# Robust Demarcation of the Family *Caryophanaceae* (*Planococcaceae*) and Its Different Genera Including Three Novel Genera Based on Phylogenomics and Highly Specific Molecular Signatures

**DOI:** 10.3389/fmicb.2019.02821

**Published:** 2020-01-14

**Authors:** Radhey S. Gupta, Sudip Patel

**Affiliations:** Department of Biochemistry and Biomedical Sciences, Faculty of Health Sciences, McMaster University, Hamilton, ON, Canada

**Keywords:** *Planococcaceae* and *Caryophanaceae* families, phylogenomic and comparative genomic analyses, conserved signature indels for different clades, *Lysinibacillus*, *Metasolibacillus* gen. nov., *Metalysinibacillus* gen. nov., *Metaplanococcus* gen. nov., emended descriptions of the *Caryophanaceae*/*Planococcaceae* genera

## Abstract

The family *Caryophanaceae*/*Planococcaceae* is a taxonomically heterogeneous assemblage of >100 species classified within 13 genera, many of which are polyphyletic. Exhibiting considerable phylogenetic overlap with other families, primarily *Bacillaceae*, the evolutionary history of this family, containing the potent mosquitocidal species *Lysinibacillus sphaericus*, remains incoherent. To develop a reliable phylogenetic and taxonomic framework for the family *Caryophanaceae*/*Planococcaceae* and its genera, we report comprehensive phylogenetic and comparative genomic analyses on 124 genome sequences from all available *Caryophanaceae*/*Planococcaceae* and representative *Bacillaceae* species. Phylogenetic trees were constructed based on multiple datasets of proteins including 819 core proteins for this group and 87 conserved *Firmicutes* proteins. Using the core proteins, pairwise average amino acid identity was also determined. In parallel, comparative analyses on protein sequences from these species have identified 92 unique molecular markers (synapomorphies) consisting of conserved signature indels that are specifically shared by either the entire family *Caryophanaceae*/*Planococcaceae* or different monophyletic clades present within this family, enabling their reliable demarcation in molecular terms. Based on multiple lines of investigations, 18 monophyletic clades can be reliably distinguished within the family *Caryophanaceae*/*Planococcaceae* based on their phylogenetic affinities and identified molecular signatures. Some of these clades are comprised of species from several polyphyletic genera within this family as well as other families. Based on our results, we are proposing the creation of three novel genera within the family *Caryophanaceae*/*Planococcaceae*, namely *Metalysinibacillus* gen. nov., *Metasolibacillus* gen. nov., and *Metaplanococcus* gen. nov., as well as the transfer of 25 misclassified species from the families *Caryophanaceae*/*Planococcaceae* and *Bacillaceae* into these three genera and in *Planococcus*, *Solibacillus*, *Sporosarcina*, and *Ureibacillus* genera. These amendments establish a coherent taxonomy and evolutionary history for the family *Caryophanaceae*/*Planococcaceae*, and the described molecular markers provide novel means for diagnostic, genetic, and biochemical studies. Lastly, we are also proposing a consolidation of the family *Planococcaceae* within the emended family *Caryophanaceae*.

## Introduction

The family *Planococcaceae* is a diverse assemblage of bacteria within the order *Bacillales* comprising 14 validly published genera (viz. *Planococcus*, *Bhargavaea*, *Chryseomicrobium*, *Filibacter*, *Indiicoccus*, *Jeotgalibacillus*, *Kurthia*, *Marinibacillus*, *Paenisporosarcina*, *Planomicrobium*, *Psychrobacillus*, *Savagea*, *Sporosarcina*, and *Ureibacillus*) (based on updated information available from the Names for Life Server in September 2019^1^). It contains >100 species with varying morphology which are Gram-variable, spore forming or non-spore forming, motile or non-motile that are usually aerobic (Ludwig et al., 2009; Shivaji et al., 2014; Yilmaz et al., 2014). Of these species, *Lysinibacillus sphaericus* is of particular importance as some strains of this species produce proteins/toxins that exhibit potent activity against mosquito larvae, and thus have been widely used as biocontrol agents for disease-transmitting mosquitoes (Baumann et al., 1991; Berry, 2012). However, the absence of any known characteristics exclusive to all members of the family *Planococcaceae* and a lack of other reliable means for classifying its members has made demarcation of this family very difficult (Ludwig et al., 2009; Shivaji et al., 2014). Although several phylogenetic studies have focused on specific genera within *Planococcaceae*, the evolutionary history of the family as a whole remains unclear as evidenced by the observation that different taxonomic databases/studies indicate different genera belonging to this family (Yoon et al., 2001a; Yarza et al., 2010; Parte, 2014; Shivaji et al., 2014; Mual et al., 2016; Maayer et al., 2019).

The current classification of species within this family relies on a limited number of phenotypic characteristics, 16S rRNA gene signature nucleotides, and the branching observed in phylogenetic trees based on 16S rRNA gene sequences (Dai et al., 2005; Ludwig et al., 2009; Shivaji et al., 2014). Utilizing these methods, several genera have been frequently added and removed from this family in recent years providing a better insight into their interrelationships than was previously attainable (Yoon et al., 2001a; Arora et al., 2011; Shivaji et al., 2014; Xu et al., 2015; Tetz and Tetz, 2018). However, the studies based on 16S rRNA gene sequences have low discriminatory power at the species and genus levels resulting in poorly resolved interrelationships of the members of the family *Planococcaceae* in phylogenetic trees based on 16S rRNA (Konstantinidis and Tiedje, 2005; Yarza et al., 2010; Maayer et al., 2019). For instance, the family remains polyphyletic as it exhibits considerable overlap with species from genera belonging to the families *Bacillaceae* and *Incertae sedis* 19 (Farrow et al., 1992; Seiler et al., 2013; Shivaji et al., 2014; Xu et al., 2015; Mual et al., 2016; Maayer et al., 2019). Furthermore, it has been well documented that several genera within the family *Planococcaceae* (viz. *Planococcus*, *Planomicrobium*, *Filibacter*, and *Sporosarcina*) do not form distinct clades and exhibit polyphyletic branching in phylogenetic trees (Shivaji et al., 2014; Xu et al., 2015; Maayer et al., 2019). The nomenclature of the family *Planococcaceae* also presents a taxonomic anomaly as highlighted recently by Tindall (2019). The taxonomic anomaly results from the fact that the family *Planococcaceae* was validly published in 1949 (Krasil’nikov, 1949), but it includes within it the family *Caryophanaceae*, which was validly published in 1939 (Peshkoff, 1939). Based on the International Code of Nomenclature of Prokaryotes (ICNP) (Parker et al., 2019), due to the earlier valid publication of the name *Caryophanaceae*, this name has priority over the family name *Planococcaceae* (Tindall, 2019). To rectify this anomaly, in the present work we are proposing a unification of the family *Planococcaceae* within the emended family *Caryophanaceae*. Therefore, hence forward, we will be referring to this family as either the *Caryophanaceae* or *Caryophanaceae*/*Planococcaceae* family. The present study was undertaken with the aim of gaining a robust understanding of the interrelationships among the *Caryophanaceae* species using multiple genomic sequences-based approaches to provide reliable means for demarcating this family and different genus level taxa within this family.

Due to significant advancements in genome sequencing technology, genome sequences are now available for the majority of the named *Caryophanaceae* species, providing an excellent representation of the overall genetic diversity that exists within this family. This genomic data has already been employed, to a limited extent, to study the phylogeny and taxonomy of a small number of *Caryophanaceae*/*Planococcaceae* species in recent years and continues to develop as a promising resource for clarifying the evolutionary history of *Caryophanaceae* species (Xu et al., 2015; Maayer et al., 2019). In addition, genome sequences are also available for >150 other species belonging to the family *Bacillaceae*, its closest phylogenetic relative (Ludwig et al., 2009; Yakoubou et al., 2010; Shivaji et al., 2014; Patel and Gupta, 2019). In the present study, we have used genomic information from *Caryophanaceae* and *Bacillaceae* species to comprehensively examine the interrelationships among species within these families using phylogenomic and comparative genomic approaches. Based on genome sequences, phylogenetic trees were constructed based on four large datasets of protein sequences. These trees not only confirm the polyphyletic nature of the family *Caryophanaceae* and the presence of polyphyletic genera within it, but they also consistently identified 18 distinct clades within the family, some of which consisted of genera that are not currently classified as belonging to the family *Caryophanaceae*/*Planococcaceae*. In parallel, detailed comparative analyses of protein sequences from these species have identified 92 novel molecular markers in the form of conserved signature indels (CSIs) which are either specific for the entire family *Caryophanaceae* or specific clades/genera within this family, which are reliably observed in all constructed phylogenomic trees. The identified CSIs provide novel and reliable means for the demarcation of the family *Caryophanaceae* as well as different observed species groups within this family in molecular terms (Gao and Gupta, 2012; Adeolu et al., 2016; Gupta, 2016; Dobritsa et al., 2017; Patel and Gupta, 2019). Based on the results from our analyses, we propose here the creation of three novel genera (viz. *Metasolibacillus* gen. nov., *Metalysinibacillus* gen. nov., and *Metaplanococcus* gen. nov.) within the family *Caryophanaceae* and also propose the transfer of 25 misclassified species from the families *Caryophanaceae* and *Bacillaceae* into the different reliably demarcated genera that are now part of the emended family *Caryophanaceae*.

## Materials and Methods

### Phylogenetic and Genomic Analysis

Phylogenetic trees were constructed for 124 species comprising all available *Caryophanaceae*/*Planococcaceae* and some representative *Bacillaceae* species whose complete genomes were available in the NCBI genome database along with *Streptococcus pyogenes*, *S. mitis*, *Lactococcus piscium*, and *L. lactis*, which were used to root the trees^2^. Phylogenetic analyses were carried out as in our earlier work (Patel and Gupta, 2018; Gupta et al., 2019) using an internally developed pipeline (Adeolu et al., 2016). Using CD-HIT program (Fu et al., 2012), protein families were identified that were present in at least 80% of the input genomes and shared >50% in sequence identity and sequence lengths. Clustal Omega program (Sievers et al., 2011) was used for the creation of multiple sequence alignments (MSAs) and after removal of poorly aligned regions with TrimAl (Capella-Gutierrez et al., 2009) sequences were concatenated. Maximum-likelihood trees based on the alignments were constructed using FastTree 2 (Price et al., 2010) based on the Whelan and Goldman (2001) model. Optimization of the trees was carried out using RAxML 8 (Stamatakis, 2014) based on Le and Gascuel (2008) model. RAxML 8 was also used to calcuate the SH-like statistical support values for different nodes and the trees were drawn using MEGA 6 (Tamura et al., 2013). The sequence alignments of the conserved core genome proteins were also used for calculation of the pairwise average amino acid sequence identity (AAI) between each pair of genomes (Thompson et al., 2013).

In addition to the protein-based trees, an unrooted 16S rRNA-based phylogenetic tree was also constructed using gene sequences for all available *Planococcaceae* and some representative *Bacillaceae* species (109 total species) retrieved from the All-Species Living Tree Project (Yilmaz et al., 2014). Type strains were obtained, if available, for all species which were then aligned using ClustalX 2.1 (Jeanmougin et al., 1998). Non-conserved regions and positions with gaps were removed from the alignment. A maximum-likelihood phylogenetic tree based on this alignment, which consisted of 1348 positions, was created using MEGA 6 (Tamura et al., 2013) using Kimura 2-parameter model (Kimura, 1980) based on 1000 bootstrap replicates as described in earlier work (Patel and Gupta, 2018). Similar results were obtained when MEGA X instead of MEGA6 was used for construction of the 16S rRNA tree.

### Identification of Conserved Signature Indels

Conserved signature indels were identified using the method detailed by Gupta (2014). For these analyses, BLASTp searches on protein sequences from the genomes of *Lysinibacillus boronitolerans*, *Lysinibacillus endophyticus*, *Planococcus citreus*, and *Sporosarcina ureae* were carried out against the NCBI non-redundant database and sequences for 10–15 divergent *Planococcaceae* and *Bacillaceae* species and six to eight outgroup species were retrieved. Subsequent analyses on these protein sequences were carried out as previously described (Patel and Gupta, 2018; Gupta et al., 2019). “Briefly, MSAs of different proteins were created using ClustalX 2.1 (Jeanmougin et al., 1998) and inspected for sequence gaps of fixed lengths which were flanked by at least four to five conserved residues in the neighboring 40–50 amino acids and appeared to be shared by either most or all homologs belonging to a certain group (Gupta, 2014). Query sequences encompassing the gap and flanking regions (40–100 amino acids long) were subjected to a second BLASTp search and the resulting top 500–1000 hits were examined to assess the group specificity of the identified CSIs. Signature files reported here were created using the SIG_CREATE and SIG_STYLE programs that are available on Gleans.net (Gupta, 2014). Unless otherwise stated, the CSIs described here are exclusive to the indicated groups of species and absent in other homologs (in the top 500 BLASTp hits examined). Due to space constraints, sequence information is shown for only a limited number of species in the main figures; however, unless otherwise indicated, the described CSIs are also present in other members of the indicated groups.” For some of the proteins containing these CSIs, homologs were not present in all species from a given clade. Detailed information for all CSIs identified in this study is provided in the Supplementary Material.

## Results

### Phylogenetic Analysis of the Family *Caryophanaceae*/*Planococcaceae*

**FIGURE 1:**
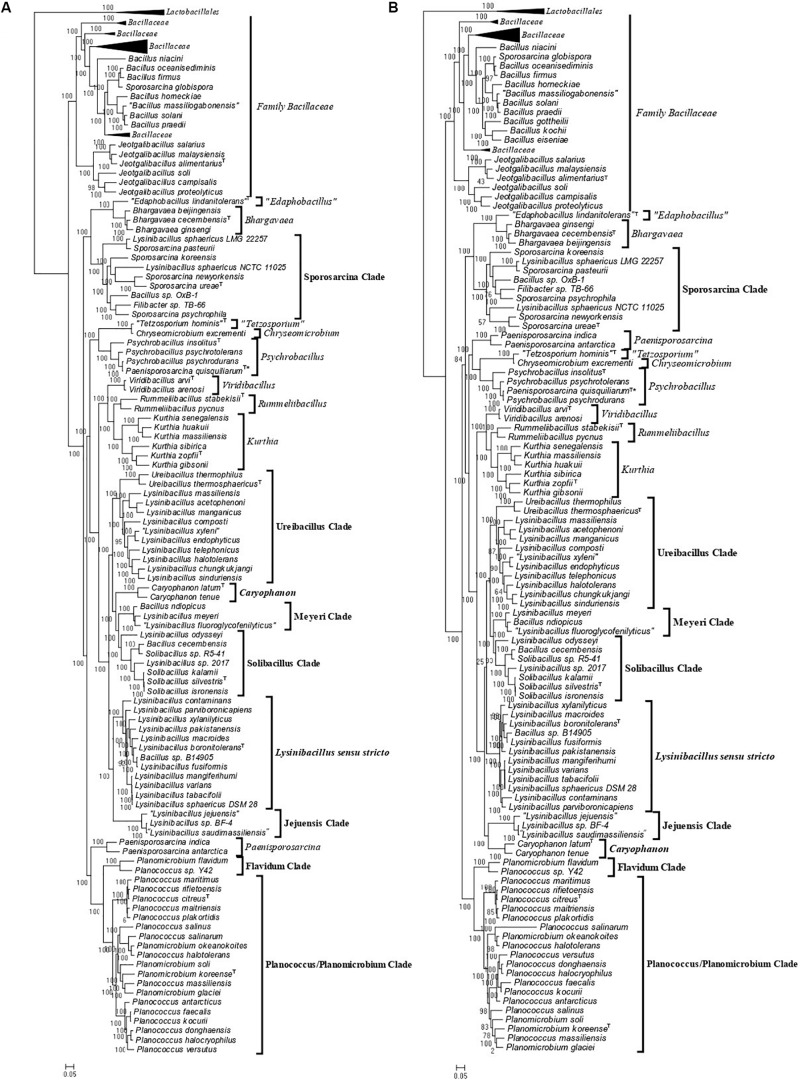
Maximum-likelihood phylogenetic trees for 124 genome sequenced members of the family *Caryophanaceae*/*Planococcaceae* and some representative members of the family “*Bacillaceae*” based on **(A)** 819 core proteins for this group of species, and **(B)** a set of 87 conserved proteins that are part of the phyloeco marker set for the phylum *Firmicutes* (Wang and Wu, 2013). Both trees were rooted using genome sequences of *Streptococcus pyogenes*, *Streptococcus mitis*, *Lactococcus piscium*, and *Lactococcus lactis* (labeled as Lactobacillales). SH-like statistical support values are indicated at each branch node. All clades observed in this study are labeled and presented with square brackets. The specific clades of interest are indicated in bold. A superscript “T” indicates the type species of a specific genus and the asterisk (^∗^) indicates the genome of *Psychrobacillus quisquiliarum* seems to be contaminated. The scale bars at the bottom represent 0.05 changes per amino acid position for each tree.

Using 124 genomes of all available genome-sequenced *Caryophanaceae* species and representative species from the family *Bacillaceae*, several phylogenetic trees were constructed based on different datasets of proteins. Each species in these trees is represented by a single genomic sequence generally of the type strain of the species when available. The trees were constructed based on concatenated sequences for (i) 819 core genome proteins for this selection of species consisting of 256,546 aligned amino acids (Figure 1A); (ii) 87 proteins commonly shared by species of the phylum *Firmicutes* (Wang and Wu, 2013) containing 26,445 aligned amino acids (Figure 1B); (iii) the DNA helicase II and DNA polymerase I proteins (Supplementary Figure 1A), and (iv) the two major subunits (RpoB and RpoC) of the RNA polymerase protein (Supplementary Figure 1B). All constructed trees exhibited nearly identical branching patterns and interrelationships among different *Caryophanaceae* and *Bacillaceae* species with high statistical support values at virtually every node. Hence, all trees reveal consistent, robust, and reliable species’ relationships among the family *Caryophanaceae*. In every tree, the family *Caryophanaceae* is comprised of 18 distinct clades of species from several different genera, some of which are currently classified as belonging to the family *Bacillaceae* (viz. *Bacillus*, *Lysinibacillus*, *Viridibacillus*, and “*Edaphobacillus*”) or the family “*Incertae sedis 19*” (viz. *Rummeliibacillus* and *Solibacillus*). In these trees, species from the genera *Caryophanon*, *Chryseomicrobium*, *Kurthia*, *Rummeliibacillus*, *Viridibacillus*, “*Edaphobacillus*,” and “*Tetzosporium*” all branched separately or formed independent monophyletic clades, while species from the remaining genera either exhibited polyphyletic branching or were not clearly separated from species of other closely related genera.

As an example of the polyphyletic and paraphyletic branching of species from genera that are part of the emended family *Caryophanaceae*, members of the genus *Lysinibacillus* were consistently found to form six different clusters, some of which were interspersed with species from other genera within the family *Caryophanaceae*. Eleven *Lysinibacillus* species branched together with the type species, *L. boronitolerans*, to form the *Lysinibacillus sensu stricto* clade; three other *Lysinibacillus* species formed a distant cluster, which we have marked as the “Jejuensis clade.” Ten other *Lysinibacillus* species are seen branching alongside *Ureibacillus* species with small branch separation to form the “Ureibacillus clade”; two *Lysinibacillus* species are seen branching with *Bacillus ndiopicus* to form the “Meyeri clade,” while two other *Lysinibacillus* species are observed to be interspersed between *Solibacillus* species forming the “Solibacillus clade.” Finally, two non-type strains of *L. sphaericus* are observed branching within the “Sporosarcina clade” which is primarily comprised of *Sporosarcina* species. Other genera which also displayed polyphyletic branching are *Planococcus* and *Planomicrobium* whose species are interspersed among one another within a larger clade of 19 species which we have called the “Planococcus/Planomicrobium clade” and a smaller clade of two species which we have called the “Flavidum clade.” Several *Bacillus* species are also seen branching within the family *Caryophanaceae*, further contributing to the polyphyly of the genera *Solibacillus*, *Sporosarcina*, and *Lysinibacillus*. In the trees based on genome sequences, *Paenisporosarcina quisquiliarum*, which is the type species of *Paenisporosarcina*, is also found to branch independently from other *Paenisporosarcina* species within a clade comprised of all genome-sequenced *Psychrobacillus* species making both *Paenisporosarcina* and *Psychrobacillus* polyphyletic. However, as clarified in the section “Discussion,” the anomalous branching of *P. quisquiliarum* within *Psychrobacillus* is very likely due to a mislabeling of this genome. Finally, the genus *Jeotgalibacillus* and *Sporosarcina globispora*, which are currently classified as belonging to the family *Caryophanaceae*/*Planococcaceae*, are seen branching with the representative species of the family *Bacillaceae* in all constructed phylogenetic trees. All 18 identified clades are depicted and labeled in the phylogenetic trees (Figure 1 and Supplementary Figure 1) with square brackets and the clades of interest, which were further investigated, are differentiated by bold labels.

**FIGURE 2:**
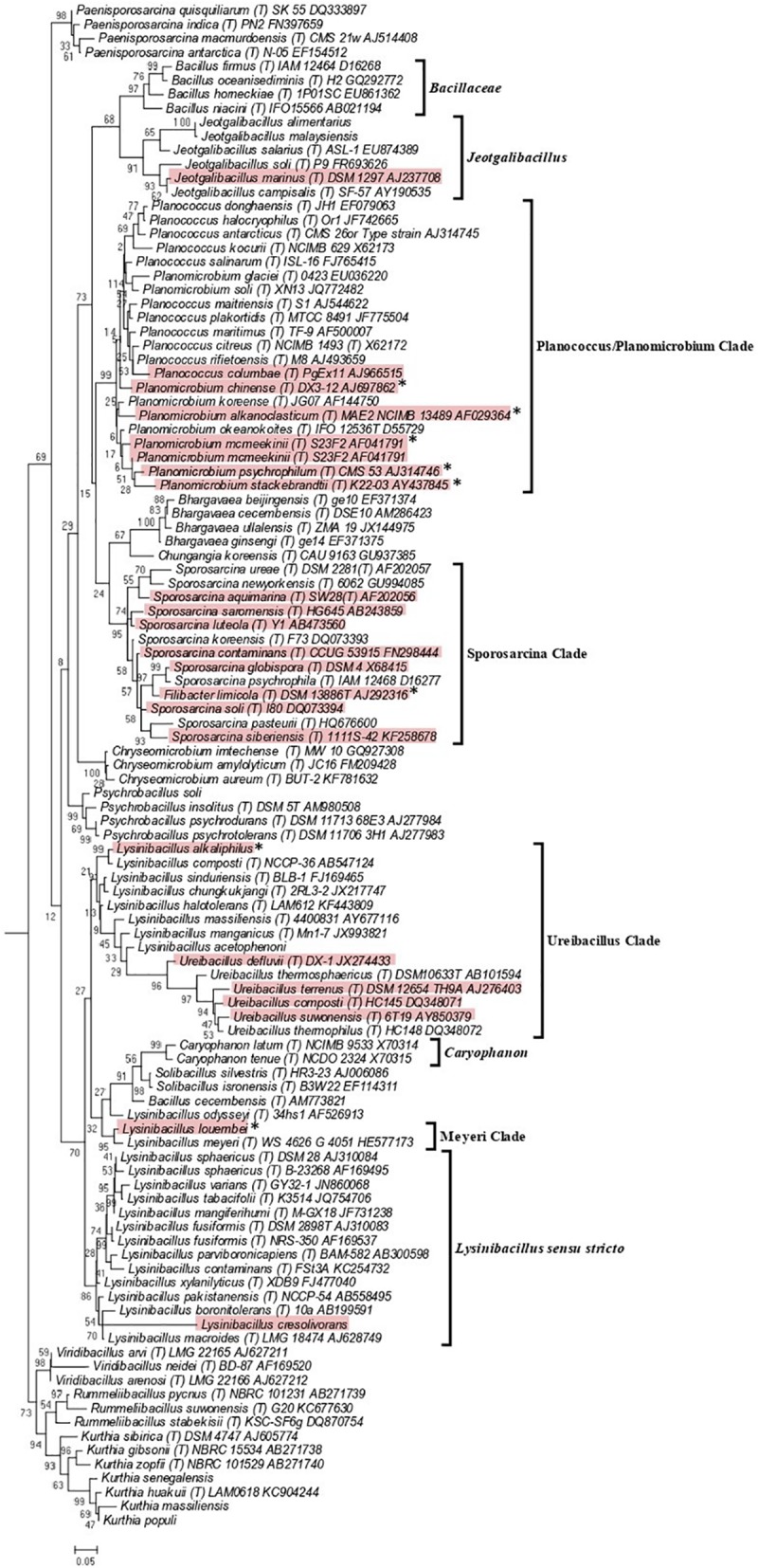
Maximum-likelihood phylogenetic tree for 109 *Caryophanaceae*/*Planococcaceae* and *Bacillaceae* species based on 16S rRNA gene sequences retrieved from the All-Species Living Tree Project (Yilmaz et al., 2014). The evolutionary history was inferred based on the Kimura 2-parameter model (Kimura, 1980). The tree is drawn to scale, with branch lengths measured in the number of substitutions per site. The scale bar at the bottom represents 0.05 changes per nucleotide position. All species which had their 16S rRNA gene sequence of their type strains are indicated by (T) followed by the name of the strains. The proposed clades seen in this tree are labeled with square brackets and all non-genome-sequenced species which are part of these clades are highlighted in red with the species which we have proposed be transferred labeled with an asterisk (^∗^). This tree is unrooted.

As the genome scale and concatenated protein-based trees were limited to only those *Caryophanaceae* species for which genomic sequences were available, a 16S rRNA-based phylogenetic tree for 109 species of the family *Caryophanaceae* and some representative *Bacillaceae* species was also constructed to discern the relative branching of all named *Caryophanaceae* species whose 16S rRNA gene sequences were available in the All-Species Living Tree Project (Figure 2; Yilmaz et al., 2014). Although the different nodes in the 16S rRNA tree are not as strongly supported, the overall branching pattern observed is very similar to that seen in the protein trees. For instance, *Lysinibacillus*, *Viridibacillus*, *Rummeliibacillus*, and *Solibacillus* are all seen branching within the family *Caryophanaceae*/*Planococcaceae* and the genera *Lysinibacillus*, *Sporosarcina*, *Planococcus*, and *Planomicrobium* also exhibited polyphyletic branching in this tree as well. Furthermore, all species of the *Lysinibacillus sensu stricto*, Meyeri, “Planococcus/Planomicrobium,” “Sporosarcina,” “Ureibacillus,” *Caryophanon*, and *Jeotgalibacillus* clades are also seen generally clustering together in the 16S rRNA-based tree and are labeled accordingly in Figure 2 with square brackets. Apart from the genome-sequenced species of the observed clades indicated in Figure 1 and Supplementary Figure 1, the 16S rRNA tree shows additional non-genome sequenced species also branching within these clades which are highlighted in red in Figure 2. *Jeotgalibacillus* is also seen branching with the family *Bacillaceae* in this tree while *S. globispora* is seen branching alongside other *Sporosarcina* species in the family *Caryophanaceae*. Lastly, unlike the genome sequenced-based trees (Figure 1 and Supplementary Figure 1), *Psychrobacillus* species and *Paeinsporosarcina* species form independent monophyletic clades in the 16S rRNA tree and *P. quisquiliarum* branches with the other *Paenisporosarcina* species, as expected.

### Genome Similarity Among *Caryophanaceae* Species

**FIGURE 3:**
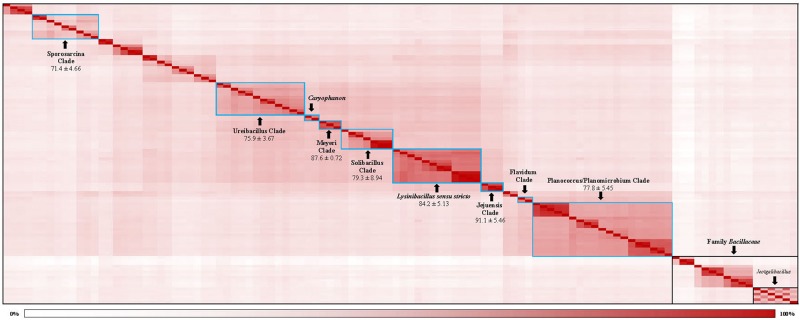
A matrix indicating the percentage of average amino acid identities shared by members of the family *Caryophanaceae*/*Planococcaceae* analyzed in this study based on 819 core proteins for this set of species. Higher amino acid identity shared by a pair of species are colored more darkly (red). The specific clades observed based on higher interspecies similarity are boxed with blue borders and labeled with arrows. The mean and standard deviation in AAI values for different clades of interest are indicated. The “Caryophanon” and “Flavidum” clades have only 1 AAI similarity values and hence the mean and standard deviation values for these clades could not be determined. The family *Bacillaceae* and the genus *Jeotgalibacillus* are indicated with boxes with black borders. A detailed amino acid matrix with the numerical values underlying this amino acid matrix and the species names is provided in Supplementary Figure 2.

The genetic relatedness across the family *Caryophanaceae* was measured by calculating the pairwise average amino acid identity (AAI) between species pairs using a concatenated sequence alignment of 819 core proteins shared by *Caryophanaceae*/*Planococcaceae* species and some representative *Bacillaceae* species (Konstantinidis and Tiedje, 2005; Konstantinidis and Stackebrandt, 2013; Thompson et al., 2013). The resulting AAI information is depicted in Figure 3 in the form of a matrix where genome pairs exhibiting a greater degree of similarity are represented with a darker shade of red. The highest AAI is observed between species that form the 18 identified clades in the phylogenetic trees mentioned above. Although comparison of AAI values provides a useful means for distinguishing prokaryotic taxa based on their genomic similarities (Konstantinidis and Stackebrandt, 2013; Sangal et al., 2016), there is no reliable threshold value that is generally applicable for the distinction of genus level taxa (Qin et al., 2014; Gupta, 2019). However, based on their AAI values, the nine clades of interest, which are boxed and labeled with black arrows in Figure 3, can be clearly distinguished from each other. The mean AAI values of species from these nine clades are all >70% and the deviation from mean in these values is 5% or less in most cases. The observed AAI values support the distinctness of the identified clades. A detailed AAI matrix with the AAI values for each pair of species is provided in Supplementary Figure 2.

### Comparative Genomic Analysis of Different Monophyletic Clades of the Family *Caryophanaceae*

The results of our comprehensive phylogenetic studies indicated the existence of several clades of species comprised of distinct genera within the family *Caryophanaceae*. However, the topology of phylogenetic trees can be influenced by many variables (Gupta, 1998, 2016; Klenk and Goker, 2010). Thus, it is important to confirm the presence of the observed clades and their genetic distinctness by other means. Molecular markers, such as conserved signature insertions and deletions (CSIs) within genes/proteins that are exclusively shared by members from a given group of species, have proven very useful in the reliable demarcation of different species clades and for clarifying their evolutionary relationships and taxonomy (Baldauf and Palmer, 1993; Rokas and Holland, 2000; Adeolu and Gupta, 2014; Bhandari and Gupta, 2014; Gupta, 2014, 2016; Naushad et al., 2014; Dobritsa et al., 2017; Hu et al., 2019). The most parsimonious explanation for the presence of these clade-specific CSIs is that the genetic changes leading to them occurred in a common ancestor of the group(s) and they were then vertically inherited by subsequent descendants (Baldauf and Palmer, 1993; Gupta, 1998, 2014, 2016; Rokas and Holland, 2000). Thus, in view of their unique shared ancestry, the CSIs represent synapomorphic characteristics that provide reliable evidence, independent of the topology observed in phylogenetic trees, of the evolutionary relatedness of a given group of species. Hence, in this study we carried out comprehensive comparative genomic analyses of protein sequences from genomes of *Caryophanaceae* and some representative *Bacillaceae* species to identify CSIs that are specific for different novel and distinct monophyletic clades of *Caryophanaceae* species. These analyses have identified 13 CSIs which are specific for the emended family *Caryophanaceae*, and 79 CSIs that are distinctive characteristics of the nine clades of interest (all of which are also observed in the phylogenetic trees) within this family. The results of these analyses are briefly described in the subsections below.

#### Conserved Signature Indels Specific for the Family *Caryophanaceae*

**FIGURE 4:**
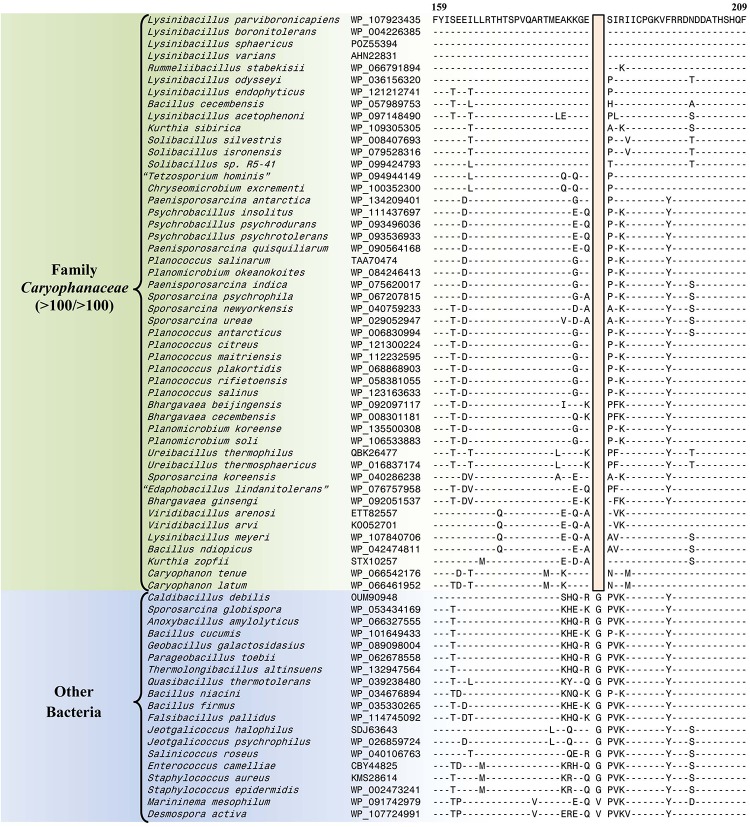
Partial sequence alignment of the phenylalanine–tRNA ligase subunit alpha protein showing a one amino acid deletion (boxed) that is exclusively shared by all members of the emended family *Caryophanaceae*. Sequence information for a limited number of *Caryophanaceae* species and other bacteria are shown here, but unless otherwise indicated, similar CSIs were detected in all members of the indicated group and not detected in any other species in the top 1000 BLASTp hits. The dashes (-) in this alignment and all other alignments presented in this paper indicate identity with the residue in their respective top sequences. Accession numbers for each sequence are indicated in the second column. Detailed sequence alignments for this CSI as well as additional CSIs specific for the family *Caryophanaceae* are presented in Supplementary Figures 3–15 and some of their characteristics are summarized in Table 1.

The emended family *Caryophanaceae* consists of >100 genome-sequenced species that consistently group together in different phylogenetic trees (Figure 1 and Supplementary Figure 1). A specific grouping of these species is also supported by our identification of 13 CSIs that are exclusively shared by all of the genome-sequenced species from this clade. One example of such a CSI consisting of a 1 aa deletion in a highly conserved region of the protein phenylalanine–tRNA ligase alpha subunit, which is specific for this family, is presented in Figure 4. Detailed sequence information for this CSI and the 12 other CSIs that are also specific for the family *Caryophanaceae* are provided in Supplementary Figures 3–15 and some of their characteristics are summarized in Table 1. The unique shared presence of these CSIs in the indicated groups of species serves to reliably demarcate the members of the family *Caryophanaceae* in molecular terms.

**TABLE 1:** Summary of conserved signature indels specific for the family *Caryophanaceae*.

**Protein name**	**Accession number**	**Figure number**	**Indel size**	**Indel position**	**Specificity**
Phenylalanine– tRNA ligase subunit alpha	WP_121176350	Figure 4 and Supplementary Figure 3	1 aa del	159–209	Family *Caryophanaceae*
Chaperonin GroEL^a^	WP_036075467	Supplementary Figure 4	2 aa ins	401–454	
Ribosome maturation factor RimP	WP_057986972	Supplementary Figure 5	1 aa ins	21–65	
BrxA/BrxB family bacilliredoxin	WP_057987839	Supplementary Figure 6	1 aa ins	29–82	
RNA methyltransferase	WP_057989758	Supplementary Figure 7	1 aa ins	4–41	
Rhomboid family intramembrane serine protease	WP_029500435	Supplementary Figure 8	1 aa del	123–171	
ATP-dependent Clp protease ATP-binding subunit	WP_057986366	Supplementary Figure 9	1 aa ins	40–99	
DNA-directed RNA polymerase subunit beta	WP_057986323	Supplementary Figure 10	27 aa ins	583–625	
Chorismate synthase^a^	WP_057982230	Supplementary Figure 11	2 aa ins	148–213	
Stage IV sporulation protein A	WP_057982892	Supplementary Figure 12	2 aa ins	53–101	
Peptidase	WP_057987802	Supplementary Figure 13	6 aa del	221–272	
KinB-signaling pathway activation protein^a^	WP_057986232	Supplementary Figure 14	3 aa ins	153–196	
DUF423 domain-containing protein	WP_042478071	Supplementary Figure 15	2–4 aa ins	39–92	

*^*a*^Minor exceptions or anomalies present (refer to figure for details).*

#### Conserved Signature Indels Specific for the *Lysinibacillus sensu stricto* Clade

**FIGURE 5:**
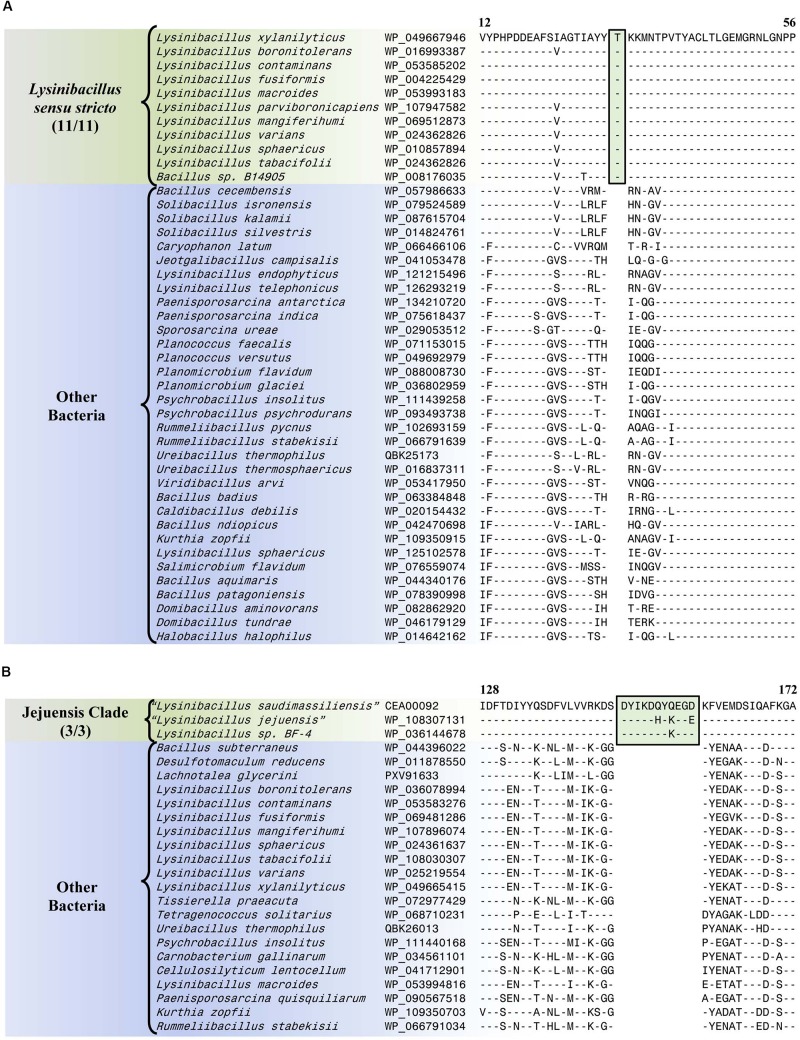
Partial sequence alignment of **(A)** the bacillithiol biosynthesis deacetylase BshB2 protein showing a one amino acid insertion (boxed) that is exclusively shared by all members of the *Lysinibacillus sensu stricto* clade, and **(B)** the arginine-binding extracellular protein ArtP precursor containing an 11 amino acid insertion that is exclusively shared by all members of the Jejuensis clade. Detailed sequence alignments for these CSIs as well as additional CSIs specific for these clades are presented in Supplementary Figures 16–21 for the *Lysinibacillus sensu stricto* clade and Supplementary Figures 22–38 for the Jejuensis clade and some of their characteristics are summarized in Table 2.

The *Lysinibacillus sensu stricto* clade is a monophyletic clade consisting of 11 genome-sequenced *Lysinibacillus* species, including *L. boronitolerans* (the nomenclatural type of the genus *Lysinibacillus*), and *Bacillus* sp. B14905 which consistently group together in different phylogenetic trees (Figure 1 and Supplementary Figure 1). Dunlap (2019) has recently indicated that of the *Lysinibacillus* species which are part of this clade, *L. mangiferihumi*, *L. tabacifolii*, and *L. varians* are later heterotypic synonyms of *L. sphaericus*. Additionally, after this work was completed, a new *Lysinibacillus* species, *L capsici*, has been described, which based on its branching in a phylogenetic tree and close similarity to other members of this clade, is also indicated to be a part of this clade (Burkett-Cadena et al., 2019). Nonetheless, a specific grouping of all genome-sequenced species, which are indicated to be part of the *Lysinibacillus sensu stricto* clade, is strongly supported by our identification of six CSIs that are exclusively shared by all of these species. One example of such a CSI consisting of a 1 aa insertion in the bacillithiol biosynthesis deacetylase BshB2 protein is presented in Figure 5A. Detailed sequence information for this CSI and the five other CSIs that are specific for the *Lysinibacillus sensu stricto* clade are provided in Supplementary Figures 16–21 and some of their characteristics are summarized in Table 2. *Lysinibacillus pakistanensis* does not appear in the CSIs presented here because it is not available in the NCBI non-redundant protein database at the time of writing this paper.

**TABLE 2:** Summary of conserved signature indels specific for the members of the *Lysinibacillus sensu stricto* clade and the Jejuensis clade.

**Protein name**	**Accession number**	**Figure number**	**Indel size**	**Indel position**	**Specificity**
Bacillithiol biosynthesis deacetylase BshB2	WP_049667946	Figure 5A and Supplementary Figure 16	1 aa ins	12–56	*Lysinibacillus sensu stricto*
PIN/TRAM domain-containing protein	WP_036077775	Supplementary Figure 17	2 aa ins	225–285	
Flagellar assembly protein FliH	WP_016994577	Supplementary Figure 18	1 aa ins	205–254	
PDZ domain-containing protein	WP_049668118	Supplementary Figure 19	3 aa ins	412–467	
TrkH family potassium uptake protein	WP_108029457	Supplementary Figure 20	2 aa ins	408–451	
D-Alanyl-D-alanine carboxypeptidase	WP_036080147	Supplementary Figure 21	21–22 aa ins	197–262	
Arginine-binding extracellular protein ArtP precursor	CEA00092	Figure 5B and Supplementary Figure 22	11 aa ins	128–172	Jejuensis clade
oxygen-independent coproporphyrinogen III oxidase	WP_108307498	Supplementary Figure 23	3 aa del	218-268	
Putative hydrolase MhqD	CEA00796	Supplementary Figure 24	1 aa del	110-160	
helix-turn-helix transcriptional regulator	WP_108306939	Supplementary Figure 25	1 aa del	82-136	
tRNA preQ1(34) S-adenosylmethionine ribosyltransferase-isomerase QueA	WP_108306792	Supplementary Figure 26	1 aa del	91-136	
DNA primase	WP_108306840	Supplementary Figure 27	1 aa del	497-535	
FMN reductase (NADPH)	CEA04024	Supplementary Figure 28	1 aa del	162–210	
UvrABC system protein C	CEA00739	Supplementary Figure 29	1 aa del	57–101	
Sensor histidine kinase YycG	CDZ99298	Supplementary Figure 30	2 aa ins	298–344	
Hypothetical protein BN1050_02162	CEA04824	Supplementary Figure 31	1 aa del	68–114	
Ribonuclease Y	CEA04602	Supplementary Figure 32	4 aa ins	20–69	
Hypothetical protein BN1050_01309	CEA02597	Supplementary Figure 33	2 aa del	55–111	
Cell division protein FtsA^a^	WP_108305708	Supplementary Figure 34	2 aa del	64–128	
ABC transporter ATP-binding protein YtrB^a^	CEA04564	Supplementary Figure 35	1 aa ins	153–206	
Cysteine–tRNA ligase^a^	CDZ99411	Supplementary Figure 36	1 aa del	318–365	
Coproporphyrinogen III oxidase^a^	WP_036144197	Supplementary Figure 37	1 aa del	435–478	
PBP1A family penicillin-binding protein^a^	WP_108306067	Supplementary Figure 38	1 aa ins	529–571	

*^*a*^Minor exceptions or anomalies present (refer to figure for details).*

#### Conserved Signature Indels Specific for the Jejuensis Clade

The “Jejuensis clade” is a monophyletic clade consisting of the genome-sequenced species “*Lysinibacillus jejuensis*,” “*Lysinibacillus saudimassiliensis*,” and *Lysinibacillus* sp. BF-4 which consistently group together in different phylogenetic trees (Figure 1 and Supplementary Figure 1). A specific grouping of these three species is also supported by our identification of 17 CSIs that are exclusively shared by them. One example of such a CSI consisting of an 11 aa insertion in the arginine-binding extracellular protein ArtP precursor is presented in Figure 5B. Detailed sequence information for this CSI and the 16 other CSIs that are specific for the “Jejuensis clade” are provided in Supplementary Figures 22–38 and some of their characteristics are summarized in Table 2.

#### Conserved Signature Indels Specific for the “Ureibacillus Clade”

**FIGURE 6:**
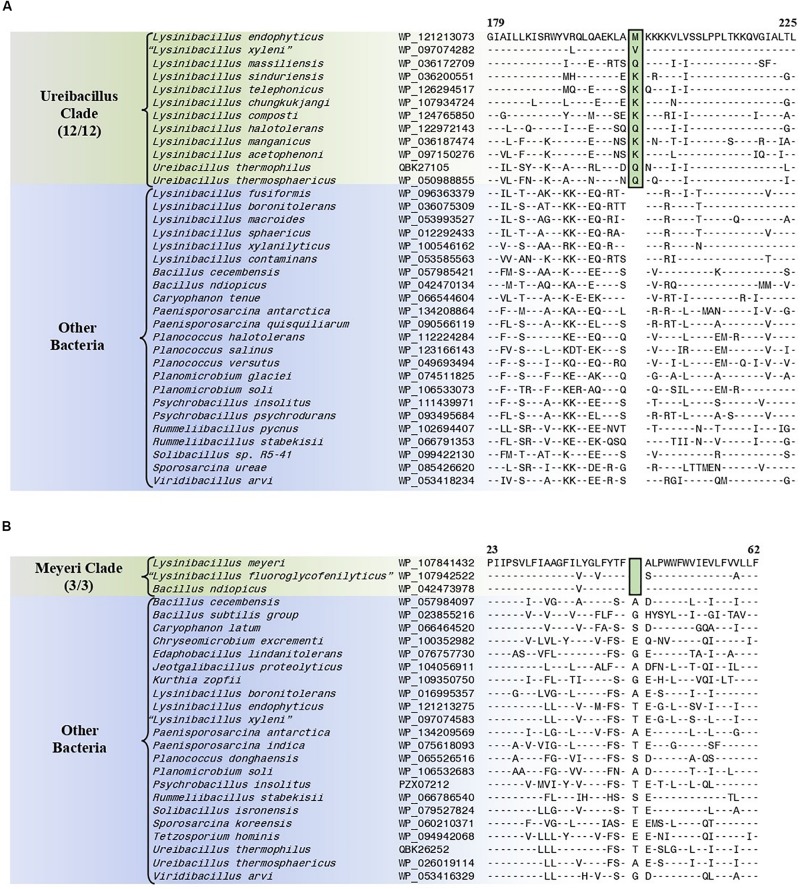
Partial sequence alignments of **(A)** the MFS transporter protein showing a one amino acid insertion (boxed) that is exclusively shared by all members of the Ureibacillus clade and **(B)** the DUF456 domain-containing protein containing a one amino acid deletion (boxed) that is exclusively shared by all members within the Meyeri clade. Detailed sequence alignments for these CSIs as well as additional CSIs specific for these clades are presented in Supplementary Figures 39–41 for the Ureibacillus clade and Supplementary Figures 42–53 for the Meyeri clade and some of their characteristics are summarized in Table 3.

The “Ureibacillus clade” is a monophyletic clade consisting of the genome-sequenced species *Ureibacillus thermosphaericus*, *U. thermophilus*, and 10 *Lysinibacillus* species which consistently group together in different phylogenetic trees (Figures 1, 2 and Supplementary Figure 1). A specific grouping of these 12 species is also supported by our identification of three CSIs that are exclusively shared by them. One example of such a CSI consisting of a 1 aa insertion in the MFS transporter protein is presented in Figure 6A. Detailed sequence information for this CSI and the two other CSIs that are specific for the “Ureibacillus clade” are provided in Supplementary Figures 39–41 and some of their characteristics are summarized in Table 3.

**TABLE 3:** Summary of conserved signature indels specific for the Ureibacillus clade, the Meyeri clade, the Solibacillus clade, and the Sporosarcina clade.

**Protein name**	**Accession number**	**Figure number**	**Indel size**	**Indel position**	**Specificity**
MFS transporter	WP_121213073	Figure 6A and Supplementary Figure 39	1 aa ins	179–225	Ureibacillus clade
EamA family transporter	WP_126296406	Supplementary Figure 40	1 aa del	177–229	
DNA internalization-related competence protein ComEC/Rec2^a^	WP_121213400	Supplementary Figure 41	1 aa del	260–320	
DUF456 domain-containing protein	WP_107841432	Figure 6B and Supplementary Figure 42	1 aa del	23–62	Meyeri clade
Toxic anion resistance protein	WP_107942781	Supplementary Figure 43	6 aa del	178–230	
Undecaprenyldiphospho-muramoylpentapeptide beta-*N*-acetylglucosaminyltransferase^a^	WP_107839309	Supplementary Figure 44	1 aa ins	164–228	
c-Type cytochrome biogenesis protein CcsB	WP_107841857	Supplementary Figure 45	15 aa ins	215–275	
Thiol-disulfide oxidoreductase ResA	WP_042477869	Supplementary Figure 46	1 aa ins	123–165	
Hypothetical protein	WP_066164326	Supplementary Figure 47	1 aa del	5–46	
Hypothetical protein	WP_107942795	Supplementary Figure 48	1 aa del	232–273	
Arginase	WP_107840234	Supplementary Figure 49	1 aa del	92–130	
Preprotein translocase subunit SecY	WP_066168906	Supplementary Figure 50	3 aa ins	291–339	
ATP-binding cassette domain-containing protein	WP_107942022	Supplementary Figure 51	2 aa del	336–373	
Purine permease	WP_042470344	Supplementary Figure 52	2 aa ins	368–416	
Thiol-disulfide oxidoreductase ResA	WP_042477869	Supplementary Figure 53	1 aa ins	123–154	
Flagellar hook–basal body protein^b^	WP_099422418	Figure 7A and Supplementary Figure 54	1 aa ins	176–213	Solibacillus clade
Aminodeoxychorismate lyase^b^	WP_057989468	Supplementary Figure 55	1 aa del	50–93	
VOC family protein	WP_057987106	Supplementary Figure 56	1 aa ins	67–116	
DNA topoisomerase IV subunit A	WP_057989369	Supplementary Figure 57	1 aa ins	266–297	
DegV family protein	WP_099423520	Supplementary Figure 58	1 aa ins	242–297	
Flagellar hook–basal body protein	WP_099422418	Supplementary Figure 59	1 aa del	206–248	
Helicase-exonuclease AddAB subunit AddB	WP_099422539	Supplementary Figure 60	6 aa ins	233–286	
Multidrug resistance efflux transporter family protein	WP_057988067	Supplementary Figure 61	2 aa ins	36–80	
Heme-dependent peroxidase	WP_057986646	Supplementary Figure 62	4 aa del	32–66	
Methionine ABC transporter ATP-binding protein	WP_099424991	Supplementary Figure 63	1 aa ins	220–265	
tRNA 4-thiouridine(8) synthase ThiI	WP_099424859	Supplementary Figure 64	1 aa ins	71–177	
AAA family ATPase	WP_099422549	Supplementary Figure 65	1 aa ins	315–361	
Aspartate–tRNA ligase	WP_083031738	Figure 7B and Supplementary Figure 66	2 aa del	435–479	Sporosarcina clade
A/G-specific adenine glycosylase	WP_029055238	Supplementary Figure 67	1 aa del	183–222	
Thymidylate synthase	WP_099690866	Supplementary Figure 68	1 aa del	138–176	
RDD family protein	WP_083032299	Supplementary Figure 69	2 aa ins	31–90	
DEAD/DEAH box helicase	WP_029055098	Supplementary Figure 70	2 aa del	332–368	
Membrane protein insase YidC	WP_099632462	Supplementary Figure 71	3 aa ins	77–124	
Cytochrome b6	WP_009765631	Supplementary Figure 72	5–6 aa ins	25–78	
Hypothetical protein	WP_083035866	Supplementary Figure 73	2 aa ins	18–53	

*^*a*^Minor exceptions or anomalies present (refer to figure for details). ^*b*^These CSIs are shared by *Lysinibacillus odysseyi.**

#### Conserved Signature Indels Specific for the “Meyeri Clade”

The “Meyeri clade” is a monophyletic clade consisting of the genome-sequenced species *Lysinibacillus meyeri*, “*Lysinibacillus fluoroglycofenilyticus*,” and *B. ndiopicus* which consistently group together in different phylogenetic trees (Figure 1 and Supplementary Figure 1). A specific grouping of these three species is also supported by our identification of 12 CSIs that are exclusively shared by them. One example of such a CSI consisting of a 1 aa deletion in the DUF456 domain-containing protein is presented in Figure 6B. Detailed sequence information for this CSI and the 11 other CSIs that are specific for the “Meyeri clade” are provided in Supplementary Figures 42–53 and some of their characteristics are summarized in Table 3.

#### Conserved Signature Indels Specific for the “Solibacillus Clade”

**FIGURE 7:**
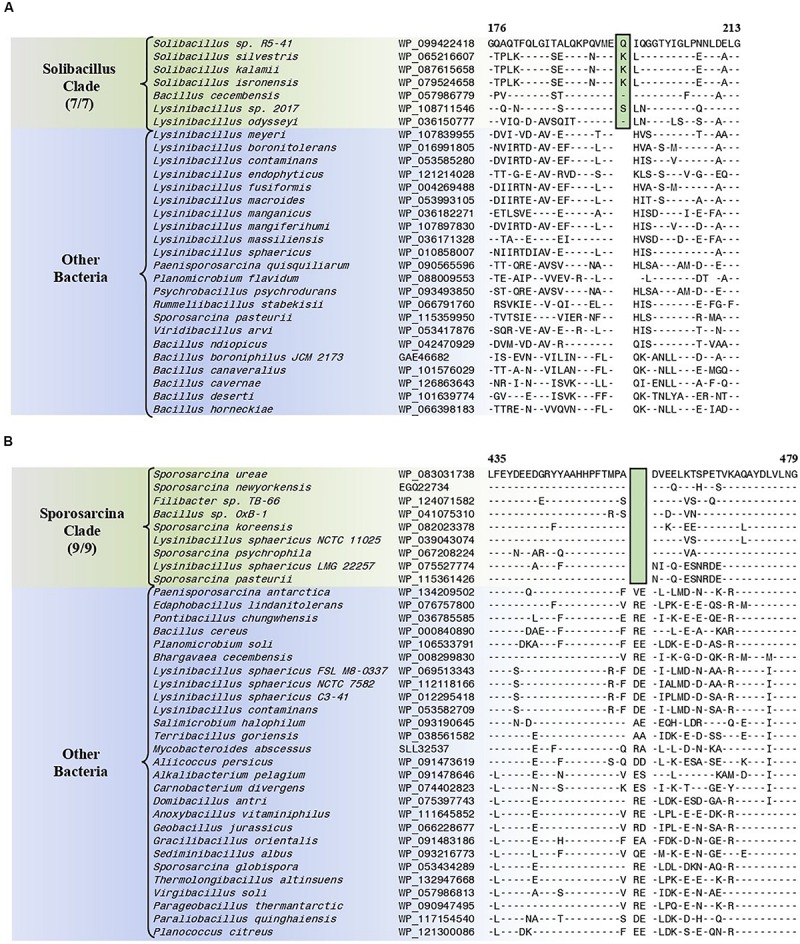
Partial sequence alignments of **(A)** the flagellar hook–basal body protein showing a one amino acid insertion (boxed) that is exclusively shared by all members of the Solibacillus clade, and **(B)** the aspartate–tRNA ligase protein containing a two amino acid deletion (boxed) that is exclusively shared by all members of the Sporosarcina clade. Detailed sequence alignments for these CSIs as well as additional CSIs specific for these clades are presented in Supplementary Figures 54–65 for the Solibacillus clade, and Supplementary Figures 66–73 for the Sporosarcina clade and some of their characteristics are summarized in Table 3.

The “Solibacillus clade” is a monophyletic clade consisting of four genome-sequenced *Solibacillus* species including *Solibacillus silvestris* (the nomenclatural type of the genus *Solibacillus*), *Lysinibacillus odysseyi*, *Lysinibacillus* sp. 2017, and *Bacillus cecembensis* which consistently group together in different phylogenetic trees (Figure 1 and Supplementary Figure 1). A specific grouping of these seven species is also supported by our identification of 12 CSIs that are exclusively shared by them. One example of such a CSI consisting of a 1 aa insertion in the flagellar hook–basal body protein is presented in Figure 7A. Detailed sequence information for this CSI and the 11 other CSIs that are specific for the “Solibacillus clade” are provided in Supplementary Figures 54–65 and some of their characteristics are summarized in Table 3.

#### Conserved Signature Indels Specific for the “Sporosarcina Clade”

The “Sporosarcina clade” is a monophyletic clade consisting of five genome-sequenced *Sporosarcina* species including *S. ureae* (the nomenclatural type of the genus *Sporosarcina*), *L. sphaericus* LMG 22257, *L. sphaericus* NCTC 11025, *Bacillus* OxB-1, and *Filibacter* sp. TB-66 which consistently group together in different phylogenetic trees (Figure 1 and Supplementary Figure 1). A specific grouping of these nine species is also supported by our identification of eight CSIs that are exclusively shared by them. One example of such a CSI consisting of a 2 aa deletion in the aspartate–tRNA ligase protein is presented in Figure 7B. Detailed sequence information for this CSI and the seven other CSIs that are specific for the “Sporosarcina clade” are provided in Supplementary Figures 66–73 and some of their characteristics are summarized in Table 3.

#### Conserved Signature Indels Specific for the “Planococcus/Planomicrobium Clade”

**FIGURE 8:**
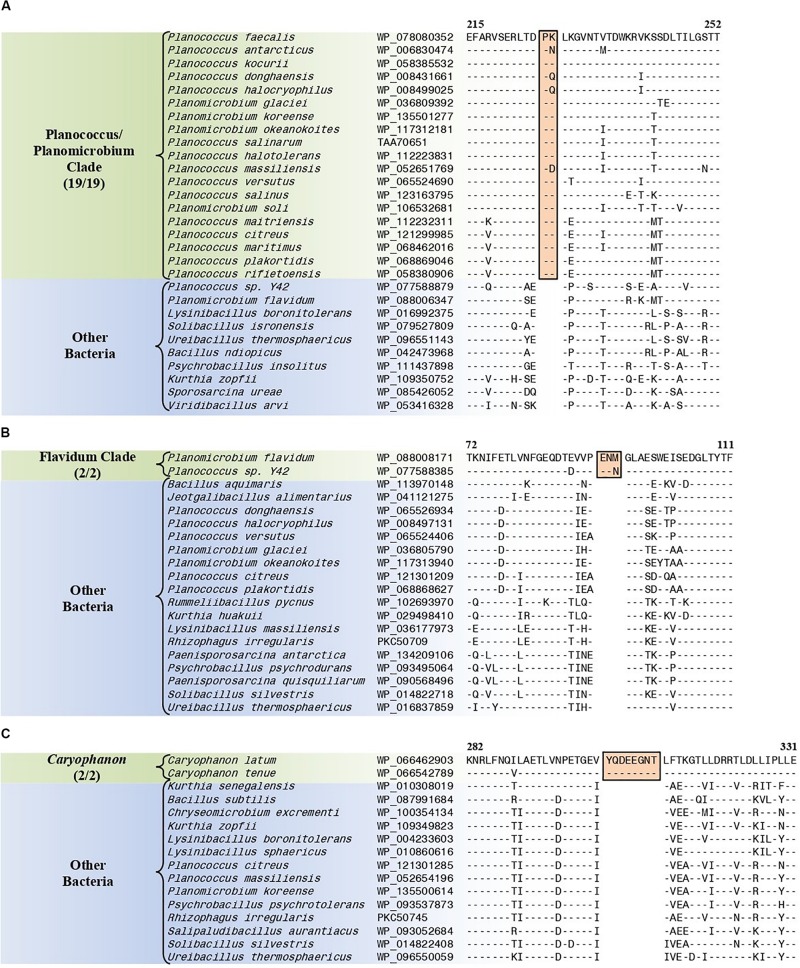
Partial sequence alignments of **(A)** the penicillin-binding protein 2 protein showing a two amino acid insertion (boxed) that is exclusively shared by all members of the Planococcus/Planomicrobium clade, **(B)** the ABC transporter substrate-binding protein containing a three amino acid insertion (boxed) that is exclusively shared by all members within the Flavidum clade, and **(C)** the DNA-directed RNA polymerase subunit beta protein containing an eight amino acid insertion that is exclusively shared by all members of the genus *Caryophanon*. Detailed sequence alignments for these CSIs as well as additional CSIs specific for these clades are presented in Supplementary Figures 74–78 for the Planococcus/Planomicrobium clade, Supplementary Figures 79–86 for the Flavidum clade, and Supplementary Figures 87–94 for the genus *Caryophanon* and some of their characteristics are summarized in Table 4.

The “Planococcus/Planomicrobium” clade is a monophyletic clade consisting of 15 genome-sequenced *Planococcus* species including *P. citreus*, and 4 *Planomicrobium* species which consistently group together in different phylogenetic trees (Figure 1 and Supplementary Figure 1). A specific grouping of these 19 species is also supported by our identification of five CSIs that are exclusively shared by them. One example of such a CSI consisting of a 2 aa insertion in penicillin-binding protein 2 is presented in Figure 8A. Detailed sequence information for this CSI and the four other CSIs that are specific for the “Planococcus/Planomicrobium clade” are provided in Supplementary Figures 74–78 and some of their characteristics are summarized in Table 4.

**TABLE 4:** Summary of conserved signature indels specific for the Planococcus/Planomicrobium clade, the Flavidum clade, and the genus *Caryophanon*.

**Protein name**	**Accession number**	**Figure number**	**Indel size**	**Indel position**	**Specificity**
Penicillin-binding protein 2	WP_078080352	Figure 8A and Supplementary Figure 74	2 aa ins	215–252	Planococcus/Planomicrobium clade
Hypothetical protein	WP_065528121	Supplementary Figure 75	3 aa ins	56–96	
NADPH-dependent 7-cyano-7-deazaguanine reductase QueF	WP_071153391	Supplementary Figure 76	1 aa del	14–65	
ACT domain-containing protein	WP_112232475	Supplementary Figure 77	1 aa del	11–64	
Methylmalonyl-CoA mutase	WP_065525509	Supplementary Figure 78	2 aa del	1009–1053	
ABC transporter substrate-binding protein	WP_088008171	Figure 8B and Supplementary Figure 79	3 aa ins	72–111	Flavidum clade
Methionine–tRNA ligase	WP_088008409	Supplementary Figure 80	1 aa ins	125–165	
MetQ/NlpA family ABC transporter substrate-binding protein	WP_088008611	Supplementary Figure 81	1 aa ins	70–129	
ABC transporter permease	WP_088005980	Supplementary Figure 82	5 aa del	122–182	
Spore protease YyaC	WP_088009144	Supplementary Figure 83	1 aa del	98–139	
*N*-acetyl-alpha-D-glucosaminyl L-malate synthase BshA^a^	WP_088007583	Supplementary Figure 84	1 aa ins	92–132	
Orotidine-5′-phosphate decarboxylase^a^	WP_088005873	Supplementary Figure 85	1 aa del	149–205	
Phospho-*N*-acetylmuramoyl-pentapeptide-transferase^a^	WP_088005773	Supplementary Figure 86	1 aa del	105–153	
DNA-directed RNA polymerase subunit beta	WP_066462903	Figure 8C and Supplementary Figure 87	8 aa ins	282–331	Genus *Caryophanon*
Peroxide-responsive transcriptional repressor PerR	WP_083998246	Supplementary Figure 88	2 aa ins	63–113	
ADP-forming succinate-CoA ligase subunit beta	WP_066543353	Supplementary Figure 89	1 aa ins	76–131	
tRNA (*N*(6)-L-threonylcarbamoyl adenosine (37)-C(2)-methylthiotransferase MtaB	WP_066542314	Supplementary Figure 90	1 aa ins	324–369	
Magnesium transporter	WP_066461515	Supplementary Figure 91	1 aa ins	325–374	
Dephospho-CoA kinase	WP_066542157	Supplementary Figure 92	1 aa del	82–120	
ATP synthase subunit I	WP_066466362	Supplementary Figure 93	1 aa ins	1–53	
Bifunctional DNA-formamidopyrimidine glycosylase/DNA-(apurinic or apyrimidinic site) lyase^a^	WP_066461928	Supplementary Figure 94	1 aa del	240–288	

*^*a*^Minor exceptions or anomalies present (Refer to figure for details).*

#### Conserved Signature Indels Specific for the “Flavidum Clade”

The “Flavidum clade” is a monophyletic clade consisting of the genome-sequenced species *Planomicrobium flavidum*, and *Planococcus* sp. Y42 which consistently group together in different phylogenetic trees (Figure 1 and Supplementary Figure 1). A specific grouping of these two species is also supported by our identification of eight CSIs that are exclusively shared by them. One example of such a CSI consisting of a 3 aa insertion in ABC transporter substrate-binding protein is presented in Figure 8B. Detailed sequence information for this CSI and the seven other CSIs that are specific for the “Flavidum clade” are provided in Supplementary Figures 79–86 and some of their characteristics are summarized in Table 4.

#### Conserved Signature Indels Specific for the Genus *Caryophanon*

The genus *Caryophanon* is a monophyletic clade consisting of the genome-sequenced species *Caryophanon latum*, and *Caryophanon tenue* which consistently group together in different phylogenetic trees (Figure 1 and Supplementary Figure 1). A specific grouping of these two species is also supported by our identification of eight CSIs that are exclusively shared by them. One example of such a CSI consisting of an 8 aa insertion in the DNA-directed RNA polymerase subunit beta protein is presented in Figure 8C. Detailed sequence information for this CSI and the seven other CSIs that are specific for the genus *Caryophanon* are provided in Supplementary Figures 87–94 and some of their characteristics are summarized in Table 4.

## Discussion

The family *Caryophanaceae* is a taxonomically heterogeneous assemblage of species from diverse genera and current methods of classification, such as 16S rRNA-based phylogenetic trees, have proven inadequate in clarifying the evolutionary history and composition of this family (Shivaji et al., 2014; Maayer et al., 2019). With no shared characteristics or reliable means for its demarcation, the family *Caryophanaceae* has become a polyphyletic assemblage of bacterial genera with conflicting classifications (Ludwig et al., 2009; Yarza et al., 2010; Parte, 2014; Shivaji et al., 2014; Maayer et al., 2019). Fortunately, recent improvements in genome sequencing technology have provided a plethora of genome sequence data for the majority of species from this family^3^, providing an exclusive and previously unavailable resource for resolving the interrelationships of different species forming the family *Caryophanaceae* via multiple independent approaches (Gupta, 2014, 2016; Dobritsa et al., 2017).

Using available genome sequences for 124 *Caryophanaceae*/*Planococcaceae* and some representative *Bacillaceae* species, in the present work, we have performed comprehensive phylogenomic analyses based on several large datasets of protein sequences. All of the constructed trees were observed to form extremely similar branching patterns and interrelationships and also consistently displayed a strongly supported monophyletic clade containing all *Caryophanaceae*/*Planococcaceae* genera (except *Jeotgalibacillus*). Within this clade, a number of genera (viz. *Lysinibacillus*, *Viridibacillus*, “*Edaphobacillus*,” *Solibacillus*, and *Rummeliibacillus*) belonging to other families within the order *Bacillales* were also interspersed. Strong independent evidence that the species from the above genera form a monophyletic grouping distinct from all other *Bacillales* families and genera is provided by our identification of 13 CSIs in 13 different proteins that are uniquely shared by species from all of the genera contained within this clade, but not by other *Bacillales* species. Thus, the identified CSIs in conjunction with the results from our comprehensive phylogenetic studies reliably demarcate a monophyletic grouping of species, which represent the emended family *Caryophanaceae*. The emended family *Caryophanaceae* now encompasses both the families *Planococcaceae* and *Caryophanaceae* and it rectifies the taxonomic anomaly that the family name *Planococcaceae* is a later heterotypic synonym of *Caryophanaceae* (Tindall, 2019).

**FIGURE 9:**
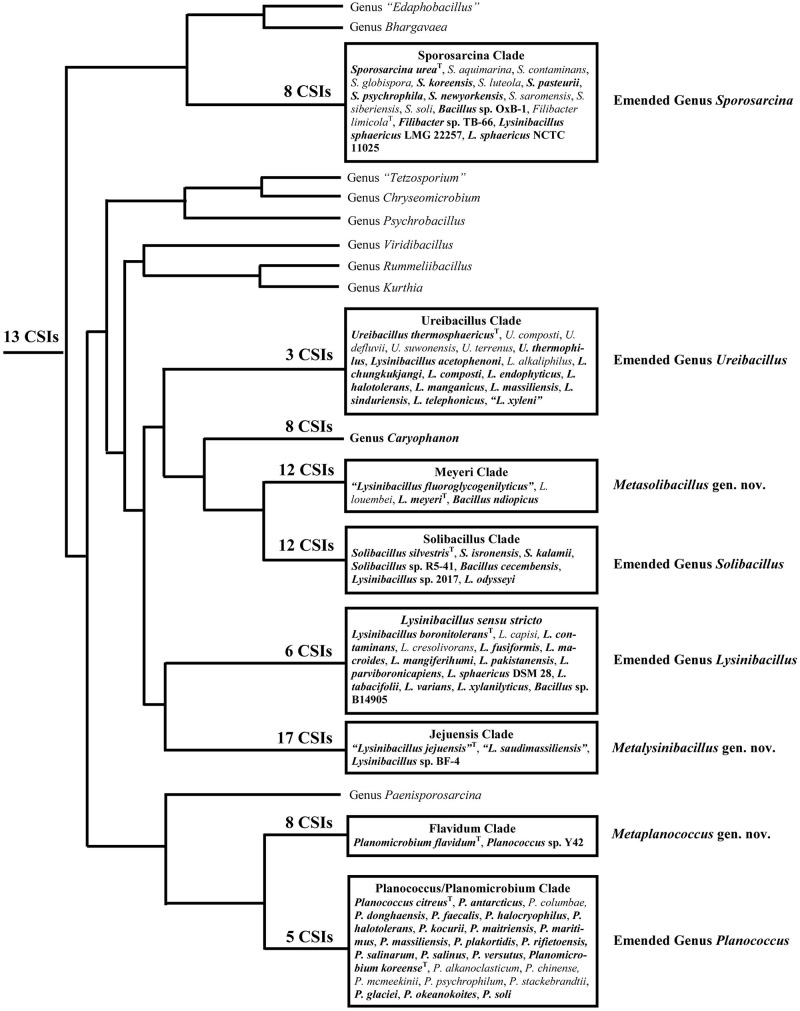
A conceptual diagram based on results obtained from phylogenetic studies, AAI similarity analysis, and several identified CSIs indicating the evolutionary relationships among the *Caryophanaceae* species. The total numbers of identified CSIs that are specifically shared by species from each of these clades are indicated at their respective nodes. All clades analyzed in this study are shown in boxes containing the species they comprise. Of these species, the ones in bold are genome-sequenced while the rest are placed within these clades based on our 16S rRNA analysis.

Within the emended family *Caryophanaceae*, in all phylogenetic trees based on genome sequences, 18 identical and distinct clades were consistently observed (labeled with square brackets in Figure 1 and Supplementary Figure 1). The distinctness of these species clades was also strongly supported by the results from our AAI analysis. More importantly, our identification of multiple highly specific molecular markers (i.e., CSIs) in important proteins that are uniquely shared by the members of these clades clearly distinguish the members of these clades from each other as well as all other *Bacillales* species. Molecular markers of this kind provide independent evidence that the species from these clades are specifically related to each other and they shared common ancestors exclusive of all other species. Figure 9 presents a summary diagram showing the species compositions of these clades as well as the numbers of distinct CSIs that were identified in this work. In addition to the genome sequenced species (shown in bold), this summary diagram also includes some additional species (non-bolded), which based on their branching in the 16S rRNA tree (Figure 2) are also a part of the indicated clades. Of these 18 clades, 10 clades were monophyletic groupings comprising of different species from known genera (viz. *Rummeliibacillus*, *Chryseomicrobium*, *Viridibacillus*, *Rummeliibacillus*, *Kurthia*, *Caryophanon*, *Psychrobacillus*, *Paenisporosarcina*, “*Edaphobacillus*,” and “*Tetzosporium*”). In contrast to these clades, the remaining eight clades were either comprised of species from multiple different genera or constituted novel species clades identified in the present work. These species clades were investigated in greater detail in this work and the taxonomic implications of these results are discussed below.

Before discussing the taxonomic implications of our results, it should be indicated that in our analyses of genome sequences, anomalous results were observed with genome sequences for two species viz. *P. quisquiliarum* (GenBank assembly accession: GCA_900109875.1) and *S. globispora* (GenBank assembly accession: GCA_001274725.1). In phylogenetic trees based on the deposited genome sequence for *P. quisquiliarum*, it branches separately from other *Paenisporosarcina* species, within a clade comprised of different genome-sequenced *Psychrobacillus* species, making both the genera *Paenisporosarcina* and *Psychrobacillus* polyphyletic. However, the 16S rRNA sequences from this genome exhibit only 94.24% sequence identity to the reference 16S rRNA sequence for the type strain of *P. quisquiliarum* (GenBank assembly accession: GCA_900109875.1), but >99.9% sequence similarity to the 16S rRNA sequence for the type strain of *Psychrobacillus psychrodurans*. Thus, we suspect that the deposited genome sequence for *P. quisquiliarum* (GenBank assembly accession: GCA_900109875.1) is closely related to *P. psychrodurans*, accounting for its anomalous branching in different protein trees (Figure 1 and Supplementary Figure 1). Similarly, in our protein trees, *S. globispora* branches separately from all other *Sporosarcina* species and deeply within the *Bacillaceae* species (Figure 1 and Supplementary Figure 1). The 16S rRNA sequences from this genome exhibit only 94.40% sequence identity to the reference 16S rRNA sequence for the type strain from *S. globispora* (GenBank assembly accession: GCA_001274725.1), indicating that the deposited genome sequence does not correspond to this species. As the sequences for both these species reliably group with the sequences of other species from these genera in the 16S rRNA trees, we have chosen to ignore the anomalous results obtained from their deposited genomes in our discussion. However, the genomes for the type strains of these two species should be sequenced again to confirm that the results obtained from the deposited genomes are anomalous.

As noted above, the work presented here allows reliable identification and demarcation of eight different species clades within the emended family *Caryophanaceae* that are comprised of either species from multiple different genera or novel species clades from the existing genera (Figure 9). Similar groupings of species from these genera have also been observed in earlier studies based on other types of analyses using a limited number of genome sequences as well as some chemotaxonomic characteristics and branching in 16S rRNA trees (Seiler et al., 2013; Shivaji et al., 2014; Xu et al., 2015; Maayer et al., 2019). The species from these clades are also observed to form distinct groupings in the Genome Taxonomy Database (GTDB) based on phylogenetic analyses of 120 ubiquitous single-copy proteins (Parks et al., 2018). Some characteristics of the species from these eight clades and their taxonomic implications are indicated below.

The genus *Lysinibacillus* is known to be highly polyphyletic (Kampfer et al., 2013; Xu et al., 2015; Gomez-Garzon et al., 2016; Mual et al., 2016), and in our work, species from this genus are seen forming six different clades. Of these clades, the clade marked *Lysinibacillus sensu stricto*, which is distinguished by six identified CSIs, contains the type species *L. boronitolerans* as well as 11 other *Lysinibacillus* species including the type strain for the important entomopathogenic bacteria *L. sphaericus*. One unnamed *Bacillus* sp. is also a part of this clade. As this clade is distinct from all other clades, we are proposing that the emended genus *Lysinibacillus* be restricted to only the species from this clade. Another clade, referred to here as the “Ureibacillus clade” is comprised of different species from the genus *Ureibacillus* as well 10 *Lysinibacillus* species. These species consistently group together in different phylogenetic trees including both protein sequences-based and 16S rRNA-based trees (Figures 1, 2 and Supplementary Figure 1). A specific grouping of different genome sequenced species from this clade is also supported by three specific CSIs that are exclusively shared by them. In contrast, no CSI was identified that is only shared by the *Lysinibacillus* species that are part of this clade. Hence, to bring taxonomic clarity to this clade of species, we are proposing the transfer of all 10 *Lysinibacillus* species which are part of this clade to the emended genus *Ureibacillus*. The name combinations proposing these transfers are listed in Table 5.

**TABLE 5:** Descriptions of the new combinations in the emended genus *Ureibacillus*.

**New name combination and etymology**	**Basonym**	**Description**	**Type strain**
*Ureibacillus acetophenoni* comb. nov. (a.ce.to.phe.no’ni. N.L. neut. n. *acetophenonum* acetophenone; N.L. gen. n. *acetophenoni* of/from acetophenone).	*Lysinibacillus acetophenoni* Azmatunnisa et al. 2015	The description of this taxon is as given by Azmatunnisa et al. (2015)	KCTC 13605^T^ (=NBRC 105754 = CCUG 57911 = DSM 23394 = JC23)
*Ureibacillus alkaliphilus* comb. nov. (al.ka.li’phi.lus. N.L. n. *alkali* (from Arabic article *al* the; Arabic n. *qaliy* ashes of saltwort) alkali; N.L. masc. adj. *philus* (from Gr. masc. adj. *philos*) friend, loving; N.L. masc. adj. *alkaliphilus* liking alkaline environments).	*Lysinibacillus alkaliphilus* Zhao et al. 2015	The description of this taxon is as given by Zhao et al. (2015)	OMN17^T^ (=DSM 28019 = CCTCC AB 2014073)
*Ureibacillus chungkukjangi* comb. nov. (chung.kuk.jan’gi. N.L. gen. n. *chungkukjangi*, of chungkukjang, a traditional Korean fermented food).	*Lysinibacillus chungkukjangi* Kim et al. 2013a	The description of this taxon is as given by Kim et al. (2013a)	NBRC 108948^T^ (=KACC 16626 = 2RL3-2)
*Ureibacillus composti* comb. nov. (com.pos’ti. N.L. gen. n. *composti*, of compost, from which the organism was isolated).	*Lysinibacillus composti* Hayat et al. 2014	The description of this taxon is as given by Hayat et al. (2014)	DSM 24785^T^ (=KCTC 13796 = NCCP-36 = JCM 18777)
*Ureibacillus endophyticus* comb. nov. (en.do.phy’ti.cus. Gr. pref. *endo* within; Gr. neut. n. *phyton* plant. N.L. masc. adj. *endophyticus* within plant, pertaining to the isolation of the type strain from plant tissues).	*Lysinibacillus endophyticus* Yu et al. 2017	The description of this taxon is as given by Yu et al. (2017)	C9^T^ (=CGMCC 1.15291 = DSM 100506)
*Ureibacillus halotolerans* comb. nov. (ha.lo.to’le.rans. Gr. masc. n. *hals*, *halos* salt; L. pres. part. *tolerans* tolerating; N.L. part. adj. *halotolerans* salt-tolerating, referring to the organism’s ability to tolerate high salt concentrations).	*Lysinibacillus halotolerans* Kong et al. 2014	The description of this taxon is as given by Kong et al. (2014)	ACCC 00718^T^ (=JCM 19611 = LAM612)
*Ureibacillus manganicus* comb. nov. (man.ga’ni.cus. N.L. neut. n. *manganum* manganese; L. suff. -*icus* suffix used with the sense of pertaining to; N.L. masc. adj. *manganicus* pertaining to manganese, referring to the isolation of the type strain from a manganese mining soil).	*Lysinibacillus manganicus* Liu et al. 2013	The description of this taxon is as given by Liu et al. (2013)	CCTCC AB 2012916^T^ (=Mn1-7 = DSM 26584)
*Ureibacillus massiliensis* comb. nov. (mas.si.li.en’sis. L. masc. adj. *massiliensis* of Massilia, the ancient Greek and Roman name for Marseille, France, where the type strain was isolated).	*Lysinibacillus massiliensis* (Glazunova et al. 2006) Jung et al. 2012	The description of this taxon is as given by Jung et al. (2012)	CCUG 49529^T^ (=CIP 108446 = 4400831)
*Ureibacillus sinduriensis* comb. nov. (sin.du.ri.en’sis. N.L. masc. adj. *sinduriensis* pertaining to the Sinduri, Republic of Korea, geographical origin of the type strain of the species).	*Lysinibacillus sinduriensis* Jung et al. 2012	The description of this taxon is as given by Jung et al. (2012)	KCTC 13296^T^ (=BLB-1 = JCM 15800)
*Ureibacillus telephonicus* comb. nov. (te.le.pho’ni.cus. N.L. neut. n. *telephonum*, telephone; L. suff. *-icus*, suffix used with the sense of pertaining to; N.L. masc. adj. *telephonicus*, pertaining to the telephone, the type strain was isolated from a cellular phone).	*Lysinibacillus telephonicus* Rahi et al. 2017	The description of this taxon is as given by Rahi et al. (2017)	S5H2222^T^ (=KACC 18714 = LMG 29294 = MCC 3065)

The “Meyeri” and “Jejuensis” clades are two new species clades identified in this work. These two clades are reliably distinguished from all other clades by different means including our identification of 12 and 17 novel CSIs, respectively, which are uniquely shared by the species from these clades. Of these two clades, the “Meyeri clade” is comprised of *B. ndiopicus* and two *Lysinibacillus* species, whereas the “Jejuensis clade” is comprised of two named and one unnamed *Lysinibacillus* species. As both these clades are novel clades, with no designated type species, we are proposing the transfer of species from the “Meyeri” and “Jejuensis” clades into two novel genera named *Metalysinibacillus* gen. nov. and *Metasolibacillus* gen. nov., respectively. The descriptions of these genera are provided below and the new name combinations proposing the transfer of species from these two clades into the two proposed genera are listed after the section “Discussion” and in Table 6, respectively. The “Solibacillus clade” demarcated by our studies not only contains all species from the genus *Solibacillus*, but also encompasses two *Lysinibacillus* species as well as *B. cecembensis.* This grouping is strongly supported by 12 CSIs that are uniquely shared by all seven species forming this clade. In light of the strong evidence supporting the distinctness of this clade, we are proposing the transfer of *B. cecembensis* and the *Lysinibacillus* species, which are observed to branch within this clade, to the emended genus *Solibacillus* forming a taxonomically homogeneous clade/genus. The name combinations proposing these transfers are listed in Table 6.

**TABLE 6:** Descriptions of the new combinations in *Metaplanococcus* gen. nov., *Metasolibacillus* gen. nov, emended genus *Solibacillus*, emended genus *Sporosarcina*, and emended genus *Planococcus*.

**New name combination and etymology**	**Basonym**	**Description**	**Type strain**
*Metaplanococcus flavidus* comb. nov. (type species of the genus *Metaplanococcus*) (fla’vi.dus. L. masc. adj. *flavidum* pale yellow).	*Planomicrobium flavidum* Jung et al. 2009	The description of this taxon is as given by Jung et al. (2009)	KCTC 13261^T^ (=ISL-41 = CCUG 56756 = DSM 27642)
*Metasolibacillus meyeri* comb. nov. (type species of the genus *Metasolibacillus*) (me’yer.i. N.L. gen. n. *meyeri* of Meyer, named in honor of Arthur Meyer, who, together with Ernst Neide in 1904, described the species *Bacillus sphaericus*, now *Lysinibacillus sphaericus*).	*Lysinibacillus meyeri* Seiler et al. 2013	The description of this taxon is as given by Seiler et al. (2013)	WS 4626^T^ (=LMG 26643 = DSM 25057)
*Metasolibacillus louembei* comb. nov. (lou.em′be.i. N.L. gen. n. *louembei* Louembe, named in honor of Professor Delphin Louembe from the Republic of the Congo for his substantial contribution to a better understanding of the microbial diversity of Congolese traditional fermented foods).	*Lysinibacillus louembei* Ouoba et al. 2015	The description of this taxon is as given by Ouoba et al. (2015)	DSM 25583^T^ (=NM73 = LMG 26837)
*Metasolibacillus ndiopicus* comb. nov. (n.dio.pi.cus. N.L. gen. n. *ndiopicus*, of Ndiop, the name of the Senegalese village where the man from whom strain FF3^T^ was cultivated lives).	*Bacillus ndiopicus* Lo et al. 2015	The description of this taxon is as given by Lo et al. (2015)	FF3^T^ (=CSUR P3025 = DSM 27837)
*Solibacillus cecembensis* comb. nov. (ce.cem.ben’sis. N.L. masc. adj. *cecembensis* arbitrary name derived from the acronym CCMB for the Centre for Cellular and Molecular Biology, where the type strain was characterized).	*Bacillus cecembensis* Reddy et al. 2008	The description of this taxon is as given by Reddy et al. (2008)	PN5^T^ (=MTCC 9127 = LMG 23935 = JCM 15113 = DSM 21993)
*Solibacillus odysseyi* comb. nov. (o.dys.se’yi. N.L. neut. n. *Odysseum* name of the spacecraft *Odyssey*; N.L. gen. n. *odysseyi* pertaining to the Mars Odyssey spacecraft, from which the organism was isolated).	*Lysinibacillus odysseyi* (La Duc et al. 2004) Jung et al. 2012	The description of this taxon is as given by Jung et al. (2012)	34hs-1^T^ (=ATCC PTA-4993 = NBRC 100172 = NRRL B-59274)
*Sporosarcina limcola* comb. nov. (li.mi′co.la. L. masc. n. *limus* mud; L. suff. *cola* (from L. masc. or fem. n. incola) dweller; N.L. fem. n. *limcola* mud dweller).	*Filibacter limcola* Maiden and Jones 1985	The description of this taxon is as given by Maiden and Jones (1985)	1SS1O1^T^ (= DSM 13886 = NCIMB 11923 = ATCC 43646)
*Planococcus alkanoclasticus* comb. nov. (al.kan.o.cla’sti.cus. N.L. neut. n. *alkanum* alkane; Gr. masc. adj. *clastos* broken; N.L. masc. adj. *alkanoclasticus* breaking alkanes).	*Planomicrobium alkanoclasticum* (Engelhardt et al. 2001) Dai et al. 2005	The description of this taxon is as given by Dai et al. (2005)	MAE2^T^ (=CIP 107718 = NCIMB 13489)
*Planococcus chinensis* comb. nov. (chin.en’sis. N.L. masc. adj. *chinensis* pertaining to China, where the type strain was isolated and studied).	*Planomicrobium chinense* Dai et al. 2005	The description of this taxon is as given by Dai et al. (2005)	AS 1.3454^T^ (=DX3-12 = JCM 12466 = DSM 17276)
*Planococcus glaciei* comb. nov. (gla.ci’e’i. L. gen. n. *glaciei* of ice, referring to the isolation source of the type strain, the China No. 1 glacier).	*Planomicrobium glaciei* Zhang et al. 2009	The description of this taxon is as given by Zhang et al. (2009)	JCM 15088^T^ (=0423 = CGMCC 1.6846 = DSM 24857)
*Planococcus koreensis* comb. nov. (ko.re.en’sis. N.L. masc. adj. *koreensis* referring to Korea).	*Planomicrobium koreense* Yoon et al. 2001a	The description of this taxon is as given by Yoon et al. (2001a)	JG07^T^ (=KCTC 3684 = JCM 10704)
*Planococcus mcmeekinii* comb. nov. (N.L. gen. n. mc.mee.kin’i.i. Named in honor of Thomas A. McMeekin, an Australian microbiologist who has studied Antarctic microorganisms).	*Planomicrobium mcmeekinii* (Junge et al. 1998) Yoon et al. 2001a	The description of this taxon is as given by Yoon et al. (2001a)	S23F2^T^ (=ATCC 700539 = CIP 105673 = DSM 13963)
*Planococcus psychrophilus* comb. nov. (psy.chro.phi’lus. Gr. masc. adj. *psychros* cold; N.L. masc. adj. *philus* (from Gr. masc. adj. *philos*) loving; N.L. masc. adj. *psychrophilus* cold-loving).	*Planomicrobium psychrophilum* (Reddy et al. 2002) Dai et al. 2005	The description of this taxon is as given by Dai et al. (2005)	DSM 14507^T^ (=MTCC 3812 = CMS 53or)
*Planococcus soli* comb. nov. (so’li. L. gen. n. *soli* of soil).	*Planomicrobium soli* Luo et al. 2014	The description of this taxon is as given by Luo et al. (2014)	CGMCC 1.12259^T^ (=XN13 = KCTC 33047)
*Planococcus stackebrandtii* comb. nov. (sta.cke.brand.ti′i. N.L. gen. n. *stackebrandtii* of Stackebrandt, to honor Erko Stackebrandt, a German microbiologist, for his valuable contributions to microbial taxonomy and molecular systematics).	*Planomicrobium stackebrandtii* (Mayilraj et al. 2005) Jung et al. 2009	The description of this taxon is as given by Jung et al. (2009)	JCM 12481^T^ (=K22-03 = DSM 16419 = MTCC 6226)

Two of the clades shown in Figures 1, 9 are comprised of species from the genera *Planococcus* and *Planomicrobium.* Of these two genera, the genus *Planomicrobium* was created in 2001 by the transfer of three *Planococcus* species on the basis of cell morphology and 16S rRNA-based phylogenetic analysis to this new genus (Yoon et al., 2001a). However, our analyses reveal that both these genera are polyphyletic, and their species are interspersed among one another within a larger clade consisting of all of the species from these two genera (see Figure 1 and Supplementary Figure 1). Within this large clade, the species *Planococcus flavidum* together with an unnamed *Planococcus* species form a deeper branching lineage (designated as the “Flavidum clade”) relative to the rest of the species from these two genera (designated as the “Planococcus/Planomicrobium clade”). In our work, while we have identified multiple CSIs that are specific for the species from the “Flavidum” or “Planococcus/Planomicrobium” clade, no CSI was identified that was specifically shared by the species from only the genus *Planococcus* or the genus *Planomicrobium.* To account for these results and to clarify the taxonomy of species from these two genera, we are proposing that all of the species from these two genera, which comprise the “Planococcus/Planomicrobium clade” be united within the emended genus *Planococcus*, which has priority [Rule 24b (1)] over the genus *Planomicrobium* (Migula, 1894; Skerman et al., 1980; Nakagawa et al., 1996; Yoon et al., 2001a, 2010; Parker et al., 2019). Further, due to the phylogenetic and molecular distinctness of the “Flavidum clade,” we are proposing the transfer of species from this clade into a new genus named *Metaplanococcus* gen. nov. The name combinations proposing these taxonomic changes are listed in Table 6. After this work was completed, a new genus *Indiicoccus* containing the sole species *I. explosivorum*, which branches peripherally to the *Planococcus* clade has also been described (Pal et al., 2019). It is unclear at present whether this new species shares any of the CSIs that are specific for members of the genera *Planococcus* or *Metaplanococcus*.

Lastly, the clade marked as the “Sporosarcina clade” includes within it all of the species from the genus *Sporosarcina* along with *Bacillus* sp. OxB-1, two strains of *L. sphaericus* and *Filibacter* sp. TB-66. In the 16S rRNA tree (Figure 2), the species *Filibacter limicola*, which is the type species of the genus *Filibacter*, also reliably branches with the other *Sporosarcina* species. To clarify the taxonomy of this clade, we are proposing that all of the species which are part of the “Sporosarcina clade” should be transferred to the emended genus *Sporosarcina*, which has priority over the genus *Filibacte*r (Kluyver and Van Niel, 1936; Skerman et al., 1980; Maiden and Jones, 1985; Yoon et al., 2001b). As the type strain of the species *L. sphaericus* is a part of the genus *Lysinibacillus*, the two *L. sphaericus* strains which branch within the “Sporosarcina clade” are described as two new species viz. *Sporosarcina sphaericus* sp. nov., and *Sporosarcina urealyticus* sp. nov.

Based on the results presented here, we have developed a reliable and coherent phylogenetic framework for understanding the evolutionary relationships as well as a classification scheme for the members of the family *Caryophanaceae*. In the proposed classification scheme, the family *Caryophanaceae* and the different monophyletic clades (genera) that form this family are reliably delineated both by means of extensive phylogenetic analyses as well as by our identification of large numbers of highly specific molecular markers (CSIs) that are specifically shared by the members of these clades. It is important to note that the CSIs described in this study for the family *Caryophanaceae* and for its different genera have several important applications. Earlier work on CSIs has shown that they exhibit a high degree of predictive ability to be found in other members of the group they represent (whose genome sequences are not yet available or in species which are not yet discovered) (Gao and Gupta, 2012; Adeolu and Gupta, 2014; Bhandari and Gupta, 2014; Sawana et al., 2014; Adeolu et al., 2016; Gupta et al., 2016, 2018; Dobritsa et al., 2017; Patel and Gupta, 2018; Gupta, 2019). Hence, the presence or absence of these CSIs in the genome sequences of other species (including unnamed species) can be used to determine if they belong to the family *Caryophanaceae* or any of the other genera for which CSIs are described in this study. Additionally, earlier studies on CSIs provide evidence that these molecular characteristics are functionally important for the group of organisms for which they are specific (Singh and Gupta, 2009; Khadka and Gupta, 2017). Hence, genetic and biochemical studies on understanding the functional significance of these CSIs are expected to lead to the identification of novel biochemical and/or other characteristics that are distinctive properties of the described groups of bacteria.

The emended descriptions of the family *Caryophanaceae* as well as the descriptions of various novel and emended species and genera are given below. The new name combinations for species that results from the proposed taxonomic changes are listed in Tables 5, 6.

### Emended Description of the Family *Caryophanaceae* Peshkoff 1939 (Approved Lists 1980)

(Ca.ry.o.pha.na.ce’ae. N.L. neut. n. *Caryophanon*, type genus of the family: suff. -*aceae*, ending to denote a family; N.L. fem. pl. n. *Caryophanaceae*, the *Caryophanon* family).

The family *Caryophanaceae* is circumscribed here based on the monophyletic grouping of different taxa from this family in phylogenetic trees based on multiple large datasets of protein sequences and conserved signatures indels in multiple proteins listed below that are specifically shared by the members of this family. The emended family *Caryophanaceae* presently contains the following genera: *Caryophanon*, *Bhargavaea*, “*Chryseomicrobium*,” “*Edaphobacillus*,” *Indii- coccus*, *Kurthia*, *Lysinibacillus*, *Metalysinibacillus*, *Metasoli- bacillus*, *Metaplanococcus*, *Paenisporosarcina*, *Planococcus*, *Psychrobacillus*, *Rummelibacillus*, *Solibacillus*, *Sporosarcina*, “*Tetzosporium*,” *Ureibacillus*, and *Virdibacillus*. As both *Caryophanon* and *Planococcus* are part of the same family and, according to the Rule 24b (1) of the Code (Parker et al., 2019), the name *Caryophanaceae* (Peshkoff, 1939) has priority over the name *Planococcaceae* (Krasil’nikov, 1949), the family name *Caryophanaceae* is used for the description of this family. As a result, the name *Planococcaceae* is now a later heterotypic synonym for the *Caryophanaceae*. Cells from members of the family *Caryophanaceae* can be cocci or rods, sometimes forming filaments or trichomes. Most species are strictly aerobic heterotrophs, although some are also facultatively aerobes. Cells are generally motile by flagella or gliding and they may or may not form endospores. Most species are catalase-positive and oxidase positive or negative. Members of this family can be reliably distinguished from all other *Firmicutes* genera based on the shared presence of CSIs described in this work in all or most of the following proteins: phenylalanine–tRNA ligase subunit alpha, chaperonin GroEL, ribosome maturation factor RimP, BrxA/BrxB family bacilliredoxin, RNA methyltransferase, Rhomboid family intramembrane serine protease, ATP-dependent Clp protease ATP-binding subunit, DNA-directed RNA polymerase subunit beta, Chorismate synthase, Stage IV sporulation protein A, peptidase, KinB-signaling pathway activation protein, and DUF423 domain-containing protein.

Type genus: *Caryophanon* Peshkoff 1939 (Approved Lists 1980).

### Emended Description of the Genus *Caryophanon* Peshkoff 1939 (Approved Lists 1980)

*Caryophanon*. (Ca.ry.o’pha.non. Gr. neut. n. *karyon*, nut, kernel, nucleus; Gr. masc.adj. *phaneros*, bright, conspicuous; N.L. neut. n. *Caryophanon*, that which has a conspicuous nucleus).

The emended genus *Caryophanon* contains the type species *Caryophanon latum*. Cells are Gram-positive, aerobic, and motile by means of peritrichous flagella. The members of this genus form a monophyletic clade in 16S rRNA gene tree and phylogenetic trees based on multiple large datasets of protein sequences. Members of this genus can be reliably distinguished from other genera within the family *Caryophanaceae* based on the shared presence of CSIs described in this work in all or most of the following proteins: DNA-directed RNA polymerase subunit beta, peroxide-responsive transcriptional repressor PerR, ADP-forming succinate-CoA ligase subunit beta, tRNA (*N*(6)-L-threonylcarbamoyl adenosine (37)-C(2)-methylthiotransferase MtaB, magnesium transporter, dephospho-CoA kinase, ATP synthase subunit I, and bifunctional DNA-formamidopyrimidine glycosylase/DNA-(apurinic or apyrimidinic site) lyase.

Type species: *Caryophanon latum* Peshkoff 1939 (Approved Lists 1980).

### Emended Description of the Genus *Lysinibacillus* Ahmed et al. 2007 emend. Jung et al. 2012

*Lysinibacillus* (Ly.si.ni.ba.cil’lus. N.L. neut. n. *lysinum* lysine; L. masc. n. *bacillus* a small staff or rod; N.L. masc. n. *Lysinibacillus* lysine bacillus, referring to the presence of the Lys–Asp type of peptidoglycan in the cell wall).

The emended genus *Lysinibacillus* contains the type species *L. boronitolerans.* The members of this genus are generally motile, rod-shaped cells that produce ellipsoidal or spherical endospores which lie terminally in a swollen sporangium. Cell-wall peptidoglycan of the studied species has been reported to contain lysine and aspartic acid (Ahmed et al., 2007). Members of this emended genus form a monophyletic clade in a 16S rRNA gene tree and trees based on multiple large datasets of protein sequences. Further, members of this genus can be reliably distinguished from all other genera within the family *Caryophanaceae* based on the shared presence of CSIs described in this work in all or most of the following proteins: bacillithiol biosynthesis deacetylase BshB2, PIN/TRAM domain-containing protein, flagellar assembly protein FliH, PDZ domain-containing protein, TrkH family potassium uptake protein, and D-alanyl-D-alanine carboxypeptidase.

Type species: *Lysinibacillus boronitolerans* Ahmed et al. 2007.

### Emended Description of the Genus *Ureibacillus* (Andersson et al. 1996) Fortina et al. 2001

*Ureibacillus* (Ur.e.i. ba.cil’lus. N. L. fem. n. *urea* urea; N. L. masc. n. *Bacillus* a bacterial genus; N.L. masc. n. *Ureibacillus* a ureolytic *Bacillus*-like organism).

The emended genus *Ureibacillus* contains the type species *U. thermosphaericus*, which was originally described as *Bacillus thermosphaericus* (Andersson et al., 1996). The members of this genus are generally motile, rod-shaped cells, and some species are known to produce ellipsoidal or spherical endospores which lie terminally in a swollen sporangium (Fortina et al., 2001). The genus includes some thermophilic bacteria. The members of this genus form a monophyletic clade in a 16S rRNA gene tree, and trees based on multiple large datasets of protein sequences. Members of this genus can be reliably distinguished from other genera within the family *Caryophanaceae* based on the shared presence of CSIs described in this work in all or most of the following proteins: MFS transporter, EamA family transporter, and DNA internalization-related competence protein ComEC/Rec2. Description of a new species that is part of this genus is provided below; new name combinations for some other species that are part of this emended genus are described in Table 5.

Type species: *Ureibacillus thermosphaericus* Fortina et al. 2001.

### Description of *Ureibacillus xyleni* sp. nov.

(xy.le’.ni. N.L. neut. n. *xylenum*, xylene; N.L. gen. n. *xyleni*: of/from xylene).

The description of this taxon is as given by Begum et al. (2016) for “*Lysinibacillus xyleni*.” The type strain is NBRC 105753^T^ (=DSM 23555 = KCTC 13604 = CCUG 57912 = JC22).

### Emended Description of the Genus *Solibacillus* Krishnamurthi et al., 2009 emend. Mual et al., 2016

*Solibacillus* (So.li.ba.cil’lus. L. neut. n. *solum* soil; N.L. masc. n. *Bacillus* a bacterial genus; N.L. masc. n. *Solibacillus* a *Bacillus*-like organism isolated from soil).

The emended genus *Solibacillus* contains the type species *S. silvestris.* The members of this genus are rod-shaped cells staining generally Gram-positive. Some species are reported to form round endospores terminally in swollen sporangia (Krishnamurthi et al., 2009). The members of this genus form a monophyletic clade in phylogenetic trees based on multiple large datasets of protein sequences. Members of this genus can be reliably distinguished from other genera within the family *Caryophanaceae* based on the shared presence of CSIs described in this work in all or most of the following proteins: Flagellar hook–basal body protein (2 indels), aminodeoxychorismate lyase, VOC family protein, DNA topoisomerase IV subunit A, DegV family protein, helicase–exonuclease AddAB subunit AddB, multidrug resistance efflux transporter family protein, heme-dependent peroxidase, methionine ABC transporter ATP-binding protein, tRNA 4-thiouridine(8) synthase ThiI, and AAA family ATPase. New name combinations for some species that are part of the emended genus are described in Table 6.

Type species: *Solibacillus silvestris* (Rheims et al., 1999) Krishnamurthi et al., 2009.

### Emended Description of the Genus *Sporosarcina* Kluyver and Van Niel 1936 (Approved Lists 1980) emend. Yoon et al. 2001b

*Sporosarcina*. (Spo.ro.sar.ci’na. Gr. fem. n. *spora* a seed, a spore; N.L. fem. n. *Sarcina* generic name; N.L. fem. n. *Sporosarcina* spore-forming *Sarcina*).

The emended genus *Sporosarcina* contains the type species *S. ureae.* Cells exhibit Gram-positive or Gram-variable staining. Studied species form round endospores and are generally motile. Facultatively anaerobic or strictly aerobic. The members of this genus form a monophyletic clade in 16S rRNA gene tree and phylogenetic trees based on multiple large datasets of protein sequences. Members of this genus can be reliably distinguished from other genera within the family *Caryophanaceae* based on the shared presence of CSIs described in this work in all or most of the following proteins: aspartate–tRNA ligase, A/G-specific adenine glycosylase, thymidylate synthase, RDD family protein, DEAD/DEAH box helicase, membrane protein insase YidC, cytochrome b6, and a hypothetical protein (accession no. WP_083035866).

Type species: *Sporosarcina ureae* (Beijerinck, 1901) Kluyver and Van Niel 1936 (Approved Lists 1980).

### Description of *Sporosarcina sphaerica* sp. nov.

(sphae’ri.ca. L. fem. adj. *sphaerica* spherical).

The type strain for this species was isolated from human lung by A.M.R. Mackenzie (Southampton General Hospital) in 1975 and characterized as *Lysinibacillus sphaericus* NCTC 11025. This strain consistently branches within the genus *Sporosarcina*, distinctly from all other *Lysinibacillus* species. This strain also shares all of the conserved indels that are specific for the genus *Sporosarcina*, leading to its assignment as a novel species within this genus. The type strain for this species is NCTC 11025.

### Description of *Sporosarcina ureilytica* sp. nov.

(u.re.i.ly’ti.ca. N.L. fem. n. *urea*, urea; N.L. masc. adj. *lyticus* from Gr. masc. adj. *lytikos*, able to loosen, able to dissolve; N.L. fem. adj. ureilytica, urea dissolving).

The type strain for this Gram-positive species was isolated by Dick (2004) and originally described as a *Bacillus sphaericus* strain. This strain was transferred along with other *B. sphaericus* strains to the genus *Lysinibacillus* when this genus was created (Ahmed et al., 2007). This strain branches reliably within the genus *Sporosarcina* and shares all conserved indels specific for this genus, leading to its assignment as a novel *Sporosarcina* species. The type strain for this species is LMG 22257.

### Emended Description of the Genus *Planococcus* Migula 1894 (Approved Lists 1980) emend. Yoon et al. 2010

*Planococcus*. (Plan.o.coc’cus. Gr. masc. n. *planes*, a wanderer; N.L. masc. n. *coccus* (from Gr. masc. n. *kokkos*, grain, seed), coccus; N.L. masc. n. *Planococcus*, motile coccus).

The emended genus *Planococcus* contains the type species *P. citreus* and it encompasses most of the species from the genus *Planomicrobium*. Cells exhibit Gram-positive or Gram-variable staining and they are cocci or short rods and generally motile. The members of this genus form a monophyletic clade in a 16S rRNA gene tree and phylogenetic trees based on multiple large datasets of protein sequences. Members of this genus can be reliably distinguished from other genera within the family *Caryophanaceae* based on the shared presence of conserved signatures indels described in this work in all or most of the following proteins: penicillin-binding protein 2, hypothetical protein (WP_065528121), NADPH-dependent 7-cyano-7-deazaguanine reductase QueF, ACT domain-containing protein, and methylmalonyl-CoA mutase.

Type species: *Planococcus citreus* Migula 1894 (Approved Lists 1980).

### Description of *Metasolibacillus* gen. nov.

*Metasolibacillus* (Me.ta.so.li.ba.cil’lus. Gr. adv. *meta* besides; N.L. masc. n. *Solibacillus* a bacterial genus name; N.L. masc. n. *Metasolibacillus* a genus besides *Solibacillus*).

Motile, rod-shaped, endospores forming cells exhibit Gram-positive staining. Grow aerobically in the range of 10–45°C, with optimal growth at 30–37°C. Studied species are reported to be positive for catalase and Voges–Proskauer tests. Species from this genus form a monophyletic clade in phylogenetic trees based on multiple large datasets of protein sequences. Members of this genus can be reliably distinguished from other genera within the family *Caryophanaceae* based on the shared presence of CSIs described in this work in all or most of the following proteins: DUF456 domain-containing protein, toxic anion resistance protein, undecaprenyldiphospho-muramoylpentapeptide beta-*N*-acetylglucosaminyltransferase, c-type cytochrome biogenesis protein CcsB, thiol-disulfide oxidoreductase ResA, hypothetical protein (accession no. WP_066164326), hypothetical protein (accession no. WP_107942795), Arginase, preprotein translocase subunit SecY, ATP-binding cassette domain-containing protein, purine permease, and thiol-disulfide oxidoreductase ResA.

Type species: *Metasolibacillus meyeri* comb. nov.

### Description of *Metasolibacillus fluoroglycofenilyticus* sp. nov.

(flu.o.ro.gly.co.fe.ni.ly’ti.cus. N.L. neut. n. *fluoroglycofenum*, fluoroglycofen; N.L. masc. adj. *lyticus* (from Gr. masc. adj. *lutikos*) able to dissolve; N.L. masc. adj. *fluoroglycofenilyticus*, fluoroglycofen degrading).

The description of this taxon is as given by Cheng et al. (2015) for “*Lysinibacillus fluoroglycofenilyticus.*” The type strain is cmg86^T^ (=KCTC 33183 = CCTCC AB 2013247).

### Description of *Metalysinibacillus* gen. nov.

*Metalysinibacillus* (Me.ta.ly.si.ni.ba.cil’lus. Gr. adv. *meta* besides; N.L. masc. n. *Lysinibacillus* a bacterial genus name; N.L. masc. n. *Metalysinibacillus* a genus besides *Lysinibacillus*).

Gram-positive, rod-shaped cells able form endospores. Some species exhibit positive catalase and Voges–Proskauer tests. Grow optimally under aerobic condition in the temperature range of 30–37°C. Species from this genus form a monophyletic clade in phylogenetic trees based on multiple large datasets of protein sequences. Members of this genus can be reliably distinguished from other genera within the family *Caryophanaceae* based on the shared presence of CSIs described in this work in all or most of the following proteins: arginine-binding extracellular protein ArtP precursor, oxygen-independent coproporphyrinogen III oxidase, putative hydrolase MhqD, helix-turn-helix transcriptional regulator, tRNA preQ1(34) *S*-adenosylmethionine ribosyltransferase-isomerase QueA, DNA primase, FMN reductase (NADPH), UvrABC system protein C, sensor histidine kinase YycG, hypothetical protein BN1050_02162, ribonuclease Y, hypothetical protein BN1050_01309, cell division protein FtsA, ABC transporter ATP-binding protein YtrB, cysteine–tRNA ligase, coproporphyrinogen III oxidase, and PBP1A family penicillin-binding protein.

Type species: *Metalysinibacillus jejuensis*.

### Description of *Metalysinibacillus jejuensis* sp. nov.

(je.ju.en’sis. N.L. masc. adj. *jejuensis*, referring to Jeju Island in the Republic of Korea, where the type strain was isolated).

The description of this taxon is as given by Kim et al. (2013b) for “*Lysinibacillus jejuensis.*” The type strain is DSM 28310^T^ (=N2-5 = KCTC 13837).

### Description of *Metalysinibacillus saudimassiliensis* sp. nov.

(sau.di.mas.si.li.en’sis. Arab. n. *as-Sa*’*udiyah*, name of Saudi Arabia: L. masc. adj. *massiliensis* pertaining to Marseille; N.L. fem. adj. *saudimassiliensis*, referring to Saudi Arabia and Marseille).

The description of this taxon is as given by Papadioti et al. (2017) for “*Lysinibacillus saudimassiliensis.*” The type strain is 13S34_air^T^ (=CSUR P1222).

### Description of *Metaplanococcus* gen. nov.

*Metaplanococcus* (Me.ta. pla.no.coc’cus. Gr. adv. *meta* besides; N.L. masc. n. *Planococcus*, a bacterial genus name; N.L. masc. n. *Metaplanococcus*, a genus besides *Planococcus*).

Cells are Gram-positive to Gram-variable and cocci or short rods (0.4–0.8 × 0.4–1.6 μm); motile by means of a single polar flagellum. Other phenotypic characteristics of this genus are as described by Jung et al. (2009) for *P. flavidum*. The members of this genus form a monophyletic clade adjoining to the genus *Planococcus* in phylogenetic trees constructed based on multiple large datasets of protein sequences. Members of this genus can be reliably distinguished from the members of genus *Planococcus* and other *Caryophanaceae* genera based on the shared presence of CSIs described in this work in all or most of the following proteins: ABC transporter substrate-binding protein, methionine–tRNA ligase, MetQ/NlpA family ABC transporter substrate-binding protein, ABC transporter permease spore protease YyaC, *N*-acetyl-alpha-D-glucosaminyl L-malate synthase BshA, orotidine-5′-phosphate decarboxylase, and phospho-*N*-acetylmuramoyl-pentapeptide-transferase.

Type species: *Metaplanococcus flavidus* comb. nov.

## Data Availability Statement

The raw data supporting the conclusions of this article will be made available by the authors, without undue reservation, to any qualified researcher.

## Author Contributions

RG obtained funding for this work, planned and supervised the entire study, carried out the construction of phlylogenetic trees, helped in the creation of sequence alignments and identification of CSIs, and wrote large sections of the manuscript and finalized it. SP was primarily responsible for the identification of CSIs from sequence alignments, determining the specificity of the CSIs, formatting of different figures and tables for publications, and in the writing of a draft manuscript.

## Conflict of Interest

The authors declare that the research was conducted in the absence of any commercial or financial relationships that could be construed as a potential conflict of interest.

## Footnotes

1 https://www.namesforlife.com/

2 http://www.ncbi.nlm.nih.gov/genome

3 http://www.ncbi.nlm.nih.gov/genome
