# Supplementary material for: Robust Demarcation of the Family Caryophanaceae (Planococcaceae) and Its Different Genera Including Three Novel Genera Based on Phylogenomics and Highly Specific Molecular Signatures
Source: Front Microbiol. 2020 Jan 14;10:2821. doi: 10.3389/fmicb.2019.02821 (PMC6971209; doi:10.3389/fmicb.2019.02821)
Supplement: Supplementary file 1 [file Data_Sheet_1.PDF]

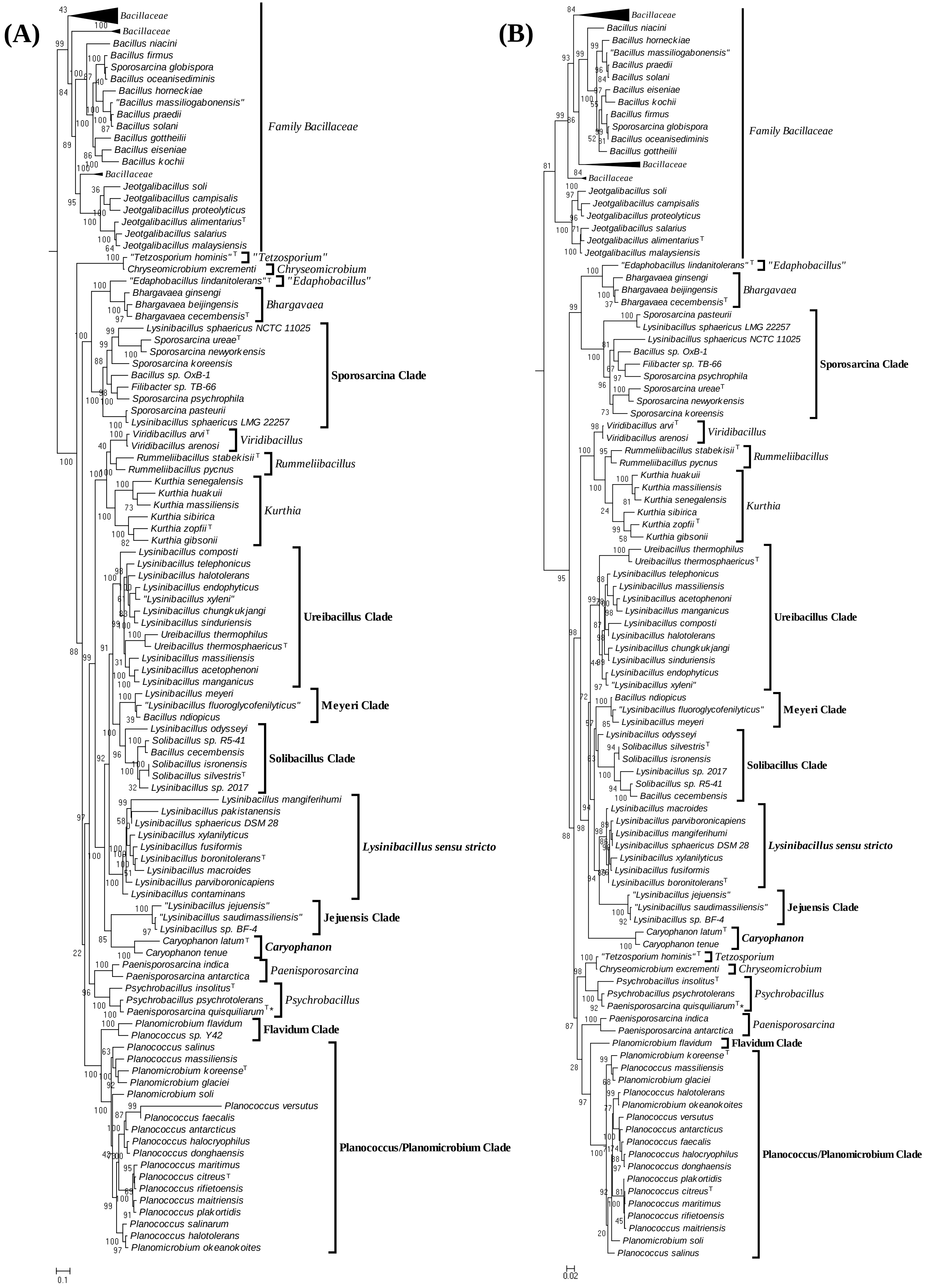
(A)
Bacillaceae
Bacillaceae
Bacillus niacini
Bacillus firmus
Sporosarcina globispora
Bacillus oceanisediminis
Bacillus horneckiae
"Bacillus massiliogabonensis"
Bacillus praedii
Bacillus solani
Bacillus gottheilii
Bacillus eiseniae
Bacillus kochii
Bacillaceae
Jeotgalibacillus soli
Jeotgalibacillus campisalis
Jeotgalibacillus proteolyticus
Jeotgalibacillus alimentarius T
Jeotgalibacillus salarius
Jeotgalibacillus malaysiensis
Family Bacillaceae
"Tetzosporium hominis" T
"Tetzosporium"
Chryseomicrobium excrementi
Chryseomicrobium
"Edaphobacillus lindanitolerans" T
"Edaphobacillus"
Bhargavaea ginsengi
Bhargavaea beijingensis
Bhargavaea cecembensis T
Bhargavaea
Lysinibacillus sphaericus NCTC 11025
Sporosarcina ureae T
Sporosarcina newyorkensis
Sporosarcina koreensis
Bacillus sp. OxB-1
Filibacter sp. TB-66
Sporosarcina psychrophila
Sporosarcina pasteurii
Lysinibacillus sphaericus LMG 22257
Sporosarcina Clade
Viridibacillus arvi T
Viridibacillus arenosi
Viridibacillus
Rummeliibacillus stabekisii T
Rummeliibacillus pycnus
Rummeliibacillus
Kurthia senegalensis
Kurthia huakuii
Kurthia massiliensis
Kurthia sibirica
Kurthia zopfii T
Kurthia gibsonii
Kurthia
Lysinibacillus composti
Lysinibacillus telephonicus
Lysinibacillus halotolerans
Lysinibacillus endophyticus
"Lysinibacillus xyleni"
Lysinibacillus chungkukjangi
Lysinibacillus sinduriensis
Ureibacillus thermophilus
Ureibacillus thermosphaericus T
Lysinibacillus massiliensis
Lysinibacillus acetophenoni
Lysinibacillus manganicus
Ureibacillus Clade
Lysinibacillus meyeri
"Lysinibacillus fluoroglycofenilyticus"
Bacillus ndiopicus
Meyeri Clade
Lysinibacillus odysseyi
Solibacillus sp. R5-41
Bacillus cecembensis
Solibacillus isronensis
Solibacillus silvestris T
Lysinibacillus sp. 2017
Solibacillus Clade
Lysinibacillus mangiferihumi
Lysinibacillus pakistanensis
Lysinibacillus sphaericus DSM 28
Lysinibacillus xylanilyticus
Lysinibacillus fusiformis
Lysinibacillus boronitolerans T
Lysinibacillus macroides
Lysinibacillus parviboronicapiens
Lysinibacillus contaminans
Lysinibacillus sensu stricto
"Lysinibacillus jejuensis"
"Lysinibacillus saudimassiliensis"
Lysinibacillus sp. BF-4
Jejuensis Clade
Caryophanon latum T
Caryophanon tenue
Caryophanon
Paenisporosarcina indica
Paenisporosarcina antarctica
Paenisporosarcina
Psychrobacillus insolitus T
Psychrobacillus psychrotolerans
Paenisporosarcina quisquiliarum T*
Psychrobacillus
Planomicrobium flavidum
Planococcus sp. Y42
Flavidum Clade
Planococcus salinus
Planococcus massiliensis
Planomicrobium koreense T
Planomicrobium glaciei
Planomicrobium soli
Planococcus versutus
Planococcus faecalis
Planococcus antarcticus
Planococcus halocryophilus
Planococcus donghaensis
Planococcus maritimus
Planococcus citreus T
Planococcus rifietoensis
Planococcus maitriensis
Planococcus plakortidis
Planococcus salinarum
Planococcus halotolerans
Planomicrobium okeanokoites
Planococcus/Planomicrobium Clade
0.1
(B)
Bacillaceae
Bacillus niacini
Bacillus horneckiae
"Bacillus massiliogabonensis"
Bacillus praedii
Bacillus solani
Bacillus eiseniae
Bacillus kochii
Bacillus firmus
Sporosarcina globispora
Bacillus oceanisediminis
Bacillus gottheilii
Bacillaceae
Bacillaceae
Jeotgalibacillus soli
Jeotgalibacillus campisalis
Jeotgalibacillus proteolyticus
Jeotgalibacillus salarius
Jeotgalibacillus alimentarius T
Jeotgalibacillus malaysiensis
Family Bacillaceae
"Edaphobacillus lindanitolerans" T
"Edaphobacillus"
Bhargavaea ginsengi
Bhargavaea beijingensis
Bhargavaea cecembensis T
Bhargavaea
Sporosarcina pasteurii
Lysinibacillus sphaericus LMG 22257
Lysinibacillus sphaericus NCTC 11025
Bacillus sp. OxB-1
Filibacter sp. TB-66
Sporosarcina psychrophila
Sporosarcina ureae T
Sporosarcina newyorkensis
Sporosarcina koreensis
Sporosarcina Clade
Viridibacillus arvi T
Viridibacillus arenosi
Viridibacillus
Rummeliibacillus stabekisii T
Rummeliibacillus pycnus
Rummeliibacillus
Kurthia huakuii
Kurthia massiliensis
Kurthia senegalensis
Kurthia sibirica
Kurthia zopfii T
Kurthia gibsonii
Kurthia
Ureibacillus thermophilus
Ureibacillus thermosphaericus T
Lysinibacillus telephonicus
Lysinibacillus massiliensis
Lysinibacillus acetophenoni
Lysinibacillus manganicus
Lysinibacillus composti
Lysinibacillus halotolerans
Lysinibacillus chungkukjangi
Lysinibacillus sinduriensis
Lysinibacillus endophyticus
"Lysinibacillus xyleni"
Ureibacillus Clade
Bacillus ndiopicus
"Lysinibacillus fluoroglycofenilyticus"
Lysinibacillus meyeri
Meyeri Clade
Lysinibacillus odysseyi
Solibacillus silvestris T
Solibacillus isronensis
Lysinibacillus sp. 2017
Solibacillus sp. R5-41
Bacillus cecembensis
Solibacillus Clade
Lysinibacillus macroides
Lysinibacillus parviboronicapiens
Lysinibacillus mangiferihumi
Lysinibacillus sphaericus DSM 28
Lysinibacillus xylanilyticus
Lysinibacillus fusiformis
Lysinibacillus boronitolerans T
Lysinibacillus sensu stricto
"Lysinibacillus jejuensis"
"Lysinibacillus saudimassiliensis"
Lysinibacillus sp. BF-4
Jejuensis Clade
Caryophanon latum T
Caryophanon tenue
Caryophanon
"Tetzosporium hominis" T
Tetzosporium
Chryseomicrobium excrementi
Chryseomicrobium
Psychrobacillus insolitus T
Psychrobacillus psychrotolerans
Paenisporosarcina quisquiliarum T*
Psychrobacillus
Paenisporosarcina indica
Paenisporosarcina antarctica
Paenisporosarcina
Planomicrobium flavidum
Flavidum Clade
Planomicrobium koreense T
Planococcus massiliensis
Planomicrobium glaciei
Planococcus halotolerans
Planomicrobium okeanokoites
Planococcus versutus
Planococcus antarcticus
Planococcus faecalis
Planococcus halocryophilus
Planococcus donghaensis
Planococcus plakortidis
Planococcus citreus T
Planococcus maritimus
Planococcus rifietoensis
Planococcus maitriensis
Planomicrobium soli
Planococcus salinus
Planococcus/Planomicrobium Clade
0.02

Supplemental Figure 1

Maximum likelihood phylogenetic trees for 124 genome sequenced members of the entire family "*Planococcaceae*" and some representative members of the family "*Bacillaceae*" based on (A) concatenated protein sequences of the DNA helicase II and DNA polymerase I (Uvrd-PolA) proteins, and (B) concatenated protein sequences of the major subunits of the RNA polymerase (RpoB-RpoC) protein. Both trees were rooted using genome sequences of *Streptococcus pyogenes*, *Streptococcus mitis*, *Lactococcus piscium* and *Lactococcus lactis* (not shown here). SH-like statistical support values are indicated at each branch node. The clades analyzed in this study are labelled in bold. The scale bars at the bottom represent 0.1 and 0.02 changes per amino acid position respectively for each tree.

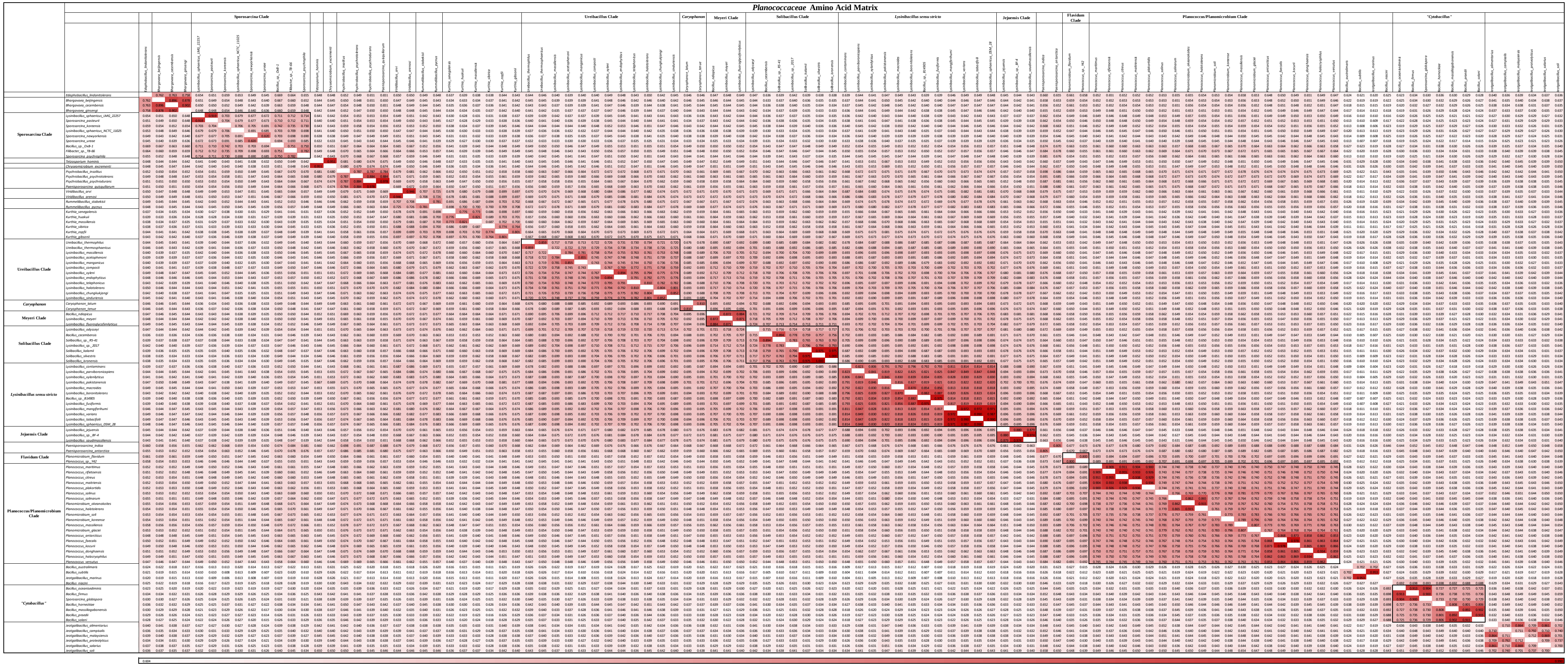

Supplemental Figure 2

A matrix depicting the percentage of average amino acid identity (AAI) shared by members of the family *Planococcaceae* based on a concatenated sequence alignment of 819 concatenated core protein families. Darker shading of red is indicative of greater AAI across genome pairs.

	Species	Accession	159	209
	Kurthia gibsonii	WP_121176350	FYISEEILMRTHTSPVQARTMEEKKGS	SIRIICPGKVFRRDNDDATHSHQF
	Kurthia huakuii	WP_029499406	----------------------KAQ-K	P-----------------------
	Kurthia massiliensis	WP_010288230	----------------------KAQ-K	P-----------------------
	Kurthia senegalensis	WP_010303700	----------------------KAE-K	P-K---------------------
	Kurthia zopfii	STX10257	------------------------D-A	--------------S---------
	Kurthia sp. 11kri321	WP_068457291	---------------------------	------------------------
	Kurthia sibirica	WP_109305305	------T-L-------------A---E	A-K-----------S---------
	Lysinibacillus contaminans	WP_053582524	--------L-------------A---E	------------------------
	Lysinibacillus fusiformis	WP_025114522	--------L-------------A---E	------------------------
	Lysinibacillus jejuensis	WP_108307641	--------L-------------K-E-A	NV------------S---------
	Lysinibacillus macroides	WP_053996413	--------L-------------A---E	A-----------------------
	Lysinibacillus odysseyi	WP_036156320	--------L-------------A---E	P-------------T---------
	Lysinibacillus parviboronicapiens	WP_107923435	--------L-------------A---E	------------------------
	Lysinibacillus saudimassiliensis	CDZ99637	--------L-------------K-E-A	NV------------S---------
	Lysinibacillus sp. B2A1	AVK83979	--------L-------------A---E	T-----------------------
	Lysinibacillus sp. BF-4	WP_036144624	--------L-------------K-E-A	NV------------S---------
	Lysinibacillus sp. BK089	WP_132357149	--------L-----------------E	------------------------
	Lysinibacillus sp. FJAT-14222	WP_053592437	--------L-------------D---E	------------------------
	Lysinibacillus sp. FJAT-14745	WP_053484936	--------L-----------------E	------------------------
	Lysinibacillus sp. YR326	WP_134021825	--------L-----------------E	------------------------
	Lysinibacillus sp. YS11	WP_103118622	--------L-------------A---E	------------------------
	Lysinibacillus sp. ZYM-1	WP_054610783	--------L-------------A---E	------------------------
	Lysinibacillus sphaericus	POZ55394	--------L-------------A---E	------------------------
	Lysinibacillus varians	AHN22831	--------L-------------A---E	------------------------
	Lysinibacillus xylanilyticus	WP_049667549	--------L-----------------E	------------------------
	Sporosarcina sp. P13	WP_099689120	---T-D--L-------------A-E--	A-K-------Y---S---------
	Sporosarcina sp. PTS2304	WP_114924002	---T-D--L-------------A-E--	A-K-------Y---S---------
	Lysinibacillus chungkukjangi	WP_107934171	---T--T-L-------------K---E	P-----------------------
	Lysinibacillus composti	WP_124762395	---T--T-L-------------L---E	P-----------------------
	Lysinibacillus endophyticus	WP_121212741	---T--T-L-------------A---E	P-----------------------
	Lysinibacillus halotolerans	WP_122971148	---T--T-L-------------A---E	P----S------------------
	Lysinibacillus sinduriensis	WP_036198733	---T--T-L-------------L---E	P-----------------------
	Lysinibacillus sp. SYSU K30002	WP_126657299	---T--T-L-------------A---E	P-----------------------
	Lysinibacillus telephonicus	WP_126293646	---T--T-L-------------A---E	P----S------------------
	Lysinibacillus xyleni	WP_097072839	---T--T-L-------------A---E	P-----------------------
	Solibacillus isronensis	WP_079528316	------T-L-------------A---E	P--V----------T---------
Family *Planococcaceae* (>100/>100)	Lysinibacillus sp. YLB-03	WP_118877305	---T----L------------LA---E	PF----------------------
	Lysinibacillus sp. 2017	WP_108713487	------T-L-------------A---E	P--V----------T---------
	Solibacillus sp. R5-41	WP_099424793	------L-L-------------A---E	T-------------T---------
	Sporosarcina koreensis	WP_040286238	-----DV-L------------AA-E-E	A-K-------Y-------------
	Sporosarcina newyorkensis	WP_040759233	---T-D--L-------------A-D-A	A-K-------Y---S---------
	Sporosarcina pasteurii	WP_115362157	--------L-----------------E	P-KV------Y---S---------
	Sporosarcina psychrophila	WP_067207815	-----D--L-------------A-G-A	P-K-------Y---S---------
	Sporosarcina sp. BI001-red	WP_116019516	---T-DV-L------------AS---E	P-K-------Y---S---------
	Sporosarcina sp. D27	WP_025783256	---T-DV-L------------AA---E	P-K-------Y---S---------
	Sporosarcina sp. EUR3 2.2.2	WP_024534277	-----DV-L-------------A-G-E	P-K-------Y---S---------
	Sporosarcina sp. HY008	WP_067402979	--------L-------------A-D-A	A-K-------Y---S---------
	Sporosarcina sp. P16b	WP_099672459	---T-D--L-------------A-D-A	A-K-------Y---S---------
	Sporosarcina sp. P18a	WP_099674441	---T-D--L-------------A-D-A	A-K-------Y---S---------
	Sporosarcina sp. P19	WP_099690680	---T-D--L-------------A-D-A	A-K-------Y---S---------
	Sporosarcina sp. P20a	WP_099677408	---T-D--L-------------A-D-A	A-K-------Y---S---------
	Sporosarcina sp. P3	WP_099639070	---T-D--L-------------A-D-A	A-K-------Y---S---------
	Sporosarcina sp. P34	WP_099695202	---T-D--L-------------A-D-A	--K-------Y---S---------
	Sporosarcina sp. ZBG7A	WP_039044419	---T-DV-L------------AA---E	--K-------Y---S---------
	Sporosarcina ureae	WP_029052947	---T-D--L-------------V-D-A	A-K-------Y---S---------
	Paenisporosarcina antarctica	WP_134209401	-----D--L-------------A-G-E	P---------Y-------------
	Paenisporosarcina indica	WP_075620017	-----D--L-------------A-G-E	P-K-------Y---S---------
	Paenisporosarcina quisquiliaru	WP_090564168	-----D--L-------------A-E-Q	P-K-------Y-------------
	Paenisporosarcina sp. HGH0030	WP_016426725	-----D--L-------------A-G-E	P-K-------Y-------------
	Paenisporosarcina sp. K2R23-3	WP_119883544	------T-L---------------G-A	P---------Y---S---------
	Paenisporosarcina sp. OV554	WP_108585314	-----D--L-------------A-G-E	P-K-------Y---S---------
	Paenisporosarcina sp. TG-14	WP_017379876	-----D--L-------------A-G-E	P---------Y-------------
	Paenisporosarcina sp. TG20	WP_019414212	-----D--L-------------A-G-E	P-K-------Y-------------
	Planococcus antarcticus	WP_006830994	---T-D--L-------------A-G-E	P-K-------Y---S---------
	Planococcus citreus	WP_121300224	---T-D--L-------------A-G-E	P-K-------Y-------------
	Planococcus donghaensis	WP_008432271	---T-DV-L-------------A-G-E	P-K-------Y---S---------
	Planococcus halocryophilus	WP_008496485	---T-DV-L-------------A-G-E	P-K-------Y---S---------
	Planococcus maitriensis	WP_112232595	---T-D--L-------------A-G-E	P-K-------Y-------------
	Planococcus maritimus	WP_068461729	---T-D--L-------------A-G-E	P-K-------Y-------------
	Planococcus massiliensis	WP_052651978	---T-D--L-------------A-G-E	P-K-------Y-------------
	Planococcus plakortidis	WP_068868903	---T-D--L-------------A-G-E	P-K-------Y-------------
	Planococcus rifietoensis	WP_058381055	---T-D--L-------------A-G-E	P-K-------Y-------------
	Planococcus salinarum	TAA70474	-----D--L-------------A-G-E	P-K-------Y-------------
	Planococcus salinus	WP_123163633	---T-D--L-------------A-G-E	P-K-------Y-------------

Group	Organism	Accession			
Family *Planococcaceae* (>100/>100)	Planococcus sp. CAU13	WP_033543611	------L-L-------------A-G-E		P-K-------Y---S---------
	Planococcus sp. PAMC 21323	WP_038703554	---T-D--L-------------A-G-E		P-K-------Y---S---------
	Planococcus sp. Y42	WP_077588716	---M-D--L-------------A-G-E		P---------Y-------------
	Bhargavaea beijingensis	WP_092097117	---T-D--L-------------I---K		PFK-------Y-------------
	Bhargavaea cecembensis	WP_008301181	---T-D--L-------------A-Q-K		PFK-------Y-------------
	Bhargavaea ginsengi	WP_092051537	---T-DV-L-------------A-E-K		-FK-------Y-------------
	Exiguobacterium acetylicum	WP_029342314	---T-----------------LAS--E		P---L-----Y---E---------
	Exiguobacterium enclense	WP_058264729	---T-----------------LAS--E		P---L-----Y---E---------
	Exiguobacterium indicum	WP_058713776	---T-----------------LAS--E		P---L-----Y---E---------
	Exiguobacterium sp. BMC-KP	WP_053454014	---T-----------------LAS--E		P---L-----Y---E---------
	Exiguobacterium sp. Leaf196	WP_056062640	---T-----------------LAS--E		P---L-----Y---E---------
	Exiguobacterium sp. MH3	WP_023469011	---T-----------------LAS--E		P---L-----Y---E---------
	Bacillus ndiopicus	WP_042474811	--------L--Q----------A-E-A		AV------------S---------
	Bacillus cecembensis	WP_057989753	---T--L-L-------------A---E		H-------------A---------
	Bacillus sp. OxB-1	WP_041075450	------V-L-------------K-D-A		A-K-------Y-------------
	Planomicrobium flavidum	WP_088006604	---T-D--L-------------Q-E-E		P---------Y-------------
	Planomicrobium glaciei	WP_036801671	---TD---L-------------I---E		P-K-------Y---S---------
	Planomicrobium okeanokoites	WP_084246413	-----D--L-------------A-G-E		P-K-------Y-------------
	Planomicrobium soli	WP_106533883	---T-D--L-------------A-G-E		P-K-------Y-------------
	Planomicrobium sp. Y74	WP_121633396	-----D--L-------------A-G-E		P-K-------Y-------------
	Psychrobacillus insolitus	WP_111437697	-----D--L-------------A-E-Q		P-K-------Y-------------
	Psychrobacillus psychrodurans	WP_093496036	-----D--L-------------A-E-Q		P-K-------Y-------------
	Psychrobacillus psychrotoleran	WP_093536933	-----D--L-------------A-E-Q		P-K-------Y-------------
	Psychrobacillus sp. OK028	WP_093060443	-----D--L-------------A-E-Q		P-K-------Y-------------
	Psychrobacillus sp. OK032	WP_093266820	-----D--L-------------A-E-K		P-K-------Y-------------
	Rummeliibacillus pycnus	WP_102694251	----------------------K-Q-A		-VK---------------------
	Rummeliibacillus sp. POC4	WP_119415593	----------------------K-Q-A		-FK---------------------
	Rummeliibacillus sp. TYF005	WP_124217200	----------------------K-Q-A		-FK---------------------
	Rummeliibacillus stabekisii	WP_066791894	--------L-------------A---E		--K---------------------
	Chryseomicrobium excrementi	WP_100352300	------L-L-------------Q-Q-E		P-----------------------
	Edaphobacillus lindanitolerans	WP_076757958	---T-DV-L-------------A-E-Q		PF--------Y-------------
	Ureibacillus thermophilus	QBK26477	---T--T-L------------LA---K		PF--------Y---T---------
	Ureibacillus thermosphaericus	WP_016837174	---T--T-L------------LA---K		PF--------Y---T---------
	Viridibacillus sp. OK051	PKA84998	--------L--Q----------K-Q-A		AVK---------------------
Other Bacteria (0/>100)	Sporosarcina globispora	WP_053434169	---T----L-------------KHE-K	G	PVK-------Y-------------
	Anoxybacillus amylolyticus	WP_066327555	---T----L-------------KHQ-R	G	PVK-------Y-------------
	Anoxybacillus ayderensis	WP_085788304	---T----L-------------KHQ-R	G	PVK-------Y-------------
	Anoxybacillus flavithermus	AST06515	---T----L-------------KHQ-R	G	PVK-------Y-------------
	Anoxybacillus gonensis	WP_035065417	---T----L-------------KHQ-R	G	PVK-------Y-------------
	Anoxybacillus pushchinoensis	WP_091701343	---T----L-------------KHQ-R	G	PVK-------Y-------------
	Anoxybacillus suryakundensis	WP_055440059	---T----L-------------KHQ-R	G	PVK-------Y-------------
	Anoxybacillus tepidamans	WP_027409555	---T----L-------------KHQ-R	G	PVK-------Y-------------
	Anoxybacillus thermarum	WP_043967854	---T----L-------------KHQ-R	G	PVK-------Y-------------
	Bacillus acanthi	WP_108669360	---T----L-------------KHE-K	G	PVK-------Y-------------
	Bacillus alveayuensis	WP_044894550	---T----L-------------KHQ-R	G	PVK-------Y-------------
	Bacillus badius	WP_041097630	---T-D--L-------------KH--Q	G	PVK-------Y-------------
	Bacillus bataviensis	WP_007087357	---T------------------KHQ-K	G	P-K-------Y-------------
	Bacillus camelliae	WP_101354894	---T--L-L-------------KHE-K	G	PVK-------Y-------------
	Bacillus campisalis	WP_046523411	---T----L--------------RQ-K	G	PVK-------Y-------------
	Bacillus cihuensis	WP_028392175	---T--T---------------KH--Q	G	PVK-------Y-------------
	Bacillus coahuilensis	WP_010174130	---T-D--L-------------KHE-K	G	PVK-------Y---S---------
	Bacillus cohnii	WP_066415406	---T-DT-L-------------KNE-K	G	P-K-------Y---D---------
	Bacillus cucumis	WP_101649433	---T----L-------------KHE-K	G	P-K-------Y-------------
	Bacillus dakarensis	WP_077215408	--------L------M------KNE-K	G	PV--------Y-------------
	Bacillus deserti	WP_101640379	---T-D--L-------------KHQ-K	G	PVK-------Y-------------
	Bacillus dielmoensis	WP_042459558	---T----L-------------KHQ-K	G	P-K-------Y-------------
	Bacillus drentensis	WP_066258044	---T-D--L-------------KHQ-K	G	P-K-------Y-------------
	Bacillus farraginis	WP_058006616	---T-DL-L-------------KHE-K	G	PVK-------Y-------------
	Bacillus firmus	WP_035330265	---T-D--L-------------KHE-K	G	PVK-------Y-------------
	Bacillus foraminis	WP_121611483	---T----L-------------KRQ-K	G	PVK-------Y-------------
	Bacillus fordii	WP_018708612	---T------------------KHE-K	G	PVK-------Y-------------
	Bacillus fortis	WP_120075072	---T------------------KHE-K	G	PVK-------Y-------------
	Bacillus freudenreichii	WP_126431565	---T------------------KHE-K	G	PVK-------Y-------------
	Bacillus fumarioli	WP_066365951	---T----L-------------KHQ-K	G	P-K-------Y-------------
	Bacillus humi	WP_057999482	---T-D--L--Q----------KHE-K	G	PVK-------Y-------------
	Bacillus kochii	WP_095372335	---T-D--L-------------KHE-K	G	PVK---------------------
	Bacillus marinisedimentorum	WP_070121835	------L-L-------------KHE-K	G	PVK-------Y---T---------
	Bacillus marisflavi	WP_063192036	------T-L-------------MAE-K	G	P-K-------Y---T---------
	Bacillus massiliglaciei	WP_110927168	---T-D---------------KKHE-K	G	PVK-------Y-------------
	Bacillus mesonae	WP_066386017	---T----L-------------KHQ-K	G	P-K-------Y-------------
	Bacillus methanolicus	WP_003347507	---T----L-------------KHQ-K	G	PVK-------Y-------------
	Bacillus mojavensis	APQ43082	---T-DM----Q-----T----KH--K	G	PVK-------Y-------------
	Bacillus niacini	WP_034676894	---TD---L-------------KNQ-K	G	P-K-------Y-------------
	Bacillus novalis	WP_066090686	---T----L-------------KHQ-K	G	P-K-------Y-------------

Other Bacteria (0/>100)

```
Bacillus obstructivus           WP_071976978  ---T-DL-L-------------KHE-K G PVK-------Y-------------
Bacillus oceanisediminis        WP_110067879  ---T----L-------------KHE-K G PVK-------Y-------------
Bacillus oleronius              WP_078110222  ---T-DL-L-------------KHE-K G PVK-------Y-------------
Bacillus onubensis              WP_099352044  ---T-D--L--Q----------KHE-K G PVK-------Y-------------
Bacillus panaciterrae           WP_028400919  ---T----L-------------KHE-K G P-K-------Y---D---------
Bacillus persicus               WP_090741492  ---T----L-------------ARN-K G PVK-------Y-------------
Bacillus rubiinfantis           WP_042354841  ---T----L-------------KHQ-K G P-K-------Y-------------
Bacillus simplex                CEG34014      ---TD-----------------KH--Q G PVK-------Y-------------
Bacillus smithii                WP_003355591  ---T----L------I------KH--K G PVK-------Y-------------
Bacillus solani                 WP_056685942  ---T----L-------------KHE-K G PVK-------Y-------------
Bacillus sporothermodurans      WP_066227040  ---T-D--L-------------KHE-K G PVK-------Y-------------
Bacillus taeanensis             WP_113804226  ---T----L-------------AHE-K G PVKV----R-Y---T---------
Bacillus timonensis             WP_010678363  ---T-D--L--Q----------KH--K G PVK-------Y-------------
Bacillus tuaregi                WP_071395980  ---T------------------MHN-Q G PVK-------Y-------------
Bacillus vireti                 WP_024029198  ---T----L-------------KHE-K G P-K-------Y-------------
Caenibacillus caldisaponilytic  WP_077615565  ---T-D--L-------------AHQ-K G PVK-------Y---T---------
Caldibacillus debilis           OUM90948      --------L-------------SHQ-R G PVK-------Y-------------
Calditerricola satsumensis      WP_054673488  ---T----L--------I----A-G-A V PVK-------Y---D---------
Desmospora activa               WP_107724991  ---TP---L--------V-----RE-Q V PVKV------Y-------------
Enterococcus camelliae          CBY44825      ---TD-----------------KRH-Q G PVK-------Y---S---------
Falsibacillus pallidus          WP_114745092  ---T-DT-L-------------KHQ-K G PVK-------Y-------------
Geobacillus galactosidasius     WP_089098004  ---T----L-------------KHQ-R G PVK-------Y-------------
Jeotgalibacillus alimentarius   WP_041120955  ---T-DV-L-------------RHA-K G PVK-------Y-------------
Jeotgalibacillus campisalis     WP_041059966  ---T-DL-L-------------RRE-K G PVK-------Y-------------
Jeotgalibacillus malaysiensis   WP_039810241  ---T-DV-L-------------RHA-K G PVK-------Y-------------
Jeotgalibacillus proteolyticus  WP_104057081  ---T-D--L-------------RHQ-K G PVK-------Y-------------
Jeotgalibacillus salarius       WP_134381591  ---T-DV-L-------------RHA-K G PVK-------Y-------------
Jeotgalicoccus halophilus       SDJ63643      --------L-----------L-AQ--E G PVK-------Y---S---------
Jeotgalicoccus halotolerans     WP_115884170  --------L-----------L-AQN-E G PVK-------Y---S---------
Jeotgalicoccus marinus          WP_026866881  -----D--L-----------L-ASN-V G PVK-------Y---S---------
Jeotgalicoccus psychrophilus    WP_026859724  -----D--L-----------L-AQ--E G PVK-------Y---S---------
Jeotgalicoccus saudimassiliens  WP_035809162  -----D--L-----------L-AQ--E G PVK-------Y---S---------
Macrococcus bovicus             WP_133450945  ---T--L-------------L-SR--Q G PVK-------Y---S---------
Macrococcus hajekii             WP_133428853  ---T--L-------------L-SR--Q G PVK-------Y---S---------
Macrococcus lamae               WP_133443841  ---T--L-------------L-SR--Q G PVK-------Y---S---------
Marininema halotolerans         WP_091837001  ---TP---L--------V------E-Q V PVK-------Y---D---------
Marininema mesophilum           WP_091742979  ---TP---L--------V----A-E-Q V PVK-------Y---D---------
Mycobacteroides abscessus subs  SHP97679      ---T----L------M------K-E-K G P-K-------Y-------------
Paraburkholderia tropica        RQN30531      ---T------------------KR--Q G PVK-------Y---S---------
Parageobacillus thermantarctic  WP_090947599  ---T----L-------------KHQ-R G PVK-------Y-------------
Parageobacillus thermoglucosid  WP_064549597  ---T----L-------------KHQ-R G PVK-------Y-------------
Parageobacillus toebii          WP_062678558  ---T----L-------------KHQ-R G PVK-------Y-------------
Quasibacillus thermotolerans    WP_039238480  ---T--L-L-------------KY--Q G PVK-------Y-------------
Salinicoccus carnicancri        WP_017549066  --F--D--L------------QA---Q G PVKV------Y---S---------
Salinicoccus haloduransc        WP_046790404  --F--D--L------------QAR--A G PVK-------Y---S---------
Salinicoccus roseus             WP_040106763  ------T-L-------------AQE-R G PVK-------Y---S---------
Salinicoccus sediminis          WP_046512042  --F--D--L------------QAR--Q G PVKV------Y---S---------
Staphylococcus agnetis          WP_037566126  ---T-D----------------KR--K G PVK-------Y---S---------
Staphylococcus argenteus        WP_047429724  ---TD---L-------------SRN-Q G PVK-------Y---S---------
Staphylococcus arlettae         WP_107376936  ---T------------------QRN-Q A PVK-------Y---S---------
Staphylococcus aureus           KMS28614      ---T------------------KR--Q G PVK-------Y---S---------
Staphylococcus capitis          TBW73926      ---T------------------KR--Q G PVK-------Y---S---------
Staphylococcus cohnii           RIM33618      ---TD-----------------KRN-E G PVK-L-----Y---S---------
Staphylococcus cornubiensis     WP_086429071  ---T--V-L-------------QRN-Q G PVK-------Y---S---------
Staphylococcus delphini         WP_019166363  ---T--V-L-------------QRN-Q G PVK-------Y---S---------
Staphylococcus epidermidis      WP_002473241  ---T------------------KR--Q G PVK-------Y---S---------
Staphylococcus felis            WP_115855797  ------T-L-------------QR--Q G PVK-------Y---S---------
Staphylococcus hyicus           WP_107633276  ---T-D----------------KRN-Q G PVK-------Y---S---------
Staphylococcus intermedius      WP_019168152  ---T--V-L-------------QRN-Q G PVK-------Y---S---------
Staphylococcus pasteuri         WP_046466578  ---T------------------QRN-Q G PVK-------Y---S---------
Staphylococcus pseudintermediu  PWZ95519      ---T--V-L-------------QRN-Q G PVK-------Y---S---------
Thermolongibacillus altinsuens  WP_132947564  ---T----L-------------KHQ-R G PVK-------Y-------------
```

Supplemental Figure 3

A partial sequence alignment of the phenylalanine-tRNA ligase subunit alpha protein containing a one amino acid deletion (boxed) that is exclusively shared by all members among the *Planococcaceae* family and absent in all other bacteria.

Family *Planococcaceae* (>100/>100)

		401		454
Bacillus cecembensis	WP_057990117	TRAAVEEGIVSGGGTALLNVYSAVVNVL	EE	VEGDVATGVKIILRALEEPVRQIA
Bacillus ndiopicus	WP_042479197	------------------------EK--	--	------------------------
Lysinibacillus odysseyi	WP_036151297	------------------------EK--	-T	------------------------
Lysinibacillus meyeri	WP_107840531	---------------------G--EK--	--	------------------------
Lysinibacillus sp. 2017	WP_108714099	---------------------G--S-I-	DT	------------------------
Lysinibacillus chungkukjangi	WP_107937450	---------------------A--EK-I	DS	---------R-V------------
Lysinibacillus contaminans	WP_053584791	---------------------G--AA-A	-K	E-------L--V------------
Lysinibacillus endophyticus	WP_121214314	---------------------G--EK--	DT	-----------V------------
Lysinibacillus fusiformis	WP_025117685	---------------------A--EK-S	-S	EA---------V------------
Lysinibacillus halotolerans	WP_122973482	---------------------G--EK--	D-	---------R-V------------
Lysinibacillus macroides	WP_053995289	---------------------A--EKAA	-S	-----------V------------
Lysinibacillus acetophenoni	WP_097150827	-------------------I----EK--	G-	-D---------V------------
Lysinibacillus manganicus	WP_036188880	-------------------I-GS-EK--	N-	-----------V------------
Lysinibacillus massiliensis	WP_036176209	-------------------I-N--DK--	S-	---------R-V------------
Lysinibacillus mangiferihumi	WP_107895784	---------------------A--EKAA	DA	-D---------V------------
Lysinibacillus parviboronicapiens	WP_107925491	---------------------A--EKAA	DV	AD---------V------------
Lysinibacillus jejuensis	WP_108307812	-----------------M---G--EKAA	-N	A--------N-V------------
Lysinibacillus saudimassiliensis	CEA03782	-----------------M---G--EKAA	-N	A-------IN-V------------
Lysinibacillus sp. BF-4	WP_036142882	-----------------M---G--EKAA	-N	A-------IN-V------------
Lysinibacillus sinduriensis	WP_036198200	---------------------A--EK-T	-T	---------R-V------------
Lysinibacillus sp. B2A1	AVK86363	---------------------A--EK-A	DA	E----------V------------
Lysinibacillus sp. BK089	WP_132364227	---------------------A--EK-A	DA	E----------V------------
Lysinibacillus sp. FJAT-14222	WP_053594821	---------------------A--EK-A	DV	E----------V------------
Lysinibacillus sp. FJAT-14745	WP_053485178	---------------------A--EK-A	DA	E----------V------------
Lysinibacillus sp. Marseille-P	WP_106781514	-------------------I-N--E---	N-	---------R-V------------
Lysinibacillus sp. SYSU K30002	WP_126659346	---------------------G--EK--	-T	---------R-V------------
Lysinibacillus sp. YLB-03	WP_118877557	---------------------G--EKI-	--	---------R-V------------
Lysinibacillus sp. YR326	WP_134024968	---------------------A--EK-A	DA	E----------V------------
Lysinibacillus sp. ZYM-1	WP_054612085	---------------------A--EKMA	-S	E-------I--V------------
Lysinibacillus sphaericus	WP_010857069	---------------------A--EKAA	DV	-D-------R-V------------
Lysinibacillus telephonicus	WP_126293786	---------------------G--EK--	D-	---------R-V------------
Lysinibacillus xylanilyticus	WP_049667832	---------------------A--EK-A	DA	E----S-----V------------
Lysinibacillus xyleni	WP_097072406	------------------------EKI-	-T	---------R-V------------
Lysinibacillus boronitolerans	WP_036075467	---------------------A--EKAA	-A	-----------V------------
Bhargavaea beijingensis	WP_092098015	-----------------V---NK-GELQ	-G	-----------V------------
Bhargavaea cecembensis	WP_063178964	-----------------V---NK-GELQ	-G	-----------V------------
Bhargavaea ginsengi	WP_092056114	-----------------V---NK-AELQ	-G	A----------V------------
Chryseomicrobium excrementi	WP_100354605	-----------------V---NK-AETV	-T	---------R-V------------
Edaphobacillus lindanitolerans	WP_076759817	-----------------V---NK-AEIQ	-A	S----------V------------
Paenisporosarcina antarctica	WP_134208655	-----------------V---KK-AE-A	AA	ET---------V------------
Kurthia massiliensis	WP_010291044	-----Q----A---V--VSI-NK-SALA	D-	F--------R-V-------I----
Kurthia sp. 3B1D	WP_126989630	-----Q----A---V--VSI-NK-AELA	DA	N--------R-V-------I----
Caryophanon latum	WP_066465529	----------A----------G--SA--	DT	-----------V------------
Caryophanon tenue	WP_066548435	----------A----------G--SA--	-Q	-----------V------------
Paenisporosarcina quisquiliarum	WP_090568932	----------A------V---NK-AELA	-T	K-------L--V------------
Planomicrobium koreense	WP_135501517	----------A------V---NK-AEL-	-T	R---E------V------------
Kurthia zopfii	WP_126342713	----------A---V--VSIHG--DA-V	NA	T-------AR-V------------
Paenisporosarcina indica	WP_075620375	----------A------V---NK-SEIA	AA	EA---------V------------
Planococcus antarcticus	WP_006831534	----------A------I---NK-AEL-	-T	Q--------N-V------------
Planococcus citreus	WP_121300307	----------A------I---NQ-AQIA	T-	Q-------IN-V------------
Planococcus donghaensis	WP_065527250	----------A------V---NK-AEL-	-S	Q-------IN-V------------
Planococcus faecalis	WP_071154661	----------A------V---NK-AEL-	-S	Q-------IN-V------------
Planococcus halocryophilus	WP_008497389	----------A------V---NK-AEL-	-S	Q-------IN-V------------
Planococcus halotolerans	WP_112223311	----------A-----YI---NN-AEI-	-T	I---E------V------------
Planococcus kocurii	WP_058386358	----------A------V---NK-AEL-	-S	Q-------IN-V------------
Planococcus maitriensis	WP_112233294	----------A------I---NQ-AE-A	TQ	Q-------IN-V------------
Planococcus maritimus	WP_068460459	----------A------I---NQ-AE-A	A-	Q--------N-V------------
Planococcus massiliensis	WP_052653084	----------A------V---NR-AEL-	-S	Q--------N-V------------
Planococcus plakortidis	WP_068872112	----------A------I---NQ-AQ-A	T-	Q--------N-V------------
Planococcus rifietoensis	WP_058381755	----------A------I---NQ-AE-A	AQ	H--------N-V------------
Planococcus salinarum	TAA71668	----------A------V---NK-AEL-	-T	T----------V------------
Planococcus sp. CAU13	WP_033541544	----------A-----YI---NS-AEI-	-T	T---E------V------------
Planococcus sp. MB-3u-03	WP_101189529	----------A------I---NQ-AQIA	T-	Q-------IN-V------------
Planococcus sp. PAMC 21323	WP_038702632	----------A------V---NK-AEL-	TA	Q-------IN-V------------
Planococcus versutus	WP_049694407	----------A------I---NK-AEL-	-T	K-------IN-V-----------V
Planomicrobium glaciei	WP_074509934	----------A------V---NK-AEL-	-T	Q---I----N-V------------
Planomicrobium soli	WP_106532350	----------A------A--HNR-AEL-	-T	ET-------R-V------------
Planomicrobium sp. Y74	WP_121633970	----------A-----YI---NN-AEI-	-T	T---E------V------------
Psychrobacillus psychrotoleran	WP_093538283	----------A------V---NK-AELA	-T	K-------L--V------------
Psychrobacillus sp. OK028	WP_093062145	----------A------V---NK-AELA	-T	K-------L--V------------
Planococcus salinus	WP_123165993	-----------------V----K-AEL-	-Q	E--------N-V-------L----
Planococcus sp. Y42	WP_077588034	-----------------I---NS-NELA	-A	Q----------V------------
Planomicrobium flavidum	WP_088008898	-----------------I-I-NS-NELA	-G	Q----------V------------
Paenisporosarcina sp. HGH0030	WP_016429474	-----------------V---NK--E-T	SD	AT---------V------------

Group	Species	Accession	Alignment	Boxed	Alignment (cont.)
Family *Planococcaceae* (>100/>100)	Paenisporosarcina sp. K2R23-3	WP_119884055	----------------V---NK-SE-T	A-	ET---------V------------
	Paenisporosarcina sp. OV554	WP_108587730	----------------V---KK-AELA	DT	EQ---------V------------
	Paenisporosarcina sp. TG-14	WP_017379543	----------------V---KK-AE-A	AT	ET---------V------------
	Paenisporosarcina sp. TG20	WP_019413609	----------------V---KK-AE-A	AA	ET---------V------------
	Psychrobacillus sp. OK032	WP_093274217	----------------V---NK-AE-A	AA	Q-------L--V------------
	Solibacillus isronensis	WP_079523186	--------------------A--EK--	DQ	---------R--------------
	Solibacillus sp. R5-41	WP_099425422	--------------------G------	--	------------------------
	Sporosarcina koreensis	WP_060205192	----------------V---TK-ETL-	-S	T----------V------------
	Sporosarcina newyorkensis	WP_009498836	----------------V---KQ-EAL-	-S	T----------V------------
	Sporosarcina pasteurii	WP_115363310	----------------I---KK-EELN	-T	ET-------N-V------------
	Sporosarcina psychrophila	AMQ04977	----------------V----K-AAL-	-S	T-------I--V------------
	Sporosarcina sp. BI001-red	WP_116015955	----------------I---KK-ESL-	-G	L----------V------------
	Sporosarcina sp. D27	WP_025782800	----------------V---KK-ESL-	-G	-----------V------------
	Sporosarcina sp. P20a	WP_099677657	----------------V---KK-EAL-	-D	T-------I--V------------
	Sporosarcina sp. P26b	WP_099693918	----------------V---KK-EAL-	-D	T-------I--V------------
	Sporosarcina sp. P33	WP_081243060	----------------V---KQ-ETL-	-S	T-------I--V------------
	Sporosarcina sp. P34	WP_099696711	----------------V---KQ-EAL-	-G	T----S--I--V------------
	Sporosarcina sp. P7	WP_099636628	----------------V---KQ-EAL-	-G	T----S--I--V------------
	Sporosarcina sp. ZBG7A	WP_039043677	----------------I---KK-ESL-	-G	-----------V------------
	Tetzosporium hominis	WP_094942652	----------------V---NK-AETV	-T	---------R-V------------
	Sporosarcina sp. EUR3 2.2.2	WP_024536836	----------------V---KK-AELV	DT	ET---------V------------
	Sporosarcina sp. HY008	WP_067408540	----------------V---GK-EEL-	NS	T----------V------------
	Sporosarcina sp. P13	WP_099687467	----------A------V---NK-EAC-	--	T---------R-V---S---------
	Sporosarcina sp. P18a	WP_099676570	----------------V---KQ-EAL-	-D	T----S--I--V------------
	Sporosarcina sp. P3	WP_099638904	----------A------V-I--K-EAS-	--	---------R-V------------
	Sporosarcina sp. PTS2304	WP_114924861	----------------V---KQ-EAL-	-N	T----------V------------
	Sporosarcina ureae	WP_083035041	----------A------V-I--K-EAS-	--	---------R-V------------
	Ureibacillus thermophilus	QBK24564	----------A------V---K--EK--	D-	-D---------V------------
	Ureibacillus thermosphaericus	WP_016839635	----------A------V---K--EK--	D-	-D---------V------------
	Viridibacillus arenosi	WP_038179462	-----Q----A---V--VSI-NK-AE-A	DA	Y----------V------------
	Viridibacillus arvi	WP_053418960	-----Q----A---V--VSI-NK-AE-A	-A	H----------V------------
	Viridibacillus sp. OK051	WP_100795715	-----Q----A---V--VSI-NK-AE-A	AA	N----------V------------
	Psychrobacillus insolitus	WP_111439689	----------------V---KK-AA-A	-A	Q-------LR-V------------
	Rummeliibacillus pycnus	WP_102693766	----------A---V--VSI-NK-AE-E	AA	ET---------V------------
	Rummeliibacillus stabekisii	WP_066790231	----------A---V--VS--NK-AEIG	QA	EA---------V------------
Other Bacteria (2/>200)	Salsuginibacillus halophilus	WP_106588273	----------A----S---I-K--KDLE	-Q	----E---IS-L--S---------
	Salsuginibacillus kocurii	WP_018923647	----------A------V---K--KSLE	-Q	T---E---IS-VA-----------
	Aeribacillus pallidus	WP_063388690	----------------V---KK-AEIE		A----Q---N-V------------
	Alkalibacillus haloalkaliphilu	WP_017187342	----------A------V-I--K-SELQ		L---E---AS-V---M--------
	Amphibacillus sediminis	WP_067841750	----------------M---KQ-GELN		L---E---AS-V------------
	Aneurinibacillus aneurinilytic	WP_021622177	----------A------VSI-NK-AEIQ		A-D-E----RL-------------
	Aneurinibacillus migulanus	WP_043067081	----------A------VSI-NK-AEIQ		A-D-E----RL-------------
	Aneurinibacillus terranovensis	WP_027417447	----------------V---N--AGLQ		----E----RLV------------
	Anoxybacillus flavithermus	WP_004888757	----------A------M---NK-AAIE		A---E------V---I--------
	Anoxybacillus pushchinoensis	WP_091704821	----------A------M---NK-AAIE		A---E------V---I--------
	Anoxybacillus tepidamans	WP_027410639	----------A----------NK-AAIQ		A---E------V---I--------
	Anoxybacillus thermarum	WP_043968485	----------A------M----K-AAIE		A---E------V---I--------
	Anoxybacillus vitaminiphilus	WP_111646473	----------A----------NK-AAIE		A---E------V---I--------
	Aureibacillus halotolerans	WP_133581947	----------------V-I-NK-AEID		A---IE-----V------------
	Bacillus abyssalis	WP_078415060	----------A------V-I-NK-AEIQ		A---E----N-V---M--------
	Bacillus acanthi	WP_108672168	--------------V------NK-AEIQ		A---A---IN-V---M-------S
	Bacillus acidicola	WP_066267221	----------------FV---NK-ASIA		A----Q--IN-V--S---------
	Bacillus aciditolerans	WP_121449571	----------------V---NK-AAIE		LA--E----N-V------------
	Bacillus alkalitelluris	WP_078543028	----------A------V---NK-ASIE		A-------I--V------------
	Bacillus alveayuensis	WP_044893077	----------A----------NK-AAIE		A---E------V---I--------
	Bacillus amyloliquefaciens	ABX45457	----------------V---NK-AA-E		A---AQ--IN-V-------I----
	Bacillus andreraoultii	WP_033826907	----------------V---NK-AAIE		A---E------V------------
	Bacillus aquimaris	WP_071617314	----------------V---NK-ASIE		ADA-I---IN-V-------I----
	Bacillus asahii	WP_119118295	----------A----------NK-AELQ		A---E------V---I--------
	Bacillus atrophaeus	ABX45512	----------------V---NK-AA-E		A---AQ--IN-V-------I----
	Bacillus badius	WP_041101410	----------------V---NK-ASLE		A-------I--V-------I----
	Bacillus camelliae	WP_101356556	--------L--------V---NK-AEIS		A-------IN-V------------
	Bacillus cavernae	WP_126865828	----------A----------NK-ASIQ		A---Q----N-V---I--------
	Bacillus cihuensis	WP_028390611	----------A-----F-----K-AEIQ		A-------IN-V---I--------
	Bacillus clausii	PAF12162	--------------V------NK-ASIQ		A---E---IN-V---M-----T--
	Bacillus coahuilensis	WP_059283757	----------------V---NK-ASLE		Q----Q--IN-V------------
	Bacillus cohnii	WP_066415716	----------A------V-I-NK-A-IT		A---E------V---I--------
	Bacillus cucumis	WP_101646334	--------------V-------K-AELQ		E-------IN-V---M-----T--
	Bacillus dakarensis	WP_077210612	--------------V------NK-AQLQ		E---AQ--IN-V---I---M----
	Bacillus endophyticus	WP_061803968	----------A------V---NK-AALS		E----Q--IN-V------------
	Bacillus fastidiosus	WP_066232397	----------------V---RK-AQIQ		E---RQ---N-V------------
	Bacillus flexus	WP_119543978	----------A------V-I-NK-AGIE		A-------IN-V---I--------
	Bacillus fordii	WP_018706778	----------------V--FNK-SAIG		D---------V------------
	Bacillus fortis	WP_120073435	----------------V---NK-AEIQ		G-D------R-V------------

Other Bacteria (2/>200)

Bacillus freudenreichii	WP_126433989	-----------------I---NK-AEIQ	G----------V------------
Bacillus ginsengihumi	WP_025731419	-----------------V---NK-ASID	A-------I--V------------
Bacillus glycinifermentans	WP_048356183	-----------------V---NK-AEIN	A---EL---N-V-------I----
Bacillus gottheilii	WP_080848772	--------------V------NK-AELQ	A---E---IN-V---M-----T--
Bacillus halmapalus	WP_078382371	----------A-----FV-I-NK-SQIQ	A---E----N-V---M--------
Bacillus halosaccharovorans	WP_078433173	-----------------V---NK-AQIQ	E---TQ---N----S---------
Bacillus haynesii	WP_043927779	-----------------V---NK-AALE	A---KL--IN-V-------I----
Bacillus hisashii	WP_095142424	-----------------V---NK-AAIE	A----------V------------
Bacillus horikoshii	WP_064098044	----------A------V-I-NQ-AAIQ	A---E---I--V---I--------
Bacillus horneckiae	WP_066400446	--------------V------NK-AALQ	A-------IN-V---M-----T--
Bacillus indicus	WP_029286195	-----------------V---NK-AS-E	G---F----N-V------------
Bacillus jeotgali	WP_079504156	--------------V------KK-SEIQ	A---E----N-V---M--------
Bacillus kribbensis	WP_026693063	----------A-----------KINEIE	A--------S-V---I--------
Bacillus kwashiorkori	WP_062352609	-----------------V---RK-SEIA	A---TL-----V------------
Bacillus lentus	WP_066145113	-----------------V---KK-AELE	AT------IN-V------------
Bacillus licheniformis	WP_044789749	-----------------V---NK-AALE	A---EL--IN-V-------I----
Bacillus litoralis	WP_066335680	-----------------V---NK-AALQ	E---TQ--IN--------------
Bacillus loiseleuriae	WP_049679667	----------A-----------KIAEIQ	A-------IN-V---I--------
Bacillus marisflavi	WP_048013535	-----------------V---NK-ASIE	ADS-----IN-V-------I----
Bacillus massiliglaciei	WP_110926844	----------A----------NK-AELT	A---E------V---I--------
Bacillus massiliogorillae	WP_042353099	---------------------NK-AEIV	A---E----N-V---M--------
Bacillus mesonae	WP_066390599	--------------V-------K-AELQ	E---E---I--V---M-----T--
Bacillus methanolicus	AGN11449	----------A----------NK-ASIQ	S---E----N-V---M--------
Bacillus mojavensis	ABX45459	-----------------V---NK-AA-E	A---AQ--IN-V-------I----
Bacillus muralis	WP_057915808	----------A----------NKIAEIQ	A----------V---I--------
Bacillus nakamurai	WP_061520158	-----------------V---NK-AA-Q	A---AQ--IN-V-------I----
Bacillus niacini	WP_045516312	--------------V-------K-AE-Q	A-------IN-V---I-----T--
Bacillus niameyensis	WP_062106544	-----------------V---NK-AA-E	A-------IN-V------------
Bacillus novalis	WP_066095316	--------------V-------K-AE-Q	A-------IN-V---M-----T--
Bacillus oleivorans	WP_097160811	----------A------V---NK-ASIQ	A----Q--IN-V------------
Bacillus onubensis	WP_099355431	-----------------V---NK-AAIE	LA--E----N-V------------
Bacillus oryziterrae	WP_017754995	-----------------V---NK-AALD	LQ--EK---N-V------------
Bacillus paralicheniformis	WP_096747897	-----------------V---NK-AALE	A---EL--IN-V-------I----
Bacillus pumilus	WP_117729039	-----------------V---NK-ASIE	AD---Q---N-V--S----I----
Bacillus rubiinfantis	WP_042357623	--------------V-------K-AELE	A---E---I--V---M---I-T--
Bacillus safensis	WP_111291478	-----------------V---NK-ASIE	AD---Q---N-V--S----I----
Bacillus salsus	WP_090858499	----------A------V---NK-ASIE	A-------I--V------------
Bacillus simplex	WP_061142110	----------A----------NKIAEIQ	A----------V---I--------
Bacillus sinesaloumensis	WP_077620357	-----------------V---NK-AAID	LA--E----N-V------------
Bacillus smithii	WP_003354043	-----------------V---NK-AAIE	A----------V-------I----
Bacillus solimangrovi	WP_069718253	-----------------V---NK-AAIE	AD--E----N-V------------
Bacillus sonorensis	ABX45472	-----------------V---NK-AAID	A---EL---N-V-------I----
Bacillus tequilensis	ABX45486	-----------------V---NK-AA-E	A---AQ--IN-V-------I----
Bacillus terrae	WP_120118278	-----------------V---NK-AEIE	GQ---------V------------
Bacillus thermoamylovorans	WP_034767858	-----------------V---NK-AAIE	A----------V------------
Bacillus timonensis	WP_010285538	-----------------V---NK-AAIE	LA--E----N-V------------
Bacillus vallismortis	ABX45462	-----------------V---NK-AA-E	A---AQ--IN-V-------I----
Bacillus velezensis	ABX45455	-----------------V---NK-AA-E	A---AQ--IN-V-------I----
Bacillus vireti	WP_024027273	--------------V-------K-AE-Q	A-------IN-V---M-----T--
Bacillus weihaiensis	WP_072581348	-----------------V---NK-AAIT	ED--TQ---N--------------
Bacillus wudalianchiensis	WP_065409735	-----------------V---NK-AA-E	A----------V-------I----
Bacillus xiamenensis	WP_008355697	-----------------V---NK-ASIE	A----Q---N-V--S----I----
Caldibacillus debilis	OUM88416	----------A------I---NK-ASIE	A----------V------------
Carnobacteriaceae	WP_047391097	S-------L-------YI--QNK-AEIE	A-------I--VV-----------
Carnobacterium divergens	TFJ44984	----------A------V--QAK-AEIE	A-------I--V--S----L---V
Carnobacterium funditum	WP_034551261	A---------------FI--Q-K-AEIA	E----------V-------I---V
Carnobacterium iners	WP_085558691	A---------------FI--QAK-AELE	E-------I--V-------I---V
Chlamydia trachomatis	CRH69616	----------A----------NKIAEIQ	A----------V---I--------
Domibacillus antri	WP_075399438	-----------------V---NK-AE-E	G----------V-------L----
Domibacillus enclensis	WP_045852576	-----------------V---NK-AE-E	G----------V------------
Domibacillus epiphyticus	WP_076764012	----------A------V---NK-AE-Q	A----------V------------
Domibacillus indicus	WP_046176602	-----------------V---NK-AE-G	----------V------------
Domibacillus iocasae	WP_069938273	-----------------V---NK-AE-G	----------V------------
Domibacillus mangrovi	WP_073712242	-----------------V---NK-TE-G	---F------V------------
Domibacillus robiginosus	WP_050184119	-----------------V---NK-AE-G	--------R-V-------L----
Domibacillus tundrae	WP_046181252	-----------------V---NK-AA-G	----------V------------
Enterococcus avium	AZQ23886	-----------------V--IKK-SELE	A---A---I--VV-----------
Enterococcus avium ATCC 14025	AAN32671	-----------------V--IKK-SELE	A---A---I--VV-----------
Enterococcus caccae	WP_010773016	--------M--------V--I-K-TALE	-----------VV------I----
Enterococcus canis	WP_067392854	--------M--------V--INK-SA-E	A---A------V-------I----
Enterococcus casseliflavus	AAN32673	--------M--------V--INK-AEIE	TD--A------V------------
Enterococcus faecium	PHK56798	-----------------V--IKK-SDLD	A-------I--VV-----------
Enterococcus gallinarum	AAN32679	--------V--------V--INK-AAIE	AD--A------V------------
Enterococcus hermanniensis	WP_071856555	-----------------V--IKK-SELA	A---A---I--VV-----------

Other Bacteria (2/>200)

```
Enterococcus hirae                WP_081186470   --------M--------V--INK-SA-E    AQ---------VV------I----
Enterococcus malodoratus          AZQ23878       -----------------V--IKK-SDLD    A-------I--VV-----------
Enterococcus pseudoavium          WP_067623250   -----------------V--IKK-SELA    A-------I--VV-----------
Enterococcus raffinosus           AAN32681       -----------------V--IKK-SDLD    A-------I--VV-----------
Enterococcus saccharolyticus      WP_005471702   --------M--------V--INK-AAIE    AD--A------V------------
Enterococcus sulfureus            WP_016185887   --------M--------V--IQK-AQLE    A-------IR-VV-----------
Enterococcus wangshanyuanii       WP_088268406   --------M--------V--INK-AE-E    A----------VV------I----
Geobacillus galactosidasius       WP_089096750   ----------A------M---NK-AAIE    A---E------V---I--------
Geobacillus lituanicus            WP_094239484   ----------A------M---NK-AAIE    A---E------V---I--------
Gracilibacillus boraciitoleran    WP_035722391   ---------------------KK-SELG    L---E----N-V-----A------
Gracilibacillus lacisalsi         WP_018934112   -----Q---------------NK-AELN    L---E----N-V-----S------
Jeotgalibacillus alimentarius     WP_041121381   -----------------V---NK-ASIE    A-D-----IN-V-------I----
Jeotgalibacillus campisalis       WP_041056435   -----------------V----K-AAIE    AQ------I--V-------I----
Jeotgalibacillus malaysiensis     WP_039807394   -----------------V---NK-AA-E    A-D-----IN-V-------I----
Jeotgalibacillus proteolyticus    WP_104059920   -----------------V---NK-ASIE    A----S--IN-V-------I----
Jeotgalibacillus salarius         WP_134382020   -----------------V---NK-AAIE    A-E-----IN-V-------I----
Jeotgalibacillus soli             WP_041086174   -----------------V---NK-AAIE    A-------I--V-------I----
Listeria booriae                  WP_036086437   -----------------V---NK-AAIE    AT--E------V--S---------
Listeria costaricensis            WP_099222412   -----------------V---NK-AELE    AT--E---I--V--S---------
Listeria fleischmannii            WP_007475471   -----------------V---NK-AAIE    AT--E------V--S---------
Listeria grandensis FSL F6-097    EUJ25115       -----------------V---NK-AAIE    AT--E------V--S---------
Listeria grayi                    WP_003758171   -----------------V---NK-AELE    AT--E---I--V--S---------
Listeria innocua FSL S4-378       EFR90107       ----------A------VSI-NK-AALE    A----E--IN-V--S---------
Listeria ivanovii                 WP_014093402   ----------A------V-I-NK-AELE    A----E--IN-V--S---------
Listeria monocytogenes FSL F2-    EFR83939       ----------A------VSI-NK-AALE    A----E--IN-V--S---------
Listeria riparia                  WP_036102137   -----------------V---NK-AAIE    AT--E------V--S---------
Listeria rocourtiae               WP_133620013   -----------------V---NK-AAIE    AA--E------V--S---------
Listeria seeligeri                WP_012986168   ----------A------V-I-NK-AELE    A----E--IN-V--S---------
Listeria weihenstephanensis       WP_118907675   -----------------V---NK-AAIE    AT--E------V--S---------
Oceanobacillus caeni              WP_060668708   ----------A-----F--I-NK-AELK    L---E----S-V---I--------
Oceanobacillus damuensis          WP_067725022   ----------A------V---NK-AELN    L---E----S-V---I--------
Oceanobacillus halophilus         WP_121206154   ----------A-----YI---NK-AELN    ----E----N-V---I--------
Oceanobacillus limi               WP_090872621   -----------------V---KK-TDLA    L---E----N-V------------
Oceanobacillus massiliensis       WP_010651724   ----------A------V---NK-AELN    L---E----S-V---I--------
Oceanobacillus rekensis           WP_087974571   --------F--------I---NK-SELK    L---E------V---I--------
Oceanobacillus senegalensis       WP_085993402   ----------A-----YM---NK-AELK    -D--E----N-V---I--------
Ornithinibacillus californiens    WP_047986092   ----------A------V---NK-AGLE    LQ--E---A--V------------
Ornithinibacillus scapharcae      WP_010098030   ----------A------V---NK-ADLA    L---E---A--V------------
Parageobacillus thermantarctic    WP_090951258   ----------A------M---NK-AAIE    A---E------V---I--------
Parageobacillus thermoglucosid    Q8VV84         ----------A------M---NK-AAIE    A---E------V---I--------
Parageobacillus toebii            WP_062756391   ----------A------M---NK-AAIE    A---E------V---I--------
Quasibacillus thermotolerans      WP_039238080   -----------------V---NK-AE-E    A-------IN-V-------I----
Staphylococcus aureus             PPK02886       ----------A------VSI-NK-AA-E    AT---------V-K---A-I----
Staphylococcus haemolyticus       RIO77664       ----------A------V-I-NK-DEIE    A--------N-V-K--SA------
Terrabacteria group               WP_095251846   --------------V------NK-AEIQ    A-------IN-V---I-----T--
Thermolongibacillus altinsuens    WP_132949652   ----------A------M---NK-ASIE    A----------V---I--------
Vagococcus entomophilus           WP_126823490   -----------------V-ASTK-AEIK    S----------V-------I----
Virgibacillus proomii             WP_077318345   ----------A-----FM-I-HK-SELQ    L---E---AN-V---M--------
```

Supplemental Figure 4

A partial sequence alignment of the Chaperonin GroEL protein containing a two amino acid insertion (boxed) that is exclusively shared by all members among the *Planococcaceae* family and absent in all other bacteria. Only 2 species belonging to the genus *Salsuginibacillus* are exceptions which share this CSI.

Family *Planococcaceae* (>100/>100)

		21		65
Bacillus cecembensis	WP_057986972	LELVDVEFIKEGRDWFLRVYVDT	R	AGGIDILQCAQVSERLSEKLD
Bacillus ndiopicus	WP_042471991	--------V----NY---I----	P	E-----D----------VI--
Bacillus sp. FJAT-22090	WP_053589944	-----I-YV-----------I--	P	------E-------K------
Bacillus sp. OxB-1	WP_041074129	-----I--V---K-----I-I--	P	Q-D---D---V---K--TE--
Bhargavaea beijingensis	WP_092096533	-----I--V----------FI--	P	G-----E---A---Q---I--
Bhargavaea cecembensis	WP_008297176	-----I--V----------FI--	P	G-----E---A---Q--VR--
Bhargavaea ginsengi	WP_092048931	-----I--V----------FI--	P	E-----E---A---Q--VR--
Brevibacillus invocatus	WP_122907513	-----I-YK---SN-----FI-N	E	V-N---DD--L---K--Q---
Brevibacillus panacihumi	WP_023557116	-----I-YK---SN-----FI-N	E	V-N---ED--L---K--Q---
Caryophanon latum	WP_066462531	-V------V--------------	P	E-----E--V-------IV--
Caryophanon tenue	WP_066543445	-V---I--V--------------	P	E-----E--V-------LV--
Kurthia huakuii	WP_029498287	--------V--------------	P	E-N---D-------K------
Kurthia massiliensis	WP_010286321	--------V--------------	P	E-H---D--------------
Kurthia senegalensis	WP_010301413	--------V--------------	P	E-N---D-------K------
Kurthia sibirica	WP_109304557	--------V-----H--------	P	V-N---S-------K---L--
Kurthia zopfii	WP_109349254	--------V--------------	P	E-N---D-----------Y--
Kyrpidia spormannii	WP_100667789	-------YV---AN-----F--R	P	E--V--DD-SR------DR--
Kyrpidia tusciae	WP_013075538	-------YV---AN-----F--R	P	E--V--DD-SR------DR--
Lysinibacillus	WP_004269252	-----I--V----N---------	P	E-----D----------LL--
Lysinibacillus acetophenoni	WP_097148046	-----I-------N------I--	P	G-----D---L-------V--
Lysinibacillus chungkukjangi	WP_107932788	----EI--L----N------I--	P	E-----E---L----V--I--
Lysinibacillus composti	WP_124763974	-----I--V----N----I-I--	P	E-----D---L------VL--
Lysinibacillus contaminans	WP_053584304	----N---V--------------	P	E-----S----------LL--
Lysinibacillus endophyticus	WP_121213018	-----I--L----N------I--	P	E-----D---I------VV--
Lysinibacillus fusiformis	WP_069480371	-----I--V----N---------	P	E-S---D----------LL--
Lysinibacillus halotolerans	WP_122970394	-----I--L----N------I--	P	E-----D---I------LV--
Lysinibacillus jejuensis	WP_108305833	--------V---SIY--------	P	E-----D---L------VV--
Lysinibacillus macroides	WP_053993958	-----I-------N---------	L	E-----D----------LL--
Lysinibacillus manganicus	WP_036181476	-----I-------N------I--	P	S-----E---L-------Q--
Lysinibacillus massiliensis	WP_036171547	-----I--VR---N------I--	P	E-----D---L---K--VV--
Lysinibacillus meyeri	WP_107840990	--------V----NY---I----	P	Q-----D----------VI--
Lysinibacillus odysseyi	WP_036152348	--------V----N---------	P	E-----D----------LI--
Lysinibacillus parviboronicapiens	WP_107923051	-----I--V----N----I----	P	E-----D----------LL--
Lysinibacillus saudimassiliensis	CEA04536	--------V---SIY--------	P	------D---L------VV--
Lysinibacillus sinduriensis	WP_036203088	----EI--L----N------I--	P	E-----E---L-------I--
Lysinibacillus sp. 2017	WP_108711937	-----I--V---------I----	P	S-------------M------
Lysinibacillus sp. B2A1	AVK83204	-----I--V----N---------	P	E-----G-------K--LL--
Lysinibacillus sp. BF-4	WP_036141489	--------V---SIY--------	P	------D---L------VV--
Lysinibacillus sp. BK089	WP_132357403	--------V----N---------	P	Q-----E----------LL--
Lysinibacillus sp. FJAT-14222	WP_053594242	--------V----N---------	P	E-S---D----------LL--
Lysinibacillus sp. FJAT-14745	WP_053483266	--------V----N---------	P	E-----E----------LL--
Lysinibacillus sp. Marseille-P	WP_106784268	-----I--L----N------I--	P	D-----D---L------VV--
Lysinibacillus sp. SYSU K30002	WP_126657514	-----I--L----N------I--	P	E-----E---I------LV--
Lysinibacillus sp. YLB-03	WP_118874619	-----I--V----N------I--	L	E-----D---L-------V--
Lysinibacillus sp. ZYM-1	WP_054610348	-----I--V----N---------	P	E-----D----------LL--
Lysinibacillus sphaericus	WP_010858481	--------V----N---------	P	E-----D----------LL--
Lysinibacillus telephonicus	WP_126292997	-----I--L-----------I--	P	E-----D----------VV--
Lysinibacillus xylanilyticus	WP_068983121	--------V----N---------	S	E-----E----------LL--
Lysinibacillus xyleni	WP_097072174	-----I--V----N------I--	P	E-----D---I------LV--
Sporosarcina koreensis	WP_040286829	-----I--L-----------I--	P	EAD---D-----------E--
Sporosarcina newyorkensis	WP_009497359	--------V-----------I--	P	E-N---D---A---K---E--
Sporosarcina pasteurii	WP_115360489	-----I--V---N----------	P	E-D---D---L-------E--
Sporosarcina psychrophila	WP_067211160	--------V-----------I--	P	N-S---D--S----K---E--
Sporosarcina sp. BI001-red	WP_116016412	-----I--L-----------I--	Q	E-N---------------E--
Sporosarcina sp. EUR3 2.2.2	WP_024534995	---I-L--VR---N----I----	P	Q-----EH--L----V--V--
Sporosarcina sp. HY008	WP_067408188	-----I--V-----------I--	P	E-N---D---L-------E--
Sporosarcina sp. P13	WP_099687081	--------L---------I-I--	P	E-A---D---A---K---E--
Sporosarcina sp. P18a	WP_099675119	--------L-----------I--	P	E-N---D---A--NK---E--
Sporosarcina sp. P34	WP_099694180	--------L-----------I--	P	E-N---D---I--NK---E--
Sporosarcina sp. PTS2304	WP_114923358	--------L---------I-I--	P	E-A---D---A---K---E--
Chryseomicrobium excrementi	WP_100352717	-------YV---------I---V	P	G-S---E---R----V--I--
Planococcus antarcticus	WP_006828245	--------L----N--------N	P	EAP---D---L---K---I--
Planococcus citreus	WP_121297650	--------V---KN--------N	P	QEP---D---I-------E--
Planococcus donghaensis	WP_008430872	--------L-------------N	P	EAP---E---L---K--GI--
Planococcus halocryophilus	WP_008497942	--------L----------F--N	P	EAP---E---L---K--GI--
Planococcus halotolerans	WP_112222792	--------V----S--------N	P	DAP---D---L-------I--
Planococcus maitriensis	WP_112231499	--------V---KN--------N	P	QEP---D---I-------E--
Planococcus maritimus	WP_068462828	--------V---KN--------N	P	QEP---D---I-------E--
Planococcus massiliensis	WP_052651272	--------V----S-----F--N	P	EAP---E---V---K---I--
Planococcus plakortidis	WP_068869381	--------V---KN--------N	P	QEP---D---I-------Q--
Planococcus rifietoensis	WP_058380468	--------V---KN--------N	P	QEP---D---I-------E--
Planococcus salinarum	TAA67493	--------V----S--------N	P	EAP---E---S-------I--
Planococcus salinus	WP_123164297	-----I--V----S--------N	P	EAP---E---L-------I--
Planococcus sp. CAU13	WP_033542075	--------V----S--------N	P	EAP---D---L-------I--

Group	Organism	Accession	Alignment	Boxed position	Alignment (cont.)
**Family *Planococcaceae* (>100/>100)**	Planococcus sp. PAMC 21323	WP_038704247	--------L----------F--N	P	EAP---E---L---K--GV--
	Planococcus sp. Y42	WP_077589619	--------V---KN--------N	P	EST---E---A---K---E--
	Planococcus versutus	WP_049693266	--------V----T--------N	P	DAP---D---L---K---I--
	Planomicrobium flavidum	WP_088005962	-----T--V---KN--------N	P	D-PM--E---V---K---E--
	Planomicrobium glaciei	WP_036806649	--------L----S--------N	P	-AP---D---L---K---I--
	Planomicrobium okeanokoites	WP_117312420	--------V----S--------N	P	DAP---D---L-------I--
	Planomicrobium soli	WP_106531885	--------L----N--------N	P	EAP---D---L---K---V--
	Planomicrobium sp. Y74	WP_121632392	--------V----S--------N	P	DAP---D---L-------I--
	Paenisporosarcina indica	WP_075617558	---I-T--V----N----I----	P	Q-----EH--L----I--V--
	Paenisporosarcina sp. HGH0030	WP_016427602	-----T--V----N----I----	P	Q-----EH--L----I--V--
	Paenisporosarcina sp. K2R23-3	WP_119883018	-----M--V----N----I----	P	S-----E---L---KV--M--
	Paenisporosarcina sp. OV554	WP_108584655	---I-L--VR---N----I----	P	Q-----EH--L----V--V--
	Paenisporosarcina sp. TG20	WP_019413990	-----L--V----S----I----	H	E-----EH--L-------V--
	Psychrobacillus insolitus	WP_111438449	----EI-YL---Q-------I--	P	S-R---E-------K---I--
	Psychrobacillus psychrotoleran	WP_093535083	-D---I-YV---------I-I--	P	N-----E-------K------
	Psychrobacillus sp. FJAT-21963	WP_056829311	-----I-YV-----------I--	P	------E-------K------
	Psychrobacillus sp. OK028	WP_093060172	-D---I-YV---------I-I--	P	T-----E-------K------
	Psychrobacillus sp. OK032	WP_093263085	---I-I-YV---------I-I--	P	------E-------K------
	Rummeliibacillus pycnus	WP_102692627	----E---V--------------	P	E-N---E-------L--V---
	Rummeliibacillus stabekisii	WP_066785707	----EI--V----E---------	P	E-S---E-------L------
	Seinonella peptonophila	WP_073157411	---F-I-YS---AN-----FI-L	P	G-AVNLDH-SK---K--KE--
	Solibacillus isronensis	WP_079525284	-----I--V---------I----	P	E--------------------
	Solibacillus sp. R5-41	WP_099422816	-----I-----------------	-	---------------------
	Edaphobacillus lindanitolerans	WP_076756505	-----I--V----E-----FI--	P	G-----D---A---Q------
	Filibacter sp. TB-66	WP_124070888	-----I--V---K-------I-N	S	E-L---D-----------E--
	Tetzosporium hominis	WP_094941664	-------YV---------I---V	P	G-----E---R----V--L--
	Ureibacillus thermophilus	QBK25670	-----I--V----A------I--	P	Q-----D---L------AL--
	Ureibacillus thermosphaericus	WP_026018948	-----I--V----A------I--	P	Q-----D---L------AV--
	Viridibacillus arvi	WP_053417436	----E---V-----------I--	P	E-S---E-------L--L---
	Viridibacillus sp. OK051	WP_100796517	----E---V-----------I-N	P	E-S---E-------L--L---
**Other Bacteria (0/>200)**	Amphibacillus jilinensis	WP_017471922	-T---I--V---KS-----FI-K		S--V--EE-GR---Q---R--
	Amphibacillus sediminis	WP_067838491	----E---T---KN-----FI-K		P--V--EE-ER---Q---R--
	Amphibacillus xylanus	WP_015010193	-----I-YV---K------FI-K		P--V--EE-G-L---------
	Anoxybacillus amylolyticus	WP_066323912	-----I-YV---KN-----FI-S		EE----E--GV---K------
	Anoxybacillus ayderensis	WP_042533893	-----I-YA---KN-----FI-S		PT----D--GV----------
	Anoxybacillus flavithermus	AST07500	-----I-YV---KN-----FI-S		PT-V--D--GV----------
	Anoxybacillus flavithermus WK1	ACJ34060	-----I-YV---KN-----FI-S		PT----D--GV---Q------
	Anoxybacillus geothermalis	WP_044741278	-----I-YV---KN-----FI-S		DT-V--E--GV----------
	Anoxybacillus gonensis	WP_009732925	-----I-YV---KN-----FI-S		PT----D--GV----------
	Anoxybacillus pushchinoensis	WP_091699958	-----I-YV---KN-----FI-S		PT----D--GV----------
	Anoxybacillus sp. 103	WP_077429654	-----I-YV---KN-----FI-S		PT-V--D--GV----------
	Anoxybacillus sp. BCO1	KHF30962	-----I-YV---KN-----FI-S		PT----D--GV----------
	Anoxybacillus suryakundensis	WP_032099687	-----I-YV---KN----IFI-S		PT-V--D--GV---Q------
	Anoxybacillus tepidamans	WP_027408723	-----I-YV---KN-----FI-S		EQ----E--GV---K------
	Anoxybacillus thermarum	WP_043964733	-----I-YV---KN-----FI-S		PT----D--GV----------
	Anoxybacillus vitaminiphilus	WP_111643760	-----I-YV---KN-----FI-S		ED----E--GV---K------
	Bacillus acidiceler	WP_088012923	-------YV---K------FI-S		ES---LE---A---K---V--
	Bacillus alkalitelluris	WP_078547058	-----I-YV---K------FI-S		E--V--ED-GT---K------
	Bacillus alveayuensis	WP_044893734	-----I-YV---KN-----FI-S		ED----E--GV---K------
	Bacillus amyloliquefaciens	KJD55131	-----I--V---PS-----FI-S		DD-V--EE--K---A------
	Bacillus aryabhattai	WP_047749910	----EI-YV---K------FI-S		DT-V--ED-GA---K----M-
	Bacillus boroniphilus JCM 2173	GAE43700	-----I-YV---KN------I-K		DN----EE-GI----------
	Bacillus cellulasensis	WP_041090498	--------V---QN----IFI-S		DK-V--EE--K---A------
	Bacillus cereus	WP_061129300	-------YV---Q------FI-S		EK-V--EE-GA-------A--
	Bacillus cereus Rock3-44	EEL49789	-------YV---K------FI-S		EK-V--EE-GA-------A--
	Bacillus cereus group	WP_000359094	-------YV---K------FI-S		EK-V--EE-GA-------A--
	Bacillus chagannorensis	WP_035210775	--------T---KH-----FI-S		PT-V-LDD-TK---Q------
	Bacillus cytotoxicus	WP_012094909	-------YV---K------FI-S		ET-V--EE-GA-------A--
	Bacillus deserti	WP_101639943	-----I-YV---KN-----FI-K		E--V--EE-GT----------
	Bacillus endophyticus	WP_124049363	-------YV---K------FI-S		EK-V--EE-GV--------M-
	Bacillus firmus	WP_035329036	-----I-YV---K------FI-K		ET-V--EE-GM----------
	Bacillus glycinifermentans	WP_048354591	-----I--V---QN-----FI-S		DD-V--EE--K---A------
	Bacillus gobiensis	WP_053602841	--------V---QN-----FI-S		EN-V--EE--N---A------
	Bacillus gottheilii	WP_066446516	-----I-YV---K------FI-K		ET-V--EE-GM----------
	Bacillus halotolerans	WP_069486536	-----I--V---QS-----FI-S		DD-V--EE--K---A------
	Bacillus haynesii	WP_043926402	-----I--V---QS-----FI-S		DD-V--EE--K---A------
	Bacillus horneckiae	WP_066393662	-----I-YV---K-----IFI-K		EN-V--EE-GL---Q------
	Bacillus indicus	WP_029281263	-----I--V---KE-----FI-S		SD----EH--I---K------
	Bacillus koreensis	WP_053401673	I----I-YV---K------FI--		EN----E--GI-------IM-
	Bacillus kribbensis	WP_026692453	-----I-YL---KN-----FI-K		E-----DE-GA----------
	Bacillus licheniformis	WP_035334067	-----I--V---QS-----FI-S		DD-V--EE--K---A------
	Bacillus luciferensis	WP_088069522	-------YV---K------FI-S		ES---LE---A---K---I--
	Bacillus marinisedimentorum	WP_066173105	M-------V---KN-----FI-S		PE-V--ED-GK-------Q--
	Bacillus marisflavi	WP_121619595	-----I-YV---TN-----F--K		PT-V--EE-GL----------

Other Bacteria (0/>200)

Bacillus massilioanorexius	WP_019242270	-------YV---K------FI-K	ET-V--EE-GV---K--V---
Bacillus massiliogabonensis	WP_102271422	-----I-YV---------IFI-K	ET-V--EE-GL---Q--V---
Bacillus massiliogorillae	WP_042348867	-------YV---KN-----FI-K	DA-V--EE-SV---Q------
Bacillus massiliosenegalensis	WP_019152751	-----I-YV---K------FI-K	D--V--EE-GL----------
Bacillus megaterium	AUO10892	----EI-YV---K------FI-S	DT-V--ED-GA---K----M-
Bacillus megaterium WSH-002	AEN87956	----EI-YV---K------FI-S	DT-V--ED-GA---K----M-
Bacillus mojavensis	WP_010334281	-----I--V---QS-----FI-S	DD-V--EE--K---A------
Bacillus nakamurai	WP_061520555	-----I--V---QN-----FI-S	DD-V--EE--K---A------
Bacillus oceanisediminis	WP_110065655	-----I-YV---K------FI-K	ET-V--EE-GL---K------
Bacillus oryziterrae	WP_017753795	-----I--V---KN-Y-K-FI-S	EK----ED-GN---Q------
Bacillus panaciterrae	WP_028399017	-------YV---K------FI-S	DA-V--EE--A-------A--
Bacillus praedii	WP_057763242	-----I-YV---------IFI-K	ET-V--EE-GL---Q--V---
Bacillus pumilus	OLP66404	-----I--V---QN-----FI-S	DK-V--EE--K---A------
Bacillus safensis	WP_081124382	--------V---QN----LFI-S	DK-V--EE--K---A------
Bacillus solisilvae	WP_087998511	-------YV---K------FI-N	ES---LE---A-------I--
Bacillus sonorensis	WP_006638132	-----I--V---QN-----FI-S	DD-V--EE--K---A------
Bacillus sp. WP8	WP_039178644	--------V---QN-----FI-S	DK-V--EE--K---A------
Bacillus stratosphericus LAMA	EMI13365	--------V---QN-----FI-S	DK-V--EE--K---A------
Bacillus subtilis	WP_015252085	-----I--V---QS-----FI-S	DD-V--EE--K---A------
Bacillus subtilis group	WP_003328902	-----I--V---QN-----FI-S	DN-V--EE--K---L------
Bacillus swezeyi	WP_076760555	-----I--V---PN-----FI-S	ED-V--EE--K---A------
Bacillus testis	WP_050614813	-----I--V---KN-----FI-K	EA-V--EE-GI---K------
Bacillus thuringiensis	WP_098856128	-------YV---Q------FI-S	EK-V--EE-GA-------A--
Bacillus tuaregi	WP_071395302	-----I-YV---K-------I-K	EA----EE-GL------D---
Bacillus vallismortis	WP_010328009	-----I--V---QS-----FI-S	DD-V--EE--K---A------
Bacillus xiamenensis	WP_008354452	--------V---QN-----FI-S	EK-V--EE--K---A------
Caldalkalibacillus thermarum	WP_007505996	--------K----K-Y----I-K	P--V--ED-SA---S--A---
Carnobacterium divergens	WP_034570000	F-------V---KN----T-I-K	P-----EE--L---KI--RM-
Carnobacterium divergens DSM 2	KRN54180	F-------V---KN----T-I-K	P-----EE--L---KI--RM-
Carnobacterium gallinarum	WP_034564667	F-------V---KN----T-I-K	P-----EE--LI--KI---M-
Carnobacterium maltaromaticum	KRN65740	F-------V---KS----M-I-K	P-----ED--L---KI---M-
Effusibacillus lacus	WP_096182287	-------YA---AN-----FI-K	P--V--ED-GR---V--A---
Enterococcus avium	WP_127978813	F---E---V---KS-----FI-K	E-----EE--F---K------
Enterococcus canintestini	OJG15334	F---E---V---KS-----FI-K	E-----EE--Y---K------
Enterococcus canis	WP_067392190	F---E---V---KS-----FI-K	E-----EE--F----------
Enterococcus casseliflavus	OJG31195	F---E---V---KN-----FI-K	E-----EE--F---Q---Q--
Enterococcus casseliflavus ATC	EGC69210	F---E---V---KN-----FI-K	E-----EE--F---Q---Q--
Enterococcus cecorum	WP_016252350	F---E---V---KS-Y---FI-K	E-----EE--L---Q------
Enterococcus columbae	WP_016183004	F---E---V---KS-----FI-K	E-----EE--W---Q------
Enterococcus columbae DSM 7374	OJG24642	F---E---V---KS-----FI-K	E-----EE--W---Q------
Enterococcus dispar	OJG38052	F---E---V---KN-----FI-K	E-----EE--Y---K------
Enterococcus faecalis	OSH20825	F---E---V---KN-----FI-K	E-----EE--F---K------
Enterococcus faecalis CBRD01	ESU75571	F---E---V---KN-----FI-K	E-----EE--F---K------
Enterococcus faecalis R508	EJV37171	F---E---V---KN-----FI-K	E-----EE--F---K------
Enterococcus faecalis TX0104	EEI11974	F---E---V---KN-----FI-K	E-----EE--F---K------
Enterococcus faecalis TX1302	EFU09500	F---E---V---KN-----FI-K	E-----EE--F---K------
Enterococcus faecium	WP_002338380	F---E---V---KS-----FI-K	E-----EE--F---K------
Enterococcus faecium R497	EJX58383	F---E---V---KS-----FI-K	E-----EE--F---K------
Enterococcus faecium TX0133a01	EFR69170	F---E---V---KS-----FI-K	E-----EE--F---K------
Enterococcus faecium TX1337RF	EJV42860	F---E---V---KS-----FI-K	E-----EE--F---K------
Enterococcus hermanniensis	OJG45159	F---E---V---KS-----FI-K	E-----EE--F---K--D---
Enterococcus massiliensis	WP_048603011	F---E---A---KS-----FI-K	E-----EE--F---Q------
Enterococcus mundtii	WP_010735193	F---E---V---KS-----FI-K	E-----EE--F---K------
Enterococcus pallens	WP_010759454	F---E---V---KN-----FI-K	E-----EE--F---K------
Enterococcus phoeniculicola	OJG71443	F---E---V---KN------I-K	D-----EE--L----------
Enterococcus pseudoavium	WP_067623586	F---E---V---KS-----FI-K	E-----EE--F---Q--D---
Enterococcus rivorum	WP_069698835	F---E---V---KN-----FI-K	D-----EE--F---Q---R--
Enterococcus sulfureus	WP_016185654	F---E---V---KS----IFI-K	E-----EE--Y---QV-----
Enterococcus wangshanyuanii	WP_088269148	F---E---V---KN-----FI-K	N-----EE--L---Q------
Fictibacillus gelatini	WP_026675840	--------V---KN-----FI-S	EK-V--EE-GV---Q------
Geobacillus genomosp. 3	WP_020959384	-----I-YV---KN-----FI-S	DT-V--E--GV---K------
Geobacillus icigianus	WP_033023085	-----I-YV---KN-----FI-S	EN-V--E--GV---K------
Geobacillus jurassicus	WP_066230017	-----I-YV---KN-----FI-S	DD-V--E--GA---K------
Geobacillus kaustophilus	WP_044731694	-----I-YV---KN-----FI-S	DD-V--E--GA---K------
Geobacillus lituanicus	WP_094239896	-----I-YV---KN-----FI-S	DT-V--E--GV----------
Geobacillus sp. 46C-IIa	WP_081206904	-----I-YV---KN-----FI-S	DD-V--E--GV---K------
Geobacillus sp. C56-T3	WP_013145731	-----I-YV---KN-----FI-S	DD-V--E--GA---K------
Geobacillus sp. LC300	AKU27736	-----I-YV---KN-----FI-S	DT-V--E--GV----------
Geobacillus sp. MAS1	ESU71621	-----I-YV---KN-----FI-S	DD-V--E--GA---K------
Geobacillus sp. T6	WP_047753361	-----I-YV---KN-----FI-S	DD-V--E--GA---K------
Geobacillus sp. WSUCF1	EPR26981	-----I-YV---KN-----FI-S	DD-V--E--GA---K------
Geobacillus thermodenitrifican	AB066487	-----I-YV---KN-----FI-S	DD-V--E--GV---K------
Geobacillus vulcani	WP_031410362	-----I-YV---KN-----FI-S	DD-V--E--GA---K------
Halobacillus hunanensis	WP_079529881	-----I--K---KN-----FL-K	PE----EE-G----K------

Other Bacteria (0/>200)

Halobacillus litoralis	WP_128522727	-----I-YK---QN-Y---FI-K	E--V--EE-G----Q------
Halobacillus mangrovi	WP_085029937	-------YK---SN-Y---FI-K	P--V--EE-G----Q------
Halobacillus massiliensis	WP_082234161	-----I--K---KN-----F--K	P-----EE-GK---Q------
Halobacillus sp. BBL2006	WP_035544487	-----I-YKH--SN-Y---FI-K	P--V--EE-G----Q------
Halobacillus sp. SKP4-6	WP_128539749	-----I-YKQ--QN-Y---FI-K	PN-V--EE-G----Q------
Lactobacillus apodemi	WP_025087511	F-------V---KS-Y---FI-K	D---N-EE--L--DQ------
Lactobacillus backii	WP_068224638	V----I--V---KS-Y----I-K	P-----EE--L---KV--Q--
Lactobacillus bifermentans	WP_057905163	F---E---V---K--Y----I-K	P---NVEE-----DL------
Lactobacillus fabifermentans	WP_024625332	F----I--VR--QS-Y--I-I-K	P---N-EE--M--DE------
Lactobacillus hammesii	WP_057731889	F-------V---KS-Y--L-I-K	P---N-EE--M--DE------
Lactobacillus hammesii DSM 163	KRL97643	F-------V---KS-Y--L-I-K	P---N-EE--M--DE------
Lactobacillus herbarum	WP_047999872	F----M--VR--QS------I-K	P---N-EE--M--DE------
Lactobacillus iwatensis	WP_125982464	V----I--V---KS-Y----I-K	P-----EE--L---KV--Q--
Lactobacillus mudanjiangensis	WP_130843290	F-------VR--QS-Y----I-K	P---N-EE--M--DE------
Lactobacillus namurensis	WP_056943560	FD------V---KS-Y----I-K	P---N-EE--T--DE------
Lactobacillus paraplantarum	WP_021730803	F----L--VR--QS-Y--I-I-K	P---N-EE--M--DE------
Lactobacillus saerimneri	WP_009554899	FD-F-I--V---KS-Y----I-K	P---N-EE--L--DQ------
Lactobacillus salivarius	WP_049154014	F-------V---KS-Y----I-K	PN--N-EE--L--DK------
Lactobacillus satsumensis	WP_056960219	F----I--V---KS-Y---FI-K	PA--N-EE--L--DQ---A--
Lactobacillus sp. 151-2B	WP_125572813	F-------V---KS-Y----I-K	P---N-EE--M--DE------
Lentibacillus sp. Marseille-P4	WP_106497590	-----I-YV---KN------I-K	D-----AE-G----Q---R--
Lentibacillus sp. SSKP1-9	WP_129674135	-----I-YV---KN------I-K	D--V--NE-GI---Q------
Listeria grayi	WP_003756098	V----IAYE---KN-----F--R	P-----DE--A---K----M-
Melissococcus plutonius	WP_013774170	F--I--------KS-Y---FI-K	K-----EE--F---Q---Q--
Oceanobacillus bengalensis	WP_121129167	-----I-YV---KN------I-K	P-----AE-GE---Q------
Oceanobacillus caeni	WP_047186079	-----I-YV---KN----I---K	P--V--TE-GE---Q------
Oceanobacillus halophilus	WP_121202598	-----I-YV---KN------I-K	P--V--TE-GE---Q--D---
Oceanobacillus iheyensis	WP_106896683	-------YV---KN------I-K	P--V--TE-GE---E------
Oceanobacillus oncorhynchi	WP_084612998	-----I-YV---KN------I-K	E-----VE-GE---Q------
Oceanobacillus picturae	CDO02197	-------YV---KN------I-K	P--V--TE-GE---E------
Oceanobacillus senegalensis	WP_085992381	-----I-YV---KN------I-K	P--V--IE-GE---Q------
Oceanobacillus sp. Castelsardo	WP_068672365	-----I-YV---KN----I-I-K	P--V--TE-GE---Q------
Ornithinibacillus californiens	WP_047984847	-----I-YV---KN-----FI-K	E-----SE-GE----------
Ornithinibacillus contaminans	WP_047981235	-----I-YV---KN-----FI-K	E-----AE-GD---Q------
Ornithinibacillus scapharcae	WP_010093377	-----I-YV---KN----IFI-K	E-----AE-GE---Q------
Parageobacillus thermantarctic	WP_090947993	-----I-YV---KN-----FI-S	DE-V--E--GV---K------
Parageobacillus thermoglucosid	WP_064551001	-----I-YV---KS-----FI-S	DE-V--E--GA---K------
Paraliobacillus quinghaiensis	WP_117152420	-Q---I--E---KN-----FI-K	P--V--EE-G----Q---Q--
Paraliobacillus ryukyuensis	WP_079709483	-----I--E---KN-----FI-K	P--V--EE-G----Q------
Pelagirhabdus alkalitolerans	WP_090793078	----ET--K---K------FI-K	P--V--EE-GR---Q---A--
Pontibacillus halophilus	WP_026799813	--------TQ--KN--------K	E-----EE-G----Q------
Pontibacillus litoralis	WP_036831902	-----I--V---KN-Y---FA-K	D---E-EE-G----Q--A---
Salirhabdus sp. Marseille-P466	WP_102028254	-----I--V---KN--------K	D-----EE-GI---Q------
Scopulibacillus darangshiensis	WP_132744904	-------YV---QN-----FI-S	PA-V-LDT---------D---
Sedimentibacter sp. SX930	WP_086627875	-----M--L---KN----I-I-K	P-----EE--LI--KV--A--
Sediminibacillus halophilus	WP_074597338	-----I--E---KN-----F--K	E--V--EE-G----Q------
Tetragenococcus halophilus	WP_014124666	F----L--V---KN-Y---FI-K	E-----EE--Y---Y------
Tetragenococcus koreensis	WP_124005424	F----L--V---KS-Y---FI-K	E-----EE--Y---Y--D---
Thermolongibacillus altinsuens	WP_132948178	-----I-YV---KN-----FI-S	ET-V--E--GM---Q------
Trichococcus collinsii	WP_086984778	-----M--L---KN----I-I-K	P-----EE--LI--KV-GA--
Trichococcus ilyis	WP_068620336	-----M--L---KN----I-I-K	P-----EE--L---KV--A--
Trichococcus paludicola	WP_107995206	-----M--L---KN----I-I-K	P-----EE--LI--KV--A--
Trichococcus palustris	WP_087031757	F----L--V---KN----I-I-K	P-----EE--LI--KV-DA--
Trichococcus patagoniensis	WP_108032599	-----M--L---KN----I-I-K	P-----EE--LI--KV--A--
Vagococcus acidifermentans	WP_126815133	F------YV---KN-----FI-K	P-----EE-VI---E--A-M-
Vagococcus elongatus	WP_126809355	F----I--V----N------I-K	PN----EE--I---Q----M-
Vagococcus entomophilus	WP_126823232	F-------V---KN-----FI-K	P-----EE--L---K----M-
Vagococcus humatus	WP_125942364	F-------V---KN-----FI-K	PK----EE--L---K---IM-
Vagococcus sp. SS1995	WP_125984038	F----I-YV----------FI-K	S-----NE-VL---Q--DI--
Virgibacillus alimentarius	WP_029266535	-----I-YV---K------FI-K	E-----AE-GA---L-G----
Virgibacillus halodenitrifican	WP_019375735	-----I-YV---KN----I-I-K	P-----TE-GD---E------
Virgibacillus indicus	WP_094883881	-----I-YV---KN------I-K	P-----TE-GD---A------
Virgibacillus senegalensis	WP_053219623	-----I--E---KN-----F--K	E--V--EE-G----Q------
Virgibacillus sp. SK37	WP_040955637	-----I-YV---KN----I-I-K	P-----TE-GD---E------

Supplemental Figure 5

A partial sequence alignment of the ribosome maturation factor RimP protein containing a one amino acid insertion (boxed) that is exclusively shared by all members among the *Planococcaceae* family and absent in all other bacteria.

Family *Planococcaceae* (>100/>100)

		29		82
Bacillus cecembensis	WP_057987839	RPDHLVTVFAGQDKEATNVARHLFG	E	DHIPSSPSFVLLKDGQVVAEVGRYEIEG
Bacillus ndiopicus	WP_042475353	---Y-------------AA--YH--	-	--L-------------------------
Bacillus sp. OxB-1	WP_041075040	-----------------AQ--MH--	D	--L------A-----KL---I--H----
Bhargavaea beijingensis	WP_092097392	---Q-------------AQ--MH--	D	--L------I-V---K--G--P-H----
Bhargavaea cecembensis	WP_040228661	-----------------AQ--MH--	D	--L----A---M---K--G--P-H----
Bhargavaea ginsengi	SEJ18759	---S----------D--AQ--MH--	D	--L--------V---K--G--P-H----
Caryophanon latum	WP_066464383	-----------------AE--Y---	-	----------------------------
Caryophanon tenue	WP_066542432	-----------------AE--Y---	-	----------------------------
Kurthia gibsonii	WP_087680232	-----------------AM--AF--	-	-----------M---------P-H----
Kurthia huakuii	WP_029499091	----F------------AM--M---	-	-----------M----A---LP-HQ---
Kurthia massiliensis	WP_010287802	---RF------------AM--MF--	-	-----------M----T---IP-H----
Kurthia senegalensis	WP_010303115	----V------------AM--MF--	-	-----------MNN-KAI--IP-H----
Kurthia sibirica	WP_109305842	----F------------AM--MF--	-	---------I-M----P----P-H----
Kurthia sp. 11kri321	AMA62922	-----------------AM--AF--	-	-----------M---------P-H----
Kurthia zopfii	WP_109350015	----F------------AM--MF--	-	-----------M----A----P-H----
Lysinibacillus acetophenoni	WP_097148184	-----------------AA--YM--	-	--L---------R--K----I--H----
Lysinibacillus chungkukjangi	WP_107934873	-----------------AA---F--	D	E------A-------K---D---H----
Lysinibacillus composti	WP_124761872	-----------------AA---F--	-	-----------M---N-------H----
Lysinibacillus contaminans	WP_053582953	-----------------AA--YH--	-	--L-------------------------
Lysinibacillus endophyticus	WP_121214829	--N--------------AA--YF--	D	N--------------K---DI--H----
Lysinibacillus halotolerans	WP_122973305	-----------------AA---F--	-	N------A-------K---DI--H----
Lysinibacillus jejuensis	WP_108307152	-----------------AA--YH--	-	--L------------E------------
Lysinibacillus manganicus	WP_036186696	-----------------AA--YM--	-	---------------K---DI--H----
Lysinibacillus massiliensis	WP_036173658	--N--------------AA--YF--	-	---------------K---DI--H----
Lysinibacillus meyeri	WP_107838579	---Y-------------AA--YH--	-	--L-------------------------
Lysinibacillus odysseyi	WP_036158251	-----------------AA--YH--	-	E-L------I-------------H----
Lysinibacillus parviboronicapi	WP_054767492	--------------D--AA--YH--	-	--L-------------------------
Lysinibacillus saudimassiliens	CEA05033	-----------------AA--YH--	-	--L------------E------------
Lysinibacillus sinduriensis	WP_036202108	-----------------AA------	D	N------A-------K---DI--H----
Lysinibacillus sp. 2017	WP_108713087	-----------------AA--YY--	-	-------------------D--------
Lysinibacillus sp. B2A1	AVK84568	--N--------------AA--YH--	-	--L-------------------------
Lysinibacillus sp. BF-4	WP_036144979	-----------------AA--YH--	-	--L------------E------------
Lysinibacillus sp. FJAT-14222	WP_053594593	-----------------AA--YH--	-	--L-------------------------
Lysinibacillus sp. Marseille-P	WP_106779775	-----------------AA--YM--	-	-------------------DI--H----
Lysinibacillus sp. SYSU K30002	WP_126657021	------------------A------	D	N--------------K---DI--H----
Lysinibacillus sp. YLB-03	WP_118875462	-----------------AT------	-	---------------K---DI--H----
Lysinibacillus sp. YR326	TDV02804	---N-------------AA--YH--	-	--L-------------------------
Lysinibacillus sp. ZYM-1	WP_054610476	-----------------AA--YH--	-	--L-------------------------
Lysinibacillus sphaericus	WP_010859724	--------------D--AA--YH--	-	--L------I------------------
Lysinibacillus sphaericus C3-4	ACA40976	-----------------AA--YH--	-	--L-------------------------
Lysinibacillus telephonicus	WP_126294070	-----------------AA---F--	D	N--------------K---DI--H----
Lysinibacillus xylanilyticus	WP_049664240	-----------------AA--YH--	-	--L-------------------------
Lysinibacillus xyleni	WP_097072983	-----------------AA---F--	-	---------------K---DI--H----
Paenisporosarcina indica	WP_075618028	-----L-----------AQ--MI--	D	--L--------M----A------H----
Paenisporosarcina sp. HGH0030	WP_016426995	--N--------------AQ--M---	D	--L-------------A--DI--H----
Paenisporosarcina sp. K2R23-3	WP_119883259	---R-L-----------A---DI--	-	--L------------KP-G-IA-H----
Paenisporosarcina sp. OV554	WP_108586795	--N--------------AQ--M---	-	E-L-------------AI--I--H----
Paenisporosarcina sp. TG20	WP_019415008	-N--------------AQ--M---	D	E-L----------------D---H----
Planococcus antarcticus	WP_006831450	---N-Y-----------AQ--AI--	D	--L-------F----KM-D-I--H----
Planococcus donghaensis	WP_008430005	---N-F-----------AQ--AI--	D	--L-------F----KM-D-I--H----
Planococcus maitriensis	WP_112232228	-----Y-----------AQ--A---	D	--L------AF----KL-D-I--H----
Planococcus maritimus	WP_068462107	-----F-------Q---AQ--D---	D	--L------AF----KL-D-I--H----
Planococcus massiliensis	WP_052651720	---R-------------AQ--AI--	-	--L-------FM---KM-D-I--H----
Planococcus plakortidis	WP_068869094	-----Y-----------AQ--A---	D	--L------AF----KL-D-I--H----
Planococcus salinarum	OHX50581	---N-Y-----------AQ--DI--	D	--L------AIV---KM-G-I--H----
Planococcus salinus	WP_123163845	---R-L-----------AQ--M---	D	--L------A-V---KL-D-I--H----
Planococcus sp. CAU13	WP_033542368	---N-Y-----------AQ--AI--	D	--L------AIV---KM-D-I--H----
Planococcus sp. MB-3u-03	WP_101190267	-----Y-----------AQ--D---	D	--L------AF----KL-D-I--H----
Planococcus sp. PAMC 21323	WP_038703770	---N-F-----------AQ--AI--	D	--L-------F----KM-D-I--H----
Planococcus sp. Y42	WP_077588927	-----Y-----------AQ--A---	-	--L------A-------------H----
Planococcus versutus	WP_065524741	---N-F-----------AQ--AI--	D	--L-------F----KM-D-I--H----
Planomicrobium flavidum	WP_088006395	-----Y-----------AQ--D---	-	--L------A------L------H----
Planomicrobium glaciei	WP_036809211	---R-L-----------AQ--AI--	-	--L------------K-AD-I--H----
Planomicrobium soli	WP_106532613	---N-Y-----------AQ--AI--	D	--L------A-M---KM-G-I--H----
Solibacillus isronensis	WP_079527551	-----------------AA--YY--	-	---------I--------G----F----
Solibacillus kalamii	WP_087616239	---Y-------------AA--YY--	-	---------I--------G----F----
Solibacillus sp. R5-41	WP_099424329	------------------A--Y---	-	----------------------------
Sporosarcina koreensis	WP_040286562	-----------------AQ--MH--	-	Y-L------A-M---EM------H----
Sporosarcina newyorkensis	WP_009496767	-----------------AQ--MH--	-	---------A-----KL-S----F----
Sporosarcina pasteurii	WP_115361128	-----------------AQM-MH--	D	--L------A-----KL-----------
Sporosarcina psychrophila	WP_067209130	-----------------AQ--M---	-	--L------------KL-----------
Sporosarcina sp. BI001-red	WP_116016985	----------------AQ--MH--	D	Y-L--------M-N-EM------H----
Sporosarcina sp. D27	WP_025784697	-----------------AQ--MH--	D	Y-L--------M---EM------H----
Sporosarcina sp. EUR3 2.2.2	WP_024534577	-----------------AQ--M---	D	E-L-------------A---I--H----

Family *Planococcaceae* (>100/>100)

```
Sporosarcina sp. HY008              WP_067403726   -----------------AQ--MH-- - --L------A-----KL---I--H----
Sporosarcina sp. P1                 WP_099626557   -----------------AQ--MH-- V ---------A-----KLIG----F----
Sporosarcina sp. P13                WP_099689400   -----------------AQ--MH-- - ---------A-----KLIG---------
Sporosarcina sp. P26b               WP_099693640   -----------------AQ--MH-- - ---------A-----KLIG----F----
Sporosarcina sp. P34                WP_099694556   -----------------AQ--MH-- - ---------A-----KLIG----F----
Sporosarcina sp. PTS2304            WP_114923702   -----------------AQ--MH-- - ---------A-----KLIG----F----
Sporosarcina sp. ZBG7A              WP_039043858    ----------------AQ--MH-- D Y-L--------M-N-EM------H----
Sporosarcina ureae                  WP_029054583   -----------------AQ--MH-- - ---------A-----KLIG----F----
Psychrobacillus insolitus           WP_111437970   --------------L--QQ--NI-- D E-L-------------M------H----
Psychrobacillus psychrotoleran      WP_093534047   --------------L--QQ--N--- D --L-------------P---I--H----
Psychrobacillus sp. OK028           WP_093060764   --------------L--QQ--N--- - --L-------------P---I--H----
Psychrobacillus sp. OK032           WP_093265621   --E-----------L--QQ--N--- D --L-------------PI-----H----
Rummeliibacillus pycnus             WP_102691466   -----------------AQ--MH-- - ---------------KP---I--H----
Rummeliibacillus stabekisii         WP_066786736   -------------Q---AQ--MH-- - ----------M-----P---I--H----
Chryseomicrobium excrementi         WP_100352489   -----------------FY--DK-- D E-L--------V----M------H----
Edaphobacillus lindanitolerans      WP_076757681   -----------------AQ--MH-- D --L----A---M---K-IG--P-H----
Filibacter sp. TB-66                WP_124070418   -----------------AQ--M--- - --L------A-M---KL---L-------
Tetzosporium hominis                WP_094942015   -----------------FY--DK-- D E-L--------V----M------H----
Ureibacillus thermophilus           QBK26110       -----------------AA--AH-- D E------A-------K---DIP-H----
Ureibacillus thermosphaericus       WP_016837771   -----------------AA--AH-- D E------A-------K---DI--H----
Viridibacillus arvi                 WP_053416215   --T--------------AQ--MH-- - --L-------------A---I--H----
Viridibacillus sp. OK051            WP_100797878   -----------------AQ--MH-- - --L-------------T---I--H----
```

Other Bacteria (0/>100)

```
Anoxybacillus amylolyticus          WP_066326093   ---Y-------------AR--EY-D   G-P------A-----KLCTMIP-H----
Anoxybacillus tepidamans            WP_027407714   ---Y-------------AR--EY-E   GYP------A-----KICTMIE-H----
Anoxybacillus vitaminiphilus        WP_111644145   ---Y-------------AR--EY-E   GYP------A-----KICTMIE-H----
Bacillaceae bacterium EAG3          WP_104848319   -----------------AR--EY-T   GYP------A-----KIIKMIE-H----
Bacillaceae bacterium SAOS 7        PAQ14666       ---Q-------------AK--SY-E   GYP------A-----KICTM-E-H----
Bacillus abyssalis                  WP_078413697   -----------------AH--DH-V   GYP------A-----KLMTM-E-H----
Bacillus acanthi                    WP_108670868   -----------------EH--SY-V   -YP------A-----KLCTMIE-H----
Bacillus acidiceler                 WP_088010895   ---Q-------------AK--EY-T   GKA------A-----EL--MIE-H----
Bacillus acidicola                  WP_066262411   -----------------AK--SY-T   GFP------A-----KL-TM-E-H----
Bacillus alkalitelluris             WP_078545876   ---Q-------------AR--EF-E   GYP------A-----KIITM-E-H----
Bacillus alveayuensis               WP_044747903   ---Y-------------AR--EY-E   GYP------A-----KICTMIE-H----
Bacillus amyloliquefaciens          WP_101669850   ---Q-------------AK--EY-E   GYP------AI----KIMKM-E-H----
Bacillus andreraoultii              WP_033828468   --N--------------AQ--SY-E   EYP------A-----KL-GM-E-H----
Bacillus anthracis                  WP_000606887   --N--------------AR--EY-E   GYP------A-----KI-TM-E-H----
Bacillus aryabhattai                WP_071270721   --TN-------------AR--EY-E   GYP------A-----KIITM-E-H----
Bacillus asahii                     WP_119115445   ---QF------------AR--EY-E   GYP------A-----KILKM-E-H----
Bacillus atrophaeus                 AT028373       ---Q-------------AK--EY-E   GYP------AI----KIQKM-E-H----
Bacillus australimaris              WP_060697283   ---Q-L-----------AR--EY-E   GFP------A-----KIIKM-E-H----
Bacillus badius                     WP_041097104   -----------------AK--SY-V   GYP------A-----ELCTM-E-H----
Bacillus bataviensis                WP_007084979   -----------------ET--SY-T   GYP------A-----KICSM-E-HQ---
Bacillus bingmayongensis            WP_017150935   -----------------AR--EY-E   GYP------A-----KI-TM-E-H----
Bacillus campisalis                 WP_046526410   ---Q-------------EK--SY-T   GYP------A------IITMIE-H----
Bacillus cereus                     WP_000606876   -----------------AR--EY-E   GYP------A-----KI-TM-E-H----
Bacillus coagulans                  WP_035188023   ---Q-------------AR--SY-T   -YP------A-----KI-KMIE-HQ---
Bacillus coahuilensis               WP_010173262   ---Q-------------AK--SY-T   GYP------A-----EIITM-E-H----
Bacillus cohnii                     WP_066413006   --N--------------EK--EF-E   GYP------A-----KILTM-E-H----
Bacillus cucumis                    WP_101651058   -----------------ET--SY-T   GYP------A-----KILTMIE-HQ---
Bacillus cytotoxicus                WP_012095310   -----------------AR--EY-E   GYP------A-----KI-TM-E-H----
Bacillus deserti                    WP_101645478   -----------------EK--SF-T   GYP------A-----KL-KM-E-H----
Bacillus endophyticus               WP_061801918   --TR-------------AR--EY-T   -YP------A-----KL-SM-E-H----
Bacillus farraginis                 WP_058002253   -----------------AK--SY-E   GYP------A-----KLLTM-E-H----
Bacillus firmus                     WP_061791239   -----------------EK--SY-T   GYP------A-----ELCTMIE-H----
Bacillus fordii                     WP_018708489   -------------R---AK--SY-T   -YP------A-----KICTM-E-H----
Bacillus fortis                     WP_120068317   -------------R---AK--SY-T   -YP------A-----KICTM-E-H----
Bacillus freudenreichii             WP_126431947   -------------RD--AK--SY-T   -YP------A-----KICTM-E-H----
Bacillus funiculus                  WP_129727141   ---Q-------------AR--EY-E   GYP------A-----KILKM-E-H----
Bacillus gaemokensis                WP_033672437   --N--------------AH--EY-E   GYP------A-----KI-TM-E-H----
Bacillus ginsengihumi               WP_025726808   ---Q-------------AK--SY-E   GYP------A-----KI-KMIE-HQ---
Bacillus glycinifermentans          WP_048356220   -----------------AR--EY-E   GYP------A-----KILKM-E-H----
Bacillus gobiensis                  WP_053606033   --------------D--AE--GY-T   GYP------A-----KL-TM-E-H----
Bacillus gottheilii                 WP_066443826   -----------------EL--TY-T   GYP------A-----KLCTM-E-H----
Bacillus halotolerans               WP_024121978   ---Q-------------AR--EY-E   GYP------AI----KIMKM-E-H----
Bacillus haynesii                   WP_043927820   ---Y-------------AR--EY-E   GYP------A-----KILKM-E-H----
Bacillus hemicellulosilyticus       WP_035346084   K--RF------------AR--EY-E   GYA------A-----KIQMM-E------
Bacillus horikoshii                 WP_063560452   --T--------------EK--EY-E   GYP------A-----KI-TM-E-H----
Bacillus horneckiae                 WP_066395763   -----------------DA--SY-T   GFP------A-----KLCQM-E-H----
Bacillus intestinalis               WP_088679266   ---Q-------------AR--DY-E   GYP------AI----KIMKM-E-H----
Bacillus jeotgali                   WP_102263519   -----------------EK--SY-T   GYP------A-----KLCTM-E-H----
Bacillus kochii                     WP_095372653   -----------------AE--SY-T   GFP------A-----EIITM-E-HD---
Bacillus koreensis                  WP_053402197   ---Q-------------AR--EF-E   GYP------A-----KIITM-E-H----
Bacillus korlensis                  WP_066047241   K--Q-------------AT--TY-T   -YP------A-----KILTM-E-H----
Bacillus krulwichiae                WP_066159172   ---RF------------AK--EY-T   GYA------A-----KIMTM-E-F----
```

Other Bacteria (0/>100)

Bacillus lentus	WP_066137512	-----------------AK--SY-T	-YP------A-----K-CTM-E-H----
Bacillus licheniformis	WP_025808082	----------------AR--EY-E	GYP------A-----KILKM-E-H----
Bacillus luciferensis	WP_088072929	---Q-------------AK--EY-T	GKA------A-----EL--M-E-H----
Bacillus manliponensis	WP_034636142	-----------------AR--EY-E	GYP------A-----KI-TM-E-H-V--
Bacillus marinisedimentorum	WP_070121624	-----------------EK--SY-T	GYP------A-----EIKTM-E-H----
Bacillus marisflavi	WP_048005983	---R-------------AK--SY-T	GYP------A------ILKM-E-H----
Bacillus massiliglaciei	WP_110927503	---R-------------GR--EY-E	GYP------A-----KIIKM-E-H----
Bacillus mediterraneensis	WP_071460064	---Q-------------AK--NY-T	GYP------A------ILTM-E-H----
Bacillus megaterium	SFG17444	--TN-------------AR--EY-E	GYP------A-----KIITM-E-H----
Bacillus methanolicus	WP_004437654	-----------------EK--SY-T	GYP------A-----KICTMIE-H----
Bacillus mojavensis	WP_010334858	---Q-------------AR--EY-E	GYP------AI----KIMKM-E-H----
Bacillus mycoides	EEK71599	--N--------------AR--EY-E	GYP------A-----KI-TM-E-H----
Bacillus niacini	WP_045517495	-----------------EK--SY-T	GYP------A-----KI-TM-E-HQ---
Bacillus niameyensis	WP_062104737	-----------------AK--SY-T	-YP------A-----KICKM-E-H----
Bacillus oleivorans	WP_097157406	K--Q-------------ES--SY-E	GYP------A-----KI-TM-E-H----
Bacillus panaciterrae	WP_034670142	-----------------QH--EY-E	GYP------AI----KIATM-E-H----
Bacillus paralicheniformis	WP_095291147	-----------------AR--EY-E	GYP------A-----KILKM-E-H----
Bacillus pseudomycoides	PEA56263	--N--------------AR--EY-D	GYP------A-----KI-TM-E-H----
Bacillus pumilus	OLP64531	---Q-L-----------AR--EY-E	GYP------A-----KIIKM-E-H----
Bacillus safensis	WP_024422921	---Q-L-----------AR--EY-E	GFP------A-----KIIKM-E-H----
Bacillus salsus	SDP90267	---Q----------D--AK--SY-E	GYP------A-----KILTM-E-H----
Bacillus selenatarsenatis	WP_041967914	-----------------EK--SY-T	GYP------A-----KLCTM-E-H----
Bacillus smithii	WP_003355270	---Q-------------AK--SY-T	GYP------A-----KL--M-E-H----
Bacillus solisilvae	WP_087999010	---Q-------------AK--EY-T	GKA------A-----EL--M-E-H----
Bacillus sonorensis	WP_006637471	-----------------AR--EY-E	GYP------A-----KILKM-E-H----
Bacillus sporothermodurans	WP_066234553	---Q-------------AK--SY-E	GYP------A-----KLLTM-E-H----
Bacillus subtilis	WP_087990646	-----------------AR--DY-E	GYP------AI----KIMKM-E-H----
Bacillus swezeyi	WP_076761794	---Q-------------AR--EY-E	GYP------A-----KILKM-E-H----
Bacillus thuringiensis	OJE14512	--N--------------AR--EY-E	GYP------A-----KI-TM-E-H----
Bacillus toyonensis	WP_098644064	--N--------------AR--EY-E	GYP------A-----KI-TM-E-H----
Bacillus vallismortis	WP_010328319	---Q-------------AR--DY-E	GYP------AI----KIMKM-E-H----
Bacillus velezensis	WP_069473496	---Q-------------AK--EY-E	GYP------AI----KTMKM-E-H----
Bacillus vireti	WP_024030452	-----------------ET--NF-T	GYP------A-----KILSM-E-HQ---
Bacillus wiedmannii	EEK65801	--N--------------AT--EY-E	GYP------A-----KI-TM-E-H----
Bacillus xiamenensis	WP_008358561	---Q-L-----------AR--EY-E	GYP------A-----KMITM-E-H----
Baia soyae	WP_131848914	----Y-----------AE--QY-E	GQP-------I----KL-HM-H-H----
Brevibacillus laterosporus	WP_003333562	K---FY-----------AK--EY-E	GYP-----YA-----KL--M-E-HQ---
Domibacillus robiginosus	WP_050180789	---R-------------AQ--SF-T	GYP------A------IQ-M-E-H----
Falsibacillus pallidus	WP_114746203	-----------------AK--SY-T	EYP------AI----KLCTM-E-H----
Fictibacillus arsenicus	WP_077365234	-----------------EK--SY-T	GYP------A-----EIQMM-E-H----
Fictibacillus phosphorivorans	WP_066242164	-----------------ET--SY-T	GYP------A-----EIQMM-E-H----
Geobacillus thermodenitrifican	AB067646	-----------------AR--EY-V	GEP------A-----KLC-MIH-HD---
Gracilibacillus lacisalsi	WP_018931638	--EQ---------R---E---QY-E	GYQ------A------ILKM-E-H----
Jeotgalibacillus alimentarius	WP_041122783	---R-------------AK--SY-E	GYP------A-M---KIISM-E-H----
Jeotgalibacillus campisalis	WP_041059103	---R-------------AK--SY-E	GYP------A--R--KILSM-E-H----
Jeotgalibacillus malaysiensis	WP_039809563	---R-------------AK--SY-E	GYP------A-----KIISM-E-H----
Jeotgalibacillus proteolyticus	WP_104056838	---R-------------AK--SY-E	GYP------A-----KILSM-E-H----
Jeotgalibacillus salarius	WP_134380288	---R-------------AK--SY-E	GYP------A-----KIISM-E-H----
Jeotgalibacillus soli	WP_041090200	---R-------------AK--SY-T	GYP------A-----EL-TM-E-H----
Lentibacillus sediminis	WP_100010720	-------------R---EK--QY-E	GYP------A-----EM-TMLE-HD---
Macrococcus bovicus	WP_133450712	--Q-------------AT--DY-E	GYP------A-M---KI-SMIE-H----
Macrococcus hajekii	WP_133429029	----------------AA--DY-E	GYP------A-----KIISMIE-H----
Oceanobacillus arenosus	WP_115772872	-------------R---ER--EY-E	GYA------A-----KI-KMIE-HD---
Oceanobacillus picturae	WP_036572699	-----------------EK--SY-E	GYP------AF----EI-TMLE-HD---
Oceanobacillus timonensis	WP_080874302	-----------------DQ--TY-E	GYA------A-----KILDM-E-NQ---
Ornithinibacillus contaminans	WP_047979969	-------------R---EK--SY-E	GYP------A-----KI-TM-E-HD---
Ornithinibacillus scapharcae	WP_010093164	-------------R---EK--TY-E	GYP------AF----KI-TMLE-H----
Paraliobacillus sediminis	WP_117170348	---N-------------EK--AY-K	GYQ------A------I-TM-E-NN---
Sediminibacillus massiliensis	WP_077621638	-------------R---ER--QY-E	GYP------AF----EI-TM-E-HD---
Staphylococcus aureus	PPJ96758	--Q-------------AT--EY-E	GYP------A-----KIISMIE-H----
Streptococcus pneumoniae	CJF71205	--TN-------------AR--EY-E	GYP------A-----KIITM-E-H----
Thalassobacillus devorans	WP_028783143	-----------------ER--QH-V	GYP------AF----EI-TM-E-HD---
Virgibacillus alimentarius	WP_029269836	-------------R---EK--TF--	-YP------AF----KI-TMLE-HD---
Virgibacillus proomii	WP_077320752	-----------------ER--EY-E	GYP------AF----KI-TMIE-HD---
Virgibacillus senegalensis	WP_053219282	-----------------EK--SY-E	GYP------AF----KI-KM-E-HD---

Supplemental Figure 6

A partial sequence alignment of the BrxA/BrxB family bacilliredoxin protein containing a one amino acid insertion (boxed) that is exclusively shared by all members among the *Planococcaceae* family and absent in all other bacteria.

Family *Planococcaceae* (>100/>100)

		4		41
Bacillus cecembensis	WP_057989758	IESTQNSLVKHWKKLL	T	QRKEREKSGEYIVEGFHLVEE
Bacillus ndiopicus	WP_042474808	------A--------M	-	T---------F----------
Bacillus sp. B14905	WP_008179195	------A---Y----A	-	T-----R---F----------
Bacillus sp. FJAT-22090	WP_053588675	----------------	S	V------TS-FLI--Y-----
Bacillus sp. OxB-1	WP_041075456	----------Y----V	-	T----D----FL------A--
Bhargavaea beijingensis	WP_092097119	---------------V	N	T----D-T--FL---E--A--
Bhargavaea cecembensis	WP_063178057	---------------V	N	T----D-T--FL---E--A--
Bhargavaea ginsengi	WP_092051539	---------------V	N	T----D-T--FL---E--A--
Butyricicoccus sp. 1XD8-22	RKJ46636	------A--------V	-	M------T--FLI--------
Caryophanon latum	WP_066461951	------AQ--A----A	Q	L----D-M---MI--------
Caryophanon tenue	WP_066542175	------AQ--A----A	Q	L------M---MI--------
Kurthia gibsonii	WP_121176262	---LS-----Y----A	-	T----DQ---FL---------
Kurthia huakuii	WP_029499407	---N------Y----A	-	T----D----FL---------
Kurthia massiliensis	WP_010288232	---N--A---Y----A	-	T----D----FL---------
Kurthia senegalensis	WP_026022528	---N--A---Y----A	-	T----D----FL---Y-----
Kurthia sibirica	WP_109305182	---SS-----Y----A	-	T----D-T--FL---------
Kurthia sp. 3B1D	WP_126989251	---N------Y----A	-	T----D----FL---------
Kurthia zopfii	WP_126343707	---SS-----Y----A	-	T----D----FL---------
Lysinibacillus acetophenoni	WP_097148497	---A--A--------V	-	M------T---L---------
Lysinibacillus boronitolerans	WP_036078296	------A---Y----A	-	T-----R---FL---------
Lysinibacillus chungkukjangi	WP_107934173	---P--A--------V	-	T---------F----------
Lysinibacillus composti	WP_124762397	---------------T	S	T------T--F----------
Lysinibacillus contaminans	WP_053582503	----------Y----A	-	T-----R---FL---------
Lysinibacillus endophyticus	WP_121212740	---------------V	-	T------T--FL---------
Lysinibacillus fluoroglycofenilyticus	WP_107942943	------A--------V	-	T---------F----------
Lysinibacillus fusiformis	WP_009373879	------A---Y----A	-	T-----R---FL---------
Lysinibacillus halotolerans	WP_122971147	------A--------V	-	T------T--F----------
Lysinibacillus jejuensis	WP_108307645	------A-I-----IA	-	T------TQTF-I--------
Lysinibacillus macroides	WP_053996414	------A---Y----A	M	TK----R---F----------
Lysinibacillus manganicus	WP_036186063	---------------V	-	M------T---LI--------
Lysinibacillus massiliensis	WP_036173856	------A--------V	-	M------T--FLI--------
Lysinibacillus meyeri	WP_107840700	------A--------V	-	T-------Q-F-------I--
Lysinibacillus odysseyi	WP_036156318	---N--A--------V	-	L---------F-I--------
Lysinibacillus parviboronicapiens	WP_054767273	------A---Y----A	-	T-----R---FL---------
Lysinibacillus saudimassiliensis	CDZ99635	----------Y----A	V	V-------K-F-I--------
Lysinibacillus sinduriensis	WP_036198732	---A--A--------V	-	T---------F----------
Lysinibacillus sp. 2017	WP_108713492	------A--------A	-	-------T---L---------
Lysinibacillus sp. B2A1	AVK83978	------A---Y----A	-	T-----R---F----------
Lysinibacillus sp. BF-4	WP_036144635	----------Y----A	V	V-------K-F-I--------
Lysinibacillus sp. BK089	WP_132357150	------A---Y---IA	-	T-----R---FL---------
Lysinibacillus sp. FJAT-14222	WP_053592435	------A---Y----A	A	T-----R---F----------
Lysinibacillus sp. FJAT-14745	WP_053484937	------A---Y----A	-	T-----R---FL---------
Lysinibacillus sp. Marseille-P	WP_106780291	------A--------T	-	L------T--F----------
Lysinibacillus sp. OL1	WP_131521377	------A---Y----A	-	T-----R---FL---------
Lysinibacillus sp. SYSU K30002	WP_126657300	---I--A--------V	-	T------T--FL---------
Lysinibacillus sp. YLB-03	WP_118877307	---P--A--------V	-	N----D------I--------
Lysinibacillus sp. YS11	WP_103118623	------A---Y----A	-	T-----R---FL---------
Lysinibacillus sp. ZYM-1	WP_054610784	------A---F----A	-	T-----RT--F----------
Lysinibacillus sphaericus	WP_069513682	------A---Y----A	-	T-----R---FL---------
Lysinibacillus tabacifolii	WP_108029935	------A---Y----A	N	T-----R---FL---------
Lysinibacillus telephonicus	WP_126293648	------A--------V	-	T------T--F----------
Lysinibacillus varians	WP_025220275	------A---Y----A	-	T-----R---FL---------
Lysinibacillus xylanilyticus	WP_100544857	------A---Y----A	-	T-----R---FL---------
Lysinibacillus xyleni	WP_097072840	---------------V	-	T------T--FL---------
Paenisporosarcina antarctica	WP_134209400	---S--AQ-----R-I	-	V----D----FL---------
Paenisporosarcina indica	WP_075620018	---M--TQ-------V	-	V----D-T--FL---------
Paenisporosarcina quisquiliaru	WP_090564170	---P------------	S	V----D-T--F-I--Y-----
Paenisporosarcina sp. HGH0030	WP_016426723	---S---Q-------V	S	V----D----FL---------
Paenisporosarcina sp. K2R23-3	WP_119883545	---S--G-----R--V	S	T------HH-FL---H-----
Paenisporosarcina sp. OV554	WP_108585315	---S--AQ-------V	-	V--D-D----FL---------
Paenisporosarcina sp. TG-14	WP_017379877	---S--AQ-----R-I	-	V----D----FL---------
Paenisporosarcina sp. TG20	WP_019414213	---S---Q-----R-V	-	V----D----FL---------
Planococcus antarcticus	WP_006830995	---N-----------G	-	T----D-FV-FL------T--
Planococcus citreus	WP_121300226	---L-----------S	-	T----D-F--FLI-----T--
Planococcus donghaensis	WP_065526655	---N-----------S	-	T----D-FA-FL------T--
Planococcus faecalis	WP_071153201	---N-----------G	-	T----D-FA-FL------T--
Planococcus halocryophilus	WP_008496484	---S-----------S	-	T----D-FA-FL------T--
Planococcus halotolerans	WP_112224682	---L-----------G	-	T----D-FS-FL------T--
Planococcus kocurii	WP_058385696	---N-----------G	-	T----D-FA-FL------T--
Planococcus maitriensis	WP_112232597	---L-----------S	-	T----D-F--FLI-----T--
Planococcus maritimus	WP_068461727	---L-----------S	-	T----D-F--FL------T--
Planococcus massiliensis	WP_052651979	---------------V	-	T----D-FT-FL------T--
Planococcus plakortidis	WP_068868902	---L-----------S	-	T----D-F--FLI-----T--
Planococcus rifietoensis	WP_058381056	---L-----------S	-	T----D-F--FLI-----T--

Group	Species	Accession			
**Family *Planococcaceae* (>100/>100)**	Planococcus salinarum	TAA70473	---L-----------G	-	T----D-FS-FL------T--
	Planococcus salinus	WP_123163632	---------------V	-	T----D-FS-FL------T--
	Planococcus sp. CAU13	WP_033543610	---L-----------T	-	T----D-FS-FL------T--
	Planococcus sp. PAMC 21323	WP_038703553	---N-----------S	-	T----D-FA-FL------T--
	Planococcus sp. Y42	WP_077588715	---P-----------V	-	T----D-FK-FL------T--
	Planococcus versutus	WP_065524268	---N-----------S	-	T----D-FA-FL------T--
	Planomicrobium flavidum	WP_088006605	---P-----------V	-	T----D-FN-FL------T--
	Planomicrobium glaciei	WP_053165468	---------------V	-	T----D-FA-FL------T--
	Planomicrobium okeanokoites	WP_084246415	---L-----------T	-	T----D-FS-FL------T--
	Planomicrobium soli	WP_106533884	---V-----------G	-	T----D-FA-FL------T--
	Planomicrobium sp. MB-3u-38	WP_101801730	---L-----------T	-	T----D-FS-FL------T--
	Planomicrobium sp. Y74	WP_121633394	---L-----------G	-	T----D-FS-FL------T--
	Solibacillus isronensis	WP_079528324	------A--------V	-	-------T-------------
	Solibacillus silvestris	WP_014823147	------A--------V	-	-------T-------------
	Solibacillus sp. R5-41	WP_099424798	------A---------	-	---------------------
	Sporosarcina koreensis	WP_040286237	---P--A--------V	-	T----DR-E-FVL--------
	Sporosarcina newyorkensis	WP_009497592	---------------V	-	T----DQTN-FL---------
	Sporosarcina pasteurii	WP_115362159	---I--A--------V	Q	T----D--K-FL---------
	Sporosarcina psychrophila	WP_067207813	---S--A--------V	-	T----DL-K-FL---------
	Sporosarcina sp. BI001-red	WP_116019515	------A--------I	-	TK----R-E-F-I--H-----
	Sporosarcina sp. D27	WP_025783255	------A--------V	-	TK----R-E-F-I--H-----
	Sporosarcina sp. EUR3 2.2.2	WP_024534276	------AQ-------V	-	V--D-D----FL---------
	Sporosarcina sp. HY008	WP_067402977	------A--------V	-	T----D--E-FLI--------
	Sporosarcina sp. P1	WP_099626804	---------------V	-	T----DQTK-FL---------
	Sporosarcina sp. P13	WP_099689119	---------------V	-	T----DQTK-FL---------
	Sporosarcina sp. P16b	WP_099672460	---------------V	-	T----DQTK-FL---V-----
	Sporosarcina sp. P18a	WP_099674440	---------------V	-	T----DQTK-FL---V-----
	Sporosarcina sp. P19	WP_099690679	------A--------V	-	T----DQTK-FV---------
	Sporosarcina sp. P20a	WP_099677409	---------------V	-	T----DQTK-FL---------
	Sporosarcina sp. P26b	WP_099692584	---------------V	-	T----DQTK-FL---------
	Sporosarcina sp. P29	WP_099661215	---------------V	-	T----DQTK-FL---------
	Sporosarcina sp. P3	WP_099639071	------A--------V	-	T----DQTK-FV---------
	Sporosarcina sp. P34	WP_099695201	---------------V	-	T----DQTK-FL---V-----
	Sporosarcina sp. PTS2304	WP_114924003	---------------V	-	T----DQTK-FL---------
	Sporosarcina sp. ZBG7A	WP_039044418	------A--------I	-	TK----R-E-F----H-----
	Sporosarcina ureae	WP_085131158	---------------V	-	T----DQTK-FL---------
	Psychrobacillus insolitus	WP_111437696	---P---IA-------	S	V----D-T--F-I--Y-----
	Psychrobacillus psychrodurans	WP_093496037	---P------------	S	V----D-T--F-I--Y-----
	Psychrobacillus psychrotoleran	WP_093536932	---P--T---------	S	V----D----FLI--Y--I--
	Psychrobacillus sp. FJAT-21963	WP_056833054	----------------	S	V------TS-FLI--Y-----
	Psychrobacillus sp. OK028	WP_093060442	---P--A---------	S	V----D-T--F-I--Y-----
	Psychrobacillus sp. OK032	WP_093266823	---P------------	S	V----D-T--F-I--Y-----
	Rummeliibacillus pycnus	WP_102691113	---P------Y----A	-	T----DR---FLI--------
	Rummeliibacillus stabekisii	WP_066788744	---P------Y----T	-	T----DR---F-I-----I--
	Tetzosporium hominis	WP_094944148	----G-A--------I	-	T--D----K-FV---Y-----
	Chryseomicrobium excrementi	WP_100352299	----G-A--------I	-	T--D----K-FV---Y-----
	Edaphobacillus lindanitolerans	WP_076757960	---------------V	N	T----D-TE-FL---E--A--
	Filibacter sp. TB-66	WP_124071671	---P--A--------V	-	T----D--E-FV---------
	Ureibacillus thermophilus	QBK26479	---K--A--------V	S	TK---D-T-------------
	Ureibacillus thermosphaericus	WP_096551457	---A--AY-------V	-	N------T--F----------
	Viridibacillus arvi	WP_053416581	---P------Y----V	-	T-----R---F----------
	Viridibacillus sp. OK051	WP_100794465	----------Y----V	-	T-----R---FL---------
**Other Bacteria (0/>100)**	Sporosarcina globispora	WP_053434168	-Q-AK-PKI-E-----		TK---D---TFL---------
	Aneurinibacillus soli	WP_096463903	-T-V--PR--Q-A--H		T------T-LFLI--P-----
	Anoxybacillus ayderensis	WP_085788303	----K-ER--Q----S		TK---D-T-LFLI--------
	Anoxybacillus flavithermus	WP_004889109	---PK-ER--Q-----		TK-G-D-T-LFLI--------
	Anoxybacillus flavithermus NBR	GAC92237	---PK-ER--Q-----		TK-----T-LFL---------
	Anoxybacillus gonensis	WP_009361816	---PK-ER--Q-----		TK---D-T-LFLI--------
	Anoxybacillus kamchatkensis	WP_026011505	---PK-ER--Q-----		TK---D-T-LFLI--------
	Anoxybacillus pushchinoensis	WP_091701340	---PK-ER--Q-----		TK-G-D-T-LFLI--------
	Anoxybacillus thermarum	WP_043967856	---PK-ER--Q-----		TK---D-T-LFLI--------
	Anoxybacillus vitaminiphilus	WP_111644542	----K-PQ--Q-----		TK-----T-LF-I--------
	Bacillus acanthi	WP_108669359	-Q-P--T---Q-R---		MK---D---LFL---------
	Bacillus alkalitelluris	WP_078545197	---VK-TQ--Q----Q		TK---D-TNTFLI--------
	Bacillus altitudinis	WP_050827541	---AK-AHI-Q----H		TK---T-T-LFL---K-----
	Bacillus amyloliquefaciens	WP_102421653	-D-AK-QK--D----Q		TK---L-TNTFLI--E--A--
	Bacillus amyloliquefaciens gro	WP_015417960	-D-AK-QK--D----H		TK---L-TNTFLI--E--A--
	Bacillus andreraoultii	WP_033828238	-Q-VK-EK--Q-----		TK---D-T-LFL---E-----
	Bacillus azotoformans	WP_039957264	------PR--Q----Q		TK---N-T-LFLI--L--I--
	Bacillus azotoformans LMG 9581	EKN67452	------PR--Q----Q		TK---N-T-LFLI--L--I--
	Bacillus azotoformans MEV2011	KEF39541	------PR--Q----Q		TK---N-T-LFLI--I--I--
	Bacillus bataviensis	WP_007087358	---VN-PK--Q-----		TK---D---TFLI--------
	Bacillus boroniphilus JCM 2173	GAE45937	-Q-DK-PQ--Q-----		T----D---MFL---------
	Bacillus cavernae	WP_126867021	---AK-PR--Q-----		TK---D-T-R-LI--------

Other Bacteria (0/>100)

Bacillus cellulasensis	WP_017367822	---AK-AHI-Q----H	TK---T-T-LFL---K-----
Bacillus cereus	WP_080349854	-D-V---R--Q----Q	TK---D-K-LFF---------
Bacillus cereus group	WP_088059829	-D-V---R--Q----Q	TK---D-K-LFF---------
Bacillus dakarensis	WP_077214189	-Q-AK-PQ--Q-----	TK---D-T-TFLI--------
Bacillus deserti	WP_101640378	---SK-PQ--Q-----	TK-D-D-T-KFLI--------
Bacillus drentensis	WP_066258046	---IN-PK--Q-----	TK---D---TFL---------
Bacillus firmus	WP_048011920	-Q-AK-PK--E-----	A----D-T-TFL---------
Bacillus funiculus	WP_129727782	-D-L--PRI-Q----Q	TK---D-T-TF----------
Bacillus glycinifermentans	WP_048353906	---AK-QK--D----H	TK---I-TNTFLI--E-----
Bacillus gottheilii	WP_080844484	-H-A--QK--Q-----	TK---D-TLTFL---Y-----
Bacillus halotolerans	WP_101860156	---AK-QK--D----H	TK---T-TNT-LI--E-----
Bacillus horneckiae	WP_066396601	-H-AK-PQI-Q-----	TK---D-T-TFL---------
Bacillus indicus	WP_029279210	---I--QK--Q-R--H	TK---DA--TFLI--M-----
Bacillus intestinalis	WP_061186727	---AK-QK--D----H	TK---T-TNTFLI--E-----
Bacillus jeotgali	WP_102263812	-Q-DK-LQ--Q-----	T----D---MFL---------
Bacillus koreensis	WP_053400885	-D-VK-PQ--G----H	TK---D-T-LF----------
Bacillus korlensis	WP_066050303	-Q-SS-PK--Q-----	TK---D-T-LF----------
Bacillus kribbensis	WP_026693705	---PK-PA--Q--R--	NK-D-D---KF----------
Bacillus lehensis	WP_038481782	----K-ER--A----H	TK-G--QT-SFLI--D---D-
Bacillus licheniformis	WP_075747907	---AK-QK--D----H	TK---T-TNTFLI--E-----
Bacillus massiliglaciei	WP_110927169	---VK-PK--Q----Q	MK---D-T-T-L---------
Bacillus massiliogabonensis	WP_102274326	-H-AK-PQI-Q-----	TK---D---TFL---------
Bacillus massilionigeriensis	WP_075982518	-S-AK-PQ--Q-----	TK---DR--TFL---------
Bacillus massiliosenegalensis	WP_019154761	-Q-A--PQ--Q-----	TK---D-T-TFL---------
Bacillus mesonae	WP_066386018	-Q-S--PQ--Q-----	TK---DR-DTFLI--------
Bacillus methanolicus	WP_003347504	-H-I--PQ--Q-----	TK---D-T-TFL---H-----
Bacillus mojavensis	WP_029441382	---AK-QK--D----H	TK---T-TNT-LI--E-----
Bacillus muralis	WP_057911489	---VK-PQ--Q-----	TK---D-T-K-L---------
Bacillus mycoides	WP_080743396	-D-V--PR--Q----Q	TK---D-K-VFF---------
Bacillus niacini	WP_045523535	---I--PK--Q-----	TK---D---TFL---------
Bacillus novalis	WP_066090688	---VN--K--Q-----	TK---D---TFL---------
Bacillus oryziterrae	WP_026092512	---A---R--Q----H	TK-----T-LFLI--R-----
Bacillus panaciterrae	WP_028400920	-D-L--PR--Q----Q	TK---D---LFL------A--
Bacillus paralicheniformis	WP_130569445	---AK-QK--D----H	TK---T-TNTFLI--E-----
Bacillus paralicheniformis ATC	AGN37441	---AK-QK--D----H	TK---T-TNTFLI--E-----
Bacillus paramycoides	WP_071718486	-D-V--PC--Q----Q	TK---D-K-LFF---------
Bacillus praedii	WP_057761501	-H-AK-PQI-Q-----	TK---D-T-TFL---------
Bacillus pseudomycoides	PEA56791	-D-V--PR--Q----Q	TK---D-K-LFF---------
Bacillus psychrosaccharolyticu	WP_040374591	---VK-TK--Q-----	TK---D---R-LI--------
Bacillus pumilus	OLP66010	---AK-TNI-Q----H	TK---T-T-QFL---K-----
Bacillus rubiinfantis	WP_042354840	-Q-L--PQ--Q-----	TK---DRT-TF-I--------
Bacillus safensis	WP_099663523	---AK-TNI-Q----H	TK---T-T-LFL---I-----
Bacillus salsus	WP_090849088	---IK-P---E----Q	T-----RTQSFLI--------
Bacillus selenatarsenatis	WP_041967562	-Q-DK-PQ--Q-----	T----D---MFL---------
Bacillus siamensis	WP_045925265	-D-AK-QK--D----H	TK---H-TNTFLI--E--A--
Bacillus simplex	WP_072273356	---VK-PQ--Q-----	TK---D-T-K-L---------
Bacillus smithii	WP_048623803	-Q-VK-PQ--Q-R--A	T----D-T-R-MM--------
Bacillus soli	WP_066063623	---LS-PK--Q-----	TK---D---TFL---------
Bacillus sonorensis	WP_029419148	---AK-QK--D----H	TK---T-TNTFLI--E-----
Bacillus stratosphericus	WP_081114422	---AK-AHI-Q----H	TK---T-T-LFL---K-----
Bacillus subterraneus	WP_044393457	-Q-DK-PQ--Q----K	T----D---MFL---I-----
Bacillus subtilis	KJJ40303	---AK-QK--D----H	TK---T-TNTFLI--E-----
Bacillus subtilis group	WP_014470805	-D-AK-QK--D----H	TK---L-TNTFLI--E--A--
Bacillus swezeyi	WP_076763141	---AK-QK--D----H	TK---T-TNTFLI--E-----
Bacillus thermoamylovorans	WP_041846481	-Q-KK-EK--Q-----	T----D---LF-I--E-----
Bacillus thermocopriae	WP_129000697	---PK-ER--Q-----	TK-G-D-T-LFLI--------
Bacillus thuringiensis	WP_094194577	-D-V--PR--Q----Q	TK---D-K-LFF---------
Bacillus timonensis	WP_010678365	---SK-PQI-Q--R-H	TK---D-T-TFMI--------
Bacillus toyonensis	WP_098644316	-D-V--PC--Q----Q	TK---D-K-LFF---------
Bacillus velezensis	RYA18227	---AK-TNI-Q----H	TK---T-T-LFL---I-----
Bacillus vireti	WP_024028557	---VN-PK--Q-----	TK---D---TFL---------
Bacillus xiamenensis	WP_008355996	---AK-TNI-Q----H	TK---T-T-LFL---K-----
Bacillus zeae	WP_119114038	-H-AK-PQ--Q----M	T----D---SF-I--------
Bacillus zhangzhouensis	WP_034320884	---AK-ASI-Q----H	TK---T-T-LFL---K-----
Balneatrix alpica	WP_051527542	-Q-AH-THF-------	N-----Q--QFL---W--C--
Caldibacillus debilis	KYD22743	-Q-AK-EK--R-----	TK---D-A-LFL---E-----
Caldibacillus debilis GB1	RKO63489	-Q-AK-EK--R-----	TK---D-A-LFL---E-----
Falsibacillus pallidus	WP_114745093	-Q-SK-PQ--Q-----	TK---S-T-Q-LI--------
Fictibacillus aquaticus	WP_094252243	---DS-PS--Q-----	TK-----T-TFLI--P-----
Fictibacillus arsenicus	WP_066291534	---DS-ASL-Q----H	T------T-TFLL--P--I--
Fictibacillus enclensis	WP_061972545	---E--AS--Q----Y	TK---D---QF-ID-P--I--
Fictibacillus solisalsi	WP_090234871	---E--ASI-Q----Y	TK---D---QF-I--P--I--
Geobacillus stearothermophilus	WP_033014394	---PK-AR--Q-----	TK-G-DET-LFLI--------
Listeria monocytogenes	CCQ20630	-Q-VK-DR--T----Q	T--G-D-T-T-L---------

Group	Species	Accession	Sequence	Sequence
**Other Bacteria (0/>100)**	Mycobacteroides abscessus subs	SHP97762	-H-SS-PK--Q-----	TK---D-T-LFL---------
	Parageobacillus caldoxylosilyt	KYD14232	---VK-PQ--Q-----	TK---D-T-HFLI--------
	Parageobacillus thermantarctic	WP_090947601	---VK-PQ--Q-----	MK---D-T-LFLI--------
	Parageobacillus thermoglucosid	WP_064549595	---VK-PQ--Q-----	MK---D-T-LFLI--------
	Pokkaliibacter plantistimulans	WP_110187500	-T----PQ--A----Q	T--Y-QQ--QFL---H-----
	Salipaludibacillus agaradhaere	WP_078579572	---V---KI-S----H	TK---DNT-LF-I--I--I--
	Salipaludibacillus aurantiacus	WP_093049580	---A--AKI-A----H	TK---D---LFLI--K--I--
	Salipaludibacillus neizhouensi	WP_110934908	---L--PKI-A----H	TK---D---LFLI--K--I--
	Salisediminibacterium haloalka	WP_093072898	---V--AKI-S----H	TKRG-D---LFLI--R-----
	Salisediminibacterium halotole	WP_121438719	---V--AKI-S----H	TKRG-D---LFLI--R-----
	Streptococcus pneumoniae	CVM33530	---AK-AHI-Q----H	TK---T-T-LFL---K-----
	Vibrio vulnificus	WP_133349586	---VK-PQ--Q-----	TK---D-T-K-L---------

Supplemental Figure 7

A partial sequence alignment of the RNA methyltransferase protein containing a one amino acid insertion (boxed) that is exclusively shared by all members among the *Planococcaceae* family and absent in all other bacteria.

Family *Planococcaceae* (>100/>100)

Species	Accession	123	171
Kurthia huakuii	WP_029500435	GASGAIFGILGAFLALVYYTRNILPQ	LRQMIVPLAVISVIITFLQPNVN
Kurthia massiliensis	WP_010290622	-----V--M---Y--------H-F--	-----L--VI-------------
Kurthia senegalensis	WP_010307252	---------------------K-F--	-K------V--------------
Kurthia sibirica	WP_109306436	---------F--YV-------K-M--	---Y-L-IV----VM--------
Kurthia sp. 3B1D	WP_126991395	---------------------K-F--	-----------------------
Kurthia zopfii	WP_109348863	---------F--Y--------KTM--	FK-L-L--VG---MM--V---I-
Lysinibacillus acetophenoni	WP_097150338	------Y--F---A-------HTM--	-K-I-M-III-GI-F-LI--GI-
Lysinibacillus boronitolerans	WP_016995330	---------F---G-------RTM-M	--KL-L-II-----M--------
Lysinibacillus chungkukjangi	WP_107937691	------Y--F---A-------HTM--	-K-V-L--V--------IS--I-
Lysinibacillus composti	WP_124766891	---------F---A-------RTM--	-K-I-M-III-----S-I---I-
Lysinibacillus contaminans	WP_053584981	---------F---G-------HTM-M	--KL-L-II-----M----S---
Lysinibacillus endophyticus	WP_121213144	---------F---S-------RTM--	-K-I-M--V---------S----
Lysinibacillus fluoroglycofeni	WP_107943336	---------I---GG-LF-M-HVM--	---I-L-II-----M------I-
Lysinibacillus fusiformis	WP_004232049	---------F---G-------RTM-M	--KL-L-II-----M--------
Lysinibacillus halotolerans	WP_122972924	---------F---A-------RTM--	-K-I-M--II--------S----
Lysinibacillus jejuensis	WP_108306951	---------M--YA--I----KMM-E	---L-L--ILV--VM--I---I-
Lysinibacillus macroides	WP_053996963	---------F---G-------RTM-M	--KL-L-III--LVM--------
Lysinibacillus manganicus	WP_036189514	----S-Y--F---A-------HTM--	-K-I-L-III--IVF-LF--GI-
Lysinibacillus massiliensis	WP_036172863	------Y-VF---A-------HTM--	-K-I-M-III----L-----GI-
Lysinibacillus meyeri	WP_107839634	---------I---G---F-M-RTM--	---I-L-II-V---M------I-
Lysinibacillus odysseyi	WP_036152611	---------F---G-ILVRY--S---	---I-L-II-----M----AG--
Lysinibacillus parviboronicapiens	WP_054768246	---------F---G-------RTM-M	--KV-L-II-----M--------
Lysinibacillus saudimassiliens	CEA03866	---------M--YA--I----KMM-E	---L-L--ILV--VM--I---I-
Lysinibacillus sinduriensis	WP_036200456	------Y--F---A-------RTM--	-K-V-M--II-------IS--I-
Lysinibacillus sp. 2017	WP_108714124	------Y--F---A--------LF--	-K-I-L--I-----MS-VT--I-
Lysinibacillus sp. B2A1	AVK86385	---------F---G-------RTM-M	--KL-L-II-----M--I-----
Lysinibacillus sp. BF-4	WP_036145803	---------M--YA--I----KMM-E	---L-L--ILV--VM--I---I-
Lysinibacillus sp. BK089	WP_132361626	-----V---F---G-------RTM-M	--KL-L-II-----M--F-SK--
Lysinibacillus sp. FJAT-14222	WP_053594014	-----V---F---G-------RTM-M	--KL-L-II-----M--------
Lysinibacillus sp. FJAT-14745	WP_053481963	---------F---G-------RTM-M	--KL-L-II-----M--------
Lysinibacillus sp. Marseille-P	WP_106781590	------Y-LF--YA-------HTM--	-K-I-M--II----L-----GI-
Lysinibacillus sp. SYSU K30002	WP_126658801	---------F---G-------RTM--	-K-I-M--II-G--M---S----
Lysinibacillus sp. YLB-03	WP_118876765	------Y--F---A-------RTM--	-K-V-L--II----L--VT--I-
Lysinibacillus sp. YR326	WP_134022869	-----V---F---G-------RTM-M	--KL-L-II-----M----S---
Lysinibacillus sp. ZYM-1	WP_054609410	---------F---G-------RTM-M	--KL-L-II-----M--------
Lysinibacillus sphaericus	WP_010860316	---------F---G-------RTM-M	--KV-L-II-----M----S---
Lysinibacillus sphaericus C3-4	ACA41765	---------F---G-------KTM-M	--KL-L-III----M--------
Lysinibacillus telephonicus	WP_126293303	---------F---VG------KTM--	-K-I-M--VI--------S----
Lysinibacillus varians	WP_025220704	---------F---G-------RTM-M	--KL-L-II-----M----S---
Lysinibacillus xylanilyticus	WP_049663434	-----V---F---G-------RTM-M	--KL-L-II-----M----S---
Lysinibacillus xyleni	WP_097074355	---------F---S-------RTM--	-K-I-L--VI--------S----
Bhargavaea beijingensis	WP_092097902	------Y--F---A-F--H-KGAF--	I--I-L-II----VM---T--I-
Bhargavaea cecembensis	WP_008301467	------Y--F---G-F--H-KGAF--	I--I-L-II-----M---T--I-
Bhargavaea ginsengi	WP_092055439	------Y--F---G-F--H-KGAF--	I--I-L-II-----M---T--I-
Butyricicoccus sp. 1XD8-22	RKJ44221	------Y-VF---A-------HTM--	-K-I-M-III----L-----GI-
Caryophanon latum	WP_066461107	--------VF--YA-------KTM-A	---I-L-II---IVM-----G--
Caryophanon tenue	WP_066546284	--------VF--YA---F---KTM-A	---I-L-IIA---VM------I-
Chryseomicrobium excrementi	WP_100354190	------Y--F---A--------V---	-K-I-L-IIG-GI-M--VT--I-
Edaphobacillus lindanitolerans	WP_076759211	------Y--F---G------K-AF--	--GI-M-II---I-M---T--I-
Falsibacillus pallidus	WP_114746641	-S------LF-FY--AALFGK-PTSR	SA-V-L-VLI--LFMS-I--G--
Falsibacillus sp. GY 10110	WP_121682085	-S------LF-FYS-M-AARKITAR-	SV-L-L--VI-ALFM--I--G--
Filibacter sp. TB-66	WP_124068857	-------------G---H--KR----	---I-L-IILV--VM--V--GI-
Bacillus cecembensis	WP_057985934	------Y--F---A--------SF--	-K-I-L--I-----M---T--I-
Bacillus ndiopicus	WP_042476954	---------I---GG-L--M-RTM--	---I-L-II----VM------I-
Bacillus sp. OxB-1	WP_041071635	---------F---G------K-AF--	---VML-II---L-M------I-
Paenisporosarcina antarctica	WP_134208610	------Y------GG----A--K---	-S-I---III--IVM---T--I-
Paenisporosarcina indica	WP_075619262	------Y-L----G-----A-KA---	---I-L-II---IVM--VSS-I-
Paenisporosarcina sp. HGH0030	WP_016429754	------Y-L----G-----A-KT---	---I-L-II---IVM---T--I-
Paenisporosarcina sp. K2R23-3	WP_119884085	---------F---G-------KAF-G	---I-L-II-----M--------
Paenisporosarcina sp. OV554	WP_108585480	------Y------G-----A--T---	---I-L-II---I-M---T--I-
Paenisporosarcina sp. TG-14	WP_017380021	------Y------GG----A--K---	-S-I---III--IVM---T--I-
Paenisporosarcina sp. TG20	WP_019414606	------Y------G-I----K-A---	---I-I-III--IVM---S--I-
Planococcus	WP_058381780	---------F---G-----GGRA---	-K-I-L-II---IVM--VT--I-
Planococcus antarcticus	WP_006830191	--------VF---G-----GGRA---	-K-I-L-II---IVM---T----
Planococcus donghaensis	WP_008432410	--------VF---G-----GGRA---	-K-I-L-II---IVM---T----
Planococcus faecalis	WP_071153532	--------VF---G-----GGRA---	-K-I-L-II---IVM---T----
Planococcus halocryophilus	WP_008498824	--------VF---G-----GGRA---	-K-I-L-II---IVM---T--I-
Planococcus halotolerans	WP_112224161	--------VF--YG--L--GGRA---	-K-I-L-IL-----M---T--I-
Planococcus kocurii	WP_058386380	--------VF---G-----GGRA---	-K-I-L-II---IVM---T----
Planococcus maritimus	WP_068460411	---------F---G-----GGRA---	-K-I-L-II---IVM--VT--I-
Planococcus massiliensis	WP_052653131	---------F--YG--L--G-RS---	---I-L-II---I-M---T----
Planococcus plakortidis	WP_068872082	---------F---G-----GGRA---	-K-I-L-II---IVM--VT--I-
Planococcus salinarum	OHX51387	--------VF--YG--L--GGRA---	-K-I-L-ILA---VL---T--I-
Planococcus salinus	WP_123166267	---------F---G--L-HGGRTM--	---I-L-II---IVM---T----

Group	Organism	Accession	Sequence	Position	Sequence
**Family *Planococcaceae* (>100/>100)**	Planococcus sp. CAU13	WP_033543549	--------VF--YG--L--GGRA---		---ILL-IL-----M---T----
	Planococcus sp. PAMC 21323	WP_038702591	--------VF---G-----GGRA---		-K-I-L-II---IVM---T--I-
	Planococcus sp. Y42	WP_077588009	---------F--YG--L--GGRA--E		---ILL-IL-----M---T----
	Planococcus versutus	WP_049695042	--------VF---G-----GGRA---		-K-I-L-II---IVM---T----
	Planomicrobium	WP_084243032	--------VF--YG--L--GGRA---		-K-I-L-IL-----M---T--I-
	Planomicrobium flavidum	WP_088006164	---------F---G--L--GGRA---		---ILL-IL-----M---T----
	Planomicrobium glaciei	WP_036807386	---------F---G--L--G-RA---		---I-L-II---I-M---T----
	Planomicrobium soli	WP_106534560	---------F---G--L--GGRV---		---I-L-IIA--IVM---T----
	Planomicrobium sp. Y74	WP_121634796	--------VF--YG--L--GGRA---		-K-I-L-IL-----M---T--I-
	Psychrobacillus insolitus	WP_111439865	------Y------G--------A---		-K-I---II--G--M--I-S-I-
	Psychrobacillus psychrodurans	WP_093495590	------Y--F---G--------FM--		-K-I-I-II--G--M-----QI-
	Psychrobacillus psychrotoleran	WP_093537571	------Y--F---G--------FM--		-K-I-I-II--G--M-----KI-
	Psychrobacillus sp. FJAT-21963	WP_056830867	------Y------G------K-L---		-K-I-L-II--G-VM----A-I-
	Psychrobacillus sp. OK028	WP_093061556	------Y--F---A-------KFM--		-K-I-I-II--G--M-----QI-
	Psychrobacillus sp. OK032	WP_093271870	------Y------G--------LM--		-K-I-I-II--G--M-----HI-
	Rummeliibacillus	WP_119415973	---------F---A-------KTM--		-K-I-M-II---I-M----SD--
	Rummeliibacillus pycnus	WP_102693749	---------F---V-------KTM--		-K-I-M-II---L-M--------
	Rummeliibacillus stabekisii	WP_066790283	-----V--LF-----------KTM--		-K-I-M-IV-----M--------
	Solibacillus	WP_008405784	-----LY--F---G-------HLF--		---I-L--I---I-M---T--I-
	Solibacillus isronensis	WP_079523299	-----LY--F---G-------HLF--		---I-L--I---I-M---T--I-
	Solibacillus kalamii	WP_087618063	-----LY--F---G-------HLF--		---I-L--I---I-M---T--I-
	Solibacillus sp. R5-41	WP_099425441	------Y--F---A--------SF--		-K-I-L-II-----M---T--I-
	Sporosarcina	WP_029054727	--------VF---G------KHAF--		---V-L-II----VM--VGT-I-
	Sporosarcina koreensis	WP_040285613	---------F---G----H-KHL---		---I-L-IIGLG-VM--VG--I-
	Sporosarcina newyorkensis	WP_009498870	--------VF---G------KHVF--		-K-V-L-II----VM--VGT-I-
	Sporosarcina pasteurii	WP_115363356	--------LF--YG------KHA---		---V-L-IIA---VM--------
	Sporosarcina psychrophila	WP_067205301	-----V-------G------KH----		---I-L-II---IVM-YV---I-
	Sporosarcina sp. BI001-red	WP_116018062	---------F---G------KKM---		---I-L-IIAL--VM--VG--I-
	Sporosarcina sp. D27	WP_025783180	-----V---F---G------KKM---		---I-L-IIGL--VM--VG--T-
	Sporosarcina sp. EUR3 2.2.2	WP_024536808	------Y-L----G-----A--T---		-K-I-L-II---I-M---T--I-
	Sporosarcina sp. HY008	WP_067407864	--------VF------I---KGNI--		---I-L-IV-L--VM----S-I-
	Sporosarcina sp. P13	WP_099687730	--------VF---G------KHVF--		-K-V-L-II----VM---GS-I-
	Sporosarcina sp. P20a	WP_099678033	--------VF---G------KHAF--		---V-L-II----VM--VGT-I-
	Sporosarcina sp. P34	WP_099694735	--------VF---G------KHAF--		---V-L-II----VM--VGT-I-
	Sporosarcina sp. PTS2304	WP_114924885	--------LF---G------KHVF--		-K-V-L-II-----M---GS-I-
	Sporosarcina sp. ZBG7A	WP_039044392	---------F---G------KKM---		---IML-IIGL--VM--VG--I-
	Tetzosporium hominis	WP_094943238	------Y--F---A--------V---		-K-I-L-IIG-GI-M--VT--I-
	Ureibacillus thermophilus	QBK24585	------Y--F-SYA-------KTM--		-K-I-M-III-GL-M------I-
	Ureibacillus thermosphaericus	WP_016838311	----S-Y--F--YA--------TM--		-K-I-M-III-GL-M------T-
	Viridibacillus arenosi	WP_038185911	---------F---A--------TM--		-K-T-L--IIV---M----S---
	Viridibacillus arvi	WP_053419213	---------F---A--------TM--		-K-T-M-IIIV-I-M----S---
	Viridibacillus sp. OK051	WP_100795697	---------F---A--------TM--		-K-T-M-II---LVM----S-I-
**Other Bacteria (0/>200)**	Ammoniphilus oxalaticus	WP_120189943	---------Y-MYAY-SR-R-D-MDR	Q	S--I-I-IL-FG--A---FTG-S
	Anaerobacillus alkalidiazotrop	WP_071388151	--------LF-IY-YM-L-RKDLID-	D	NS-VVITIL--GL-M--VNARI-
	Anaerobacillus arseniciselenat	WP_071312471	--------LF-IYAYM-L-RKDLID-	D	NS-VVMTIL--GL-M--VN-RI-
	Anaerobacillus macyae	WP_048312997	--------LF-IYFYMAF-RKELID-	A	NS-L-LMILG-GLVM--IS--I-
	Bacillaceae bacterium EAG3	WP_104850820	---------F-CYAYILL-KKHLMSR	Q	NS-T-LA-II---VLS-M-GS--
	Bacillus aciditolerans	WP_121448464	-S------LF-IY-YM-LFRKDLIS-	M	NS-L-MTILI-GLVM--INT---
	Bacillus aidingensis	WP_026702718	--------LF-VY-YM-L-RKDLIDA	M	NA-I-SIILILG-VM---N-QI-
	Bacillus alcalophilus	WP_004427163	--------LF--YFYI-LNRKDLID-	T	SS---MTIL--GLVM--VN--I-
	Bacillus alkalitelluris	WP_078543057	-S------LF-VY-YM-LKRKDLISY	A	NS-L-LTIL--G--M---N--I-
	Bacillus altitudinis	WP_047946996	--------LF-VY-Y--LFKPHMMDL	G	NS-V-LTILGV---M--VNS-I-
	Bacillus aquimaris	WP_032085351	--------LF-FYI-MILLKKAFITR	E	S--V-L-IV--G-VM--M-SGI-
	Bacillus atrophaeus	ARW05555	--------L--VY-YM-L-RKELMN-	E	NSK--IT-L-VA-LM--INS-I-
	Bacillus australimaris	WP_060699693	--------LF-VY-Y--LFKPHMMDR	G	NS-V-LTILAV---M--VNS-I-
	Bacillus bataviensis	WP_007083561	-S------LF-YYI-IIIFRKHM-SK	Q	NS-I-LV-C-V-L-M--F---I-
	Bacillus bogoriensis	WP_026675758	--------LF-LY-YMTINRKDLID-	A	NS-IVITILI-GL-M---N--I-
	Bacillus campisalis	KKK36262	-S------LF-FYISMAM-QKHM-SR	E	GS-T-LTI-A---VM--I---I-
	Bacillus canaveralius	WP_101575390	-S------LF-FY--MFTFR-YM-SR	Q	NA-I-LTIS--GI-M--I---I-
	Bacillus circulans	WP_047944184	-S------LF-YYIS--VFRKD-IS-	G	NA-I-TTI--L-L-M------I-
	Bacillus clausii	WP_095319587	----S---LF-V--FMYL-RKDLMDS	Q	SG-T-F-II-----M--VGAHI-
	Bacillus cucumis	WP_101651766	-S------LF-YYI-IIIFRKRMMS-	Q	NS-I-LV-CAV-L-M------I-
	Bacillus dakarensis	WP_077210588	-S------LF-IYI-MILFR-DLMSR	A	NT-L-LTI----L-M------I-
	Bacillus deserti	WP_101645359	--------L--YYAS--CFKKDSIGS	Q	HSRT-LT-IIVG-TM-----KI-
	Bacillus dielmoensis	WP_042454570	-S------LF-YY--IIVFRKHL-SK	Q	NS-I-LV-C---L-M---E--I-
	Bacillus drentensis	WP_066249604	-S------LF-YY--MIMFRKQM-SK	Q	NS-I-LT-C-V-L-M------I-
	Bacillus fastidiosus	WP_066234419	--------LF-VY-FMI-FRKGMMDR	S	NS-I-LSIL--GL-M--VNSDI-
	Bacillus firmus	WP_035331818	--------LF-Y-A-ILVFRKEL-TR	E	NS-I-LTITI-G--M------I-
	Bacillus foraminis	WP_121610247	-S------LF-FY--MILFR-GM-SK	E	NS-T-MTI-A-G--M--V---I-
	Bacillus fordii	WP_018709201	--------LF-IY--IIFFKKEAM--	Q	GK-V-L-I-AL---M--F-A-I-
	Bacillus fortis	WP_120072056	--------LF-IY--ITFLKKEAM--	Q	GK-V-L-I-AL---M--F-A---
	Bacillus freudenreichii	WP_126434041	--------LF-IY--ITFFKKEAM--	Q	GK-V-L-I-ALG--M--F-A-I-
	Bacillus fumarioli	WP_066369976	-S------LF-YYVSIIMFRK-L-SR	Q	NS-I-IT-LI--F-M------I-
	Bacillus glycinifermentans	WP_048356047	--------LF-VY-YI-LFRKGLMDR	A	NS-I--TILA---LM--VNY-I-

Other Bacteria (0/>200)

Bacillus gobiensis	WP_053605678	--------LF-TY-YIYF-RKELMSR	S	NS----TII---L-M--AGA-T-
Bacillus haynesii	WP_043926794	--------LF-VY-YI-LFRKELMGS	A	NS-I--TILA---LM--VNQ-I-
Bacillus horikoshii	WP_064098470	-------A-F-LYFYM-FLRPDL-DR	S	NS-V-LTIL--GLVM---NQ-I-
Bacillus horneckiae	WP_066400405	--------LF-F-M-MILFKKSK-SA	A	NS-L-ITIT--G-LM------I-
Bacillus humi	WP_057999915	-S------LF-IY-YM-MFRKDLIN-	M	NS-L-ITIL--GLVM--INS---
Bacillus infantis	WP_129614055	-S------LF-F-A-VA-FRKDLMSR	E	NS-I-ITI---GL-M--I---I-
Bacillus kochii	WP_095370828	------Y--F-F-I-IIV-R-DL-S-	Q	NT-L-LAIT--G--M----A---
Bacillus korlensis	WP_066060027	-S------LF-IY--MIFFRKEM-SR	E	NT-VVLAIV--G--M--VNA---
Bacillus licheniformis	WP_061576593	--------LF-VY-YI-LFRKELMGS	A	NS-I--TILA---LM--VNQ-I-
Bacillus litoralis	WP_066332172	--------LF-VY-FM--FRKG-ID-	A	NS-V-ISIL--GLVM--FNS-I-
Bacillus lonarensis	SDC60398	--------LF-VY-YMFLARKDL-DT	Q	NS-LVLTILA---VM--VS-QI-
Bacillus marisflavi	WP_048007234	--------LF-YY--MIATKNPFITR	E	SK-V-L-IVI-GL-M--T-SGI-
Bacillus massiliogabonensis	WP_102272745	-S------LF-F-A-IIMFRKDMISY	A	NS-L-LTI-I---FM------I-
Bacillus massilionigeriensis	WP_075980478	-S------LF-IYM-IIIFRKD--SK	E	NA-I-LSIT--GFVM--F-N-I-
Bacillus massiliosenegalensis	WP_019156413	-S-------F-IY--I-FLRKDMMSR	E	NS-V-LTI-I-G--M------I-
Bacillus megaterium	WP_026680353	--------LF-IYIFM-TFRKHLID-	T	SS-I-LTIVIVGL-M--IRQ-I-
Bacillus mesonae	WP_066390531	-S------LF-YYI-MIVFQK-R-SK	E	NS-I-IT-C-V-L-M--I---I-
Bacillus methanolicus	WP_003348445	-S------LF-FYI-IILFKKHL-SK	Q	NT-I-LTISA--L-M--V-D-I-
Bacillus nealsonii	WP_016201252	-S------LF-YYIS--VFRKD-IS-	S	NA-I-TTI--L-L-M------I-
Bacillus niacini	WP_034673293	-S------LF-YYISIILFRKHM-S-	Q	NS-I-LTICAL-L-M--I---I-
Bacillus niameyensis	WP_062106604	--------LF-VY--VILFKKEYM-I	Q	GK-V-L-I----IVM--FTS---
Bacillus notoginsengisoli	WP_118923588	-S------LF-YYVSI-LLRKRTMSR	Q	DS-T--TI-A--IVM------I-
Bacillus novalis	WP_066095261	-S------LF-YYI-IIMFRKQW-SK	Q	NS-I-LT-C---L-M------I-
Bacillus oceanisediminis	WP_019381246	--------LF-Y-A-IIVFRKEL-SR	E	NS-I-LTITI-G--M------I-
Bacillus onubensis	WP_099355757	-S------LF-IY-YM-LFRKDLIN-	M	NS-L-MTILI-GLVM--INS---
Bacillus paralicheniformis	WP_048349919	--------LF-VY-YI-LFRKELMGS	A	NS-I--TILA---LM--VNQ-I-
Bacillus patagoniensis	WP_078392389	----S---VF-V--FMYL-RNDL-DR	Q	SA-T-F-II---LVM---GS-I-
Bacillus polygoni	WP_088036212	--------LF-IY-YM-LMRKDLID-	G	SS-V-TTILI-A--M--VN-R--
Bacillus praedii	WP_057766400	--------LF-F-A-IIMFRKDMISY	A	NS-L-LTITI----M------I-
Bacillus pseudalcaliphilus	KMK76919	--------LF--YFYI-LLRKDLID-	Q	NS-V-MMILL-GL-M--VN--I-
Bacillus pumilus	WP_126741642	--------LF-VY-Y--LFKPHMMDR	G	NS-V-LTILAV---M--VNS-I-
Bacillus rubiinfantis	WP_042357747	-S------LF-YYISMIVFQK-K-TA	S	HS-V-IT-T-V-L-M---E--I-
Bacillus safensis	WP_034283954	--------LF-VY-Y-ALFKPHMMDR	G	NS-V-LTILAV---M--VNS-I-
Bacillus shacheensis	WP_059105957	--------LF-IYVYMLLNRKDL-DA	Q	SS-LVMMIV---L-M--VN-GI-
Bacillus sinesaloumensis	WP_077620339	-S------LF-IY-YM-LFRKDLIN-	M	NS-L-MTILI-GLVM--INS---
Bacillus soli	WP_066074519	-S------LF-YYI-MIIFRK-MMSK	Q	NS-I-ITIC---L-M------I-
Bacillus solimangrovi	WP_069716871	------Y-LF-IY-YM-L-RKDLIDA	N	NA-L-ITIL--GL-L---R--I-
Bacillus sonorensis	ASB90949	--------LF-VY-YM-LFRKGLMDT	A	NS-I--TILA--ILM--INY-I-
Bacillus sp. 1NLA3E	WP_015592033	-S-----ALF-FY--IILFKKHL-SR	Q	NS-IVLTIT--GLVM--I-----
Bacillus sp. AFS006103	WP_098263121	-S------LF-YY---IMFRKQV-SK	Q	NS-I-LT-C-V-L-M------I-
Bacillus sp. AFS015802	WP_098351073	--------LF-FYI-MIVQKNHFITR	E	S--I-L-IVI-G-VM--M-SGI-
Bacillus sp. AFS031507	WP_098934524	-S------LF-YY--VIMFRKHM-SK	Q	DS-I-LT-C-V-L-M------I-
Bacillus sp. AFS040349	WP_098797594	--------LF--Y-FM--FRKG-ID-	A	NT-I-ISIL--GL-M--INT-I-
Bacillus sp. AFS073361	WP_098576638	-S------LF-YY--IIMFRKHM-SK	Q	NS-I-LT-C-V-L-M------I-
Bacillus sp. B-jedd	WP_048823132	-S------LF-YYA-I-LLG-RTMSR	Q	DS-T-MTI------M------I-
Bacillus sp. EB01	WP_043934360	-S------LF-YYV-V-LLRKGRMSK	E	DS-V-LTI-A----M--F-S-I-
Bacillus sp. FJAT-18017	ALC92565	-S------LF-YYV-V-LLRKGRMSK	E	DS-V-LTI-A----M--F-S-I-
Bacillus sp. FJAT-25496	WP_057775728	-S------LF-FYA-ATKIRKDLISK	G	NS-I-LTIM--GL-M------I-
Bacillus sp. FJAT-27225	WP_066197405	-S------LF-YYV-I-L-KK-RMS-	S	DS-I-LTI-A---VM--F---I-
Bacillus sp. FJAT-27245	WP_053364848	-S------LF-YYI-I-FLRKRGMSR	Q	DS-V-LTI-A---VM------I-
Bacillus sp. FJAT-27251	WP_053361386	-S------LF-FYISMAM-QKHM-SR	E	GS-T-LTI-A---VM--I---I-
Bacillus sp. FJAT-27445	WP_059171512	-S------LF-YYI-I-LLRKRNMTR	Q	DS-V--TI-A--I-M------I-
Bacillus sp. FJAT-27916	WP_049672450	--------LF-CYAYILL-KKHLMTR	Q	NS-T-LA-I-L--VLS-M-TS--
Bacillus sp. FJAT-29814	WP_066321440	-S------LF-YYI-MIVFHKDR-SK	Q	NS-I-IT-C---L-M--IE--I-
Bacillus sp. FJAT-44876	WP_096186800	--------LF-IY-YM-M-RKDLIDP	G	NS-L-MTIVILG-VM--MN----
Bacillus sp. FJAT-44921	WP_078551273	--------LF-LY-YMSL-RKDLID-	A	NS-IVMTILI-GLVM--VNT-I-
Bacillus sp. FJAT-45505	WP_100362064	--------LF-LY-FM-FFEKKLIDP	Q	NARL-LIIS--GLVM--FRA-I-
Bacillus sp. G1(2015b)	WP_058837375	--------LF-VY-Y--LFNPHMMDR	G	NS-V-LTILAV---M--VNS-I-
Bacillus sp. HNG	WP_116354585	-S------LF-IY-YM-VFRKDLIN-	M	NS-L-ITIL--GLVM--INS---
Bacillus sp. I-2	WP_076840769	--------LF-VY-Y--LFKPHMMDR	G	NS-V-LTILAV---M--VNS-I-
Bacillus sp. J33	WP_026580731	--------LF-F-A-IIIFRKDL-SR	E	NS-I-LTIT--G--M------I-
Bacillus sp. JCM 19041	WP_054704191	----S---LF-V--FMYL-RNDL-DR	Q	SA-T-F-II---LVM---GA-I-
Bacillus sp. LF1	WP_090639870	-S------LF-YY-SIIMFRKTM-SR	Q	NS-I-IT-L--NLLM------I-
Bacillus sp. LL01	WP_047972213	-------A-F-LYFYM-FLRPDL-DR	A	NS-V-LTIL--GLVM--MNS-I-
Bacillus sp. LLTC93	WP_105928502	--------LF-VY-Y--LFKPHMMDR	G	NS-V-LTILGV--VM--VNS-I-
Bacillus sp. LNXM65	WP_106039297	--------LF-VY-Y--LFKPHMMDR	G	NS-V-ITILAV---M--VNS-I-
Bacillus sp. MRMR6	WP_075690249	-S--S---LF-FYISILAFR--T-S-	Q	NS-I-LIICAL-L-M--I---I-
Bacillus sp. MUM 116	WP_071354171	-S------LF-YY--IILFRK-W-SR	Q	NS-V-LV-CAL-L-M------I-
Bacillus sp. Marseille-P3800	WP_099303530	----S-Y-LF-VY-YIYL-RNDLISR	Q	TS-I----V--G--M--IN-GIS
Bacillus sp. NMCC4	WP_106031675	--------LF-VY-Y--LFKPHMMDR	G	NS-V-ITILAV---M--VNS-I-
Bacillus sp. NMCN1	WP_106056743	--------LF-VY-Y--LFKPHMMDR	G	NS-V-ITILAV---M--VNS-I-
Bacillus sp. NMCN6	WP_106051342	--------LF-VY-Y--LFKPHMMDR	G	NS-V-ITILAV---M--VNS-I-
Bacillus sp. NSP9.1	WP_026585990	--------LF-VY-YIALFKKELMGR	E	NS-I--TILAV--LM--INQ-I-
Bacillus sp. OG2	WP_094769350	-S------LF-F-A-VA-FRKDLMSR	E	NS-I-ITI---GL-M--I---I-

Other Bacteria (0/>200)

Bacillus sp. OK048	WP_090767836	-S------LF-YYISIIVFRKHM-S-	Q	NS-I--IICAL-L-M--I---I-
Bacillus sp. OK085	WP_132097003	-S------LF-YYISIILFRKHM-S-	Q	NS-I-LIICAL-LVM--I---I-
Bacillus sp. P003	KXI31264	--------LF-VY-Y--LFKPHMMDR	G	NS-V-LTILAV---M--VNS-I-
Bacillus sp. P14.5	WP_113927684	--------LF-IYVGMTLFHKHLIS-	Q	NK-V-I-IV--GLVM--F-S-I-
Bacillus sp. SA1-12	WP_046515952	--------LF--Y-FM-FFRKGMMD-	A	NS-I-ISIL--GL-M--FNA-I-
Bacillus sp. SG-1	WP_006838207	--------LF-IYVGMTLFHKHLIST	Q	NK-V-I-IV--GLVM--F-A-I-
Bacillus sp. SKP7-4	WP_119547299	--------LF-IYVGMALFHKHLIST	Q	NK-V-I-IV--GLVM--F-S-I-
Bacillus sp. T33-2	WP_101582488	-S------LF-FYI-IITFK-FM-SK	E	NS-T-MTI-A-GI-M--I---I-
Bacillus sp. UMB0728	WP_101552655	-S------LF-F-A-VA-FRKDLMSR	E	NS-I-ITI---GL-M--I---I-
Bacillus sp. UMB0893	WP_101569213	--------LF-IY-Y--VFRKDMIDS	S	NS-L-ITIL--G-VM--INT-I-
Bacillus sp. UNC41MFS5	WP_026563207	-S------LF-YY--MIMFRKHM-SK	Q	NS-I-LT-C-V-L-M------I-
Bacillus sp. UNC438CL73TsuS30	WP_035424107	-S------LF-YYV-IIIFRK-W-SR	Q	NSHI-LV-C-L-L-M----S-I-
Bacillus sp. URHB0009	WP_027324007	-S-------F-FYA--IVFKK-FISV	D	NSKV-LI-LA--LVM------I-
Bacillus sp. V33-4	WP_101666940	-S------LF-FY--MFTFR-YM-SR	Q	NA-I-LTIS--GI-M--I---I-
Bacillus sp. VT-16-64	WP_077111425	--------LF-IY--IIALKKGAM-P	Q	GK-V-L-M-A-G--M--F-SGI-
Bacillus sp. WN066	WP_133339278	-S------LF-YYV-IIIFRK-W-SR	Q	NSHI-LV-C-L-L-M------I-
Bacillus sp. X1(2014)	WP_038538749	-S------LF-YYI-IIVFRK---SK	Q	NS-I-IT--I--LVM--VE--I-
Bacillus sp. Y1	WP_119706358	-S------LF-IY--MIFFRKEM-SR	E	NT-VVLSIV--G--M--VNA---
Bacillus sp. YR335	WP_111618305	--------LF-VY-FM--FR-GMID-	A	NS-I-LSML--GL-M--FNS-I-
Bacillus sp. YSP-3	WP_110521606	--------LF-MYMYM-L-RKDLID-	G	NQ-VVMTILM-GL-M--VN--I-
Bacillus sp. es.034	WP_098438451	--------LF-FYI-MIVLKNHFITR	E	S--I-L-IVI-G-VM--M-SGI-
Bacillus sp. es.036	WP_098446057	--------LF-IYFYMAF-RKELID-	A	NS-L-LMILG-GLVM--IS--I-
Bacillus sporothermodurans	WP_066236177	------L-LF-MYV-TIVLKKHM-TM	S	IQ-I---IV--AL-MS--E----
Bacillus subtilis group	WP_003328136	--------LF-VY-YM-L-RKELID-	E	NSK--IT-L-VA-LM--INS-I-
Bacillus swezeyi	WP_076761265	--------LF-VY-YI-LFRKELMGS	A	NS-I--TILA---LM--INH-I-
Bacillus terrae	WP_120119270	--------LF-IY-SIIFLKKEAM--	Q	GK-V-L-I-ALG--M--F-A-I-
Bacillus tuaregi	WP_071396438	--------LF-MYISMNIFHKHL-SR	D	N--L-TTI----I-M--F---I-
Bacillus vietnamensis	WP_060673919	--------LF-FYI-MIVLKNHFITR	E	S--I-L-IVI-G-VM--M-SGI-
Bacillus vireti	WP_024030985	-S------LF-YYI-IIMFRKHW-SR	Q	NS-I-LT-C---L-M------I-
Bacillus weihaiensis	WP_072581375	--------LF-IY-FM--FRKGMID-	A	NS-I-LSIL--G--M---SA-I-
Bacillus xiamenensis	WP_008359545	--------LF-VY-Y--LFKPHMMDR	G	NS-V-LTILGV---M--VNS-I-
Bacillus zhangzhouensis	WP_034323995	--------LF-VY-Y--LFKPHMMDR	G	NS-V-LTILGV--VM--VNS-I-
Citricoccus massiliensis	WP_102415904	------Y-LF-IYVYMIVFRKDLID-	A	SS-VVLIIF--GL-M---R--I-
Clostridiales bacterium mt7	WP_101729028	-S------LF-YYIS--VFRKD-IS-	S	NA-I-TTI--L-L-M------I-
Fictibacillus aquaticus	WP_094253944	----S---LF-IYVYIL-NR-EY-NR	S	N--ILLTVL-L-L-M--MDDRI-
Fictibacillus enclensis	WP_061975868	--------LF-IY-YIL-ARKEY-GR	A	NK-ILIVVL-F-L-T--TT-GI-
Fictibacillus solisalsi	WP_090239405	--------LF-IY-YIL-ARKEY-GR	A	NK-ILIVVL-F-L-T--TT-GI-
Fictibacillus sp. S7	WP_129478425	--------LF-IY-YIL-ARKEY-GR	A	NK-ILIVVL-F-L-T--TT-GI-
Gracilibacillus boraciitoleran	WP_035723839	--------LF-LY-FM-FFEKRLIDP	Q	NAKL-LIIS---LVM---RD-I-
Gracilibacillus halophilus	WP_003470839	--------MF-LY-FM-FFEKHLID-	Q	SA-IVLI-T---LVM--IR-QI-
Gracilibacillus kekensis	WP_073202956	--------LF-LY-FM-FFEKRLIDP	Q	NAKL-LIIS---LVM--FRG-I-
Gracilibacillus lacisalsi	WP_018932009	--------LF-LY-FM-FFEKRLIDP	Q	NARL-LIIS--GLVM--FRA-I-
Gracilibacillus massiliensis	WP_058305783	--------LF-LY-FM-FFEKRLIDP	Q	NAKL-LIIS---LVM--FRG-I-
Gracilibacillus orientalis	WP_091485307	--------LF-LY-FM-FFEKRLIDP	Q	NARL-LIIS--GLVM--FRS-I-
Gracilibacillus phocaeensis	WP_130859112	--------LF-LYIFMIFFEKKLID-	Q	SA-L-FIISAVGLVM---R--I-
Gracilibacillus timonensis	WP_066187471	--------LF-LYIFMIFFEKKLID-	Q	SA-L-FIISAVGLVM---R--I-
Halalkalibacillus sp. B3227	WP_101331818	--------LF-LYVFMLLFRQDL-DS	Q	S----MVIVIFALVM--VNS-I-
Halobacillus alkaliphilus	WP_089752257	---------F-VYVFM-LFRKHLID-	A	NS-IVMVIFILGLFM--TR--I-
Halobacillus halophilus	WP_014641669	---------F-VYVFM-LFRKHLID-	A	NS-IVMVIFILGLFM--TR--I-
Halobacillus kuroshimensis	WP_027956791	---------F-VY-FM-LFQKHLID-	A	NA-I-IVIFALGLFM--TR--I-
Halobacillus litoralis	WP_128524736	---------F-VYVFM-LFRKHLID-	A	NS-I-LVIFALGLFM--AR--I-
Halobacillus mangrovi	WP_085031298	---------F-VYVYM-L-RKHLID-	A	NS-I-MVIFILGLFM--TR--I-
Halobacillus sp. BBL2006	WP_035552892	---------F-VYVYM-LFRKHLID-	A	NS-I-MVIFILGLFM--TR--I-
Jeotgalibacillus alimentarius	WP_041121349	--------LF-LYG-IWLIYKQAA-L	E	I--VM--IIA---VM--FGS-I-
Jeotgalibacillus campisalis	WP_041056353	--------LF-VYA-ILLIFKERA-A	E	---IML--LI----M--FT----
Jeotgalibacillus malaysiensis	WP_039807344	--------LF-LYA-IWLIYKQGA-M	E	I--VM--II----VM--FSS-I-
Jeotgalibacillus proteolyticus	WP_104057302	--------LF-VYA-ILFIYKERA-A	E	-K-ILL-IL-----M--FGA-I-
Jeotgalibacillus salarius	WP_134380440	--------LF-LYG-IWLIYKQAA-M	E	I--VM--VI----VM--FGS-I-
Jeotgalibacillus soli	WP_041086015	----S---LF-VYA-ILFLFKKRA-F	E	---VML--LI----M--F-----
Jeotgalibacillus sp. R-1-5s-1	WP_134374859	--------VF-LYA-IYFIY-ERA-M	E	I--VML--II----M--FGS-I-
Jeotgalibacillus sp. S-D1	WP_133378537	--------LF-LYA-ILLIFKERA-M	E	---V-L--L-----M--FSA---
Melghiribacillus thermohalophi	WP_132372174	------Y-LF-IYVYMMM-KKHLMDD	Q	SS-IVKVILILGL-M---RT-I-
Mycobacteroides abscessus subs	SHS47608	-S------LF-IY--MIFFRKEM-SR	E	NT-VVLAIV--G--M--VNA---
Natribacillus halophilus	WP_090399178	------Y-L--LY-YMIIFRKEL-DP	Q	S--VVTIILI-GIVM---V-GI-
Oceanobacillus iheyensis	WP_106895722	-----V--LF-IYIFMRFFRKHL-DY	Q	SS---TVII--GL-M---R--I-
Oceanobacillus limi	WP_090872821	------Y--F-IYIFM-AFRKHLIDS	A	NS-I-MTIFI-GLVM------I-
Oceanobacillus massiliensis	WP_010651753	--------LF-IYTFM-LFRKHLIDY	S	SS---TTILI--LVM---RN-I-
Oceanobacillus picturae	WP_036577159	-----V--LF-IYIFMRFFRKHL-DY	Q	SS---TVII--GL-M---R--I-
Oceanobacillus profundus	WP_118889732	-----V--LF-IYAFM-SFRKHLID-	G	SS-IVMTILI-GLAM---RS-I-
Oceanobacillus sp. YLB-02	WP_121524473	--------LF-VYIFM-MNRKHLID-	G	SAK--SVILA--L-M--I---I-
Ornithinibacillus californiens	WP_047985425	---------F-IY-FM-AFRKHLMDS	Q	SS-I-TVILI-GLVM---R-GI-
Paenibacillus sp. 7884-2	WP_095311876	-----V--LF-IYAFM-SFRKHLID-	G	SS-IVMTILI-GLAM---RS-I-
Paenibacillus sp. FSL R5-0490	WP_076263007	--------LF-Y-A-ILVFRKEL-SR	E	NS-I-LTITI-G--M------I-
Pontibacillus chungwhensis	WP_036786633	--------LF-VY-YMI-AKKHLID-	A	NA-IVLTIT--GLVM--IR--I-

Other Bacteria (0/>200)

Pontibacillus halophilus	WP_026801227	----S-Y-LF-FY-FMILFRQRLIDE S SK-IVTVIL--GI-M---RSGI-
Pontibacillus litoralis	WP_036834280	--------LF-IY-YM--SRKDLID- A NA-VVITI---G--M-LIS-GI-
Pontibacillus marinus	WP_027446715	--------LF-IY-YMI-AR-ELID- A NT-I-LTIS--ALVM---RS-I-
Salibacterium halotolerans	WP_093339115	--------LF-IY-YM-L-RKDLIDA M NARI-MVIT-LG-VM---S--I-
Salibacterium qingdaonense	WP_090925247	--------LF-IY-YM-L-RKDLIDA M NARI-MVIT-LG-VM---S--I-
Salimicrobium album	WP_093107977	--------LF-IYAYMLFARKDL-D- A SS-I-GVII-LGL-M------I-
Salimicrobium flavidum	WP_076559914	--------LF-IYAYMLFARKEL-D- V SS-I-GVII-LGL-M------I-
Salimicrobium halophilum	WP_093194497	--------LF-IYAYMLFARKDL-D- A SS-I-TVIIALGL-M------I-
Salimicrobium jeotgali	WP_008591051	--------LF-IYAYMLFARKDL-D- A SS-I-GVFI-LGL-M------I-
Salimicrobium salexigens	WP_076571838	--------LF-VYAYMLFARKDL-D- A SS-I-GVII-LGL-M------I-
Salinibacillus kushneri	WP_093132445	--------LF-TY-FIILFRKEL-DY Q ST-I-TAIMI-AL-M---RT-I-
Salipaludibacillus aurantiacus	WP_093055083	-----V--LF-IYVYM-VFRKDLINR M NS-L-MTIV-LG--M--IN-GI-
Salipaludibacillus neizhouensi	WP_110939291	------Y-LF-VY-YM-F-RKDLIDK A NS-L-LTVL--G-VM--IN-GI-
Salipaludibacillus sp. KQ-12	WP_110611702	--------LF-VYVYM-LVRKDLID- T NS-L-MTIV-LG--M--IN-GI-
Salirhabdus sp. Marseille-P466	WP_102029355	-----V---F--Y-FI-LFRKHLIDD Q NK-I--TIM--AL-M--VRS-I-
Salisediminibacterium haloalka	WP_093072509	--------LF-LY-YI-LMRKEL--- A SQ-I-LTII--G--M--VN-GI-
Salisediminibacterium halotole	WP_121440029	--------LF-LY-YI-LMRKEL--- A SQ-I-LTII--G--M--VN-GI-
Salsuginibacillus kocurii	WP_018924797	------Y-L--LY-YM-M-R-DLID- A SS----IILA-GM-M--IT--I-
Sediminibacillus halophilus	WP_074600600	------Y-LF-MYMYM-FFRKHLIDR A NA-IVTTILI-GL-M---R-GI-
Sinobaca qinghaiensis	WP_120192696	-----V--VF-IYMY-IWKRKDLIDA A SA-L-LI-TLF--VM---S--I-
Thalassobacillus cyri	WP_093046704	---------F-VY-FM-A-RK-MID- A NS-IVMVIF--GLVM--VR--I-
Thalassobacillus devorans	WP_028785202	--------LF-VY-FM-A-RKHLID- A NS-IVMVIFI-GLVM--IR--I-
Thalassobacillus sp. TM-1	WP_062439065	---------F-VY-YM-ANRKHLID- A NS-IVMVIL-LG-VM--VR--I-
Virgibacillus chiguensis	WP_073013057	--------LF-IYIFM-LFRKHLID- I SS-IVTTIF--GL-M--FN-GI-
Virgibacillus dakarensis	WP_088051044	------Y-LF-IYVYMIVFRKDLID- A SS-VVLIIF--GL-M---R--I-
Virgibacillus dokdonensis	WP_077702485	--------LF-IYIFM-LFRKHLID- I SS-IVTTICI-GL-M--FN-GI-
Virgibacillus halodenitrifican	WP_019379223	--------LF-IYIFM-VFRKHLID- A NS-IV-TIF--GLVM--VRS-I-
Virgibacillus indicus	WP_094885580	--------LF-IYVFM-GIRKHLID- A NS-IVMTILI-GLVM---RS-I-
Virgibacillus ndiopensis	WP_099156477	--------LF-IYVYM-LFRKDLID- A NA-IVLVISI-GL-M---R-GI-
Virgibacillus pantothenticus	WP_050351129	--------LF-IYIFM-LFRKHLID- I SS--VTTILI-GLVM--FN-GI-
Virgibacillus profundi	WP_095654673	--------LF-IYVFMIAFRKSLID- A NS-IVMTIFI-GL-M---RS-I-
Virgibacillus proomii	WP_077318373	--------LF-IY-FM-SFRKHLID- V SS-I-TTIL--GL-M--IR--I-
Virgibacillus senegalensis	WP_053217411	------Y-LF-MYMYM-FFRKHLIDR A NA-IVTTILI-GL-M---R-GI-
Virgibacillus sp. 6R	WP_072741265	--------LF-IYIFM-LFRKHLID- I SS--VTTILI-GLVM--FN-GI-
Virgibacillus sp. Bac330	WP_121640655	--------LF-IYIFM-LFRKHLID- I SS-IVTTIF--GL-M--FN-GI-
Virgibacillus sp. Bac332	WP_121604536	--------LF-IYIFM-TFRKHLID- T SS-I-LTIVIVGL-M--IRQ-I-
Virgibacillus sp. LJ137	WP_123314584	------Y-LF-IYIFI-AFRKHLMD- Q ST-IVMIIFI-GLVM--I---I-
Virgibacillus sp. SK37	WP_040954275	--------LF-IYIFM-VFRKHLID- A NS-IV-TIF--GLVM--VRS-I-

Supplemental Figure 8

A partial sequence alignment of the rhomboid family intramembrane serine protease protein containing a one amino acid deletion (boxed) that is exclusively shared by all members among the *Planococcaceae* family and absent in all other bacteria.

		40		99
Bacillus cecembensis	WP_057986366	REGGGIAAKALEAINISPQMIESGIEELVGKG	T	EEVGPIVHYTPRAKKVIELSLDESRKL
Bacillus ndiopicus	WP_042478561	--------------GV---T------------	-	-K--LT-S------------V------
Bacillus sp. FJAT-22090	WP_053591287	---S----------EVD-KV--A---------	-	--------------------V------
Bacillus sp. OxB-1	WP_041072062	--------------DV-FET--Q-V-K---T-	S	KD------------------V------
Bhargavaea beijingensis	WP_092093108	--------------GVNFET--E-V-K---T-	D	R-----I--------------------
Bhargavaea cecembensis	WP_008298940	--------------G-NFET--E-V-K---T-	D	RD----I--------------------
Bhargavaea ginsengi	WP_092053184	--------------G-NFET--E-V-K---T-	D	R-----I--------------------
Caryophanon latum	WP_066462870	--------------DV----------------	-	-K------------------V------
Caryophanon tenue	WP_066542774	--------------DV------------M---	-	-T------------------V------
Kurthia gibsonii	WP_121176059	--------------EV--E---E-------T-	S	KN------------------V------
Kurthia huakuii	WP_029500622	--------------EVT-EV--E---K---V-	-	KD---V--------------V------
Kurthia massiliensis	WP_010290458	--------------EVT-EV--E---K---V-	-	KD---V--------------V------
Kurthia senegalensis	WP_010308086	---T----------EVT-E---E---K---V-	-	KDS--V--------------V------
Kurthia sibirica	WP_109305706	--------------EV--EI--E---D---V-	-	KN------------------V------
Kurthia sp. 11kri321	WP_068450076	--------------EV--E---E-------T-	S	KN------------------V------
Kurthia zopfii	WP_109349805	--------------EV--E-----------V-	A	KN------------------V------
Lysinibacillus acetophenoni	WP_097149296	--------------DV---I------------	-	KD------------------V------
Lysinibacillus chungkukjangi	WP_107932243	--------------DV---I------------	A	KD------------------V------
Lysinibacillus composti	WP_124765581	--------------DV---I------------	S	KD------------------V------
Lysinibacillus endophyticus	WP_121214159	--------------DV---I------------	Q	KD------------------V------
Lysinibacillus fusiformis	WP_004233576	--------------D-------A---------	K	-D------------------V------
Lysinibacillus halotolerans	WP_122971242	--------------DV---I------------	S	KD------------------V------
Lysinibacillus macroides	WP_053997205	--------------D----R--A---------	K	-D------------------V------
Lysinibacillus manganicus	WP_036183236	--------------DV---I------------	-	KD------------------V------
Lysinibacillus mangiferihumi	WP_107894983	--------------D-------A---------	-	-D------------------V------
Lysinibacillus massiliensis	WP_036172376	--------------DV----------------	-	KD------------------V------
Lysinibacillus meyeri	WP_107839090	--------------GV---T--E---------	-	-K--LT-S------------V------
Lysinibacillus odysseyi	WP_036152024	--------------D-----------------	-	-N------------------V------
Lysinibacillus parviboronicapi	WP_107925065	--------------D-------A---------	-	-D------------------V------
Lysinibacillus sinduriensis	WP_036200795	--------------DV---I------------	A	KD------------------V------
Lysinibacillus sp. 2017	WP_108714320	--------------------------------	-	---------------------------
Lysinibacillus sp. AC-3	SKB94325	--------------D-------A---------	K	-D------------------V------
Lysinibacillus sp. B2A1	AVK86727	--------------D----T--A---------	K	-D------------------V------
Lysinibacillus sp. BK089	WP_132362647	--------------D-----------------	K	-D------------------V------
Lysinibacillus sp. FJAT-14222	WP_053596029	--------------D-------A---------	K	-D------------------V------
Lysinibacillus sp. FJAT-14745	WP_053482837	--------------D-------A---------	K	-D------------------V------
Lysinibacillus sp. Marseille-P	WP_106781880	--------------DV----------------	-	KD------------------V------
Lysinibacillus sp. SYSU K30002	WP_126658142	--------------D----I------------	Q	KD------------------V------
Lysinibacillus sp. YLB-03	WP_118876504	--------------DV---I------------	A	KD------------------V------
Lysinibacillus sp. YR326	TDV01291	--------------D-----------------	K	-D------------------V------
Lysinibacillus sp. ZYM-1	WP_054610153	--------------D-------A---------	K	-D------------------V------
Lysinibacillus sphaericus	WP_010860635	--------------D-------T---------	-	-D------------------V------
Lysinibacillus telephonicus	WP_126292348	--------------DV----------------	-	KD------------------V------
Lysinibacillus xylanilyticus	PJ044252	--------------D-----------------	K	-D------------------V------
Lysinibacillus xyleni	WP_097073423	--------------DV---I------------	Q	KD------------------V------
Paenisporosarcina antarctica	WP_134208103	----------M---EV-TK---EN------M-	-	GD------------------V------
Paenisporosarcina indica	WP_075620218	----------M---EV---V--E-------M-	-	GD------------------V------
Paenisporosarcina quisquiliaru	WP_090567710	---S----------EVDLKV--T-------A-	K	-------N------------V------
Paenisporosarcina sp. HGH0030	WP_016429855	----------M---EV------E-------M-	-	GD------------------V------
Paenisporosarcina sp. K2R23-3	WP_119884354	---S----------EV--EV--Q-------V-	E	GD------------------V------
Paenisporosarcina sp. OV554	WP_108585974	----------M---EV---V--D-------M-	-	GD------------------V------
Paenisporosarcina sp. TG-14	WP_017380996	----------M---EV-TK---EN------M-	-	GD------------------V------
Paenisporosarcina sp. TG20	WP_019413491	----------M---EV--K---DS------M-	-	G-------------------V------
Planococcus antarcticus	WP_040851970	--------------EVNT-L--E-VK----V-	E	KD------------------V------
Planococcus antarcticus DSM 14	EIM07670	--------------EVNT-L--E-VK----V-	E	KD------------------V------
Planococcus donghaensis	WP_008432444	--------------EVNT-L--E-VK----V-	E	K-------------------V------
Planococcus faecalis	WP_071153294	--------------EVNT-L--E-VK----V-	E	KD------------------V------
Planococcus halocryophilus	WP_065528387	--------------EVNT-L--E-VK----V-	E	K-------------------V------
Planococcus halotolerans	WP_112224731	--------------EVNT-L--E-VT----I-	E	KD------------------V------
Planococcus kocurii	WP_058386758	--------------EVNT-L--E-VK----V-	E	KD------------------V------
Planococcus maritimus	WP_068459631	--------------EVNT-L--D-VK----V-	E	KD------------------V------
Planococcus massiliensis	WP_052654245	--------------EVNT-L--E-VK----V-	E	KD------------------V------
Planococcus plakortidis	WP_068871294	--------------EVNT-L--D-VK----V-	E	KD------------------V------
Planococcus rifietoensis	WP_058382199	--------------EVNT-L--D-VK----V-	E	KD------------------V------
Planococcus salinarum	TAA67132	--------------EVNT-L--E-VT----I-	E	KD------------------V------

Family *Planococcaceae* (>100/>100)

Group	Organism	Accession	Alignment	Boxed position	Alignment (continued)
**Family *Planococcaceae* (>100/>100)**	Planococcus salinus	WP_123166219	---------S----EVNT-L--E-VK----V-	E	KD-----------------V------
	Planococcus sp. CAU13	WP_033542826	-------------EVNT-L--E-VT----I-	E	KN-----------------V------
	Planococcus sp. PAMC 21323	WP_038701838	--------------EVNT-L--E-VK----V-	E	K------------------V------
	Planococcus sp. Y42	WP_077587613	--------------GVN-KL--D--K--I-V-	E	Q------------------V------
	Planococcus versutus	WP_049694514	--------------EVNT-L--E-VK----V-	E	KN-----------------V------
	Planomicrobium	WP_101801670	--------------EVNT-L--E-VT----I-	E	KD-----------------V------
	Planomicrobium flavidum	WP_088006725	--------------GVN--L--D--K--I-V-	E	-------------------V------
	Planomicrobium glaciei	WP_036803827	--------------EVNT-L--E-VK----V-	E	KD-----------------V------
	Planomicrobium okeanokoites	WP_084242213	--------------EVNT-L--E-VT----I-	E	KD-----------------V------
	Planomicrobium soli	WP_106534504	--------------EVNT-L--E-VK----V-	E	KN-----------------V------
	Planomicrobium sp. Y74	WP_121636470	--------------EVNT-L--E-VT----I-	E	KD-----------------V------
	Solibacillus isronensis	WP_079523514	----------------------T---------	-	-D------------------------
	Solibacillus sp. R5-41	WP_099425627	-------------------K------------	-	--------------------------
	Sporosarcina koreensis	WP_040285457	--------------GV-FET----V-N-I-T-	S	KD-----------------V------
	Sporosarcina newyorkensis	WP_040759940	-----------D--SVNADT--REV-A---V-	S	KD-----------R-----V------
	Sporosarcina pasteurii	WP_115363690	--------------EV-FTT--E-V-K---T-	-	KD-----------------V------
	Sporosarcina psychrophila	WP_067204678	--------------GVNFET-VQ-V-K---A-	-	KD-----------------V------
	Sporosarcina sp. BI001-red	WP_116018394	--------------GV-FDT--T-V-T---T-	S	K------------------V------
	Sporosarcina sp. D27	WP_025782843	--------------GV-FET--T-V-T---T-	S	KD-----------------V------
	Sporosarcina sp. EUR3 2.2.2	WP_024536477	----------M---EV---V--D-------M-	-	GD-----------------V------
	Sporosarcina sp. HY008	WP_067407674	---T----------DV-LET--Q-V-K---Y-	A	K------------------V------
	Sporosarcina sp. P13	WP_099689144	---------------VNADT--REV-I---V-	S	KD-----------R-----V------
	Sporosarcina sp. P2	WP_099630515	--------------SVNADT--REV-I---V-	S	KD-----------R-----V------
	Sporosarcina sp. P20a	WP_099678374	--------------SVNADT--REV-I---V-	S	KD-----------R-----V------
	Sporosarcina sp. P33	WP_081243235	---------------V-ADT--REV-I---V-	S	KD-----------R-----V------
	Sporosarcina sp. P34	WP_099695009	--------------SV-ADT--REV-I---V-	S	KD-----------R-----V------
	Sporosarcina sp. PTS2304	WP_114925932	---------------VNADT--REV-I---V-	S	KD-----------R-----V------
	Sporosarcina ureae	WP_029053303	--------------SVNADT--REV-I---V-	S	KD-----------R-----V------
	Psychrobacillus insolitus	WP_111440109	---S----------EVD-KV--A-V-----S-	-	-D-----------------V------
	Psychrobacillus psychrodurans	WP_093495452	---S----------EVDLKV--T-------A-	K	-------N-----------V------
	Psychrobacillus psychrotoleran	WP_093537890	---S----------EVDLKV--A-------A-	K	-------N-----------V------
	Psychrobacillus sp. FJAT-21963	WP_056831206	---S----------EVD-KV--A---------	-	-------------------V------
	Psychrobacillus sp. OK028	WP_093062460	---S----------EVDLKV--A-------A-	K	-------N-----------V------
	Psychrobacillus sp. OK032	WP_093274932	---S----------EVDTKV--V-------T-	A	-------N-----------V------
	Rummeliibacillus pycnus	WP_102693520	--------------DV-SE---E-------M-	-	KN-----------------V------
	Rummeliibacillus sp. POC4	WP_119415324	--------------DV-TE---E-------M-	-	KN-----------------V------
	Rummeliibacillus sp. TYF005	WP_124217391	--------------DV-TD---E-------M-	-	KN-----------------V------
	Rummeliibacillus stabekisii	WP_066790982	---------------V-TE---E-------V-	-	KN-----------------V------
	Tetzosporium hominis	WP_094942240	---N---------S-VD-KA--E-------T-	E	KD-----------------V------
	Ureibacillus thermophilus	QBK24900	--------------DVT-E---R---------	-	KD----------------AV------
	Ureibacillus thermosphaericus	WP_016838527	--------------DV--E---R---------	-	KD-----------------V------
	Viridibacillus arvi	WP_053418519	--------------D--SE---E-------V-	-	KD-----------------V------
	Viridibacillus sp. OK051	WP_100795257	--------------DV--E---E-------V-	A	KD-----------------V------
	Butyricicoccus sp. 1XD8-22	RKJ69910	--------------DV----------------	-	KD-----------------V------
	Chryseomicrobium excrementi	WP_100354121	---N---------S-VD-KA--E-------T-	E	KD-----------------V------
	Edaphobacillus lindanitolerans	SIT87497	--------------GVNFDT--E-V-K---T-	-	K-----I-------------------
	Filibacter sp. TB-66	WP_124068584	--------------D--LET--Q-V-K---S-	-	K------------------V------
**Other Bacteria (2/>100)**	Bacillus amyloliquefaciens	KNX35631	---E---F-----LGLNSDKMQKEV-S-I-R-	Q	-STTSVP------------M--A---
	Bacillus halotolerans	WP_059336730	---E---F-----LGLNSDKVQKEV-S-I-R-	Q	-STTSVP------------M--A---
	Aeribacillus pallidus	WP_063387130	---E-------H-LGL--EK-QNEV-S-I-R-		Q-ISQTI------------M--A---
	Alkalibacillus haloalkaliphilu	WP_017186389	K--D-------KSL-VTVEQ-QEEV-Q-I-T-		QSNQQTI------------M--A---
	Alkalicoccus saliphilus	WP_107585346	---E-------TGLGLGAEK-QTEV-N-I-R-		D--TKQI------------M--A---
	Alteribacillus persepolensis	WP_091275095	---E-------Q-L-LGSEQ-QKEV-S-I-T-		Q-GSKTI------------M--A---
	Anaerobacillus macyae	WP_048313290	---E-------T-LGLGSEK-QKEV-K-I-R-		QDSVQSI------------M--A---
	Anoxybacillus flavithermus	WP_003397622	---E-------M-LGLG-DK-QKEV-S-I-R-		N---QTI------------M--A---
	Anoxybacillus kamchatkensis	WP_026011880	---E-------M-LGLG-DK-QKEV-S-I-R-		N---QTI------------M--A---
	Aureibacillus halotolerans	WP_133582023	---E-------S-LGLGAEK-QNEV-A-I-M-		Q-ASQTI------------M--A---
	Bacillaceae bacterium	REJ18045	---E-------H-LGL--EK-QNEV-S-I-R-		Q-ISQTI------------M--A---
	Bacillus acanthi	WP_108672044	---D-------Y-LGLGAEK-QKEV-N-I-R-		QDTAQTI------------M--A---
	Bacillus acidiproducens	WP_018663989	---E-------YGLGL-SEK-QEEV-N-I-N-		Q-ISQTI------------M--A---
	Bacillus cereus	BAB88968	---E-------I-LGL--EKVQKEV-A-I-R-		T-ASQT-------------M--A---
	Bacillus nakamurai	WP_061521308	---E-------Q-LGLGSDK-QKEV-S-I-R-		Q-MSQTI------------M--A---
	Citricoccus massiliensis	WP_102414317	---D-------QSLGLEVEK-QQEV-Q-I-V-		KQAMQTI--------V---Q--A---
	Domibacillus enclensis	WP_045851827	---E-------YGLGL-AEK-QEEV---I---		DGASKT-------------M--A---
	Domibacillus epiphyticus	WP_076764360	---E-------Y-LGLGAEK-QEEV---I---		DGASKT-------------M--A---

Other Bacteria (2/>100)

Domibacillus tundrae	WP_046180715	---E-------YGLGL-AEK-QEEV---I---	DGASKT-------------M--A---
Exiguobacterium alkaliphilum	WP_034817108	---E-------F-LGMTSEK-QQEV-A-I-R-	S-N-STI------------M--A---
Filobacillus milosensis	WP_134340949	K--E-------QSL-VTAEQ-QQEV-Q-I-R-	QNTSQTI------------M--A---
Geobacillus zalihae	OQP18564	---E-------M-LGLG-DK-QKEV-S-I-R-	S--SHTI------------M--A---
Gracilibacillus boraciitoleran	WP_035724202	--D-------Q--GLGADK-REEV-S-I---	NQ-SQTI------------M--A---
Gracilibacillus dipsosauri	WP_109985146	--D-------H--GLGAEK-REEV-A-I---	NQ-SQTI------------M--A---
Gracilibacillus ureilyticus	WP_089743647	--E-------H--GLGADK-REEV-S-I---	NQ-SQTI------------M--A---
Halalkalibacillus halophilus	WP_027964768	K--D-----S-K-L-VTAEQ-Q-EV-Q-I-T-	QNSAQTI------------M--A---
Halolactibacillus alkaliphilus	WP_089801879	K--D-------TSLGLKFED-QEEV-V-I-A-	QKQSQTI------------M--A---
Halolactibacillus halophilus	WP_089833798	K--D-------TSLGLKFED-QEEV-L-I-T-	QKHSQTI------------M--A---
Jeotgalibacillus campisalis	WP_041054616	---E-------KSL-L-TEK-QEEV---I---	T-QS-S-------------M--A---
Jeotgalibacillus proteolyticus	WP_104059712	---E-------KSL-L-TDK-QEEV---I---	T-QS-S-------------M--A---
Jeotgalibacillus soli	WP_041085386	---E-------QSL-L-SDK-QEEV---I---	T-QS-S-------------M--A---
Klebsiella pneumoniae	OON45095	---E-------I-LGL--EKVQKEV-A-I-R-	T-ASQT-------------M--A---
Lentibacillus amyloliquefacien	WP_068446161	---N--------SLGLEVTK-QQEV-K-I-T-	KQPMQTI---------V---Q--A---
Lentibacillus halodurans	WP_090240828	---N--------SLGMEVSK-QQEV-K-I-A-	KQPMQTI---------V---Q--A---
Listeria ivanovii FSL F6-596	EFR98339	---E-------YELGV-AEKVQQEV-G-I-H-	-KAVTTIQ-----------M--A---
Marinococcus halophilus	WP_079475081	---E-------Q-L-LDTDQ-QQEV-T-I-T-	QQGSKTI------------M--A---
Marinococcus halotolerans	WP_022794439	---E-------Q-L-LDTDQ-QQEV-T-I-T-	QQGSKTI------------M--A---
Marinococcus luteus	WP_091616765	---E-------Q-L-LDTDQ-QQEV-T-I-T-	QQGSKTI------------M--A---
Melghiribacillus thermohalophi	WP_132371857	---E-------G-L-VKADQ-QKEV-Q-I-T-	QQ-SQTI------------M--A---
Mycobacteroides abscessus subs	SHS96017	---E-------Y-LGLGSEK-QKEV-N-I-R-	QDASQTI------------M--A---
Oceanobacillus arenosus	WP_115771354	---E-------QSLGLEVPK-QEEV-K-I-T-	KQQMQSI---------V---Q--A---
Oceanobacillus senegalensis	WP_085993710	---E-------QSLGLEISQ-QEEV-K-I-V-	KQPMQSI---------V---Q--A---
Ornithinibacillus californiens	WP_047983311	---E--------SLGLEVSK-QEEV-K-I-T-	KQPMQTI---------V---Q--A---
Ornithinibacillus contaminans	WP_047981986	---E--------SLGLEVAK-QDEV-K-I-T-	KQPMQTI---------V---Q--A---
Ornithinibacillus halophilus	WP_072890061	------------SLGLEVEK-QEEV-K-I-V-	KQPMQTI---------V---Q--A---
Ornithinibacillus scapharcae	WP_010095812	---E--------SLGLEVSK-QEEV-K-I-T-	KQPMQTI---------V---Q--A---
Parageobacillus caldoxylosilyt	WP_061579341	---E-------M-LGLG-EK-QKEV-S-I-R-	Q--SHTI------------M--A---
Parageobacillus thermantarctic	WP_090949418	---E-------M-LGLGLEK-QKEV-S-I-R-	H--SHTI---------L---M--A---
Parageobacillus thermoglucosid	WP_064550569	---E-------M-LGLG-EK-QKEV-S-I-R-	H--SHTI------------M--A---
Parageobacillus toebii	WP_062677076	---E-------M-LGLG-EK-QKEV-S-I-R-	H--SHTI------------M--A---
Paraliobacillus quinghaiensis	WP_117155635	--E-------TSLGLEAEK-QQEV-N-I---	-K-SQTI------------M--A---
Paraliobacillus ryukyuensis	WP_079708193	--E-------NSLGLESEK-QQEV-N-I---	QK-SQTI------------M--A---
Paucisalibacillus globulus	WP_026907449	---E--------SLGLEVSK-QEEV-K-I-T-	KQPMQTI---------V---Q--A---
Pontibacillus chungwhensis	WP_036787646	---E-------G-LGLAADK-QEEV-Q-I-T-	DK-SQTI------------M--A---
Pontibacillus yanchengensis	WP_036819900	---E-------G-LGLASDK-QEEV-Q-I-S-	DK-SQTI------------M--A---
Salimicrobium flavidum	WP_076560487	K--E-------T-LGLEAET-QTEV-N-I---	-K-SQTI------------M--A---
Salimicrobium jeotgali	WP_008592463	K--E-------T-LGLEAET-QTEV-N-I-A-	-QKSQTI------------M--A---
Saliterribacillus persicus	WP_114354223	--E-------Q-LGLGTEK-QEEV-G-I---	NKASQTI------------M--A---
Sediminibacillus albus	WP_093217371	---E-------SSLGLETEK-QEEV-Q-I-R-	QK-SQTI------------M--A---
Sediminibacillus halophilus	WP_026771008	---E-------SSLGLETEK-QEEV-Q-I-R-	QK-SQTI------------M--A---
Sediminibacillus massiliensis	WP_077623937	--E-------SSLGLETEK-QEEV-Q-I-R-	QK-SQTI------------M--A---
Sporosarcina globispora	WP_053433210	---E-------Y-LGLGSDK-QKEV-N-I-R-	QDASQTI------------M--A---
Thalassobacillus cyri	WP_093046723	---E-------T-LGLEAEK-QGEV-Q-I-R-	-K-SQTI------------M--A---
Thalassobacillus devorans	WP_028781686	---E-------T-LGLEAEK-QGEV-Q-I-R-	-K-SQTI------------M--A---
Thermolongibacillus altinsuens	WP_132949363	---E-------M-LGLG-EK-QKEV-S-I-R-	S---QTI------------M--A---
Virgibacillus alimentarius	WP_029266880	---D-------QSLGLEV-K-QE-V-D-I-M-	KQPMQTI---------V---Q--A---
Virgibacillus dakarensis	WP_088051802	---D-------QSLGLEVEK-QQEV-Q-I-V-	KQAMQTI---------V---Q--A---
Virgibacillus siamensis	WP_077324070	---D-------QSLGLEVSK-QEEV-K-I-V-	KQPMQTI---------V---Q--A---

Supplemental Figure 9

A partial sequence alignment of the ATP-dependent Clp protease ATP-binding subunit protein containing a one amino acid insertion (boxed) that is exclusively shared by all members among the *Planococcaceae* family and absent in all other bacteria. 2 *Bacillus* species are exceptions which share this CSI.

Group	Organism	Accession	583	(boxed region)	625
Family *Planococcaceae* (>100/>100)	Bacillus cecembensis	WP_057986323	NVNLEQKTPEKYFVDLTITEE	LLKEIEATEGYADLSEEEQIQAQRRAV	LAKHFTNVPVIDPFRKKFLGNV
	Bacillus ndiopicus	WP_042478610	D------------K-A-DA-	QMA-L-----------A-KVD---K--	-Q-Y-AS---VP----------
	Butyricicoccus sp. 1XD8-22	RKJ63675	AY-----------TH-R-DD-	----V--S---SA---Q-K-D-K-Q--	-K-Y-EQA-I-H--------SI
	Caryophanon latum	WP_066462904	DS---TV------AT--VDA-	--AQ---S----E--QA---E---Q--	---Y-A-T--VA----------
	Caryophanon tenue	WP_066542790	DS---TV--D---AT---D-A	--A-V--S----E--QA---E---Q--	---Y-A-T--VA----------
	Lysinibacillus acetophenoni	WP_097149276	AY-------D---TQ-R-DD-	----V--S---SA---Q-K-TI--QV-	-K---DDA---N--------SI
	Lysinibacillus boronitolerans	WP_016993312	DF----V--D---AK---D--	T---L-SDSA-NN-D-KGK-D---T--	-R-Y-AS---VN---------I
	Lysinibacillus chungkukjangi	WP_107932283	SE----E--QQ--AS---D-D	T-QQLKTSD--NE---G-KVE-E----	-V---AS-KT-S---------I
	Lysinibacillus composti	WP_124766159	AK-------DQ--IN---DD-	--A-V--SA--SE--DA-KVEV--Q--	-K---KSI-T-N---------I
	Lysinibacillus endophyticus	WP_121214178	ST---KE------AS---D--	T-Q-L-QLDE-KNAD-S-K-EL--Q--	-R---E--KP-S--------QI
	Lysinibacillus fluoroglycofeni	WP_107943495	D-----------AK-A-DA-	Q-A-L-----------A-K-D---K--	-Q-Y-AA---VP----------
	Lysinibacillus fusiformis	WP_004233605	DF----V------AK---D--	T---L-SDNA-NN-D-KGK-D---T--	-R-Y-AS---VN---------I
	Lysinibacillus halotolerans	WP_122971262	A-----------TT---D--	T---L------NE---N-KVE---Q--	-Q---AS-KT-S---------I
	Lysinibacillus jejuensis	WP_108305974	DY---EA--MT---N-NVD--	M-A-L-QDTS-QA-A-D-K-A---Q--	-K-Y-AE--AVA---------I
	Lysinibacillus macroides	WP_053997224	DF----A--D---AK---D--	T---L-TDSA-NS-D-KGK-A---S--	-R-Y-AS---VN---------I
	Lysinibacillus manganicus	WP_036183185	EY----R--D---TN-R-ED-	----V--S---SA---Q-K-TV--QT-	-K---DDA-L-N--------SI
	Lysinibacillus mangiferihumi	WP_107894988	DF----V--D----N---D--	T-A-L--DAA-NQ-D-KGKVD---S--	-R-Y-AS---VN---------I
	Lysinibacillus massiliensis	WP_036172414	AY-----------TH-R-DD-	----V--S---SA---Q-K-D-K-Q--	-K-Y-EQA-I-H--------SI
	Lysinibacillus meyeri	WP_107839062	DT-----------AK---DA-	--A-V------V----A-K-D---K--	-Q-Y-AS---VP----------
	Lysinibacillus odysseyi	WP_036151983	DT---S-------AK---D--	T-A-L--K-E-KA---G-K-E---Q--	-Q---ASI-AVP----------
	Lysinibacillus parviboronicapi	WP_107925081	EF----E--Q----N---D--	T---L--DAA-KE-D-KGKVD---T--	-R---VS---VN---------I
	Lysinibacillus saudimassiliens	CDZ99440	DF---EA--MN---N-Q-ED-	M-A-L-SDVA-QAMT----VK---Q--	-K---IDL-AVA---------I
	Lysinibacillus sinduriensis	WP_036200767	D---------Q--AS---D--	T-QHLQ-----NE---G-KVE-E----	-V---AS-KT-S---------I
	Lysinibacillus sp. 2017	WP_108714300	TF-------------N---D--	-R----------E---A--VE------	-T-----A--V----------I
	Lysinibacillus sp. B2A1	AVK86704	DF----E--D-F--N---D--	T---L--DAT-NA-D-KGKVN---T--	-R-Y-AS---VN---------I
	Lysinibacillus sp. BF-4	WP_036143362	DF---EA--MN---N-Q-ED-	M-A-L-SDAA-QAMT----VK---Q--	-K----DL-AVA---------I
	Lysinibacillus sp. BK089	WP_132362677	AF----E--D----N---D--	T---L--DAA-NG-D-KGKVD---T--	-R-Y-AS---VN---------I
	Lysinibacillus sp. FJAT-14222	WP_053596050	DF----E--D----N---D--	T---L--DAA-NG-D-KGKVE---T--	-R---AS---VN---------I
	Lysinibacillus sp. FJAT-14745	WP_053482820	DF----E--D----K---D--	T---L--DAA-NG-D-KGKVE---T--	-R---AS---VN---------I
	Lysinibacillus sp. Marseille-P	WP_106781848	AI-------D---GH-R----	----V--S---SA---Q-KVEV--KV-	-Q-Y-AEA-I-N--------SI
	Lysinibacillus sp. OL1	WP_131521815	DF----V------AK---D--	T---L-SDSA-NN-D-KGK-D---T--	-R-Y-AS---VN---------I
	Lysinibacillus sp. SYSU K30002	WP_126658162	ST-----------AN---G--	T-N-LQ-S-E-QA---DDK-A---K--	-QR--AS-KP-S---------I
	Lysinibacillus sp. YLB-03	WP_118876528	SE----R---R--AS---D--	T-EQLK-S---N---DS-K-E-E----	-V---AS-KP-S---------I
	Lysinibacillus sp. YR326	WP_134022304	AF---EE--D----N---D--	T---L--DAD-NG-D-KGK-D---T--	-R---AS---VN---------I
	Lysinibacillus sp. ZYM-1	WP_054610136	DF----V-------N---D--	T--VL-SDSA-NN-D-KGK-D---T--	-H---ESA--VN---------I
	Lysinibacillus sphaericus	WP_010860615	AF----E--A----N---D--	T---L--DAA-NE-D-KGKVE---T--	-R-Y-AS---VN---------I
	Lysinibacillus telephonicus	WP_126292329	A-----------TT---D--	T---V--S---NA--DA-KVE---Q--	-K---AS-AP-S---------I
	Lysinibacillus xylanilyticus	PJO44212	DF----E--S----N---D--	T-N-L--DAD-NG-D-KGKVD---T--	-R---AS---VN---------I
	Lysinibacillus xyleni	WP_097073462	TT---KE------AS---D--	T-QQL-QSDD-RNAE-NVK-EL--QE-	-K---E--KP-S--------QI
	Solibacillus isronensis	WP_079523496	A-------------N---D--	----------------S-KVE-----I	-------M--V----------
	Solibacillus kalamii	WP_087617863	S-------------N---D--	----------------L-K-E-----M	-------M--V----------
	Solibacillus silvestris	WP_014822409	S-------------N---D--	----------------S-K-E-----M	-------M--V----------
	Solibacillus sp. R5-41	WP_099425606	S------------------AD-	------------G-----K--------	---------------------
	Ureibacillus thermophilus	QBK24919	SY---VA--D------R-DD-	M---L--S-E-NQ---K-K-EY--K--	-K-YIEDQEL-T--------SI
	Ureibacillus thermosphaericus	WP_016838547	SY---VA--D----S---DD-	----L--S-E-NQ---Q-K-EY--K--	-K-YIEDQEL-T--------SI
Other Bacteria (0/>100)	Bacillus fordii	WP_018708720	KE----N--------T-ENV		-EFIKKQ-LVK--K--I---I
	Bacillus fortis	WP_120072816	RE----N--------T-ENV		-EFIKKQ-LVK--K--I---I
	Bacillus fumarioli	WP_066366320	KD---V--------SKGEDV		RAKIKSM-LVE--K--I---I
	Bacillus mesonae	WP_066390087	KG---E--------EKGEDV		KAKIKSM-LV---K--V---I
	Bacillus obstructivus	WP_071976255	KT---E---------TDTNV		KE-ISKQ-I-K--I------I
	Salisediminibacterium haloalka	WP_093073176	RE---EV------AQP-EDIK		AL-AERE-VP--K-G---DI
	Psychrobacillus insolitus	WP_111440090	DF--QTE--A----PT-TD		VR--IEAAELVE--K--I---I
	Psychrobacillus sp. OK032	WP_093274844	DF--QVE-------ST-TD		VK--IESTDLVQ--K--I---I
	Geomicrobium sp. JCM 19037	GAK04721	AI----E--KE---TSDTD		IK-TIAER--VA--K-G---D-
	Burkholderia multivorans	RAA71273	QT---D---D----SASELG-	GG	-KAY-EDQELVE--N------I
	Jeotgalicoccus halophilus	WP_092597981	KE---R---N---I-AEDLG-	DG	-RGKIQEM-LV---N-----Q-
	Jeotgalicoccus halotolerans	WP_115885256	RE---R---N---I-AKDLG-	EG	-VEKIQEM-LV---D--Y--Q-
	Macrococcus bohemicus	WP_111746638	LS---TV--D---I-A-ELT-	EG	-L-YLNEQ-LVK--N-----QI
	Macrococcus lamae	WP_133443663	LS---EE--DH--IKA-DLS-	EG	-L-YLE-T-LVK--N-----Q-
	Mycobacteroides abscessus subs	SIN55735	QT---N-------IAP-EIG-	EG	-KAY-DEQ-L-K--N-N-----
	Paenibacillus macerans	OMG43670	QA---RT--D-----P-QLG-	GG	-KEY-D-AEL-E--N------I
	Salinicoccus alkaliphilus	WP_072710427	RT---E---DE---KVGELG-	GG	-KEYYKDR-LVA--D--Y--QI
	Salinicoccus kekensis	WP_097041394	RS---E---D----RVGELG-	GG	-KEYYKDR-LA---D-----QI
	Staphylococcus agnetis	WP_037566318	AT---KN--D-----ASDLG-	GG	--EY-E-TELVP--N------I
	Staphylococcus aureus	AJP21749	QE---R---N---I-P-TLG-	GG	-KEY-E-EEL-E--N------I

Group	Species	Accession	Sequence		Sequence
**Other Bacteria (0/>100)**	Staphylococcus cohnii	WP_070039469	KY---NS--D----SA-ELG-		GG -KAY-DEQ-L-E--N-N----I
	Staphylococcus nepalensis	PTK52636	QT---D---D----SASELG-		GG -KAY-EDQELVE--N------I
	Staphylococcus pasteuri	WP_108000042	QA---G---D-----P-ELG-		GG -KEF-ETKELTE--N------I
	Staphylococcus rostri	WP_103358481	AT---KN--D-----A-ELG-		GG --EY-EKTELVP--N------I
	Staphylococcus simiae	WP_088177521	QE---R---DR--I-P-TLG-		GG -KAY-E-EEL-E--N------I

Supplemental Figure 10

A partial sequence alignment of the DNA-directed RNA polymerase subunit beta protein containing a 27 amino acid insertion (boxed) that is exclusively shared by all members among the *Planococcaceae* family and absent in all other bacteria.

		148		213
Bacillus cecembensis	WP_057982230	VAKALLNELGISIVAHVTEIVGIKADPA	LL	EGKSADEIRTIVEEDPCYCVDPEASAKMVEAIDDAK
Bacillus ndiopicus	WP_042477984	------K-------------A-----AS	--	---T-N---A-I---A------V-------V--AT-
Bacillus sp. FJAT-22090	WP_053589792	---Q--KQ----------K-G--E--LT	-A	T--TIE---EV--S--V--L--V----------Q--
Bacillus sp. OxB-1	WP_041074518	---QF-R----RT-------G--PTN-D	TY	AQ-G-E-L-E---S--V--A-----RR------V--
Bhargavaea beijingensis	WP_092095140	---SF-KA---EVT---A--G-V---MT	-R	N--TVN---E-ASA-DV--I-----E--KQ------
Bhargavaea cecembensis	WP_008296988	---SF-KA---EVT---A--G-V---MT	-R	N--TVN---E-ASA-DV--I-----E--KQ------
Bhargavaea ginsengi	WP_092048703	---SF-KA---EVT---A--G-V---MT	-R	N--TVN---E-ASA-DV--I--A--E--KQ------
Butyricicoccus sp. 1XD8-22	RKJ15252	------K-------S-----G-----TS	KV	---TI----S---K--V-----S--E---QL---T-
Caryophanon latum	WP_066465140	------R----HV-S-------------	SI	-----------I---A---A------------A--
Caryophanon tenue	WP_066548664	------R----HV-S------------Q	KI	A--------A-I---A---A------Q------A--
Chryseomicrobium excrementi	WP_100352626	---Q--EQ---QVIGY--T-G-VH--RS	-A	-KLTVE-LIERTSQ--V-TA--V--EQ--A---A--
Edaphobacillus lindanitolerans	WP_076756404	---SM-KA---EVT---A--G--Q--TT	-R	N--TVS---E-ASD-AV--I-----E--KQ------
Filibacter sp. TB-66	WP_124070664	---QF-S--D-HT-S-----G-VET--S	TY	A-LGVA-L-ACI-Q--V--A-S---KQ--Q----I-
Kurthia gibsonii	WP_087680106	---I--EQ---R-----V--G--TI-RQ	AV	----VE---A------------------AL--ET-
Kurthia huakuii	WP_029498437	---Q--A----NV-GY----G--E--K-	AT	A---VE---A-I---A---A-A-------DL--AT-
Kurthia massiliensis	WP_010286116	---K--A----QV-GY----G-VE-SKE	AT	A---VE-----I-S-----A-A--------K--AT-
Kurthia senegalensis	WP_010301090	---V--EQ---RV-G-----G-VV-NAQ	TT	---TVE-----I---A---A-AQ--E-------A--
Kurthia sibirica	WP_109306906	---V--Q----T-----V--G-VV---T	ST	A--TP--L-A-I-A-AV--A--I--K---TL--ET-
Kurthia sp. 11kri321	WP_068454410	---I--EQ-D-R-----V--G--TI-RQ	AV	----VE----------------------AL--ET-
Kurthia sp. Dielmo	WP_020189612	---Q--A----N--GY----G-VE--K-	AT	A---VE---A-I---A---A-A--------L--AT-
Kurthia zopfii	WP_109348639	-S-I--EQ---K-----V--G--AV-RS	AT	--MT-----A--------------------L--AT-
Lysinibacillus acetophenoni	WP_097148946	---L--K-------S-----G-V----T	KA	---------K------V--L----------L--ET-
Lysinibacillus boronitolerans	WP_016994410	--------------------------TS	-I	---TTE---V---A--------I----------E--
Lysinibacillus chungkukjangi	WP_107937175	------S-------S-----A-----TT	KI	---TVN---E---------------SQ--QM---T-
Lysinibacillus composti	WP_124765151	------------V-------A-----TS	AV	-------------Q-A-------------QM---T-
Lysinibacillus contaminans	WP_053584021	------K-------S-----------TS	VI	---T-----E-I-A--------V----------E--
Lysinibacillus endophyticus	WP_121214726	------------V-------A-----TS	KV	---------A---------------E---QM---T-
Lysinibacillus fluoroglycofeni	WP_107942405	------K-------------A----A-S	--	---T-----A---A-A---I--V-------T--AT-
Lysinibacillus fusiformis	WP_025114711	--------------------------TS	-V	---TV----A---A--------V----------E--
Lysinibacillus halotolerans	WP_122970907	--------------S-----A-----TS	KI	----V----E---Q-------G-------QM--ET-
Lysinibacillus jejuensis	WP_108306685	I--L--A--D-H-I---I--G-VR--TT	-A	A--TVA---E---N-----L-----K----M--AT-
Lysinibacillus macroides	WP_053994336	--------------------------TS	-V	----V----A---A-------------------E--
Lysinibacillus manganicus	WP_036183775	--------------S-----G-V----T	KS	----TE---K------V--L---------QL---T-
Lysinibacillus massiliensis	WP_036179916	------K-------S-----G-----TS	KV	---TI----S---K--V-----S--E---QL---T-
Lysinibacillus meyeri	WP_107841527	------R-------------A-V---IS	--	---T------V--A-A------V--E----I--AT-
Lysinibacillus odysseyi	WP_036154477	--------------SY----A-----VS	KV	A----------I------T--------------A--
Lysinibacillus parviboronicapi	WP_107925109	--------------S-----------TS	-V	----V----A---A--------V---Q------E--
Lysinibacillus saudimassiliens	CEA04810	I--L--A----NVI---I--G-V---TT	-A	T--TVA---E---N-----L-AT--KE--AM--AT-
Lysinibacillus sinduriensis	WP_036203641	------R-------------A-----TT	KV	----VND--K---Q-------A---SQ--QM---T-
Lysinibacillus sp. 2017	WP_108712218	--------------------------TQ	--	----VA-------H---------------Q------
Lysinibacillus sp. B2A1	AVK83619	--------------S-----I-----TS	-V	---------A---A-------------------E--
Lysinibacillus sp. BF-4	WP_036141837	I--L--A----NVI---I--G-V---TT	-A	T--TVA---E---N-----L-AT--KE--AM--AT-
Lysinibacillus sp. BK089	WP_132359244	--------------------------TS	VV	---------A---A-------------------E--
Lysinibacillus sp. FJAT-14222	WP_053593568	--------------------------TS	-V	----V----A---A---------------A---E--
Lysinibacillus sp. FJAT-14745	WP_053482556	--------------------I-----TS	-V	---------A---A-------------------E--
Lysinibacillus sp. Marseille-P	WP_106784689	------------V-S-----G-----TS	IT	---------E---Q--V-----L--T---QL---T-
Lysinibacillus sp. OL1	WP_131519666	--------------------------TS	-I	---TTE---A---A-A------I----------E--
Lysinibacillus sp. SYSU K30002	WP_126657716	------------V-------A-----TS	KV	---------A-----------A--------M---T-
Lysinibacillus sp. YLB-03	WP_118874327	----------V---S-----A-----TT	KV	---------H---Q-----------QR--QM---T-
Lysinibacillus sp. YR326	WP_134019587	--------------------------TS	-V	---------S---A-------------------E--
Lysinibacillus sp. ZYM-1	WP_054609839	--------------------------TS	-I	---T-----A---A-------------------E--
Lysinibacillus sphaericus	WP_010858812	--------------S-----------TS	-V	----V----A---A--------V--TQ------E--
Lysinibacillus tabacifolii	WP_108030407	--------------S-----------TS	VI	----VA-------A--------I----------E--
Lysinibacillus telephonicus	WP_126294187	-----------T--S-----Y-----TS	KI	----I----A---Q-------ES--E---Q---E--
Lysinibacillus xylanilyticus	WP_049666777	--------------------------TS	-V	-------------A-------------------E--
Lysinibacillus xyleni	WP_097074470	------------V-------A-----TS	SI	---------A-----------AQ--E---QM---T-
Paenisporosarcina antarctica	WP_134209973	---Q--S-------S-----G-V---VK	KS	-SL-VSQ--E---A--V----Q---KL--K---E--
Paenisporosarcina indica	WP_075617676	---Q--S-------S-----G------S	KA	--L-VSQ--Q-I-T--V----E---KQ--S---E--
Paenisporosarcina quisquiliaru	WP_090561770	---E--SK----------K-G--E--LS	-A	N--TVEA--E---A--V--L--K--VR------N--
Paenisporosarcina sp. HGH0030	WP_016427414	---Q--S----N--S-----G------S	AW	--LDVKQ--E-I-A--V--L--Q--EL--K---E--
Paenisporosarcina sp. K2R23-3	WP_119883130	---I--K----EVIG--K--G--Q-NAV	DV	SNMQP--VKKAIK-N-V--L-E---KE---L--ET-
Paenisporosarcina sp. OV554	WP_108587171	---Q--S-------S-----G-----TS	KS	ADLTVAQ--G-I----V----Q---KL--Q---E--
Paenisporosarcina sp. TG-14	WP_017381838	---Q--S-------S-----G-V---VK	KS	-SL-VSQ--E---A--V----Q---KL--K---E--
Paenisporosarcina sp. TG20	WP_019414793	---Q--A-------S-----G-V---VT	KS	NDL-VSQ--E-I-S--V--I-----KL--Q---T--
Planococcus antarcticus	WP_006829866	---Q--A----K--S-----G---VN-E	SY	L---V----E---N-AV--A--TVTK--TDL--QT-

Family *Planococcaceae* (>100/>100)

Family *Planococcaceae* (>100/>100)

Planococcus citreus	WP_121297579	---Q--S---VK--S-----G---V---	SY	I--------D---Q-AV--A-SSKTKE-TDL--AT-
Planococcus donghaensis	WP_008431014	---Q--A----K--S-----G---VN-E	SY	I--------E---N-AV--A--SVTKE-TDL--QT-
Planococcus faecalis	WP_071154486	---Q--A----K--S-----G---VN-E	SY	L---V----E---N-AV--A--TVTQE-MDL--KT-
Planococcus halocryophilus	WP_008497793	---Q--A----K--S-----G---VN-E	SY	M--------E---N-AV--A--SVTKE-TDL--QT-
Planococcus halotolerans	WP_112222693	---Q--AQ---EM-S-----G---VS-E	SY	T---VA---EA--K-AV--A--SVTQQ-TDL--ET-
Planococcus kocurii	WP_058385042	---Q--A----K--S-----G---VN-E	SY	L---V----E---N-AV--A--TVTQE-MDL--KT-
Planococcus maitriensis	WP_112231664	---Q--A---VK--S-----G---V---	SY	L--------D-----AV--A-SSKTKE-TDL--AT-
Planococcus maritimus	WP_068462652	---Q--A---VKM-S-----G---V--Q	SY	I--------D---Q-AV--A-ATKTKE-TDL--AT-
Planococcus massiliensis	WP_052651397	---Q--SQ---E--S-----G---TN-E	SY	I----A---E---N-AV--A--SVTKQ-TDL--ET-
Planococcus plakortidis	WP_068869299	---Q--A---VK--S-----G---V---	SY	L--------D---Q-AV--A-ASKTKE-TDL--AT-
Planococcus rifietoensis	WP_058380562	---Q--S---VK--S-----G---V---	SY	I--------D---Q-AV--A-SSKTKE-TDL--AT-
Planococcus salinarum	TAA72547	---Q--SQ---QM-S-----G---V--E	TY	T---VE---EA--K-AV--A--SITQQ-TDL--ET-
Planococcus salinus	WP_123164200	---Q--SQ---E--SQ----G--RVN-E	TY	Q---VK---E---Q-AV--A--SVTKQ-TDL--ET-
Planococcus sp. CAU13	WP_033542130	---Q--SQ--VEM-S-----G---V--E	SF	T--TVA---EL--K-AV--A--SVTQQ-TDL--VT-
Planococcus sp. PAMC 21323	WP_038704154	---Q--A----K--S-----G---V--E	SY	I---V-D--E---N-AV--A--TVTK--TDL--QT-
Planococcus sp. Y42	WP_077589525	---QV-A-------------G--Q--VQ	KA	H--T-A-L-K-I-N--V--A---------TAL--ET-
Planococcus versutus	WP_065524473	---Q--A----K--S-----G---VN-E	SY	L-----D--E---N-AV--A--LVTKQ-TDL--ET-
Planomicrobium flavidum	WP_088007544	---Q--A-----T-------G-------	AW	--RT-A---N-I-Q--V--A--A------TAL--E--
Planomicrobium glaciei	WP_036801904	---Q--SQ--VE--------G---VN-E	SY	L----A---E---N-AV--A--SVTKQ-TDL--ET-
Planomicrobium okeanokoites	WP_084245516	---Q--SQ---EL-S-----G---VN-E	SY	T----A---EL--K-AV--A--SVTQQ-TDL--ET-
Planomicrobium soli	WP_106531775	---Q--A----K--S-----G---VN-E	SY	L--------E---N-AV--A--T-TKR-TDL--ET-
Planomicrobium sp. MB-3u-38	WP_101802913	---Q--SQ---EL-S-----G---V--E	SY	T----A---EL--K-AV--A--SVTQQ-TDL--ET-
Planomicrobium sp. Y74	WP_121632182	---Q--AQ---EM-S-----G---VN-E	SY	T---VA---EA--K-AV--A--SVTQR-TDL--ET-
Psychrobacillus insolitus	WP_111438331	---Q--S----------K-G--E--LS	-A	TT-TVE---E---A--V--L-S----------Q--
Psychrobacillus psychrodurans	WP_093494653	---E--SK---------K-G--E--LS	-A	N--TVEA--E---A--V--L------R------N--
Psychrobacillus psychrotoleran	WP_093534749	---E--SK---T------K-G--E-NLD	-A	N--TIE-------D--V--L--Q---Q------N--
Psychrobacillus sp. FJAT-21963	WP_056829478	---Q--KQ---------K-G--E--LT	-A	T--TIE---EV--S--V--L--I---------Q--
Psychrobacillus sp. OK028	WP_093060847	---E--RK---------K-G--E-NLD	-T	N--TIE---D---A--V--------E------N--
Psychrobacillus sp. OK032	WP_093273121	---Q-------------K-G--E-NLD	-T	T--TIE---E---A--V--L-S---E------N--
Rummeliibacillus	WP_119415399	------S----H--S-----G-VI---N	KI	AQ--V----A-I-------A-KI--T-------E--
Rummeliibacillus pycnus	WP_102692515	------S-------S-----G-VIV-RQ	IT	---T-N---E-I-N-----A-----V-------T--
Rummeliibacillus stabekisii	WP_066786066	---V-------H--S-----G-VI-N-E	TT	MN-TVH---Q-I-N-------QD--I-------A--
Solibacillus	WP_008404909	--------------S-----------SS	--	---T-----A-I-N-----I---------T---E--
Solibacillus isronensis	WP_079525863	--------------S-----------SS	--	---T-----A-I-N-----I---------T---E--
Solibacillus kalamii	WP_087618200	--------------S-----------SS	--	---T-----S-I-N-----I---------T---E--
Solibacillus sp. R5-41	WP_099423050	-------------------I-----T-	-V	---------A-----------S-------------
Sporosarcina koreensis	WP_040286728	---QF-R----RTA---I--G--T---D	TA	ACL------KR-----V--A--A--PA---------
Sporosarcina newyorkensis	WP_009497138	---QF-RQ---ET-------G--ET--S	TY	ANLTIEDL-QT-----V--A-K------T-----I-
Sporosarcina pasteurii	WP_115364065	---KF-E-I--TT----HD-G-V-I-SS	TY	-NL-ME-L--VIGQ--V--A-----K---Q------
Sporosarcina psychrophila	WP_067210366	---QF-R----ET-------G-FTTN-D	TY	A--GTE-L-N-I-N--V--A-----KL--Q------
Sporosarcina sp. BI001-red	WP_116016631	---QF-R----HTI---V--G--V--S-	ST	AELTME---ER--A--V--A--DV-QT---------
Sporosarcina sp. D27	WP_025784882	---QF-R----NTI---V--G--V--TT	ST	AKL-ME---AQ--A--V--A---V-QA---------
Sporosarcina sp. EUR3 2.2.2	WP_024534857	---Q--S-------------G-----TT	KS	A-LTVAQ--G-I----V----Q---KL--Q---E--
Sporosarcina sp. HYO08	WP_067407105	---QF-R----HT-------G--QT--N	TY	ADVTIE-L-ETI-Q--V--A--A--KQ--Q---EI-
Sporosarcina sp. P13	WP_099689424	---QF-RQ---ET-------G--ET--S	TY	A-LAIE-L-A-----AV--A-K---V--T-----I-
Sporosarcina sp. P16b	WP_099672060	---QF-RQ---ET-------G--ET-S-	AY	ADL-IE-L-A-----AV--A-K---V--T-----I-
Sporosarcina sp. P17b	WP_099624575	---QF-RQ---ET-------G-VQTN--	AY	ADL-I--L-A-----AV--A-QD-----T----AI-
Sporosarcina sp. P18a	WP_099675036	---QF-RQ---ET-------G--ET-S-	AY	ADL-IE-L-A-----AV--A-K---V--T-----I-
Sporosarcina sp. P20a	WP_099676753	---QF-RQ---ET-------G--ET---	AY	ADL-IE-L-A-----AV--A--D--V--TT----I-
Sporosarcina sp. P29	WP_099661458	---QF-RQ---ET-------G--ET---	AY	ANL-IE-L-A-----AV-----D--V--T-----I-
Sporosarcina sp. P3	WP_099638171	---QF-RQ---ETI------G--ET---	AY	ANL-IE-L-A-----AV--A-KD--V--T-----I-
Sporosarcina sp. P33	WP_081244524	---QF-RQ---ET-------G--ET---	AY	AEL-VE-L-A-----AV--A-K---V--T-----I-
Sporosarcina sp. P34	WP_099694289	---QF-RQ---ET-------G--ET-S-	TY	ADL-IE-L-A-----AV--A-K---V--T-----I-
Sporosarcina sp. PTS2304	WP_114923485	---QF-RQ---ET-------G--ET---	TY	AEL-MEDL-A-----AV--A-K------TA----I-
Sporosarcina sp. ZBG7A	WP_039041776	---QF-R----NTI---V--G--V--S-	ST	AELTME---AQ--A--V--A-S-V-QA---------
Sporosarcina ureae	WP_083035589	---QF-RQ---ETI------G--ET---	AY	ANL-IE-L-A-----AV--A-KD--V--T-----I-
Ureibacillus thermophilus	QBK25852	------------V-GY----Y--Q---S	KI	D--------Q---Q----TF-----V-------E--
Ureibacillus thermosphaericus	WP_016836960	------K-------G-----Y-----TS	KV	A--------Q---N----S--------------E--
Viridibacillus	WP_038190673	------A-------S-----G--I-N-E	ST	A---VA---A-I---A---A-A---L-------E--
Viridibacillus arvi	WP_053417248	------A-------S-----G-VI-N-E	ST	A---VA---A-I---A---A-A---L-------E--
Viridibacillus sp. FSL H8-0123	WP_076065188	------A-------S-----G--I-N-E	ST	A---VA---A-I---A---A-A---L-------E--
Viridibacillus sp. OK051	WP_100796716	----F-A-------S-----G--I-N-E	ST	A---VA---A-I---A---A-S----E------E--
Bacillus mannanilyticus	WP_025026765	---Q--SYF--KVAS--RR-GS-E--LS	T-	QEL-IE--QQ-T--S-VR-I-ASSAKQ-M-E--L–

Other Bacteria
(1/>100)

Caldalkalibacillus thermarum	WP_007504659	---K--RYF-MEV-G--KQ-GPVE-SLD	Q-	DQL-PQ--QR-T-DS-VR-L-Q--AQ--MQV--K–
Bacillus oleivorans	WP_097157545	---K--K----D--G--K--A-----VP		--L-VSKLKE-T-DS-VR-Y-----R--M-------
Abditibacterium utsteinense	WP_105483756	--RR--E-C--F-GS-----A-V---LT		-ILR-K-LNERADASEVR-L-EM-AHR-IHE--A-R
Anoxybacillus	WP_035050550	---RI-S----R-AS--V--G-V--EHT		AYT-LE-LQRVT-QS-VR-F-S--EK--MA---E--
Anoxybacillus amylolyticus	WP_131872549	---RI-E-V--RVAG--I--G-VR-KKL		DYR-LE-LQAVT--S-VR-F----GQ--M----W--
Anoxybacillus flavithermus	WP_041638226	---RI-A----R-AS--V--G-V--EHT		TYT-LE-LQQVT-QS-VR-F-A--EK--MA---E--
Anoxybacillus geothermalis	WP_044744927	---RI-E-V--RVAG--I--G-VR-KKL		DYR-LE-LQNVT--S-VR-F----GQ--M----L--
Anoxybacillus tepidamans	WP_027408580	---RI-A----QVAG--L--G-VR-ERL		AYE-LE-L-SVT-QS-VR-F-E--AV--M----R--
Anoxybacillus vitaminiphilus	WP_111644317	---QI-AQ---KVAG--L--G-V--KNL		TFS-LE-LQQVT-QS-VR-F-K--ET--M----E--
Bacillaceae bacterium SAOS 7	WP_095478086	---K--KL---E-AS--V--G----K-T		DVETIEQ-QQQS-DS-VR-L----EKQ-MQ------
Bacillus cereus SJ1	EFI64546	---QI-K---VE-AG--L--G-V--KHI		SNL-IE--Q--T-NS-VR-L-K-VEQ--MD---H--
Bacillus kochii	WP_095372760	---K--SS---EV-G--V--G--VSNVK		SYS-ME-LK--T--S-VR-L-SV-AEE-MV---N--
Bacillus nealsonii	WP_101176783	-G-K--SL---KVAS--L--G-V--NVE		SYE-IEKLAE-S-QS-VR-L--I-EKE-MK------
Bacillus simplex	WP_061144285	---K--SL---EVAS--L--G-V--E-P		VYETIQQLQQVT--SSVR-F-KNVEQQ-KD---E--
Carnobacterium gallinarum	WP_034561900	I--K--H----EV-G--V--G--Q-NLK		NEYTT----EGS-KS-VR-L--KVEQQ-MDK--E--
Carnobacterium maltaromaticum	WP_010053641	I--K--H----EV-G--V--G-VQ-NLK		NKYTI----EGS-NS-VR-L---VEQE-MDK--E--
Enterococcus saccharolyticus	WP_016175098	---KI-S--D-EVAG---MLG----TIP		--ITVS-VKA-S-ASDVRV---TVESEIRQL--QT-
Enterococcus sulfureus	WP_016185480	---K--Q----EVAG---KLG--Q-NIP		--LTVSQV-ELS-ASDIRV----VEQDIRDL--QT-
Escherichia coli	PSX48685	---K--H----EVAG--L--G-TR-NLT		RDYAVA--QETS-AS-VR-L-GV-AEE-MQK-----
Fictibacillus arsenicus	WP_066290026	---K--K---VEVGG--L--G-V--EQT		TYETLN-L-EKT-AS-VR-L-ET-EKM-MK---H--
Fictibacillus enclensis	WP_061969981	---Q--K----QVGG--I--G-VV-EKM		DYDTLNDLQERT-AS-VR-L-E--GL--MK---E--
Geobacillus jurassicus	WP_066229118	---RI-E-V--RVAG--I--G-VR-EKL		DYR-LE-LQAVT--S-VR-F----GQ--M-T--W--
Geobacillus kaustophilus	WP_044731010	---RI-E-V--RVAG--I--G-VR-KKL		DYR-LE-LQAVT--S-VR-F-L--GQ--M----W--
Jeotgalibacillus alimentarius	KIL48931	L-QKI-KD-N-N-AC--R--G-VQ--VI		--LP-K---SKS--SEVR-I-A---E--KT---E--
Jeotgalibacillus campisalis	WP_041058012	--QK--E----NVSC--K--G-VR-NTD		DLH-VE---RRA-NSEVR-------E--K----L--
Mycobacteroides abscessus subs	SHS35423	---K--SL---KVAS--V--G---SEVH		AYD-MESLAE-T-NS-VR-L--V-EK--MQ------
Parageobacillus caldoxylosilyt	WP_042409785	---RI-E----RVAG--L--G-VR-KRL		DYQ-LE-LQEVT-AS-VR-F-E--AV--M----N--
Parageobacillus thermoglucosid	OUM87836	---RI-E----RVAG--I--G-VR-ERL		DYR-LE-LQQVT--S-VR-F-EK-AI--M----E--
Quasibacillus thermotolerans	WP_039237762	---K--AL---EVA---L--G-V---KV		SFE-LE-L-ERS-AS-VR-L-E--EK--MQ------
Streptococcus pneumoniae	CKH28823	---QI-K---VE-AG--L--G-V--KHI		SNL-IE--Q--T-NS-VR-L-K-VEQE-MD---H--
Thermolongibacillus altinsuens	WP_132947079	---RI-A----QVAS--V--G-V--EKL		DYA-LK-LQEVT-QS-VR-F--V-EK--I----M--
Vagococcus carniphilus	WP_126791366	IC-KM-K--S-DVAG--L--G-VRGEIP		QSMTVS---EKA-NSAVR----LIEEA-K-K--AT-
Vibrio vulnificus	WP_133347582	---K--SL---EVAS--L--G-V--E-P		KYETIQQLQQVT--SSVR-F-KNVEQQ-KD---E--

Supplemental Figure 11

A partial sequence alignment of the chorismate synthase protein containing a one amino acid insertion (boxed) that is exclusively shared by all members among the *Planococcaceae* family and absent in all other bacteria. *Caldalkalibacillus thermarum* is the only exception which shares this CSI.

Family *Planococcaceae* (>100/>100)

		53		101
Bacillus cecembensis	WP_057982892	RAMDELPQSSPGPVIMTSEPKFVPAQGTSVTIGES	E	MPFRIRLVDCVGY
Bacillus ndiopicus	WP_042477968	--L--------------A-------HAAPIS--DN	G	LS-----A-----
Bacillus sp. OxB-1	WP_041074506	--Q---------------------------SV--G	D	LK-Q---A-----
Bhargavaea beijingensis	WP_092095160	--Q--------------A-------R--GISV-DG	D	LT-Q---A-----
Bhargavaea ginsengi	WP_092048718	--Q--------------A-------R--GISV-DG	D	LT-Q---A-----
Lysinibacillus acetophenoni	SOC37747	--L--------------A----------QI-----	-	I-----FA-----
Lysinibacillus boronitolerans	WP_016993351	--Q--------------A--------A-RIVV--D	-	---Q---A-----
Lysinibacillus chungkukjangi	PYF02815	--L--------------A----------EI-----	-	I-----FA-----
Lysinibacillus composti	WP_124765016	-----------------A----------QI-----	-	I-----FA-----
Lysinibacillus contaminans	WP_053584036	--Q--------------A--------A-RIAV--D	-	-S-Q---A-----
Lysinibacillus endophyticus	WP_121214735	--L--------------A----------EI-----	-	I-----FA-----
Lysinibacillus fluoroglycofeni	WP_107942402	--L--------------A-------NAAPIA--DN	G	LS-----A-----
Lysinibacillus fusiformis	WP_004232609	--Q--------------A--------A-RIVV-DD	-	---Q---A-----
Lysinibacillus halotolerans	WP_122973066	--L--------------A----------EIN----	-	I-----FA-----
Lysinibacillus macroides	WP_053994312	--Q--------------A--------A-RIVV--D	-	---Q---A-----
Lysinibacillus manganicus	WP_036183802	--L--------------A----------QI-----	-	I-----FA-----
Lysinibacillus meyeri	WP_107838555	--L--------------A-------NAAPIS--DD	G	LS-----A-----
Lysinibacillus odysseyi	WP_036154456	--L--------------A--------SAQIG--DN	G	LS-----A-----
Lysinibacillus parviboronicapi	WP_107925101	--Q--------------A--------A-RIAV--D	-	-S-Q---A-----
Lysinibacillus sinduriensis	WP_036203618	--L--------------A----------EI-----	-	I-----FA-----
Lysinibacillus sp. 2017	WP_108714436	----------------------------TIA---N	S	I------------
Lysinibacillus sp. B2A1	AVK83575	--Q--------------A--------A-RIAV--D	-	-T-Q---A-----
Lysinibacillus sp. BK089	WP_132359264	--Q--------------A--------A-RIAV--D	-	-T-Q---A-----
Lysinibacillus sp. FJAT-14222	WP_053593581	--Q----------N---A--------A-RIAV-DD	-	-S-Q---A-----
Lysinibacillus sp. FJAT-14745	WP_053482583	--Q--------------A--------A-RIAV-DD	-	-T-Q---A-----
Lysinibacillus sp. Marseille-P	WP_106784671	--L--------------A----------QI-----	-	I-----FA-----
Lysinibacillus sp. SYSU K30002	WP_126657708	--L--------------A----------EI-----	-	I-----FA-----
Lysinibacillus sp. YLB-03	WP_118874336	--L--------------A----------EI-----	-	I-----FA-----
Lysinibacillus sp. YR326	WP_134019604	--Q--------------A--------A-RIAV--D	-	-T-Q---A-----
Lysinibacillus sp. ZYM-1	WP_054609829	--Q--------------A--------A-RIVV-DD	-	---Q---A-----
Lysinibacillus sphaericus	WP_010858800	--Q--------------A--------A-RIAV--D	-	-T-Q---A-----
Lysinibacillus telephonicus	WP_126294195	--L--------------A----------EI-----	-	I-----FA-----
Lysinibacillus xylanilyticus	WP_049666804	--Q--------------A--------A-RIAV-DD	-	-T-Q---A-----
Lysinibacillus xyleni	SOC20120	--L--------------A----------EI-----	-	I-----FA-----
Paenisporosarcina antarctica	WP_134209977	--Q--------------A-------H---IQV-DD	A	FR-N---A-----
Paenisporosarcina indica	WP_075617668	--Q---------A----A-------H---IQV--D	S	FR-N---A-----
Paenisporosarcina sp. HGH0030	WP_016427422	--Q---------A----A-------H---IQV-DD	A	FR-H---A-----
Paenisporosarcina sp. K2R23-3	WP_119883121	--I-------A--A---A----------R-HLA--	S	IS-QV-F------
Paenisporosarcina sp. OV554	WP_108587178	--Q---------A----A-------H---IQV-DD	A	FR-N---A-----
Paenisporosarcina sp. TG-14	WP_017381830	--Q--------------A-------H---IQV-DD	A	FR-N---A-----
Paenisporosarcina sp. TG20	WP_019413573	--Q---------A----A-------H---IQV-DD	A	FS-N---A-----
Solibacillus isronensis	WP_079528699	-------------T---A----------T-AF---	A	I---V--------
Solibacillus isronensis B3W22	EKB45990	-------------T---A----------T-AF---	A	I---V-M------
Solibacillus sp. R5-41	WP_099423042	----------------------------T------	-	-------------
Sporosarcina koreensis	WP_052461784	--Q--------------A----------A-SV-DG	-	LS-Q---A-----
Sporosarcina newyorkensis	WP_009497147	--Q--------------A----------N-S---G	-	LA-Q---A-----
Sporosarcina pasteurii	WP_115360663	--Q--------------A------------AV-DG	-	LQ-Q---A-----
Sporosarcina psychrophila	WP_067210387	--Q---------S-----------------SV-DG	-	LK-Q---A-----
Sporosarcina sp. BI001-red	WP_116016613	--Q--------------A----------A-S--DG	-	LS-Q---A-----
Sporosarcina sp. D27	WP_051508736	--Q--------------A----------A-S--DG	-	LS-Q---A-----
Sporosarcina sp. EUR3 2.2.2	WP_024534865	--Q---------A----A-------H---IQV-DD	A	FR-N---A-----
Sporosarcina sp. HY008	WP_067407120	--Q-------------------------Q-SV-DG	D	LN-Q---A-----
Sporosarcina sp. P13	WP_099689431	--Q---------S----A------------S--DG	-	LA-Q---A-----
Sporosarcina sp. P3	WP_099638168	--Q--------------A------------S--DG	-	LE-Q---A-----
Sporosarcina sp. P34	WP_099694284	--Q--------------A------------S--DG	-	LE-Q---A-----
Sporosarcina sp. P37	ARK25920	--Q--------------A------------S--DG	-	LE-Q---A-----
Sporosarcina sp. PTS2304	WP_114923479	--Q---------S----A------------S--DG	-	LA-Q---A-----
Sporosarcina ureae	WP_083035584	--Q--------------A------------S--DG	-	LE-Q---A-----
Psychrobacillus insolitus	WP_111438340	-TQ---------N----A----------NIYL--E	Q	LRLQ---------
Psychrobacillus psychrotoleran	WP_093534765	--Q---------N----A-------H--NIHL--D	Q	LRLQ---------
Psychrobacillus sp. OK028	WP_093060839	--Q---------N----A-------H--NIHL--D	Q	LRLQ---------
Psychrobacillus sp. OK032	WP_093273154	--Q---------N----A-------H--NIHL--D	Q	LRLQ---------
Rummeliibacillus pycnus	WP_102692523	--Q--------------T-------N-AQI---NQ	D	---K--FA-----
Rummeliibacillus sp. POC4	WP_119417090	--Q--------------T-------N-AQI-V-NQ	D	---K--FA-----
Rummeliibacillus sp. TYF005	WP_124218649	--Q--------------T-------N-AQI-V-NQ	D	---K--FA-----

Group	Species	Accession	Sequence	Insertion	Sequence
**Family *Planococcaceae* (>100/>100)**	Rummeliibacillus stabekisii	WP_066786036	--Q--------------T-------N-AQISV-NQ	D	---K--FA-----
	Ureibacillus thermophilus	QBK25843	--L--------------A-------H--EIC----	-	I-----FA-----
	Ureibacillus thermosphaericus	WP_026018953	--L--------------A-------H--EIC----	-	I-----FA-----
	Butyricicoccus sp. 1XD8-22	RKJ10015	--L--------------A----------QI-----	-	I-----FA-----
	Edaphobacillus lindanitolerans	WP_076756412	--Q--------------A-------R--GISV-DG	G	LT-Q---A-----
	Filibacter sp. TB-66	WP_124070673	--Q----------I---A------------S--DG	-	LS-Q---A-----
	Viridibacillus arenosi FSL R5-	ETT81155	--Q--------------A-------K--QIAV--G	P	LTMQ---A-----
	Viridibacillus arvi	WP_053418278	--Q--------------A-------K--QIAV--G	P	LTMQ---A-----
	Viridibacillus sp. OK051	PKA87488	--Q--------------A-------K--QIAV--G	P	LTMQ---A-----
**Other Bacteria (0/>100)**	Aeribacillus pallidus	WP_063388595	--Q------AA-KT---T------N-AVT-HVD-G		LDVN---------
	Amphibacillus marinus	WP_091493402	--Q------AA-KT---T------N-AVHLNVDDG		LGINV--------
	Anaerobacillus macyae	WP_048309231	--Q------AA-RT---T------NHAVQIHVEDG		LDVNV--------
	Anoxybacillus amylolyticus	WP_066325846	--Q------AA-KT---T------N-AVK-KVDDG		LEVN---------
	Anoxybacillus flavithermus	WP_003394522	--Q------AA-KT---T------N-AVK-KVDDG		LEVN---------
	Anoxybacillus suryakundensis	WP_055440653	--Q------AA-KT---T------N-AVK-KVDDG		LEVN---------
	Anoxybacillus tepidamans	WP_027408589	--Q------AA-KT---T------N-AVK-KVDDG		LEVN---------
	Anoxybacillus vitaminiphilus	WP_111644028	--Q------AA-KT---T------N-AVK-KVD-G		LEVN---------
	Bacillus cereus	WP_000416523	--Q------AA-RT---T------N-AV-IEVD-G		LEVN---------
	Bacillus rubiinfantis	WP_042355260	--Q------AA-KT---T------N-AAT-HVD-G		LDVN---------
	Bacillus safensis	WP_048239330	--Q------AA-KT---T------N-AA-IHVSDG		LDVN---------
	Bacillus simplex	WP_076365639	--Q------AA-KT---T------N-AA-IQVA-G		LDVN---------
	Bacillus smithii	WP_003355151	--Q------AA-KT---T------N-AVK-SVD-G		LEVN---------
	Bacillus subtilis	PTU28763	--Q------AA-KT---T------N-AM--HVSDG		LDVN---------
	Caldibacillus debilis	WP_020154553	--Q------AA-KT---T------N-AVT-QVN-G		LSVNV--------
	Clostridium botulinum	WP_035784344	--K------GS-KS-H-T------NEAVEI-L-DE		TK-KV-M------
	Coprobacillus cateniformis	WP_008788828	--T------GE-KM---V------N-AARIFVE-N		LGVN---------
	Fictibacillus phosphorivorans	WP_066241881	--Q------AA-RT---T------NNAVRINV-DG		LDVN-----S---
	Firmicutes bacterium HGW-Firmi	PKM50738	--K-------A-KT---T----I-QEAARISL--D		IE-D---I-----
	Geobacillus jurassicus	WP_066229102	--Q------AA-KT---T------N-AVT-KVD-G		LEVN---------
	Geobacillus subterraneus	WP_033844075	--Q------AA-KT---T------N-AVT-KVD-G		LEVN---------
	Gracilibacillus kekensis	WP_073202108	--Q------AA-KT---T----I-N-AV-L-VE-G		LDVNV--------
	Halobacteroides halobius	WP_015327244	--Q------GE-RR---T------N-A-QISLENN		VD-NV--------
	Halothermothrix orenii H 168	ACL69811	--N-------S-RM---T------EEAIEIKLEDN		LS--V--I-----
	Hungateiclostridium cellulolyt	WP_010252315	--N------AS-RT---T------NEAIQIVLD-N		VQ-KV--------
	Hydrogenispora ethanolica	WP_132015184	--Q------GN-RT---T------SEAVE--V-DN		IQ--V--------
	Jeotgalibacillus alimentarius	WP_041122710	--Q------AA-RT---T----I-N-AVKI-VS-G		LDVNV-M------
	Jeotgalibacillus campisalis	WP_041058023	--Q-------A-RT---T------N-AVRIQVVDG		LEVN---------
	Kineothrix alysoides	TCL55704	--Q-------G-RT-T-T----I-KEAARISL--D		INVNV--I-----
	Klebsiella pneumoniae	OON61582	--Q------AA-RT---T------N-AV-IEVD-G		LEVN---------
	Melghiribacillus thermohalophi	WP_132371015	--Q------AA-KT---T----I-N-AVK-QVE-G		LEVNV--------
	Mycobacteroides abscessus subs	SHS35846	-TQ------AA-KT---T------N-AA-IHVDDG		LDVN---------
	Oceanobacillus arenosus	WP_115772814	--Q------AA-KT---T----I-N-AV--NVD-G		LDVN---------
	Oceanobacillus bengalensis	WP_121134863	--Q------AA-KT---T----I-N-AV--NVE-G		LDVN---------
	Ornithinibacillus californiens	WP_047985314	--Q------AA-KT---T----I-N-AVN--VE-G		LDVN---------
	Ornithinibacillus scapharcae	WP_010095922	--Q------AA-KT---T----I-N-AVN--VE-G		LDVN---------
	Paenibacillaceae bacterium GAS	WP_090777272	--V------AS-KT---T------N-AVRIHVA-G		LEVNV--------
	Paenibacillus beijingensis	WP_045672606	--I------AA-KT---T------N-AVRIKVA-G		LEVNV--------
	Paucisalibacillus globulus	WP_026906562	--Q------AA-KT---T----I-N-AVN-KVD-G		LDVN---------
	Pseudoflavonifractor capillosu	WP_006572541	--R------GS-RT---A------EEAVEIAMEDG		AA-SV--I-----
	Ruminococcaceae bacterium CPB6	WP_086035679	--V------AA-RT---T----I-E-AVQ-H--DN		AV-SV--I-----
	Ruminococcus lactaris	WP_023923053	-TK------AS-TT---T------KEAV--KL--D		VEVK---------
	Salimicrobium halophilum	WP_093191225	--L------AA-KT---T----I-N-AVEI-VDDH		LDL-V-M------
	Streptococcus pneumoniae	CJA53762	--Q------AA-RT---T------N-AV-IEVD-G		LEVN---------
	Thermolongibacillus altinsuens	WP_132947070	--Q------AA-KT---T------N-AVK-KVD-G		LEVN---------

Supplemental Figure 12

A partial sequence alignment of the Stage IV sporulation protein A containing a one amino acid insertion (boxed) that is exclusively shared by all members among the *Planococcaceae* family and absent in all other bacteria.

Family *Planococcaceae* (>100/>100)

		221		272
Bacillus cecembensis	WP_057987802	GQAQLLTSIDGENVETFEIEINKQDGNTFTF		FVTDERLIRKTGGIIQGMSGS
Bacillaceae	WP_008179909	-K-EIY-A-E-RK----S---T-VENERLE-		I-S--K--E-----L------
Bacillus ndiopicus	WP_042475315	-E-----A-E--K--A-T-T-D-I--ANIV-		R---PK-L------L------
Bacillus sp. FJAT-22090	ALC88078	-K-IMR------K-QD-S---TTVEDYL-Q-		T-I-KT--E-----L------
Bacillus sp. OxB-1	WP_041075059	-E-VIY-TVK-TE--S-S-R-T-IEKEQ-H-		VI--PK-LEA----L------
Bhargavaea beijingensis	WP_092098977	-E-DIY-T-K-TEIDKYS-K-TAAADDV-R-		T---KE--E-----L------
Bhargavaea cecembensis	WP_008298266	-E-EIY-T-K-TEIDKYS-K-TAAADDV-R-		T---RE--E-----L------
Bhargavaea ginsengi	WP_092051117	-E-SIY-T-K-TEINKYS-N-TASADDV-R-		T---RA--E-----L------
Lysinibacillus acetophenoni	WP_097148197	-K-E-Y-A-E-ST--K-----I-TEDSFLQ-		I---KK-LD-----L------
Lysinibacillus boronitolerans	WP_016993891	-K-EIY-A-E-SK----S---T-VENERLE-		I-S--K--E-----L------
Lysinibacillus chungkukjangi	WP_107934860	-K-EIY-A-E-SE--A-D-D-V-IED-ILQ-		V---KV-LE-----L------
Lysinibacillus composti	WP_124761898	-K-E-F-A-E-SE--A----LT-IEE-FLQ-		V---KE-LE-----L------
Lysinibacillus contaminans	WP_053582933	-K-EIY-A-E-SK--S-T-N-T-VENERLE-		I-S--K--E-----L------
Lysinibacillus endophyticus	WP_121214842	-K---Y-A-E-SQ--A-D-D-I-IED-FLQ-		I---KK-LE-----L------
Lysinibacillus fusiformis	WP_025116091	-K-EIY-A-E-RK----S---T-VENERLE-		I-S--K--E-----L------
Lysinibacillus fusiformis ZC1	EFI67338	-K-EIY-A-E-SK----S---T-VENERLE-		I-S--K--E-----L------
Lysinibacillus halotolerans	WP_122971839	-K-E-Y-A-E-SE--A---D-T-IED-FLQ-		V---KE-LE-----L------
Lysinibacillus macroides	WP_053994453	-K-EIY-A-E-SK----S---T-L-NERLE-		I-S-DK--A-----L------
Lysinibacillus manganicus	WP_036186826	-K-E-Y-A-E-SK--K---D-I-TEDSFLQ-		V---KK-LD-----L------
Lysinibacillus manganicus DSM	KGR78384	-K-E-Y-A-E-SK--K---D-I-TEDSFLQ-		V---KK-LD-----L------
Lysinibacillus mangiferihumi	WP_107894318	-K-EIY-A-E-SK----S---T-VENERLE-		I-S--K--E-----L------
Lysinibacillus massiliensis	WP_036173378	-K-E-Y-A-E-SD--K-T-D-T-IEDSFLQ-		K---KA-LE-----L------
Lysinibacillus meyeri	WP_107841164	-E--M--A-E--K--A-T-T-D-I--SNIV-		R---PK-LS-----L------
Lysinibacillus odysseyi	WP_036158198	-P--MI---E-NTI-K-D-T-H-VTEE--Q-		I---K--LE------------
Lysinibacillus parviboronicapi	WP_107923883	-K-EIF-A-E-SK--K-S---T-IENGRLE-		I-S-QK--E-----L------
Lysinibacillus sinduriensis	WP_036202069	-K-EIY-A-E-SE--A-----V-I-D-FLQ-		V---KE-LE-----L------
Lysinibacillus sp. 2017	WP_108713122	-K-EM------GD--S-T-D-GET---Q---		H---K---E------------
Lysinibacillus sp. B2A1	AVK84553	-K-EIY-A-E-SK----S---I-IENERLE-		IIS--K--E-----L------
Lysinibacillus sp. BK089	WP_132356657	-K-EIY-A-E-SK----S---T-VENERLE-		I---KK--E-----L------
Lysinibacillus sp. FJAT-14222	WP_053597076	-K-EIY-A-E-SK----S---T-VENERLE-		I-S-KK--E-----L------
Lysinibacillus sp. FJAT-14745	WP_053483723	-N-EIY-A-E-SK----S---T-VENERLE-		I---KK--E-----L------
Lysinibacillus sp. Marseille-P	WP_106779797	---E-Y-A-E-SD--K-T-D-V-IEDSFLQ-		V----K-LE-----L------
Lysinibacillus sp. PB300	WP_115673380	-K-EIY-A-E-SK----S---T-VENERLE-		I-S--K--E-----L------
Lysinibacillus sp. SYSU K30002	WP_126657036	-S-E-Y-A-E-SE--A-S-D-I-IED-FLQ-		I---KE-LD-----L------
Lysinibacillus sp. YLB-03	WP_118875494	-K-EIY-A-E-SE--A-DVD-I-IED-VLQ-		V---KE-LE-----L------
Lysinibacillus sp. YR326	WP_134019879	-K-EIY-A-E-SK----S---T-VENERLE-		I---KK--E-----L------
Lysinibacillus sp. ZYM-1	WP_054612949	-K-EIY-A-E-KK----S---T-VENERLE-		I-S--K--E-----L------
Lysinibacillus sphaericus	AOV09187	-K-EIF-TVK-TE--S-S---TNVEQDQ-H-		KL--SK-LET----L------
Lysinibacillus telephonicus	WP_126295078	---E-Y-A-E-SK--A-A-D-V-IED-FLQ-		I---KE-LQ-----L------
Lysinibacillus xylanilyticus	WP_049664215	-K-EIY-A-E-SK----S---T-VENERLE-		I---KK--E-----L------
Lysinibacillus xyleni	WP_097075315	-K-E-Y-A-E-SE--A-D-D-I-IED-FLQ-		I---KN-LE-----L------
Paenisporosarcina antarctica	WP_134209610	---E-F-----ND-RAYQVN-I-IEET--Q-		I---TE--N-----L------
Paenisporosarcina indica	WP_075618044	-K-E-Y-A-E-ND-RP-Q-D-I-VEET--E-		I----E--E-----L------
Paenisporosarcina quisquiliaru	WP_090563667	-K-VMR------K-QD-A---SSVE-HI-Q-		T---KK-LQ-----L------
Paenisporosarcina sp. HGH0030	WP_016426980	-Y-E---A---ND-NSYD---I-VEET--Q-		I---KE--K-----L------
Paenisporosarcina sp. K2R23-3	WP_119883275	-N-IIR-T-A-KE-QE-T-N-TDVHPPS-S-		E-D-QQ-LK-----L------
Paenisporosarcina sp. OV554	WP_108586808	-K-EIY-A-E-ND-QPYQ---I-V-KK--Q-		I---KV--E-----L------
Paenisporosarcina sp. TG-14	WP_017380057	---E-F-----ND-RAYQVN-I-IEET--Q-		I---TE--N-----L------
Paenisporosarcina sp. TG20	WP_019414100	-K-H-Y-A-E-N--KSY--N-L-VEDT--Q-		V---QA--D-----L------
Solibacillus isronensis	WP_079527584	-K--I--A----E-NL-D---D--TD-----		N-V-----K-----V------
Solibacillus silvestris	WP_014823485	-K--I--A----E-NL-D---D--TD-----		N-V-----K-----V------
Solibacillus sp. R5-41	WP_099424346	-K------------S---------S-----		--------Q------------
Sporosarcina koreensis	WP_040286577	-P-EI--T-E-TK--S-K-NVTDSSADA-K-		TL-----LK-----L------
Sporosarcina newyorkensis	WP_009496780	-P-EIY-TVQ-ST--K-T-Q-T-IEEEQ-QI		LM--QK-LK-----L------
Sporosarcina pasteurii	WP_115361096	-K-EIF-TVK-TE--S-S---TNVEQDQ-H-		KL--SK--ET----L------
Sporosarcina psychrophila	WP_067209875	-A-EIY-T-K-TK--S-S-R-IEVEQDQ-H-		VL--PE-LKA----L------
Sporosarcina sp. BI001-red	WP_116016955	-K-EI--T-Q-TK--S-----S-MSNDS-Q-		KL--Q--LE-----L------
Sporosarcina sp. D27	WP_025784713	-K-EI--T-Q-TE--S-----S-LSNDS-Q-		KL--K--LE-----L------
Sporosarcina sp. EUR3 2.2.2	WP_024534552	---EIY-A-E-ND-KPYQ---I-V-KK--Q-		I---KV--E-----L------
Sporosarcina sp. HY008	WP_067403685	-K-EIF-TVK-T---S-A---TSVETEQ-Q-		VL--PK-LKT----L------
Sporosarcina sp. P13	WP_099689387	-P-EIY-TVQ-S---K-S-K-T-VEEEQ-QI		LL--PK-LK-----L------
Sporosarcina sp. P16b	WP_099672196	-P-EIY-TVE-ST--K-S-Q-T-IEEEQ-HI		LL--QK-LS-----L------
Sporosarcina sp. P18a	WP_099674851	-P-EIY-TVE-ST--K-S-Q-T-IEEEQ-HI		LL--QK-LS-----L------
Sporosarcina sp. P19	WP_099692277	-P-EIY-TVE-ST--K-T-Q-T-IEEEQ-HI		LL--QK-LS-----L------
Sporosarcina sp. P20a	WP_099676972	-P-EIY-TVE-ST--K-T-Q-T-IEEEQ-HI		LL--QK-LS-----L------

Group	Organism	Accession	Alignment	Insertion	Alignment
**Family *Planococcaceae* (>100/>100)**	Sporosarcina sp. P3	WP_099637710	-P-EIY-TVE-ST--K-A-Q-T-IEEEQ-QI		LL--QK-LS-----L------
	Sporosarcina sp. P33	WP_081241905	-P-EIY-TVE-ST--K-T-Q-T-IEEEQ-QI		HL--QK-LK-----L------
	Sporosarcina sp. P34	WP_099694545	-P-EIY-TVE-ST--K-S-Q-T-IEEEQ-HI		LL--QK-LS-----L------
	Sporosarcina sp. PTS2304	WP_114923687	-P-EIF-TVQ-SS--K-S-R-T-IEEEQ-HI		LL--PI-LK-----L------
	Sporosarcina sp. ZBG7A	WP_052245963	-K-EI--T-Q-TE--S-D---S-LSNDS-Q-		KL--Q--LE-----L------
	Sporosarcina ureae	WP_029054570	-P-EIY-TVE-ST--K-T-Q-T-IEEEQ-HI		LL--QK-LS-----L------
	Psychrobacillus insolitus	WP_111437953	-K-IMR------K-QE-S-NVT-IE-HL-Q-		T-V--S--K-----L------
	Psychrobacillus psychrodurans	WP_093496656	-K-VMR------K-QD-A---SSVE-HI-Q-		T---KK-LQ-----L------
	Psychrobacillus psychrotoleran	WP_093538437	-K-VMR-----KK-QD-A---SSVE-HI-Q-		T---KK--E-----L------
	Psychrobacillus sp. FJAT-21963	KQL34466	-K-IMR------K-QD-S---TTVEDYL-Q-		T-I-KT--E-----L------
	Psychrobacillus sp. OK028	WP_093060699	-K-IMR------K-QD-T---SSVEDHI-Q-		T---KK--E-----L------
	Psychrobacillus sp. OK032	WP_093265742	-E-IMR------K-QD-T-N-SSV--HL-Q-		T---KS--Q-----L------
	Rummeliibacillus	WP_119416301	-P-K---T-S-KK--S-D-K-T-VNDKS-QI		EI--PV-KE-----L------
	Rummeliibacillus pycnus	WP_102691400	-P-K---T-S-KK--S-D-N-T-VNEKN-QI		EI--PV-KE-----L------
	Rummeliibacillus stabekisii	WP_066786687	-P-KI--T-Q-KK----D-T-T-VNNKS-QI		E---PV-KE-----L------
	Ureibacillus thermophilus	QBK26100	-K-E---A-E-SK--S-Q---V-IED-YLQ-		EI--QN-LS-----L------
	Ureibacillus thermosphaericus	WP_016837758	-K-E---A-E-SK--S-T---V-IED-YLQ-		EI--QN-LS-----L------
	Viridibacillus	WP_038184923	-K-----T-E-KK--A-DVK-T-V-DK-MQ-		E---AQ-KK-----L------
	Viridibacillus arvi	WP_053416232	-K-----T-E-KK--A-DVK-T-V-DK-LQ-		E---AQ-KQ-----L------
	Viridibacillus sp. OK051	WP_100797905	-K-----T-E-KK--A-DVK-T-I-DKSLQ-		E---AQ-KK-----L------
	Butyricicoccus sp. 1XD8-22	RKJ22435	-K-E-Y-A-E-SD--K-T-D-T-IEDSFLQ-		K---KA-LE-----L------
	Edaphobacillus lindanitolerans	WP_084186598	-D-EI--TVE-VEIGKYS-R-TSTA--A-R-		T---TK--K-----L------
	Filibacter sp. TB-66	WP_124071429	-K-EI--T-K-TK----A---T-VEKDQ-H-		VL--TA-LK-----L------
**Other Bacteria (0/>100)**	Anaerotignum neopropionicum	WP_066088093	-K-EI-SN-E-QE-KKY----ESLGKGNGKD	MMI	H---K--LE-----V------
	Aeribacillus pallidus	WP_044900270	-P-KI--VV--QK--E-A---VNVVKQH-PA	TKGMII	KI--K--LE-----V------
	Alteribacillus persepolensis	WP_091272528	-P--I--VVE--K--K-D---VQSIPQMHAA	SKGMVI	K----K-LE------------
	Ammoniphilus oxalaticus	WP_120188260	-P-HIY-VV--QK--KY----VNVIQQR-PA	TKGMII	K---PK-LE-----V------
	Anaerobacillus macyae	WP_048310757	-P-KI--VVK-SE-KAYDV-VVSSIPQK-PA	TKGMVI	K---PE-LKE----V------
	Anaerosalibacter massiliensis	WP_050069818	-K-YI--TLN-DKI-K-----V-AQNQLVPE	QKSMVL	K---KD-LQR----V------
	Aneurinibacillus soli	WP_096464776	-P-EIY-VV--QK--K-K---VNVIPQKYPS	TKGLIL	K---PV-LK-----V------
	Aneurinibacillus terranovensis	WP_027417558	-P-EIY-VV--QK-D--K-Q-VNVIQQK-PA	TKGLIL	K---P--LE-----V------
	Bacilliculturomica massiliensi	WP_130863733	-P-YI--TL--NT--KYS-S-E-INHQ-KPD	TKSMVI	R---K--LE-S---V------
	Bacillus acanthi	WP_108670852	-P--I--VV--SQ--K-D---VSTIPQK-PA	IKGMVI	K---PK-LK-----V------
	Bacillus acidicola	WP_066262305	-P-KI--VVN-DK--AYD-R-VSTVPQK-PA	TKGMVI	K---PK-LK-----V------
	Bacillus pseudomycoides	PHE45022	-P-KI--V--QDK--A-D--VVSTVPQK-PA	TKGMVI	K---K--LE-----V------
	Bacillus simplex	WP_034308274	-P-KI--VVN-SE--E-DV--VSSIPQK-PA	IKGMVL	K---KE-LQ-----V------
	Baia soyae	TCP69737	-P-KIY-VVE-QK--E---Q-THAVKQK-AA	TKGLII	K---P--LA-----V------
	Brevibacillus brevis	WP_012686042	-P-KI--VVE-QK--E-D---ANVVKQH-PA	TKGMII	K---K--LE-----V------
	Brevibacillus gelatini	WP_122904120	-P-KI--VVE-QK--E-D---ANVIKQH-PA	TKGMII	K---K--LE-----V------
	Brevibacillus invocatus	WP_122908423	-P-KI--VVN-QK--E-D---ANVVKQH-PA	TKGMII	K---K--LE-----V------
	Caldibacillus debilis	KYD20478	-P-EI--VVENNK--R-QVK-ISTVPQK-PA	TKGLVI	K---P--LE-----V------
	Chlamydia abortus	SHE11134	-P-HIY-VVN-QK--K-D---MHAAKQE-PA	TKGLVI	KI--Q--LD-----V------
	Clostridium acidisoli	WP_084113550	-D-KI--T-N--EPKYYN-K-E-LFSQDAPG	PKSMVI	K---PE-LK-----V------
	Clostridium botulinum	WP_039284548	-P-KI--T---TEPKLYD-K-E-LLPQESPG	PKSMVI	K---SE-LE-----V------
	Desulfitobacterium chlororespi	WP_072773397	-P-KIY-VLE--RI-EYDVQ-ERIMH-RTDS	KNMVI	K---PE--E------------
	Desulfotomaculum aeronauticum	WP_072910739	-P-EIY-VLQ-NKI-KYS-TVEQINLHGRAE	GKGMII	QI--QN-LK------------
	Desulfotomaculum ferrireducens	WP_077713473	-P-YIY-VLQ-NKI-KYD--VEQINLYGRAE	GKGMII	RI--KE-LA------------
	Desulfuribacillus stibiiarsena	OEH85946	-P-KIY-VVN-DKI-QYD--VVNVIPQR-SS	TKGMVI	R------LNQ-----------
	Effusibacillus pohliae	WP_018132480	-P-KI--VV--QK--EYS---VNVLRQKYPA	TKSMVI	RI--P--LE------------
	Emergencia timonensis	WP_082907621	-D-YI--T---TT--K-K---T-IHHQSSAE	SKGLEF	Q---KG-LASC----------
	Firmicutes bacterium CAG:313	CDD21595	-P-KIY-VV--QTINEYN---IDIKRQSRKG	VKGIKF	K---SN--A-----V------
	Fontibacillus phaseoli	WP_114495077	---EI--VVE-QK--RYR---VHVSKQSAPE	TKGIVL	RI--P---E-----V------
	Gorillibacterium massiliense	WP_040951330	-P--IY-VVN-QK--K-D-R-T-VARQDYPA	TKGLVI	KI--P--LEQ----V------
	Gracilibacillus massiliensis	WP_058307497	-P-HI--V-N--EI-A-DVK-VNSINQSAPA	TKGIIL	EI--K--LEA----V------
	Haloplasma contractile	WP_008826124	-S-KIY-V-N-NQI---DV--ISAEKQSKKD	IKGIKL	RI-----LS------------
	Haloplasma contractile SSD-17B	ERJ13770	-S-KIY-V-N-NQI---DV--ISAEKQSKKD	IKGIKL	RI-----LS------------
	Holdemania filiformis	WP_006060744	-P-EIY-V-Q-TTPKRYT-Q-TEVRKQNGQA	VKGIQF	Q---PA-LNQ-----------
	Holdemania massiliensis	WP_081587793	-L-EIY-VVQ-TIPQRYS-Q-TEVRKQNGQA	VKGIRF	Q---PA-LNQ-----------
	Hydrogenispora ethanolica	WP_132017186	-A-EIY-V-Q-TK--K-----I-VYPQDRPR	DKGLII	K---K--LNL-----------
	Jeotgalibacillus alimentarius	KIL49037	-G-EI--V-S-HEI-S-----LETNTELP-G	GKGLKI	RLS-PE-LE------------
	Natronincola peptidivorans	SES70613	-P-HI--TV-TNRI-KY----TRINPQARVD	SKSMII	K---KE-LE-----V------
	Novibacillus thermophilus	WP_077720462	-P-HIY-VVE-QR--KYD---VNVIEQKYAA	TKGMII	K------LN-----V------
	Numidum massiliense	WP_054949530	-P-HIY-VVE-RK--KYN---VNVIKQPYAA	TKGLII	K------LQ-----V------
	Oceanobacillus halophilus	WP_121203087	-P-KI--VVE--A-QEYDV--ISSVPQK-PA	TKGMVI	QI---E-LD-----V------
	Oceanobacillus senegalensis	WP_085991949	-P-KI--VVE--K--EY-V--ISSVPQKYPA	TKGMVV	QI---E-LD-----V------

Other Bacteria (0/>100)

Paenibacillus algorifonticola	SFE92923	-S--IY-VVN-QK--K-D---AHVMKQNSPA TKGMVI KI--K--LD-----V------
Paenibacillus amylolyticus	WP_123062247	-P-EI--VV--QQ--R-S-D-VHVADQSEPA TKGLVL RI--PK--D-----V------
Peptococcaceae bacterium BICA1	KJS65622	-P-DIY-V-E-TEIKRCSV--QDVFYPWNSR GKGMVI K---RE--AA-----------
Risungbinella massiliensis	WP_044640372	-P-KI--VV--QK--E-DVQ-THVTRQN-PA TKGIII K---K--LE-----V------
Ruminiclostridium cellobioparu	WP_004623863	-P-TI-SN-E-NEIQE-S-N-E-VARQS-SG PKGMVI K---KK-LNS----V------
Ruminiclostridium hungatei	WP_080065034	-P-TI-SN-E-SEIQE-S-Y-E-VARQS-SG PKGMVI R---K--LNS----V------
Ruminococcus flavefaciens	SFW28679	-E-EIY-TVS-NTP-RYSA--VSV-YSSG-S SKNMVI RI--R--LDAA---V------
Salipaludibacillus agaradhaere	WP_078576992	-P-EI--VVE-DE-KR-N-D-ISSTPQESPA TKGMVI K---KE-LD-----V------
Salipaludibacillus aurantiacus	WP_093047279	-P-EIY-VV---E-QR-D---ISSTPQQSPA TKGLVI K----E-LD-----V------
Sediminibacillus albus	WP_093210235	-P-KI--VVK--K--E-DV--VSSVPQK-PA TKGMII KI--KE-LNE----V------
Sediminibacillus massiliensis	WP_077621623	-P--I--VVK--K--A-DV--VSSVPQK-PA TKGMIV K---PE-LKE----V------
Seinonella peptonophila	WP_084731073	-S-KI--VVE-QR--EYK-Q-MHVMKQK-PA TKGMII K---P---------V------
Selenomonadales bacterium	PWL99768	-K-TI--TV--QGIQEY--N-I-ANKQDVRA SKSMII E---Q--LE-----V------
Sporanaerobacter acetigenes	WP_072744436	-K-YI--T-NDNK-QKY----V-AQNQP-PE QKSMII RI--KK-LQ-----V------
Tepidibacillus decaturensis	WP_068723638	-P-EI--V-E-QK--K-K---VNVVPQKYPA TKGMII K---PK-LE-----V------
Tepidibacillus fermentans	WP_132766911	-P-EI--V-EDQK--R-K---T-VVQQKYPA TKGMVI K---KK-LD------------
Thermicanus aegyptius	WP_005584354	-P-EI--VV--DK-GKYS---VNVIRQRYPS TKSMII K---P--LE-----V------
Thermoactinomyces vulgaris	WP_084712867	-P-KI--VVE-QK--EYN---VHAIKQK-PA TKGLII K---P--LK-----V------
Thermoanaerobacteraceae bacter	WP_120765620	-K-SI--V-N--KI-KYD---Q-IIRQAYPN GKGMII KI--PK--E------------
Virgibacillus necropolis	WP_089532146	-P-KI--VVE--KI-E-DV-VVSSVPQK-PA TKGMII QI--PE-LK-----V------
Virgibacillus phasianinus	WP_089062506	-P-KI--V----K--E-D---VSSVPQK-PA TKGMII QI--PE-LK-----V------

Supplemental Figure 13

A partial sequence alignment of the peptidase protein containing a six amino acid deletion (boxed) that is exclusively shared by all members among the *Planococcaceae* family and absent in all other bacteria.

Family *Planococcaceae* (>100/>100)

		153		196
Bacillus cecembensis	WP_057986232	FFMIVVTSLEWIIALMG	RQD	NIDVYVALLLFPLVAVNAFQILML
Bacillus ndiopicus	WP_042478695	-----I-----------	---	--NT---------L----Y-L---
Bacillus sp. B14905	EAZ86811	-----I-T---T-----	-DE	--NE---------L----Y-L-V-
Bacillus sp. OxB-1	WP_041071899	-----I-T---LP---V	-SG	---TW-TI-----L----Y---A-
Bhargavaea beijingensis	WP_125903940	-----I-T---LP---V	-SG	---QW-TI-----L---MY-L---
Bhargavaea cecembensis	WP_008297832	-----I-T---LP---V	-SG	---QW-TI-----L---MY-L-V-
Bhargavaea ginsengi	WP_092052728	-----I-T---LP---V	-SG	---QW-TI-----L---MY-L---
Butyricicoccus sp. 1XD8-22	RKJ57611	-----I-----------	--E	---T--------------Y-L-A-
Caryophanon latum	WP_066463004	-----------L-----	-AS	TS-S--------------Y-L---
Caryophanon tenue	WP_066542829	-------T---T-----	-DV	---S--------------Y-L-V-
Kurthia sibirica	WP_109305644	------S----L---WV	KTS	-G-MF----I--ILS--MY-L-I-
Lysinibacillus	WP_079562349	-------T---T-----	-DE	--NE---------L----Y-L-V-
Lysinibacillus acetophenoni	WP_097149212	-----------------	--G	---T--------------Y-L-A-
Lysinibacillus boronitolerans	WP_016994498	-----I-T---T-----	-DE	--NE---------L----Y-L-V-
Lysinibacillus chungkukjangi	WP_107932384	-----I-----------	--G	---T--------------Y-L-L-
Lysinibacillus composti	WP_124766210	-----I-----------	--G	---T---------L----Y-L-A-
Lysinibacillus endophyticus	WP_121216036	-----I-----------	--E	---T---------I----Y-L-A-
Lysinibacillus fluoroglycofeni	WP_107943478	-----I-----------	---	--NT--------------Y-L---
Lysinibacillus fusiformis	WP_069483220	-----I-T---T-----	-DE	--NE---------L----Y-L-V-
Lysinibacillus halotolerans	WP_122972818	-----------------	--G	---T--G-----------Y-L-V-
Lysinibacillus jejuensis	WP_108306007	-----I-T---T-----	-DA	K--E---------L------L---
Lysinibacillus macroides	WP_053997263	-----I-T---T-----	-DE	--NE---------L----Y-L-V-
Lysinibacillus manganicus	WP_036183025	-----------------	--G	---T----------V---Y-L-A-
Lysinibacillus massiliensis	WP_036172534	-----I-----------	--E	---T--------------Y-L-A-
Lysinibacillus meyeri	WP_107839019	---V-I-----------	---	--NT--------------Y-L---
Lysinibacillus odysseyi	WP_036150160	-----I-----------	---	--N----------L-T--Y-L---
Lysinibacillus parviboronicapi	WP_107950596	-----I-T---T-----	-DE	--NE---------L----Y-L-V-
Lysinibacillus saudimassiliens	CDZ99528	-----I-T---T-----	-DA	K--E---------L------L---
Lysinibacillus sinduriensis	WP_036200682	-----I-----------	--G	---T---------I----Y-L-L-
Lysinibacillus sp. 2017	WP_108714250	-----I-----------	--A	---I--------------Y-----
Lysinibacillus sp. B2A1	AVK86651	-----I-T---T-----	-DE	--NE---------L----Y-L-V-
Lysinibacillus sp. BF-4	WP_036143480	-----I-T---T-----	-DA	K--E---------L------L---
Lysinibacillus sp. BK089	WP_132362723	-----I-T---T-----	-DE	--NE---------L----Y-L-V-
Lysinibacillus sp. FJAT-14222	WP_053596079	-------T---T-----	-DE	--NE---------L----Y-L-V-
Lysinibacillus sp. FJAT-14745	WP_053482791	-------T---T-----	-DE	--NE---------L------L-V-
Lysinibacillus sp. LK3	WP_048395583	-----I-T---T-----	-DE	--NE---------L----Y-L-V-
Lysinibacillus sp. Marseille-P	WP_106781770	-----------------	--G	---T--------------Y-L-A-
Lysinibacillus sp. OL1	WP_131521826	-----I-T---T-----	-DE	--NE---------L----Y-L-V-
Lysinibacillus sp. SYSU K30002	WP_126658212	-----------------	--G	---T---------I----Y-L-A-
Lysinibacillus sp. YLB-03	WP_118876578	-----I-----------	--G	---T---------I----Y-L---
Lysinibacillus sp. YR326	WP_134022344	-------T---T-----	-DE	--NE---------L----Y-L-V-
Lysinibacillus sp. ZYM-1	WP_054610112	-----I-T---T-----	-DE	--NE---------L----Y-L-V-
Lysinibacillus sphaericus	WP_075526667	-----I-T---LP---V	SSG	---TW-TI-----L----Y---A-
Lysinibacillus telephonicus	WP_126296384	-----I-----------	--E	---T--------------Y-L-A-
Lysinibacillus xylanilyticus	WP_100545876	-------T---T-----	-DE	--NE---------L----Y-L-V-
Lysinibacillus xyleni	WP_097073524	-----I-----------	--G	---T---------I----Y-L-S-
Paenisporosarcina antarctica	WP_134208198	-----I-T---LPV--V	NEK	DV-R--T------L----Y-L-K-
Paenisporosarcina indica	WP_075620160	-----I-T---LPV--V	DEK	DV-R--T------L----Y-L-K-
Paenisporosarcina sp. HGH0030	WP_016429923	-----I-T---LPV--V	DEK	DV-R--T------L----Y-L-K-
Paenisporosarcina sp. K2R23-3	WP_119884290	-------T---LP---V	-EN	---SW-T------L----Y-L-K-
Paenisporosarcina sp. OV554	WP_108586031	-----I-T---LPV--V	DEK	DV-R--T------L----Y-L-K-
Paenisporosarcina sp. TG-14	WP_017381478	-----I-T---LPV--V	NEK	DV-R--T------L----Y-L-K-
Paenisporosarcina sp. TG20	WP_019415664	-----I-T---LPV--V	NEK	DV-R---------L----Y-L-K-
Planococcus antarcticus	WP_006828964	-----I-TV--LP---V	EEG	---SW-T-----FL----Y-L-V-
Planococcus citreus	WP_121301304	-------T---LP---V	-AG	-V-SW-T------L----Y-L-A-
Planococcus donghaensis	WP_008432546	-----I-TV--LP---V	EAG	---SW-T-----FL----Y-L-I-
Planococcus halocryophilus	WP_008497453	-----I-TV--LP---V	EAG	---SW-T-----FL----Y-L-I-
Planococcus halotolerans	WP_112224834	----A--T---LP---V	QAG	-A-SW-T-----FL----Y-L-V-
Planococcus maitriensis	WP_112233908	-------T---LP---V	-AG	-V-SW-T------L----Y-L-V-
Planococcus maritimus	WP_068459737	-------T---LP---V	-AG	-V-SW-T------L----Y-L-A-
Planococcus massiliensis	WP_052654043	-----I-TV--LP---V	EAG	---SW-T-----FL----Y-L-V-
Planococcus plakortidis	WP_068871396	-------T---LP---V	-AG	-V-SW-T------L----Y-L-V-
Planococcus rifietoensis	WP_058382132	-------T---LP---V	-AG	-V-SW-T------L----Y-L-A-
Planococcus salinarum	TAA68401	----A------LP---V	QAG	-A-SW-T-----FL----Y-L-V-
Planococcus salinus	WP_123166152	----A--T---LP---V	EAG	---SW-T-----FLS---Y-L---

Group	Species	Accession	Sequence	Boxed	Sequence
**Family *Planococcaceae* (>100/>100)**	Planococcus sp. CAU13	WP_033542769	----A--T---LP---V	QAG	---SW-T-----FL----Y-L---
	Planococcus sp. PAMC 21323	WP_038701967	-----I-TV--LP---V	EAG	---SW-T-----FL----Y-L-V-
	Planococcus sp. Y42	WP_077587681	-------T---LP---V	-SE	---SW--------L------L---
	Planococcus versutus	WP_049694573	-----I-TV--LP---V	EAG	---SW-T-----FL----Y-L-V-
	Planomicrobium flavidum	WP_088007323	-------T---LP---V	-AE	---SW--------L----Y-L-V-
	Planomicrobium glaciei	WP_074511471	-----I-TV--LP---V	DAG	---SW-T-----FL----Y-L-V-
	Planomicrobium okeanokoites	WP_117314077	----A--T---LP---V	QAG	-V-SW-T-----FL----Y-L-V-
	Planomicrobium soli	WP_106534440	-------T---LPS--V	EAG	---SW-T-----FLS---Y---V-
	Planomicrobium sp. Y74	WP_121636514	----A--T---LP---V	QAG	-A-SW-T-----FL----Y-L-V-
	Solibacillus isronensis	WP_079523466	-----I-----------	---	------------------Y-----
	Solibacillus kalamii	WP_087617842	-----I-----------	---	------------------Y-----
	Solibacillus sp. R5-41	WP_099425564	-----------------	---	------------------------
	Sporosarcina	WP_099633826	-----I-T---LP---V	TSG	-K-EW-TI-----L----Y-L-V-
	Sporosarcina koreensis	WP_060205514	-----I-T---LP---V	-SG	---SW-TI-----L----Y-L---
	Sporosarcina newyorkensis 2681	EGQ25187	-----I-T---LP---V	TSG	-K-EW-TI-----L------L-V-
	Sporosarcina pasteurii	WP_115363585	-----I-T---LP---V	-SG	---TW-TI-----L---SY---A-
	Sporosarcina psychrophila	WP_067204859	-----I-T---LP---V	-SG	---SW-TI-----L----Y---V-
	Sporosarcina sp. BI001-red	WP_116018292	-----I-TI--LP---V	-NG	EV-TW-TIM---------Y-L-A-
	Sporosarcina sp. D27	WP_025783825	-----I-T---LP---V	-KG	EV-TW-TIM---------Y-L-A-
	Sporosarcina sp. EUR3 2.2.2	WP_024536538	-----I-T---LPV--V	DEK	DV-R--T------L----Y-L-K-
	Sporosarcina sp. HY008	WP_067407756	-----I-T---LP---V	-SG	---TW-TI-----L----Y-L---
	Sporosarcina sp. P1	WP_099626735	-----I-T---LP---V	TSG	-K-EW-TI-----L----Y-L-V-
	Sporosarcina sp. P13	WP_099687659	-----I-T---LP---V	TSG	-R-EW-TI-----L------L-V-
	Sporosarcina sp. P17b	WP_099624827	-----I-T---LP---V	TSG	-K-EW-TI-----L----Y-L-I-
	Sporosarcina sp. P18a	WP_099675739	-----I-T---LP---V	TSG	-K-EW-TI-----L----Y-L-V-
	Sporosarcina sp. P19	WP_099691348	-----I-T---LP---V	TSG	-K-EW-TI-----L----Y-L-I-
	Sporosarcina sp. P20a	WP_099677981	-----I-T---LP---V	TSG	-K-EW-TI-----L----Y-L-V-
	Sporosarcina sp. P3	WP_099638291	---V-I-T---LP---V	TSG	-K-EW-TI-----L----Y-L-I-
	Sporosarcina sp. P33	WP_081243153	-----I-T---LP---V	TSG	-K-EW-TI-----L----Y-L-V-
	Sporosarcina sp. P34	WP_099694789	-----I-T---LP---V	TSG	-K-EW-TI-----L----Y-L-V-
	Sporosarcina sp. PTS2304	WP_114924967	-----I-T---LP---V	TSG	-R-EW-TI-----L------L-I-
	Sporosarcina sp. ZBG7A	WP_039043287	-----I-T---LP---V	-NG	EV-TW-TIM--------SY-L-A-
	Sporosarcina ureae	WP_085131978	-----I-T---LP---V	TSG	-K-EW-TI-----L----Y-L-V-
	Ureibacillus thermophilus	QBK25011	-----I-------P---	--G	---T--------------Y-L-A-
	Ureibacillus thermosphaericus	WP_016839512	-----I-C-----P---	-KG	---T---------I----Y-L-A-
	Viridibacillus arenosi	WP_038179191	-------T---L---WV	-T-	GG-E--T------L------L-V-
	Viridibacillus arenosi FSL R5-	ETT88714	-------T---L---WV	-T-	GG-E--T------L------L-V-
	Viridibacillus arvi	WP_053418587	-------T---L---WV	-T-	GG-E--T------L------L-V-
	Viridibacillus sp. OK051	WP_100795196	-------T---L---WV	-TE	GG-E--T------L------L-V-
	Alteribacillus iranensis	SFF00173	---Y-I-TI---P--TV	NAV	-ESKWLWIY-A--LTA-TW-L-T-
	Edaphobacillus lindanitolerans	WP_076758900	-----I-T---LP---V	-SG	---QW-TI-----L---MY-L---
	Filibacter sp. TB-66	WP_124068502	-----I-T---LP---V	-SG	---SW-TI-----L----Y-----
	Geomicrobium sp. JCM 19037	WP_042402211	---F-I-TI--VP--TV	QVV	-DS-WLWIY-A--I-A-TY-LIA-
	Psychrobacillus insolitus	WP_111440046	------ST---LPV--V	NA-	-V-SW-------IL----Y-L-A-
	Psychrobacillus psychrotoleran	WP_093537830	-----IST---LPV--V	-A-	-V-SW-T-----IL----Y-L-A-
	Psychrobacillus sp. OK028	WP_093062510	------ST---LPV--V	-A-	-V-SW-T-----IL------L-A-
	Psychrobacillus sp. OK032	WP_093274658	------ST---LPV--V	-A-	-V-SW-T-----IL----Y-L-A-
	Rummeliibacillus	WP_119415273	----F-SII--L---WV	-SS	GGNE-IT-----IL------L-E-
	Rummeliibacillus pycnus	WP_102694425	----F-SII--L---WV	-SF	GGNE-IT-----IL------L-E-
	Rummeliibacillus stabekisii	WP_066790832	---VF-SII--L---WV	-TE	GGNQ-ITI----IL------L-E-
	Saccharibacter sp. 3.A.1	WP_086431905	-----I-----------	--G	---T--------------Y-L-L-
**Other Bacteria (1/>100)**	Salsuginibacillus kocurii	WP_018924622	---Y-I-TI---P--TV	EEV	-NSKWLWIY-S--L-A-SW-L-L-
	Aeribacillus pallidus	WP_063387198	---V--SVI--MP--RV		-DRDWLY-M---ILIC--Y-L-I-
	Alkalibacillus haloalkaliphilu	WP_017186261	-------TI---P--T-		DPE WIM-MVV--L-C-TY-L-K-
	Anoxybacillus ayderensis	WP_021095315	---V---VI--FPV-RI		-EENWLY-M----LIC--Y-L-T-
	Anoxybacillus flavithermus	AST05775	---V-A-VI--FPV-RI		-EENWLY-M----LIC--Y-L-T-
	Anoxybacillus thermarum	WP_043964933	---V---VI--FPV-RI		-EENWLY-M----LIC--Y-L-T-
	Bacillus niacini	WP_034676194	-------VI--FPV-RV		-EPSW-Y-M--S-L-C--Y-L-I-
	Bacillus simplex	WP_061440403	---V---V---LPV-ST		-DESWLYFM-L--IVC--Y-L-V-
	Bacillus subtilis	KJJ42649	-L-V-I-A---FP--RV		-NEDWLY-M----M-C----L---
	Caldibacillus debilis	WP_020155541	---V---VI--LP--RN		-DPNWLQ-MIY--LVC-TY-L-S-
	Domibacillus aminovorans	WP_063964494	-------T---LPV-QA		-EESWLY-M--T-LPC----L---
	Domibacillus antri	WP_075397827	-------T---LPV-QA		-EESWLY-M--T-LPC----L-L-
	Falsibacillus pallidus	WP_114746812	-------V---LPV-TV		-EKSWLY-M----L-C--Y---V-
	Filobacillus milosensis	WP_134340863	-------T----P--NV		GEAEWIG-MIV--L-C-TY-L-T-

**Other Bacteria (1/>100)**	Jeotgalibacillus alimentarius	KIL53674	---V---T---LPV-RS	-ENYWLITIM-T-I-T--Y-M-A-
	Jeotgalibacillus campisalis	KIL52306	---V---T---LPV-RS	-EGYWLITIM-T-I-T--Y---A-
	Mycobacteroides abscessus subs	SHT64722	-------V---VPI-RV	-EKSWLY-MI---LIC--Y-L-A-
	Parageobacillus thermantarctic	WP_090949488	---V---VI--FP--RI	-DKDWLY-M----LVC--Y-L-I-
	Piscibacillus halophilus	WP_091775684	-------TV---P--RV	DEREW-M-MIV--L---TY-L-T-
	Sporosarcina globispora	WP_053433158	---V---AI--VPV-RV	-EESWLY-M----LVC-TY-L-I-
	Streptococcus pneumoniae	CEX89191	---V---I---VP--RI	-DTDWLY-MVI--LLC--Y-L-V-
	Tenuibacillus multivorans	WP_093857769	-------TV---PV-RV	GETEWIM-MIV--L-C-TY-L-T-
	Terrabacteria group	WP_095251986	-------VI--FPV-RV	-EHSW-Y-M--S-L-C--Y-L-I-
	Thermolongibacillus altinsuens	WP_132949304	---T---II--FPV-RI	-EESWLY-M----LIC--Y-L-I-
	Virgibacillus soli	WP_057984386	---V---I---LPV-RV	-EASW-Y-MI---I-C--Y-----

Supplemental Figure 14

A partial sequence alignment of the KinB-signaling pathway activation protein containing a three amino acid insertion (boxed) that is exclusively shared by all members among the *Planococcaceae* family and absent in all other bacteria. *Salsuginibacillus kocurii* is the only exception which also shares this CSI.

Family *Planococcaceae* (>100/>100)

		39		92
Bacillus ndiopicus	WP_042478071	WETAVQYHMYHALGLVAVGILGFD	VI	LGTTTVLQWASYLMVGGILLFSGSLYVL
Bacillus sp. OxB-1	WP_041073048	-------Q-F--I--L-I---MSS	SL	F-PS-S-S--G--ILA--II--------
Bacillaceae	WP_008177401	-------Q-----AIIGL---SNE	AL	--SSSL-S--G---FT--VF--------
Bhargavaea beijingensis	WP_092097414	----AK-Q----I--ILI---QSR	AL	--AASQ-N--GW--LA------------
Bhargavaea cecembensis	WP_008296590	----AR-Q-------ILI---QSQ	AL	--AVSQ-N--GW--LA--VI--------
Bhargavaea ginsengi	WP_092054644	----AR-Q-------ILI---QSQ	AL	--AVSQ-N--GW-LLA--VI--------
Caryophanon latum	WP_066464920	---GT--------A-------SN-	AL	--AS-E-S--A-CFIA--VF--------
Caryophanon tenue	WP_066547467	---GT--------AILL--V-LN-	SL	--DREQ-M--G-CFIA--VF--------
Kurthia gibsonii	WP_087680850	--K--T-Q-S-----I-I---MAP	S-	--PVKE-K--GN-LTI--I---------
Kurthia huakuii	WP_029498334	-----K-Q-------IVI---QL-	SL	F-AQ-IFST--LF-LI------------
Kurthia massiliensis	WP_010286239	-D---K-Q-----A-I-I---QT-	-L	F-SQ-IFAT--T--LI--I---------
Kurthia sp. 11kri321	WP_068457567	--K--T-Q-S-----I-I---MAP	S-	--PVKE-K--GN-LTI--I---------
Kurthia sp. Dielmo	WP_083851438	-----K-Q-------ILI---Q--	TL	---QSIFSA--W---I--I---------
Kurthia zopfii	WP_109350817	-DK--T-Q-S-----ILI---MSK	N-	F-PVKQ-N--G--LLV--I-------AM
Lysinibacillus acetophenoni	WP_097147583	-N-----Q-F--VAIIII-V-MST	KL	M-NVKT-K--AI--NL--VF-A------
Lysinibacillus boronitolerans	WP_016992209	-------Q------IL-L---SNE	AF	--SSSL-S--V---FT--VF--------
Lysinibacillus chungkukjangi	WP_107936206	-N-----Q-------ILL---SM-	AL	--NSGL-S--G---LA-VVF--------
Lysinibacillus composti	WP_124763480	-D-----Q-F--SA-LVI---MSS	KL	F-DVKQ-K--TIC-NL--VF-----FI-
Lysinibacillus contaminans	WP_053583358	-------Q----I--LIL---SS-	-L	---SSM-S--G---FA--IF------I-
Lysinibacillus endophyticus	WP_121214288	-N-----Q-------IIL-T-SN-	-L	--E-SL-M--G---LA-VIF--------
Lysinibacillus fusiformis	WP_004225138	-------Q------IL-L---SN-	AF	--SSSL-S--G---FT--VF--------
Lysinibacillus halotolerans	WP_122972231	-N-G---Q-------ILI---SI-	SL	--ASNL-S--G---FT-VIF--------
Lysinibacillus jejuensis	WP_108306086	-N-----Q-F--G--L-----MSR	KL	M-DVKL-NA-GWC-NI-----A----T-
Lysinibacillus macroides	WP_053995591	-------Q------IL-L---SN-	AF	--SSSL-S--V---FT--VF--------
Lysinibacillus manganicus	WP_036182553	-N------I------I-I---SM-	AL	V-ESSL-T--C---FT-VIF--------
Lysinibacillus mangiferihumi	WP_107895992	-------Q-F--T--LVI-L-MSS	KL	--EVKQ-KL-GIFLNL--VF-A------
Lysinibacillus massiliensis	WP_036170830	-N------------MI-I---SM-	AL	---SAL-S------FA-VIF--------
Lysinibacillus meyeri	WP_107841356	---------------I------Y-	A-	----S---------A--A-------I-
Lysinibacillus parviboronicapi	WP_054768126	-----------S---LVL-M-SS-	-L	---SSM-S--G---FA--IF------M-
Lysinibacillus saudimassiliens	CEA01618	-N-----Q-F--G--L-----MSR	KL	M-DVKL-NI-GWC-NI-----A----T-
Lysinibacillus sinduriensis	WP_036198288	-N-----Q---G---ILL---SM-	AL	--SSSL-S--G---FA--VF--------
Lysinibacillus sp. AR18-8	WP_066037288	-------Q------IL-L---SN-	AF	--SSSL-S--G---FT--VF--------
Lysinibacillus sp. B2A1	AVK82623	-------Q-F--T--LVI-L-MSS	KL	--EVKQ-NL-GIFFNL--VF------I-
Lysinibacillus sp. BK089	WP_132360246	-------Q-F--TAILVI---MSS	KL	--QVKQ-NL-GIFFNL--VF--------
Lysinibacillus sp. F5	WP_058843667	-------Q------IMGL---SNE	AL	--SSSL-S--V---FT--VF--------
Lysinibacillus sp. FJAT-14222	WP_053592586	-----------S--ILGL---SS-	AL	--ASSM-S--G---LA--VF--------
Lysinibacillus sp. FJAT-14745	WP_053484100	----------------GL---SS-	AL	--ASSM-S--G---FA--VF--------
Lysinibacillus sp. LK3	WP_048392583	-------Q------IL-L---SNE	AF	--SSSL-S--G---FT--VF--------
Lysinibacillus sp. Marseille-P	WP_106783246	-N-----Q-------FII---SME	NL	-----LFT------FA-VVF--------
Lysinibacillus sp. OL1	WP_131520076	-------Q------IL-L---SNA	AF	--SSSL-S--G---FT--VF--------
Lysinibacillus sp. PB300	WP_115674191	-------Q-F--T--LII-L-MSS	KL	--EVKQ-NL-GIFFNL--VF--------
Lysinibacillus sp. SYSU K30002	WP_126659321	-N-----Q---G--ILLIA--SM-	SL	--A-SL-R--G---FV-V-F--------
Lysinibacillus sp. YLB-03	WP_118875063	-D-----Q-F--AA-LGI---RSP	NL	A-SAKQ-KI-AVC-NL---F-A----I-
Lysinibacillus sp. YR326	WP_134020380	-------Q-F--TA-LVI---MSS	KL	--EVKQ-NL-GIFFNL--VF--------
Lysinibacillus sp. YS11	WP_103117406	-------Q-F--T--LII-L-MSS	KL	--EVKQ-NL-GIFFNL--VF--------
Lysinibacillus sp. ZYM-1	WP_054612448	-------Q------IIGL---SNE	AL	--SSSL-S--G---FT--VF--------
Lysinibacillus sphaericus	WP_010857904	-------Q-F--T--LVI-L-MST	KL	--EVKQ-NV-GIFFNL--VF--------
Lysinibacillus telephonicus	WP_126293227	-D--I--Q-F--TA-LVI---MSS	KL	--ASKQ-K--AIC-NL--IF-T---L--
Lysinibacillus xylanilyticus	WP_049664900	---------------LGL---SS-	AL	--ASSM-S--G----A--VF--------
Lysinibacillus xyleni	WP_097072435	-N-----Q------MI-LA--SS-	GL	I-E-NF-I--G---FA-VIF--------
Paenisporosarcina antarctica	WP_134210714	-------Q----I--IVI---MST	N-	I-NVSQ-S--G---LT--VI--------
Paenisporosarcina indica	WP_075618429	-------Q----I--IVI---MSS	S-	I-NVSQ-N--G-F-LA--VI--------
Paenisporosarcina sp. HGH0030	WP_016428326	-D-----Q----I--IVI---MSS	N-	I-NVSQ-S--G---LA--VI--------
Paenisporosarcina sp. K2R23-3	WP_119882520	-------Q-F--VA-LVI-V-LST	SL	--PVSS-N--G--LLA--II--------
Paenisporosarcina sp. OV554	WP_108586159	--I----Q----I--IVI---MST	N-	I-NVSQ-S--G---LA--VI--------
Paenisporosarcina sp. TG-14	WP_017381766	-------Q----I--IVI---MST	N-	I-NVSQ-S--G---LS--VI--------
Paenisporosarcina sp. TG20	WP_019414494	-------Q----I--IVI---MST	N-	I-NVSQ-G--G---LA--II--------
Planococcus antarcticus	WP_006829292	-Q-----Q-F-SI--IV-A--MSS	SL	--SLGA-N--G---LA--VI--------
Planococcus citreus	WP_121298422	-Q-----Q-F-SI--I--A--MSS	AL	I-PLGS-N--G---LA--VI--------
Planococcus halotolerans	WP_112221258	-Q-----Q-F-SI--MV-AV-MST	TL	I-PLGS-S--G---LA--II--------
Planococcus maitriensis	WP_112230388	-Q-----Q-F-SI--I-IA--MSS	AL	--PLGS-S--G---LA--VI--------
Planococcus maritimus	WP_068464303	-Q-----Q-F-SI--I--A--MSS	AL	I-PLSS-S--G---LA--VI--------
Planococcus massiliensis	WP_052650636	-Q-----Q-F-SI--I-IA--MSS	SL	I-NLGS-S--G---LA--II--------
Planococcus plakortidis	WP_068870138	-Q-----Q-F-SI--I--A--MSS	AL	I-PLGS-S--G---LA--VV--------
Planococcus rifietoensis	WP_058382908	-Q-----Q-F-SI--I--A--MSS	AL	I-PLGS-N--G---LA--VI--------

Group	Organism	Accession			
Family *Planococcaceae* (>100/>100)	Planococcus salinarum	OHX57190	-Q-----Q-F-SI--MV-A--MSS	SL	--PLGS-S--G---LA--VI--------
	Planococcus salinus	WP_123164578	-Q-----Q-F-SI--MV-AL-MSS	TL	I-QLGS-S--G---LA--VI--------
	Planococcus sp. CAU13	WP_033541346	-Q-----Q-F-SI--II----MSS	TL	I-SLGS-S--G---LA--II--------
	Planococcus sp. PAMC 21323	WP_038704766	-Q-----Q-F-SI--MV-A--MSS	SL	--SLGS-N--G---LA--VI--------
	Planococcus sp. Y42	WP_077590221	-Q-----Q-F--T--LI--L-MST	AF	I-PISS-N--G--LLA--VI--------
	Planococcus versutus	WP_049692971	-Q-----Q-F-SI--IV-AV-MSS	SL	--SLGS-S--G---LA--VI--------
	Planomicrobium flavidum	WP_088007662	-Q-----Q-F--T--LI--L-MSA	AL	I-PLSS-S--G--LLA--II--------
	Planomicrobium glaciei	WP_036802941	-Q-----Q-F--I--MV----MSS	SL	I--LGS-S--G---LA--II--------
	Planomicrobium okeanokoites	WP_084244156	-Q-----Q-F-SI--M--A--MSS	AL	--PLGS-S--G---LA--VI--------
	Planomicrobium soli	WP_106532800	-Q-----Q-F-SI--MIIA--MSS	AF	I-PLSS-S--G---LA--VF--------
	Planomicrobium sp. MB-3u-38	WP_101801393	-Q-----Q-F-SI--IV-AV-MST	TL	I-PLGS-N--G---LA--II------I-
	Planomicrobium sp. Y74	WP_121635623	-Q-----Q-F-SI--MV-AV-MST	TL	I-PLGS-S--G---LA--II--------
	Sporosarcina koreensis	WP_060207032	-------Q-F--I--I-----MST	SL	--QS-T-T--G--LLA--II------I-
	Sporosarcina newyorkensis	WP_009766268	-------Q-F--I--L-----MSS	TL	--PS-Q-T--G--LLA--VI------A-
	Sporosarcina pasteurii	WP_115359886	-------Q-F-----L-I---ASS	S-	W-ASPQ-N--G--LLA--II------A-
	Sporosarcina psychrophila	WP_067213264	-------Q-F---A-L-I---MSP	SL	F-PV-Q-S--G--ILA--II--------
	Sporosarcina sp. BI001-red	WP_116019636	--K--H-Q-F--IA-L-I---MSP	AL	F-SS-A-S--G---LA--II--------
	Sporosarcina sp. D27	WP_025785540	--K--H-Q-F--IA-L-I---MSP	AL	F-SS-A-S--G--LLA--II--------
	Sporosarcina sp. EUR3 2.2.2	WP_024535624	-------Q----I--IVI---MHS	N-	I-NVSQ-S--G---LA--VI--------
	Sporosarcina sp. HY008	WP_067405352	-------Q-F-S-----I---MST	SL	M-A-AQ-S--G--LLA--II------A-
	Sporosarcina sp. P13	WP_099687957	-------Q-F--I--L-----MSS	AL	--PS-Q-S--G--LLA--II------A-
	Sporosarcina sp. P16b	WP_099672296	-----H-Q-F--IA-L-I---MS-	TL	--PS-Q-S--G--ILT--VI--------
	Sporosarcina sp. P18a	WP_099675588	-----H-Q-F--IA-L-I---MS-	TL	--PS-Q-L--G--ILA--VI--------
	Sporosarcina sp. P19	WP_099691866	-------Q-F--IA-L-I---MSS	TL	--PS-Q-S--G--ILA--IV--------
	Sporosarcina sp. P2	WP_099631204	-----H-Q-F--IA-L-I---MS-	TL	--PS-Q-S--G--ILA--VI--------
	Sporosarcina sp. P34	WP_099695462	-----H-Q-F--IA-LVI---MSS	TL	--PS-Q-S--G--ILA--VI--------
	Sporosarcina sp. P7	WP_099637574	-----H-Q-F--IA-L-I---MS-	TL	--PS-Q-S--G--ILA--VI--------
	Sporosarcina sp. PTS2304	WP_114925630	-------Q-F--I--L-I---MSS	SL	--PSAQ-S-SG--LLI--IV------A-
	Sporosarcina sp. ZBG7A	WP_039042733	--K--H-Q-F--IA-L-I---MSP	AL	F-SSNA-S--G--LLV--II--------
	Sporosarcina ureae	WP_029053505	-------Q-F--IA-L-I---MSS	TL	--PS-Q-S--G--ILA--II--------
	Psychrobacillus insolitus	WP_111439264	-G-----Q-F---A---I---MSS	KV	--HVSQ-NI-G--ILA--VI--------
	Psychrobacillus psychrodurans	WP_093493750	-D-----Q-F-S-A-I-I---MSS	KV	--PLST-NT-G--ILA--VI--------
	Psychrobacillus psychrotoleran	WP_093535853	-------Q-F---A---I---MSS	KV	--PIST-NT-G-FILA--II--------
	Psychrobacillus sp. OK028	WP_093061371	-------Q-F---A---I---MSS	KL	--PVSS-NT-G---IV--V--A------
	Psychrobacillus sp. OK032	WP_093267714	-------Q-F---A-I-I---MSS	KL	I-SVSS-NT-G--ILA--I---------
	Rummeliibacillus pycnus	WP_102693150	---G---Q-F--I--IVI---MSK	SL	I-PKSL-SK-GIM-FI-VI---------
	Rummeliibacillus stabekisii	WP_066791652	Y------Q-F--V--ILI---MSK	SL	F-PIPL-SR-GMI-LI-TIV--------
	Butyricicoccus sp. 1XD8-22	RKJ17424	-N-----------MI-I---SM-	AL	---SAL-S------FA-VIF--------
	Edaphobacillus lindanitolerans	WP_076756833	--K-AM-Q----I--III-V-KHP	SL	--NA-Q-S--GW--FA--I---------
	Filibacter sp. TB-66	WP_124068746	-------Q-F-----III-V-MSK	S-	--PI-Q-S--G--ILA--II------I-
	Scopulibacillus darangshiensis	WP_132747894	F--G---Q-M------LI---ANT	M-	R-ESGL-N--GW--LA--VI------L-
	Sporolactobacillus inulinus	WP_010026414	FQ-G-H-Q-------IVI---ALT	IF	S-QS-L-S-SGW--TV-V--------A-
	Sporolactobacillus nakayamae	WP_093670887	FQ-G-H-Q-------IVI-L-ALT	-F	S-QSML-S-SGW--TV----------A-
	Sporolactobacillus sp. THM19-2	WP_129928463	FQ-G---Q---S---ILIA--AMT	-L	Q-SAGL----GWMLTL-VV---------
	Sporolactobacillus sp. THM7-4	WP_130030849	YQ-G---Q-F-S---MII---AAG	AV	HESSGL----GW--LA--I-------A-
	Sporolactobacillus terrae	WP_028976153	FQ-G---Q-V-----ILI---ALT	-F	T-Q--L-A--GW--TL-VV-------A-
	Sporolactobacillus vineae	WP_010630674	Y--G---Q---S---ILF-L-ALT	-L	H-SASL-S--GG--IL--V-------A-
	Viridibacillus	WP_038180164	-D-----Q-F-----I-I---MSS	SL	I-PV-Q-SR-G---LA--VF-A------
	Viridibacillus sp. OK051	WP_100794898	-D-----Q-F--IA-I-I---MSS	SL	I-PVAQ-SR-G---LA--VF-A------
	Chryseomicrobium excrementi	WP_100353352	-------Q-F--VA-I-IA--MHP	NL	--ASGA-ST-G-AILI--II-----FA-
Other Bacteria (0/>100)	Bacillus sporothermodurans	WP_066235221	-K-G-T-Q-F----IL-I---AGY	-	QQGSSLIT-SGW--FI--IF--------
	Sporosarcina globispora	WP_053433419	-K-G-T-Q-F--T-ILII-V-LGK		-PA-SL-S-SGW--LI--I---------
	Aliicoccus persicus	WP_091475310	-------Q-F-TG--L-I-V-LNV		PSKHLVR -GW--TA--VF------I-
	Alteribacillus iranensis	WP_091659568	------QTM-----L---LIAAY		A-SSST-S--GW-LFI---I------TM
	Bacillaceae bacterium EAG3	WP_104848491	-Q-G---Q-F--G--FVI-L-WNH		-SSHNL-S-SGWA-LA--I-------I-
	Bacillus deserti	WP_101641292	-K---H-Q-F--S--LII---AST		-KAGNL-N-SGW--LI--I-------I-
	Bacillus kochii	WP_095371531	-Q-----Q-F--I-IFI-AL-LSK		MPDSSL-T-SGW--AF---F------I-
	Bacillus nealsonii	WP_101178322	-K-G-T-Q-F-SM-ILII-A-MMK		IPQSSL-T-SGW---A--I--A------
	Bacillus simplex	WP_063234196	-Q-----Q-F--V--LVI-L-AGK		ISSPLIN-SGW--LI--I------F--
	Bacillus smithii	WP_003354337	-K-G---Q-F-----LI-A--AGK		YPASSLIS-SGW-LFI--V---------
	Bacillus subterraneus	WP_044393690	-K-G-T-Q-F--I-ILFI-V-LGK		-PA-SL-N-SGW--LI--I-------T-
	Bacillus subtilis group	WP_013354008	-H-G----------L--AF-AEK		-SGVGSVSV-GW--FA--V---------
	Domibacillus aminovorans	WP_063966612	-Q-----Q-F--I--II--F-MGQ		FPDSSA-N-SGW--LA--I------F--
	Domibacillus tundrae	WP_046179081	-K-----Q-F--I--MI--F-MGQ		P-SSA -N-SGW--MA--V------F--
	Falsibacillus pallidus	WP_114745850	-H-----Q-F----II-I---AGN		YASSQL-S-SGW--LI--I---------

Other Bacteria (0/>100)

```
Fictibacillus gelatini           WP_026678816  YK-G----------IL--ALIASK    -TNSSLVN-SGW-LFI--I---------
Fictibacillus macauensis         WP_007203920  YK-G----------IL---F-AMK    I--PSS-V-SGW---I--I-------I-
Gammaproteobacteria bacterium    PKM33647       K-G-T-Q-W-----C-I-L-HRQ    NPRQAL-S-SAW--FA--V-------L-
Jeotgalibacillus campisalis      WP_041053456  -------Q-F--A-ILII---MGT    -P--GL-S--GW--LV--IF--------
Jeotgalibacillus soli            WP_041086914  -N-----Q-F--T-ILVI---MAN    -PA-GL-N--GW--LV--IF------I-
Listeria innocua                 WP_010990547  ---G---Q-F--V-ILI----MEK     QASRLYA--AI-FSI--VF--------
Listeria ivanovii                WP_014092115  ---G---Q-F--V-ILV--L-MEK     QASQLYA--VI-FSV--AF------I-
Macrococcus lamae                WP_133443638  --K----Q-------LVI-V-SGT      --LN-NV-GW--TA--IF------I-
Methylobacter tundripaludum      WP_104422023  YQ-G-T-Q-------IG-ALISRQ    ASDSRL-H--GW--FA----------L-
Methylomicrobium alcaliphilum    WP_014150280   K-G-T-Q-W-----C-I-L-HRQ    NPRQAL-S-SAW--FA--V-------L-
Nitrosococcus halophilus         WP_013033830  -Q-G-E---------ILI--ISHW    I-GGSLIK-SGI--FI----------L-
Nitrosococcus oceani             WP_002809289  -Q-G-E---------ILI-MISHW    --SSALIK-SGG--LA------------
Paenibacillus amylolyticus       WP_123064997  Y--G-----I---A-LI--LTAGQ    --AS-K-K--AR-LFA--II--------
Paenibacillus xerothermodurans   WP_089199614  Y--G---Q-M-S---I-L--AAAF    -PSSGK-SA-GW--LA--V---------
Parageobacillus caldoxylosilyt   WP_042406875  -K-----Q-F-----F---L-LGK    FPQAGLISI-GWM-FV--V---------
Salinicoccus alkaliphilus        WP_072710466  -------Q-F--A--I-I---VGL      -GVPMNA-GW--FA--IF--------
Salinicoccus sediminis           WP_046513222  --K----Q-------L-----MQI     TNAPL-GASGW--LL--IF------I-
Salsuginibacillus kocurii        WP_018923238   Q-G-----F----IILI-LVAAF    A-SSGL-T--GWV-FV---F------TM
Staphylococcus argensis          WP_103371574  --K-TM-Q---G---L-I--ISGT      -NINVN--GW--FI--I-------I-
Staphylococcus aureus            PAJ05573      --K-TT-Q---G-A-LII-VISGT      -SINVN--GW-IFA--IF------I-
Sulfobacillus thermosulfidooxi   WP_053959116  YQ-G---Q----G--IVT-L-SHI    IAQS-L-T--GW-FLA--V-------I-
Sulfobacillus thermotolerans     AUW93618      QTGVQYQMYHALGL--TGLVSIKA     PG--L-A--GW-F-V--V---------
```

Supplemental Figure 15

A partial sequence alignment of the DUF423 domain-containing protein containing a 2-4 amino acid insertion (boxed) that is exclusively shared by all members among the *Planococcaceae* family and absent in all other bacteria.

			12		56
	Lysinibacillus xylanilyticus	WP_049667946	PHPDDEAFSIAGTIAYY	T	KKMNTPVTYACLTLGEMGRNLGNPP
	Lysinibacillus boronitolerans	WP_016993387	---------V-------	-	-------------------------
	Lysinibacillus contaminans	WP_053585202	-----------------	-	-------------------------
	Lysinibacillus fusiformis	WP_004225429	-----------------	-	-------------------------
	Lysinibacillus macroides	WP_053993183	-----------------	-	-------------------------
***Lysinibacillus sensu stricto* (11/11)**	Lysinibacillus parviboronicapiens	WP_107947582	---------V-------	-	-------------------------
	Lysinibacillus mangiferihumi	WP_069512873	---------V-------	-	-------------------------
	Lysinibacillus varians	WP_024362826	---------V-------	-	-------------------------
	Lysinibacillus sphaericus	WP_010857894	---------V-------	-	-------------------------
	Lysinibacillus tabacifolii	WP_024362826	---------V-------	-	-------------------------
	Bacillus sp. B14905	WP_008176035	---------V---T---	-	-------------------------
	Anoxybacillus geothermalis	WP_044743040	--------GVS----QH		VQSG----------------M-V--
	Anoxybacillus sp. UARK-01	WP_080859758	--------GVS--L-MH		AEKG--I-------------M-T--
	Bacillus acanthi	WP_108668797	--------GVS----S-		I-AGV----------Q---------
	Bacillus acidicola	WP_066267490	--------GVS---SLH		V-NG-----------Q---------
	Bacillus acidiproducens	WP_018661301	--------GVS----TH		VNHG-------------A-------
	Bacillus alcalophilus	WP_004428660	--------GVS---SA-		RAKQI--------------------
	Bacillus andreraoultii	WP_033827693	--------GVS----SH		V-QG---------------------
	Bacillus anthracis	OON45435	------SYCV----LA-		TQR-V-L--V---------AM----
	Bacillus aquimaris	WP_044340176	--------GVS---STH		V-NE---------------------
	Bacillus asahii	WP_127762702	------S-AV---L-LH		IQAG-----M---------------
	Bacillus azotoformans	WP_003329509	--------GAS---SL-		SEKG-------C--------M----
	Bacillus badius	WP_063384848	--------GVS----TH		R-RG---------------------
	Bacillus bataviensis	WP_007085204	--------GVS--L-TH		VQNG----------------M----
	Bacillus boroniphilus JCM 2173	GAE45025	--------GVS----SH		VN-G-----------Q----M----
	Bacillus butanolivorans	WP_098177115	---------VS--L-LH		REAG-----L---------------
	Bacillus camelliae	WP_101354450	---------VS---STH		V-NG-----------Q---------
	Bacillus campisalis	WP_046523833	--------GVS----SH		IN-G----------------M----
	Bacillus canaveralius	WP_101578132	--------GVS----TH		VRQGS---------------M----
	Bacillus cavernae	WP_126863591	--------GVS----LN		IENG---------------------
	Bacillus cecembensis	WP_057986633	---------V---VRM-		RN-AV--------------------
	Bacillus firmus	WP_081757231	--------GVS---SLH		IDNG----------------M----
	Bacillus foraminis	WP_121610492	--------GVS----SH		VN-G----------------M----
	Bacillus fumarioli	WP_066367228	--------GVS----TH		I-SG----------------M----
	Bacillus ginsengihumi	WP_025727588	--------GVS---STH		I-QG---------------------
	Bacillus gottheilii	WP_066442193	--------GVS----TH		VQNG----------------M----
	Bacillus halodurans	WP_010899461	--------GVS----LF		R-QGV--------------------
	Bacillus hemicellulosilyticus	WP_035343448	------T-GA-----MH		ANQG--I------------------
	Bacillus horneckiae	WP_066398475	--------GVS----S-		T-RG----------------M----
	Bacillus jeotgali	WP_102264652	--------GVS----SH		VN-G-----------Q----M----
	Bacillus korlensis	WP_066057020	--------GVS--L-LH		IQNG---------------------
	Bacillus kribbensis	WP_035324245	--------GVS----SH		VQNG-----------Q---------
**Other Bacteria (0/>300)**	Bacillus krulwichiae	WP_066149477	--------GVS--L-MH		IANG--I------------------
	Bacillus kwashiorkori	WP_062351758	--------G-S--L-T-		IQNEV--------------------
	Bacillus lentus	WP_066143784	--------GVS----A-		IQQGV--------------------
	Bacillus ligniniphilus	WP_026104979	--------GVS---SLH		V-NG---------------------
	Bacillus loiseleuriae	WP_049683369	--------GVS----S-		IESG---------------------
	Bacillus marisflavi	WP_121621044	--------GVS---STH		TNSG---------------------
	Bacillus marmarensis	WP_022627929	--------GVS----MH		TSSG---------------------
	Bacillus mediterraneensis	WP_071461461	--------GVS----SH		VN-G----------------M----
	Bacillus mesonae	WP_066389668	--------GVS----TH		V-NG----------------M----
	Bacillus methanolicus	WP_003346920	--------GVS----S-		TRNGI---------------M----
	Bacillus muralis	WP_064467058	---------VS--V-IH		REAG-----L---------------
	Bacillus ndiopicus	WP_042470698	---------V--IARL-		HQ-GV--------------------
	Bacillus nealsonii	WP_101178312	---------VS---SRH		IHEG-----------Q---------
	Bacillus niacini	WP_045514802	--------GVS----TH		VQNG----------------M----
	Bacillus niameyensis	WP_062108320	--------GVS--M-SH		V-NG---------------------
	Bacillus notoginsengisoli	WP_118921026	--------GVS----MH		T-NG----------------M----
	Bacillus novalis	WP_066094092	--------GVS----TH		VQNG----------------M----
	Bacillus oceanisediminis	WP_019380556	--------GVS---SLH		IDNG----------------M----
	Bacillus patagoniensis	WP_078390998	--------GVS----SH		IDVG---------------------
	Bacillus persicus	WP_090748551	--------GVS----SH		VN-G----------------M----
	Bacillus praedii	WP_057760018	--------GVS----S-		T-SGI---------------M----
	Bacillus pseudofirmus	WP_012959161	--------GVS----MH		TSSG---------------------
	Bacillus psychrosaccharolyticu	WP_040376349	--------GVS----LH		IEN------I-M-------------
	Bacillus rubiinfantis	WP_042354439	--------GVS----TH		I-DG---------------------
	Bacillus selenatarsenatis	WP_041966315	--------GVS----SH		VNLG-----------Q----M----
	Bacillus shacheensis	WP_059104822	--------GVS----GH		IDNG---------------------
	Bacillus smithii	WP_048624560	-------LGVS--L-I-		A-N-V-I------------------
	Bacillus sporothermodurans	WP_066230497	--------GVS----TH		V-NG---------------------
	Bacillus subterraneus	WP_125480197	--------GVS----SH		VN-G----------------M----
	Bacillus thermoamylovorans	CEE03174	--------GVS----SF		V-QG---------------------
	Bacillus vireti	WP_024029473	--------GVS----TH		VQNG----------------M----
	Bacillus wakoensis	WP_034741547	------T-GV-----MH		VANG---------------------

Other Bacteria (0/>300)

Bacillus wudalianchiensis	WP_065410119	--------GVS----TH	R-RG---------------------
Bacillus zeae	WP_119111999	------S-GVS----SH	VN-G----------------M----
Bhargavaea cecembensis	WP_040227486	--------GVS---ST-	IQ-GV-----------------M--
Butyricicoccus sp. 1XD8-22	RKJ31855	---------S----RL-	RN-GV--------------------
Caldibacillus debilis	WP_020154432	--------GVS----T-	IRNG--L------------------
Caryophanon latum	WP_066466106	---------C--VVRQM	T-R-I--------------------
Couchioplanes caeruleus	WP_071808666	------T--S---L-S-	IQKG-----------Q---------
Dietzia cinnamea	WP_063973099	--------GVS----SH	IN-G-----------Q----M----
Domibacillus aminovorans	WP_082862920	--------GVS----IH	T-RE---------------------
Domibacillus antri	WP_075397070	--------GVS----VH	TARE---------------------
Domibacillus enclensis	SIQ23599	--------GVS----VH	TERK---------------------
Domibacillus epiphyticus	WP_083711496	--------GVS----IH	TGRE---------------------
Domibacillus indicus	WP_046174713	--------GVS----VH	TERK---------------------
Domibacillus iocasae	WP_069938662	--------GVS----VH	TERK---------------------
Domibacillus mangrovi	WP_083566268	--------GVS----IH	T-RE---------------------
Domibacillus robiginosus	WP_050180456	--------GVS----IH	TGRK---------------------
Domibacillus tundrae	WP_046179129	--------GVS----IH	TERK---------------------
Edaphobacillus lindanitolerans	WP_076756839	--------GVS-S-ST-	IQ-GV-----------------M--
Falsibacillus pallidus	WP_114745840	--------GVS----TH	IQNG-----------Q---------
Falsibacillus sp. GY 10110	WP_121681895	--------GVS----TH	V-HG---------------------
Fictibacillus arsenicus	WP_066286048	--------GVS----KH	T-KGS----I-----Q----M----
Fictibacillus phosphorivorans	WP_066243206	--------GVS----QH	T-KGS----I-----Q----M----
Filibacter sp. TB-66	WP_124068740	--------GVS---SI-	RE-GV--------------------
Geobacillus kaustophilus	WP_044732348	--------GVS----EH	AQNG----------------M-T--
Halobacillus halophilus	WP_014642162	--------GVS---TS-	I-QG--L------------------
Jeotgalibacillus campisalis	WP_041053478	--------GVS----TH	LQ-G-G-------------------
Jeotgalibacillus salarius	WP_134382214	--------GVS----SH	I-KG-D---------Q---------
Jeotgalibacillus soli	WP_041086908	--------GVS----SH	IQA------------Q---------
Kurthia massiliensis	WP_010287167	--------GVS--L-Q-	AQAGV-I------------------
Kurthia zopfii	WP_109350915	--------GVS--L-Q-	ANAGV-I------------------
Lysinibacillus acetophenoni	WP_097147578	---------S----RL-	RN-GV--------------------
Lysinibacillus chungkukjangi	WP_107936772	---------S----RM-	RN-GV--------------------
Lysinibacillus composti	WP_124763490	---------S----RL-	RN-GV--------------------
Lysinibacillus endophyticus	WP_121215496	---------S----RL-	RNAGV--------------------
Lysinibacillus halotolerans	WP_122972436	---------S----RL-	RN-GV--------------------
Lysinibacillus jejuensis	WP_108306092	------S--V--IA-MQ	AA-GV-I------------------
Lysinibacillus manganicus	WP_036182408	---------S----RL-	RN-GV--------------------
Lysinibacillus massiliensis	WP_036171102	---------S----RL-	RN-GV--------------------
Lysinibacillus meyeri	WP_107838270	---------V--ITRL-	HE-SV--------------------
Lysinibacillus odysseyi	WP_036153162	---------C--SVRL-	AN-GV--------------------
Lysinibacillus saudimassiliensis	CEA01601	------S--V--IA-MQ	AS-GV--------------------
Lysinibacillus sinduriensis	WP_036200036	---------S----RL-	RN-GV--------------------
Lysinibacillus sp. 2017	WP_108711471	---------V--SVRM-	RN-GV--------------------
Lysinibacillus sp. BF-4	WP_036145405	------S--V--IA-MQ	AS-GV--------------------
Lysinibacillus sp. Marseille-P	WP_106783408	---------S----RL-	RN-GV--------------------
Lysinibacillus sp. SYSU K30002	WP_126658849	---------S----RL-	RN-GV--------------------
Lysinibacillus sp. YLB-03	WP_118875069	---------S----RL-	RN-GV--------------------
Lysinibacillus sphaericus	WP_125102578	--------GVS----T-	IE-GV--------------------
Lysinibacillus telephonicus	WP_126293219	---------S----RL-	RN-GV--------------------
Lysinibacillus xyleni	WP_097071621	---------S----RL-	RN-GV--------------------
Paenisporosarcina antarctica	WP_134210720	--------GVS----T-	I-QG---------------------
Paenisporosarcina indica	WP_075618437	------S-GVS----T-	I-QG---------------------
Paenisporosarcina quisquiliaru	WP_090564572	--------GVS----T-	INQGI--------------------
Paenisporosarcina sp. HGH0030	WP_016428339	--------GVS---SM-	I-QG---------------------
Paenisporosarcina sp. K2R23-3	WP_119882510	--------GVS---SHF	RQEG---------------------
Paenisporosarcina sp. OV554	WP_108586168	--------GVS---SM-	I-QG---------------------
Paenisporosarcina sp. TG-14	WP_017381773	--------GVS---ST-	I-QG---------------------
Paenisporosarcina sp. TG20	WP_019412402	--------GVS---ST-	I-QG---------------------
Paraburkholderia tropica	RQN27000	------T--SS----S-	IEKGI----------Q---------
Planococcus antarcticus	WP_006829302	--------GVS---TTH	IQQG---------------------
Planococcus donghaensis	WP_008430438	--------GVS---TTH	IQQG---------------------
Planococcus faecalis	WP_071153015	--------GVS---TTH	IQQG---------------------
Planococcus halocryophilus	WP_008496839	--------GVS---TTH	IQQG---------------------
Planococcus halotolerans	WP_112221268	--------GVS--MTTH	I-QG---------------------
Planococcus kocurii	WP_058384407	--------GVS---TTH	IQQG---------------------
Planococcus maitriensis	WP_112230374	--------GVS---TTH	IQQG---------------------
Planococcus maritimus	WP_069576547	--------GVS---TTH	IQQG---------------------
Planococcus massiliensis	WP_052650625	--------GVS---STH	I-RG---------------------
Planococcus plakortidis	WP_068870150	--------GVS---TTH	IQQG---------------------
Planococcus salinarum	OHX57186	--------GVS---TTH	I-QG---------------------
Planococcus salinus	WP_123164587	--------GVS--MSTH	VDRG---------------------
Planococcus sp. CAU13	WP_033541336	--------GVS--MTTH	I-QG---------------------
Planococcus sp. PAMC 21323	WP_038704773	--------GVS---TTH	IQQG---------------------
Planococcus sp. Y42	WP_077590231	--------GVS---SS-	IEQG---------------------

Other Bacteria (0/>300)	Planococcus versutus	WP_049692979	--------GVS---TTH	IQQG--------------------
	Planomicrobium flavidum	WP_088008730	--------GVS---ST-	IEQDI-------------------
	Planomicrobium glaciei	WP_036802959	--------GVS---STH	I-QG--------------------
	Planomicrobium okeanokoites	WP_117313702	--------GVS--MTTH	INQG--------------------
	Planomicrobium soli	WP_106532793	--------GVS---ST-	I-QG--------------------
	Planomicrobium sp. MB-3u-38	WP_101801383	--------GVS--MTTH	I-QG--------------------
	Planomicrobium sp. Y74	WP_121635614	--------GVS--MTTH	I-QGS-------------------
	Pseudomonas syringae pv. pisi	PYD83933	------T--S---L-S-	IQKGI----------Q---------
	Psychrobacillus insolitus	WP_111439258	--------GVS----T-	I-QGV-------------------
	Psychrobacillus psychrodurans	WP_093493738	--------GVS----T-	INQGI-------------------
	Psychrobacillus psychrotoleran	WP_093535869	--------GVS----T-	I-QGI-------------------
	Psychrobacillus sp. OK028	WP_093061379	--------GVS----T-	INQGI-------------------
	Psychrobacillus sp. OK032	WP_093267694	--------GVS----T-	V-QGI-------------------
	Quasibacillus thermotolerans	KKB36467	--------GVS--L-VH	I-RG--------------------
	Rhodococcus qingshengii	WP_133369805	--------GVS----TH	VQNG---------------M----
	Rummeliibacillus pycnus	WP_102693159	--------GVS--L-Q-	AQAG--I-----------------
	Rummeliibacillus stabekisii	WP_066791639	--------GVS--L-Q-	A-AG--I-----------------
	Salimicrobium flavidum	WP_076559074	--------GVS--MSS-	INQGV-------------------
	Solibacillus isronensis	WP_079524589	---------V---LRLF	HN-GV-------------------
	Solibacillus kalamii	WP_087615704	---------V---LRLF	HN-GV-------------------
	Solibacillus silvestris	WP_014824761	---------V---LRLF	HN-GV-------------------
	Solibacillus sp. R5-41	WP_099422347	---------V---VRM-	SN-GV-------------------
	Sporosarcina globispora	WP_053433407	--------GVS---SLH	IDNG---------------M----
	Sporosarcina koreensis	WP_040287606	--------GVS----T-	IE-GV-------------------
	Sporosarcina newyorkensis	WP_040758536	--------GVS---SK-	IE-GV-------------------
	Sporosarcina pasteurii	WP_115359879	--------G-S-S--A-	R--GV-------------------
	Sporosarcina psychrophila	WP_067213280	--------GVS---SS-	RDIGV-------------------
	Sporosarcina sp. BI001-red	WP_116019809	--------GVS----T-	IE-GV-------------------
	Sporosarcina sp. EUR3 2.2.2	WP_024535633	--------GVS----T-	I-QG--------------------
	Sporosarcina sp. HYO08	WP_067405335	--------G-S-S-SM-	RE-GV-------------------
	Sporosarcina sp. P1	WP_099628068	------S-GT-----E-	IE-GV-------------------
	Sporosarcina sp. P13	WP_099687964	------S-GS-----T-	VE-GV-I-----------------
	Sporosarcina sp. P17b	WP_099625015	------S-G------E-	IE-GV-------------------
	Sporosarcina sp. P18a	WP_099675592	------S-G------Q-	IE-GV-------------------
	Sporosarcina sp. P19	WP_099691862	------S-GT-----Q-	IE-GV-------------------
	Sporosarcina sp. P20a	WP_099678769	------S-GT-----Q-	IE-GV-------------------
	Sporosarcina sp. P21c	WP_099628206	------S-GT-----E-	IE-GV-------------------
	Sporosarcina sp. P29	WP_099662552	------S-G------E-	IE-GV-------------------
	Sporosarcina sp. P3	WP_099638796	------S-GT-----Q-	IE-GV-------------------
	Sporosarcina sp. P33	WP_081243788	------S-GS-----E-	IE-GV-------------------
	Sporosarcina sp. P34	WP_099695468	------S-GS-----Q-	IE-GV-------------------
	Sporosarcina sp. PTS2304	WP_114925623	------S-GL----SK-	IE-DV-------------------
	Sporosarcina ureae	WP_029053512	------S-GT-----Q-	IE-GV-------------------
	Staphylococcus aureus	WP_084978434	------T--S---L-S-	IQQGI----------Q---------
	Ureibacillus thermophilus	QBK25173	---------S--L-RL-	RN-GV-------------------
	Ureibacillus thermosphaericus	WP_016837311	---------S--V-RL-	RN-GV-------------------
	Vibrio vulnificus	WP_133350894	---------VS--L-LH	REAG-----L--------------
	Viridibacillus arvi	WP_053417950	--------GVS---ST-	VNQG--------------------
	Viridibacillus sp. OK051	WP_100794906	--------GVS---ST-	VNQG--------------------

Supplemental Figure 16

A partial sequence alignment of the bacillithiol biosynthesis deacetylase BshB2 protein containing a one amino acid insertion (boxed) that is exclusively shared by all members belonging to the *Lysinibacillus sensu stricto* clade and absent in all other bacteria. *Lysinibacillus pakistanensis* was not in the NCBI non redundant protein sequences database at the time of writing this paper.

			225		285
Lysinibacillus sensu stricto (10/10)	Lysinibacillus boronitolerans	WP_036077775	LQDERKTKVEITDEDFEDVQEVDLKLVRLAKK	MG	NDTQIVTNDFNLNKVCELHHVKVLNIN
	Lysinibacillus fusiformis	WP_025116463	-------N------------------------	--	---------------------------
	Lysinibacillus macroides	WP_053997207	-----Q-Q-------------------K----	--	---------------------------
	Lysinibacillus parviboronicapiens	WP_107925067	-----M------E-------------------	--	-------------------Q-R-----
	Lysinibacillus varians	WP_025220851	-----M------E-------------------	-D	-------------------Q-------
	Lysinibacillus xylanilyticus	WP_100542793	-----M---Q--E-------------------	--	H------------------N-------
	Lysinibacillus tabacifolii	TKI45480	-----M------E-------------------	-D	-------------------Q-------
	Lysinibacillus mangiferihumi	TKI65133	-----M------E-------------------	-D	-------------------Q-------
	Lysinibacillus sphaericus	WP_010860633	-----M------E-------------------	R-	-------------------Q-------
	Bacillus sp. B14905	WP_008180605	-------N------------------------	--	---------------------------
**Other Bacteria (0/>300)**	Kurthia huakuii	WP_029500620	----PA-T-LL-EV--D--P------------	M	GGG----------------N-Q-----
	Kurthia sp. 3B1D	WP_126991991	---DPL-T-LL-EV--D--P------------	M	GGA----------------N-Q-----
	Kurthia massiliensis	WP_010290455	----PSLN-ML-EI--D--P------------		MGG----------------N-R-----
	Kurthia senegalensis	WP_010308078	--K-YASQ-LL-EV-----P------------		M-G-V--------------K-------
	Kurthia sibirica	WP_109305704	---DHTSN-I--EV--D-IL------------		MGG--L----------D--K-------
	Kurthia zopfii	WP_109349807	---APTSN-L--EV----IP------------		MNG--L----------D--K-H-----
	Lysinibacillus acetophenoni	WP_097149294	-----A---I--E------H------------		M-A--L-------------K-Q-----
	Lysinibacillus chungkukjangi	WP_107932247	-----V-T-Q-SE-------------------		MGA--L----------D----Q-----
	Lysinibacillus composti	WP_124766130	-----L---S-SE-------------------		MGA--L----------D--N-Q-----
	Lysinibacillus endophyticus	WP_121214161	-----A-T-Q-SEV----IN------------		M-A--L---------------Q-----
	Lysinibacillus halotolerans	WP_122971244	-----A-T-Q-SE-------------------		MGA--L---------------E-----
	Lysinibacillus jejuensis	WP_108305958	---DKTS--V---------A--------Y---		HHA-V-----------D--Q-D-----
	Lysinibacillus manganicus	WP_036183232	-----A---D--E------H------------		MSA--L-------------N-Q-----
	Lysinibacillus massiliensis	WP_036172381	-----A-N-I-SE-----IH------------		MGA--L----------D----P-----
	Lysinibacillus meyeri	WP_107839087	-----ASQ-L-V-D-----H-----------Q		RNA--L-------------S-Q-----
	Lysinibacillus odysseyi	WP_036152020	-----QS--L-VE---D-IA-----------Q		M-A--L-------------Q-------
	Lysinibacillus saudimassiliensis	CDZ99402	-K-DKTSN-V--E------A--------Y---		YSA-V-----------D--Q-D-----
	Lysinibacillus sinduriensis	WP_036200791	-----V-T-Q-SE-------------------		IGA--L----------D----Q-----
	Lysinibacillus sp. 2017	WP_108714318	-----AS--L--EI-----A------------		M-A--L-------------R-Q-----
	Lysinibacillus sp. BF-4	WP_036143312	---DKTSN-V---------A--------Y---		YSA-V-----------D--Q-D-----
	Lysinibacillus telephonicus	WP_126292346	-----A-T-Q-SE-----I-------------		MGA--L---------------Q-----
	Lysinibacillus xyleni	WP_097073425	-----A-T-Q-SEV-----N------------		M-A--L---------------Q-----
	Acidibacillus ferrooxidans	WP_067561503	V-K-L-V---VLET-----A---S---K---Q		L-GKV-------------QG-S-----
	Acidibacillus sulfuroxidans	WP_109429578	I-K-L-V--QVLEI---ELS---S---K---Q		LGGKV----------A--QG-S-----
	Aeribacillus pallidus	WP_063387133	I-K-LDI----WEG----I----S---K---I		IQGVV-------------QN-S-----
	Alicyclobacillus pomorum	WP_026964729	I-K-L-V--QVHEV--D-I----S-------Q		MRGKV-------------QG-P-----
	Alicyclobacillus sacchari	WP_134160563	I-K-S-AT-Q-VEI--D-I----S-------Q		MHGKVM------------QG-P-----
	Alicyclobacillus vulcanalis	WP_076345567	I-K-S-VQ-Q-VET----I----A-------Q		MRGKV---------I---QG-Q-----
	Alteribacillus iranensis	WP_091663759	I-K-LPI----YEG----I----S---K---I		QNGMV-----------D-QG-D-----
	Ammoniphilus oxalaticus	WP_120190242	I-K-L-V--I-EER----I----S-------V		TSGKV-------------QG-P-----
	Aneurinibacillus aneurinilytic	WP_021623658	I-K-L-V--I---K---EIS---S---K---V		MKGKV-------------QG-S-----
	Aneurinibacillus migulanus	WP_043063773	I-K-L-V--I---K---EIS---S---K---L		MKGKV-------------QG-P-----
	Aneurinibacillus soli	WP_096463218	I-K-L-V--I---Q---EIS---S---K---V		MQGKV-------------QG-S-----
	Aneurinibacillus terranovensis	WP_027415304	I-K-LNV--I--EQ----IS---S---K---V		MKGKV-------------QG-S-----
	Aneurinibacillus tyrosinisolve	WP_047154529	I-K-L-V--I---Q----IS---S---K---V		MKGKV-------------QG-S-----
	Anoxybacillus amylolyticus	WP_066322113	I-K-LEM----YEG--D-I----S---K---L		TSGVV-------------QN-R-----
	Anoxybacillus tepidamans	WP_027410211	I-K-LEM----YEG--D-I----S---K---L		TSGVV-------------QN-R-----
	Anoxybacillus vitaminiphilus	WP_111646095	I-K-LDI----YEG----I----S---K---L		ISGVV-------------QN-P-----
	Anoxybacter fermentans	WP_127018058	I-KDMNV----V-A--D--D---S---K---M		M-GK-I---------A---G-P-----
	Bacillus alkalitelluris	WP_088073093	I-K-LPVN---YEG----I----S---K---L		L-GYV-----------D-QG-S-----
	Bacillus altitudinis	WP_073415646	I-K-LDI----YEG----I----S---K---L		TSGVV-------------QK-A-----
	Bacillus andreraoultii	WP_033827315	I-K-LPI----YEG----I----S---K---T		-G- V----Y--------QQ-H-----
	Bacillus atrophaeus	WP_061570389	I-K-LDI----YEG----I----S---K---L		TSGVV-------------QK-A-----
	Bacillus australimaris	WP_060697858	I-K-LDI----YEG----I----S---K---L		TSGVV-------------QK-A-----
	Bacillus badius	WP_063384352	I-K-IPVE---IER---------S---K---E		MNGIV-------------QK-Q-----
	Bacillus bogoriensis	WP_026673470	I-K-LPI----YEG---EI----S---K---L		VSGIV-------------QG-S-----
	Bacillus butanolivorans	WP_053349086	I-K-LPI---MYEG----I----S---K---I		SGGMV------------FQN-A-----
	Bacillus caseinilyticus	WP_090892754	I-K-LPVN---YEG----I----S---K---L		L-GYV-----------D-QG-S-----
	Bacillus cecembensis	WP_057986361	-----AS--L--EI--D--A------------		M-A--L-------------S-Q-----
	Bacillus glycinifermentans	WP_048355826	I-K-LDI----YEG----I----S---K---L		TSGVV-------------QK-A-----
	Bacillus gottheilii	WP_080849056	I-K-LSI----YEG---EI----S---K---I		SNGVV-------------QQ-A-----
	Bacillus halotolerans	WP_044153446	I-K-LDI----YEG----I----S---K---L		TSGVV-------------QK-A-----
	Bacillus indicus	WP_029282881	I-K-LSI----YEG---EI----S---K---L		TSGVV-------------QG-G-----
	Bacillus jeotgali	WP_102264839	I-K-L-I----YEG--D-I----S---K---L		TNGVV-------------QK-A-----
	Bacillus koreensis	WP_053403255	IRK-LPVR---YEG----I----S---K---I		TSGVV-------------QN-------
	Bacillus kwashiorkori	WP_062349756	I-K-LSI----YEG----I----S---K---L		TNGIV----Y--------QK-Q-----
	Bacillus marinisedimentorum	WP_070121448	I-K-LPVN---YEG---------S---K---L		LSGVV-------------QK-A-----
	Bacillus massiliglaciei	WP_110925979	I-K-LAV---MYEG----I----S---K---I		SGGMV------------FQN-A-----
	Bacillus massilionigeriensis	WP_075980291	I-K-LSI----YEG----I----S---K---I		TNGIV-------------QQ-S-----
	Bacillus mediterraneensis	WP_071458918	I-K-IDI----YEG----I----S---K---L		SGGIV-------------QG-S-----
	Bacillus megaterium	WP_116077820	I-K-LAM----YEG----I----S---K---L		TSGLV-------------QN-G-----
	Bacillus mesonae	WP_127484390	I-K-LAI----YEG---EI----S---K---L		TSGIL-------------QN-A-----
	Bacillus methanolicus	WP_003348663	I-K-LAI----YEG---EI----S---K---I		TNGVV-------------QN-S-----
	Bacillus mojavensis	WP_010332752	I-K-LDI----YEG----I----S---K---L		TSGVV-------------QK-A-----

Other Bacteria (0/>300)

Bacillus muralis	WP_057915193	I-KDLPI---MYEG----I----S---K---I	SGGMV------------FQN-A-----
Bacillus ndiopicus	WP_042478567	-----ASQ-L-V-D-----------------Q	RNA--L-------------N-Q-----
Bacillus nealsonii	WP_101176287	I-K-LAI----YEG----I----S---K---L	TNGVV-------------QK-S-----
Bacillus notoginsengisoli	WP_118923772	I-K-IAVN---SET----I----S---K----	TGGIL----------S-FQN-S-----
Bacillus panaciterrae	WP_028400832	I-K-LAI----YEG----I----S---K---V	MNGAV-------------QG-------
Bacillus selenatarsenatis	WP_041966545	I-K-L-I----YEG--D-I----S---K---L	TNGVV-------------QK-A-----
Bacillus simplex	WP_096340940	I-KDLPI---MYEG----I----S---K---I	SGGMV------------FQN-A-----
Bacillus sonorensis	WP_006640294	I-K-LDI----YEG----I----S---K---L	TSGVV-------------QK-A-----
Bacillus stratosphericus	WP_103132748	I-K-LDI----YEG----I----S---K---L	TSGVV-------------QK-A-----
Bacillus subterraneus	WP_125482159	I-K-L-I----YEG--D-I----S---K---L	TNGVV-------------QK-A-----
Bacillus subtilis	AAA21793	I-K-LDIE---YEG----I----S---K---L	TSGVV-------------QK-A-----
Bacillus swezeyi	WP_076762608	I-K-LDI----YEG----I----S---K---L	TSGVV-------------QK-A-----
Bacillus thermoamylovorans	WP_041903701	I-K-LPI----YEG----I----S---K---T	-G- V-------------QQ-N-----
Bacillus trypoxylicola	WP_061947733	I-K-LPV----YEG---EI----S---K---L	TSGVV-------------QG-P-----
Bacillus tuaregi	WP_071396739	I-K-LAI----YEG---EI----S---K---L	TNGIV-------------QQ-S-----
Bacillus xiamenensis	WP_008356484	I-K-LDI----YEG----I----S---K---L	TSGVV-------------QK-A-----
Bacillus zeae	WP_119112268	I-K-LEI----YEG---EI----S---K---L	SSGIV-------------QG-S-----
Brevibacillus borstelensis	WP_003392038	I-K-M-V--Q-YEG---E-S---S--IK---V	MNGKV-------------QG-A-----
Brevibacillus choshinensis	WP_055746186	I-K-M-V--Q-WEG---E-S---S--IK---V	MNGKV-------------QG-S-----
Brevibacillus sp. SCSIO 07484	WP_126424753	I-K-L-V--Q-YEG--D------S--IK---V	LNGKV-------------QG-P-----
Butyricicoccus sp. 1XD8-22	RKJ70995	-I-SE-----IH------------	MGA--L----------D----P-----
Caenibacillus caldisaponilytic	WP_077615908	I-K-LAV----YEG--D--S---S---K--QL	TGGVV-------------QG-------
Caldalkalibacillus thermarum	WP_007502769	I-K-KTV--H-YEG----IH---S--I----V	LNGKV-------------QG-P-----
Caldanaerovirga acetigignens	WP_073257541	M-K-MNIE-Q-CEK----IA---S---K--QM	L-GK-I---------A---K-P-----
Caldibacillus debilis	WP_020155364	I-KDLPI----YEGN-D------S--IK----	TNGTV----Y--------QN-------
Caryophanon latum	WP_066462878	----Y-SG-M---R--D--A-----------E	LNG------------S---Q-H-----
Caryophanon tenue	WP_066542777	-------E-I--ER---------M-------E	MGGK-----------S---N-H-----
Centipeda periodontii	WP_040394623	IRK-SRM---V-EA----IA---S--LQ--QE	VGGK-I---------AQ-RG-P-----
Chlamydia abortus	SHE14368	I-K-LEV--L-YEG----IS---S-------L	LRGKVI------------QG-S-----
Clostridiales bacterium PH28_b	KKM12740	IRK-LDVM-K-YEQ----LV---S------Q-	L-A-VI---Y-----A--QG-------
Desulfuribacillus stibiiarsena	WP_069701351	I-K-LQV----WEG---ETS---A---K---L	I-G-V-------------QG-S-----
Domibacillus antri	WP_075397879	M-K-ISVD--M-EK-Y--IH---S---K---E	IGGIV------------FQQ-P-----
Domibacillus epiphyticus	WP_076764358	M-K-IPIE--M-EK-Y--IH---S---K---E	MGGIV------------FQQ-P-----
Domibacillus indicus	WP_046176526	--K-VPIN--M-EK-Y--IH---S---K---E	IGGIV------------FQK-Q-----
Domibacillus robiginosus	WP_050182416	--KDVPID--M-EK-Y-------S---K---E	TGGIV------------FQK-Q-----
Edaphobacillus lindanitolerans	WP_076758768	I-E----GAV------D--P---M--------	-GGVV----------------P-----
Effusibacillus lacus	WP_096181040	I-K-L-I--N-L-I--DEI----S--I----Q	MNGKV-------------QG-E-----
Effusibacillus pohliae	WP_018131254	I-K-L-I--NVMEI----I----S--I----Q	LNGKV-------------QG-P-----
Escherichia coli	OTE83120	--K-EGIS--MY-G----IS---S--IK---L	L-GV-----Y-----S-FQN-P-----
Exiguobacterium acetylicum	WP_029343077	---IPGVE---Y-G-----P---I--IK--EL	VKGIV----Y-------VR--P-----
Firmicutes bacterium	REJ36841	M-K-S-VA---L-R--D-ITD--A------RR	L-AKVL---------A--QG-E-----
Fusibacter sp. A1	WP_129489508	I-K-LDVE-V--SQ-----N---I--LK--QM	L-GAV----------A-I-G-------
Geobacillus icigianus	WP_033021062	I-K-MAM----HEA--N------S---K---Q	LQGVV-------------QN-R-----
Geobacillus jurassicus	WP_066234050	I-K-MAM----HEA--S------S---K---Q	LQGVV-------------QN-R-----
Geobacillus stearothermophilus	WP_033017264	I-K-MAM----HEA--S------S---K---Q	LQGVV-------------QN-R-----
Geobacillus thermocatenulatus	WP_025950550	I-K-MAM----HEA--S------S---K---Q	LQGVV-------------QN-R-----
Geobacillus thermodenitrifican	WP_011886622	I-K-MAM----HEA--S------S---K---Q	LQGVV-------------QN-R-----
Geobacillus vulcani	WP_031406249	I-K-MAM----YEA--S------S---K---Q	LQGVV-------------QN-R-----
Geosporobacter ferrireducens	WP_069978626	I-K-LQVE-Q---K-----A---I--LK--QF	MGGKV----Y----IA--QG-P-----
Geosporobacter subterraneus	WP_110941071	I-K-LQVE-Q---K-----A---I--LK--QF	MSGKV----Y----IA--QG-P-----
Gorillibacterium timonense	WP_058300170	I-K-LDV----YEG---EIS---S-------V	LGGKVL------------QG-S-----
Halobacillus alkaliphilus	WP_089752364	--KDLPVN---YEG----I----S---K---V	MNGIV------------FQN-Q-----
Halobacillus halophilus	WP_014641396	--KDLPVN---YEG----I----S---K---V	MNGIV------------FQN-Q-----
Halolactibacillus alkaliphilus	WP_089801870	--K-IGVD--MY-G---EI----S---K---V	M-GIV------------FQN-P-----
Halolactibacillus halophilus	WP_089831709	--K-IDVD--MY-G---EI----S---K---V	M-GIV------------FQN-P-----
Inediibacterium massiliense	WP_053955604	I-K-LNME-Q---K-----A---I--LK--QF	M-GKV----Y-----A--QG-P-----
Kyrpidia spormannii	WP_100666529	I-K-ATV--Q-VET---------S--------	MQGKV-------------QG-P-----
Kyrpidia tusciae	WP_013074198	I-K-TSV--Q-VET---------S--------	MQGKV-------------QG-P-----
Listeria booriae	WP_036088170	--K-D-I---MYEG----IA---S---K---V	MQGKV----Y-------FQN-A-----
Listeria fleischmannii	WP_007477494	I-K-E-I---MYEG----IA---S---K---I	MNGTV----Y-------FQN-P-----
Listeria thailandensis	WP_122865633	I-K-ENIR--MYEG-----P---A---K---T	MNGTV----Y-------FQN-P-----
Marinococcus halophilus	WP_079475083	I-K-LPIE-----T----IH---S---K---V	RNGIV-----------D-QG-S-----
Marinococcus halotolerans	WP_022794441	I-K-LPIE-----T----IH---S---K---V	RNGIV-----------D-QG-S-----
Marinococcus luteus	WP_091616761	I-K-LPIE-----T----IH---S---K---V	RNGIV-----------D-QG-S-----
Novibacillus thermophilus	WP_077721179	I-K-L-V--L-YEG----IH---S---K---V	LSGKV----Y--------QN-P-----
Numidum massiliense	WP_054951023	I-K-L-V--L-YEG----I----S---K---V	LKGKV-------------QQ-P-----
Ornithinibacillus contaminans	WP_047981984	I-K-LPI----YEG----I----S--IK---V	I-GIV-----------D-QG-R-----
Paenibacillus cellulosilyticus	WP_110046837	I-K-LDVR-L-YEG---EIG---S---K---A	LGGKV-------------QG-S-----
Paenibacillus contaminans	WP_113036250	I-K-LEV--L-YEG---EIS---S-------V	LGGKVI------------QG-P-----
Paenibacillus curdlanolyticus	WP_006037975	I-K-LDVR-L-YEG---EIG---S---K---A	LGGKV-------------QG-S-----
Paenibacillus xylaniclasticus	WP_127571944	I-K-LDVR-L-YEG---EIG---S---K---A	LGGKV-------------QG-S-----
Paenisporosarcina antarctica	WP_134208108	--T--ASALL--E---DE--------M-----	MGGVV-----------A--Q-P-----
Paenisporosarcina indica	WP_075620216	--K--ASA-L----------------M-----	MEGMV--------------Q-P-----
Paenisporosarcina quisquiliaru	WP_090567716	--T--ASA-L-VE-----I-------------	MNGIV-----------D--Q-R-----

Other Bacteria (0/>300)

Parageobacillus caldoxylosilyt	WP_017434043	I-K-LDI----YEG--D-I----S---K---L	LSGVV-------------QN-R-----
Parageobacillus genomosp. 1	WP_043903297	I-K-LDI----YEG--D-I----S---K---L	ISGIV-------------QN-R-----
Paucisalibacillus globulus	WP_096271397	I-K-LPI----YEG----I----S--IK---V	I-GIV-----------D-QG-H-----
Pelagirhabdus alkalitolerans	WP_090796867	--K-IPVD--MY-G----I----S---K---V	S-GIV------------FQN-P-----
Planococcus antarcticus	WP_006828894	--S--VDAIM--E-N-DE-S------M-A---	MGG-V--------------N-P-----
Planococcus donghaensis	WP_065527585	--S--VDAIM--E-S-DE-S------M-A---	MGGKV--------------N-P-----
Planococcus halocryophilus	WP_008497404	--S--VDAIM--E-S-DE-S------M-A---	MGGKV--------------N-P-----
Planococcus halotolerans	WP_112224733	--S--EGAIM--E-S-DE-A------M-A---	MGG-V--------------N-P-----
Planococcus maitriensis	WP_112233949	--T--VGAIM--E---G-AA---M--M-A-Q-	MGGKV--------------N-P-----
Planococcus maritimus	WP_068489142	--T--VGAIM--E---G-AA---M--M-A-Q-	MGGKV--------------N-P-----
Planococcus massiliensis	WP_052654240	--S--EGAIM--E-S-DE-S------M-A---	MGG-V--------------N-P-----
Planococcus plakortidis	WP_068871298	--T--VGAIM--E---G-AA---M--M-A-Q-	MGGKV--------------N-P-----
Planococcus rifietoensis	WP_058382197	--T--VGAIM--E---G-AA---M--M-A-Q-	MGGKV--------------N-P-----
Planococcus salinarum	TAA67134	--S--EGAIM--E-S-DE-A------M-A---	MGG-V--------------N-P-----
Planococcus salinus	WP_123166217	--S--LDAIM--E-S-DE-S------M-A---	MGG-V--------------N-P-----
Planococcus sp. Y42	WP_077587615	-----TGS-V---L--D--P---M--M-A-Q-	MGG-V--------------N-P-----
Planococcus versutus	WP_049694516	--S--VDAIM--E-N-DE-S------M-A---	MGGKV--------------N-P-----
Planomicrobium flavidum	WP_088006727	-----AE-IT---L---------M--M-A-Q-	MGG-V--------------G-P-----
Planomicrobium glaciei	WP_036803829	--S--EGAIM--E-N-DE-S------M-A---	MGG-V--------------N-P-----
Planomicrobium okeanokoites	WP_117314089	--S--EGAIM--E-S-DE-A------M-A---	MGG-V--------------N-P-----
Planomicrobium soli	WP_106534502	--S--EGAIM--E-S-DE-S------M-A---	MGG-V--------------N-P-----
Planomicrobium sp. Y74	WP_121636472	--S--EGAIM--E-S-DE-A------M-A---	MGG-V--------------N-P-----
Psychrobacillus insolitus	WP_111440107	--T--ASA-L-VEQ----I-------------	MNGIV-----------D--Q-R-----
Psychrobacillus psychrodurans	WP_093495451	--T--ASA-L-VE-----I-------------	MNGIV-----------D--Q-R-----
Psychrobacillus psychrotoleran	WP_093537888	--T--SSA-L-VE-----I-------------	MNGIV-----------D--Q-R-----
Rhizophagus irregularis	PKC50859	-----A-N-I-SE-----IH------------	MGA--L----------D----P-----
Rummeliibacillus pycnus	WP_102693522	-----ASQ-L--EI----------------Q	MGG--L----------D--N-------
Rummeliibacillus stabekisii	WP_066790978	-----A-Q-L--EV------------------	MGG--L----------D--N-------
Salimicrobium flavidum	WP_076560482	--R-VPVN--MY-G----I----S---K---V	MNGIV------------FQG-Q-----
Salimicrobium halophilum	WP_093194627	--R-VPVN--MY-G----I----S---K---V	M-GIV------------FQG-Q-----
Salipaludibacillus aurantiacus	WP_093052736	I-K-LSV----YEG---EI----S---K---V	LGGFV-----------D-QG-D-----
Sediminibacillus halophilus	WP_074601221	M-K-LPVN---YEG---EI----S---K---V	I-GVV------------FQ--Q-----
Selenomonas artemidis	WP_026762182	IRK-SRM-----EV----IA---S--LQ--QE	VGGK-I---------AQ-RG-P-----
Selenomonas bovis	WP_031587569	IRTDS-M-----N---D-IS---S-----GQ-	VGAK-I---------AK-RG-E-----
Selenomonas ruminantium	WP_074814227	IRQ-SRM--K--E-----IP---S---Q--QQ	VGGK-I---------AQ-RG-E-----
Shimazuella kribbensis	WP_028777667	I-K-LDV--M-YEG----I----S---K---V	LSGKV-------------QG-------
Solibacillus isronensis	WP_079523511	-----AS--L--EV-----S-----------N	MSA--L-------------R-Q-----
Solibacillus sp. R5-41	WP_099425624	-----AS--L--EI-----A------------	M-A--L-------------R-Q-----
Sporosarcina koreensis	WP_060203367	--TDDGPSIL------A--A-----------S	MNG-V----------AD--G-A-----
Sporosarcina newyorkensis	WP_009499082	--NDDGPT-L------N--A-------K----	MEGLV----------SD--G-A-----
Sporosarcina psychrophila	WP_067204683	M-TDDGPNIL------AG-P---M---H----	MGGLV----------AD--G-S-----
Sporosarcina ureae	WP_083065581	--NDDGPQ-L-----IPN-A-------K----	M-GLV----------SD--G-A-----
Thalassobacillus devorans	WP_028781689	--KDLPVN---YEG---EI----S---K---V	M-GVV------------FQN-A-----
Thalassobacillus sp. TM-1	WP_062438207	--KDLPVN---YEG---EI----S---K---V	M-GVV------------FQN-A-----
Thermanaeromonas toyohensis	WP_084662938	IRK-LGVN-K-YE-----IT---T------Q-	LKAP-L---Y-----A---G-------
Thermoflavimicrobium dichotomi	WP_093229923	I-K-LNV--L-YEG-----S---S---K---V	LSGKV-------------QG-Q-----
Thermoflavimicrobium sp. FBKL4	WP_113659530	I-K-LDV--L-YEG-----S---S---K---V	LSGKV-------------QG-Q-----
Thermosediminibacter oceani	WP_013276841	M-K-M-ID-K-YEG----IS---S---K--QI	L-GK-I---------A---N-P-----
Tumebacillus algifaecis	WP_094238174	I-K-L-I--QVMEI---EI----S-------Q	INGKV-------------QG-G-----
Tumebacillus avium	WP_087456428	I-K-L-I--QVMEI---EI----S-------Q	INGKV-------------QG-G-----
Tumebacillus flagellatus	WP_038092191	I-K-L-I--QVMEI--D-I----S-------N	ISGKV-------------QG-A-----
Tumebacillus permanentifrigori	WP_109691003	I-K-L-I--QVLEI--D-I----S-------N	IGGKV-------------QG-L-----
Tumebacillus sp. BK434	WP_132946765	I-K-L-I--QVMEI---EI----S-------Q	INGKV-------------QG-G-----
Ureibacillus thermophilus	QBK27097	---GKSV--M-SE---D---------------	MGAK-L---------------Q-----
Ureibacillus thermosphaericus	WP_016838530	---GKTVN-I-SE---D---------------	MGAK-L----------D----Q-----
Vagococcus humatus	WP_125943672	--K-SSVQ--MYEG----IA---S--IK---L	L-GVV------------FQN-P-----
Vagococcus sp. SS1995	WP_125983383	--K-EGIS--MYEG----I----S--IK---L	L-GIV------------FQN-P-----
Virgibacillus halodenitrifican	CDQ37356	I-K-LPV----YEG---EI----S--IK---V	MNGIV-----------D-QG-A-----
Viridibacillus arvi	WP_053418522	-----A-DIL--EV--D-I-------------	MSG--L----------D--N-A-----
Viridibacillus sp. OK051	WP_100795254	-----SSDIL--EV--D-I-------------	MSG--L----------D--N-S-----

Supplemental Figure 17

A partial sequence alignment of the PIN/TRAM domain-containing protein containing a two amino acid insertion (boxed) that is exclusively shared by all members belonging to the *Lysinibacillus sensu stricto* clade and absent in all other bacteria. *Lysinibacillus pakistanensis* was not in the NCBI non redundant protein sequences database at the time of writing this paper and *Lysinibacillus contaminans* did not have a homolog of this protein.

			205		254
Lysinibacillus sensu stricto **(11/11)**	Lysinibacillus boronitolerans	WP_016994577	ELAEMFPIDVPFMIFVNEDLD	N	ETDCYIETNHGRIMVSIDEQLNALRLKL
	Lysinibacillus contaminans	WP_053584341	-------T-----V------E	-	D---F--------V--------E-----
	Lysinibacillus fusiformis	WP_004269300	---------------------	-	----------------------------
	Lysinibacillus macroides	WP_053993923	-------V---L-------F-	-	D------------V--------E-----
	Lysinibacillus mangiferihumi	WP_107894686	-------T-----------FA	-	---------Y---V--------E--I--
	Lysinibacillus parviboronicapiens	WP_107947753	-------T------------E	-	----F--------V--------E-----
	Lysinibacillus tabacifolii	WP_108029520	-------T-----------FA	-	---------Y---V--------E--I--
	Lysinibacillus varians	WP_025218792	-------T-----------FA	-	---------Y---V--------E--I--
	Lysinibacillus xylanilyticus	WP_049668440	-------T----------E-E	-	----F--------V--------E-----
	Lysinibacillus sphaericus	WP_024362035	-------T-----------FA	-	---------Y---V--------E--I--
	Bacillus sp. B14905	EAZ83847	-------V------------N	-	-------------V--------E-----
**Other Bacteria (0/>300)**	Alteribacillus persepolensis	WP_091270374	--ESQL-HSKELIVLPDSQ--		DNQ-I---SY--VDA---S--QE-KT--
	Amphibacillus jilinensis	WP_083855749	Q-MSVLEP--SLT-YPSDK-N		DG--I---PYS-LD----Q---KI-TG-
	Anoxybacillus amylolyticus	WP_066323799	--KA--NEP-DLFLYPD-Q-Q		--G-I---PF---DA-V-T--QQIKEQ-
	Bacillus acanthi	WP_108669692	--LSL--KETNLY-YPDDE-T		-SN-I--SSN---DA-V-S--DEV-R--
	Bacillus acidiproducens	WP_018660060	--DAI--N-TKCY-YPD-S-H		PLQV----DS---DA---S--SE-KA--
	Bacillus aidingensis	WP_026701059	-MENLLAYSQEL---PDSS-N		-N--I---PY-KVDA-L-S--QE-KEQ-
	Bacillus alkalitelluris	WP_078547133	D-KALINR-CDVFV-P-P---		-IG-F--SSY-K-EA-V-S--EEIKS--
	Bacillus aquimaris	WP_113968223	--EV---T--QCF-YA-D--E		PEE------Q--VI--V-S--RE-KQ--
	Bacillus aryabhattai	WP_033579779	--FDLLNGETGLF-YAD-N-Q		-M--V-DSSY--LVA-F-S--TEMKK--
	Bacillus asahii	WP_119116563	--EAI--KNTDLYVYP-DE-A		-SS-I--SES---DA-V-S--REMKD--
	Bacillus badius	WP_082028220	-IDA---V-TLCY-YP--K-E		-GG-----S----EIGL-S--QQ--KQ-
	Bacillus boroniphilus JCM 2173	GAE43648	--IAI--K-ID-Y-YPD-E-E		--S-I--SEN--VDA-V-S--EEIKN--
	Bacillus butanolivorans	WP_116821779	--ISI--K-TELY-YPDD--N		-SS-I--SEN---DA-V-S--QEIKM--
	Bacillus canaveralius	WP_101576619	--LAL--KETN-Y--PDD--S		-ES-L--SAN--MDA-V-S--LEVKQ--
	Bacillus cavernae	WP_126864468	--SAI-HKETR-Y-YPDQGIG		--S-I--ST----DAG--S--KEIK---
	Bacillus circulans	WP_082138199	--LQL---KPNLF--PD-TI-		-NA-I---AN--LDA-V-T--LMIKS--
	Bacillus coagulans	WP_110133960	--DAL--N-TKCYVYPDDS-E		PYQVF--SGS---DA---S--SE-KA--
	Bacillus coahuilensis	WP_010172474	D-KRS--PH-HIYFYP-DE-G		ADE--L-SKQ------V-S--EE--RQ-
	Bacillus dakarensis	WP_077211520	D-LKI--KETELY-YPDP--P		-QS-I--SAS---DA-V-Q--EEIKR--
	Bacillus deserti	WP_101639904	K-TAL--KNSELY-YPD-E-A		--S-V--SSN---DA-V-S--CEIQE--
	Bacillus enclensis	KSU62512	-IETV--AN-QCF-YA-D--E		DEQ------Q---I----S--SE-K---
	Bacillus farraginis	WP_083498338	--DAI--SNQQCYVYA-DE-S		-FQ-L---S---VDA-V-S--TE-KV--
	Bacillus fastidiosus	WP_082799822	--MSILTSETDLY-YA-DEVE		-NG----SPY---DA---T--FE-KKQ-
	Bacillus firmus	WP_048009026	--IRV--KETDLY-YPD-E-S		N-S-I--SAN---EA-V-S--EEIKR--
	Bacillus foraminis	WP_121609263	--EAV--R-CLLF-YPEDE-S		--S-I---GN---DA-V-S--EEIKQR-
	Bacillus fortis	WP_120069667	-ESI--RE-QCY-YP--E-N		-N--VV-CETI--DTG--S--SE-KER-
	Bacillus freudenreichii	WP_126432444	-ESI--RE-L-Y-YPS-E-N		VH--V--CETT--DAG--S--TE-KGR-
	Bacillus ginsengihumi	WP_035352909	--DAI--N-TKLY-YPD---K		-FQ----SDY---DA---S--TE-KQ--
	Bacillus gobiensis	WP_082363743	--IHCL-HHCHLN-YADTEAS		KGT-----DF---DA---T---E-KA--
	Bacillus gottheilii	WP_080846448	--MAV--KETKLY--P-DE-A		--S-V--SDS---DAAV-S---EIKV--
	Bacillus horneckiae	WP_066393583	--LML--KEID-Y-YP-D--A		--G-F--SAS---DA-V-S--EEIKQ-I
	Bacillus humi	WP_057996908	--TAI-GRETDLFVYP----G		-D--V--SSF-K-DA-V-T--AEIKE--
	Bacillus jeotgali	WP_102262191	--IAI--K-ID-Y-YPDDE-E		--S-I--SEN--VDA-V-S--EEIKN--
	Bacillus korlensis	WP_066053259	D-LRI--KETELY--PDSE-T		DQS-I--SAN---DA-V-Q--DEIKR--
	Bacillus kribbensis	WP_026692491	-TSL--RETDLY-YPD-E-A		V-G-I--SSN---DA-V-S--QEIKS--
	Bacillus lentus	WP_082788627	--DAI--NNITCF-YPDT--E		-HA-F--SDN---DA--SS--VE-KES-
	Bacillus loiseleuriae	WP_082190981	--EA--TKETALI-YPDPE-S		-DS-L--SAN---DA---S--REIKD--
	Bacillus marisflavi	WP_121619556	--ESI--AV-QCY-YP-DE-Q		P------S-Q--V---V-S--KE-KV--
	Bacillus massiliglaciei	WP_110929414	--LAI--RETELY-YPD-EMS		--G----SDK---DA-V-T--EEIKQ--
	Bacillus massilioanorexius	WP_081591227	--LSI--KETE-F--PDDN-T		P-S-M---PS---DA---S--YEVKE--
	Bacillus massiliogabonensis	WP_102271460	-IMAL--K-ID-Y-YPDD--F		--S-L--SEN---DA---S--EEIKQ--
	Bacillus massiliogorillae	WP_081707642	--LGI--KETD-Y--PDDEIK		--S-I--SAN---DA---S---E-KQ--
	Bacillus massilionigeriensis	WP_097677901	--VRI--KETDLY-YPDD--S		-LT-I--SSN---DA-V-G--VEIKE--
	Bacillus mediterraneensis	WP_071459443	--EAV--R-TD-F-YP--E-E		DSS-L--SAN---DATV-S--AEIKQ--
	Bacillus megaterium	WP_050689645	--FDLLNGETGLF-YAD-N-Q		-M--V-DSSY--LVA-F-S--TEMKK--
	Bacillus methanolicus	WP_003349044	--LSI--RETN-Y-YP----S		K-S-I--SAN---DA---S--EQVKK--
	Bacillus muralis	WP_083231991	--LAI--K-TELY--PDDE--		-NS-I--SEN---DA-V-S--EVIKT--
	Bacillus ndiopicus	WP_042471916	-------P---LLF------E		N-ES---------V--------E-----
	Bacillus nealsonii	WP_040342534	--LQL---KPNLF--PD-TIE		-NG-I---AN--LDA-V-T--LMIKN--
	Bacillus obstructivus	WP_079991271	--DAI--SNQQCYVYA-DE-S		-FQ-L---S---VDA-V-S--TE-KV--
	Bacillus oceanisediminis	WP_110065692	--IMV--KETDLY-YPD-E-S		Q-S-I--SAN---EA-L-S--EEIKRR-
	Bacillus oleivorans	WP_097159586	--KLIL-AEAEIY-YA-PE-N		S-G-----EG-KLDA-VEV--TQVKAH-
	Bacillus oleronius	WP_078110785	--DAI--SNQQCYVYA-DE-S		-FQ-L---S---VDA-V-S--TE-KV--
	Bacillus praedii	WP_057763207	-IIAL--K-IE-Y-YP-D--S		--S-L--SEN---DA---S--EEIKQ--
	Bacillus psychrosaccharolyticu	WP_040373615	--QAI--KEINVY-YPDS--E		VNS-I--SGN---DA-V-S--AEIKR--
	Bacillus selenatarsenatis	WP_052442228	--IAI--K-ID-Y-YPDD--E		--S-I--SEN--VDA-V-S--EEIKN--
	Bacillus simplex	WP_081092583	--IAI--R-TELY--PDDE-E		-SS-I--SEK---DA-V-S--EVIKT--
	Bacillus sinesaloumensis	WP_077617814	Q-TAILNGETDLF-YP--E-N		----V--SSF-K-NA-V-T--SEIKE--
	Bacillus smithii	AKP46864	--EAI--AV-ELYLYPD----		IN--I---I---LDA-V-S--FKI-QT-
	Bacillus solani	WP_056684167	-IMAL--K-ID-Y-YP-D--S		--S-L--SEN---DA---S--EEIKQ--
	Bacillus sporothermodurans	WP_084347731	--DALL-NNQQCYLYSDDQ-N		-YQ-L---S--K-DA---S--SE-K---
	Bacillus subterraneus	WP_125479119	--IAI--KEID-Y-YPDD--P		--A-L--SEN---DATV-S--EEIKQ--
	Bacillus terrae	WP_120115172	-KSV--RE-QCY-YP--E-N		-N--IV-CETI--NAG--S--TE-KER-

Other Bacteria (0/>300)

Bacillus testis	WP_050614775	--LAI--KETNLVLYPDDE-N	--G-I--SPA-L-DAT--S--AEVKN--
Bacillus timonensis	WP_026021677	--TAILNRESDLF-YP----K	-A--V--SSF-K-NA-V-T--TEIKER-
Bacillus velezensis	WP_129193145	--IRILQK-THLA-YSD-KAP	KGT--V--SF-KVEA-V-T---Q-KE--
Bacillus wudalianchiensis	WP_077247410	-IDA-----TLCY-YP--K-E	-DG-----S----EIGL-S--QQ--KQ-
Bacillus zeae	WP_119111563	--EAV--RETD-Y-YP-D--P	-GS-V--SAN---DA-V-S--AEIKQ--
Caryophanon tenue	WP_066543378	-------P----L-------Q	S-ES---------V----T--QQ--KA-
Domibacillus aminovorans	WP_063964932	-IEAL--GG-NCY--P--EAK	-EA-FV--TS---DA---A--QQ--KT-
Domibacillus antri	WP_075398134	-IEAL--G--NCY--P--EAA	-NA-FV--AS---DA---T--QQ--KT-
Domibacillus enclensis	WP_082084563	-IEAL--SEINCY-YP--EA-	-DA-F---AN---DTA--T--QQ--KT-
Domibacillus epiphyticus	WP_083711203	-IEAL--G-INCY--P--EA-	-EA-FV--AS---DA---A--QQ--KT-
Domibacillus indicus	WP_082087901	-IEAL--A-INCY-YPS-EAG	-NT-F---AN---DAG--T--QQ--KT-
Domibacillus iocasae	WP_069939401	-IEAL--SEINCY--P--EA-	-NT-F---SN---DAG--T--HQ--KT-
Domibacillus mangrovi	WP_073711016	-IEAL--GG-NCY--P--EAE	-EA-F---AS---DA-L-A--QQ--KT-
Domibacillus robiginosus	WP_082220302	-IEAL--NEINCY--P--EAG	-DA-F---SN---DAGV-T---Q--KT-
Domibacillus tundrae	WP_046179171	-IEAL--SE-NCY--P--EAY	-NT-F---SN---DAG--T--HQ--KT-
Falsibacillus pallidus	WP_114743682	D-ETL--F--NCY-YPD---P	-NS----SQN--VDAG--S--IEIKRQ-
Falsibacillus sp. GY 10110	WP_121680272	--ESV--KNTQVF-YPD---G	-MN-F--SPN--VDA---S--IE-KGR-
Filibacter sp. TB-66	WP_124070925	---AI--P----L--A---F-	S-Q-F--------V--------V--ER-
Filobacillus milosensis	WP_134339287	--E-L--NKGVLT--P-K--S	-NS-L--SPF-M-DS---T--HQ-KIH-
Geobacillus sp. 44B	WP_081159529	--KSL--HE-DLF-HPDDE-Q	-HG-LV--PF---DA-V-T--AQIKE--
Gracilibacillus boraciitoleran	WP_035721435	--NSLIQS-IE---YPST--I	GEQIV---PF---DA-V-S--QEI-SR-
Gracilibacillus kekensis	WP_073201877	--SSILHNQME---YPSA--K	-EQVL---PF---DA-L-T--EEI-TR-
Jeotgalibacillus alimentarius	WP_052474049	--LAI--T--KLY-YPE-E-E	PYK-F---AN---DI---T--TE-KTG-
Jeotgalibacillus campisalis	WP_084215536	--LAI--V--QLS-YPEDE-E	PYQ-----TQ---DL---T--TQ-K---
Jeotgalibacillus malaysiensis	WP_052268637	--LAI--T--RLY-YPE-E-E	PYQ-F---AN---DI---T--TE-KAG-
Jeotgalibacillus proteolyticus	WP_104055968	--FGL--PEIQLYLYPE-E-E	PYQGF---KN---DI-L-T--DEMKQ--
Jeotgalibacillus salarius	WP_134379472	--LAI--T--KLY-YPE-E-E	PYK-F---GN---DI---T--SE-KAG-
Jeotgalibacillus soli	WP_041089050	--RAI--V--QLY-YPE-E-A	PYQ-F--S-Q---D--V-A--TQMKR--
Jeotgalibacillus sp. R-1-5s-1	WP_134371837	--LSI--V--KLY-YPE-E-E	PFQ-F--SSQ---DI---T--TE-KAG-
Kurthia huakuii	WP_029498236	--VA---PNM-----ID---S	NQE---D-----LIA-------E--HH-
Kurthia massiliensis	WP_010286419	--VA---P-M-----IDDT-S	D-ES--D-----L-A-V-A---E--YH-
Kurthia senegalensis	WP_010301534	--VA---P-M-----IDDV-G	DQE---D-----LVAT------E--KH-
Kurthia sp. 3B1D	WP_126990761	--VA---V-M-----IDDT-S	IQE---D-----LIA-------E--HH-
Kurthia sp. Dielmo	WP_020189442	--VA---A-M-----IDDT-S	IQE---D-----LIA-------E--HH-
Kurthia zopfii	WP_109350586	--ETLL-P-LL-T--IDGS-N	KL-----S-T--M---V-A---E-KKT-
Lysinibacillus acetophenoni	WP_097148007	--V-L--T----F-----EFN	D------------VI-------E-----
Lysinibacillus chungkukjangi	WP_107934003	--V----T---LLV--D-E-N	--E----------V--V----SE--I--
Lysinibacillus composti	WP_124764011	--V----T----L-------N	D-ES---------V----S---E-----
Lysinibacillus endophyticus	WP_121212978	--V--------LL-------S	S-ES---------VI------HE-----
Lysinibacillus fluoroglycofeni	WP_107942285	-------P---LLF------E	N-ES---------V--------E-----
Lysinibacillus halotolerans	WP_122970357	--VA---T----L---D---N	D--G---------V--------V--I--
Lysinibacillus jejuensis	WP_108305796	-------P---LL---I-EFE	D-E----------V-T-----Q---N--
Lysinibacillus manganicus	WP_036181580	-------T-I--F-------N	D--------R---V----D---E-----
Lysinibacillus massiliensis	WP_036171651	--V-I--T----F---L-E-A	D-E----------V-------HE--M--
Lysinibacillus meyeri	WP_107837882	-------P---LL-------E	N-ES---------VI------HE-----
Lysinibacillus odysseyi	WP_036152257	-------P----L-------A	NAES----S----V----T---E-----
Lysinibacillus saudimassiliensis	CEA04454	----I--P---LLL--I-EFE	D-E----------V-T-----SE--S--
Lysinibacillus sinduriensis	WP_036202977	--V----T---LLV--D---N	Q-ES---------V-------SE-----
Lysinibacillus sp. 2017	WP_108711899	-------AN-----------N	D-ES---------V-------KE--QQ-
Lysinibacillus sp. BF-4	WP_036141386	----I--P---LLL--I-E-G	D-E----------V-T-----SE--S--
Lysinibacillus sp. Marseille-P	WP_106784193	--M-I--P----I----DE-N	D------------V--------E-----
Lysinibacillus sp. SYSU K30002	WP_126657554	-------A---LL------FT	S-ES---------V--------E-----
Lysinibacillus sp. YLB-03	WP_118874659	--V-I--T---LF---DDE-M	D-Q----------VL-V-Q--SE-----
Lysinibacillus sphaericus	WP_125102062	---SI--P---ML----DEFE	S-E----------V--V-----EMKER-
Lysinibacillus telephonicus	WP_126292957	-------T---LL-------T	D-ES---------V-------HE-----
Lysinibacillus xyleni	WP_097072136	--V----T---LL-------N	SSES---------V--------E-----
Mycobacteroides abscessus subs	SHR14962	D-IRI--KETELY--PDSE-T	-QS-I--SAN---DA-V-Q--DEIKR--
Oceanobacillus iheyensis	WP_011065957	--KR-LGK-SKLA-Y-----K	----L--HPF-Q-EAN--S--TKI-EA-
Oceanobacillus kimchii	WP_017796670	--QRIIGK-SKLA-Y-----K	----L--HPF-Q-EAN--S--TKI-EA-
Paenibacillus sp. FSL R5-0490	WP_083675265	--IRV--KETDLY-YPD-E-S	N-S-I--SAN---EA-V-S--EEIKR--
Paenisporosarcina quisquiliaru	WP_090562585	--SSI--V--------D--MN	-------------V------VQE--R--
Paenisporosarcina sp. K2R23-3	WP_119882980	D-SAI--VN-----------Q	DKQS---------VIG--A---E-----
Parageobacillus caldoxylosilyt	WP_026078624	--KSL--HE-DLF-HPDDE-Q	-HG-LV--PF---DA-V-T--AQIKE--
Parageobacillus genomosp. 1	WP_043904113	--ESL--HE-DLF-HPDDE-E	-YG-L---PF---DA-V-T--AQIKE--
Planomicrobium flavidum	WP_088005924	--VSLL-A-I-LI--AEDG-E	--AGF----F---DF------SG--T--
Psychrobacillus insolitus	WP_111438484	---SI--V--------D--IQ	A------------V-G-----QE--I--
Psychrobacillus psychrodurans	WP_093494457	--SSI--V--------D--MN	-------------V------VQE--R--
Psychrobacillus psychrotoleran	WP_093535154	--SSI--V-----V--D--MN	-------------V------VQE--R--
Psychrobacillus sp. FJAT-21963	WP_056829271	---SI--V--------D--MN	D------------V-------QE-----
Psychrobacillus sp. OK028	WP_093060101	--SSI-----------D--MN	D------------V------VQE--S--
Psychrobacillus sp. OK032	SER53892	---SL--V--------D-EMN	A---F--------V-------QE--Q--
Quasibacillus thermotolerans	WP_083965742	--EA---LEILCY-YP--K-S	-NG-F---S----DI-L-S--EE--KQ-
Rummeliibacillus pycnus	WP_102692665	--SS---VG--L-------IG	D--S---------I----Q---E-----
Rummeliibacillus sp. POC4	WP_119414283	--S-----G---------EIG	D------------I----Q---E-----

**Other Bacteria (0/>300)**	Rummeliibacillus sp. TYF005	WP_124217250	--S-----G---------EIG	D-----------I----Q---E-----
	Rummeliibacillus stabekisii	WP_066785599	--KA---VG--V-V--D---A	D--S----------G--Q---E-----
	Solibacillus isronensis	WP_079525204	-------VN----V------L	DSES---------V-------QE--RQ-
	Solibacillus kalamii	WP_087615413	-------VN----V------L	DSES---------V-------QE--RQ-
	Solibacillus silvestris	WP_065216423	-------VN----V------L	DSES---------V-------QE--RQ-
	Solibacillus sp. R5-41	WP_099422780	-------VN-----------L	DDES---------V--L----QE--IQ-
	Sporolactobacillus sp. THM7-7	WP_130000396	-L-SAAQ-AKII-YADG--S	-N--M---AF---EA-V-A--SVIKE--
	Sporosarcina globispora	WP_053436476	--IRV--KETDLY-YPD-E-S	N-S-I--SAN---EA-V-S-IEEVKR--
	Sporosarcina koreensis	WP_082713655	---SI--P----L--A-D-F-	TNE-I----Q---V--V-----E-KE--
	Sporosarcina newyorkensis	WP_078817750	--SSI--PET--L------F-	A---F--------V--V-----E-KEQ-
	Sporosarcina pasteurii	SUJ00809	---AI--P-T--L--AT--F-	A-E----------V--------E--ER-
	Sporosarcina psychrophila	WP_082786635	---SV--PN---L--A-D-FE	SNE----------V--------E--E--
	Sporosarcina sp. BI001-red	WP_116016340	--GSI--P---MLL---DEFE	A-E----------V--------EMKER-
	Sporosarcina sp. D27	WP_025785049	---SI--P---ML----DEFE	S-E----------V--V-----EMKER-
	Sporosarcina sp. HY008	WP_067408151	---AI--P-I--L--A-D-FE	S-Q----------V--------E--E--
	Sporosarcina sp. P13	WP_099687118	---SI--PET--L------FS	----I--------V--V-----E-KEQ-
	Sporosarcina sp. P16b	WP_099671964	---SI--PET--L------FN	A---F--------V--V-----E-KVQ-
	Sporosarcina sp. P18a	WP_099675138	---SI--PET--L------FN	A---F--------V--V-----E-KEQ-
	Sporosarcina sp. P2	WP_099630915	---SI--PET--L------FN	A---F--------V--V-----E-KEQ-
	Sporosarcina sp. P20a	WP_099676639	---SI--PET--L------FN	A---F--------V--V-----E-KEQ-
	Sporosarcina sp. P26b	WP_099692668	---SI--PET--L------FN	A---F--------V--V-D---E-KEQ-
	Sporosarcina sp. P29	WP_099661541	---SI--PET--L------FN	A---F--------V--V-----E-KEQ-
	Sporosarcina sp. P3	WP_099638057	---SI--PET--L-----NFN	A---F--------V--------E-KEQ-
	Sporosarcina sp. P33	WP_081244360	---SI--PET--L------FN	A---F--------V--V-D---E-KEQ-
	Sporosarcina sp. P34	WP_099694150	---SI--PET--L------FN	A---F--------V--V-----E-KEQ-
	Sporosarcina sp. P37	ARK25768	---SI--PET--L------FN	A---F--------V--V-D---E-KEQ-
	Sporosarcina sp. P7	WP_099635944	---SI--PET--L------FN	A---F--------V--V-----E-KEQ-
	Sporosarcina sp. PTS2304	WP_114923321	---SI--PET--L------FS	A---F--------V--V-----E-KEQ-
	Sporosarcina sp. ZBG7A	WP_052245738	---SI--P---IL----DEFE	S-E----------V--V-----EMKER-
	Sporosarcina ureae	ARF15317	---SI--PET--L------FS	A---F--------V--V-----E-KEQ-
	Ureibacillus thermophilus	QBK25632	---V---P-I--C------M-	DL-A---------VL------KE-----
	Ureibacillus thermosphaericus	WP_096550658	---A---P----L-------N	D--S---------VL------KE-----
	Viridibacillus arenosi	WP_038184096	---SI--V----------E-N	D--GF--------V--------E-----
	Viridibacillus arvi	WP_053417470	---SI--V----------E-N	D--G---------V--------E-----
	Viridibacillus sp. FSL H7-0596	WP_076034014	---SI--V----------E-N	D--G---------V--------E-----
	Viridibacillus sp. FSL H8-0123	WP_076065422	---SI--V----------E-N	D--G---------V--------E-----
	Viridibacillus sp. OK051	WP_100796478	---SI--V----------E-N	D--G---------V--------E-----

Supplemental Figure 18

A partial sequence alignment of the flagellar assembly protein FliH containing a one amino acid insertion (boxed) that is exclusively shared by all members belonging to the *Lysinibacillus sensu stricto* clade and absent in all other bacteria. *Lysinibacillus pakistanensis* was not in the NCBI non redundant protein sequences database at the time of writing this paper.

Group	Species	Accession	412		467
Lysinibacillus sensu stricto (11/11)	Lysinibacillus xylanilyticus	WP_049668118	RLDGYLDESTEEAVKLYREDQKL	KKA	EDNSNMDTTFFNSLNETLKKYKEDRQNDLQ
	Lysinibacillus tabacifolii	WP_024362504	--------T-Q--------Q---	AQP	DNDMYV-S-------G---A----------
	Lysinibacillus boronitolerans	WP_016991518	-----M--T-A---N---KQK--	-L-	--EFY--------------DF-A-----Q-
	Lysinibacillus contaminans	WP_053585400	--------A---------KQK--	VS-	-GDVYV-----S-------V-----K--A-
	Lysinibacillus fusiformis	WP_069480119	-----M-DT-A---N---KQK--	-L-	--EFY--------------N--V-----Q-
	Lysinibacillus macroides	WP_053993652	-----M-DT-A---N---KQK--	EQ-	-GEFY--------------N--T---H-Q-
	Lysinibacillus mangiferihumi	WP_107895565	--------T-Q--------Q---	AQP	DNDMYV-S-------G---A----------
	Lysinibacillus parviboronicapiens	WP_107925296	--------A-D--------QK--	T-T	DNDVS---A----------N-----T----
	Lysinibacillus varians	WP_024362504	--------T-Q--------Q---	AQP	DNDMYV-S-------G---A----------
	Lysinibacillus sphaericus	WP_010858080	--------A-D-------KQK--	P-S	D-DVY---A----------N-----T----
	Bacillus sp. B14905	WP_008178788	-----M-DT-A---N---KQK--	-L-	--EFY--------------N--V-----Q-
Other Bacteria (0/>100)	Alkalicoccus saliphilus	WP_107584397	-T---F--A-----RSFQ--SD-		-ATGTITEETAD--Q-QVIEAVQEKE--V-
	Aneurinibacillus aneurinilytic	ERI04504	-----F-NR--M---TFQSLHQM		PVTGKV-KETAGAMQ-E-I-RIQ-PK----
	Aneurinibacillus migulanus	WP_043071515	-----F-NR--M---TFQSLHQ-		PVTGKV-KKTAGAMQ-E-I-RIQ-PK----
	Aneurinibacillus sp. XH2	WP_057900213	-M---F-AR--A---IFQNLHQ-		PMTGKV-KKTAGLMQ-E-I-RIR-PK----
	Aneurinibacillus thermoaerophi	WP_091261178	-M---F-AR--A---IFQNLHQ-		PMTGKV-KKTAGLMQ-E-I-RIR-PK----
	Bacillus cecembensis	WP_057984331	-K--FF-ME-AQ--SNF-KRYD-		Q-GRH--EQ--AK-T-Q-QT--DSQI--T-
	Bacillus cihuensis	WP_084552481	-V---Y--K--I--RAFQNTND-		TVSG-INSKTAAA-E-VVA-K-K-EK----
	Bacillus daliensis	SDO45986	-E---F--A-----MNFQ--EG-		-VTGEINEETADT-Q-RVIESVQ-KE----
	Bacillus glycinifermentans	WP_048354956	-Y---YS-G-KK--TAFQAQN--		KRSGTI-QKTA-TI-LRIEEK-L-EK----
	Bacillus gobiensis	WP_053604235	-M---FNK--KK--RSFQSKHG-		KETGVI-NRTAQRI--KINSL---EK----
	Bacillus kwashiorkori	WP_087941738	-T---FS-T--Y---AFQQEVN-		SS-GII-QVTA-AME-KV--MMA-VK--R-
	Bacillus ndiopicus	WP_042471190	-Q--FF-KE-A-V-QQF-KRH--		VAGEELNED--TA-Q-EALS---KPEH---
	Bacillus shacheensis	WP_082676301	-N---F--Q--Q---AFQQSVD-		--SGEI-QETAGR-Q-EILEHIR-SD----
	Bacillus sonorensis	WP_029419575	-Y---FN-G-KK--AAFQAQN--		KKSGVI-QKTA-T--LRIEEK-L-EK----
	Bacillus sp. FJAT-22090	WP_053590269	-K---F--D-MDG-EK--QERS-		-PS-DI-DAL-Q--TK--ME--TRKE--K-
	Bacillus sp. JCM 19047	GAF21746	-T---F--Q--T--REFQ--HD-		DETGQL-EATAQKIQ-EIQAHIR-VE--A-
	Bacillus sp. OxB-1	WP_041073586	---F----A---HAF-I-AEV		DAGDA--RV--TA-R-QVET-RK-IK--S-
	Bacillus sp. SA1-12	WP_046589191	-E---YSKE-AA---EFQ-ENE-		AISGKI-QQTA-T--QKI-E---NEK----
	Bacillus sp. T33-2	WP_101585188	-----FS----T---AFQKKKGM		-A-GVI-AKTAAA-EDAARDAMKQEK--I-
	Bacillus sp. V5-8f	WP_101635065	-E---F--K--I-I-AFQQQ---		KVTGEL-KETAEQ-EK-IIEKVQ-EA--I-
	Bacillus subtilis	WP_087991539	-E---FSKDMKK--MAFQ-QN--		VKTGVI--RTAE---QQIE-K-S-EK----
	Bacillus terrae	WP_120116055	-A---F-GQ--R--RAFQSIE-I		PVTGVI--RTAEK-E-AVI-EIQ-E-----
	Bacillus testis	WP_050616446	-T---F-VK--T--RAFQNGEH-		KNTGTINLKTAQA-EQ-IN-A-K-DE--M-
	Bhargavaea beijingensis	WP_092096112	-E---Y--T-LK--EQF-DHE--		NPGGA--DA--RA-RQKVID--S-EK--D-
	Bhargavaea cecembensis	WP_063179788	-E---Y--T-LK--ERF-DNE--		DPGGA--DA--RA-RAKVIE--A-EK--D-
	Bhargavaea ginsengi	WP_092054224	-E---Y--T-LK--ERF-DNE--		-PGGA--DA--RA-RAKVIE--S-EK--D-
	Brevibacillus agri	WP_005829891	-D---F--K--Q-I-QFQTAK--		PVTGKV-AATRSQ-E-S-RETMRKPE----
	Brevibacillus brevis	WP_017252239	-E---F--K----I-QFQTSS--		-VTGKV-AKTRS--E-S-R-TMTKPE----
	Brevibacillus formosus	WP_088909666	-E---F--K----I-QFQTSN--		-VTGKV-AKTRS--E-S-R-TMTKPE----
	Brevibacillus gelatini	WP_122906185	-D---F--K--Q---QFQTAK--		PVTGKV-AATRSQ-E-S-RETMRKPE----
	Brevibacillus parabrevis	WP_063229298	-D---F--K--Q-I-QFQTAK--		PATGKV-AATRSQ-EDS-RETMRKPE----
	Brevibacillus reuszeri	WP_103108993	---F--K--QVI-QFQTSH--		PATGKV-AATRTQ-E-G-R-VMR-P-----
	Brevibacillus sp. BC25	WP_007718275	-E---F--K---VI-QFQTSN--		-VTGKV-AKTRS--E-S-R-TMTKPE----
	Brevibacillus sp. Leaf182	WP_056487150	-E---F--K--D-I-QFQTSN--		-VTGKV-AKTRS--E-S-R-TMTKPE----
	Brevibacillus sp. NRRL B-41110	WP_106835117	-E---F--K---VI-QFQTSN--		-VTGKV-AKTRS--E-S-R-TMTKPE----
	Brevibacillus sp. NRRL NRS-121	WP_106838096	-E---F--K----I-QFQTSN--		-VTGKV-AKTRS--E-S-R-TMTKPE----
	Brevibacillus sp. NRRL NRS-603	WP_106785327	-E---F--K----I-QFQTSN--		-VTGKV-AKTRS--E-S-R-TMTKPE----
	Brevibacillus sp. SCSIO 07484	WP_126428682	-Q---F-KR------RFQAE-R-		AVTGRV-QATVEA-HTA-LEKIR-P-----
	Butyricicoccus sp. 1XD8-22	RKJ47701	-T--FF-SE-A---E----KNN-		IEGR--TEDL-E-IR-QVML---AQEH---
	Caldibacillus debilis	WP_120667045	-T---F--T--A---VFQH-NR-		PETGVI-GKTAKTMEKVILEE--NKA--R-
	Edaphobacillus lindanitolerans	WP_076756727	-K---F--T--K--EKF-N-ED-		QPGRA--RD--R--RSRIT--QA-VK--E-
	Fontibacillus panacisegetis	WP_091235336	-K---FGKG--N---KFQD-N--		-VTGVL-AKTAEA-ESS-I-QIQ-KK--N-
	Hazenella coriacea	WP_131923658	-T---F-QQ--L---AFQKTKNI		AVTGQL-PKTADH-R-S-NDL-R-PK--V-
	Jeotgalibacillus proteolyticus	WP_104058165	-T---FSA---K--QNFQKTVG-		TQ-GEAN-ATL-E-DKAIQS---NPK--H-
	Jeotgalibacillus sp. S-D1	WP_133376483	-T---FST---K-ITAFQKKSG-		KE-GEAN-ATLTE-DKAIQS--DNPK----
	Laceyella sediminis	WP_022737509	-Q---F--R--QSL-MFQKTKG-		PQTG-L-Q-TAEAVQAAFIDFVS-PK--V-
	Lysinibacillus acetophenoni	WP_097147779	-T--FY-KE-GD--A----RSN-		LEGRH-NEEL---IS-QVVA--QEQSH---
	Lysinibacillus chungkukjangi	WP_107932718	-T--FY--N-A---M----QND-		KKGRH--DD--A-IS-QVI---QNVE--A-
	Lysinibacillus composti	WP_124763706	-T--FF-DQ-AD--NQ---KNE-		RTGAV--EE--E-IRKQVIA----VEH---
	Lysinibacillus endophyticus	WP_121213914	-T--FY--Q-AD--A----RND-		RKGRH--DA--S-IR-QVT-----MD--V-
	Lysinibacillus fluoroglycofeni	WP_107942143	-Q--FF--E-A-V-QQF-KKHN-		IAGEELEES--AA-QKEALS---KPEH---
	Lysinibacillus halotolerans	WP_122971033	-T--FF--D-A-S-VQ--KKN--		KNGRQ--DE--A-IR-QVTS--QNME--V-
	Lysinibacillus manganicus	WP_088322074	-T--FY-KE-AD--A----RSN-		AEGRQ-NDEL---IS-KVVA--QEQ-H---
	Lysinibacillus massiliensis	WP_036176407	-T--FF-SE-A---E----KNN-		IEGR--TEDL-E-IR-QVTL---AQEH---
	Lysinibacillus meyeri	WP_107840361	-T--FF--E-AQV--QF-KQHD-		VKGEEL-ES--TA-Q-EALT---KPEH---
	Lysinibacillus odysseyi	WP_036156105	-Q---F--T-Q---IDF--RYD-		DEGDAL-EL--EK-Q-QITL--SKKE----
	Lysinibacillus sinduriensis	WP_036198975	-T--FY--D-A---V----KNE-		KKGRH--DE--A-IR-QVAS---NAE--A-
	Lysinibacillus sp. 2017	WP_108711654	-T--FF-MD-A-E-SKF-KQND-		-EGRHI-EL--TE-SSQVQ-F-ASPL--M-
	Lysinibacillus sp. Marseille-P	WP_106785129	-T--FF-AE-A---E---DRNQ-		N-GKVINDEL-E-IRDQVTS----A-H---
	Lysinibacillus sp. SYSU K30002	WP_126658310	-T--FY--D-GD--A----KND-		KEGRY--NE--TNVR-QII-----TD--A-
	Lysinibacillus sp. YLB-03	WP_118874901	-T--FY--Q-AA--L----KND-		K-GRH--DQ--A-IR-QVMN--QNVE----
	Lysinibacillus sphaericus	WP_075528644	KA---F-KE-VR--MAFQK-KT-		-A-GE-NRE-YMA-K-EVEAFRANKE--Q-
	Lysinibacillus sphaericus	WP_125103469	-V--FF--T-SLTIADFM-TE-V		-STGE--GK--QT-KKSAEA-RN--A--E-

Other Bacteria (0/>100)

Lysinibacillus telephonicus	WP_126293685	-T--FY--D-AQ--A-F--QND-	RKGRH--DA--STIR-QVTA---NED--V-
Lysinibacillus xyleni	WP_097071897	-T--FY--E-A---A----KND-	RKGRY--DI--T-IR-QVT-----MD--V-
Paenibacillus antibioticophila	WP_044480980	-S---FNRA--A---KFQG----	-ATGII-EKTAAA-ERQ-IEHIR-PK----
Paenibacillus barengoltzii	WP_085278207	-K---F-K---K-L-KFQT----	TA-GVL-EKTAGA-EASIIQQIR-PK----
Paenibacillus bovis	WP_060536494	-K---F-R---I---TFQK-H--	VV-GTL-AKTAAE-EAE-I-HIQ-P-Y-V-
Paenibacillus crassostreae	WP_068658995	-V---F-VG-QK---TFQTS--I	KA-GII-SKTAEA-EKV-IEHIQ-PL--T-
Paenibacillus glacialis	WP_068532476	-T---F-QA--KS--SFQTSK--	KV-GII-AKTAEA-ENE-I-HIQ-PL--T-
Paenibacillus macquariensis	WP_068584243	-T---F-QA--KS--SFQTSK--	KA-GII-AKTAVA-ESE-I-HIR-PL--T-
Paenibacillus sp. CAA11	WP_108467113	-R---F-AG-RD--RKFQ---N-	PVSGDI-AKTADAME-N-V-KIA-P---A-
Paenibacillus sp. J14	WP_028540287	-K---F-K---K-L-KFQT----	TA-GVL-EKTAGA-EASIIQQIR-PK----
Paenibacillus sp. J6	WP_085170102	-K---F-K---K-L-KFQT----	TA-GVL-EKTAGA-EASIIQQIR-PK----
Paenibacillus sp. oral taxon 7	WP_009225390	-K---F-KN--K-L-KFQT----	TA-GVL-AKTAEA-EASIIQQIR-PK--M-
Paenibacillus wulumuqiensis	WP_046216438	-K---F-RE--A--RSFQQ-H--	TGTGTI-AKTAAA-EAE-I-HIQ-P-Y-V-
Paenisporosarcina sp. TG20	WP_019414442	-V---F--D-AK--LTFKKEK--	VTDA---QP--K-IRD-ILT--TSKKH-E-
Psychrobacillus insolitus	WP_111438815	-K---FG-D-L--IEG--KENS-	NKGTGINNEL-Q--SK-VTD--ARKE--K-
Psychrobacillus psychrotoleran	WP_093537320	-Q---F--D-QD-IEK--QERS-	DEYNEI-DVL-Q--SK-ILD--TS-E--K-
Psychrobacillus sp. FJAT-21963	WP_056827579	-K---F--D-MDG-EK--QERS-	-PS-DI-DAL-Q--SK--ME--TRKE--K-
Psychrobacillus sp. OK028	WP_093059597	-Q---F--D--D-IEK--QERS-	D-LNEI-DAL-K-IT--ILE--TS-D--K-
Psychrobacillus sp. OK032	WP_093264634	-E---F--G--D-IEN--QERS-	GEG-EINSVL-Q--T--VAA--TKKE--K-
Rummeliibacillus pycnus	WP_102692901	-V---F-RT-AK-IEE-SKKHQ-	TNGNK--D--LFA-H-EMV--RNNLD----
Rummeliibacillus stabekisii	WP_066784739	-----Y-K--AK--QQ-SDKHN-	SGK-E-NEE-LTT-R-EMVQ-R-KEE--T-
Sinobaca qinghaiensis	WP_120191936	-S---FN-E--T--QNFQ-E-D-	-A-GTI-EDTAEA--QA-LENIR-PE--A-
Solibacillus isronensis	WP_079524755	-T--FF--D-AQE-ESF-KKHDI	AEGKY--EV--HE-TKE-QTF-QSKV--M-
Solibacillus kalamii	WP_087615587	-T--FF-KD-AQE-ESF-KKHDI	AEG-Y--EV--HE-T-E-QTF-QSKV--M-
Solibacillus sp. R5-41	WP_099422536	-K--FF-ME-AQ--SNF-KRYD-	QEGRH--EQ--AK-T-Q-QA--ASQI--M-
Sporosarcina koreensis	WP_060209281	-D---F--A-AL--HEF-V-A-V	SPE-I--RK--AAIR-HVEN-RK-KA--K-
Sporosarcina newyorkensis	WP_009765746	-V--FF-KD-A---QAF-A-HQI	A--DA--RE--TT-KAAVEE-RG-AK--K-
Sporosarcina pasteurii	WP_115360245	KA---F-KE-VS--LAFKK-KE-	-V-GK-NRE-YMA-K-EVEVFRA-KE--K-
Sporosarcina psychrophila	WP_067211947	-N--FF----SR--SAF---AEV	-AGPT--RL--STVKIKVEEFRN--K--D-
Sporosarcina sp. BI001-red	WP_116015676	-V--FF--G-G--LAKFMDHE-V	DSSGD--SK--QT-KDSSEA-RN--A--E-
Sporosarcina sp. D27	WP_025786037	-V--FF--T-SQTIADFMK-E-V	-SAGE--SK--QT-KASAEE-RS--A--E-
Sporosarcina sp. HY008	WP_067407919	-K---F----VMG-TDF-A-A--	DEGDV--RK--TA-K-KVE-FRK-EK--E-
Sporosarcina sp. P13	WP_099690007	-T---F-KD-VN--HA--A-RQ-	TE-HV--RE--AD-KKAAEE-RA-VK--K-
Sporosarcina sp. P16b	WP_099673808	-T---F-RE-A---HAF-AEYEI	KEGYD--RE--TA-KTAAEE-RAEPK--K-
Sporosarcina sp. P17b	WP_099626108	-T---F-QE-A---QAF-TKHEI	KEGYD--RE--TT-KTAAEE-RAEPK--K-
Sporosarcina sp. P18a	WP_099675264	-T---F-RE-A---HAF-AEYEI	KEGYD--RE--TA-KTAAEE-RAEPK--K-
Sporosarcina sp. P2	WP_099631058	-T---F-RE-A---HAF-AEYEI	KEGYD--RE--TA-KTAAEE-RAEPK--K-
Sporosarcina sp. P20a	WP_099679183	-T---F-RE-A---QSF-TEYEI	KEGYD--RE--MA-KTAAEE-RAEPK--K-
Sporosarcina sp. P26b	WP_099693602	-T---F-QE-A---QAF-TKHEI	KEGYD--RE--TT-KIAAEE-RAEPK--K-
Sporosarcina sp. P3	WP_099638534	-T---F-SE-A---HAF-AE-EI	KEGYD--RE--TA-KTATEE-RAESK--K-
Sporosarcina sp. P33	WP_081244155	-T---F-SD-AD--HAF-I-REI	KEGYD--RE--TA-KTAAEE-RAEEK--K-
Sporosarcina sp. P34	WP_099696134	-T---F-RE-A---HAF-AEYEI	KEGYD--RE--TA-KTAAEE-RAEPK--K-
Sporosarcina sp. P7	WP_099637509	-T---F-RE-A---HAF-AEYEI	KEGYD--RE--TA-KTAAEE-RAEPK--K-
Sporosarcina sp. PTS2304	WP_114923112	-T---F-KD-AN--HA--A-RE-	TESRI--RE--ADIKKAAEE-RA-EK--K-
Sporosarcina sp. ZBG7A	WP_039042106	-V--FF--T-GQT-ADFMKAE-V	-STGE--GK--QT-KTSAEA-RN--A--E-
Sporosarcina ureae	WP_083035237	-T---F-SE-A---HAF-AE-EI	KEGYD--RE--TA-KTATEE-RAESK--K-
Ureibacillus thermophilus	QBK27126	-T--FY--E-A---AS---KF--	KRGRH--DE--A-IR-KVVLF---MEQ-V-
Ureibacillus thermosphaericus	WP_016839257	-T--FY--Q-A---AS--QKHD-	KSGRH--DE--A-IRDQVAL----MEH-V-
Viridibacillus arvi	WP_053417747	-T---F-RQ-A---L---KTK--	AQEPIINEA--SAIHKEMVE--DSNE--H-
Viridibacillus sp. OK051	PKA85231	-T---F-RE-A---LM--KKK--	AQEPIINES--SA-HKEMVE--GNN---H-

Supplemental Figure 19

A partial sequence alignment of the PDZ domain-containing protein containing a three amino acid insertion (boxed) that is exclusively shared by all members belonging to the *Lysinibacillus sensu stricto* clade and absent in all other bacteria. *Lysinibacillus pakistanensis* was not in the NCBI non redundant protein sequences database at the time of writing this paper.

Group	Organism	Accession	408		451
Lysinibacillus sensu stricto (11/11)	Lysinibacillus tabacifolii	WP_108029457	GKIIIIILMFIGRVGLISFLYSV	GG	GGRGKKSKYHYPKERVIIG
	Lysinibacillus contaminans	WP_053584549	--V--M---------------TL	--	------TNF----------
	Lysinibacillus macroides	WP_053993708	------V--------------TL	--	-------SF----------
	Lysinibacillus xylanilyticus	WP_100544213	-----M----V----------TL	--	-------S-----------
	Lysinibacillus mangiferihumi	WP_107897142	-----------------------	--	-------------------
	Lysinibacillus varians	WP_024362269	-----------------------	--	-------------------
	Lysinibacillus fusiformis	WP_036123502	--V---M--------------TL	--	--------F----------
	Lysinibacillus boronitolerans	WP_036075771	---------------------TL	--	-------GF----------
	Lysinibacillus parviboronicapiens	WP_107923265	--F------------------TL	--	---A---S-----------
	Lysinibacillus sphaericus	WP_103977362	-----------------------	--	----------F--------
	Bacillus sp. B14905	WP_008174151	---------------------TL	--	--------F----------
Other Bacteria (0/>200)	Bacillus novalis	WP_066085545	A-C-LM-------I--T--FLII	-	-EGN-ED-------K--T-
	Bacillus shackletonii	WP_055741895	S-C-LM----V--I--T----II	-	-DNN--A---------VT-
	Lysinibacillus sphaericus	WP_075528581	-----MV------------FTL		---T--PH-----------
	Lysinibacillus sphaericus	WP_125101956	--V--MA------------FTL		--KVN-TSFR---------
	Anoxybacillus pushchinoensis	WP_091700821	--LV--V------I-IL---FLI		R-NNQ-ET-----------
	Bacillus acanthi	WP_108670199	--FV-MV--------I----FMI		--KE--IS-------I---
	Bacillus aciditolerans	WP_121445314	---L-ML--------ILN-IFII		-KK--DVN-------L---
	Bacillus aidingensis	WP_026701373	--VV-MM------I-IF---FIM		R-KV-GDT-----------
	Bacillus alkalitelluris	WP_078544384	--V---L------I-IL--IFLI		NRETDEV--------I---
	Bacillus andreraoultii	WP_033829378	-----ML--------ILT-IFMI		--KE--ENF------I---
	Bacillus anthracis	WP_071737421	--LVL-V--------ILT-ILAS		---EQPPR-K-----I---
	Bacillus aquimaris	WP_113968441	--SV--G--------IL---FMI		--KE-TT------------
	Bacillus australimaris	KPN13725	--C--MVV-----I-IP----LI		-R-ESEAN-----------
	Bacillus campisalis	WP_046525962	--F--M-----------Q--LAM		--KE-NQSI----------
	Bacillus cavernae	WP_126864248	----DMT--------IV---FII		-R-E-TDN-------I---
	Bacillus cecembensis	WP_057988601	--V--MA---------------L		--KAN-KN-----------
	Bacillus cereus	WP_046392499	--LVL-V--------ILT-ILAS		---EQPPR-K-----I---
	Bacillus cihuensis	WP_028393276	--FVLM-------I--T---FII		--KK-EAN-----------
	Bacillus circulans	WP_047942949	--CV-ML------I--T--I-II		--KE--DNF------I-V-
	Bacillus coahuilensis	WP_059350618	--VVLM---------IL---FI-		--KE-RVM-------I---
	Bacillus dakarensis	WP_077211302	--CV-MV------I--T---FMI		--KKREVN-R---------
	Bacillus firmus	WP_035327469	--C--MA------I--T---FII		--KE-QPN-----------
	Bacillus glycinifermentans	WP_048354382	---V-M-------I-IVTL--LI		-RKESEAN-----------
	Bacillus gottheilii	WP_066447245	--TV-ML------I--T--IFII		--KMT-PN-----------
	Bacillus hisashii	WP_095141671	-----ML--------ILT--LMI		--KE--ENF----------
	Bacillus horneckiae	WP_066393050	--CVLMV------I--T---FII		--KE--AN-----------
	Bacillus humi	WP_057996549	---L-ML--------ILN-IFII		-KK--DVN-------L---
	Bacillus indicus	WP_029281802	--C--MV------I-IVT-I-II		-SKAS-PN-----------
	Bacillus jeotgali	WP_079508204	---VL----------V----LII		--KTE-ESI----------
	Bacillus litoralis	WP_066331103	--VV-M-------I-IL----LL		-TKER-DN--F--------
	Bacillus loiseleuriae	WP_049680288	--FVLM-------I--T---FII		--KK-DAN-----------
	Bacillus marisflavi	WP_048004261	--CV-M---------IL---FMI		--KE--AN-------L---
	Bacillus massilioanorexius	WP_019242526	--CV--L--------IVT--FMI		--KE--VN-R--E--I---
	Bacillus ndiopicus	WP_042471330	---V-M---------------TL		--NT--L------------
	Bacillus nealsonii	WP_016200908	--CV-ML------I--T--I-II		--KE--DNF------I---
	Bacillus oceanisediminis	WP_110067489	--C--MA------I--T-S--II		--KE-QPNF----------
	Bacillus oleivorans	WP_097159703	--FV-MF---V----ILA--FML		---NNNVN-----------
	Bacillus oleronius	WP_078110803	--CLL-V---------V-LM-LM		---DQSNN-------L---
	Bacillus onubensis	WP_099354121	---L-ML--------ILN-IFII		-KK--DAN-------L---
	Bacillus safensis	WP_111291302	--C--MVV-----I-IP----LI		-R-ESEAN-----------
	Bacillus selenatarsenatis	WP_041964831	--MVL----------V----LII		--KTE-ESI----------
	Bacillus simplex	WP_076370355	--VV-M------KI-MFT--F-M		--NPV-HA-------I---
	Bacillus sinesaloumensis	WP_077617377	---LL-L--------ILN-IFII		-KK--DV--------L---
	Bacillus sp. 100374	WP_099683738	--LVL-V--------ILT-ILAS		---EQPPR-K-----I---
	Bacillus sporothermodurans	WP_066227880	--CLL-V--------IV-LM-MM		---DQSNN-------L---
	Bacillus stratosphericus	WP_081114179	--C--MVV-----I-IP----LI		-R-ESEAN-----------
	Bacillus subterraneus	WP_125479331	--SVL----------V----LII		---TY-ESV----------
	Bacillus taeanensis	WP_113806330	---V-M-------I--L---FII		R-NTSAD------------
	Bacillus testis	WP_050614560	--V-L-L------I-I----FTM		--KE-RLG-R--E------
	Bacillus thermoamylovorans	WP_108897863	-----ML--------ILT--LMI		--KE--ENF----------
	Bacillus thuringiensis	WP_061883914	---VL-V--------ILT-ILAS		---EQPPR-K-----I---
	Bacillus timonensis	WP_026021711	--LL-ML--------ILN-IFII		-KK--DVN-------L---
	Bacillus toyonensis	WP_098710175	--LVL-V--------ILT-ILAS		---EQPPR-K-----I---
	Bacillus velezensis	OQV37360	--CV-M-V-----I-ILT---LI		-RKEIEAN-------M---
	Bacillus vietnamensis	WP_060670125	--CV--A--------IL---FMI		--KE-TA------------
	Bacillus vireti	WP_024026241	--FVLMM------I--T---FII		--SN---N-N---------
	Bacillus weihaiensis	WP_072581882	--V--M---------ILT---LL		-TKE--AN-----------
	Bacillus wiedmannii	WP_098147064	--LVL-V--------ILT-ILAS		---EQPPR-K-----I---
	Bacillus xiamenensis	WP_008361372	--C--MVV-----I-IP----LI		-R-ESEAN-----------
	Bacillus zeae	WP_119111361	--TV-MV--------IV---V-M		A-SQ-S-Q--F---K----
	Bacillus zhangzhouensis	KEP26099	--C--MMV-----I-IP----LI		-R-ESEAN-----------
	Bhargavaea beijingensis	WP_092095937	--AVLMF---V---------FTL		--KPQ-LN-R---------
	Bhargavaea cecembensis	KZE39085	--V-LMF---V---------FTL		--KPQ-LN-R---------

**Other Bacteria (0/>200)**	Bhargavaea ginsengi	WP_092050722	--S--MF---V---------FTL	--KPQ-LN-R--E------
	Caryophanon latum	WP_066461550	--VV-LL---------------I	--NTR-R--D--T--I---
	Caryophanon tenue	WP_066543676	---V-ML---------------L	--NKR-R--D--T------
	Chlamydia trachomatis	CRH76753	--VV-MT-----KI-MFT--F-M	--KPL-HA-------I---
	Chryseomicrobium excrementi	WP_100352854	--VT-MF-------------FTL	--KSNST-F----------
	Domibacillus indicus	WP_046174059	A---L-----------F-L-FLI	R--EI-DS-------IL--
	Domibacillus tundrae	WP_046179020	--VV-M------KI-MFT--FFM	--KPV-HP-------I---
	Edaphobacillus lindanitolerans	WP_076756670	--V-LMF---V---------FTL	--KP--LNF----------
	Filibacter sp. TB-66	WP_124071256	--S--M--------------FTL	--KT--PH--F--------
	Geobacillus kaustophilus	WP_044736860	--LVLV-V-------I----WL-	R--AV-DS-----------
	Geobacillus stearothermophilus	KFL16752	--LVLV-V-------I----WL-	R--AV-DS-----------
	Geobacillus thermodenitrifican	WP_100660565	--LVLVM--------I----WLI	R--AV-DS-----------
	Halobacillus aidingensis	WP_089650858	------T------I-IF---FII	R-K-I-D-------PM---
	Halobacillus dabanensis	WP_075035960	--V---C------I-IF---FII	R---T-DR------PM---
	Halobacillus trueperi	WP_115894003	------T------I-IF---FII	R-K-I-D-------PM---
	Jeotgalibacillus alimentarius	WP_041122519	S---LMF---V---------FMI	--NN--E-FR---------
	Jeotgalibacillus campisalis	WP_041057095	S-F--MV-------------FMI	--NN--A------------
	Jeotgalibacillus malaysiensis	WP_039813403	S-M-LMV-------------FMI	--NN--E------------
	Jeotgalibacillus proteolyticus	WP_104056364	S-F--MM-------------FMI	--NN--A------------
	Jeotgalibacillus salarius	WP_134382208	S-V-LM--------------FMI	--NN--E-FR---------
	Jeotgalibacillus soli	WP_041088640	S-F-LMF-------------FMI	--NN--A------------
	Jeotgalibacillus sp. R-1-5s-1	WP_134372216	S-F-LM----V---------FMI	--NN--E-F----------
	Jeotgalibacillus sp. S-D1	WP_133376070	S-F--ML-------------FMI	--NN--A------------
	Kurthia gibsonii	WP_087682385	--V--M----V-----------L	--KSE-P------------
	Kurthia huakuii	WP_029498067	--VV-ML---------------L	--KAE-P-F----------
	Kurthia massiliensis	WP_010286688	---VVML---------------L	--KSE-P------------
	Kurthia senegalensis	WP_010301958	--C--ML-------------F-I	--KSE-T-F--------T-
	Kurthia sibirica	WP_109304359	--C--MV---V-----------I	--K-D-P------------
	Kurthia sp. 11kri321	WP_068455303	--V--M----V-----------L	--KSE-P------------
	Kurthia zopfii	WP_109349993	--V--M----V-----------L	--K-E-P------------
	Lysinibacillus acetophenoni	WP_097147835	-----M----------------L	--KEN-AH-R---------
	Lysinibacillus chungkukjangi	WP_107934982	--FV-M---------------TL	---VD-PP-R---------
	Lysinibacillus composti	WP_124763652	---V-M---------------TL	--KAN-AI-R--Q--I---
	Lysinibacillus endophyticus	WP_121214099	--FVVM---------------TL	--KAE-PP-R---------
	Lysinibacillus halotolerans	WP_122973358	--FV-M---------------TL	--KAE-AP-R---------
	Lysinibacillus jejuensis	WP_108306235	--VV-ML---------------L	--KT--T-F----------
	Lysinibacillus manganicus	WP_036181952	-----M----------L---F-L	--KAN-TNF----------
	Lysinibacillus massiliensis	WP_036172130	---VVM----------------L	--KA---Q-R---------
	Lysinibacillus meyeri	WP_107841896	---V-MV--------I-----TL	--NR--L------------
	Lysinibacillus odysseyi	WP_036155835	--GV--V--------------TL	--KT--VN-R---------
	Lysinibacillus saudimassiliensis	CEA02308	---V-ML---------------I	--KT--T-F----------
	Lysinibacillus sinduriensis	WP_036202521	--FVMM---------------TL	--KVD-PP-R---------
	Lysinibacillus sp. 2017	WP_108711720	--F--MG---------------I	T--PT-K------------
	Lysinibacillus sp. BF-4	WP_036143557	---V-ML---------------I	--KT--T-F----------
	Lysinibacillus sp. Marseille-P	WP_106783887	--F-MM----------------L	--KAN-AQ-R---------
	Lysinibacillus sp. SYSU K30002	WP_126658412	--FV-MV--------------TL	--KAD-LP-R---------
	Lysinibacillus sp. YLB-03	WP_118874840	--FVMML--------------TL	--KVD-PP-----------
	Lysinibacillus telephonicus	WP_126296086	--FVVMA--------------TL	--KAD-PP-R---------
	Lysinibacillus xyleni	WP_097071953	--FVVM---------------TL	--KAE-PP-R---------
	Macrococcus bovicus	WP_133451265	S--V-M-V---------T-VVML	--KAEPV---------M--
	Macrococcus brunensis	WP_133432797	S--V-M-V---------T-VIML	--KAEPV--------IM--
	Melghiribacillus thermohalophi	WP_132372807	------L------I-ILT--F-F	NTEK-QGE-------I---
	Mycobacteroides abscessus subs	SLK30065	--CV-ML------I--T--I-II	--KE--DNF------I---
	Oceanobacillus sp. YLB-02	WP_121523995	--VV-M------KI-IFT--FFI	--KTV-H-H------L---
	Paenibacillus anaericanus	WP_127191591	ARCLLM-------I--T---FII	---Q--A-FN-------T-
	Paenibacillus selenitireducens	WP_078498493	A-C-L-V------I--T----II	--NN-DA-F---V----T-
	Paenibacillus sp. 18JY21-1	WP_132417606	A-C-LMV------I--T---FII	--KQ--A-L-----K--T-
	Paenibacillus sp. 1_12	WP_091170863	--C-LMV------I--T---FII	--EK--ANF---E----T-
	Paenibacillus sp. FSL R5-0490	WP_076256917	--C--MA------I--T---FII	--KE-QPN-----------
	Paenibacillus terrigena	WP_018754348	A-S-L-V------I--T----II	--NK-EAR----V----T-
	Paenisporosarcina antarctica	WP_134210225	--SL-M----V---------FTI	---TT-T------------
	Paenisporosarcina indica	WP_075617365	---L-MV-------------FTI	---RI-T---F--------
	Paenisporosarcina quisquiliaru	WP_090563114	---T-MV-------------FTL	--KHN-T------------
	Paenisporosarcina sp. HGH0030	WP_016427882	---L-ML---V--I-------TI	---TT-T-F----------
	Paenisporosarcina sp. K2R23-3	WP_119882798	--VL-ML------------IFTI	---AE-T---F--------
	Paenisporosarcina sp. OV554	WP_108584361	---L-ML---V--I-------TI	---TT-T-F----------
	Paenisporosarcina sp. TG-14	WP_017379358	--SL-M----V---------FTI	---TT-T------------
	Paenisporosarcina sp. TG20	WP_019413110	--TL-MV---V---------FTI	---TTTT-F----------
	Planococcus antarcticus	WP_006831011	--F--MA---V---------FTI	--KTDPT-F----------
	Planococcus citreus	WP_121297829	--V-VMV-------------FTI	--KTDPT-F----------
	Planococcus donghaensis	WP_065525841	--V--MA---V---------FTI	--KTDPT-F----------
	Planococcus halocryophilus	WP_008498098	--F--MA---V---------FTI	--KTDPT-F----------
	Planococcus halotolerans	WP_112223019	--LT-MV-------------FTI	--KTDPT-F----------
	Planococcus maitriensis	WP_112231228	--V-VMV-------------FTI	--KTDPT-F----------

Other Bacteria (0/>200)

Planococcus maritimus	WP_068463103	--V-VM-------------FTI	--KTDPT-F----------
Planococcus massiliensis	WP_052651009	--F--MA------------FTI	--KTDPT-F----------
Planococcus plakortidis	WP_068869529	--V-VM-------------FTI	--KTDPT-F----------
Planococcus rifietoensis	WP_058383434	--L--MV------------FTI	--KTDPT-F----------
Planococcus salinarum	TAA72165	--VTVMV------------FTI	--KMDPT-F----------
Planococcus salinus	WP_123166332	--L--MG------------FTI	--KTDPT-F----------
Planococcus sp. CAU13	WP_033542236	--LT-MV------------FTI	--KTDPT-F----------
Planococcus sp. PAMC 21323	WP_038704406	--F--MA---V--------FTI	--KTDPT-F----------
Planococcus versutus	WP_049693433	--F--MA---V--------FTI	--KNDPT-F----------
Planomicrobium flavidum	WP_088005853	--FV-MA-----------IF-L	--KSDPTNF----------
Planomicrobium glaciei	WP_036807208	--VV-MA------------FTI	--KSD-T-F----------
Planomicrobium okeanokoites	WP_084244810	--LT-MV------------FTI	--KTDPT-F----------
Planomicrobium soli	WP_106534753	--L--MV------------FTI	--KTDPTNF----------
Planomicrobium sp. MB-3u-38	WP_101801475	--LT-MV------------FTI	--KTDPT-F----------
Planomicrobium sp. Y74	WP_121632956	--LT-MV------------FTI	--KTDPT-F----------
Pontibacillus halophilus	WP_026800676	--VT---------I-IL---FTF	KKE--GN----T--I---
Psychrobacillus insolitus	WP_111438742	--VT-M-------------F-L	--KSNQT-----------
Psychrobacillus psychrodurans	WP_093494321	---T-MV------------FTL	--KHN-T-----------
Psychrobacillus psychrotoleran	WP_093535518	--V--M-------------FTL	--KHN-T-----------
Psychrobacillus sp. FJAT-21963	WP_056827504	--VT-M--------------TL	--KSN-------------
Psychrobacillus sp. OK028	WP_093059744	--VT-M-------------FTL	--KHN-T-----------
Psychrobacillus sp. OK032	WP_093264319	--LTMM---------ML---F-L	--KSN-T-----------
Rhizophagus irregularis	PKC52374	---VVM---------------L	--KA---Q-R---------
Rummeliibacillus pycnus	WP_102692846	--VVM-V--------I------I	--KSE-TN----------
Rummeliibacillus sp. POC4	WP_119414687	--VVMM----V----I-----FI	--KPE-TNF---------
Rummeliibacillus sp. TYF005	WP_124217589	--VVMM----V----I-----FI	--KPE-TNF---------
Rummeliibacillus stabekisii	WP_066784932	--VVM-F--------M------L	--KA-QT-----------
Salinicoccus halodurans	WP_046789577	S-V--MMF-----I-F----F-I	---QHELP-------IL--
Salinicoccus luteus	WP_084185027	S--VVM-F-----I-F----FTM	A--Q-ELP--F----IL--
Salinicoccus roseus	WP_124010657	S--VVM-F-----I-F----F-M	A--Q-ELP--F----IL--
Sediminibacillus halophilus	WP_074598333	------F------I-IF---FLL	R--VT-DI----------
Solibacillus isronensis	WP_079524842	--MV-MV---V----------I	--SAQ-K--------I---
Solibacillus kalamii	WP_087615541	--MV-MV---V----------I	--SA--K--------I---
Solibacillus silvestris	WP_065216498	--MV-MV---V----------I	--SA--K--------I---
Solibacillus sp. R5-41	WP_099422592	--VV-M---------------L	--KTN-KN----------
Sporosarcina globispora	WP_053436704	--C--MA------I--T---FII	--KE-QPNI---------
Sporosarcina koreensis	WP_040287294	-----MA------------FTL	--KVY-TSFR--------
Sporosarcina newyorkensis	WP_078817860	--L--MV-------------TI	--KTN-TP----------
Sporosarcina pasteurii	WP_115360303	-----MV------------FTL	---TQ-PH----------
Sporosarcina psychrophila	WP_067214027	--V--ML------------FTL	--KT--L-----------
Sporosarcina ureae	WP_029052447	--L--MA-----------I-TI	--KSN-TP----------
Staphylococcus aureus	KMR14028	S-VVLM------------IIMI	A--REPD-F------IQ--
Staphylococcus devriesei	WP_107506941	-V-LM------------IIMI	A--KEPD-F-------Q--
Staphylococcus equorum	WP_107515396	--VLM------------IIMI	---REPD--------IQ--
Staphylococcus gallinarum	SUM32379	-L-LML-----------IIMI	---REPD-F-------Q--
Staphylococcus haemolyticus	WP_037557767	-V-LM------------IIMI	A--KEPD---------Q--
Staphylococcus kloosii	WP_061855083	-V-LM------------VIMI	A--REPD--------IQ--
Staphylococcus pasteuri	WP_108016841	---LM------------IIMI	---KEPK--------IQ--
Staphylococcus saprophyticus	WP_048792736	-V-LM------------VIMI	A--REPD--------IQ--
Staphylococcus simiae	WP_002461904	S--VLMV-----------IIMI	A--REPD--------IQ--
Staphylococcus sp. AOAB	ODB64349	---LM------------IIMI	---KEPK--------IQ--
Staphylococcus sp. NAM3COL9	WP_057512675	S-VVLM------------IIMI	---REPD--------IQ--
Staphylococcus xylosus	WP_039067720	S-MALM------------IIMI	---REPD---------Q--
Streptococcus pneumoniae	COH31880	--LVL-V--------ILT-ILAS	---EQPPR-K-----I---
Tetzosporium hominis	WP_094941512	--VT-MF------------FTL	--KSNST-F---------
Ureibacillus thermophilus	QBK25456	---VVMA---------L----TL	--KAQ-P--R--E--I---
Ureibacillus thermosphaericus	WP_096550537	--LV-MT------------FTL	--KAQTP--R--E--I---
Vibrio vulnificus	WP_133352585	--VV-MT-----KI-MFT--F-M	--KPL-HA-------I---
Virgibacillus ndiopensis	WP_099160075	---V--S------I-IL--IFLF	NR-N-QTN---------V-
Virgibacillus senegalensis	WP_053218816	--C---F------I-IF---FLL	R--VT-DI----------
Viridibacillus arvi	WP_053417636	--V-MML--------------L	--KA--------------
Viridibacillus sp. OK051	WP_100796277	--V-MML--------------I	--K---T-----------

Supplemental Figure 20

A partial sequence alignment of the TrkH family potassium uptake protein containing a two amino acid insertion (boxed) that is exclusively shared by all members belonging to the *Lysinibacillus sensu stricto* clade and absent in all other bacteria. *Lysinibacillus pakistanensis* was not in the NCBI non redundant protein sequences database at the time of writing this paper.

Group	Organism	Accession	197		262
***Lysinibacillus sensu stricto* (11/11)**	Lysinibacillus boronitolerans	WP_036080147	WENKHRLL	REGSGIAADIDDETEQPVSSLK	SATGTAFAGKTGFTKVAGRTLATAFQKDGQTCIVVT
	Lysinibacillus contaminans	WP_053584139	-------V	--Q--PVS-SK--E----T---	RS--S-Y-----Y-----------------------
	Lysinibacillus fusiformis	WP_004232205	--------	-----M----------------	-----------------------------I-----
	Lysinibacillus macroides	WP_053994126	--------	-----SV-EM------------	----M------------------------I-----
	Lysinibacillus parviboronicapiens	WP_107949735	--------	-----V-SE-N--A--------	------------------------------------
	Lysinibacillus varians	WP_025218905	--------	-----V-VEV------------	------------------------------------
	Lysinibacillus xylanilyticus	WP_100545069	--------	-----T-IE-G---Q---NA--	--K-S-------------------------------
	Lysinibacillus tabacifolii	TKI48171	--------	-----V-VEV------------	------------------------------------
	Lysinibacillus mangiferihumi	TKI61944	--------	-----V-VEV------------	------------------------------------
	Lysinibacillus sphaericus	WP_051112139	--------	-----V-TE---A---------	----S-------------------------------
	Bacillus sp. B14905	EAZ87255	--------	-----M--E-------------	----V-Y-----------------------------
**Other Bacteria (0/>100)**	Bacillus cecembensis	WP_057982158	-R------		HLKDG-V-----Y----------YYER-DKKFV---
	Bacillus ndiopicus	WP_042472244	-K------		-ENNG-I----------------Y---EQKQF----
	Bacillus sp. 72	WP_034286004	-------V		RDG-MITG----Y--KT----V-TAK-N-LHLVA--
	Bacillus sp. FJAT-22090	WP_053589830	-K---K--		HYSDQ-I--------R-----V-Y-KDGSKEI----
	Bacillus sp. OxB-1	WP_084212558	-R------		HSEP--V--------A-------Y-E---KRI----
	Bhargavaea beijingensis	WP_125903218	-Q-----V		R-E-D-V--------T-------L-E---KRV----
	Bhargavaea cecembensis	WP_008297045	-Q-----V		R-E-D-V--------T-------L-E---KRV----
	Bhargavaea ginsengi	WP_092048778	-Q-----V		RGQ-E-V--------T-------L-E---KRV----
	Butyricicoccus sp. 1XD8-22	RKJ32255	-L------		REKVG-I----------------Y-EEN-KKV----
	Domibacillus aminovorans	WP_063966870	-------V		RDG-MITG----Y--KT----V-TAK-N-LHLVA--
	Domibacillus antri	WP_075397566	-------V		REG-RITG----Y--KS----V-TAKQN-LEFVA--
	Domibacillus epiphyticus	WP_095532695	-------V		REG-RITG-------RS----V-TAKQN-LHLVT--
	Domibacillus mangrovi	WP_073710034	-------V		RTG-MVTG----Y--KT----V-TAK-N-LQLVA--
	Domibacillus robiginosus	WP_082220247	-------V		RSG-MITG-------KS----V-TAE---M-LA---
	Edaphobacillus lindanitolerans	SIT66704	-Q-----V		RGE---V--------R-------Q-E-E-KAV----
	Filibacter sp. TB-66	WP_124070699	-Q-----I		GSDSG-I----------R-----Y-E---KNI----
	Lysinibacillus acetophenoni	WP_097149034	-L------		REKVG-I----------------Y-EQ--KRV----
	Lysinibacillus boronitolerans	KGR87370	-Q-----V		QEQNG-V--------A-------Y-E-EDKKV----
	Lysinibacillus chungkukjangi	WP_107935383	-Q------		RQQVG-VS---------------Y-EQ-NKKV----
	Lysinibacillus composti	WP_124765131	-Q------		REQVG-V----------------Y-E--QKKV----
	Lysinibacillus endophyticus	WP_121215603	-Q---K--		RQQVG-VS-------I-------Y-EQGEEKV----
	Lysinibacillus fluoroglycofeni	WP_107942368	-K------		-ENNG-V-----Y----------Y---EQKHF----
	Lysinibacillus halotolerans	WP_122972872	-Q------		RQQVD-V----------------Y-E--NKKV----
	Lysinibacillus manganicus	WP_036184092	-L------		RENVG-I--------I-------Y-E---KRV----
	Lysinibacillus massiliensis	WP_036177023	-L------		REKVG-I----------------Y-EEN-KKV----
	Lysinibacillus meyeri	WP_107839350	-K------		-ENNG-I----------------Y---EQKQF----
	Lysinibacillus odysseyi	WP_036154276	-Q---K--		-QGIG-I-----Y----------Y-ERGDKEFV---
	Lysinibacillus sinduriensis	WP_036204173	-Q------		RQQVG-V----------------Y-EEGDKKV----
	Lysinibacillus sp. 2017	WP_108714431	-K------		HEDVG-VS----Y----------F-EREQKQF----
	Lysinibacillus sp. Marseille-P	WP_106785163	-L------		REKVG-I----------------Y-EQN-KRV----
	Lysinibacillus sp. SYSU K30002	WP_126658054	-Q---K--		RQQVG-L----------------Y-EE--KKVV---
	Lysinibacillus sp. YLB-03	WP_118875184	-Q-----I		REQVG-V----------------Y-EQGQKKVV---
	Lysinibacillus sphaericus	WP_125102288	-------I		TSDTD-V--------A-------Y-E-N-KKV----
	Lysinibacillus telephonicus	WP_126295446	-Q------		REQVG-V----------------Y-EQGQKKV----
	Lysinibacillus xyleni	SOB94188	-Q------		RQQVG-VS---------------Y-EQ-QEKV----
	Paenisporosarcina antarctica	WP_134210006	-----K-V		RLES--I-----Y--A-------Y-KDGEKEV----
	Paenisporosarcina indica	WP_084543941	-----K-V		RSDTK-I--------T-------Y-KDGEEEV----
	Paenisporosarcina quisquiliaru	WP_090561846	-R---K--		HFNKY-V-----Y--------V-Y-EDN-KKV----
	Paenisporosarcina sp. HGH0030	WP_016427451	-D---K-V		RLETK-I--------T-------Y-KDGDEEV----
	Paenisporosarcina sp. K2R23-3	WP_119883093	------		HENNY-VS-------A-------F-E--NESV----
	Paenisporosarcina sp. OV554	WP_108587637	-----K-V		RSETK-I--------A-------Y-KDGEKEV----
	Paenisporosarcina sp. TG-14	WP_017380392	-----K-V		RLES--I-----Y--A-------Y-KDGEKEV----
	Paenisporosarcina sp. TG20	WP_019415249	-------V		R-EPS-I--------A-------Y-KN-KEEEVI--
	Psychrobacillus insolitus	WP_111438368	-R---K-M		HFEDN-V-----Y--R-----I-Y-E--KKEI-I--
	Psychrobacillus psychrodurans	WP_093494629	-R---K--		HFNKY-V-----Y--------V-Y-EDN-KKV----
	Psychrobacillus psychrotoleran	WP_093534817	-R---K--		HYNKF-I-----Y--------V-Y-ED--KKV----
	Psychrobacillus sp. FJAT-21963	WP_056829428	-K---K--		HYSDQ-I--------R-----V-Y-KDGSKEI----
	Psychrobacillus sp. OK028	WP_093060812	-K---K--		HYNES-I-----Y--A-----V-F-EE--KKV----
	Psychrobacillus sp. OK032	SES34121	-N-----I		HESE--I-----Y--R-----V-Y-KE-EKEIV---
	Rummeliibacillus pycnus	WP_102694353	-K---K--		HYNKY-I----------------Y-E---KRV----
	Rummeliibacillus stabekisii	WP_066791843	-Q---K--		HSNKY-I-----Y----------Y-E---KRV----
	Solibacillus isronensis	WP_079525757	-R-----M		HEKIG-I-----Y----------Y-EREQKSFV---
	Solibacillus kalamii	WP_087618241	-R-----M		HEKIG-I-----Y----------F-EREQKSFV---
	Solibacillus silvestris	WP_014824345	-R-----M		HEKIG-I-----Y----------F-EREQKSFV---
	Solibacillus sp. R5-41	WP_099423017	-K------		HLKDG-V-----Y----------YYER-DKKFV---
	Sporosarcina koreensis	WP_060207906	-R----MI		HTNDFVI--------A-------Y-E---KKL-I--
	Sporosarcina newyorkensis	WP_078816439	-Q------		LSEP--I----------------Y-E---KSV----
	Sporosarcina pasteurii	WP_115360638	-A-----V		RS-E--I--------A-----V-Y-E--NKRV----
	Sporosarcina psychrophila	WP_067210462	-R------		HSES--I--------A-------Y-E---KNI----
	Sporosarcina sp. BI001-red	WP_116017165	-------I		TSDTD-V--------A-------Y-E-N-KKV----
	Sporosarcina sp. D27	WP_081760225	-------I		TSDTD-V--------A-------Y-E-N-KKV----
	Sporosarcina sp. EUR3 2.2.2	WP_024534901	-----K-V		RLDSK-I--------A-------Y-KDGDEEV----
	Sporosarcina sp. HY008	WP_082731993	-G-----V		RNEP--I--------A-----V-Y-E---KSI--C-
	Sporosarcina sp. P1	WP_099626579	-Q-----V		LSEP--V--------A-------Y-E---KSV----
	Sporosarcina sp. P13	WP_099686989	-Q------		LSEPS-I--------A-------Y-E---KSV----
	Sporosarcina sp. P2	WP_099631657	-Q-----V		LSEP--V--------A-------Y-E---KSV----
	Sporosarcina sp. P20a	WP_099677113	-Q-----V		ISEP--V--------A-------Y-E---KSV----
	Sporosarcina sp. P29	WP_099661486	-Q-----V		ISEP--V--------A-------Y-E---KSV----
	Sporosarcina sp. P3	WP_099638148	-Q-----V		ISEP--V--------A-------Y-KQ--KSV----

Other Bacteria (0/>100)

Sporosarcina sp. P33	ARD49778	-Q-----V		LSEP--I--------A-------Y-E---KSV----
Sporosarcina sp. P34	WP_099694632	-Q-----V		LSEP--V--------A-------Y-E---KSV----
Sporosarcina sp. P35	WP_099662755	-Q-----V		LSEP--I--------A-------Y-E---KSV----
Sporosarcina sp. P37	WP_085431374	-Q-----V		LSEP--I--------A-------Y-E---KSV----
Sporosarcina sp. P7	WP_099636079	-Q-----V		LSEP--V--------A-------Y-E---KSV----
Sporosarcina sp. PTS2304	WP_114925818	-Q------		LSEP--I--------A-------Y-E---KAV----
Sporosarcina sp. ZBG7A	WP_082003284	-------I		TSDTD-V--------A-------Y-E-N-KKV----
Sporosarcina ureae	ARF15807	-Q-----V		ISEP--V--------A-------Y-E---KSV----
Ureibacillus thermophilus	QBK25812	-L------		RENVG-V-----Y---------Y-ER-DERI----
Ureibacillus thermosphaericus	WP_050988822	-L------		RENVG-V-----Y---------Y-E-GNERI----
Viridibacillus arenosi FSL R5-	ETT81217	-----K-V		RNNKF-I-----Y---------Y-E---KKV----
Viridibacillus arvi	WP_053418286	-----K-V		RNNKF-I-----Y---------Y-E---KKV----
Viridibacillus sp. FSL H8-0123	WP_076065253	-----K-V		RNNKF-I-----Y---------Y-E---KKV----
Viridibacillus sp. OK051	WP_100796612	-----K-V		RNNKF-I-----Y---------Y-E---KKV----
unclassified Erysipelotrichace	WP_120146182	-K--N---		FDYPFTIG-------K-----V--AEH--VES----
Bacillus acidicola	WP_066262546	-K--N---	T	EKYKYCTG-------L-R---V-TAS---ENL-S--
Bacillus albus	WP_128974598	-K---K-V	T	-YYEF-TG-------K-----V-TAS---LDL----
Bacillus anthracis	AIM10792	-K---K-V	T	-YYEF-TG-------K-----V-TAS---LDL----
Bacillus bingmayongensis	WP_017152557	-K---K-V	T	-YYEF-TG-------K-----V-TAS---LDL----
Bacillus cereus	WP_098540377	-K---K-V	T	-YYEF-TG-------K-----V-TAS---LDL----
Bacillus funiculus	WP_129728229	-H---K--	T	-MYKY-TG-------K-----V-TAS---LDL----
Bacillus gaemokensis	WP_033675293	-K---K-V	T	-YYEF-TG-------K-----V-TAS---LNL----
Bacillus luti	WP_071709807	-K---K-V	T	-YYEF-TG-------K-----V-TAS---LDL----
Bacillus mobilis	WP_071722003	-K---K-V	T	-YYEF-TG-------K-----V-TAS---LDL----
Bacillus mycoides	WP_131246001	-K---K-V	T	-YYEF-TG-------K-----V-TAS---LDL----
Bacillus paramycoides	WP_071721181	-K---K-V	T	-YYEF-TG-------K-----V-TAS---LDL----
Bacillus pseudomycoides	EEM11909	-K---K-V	T	-YYEF-TG-------K-----V-TAS---LDL----
Bacillus sp. 100374	WP_099683828	-K---K-V	T	-YYEF-TG-------K-----V-TAS---LDL----
Bacillus sp. 123MFChir2	WP_020059651	-K---K-V	T	-MYEY-TG-------K-----V-TAS---LDL----
Bacillus sp. 7_6_55CFAA_CT2	EHL70163	-K---K-V	T	-YYEF-TG-------K-----V-TAS---LDL----
Bacillus sp. AFS018417	WP_098309450	-K---K-V	T	-MYEY-TG-------K-----V-TAS---LDL----
Bacillus sp. AFS059628	WP_098670395	-K---K-V	T	-YYEF-TG-------K-----V-TAS---LDL----
Bacillus sp. AFS094611	WP_098903581	-K---K-V	T	-YYEF-TG-------K-----V-TAS---LDL----
Bacillus sp. GZT	WP_062922121	-K---K-V	T	-YYEF-TG-------K-----V-TAS---LDL----
Bacillus sp. H1a	WP_025147260	-K---K-V	T	-YYEF-TG-------K-----V-TAS---LDL----
Bacillus sp. HBCD-sjtu	WP_101195567	-K---K-V	T	-YYEF-TG-------K-----V-TAS---LDL----
Bacillus sp. LK2	WP_048370474	-K---K-V	T	-YYEF-TG-------K-----V-TAS---LDL----
Bacillus sp. MYb209	WP_105584040	-K---K-V	T	-YYEF-TG-------K-----V-TAS---LDL----
Bacillus sp. N35-10-4	WP_071716571	-K---K-V	T	-YYEF-TG-------K-----V-TAS---LDL----
Bacillus sp. SN10	WP_101165940	-K---K-V	T	-YYEF-TG-------K-----V-TAT---LDL----
Bacillus sp. SRB_28	WP_113770086	-K-----V	T	-YYEF-TG-------K-----V-TAS---LDL----
Bacillus sp. SRB_331	WP_113733916	-K---K-V	T	-YYEF-TG-------K-----V-TAS---LDL----
Bacillus sp. SYJ	WP_131101751	-K---K-V	T	-YYEF-TG-------K-----V-TAS---LDL----
Bacillus sp. ok061	WP_103945624	-K---K-V	T	-YYEF-TG-------K-----V-TAS---LDL----
Bacillus thuringiensis	WP_098250432	-K---K-V	T	-YYEF-TG-------K-----V-TAS---LDL----
Bacillus toyonensis	WP_097854678	-K---K-V	T	-YYEF-TG-------K-----V-TAS---LDL----
Bacillus tropicus	WP_071723317	-K---K-V	T	-YYEF-TG-------K-----V-TAS---LDL----
Bacillus wiedmannii	WP_001252629	-K---K-V	T	-YYEF-TG-------K-----V-TAS---LDL----
Gracilibacillus halophilus	WP_003466247	-R--NK--	T	-LYENSTG-------A-----VSTAE----RL-A--
Jeotgalibacillus campisalis	WP_052476980	-N-----I	T	GMYPH-TG----Y--Q-----V-TAKR--M-L----
Jeotgalibacillus sp. S-D1	WP_133375533	-S-----V	T	GMYPH-TG----Y--L-----V-SAKR--M-L----

Supplemental Figure 21

A partial sequence alignment of the D-alanyl-D-alanine carboxypeptidase protein containing a 21-22 amino acid insertion (boxed) that is exclusively shared by all members belonging to the *Lysinibacillus sensu stricto* clade and absent in all other bacteria. *Lysinibacillus pakistanensis* was not in the NCBI non redundant protein sequences database at the time of writing this paper.

Group	Organism	Accession	128		172
**Jejuensis Clade (3/3)**	Lysinibacillus saudimassiliens	CEA00092	IDFTDIYYQSDFVLVVRKDS	DYIKDQYQEGD	KFVEMDSIQAFKGA
	Lysinibacillus sp. BF-4	WP_036144678	--------------------	-------K---	--------------
	Lysinibacillus jejuensis	WP_108307131	--------------------	-----H-K--E	--------------
**Other Bacteria (0/>300)**	Acetanaerobacterium elongatum	WP_092638080	-----N--K--L-M--K-GG		PYEGAK---D----
	Acetitomaculum ruminis	WP_092873248	L--S-N--T--L-V--K--G		D-AKAKT-ND----
	Aerococcus sp. HMSC035B07	WP_070817350	---S-P--N--I-V-T----		PYANAK-LED-N-G
	Aerococcus sp. HMSC062A02	WP_070616944	---S-P--N--I-V-T----		PYANAK-LED-N-G
	Aerococcus urinaehominis	WP_067977518	---SNS--D-NI--------		PYAQAK-LAD-N--
	Alkaliphilus oremlandii	WP_012158595	-----F--S--L-M-----G		NYTNAE---D----
	Anaerobium acetethylicum	WP_091231173	---S----K--LII--K-GE		AYEDAT--AD----
	Anaerococcus hydrogenalis	WP_037376773	----NS--V--L-V-LK---		P-AKAK-LKD--D-
	Anaerococcus obesiensis	WP_019118511	----NS--I--L-I-LK---		S-AKAK--AD--D-
	Anaerococcus rubeinfantis	WP_058989875	-----S--V--L-I-LK---		S--NAK-LND--K-
	Anaerococcus senegalensis	WP_040398026	----NS--V--L-V-LK---		P-AKAK-LKD--N-
	Anaerococcus sp. SB3	WP_049690117	-----S--V--L-I-LK---		S--NAK-LND----
	Anaerococcus vaginalis	WP_004839318	----NS--I--L-I-LK---		S-AKAK--AN--D-
	Anaerocolumna aminovalerica	WP_091689250	---S-S--K-NL-M--K-GG		VYENAT---D----
	Anaerocolumna jejuensis	WP_073279751	---S-N--K-NL-M--K-GG		A-EKAT---D----
	Anaerocolumna xylanovorans	WP_073587406	-----N--R--L-M--K-GG		PYENAA---D----
	Bacillus acanthi	WP_108671573	---S-N--K-NL-M--K-GG		E-EGAT---D-N--
	Bacillus aciditolerans	WP_121446933	---S-N--T-NL-M--K-G-		TY ---D----
	Bacillus andreraoultii	WP_033826787	--------N-NL-M--K-G-		-YEDAT---D----
	Bacillus azotoformans	WP_003330624	---S-N--K--L-M--K-GG		-YESAT---D-N--
	Bacillus infantis	WP_129613085	---SEN--N-QL-M--K-GG		-YENAT-LND-N--
	Bacillus jeotgali	WP_079507698	---S-N--K-NL-M--K-GG		-YEGAA---D----
	Bacillus kochii	WP_095371434	---S-N--E--L-M--K-GG		DYENAT---D-S--
	Bacillus korlensis	WP_066054123	----EN--K-NL-M--K-G-		-Y AT-LED----
	Bacillus marisflavi	WP_079513996	---S-N--K--L-M--K-GG		-YEGAK---D----
	Bacillus massilioanorexius	WP_026089364	---S-N--K--L-M--KNGG		-Y-DAT---D----
	Bacillus massiliogabonensis	WP_102273497	---S-N--K--LIM--K-GG		-YEGAT---D----
	Bacillus massiliogorillae	WP_042351962	-Y-S-N--K--L-M--KNGG		-Y-NAK---D----
	Bacillus massilionigeriensis	WP_075983312	---S-N--K-NL-M--K-GG		-YEGAT---D----
	Bacillus ndiopicus	WP_042475566	----SN--T----I--K-G-		PY TLAD-S--
	Bacillus nealsonii	WP_016201540	---S-S--K-NL-M--K-G-		-YENAA---E----
	Bacillus niacini	WP_063253979	---S-N--T-NL-M--K-G-		-Y AT---D----
	Bacillus onubensis	WP_099354452	---S-N--T-NL-M--K-GI		TYEGAT---D-N--
	Bacillus praedii	WP_057759910	---S-N--K--LIM--K-GG		-YEGATA--D----
	Bacillus solani	WP_056686904	---SVN--K--LIM--K-GG		-YEGAT---D----
	Bacillus subterraneus	WP_044396022	---S-N--K-NL-M--K-GG		-YENAA---D----
	Bacillus thermoamylovorans	WP_034768266	---S-N--K--L-M--K-G-		PY ---D-S--
	Bacillus timonensis	WP_010677717	---S-N--T-NL-M--K-G-		-YENAT---D----
	Bacillus vireti	WP_024028813	-----N--K-NL-M--K-GG		--DGAS---D----
	Bavariicoccus seileri	WP_022796671	---SNY--A-N--I--Q---		-WTDAT--SD----
	Brevibacillus borstelensis	WP_031932456	---S-N--K-NL-M--K-GG		-YENAT---D----
	Brevibacillus centrosporus	WP_122960459	---S-N--K-NL-M--K-GG		-YEGAT---D----
	Brevibacillus invocatus	WP_122909270	---S-N--K-NL-M--K-G-		-Y AT---D----
	Brevibacillus laterosporus	WP_113756625	---S-N--K--L-M--K-GG		EYENAS---D----
	Brevibacillus nitrificans	WP_122926396	---S-N--K-NL-M--K-GG		-YEGAT---D----
	Brevibacillus panacihumi	WP_051410066	---S-N--K-NL-M--K-G-		-Y AT---D----
	Brevibacillus sp. CFH S0501	WP_134683653	---S-N--K-NL-M--K-GG		-YEGAT---D----
	Brevibacillus sp. OK042	WP_092271660	---S-N--K-NL-M--K-GG		-YEGAT---D----
	Carnobacterium gallinarum	WP_034561101	---S-N--K-HL-M--K-GG		PYENAT---D-A--
	Carnobacterium maltaromaticum	WP_010053924	---S-S--K-NL-M--KNG-		--QSAT---D-E--
	Caryophanon latum	WP_066464248	---S-T--A----I-TKG--		PYAKARTLAD-Q--
	Caryophanon tenue	WP_066542565	---S-T--V----I-TKAG-		EYANAKTLAD-E--
	Cellulosilyticum lentocellum	WP_041712901	---S-N--K--L-M--K-GG		IYENAT---D-S--
	Cellulosilyticum sp. WCF-2	WP_127065666	---S-N--K--L-M-IK-G-		AYENAT---D-S--
	Chlamydia trachomatis	CRH77201	---S-N--K-QL-MI-K-G-		--EQAKTLAD-S--
	Clostridium amylolyticum	WP_073010206	---S-N--K-NL-M--K-GG		-YDGAA-L-D-N--
	Clostridium bartlettii CAG:132	CDA11755	-----V--T--L-M--K-GG		-YENATKLSD----
	Clostridium cadaveris	PWL53284	L-------K--L-ML-K-GG		AYENANTLKD-S--
	Clostridium cuniculi	WP_133016005	---S-N--K--L-ML-K-GG		AYENAKTLSD-S--
	Clostridium gasigenes	WP_089966274	---SNN--K--L-M--K-GG		-YEGAT---D-N--
	Clostridium grantii	WP_073337123	---S-N--K--L-M--K-GG		AYDGAT---D-S--
	Clostridium homopropionicum	WP_052221864	---S-N--K--L-M--K-GG		-YEGAT---D-N--
	Clostridium intestinale	WP_073019368	---S-N--K-EL-M--K-GG		-YNGAT-LKD-S--
	Clostridium nigeriense	WP_066896133	--------K--L-ML-K-GG		AYENAETLKD-S--
	Clostridium tunisiense	WP_017414219	---S-N--K--L-M--K-GG		-YESAS--KD-Q--
	Desnuesiella massiliensis	WP_055666693	---S-N--K--L-M--K-GG		-YDGAT---D----
	Desulfitobacterium chlororespi	WP_072773082	---S-------L-I--KSG-		-YENAT-LKD-N--
	Desulfitobacterium dehalogenan	WP_014793709	---S-------L-M--KNG-		-YENAT-LKD-N--
	Desulfonispora thiosulfatigene	WP_084051867	---S-N--K--L-M--K-GG		E-EKAT---D----
	Desulfosporosinus sp. I2	WP_045576032	---SN---K--L-M--K-GG		-YDGAS---D---S
	Desulfotomaculum aeronauticum	WP_072910560	---S-N--K-NL-M--K-GG		-YEGATA--D----

Other Bacteria (0/>300)

Desulfotomaculum ferrireducens	WP_077713309	---S-N--K--L-M--K-G-	-YENAT--HD----
Desulfotomaculum reducens	WP_011878550	---S----K--L-M--K-GG	-YEGAK---D-N--
Enterococcus aquimarinus	WP_071874930	-----P--E-RL-V-T--N-	E-ANAT-LAD-A--
Enterococcus avium	WP_048717818	V--S-P--E-QL-I-T---T	-YAKAT--KD-A--
Enterococcus canintestini	WP_071864309	----NP--E-HL-I-T----	-YADAKDLAD----
Enterococcus casseliflavus	WP_128432301	-----P--E--L-V-----G	A-ADAK-LADLS--
Enterococcus cecorum	WP_016250940	-----P--E-HL-M-TQ-NG	-YANAK--ED----
Enterococcus columbae	WP_016184389	----KP--E-HL-I-TK-NG	-YANAK--ED----
Enterococcus dispar	WP_016173678	----NP--E-HL-I-T----	-YAKAKDLAD----
Enterococcus durans	WP_005875301	-----P--E-QL-V--Q--G	-YANAK-LADLS--
Enterococcus faecium	WP_099704194	-----P--E-QL-V--Q---	-YAKAK-LADLS--
Enterococcus hirae	WP_010720364	-----P--E-QL-V--K---	-YANAK-LADLSN-
Enterococcus phoeniculicola	WP_010770108	V----A--E-QL-V-TK---	PYAKAK-LKDLS--
Enterococcus pseudoavium	WP_067621809	V--S-P--E-QL-I-T----	-YAKAS--KD-S--
Enterococcus raffinosus	OJG88447	V--S-P--E-QL-I-T---T	-YAKAT--KD-A--
Enterococcus rivorum	WP_084386978	V----V--E-NL-IL--A--	I-AKAT-LKD-S--
Enterococcus villorum	WP_081184517	-----P--E-QL-V--Q-NG	-YANAK-LADLS--
Facklamia hominis	WP_006908527	-----S--T--I-V--K--G	PYA-AK-LKD-A--
Facklamia sp. HMSC062C11	WP_070609721	---S-S--T--I-V--K--G	PYA-AK-LKD-A--
Faecalicatena contorta	WP_109708431	-----V--E-NL-M-I-A--	DYADAK-LTD-S--
Fastidiosipila sanguinis	WP_106012755	---S-T--T--L-M--Q-G-	NYEKAS---E--D-
Finegoldia magna	WP_002835006	---S-T--S--L-I-TK---	-YINAK-LNDL--F
Flexilinea flocculi	WP_062281908	V--S-P--E--L-I--KA--	EYAKAT---D--D-
Fonticella tunisiensis	WP_133628284	---S-N--K--L-M--K-GG	-YEGAS---D-N--
Fusobacterium mortiferum	WP_005885148	-----T----NL-ML-KNGG	DY-NAKTLAD-S--
Fusobacterium necrogenes	WP_115270955	----------NL-ML-KNGG	-YINAKTLAD-S--
Gottschalkia acidurici	WP_014966951	---S-N--K--L-M--K-G-	-YENAA---D----
Granulicatella adiacens	WP_039848743	-----S--D-QL----K-G-	-YANAT-LAD-S--
Granulicatella elegans	WP_006702309	---S-N--K-QL-MI-K-G-	-YEQAKTLAD-S--
Granulicatella sp. HMSC30F09	WP_070438790	-----S--D-QL----K-G-	-YANAT-LAD-S--
Granulicatella sp. HMSC31F03	WP_070444247	-----S--D-QL----K-G-	-YANAT-LAD-S--
Granulicatella sp. WM01	WP_135125993	-----S--M-TL---I---G	AYANAH-LKD-S--
Herbinix hemicellulosilytica	WP_125475862	--------T--L-M--K-GG	-YDNAS---D-A--
Intestinibacter bartlettii	WP_007285363	-----V--T--L-M--K-GG	-YENATKLSD----
Kallipyga gabonensis	WP_053942283	----AP-----L-M-----G	PYTQAK-LED-S--
Kallipyga massiliensis	WP_019134727	----AP-----L-M-----G	LYT-AK-LED-R--
Kurthia huakuii	WP_029500113	---S-V--T-NL-M--K-GG	-YEKAT-LAD--D-
Kurthia sibirica	WP_109305737	---S----T--L-M---A--	-YADAK--KD--D-
Kurthia sp. 3B1D	WP_126991322	---S-V--T-NL-M--K-G-	-YENAT-LAD-S--
Kurthia zopfii	WP_109350703	V--S----A-NL-M--KS-G	-YADAT--DD-S--
Lachnotalea glycerini	PXV91633	--------K--LIM--L-GG	-YENAK---D-S--
Lactobacillus concavus	WP_057823813	-----T--K-KL-II-----	-YANVNDLKD--N-
Lactobacillus dextrinicus	WP_057756250	-----T--H-KL-II---N-	-YAQAT-LKD--N-
Lactobacillus perolens	WP_057817733	----NT--V-NMTI-----G	-YANAT-LKD----
Lactobacillus shenzhenensis	WP_040535284	----NT--V-NMTM-----G	-YANAT-LKD----
Lactobacillus sp. 143-6	WP_125578524	-----T--K-QL-VI----G	--TNAK-LKD--N-
Lactobacillus sp. 17-4	WP_125767682	---S-T--K-QL-II---NG	-YADATKLSD----
Lactobacillus sp. 247-4	WP_125599310	---S-T--A-QL-I------	-YAQATKLSD-S--
Lactobacillus sp. 33-1	WP_125696336	---SST--A-KLTV---R--	-YAKAT--KD-N--
Lactobacillus sp. 54-5	WP_125606341	-----T--R-KL-I------	-YAKAT-LKD--N-
Leptotrichia goodfellowii	WP_006807313	LS--KP--E--L-V--K--G	-YANAK--ND-A--
Levyella massiliensis	WP_019190285	L----A--A--L-I--Q--G	-Y -LED-R--
Lysinibacillus boronitolerans	WP_036078994	----EN--T----M-IK-G-	-YEDAK---D-S--
Lysinibacillus contaminans	WP_053583276	----EN--T----M-IK-G-	-YENAK---D-S--
Lysinibacillus fusiformis	WP_069481286	----EN--T----M-IK-G-	-YEGVK---D-S--
Lysinibacillus macroides	WP_053994816	----EN--T----I--K-G-	E-ETAT---D-S--
Lysinibacillus mangiferihumi	WP_107896074	----EN--T----M-IK-G-	-YEDAK---D-S--
Lysinibacillus parviboronicapi	WP_107925204	----EN--T----M-IK-G-	PY ---D-S-T
Lysinibacillus sp. B2A1	AVK85105	----EN--T----M-IK-G-	-YEKAK---D-S--
Lysinibacillus sp. BK089	WP_132357907	V---EN--T----M-IK-G-	-YEKAT---D-S--
Lysinibacillus sp. FJAT-14222	WP_053595538	----EN--T----M-IK-G-	-YEKAK---D-S--
Lysinibacillus sp. FJAT-14745	WP_053485870	----EN--T----M-IK-G-	EYEKAK---D-S--
Lysinibacillus sp. Marseille-P	WP_106778898	---SEN--T-EY-I-T-A-G	NYANAKTLED-A--
Lysinibacillus sp. SYSU K30002	WP_126658563	----EN--T-E--I--KA-G	-YANAKTLED-T--
Lysinibacillus sp. YR326	WP_134024605	----EN--T----M-IK-G-	-YEKAT---D-S--
Lysinibacillus sp. ZYM-1	WP_054611901	----EN--T----M-IK-GN	-YEGAK---D-S--
Lysinibacillus sphaericus	WP_010859252	----EN--T----M-IK-G-	--ENAK---D-S--
Lysinibacillus sphaericus	WP_024361637	----EN--T----M-IK-G-	-YEDAK---D-S--
Lysinibacillus sphaericus	WP_031418189	----EN--T----M-IK-G-	-YEGVK---D-S--
Lysinibacillus sphaericus	WP_036216118	----EN--T----M-IK-G-	-YEGVK---D-S--
Lysinibacillus sphaericus	WP_069512544	----EN--T----M-IK-G-	-YEDAK---D-S--
Lysinibacillus sphaericus C3-4	ACA40321	----EN--T----M-IK-G-	-YEGVK---D-S--
Lysinibacillus tabacifolii	WP_108030307	----EN--T----M-IK-G-	-YEDAK---D-S--
Lysinibacillus varians	WP_025219554	----EN--T----M-IK-G-	-YEDAK---D-S--

Other Bacteria (0/>300)

Lysinibacillus xylanilyticus	WP_049665415	----EN--T----M-IK-G-	-YEKAT---D-S--
Melissococcus sp. OM08-11BH	WP_117974134	----ND--H-NL----A--G	-YANAK-LKD-E--
Miniphocibacter massiliensis	WP_100065309	----EN--T--L-I--K-EG	NYA-AE---D----
Murdochiella massiliensis	WP_066924464	V---EQ--A--L-M--Q--G	--AHAE-LSD-A--
Murdochiella vaginalis	WP_083428182	V---EQ--A--L-M-----G	-YANAKALAD-R--
Muricomes intestini	WP_132381363	-----V--E-NL-M-TKA--	EYAKAK-LDD-S--
Mycobacteroides abscessus subs	SLL36225	---SEN--T-NL-M--K-G-	--ENAT---D----
Ndongobacter massiliensis	WP_083430915	-----Y--T--L-V--K-GN	AWEKAQTLSD-A--
Neglecta sp. Marseille-P3890	WP_099205570	-----N--T-HL-M--K-G-	AYENAT---D-S--
Oceanivirga salmonicida	WP_067140064	L--SNS--E--VL---K---	PY-NGKTKKD--N-
Paenibacillus antibioticophila	WP_044481308	-----N--K-NLIM--K-GG	AY AT---D-S--
Paenibacillus barengoltzii	WP_016313029	---S-N--K-NLIM--K-G-	PY AT---D-S--
Paenibacillus humicus	WP_127509453	---S-N--K-NL-M--K-GG	-YEGAT---D-N--
Paenibacillus phocaensis	WP_068786787	---S-N--K-NL-M--K-G-	SYENAT---D-S--
Paenibacillus rubinfantis	WP_059046460	---S-N--K-NL-M--K-G-	SY AT---D-S--
Paenisporosarcina quisquiliaru	WP_090567518	---SEN--T-N--M--K-GG	A-EGAT---D-S--
Paenisporosarcina sp. HGH0030	EPD50976	---S-N--T-EL-M--K-GG	-YEGAA--HD----
Paenisporosarcina sp. HGH0030	WP_036659550	---S-N--T-EL-M--K-GG	-YEGAA--HD----
Paenisporosarcina sp. OV554	WP_108585867	---S-N--T--L-M--K-GG	EY ---D----
Peptoniphilus grossensis	WP_019125467	---SNT--T--L-I--K---	PYANAK---D----
Peptoniphilus harei	WP_126438824	L--S-T--T--L-V--K---	PL-NAK--ND-A--
Peptoniphilus phoceensis	WP_062551648	L--SNT--T--L-I--K--G	AYANAS-LND----
Peptoniphilus senegalensis	WP_019108348	---SNT--T--L-I--K---	PYANAK-L-D----
Peptoniphilus vaginalis	WP_085875329	L--S-T--T--L-I--K---	PYANAK--ND----
Planomicrobium glaciei	WP_074509043	----EP--T-EL-M--K-GG	EY AT-LSD-S--
Pseudoclostridium thermosuccin	WP_103089464	-----D--K--L-M--K-G-	-YENAT---D----
Psychrobacillus insolitus	WP_111440168	---SEN--T----MI-K-GG	P-EGAT---D-S--
Psychrobacillus psychrodurans	WP_093495510	---SEN--T-N--M--K-GG	P-EGAT---D-S--
Psychrobacillus psychrotoleran	WP_093537965	---SEN--T-N--M--K-GG	A-EGAT---D-S--
Psychrobacillus sp. FJAT-21963	WP_056831356	---SEN--T-N--M--K-GG	PYEGAT---D-S--
Psychrobacillus sp. OK028	WP_093062373	---SEN--T-N--M--K-GG	P-EGAT---D-S--
Psychrobacillus sp. OK032	WP_093275247	---S-N--T-N--M--K-GG	PYEGAT---DL---
Rhodococcus qingshengii	WP_133370605	---S-N--T-NL-M--K-G-	-YEGAT---D----
Rummeliibacillus	WP_119416585	---S----K-NL-M--K-G-	-YDGAT--TD----
Rummeliibacillus stabekisii	WP_066791034	---S-N--T-HL-M--K-G-	-YENAT--ED-N--
Sebaldella termitidis ATCC 333	ACZ09159	L---SP--K--L-V--A--G	-YANAKTLED-Q--
Senegalia massiliensis	WP_130807598	---SNN--K--L-MI-K-G-	-YEDST--ND----
Sneathia amnii	WP_046328321	L---KS--D--I-V--K--G	--KDAKTLND----
Sporanaerobacter	WP_071138688	---S-N--K--L-M--K--G	AYEKA--LND----
Sporosarcina sp. HY008	WP_067408500	---SEN--T-N--MI-K-GG	PYEGAT---D-S--
Streptobacillus hongkongensis	WP_066896437	LE--SP--E--L-V------	-YINAKKLDDL---
Streptobacillus moniliformis	WP_012858628	LE---P--E--L-I-IK---	-YLNANN--E-SNS
Streptococcus sp. 263_SSPC	WP_048782710	-----S--D-QL----K-G-	-YANAT-LAD-A--
Tepidanaerobacter acetatoxydan	WP_013779017	---STN--K--L-M--K-GG	-YEGAT---D----
Tetragenococcus solitarius	WP_068710231	-----P--E--L-I-T----	DYAGAK-LDD----
Tissierella creatinophila	WP_075726550	V--S-G--T--L-M--K-GG	PYEAAKKLED----
Tissierella praeacuta	WP_072977429	-----N--K-NL-M--K-GG	-YENAT---D----
Tissierella sp. P1	WP_094903526	---SEN--K-NL-M--Q-GG	-YEDAT---D----
Ureibacillus thermophilus	QBK26013	-----N--T----I--K--G	PYANAK--HD----
Vagococcus acidifermentans	RSU11730	-----T--R-HL-ML-K-G-	A-EQASQLAD--H-
Vagococcus fluvialis	WP_114290428	L--S-Y--S-QLTIL-K---	PYANAT-LKD----
Vagococcus humatus	WP_125944042	-----V--K-HL-ILTK-GG	DYA-AT-LAD--H-
Vagococcus lutrae	WP_126763173	---S-V----HL--L-K-GG	P-E-ATRLSD--Q-
Vagococcus martis	WP_079346619	----ND--H-NL----A--G	-YANAK-LKD-E--
Vagococcus penaei	WP_126844211	V----V--N-NL-ML-NSEG	N-TDAK-LAD--D-
Vagococcus sp. AM17-17	WP_118250975	----ND--H-NL----A--G	-YANAK-LKD-E--
Vagococcus sp. SS1994	WP_125957888	----ND--H-NL----A--G	-YANAK-LKD-E--
Vagococcus sp. SS1995	RST96764	-----V--T-NL-MLINSEG	NYKNAT-LDD----
Vagococcus sp. SS1995	WP_125984725	-----V--T-NL-MLINSEG	NYKNAT-LDD----
Vagococcus teuberi	WP_071455991	----ND--H-NL----A--G	-YANAK-LKD-E--
Varibaculum timonense	WP_080913359	---S----T--L-M--K-G-	-YESAK---D-S--
Viridibacillus arvi	WP_053418467	-----N--T-NY-M--K-G-	-YEKAT---D-S--
Viridibacillus sp. FSL H8-0123	WP_076065731	-----N--T-NY-M--K-G-	-YEKAK---D-S--
Viridibacillus sp. OK051	WP_100795323	-----N--T-NY-M--K-G-	-YEKAT---D-SD-
Youngiibacter fragilis	WP_023388031	---S-N--K--LIM--K-GG	AYENAKTLKD-T--

Supplemental Figure 22

A partial sequence alignment of the Arginine-binding extracellular protein ArtP precursor containing an 11 amino acid insertion (boxed) that is exclusively shared by all members belonging to the Jejuensis clade and absent in all other bacteria.

Group	Organism	Accession	218		268
Jejuensis Clade (3/3)	Lysinibacillus jejuensis	WP_108307498	EGEMYDVLMREMEAAGLAQYEISNF		GQNSVHNTIYWDNDEYIGIGAGAHGY
	Lysinibacillus sp. BF-4	WP_036144454	---------------NV--------		--------------------------
	Lysinibacillus saudimassiliensis	CEA00002	---------------NV--------		--K-----------------------
Other Bacteria (0/>200)	Alteribacillus iranensis	WP_091656108	-A----L--SKL-K--FSA------	ARN	-YE-K--KT--K-E--Y-F-------
	Bacillus abyssalis	WP_078413828	-AK---E--MQ--KH-FK-------	AKD	-FE-R--LT--N-E--Y---------
	Bacillus acanthi	WP_108669592	-AA--EL--EQ-DKK--I-------	AKP	-FE-K--LT--N--Q-F-F-------
	Bacillus acidiceler	WP_129688146	-AA---F--NYL-KK-FH-------	EKN	-LE-K--LV--N-E--F-F-------
	Bacillus acidicola	WP_066262048	-AN--SL--N---K--YH-------	AKP	-FE-R--LV--N-E--F-F-------
	Bacillus acidiproducens	WP_018661406	-AQ---I--N---KH-YR-------	ARP	-FE-I--LV----E--F-F-------
	Bacillus aciditolerans	WP_121447259	-AR--EI--A---EH-FR-------	SKD	-YE-R--LT--N-E--Y-------S-
	Bacillus amyloliquefaciens	WP_072176719	-A---ETV--Q-REV-IR-------	AKP	-YE-Q--LT--S-E--F-F-------
	Bacillus aquimaris	WP_044339596	-AA--EI-IET--KY-IN-------	AKN	-FE-K--LV----N--Y-L-------
	Bacillus aryabhattai	WP_045292086	-AA--EL--K--DKQ-FR-------	AKP	-YE-I--LT--N--D-Y-------S-
	Bacillus butanolivorans	WP_053347015	-AS--ER--E---KH--H-------	ARP	-FE-R--LT----V--Y---------
	Bacillus camelliae	WP_101354104	-AR---I--T---KH--H-------	AKP	-YE-K--LV----E--F-F-----S-
	Bacillus cecembensis	WP_057989649	-AD--G---DT-Q-H--Q-------	AQD	-FL-T--K--------A-F-------
	Bacillus coagulans	WP_133536702	-AD--SM--D---KH--K-------	AKP	-YE-R--LV--N-E--F-F-------
	Bacillus coahuilensis	WP_059351314	-AS------NT--KK-IS-------	AKK	-YE-K--LV--S-E--Y-F-----S-
	Bacillus dakarensis	WP_077214031	-AS--GT--E---KH-YR-------	SKP	-YE-N--IT--N----Y-------S-
	Bacillus firmus	WP_035331171	-A----L--EQ-DKH-F--------	AKQ	-YE-R--IT----EW-F-F-------
	Bacillus foraminis	WP_121611683	-A---E---EQ--LH--K-------	ALQ	-YE-R--LT--E-E--F-F-----S-
	Bacillus fordii	WP_026319694	-AA--EI-IEQ--KN--K-------	SKA	-FQ-T--LV--N----F-F-------
	Bacillus fortis	WP_120068995	-AA--EI-IEQ--KS--Q-------	SKP	-FQ-I--LV--N----F-F---S---
	Bacillus freudenreichii	WP_126431847	-AA--N--IE---RS--R-------	AKS	-FQ-I--LV--N-E--F-F-------
	Bacillus funiculus	WP_129727322	-AQ--EIV-D---KN-YH-------	SKA	-YE-R--LT--N-E--Y-F-------
	Bacillus galactosidilyticus	KRG16787	-AD--R---E---KK--Q-------	SRP	-YE-K--L---N-E--Y-F-------
	Bacillus ginsengihumi	WP_025726657	-AD--QL--M--DKH--N-------	ALP	-YE-Q--L---N-E--F-F-------
	Bacillus glycinifermentans	WP_048355477	-A---ELV-KT-AE--IH-------	AKK	-FE-R--LT--S-E--F-F-------
	Bacillus halotolerans	WP_105990417	-A---ETV-SR-----IH-------	AKQ	-ME-K--LT--S-E--F-F-------
	Bacillus haynesii	WP_043928582	-A---ELV--K--E--IG------Y	AKK	-YE-K--LT--N-ED-F-F-------
	Bacillus horikoshii	WP_064100014	-TS-----LN---KH-YH-------	AKE	-YE-K--LT----ES-Y---------
	Bacillus indicus	WP_029279548	-AA--EL--E--DKH-F--------	AKP	-YE-M--LT--N-E--F-------S-
	Bacillus intestinalis	WP_079287976	-A---EMV--R---H-IH-------	AKA	DLE-K--LT--S-E--F-F-------
	Bacillus lentus	WP_066137364	-T--FTI--E---KK--R-------	SEP	-FE-K--L---N-E--F-F-------
	Bacillus licheniformis	WP_017474324	-A----LV--K--E--IG------Y	AKK	-YE-K--LT--N-ED-F-F-------
	Bacillus marisflavi	WP_121620784	-A----L-IE---K--MH-------	SKK	-YE-L---V----E--Y-F-------
	Bacillus mediterraneensis	WP_071460184	-A---EI--D---RH-RE-------	AVP	-YE-I--LT--N-E--Y-F-------
	Bacillus megaterium	WP_043976183	-AA--EL--K--DKQ-FH-------	AKP	-YE-I--LT--N--D-Y-------S-
	Bacillus mojavensis	WP_010334998	-A---ETV-SR--K--IH-------	AKQ	-ME-K--LT--S-E--F-F-------
	Bacillus muralis	WP_057911680	-AS--ER--E---KH-IH-------	AKA	-FE-R--LT----V--Y---------
	Bacillus ndiopicus	WP_042478180	-AD------H---V-----------	ARE	-FA----K--------A-F-------
	Bacillus niameyensis	WP_062104630	-AK--E---ET--NH----------	SKS	-YE-R--LV--N-E--F-F-------
	Bacillus oceanisediminis	WP_110066107	-A---AL--EQ-DKH-FK-------	AKP	-HE-R--IT----EW-F-F-------
	Bacillus onubensis	WP_099352209	-AR--EI--A---KH-FH-------	SKD	SYE-R--LT--N-E--Y-------S-
	Bacillus paralicheniformis	WP_101561096	-A---ELV--K--E--IG------Y	AKK	-YE-K--LT--N-E--F-L-------
	Bacillus persicus	WP_090741086	-AQ--EL--DA--KK--K-------	ALP	-FE-Y--LT--R-E--F-F-------
	Bacillus safensis	WP_111291681	-A---ELV-D---SH--K------Y	AKP	-FE-Q--LT--S-ED-F-F-------
	Bacillus selenatarsenatis	WP_041966908	-A---ET--EQ--I---R-------	AVQ	-FE-R--LT--N-E--Y-F-------
	Bacillus shackletonii	WP_055739223	-AR--EI--T---KH--H-------	AKP	-FE-K--LV--N-E--F-F-----S-
	Bacillus siamensis	WP_029575368	-A---ETV--Q-SE--IR-------	AKP	-YE-Q--LT--S-E--F-F-------
	Bacillus simplex	WP_061144000	-AS--EK--ED--KQ-IH-------	ARP	-FE-R--LT----V--Y---------
	Bacillus smithii	WP_048623718	-A----L--D---RH-IH-------	AKK	-YE-K--LV--N-G--Y-F-----S-
	Bacillus sp. 2_A_57_CT2	WP_009333020	-A---EL--EQ-GKH-F--------	AKP	-YE-R--IT----EW-F-F-------
	Bacillus sp. 3-2-2	WP_126051321	-AA--N--IE---RS--R-------	AKS	-FQ-I--LV--N-E--F-F-------
	Bacillus sp. 7894-2	WP_095245636	-A----L-IEQ-DKH-F--------	AKP	-YE-K--IT----EW-F-F-------
	Bacillus sp. mrc49	WP_100530827	-AS--EK--E---KQ-IH-------	ARP	-FE-R--LT----V--Y---------
	Bacillus subterraneus	WP_044395389	-AQ---T-IDQ--KH--H-------	AIP	-YE-R--LT--N-E--Y-F-------
	Bacillus subtilis	WP_100506289	-A---EMV-SQ---H-IH-------	AKA	-ME-K--LT--S-EQ-F-F-------
	Bacillus swezeyi	WP_076761922	-A---ELV-QK-NE--IS------Y	AKK	-YE-K--LT--S-E--F-F-------
	Bacillus terrae	WP_120114917	-AA--EM-INQ--KS--Q---V---	SKP	-FQ-I--LV--N-E--Y-F-------
	Bacillus vallismortis	WP_010330719	-A---EMV-SR---H-IH-------	AKR	-ME-K--LT--S----F-F-------
	Bacillus velezensis	WP_025649831	-A---ETV--Q-SE--IR-------	AKP	-YE-Q--LT--S-EA-F-F-------
	Bacillus vietnamensis	WP_061811130	-AA--EI-IET--KY-IN-------	AKP	-FE-K--LV----N--Y-L-------
	Bacillus wudalianchiensis	WP_065408999	-AS--EL--E--A----R-------	AIP	-FE-L--LV--N-EY-Y-F-----A-
	Bacillus xiamenensis	WP_008356227	-A---ELV-D---RH--K------Y	AKP	-FE-Q--LT--S--D-F-F-------
	Bacillus zeae	WP_119113566	-A---EI--E--DRH--K-------	AIP	-YE-R--LT--N-E--F-F-----S-
	Butyricicoccus sp. 1XD8-22	RKJ36553	-A----I--KK--LHD-K-------	AKD	-HI-T--K--------V---------
	Caldibacillus debilis	KYD11120	-AD--ERI-E---KR--H-------	AKK	-FE-M--R-----EN-Y-F-----S-
	Caryophanon latum	WP_066460926	-AT--GH-ID---RH--K-------	AKD	-YA----K--------A-F-------
	Caryophanon tenue	WP_066542639	-AD--GR--D---RH--H-------	AQP	-YD-I--K--------A-F-------
	Chryseomicrobium excrementi	WP_100352980	-AQ------TRTKEH-FE-------	AKN	-LY-E--S---R-E--L-L-------
	Domibacillus robiginosus	WP_050180941	-AD--EAV-----LQ--H-------	AQP	-FE-L--IT--N----Y-F-------
	Domibacillus tundrae	WP_046178749	-AD--EAV----A-G--N-------	ARP	-FE-L--LT--N----Y-F-------
	Exiguobacterium oxidotolerans	WP_029332896	-A---RLMIETI--G--K-------	SLP	-KE-R--RV--E----Y-F---S-S-
	Exiguobacterium sibiricum	WP_026829022	-AD--RLMIETI--G--Q-------	SLP	-RE-R--RV--E----Y-F---S-S-
	Exiguobacterium sp. B203-G5 25	WP_131973228	-AD--RLMIETI--G--H-------	SLP	-RE-R--RV--E----Y-F---S-S-
	Exiguobacterium sp. HF60	WP_114570449	-AD--RLMIERI--G----------	SLP	-KE-R--RV--E----Y-F---S---
	Exiguobacterium sp. N4-1P	WP_088836523	-A---RLMIETI--G--K-------	SLP	-KE-R--RV--E----Y-F---S-S-
	Exiguobacterium undae	WP_028106821	-AD--RLMIETI--G--H-------	SLP	-RE-R--RV--E----Y-F---S-S-
	Falsibacillus pallidus	WP_114744919	-A---GL--EK--VS--K-------	AKP	-FE-R--LV--N-EQ-F-F-----S-

Other Bacteria (0/>200)

Fictibacillus macauensis	WP_040340527	AR--E---E---KH-YT-------	AKK	-YE-K--LQ--N-N--Y---------
Fictibacillus sp. FJAT-27399	WP_062234849	-AQ--E---EQ--KH-YV-------	ARP	-YE-K--LQ----N--Y-------S-
Halobacillus dabanensis	WP_075035083	-A---EL-Q-KLT---AV-------	AKP	-FE-K--LT--N-EH-Y---------
Jeotgalibacillus campisalis	WP_041059462	-AG--EL--KK-KER--N-------	SKK	-FE-R--IV--N----F-F-------
Jeotgalibacillus proteolyticus	WP_104056955	-AR--EM--DK-KS--YG-------	AKP	-FE-K--IV----S--F-F-------
Jeotgalibacillus salarius	WP_134381718	-AA---S--NK-A-K--E-------	SKT	-YE-I--LT--E-ES-Y-F-------
Jeotgalibacillus soli	WP_041090641	-AS--EL--EK---H-RK-------	ARP	-YE-K--LV-------Y-F-G-----
Jeotgalibacillus sp. R-1-5s-1	WP_134376679	-AA--EL--KQ-----RN-------	ALP	-FE-E--KV--K-ES-F-L-------
Jeotgalibacillus sp. S-D1	WP_133375331	-AQ--EL--KK-AEH----------	AKS	-FE-R--LV----A--F-F-------
Kurthia gibsonii	WP_121178020	-AA--AL--K---KR--H-------	GRT	DFE-K--L--------A-F----S--
Kurthia huakuii	WP_029499231	-AK---M--QQ---H--T-------	AKK	-FE-T--C---E----A-F----S--
Kurthia massiliensis	WP_010288001	-AK---M--KQ---H--T-------	ARP	-CE-I--C---E----A-F----S--
Kurthia senegalensis	WP_010303425	-A---EM--QQ--SN--M-------	AKR	-LE----C---E----A-F----S--
Kurthia sibirica	WP_109306545	-AD--NY--DT--KS--E-------	GKA	-HE-L--L-----E--A-F----S--
Kurthia sp. 11kri321	WP_068452556	-AA--AL--K---KR--H-------	GRT	DFE-K--L--------A-F----S--
Kurthia sp. 3B1D	WP_126991148	-AK---M--HQ---H--K-------	AKP	-FE-T--C---E----A-F----S--
Kurthia zopfii	WP_109348351	-AT--EM--AQ---H--H-------	AKP	EHE-A--L-----E--A-F----S--
Listeria aquatica	WP_036074039	-AN---Y--S---K--KK-------	AET	-FE-R--LV--S-EH-F-F-------
Listeria costaricensis	WP_099221170	-AN---Y--T---R--KS-------	AAK	-YQ-R--LV--S-EH-F-F-------
Listeria grayi	WP_003755793	-AN---T--N---KH-RR-------	AKP	-FE-R--IV--S-EH-Y-F-------
Listeria kieliensis	WP_115752337	-AN---Y--S---K--KK-------	AEP	CFQ-R--LV--S--H-F-F-------
Listeria thailandensis	WP_122865473	-AN---Y--S---K--KK-------	AEP	CFQ-R--LV--S--H-F-F-------
Lysinibacillus acetophenoni	WP_097148327	-A----L--N---RN-HH-------	ARD	-LI-T--KV-----------------
Lysinibacillus boronitolerans	WP_016992467	-AD------QQ-ALQ--Q-------	AKP	-YH-E--K--------A-F-------
Lysinibacillus chungkukjangi	WP_107935392	-AD---L---R-KEKEKI-------	AID	-H--T--K--------A-F-------
Lysinibacillus composti	WP_124762148	-A---GL--NK-K-N--N-------	ASK	-FE-I--K--------A-F-------
Lysinibacillus contaminans	WP_053582788	-A-------Q----H--H-------	AYE	-FS----K---E----A-F-------
Lysinibacillus endophyticus	WP_121213336	-AD---L--NK--QN--K-------	ALD	-YQ-T--K--------A-F-------
Lysinibacillus fluoroglycofeni	WP_107942464	-AD---M-L----------------	ARD	-FA----K--------A-F-------
Lysinibacillus fusiformis	WP_069482038	-AD------QQ--LH--Q-------	AKP	-YH-E--K--------A-F-------
Lysinibacillus halotolerans	WP_122971358	-AD--EL--KK-AEKQKE-------	AMD	-HI-T--K--------A-F-------
Lysinibacillus macroides	WP_053996201	-AD------QQ--LQ--Q-------	AKA	-FE-G--K--------A-F-------
Lysinibacillus manganicus	WP_036186301	-A----Y--N---RNN-H-------	AKG	DNI-T--K--------V---------
Lysinibacillus mangiferihumi	WP_107893893	-AD--E---Q--KLH-FM-------	AKA	-FT-T--K---E----A-F-------
Lysinibacillus massiliensis	WP_036176046	-A----I--KK--LHD-K-------	AKD	-HI-T--K--------V---------
Lysinibacillus meyeri	WP_107841784	-AD---M----------V-------	AEE	-NE----K--------A-F-------
Lysinibacillus odysseyi	WP_036157755	-AD------K----HD---------	AKP	-FA-I--K--------A-F-------
Lysinibacillus parviboronicapi	WP_107926116	-A-------Q----H--Q-------	AQP	-FA-T--N---E----A-F-------
Lysinibacillus sinduriensis	WP_036201792	-AD---L---R--EKEKK-------	AID	-HI-T--K--------A-F-------
Lysinibacillus sp. 2017	WP_108712003	-AN--G---E----H--H-------	AHK	-YE-T--K--------A-F-------
Lysinibacillus sp. AR18-8	WP_066036451	-AD------QQ-ALH--Q-------	AKP	-YH-E--K--------A-F-------
Lysinibacillus sp. BK089	WP_132357274	-AD------NQ---H--Q-------	AQS	-FS-K--K---E----A-F-------
Lysinibacillus sp. FJAT-14222	WP_053595275	-AD------NQ--VH--Q-------	AQA	-FS-S--K---E----A-F-------
Lysinibacillus sp. FJAT-14745	WP_053484678	-AD------NQ---HAHQ-------	AQL	-FS-R--K---E----A-F-------
Lysinibacillus sp. LK3	WP_048392178	-AD------QQ-ALH--Q-------	AKP	-YH-E--K--------A-F-------
Lysinibacillus sp. Marseille-P	WP_106780024	-A----L--K---RN--E-------	AKN	-LI-T--K--------V---------
Lysinibacillus sp. OL1	WP_131520544	-AD------QQ-ALH--Q-------	AKP	-YH-E--K--------A-F-------
Lysinibacillus sp. PB300	WP_115674113	-AD------QQ-ALH--Q-------	AKP	-YH-E--K--------A-F-------
Lysinibacillus sp. SYSU K30002	WP_126657175	-AD---L--KR-GKS--Q-------	ALE	-YQ-T--K--------A-F-------
Lysinibacillus sp. YLB-03	WP_118875621	-A----L---R--DNHKQ-------	AIE	-RQ-T--K--------A-F-------
Lysinibacillus sp. YR326	WP_134018471	-AD------NQ---H--Q-------	AQS	-FS-K--K---E----A-F-------
Lysinibacillus sp. YS11	WP_103118454	-AD------QQ-ALH--Q-------	AKP	-YH-E--K--------A-F-------
Lysinibacillus sp. ZYM-1	WP_054609301	-AD------QQ--LH--Q-------	AKP	-YH-E--K--------A-F-------
Lysinibacillus sphaericus	POZ55659	-AD--E---Q--RLH-FK-------	AKA	-FT-T--K---E----A-F-------
Lysinibacillus sphaericus	WP_010861218	-AD------Q--D-H--Q-------	AKP	-FA-T--K---E----A-F-------
Lysinibacillus sphaericus	WP_012295330	-AD------QQ--LH--Q-------	AKP	-YH-E--K--------A-F-------
Lysinibacillus sphaericus	WP_031417052	-AD------QQ--LH--Q-------	AKP	-YH-E--K--------A-F-------
Lysinibacillus sphaericus	WP_036121703	-AD------QQ-ALH--Q-------	AKP	-FH-E--K--------A-F-------
Lysinibacillus sphaericus	WP_036221387	-AD------QQ--LH--Q-------	AKP	-YH-E--K--------A-F-------
Lysinibacillus sphaericus	WP_054550092	-AD------QQ-ALH--Q-------	AKP	-YH-E--K--------A-F-------
Lysinibacillus sphaericus	WP_069513478	-AD--E---Q--RLH-FK-------	AKA	-FT-T--K---E----A-F-------
Lysinibacillus sphaericus	WP_099806032	-AD------QQ--LH--Q-------	AKP	-YH-E--K--------A-F-------
Lysinibacillus sphaericus	WP_112118209	-AD------QQ-ALH--Q-------	AKP	-YH-E--K--------A-F-------
Lysinibacillus sphaericus	WP_125103638	-TV-FAE-IDR--R--RS-------	AIP	-RE-I--M---E----A---------
Lysinibacillus tabacifolii	WP_108029804	-AD--E---Q--KLH-YL-------	AKA	-FT-T--K---E----A-F-------
Lysinibacillus telephonicus	WP_126292787	-AD---L--KK-DEK-IR-------	ALD	-HI-T--K--------A-F-------
Lysinibacillus xylanilyticus	WP_100545938	-AD------NQ---H--Q-------	AQS	-FS-K--K---E----A-F-------
Lysinibacillus xyleni	WP_097074522	-A----L---K--EN--K-------	ALD	-YQ-T--K--------A-F-------
Paenibacillus chondroitinus	WP_127449827	-VD--ELI-KRL----YK-------	AKP	-RE-R---M--R-RS-Y-L-------
Paenibacillus sp. FSL R5-0490	WP_076257438	-A----L--EQ-DKH-F--------	AKP	-YE-R--IT----EW-F-F-------
Paenisporosarcina antarctica	WP_134209531	-AD--E---N---SK-IN-------	AKP	DFH-K--NL----VS-A-F-------
Paenisporosarcina quisquiliaru	WP_090563852	-A---SY-LE--KNH-YT-------	AYE	-KE-K--LL--N------L-------
Paenisporosarcina sp. HGH0030	WP_036659638	-AD------T--KSR-IH-------	AKP	-FH-K--NL----V--A-F-------
Paenisporosarcina sp. OV554	WP_108586326	-A---E---TS--SK-IH-------	AQP	-FH-K--NL----VS-A-F-------
Planococcus antarcticus	WP_006828706	-AD--EK--N--DKR--K-------	ARP	-HE-H--LL----V----V-------
Planococcus citreus	WP_121300040	--D--ER--D---KH--H-------	AKP	-HE-R--LL----E----V-------
Planococcus donghaensis	WP_065526552	-AD--EK--N--DKR--K-------	ARP	-HE-H--LL----V----V-------
Planococcus faecalis	WP_071154367	-AD---K--N--DKR--K-------	ARP	-HE-R--LL----V----V-------
Planococcus halocryophilus	WP_008498985	-AD--EK--N--DKR--K-------	ARP	-HE-H--LL----V----V-------
Planococcus halotolerans	WP_112223796	-AD--QK--T--NQR--L-------	ARE	-ME-H--LL----E----A-------

Other Bacteria (0/>200)

Planococcus kocurii	WP_058385574	-AD---K--N--DKR--K-------	ARP -HE-R--LL----V----V-------
Planococcus maitriensis	WP_112232385	--D--ER--D---KH--H-------	AKP -HE-R--LL----E----V-------
Planococcus maritimus	WP_068461942	--D--ER--D--KKH--H-------	AKP -HE-R--LL----E----V-------
Planococcus massiliensis	WP_052651808	-AD--EK--A---QR--K-------	GRP -FQ-R--L-----E----A-------
Planococcus plakortidis	WP_068869010	--D--ER--D--AKQ--H-------	AKP -HE-R--LL----E----V-------
Planococcus rifietoensis	WP_058380943	--D--ER--D---KH--H-------	AKP -HE-R--LL----E----V-------
Planococcus salinarum	TAA68333	-AD--AK--N---QH--L-------	ARP -FE-R--LL----E----A-------
Planococcus salinus	WP_123163756	-AA---K--A--DRH--E-------	AKP -FE-H--LL--N-E----A-------
Planococcus sp. CAU13	WP_033543572	-AD--ER--Q---SR--G-------	ARP -FE-R--LL----V----A-------
Planococcus sp. PAMC 21323	WP_038703673	-AD--EK--N--DKR--K-------	ARP -HE-H--LL----V----V-------
Planococcus sp. Y42	WP_077588837	-AD--K---D--A-H--E-------	AKS -YQ-R--LL--N-E----A-------
Planococcus versutus	WP_065524161	-AD--EK--N--DQH--K-------	ARP -HE-H--LL--N-V----V-------
Planomicrobium flavidum	WP_088008038	-A---R---ER-A-H--H-------	AKP -RK-R--LL--N-E----A---S---
Planomicrobium glaciei	WP_036809507	-AD--EK--A---KR--K-------	GRP -FE-R--LL----A----V-------
Planomicrobium okeanokoites	WP_117312164	-AD--EK--T--NQR--L-------	ARE -ME-R--LL----E----A-------
Planomicrobium soli	WP_106532722	-AD---K--D--DIR--K-------	ARP NFE-R--LL----A----V-------
Planomicrobium sp. MB-3u-38	WP_101803725	-AD--EK--T--NQR--L-------	ARE -ME-R--LL----E----A-------
Planomicrobium sp. Y74	WP_121633602	-AD--EK--T--NQH--L-------	ARE -ME-H--LL----E----A-------
Psychrobacillus insolitus	WP_111437849	-A---GY--D--KKQ-YS------Y	SHE -KQ-K--L--------F-F-------
Psychrobacillus psychrodurans	WP_093496125	-A---SY-LE--KNH-YT-------	AYE -KE-K--LL--N------L-------
Psychrobacillus psychrotoleran	WP_093538492	-A---GY-LE--QSH-YI-------	AYV -KE-K--LL--N------L-------
Psychrobacillus sp. FJAT-21963	WP_056832094	-AK--GY-LD--TKQ-YN-------	AKE -KE-K--L----------L-------
Psychrobacillus sp. OK028	WP_093060600	-A---SY-LE--KSH-YS-------	AYE -KE-K--LL--N------L-------
Psychrobacillus sp. OK032	WP_093266112	-A---SY--D--KMN-YD-------	AHK -KE-K--LL--N------L-------
Rummeliibacillus pycnus	WP_102691275	-A----L--QQ--QQ--H-------	SKK -YS-I--Q--------A-F-------
Rummeliibacillus sp. POC4	WP_119414809	-A-------QQ--QH--H-------	SKE -YS-L--R--------V-L-------
Rummeliibacillus sp. TYF005	WP_124217192	-A-------QQ--QH--H-------	SKD -YS-L--R--------V---------
Rummeliibacillus stabekisii	WP_066786283	-A-------E--KKH-YH-------	SKP -YP-I--Q--------A-F-------
Sediminibacillus halophilus	WP_026771149	-A---QL-TEQ---H----------	AKP -YE-R--LT--N--H-Y-M-------
Solibacillus isronensis	WP_079525552	-AD--G---DT---H--R-------	ANE -YE-T--K--------A-F-------
Solibacillus kalamii	WP_087615339	-TD--S---DT---H--K-------	ANE -YA-T--K--------A-F-------
Solibacillus silvestris	WP_014824437	-TD--S---DT---H--K-------	ANE -YE-T--K--------A-F-------
Solibacillus sp. R5-41	WP_099422881	-A---G---DT-Q-H--H-------	AHE -FL-T--K--------A-F-------
Sporosarcina globispora	WP_053434339	-A---EL--EH-DKH-F--------	AKS -FE-R--IT----EW-F-F-------
Sporosarcina koreensis	WP_040286658	-TA-FES-ISR--Q--RG-------	AIP -HE----M---E----A-V-------
Sporosarcina sp. EUR3 2.2.2	WP_024534458	-AD--E---HV--SK-VH-------	AQP -YH-I--NL----VS-A-F-------
Streptococcus castoreus	WP_027970242	-A--F-YILS----N-FEH------	TKP -FE-R--LV--N----F-C----S--
Streptococcus pseudoporcinus	WP_077321784	-A--F-YIIQ----H-FEH------	GKP -YY-Q--LM--N----Y-C----S--
Tetzosporium hominis	WP_094942121	-A---E----RT-EH-FE-------	AKK -LY-E--S---R-E--L-L-------
Ureibacillus thermophilus	QBK26290	-AK--EM--D---EK-IH-------	AKE -YE-I--K--------A-F-------
Ureibacillus thermosphaericus	WP_016838580	-AK---L--ER--DS--S-------	AKE -FQ-L--K--------A-F-------
Vagococcus entomophilus	RSU08533	--D-FELAASK-S------------	ARA -KE-Q--LV----EQ-Y-F----S--
Vibrio vulnificus	WP_133352863	-AL--ER--E---KH--H-------	ARA -FE-R--LT----V--Y---------
Viridibacillus arenosi	WP_038187166	-AD--EE--QQ---H-WH-------	AKD -HS-I--R--------A-F-------
Viridibacillus arvi	WP_053416411	-AD--EE--QQ--TH-WH-------	AKE -HS-I--R--------A-F-------
Viridibacillus sp. OK051	WP_100794307	-AD------QQ--EH-FH-------	SKD -HS-I--R--------A-F-------

Supplemental Figure 23

A partial sequence alignment of the oxygen-independent coproporphyrinogen III oxidase protein containing a three amino acid deletion (boxed) that is exclusively shared by all members belonging to the Jejuensis clade and absent in all other bacteria.

Group	Species	Accession	110		160
Jejuensis Clade (3/3)	Lysinibacillus saudimassiliensis	CEA00796	LFHYSEAFAAAILHHPMVPLRNHVLP		QQATRVFIAAGDNDPLCPKEEAIEL
	Lysinibacillus sp. BF-4	WP_036144850	-----D--------------------		-------------------------
	Lysinibacillus jejuensis	WP_108306347	----AD--E------------VSV-		L-K--------E------AQ-S-D-
Other Bacteria (0/>200)	Alkalibacillus haloalkaliphilu	WP_017185668	M---QD-LK--S-------R-GIE--	D	LTGKK------T---I--AQ-SED-
	Alkalibacterium olivapovliticu	WP_106190935	---FKD-L-G----------GLN--	D	LKD-P---G--T---I--P--ST--
	Alkalibacterium pelagium	WP_091481362	-----D-L-G----------GLD--	D	LTGLP---G--T---I--P--ST--
	Alkalibacterium sp. AK22	WP_034301991	----QD-L-G---------I-ELE--	D	MTGLP---G--T---I--S--SS--
	Alkalibacterium subtropicum	WP_091528426	-----D-LTG-V---------GLQ--	D	LSK-P---G--K--SI--S--SE--
	Alkalibacterium thalassium	WP_091265587	-----D-L-G----------GLD--	D	LTGLP---G--T---I--P--ST--
	Amphibacillus jilinensis	WP_017471697	----TH-LK----------R-GIT--	P	-PQVP---G--L---I---Q-SQD-
	Amphibacillus sediminis	WP_067839962	-----KPLR----------R-DIK--	D	-TGLP---G--K------QR-SV--
	Amphibacillus xylanus	WP_015010383	----ENVLLG---------R-DIDI-	N	-NQIP---G--E---M--P--SV--
	Aquisalibacillus elongatus	WP_124219772	----NDSLK--M-F-----R-GIQ--	D	--GVN---G--E---I--AQ--QD-
	Atopococcus tabaci	WP_028273187	----QD-L-G---------R-GID--	D	LTG-P---G--T---I--AK-SE--
	Bacillus acidicola	WP_066268817	----KDSLKG---------R-GID--	D	LTG-E---G--T---M--AA-SE--
	Bacillus aciditolerans	WP_121446258	----KN-LK----------R-GIE--	D	LTG-P---T--K---I--AQ-T-D-
	Bacillus albus	WP_128805380	----EN-LKG-V-------R-GTQ--	N	LAGKS------T---I-SSA-SE--
	Bacillus alkalitelluris	WP_078549082	---HEN-LKG---------R-GID--	E	LKG-S---G--K------LP--S---
	Bacillus altitudinis	WP_050826815	----GD-LKG---------R-GVS--	S	LAGKQ------E---M-RP--SQ--
	Bacillus amyloliquefaciens	WP_045511270	----KDVLKG---------I-GVK--	D	MAGQP---G--KR----T---SE--
	Bacillus andreraoultii	WP_033827838	---EEN-LE----------R-GIE--	N	LEGK--L-T--T---I--QQ-S---
	Bacillus anthracis	KOS26552	----DN-LKG-V-------R-GTQ--	N	LAGKS------T---I-SSA-SE--
	Bacillus australimaris	WP_060698944	----GD-LKG---------R-GVS--	S	LEGKQ------E---M-RP--SQ--
	Bacillus badius	WP_041098599	---FQD-LKG-V-F-----I-GRQ--	D	LSG-E---G--E------PQ-TK--
	Bacillus beveridgei	WP_069365863	----KD-LQG---------R-GIE--	D	LTG-P------E---I--AQ-SED-
	Bacillus boroniphilus JCM 2173	GAE47772	----QN-LKG---------R-GID--	D	LTG-S------T---I-SPQ-SE--
	Bacillus campisalis	WP_046522433	----QD-LQ----------R-GIQ--	E	LGG-SI--G--T---I--P--SR--
	Bacillus cecembensis	WP_057990127	--N-ENTLKG---------D-DAK--	N	-TG-K------V-----T-----D-
	Bacillus cellulasensis	WP_057078783	----GD-LKG---------R-GVS--	S	LAGKQ------E---M-RP--SQ--
	Bacillus cereus	WP_074569667	----EN-LKG-V-------R-GTQ--	N	LAGKS------T---I-SSS-SE--
	Bacillus circulans	WP_123259009	----QD-LK----------R-GIT--	D	LTGKG---T--T---I-S---SN--
	Bacillus dakarensis	WP_077212085	----QD-LKG---------R-GID--	D	LSG-S--MG--T---I--AA-ST--
	Bacillus dielmoensis	WP_042455510	----QN-LSG---------R-GID--	D	LSGKA---S--T---I--Q--S---
	Bacillus endophyticus	WP_124050630	----ED-LKG---------R-GIE--	N	LAH-Q---G--E---M-TA--SK--
	Bacillus fastidiosus	WP_066233288	----ED-LRG---------R-GIE--	D	LSGKSL-----T---M--P---D--
	Bacillus firmus	WP_048009940	---HEN-LK----------R-GAE--	D	LSG-S------T---I--A--STD-
	Bacillus fordii	WP_018708100	----QD-LKG---------R-GIE--	D	LSDKS------T---I--PS-SK--
	Bacillus fortis	WP_120070437	---SEDSLKG---------R--IE--	N	LTGRHI-----K---M--PH-SL--
	Bacillus galactosidilyticus	WP_064468523	----QQ-LKG---------R-GIE--	D	LNG-P---G--T---I--SK-SQ--
	Bacillus gottheilii	WP_066442597	M---ADSLKG---------R-GIQ--	D	LTG-S------T---I--SS-SED-
	Bacillus halotolerans	WP_105955014	----ADVLK----------I-GLE--	D	MTGLP---G--KY----T---SE--
	Bacillus horneckiae	WP_066400014	----QS-LKG---------R-GVE--	D	LTGKS------T---I--PT-ST--
	Bacillus humi	WP_057998955	----KD-IKG---------R-GIE--	D	LTG-P-L-T--K---I--AQ-TVD-
	Bacillus infantis	WP_129612678	----KD-LKG---------R-GVE--	D	LTG-S------K---I--SS-SE--
	Bacillus jeotgali	WP_079504754	----QK-LKG---------R-GID--	D	LKG-P------T---I--PQ-SE--
	Bacillus kochii	WP_095371862	M---KD-VK----------R-GIE--	D	LTGQK---G--T---I-SP--SE--
	Bacillus korlensis	WP_066051720	----KN-LK------------GVE--	N	LDG-S---G--T---I-SP--ST--
	Bacillus kwashiorkori	WP_062354448	----KN-LK----------R-GIA--	D	LSGVP---G--A---I--P--STD-
	Bacillus massilioanorexius	WP_019243590	-----HSLKG---------R-GID--	D	LTG-FT-----K---I--AQ-SE--
	Bacillus massiliogabonensis	WP_102273875	---FQN-LKG---------R-GIE--	D	LSGKS------T---I-SP--SV--
	Bacillus massiliogorillae	WP_042351949	M---GD-LKG---------R-GIE--	N	LDGAS------K---I--AA-SE--
	Bacillus selenatarsenatis	WP_041965338	----KN-LKG---------R-GIE--	E	LSD-E--L---K---I--AQ-SE--
	Bacillus siamensis	WP_029575225	----KDVLKG-V-------I-GVK--	D	MAGLP---G--KR----T---SE--
	Bacillus simplex	WP_095392095	----EG-LQG---------R-GIA--	S	LSDIP------K---I--A--TD--
	Bacillus sinesaloumensis	WP_077619297	----KDSLKG---------R-DVE--	D	LTG-P---T--K---I--AQ-T-D-
	Bacillus timonensis	WP_010676490	----KD-LKG---------R-GIE--	E	LTD-P---T--K---I--AQ-T-D-
	Bacillus toyonensis	WP_000975466	----ED-LK--V-------R-GIQ--	N	LAE-A------T---I-APS-SE--
	Bacillus velezensis	WP_043021824	----KDVLKG-V-------I-GVK--	D	MEGLP---G--KR----T---SE--
	Bacillus wiedmannii	EJQ54666	----EN-LKG-V-------R-GTQ--	N	LAGKS------T---I-SSA-SE--
	Bacillus wudalianchiensis	WP_065409727	----AHSLK----------R-GID--	D	LSGKK------I---I--QQ-SL--
	Bacillus xiamenensis	WP_071168277	----GD-LKG---------R-GVS--	S	LAGKQ------E---M-RP--SQ--
	Bacillus zhangzhouensis	WP_034323880	----GD-LKG---------R-GVS--	S	LAGKQ------E---M-RP--SQ--
	Bhargavaea beijingensis	WP_092098920	M---AD-LRG---------I-DQE--	A	LTG-S---G--T---I--VD--KD-
	Bhargavaea cecembensis	WP_063177895	M---EN-LKG---------I-DKE--	P	LKG-S---G--T---I--MD--KD-
	Bhargavaea ginsengi	WP_092051762	M---AD-LKG---------V-DKG--	S	LDG-S---G--T---I--MD--KD-
	Carnobacterium alterfunditum	WP_034546548	----AD-LSG---------I-DLS--	D	LTN-PI--S--T---I--AK-SE--
	Carnobacterium inhibens	WP_023179212	----GDSLTG---------R-GIT--	D	LSDKAI--S--T---I--SK-SE--
	Caryophanon latum	WP_066464155	--T-AD-L-G-V-------R-DADM-	N	L-NVP---G--T-----T---SE--
	Cohnella lupini	WP_115993744	----EGS-KG---------I--VA--	D	LTGVPI--G--E---I-VPQ-TE--
	Cohnella panacarvi	WP_027087045	----AD--KG----------GLK--	D	LSGIP------E---I-SAA-TE--
	Cohnella sp. AR92	WP_126958004	----KD--DK---------M-GLK--	D	LAG-AI--G--T---I-APQ-TE--
	Domibacillus antri	WP_075396740	----KN-LKG---------R-GIN--	D	LTG-P--M---E---M--PQ-TE--
	Domibacillus epiphyticus	WP_076763851	----EK-LK----------R-GIE--	S	LTGVPI-M---E---I--SQ-T-D-
	Domibacillus mangrovi	WP_073711787	----EK-VKG---------R-GIE--	N	LTGIP--M---K------P--TE--

Other Bacteria (0/>200)

Organism	Accession			
Edaphobacillus lindanitolerans	WP_076758086	--Q-EQSLKG-----------GVE--	G	-NGVP---G--T---I--RS--E--
Fictibacillus enclensis	SCC13559	----E-SLRG-V---------GIQ--	D	LNGVN---G--K---I--PQ-TE--
Fictibacillus solisalsi	WP_090235436	----KTSLRG-V---------GIQ--	D	LKGV----G--K---I--PQ-TE--
Gracilibacillus dipsosauri	WP_054859609	----QDGL-E---------R-GIG--	D	LTN-K---T--R-----TEQ-SLD-
Gracilibacillus halophilus	WP_003467133	----QD-L---V-------R-GIE--	D	LATVK-L-T--R-----TEQ-SND-
Gracilibacillus orientalis	WP_091484047	----QD-LN--V-------R-GIE--	D	LT-VK-L-T--L-----TEQ-S-D-
Gracilibacillus ureilyticus	WP_089742058	----QD-LN----------R-GIE--	D	LAGVK--LT--H---I--EQ-S-D-
Halalkalibacillus sp. B3227	WP_101330535	----EKVLK----------R-GID--	E	-HE-P------E---I-SPQ-SKD-
Halobacillus dabanensis	WP_075036923	----EKSL-G-V-------R-GIEVA	N	-TGLP---G--E---I--AQ-TKD-
Halobacillus massiliensis	WP_082234055	----Q-SLKS---Y--------VP--	E	HSSLK---G--E---I--P--TS--
Halobacillus salinus	WP_079480026	----ENSLN--L-F-----R-GIQ--	D	-DGVP---G--E---I--AQ-TVD-
Halobacillus sp. SKP4-6	WP_128539630	----KDSLKG-L-F-----R-GIE--	D	-SGVP---G--E---I--AQ-TVD-
Jeotgalibacillus alimentarius	WP_041122592	-----DSLK--S---------DKE--	D	LSKIS---G--E---I--P--TKD-
Jeotgalibacillus campisalis	WP_041057811	I---EN-LKS---------I-DKK--	E	LNGVH-L-T------I--P--TRD-
Jeotgalibacillus malaysiensis	WP_039809178	-----DSIK--M---------D-K--	S	LAG-K---G--E---I--AV-TTD-
Jeotgalibacillus proteolyticus	WP_104055563	----EN-LKG-V-------I-GLD--	D	SS-IN-L-T------I--P--TTD-
Jeotgalibacillus salarius	WP_134379877	--N--DSLR--M---------D-K--	D	LTG-----G--E---I-AP--TKD-
Jeotgalibacillus sp. R-1-5s-1	WP_134371508	-----DSLKG-----------D-K--	Q	LTDVP---G--K---I--P--TTD-
Jeotgalibacillus sp. S-D1	WP_133375716	----EN-LKG-----------DKK--	D	LTNVP---T--I---I--P--TN--
Lacticigenium naphtae	WP_027109243	----AD--KG---------IHGLT--	A	LSHSS-L-T--E---I--P--SE--
Lentibacillus amyloliquefacien	WP_068445963	----KD-LKS---------R-GIE--	D	LSGKK---G--T---I-SA--SED-
Lentibacillus halodurans	WP_090236846	----RS-LKG---------R-DIG--	D	LSGKQI--G--T---I--SQ-SED-
Lentibacillus sediminis	WP_100012579	----QD-LK----------R-GID--	D	LS-KD---G--T---I-SA--SE--
Lentibacillus sp. SSKP1-9	WP_129673498	----QD-LKG---------RKGVE--	N	LSGKN---G--T---I--SQ-SE--
Lysinibacillus acetophenoni	WP_097150472	----AD-LKG---------R-GIE--	N	LDGKN------V---I--QQ-SV--
Lysinibacillus boronitolerans	WP_036079458	----DD-L-G---------R-GIE--	Q	LSP-P---G--T---M-TAQ-SED-
Lysinibacillus chungkukjangi	WP_107937905	----KD-LKG---------R-GID--	D	LKE-K-L-T--T---I--QQ-SL--
Lysinibacillus composti	WP_124766781	---F-G-LKG---------R-GID--	N	LEGAK------T---I--QQ-S---
Lysinibacillus endophyticus	WP_121213420	----ET-IKG---------R-GIQ--	N	LAG-H------T---I--QQ-SV--
Lysinibacillus fusiformis	WP_004231919	----DD-L-G---------R-GIE--	Q	LSP-P---G--T---M-TAQ-SED-
Lysinibacillus halotolerans	WP_122972035	---IQDTLKG---------R-GIQ--	N	LEGINI-----V---I--QQ-S---
Lysinibacillus macroides	WP_053996897	----ED-L-G---------R-GIE--	K	LS--P---G--T---M-IPQ-SED-
Lysinibacillus manganicus	WP_036184261	---FAD-LKG---------R-GID--	N	LDGKN------V---I--QH-SVD-
Lysinibacillus massiliensis	WP_036177333	----EN-LKG---------R-GIE--	Q	LTD-KI-----T---I--QQ-SVD-
Lysinibacillus sinduriensis	WP_036198017	----KD-LMG---------R-GIE--	D	LT-KN-Y----T---I--QQ-SV--
Lysinibacillus sp. 2017	WP_108712814	--E-ENT-KG---------N-DAK--	N	--G-H------I-----S----VD-
Lysinibacillus sp. LK3	WP_048394178	----DD-L-G---------R-GIE--	Q	LSP-P---G--T---M-TAQ-SED-
Lysinibacillus sp. Marseille-P	WP_106782373	----ADSLKG---------R-GME--	N	LDG-D------V---I--QQ-SV--
Lysinibacillus sp. OL1	WP_131520896	----DD-L-G---------R-GIE--	Q	LSP-P---G--T---M-TAQ-SED-
Lysinibacillus sp. SYSU K30002	WP_126660017	---FKD-LKG---------R-GIE--	N	LEG-P------V---I--QQ-S-D-
Lysinibacillus sp. YLB-03	WP_118877826	----KD-LKG---------R-GIE--	D	LKEAK------T---I--QQ-SV--
Lysinibacillus sp. ZYM-1	WP_054609472	----E--L-G---------R-GID--	K	MS--P---G--T---M-AAQ-SE--
Lysinibacillus sphaericus	VDG98124	----DD-VKG---------I--VPM-	D	LAGIH-W-G--A---I-TQ--ST--
Lysinibacillus sphaericus	WP_010860391	----KD-L-G---------R-DIE--	V	LTSMP------T---M-TA--SQD-
Lysinibacillus sphaericus	WP_031416552	----EDVL-G---------R-GIE--	K	LS--P---G--T---M-TAQ-SE--
Lysinibacillus sphaericus	WP_036164814	----EDVL-G---------R-GIE--	K	LS--P---G--T---M-TAQ-SE--
Lysinibacillus sphaericus	WP_036217643	----EDVL-G---------R-GIE--	K	LS--P---G--T---M-TAQ-SE--
Lysinibacillus sphaericus	WP_075526580	----EGSLKG-S-------R-GLQ--	N	LEG-K------K---I--MA-SE--
Lysinibacillus sphaericus	WP_112116776	----DD-L-G---------R-GIE--	Q	LSP-P---G--T---M-TAQ-SED-
Lysinibacillus sphaericus	WP_125102479	----DD-VKG---------I--VPM-	D	LAGIH-W-G--A---I-TQ--ST--
Lysinibacillus sphaericus C3-4	ACA41834	----EDVL-G---------R-GIE--	K	LS--P---G--T---M-TAQ-SE--
Lysinibacillus telephonicus	WP_126292490	---HKD-LKG---------R-GIE--	N	LKDIN------T---I--QQ-S---
Lysinibacillus xyleni	WP_097073546	---FKDSLKG-----------GIT--	N	LTG-H---S--T---I---Q-SD--
Mycobacteroides abscessus subs	SHS08795	----EN-LK--V---------GIE--	N	LEG-S---G--T---I-SP--ST--
Oceanobacillus arenosus	WP_115772768	----QD-LK----------R-GIE--	N	LSSKG------T---I--ST-SE--
Oceanobacillus bengalensis	WP_121131825	----QD-LKG---------R-GIQ--	N	LSDKN------T---I--AQ-SE--
Oceanobacillus chungangensis	WP_115750736	----QD-LK----------R-GIE--	D	LSSNG------T---I--AT-SED-
Oceanobacillus halophilus	WP_121206042	M---QD-LNT---------RKGID--	D	LSGKK------T---I--AS-SA--
Oceanobacillus iheyensis	WP_106895551	M---KD-LN----------R-GIS--	D	LSGKK-L-T--T---I--SQ-S-D-
Oceanobacillus jeddahense	WP_040983295	----KNSLQK---------RKSID--	D	LTG-D------N---M-SP---E--
Oceanobacillus sp. Castelsardo	WP_068677390	----KDSLN----------R-GIE--	D	LSDKK------T---I--AQ-SED-
Oceanobacillus sp. YLB-02	WP_121524673	----KD-LKG---------R-GID--	D	LSGKK------T---I--A--SE--
Oceanobacillus timonensis	WP_080875530	----KDSLQK---------RKGID--	D	LTG-E----------M--P---E--
Ornithinibacillus halophilus	WP_072888053	----QD-LK----------R-GVE--	D	LSTKH------K---I--SQ-SE--
Paenibacillus agaridevorans	WP_108995899	----KDSLKG---L-------GIK--	D	LSGVP------E------PT-TK--
Paenibacillus antarcticus	WP_068650833	----ED--KG------------KE--	D	LSCIP---G--R--MM--AQ-SED-
Paenibacillus bouchesdurhonens	WP_110933418	----EH--KG-----------GMQ--	N	LAEVP---G--S---I--PS-TE--
Paenibacillus macquariensis	WP_068592227	----ED--KG------------KE--	D	LSGIP---G--R--MM--AQ-SED-
Paenibacillus sp. 18JY67-1	WP_126018552	----KD--K----------I-SIA--	D	LSNVPI--G--K---I-SAS-TE--
Paenibacillus taihuensis	WP_116191684	----EQ--KG---------I--IA--	D	-SGIP---G--E---I-SSD-TE--
Paenibacillus terrigena	WP_018758615	----AN-VRG-V-------R-GVA--	N	LEG-E---G--T---I-S-S-SE--
Paenibacillus thiaminolyticus	WP_087440091	---IQ--W-G---------R-GV---	E	LSGLP---G--K---I-SPQ-TE--
Paenisporosarcina antarctica	WP_134211083	---FDN-IRG---------R-GIA--	D	LSGKH---T--K---I--QQ-SVD-
Paenisporosarcina indica	WP_075618673	M---Q--L-G---------R-GID--	D	LSSK-------T---I--AQ-SED-

Group	Species	Accession	Sequence
Other Bacteria (0/>200)	Paenisporosarcina sp. OV554	WP_108586522	---FKDSIKG---------R-GIT-- D LSGKH------K---I-SQQ-SVD-
	Paenisporosarcina sp. TG-14	WP_017380587	---FDN-IRG---------R-GI--- D LSGKH---T--K---I--QQ-SVD-
	Paenisporosarcina sp. TG20	WP_019416141	---FKH-IKG---------R-GIS-- D LTG-H---T--K---I--QQ-SVD-
	Paraliobacillus ryukyuensis	WP_113869757	----KK-LK----------R--IE-- D LTG-AI--G--E----ISIQ-SN--
	Paraliobacillus sediminis	WP_117169895	---HEQ-LKG---------R--IT-- D LKSVD---G--H-----KEA-SK--
	Paraliobacillus sp. PM-2	WP_090854852	---EQD-LKG---------R-GII-- D LTGKAI-----T---I-SS--ST--
	Pelagirhabdus alkalitolerans	WP_090792611	----EKPLKG---------R-GLT-- D -NQLP---G--T------VD-TK--
	Planococcus citreus	WP_121299512	--S-EDSLK----------D--KE-- N LEG--------T---I-----SED-
	Planococcus donghaensis	WP_008430311	--T--N-LKG---------N-DTN-- D LTNIP------T---I--A--STD-
	Planococcus faecalis	WP_071153118	--T-TG-LKG---------N-HTD-- D LAN-P------T---I--A--S-D-
	Planococcus kocurii	WP_058384326	--T-TG-LKG---------N-HTD-- D LAN-P------T---I--A--S-D-
	Planococcus maritimus	WP_068465784	--S-EDSLK----------D-DK--- N LEE--------T---I---V-SED-
	Planococcus massiliensis	WP_052650527	--T--D-LKG---------N-ETE-- D LAGVP---S--T---I--S--STD-
	Planococcus plakortidis	WP_068872600	--T-EDSLK----------D-HKE-- N LDG--------T---I-----SED-
	Planococcus rifietoensis	WP_058383789	--S-EDSLK----------D--KQ-- N SEG--------T---I-----SED-
	Planococcus sp. PAMC 21323	WP_038705485	-Y-FKD-ITG------------IE-- D LSERK------T---IS-VK--E--
	Planococcus sp. Y42	AQQ53169	-Y---D-LNG-----------GME-- D LTGKH------T---IS-IV--E--
	Planococcus sp. Y42	WP_077589054	-Y---D-LNG-----------GME-- D LTGKH------T---IS-IV--E--
	Planococcus sp. Y42	WP_077590311	----GD-LRG---------V-DME-- D LTG-P---G--T-----TA--SED-
	Planococcus versutus	WP_049693856	-Y-FKN-ITG------------IE-- D LSGRK------T---IS-VK--E--
	Planomicrobium glaciei	WP_036805558	--T--D-LKG---------N-ETQ-- D LSGVQ------T---I--A--STD-
	Planomicrobium soli	WP_106533954	--T-AD-LKG---------N-ETE-- D LSG-P------T---I--A--STD-
	Psychrobacillus sp. OK028	WP_093061526	---FKD-LKG---------R-GMI-- D LTG-E------T---I--QR-SVD-
	Psychrobacillus sp. OK032	WP_093269495	---FNDSLKG---------R-GIP-- D LTGIK------T---I--QK-SV--
	Quasibacillus thermotolerans	KKB38305	M---QD-LKG---------R-GIR-- D LSGKA------T---I-SP--SK--
	Salirhabdus sp. Marseille-P466	WP_102028986	----ADSLKG---------R-GIE-- S LNG-P------T---I--AQ-SND-
	Saliterribacillus persicus	WP_114352443	----QD-LNG-V-------R--IE-- D LSSVK-L-T--H-----TE--SY--
	Solibacillus sp. R5-41	WP_099423396	----ENTLKG---------D-QAK-- N -TG-K------V-----T--D-TD-
	Sporosarcina globispora	WP_053435074	----ENSIK----------R-GVE-- D LSGKSI-----T---I--A--STD-
	Sporosarcina koreensis	WP_040287189	---FADVLKG-V-------I--RD-- D LTGIP-W-G--A---I--QA-ST--
	Sporosarcina newyorkensis	WP_009766604	-----D-LKG---------R-GIA-- N LAG-S--L---T---I--PS-SED-
	Sporosarcina pasteurii	WP_115363725	----EGSLKG-S-------R-GLQ-- N LEG-K------K---I--MV-SE--
	Sporosarcina psychrophila	WP_067204585	----A--LKG---------R-GID-- D LAGIP---T--K---I--AK-SED-
	Sporosarcina sp. BIO01-red	WP_116019840	----AD-VKG---------I--VPM- D LTGIH-W-G--A---I-TQ--SV--
	Sporosarcina sp. D27	WP_025782805	----DD-VKG---------I--VTM- D LAGIH-W-G--A---I-TQ--ST--
	Sporosarcina sp. P13	WP_099687423	----QH-LKG---------R-GIE-- D LSGKP------S---M--P---E--
	Sporosarcina sp. ZBG7A	WP_039043667	----DD-VKG---------I--VPM- D LTGIH-W-G--A---I-TQ--ST--
	Terribacillus aidingensis	WP_097038822	-Y---NPLKG---------R--IT-A Q --G-P-L-T--T---I---S--TD-
	Terribacillus goriensis	WP_038563581	-Y--TDSLKG---------R--II-A Q --G-P---T------I--RS-----
	Terribacillus halophilus	WP_077306721	-Y---NPLKG---------R--II-A Q --G-P-L-T--T---I---S--TD-
	Terribacillus saccharophilus	WP_095227723	-Y--TASLKG---------R--IT-A Q --G-P---T------I--RS-----
	Terribacillus sp. 7520-G	WP_095214383	-Y-F-DSLKG---------R-HIE-A K --G-P---T------I--RS-----
	Vibrio vulnificus	WP_133346866	----EG-LQG---------R-GIA-- S LSDIP------K---I--A--TD--
	Virgibacillus dokdonensis	WP_116277820	----QH-LCG---------R-GIA-- N LAHKH------M---I--AQ-SKD-
	Virgibacillus indicus	WP_094887000	----QD-LKG---------R-GVD-- D LTGKD------T---I--AQ-SED-
	Virgibacillus massiliensis	WP_038245837	----PH-LYG---------R-GLS-- D LSGIN------T---I--A--SE--
	Virgibacillus pantothenticus	WP_077302990	----QDSLRG---------R-GIE-- E LTKKD------T---I--A--SED-
	Virgibacillus profundi	WP_095656949	----KD-LKG---------R-GID-- D LSGKG------K---I--AQ-SED-
	Virgibacillus soli	WP_057984943	----QQ-LKG---------R-GIE-- D LNG-P---G--T---I--SK-SQ--
	Virgibacillus sp. 7505	WP_095221291	-Y--TGSLKG---------R--IT-A E --G-P---T------I--RS-----
	Virgibacillus sp. Bac330	WP_121638912	----QH-LCG---------R-GIA-- N LAHKH------M---I--AQ-SKD-
	Virgibacillus sp. Bac332	WP_121604398	----KNPLKG---------R-GIS-- N LSGIN------T---I--A--SE--

Supplemental Figure 24

A partial sequence alignment of the putative hydrolase MhqD protein containing a three amino acid insertion (boxed) that is exclusively shared by all members belonging to the Jejuensis clade and absent in all other bacteria.

Group	Organism	Accession	82		136
**Jejuensis Clade (3/3)**	Lysinibacillus jejuensis	WP_108306939	SSLVARKLLDNQVPPKKAFAFNYACTEVI		SAMSQASFLQVAEELIEFYIDFIADRK
	Lysinibacillus saudimassiliensis	CEA03907	--II----------A-------F------		-T--E-------D--------------
	Lysinibacillus sp. BF-4	WP_036147033	--II----------A-------I------		-N--D-------D--------------
**Other Bacteria (0/>100)**	Bacillus cecembensis	WP_057985960	--I-----------S-------L--ADM-	E	NS-KD-E---F-DD--D--VY------
	Bacillus ndiopicus	WP_042476990	--I-------------------L---DM-	E	NK-KD-E---F-DD-----VY------
	Bacillus sp. B14905	EAZ85853	--I-----------S-------I--NDM-	E	NQ-KD-E---F-DD--D-FVY------
	Caryophanon latum	WP_066461123	--I-----------A------SV--DDM-	D	NQ-RD-E---F-DD--D-FVN------
	Caryophanon tenue	WP_066546313	--I-----------A-------V--VDM-	D	NH-RD-E---F-DD-VD-FVN------
	Chryseomicrobium excrementi	WP_100354207	M----I-S---AV-----SRS-MD--	D	NR--DVL---CG-------VHVLSE--
	Kurthia huakuii	WP_029500451	--V-T---IE--A-L-------S--VQMV	D	ER-ND-E---F-DD-V----YV-SE--
	Kurthia massiliensis	WP_010290643	--V-T---I---A-L-------S--VQMV	D	ER-ND-E---F-DD------YV-SE--
	Kurthia senegalensis	WP_010307315	--V-T-R-IE--A-L-------T--V-M-	D	ER-ND-E---F-DD------YV-SE--
	Kurthia sibirica	WP_109306452	--V-T---VE--A-F-------A--V-M-	D	QR-ND-E---F-DD------YV-SE--
	Kurthia sp. 3B1D	WP_126991408	--V-T---I---A-L-------S--IQMV	D	ER-ND-E---F-DD-V----YV-SE--
	Kurthia zopfii	VEI05745	--V-T---VE--A-F-------A--V-M-	D	QR-ND-E---F-DD------YV-SE--
	Lysinibacillus acetophenoni	WP_097150326	--I-----------A----------ADM-	E	NK-KD-E---F-D------V-------
	Lysinibacillus chungkukjangi	WP_107934778	--I-----------S-------L--ADM-	E	NN-KD-E---F-DD-VD--VY------
	Lysinibacillus composti	WP_124766904	--I-----------S-------L--ADM-	E	NK-KD-E---F-D---D--VY------
	Lysinibacillus contaminans	WP_053584967	--I-----------S-------V--NDM-	E	NQ-KD-E---F-DD--D-F-Y------
	Lysinibacillus fluoroglycofeni	WP_107943467	--I-------------------L--ADM-	E	NK-KD-E---F-DD-----VY------
	Lysinibacillus fusiformis	WP_069482838	--I-----------S-------I--NDM-	E	NQ-KD-E---F-DD--D-FVY------
	Lysinibacillus halotolerans	WP_122972911	--I-----------SR------L--ADM-	E	NK-KD-E---F-DD--D--VH------
	Lysinibacillus manganicus	WP_036189540	--I-----------A----------ADM-	E	NK-KD-E---F-D------V-------
	Lysinibacillus massiliensis	WP_036172824	--I-----------S-------L--SDM-	E	NK-KD-E---F-D------V-------
	Lysinibacillus meyeri	WP_107839648	--I-------------------L---DM-	E	NK-KD-E---F-DD-----VY------
	Lysinibacillus odysseyi	WP_036152666	--I-----------S-------L--ADM-	E	NR-KD-E---F-DD--D--VY------
	Lysinibacillus parviboronicapi	WP_107925440	--I-----------S-------I--NDM-	E	NQ-KD-E---F-DD--D-FVY------
	Lysinibacillus sinduriensis	WP_036200467	--V-----------S-------L--ADM-	E	NN-KD-E---F-DD--D--VY------
	Lysinibacillus sp. 2017	WP_108714137	--I-----------S-------L--ADMV	E	NK-KD-E---F-DD--D--VY------
	Lysinibacillus sp. AC-3	SKB96634	--I-----------S-------I--NDM-	E	NQ-KD-E---F-DD--D-FVY------
	Lysinibacillus sp. BK089	WP_132361596	--I-----------S-------I--NDM-	E	NQ-KD-E---F-DD--D-FVY------
	Lysinibacillus sp. FJAT-14222	WP_053593998	--I-----------S-------I--NDM-	E	NQ-KD-E---F-DD--D-FVY------
	Lysinibacillus sp. FJAT-14745	WP_053481947	--I-----------S-------I--NDM-	E	NQ-KD-E---F-DD--D-FVY------
	Lysinibacillus sp. Marseille-P	WP_106781614	--I-----------S-------L--ADM-	E	NK-KD-E---F-D---D--V-------
	Lysinibacillus sp. SG9	SCZ01290	--I-----------S-------I--NDM-	E	NQ-KD-E---F-DD--D-FVY------
	Lysinibacillus sp. SYSU K30002	WP_126658787	--I-----------S-------L--ADM-	E	NK-KD-E-I-F-DD-VD--VY------
	Lysinibacillus sp. YLB-03	WP_118876778	--II----------S-------L--ADMV	E	NK-KD-E---F-DD--D--VY------
	Lysinibacillus sp. YR326	TDV00865	--I-----------S-------I--NDM-	E	NQ-KD-E---F-DD--D-FVY------
	Lysinibacillus sp. ZYM-1	WP_054609423	--I-----------S-------I--NDM-	E	NQ-KD-E---F-DD--D-FVY------
	Lysinibacillus sphaericus	POZ54895	--I-----------S-------I--NDM-	E	NQ-KD-E---F-DD--D-FVY------
	Lysinibacillus sphaericus	SPT97223	--I-----------S-------I--NDM-	E	NQ-KD-E---F-DD--D-FVY------
	Lysinibacillus sphaericus	WP_010860333	--I-----------S-------I--NDM-	E	NQ-KD-E---F-DD--D-FVY------
	Lysinibacillus sphaericus	WP_031416499	--I-----------S-------I--NDM-	E	NQ-KD-E---F-DD--D-FVY------
	Lysinibacillus sphaericus	WP_061504537	--I-----------S-------I--NDM-	E	NQ-KD-E---F-DD--D-FVY------
	Lysinibacillus sphaericus	WP_062794077	--I-----------S-------I--NDM-	E	NQ-KD-E---F-DD--D-FVY------
	Lysinibacillus sphaericus	WP_069508948	--I-----------S-------I--NDM-	E	NQ-KD-E---F-DD--D-FVY------
	Lysinibacillus sphaericus C3-4	ACA41780	--I-----------S-------I--NDM-	E	NQ-KD-E---F-DD--D-FVY------
	Lysinibacillus telephonicus	WP_126293323	--I-----------S-------L--ADM-	E	NK-KD-E---F-DD--D--VY------
	Lysinibacillus varians	AHN23516	--I-----------S-------I--NDM-	E	NQ-KD-E---F-DD--D-FVY------
	Lysinibacillus xylanilyticus	WP_068986739	--I-----------S-------V--NDM-	E	NQ-KD-E---F-DD--D-FVY------
	Paenisporosarcina antarctica	WP_134208553	--IM----YE----F--T----T--IDMV	D	NHLND-----F-DD--D-FVSV-SE--
	Paenisporosarcina indica	WP_075619281	--IM----YE----S---L------IDM-	D	NH-ND-----F-DD--D-FVMV-SE--
	Paenisporosarcina sp. HGH0030	WP_016429722	--IM----YE----S-------A--I-M-	D	FH--D-A---F-DD--D-FVSV-SE--
	Paenisporosarcina sp. K2R23-3	WP_119884101	--VMS---YE--------Y---T--VDM-	E	NY-TD-----F-DD--D-F-QT--E--
	Paenisporosarcina sp. OV554	WP_108585515	--IM--Q-YE----A-------S--IGMV	D	FH-ND-Q---F-DD--D-FVSV-SE--
	Paenisporosarcina sp. TG-14	WP_026045745	--IM----YE----F--T----T--IDMV	D	NHLND-----F-DD--D-FVSV-SE--
	Planococcus antarcticus	WP_006830219	--VM----YEMR----------S--V-LV	D	KH-NDSE-MF--D-----FVSI-SE--
	Planococcus donghaensis	WP_008432372	--VM----YEMR----------A--V-LV	D	KH-NDSE-MF--D-----FVSI-SE--
	Planococcus halocryophilus	WP_008498798	--VM----YEMR----------S--V-LV	D	KH-NDSE-MF--D-----FVSI-SE--
	Planococcus maritimus	WP_068489013	--VM----YEMR----------A--V-LV	D	KH-NDSE-MY--D-----FTSI-SE--
	Planococcus massiliensis	WP_052653177	--VM----YEMR----------T--I-LV	E	KH-NDSE--Y--D-----FVSV-SE--
	Planococcus plakortidis	WP_068872033	--VM----YEMR----------A--V-LV	D	KH-NDSE-MY--D-----FTSI-SE--
	Planococcus salinarum	OHX48564	--VM----FEMR----------N--I-LV	D	NR-NDSE--Y--D-----F-SV-SE--
	Planococcus salinus	WP_123166285	--VM----YEMR----------A--V-MV	D	NR-NDSE--Y--D-----FVSI-SE--
	Planococcus sp. CAU13	WP_033541020	--VM----FEMR----------N--I-LV	D	NR-NDSE--Y--D-----F-SV-SE--
	Planococcus sp. PAMC 21323	WP_038702540	--VM----YEMR----------S--V-LV	D	KH-NDSE-MF--D-----FVSI-SE--
	Planococcus sp. Y42	WP_077587984	--IM----YET-----------A--V-IV	E	NR-NDSE--L--D-----FVSILSE--
	Planococcus versutus	WP_049695056	--VM----YEMR----------S--V-LV	D	KH--DSE-MF--D-----FVSI-SE--
	Planomicrobium flavidum	WP_088006141	--IM----YET-----------A--I-IV	E	NR-NDSE--L--D-----FVSILSE-Q
	Planomicrobium glaciei	WP_036807471	--VM----FEMR----------V--V-LV	E	KH-NDSE--Y--D-----FTSI-SE--
	Planomicrobium okeanokoites	WP_084243052	--VM----FEMR----------N--I-LV	D	NR-NDSE--Y--D-----F-SV-SE--
	Planomicrobium soli	WP_106534537	---M----YEMR----------S--I-LV	D	NR-NDSE--Y--D-----FVSI-SE--
	Planomicrobium sp. MB-3u-38	WP_101801574	--VM----FEMR----------N--I-LV	D	NR-NDSE--Y--D-----F-SV-SE--
	Psychrobacillus insolitus	WP_111439900	AFM-S--YE----SD-VL---NS-V---	E	TK-ND-Q-M-CGY--V--FVKIVTE--

Other Bacteria
(0/>100)

```
Psychrobacillus psychrotoleran     WP_093537549  AAIMG---NES---TD-VS---I--V-T- E TK--D-Q--LC-D--V--FVMV-TE--
Psychrobacillus sp. OK028          WP_093061579  -AVMG---NES---ID-IR---I--L-T- E TK--D-Q--LC-D--V--FVMV-TE--
Psychrobacillus sp. OK032          WP_093271958  A-IM----YE----TD-VM---I-SVDI- E TQ-ND-Q---C-D--V--FVLV-TE--
Rhizophagus irregularis            PKC50643      --I-----------S-------L--SDM- E NK-KD-E---F-D------V-------
Rummeliibacillus pycnus            WP_102693729  --V-T---IE--A-S--V----A--V-M- D ERLND-EY--F-DD--D---YV-SE--
Rummeliibacillus stabekisii        WP_066790306  --V-T----E--A-S--V----T--IQM- D KQ-KD-E---F-DD------YV-SE--
Solibacillus isronensis            WP_079523312  --I-----------S-------L--ADM- E NK-KD-E---F-DD--D--VY------
Solibacillus kalamii               WP_087618074  --I-----------S-------L--ADM- E NK-KD-E---F-DD--D--VY------
Solibacillus silvestris            WP_014822526  --I-----------S-------L--ADM- E NK-KD-E---F-DD--D--VY------
Solibacillus sp. R5-41             WP_099425455  --I-----------S-------L--ADM- E NS-KD-E---F-DD--D--VY------
Sporosarcina pasteurii             WP_115363389  AGII--QMKQLHI-VE---Y--ST-IKL- E DKLNEHNAAD--D-----F-YSVEE--
Sporosarcina sp. EUR3 2.2.2        WP_024536769  --IM----YE----A-------S--VDMV D -H-ND-E---F-DD--D-FVSV-SE--
Sporosarcina sp. PTS2304           WP_114924904  --M-T-RMKEEKLLNDENAFTFN-TCI-L I EQNLTEANAVEIGDELIEFYCY-LKER
Sporosarcina ureae                 ARK21415      ----T-R-QTECILTDENAFTFSTACIEM I ENNLHEDNMKEIGDELIEFYCY-LKER
Tetzosporium hominis               WP_094943211      MARK-IDSQV-AVKAFAFSRSCMDV I DNRMSDVLFLQCG-ELIEFYVHVLSER
Ureibacillus thermophilus          QBK24599      --I-----ME----S-------V--VDM- Q NK-KD-E---F-D---D--VS------
Ureibacillus thermosphaericus      WP_016838327  --I-----ME----S-------V--IDM- Q NK-KD-E---F-D---D--VS------
Viridibacillus sp. OK051           WP_100795679  --V-T-R-IE--A-S-------V--V-M- D QR-ND-E---F-DD------YV-SE--
```

Supplemental Figure 25

A partial sequence alignment of the helix-turn-helix transcriptional regulator protein containing a one amino acid deletion (boxed) that is exclusively shared by all members belonging to the Jejuensis clade and absent in all other bacteria.

Group	Organism	Accession	91		136
**Jejuensis Clade (3/3)**	Lysinibacillus jejuensis	WP_108306792	WETLVKPAKRVKVGTVVTFG		NLLTATCTEILAHGGRKFKFSYDGIF
	Lysinibacillus saudimassiliensis	CDZ99862	--------------------		--------------------------
	Lysinibacillus sp. BF-4	WP_036142697	--------------------		--------------------------
**Other Bacteria (0/>200)**	Amphibacillus jilinensis	WP_017472695	--V------K--E---IS--	E	G------K-V-NQ---IVE-T-Q---
	Amphibacillus xylanus	WP_015009774	--V------K--L--KLS--	E	GR---------E----IVR-D-----
	Anaerobacillus macyae	WP_048312956	------------P--EIS--	D	GR---V---S-E----VLE-K---V-
	Anoxybacillus flavithermus	WP_004889269	---------------IIS--	D	GR-R-E-VAE-E----ILS-------
	Anoxybacillus pushchinoensis	WP_091702227	----------------IS--	D	GR-R-E-VAE-E----VLS-------
	Bacillus acanthi	WP_108669470	--A-------------LS--	D	GK-----E-E-D----IIS-K-E---
	Bacillus aidingensis	WP_026700425	----I------TL---IY--	G	-K-Q-E---L-E----MLR-T-K-V-
	Bacillus altitudinis	WP_073413640	------------N---I---	D	GR-K-V---EME-----VE-H-T---
	Bacillus alveayuensis	WP_044894670	------------I--TIS--	D	GR-K---IDTVE----MLE-----V-
	Bacillus amyloliquefaciens	WP_045510303	-----------RK---L---	D	GR---V---E-D-----I--R-----
	Bacillus anthracis	WP_044025097	------------E---IS--	E	GK-K----GTADQ---QLE-------
	Bacillus atrophaeus	WP_106044036	------------K---L---	D	G--Q-V---E-E-----VE-R-----
	Bacillus australimaris	WP_060697486	------------K---I---	D	GR-Q-V---EME-----VE-Q-T---
	Bacillus azotoformans	WP_003331778	----A----------II---	D	GR-K-K-ISEGD----H-E-------
	Bacillus boroniphilus JCM 2173	GAE43922	----I-------E--KIV--	D	G----E--GEAE----NL--------
	Bacillus cavernae	WP_126865574	----------I-E--KIV--	D	GK------DV-D----VLA-A-E---
	Bacillus cecembensis	WP_057988181	--------------------	E	G-------GE-E----L---D-----
	Bacillus cereus	WP_000354024	------------E---IS--	E	GK-K----GMADQ---QLE-------
	Bacillus cihuensis	WP_028392062	----------I-E-S-IV--	D	GK-K-V--GV-E----ILD-------
	Bacillus coagulans	WP_029141942	------------T----R--	D	GK-S-V-A-E-D----VM--R-E---
	Bacillus fastidiosus	WP_066230424	----I-------E---I---	N	GA-K---VGTAD----QL--D-E---
	Bacillus glycinifermentans	WP_048353542	------------K---L---	D	GR---V-K-E-D-----IE-R-E---
	Bacillus gottheilii	WP_080844700	----I-------E---I---	D	G--S---IGEAE----LLE---E---
	Bacillus halodurans	WP_010897395	----------L----IIR--	N	GE-----VQE-E----VLE---E---
	Bacillus halotolerans	WP_106020188	------------K-------	D	GR-K-V---E-E-----VE-Q-----
	Bacillus haynesii	WP_043925417	------------R---LS--	D	GR---V--DE-E-----IE-R-E---
	Bacillus jeotgali	WP_102263709	----I-------E--KIV--	D	G----E--GEAE----NL--------
	Bacillus korlensis	WP_066050041	------------E--IIS--	N	G----V-VGETE----TLE---E---
	Bacillus licheniformis	WP_124932249	------------R---LS--	D	GR---V---E-E-----IE-R-E---
	Bacillus litoralis	WP_066334421	------------E---I---	S	GI-K---VGTSE----LLQ-D-----
	Bacillus lonarensis	WP_090775823	----------------L---	E	GR-Q----AV------M-S-----V-
	Bacillus mesonae	WP_127488162	------------E----E--	D	G----V--GESD----T-E-K-E---
	Bacillus mojavensis	WP_010335133	------------K-------	D	GR-K-V---E-E-----VE-H-----
	Bacillus nakamurai	WP_061522919	------------K---L---	D	GR-K-V---E-D-----VE-R-----
	Bacillus ndiopicus	WP_042475086	----------------I---	E	G--R-E-VGE-E----I---Q-E---
	Bacillus nealsonii	WP_101176361	--S-------------IS--	D	GH-K-V-VQE-E----VLD-Q-----
	Bacillus niameyensis	WP_062104576	----------I-I--KIS--	N	GE---V--KEMD----V-E-H-E---
	Bacillus novalis	WP_066090442	---------------EID--	D	G----V--GTSD----V-E-K-E---
	Bacillus okuhidensis	WP_053431596	----------L----IIR--	N	GE-----VQE-E----VLE---E---
	Bacillus psychrosaccharolyticus	WP_040373107	----------IR---K-V--	D	GK---V---E-E----I-D-Q-E---
	Bacillus pumilus	WP_126740763	------------K---I---	D	GR-Q-V---E-E-----VE-Q-T---
	Bacillus rubiinfantis	WP_042354939	------------K---IE--	E	GV---V---EAE----V-Q-T-----
	Bacillus safensis	WP_126681528	------------K---I---	D	GR-K-V---EME-----VE-Q-T---
	Bacillus salarius	WP_125554632	------------S--ILS--	E	GK-K-E--D--E----MLR---E-V-
	Bacillus salsus	WP_090854830	----------I---S-IS--	D	G--K-V--AESE----YLT-Q-----
	Bacillus selenatarsenatis	WP_041967764	----I-------E--KIV--	D	G----E--VEAE----SLR-------
	Bacillus siamensis	WP_099744703	------------K---L---	D	GR---V---E-D-----IE-R-----
	Bacillus sinesaloumensis	WP_077618935	------------E---L---	D	GR-K----A-GE----MLE-H-E---
	Bacillus sonorensis	WP_118310502	------------R---L---	D	GR---V---E-D-----IE-R-E---
	Bacillus sp. 17376	WP_023627167	----I-------E--KIV--	D	G----E--GEAE----NL--------
	Bacillus sp. 2D02	WP_125586676	------------K---I---	D	GR-Q-V---EME-----VE-Q-T---
	Bacillus sp. 5B6	EIF14091	------------K---L---	D	GR---V---E-D-----IE-R-E---
	Bacillus sp. 7586-K	WP_095303157	------------E---IS--	D	GI-----VGTSE----LLT-D-----
	Bacillus sp. 7705b	WP_095714302	------------K-------	D	GR-K-V---E-E-----VE-Q-----
	Bacillus sp. AFS017274	WP_098369654	----------I---STIV--	D	GK-S-V--GV-E----ILE-K-----
	Bacillus sp. F56	WP_069839608	----A-------K-------	D	GR-K-V---E-E-----ME-Q-----
	Bacillus sp. FJAT-22090	WP_053588780	---------------KIS--	D	G----V---LKE----V-N-E-E-V-
	Bacillus sp. FJAT-26652	WP_053357718	------------P----S--	N	G--K-V-L-ETDQ---T---E-E---
	Bacillus sp. FJAT-27986	WP_066105476	----A-----IR---E-V--	D	GQ---V-VKEME----E-E-K-----
	Bacillus sp. FJAT-42376	WP_123918123	----I-------A---I---	D	GK-----V-EQE----TLH-------
	Bacillus sp. FJAT-44876	WP_096188244	------------K----Q--	D	G------V-E-PE-R--LQ--FQ---
	Bacillus sp. FJAT-46582	WP_100332079	----------I-E-S-IS--	D	GR------G-KD----MLE-------
	Bacillus sp. G1(2015b)	WP_058838996	------------K---I---	D	GR-K-V---EME-----VE-Q-T---
	Bacillus sp. HMF5848	WP_125907006	----I-----I-E-S-IH--	D	G--K----KM-D----HLE-T-----
	Bacillus sp. HNG	WP_116351616	------------E--ML---	D	GR------D-GD----MLE-H-E---
	Bacillus sp. LF1	WP_090637780	------------E--EID--	N	G----V--GTSD----I-N-T-----
	Bacillus sp. LLTC93	WP_105926061	------------K---I---	D	GR-Q-V---E-E-----VE-R-T---
	Bacillus sp. MUM 13	WP_071350030	----------I---SKII--	D	GK---V--GV-D----ILD-Q-----
	Bacillus sp. Marseille-P3661	WP_102345434	-----------QE--I-S--	D	GR-K-V-V-TSE----H-E-K-----
	Bacillus sp. NMCC46	WP_106038021	------------K---I---	D	GR-Q-V---EME-----V--Q-T---
	Bacillus sp. NMTD17	WP_106071785	------------K---I---	D	GR-Q-V---EME-----V--Q-T---

Other Bacteria (0/>200)

Bacillus sp. NSP9.1	WP_026588089	------------R---L--- D GR---V---E-D-----IE-R-K---
Bacillus sp. Nf3	WP_107163295	------------K---I--- D GR-K-V---E-E-----VE-Q-T---
Bacillus sp. OV194	WP_091004231	------------P----S-- N G--K-V-L-ETDQ---T---E-E---
Bacillus sp. OxB-1	WP_041075323	------------I--I---- D GR-K-R--S--EQ---T-Q-L-----
Bacillus sp. SJS	WP_035405284	----I-------E--II--- D GK---K-V-EQE----TLH---E---
Bacillus sp. TE3	WP_129508043	----A-------K------- D GR-K-V---E-E-----ME-Q-----
Bacillus sp. TS-2	WP_045485777	------------K--EI--- D G--K---L-E-E----V-RLE-E---
Bacillus sp. UMB0899	WP_102229521	------------A---I--- S GS-K---VGTSD----LL--D-----
Bacillus sp. es.036	WP_098444385	------------P---IS-- D GR---V-K-S-E----VLE-Q-E-V-
Bacillus subterraneus	WP_125481703	----I-------E--KIV-- D G----E--GEAE----NL--------
Bacillus subtilis	WP_003222672	----A-------K------- D GR-K-V---E-E----RME-Q-----
Bacillus swezeyi	WP_076763361	------------R---L--- D GR---V---E-D-----IE-R-E---
Bacillus taeanensis	WP_113804871	------------N---LS-- D GR-----IKE-E----ILE-H-E---
Bacillus tequilensis	WP_024714881	----A-------K------- D GR-K-I---E-E-----ME-Q-----
Bacillus tuaregi	WP_071395886	----------I-N--KIV-- N G----V--QE-E----ILD-Q-E---
Bacillus vallismortis	WP_100740707	------------K------- D GR-K-V--DE-E-----VE-Q-E---
Bacillus velezensis	WP_104842707	------------K---L--- D GR---V---E-D-----IE-R-----
Bacillus xiamenensis	WP_008360998	------------K------- D GR-K-V---E-E-----VE-Q-T---
Bacillus zeae	WP_119113499	------------A---IA-- D G----V---ESD----VLD-K-E---
Bacillus zhangzhouensis	WP_034321311	------------K-M-I--- D GR-Q-V---E-E-----VE-Q-T---
Bavariicoccus seileri	WP_022795603	----------A-----IS-- D GQ--GEVI-V-E----RV----Q-V-
Bhargavaea beijingensis	WP_092096607	------------P------- D G--K-E--GVGD----T-----E---
Bhargavaea cecembensis	WP_035002117	------------P------- D G--K-E--GVGD----T-----E---
Bhargavaea ginsengi	WP_092051346	------------P------- D G--K-E--GVGD----T-----E---
Brochothrix campestris FSL F6-	EUJ41703	-----------R---KMS-- E GQ---V---E-E-----L----E---
Caryophanon latum	WP_066461025	----A-------I----S-- D G--Q-E-IGE-D----I---M-----
Caryophanon tenue	WP_066542253	----A-------I----S-- D G--Q-E-IGE-D----I---I-----
Chryseomicrobium excrementi	WP_100352364	------------I---IS-- D G-------A-KD----V--MN---V-
Edaphobacillus lindanitolerans	WP_084186606	----A------RP---IE-- D G--K-E--GVSD----T---T-----
Exiguobacterium alkaliphilum	WP_034817964	--A------K-R--AI-E-- D G--R-E-V-E-PE---R---D-----
Exiguobacterium aurantiacum	WP_081828342	--A------K-R--AI-E-- D G--R-E-V-E-PE---R---D-----
Exiguobacterium chiriqhucha	WP_058763242	--A------K-R--AI-E-- N G--R-E-V-E-PE---R---D-----
Exiguobacterium marinum	WP_026826454	--A------K-RI-AI-E-- D G--R-E-V-E-PE---R---D-----
Exiguobacterium mexicanum	WP_034776879	--A------K-R--AI-E-- N G--R-E-V-E-PE---R---D-----
Exiguobacterium oxidotolerans	WP_029331037	----A-------P---I--- D G--K-E-V-A-DD---IL--------
Exiguobacterium profundum	WP_074037470	--A------K-R--SI-E-- D G--R-E-V-E-PE---R---D-----
Exiguobacterium sp. AB2	WP_034809674	--A------K-R--AI-E-- D G--R-E-V-E-PE---R---D-----
Exiguobacterium sp. AM39-5BH	WP_128122996	--A------K-R--AI-E-- N G--R-E-V-E-PE---R---D-----
Exiguobacterium sp. AT1b	WP_074035193	--A------K-R--SI-E-- D G--R-E-V-E-PE---R---D-----
Exiguobacterium sp. JLM-2	WP_047795489	--A------K-R--SI-E-- D G--R-E-V-E-PE---R---D-----
Exiguobacterium sp. N4-1P	WP_088837523	----A-------P---I--- D G--K-E-V-A-DD---IL--------
Exiguobacterium sp. S17	WP_084675460	--A------K-R--AI-E-- N G--R-E-V-E-PE---R---E-----
Exiguobacterium sp. SH31	WP_071399449	--A------K-R--AI-E-- N G--R-E-V-E-PE---R---E-----
Exiguobacterium sp. SH5S13	WP_131468673	--A------K-R--AI-E-- N G--R-E-V-E-PE---R---E-----
Exiguobacterium sp. SH5S4	WP_131457389	--A------K-R--AI-E-- N G--R-E-V-E-PE---R---E-----
Exiguobacterium sp. SL-10	WP_131434280	--A------K-R--AI-E-- N G--R-E-V-E-PE---R---D-----
Exiguobacterium sp. SL-9	WP_131484806	--A------K-R--AI-E-- N G--R-E-V-E-PE---R---D-----
Exiguobacterium sp. TNDT2	WP_114165842	--A------K-R--AI-E-- N G--R-E-V-E-PE---R---D-----
Exiguobacterium sp. ZOR0005	WP_047374006	--A------K-R--AI-E-- N G--R-E-V-E-PE---R---D-----
Fictibacillus sp. FJAT-27399	WP_062234202	------------P----S-- N G--K-V-L-ETDQ---T---E-E---
Filibacter sp. TB-66	WP_124071595	---------------I---- D GR-K-Q--G--EQ---I-----E---
Geobacillus sp. 44B	WP_081159660	---------------EI--- D GR-K-V-VDT-E----VLE---Q---
Geobacillus sp. WCH70	WP_015864627	---------------EI--- D GR-K---VDT-E----ILE---Q---
Geomicrobium sp. JCM 19037	WP_042403068	--A---------E--TIS-- D GR-Q-V--NV-P----ELS-------
Geomicrobium sp. JCM 19038	WP_042413365	--A------------TIS-- D GR-K-S-----------LS---E-V-
Geomicrobium sp. JCM 19039	WP_042424323	--A---------E--TIS-- D GR-Q-V--NV-P----ELS-------
Geomicrobium sp. JCM 19055	WP_042359432	--A------------TIS-- D GR-K-------------LS---E-V-
Gracilibacillus halophilus	WP_003467443	--V------KL----K---- H GQ-----VDTME----VLR-D-----
Gracilibacillus lacisalsi	WP_018931466	--V------K-----TIS-- E GK-----VDTKE----V-R-Q-E---
Halobacillus aidingensis	WP_089652643	-DV------K-----KIV-- E GK-V-E---LQE-----V--------
Halobacillus kuroshimensis	WP_027955282	--V------K--E--KIV-- G GK---E----QE-----V----E---
Halobacillus litoralis	WP_128526346	--V-I----K-----RIV-- E GQ---E----QE-----V--------
Halobacillus mangrovi	WP_085029424	--V------K--A--KI--- N GQ-V-E----QE-----VR---E---
Halobacillus massiliensis	WP_082233721	--V-I----K--A--IIS-- D GQ---E---V-E-----VR-------
Halobacillus sp. BBL2006	WP_035550056	--V------K--I--KIV-- E GQ-V-E----QE-----VR---E---
Halobacillus sp. Marseille-P38	WP_101844414	--V-I----K--E--RIV-- N GQ-I-E---VQD-----V--L-----
Halobacillus trueperi	WP_115893515	-DV------K-----KIV-- E GK-V-E---LQE-----V--------
Halolactibacillus alkaliphilus	WP_089800332	--V-A----K------IS-- E GK-K-----L-E----LVS---E---
Jeotgalibacillus malaysiensis	WP_039810059	------------P---I--- D G----E-V-TGDQ-A-ILE-------
Jeotgalibacillus salarius	WP_134381666	----------I-P---I--- E G----E-IDTGDQ---ILE-------
Jeotgalibacillus sp. R-1-5s-1	WP_134376733	----------I-P--KIS-- N G----E-L-TGEQ---ILE-------
Kurthia gibsonii	WP_087680344	----------I--------- D G----E--GE-E----L---N-E---
Kurthia huakuii	WP_029499319	----------I-K----S-- D G-------NE-E----Q---D-E---
Kurthia massiliensis	WP_010288112	----------I-E--I-S-- D G-------GEMD--------N-E---

Other Bacteria
(0/>200)

Kurthia senegalensis	WP_010303554	----------I----T---- K G-------GEMD----T---N-E---
Kurthia sibirica	WP_109307044	---------------EL--- D G--K-V--GV-D----I---D-E---
Kurthia sp. 11kri321	WP_068452317	----------I--------- D G----E--GE-E----L---N-E---
Kurthia sp. 3B1D	WP_126991092	----------I-E----S-- N G-------SEMD--------D-E---
Kurthia zopfii	WP_109348272	----------I-K--I---- E G--K-V--AE-D----Q---E-E---
Lactobacillus acidipiscis	WP_056971654	----------A----EIS-- N GK----V-KE-D----MIE-H-----
Lactobacillus apodemi	WP_025087849	----------A----KI--- N GE---VVK-E-E----IIE-A-----
Lactobacillus aviarius	WP_057827630	--------R-A----KIV-- N GE----V-KE-E----MI--E-----
Lactobacillus collinoides	WP_054761108	----M-----I----T---- D GQ----V-KE-E----MIE-H-----
Lactobacillus mixtipabuli	WP_089108950	----M----KM---STLS-- D G-----V--E-E----MIE-H-----
Lactobacillus odoratitofui	WP_056949031	----M-----I----T-S-- D G-----V--E-E----MIE-H-----
Lactobacillus oligofermentans	WP_057889386	-------G-KI-----L--- N GE----V--E-E----MIE-H-E---
Lactobacillus paracollinoides	WP_054710074	----M-----I----T---- D GQ----V-KE-E----MIE-H-----
Lactobacillus ruminis	WP_003698862	----------A----EIS-- D GK----V--E-E----MIE-H-----
Lactobacillus silagincola	WP_098825708	----M-----IR--S-IS-- D G-----V-KE-E----MIE-H-----
Lactobacillus sp. YK43	WP_124976129	----------A----EIK-- D GE----V--E-E----MIE-H-----
Listeria welshimeri	WP_011702333	----------IRK-GTI--- N GA-K---L-E-E----ILE---E---
Lysinibacillus acetophenoni	WP_097148390	------------I--EI--- N G--K----GE-D----M---------
Lysinibacillus boronitolerans	WP_016991900	----------------I--- D G-------GE-E----T-E-Q-----
Lysinibacillus chungkukjangi	WP_107934597	---------------E---- D G--K-I--GE-D----L---------
Lysinibacillus composti	WP_124762269	---------------EI--- D G--K-----E-D----L---------
Lysinibacillus contaminans	WP_053582685	------------I------- D G-------GE-E----L-E-K-----
Lysinibacillus endophyticus	WP_121212841	----A-----------IS-- E G--K---IGE-D----L-E-------
Lysinibacillus fusiformis	WP_069482144	----------------I--- D G-------GE-D----T-E-H-----
Lysinibacillus halotolerans	WP_122973231	---------------EIS-- D G--K---IGE-D----L---N-E---
Lysinibacillus macroides	WP_053996294	----------------I--- D G-------GE-E----T-E-Q-E---
Lysinibacillus manganicus	WP_036185806	---------------EL--- E G--K----GE-D----M-----E---
Lysinibacillus mangiferihumi	WP_107893764	-------------------- D G-------AE-D----M---K-----
Lysinibacillus massiliensis	WP_036176775	------------I--EI--- D G--K----GE-D----L---------
Lysinibacillus meyeri	WP_107839663	------------I--M---- E G--R-Q-VGE-D----I---Q-E---
Lysinibacillus odysseyi	WP_036156609	------------I---I--- G G--K----GM-E----S---------
Lysinibacillus parviboronicapi	WP_054768093	-------------------- D G-------GE-D----L-A-N-----
Lysinibacillus sinduriensis	WP_036201735	---------------EI--- D G--K---IGE-D----V---D-----
Lysinibacillus sp. 2017	WP_108713365	-------------------- D G-------GE-D----T---E-E---
Lysinibacillus sp. B2A1	AVK84150	-------------------- D G-------GE-D----T-E-N-N---
Lysinibacillus sp. BK089	WP_132356977	-------------------- D G-------GE-E----T-E-K-N---
Lysinibacillus sp. FJAT-14222	WP_053596161	-------------------- D G-------GE-D----T-E-K-N---
Lysinibacillus sp. FJAT-14745	WP_053484806	-------------------- D G-------GE------T-E-K-N---
Lysinibacillus sp. Marseille-P	WP_106780115	------------T---I--- D GI-K---IGE-E----L---N-----
Lysinibacillus sp. OL1	WP_131520492	----------------I--- D G-------GE-E----T-E-Q-----
Lysinibacillus sp. SYSU K30002	WP_126657240	---------------EIS-- E G--K---INE-E----V---H-----
Lysinibacillus sp. YLB-03	WP_118875695	----------------I--- D G--K----GE-D----V-Q---E---
Lysinibacillus sp. ZYM-1	WP_054609188	----------------I--- D G-------GE-D----T-E-H-----
Lysinibacillus sphaericus	WP_075527757	----------I---N--S-- D G--K-E--AV-PQ---E---T-N---
Lysinibacillus telephonicus	WP_126292709	----------------I--- D G--K----GE-D----M---------
Lysinibacillus varians	WP_025220112	-------------------- D G-------AE-D----M-E-K-E---
Lysinibacillus xylanilyticus	WP_068983754	-------------------- D G-------GE-D----T-E-K-N---
Lysinibacillus xyleni	WP_097072733	----A-----------IS-- E G--K---IGE-D----L---------
Marinococcus halophilus	WP_079476912	------------P------- D GR---E--KV-E----EMV-T-----
Marinococcus halotolerans	WP_022793794	------------P------- D G----E--KV-E----EMV-T-----
Marinococcus luteus	WP_091612058	------------P------- D GR---E--KV-E----EMV-T-----
Mycobacteroides abscessus subs	SHP92906	------------E--IIS-- N G----E-VGESE----TLE---E---
Oceanobacillus sojae	WP_077603154	--V------K--E---ID-- D G--K-V----KE----TVT-T-----
Paenisporosarcina antarctica	WP_134209483	------------I--M-S-- E G--R-Q-VGEAD----L---T-E---
Paenisporosarcina indica	WP_075618182	------------I----S-- E G--R-Q-VGEAD----L---T-----
Paenisporosarcina sp. HGH0030	WP_016426810	--S---------I----S-- E G--R-E-VGEAN----L---T-----
Paenisporosarcina sp. OV554	WP_108586414	-------S----I----S-- E G--R-Q-VGEAN----L---T-E---
Paenisporosarcina sp. TG-14	WP_017381203	------------I--M-S-- E G--R-Q-VGEAD----L---T-E---
Paenisporosarcina sp. TG20	WP_019414055	------------K--I-S-- E G--R-Q-VGETN----L---------
Parageobacillus caldoxylosilyt	WP_017435546	---------------EI--- D GR-K-V-VDT-E----VLE---Q---
Parageobacillus genomosp. 1	WP_043905598	---------------EI--- D GR-K-V-IDT-E----ILE---Q---
Parageobacillus thermantarctic	WP_090947508	---------------TII-- D GR-K---VNT-E----ILQ---R---
Parageobacillus toebii	WP_062677162	---------------EI--- D GR-K---VDT-E----ILE---Q---
Paucisalibacillus globulus	WP_026906320	--V-A-----I----KL--- D GK-K----D--E----I-D-E-----
Pediococcus argentinicus	WP_057799179	----------A-L--KID-- D G-----V-KE-E----EIE-------
Planococcus antarcticus	WP_081487824	----A----K-----I-S-- E G--R-E--G--D----H--MI-----
Planococcus citreus	WP_121300112	---------K--I----S-- D G--R-E--GV-D----H---I-----
Planococcus donghaensis	WP_008431515	---------K--I----S-- E G--R-E--G--D----H--MI-----
Planococcus faecalis	WP_078080324	----T----K-------S-- D G--R-E--G--D----H--MI-----
Planococcus halocryophilus	WP_008498938	---------K--I----S-- E G--R-E--G--D----H--MI-A---
Planococcus halotolerans	WP_112223753	---------K-------S-- E G--R-E--G--D----H--MI-----
Planococcus kocurii	WP_058385622	----T----K-------S-- D G--R-E--G--D----H--MI-----
Planococcus maitriensis	WP_112232465	---------K-------S-- D G--R-E--GV-D----Y---I-----
Planococcus maritimus	WP_083249489	---------K--I----S-- D G--R-E--GV-D----Y---I-----

Other Bacteria (0/>200)

Planococcus massiliensis	WP_052651853	---------K--I---IS-- D G--K-E--G--D----H--MI-----
Planococcus plakortidis	WP_084632783	---------K-------S-- D G--R-E--GV-D----Y---I-----
Planococcus rifietoensis	WP_083509125	---------K--I----S-- D G--R-E--GV-D----H---I-----
Planococcus salinarum	TAA69204	---------K-------S-- E G--R-E--G--D----H--MI-----
Planococcus salinus	WP_123164027	---------K-------S-- G G--R-E--G--E----H--MI-----
Planococcus sp. CAU13	WP_033544125	---------K-------S-- E G--R-E--G--D----H--MI-----
Planococcus sp. PAMC 21323	WP_038703627	---------K--I----S-- E G--R-E--G--D----H--MI-----
Planococcus versutus	WP_065524203	----A----K-----I-S-- D G--R-E--G--D----H--MI-----
Planomicrobium glaciei	WP_036806250	---------K------IS-- D G--K-E--AL-D----H--MI-----
Planomicrobium okeanokoites	WP_084246254	---------K-------S-- E G--R-E--G--D----H--MI-----
Planomicrobium soli	WP_106533805	---------K-------S-- E G--R-E--A--D----H--MI-----
Planomicrobium sp. MB-3u-38	WP_101803757	---------K-------S-- E G--R-E--G--D----H--MI-----
Planomicrobium sp. Y74	WP_121633541	---------K-------S-- E G--R-E--G--D----H--MI-----
Psychrobacillus insolitus	WP_111437794	-------S----I--EI--- E G--K-V----KD----I-R-V-S-V-
Psychrobacillus psychrotoleran	WP_093537025	---------------EI--- D G--K-I--D-KD----I-R-E-T-V-
Psychrobacillus sp. FJAT-21963	WP_056831561	------------I--KIS-- D G----V---LKE----V-N-E-E-V-
Psychrobacillus sp. OK028	WP_093060545	------------I--EI--- E G--K-V--D-KD----I-R-E-T-V-
Psychrobacillus sp. OK032	WP_093266222	------------I--TIV-- D G--R-V----KE----I-R-E-A-V-
Rummeliibacillus pycnus	WP_102691208	---------------EI--- D G--K----GE-E----M---N-E---
Rummeliibacillus stabekisii	WP_066788477	----------------L--- E G--K----GE------M---E-E---
Saliterribacillus persicus	WP_114353424	--V------K--I--TI--- D -K-----IKE-E----ILR---S---
Sinobaca qinghaiensis	WP_120193484	---------KI-T---I--- G GK---E---LQE----ILQ---E-V-
Solibacillus isronensis	WP_079528011	------------T------- D G------GE-D----T---T-----
Solibacillus silvestris	WP_065215613	------------T------- D G------GE-D----T---T-----
Solibacillus sp. R5-41	WP_099424663	----------I--------- E G------GE-E----L---D-----
Sporosarcina koreensis	WP_060205345	-------------------- D GR-K-E-----EQ---I---R-E---
Sporosarcina newyorkensis	WP_040759190	-------------------- D GR-Q-K--A-GEQ---E-A-M-----
Sporosarcina pasteurii	WP_115361455	----------I-E-N--S-- N G--N-E--AV-PQ---E---T-N---
Sporosarcina psychrophila	WP_067208188	----A-------L--I---- D GR-K-E--G--EQ---T---I-----
Sporosarcina sp. EUR3 2.2.2	WP_024534394	------------M----S-- E G--R-Q-VGEAN----L---T-----
Sporosarcina sp. HYO08	WP_067403244	-------------------- D GR-K-E--G--EQ---T---------
Sporosarcina sp. P13	WP_099688040	-------------------- D GR-Q-K--A-GEQ---E-T-I-----
Sporosarcina sp. P16b	WP_099672907	-------S--I--------- D G--Q-E--ALGEQ---E---------
Sporosarcina sp. P18a	WP_099674590	-------S--I--------- D G--Q-E--ALGEQ---E---------
Sporosarcina sp. P19	WP_099692182	-------S--I----I---- D G--Q-E--ALGEQ---E---------
Sporosarcina sp. P2	WP_099629832	-------S--I--------- D G--Q-E--ALGEQ---E---------
Sporosarcina sp. P20a	WP_099677283	-------S--I----I---- D G--Q-E--ALGEQ---E---------
Sporosarcina sp. P29	WP_099662197	-------S--I--------- D G--Q-E--ALGEQ---E---------
Sporosarcina sp. P3	WP_099638978	-------S--I--------- D G--Q-E--ALGEQ---E---------
Sporosarcina sp. P33	WP_081242072	----------I--------- D G--R-E--A-GEQ---E-T-A-----
Sporosarcina sp. P34	WP_099695364	-------S--I----I---- D G--Q-E--ALGEQ---E---------
Sporosarcina sp. P35	WP_099662736	----------I--------- D G--R-E--A-GEQ---E-T-A-----
Sporosarcina sp. P37	WP_085430061	----------I--------- D G--R-E--A-GEQ---E-T-A-----
Sporosarcina sp. P7	WP_099636694	-------S--I--------- D G--Q-E--ALGEQ---E---------
Sporosarcina sp. PTS2304	WP_114923897	-D----------I------- D GR-Q-K--A-GEQ---E-E-I-----
Sporosarcina ureae	WP_085426375	-------S--I----I---- D G--Q-E--ALGEQ---E---------
Streptohalobacillus salinus	WP_110251545	--V------K-----KIS-- Q GK-M----DV-E----LVR---E---
Terribacillus saccharophilus	WP_095218195	--V------K------LS-- D GK---V-K-L-E----IVE-R-----
Terribacillus sp. 7520-G	WP_095215520	--V------K-----ELS-- D GK---V-K-L-E----IIE-K-----
Tetzosporium hominis	WP_094944226	------------I---IS-- D G-------A-KD----V--MN---V-
Thalassobacillus sp. C254	WP_054635547	------------P---IE-- D GR-K-V--DV-E----VLD-Q-E-V-
Thermolongibacillus altinsuens	WP_132947656	---------------TLS-- D GR-K---V-A-E----VLE-A-----
Ureibacillus thermophilus	QBK26367	------------I--KI--- D GRM-----D--E----I-E-H-----
Ureibacillus thermosphaericus	WP_016838395	------------I--TIS-- D G--R----DV-D----I-E---E---
Viridibacillus arvi	WP_053416468	---------------EI--- E G--K----GE-D----L---N-E---
Viridibacillus sp. OK051	WP_100794381	------------I--EI--- E G--K----GE-D----L---D-E---

Supplemental Figure 26

A partial sequence alignment of the tRNA preQ1(34) S-adenosylmethionine ribosyltransferase-isomerase QueA protein containing a one amino acid deletion (boxed) that is exclusively shared by all members belonging to the Jejuensis clade and absent in all other bacteria.

Group	Organism	Accession	497		535
**Jejuensis Clade (3/3)**	Lysinibacillus jejuensis	WP_108306840	PFLRDAYNAVFVHLLAFYE		KNAPDYQRFLMQLDEGELRK
	Lysinibacillus sp. BF-4	WP_036145758	-------------------		--E-N-----T--E-A----
	Lysinibacillus saudimassiliensis	CEA05101	-------------------		--E-N-----T--E-A----
**Other Bacteria (0/>100)**	Bacillus cecembensis	WP_057989591	--IHEE-LS---R-VG---	E	YEMA-FH--VEV-NDN----
	Bacillus ndiopicus	WP_042478277	--V-EE-MV---R-IG---	E	FSTA-----VEVIEDS----
	Bacillus sp. B14905	WP_083796504	--V----M----R-VG---	E	YGH------MEI--DA----
	Bacillus sp. FJAT-22090	WP_053588853	-I--V-E-AY-R-TG---	D	YPEG-F----EVINDADI--
	Bacillus sp. OxB-1	WP_041075208	-IQ---Q-I-IR-AG---	Q	HGN--FH--SES-EDRD---
	Bhargavaea beijingensis	WP_092096800	--FH-E-T-I--R-AG---	E	YGE--VH--CET--DS---R
	Bhargavaea cecembensis	WP_063178335	--FH-E-T-I--R-AG---	E	YGE--VH--CET--DP----
	Bhargavaea ginsengi	WP_092051229	--FH-E-T-I--R-AG---	E	YGE--VH--CET--DP----
	Butyricicoccus sp. 1XD8-22	RKJ07925	--VS-E------R-VG---	E	HESS------EM--DSS---
	Caryophanon latum	WP_066464571	--IH-VFT--YIR-IG---	Q	HDK--F----E--EDV----
	Caryophanon tenue	WP_066542642	--IH-VFT--YIR-IG---	Q	HDRA-----IE--EDI----
	Domibacillus antri	WP_075397677	--TI-EHQ-LYTY--GY--	K	AEH--TAS---V-QDDQ--R
	Domibacillus mangrovi	WP_073710142	--TM-EHQ-L-TY--GY--	Q	A----TAS--TV-PDDQ--R
	Edaphobacillus lindanitolerans	WP_076758390	--FH-E-T-IY-N-AG---	E	YGE--VH--AET-QDPD--R
	Filibacter sp. TB-66	WP_124071494	-V--E-A-I--R-AG---	E	HGT--FH--AET-EDRN---
	Kurthia gibsonii	WP_121175897	--V-ED-L-IY---MG---	S	HEKA-----AEIVEDR----
	Kurthia huakuii	WP_029499195	--V--E-V-IYI---S--A	H	HDKA-F---AEM-TDS----
	Kurthia massiliensis	WP_010287943	--V--E-V-IYI---S--N	H	HDK--F---AEM-NDA----
	Kurthia senegalensis	WP_010303334	--V-EE-K-I-NE-VD---	Q	HEQA-FH--AEG-EDRN---
	Kurthia sibirica	WP_109305939	--V-ED-L-IYA--MG---	T	FEKE-----VEI-EDR----
	Kurthia sp. 11kri321	WP_068452651	--V-ED-L-IY---MG---	S	HEKA-----AEIVEDR----
	Kurthia sp. 3B1D	WP_126990304	--V--E-V-IYI---S--A	H	HDK--F---AEM-TDS----
	Kurthia zopfii	WP_109348378	--V-ED-I-I----MG---	T	HDK------AEVVEDR----
	Lysinibacillus acetophenoni	WP_097148303	--VH-E-V----R-IG---	E	HDTA------EI--DN----
	Lysinibacillus boronitolerans	WP_081977457	--V----M----R-VG---	E	YGH------VEI--DA----
	Lysinibacillus chungkukjangi	WP_107935413	--V--E-S----R-IG---	E	HGSA------EI-EDS----
	Lysinibacillus composti	WP_124762100	--A--E-I----R-IG---	E	HVSA------EV--DS----
	Lysinibacillus contaminans	WP_082332671	--V----M----R-IG---	E	FGH------VEV--DP----
	Lysinibacillus endophyticus	WP_121213313	--AK-D-A----R-IG---	E	HSSA------EIIEDPA---
	Lysinibacillus fusiformis	WP_004227161	--V----M----R-VG---	E	YGH------VEI--DA----
	Lysinibacillus halotolerans	WP_122971336	--A--E-A----R-IG---	E	HGTA------EI--DS----
	Lysinibacillus macroides	WP_083448598	--V----M----R-VG---	E	YGH------VEI--DA----
	Lysinibacillus manganicus	WP_036186253	--VH-E-A----R-IG---	E	HETA------EI--DSV---
	Lysinibacillus massiliensis	WP_036175949	--VS-E------R-VG---	E	HESS------EM--DSS---
	Lysinibacillus meyeri	WP_107841726	--V--E-M----R-IG---	E	YSMA-----VEVIEDS----
	Lysinibacillus odysseyi	WP_036157860	--I--D-KT--IK-IG---	E	YSSS--H--VEL---AD---
	Lysinibacillus parviboronicapi	WP_107950241	--V----L----R-VG---	E	YGHS-----VEI--DA----
	Lysinibacillus sinduriensis	WP_036201812	--V--E-G----R-VG---	E	HGSA------EI-EDS----
	Lysinibacillus sp. 2017	WP_108714429	--IHEE-L----R-IG---	E	YEMA-FH--VEE-NDN----
	Lysinibacillus sp. AC-3	SKC17133	--V----L----R-VG---	E	FGH------VEI--DS----
	Lysinibacillus sp. AR18-8	WP_083225209	--V----M----R-VG---	E	YGH------VEI--DA----
	Lysinibacillus sp. B2A1	AVK86983	--V----M----R-VG---	E	YGH------VEI--DA----
	Lysinibacillus sp. BK089	WP_132356814	--V----M----R-VG---	E	YGH------VEI--DA----
	Lysinibacillus sp. F5	KUF30966	--V----M----R-VG---	E	YGH------VEI--DA----
	Lysinibacillus sp. FJAT-14222	WP_082340141	--A----M----R-VG---	E	YGH------VEI--DS----
	Lysinibacillus sp. FJAT-14745	WP_082337536	--A----M----R-VG---	E	YGH------VEI--DS----
	Lysinibacillus sp. LK3	KMN39857	--V----M----R-VG---	E	YGH------VEI--DA----
	Lysinibacillus sp. Marseille-P	WP_106779981	--V--E-K----R-IG---	E	HSSA------EI-ED-S---
	Lysinibacillus sp. SG8	SCX56892	--V----M----R-VG---	E	YGH------MEI--DA----
	Lysinibacillus sp. SG9	SCY75295	--V----M----R-VG---	E	YGH------VEI--DA----
	Lysinibacillus sp. SYSU K30002	WP_126657149	--A--E-R----R-IG---	E	HQTA------EMI-DPA---
	Lysinibacillus sp. YLB-03	WP_118875596	--I--G------R-IG---	E	HGS-------EV--DPD---
	Lysinibacillus sp. YR326	WP_134018777	--V----M----R-VG---	E	YGH------VEI--DS----
	Lysinibacillus sp. ZYM-1	WP_082389355	--V----M----R-VG---	E	YGH------MEI--DA----
	Lysinibacillus sphaericus	AMO32895	--V----M----R-VG---	E	YGH------MEI--DA----
	Lysinibacillus sphaericus	KEK12187	--V----M----R-VG---	E	YGH------MEI--DA----
	Lysinibacillus sphaericus	POZ55693	--V----M----R-VG---	E	YGH------VEI--DA----
	Lysinibacillus sphaericus	SPU38111	--V----M----R-VG---	E	YGH------VEI--DA----
	Lysinibacillus sphaericus	SUV18015	--V----M----R-VG---	E	YGH------VEI--DA----
	Lysinibacillus sphaericus	WP_012295243	--V----M----R-VG---	E	YGH------MEI--DA----
	Lysinibacillus sphaericus	WP_075528024	-FHED-ATI--R-AG---	Q	YKK--FH--AESIEDR----
	Lysinibacillus sphaericus	WP_080647672	--V----M----R-VG---	E	YGHS-----VEI--DL----
	Lysinibacillus sphaericus	WP_080709215	--V----M----R-VG---	E	YGH------MEI--DA----
	Lysinibacillus sphaericus	WP_081328041	--V----M----R-VG---	E	YGH------VEI--DA----
	Lysinibacillus sphaericus	WP_103976210	--V----M----R-VG---	E	YGH------VEI--DA----
	Lysinibacillus sphaericus	WP_125103623	-VH-D-ATIYI--AG---	Q	YG---FH--AETIEDR----
	Lysinibacillus sphaericus OT4b	EON71767	--V----M----R-VG---	E	YGHS-----VEI--DL----
	Lysinibacillus telephonicus	WP_126292832	--A--E-A----R-IG---	E	HAS---H---EI-EDQ----
	Lysinibacillus varians	AHN22520	--V----M----R-VG---	E	YGH------VEI--DA----
	Lysinibacillus xylanilyticus	WP_068983601	--V----M----R-VG---	E	YGH------VEI--DS----
	Lysinibacillus xyleni	WP_097074545	--AK-E-A----R-VG---	E	HS-A------EI-EDPA---

Other Bacteria (0/>100)

Paenisporosarcina indica	WP_075618111	-IH-S-E-I--R-IG--D S HSQA-TH---EI-EDT----
Paenisporosarcina quisquiliaru	WP_090563809	-I----EV---R-TG--- E YPDG-F----EV-NDAQ---
Paenisporosarcina sp. HGH0030	WP_016426906	-IH-P-E-I--R-IG--D T YPQA-TH---EI-EDT----
Paenisporosarcina sp. OV554	PUB12546	-IH-S-E-I--R-IG--D M YPQA-TH---EI-EDTD---
Planococcus antarcticus	WP_006828684	--ISEEFQ-LY-Q--G--- E WDKA-FHK--ET-QDA----
Planococcus citreus	WP_121300013	--ISEE-Q-LY-Q--G--- E WEKA-FHK--ET-TDQ----
Planococcus donghaensis	WP_008431630	--VSEEFQ-LY-Q--G--- E WDKA-FHK--ET-QDA----
Planococcus faecalis	WP_071154373	--ISEEFR-LY-Q--G--- E WDKA-FHK--ET-QDT----
Planococcus halocryophilus	WP_008499003	--VSEEFQ-LY-Q--G--- E WDKA-FHM--ET-QDA----
Planococcus halotolerans	WP_112223816	--ISEEFQ-IY-Q--G--- E W-KA-FHK--ET-KDP----
Planococcus kocurii	WP_058385554	--ISEEFR-LY-Q--G--- E WDKA-FHK--ET-QDT----
Planococcus maitriensis	WP_112232343	--ISEE-Q-LY-Q--G--- E WEKA-FHK--ET-TDQ----
Planococcus maritimus	WP_068461985	--ISEE-Q-LY-Q--G--- E WEKA-FHK--ET-TDQ----
Planococcus massiliensis	WP_052651787	--VSEEFK-LY-Q--G--- E WEKA-FHK--ET-RDP----
Planococcus plakortidis	WP_068869031	--ISEE-Q-LY-Q--G--- E WEKA-FHK--ET-TDQ----
Planococcus rifietoensis	WP_058380924	--ISEE-Q-LY-Q--G--- E WEKA-FHK--ET-TDQ----
Planococcus salinarum	TAA66004	--ISEEFQ-IY-Q--G--- E W-KA-FHK--ET-QDA----
Planococcus sp. CAU13	WP_033541085	--ISEEFQ-IY-Q--G--- E W-KA-FHK--ET-QDA----
Planococcus sp. PAMC 21323	WP_038703693	--VSEEFQ-LY-Q--G--- E WDKA-FHK--ET-QDA----
Planomicrobium glaciei	WP_036809442	--VSEEFK-LY-Q--G--- E WDKA-FHK--ET-QDA----
Planomicrobium okeanokoites	WP_084246816	--ISEEFQ-IY-Q--G--- E W-KA-FHK--ET-QDP----
Planomicrobium soli	WP_106532699	--ASEEFQ-LY-Q--G--- E WDKA-FHK--ET-QDA----
Planomicrobium sp. MB-3u-38	WP_101803790	--ISEEFQ-IY-Q--G--- E W-KA-FHK--ET-QDP----
Planomicrobium sp. Y74	WP_121633636	--ISEEFQ-IY-Q--G--- E W-KA-FHK--ET-KDP----
Psychrobacillus insolitus	WP_111437870	-I--I-E-A--R-TG--- D FPNG------EMVNDP----
Psychrobacillus psychrodurans	WP_093496109	-I----EV---R-TG--- E YPDG-F----EV-NDAQ---
Psychrobacillus psychrotoleran	SFQ76097	-I----EV---R-TG--- E YPDG------EV-NDA----
Psychrobacillus sp. FJAT-21963	WP_056831662	-I----E-AY-R-TG--- D YPEG-F----EVINDADI--
Psychrobacillus sp. OK028	WP_093060622	-I----EV---R-TG--- E YPDG------EV-NDA----
Psychrobacillus sp. OK032	SER83069	-I----E--Y-R-TG--- Q YPDG------EV-NDAQ---
Psychrobacillus sp. OK032	WP_093266055	-I----E--Y-R-TG--- Q YPDG------EV-NDAQ---
Rummeliibacillus pycnus	WP_102691313	--V--D-M-IYI--IG--- D HQKA-----AEVIGDR----
Rummeliibacillus sp. POC4	WP_119414847	--V-ED-M-IYI--IG--- E HQKA-----AEVIGDR----
Rummeliibacillus sp. TYF005	WP_124218002	--V-ED-M-IYI--IG--- E HQKA-----AEVIGDR----
Rummeliibacillus stabekisii	WP_066786428	--V-ED-M-IYI--IG--- E YEQA--K--AEVISDR----
Sinobaca qinghaiensis	WP_120193408	--HM--AA--Y---A D G-E--PGE--SY-ED-----
Solibacillus isronensis	WP_079525623	--IHEE-LS---R-IG--- E YEMA-FH--VEV--DHD---
Solibacillus sp. R5-41	WP_099422910	--IHEE-LS---R-VG--- E YEMA-F---VEV-NDN----
Sporosarcina koreensis	WP_040286635	-IH-E-ATLYIR-AG--- Q YGS--FH--SEMIE-R---R
Sporosarcina newyorkensis	WP_078816533	-V--DVIKI-FK-A---- Q YDL-N----AEM-EDRQ-K-
Sporosarcina pasteurii	WP_115360958	-FHED-ATI--R-AG--- Q YKK--FH--AESI-DR----
Sporosarcina psychrophila	WP_067210250	-V-ED-V-I-IR-AG--- Q HGI--FH--AES-EDR----
Sporosarcina sp. D27	WP_025784779	-VH-D-ATIYI--AG--- Q YG---FH--AETIEDR----
Sporosarcina sp. EUR3 2.2.2	WP_024534482	-IH-S-E-I--R-IG--D T YPHA-IH---EI-EDTD---
Sporosarcina sp. HYO08	WP_067403463	-IQ-N-S------AG--- Q YGT--FH--AES-QDR---R
Sporosarcina sp. P1	WP_099626515	-V--DIIKI-FK-A---- Q YDL-N----AET-EDRQ-K-
Sporosarcina sp. P16b	WP_099672146	-V--DVIKI-FK-A---- Q YDL-N----AEM-EDRQ-K-
Sporosarcina sp. P17b	WP_099625688	-V--DVIKI-FK-A---- Q YDL-N----AET-EDRQ-K-
Sporosarcina sp. P18a	WP_099674969	-V--DVIKI-FK-A---- Q YDL-N----AEM-EDRQ-K-
Sporosarcina sp. P2	WP_099630209	-V--DVIKI-FK-A---- Q YDL-N----AEM-EDRQ-K-
Sporosarcina sp. P20a	WP_099676844	-V--DVIKI-FK-A---- Q YDL-N----AET-EDRQ-K-
Sporosarcina sp. P26b	WP_099693249	-V--DVIKI-FK-A---- Q YDL-N----AET-EDRQ-K-
Sporosarcina sp. P3	WP_099637659	-V--DVIKI-FK-A---- Q YDL-N----AET-EDRQ-K-
Sporosarcina sp. P34	WP_099694406	-V--DVIKI-FK-A---- Q YDL-N----AEM-EDRQ-K-
Sporosarcina sp. P7	WP_099637299	-V--DVIKI-FK-A---- Q YDL-N----AEM-EDRQ-K-
Sporosarcina sp. PTS2304	WP_114923600	-V-EDIMKI-FK-A---- Q YDV-N----TEM--DRQ-Q-
Sporosarcina sp. ZBG7A	WP_039041685	-VH-D-ATIYI--AG--- Q YG---FH--AETIEDR----
Sporosarcina ureae	WP_085428930	-V--DVIKI-FK-A---- Q YDL-N----AET-EDRQ-K-
Ureibacillus thermophilus	QBK26265	--VH-E-I----R-IG-C- E HGTS------EI--DA----
Ureibacillus thermosphaericus	WP_016838554	--IH-E-M----R-IG--- E HGTA------EI--DA----
Viridibacillus arvi	WP_053416368	--V--D-L--Y---IG--- N HQKA-----AEV-EDS----
Viridibacillus sp. FSL H8-0123	WP_076064893	--V--D-L--Y---IG--- N HQKA-----AEV-EDS----
Viridibacillus sp. OK051	WP_100794283	--V--D-L-IY---IG--- S HQKS-----AEI-EDS----

Supplemental Figure 27

A partial sequence alignment of the DNA primase protein containing a one amino acid deletion (boxed) that is exclusively shared by all members belonging to the Jejuensis clade and absent in all other bacteria.

Group	Organism	Accession	162		210
Jejuensis Clade (3/3)	Lysinibacillus saudimassiliensis	CEA04024	PARRNEVKPRLPVGAVLHENTY		DENYDEQLQAFNATMENYYGSRSSNRK
	Lysinibacillus sp. BF-4	WP_036145034	----------------------		--------------------N------
	Lysinibacillus jejuensis	WP_108307705	----------------------		--------H----------S-------
Other Bacteria (0/>100)	Acidithiobacillus caldus	WP_004871403	-DQTV-------L----Q--R-	S	E-HDA-LIAEYD---QA------DH--
	Bacillus albus	WP_128974621	-DEDHG-------A-I----G-	D	EKK-G-L-NEYDE--SS--KA----K-
	Bacillus anthracis	WP_098760489	-DENHG-------A-I----G-	D	EKK---S-NEYDE--SS--KE----K-
	Bacillus badius	WP_063385451	--ES--------LE-----D--	N	EAK--QL-AEYDKQ-H---AA-TT-K-
	Bacillus cecembensis	WP_057988059	-TK---T------A------S-	D	VDQ-ETL-ADYD-M--S--A-----Q-
	Bacillus cereus	WP_046955178	-DENHG-------A-I----G-	D	EKK---L-NEYDE--SS--KE--A-K-
	Bacillus cytotoxicus	WP_087098248	-DQEH--------E-I------	D	VAK-ESL-ETYDE--SA--NA--T-Q-
	Bacillus fordii	WP_018705379	-EET---------D-I---D--	D	ASK-EKI-KEYDS---E--RN--A-N-
	Bacillus fortis	WP_120075672	--EK--------IE-I---D--	D	ENK--KI-KEYDSV--E--RN--A-S-
	Bacillus freudenreichii	WP_126432810	-EET---------E-I---D--	D	ESK---I-TKYDSV--E--R--TA-N-
	Bacillus luti	WP_071709718	-NEEHG-------A-I----G-	D	EKK---L-SEYDE--SA--KE----Q-
	Bacillus mobilis	WP_071721931	-DEDHG-------A-I----G-	D	EKK---L-NEYDE--SS--KE----K-
	Bacillus mycoides	WP_050000286	-NEEHG-------A-I----G-	D	EKK---L-NEYDE--SG--KE----Q-
	Bacillus ndiopicus	WP_042470462	-TK---T-------------A-	D	EVR-ATL-DEYDT------T-----Q-
	Bacillus proteolyticus	WP_071744985	-DQEHG-------A-I----G-	D	EKK---L-NEYDE--SA--KE----Q-
	Bacillus pseudomycoides	WP_098719401	-DESHA-------K-I----V-	D	ENK---L-EEYDR-TNE--KG--T-Q-
	Bacillus sp. 007/ AIA-02/ 001	WP_135238191	-DEEHG-------A------G-	D	EQK---L-NEYDE--NA--KE----K-
	Bacillus sp. 100374	WP_099683903	-NEDHG-------A-I----G-	D	EKK---L-NEYDE--NS--KE----K-
	Bacillus sp. 103mf	SFS75997	-DEKHG---------I------	D	ENK-N-L-DEYDR-TNE--KE-LT-Q-
	Bacillus sp. 3-2-2	WP_126049599	-EET---------E-I---D--	D	ESK---I-TKYDSV--E--R--TA-N-
	Bacillus sp. AFS033286	WP_098841497	-NEEHG-------A-I----G-	D	EKK---L-NEYDE--SA--KE----K-
	Bacillus sp. AFS059628	WP_098667199	-DEDHG-------A-I----G-	D	EKK---L-NEYDE--SS--KE----K-
	Bacillus sp. AG1163	WP_134197905	-DEDHG------IA-I----G-	D	EKK---L-NEYDE--SA--KE----Q-
	Bacillus sp. COPE52	AXK17704	-DEEHG-------A-I----G-	D	EKK---L-NEYDE--SA--KE----Q-
	Bacillus sp. G3(2015)	WP_058839610	-DEEHG-------A------G-	D	EQK---L-NEYDE--NA--KE----K-
	Bacillus sp. GZT	WP_062922172	-DEDHG-------T-I----E-	D	EKK---L-NEYDE--SA--KE----K-
	Bacillus sp. GeD10	WP_006922650	-DEDHG-------A-I----G-	D	EQK---L-NEYDE--NA--KE----K-
	Bacillus sp. MB366	WP_075677470	-DEDHG-------A------G-	D	EQK---L-NEYDE--NA--KE----K-
	Bacillus sp. MYb209	WP_105583942	-DEDHG-------A-I----G-	D	EKK---L-NEYDE--SA--KE----Q-
	Bacillus sp. NH11B	WP_071770102	-DQEHG-------A-I----G-	D	EKK---L-NEYDE--SA--KE----Q-
	Bacillus sp. OV752	WP_107887795	-NEDHG-------A-I----G-	D	EKK---L-NEYDE--SA--KE----Q-
	Bacillus sp. OxB-1	WP_041071754	-DES---------E-II--D--	D	A-K---L-P-YDE--NA--QN-GT-Q-
	Bacillus sp. SN10	WP_101165753	-DEDHG------IA-I----G-	D	EKK---L-NEYDE--SA--KE----K-
	Bacillus sp. SRB_28	WP_113770140	-NEEHG-------A-I----G-	D	EKK---L-NEYDE--SA--KE----Q-
	Bacillus sp. UNC438CL73TsuS30	WP_026574340	--E---------LD-I----Q-	N	T-K-ESL-DEYDQ--NT--QQ--T-N-
	Bacillus subtilis	QBJ67445	-DEDHG-------A-I----G-	D	EQK---L-NEYDE--SA--KE----K-
	Bacillus terrae	WP_120117099	-EEA---------E-I---D--	D	ENK-A-I-KQYDSV--E--RK--T-N-
	Bacillus thuringiensis	WP_098400471	-DEEHG-------A------G-	D	EQK---L-NEYDE--SA--KE----Q-
	Bacillus toyonensis	WP_098227871	-DEEHG-------A-I----G-	D	EKK---L-NEYDE--SA--KE----Q-
	Bacillus wiedmannii	WP_098057657	-DENHG-------A-I---DG-	D	EKK---L-NEYDE--SS--KE----K-
	Bhargavaea beijingensis	WP_125904398	-KF----------N------E-	N	S-K-E---SEYDR--NG--L--G----
	Bhargavaea cecembensis	WP_008297526	-KF----------S------E-	N	S-K-E-L-PEYDR--ND--LN-G----
	Bhargavaea ginsengi	WP_092055224	-KF----------TSI----E-	D	SKK-E---PEYDR--ND--L--G----
	Butyricicoccus sp. 1XD8-22	RKJ69005	-TK---T-----IE-II-----	D	V-K--HL-DEYD-I-----A-----Q-
	Caryophanon latum	WP_066461268	-TKV--T------A------S-	N	V-Q--TL-DEYDQM--D--A-----Q-
	Caryophanon tenue	WP_066546727	-TKV--T------A------S-	N	V-Q--TV-DEYDRI--D--A-----Q-
	Domibacillus antri	WP_075398703	--QT---------E-I--T-E-	D	ETK-ETL-DDYDE--RQ--A-----N-
	Domibacillus tundrae	WP_046178318	--QT-----------I--T-E-	N	ETK-ETL-DEYDDVIRD--A---T-N-
	Edaphobacillus lindanitolerans	WP_076759067	--V-H-------LE------E-	D	--K--RL-PEYDR-IRE--A---A-T-
	Lysinibacillus acetophenoni	WP_097150375	-TK---T------E--------	N	VDK---L-DQYDSI--D--A-----Q-
	Lysinibacillus boronitolerans	WP_016994441	-------------S------E-	N	V-K-E-L-P-Y-EE--K--N-------
	Lysinibacillus chungkukjangi	WP_107936933	-TK---T------E-I----G-	D	V-K--TL-DQYDT---E--A-----Q-
	Lysinibacillus composti	WP_124766966	-TK---T------S------E-	H	A-K-HSL-NDYDQV--D--A-------
	Lysinibacillus contaminans	WP_053585116	-S-----------A------E-	N	I-K-E-L-P-Y-E---A--N-------
	Lysinibacillus endophyticus	WP_121213439	-TK---T--------I----S-	N	T-K-N-L-DEYDSI-----A-----Q-
	Lysinibacillus fluoroglycofeni	WP_107941940	-TK---T------P------A-	D	EAK--AL-EEYD----G--A-----Q-
	Lysinibacillus fusiformis	WP_004229757	-------------S------E-	K	V-K-E-L-P-Y-EE--I--N-------
	Lysinibacillus halotolerans	WP_122971802	-TK---T------E-I----D-	N	A-K--TL-DEYD-------A-----Q-
	Lysinibacillus macroides	WP_053993299	-------------S-I----D-	N	I-K-ETL-P-Y-E---A--N-------
	Lysinibacillus manganicus	WP_036184312	-TK---T-----LD------S-	N	V-K--VI-DQYDSI--D--A-----Q-
	Lysinibacillus mangiferihumi	WP_107897385	-------------A------E-	N	C-K-E-L-P-Y-E---A--N-------
	Lysinibacillus massiliensis	WP_036178796	-TK---T-----IE-II-----	D	V-K--HL-DEYD-I-----A-----Q-
	Lysinibacillus meyeri	WP_107840062	-TK---T------A-----DS-	D	TAK--TL-DDYD-M--S--T---A-Q-
	Lysinibacillus odysseyi	WP_036153445	-TK---T------A------G-	D	E-K-ETL-DEYD-I--S--A-----Q-
	Lysinibacillus parviboronicapi	WP_107924541	-------------A------E-	N	AQK-E-L-P-Y-E-L-A--N-------
	Lysinibacillus sinduriensis	WP_036199636	-TK---T------E------G-	D	E-K--TL-DEYD---A--A-----Q-
	Lysinibacillus sp. 2017	WP_108711352	-TK---T------A------G-	N	VAQ--TL-DEYD----S--T-----Q-
	Lysinibacillus sp. B2A1	AVK82504	-------------A-I----E-	N	AQQ-E-L-PSY-E---A--N-------
	Lysinibacillus sp. BK089	WP_132363684	-------------A------E-	N	ADK-E-L-P-Y-D---T--NN------
	Lysinibacillus sp. FJAT-14222	WP_053594078	-------------S------E-	N	A-K-E-L-P-Y-D---A--NN------
	Lysinibacillus sp. FJAT-14745	WP_053486054	-T-----------A------E-	N	T-K-E-L-P-Y-D---A--NN------

Other Bacteria (0/>100)

Lysinibacillus sp. Marseille-P	WP_106782425	-TK---T------E------E-	D	IQK-EQL-DEYD----S--T-----N-
Lysinibacillus sp. SYSU K30002	WP_126659179	-TK---T------E-I----Q-	D	V-K--TL-NDYD----S--A-----Q-
Lysinibacillus sp. YLB-03	WP_118876615	-TK---T------E------N-	D	A-K-ETL-D-YD----D--A-----Q-
Lysinibacillus sp. YR326	WP_134022677	-------------A------E-	N	T-K-E-L-P-Y-D---A--NT------
Lysinibacillus sp. ZYM-1	WP_054612795	-T---D-------S------E-	N	A-K-Q-L-P-Y-E---M--NN------
Lysinibacillus sphaericus	VDG97901	--KS---------D------M-	D	AS--M-I-RDYD----T--ET------
Lysinibacillus sphaericus	WP_010857762	-V-----------A------E-	N	SQK-E-L-P-Y-ESLAA--T-------
Lysinibacillus sphaericus	WP_012292602	-V-----------S------E-	N	S-K---L-P-Y-E---A--N-------
Lysinibacillus sphaericus	WP_036117770	-------------S-I----E-	N	V-K-E-L-P-Y-----M--NN------
Lysinibacillus sphaericus	WP_036218110	-V-----------S------E-	N	S-K---L-P-Y-K---A--N-------
Lysinibacillus sphaericus	WP_069512950	-------------A------E-	N	C-K-E-L-P-Y-----D--N-------
Lysinibacillus sphaericus	WP_099805717	-V-----------S------E-	N	S-K---L-P-Y-E---A--N-------
Lysinibacillus sphaericus	WP_103977516	----------F--A------E-	N	C-K-E-L-P-Y-----D--N-------
Lysinibacillus sphaericus	WP_125102319	--KS---------D------M-	D	AS--M-I-RDYD----T--ET------
Lysinibacillus tabacifolii	WP_108030682	-------------A------E-	N	CDK-E-L-P-Y-E---D--N-------
Lysinibacillus telephonicus	WP_126295345	-TK---T------E------E-	D	V-K--SL-DEYDSI-----A-----N-
Lysinibacillus xylanilyticus	WP_100545183	-------------A------E-	N	T-K-E-L-P-Y-D---A--NN------
Lysinibacillus xyleni	WP_097073557	-TK---T--------I----S-	D	SAI-NDL-DEYDSI-----A-----Q-
Oceanobacillus caeni	WP_060667682	-SVI-D-------N-I------	L	E-K-EDM-EEYDQ--SD--Q--A--N-
Paenisporosarcina antarctica	WP_134210487	--EH---------D-I----K-	D	EVK---L-PTYDKEI-R--KN--T-E-
Paenisporosarcina indica	WP_075618195	-DEQH--------E-I----G-	D	S-K-HSL-S-YDK---E--ET-G--Q-
Paenisporosarcina sp. OV554	WP_108587240	-DEQH--------D------G-	D	SGK-NTL-PKYDK-I-E--A-----Q-
Paenisporosarcina sp. TG-14	WP_017380764	--EH---------D-I----K-	D	EVK---L-PTYDKEI-R--KN--T-E-
Paenisporosarcina sp. TG20	WP_019412803	-NEQH--------E-I----S-	D	S-K-NTL-P-YDK--DD--ST----Q-
Planococcus antarcticus	WP_006830083	-DEA-G---------I----E-	D	EAK-ETL-PEYDS-IQA--AG-G--Q-
Planococcus citreus	WP_121300884	-NES---------E------E-	D	EAK-A-I-P-YDE-IMS--AA--N-Q-
Planococcus maitriensis	WP_112232976	-KEA---------E------E-	D	EAK-E-I-P-YDE-IMA--AA--N-Q-
Planococcus plakortidis	WP_068871899	-NEA---------E------E-	D	EAK-A-I-P-YDE-IMH--SA--N-Q-
Planococcus rifietoensis	WP_058381878	-NEA---------E------E-	D	EAK-A-I-P-YDE-IMA--AA--N-Q-
Planococcus salinarum	TAA72635	-NEK-D-------E-I----D-	N	E-K-ERI-P-YDK-IQD-----G--Q-
Planococcus salinus	WP_123165672	-NEA---------E-I----E-	D	EAK-ETL-PEYDQ-LK---A--G--Q-
Planococcus sp. CAU13	WP_033543738	-NE----------E-I----N-	D	EKK-E-I-P-YDK-IQE--S--G--Q-
Planococcus sp. Y42	WP_077587907	--T----------E-I----A-	D	EDK-E-I-P-YDE-IQA------A-Q-
Planomicrobium flavidum	WP_088009175	-EK----------E------A-	D	EQK-KMI-PEYDE-VQA-----G--Q-
Psychrobacillus insolitus	WP_111440150	---Q---------A-I----A-	D	SSK-E-I-KEYDQ---A--A--G--Q-
Psychrobacillus psychrotoleran	WP_093537935	-DQQ---------T------N-	N	SAK-EVL-PEYDQ---D--K--G--Q-
Psychrobacillus sp. OK032	WP_093275098	-DQQ---------A------S-	D	SAK-ESL-TEYDR---E--A--G--Q-
Quasibacillus thermotolerans	WP_039236964	--E----------Q-IV---S-	D	E-K---L-SSYDEK--E--ST-AT-Q-
Rummeliibacillus pycnus	WP_102693237	-TK---T-----LD------S-	D	V-K--HL-DDYD--L-K--A-----Q-
Rummeliibacillus sp. POC4	WP_119415954	-TK---T-----LN-I----N-	D	V-K--QL-DEYD--L-T--A-----K-
Rummeliibacillus sp. TYF005	WP_124218204	-TK---T-----LN-I----N-	D	V-K--QL-DEYD--L-T--A-----K-
Rummeliibacillus stabekisii	WP_066791477	-TK---T-----LD------G-	D	E-K--TL-DEYD--L-E--QH----Q-
Solibacillus isronensis	WP_079523847	-TK---T------A------G-	D	VDK-ETL-DEYDGI--D--S-----Q-
Solibacillus kalamii	WP_087617153	-TK---T------A------G-	D	VDK--TL-DEYDGI--D--S-----Q-
Solibacillus silvestris	WP_014824880	-TK---T------A------G-	D	VDQ--TL-DEYDGI--D--S-----Q-
Solibacillus silvestris	WP_065217477	-TK---T------A------G-	D	VDQ--TL-DEYDGI--D--SN----Q-
Solibacillus sp. R5-41	WP_099422248	-TK---T------S------S-	D	VDQ-ETL-ADYD-M--T--A-----Q-
Sporosarcina koreensis	WP_060205740	-DEA---------E-II---S-	D	TAK---L-P-YDQ--HE--LN-G--Q-
Sporosarcina psychrophila	WP_067205112	-DEA---------E-II---S-	N	TDK---LIPKYDE--ND--L--G----
Sporosarcina sp. BI001-red	WP_116018178	--KV---------D------M-	D	AS-FE-V-REYDT--TT--ET----Q-
Sporosarcina sp. D27	WP_025785514	--KS---------D------M-	D	ASH-E-I-REYD----T--ET------
Sporosarcina sp. EUR3 2.2.2	WP_024535445	-DEQH--------D-I----G-	N	S-K-QTL--NYDK-L-E--A-----Q-
Sporosarcina sp. HY008	WP_067407815	-DEE---------E-II---S-	D	P-K-E-I-P-YDQ--NA--L--G--Q-
Sporosarcina sp. P18a	WP_099675724	-NES---------E--------	N	EDQ-KDL-P-YDEI-KE--A--DT-QR
Sporosarcina sp. P19	WP_099691334	-NES-G-------E-I------	N	E-K-T-L-P-YDE--KE--A--D--QR
Sporosarcina sp. ZBG7A	WP_039044332	--KS---------D------M-	D	AS--E-I-RDYD----T--ET------
Streptococcus pneumoniae	CKH13590	-DEEHG-------A------G-	D	EQK---L-NEYDE--NA--KE----K-
Ureibacillus thermophilus	QBK27071	-TK---T------E------E-	D	A-K---L-DKYD-I-----A-----Q-
Ureibacillus thermosphaericus	WP_016837656	-TKV--T------E------E-	N	V-K---L-DEYD-I--K--A-----Q-
Viridibacillus arvi	WP_053418042	-TK---T------Q------N-	D	VDQ--TL-DEYD----D--A--G----
Viridibacillus sp. FSL H7-0596	WP_083671635	-TK---T------Q-I----N-	D	VDR--TL-DEYD----D--A--G----
Viridibacillus sp. OK051	PKA85572	-TKC--T------Q------N-	D	V-Q--TL-DEYD----A--TN-G----

Supplemental Figure 28

A partial sequence alignment of the FMN reductase (NADPH) protein containing a one amino acid deletion (boxed) that is exclusively shared by all members belonging to the Jejuensis clade and absent in all other bacteria.

Group	Organism	Accession	57		101
**Jejuensis Clade (3/3)**	Lysinibacillus saudimassiliensis	CEA00739	LFVGKARKLRQRIKKHFNDNGLMVK		HRDEIHKIEVFEVETPMERE
	Lysinibacillus jejuensis	WP_108306330	-------------------------		--------------------
	Lysinibacillus sp. BF-4	WP_036144869	-------------------------		-----------------D--
**Other Bacteria (0/>100)**	Bacillus acanthi	WP_108670990	-----------------E--VSPIR	N	----VY----CV--D-T---
	Bacillus acidicola	WP_066266914	------------VQ---E--VSPI-	G	--------C--Y--D-A---
	Bacillus aciditolerans	WP_121445964	-----------------E-AVSPI-	T	----V---DICI--D-----
	Bacillus akibai JCM 9157	GAE34051	-----------------E-TVSPI-	M	--N-VT--D-CV--G-VH--
	Bacillus albus	WP_130068832	--------I--------E--VSPI-	N	----VYR-DACI--D-T---
	Bacillus alkalitelluris	WP_078544922	-----------------E--VSPI-	N	--N-VYR-D-CV--D-----
	Bacillus amyloliquefaciens	WP_101669755	-----------------E--VSP--	H	----VY----CV-ND-----
	Bacillus anthracis	KOS27676	--------I--------E--VSPI-	N	----VYR-DASI--D-T---
	Bacillus asahii	WP_127761862	--I-----------R--E-QVSPM-	H	--K-VYQ-D-CV--S-----
	Bacillus atrophaeus	WP_061669309	-----------------E--VSPI-	Q	----VY----CL--D--D--
	Bacillus badius	WP_063384199	M---------M--R-S-E-AASPI-	L	---DVY----CY--D-----
	Bacillus bataviensis	WP_007086758	----------P-V-R--E-TVSPI-	A	----VN--A-IV-DE-----
	Bacillus bingmayongensis	WP_017149435	--------I--------E--VSPI-	A	----VYR-D-CI--D-T---
	Bacillus camelliae	WP_101354699	-Y----------VT---E--VSPI-	N	----V---A--Y--D-T---
	Bacillus campisalis	WP_046521722	-----------------E-TVSPI-	M	----VTR---CVIDD-VH--
	Bacillus cavernae	WP_126865873	------------V----E--VSPI-	N	----V---VISFE-D--Y--
	Bacillus cecembensis	WP_057983291	--------I--------E-TVSPM-	N	--T-V-----Y-I-D-----
	Bacillus cereus	WP_025709425	--------I--------E--VSPI-	N	-----YR-DACI--D-T---
	Bacillus cihuensis	WP_034302406	-----------------E-SVSPI-	L	--N-VSS--MCY--D-----
	Bacillus cohnii	WP_066411248	-----------------E-TVSPI-	D	----V-R-DICL--D----D
	Bacillus cucumis	WP_101646913	----------P-V-R--E-TVSPI-	A	---D-Y--A-LV-DS-----
	Bacillus cytotoxicus	WP_012094330	--------I--------E--VSPM-	A	----VYR-D-CL--D-----
	Bacillus dakarensis	WP_077215180	-----------------E--VSPI-	M	----VT--A-CL--D-VH--
	Bacillus deserti	WP_101645050	-----------------E-TVSPI-	Q	----VYT-DICY--E-VD--
	Bacillus dielmoensis	WP_042464148	-----------------E--VSPM-	M	----VAR-D-CI-DD-AH--
	Bacillus drentensis	WP_066253712	----------P-V----E-TVSPI-	L	----VYR-A-CV--D-V---
	Bacillus fastidiosus	WP_066230664	-----------------E--VSPI-	N	----VYR-D-CV--D-TD--
	Bacillus firmus	WP_035329553	------------V----E--VSPI-	N	---DV---A-IY--DS----
	Bacillus foraminis	WP_121613095	------------V----E-QVSPI-	L	----VY---IAL--E-----
	Bacillus gaemokensis	WP_033675580	--------I--------E--VSPI-	E	----VYR-D-CV--D-T---
	Bacillus gottheilii	WP_066444909	M-----------V----E--VSPI-	K	Y--DVY--AIIL--DA----
	Bacillus halmapalus	WP_078380476	------------V----E--VSPI-	N	----VYR-D-CI--D-----
	Bacillus halosaccharovorans	WP_078434030	------------V---LE-TVSPL-	N	----VFR-D-SI--D-----
	Bacillus halotolerans	WP_105953840	-----------------E-TVSPI-	N	----VY----CV--D--D--
	Bacillus horikoshii	WP_064100940	-Y---------------E--VSPI-	H	----VYR-D-CI--D-----
	Bacillus horneckiae	WP_066394727	----------P-V----E-TVSPIL	K	----VT--A-CLI-D--D--
	Bacillus humi	WP_058000194	-Y--------P-L----E-TVSPI-	M	----VY--A-LV--D-V--D
	Bacillus indicus	WP_029281009	-----------------E-QVSPI-	N	----V-E-A-CI--S-----
	Bacillus intestinalis	WP_088679747	-----------------E-TVSPI-	H	----VY----CV--D-----
	Bacillus kribbensis	WP_026694873	-----------------E--VSPL-	N	----VYS---CI--E--D--
	Bacillus litoralis	WP_066340743	------------V---LE-TVSPI-	N	--N-VF--D-CV--D----D
	Bacillus loiseleuriae	WP_049682978	-----------------E-SVSPI-	L	--N-VSR--ICY--D-----
	Bacillus luti	WP_071712080	--------I--------E--VSPI-	N	----VYR-DACI--D-T---
	Bacillus manliponensis	WP_084158013	-----------------Q--TSPI-	D	----VYR-D-CV--DA-H--
	Bacillus marisflavi	WP_121618603	------------V---LE-TVSPL-	N	----VYR-D-CL--D----D
	Bacillus massiliglaciei	WP_110928684	-----------------E--VSPI-	N	--G-VNR--IAY--E-V---
	Bacillus massilioanorexius	WP_019241643	----------------LE--VSPM-	K	Y----YS-D-CI-DD-----
	Bacillus massiliogabonensis	WP_102275115	----------P-V----E-TVSPL-	K	Y--D-Y--A-CL--D-----
	Bacillus massiliogorillae	WP_042347988	-----------------E--VSPM-	K	----VYT---CY--DS----
	Bacillus massilionigeriensis	WP_075983332	-----------------E-TVSPI-	M	----VAR-D-CV--D-VQ--
	Bacillus mediterraneensis	WP_071461557	-----------------E-TVSPI-	N	----VT--D-CV--D-VD--
	Bacillus mesonae	WP_066392190	-----------------E--VSPM-	E	----VT--A-CI--E-VH--
	Bacillus mobilis	WP_120450272	--------I--------E--VSPI-	N	----VYR-DACI--D-T---
	Bacillus mojavensis subgroup	WP_010331777	-----------------E-TVSPI-	N	----VY----CV--D-----
	Bacillus muralis	WP_057912204	I---------P-V----E--VSPI-	E	--N-VK--DILI--D-----
	Bacillus mycoides	WP_002069202	-----V--V--------E--VSPI-	N	----VYR-D-CI--N-----
	Bacillus nakamurai	WP_061527044	-----------------E-TVSP--	N	----VY--Q-CV-ND----D
	Bacillus ndiopicus	WP_042472975	--------I--------E--VSPM-	K	Y----F----Y-I-D-----
	Bacillus niameyensis	WP_062105582	-Y--------P-V----E-TASAI-	K	----VY-VA-SI-DD-----
	Bacillus nitratireducens	WP_071734493	--------I--------E--VSPI-	N	----VYR-DACI--D-T---
	Bacillus notoginsengisoli	WP_118921870	M---------P-V-R--E-TVSPI-	N	----VY--A-LV--E--D--
	Bacillus oceanisediminis	WP_110065251	------------V----E--VSPI-	N	----VY--A-IY--DS----
	Bacillus onubensis	WP_099363610	-----------------E-TVSPI-	M	----VY--DICI--D----D
	Bacillus panaciterrae	WP_028401545	-----------------E-TASPI-	M	Y---VY--DICL--D-T--D
	Bacillus persicus	WP_090748910	------------V----E-TVSPI-	N	----V----ICV--D-TD--
	Bacillus praedii	WP_057762345	----------P-V----E-TVSPM-	K	Y---VY--A-CL--D-----
	Bacillus pseudofirmus	WP_012958795	-----------------E-TVSPI-	V	----VA--D-CII-D-VH--
	Bacillus pseudomycoides	WP_098927955	--------I--------E--VSPIQ	A	----VYR-DACI--D-----
	Bacillus psychrosaccharolyticu	WP_040374316	------------V----E--VSPI-	L	--KDVT---ILV--D-----
	Bacillus salsus	WP_090849315	-----------------E--VSPM-	K	Y---VYR-DISY--D--D--
	Bacillus siamensis	WP_047477650	-----------------E--VSP--	H	----VY----CV-ND-----

Other Bacteria (0/>100)

Bacillus simplex	WP_061143923	I---------P-V----E--VSPI-	D	--N-VK--DILI--D-----
Bacillus sinesaloumensis	WP_077616980	-----------------E-TVSPI-	N	---QV---DICI--D-----
Bacillus soli	WP_066064561	----------P-V-R--E-TVSPI-	T	----VY--ALCVI-D-----
Bacillus sporothermodurans	WP_066229550	------------VT---D--VSAI-	K	----VQ--A-IY--D-----
Bacillus subterraneus	WP_125481113	M---------P-V----E--VSPI-	N	--H-VY--A-LT--D--D-D
Bacillus subtilis	WP_015483665	-----------------E-TVSPI-	H	----VY----CV-DD-----
Bacillus tequilensis	WP_024713617	-----------------E--VSPI-	H	----VY----CV-DD-----
Bacillus testis	WP_050615086	-----------------E-SVSPM-	N	----VYT-D-CI-ND----D
Bacillus thuringiensis	AJG75859	--------I--------E--VSPI-	N	----VYR-DACV--D-T---
Bacillus timonensis	WP_042341379	-----------------E-TVSPI-	M	----V---DICI--D----D
Bacillus toyonensis	WP_098954466	--------I--------E--VSPI-	N	----VYR-D-CI--D-T---
Bacillus tuaregi	WP_071394037	-----------------E--VSPI-	T	----VYR-DICL-DD-----
Bacillus vallismortis	WP_121643238	-----------------E--VSSI-	H	----VY----CV-DD----D
Bacillus wudalianchiensis	WP_065410596	M---------M--R-S-E-AASPI-	L	Y--DVY----CY--D-----
Butyricicoccus sp. 1XD8-22	RKJ49322	-----------------E--VSPI-	N	--E-VY----Y---D-----
Candidatus Parcubacteria bacte	RJR13826	------------V---LE--VSPI-	N	----VYR-D-CI--DA----
Caryophanon latum	WP_066464107	--------V------Y-DG-VAPLN	N	-KH-V-----Y-I-D-----
Caryophanon tenue	WP_066545024	--------V------F-DG-IAP-N	N	-KH-------Y-I-D-----
Cohnella sp. 18JY8-7	WP_123042545	----------P------E-SVSPI-	K	----VT----CI--D-VH--
Cohnella sp. K2E09-144	WP_119149846	----------P------E-SVSVI-	A	----VA----CVI-N-VY--
Cohnella sp. M2MS4P-1	WP_120978313	----------P------E-SVSPI-	E	----VA----CV--D-VH--
Domibacillus antri	WP_075398957	----------M--R---E--VSDI-	N	---DV---V-CI-DS----D
Domibacillus enclensis	WP_045851616	M---------M--R---E-TVSDI-	N	---DV---V-LV-DS----D
Domibacillus tundrae	WP_046179784	----------M--R---E--VSDI-	N	---DV---V-LV-DD----D
Filibacter sp. TB-66	WP_124071719	--A---------V----E--VSPI-	E	--N-VKE-S-III-D-----
Jeotgalibacillus campisalis	WP_041057013	------------V----E-SVSP--	N	----VYR-D-SI-DH--D-D
Jeotgalibacillus proteolyticus	WP_104056440	------------V---LQ--VSP--	E	-----YR-D-SI--DS----
Jeotgalibacillus soli	WP_041088565	-----------------E-TVSPI-	N	Y---VYR-D-SI--E--D--
Jeotgalibacillus sp. S-D1	WP_133376352	--------I---V----E--VSPM-	N	----VYR-D-SI--D-T---
Kurthia huakuii	WP_029499623	--------I---V----E--VSPM-	N	----VF----Y-I-D-----
Kurthia massiliensis	WP_010288986	--------I--------E--VSP--	D	--N--F----Y-I-DQ----
Kurthia senegalensis	WP_010305257	--------I--------E-TVSPM-	D	----VF----Y-I-DQ----
Kurthia sibirica	WP_109305499	----------P-L----S-TVSPI-	D	--K-VA-----VI-DA----
Kurthia sp. 3B1D	WP_126991813	--------I---V----E--VSP--	D	--N--F----Y-I-D-----
Kurthia zopfii	WP_109348981	----------P-L----Q-QVSPL-	D	----VA---A-VI-DA----
Lysinibacillus acetophenoni	WP_097148769	-----------------E--VSPI-	K	Y---VY----Y---D-----
Lysinibacillus chungkukjangi	WP_107933460	--------I--------E-TVSPM-	N	--N--Y----Y-IDDA----
Lysinibacillus composti	WP_124762711	--------I--------E--VSPM-	T	-----Y----Y-I-D-----
Lysinibacillus contaminans	WP_053583331	--------I--------E--VSPLQ	N	-----F----Y-I-D-----
Lysinibacillus endophyticus	WP_121214393	--------I--------E--VSPM-	N	-----Y----Y-I-D-----
Lysinibacillus halotolerans	WP_122971230	--------I--------E--VSPM-	N	-----Y----Y-I-D-----
Lysinibacillus macroides	WP_053995695	--------I--------E--VSP--	N	-----F----Y-I-D----D
Lysinibacillus manganicus	WP_036185444	-----------------E--VSPI-	K	Y---VY----Y---D-----
Lysinibacillus massiliensis	WP_036177107	-----------------E--VSPI-	N	--E-VY----Y---D-----
Lysinibacillus meyeri	WP_107837982	--------I--------E--VSPM-	K	Y----F----Y-I-DA----
Lysinibacillus odysseyi	WP_036159210	--------I--------E--VSPM-	N	-----F----Y-I-D-----
Lysinibacillus parviboronicapi	WP_107924328	-Y------I--------E--VSPM-	K	Y----F----Y-I-D-----
Lysinibacillus sinduriensis	WP_036201458	--------I--------E--VSP-R	K	Y----Y----Y---DA----
Lysinibacillus sp. 2017	WP_108712764	--------I--------E-TVSPM-	N	----------Y---D-----
Lysinibacillus sp. B2A1	AVK84983	--------I--------E--VSPM-	N	--Q--F----Y-I-D-----
Lysinibacillus sp. BK089	WP_132360684	--------I--------E--VSPM-	N	----VF----Y---D-----
Lysinibacillus sp. FJAT-14222	WP_053593439	--------I--------E--VSPL-	N	-----F----Y---D-----
Lysinibacillus sp. Marseille-P	WP_106778997	-----------------E-QVSPI-	N	----VY----Y---D-----
Lysinibacillus sp. SYSU K30002	WP_126659002	--------I--------E--VSPM-	N	-----Y----Y---DA----
Lysinibacillus sp. YLB-03	WP_118875268	--------I--------E--VSPM-	N	-----Y----Y---DA----
Lysinibacillus sp. ZYM-1	WP_054611790	--------I---V----E--VSPMR	N	----VF----Y---D-----
Lysinibacillus sphaericus	WP_010859383	-Y------I--------E--VSPM-	K	Y----F----Y-I-D-----
Lysinibacillus telephonicus	WP_126294845	--------I--------E--VSPM-	N	-----Y----Y-I-D-----
Lysinibacillus xylanilyticus	WP_049665153	--------I--------E--VSPM-	N	----VF----Y---D-----
Lysinibacillus xyleni	WP_097073198	--------I--------E--VSPM-	N	-----Y----Y---DA----
Mycobacteroides abscessus subs	SHQ79240	M---------P-V----E-QVSPI-	K	----VY--A-IT--DA----
Oceanobacillus bengalensis	WP_121134728	------------V----E--VSPI-	M	----VN--DICL--D-----
Oceanobacillus rekensis	WP_087974797	----------P------Q--VSPM-	T	----VT---ICL--E-AD--
Oceanobacillus sp. Castelsardo	WP_068672163	-----------------E--VSPI-	N	----VT--DICL--D-----
Ornithinibacillus californiens	WP_047984309	-----------------E-TVSPI-	M	----VYR-DICL--D-----
Paenibacillus agaridevorans	WP_108995141	-----------------E-TVSVM-	N	--E-VT------N-S-L---
Paenibacillus amylolyticus	WP_123063941	----------P------E-TVSPI-	N	--E-VT----CL--D--D--
Paenibacillus borealis	WP_076110926	----------P------E--VSVM-	P	--G-VT----CL--D-VD--
Paenibacillus cellulosilyticus	WP_110042374	----------P------E-TVSLM-	N	--E-VAT---YI--D-V---
Paenibacillus elgii	WP_029191906	----------P------E-TVSIM-	N	----VA----CI--D-VH--
Paenibacillus herberti	WP_089524061	----------P------E-SVSPM-	K	----VA----YL-DD----D
Paenibacillus ihuae	WP_054942469	----------P------E-TVSVM-	G	--E-VT----CI--D-VD--
Paenibacillus koleovorans	WP_127580416	----------P------E-TVSA--	D	--G--TR---CL--D-V---
Paenibacillus mucilaginosus	WP_013921550	-----------------E-TVSPI-	N	--N-VT----YI--D-V---

Other Bacteria (0/>100)

Paenibacillus paeoniae	WP_116048304	----------P------E-SVSIM-	N	----VT----CI--D-VH--
Paenibacillus pasadenensis	WP_028600151	----------P------E-ASSVM-	N	--S-VA----CLI-E--H--
Paenibacillus pinisoli	WP_120106193	----------P------E-TVSVM-	N	----VV----CV--D-VD--
Paenibacillus polysaccharolyti	WP_090921395	----------P------E-TVSPM-	N	----VT----CL--D--D--
Paenibacillus selenitireducens	WP_078498139	----------P------E-TVSV--	N	-----T-----VN-DAV---
Paenibacillus sp. 7197	WP_124697380	----------P------E--VSPI-	D	--N-VA----CV--D-V---
Paenibacillus sp. HW567	WP_019914748	----------P------E-TVSVM-	P	----VT----CI-DD-VD--
Paenibacillus sp. MY03	WP_087572060	-----------------E-AVSVM-	N	--E-VT------N-S-L---
Paenibacillus sp. P46E	WP_074110246	----------P------E--VSVM-	P	----VT----CL--EAVD--
Paenibacillus sp. Root52	WP_056703262	----------P------E-TVSPI-	N	--E-VT----CL--D--D--
Paenibacillus sp. SMB1	WP_111147218	-----------------E-TVSPM-	N	----VV----AI-DD-VD--
Paenibacillus taichungensis	WP_094937360	----------P------E-TVSPM-	A	----VTT---CVI-D-VD--
Paenibacillus taiwanensis	WP_028543692	----------P------E-SVSV--	N	--N--A-----VN-DAV---
Paenibacillus typhae	WP_090715649	----------P------E--VSVM-	P	----VT----CI--D-VD--
Paenibacillus wulumuqiensis	WP_046216312	----------P------E-TVSPI-	D	----VTR-D-CI--D-T---
Paenisporosarcina antarctica	WP_134210832	--------V---V----E--VSPL-	E	----VT--KI-Y-DDA----
Paenisporosarcina indica	WP_075618640	--------V---V----E--VSPL-	D	----VT--KICY--DA----
Paenisporosarcina quisquiliaru	WP_090566171	--------I--------E--VSPMI	K	----VY--S-CI--EA-D--
Paenisporosarcina sp. HGH0030	WP_016428469	--------V---V----E--VSPL-	E	----VA--KI-Y-DDA----
Paucisalibacillus sp. EB02	WP_042147211	-----------------E-TVSPI-	M	----VY--DICL--D-----
Planococcus donghaensis	WP_008428720	------------V----E-SVSPL-	N	--E-VYR-T-SF--D-----
Planococcus halocryophilus	WP_008496293	------------V----E-SVSPL-	N	--E-VYR-T-SF--D-----
Planococcus halotolerans	WP_112224258	------------V----E--VSPL-	N	----V-R-T-SI--D-----
Planococcus maitriensis	WP_112232815	------------V----E--VSPL-	N	----V-R-A-SI--D-----
Planococcus maritimus	WP_068459970	------------V----E--VSPL-	N	----V-R-A-SI--D-----
Planococcus salinarum	TAA72059	------------V----E--VSPL-	N	----V-H-TISI--D-----
Planococcus salinus	WP_123166035	------------V----E--VSPL-	N	--E-V-R-T-SI-DD-----
Planococcus sp. CAU13	WP_033542490	------------V----E--VSPL-	N	----V-R-TISI--D----D
Planococcus sp. PAMC 21323	WP_038702153	------------V----E-SVSPL-	N	----VYR-T-SF--D-----
Planococcus versutus	WP_049693513	------------V----E-SVSPL-	N	--E-VYR-A-SF--D----D
Planomicrobium flavidum	WP_088006697	------------V---LQ--VSPLR	T	--E---R-D-TF--DA---D
Planomicrobium glaciei	WP_036802782	------------V----E--VSPL-	N	--E-VYR-T-SI--D-----
Planomicrobium soli	WP_106533093	------------V---LE-TVSPL-	N	--E---R-S-TI--D----D
Planomicrobium sp. Y74	WP_121635018	------------V----E--VSPL-	N	----V-R-T-SI--D-----
Psychrobacillus insolitus	WP_111439956	--------I--------E--VSPMI	K	----VY--S--I--EA-D--
Psychrobacillus psychrodurans	WP_093495671	--------I--------E--VSPMI	K	----VY--S-CI--EA-D--
Psychrobacillus psychrotoleran	WP_093537476	--------I--------E--VSPMI	K	----VY--S-CI--EA-D--
Psychrobacillus sp. OK028	WP_093061661	--------I--------E--VSPMI	K	----VY--S-CI--EA-D--
Psychrobacillus sp. OK032	WP_093272223	--------I--------E--VSPMI	K	----VY--S-CI--EA-D--
Quasibacillus thermotolerans	WP_039238603	-----------------E--VSPI-	T	----VT--Q-CV-DD-VH--
Rhizophagus irregularis	PKC52305	-----------------E--VSPI-	N	--E-VY----Y---D-----
Rummeliibacillus pycnus	WP_102691549	--------M-P-L----E--VSPL-	N	----V----A-I-DD-----
Rummeliibacillus sp. POC4	WP_119417279	--------M---V----E--VSPL-	D	----V----AYV-QD-----
Rummeliibacillus sp. TYF005	WP_124218582	--------M---V----E--VSPL-	D	----V----AYV-QD-----
Rummeliibacillus stabekisii	WP_066786822	--------M-P-L----E--VSPL-	D	----V----A-IIDD-----
Saccharibacillus kuerlensis	WP_018977977	----------P------E--VSPM-	N	----VRR-D-CL-AD-T---
Salipaludibacillus sp. KQ-12	WP_110608631	-----------------D--VSVI-	N	--K-VT----CIIDD-----
Solibacillus isronensis	WP_079526647	--------I--------E--VSPM-	N	--A-------Y-I-D-----
Solibacillus sp. R5-41	WP_099423859	--------I--------E-TVSPM-	N	----V-----Y-I-D-----
Sporosarcina globispora	WP_053435047	------------V----E--VSPI-	N	---DVY--A-IY--DS----
Sporosarcina koreensis	WP_082023379	----------P-V---LE--VSPL-	P	----VN--G-IL--D-----
Sporosarcina psychrophila	WP_067207386	--A---------V----E-TVSPI-	N	--N-V-E-AIIV--D-----
Sporosarcina sp. BI001-red	WP_116019053	----------P-V---LE--VSPL-	P	----VN--A-IS--DA----
Sporosarcina sp. EUR3 2.2.2	WP_024535764	--------V---V----E--VSPI-	D	----VT--KI-Y-DDA----
Streptococcus pneumoniae	CRG03572	--------I--------E--VSPI-	N	----VYR-DACI--D-T---
Virgibacillus salinus	SDQ07316	----------P---------VSPI-	L	----VS----CI-RD-AH--
Viridibacillus arenosi	WP_038184681	--------I---V----E--VSPI-	D	--N-V------I--DQ----
Viridibacillus sp. OK051	WP_100797807	--------I---V----E-SVSPI-	D	--K-VY-----V--DQ----

Supplemental Figure 29

A partial sequence alignment of the UvrABC system protein C protein containing a one amino acid deletion (boxed) that is exclusively shared by all members belonging to the Jejuensis clade and absent in all other bacteria.

Group	Species	Accession	298		344
**Jejuensis Clade (3/3)**	Lysinibacillus saudimassiliensis	CDZ99298	PALQLLSSSFEETIGRPITTVLGIE	QD	YDYTFENLINMKEPLNLDFS
	Lysinibacillus sp. BF-4	WP_036143833	-------------------------	--	--------------------
	Lysinibacillus jejuensis	WP_108305916	-------------------------	--	------D-L-----------
**Other Bacteria (0/>200)**	Aeribacillus pallidus	WP_063387768	--HEM-NV-R-TAL-KS-VEL-DLT		DEC---D-V-QQ--ML----
	Anoxybacillus ayderensis	WP_085788905	A--NI-NV-R-TVLSA--VS---LD		EQ----M-LEER-T-I----
	Anoxybacillus flavithermus	WP_064221292	A--NI-NV-R-TVLSA--V----LD		EQ----T--EER-T-I----
	Anoxybacillus gonensis	WP_009361069	A--NI-NV-R-TVLSA--VS---LD		EQ----T-LEER-T-I----
	Anoxybacillus kamchatkensis	WP_019416600	A--NI-NV-R-TVLSA--VS---LD		EQ----T-LEER-T-I----
	Anoxybacillus pushchinoensis	WP_091702710	A--NI-NV-R-TV-SA--V----LD		EQ----T--EER-T-I----
	Anoxybacillus sp. 103	WP_077429099	A--NI-NV-R-TVLSA--V----LD		EQ----T--EER-T-I----
	Anoxybacillus suryakundensis	WP_055440859	A--NI-NV-R-TVLSA--V----LD		EQ----T--EER-T-I----
	Anoxybacillus thermarum	WP_043964376	A--NI-NV-R-TVLSA--VS---LD		EQ----T-LEER-T-I----
	Bacillus aquimaris	WP_113969390	--ADM-NV-R-TVVSQ--V-L--LD		EE---DD-S-EQDSII--Y-
	Bacillus asahii	WP_119119017	--AK--NVTR-TVLNQ--IEL--L-		EE---DD-LEQR-SII--Y-
	Bacillus badius	WP_041095647	--AK---V-R-TV-SQ---HL--L-		K----DD-L-EQDSVI--Y-
	Bacillus boroniphilus JCM 2173	GAE47822	--T-M-GV-R-TVLSQ-LVSI--LD		EN-N--D-LQE-DSIV--Y-
	Bacillus butanolivorans	WP_116821251	--AR--NV-H-TVMNQ--IE---L-		EE-K--D-LEER-SII--Y-
	Bacillus campisalis	WP_046524959	--ANM-DV-R-TVLSE--VS---L-		E---L-D-LQEQ-SVI--Y-
	Bacillus cavernae	WP_126867241	--AK--DV-R-TVLSQ--INL--L-		EE-S--D-LEER-SVI--Y-
	Bacillus cecembensis	WP_057986483	---S--HDTR-S-LN---AS---LD		QE-S--D--H---SVS----
	Bacillus cohnii	WP_066413574	---KM-NVPR-TVLAK--VE-----		E-H---T-MSEQDS-I----
	Bacillus firmus	WP_061793144	--AKM-NV-R-TVLSS--VSL--L-		E-----E-LDEQDSVI--Y-
	Bacillus flexus	WP_061785683	--ASM-NV-R-TVLSE--V-L--LD		ETH---S-LANQDS-I----
	Bacillus foraminis	WP_121610440	--AKI-DV-R-TVLSD--VS-----		DE----D-LEQQ-SVI--Y-
	Bacillus funiculus	WP_129728641	--EK--NV-R-TALDQ---KI----		EE--LD--YEEPDSVI--Y-
	Bacillus horikoshii	WP_088020036	---GM-NV-R-TVLNK--VE-----		ETHS-DQ-VSEQ-S-I----
	Bacillus horneckiae	WP_066398816	--AEM-NV-R-TVLTL--VSL--L-		E----DE-LAEQDSVI--Y-
	Bacillus indicus	WP_029282966	--IEM-NV-R-TV-DV---K----D		GE---DS--DQQDS-I--A-
	Bacillus jeotgali	WP_079505993	--T-M-GV-R-TILSQ-LVS---LD		EN-S--D-LQE-DSIV--Y-
	Bacillus kochii	WP_095371357	--AEM-HV-R-TVLSNS-VSL--L-		E-----E-LEER-SVI--Y-
	Bacillus korlensis	WP_066059574	--AK---V-R-TILSSS-VSL--L-		E--S--D-LVE--S-I--Y-
	Bacillus kwashiorkori	WP_062351055	A-EKM-NVTR-TV-SM--V-L--L-		EK---D--LEQ-DSII--Y-
	Bacillus loiseleuriae	WP_049683479	--AK--DV-R-TVLNQ--IEL-DL-		D-----D-LEERDSVI--Y-
	Bacillus marisflavi	WP_121617848	--AKM-NV-R-TVLSQ--VSL--LD		EN----D-SKEQDSII--Y-
	Bacillus massiliglaciei	WP_110928063	--AK--NV-R-TVLNQ--IQ---L-		DE-S-DD-LEER-SVI--Y-
	Bacillus massilioanorexius	WP_019243369	--A---DV-R-TVLSTS-VSL--L-		KE----D-LALQDSIT----
	Bacillus massiliogabonensis	WP_102273372	--AEM-NV-R-TVLSS--VPL--L-		E----DE-LAERDSVI--Y-
	Bacillus massiliogorillae	WP_042352931	--A---NV-R-TVLSM--VSL--L-		S--S--D-LSQQDS-I----
	Bacillus mediterraneensis	WP_071461045	--AEM-NV-R-TVLSQQ-VS---L-		E--SL-D-LQE--SII--YT
	Bacillus mesonae	WP_066389886	--VEM-DV-R-TILSQ--EQ---L-		DSN---D-LEE-DS-I--Y-
	Bacillus muralis	WP_057914121	--A---NI-R-TVMNQ--IE---L-		EE-K-DD-LEER-SVI--Y-
	Bacillus ndiopicus	WP_042478424	---N--HVTR-AMMN---VS---LD		QN-S--D--H-------N--
	Bacillus oceanisediminis	WP_110063597	--AKM-NV-R-TVLSSS-DSL--L-		EE----E-L-ERDSVI--Y-
	Bacillus oleivorans	WP_097156586	S-IG--NV-R-TALSQQ---L----		DK----D---EQ-SVI--Y-
	Bacillus persicus	WP_090748392	--AKM-DV-R-TVLSNS-VS---V-		D--S-DD-LLEQ-SVI----
	Bacillus praedii	WP_057759564	--AEM-NV-R-TVLSS--V-L--L-		D----DE-LAERDSVI--Y-
	Bacillus psychrosaccharolyticu	WP_040374700	--AR--DV-R-TVL-Q--IEL--L-		EE----D-LEER-SVI--Y-
	Bacillus rubiinfantis	WP_042354556	--AEM-NV-R-TILSL--E----L-		NSI--DD-LEE-DS-I--Y-
	Bacillus selenatarsenatis	WP_041965135	----M-GVPR-TVLSQ-LVS---LD		EN---DD-LQE-DSIV--Y-
	Bacillus shackletonii	WP_055741610	A-TEMIDV-R-TILSQN-V-A----		R-HS-ND-L-E--SII----
	Bacillus simplex	WP_034316130	--A---NV-S-TVMNQ--IE---L-		EE-K--D-LEER-SII--Y-
	Bacillus smithii	WP_048624322	--TEM-NV-R-TVMSES-V-L-HL-		GQ-E--D-L---DSI-----
	Bacillus sp. 17376	WP_023613396	--T-M-GV-R-TVLSQ-LVSI--LD		EN-N--D-LQE-DSIV--Y-
	Bacillus sp. 1NLA3E	WP_015596207	--VKM--V-R-TVLSQS-VSL----		DK-G--D-LEEQDSVI--Y-
	Bacillus sp. 72	WP_034287765	--SKM-NV-R-TI-SQ--VSL-NL-		E--S-DE-L-EQDSVI--Y-
	Bacillus subterraneus	WP_044392014	--A-M-GV-R-TVLSQ-LVSI--LD		EN-S--D-LQE--SII--Y-
	Bacillus subterraneus	WP_125482069	--E-M-GV-R-TILSQ-LVS---LD		EN----D-LQEQ-SIV--Y-
	Bacillus subtilis	WP_014115845	---E--NV-R-TALEM---SL---Q		EN----D-VEQQDSML-EIK
	Bacillus terrae	WP_120115831	-S-M-NV-R-TM-SQ-V-EL-LLD		DE-S--D-LDQQ--IV--Y-
	Bacillus testis	WP_050613499	--A---NV-R-TVLSM--VSL----		D-----D-LDQQDS------
	Bacillus thermocopriae	WP_128999536	A--NI-NV-R-TVLSA--V----LD		EQ----T--EER-T-I----
	Bacillus vietnamensis	WP_034764813	--ADM-DV-R-TVVSQ--VSL--LD		EE---DD-S-EQDSII--Y-
	Bacillus vietnamensis	WP_060674574	--ADM-DV-R-TVVSQ--VSL--LD		EE---DD-S-AQDSII--Y-
	Bacillus wudalianchiensis	WP_065409483	--SK--NV-R-TV-SQH--NL--L-		KE---DD-L-EQDSVI--Y-
	Bacillus zeae	WP_119112117	--A-M-DV-R-TILSA--VS---L-		E---L-D-LQE--SVI--Y-
	Brochothrix thermosphacta	WP_120481709	--ADM-KINQ-DAMS---AE--K--		S-H-Y-T-LEE-DSVTI-L-
	Butyricicoccus sp. 1XD8-22	RKJ59671	---NF-NT-R-A-LN---AS---L-		Q--S--D-L---DSV-----
	Caryophanon latum	WP_066466489	------QLERN--LN---IS---LD		Q--D--D--H--DSV--N--
	Caryophanon tenue	WP_066542722	------QFERT--LN---IS---LD		Q--E--D--H--DSV--N--
	Chryseomicrobium excrementi	WP_100353864	---E--NVNRDSVLN----S--T-D		D-A---D--HQQ--MS--Y-
	Domibacillus aminovorans	WP_063966537	--SKM-NV-R-TI-SQ--VSL-NL-		E--S-DE-L-EQDSVI--Y-
	Domibacillus antri	WP_075398629	--SKM-NV-R-TV-SQ--VSL-NL-		E--S-DD-L-EQDSVI--Y-
	Domibacillus enclensis	WP_045851231	--SKM--V-R-TV-SQ--VSL-NL-		E--S-DD-L-EQDSVI--Y-
	Domibacillus epiphyticus	WP_076766562	--SKM-NV-R-TV-SQ--V-L-NL-		E--S-DD-L-EQDSVI--Y-

Other Bacteria (0/>200)

Domibacillus indicus	WP_046176399	--SKM--V-R-TV-SQ--ISL-NL-	E--S-DD-L-EQDSVI--Y-
Domibacillus iocasae	WP_069937051	--SKM-NV-R-TV-SQ--VSL-NL-	E--S-DD-L-EQDSVI--Y-
Domibacillus mangrovi	WP_073710582	--SKM-NV-R-TI-SQ--VSL-NL-	E--S-DD-L-EQDSV---Y-
Domibacillus robiginosus	WP_050180244	--SKM-NV-R-TV-SQ--VSL-NL-	E--S-DD-L-EQ-SVI--Y-
Domibacillus tundrae	WP_046180624	--SKM-NV-R-TV-SQ--VSL-NL-	E--S-DD-L-EQDSVI--Y-
Falsibacillus pallidus	WP_114746421	--SK--DV-R-TVLAQ--VS---L-	EE---DD-LVEQNSVI--Y-
Falsibacillus sp. GY 10110	WP_121681505	--AK--NV-R-TVLSQ--ISL--L-	E----DD-LVEQNSVI----
Filibacter sp. TB-66	WP_124069120	A--AM-RMTRDVVLN---SSI--L-	QE-S--E--QI--S-A----
Jeotgalibacillus campisalis	WP_041054293	--AG--NV-R-TVLSQS--DL--V-	EEF--DD-L-Q-DSVI-N--
Jeotgalibacillus proteolyticus	WP_104059201	--AGM-NV-R-TVLSQS--E-----	GE--L-D-L-QQDS-I-N-G
Jeotgalibacillus soli	WP_041086316	--AGM-NV-R-TVLSQ----L--V-	EE-S--E-LEQQ-SVI----
Jeotgalibacillus sp. S-D1	WP_133377414	--AGM-NV-R-TVLSQS-SDL--V-	DE-S--D-LSQQDS-I----
Kurthia huakuii	WP_029500685	--QK--K-EQN-LM--SL-A---LD	EE----D--H-Q-SV-----
Kurthia massiliensis	WP_010290544	--QK--N-EQN-LM--SL-----LD	EQ----D--H-Q-SV-----
Kurthia senegalensis	WP_010308256	--QN--G-EQS-MM--SL-----VD	DE----D--H---SI-----
Kurthia sibirica	WP_109305768	--QK--QGEKN-LM--S--E---VD	DE----D--H-QDSI-----
Kurthia sp. 3B1D	WP_126989949	--QK--K-EQN-LM--SL-A---LD	EE----D--H-Q-SI--N--
Kurthia zopfii	WP_109350768	--QK--A-ENN-LM--S--E--SVD	DE----D--H-QDSI-----
Listeria cornellensis	WP_036082166	--EKM-KMAH-SAN-K--ID---VS	E--S-DD-MELE---TM-R-
Listeria fleischmannii	WP_007476176	--EKM-RVAH-TAN-K-L-E---VQ	DE-E--D-MD-T---TM-R-
Listeria grandensis	WP_036067507	--EKM-KAAH-SAN-K--ID---VS	EE-S-DD-MELE---TM-R-
Listeria riparia	WP_036102089	--EKM-KVAH-SAN-K--ID--DVA	DE-S-DD-MELD---TM-R-
Listeria weihenstephanensis	WP_036061449	--EKM-KVPH-SAN-K--ID---VS	E--S-DD-MELE---TM-R-
Lysinibacillus acetophenoni	WP_097149364	---SF-KI-RDT-LN---AS---L-	KE-S--D---T--SV---I-
Lysinibacillus boronitolerans	WP_016992311	---E--HI-RDI-L----AS----D	QE-S--D--H-NDAV--N--
Lysinibacillus chungkukjangi	WP_107932063	---NF-DVTR-T-L----AS---LD	EE-N--D--HTQ-SI---Y-
Lysinibacillus composti	WP_124765697	---S--NVLR---LN---AS---LD	KE-S--D-----D-I-----
Lysinibacillus endophyticus	WP_121214581	---NF-NVTR-TMLN----S---VD	QE-S--D--Y--DS---N--
Lysinibacillus fusiformis	WP_025115722	---E--HI-RDI-L----AS----D	QE-S--D--H-SDAI--N--
Lysinibacillus halotolerans	WP_122972070	---KF-NVTR-T-LN---AS---LD	QE-S--D--H---SI---Y-
Lysinibacillus macroides	WP_053997103	---E--HI-RDI-L----AS----D	QE-S--D--H-HDAI--N--
Lysinibacillus manganicus	WP_036183426	--ISF-KI-R-T-LN---AS---L-	KE-S--D---TQDSV-----
Lysinibacillus mangiferihumi	WP_107894911	---E--HI-RDI-L----AS----D	QE-S--D--H-NDAV-----
Lysinibacillus massiliensis	WP_036172247	---NF-NT-R-A-LN---AS---L-	Q--S--D-L---DSV-----
Lysinibacillus meyeri	WP_107841899	---N--HVTR-AMMN---ASA---D	QN-S--D--H-----S-N--
Lysinibacillus odysseyi	WP_036152137	---D--HV-R-M-LN---AS-----	QE-S--D--Y--D-V--N--
Lysinibacillus parviboronicapi	WP_107951468	---E--HI-RDI-L----AS----D	QE-S--D--Y-NDAV--N--
Lysinibacillus sinduriensis	WP_036200933	---NF-NVTR-T-LS---AS---LD	Q--S--D--H---SI---Y-
Lysinibacillus sp. 2017	WP_108714362	---S--HETR-S-LN---ASL--LD	QE-S--D--H--DSV-----
Lysinibacillus sp. A1	WP_039392495	---E--HI-RDI-L----AS----D	QE-S--D--H-SDAI--N--
Lysinibacillus sp. B2A1	AVK86845	---E--HI-RDI-L----AS----D	QE-S--D--H-NDAV--N--
Lysinibacillus sp. BK089	WP_132362525	---E--HI-RDI-L----AS----D	QE-S--D--H-NDAV--N--
Lysinibacillus sp. FJAT-14222	WP_053595926	---E--HI-RDI-L----AS----D	QE-S--D--H-NDAV--N--
Lysinibacillus sp. FJAT-14745	WP_053482922	---E--HI-RDI-L----AS----D	QE-S--D--H-NDAV-----
Lysinibacillus sp. Marseille-P	WP_106782015	---SF-KI-R-A-LN---AA---VN	Q--S--D-L--NDSI-----
Lysinibacillus sp. OL1	WP_131521732	---E--HI-RDI-L----AS----D	QE-S--D--H-NDAV--N--
Lysinibacillus sp. SYSU K30002	WP_126660035	---NF-DVTR-TSLN----S---LD	QE-S--D--Y---SV--N--
Lysinibacillus sp. YLB-03	WP_118876424	--MSF-EVTR-T-L----AS--ELD	QE-S--D--H-Q-SI--NY-
Lysinibacillus sp. YR326	WP_134022143	---E--HI-RDI-L----AS----D	QE-S--D--H-NDAV--N--
Lysinibacillus sp. ZYM-1	WP_054610242	---E--HI-RDI-L----AS----D	QE-S--D--H-NDAV--N--
Lysinibacillus sphaericus	WP_010860712	---E--HI-RDI-L----AS----D	QE-S--D--H-NDAV--N--
Lysinibacillus sphaericus	WP_031415817	------HI-RDI-L----AS----D	QE-S--D--H-SDAI--N--
Lysinibacillus sphaericus	WP_036119369	---E--HI-RDI-L----AS----D	QE-G--D--H-NDAI--N--
Lysinibacillus sphaericus	WP_036162489	------HI-RDI-L----AS----D	QE-S--D--H-SDAI--N--
Lysinibacillus sphaericus	WP_069514076	---E--HI-RDI-L----AS----D	QE-S--D--H-NDAV-----
Lysinibacillus sphaericus C3-4	ACA42202	------HI-RDI-L----AS----D	QE-S--D--H-SDAI--N--
Lysinibacillus telephonicus	WP_126293843	---NF-DVTR-T-L----AS---LD	QE-S--D--H---SI--NY-
Lysinibacillus xylanilyticus	WP_049662631	---E--HI-RDI-L----AS----D	QE-S--D--H-NDAV--N--
Lysinibacillus xyleni	WP_097073308	---NF-NVTR-TMLN---AS---VD	QEFS--D--Y--DS---N--
Mycobacteroides abscessus subs	SHP62808	--AK---V-R-TILSSS-VSL--L-	E-----D-LVE--S-I--Y-
Paenibacillus sp. FSL R5-0490	WP_076259548	--AKM-NV-R-TVLSS--VSL--L-	E-----E-LDE-DSVI--Y-
Paenisporosarcina antarctica	WP_134211637	---R--RVPRDMVLH---SS-----	TQ----D--H---SI-----
Paenisporosarcina indica	WP_075619684	---R--RVPS-MV-H---SS----D	LQFA--D--H--DSI-----
Paenisporosarcina quisquiliaru	WP_090567371	---R--RIPR-MVLN---IS----D	SEHS--D--HL--A----L-
Paenisporosarcina sp. HGH0030	WP_016428910	---R--RV-R-LVLH---SS-----	AE-A--D--H---SI-----
Paenisporosarcina sp. K2R23-3	WP_119882148	---R--RV-R-MVLQ---AS---L-	AN-S--D--H--DF---N--
Paenisporosarcina sp. OV554	WP_108586878	---R--RVPR-LVLH---SSA----	SQ-V--D--H---SI-----
Paenisporosarcina sp. TG-14	WP_017378785	---R--RVPRDMVLH---SS-----	TQ----D--H---SI-----
Planococcus antarcticus	WP_006830572	------NV-R-TV-N--V-SI--L-	EE----D--D---AVT----
Planococcus donghaensis	WP_008427726	------NV-R-TV-N--V-S---L-	EE-------D---SVT--L-
Planococcus faecalis	WP_058383872	------NV-R-TV-N--V-SI--LD	EE----D--D---AIT----
Planococcus halocryophilus	WP_040850809	---H--NV-R-TV-N--V-S---L-	EE----D--D---SVT--L-
Planococcus halotolerans	WP_112222190	---R--NV-R-TVMN--------L-	SE----D--H--DAIT---G
Planococcus kocurii	WP_062429931	------NV-R-TV-N--V-SI--LD	EE----D--D---AIT----

Other Bacteria (0/>200)

Planococcus maitriensis	WP_112233341	------NV-R-TV-N--V-----LD		ES----D--D-RDAIT--I-
Planococcus maritimus	WP_069576998	------NV-R-TV-N--V-----LD		EA----D--D-RDAIT--I-
Planococcus massiliensis	WP_052649580	---R--NV-R-TV-N----S--NL-		SE----D--HI--AIT----
Planococcus plakortidis	WP_068871132	---H--NV-R-TV-N--V-----LD		ES----D--D-RDAIT--I-
Planococcus rifietoensis	WP_058382290	------NV-R-TV-N--V-----LD		ES----D--D-RDAIT--I-
Planococcus salinarum	TAA70628	---R--NV-R-TVMN--------L-		SE----D--H--DAIT---G
Planococcus salinus	WP_123165110	------NV-R-TV-N--V-A---LD		N-----D--H---AIT--L-
Planococcus sp. CAU13	WP_033543227	---R--NV-R-TVMN----S---L-		NE----D--H--DAIT---G
Planococcus sp. PAMC 21323	WP_038705246	------NV-R-TV-N--V-S---L-		EE----D--D--DSVT--L-
Planococcus versutus	WP_049693568	---Y--NV-R-TV-NQ-V-S---LD		EE----D--D---AIT-NL-
Planomicrobium glaciei	WP_036810907	-G-R--NV-R-TV-N----S---LD		SE----D--HL-DAIT----
Planomicrobium okeanokoites	WP_084243452	---R--NV-R-TVMN--------L-		SE----D--H--DAIT---G
Planomicrobium soli	WP_106533349	---R--DV-R-TV-N--L-M---LD		SE----D--HL--TIT----
Planomicrobium sp. MB-3u-38	WP_101800999	---R--NV-R-TVMN--------L-		SE----D--H--DAIT---G
Planomicrobium sp. Y74	WP_121635722	---R--NV-R-TVMN--------L-		SE----D--H--DAIT---G
Psychrobacillus insolitus	WP_111440175	---K--RIPR-MVLN----S--A-D		TANS--D--H--DA-T----
Psychrobacillus psychrodurans	WP_093495561	---R--RIPR-MVLN---IS----D		SEHS--D--HL--A----L-
Psychrobacillus psychrotoleran	WP_093537992	---R--RIPR-MALN---IS----D		PV-S--D--HL--AI---L-
Psychrobacillus sp. FJAT-21963	WP_056831413	---R--RIPR-MVLN---IS----D	A	SEQS--D--YS--SIT----
Psychrobacillus sp. OK028	WP_093062329	---R--RIPR-MVLN---IS----D		PEHS--D--HL---I---L-
Psychrobacillus sp. OK032	WP_093275400	--IR--RIPR-MVLN---IS----D		SAHS--D--HL--SM---L-
Quasibacillus thermotolerans	WP_039238624	--AR--NV-R-TV-SQ---S---L-		EE-S-DD-L-EQDSVI--YG
Rummeliibacillus pycnus	WP_102693451	--QK--NGEQN-LM---LIS---L-		NEH---D--H--DSI-----
Rummeliibacillus stabekisii	WP_066791154	--EK--N-GHN-LM---LVS---L-		KQH---D--H--DAI-----
Solibacillus isronensis	WP_079523570	---E--HD-R-T-LN---AS--RLD		QE-S--D--H--D-V-----
Solibacillus kalamii	WP_087617912	---E--HD-R-T-LN---AS--RLD		QE-S--D--H--D-V-----
Solibacillus silvestris	WP_014822362	---E--HD-R-T-LN---AS--RLD		QE-S--D--H--D-V-----
Solibacillus sp. R5-41	WP_099425678	---S--HDTR-S-LN---AS---LD		QE-S--D--H---SVS----
Sporosarcina globispora	WP_053433315	--AKM-NV-R-TVLSS--VSL--L-		E-----E-L-ERDSVI--Y-
Sporosarcina newyorkensis	WP_009499320	S---M-NLTTDLV-N---ASI----		SE----E--QV--SIP----
Sporosarcina psychrophila	WP_067204143	S--LM-RLTRDLVLN---SSI--F-		QE-A--D--QV--SIA----
Sporosarcina sp. EUR3 2.2.2	WP_024536260	---R--RVPR-LVLH---SSA----		SQ-A--D--H--DSI-----
Sporosarcina sp. P1	WP_099626601	S---M-GVTNDLV-N---ANI---D		DA----E--Q---SIP----
Sporosarcina sp. P13	WP_099689274	S---M-GVTNDLV-N-S--SI----		S-----E--QV--SIP----
Sporosarcina sp. P16b	WP_099673591	S---M-GVTNDLV-N---ASI---D		EEF---E--Q---SIP----
Sporosarcina sp. P19	WP_099691005	S---M-GVTNDLV-N---ASI---D		DE----E--Q---SIP----
Sporosarcina sp. P20a	WP_099678566	S---M-GVTNDLV-N---ASI---D		DE----E--Q-R-SIP----
Sporosarcina sp. P29	WP_099661859	S---M-GVTNDLV-N---ASI---D		DE----E--Q-R-SIP----
Sporosarcina sp. P34	WP_099696564	S---M-GVTNDLV-N---ASI---D		DE----E--Q---SIP----
Sporosarcina sp. P7	WP_099635878	S---M-GVTNDLV-N---ASI---D		DEF---E--Q---SIP----
Sporosarcina sp. PTS2304	WP_114925234	S---M-DVTNDLV-N----KI---D		L-----E--QV--SIP----
Sporosarcina ureae	WP_029054364	S---M-GVTNDLV-N---ASI---D		DE----E--Q---SIP----
Tetzosporium hominis	WP_094941835	---E--NVNRDSVLN----S--T-D		E-A---D--HQQ--MS--Y-
Thermolongibacillus altinsuens	WP_132947254	A--NI-NV-R-TVLSA--VE---LD		EQ----T-VQERDS-I----
Ureibacillus thermophilus	QBK24845	---SF-KVTR-D-LN---AS-----		NQ-S--D--------S----
Ureibacillus thermosphaericus	WP_016838906	---S--NVTR---LN---AS-----		EN-S--D--H-------N--
Vibrio vulnificus	WP_133350715	--A---NV-S-TVMNQ--IE---L-		EE-K--D-LEER-SII--Y-
Viridibacillus arvi	WP_053418437	--Q---N-EDD-LM---LIS---L-		EE----D--H--DAV-----
Viridibacillus sp. OK051	WP_100795362	--Q--FKGEHD-LM---LIS---LD		EG----D--H--DAV-----

Supplemental Figure 30

A partial sequence alignment of the Sensor histidine kinase YycG protein containing a two amino acid insertion (boxed) that is exclusively shared by all members belonging to the Jejuensis clade and absent in all other bacteria.

Group	Species	Accession	68		114
**Jejuensis Clade (3/3)**	Lysinibacillus saudimassiliensis	CEA04824	FQYYAHGLLVLDAEKAFHDIRL		HEEPVYIEMILPNAMQNPHYVAVLE
	Lysinibacillus jejuensis	WP_108306735	----TG--HIQ--------V--		-DGAI-M-IKM-Q--T--Y------
	Lysinibacillus sp. BF-4	KFL44510	----S-----------------		----------------S--------
**Other Bacteria (0/>300)**	Amphibacillus jilinensis	WP_017472189	-RF-KDHIVTT----S------	N	RQD-I--QLSFE--H-SAE-S----
	Amphibacillus marinus	WP_091493435	-RF-KDHIVTT----S------	N	RH--I--QLSFE--N--AE-S----
	Amphibacillus sediminis	WP_067836507	-RF-KDHIVTT----S------	N	RH--I--QLSFE--H-SAE-S----
	Amphibacillus xylanus	WP_015009988	-RF-KDHIVTT----S------	N	RH--I--QLSFK-SH-SIE-N----
	Anaerobacillus arseniciselenat	WP_071311761	-SFHKRQHVTM----S------	N	K--EI--QLNFKD-KK--Q------
	Anaerobacillus isosaccharinicu	WP_071319382	-SFHKRQHVTM----S------	N	K--EI--QLNFKD-KK--Q--S---
	Anaerobacillus macyae	WP_048309252	-RF-K-NIITT-P--S------	N	QD--I--QLNFKSKNVS-QF-----
	Anoxybacillus amylolyticus	WP_066325793	-RF-KENVMTT----S------	N	RD-DI--QLNFRASFHS-Q------
	Anoxybacillus flavithermus	WP_003394563	-RF-KQNVVTT----S------	N	RD-DI--QLNFRG-FHS-Q------
	Anoxybacillus geothermalis	WP_044743309	-RF-KGNVMTT----S------	N	RD-DI--QLNFRASFHS-Q------
	Anoxybacillus gonensis	WP_009373663	-RF-KQNVITT----S------	N	RD-DIF-QLNFRG-FHS-Q------
	Anoxybacillus pushchinoensis	WP_091700355	-RF-KQNVVTT----S------	N	RD-DI--QLNFRG-FHS-Q------
	Anoxybacillus sp. P3H1B	WP_066145428	-RF-KENVMTT----S------	N	R--DI--QLNFRASFHS-Q------
	Anoxybacillus sp. UARK-01	WP_080862410	-RF-KENVMTT----S------	N	R--DI--QLNFRASFHS-Q------
	Anoxybacillus suryakundensis	WP_055440636	-RF-KQNVITT----S------	N	RD-DI--QLNFRG-FHS-Q------
	Anoxybacillus tepidamans	WP_027408565	-RF-KENVMTT----S------	N	RD-DI--QLNFRASFHS-Q------
	Anoxybacillus thermarum	WP_043967450	-RF-KQNVITT----S------	N	RD-DIF-QLNFRG-FHS-Q------
	Anoxybacillus vitaminiphilus	WP_111644008	-RF-KENVMTT----S------	N	RD-DI--QLNFRASFHS-Q------
	Aureibacillus halotolerans	WP_133579389	-RF-KDNIVTT---------M	H	DSDRI-VQLNFK-SY-T-QFA----
	Bacillus acidicola	WP_066262699	-RF-KENIMTT----S------	N	R--DI--QLNFKSSNLSYQ-A----
	Bacillus aciditolerans	WP_121448972	-RF-KENVMTT----S------	N	R--EI--QLNFRSSFHS-N------
	Bacillus alkalinitrilicus	WP_078428486	-CFHK-KHVTM-----------	N	NT-E---QLNFS--KT---------
	Bacillus alkalitelluris	WP_078546097	-RF-KENVMTT----S------	N	RD-DI--QLNFRSSFHT-Q------
	Bacillus alveayuensis	WP_044749085	-KF-KKNVMTT----S------	N	KD-DL--QLNFRL-YNS-E-A----
	Bacillus anthracis	WP_097852577	-HFFKQNVMTT----S------	N	RD-DI--QLNFKSSF--AN------
	Bacillus atrophaeus	WP_061572976	-RF-KENVMTT----S------	N	KQQDLF-QLNFRS-YNS-E-A----
	Bacillus badius	WP_041096942	-RFFKENIMTT----S------	N	K--DI---LKFKS-NVSHQ-A--M-
	Bacillus bingmayongensis	WP_017152508	-HFFKQNVMTT----S------	N	RD-DI--QLNFKSSF--AN------
	Bacillus bogoriensis	WP_026673057	-SFHKKKHVTM-----------	H	DD-D---QLNFIGSKV-H-------
	Bacillus camelliae	WP_101353270	-RF-KENIMTT----S------	N	RD-DI--QLNFKD-NISY--A----
	Bacillus cecembensis	WP_057982897	-RF-KKNIMTS----S---L--	N	PQ-LM-VQLNF--VPP--V-L----
	Bacillus cereus	WP_098912437	-HFFKQNVMTT----S------	N	RD-DIF-QLNFKSSF--AN------
	Bacillus cihuensis	WP_028391544	-RF-KSNIMTT----S------	N	RD-DI--QLNFKS-YSSY--A----
	Bacillus coagulans	WP_019720942	-RF-KDNIMTT----S------	N	R--DI--QLNFKKSQLSYQ-A----
	Bacillus cohnii	WP_066416842	-RF-KQNVMTT----S------	N	RD-DI--QLNFRS-YHS-Q-A----
	Bacillus cytotoxicus	WP_011984317	-HFFKQNVMTT----S------	N	RD-DI--QLNFKSSF---N------
	Bacillus endophyticus	WP_113749138	-RF-KENVMTT----S------	N	RD-DI--QLNFRSSYLS-Q------
	Bacillus fastidiosus	WP_066233704	-RF-KENVMTT----S------	N	KD-ELF-QLNFRS-YKS-Q-A----
	Bacillus funiculus	WP_129728142	-HF-KQNVMTT----S------	N	RD-DI--QLNFKSSF--AN------
	Bacillus gaemokensis	WP_033676952	-HFFKQNVMTT----S------	N	RD-DI--QLNFKSSF--AN------
	Bacillus ginsengihumi	WP_025729113	--F-REHIMTT----S---L--	N	RD-DI--QLNFKSSQLSH--A----
	Bacillus glycinifermentans	WP_048352934	-RF-KENVMTT----S------	N	KQQDLF-QLNFRS-YNS-E-A----
	Bacillus gobiensis	WP_053603160	-RF-KQNVMTT----S------	N	KNQSLF-QLNFRSSYHS-E-A----
	Bacillus halmapalus	WP_078380196	-RF-KENVMTT----S------	N	RD-DI--QLNFRSVYHS-Q-A----
	Bacillus halodurans	WP_134228679	-CFHKYQQVTM---------M	N	P--AI--QLHFSG-KH--N-I----
	Bacillus halosaccharovorans	WP_078432792	-RF-KENVMTT----S------	N	KD-ELF-QLNFRS-Y-S-Q-A----
	Bacillus halotolerans	WP_105991734	-RF-KENVMTT----S------	N	KQQDLF-QLNFRS-YSS-E-A----
	Bacillus horikoshii	WP_064097225	-RF-KENVMTT----S------	N	RD-DI--QLNFRSVYHS-Q-A----
	Bacillus humi	WP_057997406	-RF-KENVMTT----S------	N	R--EI--QLNFRSSFHS-N------
	Bacillus indicus	WP_029280283	-RF-KENVMTT----S------	N	KD-DL--QLNFRSSY-S-N-A----
	Bacillus intestinalis	WP_087986937	-RF-KENVMTT----S------	N	KQQDLF-QLNFRS-YSS-E-A----
	Bacillus koreensis	WP_053402315	-RF-KESVMTT----S------	N	RN-DI--QLNFRSSFLS-Q------
	Bacillus ligniniphilus	WP_017728016	-SFHKYHKVAM-----------	N	DD-EI--QLNFSG-KS--N-I----
	Bacillus litoralis	WP_066331916	-RF-KENVMTT----S------	N	KD-ELF-QLNFRSVY-S-Q-A----
	Bacillus luciferensis	WP_088069193	-HF-KQNVMTT----S------	N	RD-DI--QLNFKSSF-Y-MF-S---
	Bacillus manliponensis	WP_034636694	-HF-KQNVMTT----S------	N	RD-DI--QLNFKSSF--AN------
	Bacillus marinisedimentorum	WP_070121129	-RF-KENIMTT-V--S------	N	RD-----QLNFQG-FHS----L---
	Bacillus marisflavi	WP_048005875	-RF-KENIMTT----S------	N	K--DI--QLNFSK-NASYQFA----
	Bacillus massiliglaciei	WP_110927622	-RF-KSNIMTT----S------	N	RD-DI--QLNFKS-YSSHQ-A----
	Bacillus megaterium	WP_098980420	-RF-KENIMTT----S------	N	R--DI--QLNFRSLYSS-Q--S---
	Bacillus methanolicus	WP_004435270	-RF-KENVMTT----S------	N	RD-EI--QLNFHASFKA-Q-A----
	Bacillus muralis	WP_057914375	-RF-KSNIMTT----S------	N	RD-DI--QLNFKS-YSSYQ-A----
	Bacillus mycoides	WP_131231664	-HFFKQNVMTT----S------	N	RD-DI--QLNFKSSF--AS------
	Bacillus nakamurai	WP_061520964	-RF-KENVMTT----S------	N	KQQDLF-QLNFRS-YSS-E-A----
	Bacillus onubensis	WP_099351679	-RF-KENVMTT----S------	N	R--EI--QLNFRSSFHS-N------
	Bacillus oryziterrae	WP_017755568	-RF-KENVMTT----S------	N	RDQDI--QLNFRS-FSCAQ------
	Bacillus panaciterrae	WP_028400493	-HF-KQNVMTT----S------	N	RD-DI--QLNFKASF--AN------
	Bacillus persicus	WP_090744556	-RF-KENIMTT----S------	N	RD-EI--QLNFHS-NKA-QFA----
	Bacillus pseudofirmus	WP_075384754	-CFHK-KHVTM-----------	N	NT-E---QLNFS--KT---------
	Bacillus pseudomycoides	WP_041488369	-HFFKQNVMTT----S------	N	RD-DI--QLNFKSSF--AN------
	Bacillus pumilus	WP_099680979	-RF-KENIMTT----S------	N	KQQDLF-QLNFRS-YRS-E-A----

Other Bacteria (0/>300)

Bacillus siamensis	WP_045927201	-RF-KENVMTT----S------ N	KQQDLF-QLNFRS-YSS-E-A----
Bacillus simplex	WP_061144278	-RF-KSNIMTT----S------ N	RD-DI--QLNFKS-YSSYQ-A----
Bacillus sinesaloumensis	WP_077618588	-RF-KENVMTT----S------ N	RD-EI--QLNFRSSFHS-N------
Bacillus solimangrovi	WP_069716341	-RF-KENIMTT----S------ N	R-QDI--QLNFRG--TTTK------
Bacillus solisilvae	WP_087999571	-HF-KQNVMTT----S------ N	RD-DI--QLNFKSSF-Y-MF-S---
Bacillus sonorensis	WP_006636393	-RF-KENVMTT----S------ N	KQQDLF-QLNFRS-YNS-E-A----
Bacillus sp. 123MFChir2	WP_020059595	-HF-KQNVMTT----S------ N	RD-DI--QLNFKSSF--AN------
Bacillus sp. 7586-K	WP_095301144	-RF-KENVMTT----S------ N	KD-ELF-QLNFRS-Y-S-Q-A----
Bacillus sp. AFS018417	WP_098309402	-HF-KQNVMTT----S------ N	RD-DI--QLNFKSSF--AN------
Bacillus sp. AFS040349	WP_098796139	-RF-KENVMTT----S------ N	K--ELF-QLNFRSSY-S-Q-A----
Bacillus sp. AFS053548	WP_098862997	-HF-KQNVMTT----S------ N	RD-DI--QLNFKSSF-Y-MF-S---
Bacillus sp. AFS055030	WP_025671400	-HF-KQNVMTT----S------ N	RD-DI--QLNFKSSF-Y-MF-S---
Bacillus sp. AFS059628	WP_098670515	-HFFKQNVMTT----S------ N	RD-DI--QLNFKSSF--SN------
Bacillus sp. BA3	WP_101222412	-RF-KSNIMTT----S------ N	RD-DI--QLNFKSSYSSYQ-A----
Bacillus sp. BK245	WP_131841613	-HF-KQNVMTT----S------ N	RD-DI--QLNFKSSF-Y-MF-S---
Bacillus sp. CGMCC 1.16541	WP_110112958	-RF-KENIMTT----S------ N	RD-DI--QLNFRSSYSS-Q--V---
Bacillus sp. CHD6a	WP_060663948	-RF-KENVMTT----S------ N	RD-DI--QLNFRSVYHS-Q-A----
Bacillus sp. EAC	WP_088044140	-HF-KQNVMTT----S------ N	RD-DI--QLNFKSSF-Y-MF-S---
Bacillus sp. FJAT-25509	WP_056466666	-HF-KQNVMTT----S------ N	RD-DI--QLNFKSSF-Y-MF-S---
Bacillus sp. FJAT-27916	WP_049670568	-RF-KSNIMTT----S------ N	RD-DI--QLNFRT-YSSY--A----
Bacillus sp. FJAT-27986	WP_066107436	-RF-KSNIMTT----S------ N	KD-DI--QLNFRT-YSSYQ-A----
Bacillus sp. FJAT-42315	WP_100402464	-RFFKENIMTT----S------ N	K--NI---LKFRGSNTSHQ-A----
Bacillus sp. FJAT-42376	WP_123917250	-RF-KENVMTT----S------ N	RD-EL--QINFRSSY-S-Q-A--M-
Bacillus sp. FJAT-44921	WP_078553288	-SFHK-KHVTM----------- N	CD-NI--QLNFLGSKS--Q-A----
Bacillus sp. FJAT-45350	WP_096200923	-CFHKQQHVSM----------- N	N---I--QLNFQGSRT----I----
Bacillus sp. HMF5848	WP_125906420	-RF-KENVMTT----S------ N	RD-DI--QINFRSSYSC-Q------
Bacillus sp. HMSC76G11	WP_070878132	-RF-KENVMTT----S------ N	KD-DL--QLNFRS-Y-S-N-A----
Bacillus sp. HNG	WP_116352356	-RF-KENVMTT----S------ N	R--EI--QLNFRSSFHS-N------
Bacillus sp. J37	WP_026560198	-RF-KENVMTT----S------ N	KD-ELF-QLNFRS-Y-S-Q-A----
Bacillus sp. LL01	WP_047968950	-RF-KENVMTT----S------ N	RD-DI--QLNFRSVYHS-Q-A----
Bacillus sp. M6-12	WP_101594518	-RF-KSNVMTT----S------ N	RD-EI--QLNFRS-YSSHQ-A----
Bacillus sp. Marseille-P3661	WP_102345786	-RF-KENVMTT----S------ N	RDQDI--QLNFRSSFSCAQ------
Bacillus sp. OV186	WP_107901722	-HF-KQNVMTT----S------ N	RD-DI--QLNFKSSF-Y-MF-S---
Bacillus sp. OxB-1	WP_041074530	-RF-KGN-MTA----S---L-- H	PDDKM--QLNF--SHAC-Q-AS-R-
Bacillus sp. PK3_68	WP_120032586	-RFFKENIMTT----S------ N	K--DI---LKFKSSNVSHQ-A--M-
Bacillus sp. SA1-12	WP_046590378	-RF-KENVMTT----S------ N	KD-ELF-QLNFRS-Y-S-Q-A----
Bacillus sp. SA5d-4	WP_094923817	-RF-KENVVTT-P--T------ N	QQDEL--QLNFKS--TS---A----
Bacillus sp. SJS	WP_035408680	-RF-KENVMTT----S------ N	R--EL--QINFRS-Y-S-Q-A--M-
Bacillus sp. SYSU K30001	WP_124564035	-HF-KQNVMTT----S------ N	RD-DI--QLNFTSSF--AN------
Bacillus sp. UMB0893	WP_101568268	-RF-KENVMTT----S------ N	KD-DLF-QLNFRS-Y-S-N-A----
Bacillus sp. UMB0899	WP_102229199	-RF-KENVMTT----S------ N	KD-ELF-QLNFRS-Y-S-Q-A----
Bacillus sp. V44-8	WP_117322240	-RF-KSNIMTT----S------ N	RD-EI--QLNFKS-YSSYQ-A----
Bacillus sp. V47-23a	WP_117327012	-RF-KSNIMTT----S------ N	RD-EI--QLNFRS-YSSHQ-A----
Bacillus sp. V5-8f	WP_101632289	-RF-KSNIMTT----S------ N	R--EI--QLNFKS-YSSYQ-A----
Bacillus sp. V59.32b	WP_117305171	-RF-KSNIMTT----S------ N	R--EI--QLNFKS-YSSYQ-A----
Bacillus sp. YR335	WP_111614271	-RF-KENVMTT----S------ N	KD-ELF-QLNFRSVY-S-Q-A----
Bacillus sp. es.036	WP_098443960	-RF-K-NIITT-P--S------ N	QD--I--QLNFKSKNVS--------
Bacillus sp. mrc49	WP_100531410	-RF-KSNIMTT----S------ N	RD-DI--QLNFKS-YSSYQ-A----
Bacillus sporothermodurans	KYD09258	-RF-KENIMTT----S------ N	R--DI--QLNFKA-NISYQ-A----
Bacillus stratosphericus LAMA	EMI12062	-RF-KENIMTT----S------ N	KQQDLF-QLNFRS-YRS-E-A----
Bacillus subtilis	WP_019714768	-RF-KENVMTT----S------ N	KQQDLF-QLNFRS-YSS-E-A----
Bacillus taeanensis	WP_113805174	-RF-KQNIITT----S------ N	Q--DI--QLNFLA-RK--K-A--V-
Bacillus tequilensis	WP_024715261	-RF-KENIMTT----S------ N	KQQDLF-QLNFRS-YSS-E-A----
Bacillus testis	WP_050615420	-KF-KSNVMTT----S------ N	R--DI--TLHFRS-YSSY--A----
Bacillus thuringiensis	WP_001095927	-HFFKQNVMTT----S------ N	RD-DI--QLNFKSSF--AN------
Bacillus timonensis	WP_010284166	-RF-KENVMTT----S------ N	R--EI--QLNFRSSFHS-N------
Bacillus toyonensis	WP_100062446	-HFFKQSVMTT----S------ N	RD-DI--QLNFKSSF--AN------
Bacillus velezensis	WP_053104006	-RF-KENVMTT----S------ N	KQQDLF-QLNFRS-YSS-E-A----
Bacillus weihaiensis	WP_072579404	-RF-KENVMTT----S------ N	K--ELF-QLNFRSSY-S-N-A----
Bacillus wiedmannii	WP_098625022	-HFFKQNVMTT----S------ N	RD-DI--QLNFKSSF--AN------
Bhargavaea beijingensis	WP_092095125	-RF-KGN-MTA----S---L-- H	P--NM-LQLNF--RHAC-E-A----
Bhargavaea cecembensis	WP_063179173	-RF-KGN-MTA----S---L-- H	P--NM-LQLNF--RHAC-E-A----
Bhargavaea ginsengi	WP_092048687	-RF-KGN-MTA----S---L-- H	P--NM-LQLNF--RHAC-E-A----
Brochothrix thermosphacta	WP_120487328	-LFFKKH-VTT-PQ-------M H	P-QAI-VQVDF-H-LTS-E--G-K-
Caenibacillus caldisaponilytic	WP_077616344	-I-RVRNVVTEQP------L-- N	TD-----RL-F-D-SRSAE-A----
Caldibacillus debilis	WP_061567919	-RF-KESVMTT----S------ N	RN-DI--QL-FKEMY-SHR-A----
Candidatus Parcubacteria bacte	RJR08861	-RF-KENVMTT----S------ N	R--DI--QLNFRSVYHS-Q-A----
Caryophanon latum	WP_066465126	-RF-KGNIMTS----S---L-- H	DD-AI--QLNFQ-VPPSAE-L----
Chryseomicrobium excrementi	WP_100352620	-RF-KGTIVSA----S---M-- H	PDQ-M---LKF-DKQRV-EFI----
Dolosigranulum pigrum	WP_112790691	-RL-KE-FA-Q-P-R---EM-M H	WT-KL---LNF-D-FL--L--K---
Domibacillus aminovorans	WP_063966890	-RFFKDNIMTT----S------ N	K--DIF--LKFNG-P-SHQ-A--R-
Domibacillus epiphyticus	WP_076767720	-RFFKDNIMTT----S------ N	K--DIF--LKFYG-P-SH--A--R-
Domibacillus mangrovi	OKL37982	-RFFKDNIMTT----S------ N	K--DIF--LKFNG-P-SHQ-A--R-
Edaphobacillus lindanitolerans	WP_076756398	-RF-KGN-MTA----S---L-- H	P--NMFLQLNF-GRHSC-E-A----

Other Bacteria (0/>300)

Falsibacillus pallidus	WP_114745325	-RF-KENIMTT----S------	N	R--EIF-QLNFRSSQASHQ------
Fictibacillus aquaticus	WP_094250497	-RF-KQNV-TT----S------	N	Q--DI-L-LNFNSSRLT-E-AS---
Fictibacillus gelatini	WP_026676961	-RF-KQNV-TT----S------	N	QD-DI---LNFNSSRLT-E-AS---
Fictibacillus macauensis	WP_007200796	-RF-KQNV-TT----S------	N	Q--DIF--LNFNS-RLT-E-AS---
Fictibacillus phosphorivorans	WP_066241823	-RF-KQNV-TT----S------	N	Q--EI-L-LNFNASRLT-E-AS---
Geobacillus jurassicus	WP_066229158	-RF-KGNVMTT----S------	N	RD-DI--QLNFRASFHS-Q------
Geobacillus kaustophilus HTA42	BAD76479	-RF-KGNVMTT----S------	N	RD-DI--QLNFRASFHS-Q------
Geobacillus sp. 12AMOR1	AKM19424	-RF-KGNVMTT----S------	N	RD-DI--QLNFRASFHS-Q------
Geobacillus stearothermophilus	KZE97477	-RF-KGNVMTT----S------	N	RD-DI--QLNFRASFHS-Q------
Gracilibacillus dipsosauri	WP_054859368	-RF-KKHIVTT--D-S------	H	ND--I--QLNFKG-Q-SVQ-MN---
Gracilibacillus lacisalsi	WP_018933720	-RF-KKHIVTT--D-S------	H	AK--I--QLNFKDSK-CIP-MR---
Gracilibacillus orientalis	WP_091483458	-RF-KKHIVTT--D-S------	H	AK--I--QLNFKDSR-CIP-IK---
Gracilibacillus ureilyticus	WP_089738323	-RF-KKHIVTT--D-S------	H	NK--I--QLNFK-SN-SVE-MK---
Halobacillus dabanensis	WP_075036711	-RF-KGQVMTN----S---L-M	N	QG--I--Q-NFD--Q-CSK-AL---
Halobacillus hunanensis	WP_079530121	-RF-KGQVMTN----S---L-M	N	Q------Q-NFED-HKCSM-AL---
Halobacillus kuroshimensis	WP_027954409	-RF-KGQVMTN----S---L-M	N	QG-----Q-NFE--Q-CSK-AL---
Halobacillus litoralis	WP_128522553	-RF-KGQVMTN----S---L-M	N	QG-----Q-NFE--Q-CSK-AL---
Halobacillus mangrovi	WP_085029731	-RF-KGQVMTN----S---L-M	N	QD-----Q-NFD--Q-CSK-AL---
Halobacillus salinus	WP_079479966	-RF-KGQVMTN----S---L-M	N	KD-----Q-NFE--HACSK-AL---
Halobacillus sp. BBL2006	WP_035551165	-RF-KGQVMTN----S---L-M	N	QD-----Q-NFDK-Q-CSK-AL---
Halobacillus sp. Marseille-P38	WP_101844082	-RF-KGQVMTN----S---L-M	N	QD--I--Q-NFE--H-CSM-AL---
Jeotgalibacillus alimentarius	WP_041122698	-RF-KGHVMTT----S------	H	RD-DL--QLNFES-YSSYQ-A----
Jeotgalibacillus malaysiensis	WP_039809393	-RF-KEQVMTT----S------	H	R--DL--QLNFES-YSSYQ-A----
Kurthia gibsonii	WP_121177523	-RF-KGN-MTA----S---L--	N	S--DM--QLNF-KIPP--A-L----
Kurthia sibirica	WP_109306913	-RF-KGNIMTA----S---L--	H	PD-DM--QLNF-KLPPT-S-L----
Kurthia sp. 11kri321	WP_068454388	-RF-KGN-MTA----S---L--	N	S--DM--QLNF-KIPP--A-L----
Lentibacillus persicus	WP_090087549	-RF-KKQ-VTT---------M	N	QG--L--QLNFSKSY--SFFA----
Lentibacillus sediminis	WP_100011188	-RF-KNH-VTT----S------	N	RDQ-LFLQLNFK-SK--AC-A----
Lentibacillus sp. Marseille-P4	WP_106497413	-RF-KSQ-VTT----S------	N	QK-AL--QLNFN--K--SL------
Lentibacillus sp. SSKP1-9	WP_129674570	-RF-KKQ-VTT----S-----M	N	QN--L--QLNFNKSH--SN-A----
Lysinibacillus acetophenoni	WP_097148940	-RF-KGNIMTS----S---L--	H	PN-SM--QLNF--VPPSQQ-L----
Lysinibacillus chungkukjangi	WP_107937161	-RF-KGNIMTS----S---L--	H	PN-SM--QLNF--IPPSQQ-L----
Lysinibacillus composti	WP_124764988	-RF-KGNIMTS----S---L--	H	PS-SM--QLNF--IPPSQQ-L----
Lysinibacillus contaminans	WP_053584014	-RF-KGNVMTA----S---L--	N	EK-DM-LQLNF--IPPSTQ-L----
Lysinibacillus endophyticus	WP_121214720	-RF-KGNIMTS----S---L--	H	PN-SM--QLNF--IPPSQQ-L----
Lysinibacillus halotolerans	WP_122970913	-RF-KGNIMTS----S---L--	H	PN-SM--QLNF--IPPSGQ-L----
Lysinibacillus manganicus	WP_036183758	-RF-KGNIMTS----S---L--	H	PN-SM--QLNF--VPPSRQ-L----
Lysinibacillus massiliensis	WP_036179898	-RF-KGNIMTS----S---L--	H	PN-SM--QLNF--IPPSTQ-L----
Lysinibacillus parviboronicapi	WP_054768035	-RF-KGNVMTA----S---L--	H	EHDDM--QLNF--IPPSAQ-L----
Lysinibacillus sinduriensis	WP_036203660	-RF-KGNIMTS----S---L--	H	PG-SM--QLNF--IPPSQQ-L----
Lysinibacillus sp. B2A1	AVK83626	-RF-KGNVMTA----S---L--	H	EQ-DM--QLNF--IPPSAQ-L----
Lysinibacillus sp. BK089	WP_132359230	-RF-KGNVMTA----S---L--	H	EH-DM--QLNF--VPPSAQ-L----
Lysinibacillus sp. FJAT-14222	WP_053593562	-RF-KGNVMTA----S---L--	H	EH-DM--QLNF--VPPSAQ-L----
Lysinibacillus sp. FJAT-14745	WP_053482550	-RF-KGNVMTA----S---L--	H	EH-DM--QLNF--IPPSAQ-L----
Lysinibacillus sp. Marseille-P	WP_106784699	-RF-KGNIMTS----S---L--	H	PN-SM--QLNF--VPPSQQ-L----
Lysinibacillus sp. YLB-03	WP_118874321	-RF-KGNIMTS----S---L--	H	PN-SM--QLNF--VPPSQQ-L----
Lysinibacillus sphaericus	WP_010858819	-RF-KGNVMTA----S---L--	H	EH-DM--QLNF--IPPSAQ-L----
Lysinibacillus sphaericus	WP_031417985	-RF-KGNVMTA----S---L--	H	EQ-DM--QLNF--IPPSAQ-L----
Lysinibacillus sphaericus	WP_036215338	-RF-KGNVMTA----S---L--	H	EQ-DM--QLNF--IPPSAQ-L----
Lysinibacillus sphaericus	WP_075528201	-RF-KGN-MTA----S---L--	Y	EN-KM--QLNF--NHAC---AS-R-
Lysinibacillus sphaericus	WP_112117666	-RF-KGNVMTA----S---L--	H	EQ-DM--QLNF--IPPSAQ-L----
Lysinibacillus sphaericus	WP_125103689	-RF-KGS-MTT----S---L--	H	PD-KMF-QLNFLKSNSS---AS---
Lysinibacillus sphaericus C3-4	ACA39555	-RF-KGNVMTA----S---L--	H	EQ-DM--QLNF--IPPSAQ-L----
Lysinibacillus telephonicus	WP_126294180	-RF-KENIMTS----S---L--	H	PNDSM--QLNF--IPPSQQ-L----
Lysinibacillus xylanilyticus	WP_068982835	-RF-KGNVMTA----S---L--	H	EQ-DM--QLNF--VPPSAQ-L----
Lysinibacillus xyleni	WP_097074465	-RF-KGNIMTS----S---L--	H	PN-SM--QLNF--IPPSQQ-L----
Melghiribacillus thermohalophi	WP_132371099	-RF-KEQIMTT-P--S------	N	DM--I--QLNFK--ARH-R-AV---
Oceanobacillus bengalensis	WP_121133123	-RF-KNH-VTT----S---V--	N	QD--L--QLNFKK-H--AI-AS-I-
Oceanobacillus damuensis	WP_067725485	-RF-KNH-VTT----S--E---	N	QN--L-LQLNFKKSN-HSY-AS---
Oceanobacillus halophilus	WP_121202731	-RF-KNH-VTT----S------	N	QD-DL--QLNFSKSH--SN--S---
Oceanobacillus iheyensis	WP_106896803	-RF-KNQ-VTT----S------	N	-N--L-LQLNFKKSY--AA------
Oceanobacillus manasiensis	WP_042223598	-RF-KNQ-VTT----S------	N	-N--L-LQLNFKKSY--AA------
Oceanobacillus massiliensis	WP_010650653	-RF-KNH-VTT----S--E---	N	QN--L-LQLNFKRSN-HSY-AS---
Oceanobacillus picturae	WP_036572827	-RF-KNQ-VTT----S------	N	-N--L-LQLNFKKSY--AA------
Oceanobacillus rekensis	WP_087971523	-RF-KNH-VTT----S--E---	N	QD--L-LQLNFKKSN-HSY-AS---
Oceanobacillus sp. 160	WP_114916630	-RF-KNQ-VTT----S------	N	QQ-DL--QLNFK--N--AY-ASIM-
Oceanobacillus sp. YLB-02	WP_121521184	-RF-KNH-VTT----S------	N	QD-DL--QLNFL-SQ-D-N--I---
Ornithinibacillus californiens	WP_047985304	-RF-KNH-VTT-----------	N	QH-SL--QLNFNKSN--HK-A----
Ornithinibacillus contaminans	WP_047980050	-RF-KNH-VTT-----------	N	QQ-SL--QLNFT--N--HM-A----
Ornithinibacillus halophilus	WP_072887331	-RF-K-Q-VTT-PD-S------	N	Q---L--QLNFK-SF--TM-A----
Ornithinibacillus scapharcae	WP_010095899	-RF-KNH-VTT-----------	N	QH-SL--QLNFNK-N--HM-A----
Paenibacillus sp. VT-16-81	OPG94634	-RF-KENVMTT----S------	N	KQQDLF-QLNFRS-YNS-E-A----
Paenisporosarcina indica	WP_075617683	-RF-KGN-MTA----S---L--	H	PN-DMF-QLNF--VPPSSE-L----
Paenisporosarcina sp. HGH0030	WP_016427407	-RF-KGN-MTA----S---L--	N	PN-DMF-QLNF--VPPSSE-L----

Other Bacteria (0/>300)

Paenisporosarcina sp. K2R23-3	WP_119883136	-RF-KGN-MTS----S---M--	N	PK--L--QLNF-LFPPS-E-L----
Paenisporosarcina sp. TG20	WP_019414786	-RF-KGN-MTA----S---L--	H	PN-DMF-QLNF--VPPSSE-L----
Parageobacillus caldoxylosilyt	WP_042409745	-RF-KGNVMTT----S------	N	RD-DI--QLNFRASFHS-Q------
Parageobacillus genomosp. 1	WP_043905251	-RF-KGNVMTT----S------	N	RD-DI--QLNFRASFHS-Q------
Parageobacillus thermantarctic	WP_090948803	-RF-KGNVMTT----S------	N	RD-DI--QLNFRASFHS-Q------
Parageobacillus thermoglucosid	WP_064550228	-RF-KGNVMTT----S------	N	R--DI--QLNFRASFHS-Q------
Paraliobacillus quinghaiensis	WP_117152152	-RF-KNHIVTT----S------	N	RDD-I--QLSFE-VH--SN------
Paraliobacillus ryukyuensis	WP_079709690	-RF-KNHIVTT----S------	N	R---I--QLSFE--H-TAN------
Paraliobacillus sediminis	WP_117169366	-RF-KNHIVTT----S------	N	RSD-I-VQLSFE--H--AN------
Paraliobacillus sp. PM-2	WP_090853474	-RF-KNHIVTT----S------	N	RSD-I--QLSFE--H-TAN------
Paucisalibacillus globulus	WP_096270455	-RF-KNH-VTT-----------	N	QH-SL--QLNFN--N--HM-A----
Paucisalibacillus sp. EB02	WP_042145659	-RF-KNH-VTT-----------	N	QH-SL--QLNFN--N--HM-A----
Planococcus citreus	WP_121297575	--F-KGK-MTA----S---L--	H	PD--L-VQLNF-SIPPA-L-L----
Planococcus maitriensis	WP_112231674	--F-KGK-MTA----S---L--	H	PD--L-VQLNF-SIPPA-L-L----
Planococcus maritimus	WP_068485788	--F-KGK-MTA----S---L--	H	PD--L--QLNF-SIPPASL-L----
Planococcus massiliensis	WP_052651403	-RF-KGN-MTA----S---L--	N	PDDDL-VQLNF--RPPS-L-LS---
Planococcus plakortidis	WP_068869293	--F-KGK-MTA----S---L--	H	PD--L-VQLNF-SIPPA-L-L----
Planococcus rifietoensis	WP_058380567	--F-KGK-MTA----S---L--	H	PD--L-VQLNF-SIPPA-L-L----
Planococcus salinus	WP_123164194	-RF-KGN-MTA----S---L--	N	P--DL-VQLNF--RPPA-L-IS---
Planococcus sp. Y42	WP_077589518	-RF-RGH-MTA----T---L--	H	PD-EL-VQLNFR-LPRS-E------
Planomicrobium flavidum	WP_088007556	-RF-RGH-MTA----T---L--	H	P--EL-VQLNFR-MPPS-EFM----
Planomicrobium soli	WP_106531769	-RF-KGN-MTA----S---L--	N	P-QEL-VQLNF--RPPS-L-LS---
Pontibacillus chungwhensis	WP_036779385	-RF-KDQIMTT----S------	N	RN-A---QLNFK-SH-SAS-A----
Pontibacillus litoralis	WP_036832909	-RF-KNQVMTT----S------	N	RS-A---QLNFKEGL-TA-------
Pontibacillus marinus	WP_081673029	-RF-KDQIMTT----S------	N	RN-----QLNFKS-H-CAQ-A----
Pontibacillus yanchengensis	WP_036818564	-RF-KDQIMTT----S------	N	RN-A---QLNFK-GH-SSQ-A----
Psychrobacillus insolitus	WP_111438324	-RF-KGSIMTA----S---L--	H	PD-EM--QLNFQTLPPS-E-L----
Psychrobacillus psychrotoleran	WP_093534735	-RF-KGSIMTA----S---L--	H	P--DM--QLNFQMMPPS-E-L----
Psychrobacillus sp. OK028	WP_093060854	-RF-KGSIMTA----S---L--	H	P--DM--QLNFQMMPPS-E-L----
Psychrobacillus sp. OK032	WP_093273092	-RF-KGSVMTA----S---L--	H	P--DM--QLNFQTLPPS-E-L----
Quasibacillus thermotolerans	WP_039237768	-RFFKENVMTT----T------	N	K--DI---LKFKSSNVSHQ-A----
Salipaludibacillus agaradhaere	WP_078576855	-AFHKNQHVTM----S------	N	RN-DIF-QLNFKDKQM--S------
Salipaludibacillus sp. KQ-12	WP_110608158	-AFHKNQHVTM----S------	N	RN-DI--QLNFKEKQSS---M----
Sediminibacillus albus	WP_093210374	-RF-KNHIVTT----S------	N	RKD----QLNFK--N-SAP------
Sediminibacillus halophilus	WP_074597143	-RF-KNHIVTT----S------	N	RKD----QLNFK--N-SAS------
Sediminibacillus massiliensis	WP_077621742	-RF-KNHIVTT----S------	N	RK-----QLNFK-SH-CAS------
Solibacillus kalamii	WP_087618259	-RF-KENIMTS--------L-I	N	AH-AI-FQLNF--VPPD-L-L----
Solibacillus sp. R5-41	WP_099425768	-RF-KKNIMTS----S---L--	N	PQ-LM-VQLNF--VPP--V-L----
Sporosarcina koreensis	WP_040286723	-RF-KGS-MTT----S---L--	H	PD-KM--QLNFQKSNSS---AS---
Sporosarcina pasteurii	WP_115360676	-RF-KGN-MTA----S---L--	Y	EN-KM--QLNF--NHAC---AS-R-
Sporosarcina psychrophila	WP_067210355	-RF-KGN-MTA----S---L--	H	PDDKM--QLNFS-SHSC-E-AS-R-
Sporosarcina sp. BI001-red	WP_116016643	-RF-KGS-MTT----S---L--	H	PD-KMF-QLNFLKSNSS---AS---
Sporosarcina sp. D27	WP_025784876	-RF-KGS-MTT----S---L--	H	PD-KMF-QLNFLKSNSS---AS---
Sporosarcina sp. EUR3 2.2.2	WP_024534850	-RF-KGN-MTA----S---L--	H	PN-DM--QLNF-LVPPS-E-L----
Sporosarcina sp. HYO08	WP_067407095	-RF-KGN-MTA----S---L--	H	PDDKM--QLNF--NHTC-Q-AS-R-
Sporosarcina sp. ZBG7A	WP_039041770	-RF-KGS-MTT----S---L--	H	PD-KMF-QLNFLKSNSS---AS---
Streptococcus pneumoniae	CKG52787	-HFFKQNVMTT----S------	N	RD-DI--QLNFKSSF--AN------
Streptohalobacillus salinus	WP_110251031	-VF-KDK-VST-V------M-M	N	KH--L---LDFL-RKRDI--MK---
Tenuibacillus multivorans	WP_093857081	-R--KDHVVTT-VD--------	N	P--TL----VYD-VR-SL--AE---
Terribacillus aidingensis	WP_097041132	-RF-KDNIVTT----S-----M	N	KKD----QLNFK--N-SME------
Terribacillus halophilus	WP_077308995	-RF-KDNIVTT----S-----M	N	KKD----QLNFK--N-SME------
Terribacillus saccharophilus	WP_095227049	-RF-KDNIVTT----S-----M	N	KKD----QLNFK-SH-SME------
Terribacillus sp. 7520-G	WP_095215253	-RF-KDNIVTT----S-----M	N	KKD----QLNFK-SH-SME------
Tetzosporium hominis	WP_094941776	-RF-KGTIVSA----S---M--	H	PDQ-M---LKF-DKQRV-EFI----
Thalassobacillus cyri	WP_093044689	-RF-KDKVMTT----S------	N	Q-D-L--QLNFDQ-N-SAR-A----
Thalassobacillus devorans	WP_085508688	-RF-KDKVMTT----S------	N	Q---L--QLNFDQ-N-SAR-A----
Thalassobacillus sp. TM-1	WP_062446359	-RF-KDKVMTT----S------	N	Q---L--QLNFDQ-N-SAR-A----
Thermolongibacillus altinsuens	WP_132947092	-RF-KENVVTT----S------	N	RD-DI--QLNFRG-FHS-Q------
Ureibacillus thermophilus	QBK25858	-RF-KGNIMTS----S---L--	Y	PN-DM--QLNF--VPP--L-L----
Ureibacillus thermosphaericus	WP_016836969	-RF-KGNIMTS----S---L--	Y	PN-DM--QLNF--VPP-QL-L----
Vibrio vulnificus	WP_133347570	-RF-KSNIMTT----S------	N	RD-DI--QLNFKSSYSSYQ-A----
Virgibacillus alimentarius	WP_029267888	-RF-KKQ-VTT----S------	N	QD--L--QLNFK--K-SAF------
Virgibacillus halodenitrifican	WP_121615001	-RF-KNQ-VTT----S------	N	Q-D-L-LQLNFKKSY--AF--S---
Virgibacillus indicus	WP_094883717	-RF-KNQ-VTT----S------	N	QN-DL-LQLNFK--N--SF------
Virgibacillus massiliensis	WP_038243936	-RF-KNQ-VTT----S------	N	KN-AL-LQLNFSKSY--EL-IS---
Virgibacillus ndiopensis	WP_099158352	-RF-KNQ-VTT----S------	N	QK--L--QLNFK--N--AF------
Virgibacillus necropolis	WP_089532365	-RF-KNH-VTT----S-----M	N	R--AL-VQLNFH-SI--AS------
Virgibacillus pantothenticus	SIS53627	-RF-KNQ-VTT----S------	N	KT-AL-LQLNFKKSY--AL-I----
Virgibacillus phasianinus	WP_089062603	-RF-KNH-VTT----S-----M	N	R---L-VQLNFH-SI--AS------
Virgibacillus profundi	WP_095655692	-RF-KNQ-VTT-S--S------	N	QND-L-LQLNFK--N--SF------
Virgibacillus proomii	WP_077321530	-RF-KNQ-VTT----S------	N	KN--L-LQLNFHKSY--AL-IS---
Virgibacillus senegalensis	WP_053219407	-RF-KNHIVTT----S------	N	RKD----QLNFK--N-SAS------
Virgibacillus siamensis	WP_077326674	-RF-KNQ-VTT----S------	N	QHD-L--QLNFDK-N--IY-A----
Virgibacillus sp. 7505	WP_095222668	-RF-KDNIVTT----S-----M	N	KKD----QLNFK-SH-SME------

Supplemental Figure 31

A partial sequence alignment of the hypothetical protein BN1050_02162 containing a one amino acid deletion (boxed) that is exclusively shared by all members belonging to the Jejuensis clade and absent in all other bacteria.

Group	Organism	Accession	20		69
**Jejuensis Clade (3/3)**	Lysinibacillus saudimassiliensis	CEA04602	YFYMKKVNNDKVNGAKYQAQAIIDD	AHKT	AQREAEALKKEAILEAKDETH
	Lysinibacillus sp. BF-4	WP_036141570	-----------I-------------	----	---------------------
	Lysinibacillus jejuensis	WP_108305860	-----------I---------L--	----	---------------------
**Other Bacteria (0/>300)**	Aeribacillus pallidus	WP_063386730	--VR-SIAEA-IA---NA-EQ-LE-		-K-----T----L------I-
	Anoxybacillus vitaminiphilus	WP_111643797	F-VR-SIAEA-IG--QAA-NQ--EE		-K---D------L------I-
	Aureibacillus halotolerans	WP_133580499	--VR-SIAEA-ISS-ENA--Q-V-E		GK---D------L------I-
	Bacillus acanthi	WP_108669327	--VR-SISEN-IA---SE-ER----		-K----------L------I-
	Bacillus acidicola	WP_066264558	-I-R-TIAEA-IA--RNA-EQ-LEE		-K----------L------N-
	Bacillus aciditolerans	WP_121446645	FYVR-SIAET-IA---SV-EQ--E-		-N---DS-----L------I-
	Bacillus alkalitelluris	WP_078546989	F-VR-SIAET-IA---GA-EQ----		-N---DS-----L------I-
	Bacillus alveayuensis	WP_044746904	--AR-SIAEA-IA---NA-VQ--E-		-K-----T----L------I-
	Bacillus amyloliquefaciens	AOC91112	-YVR-TIAEA-IA--RGA-EQ-LE-		-K-D--------L------I-
	Bacillus andreraoultii	WP_033829018	--IR-SIAEE-IA---DA-EK-LE-		-K-D--S-----L------I-
	Bacillus anthracis	WP_098313580	F-VR-SIAEA-I---ANE-RR-L-E		-N-D--------L------I-
	Bacillus aquimaris	WP_064093641	-LIR-SIAEA-IA---GS-EH-LE-		-K---D------L------N-
	Bacillus asahii	WP_127762496	--VH-SKFDN-IA--RGS-EQ-VE-		-K----------L------I-
	Bacillus azotoformans	WP_003330384	--VR-SIAEA-ITS-EHA--Q--E-		-K-D---S----L------N-
	Bacillus badius	WP_041100117	--VR-SIAES-IA---SA-DQ-V--		-K----S-----L------I-
	Bacillus bataviensis	WP_007087156	--VH-SIAEA--A---NV-EQ-LE-		-K-D-DSM----L------I-
	Bacillus boroniphilus JCM 2173	GAE43747	---R-S-AEA-IA---NA-EQ-VE-		-K-----M---SL------N-
	Bacillus butanolivorans	WP_116821795	--IH-SKFES-IA---GS-EQ-LE-		-K----------L------I-
	Bacillus camelliae	WP_101355729	--VR-SFAEARIT---NA-EQ-LE-		-K----------L------N-
	Bacillus campisalis	WP_046522103	---R-S-AEA-IA---NA-EQ-LE-		-K-D--------L-----DI-
	Bacillus cavernae	WP_126864538	--VS-S-SQN-IA---NS-EH-LE-		-R-----A----L------I-
	Bacillus cecembensis	WP_057986892	-V------ES--T---HV-EQ-VE-		-K---D------L--------
	Bacillus cereus	WP_097949862	F-VR-SIAEA-I---SNE-KR-L-E		-N----------L------I-
	Bacillus cihuensis	WP_028392770	--VF-NITQN-IA--RNS-EQ-LE-		-K----------L-----DI-
	Bacillus coagulans	WP_017553388	--IRRSIYEA-IA---NT-DQ-V-E		-K---DR-----L------N-
	Bacillus coahuilensis	WP_010172577	---R-SIAEA-I---ENA-EQ-LEK		GK----------L------I-
	Bacillus cucumis	WP_101647904	--VH-SIADA--A---NA-EQ-LE-		-K-D-DS-----L------I-
	Bacillus cytotoxicus	WP_012094870	F-VR-SIAEA-I---ANE-KR-L--		-N----------L------I-
	Bacillus dakarensis	WP_077211594	--IR-S-AEA-IA---DA-EQ-LE-		-K---DS-----L------I-
	Bacillus daliensis	WP_090840075	--VR-SIAEA-ISS-EML-KTT---		-K----SS-----------A-
	Bacillus deserti	WP_101639979	--VR-SITEN-IA---VS-EQ--E-		-K---DSK----L------I-
	Bacillus dielmoensis	WP_042456501	--IR-SIAEA-IA---NA-EQ-LE-		-K-D-DT-----L------I-
	Bacillus drentensis	WP_066252801	--VH-SIAEA--A---NA-EQ-LE-		-K-D-DS-----L------I-
	Bacillus endophyticus	WP_061801667	--IRRSIAEA-IA---SA-EQ-V-E		GK----S-----L------I-
	Bacillus firmus	WP_048008961	--VR-SIAEA-IA---DA-EQ-LE-		-K-----T----L------I-
	Bacillus foraminis	WP_121609191	---R-S-AEA-IA--RNA-EQ-LE-		-K-D-------SL-----DI-
	Bacillus fordii	WP_018705047	--IF-S-AQA-IT--HGS-DQ-LEE		-K----S-----L------N-
	Bacillus fortis	WP_120069848	--IF-S-AQA-IT--QGS-EQ-LEK		-K----S-----L------N-
	Bacillus fumarioli	WP_066368230	--IR-SIAEA-IA---NA-EQ--E-		-K-D--S-----L------I-
	Bacillus funiculus	WP_129726733	F-VR-SIAEA-I---TNA-KQ-VEE		-R--G-------L------I-
	Bacillus gaemokensis	WP_033672018	F-VR-SIAEA-I---ANE-RR-L-E		-N----------L------I-
	Bacillus glycinifermentans	WP_048354765	-YVR-IIAEA-IS--RNA-EQ-LG-		-K-D--------L------I-
	Bacillus gobiensis	WP_053602868	--VR-TIAEA-IA--RTA-EQ-VA-		-K-D--------L-----DI-
	Bacillus gottheilii	WP_066446406	--VR-SISEA-IA---DA-EQ-LE-		-K-----T----L-----DI-
	Bacillus haynesii	WP_043926428	-YVR-IIAEA-IS--RNA-EQ-LG-		-K-D--------L------I-
	Bacillus horikoshii	WP_088018145	FYVR-SIAEQ-IA--RSV-DQ-VE-		SR----S-----L------I-
	Bacillus horneckiae	WP_066393755	--VC-SIAKA--A---DA-EQ-LE-		-K---D-I----LI-----I-
	Bacillus humi	WP_057999890	FYVR-S-AET-IA---GV-EQ--E-		-N---DS-----L------I-
	Bacillus indicus	WP_029565644	--VR-SIAEA-IA---GT-EQ-LE-		-R-D---T----L-----DI-
	Bacillus jeotgali	WP_079508504	---R-S-AEA-IA---NA-EQ-VE-		-K-----M----L------N-
	Bacillus kochii	WP_095369460	--FR-S-TEA-IA--RGA-DQ-LE-		-K----------L------N-
	Bacillus koreensis	WP_053401622	F-VR-SIAEARIA---SI-DQ-V-E		-K----S-----L------I-
	Bacillus korlensis	WP_066058425	--VR-SFSEA-IA--RGA-EQ-LE-		-K---------SL------I-
	Bacillus kribbensis	WP_026692410	-LVR-NMTEN-IA---VS-EQ-VE-		-K---DSK----L------I-
	Bacillus lentus	WP_066139459	--IF--IAEV--T---HA-EH--E-		-N----------L------N-
	Bacillus licheniformis	WP_120155685	-YVR-IIAEA-IS--RNA-EQ-LG-		-K-D--------L------I-
	Bacillus litoralis	WP_066337576	--VR-SIAEA-IA--NHA-EQ-LE-		-K-D---T----L------I-
	Bacillus loiseleuriae	WP_049680852	--VF-NITQN-IA--RNS-EQ-LE-		-K----------L-----DI-
	Bacillus luti	WP_071713258	F-VR-SIAEA-I---ANE-KR-L-E		-N----------L------I-
	Bacillus manliponensis	WP_034635662	F-VR-SIAEA-I---ANE-KR-LE-		-N----------L------I-
	Bacillus marisflavi	WP_121619631	--IRRSIAEA-IA--RGT-EQ-LE-		-K-D--------L------N-
	Bacillus massiliglaciei	WP_110929487	--VH-SMFEN-IA---GS-EQ--E-		-K-G--------L------I-
	Bacillus massilioanorexius	WP_019242230	--VF--LSEN-IA---NE-ER--E-		GI--S-------LI-----I-
	Bacillus massiliogorillae	WP_042348818	--VR-SIAEN-IA---NE-ER--E-		GK--S-------L-----DI-
	Bacillus massilionigeriensis	WP_075980855	--VR-SFSEA-IA---NS-EL-LE-		-K---DS-----L------I-
	Bacillus mediterraneensis	WP_071459515	---R-S-AEA-IA---NA-EQ-LE-		-K-D-D-----SL------I-
	Bacillus megaterium	WP_043976549	F-VC-SIAEA-IA---SS-EQ---E		GT----------L------I-
	Bacillus mesonae	WP_066396304	--VH-SIAEA--T---NA-EQ--E-		-K-D-D------L------I-
	Bacillus methanolicus	WP_003350106	-LIR-SIAEA-IT---NA-EQ-LE-		-R-Q-D------L------I-
	Bacillus mojavensis subgroup	WP_010334309	-YVR-TIAEA-IA--RGA-EQ-LE-		-K-D--------L------I-
	Bacillus muralis	WP_057913343	--IH-SKFES-IA---GS-EH-LE-		-K----------L------I-

Other Bacteria (0/>300)

Bacillus mycoides	WP_128281261	F-VR-SIAEA-I---ANE-KR-L-E	-N-D--------L------I-
Bacillus nakamurai	WP_061520590	-YVR-TIAEA-IA--RGA-EQ-LE-	-K-D--------L------I-
Bacillus ndiopicus	WP_042472420	--------DS-IT--THT-RS-VEE	-K---D------L--------
Bacillus nealsonii	WP_016201202	--IR-SNA-A-ISN-TMT-EQ--EN	-K---D------L------I-
Bacillus niacini	WP_045515127	--VR-SIAEA-IA---NA-EQ-LE-	-K-D-DS-----L------I-
Bacillus niameyensis	WP_062105010	--IFNY-TKV-IA---NS-EQ-LES	-K-D--------L------N-
Bacillus notoginsengisoli	WP_118922504	--IR-SFAEKQ-A---SE-AQ-L--	-K-D---S----L------I-
Bacillus novalis	WP_066085448	--IR-SIAEA-IA---NA-EQ-LE-	-K-D-D------L------I-
Bacillus oceanisediminis	WP_110065618	--VR-SIAEA-IA---DA-EQ-LE-	-K-----S----L------I-
Bacillus oleivorans	WP_097160450	--LR-NIYEA--T---NA-VQ-LE-	-K-----I---SL------I-
Bacillus onubensis	WP_099355306	FYVR-SIAET-IA---SV-EQ--E-	-N---DS-----L------I-
Bacillus oryziterrae	WP_017753831	--GR-TIAEA-ISS-EHA-EQ-LE-	-K-----C----L------N-
Bacillus panaciterrae	WP_028399054	F-VR-SIAEA-I---T-A-NQ--E-	-K-QG--S-R-VL------I-
Bacillus persicus	WP_090742134	---R-S-AEA-IA--RNA-EQ-LE-	-K-D--------L-----DI-
Bacillus psychrosaccharolyticu	WP_040376538	--IF-TTFEN-IT---GS-ET-L--	-K-----T----L------I-
Bacillus pumilus	OLP66366	--VR-TIAEA-IS--RNM-EQ-VE-	-K-D--------L------I-
Bacillus pumilus	WP_099680619	--VR-TIAEA-IS--RNM-EQ-VE-	-K-D--------L------I-
Bacillus rubiinfantis	WP_042356199	--VH-SIAEA--A---NA-EQ----	-K-D-D------L------I-
Bacillus salsus	WP_090850140	F-VR-SIAET-IA---GA-EQ----	-N---DS-----L------I-
Bacillus selenatarsenatis	WP_041966663	---R-S-AEA-IA---NA-EQ-VE-	-K-----M---SL------N-
Bacillus simplex	WP_096338182	--IH-SKFENRIA---GS-EH-LE-	-K----------L------I-
Bacillus sinesaloumensis	WP_077617894	FYVR-SIAET-IA---SV-EQ--E-	-N---DS-----L------I-
Bacillus smithii	WP_003352409	--VR-SIAEH-IA---SI-DQ--E-	-K----SV---SL------I-
Bacillus soli	WP_066068755	--VH-SIADA--A---NA-EQ-LE-	-K-D-DS-----L------I-
Bacillus sonorensis	WP_006638098	-YVR-IIAEA-IS--RNA-EQ-LG-	-K-D--------L------I-
Bacillus sp. 123MFChir2	WP_020059067	F-VR-SIAEA-I---ANE-KR-VE-	-N-Q--------L------I-
Bacillus sp. 1NLA3E	WP_015593185	--IR-SIAEA-IT---DA-EQ-LE-	-K----------L------I-
Bacillus sp. 2_A_57_CT2	EFV79329	--VR-SIAEA-IA---DA-EQ-LE-	-K-----T----L------I-
Bacillus sp. 7586-K	WP_095297671	--VR-SIAEA-IA--NHA-EQ-LE-	-K-D---T----L------I-
Bacillus sp. 7705b	PAY13037	-YVR-TIAEA-IA--RGA-EQ-LE-	-K-D--------L------I-
Bacillus sp. 7884-1	WP_095249335	--VR-SIAEA-IA---NA-EQ-LE-	-K-D-DS-----L------I-
Bacillus sp. 7894-2	WP_095242414	--VR-SIAEA-IA---DA-EQ-LE-	-K-----T----L------I-
Bacillus sp. AFS006103	WP_098261215	--VH-SIAEA--A---NA-EQ-LE-	-K-D-DS-----L------I-
Bacillus sp. AFS015802	WP_098351712	-LIR-SIAEA-IA---GS-EQ-LE-	-K---D------L------N-
Bacillus sp. AFS015896	WP_098356865	F-VR-SIAEA-I---ANE-KR-L-E	-N----------L------I-
Bacillus sp. AFS018417	WP_098309730	F-VR-SIAEA-I---ANE-KR-VE-	-N-Q--------L------I-
Bacillus sp. AFS073361	WP_098571727	--VH-SIAEA--A---NA-EQ-LE-	-K-D-DS-----L------I-
Bacillus sp. B-jedd	WP_048824525	--IR-SFAEKQ-A---SE-AL-L--	-K-D---S----L------I-
Bacillus sp. CGMCC 1.16541	WP_110112761	F-VR-SIAEA-IA---TV-DQ-V-E	-N---D------L------I-
Bacillus sp. CHD6a	WP_060666480	FYVR-SIAEQ-IA--RSV-DQ-VE-	SR----S-----L------I-
Bacillus sp. FJAT-14578	WP_028394480	F-GR-SIAEA-IA---SA-EQ---E	-N---D------L------I-
Bacillus sp. FJAT-25496	WP_057769952	--VS-SIAQA-IA---DS-DQ-LE-	-N----------L------I-
Bacillus sp. FJAT-27245	WP_053366237	--IR-SFAEKQ-A---SE-AL-L--	-K-D---S----L------I-
Bacillus sp. FJAT-27251	WP_053362191	---R-S-AEA-IA---NA-EQ-LE-	-K-D--------L-----DI-
Bacillus sp. FJAT-27916	WP_049671363	-LLR-SIAES-IS--TNA-EK--E-	-K---D-----SL------I-
Bacillus sp. FJAT-27986	WP_066100258	-LLRRSIAES-IS--TNA-EK--E-	-K---D-----SL------I-
Bacillus sp. FJAT-29814	WP_066320734	--IR-SIAEA-IA---NA-ES-LE-	-K-D-DS-----L------I-
Bacillus sp. FJAT-29937	WP_066288780	--VC-SIAQA-IA---DS-EQ-LE-	SK---D------L------I-
Bacillus sp. FJAT-42315	WP_100403010	--VR-SIAES-IA---GA-EQ--E-	-N----------L------I-
Bacillus sp. FJAT-42376	WP_123916654	--VR-SIAEA-IA---SA-EQ-LE-	GK-----A----M------I-
Bacillus sp. FJAT-46582	WP_100330978	--VR-SIAES-IA---SA-EQ----	-K----------L------I-
Bacillus sp. HMF5848	WP_125906101	F-VR-SIAETRIA---SA-EQ-LE-	-K---DS-----L------I-
Bacillus sp. HMSC76G11	WP_070877321	--VR-SIAEA-IA---GT-EQ-LE-	-K-D---T----L-----DI-
Bacillus sp. HNG	WP_116350513	FYVR-SIAET-IA---SV-EQ--E-	-N---DS-----L------I-
Bacillus sp. J33	WP_026585461	--VR-SIAEA-IA---DA-EQ-LE-	-K-----T----L------I-
Bacillus sp. LF1	WP_090629941	--VH-SIADA--T---NA-EQ-LE-	-K-D-DS-----L------I-
Bacillus sp. M6-12	WP_101593992	--VQ-SISSN-IA---SS-EQ-LE-	-K-----V----L------I-
Bacillus sp. MBGLi97	WP_103748761	--VR-TIAEA-IA--RGA-EQ-LE-	-K-D--------L------I-
Bacillus sp. MRMR6	WP_075685566	--VR-SIAEA-IA---NA-EQ-LE-	-K-D-DS-----L------I-
Bacillus sp. MUM 116	WP_071357311	--IR-STAET-IA---SA-EQ-L--	-K-D--------L------I-
Bacillus sp. Marseille-P3661	WP_102346870	--VR-SIAEA-ITS-EHA--Q--E-	-K-----S----L------N-
Bacillus sp. NRRL B-14911	EAR63733	--IR-SIAEA-IA---NA-EQ-LE-	-R---D-S----L------I-
Bacillus sp. OK048	WP_090757982	--VR-SIAEA-IA---NA-EQ-LE-	-K-D-DS-----L------I-
Bacillus sp. OK085	WP_132086168	--VR-SIAEA-IA---NA-EQ-LE-	-K-D-DS-----L------I-
Bacillus sp. OxB-1	WP_041074191	--VN----ES--T----S-EQ-VEE	-K-----M----L--------
Bacillus sp. P14.5	WP_113929961	--IR-SIAEA-IA---GS-EQ-LE-	-K---------KL------N-
Bacillus sp. PK3_68	WP_120037374	--VR-SIAES-IA---SA-EQ----	-K----S-----L------I-
Bacillus sp. SA1-12	WP_046514852	--VR-SIAEA-IA--NHA-EQ-LE-	-K-D---T----L------I-
Bacillus sp. SG-1	WP_044023060	--IR-SIAEA-IA---GS-EQ-LE-	-K---------KL------N-
Bacillus sp. SJS	WP_035411728	--VR-SIAEA-IA---SA-EQ-LE-	GK-----A----M------I-
Bacillus sp. SKP7-4	WP_119548944	--IR-SIAEA-IA---GS-EQ-LE-	-K---------KL------N-
Bacillus sp. SUBG0010	WP_128822428	-YVR-IIAEA-IS--RNA-EQ-LG-	-K-D--------L------I-
Bacillus sp. SYSU K30001	WP_124563706	F-VR-SIAEA-I---T-A-NQ-LE-	-K-Q--------L------I-
Bacillus sp. T33-2	WP_101583246	-LIR-SIAEA-IA---NA--Q-LE-	GK-D--------L------I-

Other Bacteria (0/>300)

Bacillus sp. UMB0899	WP_102229762	--VR-SIAEA-IA--THA-EQ-LE-	-K-D---T----L------I-
Bacillus sp. UNC41MFS5	WP_026566503	--VH-SIAEA--A---NA-EQ-LE-	-K-D-DS-----L------I-
Bacillus sp. V3-13	WP_101659912	--IR-SFAEA-IA---NA--Q-LE-	-K-D---S----L------I-
Bacillus sp. V44-8	WP_117323847	--VS-S-SQN-IA---NS-EQ--E-	-K-----A----L------I-
Bacillus sp. V47-23a	WP_117325040	--VQRSIS-N-IA---SS-EQ-LE-	-K-----V----L------I-
Bacillus sp. V5-8f	WP_101633007	--VS-T-SQN-IA---NS-EQ--E-	-K-----A----L------I-
Bacillus sp. V59.32b	WP_117306506	--VS-N-SQN-IA---NS-EH-LA-	-K-----A----L------I-
Bacillus sp. VT-16-64	WP_077111667	--IF-TFAQA-IT--QGS-TQ-LE-	-K----S-----L------N-
Bacillus sp. WBUNB004	WP_017656591	F-VR-SIAEA-I---ANE-RR-L-E	-N-D--------L------I-
Bacillus sp. X1(2014)	WP_038541253	--IRRSIAEA-IA--RNA-EQ-LE-	-K-D--S-----L------I-
Bacillus sp. Y1	WP_119707609	--VR-SFSEA-IA--RGA-EQ-LE-	-K---------SL------I-
Bacillus sp. es.034	WP_098440718	-LIR-SIAEA-IA---GS-EQ-LE-	-K---D------L------N-
Bacillus sp. m3-13	WP_087942037	FYVR-SIAEQ-IA--RSV-DQ-VE-	SR----S-----L------I-
Bacillus sp. mrc49	WP_100534144	--IH-SKFES-IA---GS-EH-LE-	-K----------L------I-
Bacillus stratosphericus	WP_052320345	--VR-TIAEA-IS--RNM-EQ-VE-	-K-D--------L------I-
Bacillus subterraneus	WP_125479365	---R-S-AEA-IA---NA-DQ-L--	-K-----M----L------N-
Bacillus subtilis	WP_042975808	--VR-TIAEA-IA--RGA-EQ-LE-	-K-D--------L------I-
Bacillus swezeyi	WP_076760588	-YVR-IIAEA-IS--RHA-EQ-LG-	-K-D--------L------I-
Bacillus taeanensis	WP_113805646	-LVR-SIAEA-ISS-EHA--Q--E-	GK----------L--------
Bacillus terrae	WP_120116176	--IF-S-AQA-IT--QGS-EQ-LEE	-K-T--S-----L------N-
Bacillus testis	WP_050614849	--VR-SFSEN-IA---NE-ER--E-	GK--S-------LI----DI-
Bacillus thuringiensis	WP_071729824	F-VR-SIAEA-I---ANE-KR-L-E	-N----------L------I-
Bacillus timonensis	WP_010282273	FYVR-SIAET-IA---SV-EQ-VE-	-N---DSI----L------I-
Bacillus toyonensis	WP_097953974	F-VR-SIAEA-I---ANE-KR-L-E	-N-D--------L------I-
Bacillus tuaregi	WP_071395269	--VR-SIAEA-IA--RNA-EQ-VE-	-K-D-DS-----M------I-
Bacillus velezensis	WP_099762529	-YVR-TIAEA-IA--RGA-EQ-LE-	-K-D--------L------I-
Bacillus vietnamensis	WP_060669609	-LIR-SIAEA-IA---GS-EQ-LE-	-K---D------L------N-
Bacillus vireti	WP_024028275	--IR-SIAEA-IA---NA-EQ-LE-	-K-D-D------L------I-
Bacillus wiedmannii	WP_098093386	F-VR-SIAEA-I---ANE-KR---E	-N----------L------I-
Bacillus zeae	WP_119111639	---R-S-AEA-IA---NA-EQ-LE-	-K-D-D-----SL------I-
Bhargavaea beijingensis	WP_092095330	F-AL-Q--DS--T---QS-ES-VEE	GR----------L--------
Bhargavaea cecembensis	WP_008297107	--AL-Q--DS--T---QS-ES-VE-	GR----------L--------
Bhargavaea ginsengi	WP_092048857	--AL-Q--DS-MT---QS-ES-VE-	GR----------L--------
Butyricicoccus sp. 1XD8-22	RKJ45014	--------ES------G--EL-V-E	-K----------L--------
Caenibacillus caldisaponilytic	WP_077615807	--VR-SIAEARIAT-EQ--RQ-VEE	-R-----V----L------I-
Caryophanon latum	WP_083995284	---V-Q--ES------FI-DQ-V-E	-K---D------L--------
Caryophanon tenue	WP_083998228	---V-Q--ES------FV-EQ-V-E	-K----------L--------
Chryseomicrobium excrementi	WP_100352694	-W-L--L-DS--T---QSSEL-LEE	-K----------L------V-
Domibacillus antri	WP_075398071	--VR-SIAES-IA---NA-EQ-V--	-K-----S----L------I-
Domibacillus enclensis	WP_045849972	--VR-SIAES-IA--RSA-DQ-VE-	-K-----S----L------I-
Domibacillus epiphyticus	WP_076763142	--VR-SIAES-IA---NA-DQ-V--	-K-----S----L------I-
Domibacillus indicus	WP_046174987	--VR-SIAES-IA--RSA-DQ-VE-	-K-----S----L------I-
Domibacillus iocasae	WP_069939331	--VR-SIAES-IA--RSA-EQ-VE-	-K-----S----L------I-
Domibacillus robiginosus	WP_050181381	--VR-SIAES-IA--RSA-DQ-VE-	-K-----S----L------I-
Domibacillus tundrae	WP_046179253	--VR-SIAES-IA--RSA-DQ-VE-	-K-----S----L------I-
Edaphobacillus lindanitolerans	WP_076757447	--AL-N--DS--T---QS-AL-VEE	GR----------L--------
Falsibacillus pallidus	WP_114743606	-LIR-SIAEA-IS---NA-EQ-VE-	-K----S-----L------N-
Falsibacillus sp. GY 10110	WP_121682580	--IR-SIAEARIT---NA-EQ-VE-	-K----S-----L------N-
Filibacter sp. TB-66	WP_124070857	--VNR---DS--T---HS-EQ-VE-	-K-----M----L--------
Isobaculum melis	WP_092650738	-MVR-STHEKEIA---NT-T--L--	-RK-S-T-----L------N-
Jeotgalibacillus soli	WP_041089185	-MIH-FMTKA-L----GSVEQ--EN	GK----------L------N-
Klebsiella pneumoniae	OON57291	F-VR-SIAEA-I---ANE-KR-L-E	-N----------L------I-
Kurthia huakuii	WP_029498320	--A-----DS--T---ST-EM--E-	GK----------L--------
Kurthia massiliensis	WP_026021969	--A-----DS--S---SS-EM--E-	GK----------L--------
Kurthia senegalensis	WP_010301328	--V-----DS--T---SS-EM--E-	GK----------L--------
Kurthia sibirica	WP_109304583	-VII-L---S--T---SS-KL-VEE	GE--------G------E---
Kurthia sp. 3B1D	WP_126990726	--V-----DS--T---ST-EM--E-	GK----------L--------
Kurthia sp. Dielmo	WP_020189512	--V-----DS--T---ST-EM--E-	GK----------L--------
Lysinibacillus acetophenoni	WP_097149179	--------ESN-----HT-SL---E	-K-D--T-----L--------
Lysinibacillus boronitolerans	WP_036080352	-I------DS-IT---HV-ET-VEE	GK-----M----L--------
Lysinibacillus chungkukjangi	WP_107933126	-L------ES------NS-EL---E	-K----------L--------
Lysinibacillus composti	WP_124766106	--------ES------HS-EL----	-K----------L--------
Lysinibacillus contaminans	WP_053584262	-I------DS-IT---NV-ET-V-E	GK----------L--------
Lysinibacillus endophyticus	WP_121215006	--------ES------HASEL---E	-K----------L--------
Lysinibacillus halotolerans	WP_122970503	--------ES------HS-EL---E	-K----------L--------
Lysinibacillus macroides	WP_053993993	-I------DS-IT---HV-ET-VEE	GK-Q---M----L--------
Lysinibacillus manganicus	WP_036189196	-L------ES------HT-SL-V-E	-K-D--T-----L--------
Lysinibacillus massiliensis	WP_036174384	--------ES-----RSS-EL-L-E	-K----------L--------
Lysinibacillus meyeri	WP_107840784	--------DS-IT--THT--S-VEE	-K---D------L--------
Lysinibacillus odysseyi	WP_036152563	-----Q--ES--T---H--AT-VEE	-K---D------L--------
Lysinibacillus parviboronicapi	WP_107923023	-I------DS-IT---HV-ET-VEE	GK----------L--------
Lysinibacillus sinduriensis	WP_036204102	--------ES------NS-EL---E	-K----------L--------
Lysinibacillus sp. 2017	WP_108714424	-----Q--ES--T---HV-SQ-V-E	-K---D------L--------
Lysinibacillus sp. B2A1	AVK83243	-I------DS-IT---HV-ET-VEE	GK----------L--------

Other Bacteria (0/>300)

Lysinibacillus sp. BK089	WP_132357371	-I------DS-IT---HV-ET-VEE	GK----------L--------
Lysinibacillus sp. FJAT-14222	WP_053594275	-I------DS-IT---HV-ET-VEE	GK----------L--------
Lysinibacillus sp. FJAT-14745	WP_053483231	-I------DS-IT---HV-ET-VEE	GK----------L--------
Lysinibacillus sp. Marseille-P	WP_106784335	--------ES------S--EL-V-E	-K----------L--------
Lysinibacillus sp. SYSU K30002	WP_126658034	--------ES------HASEL---E	-K----------L--------
Lysinibacillus sp. YLB-03	WP_118875191	--------ES------HSSML---E	-K----------L--------
Lysinibacillus sp. ZYM-1	WP_054610384	-I------DS-IT---HV-ET-VEE	GK----------L--------
Lysinibacillus sphaericus	WP_010858523	-I------DS-IT---HV-ET-VEE	GK----------L--------
Lysinibacillus sphaericus	WP_012293332	-I------DS-IT---HV-ET-VEE	GK----------L--------
Lysinibacillus sphaericus	WP_069510052	-I------DS-IT---HV-ET-VEE	GK----------L--------
Lysinibacillus sphaericus	WP_075528360	--VNR---ES--T---HS-E---EE	-K-----M-R--L--------
Lysinibacillus telephonicus	WP_126293030	--------ES------NS-EL---E	-K----------L--------
Lysinibacillus xylanilyticus	WP_049668515	FI------DS-IT---HV-ET-VEE	GK----------L--------
Lysinibacillus xyleni	WP_097072365	--------ES------HASEL---E	-K----------L--------
Massilibacterium senegalense	WP_062197928	--IR--IAEA-ISS-EFA--Q-VEE	-K-NS------KL---R--I-
Paenibacillus baekrokdamisoli	WP_125656234	--IR-SIAEA-ISS-EQA-SQ-V-N	-RK--------TV------V-
Paenibacillus kobensis	WP_127531705	--IR-SIAEA-IQS-EQA-TV-V-N	-KK--------TV------V-
Paenibacillus larvae	WP_036654059	--IR-SLAEA-ISS-EFA--Q-MES	-KK--------TV------V-
Paenibacillus montanisoli	WP_112883090	--IR-SIAEA-ISS-EQA-SQ-V-N	-RK--------TV------V-
Paenibacillus sp. 18JY67-1	WP_126016474	--IR-SIAEA-ISS-EQA-SQ---N	-RK--------TV------V-
Paenibacillus sp. BK736	WP_132480154	--IR-SIAEA-ISS-EQA-SQ-V-N	-RK--------TV------V-
Paenibacillus sp. OV219	WP_090574259	--IR-SIAEA-ISS-EQA-SQ---N	-RK--------TV------V-
Paenibacillus taihuensis	WP_116187214	--IR-SIAEA-ISS-EQA-SQ---N	-RK--------TV------V-
Paenibacillus validus	WP_127604450	--IR-SLAEA-ISS-EHA--Q---N	-RK--------TV------I-
Paenibacillus xerothermodurans	WP_089198152	--IR-SIAEA-ISS-EHA--Q-KES	-SK---------V------I-
Paenibacillus xylaniclasticus	WP_127568532	--IR-SIAEA-IQS-EHA-TV---S	-KK--------TV------V-
Paenisporosarcina indica	WP_075617590	-YVI-N--DS--T---QS-VL-V-E	-K----------L------I-
Paenisporosarcina quisquiliaru	WP_090562388	-I-L-N--DS--T---QSSAI---E	-K----------L--------
Paenisporosarcina sp. HGH0030	WP_016427567	-ILI-N--DS--T---QS-VL-V-E	-K----------L--------
Paenisporosarcina sp. K2R23-3	WP_119883046	--VV-S--DS--T---QS--T-L--	-K---------VL--------
Paenisporosarcina sp. OV554	WP_108584706	--FI-N--DS--T---QS-VL-V-E	-K----------L--------
Paenisporosarcina sp. TG20	WP_019414860	--LI-N--DS--T---QS-VL-V-E	-K----------L--------
Planococcus antarcticus	WP_006828217	--AL--A-DSNIA---NS-KQ-VE-	-K----------L------N-
Planococcus citreus	WP_121297632	--AL--A-DS-MA---HS-EQVVE-	-K----------L------N-
Planococcus donghaensis	WP_065526001	--AL--A-DSNIA---NS-EQ-VE-	-K----------L------N-
Planococcus halocryophilus	WP_065528084	--AL--A-DSNIA---NS-EQ-VE-	-K----------L------N-
Planococcus maritimus	WP_068462777	--AL--A-DS-MA---HS-EQVVE-	-K----------L------N-
Planococcus massiliensis	WP_052651314	-VVL--A-DSNIA---NS-EQ--E-	-K----------L------N-
Planococcus salinarum	TAA72473	--V---S-ES-MA--RNS-EQV-E-	-K----------L------N-
Planococcus salinus	WP_123164269	-YAL-R--DS-MA---GS-EQ--E-	-K----------L------N-
Planococcus sp. CAU13	WP_033544075	--V---S-ES-MA--RNS-EQ--E-	-K----------L------N-
Planococcus sp. PAMC 21323	WP_038704220	--AL--A-DSNIA---NS-EQ-VE-	-K-----Q----L------N-
Planococcus sp. Y42	WP_077590983	-WAL----DSN-A--RSV-NQ-LE-	-N-Q--------L------N-
Planococcus versutus	WP_049693241	--V---S-ES-MA--RNS-EQ--E-	-K-----Q----L------N-
Planomicrobium flavidum	WP_088005988	-VVL----DSN-A--RSV-EQ-LE-	-K-Q--------L------N-
Planomicrobium glaciei	WP_036806568	-YAL--A-DSNIA---NS-EQ-VE-	-KK---------L------N-
Planomicrobium okeanokoites	WP_084245362	--V---S-ES-MA--RNS-EQV-E-	-K----------L------N-
Planomicrobium soli	WP_106531858	--AL--S-DS--A---NS-EQ--E-	-K----------L------N-
Planomicrobium sp. MB-3u-38	WP_101802859	--V---S-ES-MA--RNS-EQV-E-	-KK---------L------N-
Psychrobacillus insolitus	WP_111438419	-N-L----DS--T---QSSAL-LEE	-K----S-----L--------
Psychrobacillus psychrodurans	WP_093494503	-I-L-N--DS--T---QSSAS---E	-K----------L--------
Psychrobacillus psychrotoleran	WP_093535027	-I-L-N--DS--T---QSSANL--E	-K----------L------A-
Psychrobacillus sp. OK028	WP_093060205	-I-L-N--DS--T---QSSAI---E	-K----------L--------
Psychrobacillus sp. OK032	WP_093262950	-I-L-N--DS--T---QSSAL-L-E	-K----------L--------
Pueribacillus theae	WP_116553075	-LGR-SIAEA-ISS-EHA--Q--E-	GK-----S----L--------
Quasibacillus thermotolerans	WP_039232569	--VR-SIAES-IA---SA-EQ----	-K----SS----L------I-
Rhodococcus qingshengii	WP_133369391	--VR-SIAEA-IA---NA-EQ-LE-	-K-D-DS-----L------I-
Rummeliibacillus pycnus	WP_102694361	--V-----DS--T---NS-EL--EE	GK----------L--------
Rummeliibacillus sp. POC4	WP_119414375	--V-----ES------SA-EL--EE	GK----------L--------
Rummeliibacillus sp. TYF005	WP_124217362	--V-----ES------SA-EL--EE	GK----------L--------
Rummeliibacillus stabekisii	WP_066785809	--IT----DS--T---GT-EL-VQE	GK----------L--------
Salibacterium halotolerans	WP_093339682	--VR-STAEA-IAS-EQASRQ----	-H-Q---D----V------A-
Solibacillus isronensis	WP_079525364	-----N--ES--T---HV-EQ-VE-	-K---D------L--------
Solibacillus silvestris	WP_014824446	-----N--DS--T---HV-EQ-VE-	-K---D------L--------
Solibacillus sp. R5-41	WP_099425751	--------ES--T---HV-EQ-VEE	-K---D------L--------
Sporolactobacillus inulinus	WP_039744192	-YIR-NIAEA-ITS-ETA-HQ-VE-	-H-D--S-----L------I-
Sporolactobacillus laevolactic	WP_031263588	-YIR--IAEA-ITS-EVA-RQ-VE-	-H-D--------L------I-
Sporolactobacillus pectinivora	WP_100487742	-YFR--IAEA-IAS-ETA-HQ-VE-	-H-D-------SL----E-I-
Sporolactobacillus sp. THM7-4	WP_130030570	-YIR--IAEA-IVS-ETA-RQ-V--	-H-D--------L------I-
Sporolactobacillus sp. THM7-7	WP_130000335	-YIR--IAEA-IAS-ETA-RQ-V--	-K-N--------L------I-
Sporosarcina globispora	WP_053436404	--VR-SIAEA-IA---DA-EQ-LE-	-K-----S----L------I-
Sporosarcina koreensis	WP_040286798	FV-N-N--ES--T---HS-ET-VEE	-K----------L------N-
Sporosarcina newyorkensis	WP_078817724	F-IL-N--DS--T---QT-NQ-VEE	-K----SV----L--------
Sporosarcina pasteurii	WP_115360521	--VNR---ES--T---HS-E--VEE	-K-----M-R--L--------

**Other Bacteria (0/>300)**	Sporosarcina psychrophila	WP_067211083	--FN----DS--S---FT-EQ-VEE	-K---D------L--------
	Sporosarcina sp. BI001-red	WP_116016473	-L-N-N--ES--T---HS-AT-VEE	-K----------L------N-
	Sporosarcina sp. D27	WP_025784980	FV-N-N--ES--T---HS-AT-VEE	-K----------L------N-
	Sporosarcina sp. EUR3 2.2.2	WP_024534959	--FI-N--DS--T---QS-VL-V-E	-K----------L--------
	Sporosarcina sp. HY008	WP_067408221	--VI----DS--K---DS-EY-VEE	-K-----M----L--------
	Sporosarcina sp. P13	WP_099687051	F-IL-N--DS--T---QT-NQ-VEE	-K----SV----L--------
	Sporosarcina sp. P34	WP_099694207	F-IL-N--DS--T---HT-NQLVEE	-K----SV----L--------
	Sporosarcina sp. PTS2304	WP_114923389	F-IL-N--DS--T---QT-NQ-VEE	-K----SV----L--------
	Streptococcus pneumoniae	CKF51546	F-VR-SIAEA-I---ANE-KR---E	-N----------L------I-
	Tetzosporium hominis	WP_094941693	-W-L--L-DS--T---QSSEL-LEE	-K----------L------V-
	Trichococcus ilyis	WP_068620706	-S-R-SNHEKEIA---NT-SQ-L--	-RK---T-----M------N-
	Ureibacillus thermophilus	QBK25702	--------ES------HS-EL---E	-K-Q--------L--------
	Ureibacillus thermosphaericus	WP_016836829	--------ES------Q-SLL---E	-K----------L--------
	Vibrio vulnificus	WP_133348168	--IH-SKFESRIA---GS-EH-LE-	-K----------L------I-
	Viridibacillus arvi	WP_053417409	-YVN-R--DS--T---SS-EL---E	GK----------L--------
	Viridibacillus sp. FSL H7-0596	WP_076033994	-YVN-R--DS--T---SS-EI---E	GK----------L--------
	Viridibacillus sp. OK051	WP_100796547	-YV------S--T---SS-EL---E	GK----------L--------

Supplemental Figure 32

A partial sequence alignment of the Ribonuclease Y protein containing a four amino acid insertion (boxed) that is exclusively shared by all members belonging to the Jejuensis clade and absent in all other bacteria.

			55		111
Jejuensis Clade (3/3)	Lysinibacillus saudimassiliensis	CEA02597	LIDQQERNKQLTAEVHDLTNAVRDY		EKKVADTDYEQVVEEADDLRLMLGNISTKGKG
	Lysinibacillus sp. BF-4	WP_051910113	-----------A----E--T-----		-------------------------T-------
	Lysinibacillus jejuensis	WP_108305705	------------T-L-EM-V-I---		---MV-A---KL-AQ------L--S--A----
Other Bacteria (0/>100)	Anoxybacillus sp. B2M1	ANB56120	--QS-KE-QK-K--LMSKQEK--KM	EA	SLADKKKA-SDLAT--EQ--MYI-KVKVR-A-
	Anoxybacillus sp. P3H1B	KXG10461	--QS-KE-QK-K--LMSKQEK--KM	EA	SLADKKKA-SDLAT--EQ--MYI-KVKVR-A-
	Anoxybacillus vitaminiphilus	WP_111643633	I-E---K-RK-HE-LVKKQQK-QKM	EK	-LADEEQI-YNL--D-EK--MH--KVTVQ-Q-
	Bacillus aidingensis	WP_084413973	VLEE--K-YE--E-WRS-QSR--EM	EE	TLAENEASSFNL--DLEK--MLT-EVPV--P-
	Bacillus alkalitelluris	WP_088075795	ILME-MV-RS--E-LR-IQ-QLTEF	EE	-IVNQQ-R-FNL--DL-R--MIT--VGVV-E-
	Bacillus alveayuensis	WP_044893852	I-E---K-QK-HE-LVQKQQKI-EI	EK	-LANQEQI-YNL--D-EK--MY--KVKVR-Q-
	Bacillus atrophaeus	WP_061571500	-LK-EKQ--E-KK-LYEKQSK--QT	EN	KL-KEK-E-YN-L-DVEKY-MYV-EVGV--E-
	Bacillus campisalis	WP_046522224	--E-E-L-RTMQQ-L-EKQEM--EF	EA	DLANEAQV-FNLA-D-EKY-MH--KVKV--P-
	Bacillus cecembensis	WP_057985793	--V------E--D-LAE-EKKI-G-	EK	-YSSNEVQ--RNIK--QQ---L--IG-GE---
	Bacillus coahuilensis	WP_081447444	--K-S-LE-QEKLYA-Q-ELK--	ET	-LSKKEDV-FNL--DVNKY-MF--EVDV--P-
	Bacillus cohnii	WP_066417619	I-NTE---RE-QN-LFEKQEI--QI	EE	-IAKEEQLFFNL--DVEK--MYN--VKV--T-
	Bacillus deserti	WP_101645249	-VE-E-KTRK-QK-LFSKQAE-TSM	EK	QISKETEN-SNITK-M-KY-MY--KVKV----
	Bacillus fastidiosus	WP_066232482	--K-E-S-AK-QK-LKQKQEK--EF	EK	-L-DQKQV-FNL--DVEKY-MFV-EVGVS-S-
	Bacillus foraminis	WP_132005890	--E-E-T-RA-QK-LNEKQEM--N-	EA	-LAKEAEI-FNLA-D-EKY-MF--KVKV--N-
	Bacillus halmapalus	WP_078380980	--KTE---LE-QQ-LL-IQEE--KI	EE	-LATGEQLLFNL--DVEN--MYN--VKVQ-Q-
	Bacillus halotolerans	WP_044155974	-LK-EKE--D-EK-LYQKQ-K--QT	EN	KL-NEK-E-YN-L-DVEKY-MYI-EVGVQ-E-
	Bacillus indicus	WP_029285006	--K-E---AE-QKQLS-TQEK--KI	EE	DL-NEKQL-FNL--D-EKY-MYV-ELGV--E-
	Bacillus intestinalis	WP_061187423	-LK-EKE--N-EK-LYQKQ-K--QA	EN	KL-KEK-E-YN-L-DTEKY-MYI-EVGVQ-E-
	Bacillus kochii	WP_095373479	---LE-K-LS-QQ-LYKSQD-LQKI	EK	-FSQDEQV-SNLA-D--KY-MY--KVKVS-P-
	Bacillus koreensis	WP_053403369	----E---RK-QR-LFAKQEK--KT	EE	GLAKKEQ-LAKI-DDVKKY-MYT-KVGV----
	Bacillus marmarensis	WP_022628247	QERN--LQEE-REYQTEVRLLEDEI	AS	-SAEQELRTTNLI-DL-R--KVV--VPV--P-
	Bacillus massiliogabonensis	WP_102271543	-V-LEKK-LT-QK-L-EKQER--EI	EN	-LSQEAQV-FNLA-D-EK--MY--K-GV--Q-
	Bacillus methanolicus	WP_003350304	----EQA-RE-QK-LNQEQEK-LEI	EK	QLAKEAQV-FNLA-D-EKY-MY--KVAV----
	Bacillus mojavensis	WP_010334162	-LK-EKE--D-EK-LYQKQ-K--QT	EN	KL-NEK-E-YN-L-DVEKY-MYI-EVGVQ-E-
	Bacillus ndiopicus	WP_042471701	--T------E-AD-LNH-QQTI-K-	EK	-LTSNAEQH-DL-A--ER---L--D-AGQ-Q-
	Bacillus niameyensis	WP_062105157	--SI-D--RD-QK-LYEKQES--QK	EK	-LSQEEET-MNLAKD-EKF-MF--KVKV--P-
	Bacillus panaciterrae	WP_028398920	--E-EKT-AK-QTK-AA-QEN--NE	EK	NIASQQEKLSDT-K-VE---MY--KVKV--P-
	Bacillus persicus	WP_090747457	-NE-R-K-RD-QE-LNKRQEM---F	ET	QLANEAQV-FNLA-D-EKY-MF--KVKV--A-
	Bacillus pseudofirmus	WP_041822901	QERN--LQEE-REYQTEVRLLED-I	AS	-SAEQELRTTNLI-DL-R--KVV--VPV--P-
	Bacillus rubiinfantis	WP_042356330	--E-E-E-RR-QK-LSQKQVE-QLN	EK	-FAKEAQVFSNLA-D--K---Y--KVKV--N-
	Bacillus solimangrovi	WP_069718688	--K-E--TR--QQ-LFEKQEE--EI	ES	-LATKEKGLFNL--DVEK--MFA-D-KVS-P-
	Bacillus sp. B-jedd	WP_048827828	--SLE-A-RS-QK-YNTKQSKI-EI	EK	-LSKEEET-SAIA---EKY-MF--KMKV--P-
	Bacillus sp. FJAT-21945	KOP82049	-V-LEKK-LT-QK-L-EKQEQ--EI	EN	-LSQEAQV-FNLA-D-EK--MY--K-DV--Q-
	Bacillus sp. FJAT-22090	ALC86144	--E-K---NE-SD-INEKQHEIKEF	EQ	SFVNSEENV-EL-D--EM---LI-D-PSE---
	Bacillus sp. FJAT-27225	WP_077247753	--SLE-T-RE-QK-FNSKQAQ-LE-	EK	ALSKEEQTFSKIA---EKY-MF--HVKVQ-P-
	Bacillus sp. FJAT-27251	WP_053362080	--E-EKL-RTMQQ-L-EKQEM--EF	EA	DLANEAQV-FNLA-D-EKY-MY--KVKV--S-
	Bacillus sp. FJAT-45037	WP_100373288	QERN-ILQDE-RNYQSEVRQLEEEL	AE	KNVEQEIRTTNLI-DL-R--KVV--VPV--P-
	Bacillus sp. FMQ74	WP_071576793	-LK-EKE-RK-EK-LYQKQ-K--QA	EN	KL-KEK-E-YN-L-DTEKY-MYI-EVGVQ-E-
	Bacillus sp. OxB-1	BAQ10431	-VN------E-FD-IAAKQEEI-Q-	EQ	TFSKGKENHADL----K----L--V-PAT-S-
	Bacillus sp. OxB-1	WP_052483977	-VN------E-FD-IAAKQEEI-Q-	EQ	TFSKGKENHADL----K----L--V-PAT-S-
	Bacillus sp. P14.5	WP_113929811	--NH--K-SK-QE--FEKQQK-V-I	EK	DLSQEKQI-FNLA-D-EKY-MF--KVKVE-Q-
	Bacillus sp. SJS	WP_035412926	--E-E-K-LK-Q-ALQKKQKN-Q--	EK	KL-KDKQAASGL--QIEN--MYV-ETGV--E-
	Bacillus sp. SYSU K30001	WP_124563806	--R-EKA-AT-EKK-VN-QKK--AE	EQ	KLGDQQKQFSDA-K-VEE--MY--RVKV--A-
	Bacillus sp. TE3	WP_129505775	-LK-EKE--N-EK-LYQKQ-K--QA	EN	KL-KEK-E-YN-L-DTEKY-MYI-EVGVQ-E-
	Bacillus sp. UMB0893	WP_101565670	--Q-E----E-QK-LF-NQER--KI	EE	NL-DEKQI-FNL--DVEKY-MYV-ELGVS-E-
	Bacillus subtilis	WP_003221430	-LK-EKE--N-EK-LYQKQ-K--QA	EN	KL-KEK-E-YN-L-DTEKY-MYI-EVGVQ-E-
	Butyricicoccus sp. 1XD8-22	RKJ09797	--E-----NE--K-LNN-HLKI-E-	EQ	NFASDEKK---L-N--EK---LI-K-PAQ-Q-
	Caldibacillus debilis	WP_120665838	--AEEKK-RE-AE-L-KKQ-E-LQF	EK	KLADEEKVLSGMA---EQ--RY--KVKVS---
	Caryophanon tenue	WP_066543239	--E------A-AE-LRAVQE--FA-	EQ	QLATEARE---N-D--ER--QLI-LLPAE-Q-
	Chryseomicrobium excrementi	WP_100352784	--E-K----E-SE-LR-VQETI-E-	EQ	QAGSNTSSLDEL-K--EA---LT-EV-V--E-
	Falsibacillus sp. GY 10110	WP_121680353	--E--KA-RK-QK-LYAKQSK-L-S	EK	-LSKEEQVFYNLA-D-EKY-MY--KVKV----
	Fictibacillus arsenicus	WP_083207750	ILSL-KT-RE-SN-LKG-QQS-EKK	EN	KFADQEEISSKL---LKE--MVV--VKV--E-
	Fictibacillus gelatini	WP_026675960	IL-V-KT----SN-LKVIQQK-Q-K	EE	NVANEEKNSAGL-K-LKQ--MIV-EVKV--E-
	Fictibacillus phosphorivorans	WP_082820696	ILNL-KT-RE-AV-LQG-QQN-QKK	ES	KLANQEEISSKL--DLNE--MIV--VKV--E-
	Fictibacillus sp. BK138	WP_130295760	ILNL-KT-RE-AN-LKG-QQS-EKK	EN	KFADQEEISSKL---LKE--MVV--VKV--E-
	Filibacter sp. TB-66	WP_124071240	--N------E-SD-IAAKQEKI-K-	EQ	SFSNREEEHAGL----K----L--VVPAM-Q-
	Jeotgalibacillus soli	WP_041088900	-L-K-QE-IA-QDRLYEQQDI--E-	EK	SMSSEEQAFFNLA-D-ENY-MF--K-AVE-P-
	Lysinibacillus acetophenoni	WP_097148097	--R------E-SE-LNE-QQTI--F	EK	TFASNKDEF-ILQQ--EK------E-PAV-Q-
	Lysinibacillus boronitolerans	WP_026023384	--T------E--E-LNN-QEQI-K-	EK	SFASNEK--KKL--Q-E----L--ELKSE---
	Lysinibacillus chungkukjangi	WP_107934057	---------E-AE-LN--Q-RL-E-	EK	SFAENEVE--HLI---EK---L--I-PAQ-G-
	Lysinibacillus composti	WP_124764093	----K----E-MD--NH-QEQI---	EK	SFASNQNE---M-DK-EQI--L--Q-PAV-E-
	Lysinibacillus contaminans	WP_053584443	--E------E-AE-SNR-QEKI-E-	EK	KFASNENE---L----E----L---LTGE---
	Lysinibacillus endophyticus	WP_121212900	--S------E--E-LIA-QTKR-E-	EK	DFAENEEE--HLLK--EN---L--E-PAH-E-
	Lysinibacillus fluoroglycofeni	WP_107942241	--T------E--E-LNY-QQTI-N-	EK	QLTSSAEQH-DL-A--ER---L--D-AGQ-Q-
	Lysinibacillus fusiformis	WP_069480283	--T------E--E-LNS-QEQI-K-	EK	SFASDEK--KKL--Q-E----L--ELKSD---
	Lysinibacillus halotolerans	WP_122970274	--E------E-AE-LN--QDQI---	EK	SIAKNEDQ--DLI-D-EK---Q--L-PV--E-
	Lysinibacillus macroides	WP_053993836	--A------E-SE-LNS-QEQI-K-	EK	SFAADEKA-QKL--Q-E----L--ELKGE---
	Lysinibacillus manganicus	WP_036182115	--K------E-SE-LNE-NQRI---	EK	SFASNQDE-DVLLQ--EK------D-PAV-Q-
	Lysinibacillus mangiferihumi	WP_107895682	--E------E--E-ANM-QEKI-H-	EK	KFASNEK-SKKL-Q--E----L--DLNSE---
	Lysinibacillus massiliensis	WP_036172154	--G------E-ME-LNS-Q-KI---	EK	TFASNEDQ--NL-Q--ER---I--E-PAV-Q-
	Lysinibacillus meyeri	WP_107839248	--T------E-AD-LNH-QQTI-N-	EK	-LTLNTEQHDDL-A--ER---L--D-EGQ-Q-

Other Bacteria (0/>100)

Lysinibacillus odysseyi	WP_036156070	--E------E-AE-LDGILGTI-K-	EK	-YATHEEH-DEM-K--ER--ILM-ELPVT---
Lysinibacillus parviboronicapi	WP_054767104	--E------E--E-ASA-QEKI-K-	EK	QFASSEK--K-L--Q-E----I--DLNSE---
Lysinibacillus sinduriensis	WP_036204077	--H------E-AE-LR--QERL---	ER	SFAKDEDK--HLL---EK------K-PSQ-P-
Lysinibacillus sp. 2017	WP_108714419	--E-K----E-AE-LAN-EEKI-T-	EK	QFSSNEEQ---NIS--EQ---L--YTAGQ---
Lysinibacillus sp. B2A1	AVK83061	--T------V-AE-ANT-QEKI-K-	EK	SFVADEKANKKL----E----L--DLQAE---
Lysinibacillus sp. BK089	WP_132357547	--A------E-SE-ANA-QEKI-K-	EK	SFVASKK--TKL----E----L--DLNSE---
Lysinibacillus sp. FJAT-14222	WP_053594639	--A------E-SE-ANA-QEKI-KH	EK	SFVASEK--TKL----E----L--DLNAE---
Lysinibacillus sp. FJAT-14745	WP_053483391	--V------E--D-ANA-QEKI-K-	EK	SFVASKK--TKL-A--E----L--DLNSE---
Lysinibacillus sp. Marseille-P	WP_106784049	--L------E--E-LNL-Q-KI---	EK	SFASNKDE--LL-QD-EK---L--E-PGV-Q-
Lysinibacillus sp. OL1	WP_131521626	--T------E--E-LNN-QEQI-K-	EK	SFASNEK--KKL--Q-E----L--ELKSE---
Lysinibacillus sp. SYSU K30002	WP_126657650	--S-K----E-IE-LIA-QTKR-E-	EK	TFAENEE--DHLLQ---K---L--E-PAY-E-
Lysinibacillus sp. YLB-03	WP_118874745	--E------E-AE-YYS-Q-KM-E-	EK	TFAENEEE--HLI---ER---V--YLPAQ-E-
Lysinibacillus sp. YR326	WP_134019094	--A------E-SE-ANA-QEKI-K-	EK	SFVASKK--T-L-D--E----L--DLNAE---
Lysinibacillus sp. ZYM-1	WP_054611684	--T------E-SE-LNN-QDQI-K-	EK	SFASNEK--KKL--Q-E----L--ELKSD---
Lysinibacillus sphaericus	POZ57773	--E------E--E-ANM-QEKI-H-	EK	KFASNEK-SKKL-Q--E----L--DLNSE---
Lysinibacillus sphaericus	SUV15935	--E------E--E-ANM-QEKI-H-	EK	KFASNEK-SKNL-Q--E----L--DLDSE---
Lysinibacillus sphaericus	VDG97412	--E-K-------D-ISSKQKTIAEV	EQ	TLSDTEENHDLL-K--KS---L--VVPAS-Q-
Lysinibacillus sphaericus	WP_010858335	--E------E--E-ASA-QEKI-K-	EK	-FASSEKN-K-L--Q-E----L--DLDSE---
Lysinibacillus sphaericus	WP_031418920	--T------E-SD-LNS-QEQI-K-	EK	SFASNEK--KEL--Q-E----L--ELKSE---
Lysinibacillus sphaericus	WP_069510254	--E------E--E-ANM-QEKI-H-	EK	KFASNEK-SKKL-Q--E----L--DLNSE---
Lysinibacillus sphaericus	WP_075528500	--E-----RE-SDDIGVKQEEI-A-	ER	SFLEQEE-HADL----K----L--VVPAM-Q-
Lysinibacillus sphaericus	WP_125102137	--E-K-------D-ISSKQKTIAEV	EQ	TLSDTEENHDLL-K--KS---L--VVPAS-Q-
Lysinibacillus sphaericus C3-4	ACA39040	--T------E-SD-LNS-QEQI-K-	EK	SFASNEK--KEL--Q-E----L--ELKSE---
Lysinibacillus telephonicus	WP_126292868	--E------E--E-LNN-QVRI-EF	EK	SFAENKDE--HLLD--EK---IV-E-PAQ-E-
Lysinibacillus varians	WP_025218754	--E------E--E-ANM-QEKI-H-	EK	KFASNEK-SKNL-Q--E----L--DLDSE---
Lysinibacillus xylanilyticus	WP_100544027	--A------E-SE-ANA-QEKI-K-	EK	SFVASKK--TKL-D--E----L--DLNAE---
Lysinibacillus xyleni	WP_097072050	--S------E--E-LTA-QTKR-E-	EK	NFAENEE--GHLL---EN---L--E-PAH-E-
Paenisporosarcina antarctica	WP_134210145	--H-K----E-EN-LVTRQLE--EI	EK	SLSGSEEQ--SL-G--E----LM-MLPAT-E-
Paenisporosarcina indica	WP_075617475	-VQ-K----E-AE-LEARQLQ--EI	EK	SLSGSEEK--GLIN--E----LI-LLPAT-E-
Paenisporosarcina sp. HGH0030	WP_016427769	--Q-K----E-ET-LVARQQQ--EI	EK	SLSGSEEQ--SLID--E----L--ILPST-E-
Paenisporosarcina sp. K2R23-3	WP_119884472	--E-K----E--D-LEKKQTQ--EI	EQ	QLSGSEES-DAL-K--E----L--T-PVE-E-
Paenisporosarcina sp. OV554	WP_108584527	--H-K----E--T-LEARQQQ--EI	EK	SLSGSEEQ--NLIQ--E----L--LLPAT---
Paenisporosarcina sp. TG-14	WP_017379356	--H-K----E-EN-LLTRQLE--EI	EK	SLSGSEEQ--SLIG--E----LI-MLPAT-E-
Paenisporosarcina sp. TG20	WP_019413885	--G------E-EN-LVTRQQK--EV	EM	SLSGSEEQ-QSLIK--E----L--V-PSI-E-
Parageobacillus thermantarctic	WP_090948866	--QM-KE-R--KN-LVQKQDKIAEI	EK	TLADER-A-TDLAKQ-EK--MYV-ESKV----
Parageobacillus thermoglucosid	WP_064551114	--QI-KE-R--KN-LVQKQDK-AEM	EK	TLADER-A-TDLA---EK--MYV-ESKV----
Planococcus antarcticus	WP_006828832	---------D-RD-LQKKQSE-QE-	ER	SFADGENR-TEYAA--EE--RF--LVPIQ-N-
Planococcus citreus	WP_121297759	--E------E-R--LD-KQK--QEF	ER	SVVDGEEN-TAYAR--EE--RF--LVPV--Q-
Planococcus donghaensis	WP_065525916	--N------A-RD-LQEKQSE-QE-	EK	SFADGENR-TEYAK--EE--RY--LVPVQ-N-
Planococcus faecalis	WP_071153617	---------E-KN-LQEKQSE-QE-	EK	SFADGENR-TEYA---KE--RY--LVPVQ-S-
Planococcus halocryophilus	WP_008498013	--N------A-RD-LQEKQSE-QE-	EK	SFADGENR-TEYAK--EE--RY--VVPVQ-S-
Planococcus halotolerans	WP_112222935	--E------T-RE-MLAKQE-LQEF	ES	AFSAGQE--SEIAA--EK--RYV-LMPVE-E-
Planococcus kocurii	WP_058384872	---------E-KN-LQEKQSE-QE-	EK	SFADGENR-TEYA---KE--RY--LVPVQ-S-
Planococcus maitriensis	WP_112231368	--E------E-RD-LG-KQK--QEF	ER	SVVDGEEN-TEYAR--EE--RF--LVPVE-Q-
Planococcus maritimus	WP_068462964	--E------E-RE-LG-KQET-QKF	ER	SVVDGEEN-TAYAR--EE--RF--LVPV--E-
Planococcus massiliensis	WP_052651102	--E------E-R--IEEKQKS-QQF	EQ	SFADGEEHFADYAK--EA---Y--VLPVE-N-
Planococcus plakortidis	WP_084632825	--E------E-RE-LG-KQK--QEF	ER	SVVDGEEN-TEYAR--EE--RF--LVPVM---
Planococcus rifietoensis	WP_058383512	--E------E-R--LD-KQK--QEF	ER	SVVDGEEN-TAYAR--EE--RF--LVPV--Q-
Planococcus salinarum	TAA68703	--E------T-RE-MQNKQ--LQEF	ES	SFSTEEA--SGMAA--EK--RYI-LMPVE-E-
Planococcus sp. CAU13	WP_033543519	--E-K----T-RE-ILAKQEQLQ--	ES	SFSDGEEIFGEIAS--EE--RYI-LMPVE-E-
Planococcus sp. PAMC 21323	WP_038704321	--V------A-RD-LQAKQSD-QE-	EK	SFADGENR-TEYAK--EE--RY--LVPVQ-S-
Planococcus versutus	WP_049693346	--S------E-RV-LQNKQSN-QEF	EQ	SFADGENR-TEYA---KE--RY--LVPVQ-T-
Planomicrobium glaciei	WP_036806891	--E------S-RE-LQEKQAT-QE-	ED	SFSDGENRFADYT---EA--RYI-ILPI--D-
Planomicrobium okeanokoites	WP_117312478	--E------T-RE-MQSKQDVLQEF	ES	-FSSGQEN-SEIAA--EK--RYV-LMPVE-E-
Planomicrobium soli	WP_106534886	--E------A-RE-LGEKQA--QKF	EQ	SFLEGESHETDFAK--EA--HA--LLPVQ-S-
Planomicrobium sp. MB-3u-38	WP_101801992	--E------T-RE-MQSKQDVLQEF	ES	-FSSGQEN-SEIAA--EK--RYV-LMPVE-E-
Planomicrobium sp. Y74	WP_121632792	--E------T-RE-MLAKQE-LQEF	ES	AFSAGQE--SEIAT--EK--RYV-LMPVE-E-
Psychrobacillus insolitus	WP_111438643	--L-K----E--E-MN-KQQ-I--I	EQ	SLVDSEGNI-GL-N--EM---LI-D-TSQ---
Psychrobacillus psychrotoleran	WP_093535741	-VE-K----E--E-IT-KQQKI-AF	EH	SFVESEESV-EL----EI---LI-D-PSQ---
Psychrobacillus sp. OK028	WP_093059940	-VE-K----E-NE-IIVKQQK---F	EQ	SFVESEESV-DL----EL---LI-D-PSQ---
Psychrobacillus sp. OK032	WP_093263870	----K----E--E-INEKQQGI--L	EQ	SFANSEESA-AL-K--EK---LM-DLPSQ---
Salibacterium halotolerans	WP_093335162	VLEE--K--E--E-WRN-QQQ---M	EE	NLAENE-SSFNL---LKKM-MLT-EVPV----
Salibacterium qingdaonense	WP_090925595	VLEE--K--K-SE-WRN-QQQ--EM	EE	SLAESE-SSFNL---LKKM-MLT-EVPV--E-
Salipaludibacillus agaradhaere	WP_078576148	E--TTRE--EQLREIQSEIKQI	EE	TVSDQEQL-FNL--DM-K--MVT-E-GV--E-
Salipaludibacillus aurantiacus	WP_093047680	VLRE-QV-RE-AG-LKEIQTEIKTV	EE	-ISHRERT-FNL--DL-H--MVT--VGV--E-
Solibacillus isronensis	WP_079525026	--EK-----E-AD-LAQ-EEKI-T-	EK	QFSSNE-Q---NRQD-EQ---L--LTDGM-Q-
Solibacillus kalamii	WP_087615476	-TGK-----E-AD-LAQ-EEKI-M-	EK	QYSSNE-Q---NRQD-EQ---L--LTDGI-Q-
Solibacillus sp. R5-41	WP_099422688	--V------E-SD-LVE-EEKI-T-	EK	-YSSNEVQ--RNIK--EQ---L--IQ-GE---
Sporosarcina koreensis	WP_060209326	--A-K----E-AD-IQTKQEEI--F	EK	SFSENEDNHTVL----K----L--V-PAS-P-
Sporosarcina newyorkensis	WP_009765594	--G------E--D-LLSKQKK-HEF	EQ	DFSSKQK-HAVL-DK-Q----L--V-PA--T-
Sporosarcina pasteurii	WP_115360384	--E-----RE-SDDIGVKQEEI-A-	ER	SFLEQEE-HADL----K----L--VVPAM-Q-
Sporosarcina psychrophila	WP_067214023	--S------E--D-IAAKQEEI-E-	ER	SFSMGEK-HASL-D--KN---L--A-PAI-Q-
Sporosarcina sp. BI001-red	WP_116016194	--E-K------ND-ISSKQKKIAKV	EQ	SLSDSEENHDLL-K--RS---L--VVPSS-Q-
Sporosarcina sp. D27	WP_037593187	--E-K-------D-ISSKQKTIAEV	EQ	TLSDTEENHDLL-K--KN---L--VVPAS-Q-

Other Bacteria (0/>100)

```
Sporosarcina sp. EUR3 2.2.2       WP_024535080  --I-K----E-AT-LEAKQQQ--EI EK SLSGSEEQ-DSLIQ--E----L--ILPAT---
Sporosarcina sp. HYO08            WP_067408267  --G------E-SD-IASKQ-EI-E- EQ SFSTQEK-H-VL----K----L--A-PAV-Q-
Sporosarcina sp. P13              WP_099687227  --T------E--N-LLEKQRKIQEF EE NFSNQEENHAVL--K-R----L--VMPAT-A-
Sporosarcina sp. P16b             WP_099671921  --E------E--N-LLEKQKK-HSF EQ DFSDKEKNHAVL--K-R----L--D-PS--A-
Sporosarcina sp. P17b             WP_099624599  --E------E--N-LLEKQKE-HSF EQ DFSDKEKNHAVL--K-R----L--D-PS--P-
Sporosarcina sp. P18a             WP_099675180  --E------E--N-LLEKQKK-HSF EQ DFSDKEKNHAVL--K-R----L--D-PS--A-
Sporosarcina sp. P34              WP_099694091  --E------E--N-LLEKQKK-HSF EQ DFSDKEKNHAVL--K-R----L--D-PS--A-
Sporosarcina sp. PTS2304          WP_114925807  --T------E--N-LLEKQKKIQ-F ED RFSNQEENHAVL--K-R----L--VMPAT-S-
Sporosarcina sp. ZBG7A            WP_039041859  --E-K-------D-ISSKQKTIAEV EQ TLSDTEENHDLL-K--KS---L--VVPAS-Q-
Sporosarcina ureae                ARF15245      ---------E--N-LLEKQKD--SF EQ VFSDKEKNHAIL--K-R----L--D-PS--A-
Sporosarcina ureae                ARK22638      --E------E--N-LLEKQKE-HSF EQ DFSDKEKNHAVL--K-R----L--D-PS--P-
Tetzosporium hominis              WP_094941588  --E-K----E-SE-LR-VQETI--- EQ QAGSNTSSLDEL-K--EA---LT-EVPV--E-
Thermolongibacillus altinsuens    WP_132948054  --E--KE-RK-QQQLFAKQQK--EI EK -LADERRI-FNL--D-EK--MY--KV-V----
Ureibacillus thermophilus         QBK27137      --E---KT-E-MD-LNE-KEQISK- EK SFTENEN--KELA---EK------ELPAH-E-
Ureibacillus thermosphaericus     WP_026019002  --E------E-MD-LNA-KAQI-E- EK SFTESTN--KELAQ--EK---I--ELPAH-E-
Viridibacillus arvi               WP_053417535  --A------E-EE-KLL-QQKIK-- EQ DFSGSKEG-KDL-KQ------L--K-PGR-Y-
Viridibacillus sp. OK051          WP_100796388  --A------E-EE-KLQ-QQKIK-- EQ DFSGSKEGSRNL-KQ--N------K-PGQ-S-
```

Supplemental Figure 33

A partial sequence alignment of the hypothetical protein BN1050_01309 containing a two amino acid deletion (boxed) that is exclusively shared by all members belonging to the Jejuensis clade and absent in all other bacteria.

Group	Organism	Accession	64		128
**Jejuensis Clade (3/3)**	Lysinibacillus jejuensis	WP_108305708	AERMTGYKVERAVVSISAHQADIQRVRGVVTV		TNSEVTNDDLHRVMDSASSYKLPEGRDLVNVIP
	Lysinibacillus saudimassiliensis	CEA02606	-------Q-RE----------E----K-----		-SG----A--Q---------------EC---V-
	Lysinibacillus sp. BF-4	WP_036141123	-------E-RE----------E----K-----		-SG----A--Q---------------EC---V-
**Other Bacteria (1/>200)**	Deferribacter desulfuricans	WP_013007330	--K-C-LQIRN-T-G-AGGHIKSFNS--IIA-		K-R---KK-VE--IE---AVDI-I-SEVLH---
	Alteribacillus iranensis	WP_091657490	----V-LD--QV--GVNGNHI-M-FCS---A-	SS	ADR-IRDE-IE----A-QVMPI-PE-EIID---
	Alteribacillus persepolensis	WP_091270239	----V-LQI-QVILGVNGNHI-M-ECS---A-	SS	PDR-IRTE-ID----A-QVLSI-PE-EVID---
	Anaerobacillus alkalidiazotrop	WP_071390838	----I-LSI-NVI-GV-GNHVQL-LCH---A-	SS	PDR-IGDE-IK--I-A-QVFSI-PE-EIID---
	Anaerobacillus alkalilacustris	WP_071309851	----I-LSI-NVI-G--GNHIQL-LCH---A-	SS	PDR-IGEE-IK--I-A-QVFSI-PE-EIID---
	Anaerobacillus arseniciselenat	WP_071314202	----I-MSI-NVI-GV-GNHVQL-QCH---A-	SS	ADR-IGEE-IS--I-A-QVFSI-PE-EIID---
	Anoxybacillus amylolyticus	WP_066323598	----I-LTIQ-V--GVNGNHIQLHDCH-I-A-	SS	E-R-I-DE-VA------QVVSI-PD-EIIG---
	Anoxybacillus ayderensis	WP_085788146	----V-VTIKG-I-GVNGNHIQL-PCN-I-A-	SS	E-K-I--E-VT--IEA-QVVSI-PE-EIID---
	Anoxybacillus flavithermus	WP_006321461	----V-VTIKG-I-GVNGNHIQL-PCN-I-A-	SS	E-K-I--E-VT--IEA-QVVSI-PE-EIID---
	Anoxybacillus gonensis	WP_035065970	----V-VTIKG-I-GVNGNHIQL-PCN-I-A-	SS	E-K-I--E-VT--IEA-QVVSI-PE-EIID---
	Anoxybacillus kamchatkensis	WP_019418350	----V-VTIKG-I-GVNGNHIQL-PCN-I-A-	SS	E-K-I--E-VT--IEA-QVVSI-PE-EIID---
	Anoxybacillus pushchinoensis	WP_091703736	----V-VTIKG-I-G-NGNHIQL-PCH-I-A-	SN	E-K-I--E-VT--IEA-QVVSI-PE-EIID---
	Anoxybacillus sp. BCO1	KHF28564	----V-VTIKG-I-GVNGNHIQL-PCN-I-A-	SS	E-K-I--E-VT--IEA-QVVSI-PE-EIID---
	Anoxybacillus thermarum	WP_043963598	----V-VTIKG-I-GVNGNHIQL-PCN-I-A-	SS	E-K-I--E-VT--IEA-QVVSI-PE-EIID---
	Anoxybacillus vitaminiphilus	WP_111643635	----V-LDIR-V--GV-GNHIQL-NCH---A-	SS	E-R-IS-E-VA--IEA-QVISI-PE-EIID---
	Bacillus acidiceler	WP_088013035	----V-IHIDKVI-GV--N-VQLISTN---A-	SK	E-K-ID-E-VL----Q-QVISI-PE-EIID-V-
	Bacillus acidiproducens	WP_018664073	----V-MEIGQVI-G-ASNHVML-PCH---A-	QS	E-R-I-DG-VQ----A-QVLSI-PEKEI-----
	Bacillus aidingensis	WP_026700150	----V-LDI-QV--GVNGNHI-M-TCS---A-	SS	PDR-IR-E-IE----A-QVLSI-PE-EIID---
	Bacillus alveayuensis	WP_044896116	----V-IPINKV--G--GNHVQL-DCH---A-	SS	E-R-I--E-VK--IEA-QVLSI-PE-EIIDC--
	Bacillus andreraoultii	WP_033829300	----V-MEIRQVI-G-P--HV-LLDCH--IA-	NG	E-R-I-DE-VA--V-A-QVMSI-PD-EII-IV-
	Bacillus aquimaris	WP_044337472	----V-LSISQVI-G-TGNHVSL-SCH---A-	SS	D-R-IGDE-VL----A-QVVSI-PE-EII----
	Bacillus aryabhattai B8W22	SDC36865	----V-ITIKKV--G--GNHVFL-DCH---A-	SS	E-R-I--E-I---I-A-QVMSI-PD-EIID---
	Bacillus badius	WP_063383468	----V-IEIKQV--G-TGNH-LL-PCS---A-	SS	E-R-I-DV-VA--I-ASQVMSI-PD-EII----
	Bacillus campisalis	WP_046522221	----I-MEIR-VI-GVTGNNVTL-PCH---A-	SS	E-R-I--E-VA--I-A-QVMSI-PE-EIID---
	Bacillus cecembensis	WP_057985798	------I-IDEV-LGVP-N-TFL-P-K---A-	NG	E-R-I-D---E--I---QVMSI-PE-E---L--
	Bacillus coagulans	WP_017553747	----V-M-IDHVI-G-ASNHVML-PCH---A-	QS	E-R-I-DG-VQ----A-QVLSI-PE-EI-----
	Bacillus coahuilensis	WP_059282536	----I-MNIKQVI-G--GNHVAL-PCH-I-A-	SS	D-R-I--E-VG--L-A-QVVSI-PD-EII----
	Bacillus cohnii	WP_066417611	----V-MSIN-VI-GV-GNHVHL-NCH---A-	SS	D-K-I--E-IT--I-A-QVFSI-PE-EIIDC--
	Bacillus dakarensis	WP_077211441	----I-MEINQVI-GVAGNHVML-PCH---A-	SS	D-R-I-D--VA--I-A-QVVSI-PE-EIID---
	Bacillus deserti	WP_101645247	----V-MEIK-V--G--GNHISL-PCH---A-	SS	N-K-I-EE-VA--REA-ELLTI-SD-EIID-V-
	Bacillus endophyticus	WP_061801784	----V-MEIH-V--GV-GNHVRL-ECH---A-	SS	E-R-IM-S-IA--I-A-QVMSI-PD-EIID---
	Bacillus farraginis	WP_058002564	----I-MNINHVI-G-AGNHVML-SCH---A-	SS	E-R-I-DE-VA----A-QVISI-PE-EII----
	Bacillus fastidiosus	WP_066227841	----V-IPLR-V--G-TGNHVHL-DCH---A-	SS	E-R-IS-E-VR--IEA-QVMSI-PE-EIID---
	Bacillus filamentosus	WP_019391731	----V-MEIH-V--GV-GNHVRL-DCH---A-	SS	E-R-IM-S-IA--I-A-QVMSI-PD-EIID---
	Bacillus firmus	WP_035328805	----I-MEIKNVI-G-TGNHVML-PSH---A-	SS	E-R-I-DE-VA--I-A-QVVSI-PE--IID---
	Bacillus fordii	WP_018705220	----I-M-INDVI-G-AGNHVAL-PCH---A-	SS	Q-R-I--E-VA----A-QVISI-PE-EII----
	Bacillus fortis	WP_120073860	----I-M-INDVI-G-AGNHVAL-PCH---A-	SS	Q-R-I----VA----A-QVISI-PE-EII----
	Bacillus ginsengihumi	WP_025728817	----V-MNINNVI-G-PSNHVVL-TCH---A-	SS	E-R-I-DE-VA----A-QVMSI-PE-EI-----
	Bacillus gottheilii	WP_066446849	----I-MEIKSVI-G-TGNHVML-PSH---A-	SS	E-R-ISD--VA--I-A-QVVSI-PE--IID---
	Bacillus halmapalus	WP_078380978	----V-LPIN-V--GVTGNHVQL-DCH---A-	SS	D-R-IADE-IA--I-A-QVFSI-PE-EIIDC--
	Bacillus horikoshii	WP_064101190	----V-MPIN-V--GVTGNHVQL-DCH---A-	SS	E-R-IGDE-IA--I-A-QVFSI-PE-EIIDC--
	Bacillus indicus	WP_029285000	----V-IPLR-V--GVTGNHVQL-DCH---A-	SS	E-R-IA-E-IR--I-A-QVMSI-PE-EIID---
	Bacillus jeotgali	WP_079508354	----I-L-INQVI-GVTGNHVSLLPCH---A-	SS	D-R-I--E-VA--I-A-QVVSI-PE-EIID---
	Bacillus kochii	WP_095369610	----I-MEIR-VI-G-TGNHVML-PSH---A-	SS	E-K-ISD--VA----A-QVVSV-PE-EIID---
	Bacillus niacini	WP_045515372	----I-MEIRQVI-G--GN-VALEPC--I-G-	SS	Q-R-I--E-VR--IEE-QVVSI-QD-EIIG---
	Bacillus niameyensis	WP_062105154	----I-MQIKQVI-G-AGN-VGL-PCH---A-	SS	E-R-I--E-VS--I-A-EVVSI-PE-EII-IL-
	Bacillus obstructivus	WP_071976630	----I-MNINHVI-G-AGNHVML-SCH---A-	SS	E-R-I-DE-VA----A-QVISI-PE-EII----
	Bacillus oceanisediminis	WP_110065770	----I-MEIKNVI-G-TGNHVML-PSH---A-	SS	E-R-I-DE-VA--I-A-QVVSI-PE--IID---
	Bacillus oleivorans	WP_097159501	----I-MTIRDVI-G--GNHVML--SH---A-	SS	D-R-I--E-IA----A-QVVSI-PE-EII-I--
	Bacillus oleronius	WP_078109731	----I-MNINHVI-G-AGNHVML-SCH---A-	SS	E-R-I-DE-VA----A-QVISI-PE-EII----
	Bacillus persicus	WP_090747449	----I-MEIRQVI-GVTGNHVTL-SCH---A-	SS	P-R-I--E-VS----A-QVVSI-PE-EIID---
	Bacillus praedii	WP_057766616	----I-MEITSVI-G-TGNHVML-PSH---A-	SS	E-R-I--E-VA--I-A-QVVSI-PE--IID---
	Bacillus salarius	WP_125558946	----V-LDI-QV--GVNGNHI-M-TCS---A-	SS	PDR-IR-E-IE----A-QVLSI-PE-EIID---
	Bacillus selenatarsenatis	WP_041966020	----I-L-INQVI-GVTGNHVSLLPCH---A-	SS	D-R-I--E-VA--I-A-QVVSI-PE-EIID---
	Bacillus smithii	WP_048623106	----V-MEIKQVI-G-P-N-VQL-PCS---A-	SS	E-R-I-DE-VA--I-ACQVMPI-QD-EII--L-
	Bacillus sporothermodurans	WP_066230079	----I-MNINHVI-G-AGNHVML-NCH---A-	SS	E-R-ISDE-VA----A-QVISI-PE-EII----
	Bacillus subterraneus	WP_125479195	----I-M-INQVI-GVTGNHVSLLPCH---A-	SS	D-R-I--E-VA--I-A-QVVSI-PE-EIID---
	Bacillus taeanensis	WP_113805112	----V-L-INSVI-G-TGNHIQL--CH---A-	SS	EDR-IG-E-VA--I-A-QVVSI-PE-EIID---
	Bacillus terrae	WP_120115239	----I-M-INDVI-G-AGNHVAL-PCH---A-	SS	Q-R-I----VA----A-QVISI-PE-EII----
	Bacillus vietnamensis	WP_060669871	----V-LSISQVI-G-TGNHVSL-SCH---A-	SS	D-R-IGDE-VL----A-QVVSI-PE-EII----
	Bacillus weihaiensis	WP_072580134	----V-ISLK-V--GVTGNHVHL-DCH---A-	SS	E-R-IS-E-VR--I-A-QVISI-PE-EIID---
	Bacillus wudalianchiensis	WP_065411540	----V-IEIKQV--G-AGNH-LL-PCS---A-	SS	E-R-I-DV-VA--I-ASQVMSI-PD-EII----
	Bacillus zeae	WP_119111486	----I-MQIRQVI-G--GNHVSLKPCH---A-	AS	E-R-I--E-VS--I-A-QVVSI-PE-EIID---
	Bhargavaea beijingensis	WP_092096331	----I-MSIREV-LG-P-N-VML-Q-K---A-	NS	E-R-I-D---D---R--EVM I-AE-E-I-I--
	Bhargavaea cecembensis	WP_063179511	----I-MSIREV-LG-P-N-VML-Q-K---A-	NS	E-R-I-D---D---R--EVM I-AE-E-I-I--
	Bhargavaea ginsengi	WP_092049090	----I-MSIREV-LG-P-N-VML-Q-K---A-	NS	E-R-I-D---D---R-- EVMI-AE-E-I-I--
	Caldibacillus debilis	WP_020155605	----V-MEIRKVI-G-PC-HISLIDCH---A-	SG	E-R-I--E-VL--I-A-QVISI-PE-EII----
	Caryophanon latum	WP_083995306	-----NL-IDEVILG-P-N-IAL-SAK---A-	NS	E-K-I-DE------E--QVMSISPD-E---I--
	Caryophanon tenue	WP_083998221	-----DL-IDEVILG-P-N-VAL-AAK---A-	NS	E-K-ISD---------QVMSI-PD-E-I-L--
	Chryseomicrobium excrementi	WP_100352782	----A-MEIREV-LG-P-N--S-HH-K---A-	QS	EDK---GK--E--L---KVMSI-PE-EYL-I--
	Diaphorobacter sp. DS2	TFI49057	----I-MEIKNVI-G-TGNHVML-PSH---A-	SS	E-R-I-DE-VA--I-A-QVVSI-PE--IID---
	Edaphobacillus lindanitolerans	WP_076756583	----I-MSIREV-LG-P-N-VML-Q-K---A-	NS	E-R-I-D---D---H-- EVMI-AE-E---I--
	Falsibacillus pallidus	WP_114743761	----I-MNIDKVI-GVAGNHVAL-PCH---A-	SS	D-R-I-D--VA----A-QVISI-PE-EII----
	Falsibacillus sp. GY 10110	WP_121680350	----I-MNINKVI-GVAGNHVTL-PCH---A-	SS	E-R-ISD--VA----A-QVVSI-PE-EII----
	Filibacter sp. TB-66	WP_124070998	----I-MQIQEVILG-P-NGVVL-D-K---A-	NS	E-R-I-D---D---K--QVMSV-PE-EII-I--
	Geomicrobium sp. JCM 19037	GAK04048	----V-MSIKQV--G-NGNHI-LHQSH-I-A-	SS	--K-I-DE-ID--K-A--VVSI-HD-EI-D-V-
	Geomicrobium sp. JCM 19038	GAK07429	----V-MSIKQV--G-NGNHI-LHQTH-I-A-	SS	P-K-I-AE-ID--T-A--VVSI-HD-EI-DIV-

Other Bacteria (1/>200)

Geomicrobium sp. JCM 19039	WP_042430651	----V-MSIKQV--G-NGNHI-LHQSH-I-A-	SS	--K-I-DE-ID--K-A--VVSI-HD-EI-D-V-
Geomicrobium sp. JCM 19055	GAJ99637	----V-MSIKQV--G-NGNHI-LHQTH-I-A-	SS	P-K-I-AE-ID--T-A--VVSI-HD-EI-DIV-
Gracilibacillus boraciitoleran	GAE91730	----VDIHIDQVI-GVNGNHVQL-PCH---A-	SS	D-K-ISEE-I-----A-QVMSI-PE-EI-D---
Gracilibacillus halophilus	WP_003463146	----VDIHID-VI-G-NGNHVQL-PCH---A-	SN	E-K-IAEE-I-----A-QVISI-PE-EI-D---
Gracilibacillus kekensis	WP_073201795	----VDIQIDQVL-G-NGNHVQL-ACH---A-	SS	D-K-IAEE-I-----A-QVMSI-PE-EI-D---
Gracilibacillus massiliensis	WP_058307902	----VDIHIDQVL-G-NGNHVQL-PCH---A-	SS	D-K-IAEE-I-----A-QVMSI-PE-EI-D---
Gracilibacillus orientalis	WP_091483932	----VDIHIDQVI-G-NGNHVQL-SCH---A-	SS	DDK-ISEE-I-----A-QVMSI-PE-EI-D---
Gracilibacillus ureilyticus	WP_089737732	----VDVHID-V--G-NGNHVQL-PCH---A-	SS	EDK-IKEE-I-----A-QVVSI-PE-EI-D---
Halobacillus alkaliphilus	WP_089751883	----VDM-ID-V--G-NGNHIQL-SCH---A-	SS	ESR-IG-E-IT--I-A-QVLSI-PE-EIID---
Halobacillus halophilus	WP_014643119	----VDM-ID-V--G-NGNHIQL-SCH---A-	SS	ESR-IG-E-IT--I-A-QVLSI-PE-EIID---
Kurthia senegalensis	WP_010301748	----I-MSIHKVILG-PGN-VVL-DAK-I-A-	ND	---DI-T--VY--L-A-EMIS-SPD-EKI-LV-
Lentibacillus halodurans	WP_090238305	----V-MQIDSV--GVNGNHIQL-PCH---A-	QS	E-R-IG-E-IQ--I-G-QVISI-PE-EIID---
Lentibacillus sp. Marseille-P4	WP_106497702	----V-MQID-V--G-NGNHVQL-ACH---A-	QS	E-R-IGDE-IT--I-G-QVVSI-PE-EIID---
Listeria cornellensis	WP_036080143	----V-VH-SQVI-G-VSS--RLEAC----AI	SG	E-R-I-D--VWN-I---QVIS-APD-EII----
Listeria newyorkensis	WP_059141378	----V-VH-SQVI-G-VSS-VRLEAC----AI	SG	E-R-I-D--VWN-I---QVIS-APD-EII----
Listeria sp. SHR_NRA_18	WP_036130708	----V-VH-SQVI-G-VSS-VRLEAC----AI	SG	E-R-I-D--VWN-I---QVIS-APD-EII----
Lysinibacillus acetophenoni	WP_097147928	------ISINEV-LG-P-N-TTL-S-K---A-	NS	E-R-I-D---E--IE--QVMSI-PE-E---LL-
Lysinibacillus boronitolerans	WP_016992531	-------QIHEV-LGVP-N-TML-L-K---A-	NS	E-R-I-D---D--VE--QVMSI-PE-E---I--
Lysinibacillus chungkukjangi	WP_107933855	------I-INEV-LG-P-N-TTL-P-K---A-	NS	E-R-I-DE--D--IE--QVMSI-PE-E---L--
Lysinibacillus composti	WP_124764091	------LA--EVILG-P-I-TSLKS-K---A-	NS	E-R-I-D---E--I---QAMSV-PESV---I--
Lysinibacillus contaminans	WP_053584440	--------I-EV-LGVP-N-TML-L-K---A-	SS	E-R-I-D---D--VE--QVMSI-PE-E---I--
Lysinibacillus endophyticus	WP_121212902	------I-IDEVILG-P-N-TTL-P-K---A-	NS	E-R-I-DE--E--IE--QVMSI-PE-E---L--
Lysinibacillus fluoroglycofeni	WP_107942243	------I-IHDVILGVP-N-TFL-P-K---A-	NS	E-R-IGD---E--IE--QVMSI-PE-E---L--
Lysinibacillus fusiformis	WP_025113848	-------QIHEV-LGVP-N-TML-L-K---A-	NS	E-R-I-D---D--VE--QVMSI-PE-E---I--
Lysinibacillus halotolerans	WP_122970277	------I-IDEV-LG-P-N-TTL-P-K---A-	NS	E-R-I-DE--E--IE--QVMSI-PE-E---L--
Lysinibacillus macroides	WP_053993839	-------QIQEV-LGVP-N-TML-L-K---A-	NS	E-R-I-D---D--VE--QVMSI-PE-E---I--
Lysinibacillus manganicus	WP_052123927	------IAINEV-LG-PSN-TAL-S-K---A-	NS	D-R-I-D---E--IE--QVMSI-PE-EV--LL-
Lysinibacillus massiliensis	WP_036171871	------ISINQV-LG-P-N-TTL-S-K---A-	NS	E-R-I-DE--E--IE--QVMSI-PE-E---L--
Lysinibacillus meyeri	WP_107839245	------I-IHDVILGVP-N-TFL-P-K---A-	NS	E-R-IGD---E--IE--QVMSI-PE-E---L--
Lysinibacillus odysseyi	WP_036156075	------L-I-EV-LGVP-N-TVL-P-K---A-	NG	DGR-ISD---A--L---E-IAT-PEKEI--LL-
Lysinibacillus parviboronicapi	WP_107923155	-------QIDEV-LGVP-N-TML-L-K---A-	NS	E-R-I-D---D--VE--QVMSI-PE-E---I--
Lysinibacillus sinduriensis	WP_036204080	------I-INEV-LG-P-N-TTL-P-K---A-	NS	E-R-I-DE--D--IE--QVMSI-PE-E---I--
Lysinibacillus sp. 2017	WP_108711812	------I-I-EV-LGVP-N-TFL-P-K---A-	NG	EDR-I-D---A--I---QVMSI-PE-E---L--
Lysinibacillus sp. B2A1	AVK83064	-------QINEV-LGVP-N-TML-L-K---A-	NS	E-R-I-D---D--VE--QVMSI-PE-E---I--
Lysinibacillus sp. BK089	WP_132357541	-------QINEV-LGVP-N-TML-L-K---A-	NS	E-R-I-D---D--VE--QVMSI-PE-E---I--
Lysinibacillus sp. FJAT-14222	WP_053594636	-------QINEV-LGVP-N-TML-L-K---A-	NS	E-R-I-D---D--VE--QVMSI-PE-E---I--
Lysinibacillus sp. FJAT-14745	WP_053483388	-------QINEV-LGVP-N-TML-L-K---A-	NS	E-R-I-D---D--VE--QVMSI-PE-E---I--
Lysinibacillus sp. Marseille-P	WP_106784054	------I-INQV-LG-P-N-TAL-P-K---A-	NS	E-R-I-D---E--IE--RVMSI-PE-E---LL-
Lysinibacillus sp. SYSU K30002	WP_126657647	------I-IDEV-LG-P-N-TTL-P-K---A-	NS	E-R-I-D---E--IE--QVMSI-PE-E---L--
Lysinibacillus sp. YLB-03	WP_118875198	------I-I-EV-LG-P-N-TTL-P-K---A-	NS	E-R-I-DE--D--IE--QVMSI-PE-E---L--
Lysinibacillus sp. YR326	WP_134019090	-------QINEV-LGVP-N-TML-L-K---A-	NS	E-R-I-D---D--VE--QVMSI-PE-E---I--
Lysinibacillus sp. ZYM-1	WP_054611682	-------QIHEV-LGVP-N-TML-L-K---A-	NS	E-R-I-D---D--VE--QVMSI-PE-E---I--
Lysinibacillus sphaericus	WP_010858338	-------QIDEV-LGVP-N-TML-L-K---A-	NS	E-R-I-D---D--VE--QVMSI-PE-E---I--
Lysinibacillus sphaericus	WP_012293163	-------QIHEV-LGVP-N-TML-L-K---A-	NS	E-R-I-D---D--VE--QVMSI-PE-E---I--
Lysinibacillus sphaericus	WP_054549861	-------QIHEV-LGVP-N-TML-L-K---A-	NS	E-R-I-D---D--VE--QVMSI-PE-E---I--
Lysinibacillus sphaericus	WP_075528497	------MAIHEV--G-P-NGVTL-D-K---A-	NS	E-R-I-D---D---E--QVMSV-PE-EI--I--
Lysinibacillus telephonicus	WP_126292865	------I-IDDV-LG-P-N-TAL-P-K---A-	NS	E-R-I-DE--E--IE--QVMSI-PE-E---L--
Lysinibacillus xylanilyticus	WP_049668346	-------QINEV-LGVP-N-TML-L-K---A-	NS	E-R-I-D---D--VE--QVMSI-PE-E---I--
Lysinibacillus xyleni	WP_097072053	------I-IDEVILG-P-N-TTL-P-K---A-	NS	E-R-I-D---E--IE--QVMSI-PE-E---L--
Massilibacterium senegalense	WP_062197777	--N-V-I-INQVLLG-PS--VQ-HPCN-----	AN	EDR-I-DHEID--I-A-Q-VII-PDQEM--LL-
Mycobacteroides abscessus subs	SHT22413	----I-MEINKVI-GVTGNHVML-PCH---A-	SS	E-R-I--E-VA--I-A-QVISI-PE-EIID---
Oceanobacillus arenosus	WP_115773650	----V-MQI-SVI-G-NGNHIQL-PCH---A-	QS	E-R-IGD--IR--I-G-QVISI-PE-EIID---
Oceanobacillus bengalensis	WP_121130218	----V-MQI--V--G-NGNHI-LRPCH---A-	QS	EDR-IGDE-IR--I-G-QVISI-PE-EIID---
Oceanobacillus caeni	WP_060667907	----V-MHI--V--G-NGNHI-L-PCH---A-	QS	EDR-I-DE-IR--I-G-QVVSI-PE-EIID---
Oceanobacillus halophilus	WP_121202481	----V-MHI--V--G-NGSHIEL-PCH---A-	QS	E-R-ISDE-IR--I-G-QVVSI-PE-EIID---
Oceanobacillus senegalensis	WP_085992469	----V-M-I--V--G-NGSHIEL-PCH-I-A-	QS	E-R-IADE-IR--I-G-QVVSI-PE-EIID---
Oceanobacillus sojae	WP_077601089	----V-MEIH-V--GVNG-HIQL-SCH---A-	QS	E-R-INDE-VT--I-G-QVISI-PE-EIID---
Oceanobacillus sp. Castelsardo	WP_068672473	----V-MHI--VI-G-NGNHVEL-PCH---A-	QS	EDR-I-DE-IR--I-G-QVISI-PE-EIID---
Ornithinibacillus californiens	WP_047984552	----V-MQIDSV--G--GNHIQL-PCH---A-	QS	E-R-I--E-VK--I-G-QVISI-PE-EIID---
Ornithinibacillus contaminans	WP_047981349	----V-MQIDSV--G--GNHVQL-PCH---A-	QS	E-R-I--E-VK--I-G-QVISI-PE-EIID---
Ornithinibacillus halophilus	WP_072886791	----V-MQIDSV--G--GNHIQL-PCH---A-	QS	D-R-I-D--VK--I-G-QVVSI-PE-EIID---
Ornithinibacillus scapharcae	WP_010096776	----V-MQIDSV--G--GNHIQL-PCH---A-	QS	E-R-I--E-VK--I-G-QVISI-PE-EIID---
Paenibacillus sp. FSL R5-0490	WP_076255638	----I-MEIKNVI-G-TGNHVML-PSH---A-	SS	E-R-I-DE-VA--I-A-QVVSI-PE--IID---
Paenisporosarcina antarctica	WP_134210143	----I-MSINEV-LG-P-N--ML-H-K---A-	NS	E-R-I-DE--E--I---QVMSI-PE-E---LV-
Paenisporosarcina indica	WP_075617478	----I-MSINEV-LG-P-N-SML-Q-K---A-	NS	E-R-I-D---D---E--QVMSI-PE-E---LV-
Paenisporosarcina quisquiliaru	WP_090562817	----I-MSINEI-LG-P-NM-N--N-K---A-	NR	EDR-I-DG--E--IE--QVMSI-PE-E-I-IA-
Paenisporosarcina sp. HGH0030	WP_016427766	----I-MSINEV-LG-P-N--MLHQ-K---A-	NS	E-R-I-D---D--I---QVMSI-PE-E---LV-
Paenisporosarcina sp. K2R23-3	WP_119882906	----I-MSINEV-LG-P-G--LL-P-K---A-	NS	ETR-I-DE--D------QVMSI-PE-E-ISI--
Paenisporosarcina sp. OV554	WP_108584531	----I-MSINEV-LG-P-N--MLHH-K---A-	NS	E-R-I-D---D--IE--QVMSI-PE-E---LV-
Paenisporosarcina sp. TG-14	WP_017380782	----I-MSINEV-LG-P-N--ML-H-K---A-	NS	E-R-I-DE--E--I---QVMSI-PE-E---LV-
Paenisporosarcina sp. TG20	WP_019413882	----I-MSINEV-LG-P-N--ML-H-K---A-	NS	E-R-I-D---E--IE--QVMSI-PE-E---LV-
Paracoccus sp. DMF	TFE38587	----I-MEIKNVI-G-TGNHVML-PSH---A-	SS	E-R-I-DE-VA--I-A-QVVSI-PE--IID---
Parageobacillus thermantarctic	WP_090948863	----I-LTIR-VI-GVTGNHVQL-DCH-I-A-	SS	E-R-I-DE-VA----A-QVVSI-PD-EIIG---
Parageobacillus thermoglucosid	WP_064551112	----V-LTIR-VI-GVTGNHVQL-DCH-I-A-	SS	E-R-I-DE-VA----A-QVVSI-PD-EIIG---
Parageobacillus toebii	WP_062753472	----V-LTIR-VI-GVTGNHVQL-DCH-I-A-	SS	E-R-I-DE-VA----A-QVVSI-PD-EIIG---
Paucisalibacillus globulus	WP_096269968	----V-MQIDSV--G--GNHIQL-PCH---A-	QS	E-R-I----VK--I-G-QVISI-PE-EIID---
Paucisalibacillus sp. EB02	WP_042144994	----V-MQIDSV--G--GNHIQL-PCH---A-	QS	E-R-I--E-VK--I-G-QVISI-PE-EIID---
Planococcus antarcticus	WP_006828829	----I-KSIHEV-LG-P-NK-VL-P-K-I-A-	NS	E-R-I-D---D--LEA-QVMSI-PE-E---I--
Planococcus donghaensis	WP_008430748	----I-KSIHEV-LG-P-NK-VL-P-K-I-A-	NS	E-R-I-DE--D--LEA-QVMSI-PE-E---I--
Planococcus halocryophilus	WP_065528111	----I-KSIHEV-LG-P-NK-VL-P-K-I-A-	NS	E-R-I-DE--D--LEA-QVMSI-PE-E---I--

Other Bacteria (1/>200)

Planococcus halotolerans	WP_112222932	----I-KSIHEV-LG-P-NKMML-P-K---A-	NS	E-R-I-D--MD--LEA-QVMSI-PE-E---I--
Planococcus maitriensis	WP_112231371	----I-KEIHEV-LG-P-N-VAL-P-K-I-A-	NS	E-R-I-DE--E--LEA-QVMSI-PE-E---I--
Planococcus maritimus	WP_068486058	----I-KEIHEV-LG-P-N-VAL-P-K-I-A-	NS	E-R-I-DE--E--LEA-QVMSI-PE-E---I--
Planococcus massiliensis	WP_052651111	----I-KSIHEV-LG-P-NK-ML-P-K---A-	NS	E-R-ISD---D--L-A-QVISI-PE-E---I--
Planococcus salinarum	TAA68706	----I-KSIHEV-LG-P-NKMML-P-K---A-	NS	E-R-I-DE-MD--LEA-QVISI-PE-E---I--
Planococcus salinus	WP_123164372	----I-RSIHEV-LG-P-N--RL-P-K---A-	NS	E-R-I-DE--D--LEA-EVISI-PE-E---I--
Planococcus sp. CAU13	WP_033543516	----I-KSIHEV-LG-P-NKMVL-P-K---A-	NS	E-R-I-DE-MD--LEA-QVMSI-PE-E---I--
Planococcus sp. PAMC 21323	WP_038704318	----I-KSIHEV-LG-P-NK-VL-P-K-I-A-	NS	E-R-I-DE--D--LEA-QVMSI-PE-E---I--
Planococcus sp. Y42	WP_077590991	----I-KSINEV-LG-P-N-VKL-P-K---A-	NS	E-R-I-DM--E--HEA-QVVSI-TD-EF---V-
Planococcus versutus	WP_049693343	----I-KSIHEV-LG-P-NK-LL-P-K-I-A-	NS	E-R-I-D---D--LEA-QVMSI-PE-E---I--
Planomicrobium flavidum	WP_088005770	----I-KSINEV-LG-P-N-VQL-P-K---A-	NS	E-R-I-DI--E--HEA-QVVSI-PD-EF---V-
Planomicrobium glaciei	WP_036806882	----I-KSIHEV-LG-P-NK-ALHP-K---A-	NS	E-R-I-D---D--LEA-QVISI-PE-E---I--
Planomicrobium soli	WP_106534889	----I-KSIQEV-LG-P-SKVALHP-K---A-	NS	E-R-I-DE--D--LE--QVMSI-PE-E------
Planomicrobium sp. Y74	WP_121632786	----I-KSIHEV-LG-P-NKMML-P-K---A-	NS	E-R-I-D--MD--LEA-QVMSI-PE-E---I--
Pontibacillus chungwhensis	WP_036778477	----V-MQIN-V--G-NGSHIQL-PCH---A-	QS	E-R-IG-E-IA--I-A-QVISV-PE-EI-D---
Pontibacillus litoralis	WP_036832151	----V-MNIDKV--G-NGNHVQL-PCH-I-A-	QS	E-R-I-DE-IT--I-A-QVVSI-PE-EIID-V-
Pontibacillus marinus	WP_027448201	----V-MQIN-V--G-NGNHIQL-PCH---A-	QS	E-R-IDEE-IN--L-A-QVMSI-PE-EIID---
Psychrobacillus insolitus	WP_111438640	----I-MSIHEI-LG-P-NL-NV-H-K---A-	NR	EDR-I-DG--E--IE--EVMSI-PE-E-I-IV-
Psychrobacillus psychrodurans	WP_093494402	----I-MSINEI-LG-P-NM-N--N-K---A-	NR	EDR-I-DG--E--IE--QVMSI-PE-E-I-IA-
Psychrobacillus psychrotoleran	WP_093535310	----I-MSINEI-LG-P-NM-N--N-K---A-	NR	DDR-I-DG--E--IE--QVMSI-PE-E-I-IA-
Psychrobacillus sp. FJAT-21963	WP_056827377	----I-MSIQEI-LG-P-NL-N--N-K---A-	NR	EDR-I-DT--D--IE--QVMSI-PE-E-I-IA-
Psychrobacillus sp. OK028	WP_093059946	----I-MSINEI-LG-P-NM-N--N-K---A-	NR	EDR-I-DG--E--IE--QVMSI-PE-E-I-IA-
Psychrobacillus sp. OK032	WP_093263854	----I-MAIHEI-LG-P-NL-N--N-K---A-	NR	EDR-I-DS--E--IE--QVMSI-PE-E-I-IA-
Quasibacillus thermotolerans	WP_039231976	----V-IEIKKV--G-AGNH-LL-PCN---A-	SS	E-R-I-DV-VG--I-ASQVMSI-PD-EII----
Rummeliibacillus pycnus	WP_102692747	----V-MSIHKVILG-P-N-VQLHD-K---A-	NS	E-R-I-DV--D--IE--QLMSI-PD-E---IV-
Rummeliibacillus sp. POC4	WP_119414206	----V-LTINKVILG-P-N-VQLHP-K---A-	NS	E-R-I-DV--D--IE--ELMSI-PD-E---IV-
Rummeliibacillus sp. TYF005	WP_124217212	----V-LTINKVILG-P-N-VQLHP-K---A-	NS	E-R-I-DV--D--IE--ELMSI-PD-E---IV-
Rummeliibacillus stabekisii	WP_066785209	----V-MTINKVILG-P-N-VQLHA-K---A-	NS	E-R-I-DL--D---E--QLMSI-PD-E-I-IV-
Salibacterium halotolerans	WP_093334959	----V-LEI-QV--GVNGNHIQM-NCS---A-	SS	PDR-IS-E-IE----A-QVLSI-PE-EIID---
Salibacterium qingdaonense	WP_090925536	----V-LEI-QV--GVNGNHIHM-NCS---A-	SS	PDR-IN-E-IE----A-QVLSI-PE-EIID---
Salisediminibacterium haloalka	WP_093071725	----INS-INNV--GVTGNHLEL-PCH---A-	SS	DD--INDE-IN--I-A-QVISI-QE-EI--I--
Salisediminibacterium halotole	WP_121439715	----INS-INNV--GVTGNHLEL-PCH---A-	SS	DD--INDE-IN--I-A-QVISI-QE-EI--I--
Saliterribacillus persicus	WP_114352788	----VDI-I--VI--VNGSHVQL-PCH---A-	SS	E-R-IG-E-I---IEA-QVISI-PE-EI-D---
Salsuginibacillus halophilus	WP_106587493	---SC-FAI--VI-GVNG-HV-L-PSH---A-	SS	E-R-I--E-ID--I-A-QVVSI-PE-EI-D-V-
Salsuginibacillus kocurii	WP_018921336	---TA-LTI-K-I-GVNGNHIQL-PCH---A-	SD	P-R-IGA--IE--L-A-QVLSI-PE-EV-D-V-
Scopulibacillus darangshiensis	WP_132745994	----V-LQIKSVI-GVTGNHIQL-PCH---A-	SS	E-R-IG-I-IA----A-QVMSI-PE-EIID---
Solibacillus isronensis	WP_079525030	------INI-EV-LGVP-N-TVL-P-K---A-	NG	EDR-I-D---A--I---QVMSI-PE-E---L--
Solibacillus silvestris	WP_014824540	------INI-EV-LGVP-N-TVL-P-K---A-	NG	EDR-I-D---A--I---QVMSI-PE-E---L--
Solibacillus sp. R5-41	WP_099422691	------I-IDEV-LGVP-N-TFL-S-K---A-	NG	E-R-I-D---E--I---QVMSI-PE-E---L--
Sporolactobacillus laevolactic	WP_023508981	----V-INIKSVI-G--GNHIQLR-CH---A-	SG	ETH--G-E-IA--I-A-QVMSI-PE-EI-D---
Sporolactobacillus sp. THM7-7	WP_130000470	----V-LSIQSV--GV-GNHVKL-NC--I-A-	SN	E-R-IGDV-IA--I-A-QVMSI-PE-EIID---
Sporosarcina globispora	WP_053436556	----I-MEIKNVI-G-TGNHVML-PSH---A-	SS	E-R-I-DE-VA--I-A-QVVSI-PE--IID---
Sporosarcina koreensis	WP_040286932	------LRIQEVILG-PSNGVAL-D-K---A-	NS	E-R-I-D-E-D---E--QVMSVAPE-EI--I--
Sporosarcina newyorkensis	WP_078817809	------L-I-DV-LG-P-NGVQL-D-K---A-	NS	E-R-I-D---E---K-SQVMNV-PE-EI--LV-
Sporosarcina pasteurii	WP_115360387	------MAIHEV--G-P-NGVTL-D-K---A-	NS	E-R-I-D---D---Q--QVMSV-PE-EI--I--
Sporosarcina psychrophila	WP_067211461	----I-MQIREVILG-P-NGVSL-D-K---A-	NS	E-R-I-D---D---K--QVMSV-PD-EI--I--
Sporosarcina sp. BI001-red	WP_116016200	------LQ-TEV-LG-PSNGVTL-D-K---A-	NS	V-R-I-D---D---E--QVMSVAPE-EI--I--
Sporosarcina sp. D27	WP_025785138	------LQ-KEV-LG-PSNGVTL-D-K---A-	NS	V-R-I-D---D---E--QVMSVAPE-EI--I--
Sporosarcina sp. EUR3 2.2.2	WP_024535077	----I-MSINEV-LG-P-N--MLHH-K---A-	NS	E-R-I-D---D--IE--QVMSI-PE-E---LV-
Sporosarcina sp. HY008	WP_067408074	----I-MQIREVILG-P-TGVVL-D-K---A-	NS	E-R-I-D---D---K--QVMSV-PE-EI--I--
Sporosarcina sp. P13	WP_099687186	------L-I-EV-LG-P-NGVQF-N-K---A-	NS	E-R-I-D---E---K-SQVMNV-QE-EF--LV-
Sporosarcina sp. P34	WP_099694093	------L-I-EV-LG-P-NGVQF-N-K---A-	NS	E-R-I-D---E---K-SQVMNV-PE-EF--LV-
Sporosarcina sp. PTS2304	WP_114923251	------L-I-EV-LG-P-NGVQF-D-K---A-	NS	E-R-I-D---E---K-SQVMNV-QE-EF--LV-
Streptohalobacillus salinus	WP_110250483	----VDMRIDNVI-G-NGN-VEL-PCQ-I-A-	AS	E-R-IGD--VT--I-A-KVIS--PE-EIID---
Terribacillus aidingensis	WP_097040285	----V-MQI--V--GVNGN-IQL-SCH---A-	SS	ETR-IDDE-IS--IEA-QVVSI-PE-EI-D---
Terribacillus halophilus	WP_077309748	----V-MQI--V--GVNGN-IQL-SCH---A-	SS	ETR-IDDE-IS--IEA-QVVSI-PE-EI-D---
Terribacillus saccharophilus	WP_095217529	----V-MQI--V--GVNGN-IQLHPCH---A-	SS	ETR-IDDE-IA--L-A-QVVSI-PE-EI-D---
Terribacillus sp. 7520-G	WP_095214623	----V-MQI--V--GVNGN-IQL-SCH---A-	SS	ETR-IDDE-VA--I-A-QVVSI-PE-EI-D---
Tetzosporium hominis	WP_094941590	----A-MEIREV-LG-P-N--S-HQ-K---A-	QS	EDK---GI--E--L---KVMSI-PE-EYL-I--
Thermolongibacillus altinsuens	WP_132948057	----V-I-IN-VI-GVNGNHIQL-DCH-I-A-	SS	E-R-IS---VA--IEA-QVVSI-PE-EIID---
Ureibacillus thermophilus	QBK25556	------IQIREV-LG-P-N-TVL-P-K---A-	NS	E-R-I-D---D--FE--QVMSI-PE-E---I--
Ureibacillus thermosphaericus	WP_016837501	------IQIREV-LG-P-N-TAL-P-K---A-	NS	E-R-I-D-E-E--FE--QVMSI-PE-E---L--
Virgibacillus dokdonensis	WP_116276904	----V-M-I--V--GVNGNHIQL-PCH---A-	QS	E-R-I-DE-VT--I-G-QVMSI-PE-EIID---
Virgibacillus halodenitrifican	WP_019378517	----V-MHI--V--G-NGNHIQL-NCH---A-	QS	E-R-I--E-IA--I-G-QVISI-PE-EIID---
Virgibacillus indicus	WP_094883998	----V-MQI-SV--G-NGNHIQL-PCH---A-	QS	E-R-I-DE-IT--I-G-QVISI-PE-EIID---
Virgibacillus sp. SK37	WP_040955743	----V-MHI--V--G-NGNHIQL-NCH---A-	QS	E-R-I--E-IA--I-G-QVISI-PE-EIID---
Viridibacillus arvi	WP_053417533	----I-MTINKVILG-P-N--ML-P-K---A-	NS	D-R-IGDE--D---E--QVMSI-PE-E---LV-
Viridibacillus sp. OK051	WP_100796391	----I-MSINKVILG-P-N--ML-P-K---A-	NS	D-R-IGDE--D--LE--QVMSI-PE-E---LV-

Supplemental Figure 34

A partial sequence alignment of the cell division protein FtsA protein containing a two amino acid deletion (boxed) that is exclusively shared by all members belonging to the Jejuensis clade and absent in all other bacteria. *Deferribacter desulfuricans* is the sole exception which also shares this CSI.

				153		206
**Jejuensis Clade (3/3)**	Lysinibacillus saudimassiliensis		CEA04564	DEPTSGLDPIMRDKILDLLVQFM	D	EDATRSILFSSHITTDLDKIADMITFIHKG
	Lysinibacillus jejuensis		WP_108305846	-------------E---------	-	------------------------------
	Lysinibacillus sp. BF-4		WP_036141530	-------------E---------	-	------------------------------
**Other Bacteria (1/>200)**	Anaerococcus sp. Marseille-P35		WP_105301823	---------S---ELV--ILD--	E	--EDHTV---T---Q---R---FLI--DE-
	Acetivibrio ethanolgignens		WP_058353309	---------VI-NE---VFLEYI		Q-ERN---L-----S--E----Y-----Q-
	Acutalibacter muris		WP_066541223	--A------V---D---VFLE-V		Q-EEH---I--------E-V--S-----Q-
	Agathobaculum butyriciproducen		WP_117491057	---------VV--EV----YD--		Q-ENHA--L-----S-------E-------
	Agathobaculum desmolans		WP_031472701	---------VV--EV---FYE--		QEESHA--L-----S-------Y-----Q-
	Agathobaculum sp. Marseille-P7		WP_125116172	---------VV--EV---FYD--		Q-DGH---L-----S-------Y-----Q-
	Alkaliphilus transvaalensis		WP_026478811	---------VI-SEL--V-YDII		Q-ER------T---R-------Y----D--
	Anaerocolumna aminovalerica		WP_097014574	---------AI--EF-EI-QEYI		K-GE--V---T------ERV--Y----NR-
	Anaerosalibacter sp. Marseille		WP_077369052	---------VF-RE---I-YNVI		Q-ESK--F--T------E----Y----NN-
	Anaerovirgula multivorans		WP_089284940	----------F-RE---I-YSII		Q-E-K--F--T--------V--Y----DN-
	Aneurinibacillus migulanus		WP_043068910	----------F-REL--I-HNI-		Q-GKKT-F--T-------R---Y-------
	Aureibacillus halotolerans		WP_133579311	---------VV-REV--I-SEVI		Q-ENKT-F---------ER---Y-----N-
	Bacilliculturomica massiliensi		WP_130863860	----G----VV-NE----FLD-I		Q-EEH-----T------E----Y-----E-
	Bacillus atrophaeus		WP_106046777	----------F-REF--I-HNI-		Q-GKKT-F--T-------R---Y-----N-
	Bacillus bogoriensis		WP_026674830	----------F-REL--I-HSL-		Q-GEKT-F--T-------R---Y-----N-
	Bacillus cecembensis		WP_057986943	----------V--EV-E--QK--		KNDEHA--------S--E----T-------
	Bacillus cereus		WP_065223308	---------VF-REV--IVYDL-		I-QNKT----T-------R---Y-V-----
	Bacillus endophyticus		WP_111925480	---------VV-RE---I-YDL-		Q-EQKT-F----------R---Y-------
	Bacillus farraginis		WP_058006654	----------F-REL--I-H-L-		Q-GNKT-F--T-------T---Y-----Q-
	Bacillus filamentosus		WP_019392197	---------VV-RE---I-YDL-		Q-EQKT-F----------R---Y-------
	Bacillus glycinifermentans		WP_082634631	----------F-REL-EV-H-LI		Q-GDKT-F--T-----------Y-------
	Bacillus gottheilii		WP_066451000	----------F-REL-EI-H-L-		Q-GDKT-F--T-------M---Y-----N-
	Bacillus halotolerans		WP_059293557	----------F-REF--I-HNI-		Q-GKKT-F--T-------R---Y-----N-
	Bacillus haynesii		WP_083698322	----------F-REL-EI-H-L-		Q-GDKTVF--T-----------Y-------
	Bacillus kochii		WP_095370379	---------VF-REL--I-QEI-		--G-KT-F--T---S---R---Y-----N-
	Bacillus kwashiorkori		WP_087941729	----------F-REL--I-HDL-		Q-SEKT-F--T-------R---Y-----H-
	Bacillus lentus		WP_066136667	----------F-REL--I-QE-I		Q-ENK--FY-T------EQ---Y-----D-
	Bacillus licheniformis		WP_011201535	----------F-REL-EI-H-L-		Q-GDKT-F--T-----------Y----D--
	Bacillus manliponensis		WP_034641608	----------F-REL--I-HNI-		Q-GEKT-F--T------ER---Y-----N-
	Bacillus massiliogabonensis		WP_102275107	----------F-RD---I-YDI-		Q-EKKT-F----------R---Y---L-N-
	Bacillus mojavensis		WP_010335051	----------F-REF--I-HNI-		Q-GKKT-F--T-------R---Y-----N-
	Bacillus mycoides DSM 2048		EEL98777	---------VF-REL--V-QNI-		Q-GEKT-F----------R---Y----QN-
	Bacillus obstructivus		WP_071977614	----------F-REL--I-H-L-		Q-GNKT-F--T-------T---Y-----Q-
	Bacillus oleronius		WP_078109436	----------F-REL--I-H-L-		Q-GNKT-F--T-------T---Y-----Q-
	Bacillus paralicheniformis		KFM89913	----A-----F-REL-EI-H-L-		Q-GDKT-F--T-----------Y-------
	Bacillus sonorensis		WP_081605421	----------F-REL--I-H-LI		Q-GDKT-F--T-------T---Y-------
	Bacillus subtilis group		WP_041817138	----------F-REL--I-H-L-		Q-GDKT-F--T-----------Y-------
	Bacillus swezeyi		OMI05376	----------F-REL--I-H-LI		Q-GDKT-F--T-------T---Y-------
	Bacillus thuringiensis		WP_098851398	---------VF-REL--V-QNI-		Q-GEKT-F----------R---Y----QD-
	Bacillus wiedmannii		WP_097908592	---------VF-REL--V-QNI-		Q-GEKT-F----------R---Y----QN-
	Bifidobacterium merycicum		KFI71406	--A------V---EM--VFLD-V		QEEGH---L-----S--E----Y-----N-
	Blautia hominis		WP_095173044	--A------V---D---VFMD-V		Q-ESH---M--------E----Y-------
	Blautia hydrogenotrophica		WP_005948539	--A-------V-EE----FLE-V		--EK-TV-I-----S--E-V--Y---M-Q-
	Blautia marasmi		WP_104804770	---------VS--EM--I-LEYT		D-KKH-V---T---P-------Q----N--
	Blautia obeum		WP_118034853	--A----------E---MFLD-I		Q-EEN---V-----S--E-V--Y-V---N-
	Blautia producta		WP_033140106	--A------V---D---VFLD-V		Q-ENH---M--------E-V--Y-----Q-
	Blautia schinkii		WP_044945162	--I--A---L--EE--S-FLE-I		--ES---------I---E----Y----NN-
	Blautia sp. AF17-9LB		WP_118595614	--A------V---D---VFLE-V		Q-ENH---M--------E-V--Y-----Q-
	Blautia sp. OM06-15AC		WP_118697153	--A-------I--DV--M-ID-V		Q-ENH-V-V-----S--E-V--Y-------
	Butyricicoccus pullicaecorum		WP_087415152	---------VI--E---IFYD--		Q-DSH---I-----S--E--C-YVA---Q-
	Butyricicoccus sp. OM06-6AC		WP_117751642	---------VV--EV----YD--		Q-ENHA--L-----S-------E-------
	Caldibacillus debilis		WP_120670103	---------LV-NEL--I-RTL-		--GNT-VF--T---S-------Y--L--E-
	Candidatus Arthromitus sp. SFB		EIA25667	---------VS--EL--I-LEYI		--GEH-V---T---E--ERV--Y--Y-NY-
	Chlamydia abortus		SHE14477	---------VF-REM----ADM-		Q-ERN--I--T-------R---Y-A---R-
	Clostridioides difficile		SUY25142	--A--S----V-EE---IFLD-I		Q-EEH--IL-----S-------Y-------
	Clostridium amylolyticum		WP_073012477	---------V---EF-EI-QDYI		K-GE--V---T------E----Y----NQ-
	Clostridium botulinum		WP_012422726	---------VI-NE--E--QEYI		--GEK-V---T---S--E----Y-F--N--
	Clostridium cadaveris		WP_027639322	----G----VA--EFI---GEYI		Q-GE--VI--T---S--E----Y----R--
	Clostridium cochlearium		STA91979	---------VF-RE---I-LELI		QNENK-VF--T------E----Y--Y-N--
	Clostridium grantii DSM 8605		SHI01159	----A----MA-IEV--I-RE-V		A-GEK-VF--T---S-------Y--L----
	Clostridium jeddahense		WP_042436409	--A------V--SE-----LE-I		Q-EEHAV-------A--ER---YV----E-
	Clostridium liquoris		WP_106063180	----------F-CE---I-CELI		Q-ENK-VF--T------E----Y----NN-
	Clostridium lundense		WP_027624175	---------VF-CE---I-YELI		Q-ENK-VF--T------E----Y----NN-
	Clostridium oryzae		WP_079422141	---------VF-RE---I-KYVI		Q-ENK--F--T------E----Y----NN-
	Clostridium peptidivorans		WP_097026845	---------VF-RE---I-YSLI		Q-ENK--F--T------E----Y----N--
	Clostridium perfringens		SUY32549	---------VF-SEL--I-FEII		GEEEV---Y-T------E-L--Y---LN--
	Clostridium tetani	E	WP_023437641	---------VF-RE---I-LEL-		QNENK-VF--T------E-V--Y--Y-N--
	Cohnella sp. HS21		WP_130609047	---------VF-REL----ADM-		Q-ERN--I--T-------R---Y----NR-
	Coprobacillus cateniformis		WP_008788679	--A----------E---VFME-V		QNENHA--L----SS--E-V--Y-----E-
	Defluviitalea phaphyphila		WP_058485024	----------I-NEL--I-TS-I		QNENK-VF--T-----------Y----NN-
	Desulfonispora thiosulfatigene		WP_084052429	---------VF-RE---V-YDII		Q-ENK--F--T------E----Y----N--

Other Bacteria (1/>200)

Dethiobacter alkaliphilus		WP_008513798	---------VF-REL--IFAEMI	--G-------T---S---RT--Y----NN-
Dorea sp. 5-2		WP_016217833	--A------VI--D---I-LD-A	Q-E-H---I--------E----Y-V---E-
Eisenbergiella massiliensis		WP_025487779	--A------V---D---VFLD-I	Q-E-H---M--------E-V--Y-----Q-
Eisenbergiella sp. OF01-20		WP_118679402	--A------V---D---VFLD-I	Q-ENH---M--------E-V--Y-----E-
Eisenbergiella tayi		WP_071699491	--A------V---D---IFLDYI	Q-ENH---M--------E----YV----E-
Enterococcus faecium		WP_023042869	--A------V---D---VFLD-V	Q-ENH---M--------E-V--Y-----Q-
Erysipelatoclostridium sp. An1		WP_087373202	--A----------E---VFME-V	Q-ENHA--L----SS--E----Y-----E-
Eubacterium sp. CAG:786		CCY17864	----G----VV-SE---M-LE--	Q-E-H---I-T---S--EH---Y-G---Q-
Faecalibacterium prausnitzii L	E	CBK99182	--A------V---D---VFLD-V	Q-ENH---M--------E-V--Y-----Q-
Faecalibacterium sp. An122		WP_087384952	--A------V---DM--VFLE-V	Q-EEH---M--------E-V--Y-----Q-
Faecalibacterium sp. An192		WP_087369015	--A------V---DM--VFLE-V	Q-EEH---M--------E-V--Y-----Q-
Faecalibacterium sp. An77		WP_087248117	--A------V---DM--VFLE-V	Q-EEH---M--------E-V--Y-----Q-
Fenollaria massiliensis		WP_019214845	--A----------E---V-LE-V	KEENH---L-----S--E----Y-V---N-
Fontibacillus panacisegetis		WP_091225735	--A----------D---IFLE-V	Q-ENH---M--------E----Y-----Q-
Frisingicoccus caecimuris		WP_132093775	--A------VV--E---MFQE-I	Q-EAH-V-I-----S--E-V--Y-------
Fusicatenibacter sp. 2789STDY5		CUQ40001	----------I--E---IFLD-I	Q-E-H--FV-T--VE--E----Y-------
Gemmiger sp. An194		WP_087296284	--A------VV--D-----LE-V	Q-E-H---V-----S--E----Y-V-L---
Gemmiger sp. An87		WP_087182880	--A------V---D---VFLD-V	Q-ENH---M--------E-V--Y-----Q-
Geobacillus galactosidasius		WP_089096827	----------I-SEL--I-SS-I	Q-ENKGVF--T---S-------Y-----D-
Geobacillus zalihae		WP_081212683	----------I-SEL--I-SS-I	Q-EN-GVF--T---S-------Y----ND-
Holdemania filiformis		WP_006059166	----------V--EM---FYD-I	Q-E-H-----------IE----RV----Q-
Holdemania massiliensis		WP_020223635	----------V--E----FYD-I	Q-E-H-----------IE----VV----Q-
Holdemania sp. Marseille-P2844		WP_072684335	----------V--EM---FYD-I	Q-E-H-----------IE----TV----Q-
Intestinibacillus massiliensis		WP_087065684	---------VV--EV---FWE-L	Q-ENH-V-I-----S-------Y-----E-
Intestinibacillus sp. Marseill		WP_054326037	---------VV--EV---FYD--	QEDSHA--L-----S-------H-----Q-
Keratinibaculum paraultunense		WP_132028603	---------VF-RE---I-YNII	Q-D-K--F--T------E----Y----NE-
Lachnoclostridium pacaense		WP_130789432	--A------VV--E---I-FG-I	--GGN-V-I-----S----V--Y--M--N-
Lachnoclostridium phocaeense		WP_076776056	--A-------I--DV--M-ID-V	Q-ENH-V-V-----S--E-V--Y---L---
Lachnoclostridium sp. An169		WP_087159553	--A------V---D---VFLD-V	Q-ENH---M--------E----Y-----Q-
Lysinibacillus acetophenoni		WP_097148067	----------V--E----FLD--	Q-DEH---------S--E----Y-----E-
Lysinibacillus boronitolerans		WP_036080400	----------I--E----FLA--	Q-E-H---------S--E----Y--L--N-
Lysinibacillus contaminans		WP_053584286	----------I--E----FLE--	Q-N-H---------S--E----Y-----N-
Lysinibacillus endophyticus		WP_121213033	----------V--E----FLE--	Q-EKH---------S--E----Y-----E-
Lysinibacillus fusiformis		WP_096365657	----------F-REL-NI-HEL-	Q-GDKT-F--T-------R---Y-----N-
Lysinibacillus halotolerans		WP_122970409	----------V-EE----FLE--	Q-EEH---------S--E----Y-----E-
Lysinibacillus macroides		WP_053993971	----------I--E----FLE--	Q-E-H---------S--E----Y--L--N-
Lysinibacillus manganicus		WP_052124232	----------V--E---IFLD--	QEEEH---------S--E----Y-----E-
Lysinibacillus mangiferihumi		WP_107894654	----------I--E----FLA--	Q-E-H---------S--E----Y-----N-
Lysinibacillus massiliensis		WP_052126086	----------V--E----FLEY-	Q-E-N---------S--E----Y--L--D-
Lysinibacillus odysseyi		WP_036152381	----------V--E----FLH--	-EEEHT--------S--E----S-----E-
Lysinibacillus parviboronicapi		WP_107947814	----------I--E----FLA--	Q-ERH---------S--E----Y--C--N-
Lysinibacillus sp. 2017		WP_108711951	----------V--EV-GI-Q---	-QEEHA--L-----S--E----T-----E-
Lysinibacillus sp. B2A1		AVK83219	----------I--E----FLA--	Q-E-H---------S--E----Y-----N-
Lysinibacillus sp. BK089		WP_132357393	----------I--E----FLT--	Q-E-H---------S--E----Y-----N-
Lysinibacillus sp. FJAT-14745		WP_082337432	----------I--E----FLT--	Q-E-H---------S--E----Y-----N-
Lysinibacillus sp. OL1		WP_131520102	----A-----F-REL----QEL-	V-GN-T-F--T-------R---Y-A--Q--
Lysinibacillus sp. OL1		WP_131521872	----------I--E----FLA--	Q-E-H---------S--E----Y--L--N-
Lysinibacillus sp. PB300		WP_115673689	----A-----F-REL----QEL-	V-GN-T-F--T-------R---Y-A--Q--
Lysinibacillus sp. YR326		WP_134018884	----------I--E----FLT--	Q-E-H---------S--E----Y-----N-
Lysinibacillus sp. YS11		WP_103118243	----A-----F-REL----QEL-	V-GN-T-F--T-------R---Y-A--Q--
Lysinibacillus sp. ZYM-1		WP_054610363	----------I--E----FLA--	Q-E-H---------S--E----Y--L--N-
Lysinibacillus sphaericus		POZ57611	----------I--E----FLT--	Q-E-H---------S--E----Y-----N-
Lysinibacillus sphaericus		WP_010858500	----------I-EE----FLD--	Q-E-H---------S--E----Y--C--N-
Lysinibacillus sphaericus		WP_024361983	----------I--E----FLA--	Q-E-H---------S--E----Y-----N-
Lysinibacillus sphaericus		WP_036128810	----------F-REL-NI-HEL-	Q-EHKT-F--T-------R---Y-----N-
Lysinibacillus sphaericus		WP_036223742	----------F-REL-NI-HEL-	Q-GDKT-F--T-------R---Y-----N-
Lysinibacillus sphaericus		WP_036226492	----------I--E----FLA--	Q-E-H---------S--E----H--L--N-
Lysinibacillus sphaericus		WP_069510082	----------I--E----FLT--	Q-E-H---------S--E----Y-----N-
Lysinibacillus xylanilyticus		WP_100543897	----------V--E----FLT--	Q-E-H---------S--E----Y-----N-
Lysinibacillus xyleni		WP_097072189	----------V--E----FL---	Q-ENN---------S--E----Y-----E-
Maledivibacter halophilus		WP_079492156	------I---F-NE---I-ME-I	Q-EKK--F--T------E----Y----ND-
Marinisporobacter balticus		WP_132243698	---------VF-NEL-GI-YDL-	Q-ENKG----T-----------Y----NN-
Massiliomicrobiota timonensis		WP_129736441	--A----------E---VFME-V	Q-ENHA--L----SS--E----Y---V-E-
Methanocorpusculum bavaricum		WP_042697877	---------VV-EE---IFLE-I	Q-EEHA--L-----S-------YL----E-
Methanocorpusculum labreanum		WP_011833915	---------VI-EE---IFLE-I	Q-EEH---L-----S-------YL----E-
Methanocorpusculum parvum		WP_095642516	---------VV-EE---IFLE-I	Q-EEHA--L-----S-------YL----E-
Methanocorpusculum sp. MCE		RBQ24283	---------VI-EE---IFLE-I	Q-EEH---L-----S-------YL----E-
Mobiluncus mulieris		WP_004015108	---------VV--DV--IIMD--	Q-ESH-V------IS--Q-A--YVA-----
Oribacterium parvum		WP_009535475	--A----------E---V-LE-V	KQENH---L-----S--E----Y-V---N-
Paenibacillus algorifonticola		WP_046232805	--A------VV-EEM---FLE-V	--EEH---M-----S--E----Y-----N-
Paenibacillus amylolyticus		WP_133386791	---------VF-REL----ADMI	Q-E----I--T------ER---Y-----Q-
Paenibacillus antibioticophila		WP_044478465	----A-----F-REL-E--AE-I	Q-EK-TV---T-L------V--Y-----R-
Paenibacillus apiarius		WP_087432814	----------F-REL-NI-HDL-	QEEDKT-F--T-------R---Y-------
Paenibacillus borealis		WP_076109203	--A-------T-EE--EVFLE-V	--ESH---M-----S--E----F-----Q-

**Other Bacteria (1/>200)**	Paenibacillus bouchesdurhonens	WP_110930271	--A------V---D---VFLD-V	Q-EHH---M--------E-V--Y-----Q-
	Paenibacillus glacialis	WP_068538141	---------VF-RE-----ADI-	Q-ESN-----T-------Q---Y-A--NR-
	Paenibacillus glucanolyticus	WP_063480672	---------VF-REL----ADM-	Q-ERNT-I----------R---Y----NR-
	Paenibacillus graminis	WP_025703780	--A----------DM--VFLD-V	Q-ENH---M--------E-V--Y-----G-
	Paenibacillus jilunlii	WP_062524317	--A----------DM--VFLD-V	Q-ENH---M--------E-V--F-----E-
	Paenibacillus larvae	WP_104932675	----A-----F-REL----SNLI	Q-ESKTV---T---A---R---Y-----Q-
	Paenibacillus oryzae	WP_068683419	--A-------V--E--GVFLE-V	--ENH---M-----S--E----Y-----Q-
	Paenibacillus pabuli	WP_076291812	---------VF-REL----ADVI	Q-E----I--T------ER---Y-A-----
	Paenibacillus riograndensis	KWX86743	--A----------DM--VFLD-V	Q-ENH---M--------E-V--H-----E-
	Paenibacillus sanguinis	WP_018753020	--A------V--ED---VFLD-V	Q-ENH---M--------E----Y-----Q-
	Paenibacillus sonchi	WP_039835242	--A----------DM--VFLD-V	Q-ENH---M--------E-V--Y-----E-
	Paenibacillus vortex	WP_006210429	---------VF-REL----ADM-	Q-ERNT-I----------R---Y----NR-
	Peptoniphilus grossensis	WP_075658722	----------V-EE-----LDY-	Q-ENHT--I----LS--E----Y-A---E-
	Peptoniphilus lacrimalis	WP_019035018	---------VI--E---IFFE-I	K-ENS-----T---S--E----Y-S--SQ-
	Peptoniphilus obesi	WP_019131995	---------VI--D---I-LE--	--EKH---------E--E----Y--Y-ND-
	Peptoniphilus sp. oral taxon 8	WP_009345770	---------VI--E---IFFE-I	K-ENS-----T---S--E----Y-S--SQ-
	Pontibacillus chungwhensis	WP_036783589	----------F-REL-EV-RSL-	--GGKT-F--T-------S---Y-----N-
	Pseudobacteroides cellulosolve	WP_036943396	---------VA--ELM-I-SE-V	IND---V---T---S----V--Y----NN-
	Pygmaiobacter massiliensis	WP_102049397	--A-------V--E---VFLE-I	S-EQH---------S--E----YV----N-
	Robinsoniella sp. RHS	KLU73828	--A-------V--EV--IFLD-V	Q-E-HAV-V-----S--E----Y-----E-
	Roseburia inulinivorans	WP_118093828	--A------V---EM--VFLE-V	QEENH---L-----S--E-V--Y-----N-
	Roseburia sp. 1XD42-69	WP_120407036	--A------VI--D---M-LD-V	Q-EEH---V-----S--E----Y-V---E-
	Ruminiclostridium cellobioparu	WP_004625581	---------VA--EF--I-SE-I	Q-GK--V---T---S-------Y----NN-
	Ruminiclostridium cellulolytic	WP_012634675	---------VA-EEL--IMTE-I	G-GKS-V---T---S-------Y----NQ-
	Ruminiclostridium hungatei	WP_080066498	---------VA--EL-EI-SE-I	Q-G---V---T---S-------YL---NN-
	Ruminiclostridium josui	WP_024832910	---------VA--EL--IMTE-I	G-GK------T---S-------Y----NQ-
	Ruminiclostridium papyrosolven	WP_004619652	---------VA--EF--IMTE-I	G-GK------T---S-------Y----NQ-
	Ruminococcus albus	WP_074960027	--A-A----VV--E--HIFMEYL	Q-GE----M-----S--E-----V---N--
	Ruminococcus flavefaciens	WP_080770529	---------VA--EL--I-AEYI	-NEN--V---T---S-VER---YV-IL-N-
	Ruminococcus gauvreauii	WP_028529310	--A------V---D---VFLD-V	Q-ENH---M--------E-V--Y-----Q-
	Ruminococcus sp. AM40-10AC	WP_118572170	--A-------I--DV--M-ID-V	Q-ENH-V-V-----S--E-V--Y-------
	Ruminococcus sp. AM46-18	WP_119254090	--A------VI--D---M-LD-V	Q-EEH---V-----S--E----Y-V-----
	Saccharibacillus sp. O16	WP_087797676	----A----VF-REL----AEQI	Q-EN----------N---R---Y-V-VN--
	Saccharibacillus sp. O23	WP_088489738	----A----VF-REL----AEQI	Q-ER----------N---R---Y-V-VN--
	Schaalia canis	WP_124867840	--A------VV--EV--IMLE-I	Q-P-H--------VS-I--A--YVA---E-
	Solibacillus isronensis	WP_079525312	----------V--EV-GM-QN--	-QEDHA--------S--E----T-----D-
	Solibacillus kalamii	WP_087615382	----------V--EV-SM-QH--	-QEDHA-----------E----T-----E-
	Solibacillus silvestris	OBW58471	----------V--EV-SM-QH--	-QEDHA-----------E----T-----E-
	Sporanaerobacter acetigenes	WP_132995955	---------VF-RE---I-YNVI	Q-E-K--F--T------E----Y----N--
	Sporosarcina koreensis	WP_060204946	----------F-REL--I-HDL-	--EEKT-F--T-------R---Y-----N-
	Stenotrophomonas rhizophila	AXQ50855	----------F-REL-NI-HEL-	Q-GDKT-F--T-------R---Y-----N-
	Stomatobaculum longum	WP_009533352	--A----------EM--VFLD-V	Q-ERH---L-----S--E----Y-----N-
	Streptococcus sp. DD04	WP_082757326	--A----------E---V-LE-V	KQENH---L-----S--E----Y-V---D-
	Terribacillus halophilus	WP_077308215	----A-----F-REL-G--QEL-	I-GN-T-F--T-------R---Y-A-----
	Thermincola ferriacetica	WP_052217471	---------VF-NE---I-RDL-	Q-ESKGV---T--------V--Y----N--
	Thermincola potens	WP_013121529	---------VF-NEV--I-RDL-	Q-ESKGV---T--------V--Y----N--
	Thermobacillus composti	WP_015253358	---------VV-EEM---FLE-V	-NEEH---M-----S--E-V--Y-----N-
	Thermobacillus sp.	REK55937	---------VV-EEM---FLE-V	-NEEH---M-----S--E-V--Y-----N-
	Thermosyntropha lipolytica	WP_084728477	----------V--EL--I-SE-I	QNEN-AVF--T---S-------Y----NE-
	Tissierella sp. P1	WP_094903131	----------F-AE---V-YSII	Q-EKK--F--T------E----Y----N--
	Turicibacter sanguinis	WP_006783211	----------V-NE---IFLD-I	Q-EEH---I-----S--E----Y-----E-
	Ureibacillus thermophilus	QBK25683	----------V--E----FME--	QEEEN-----T---S--E----Y-----D-
	Ureibacillus thermosphaericus	WP_096550691	----------V--E----FME--	Q-EDN-----T---S--E----Y-----E-
	Vallitalea guaymasensis	WP_113671783	---------VA--N---VFQELV	--GE------T---S--E-C--Y--Y-NN-
	Viridibacillus sp. FSL H8-0123	WP_076065076	----------V--E--E-FQD--	Q-KSH------------E----Y-----E-

Supplemental Figure 35

A partial sequence alignment of the ABC transporter ATP-binding protein YtrB containing a one amino acid insertion (boxed) that is exclusively shared by all members belonging to the Jejuensis clade and absent in all other bacteria. One unnamed *Anaerococcus* species also shared this CSI.

			318		365
**Jejuensis Clade (3/3)**	Lysinibacillus saudimassiliensis	CDZ99411	RIKTAYTNVKHRLSTTTDIVDAGD		WLTQIKEQRVAFETAMDDDFNTAN
	Lysinibacillus sp. BF-4	WP_036143323	------------------V-----		------------------------
	Lysinibacillus jejuensis	WP_108305962	------A--A---TA---V-P---		-----AQ-QE---L----------
**Other Bacteria (1/>100)**	Oceanobacillus sp. YLB-02	WP_121524911	------F-LE--KASSLNL--QK-		--EK-HRFKKQ--AE---------
	Bacillus altitudinis	WP_110487777	-L--S-S-LH---ESS-NLTEDNT	E	---K-E---AT-ISE-N-------
	Bacillus amyloliquefaciens	WP_015388782	-L----S-LN---ISS-NLT-NDE	E	--AK-E-H-T---E----------
	Bacillus anthracis	WP_000152267	------G-L---MESS--LT-HNE	K	--ADLEKFQT---E--N-------
	Bacillus atrophaeus	WP_106361266	-L----S-LL---NSS-NLTADN-	E	--KK-E-H-T---E----------
	Bacillus bingmayongensis	WP_017150113	------G-L---MESS--LT-HNE	K	--AEVEKFQL---E--N-------
	Bacillus cellulasensis	WP_041091507	-L--S-A-LH---ESS-N-TEDKT	E	---K-E---AT-ISE-N-------
	Bacillus cereus	WP_098585952	------G-L---MESS--LT-HNE	N	--AELEKFQT---E--N-------
	Bacillus cytotoxicus	WP_087094329	----T-R-L---IESS--LT-HNE	K	--AEM--FQS---A----------
	Bacillus funiculus	WP_129730283	--Q---A-L---MDSS-NLTENDE	S	-ISEV-AF-E---Q----------
	Bacillus gaemokensis	WP_033677208	------G-L---MESS--LT-HNE	K	--AEMEKFQA---E--N-------
	Bacillus glycinifermentans	WP_048355831	-L----S-LQ---KSS-NLTEGDS	E	--EK-E---E--VA----------
	Bacillus halotolerans	WP_059336715	-L----S-LQ---NSS-NLTEDD-	K	--AK-E-H-T---E----------
	Bacillus haynesii	WP_076794190	-L----S-LQY--KSS-NLTEGDS	E	--EK-E---K---K----------
	Bacillus intestinalis	WP_061188091	-L----S-LQ---NSS-NLTEDD-	Q	--EKVE-H-K---E----------
	Bacillus licheniformis	WP_107661537	-L----S-LQ---KSS-NLTEGDS	E	--EK-E---K--Q-----------
	Bacillus luti	WP_071711515	------G-L---MESS--LT-HNE	K	--AEMEKFQA---E----------
	Bacillus manliponensis	WP_034642647	------G-L----ESS-NLTEHDE	K	--AE--KFQT---E----------
	Bacillus mojavensis	WP_010332757	-L----S-LQ---NSS-NLTEDD-	K	-VAK-E-H-T---E----------
	Bacillus mycoides	WP_070178726	------G-L---MESS--LT-HNE	K	--AEMGKFQ----E----------
	Bacillus nakamurai	WP_061527641	-L----S-LN---NSS-NLTEND-	E	--AK-E-H-T---E----------
	Bacillus ndiopicus	WP_042478584	--R---S--E---VSAA-L-NNSE	E	-----AAIQQQ--E--N-------
	Bacillus panaciterrae	WP_028400837	--L---H-L---MESS--LTENDE	K	-IAEV-AF-A---E----------
	Bacillus paralicheniformis	WP_130569521	-L----S-LQ---KSS-NLTEGDS	E	--GK-E---K--------------
	Bacillus proteolyticus	WP_071747345	------G-L---MESSA-LT-HNE	K	--AEMEKFQA---E----------
	Bacillus pseudomycoides	WP_098673713	------G-L---MESS--LT-HNE	K	--AEMERFQ----A----------
	Bacillus pumilus	WP_066031440	-L--S-A-LH---ESS-N-TEDNA	E	--AK-E---AT-ISE-N-------
	Bacillus safensis	WP_111927728	-L--S-A-LH---ESS-N-TEDNA	E	--AK-E---AT-ISE-N-------
	Bacillus siamensis	WP_016935910	-L----S-LN---ISS-NLT-ND-	E	--AK-E-H-A---E----------
	Bacillus sonorensis	WP_077735270	-L----S-LQ---KSS-NLTEGDS	E	--EK-E---N--------------
	Bacillus sp. 100374	WP_099685883	------G-L---MESS--LT-HNE	K	--AELEKFQT---E--N-------
	Bacillus sp. 123MFChir2	WP_020062275	------G-L---MESS--LT-HNE	K	--AEMGKFQ----ES---------
	Bacillus sp. 491mf	WP_090999071	------V-L---MESS--LT-HNE	K	--AELEKFQ----E----------
	Bacillus sp. 7705b	WP_095714846	-L----S-LQY--NSS-NLTEDD-	K	--AK-E-H-T---E----------
	Bacillus sp. AFS018417	WP_098309983	--E---G-L---MESS--LT-HNE	K	--DEMEKFQA---A----------
	Bacillus sp. AFS023182	WP_098303059	------G-L---MESS--LT-HNE	K	--VE-EKFQLT--E--K-------
	Bacillus sp. AFS029637	WP_098945958	------G-L---MESS--LT-HNE	K	--AEMEKFQ----E----------
	Bacillus sp. AFS059628	WP_098669545	------G-L---MESS--LT-HNE	K	--AELEKFQA---E----------
	Bacillus sp. BT1B_CT2	EFV69656	-L----S-LQ---KSS-NLTEGDS	E	--EK-E---K--Q-----------
	Bacillus sp. CDB3	WP_128853295	------G-L---MESS--LT-HNE	K	--AEMEKFQA---E----------
	Bacillus sp. CPSM8	ETB72041	-L----S-LQY--KSS-NLTEGDS	E	--GK-E---K--------------
	Bacillus sp. H1a	WP_025149632	------G-L---MESS--LTEHNE	K	--AEMEKFQA---E----------
	Bacillus sp. HMG	WP_116307696	-L--S-S-LH---ESS-NLTEDNT	E	---K-E---AT-ISE-N-------
	Bacillus sp. JFL15	WP_049627780	-L----S-LN---ISS-NLT-NDN	E	--AK-E-H-A---E----------
	Bacillus sp. MYb209	WP_105587622	------G-L---MESS--LT-HNE	K	--AEMEKFQ----E----------
	Bacillus sp. NSP9.1	WP_026586084	-L----S-LQ---KSS-NLTEGDS	E	--EK-E---N--------------
	Bacillus sp. Nf3	WP_107164233	-L--S-A-LH---ESS-N-TEDNA	E	--AK-E---AT-ISE-N-------
	Bacillus sp. SB47	WP_026578985	-L----S-LQY--KSS-NLTEGDS	E	--GK-E---K--------------
	Bacillus sp. SN10	WP_101168950	------G-L---MESS--LT-HDE	K	--ADLEKFQT---E--N-------
	Bacillus sp. SRB_331	WP_113735879	------G-L---MESS--LT-HNE	K	--AEMEKFQ----E----------
	Bacillus sp. SRB_8	WP_113710117	------G-L---MESS--LT-HNE	K	--AEMEKFQ-V--E----------
	Bacillus sp. TE3	WP_129506940	-L----S-LQ---NSS-NLTEDD-	Q	--EKVE-H-K---E----------
	Bacillus sp. UNC322MFChir4.1	WP_035433742	------G-L---MESS--LT-HNE	K	--AELEKFQ----E----------
	Bacillus sp. UNC69MF	WP_032870902	-L----S-LN---ISS-NLT-NDE	E	--AK-E-H-T---E----------
	Bacillus sp. VMFN-A1	WP_109567814	-L----S-LN---ISS-NLT-NDQ	E	--AK-E-H-T---E----------
	Bacillus sp. ok061	WP_103945739	------G-L---MESS--LT-HNE	K	--AELEKFQT---E--N-------
	Bacillus subtilis	WP_003225751	-L----S-LQ---NSS-NLTEDD-	Q	--EKVE-H-K---E----------
	Bacillus swezeyi	WP_076762602	-L----S-LQ---KSS-NLTEGDS	E	--EK-DG--K--------------
	Bacillus tequilensis	WP_024714035	-L----S-LQ---NSS-NLTEDD-	Q	--EK-E-H-K---E----------
	Bacillus thuringiensis	WP_098672532	------G-L---MESS--LT-HNE	K	--AELEKFQT---E--N-------
	Bacillus toyonensis	WP_100062062	------G-L---MESSANLT-HNE	K	--AEMEKFQA---E----------
	Bacillus velezensis	WP_014304214	-L----S-LN---ISS-NLT-NDE	E	--AK-E-H-T---E----------
	Bacillus wiedmannii	WP_064449385	------G-L---MESS--LT-HNE	K	--AELEKFQT---E--N-------
	Bacillus xiamenensis	WP_071168234	-L--S-A-LH---ESS-NLTEDNA	E	---K-E---AT-ISE-N-------
	Bacillus zhangzhouensis	WP_034323256	-L--S-A-LH---ESS-NLT-NNT	E	---K-E---AT-ISE-N-------
	Bhargavaea cecembensis	WP_040225933	-LR---R--L---D--A-NG-QE-	I	--HKLEGE-K--------------
	Caryophanon latum	WP_066462883	-LR-SFN-T-----V-AELG--N-	E	---K-EAV-E----S---------
	Caryophanon tenue	WP_066542780	-LR-SFN-------V-AELGAEN-	E	--AK-EAV-AS---S---------
	Chryseomicrobium excrementi	WP_100354127	--R-S----L---EHSA-LG-QD-	M	-TF-LN--IK---D----------
	Cronobacter sakazakii	PUW00845	------G-L---MESS--LT-HNE	K	--AEMEKFQ----E----------
	Edaphobacillus lindanitolerans	WP_076758776	-LR--FR--S---E--A-NG-QT-	I	--YK-GKA-K---E----------

Other Bacteria (1/>100)

Filibacter sp. TB-66	WP_124068554	--R---N-L------SA-LG-QK- V -MHKVD-VKIQ--I---------
Geobacillus sp. 44C	WP_081189182	-L--S-F-L----QSS-NLT-DD- Q --AR-Q--HE--IRE--------
Klebsiella pneumoniae	OON74969	------G-L---MESS--LT-HNE K --ADLEKFQT---E--N-------
Lysinibacillus acetophenoni	WP_097149290	-LR---L--Q---T----LL-N-- E -I-K-A-VKQQ--D---------
Lysinibacillus boronitolerans	WP_016992140	--R---N------T--VSLG-HSE E --NK-A--KAH--E---------
Lysinibacillus chungkukjangi	WP_107932255	-LR---S-IQY---AS-NLA-N-G E Y-DKLE-VKKQ--I---------
Lysinibacillus composti	WP_124766138	-LR---S-L----K----LTNQA- E Y-VK-E-VKKQ--E--------S-
Lysinibacillus fusiformis	WP_069483205	--R---N------T--VSLG-HS- E --NK-S--KAQ--E---------
Lysinibacillus macroides	WP_053997212	--R---N------T--VSLG-HTE E --SK-AD-KAL--E---------
Lysinibacillus manganicus	WP_036183218	-LR---S--Q---T----LL-N-- E -TQK-A-VKKQ--D---------
Lysinibacillus massiliensis	WP_036172387	-LR---S--Q---TA---LSEN-- E --EKVAKVKGQ--E---------
Lysinibacillus meyeri	WP_107839075	--R---S--E---A-SA-L--HA- E -----AAIQQQ--E--N-------
Lysinibacillus odysseyi	WP_036152011	-LR------Q---TA-AGLGES-- E ---K-A-VKGQ--A--N-------
Lysinibacillus parviboronicapi	WP_107950584	--R---N------TA-ASLG-NSV E --EK-AA-K-Q--E---------
Lysinibacillus sp. B2A1	AVK86718	--L---N-I----TA-ASLG-HSE Q --EK-E--KAH--E--N-------
Lysinibacillus sp. BK089	WP_132362661	--R---N------TA-VSLG-HSE E --KK-A--KAH--E---------
Lysinibacillus sp. FJAT-14222	WP_053596038	--R---N------TA-ASLG-HSE E --EK-A--KAD--E---------
Lysinibacillus sp. FJAT-14745	WP_053482830	--R---N------TA-ASLG-HSE E --EK-A--KAQ--E---------
Lysinibacillus sp. Marseille-P	WP_106781871	-LR---S------TA--NLINN-- E --AKVTDIKNQ--E---------
Lysinibacillus sp. OL1	WP_131521812	--R---N-A----T--VSLG-HSE E --NK-A--KAH--E---------
Lysinibacillus sp. SYSU K30002	WP_126658149	-L----A--EY--NASANLSSN-- E Y----NQVKS---E---------
Lysinibacillus sp. YR326	WP_134022290	--R---N------TA-ASLG-HSE E --GK-A--KGY--E---------
Lysinibacillus sp. ZYM-1	WP_054610147	--R---N------T--VSLG-HSE E --SK-A--KAQ--E---------
Lysinibacillus sphaericus	WP_010860628	--R---N------TA-ASLG-NSA E --QK-A--K-Q--E---------
Lysinibacillus sphaericus	WP_012296088	--R---N------T--VSLG-HS- E --NK-T--KAQ--E---------
Lysinibacillus sphaericus	WP_024364390	--R---N---Y---A-ASLG-CSE E --R--AQ-KAQ--E---------
Lysinibacillus sphaericus	WP_036123749	--R---N------T--ASLG-HSE E --SK-A--KAQ--E---------
Lysinibacillus sphaericus	WP_069514303	--R-S-N---Y---A-ASLG-CTE E --R--AQ-KAL--E---------
Lysinibacillus sphaericus	WP_080718177	--R---N------T--VSLG-HSN E --NK-T--KAQ--E---------
Lysinibacillus sphaericus	WP_112116889	--R---N------T--VSLG-HSE E --NK-A--KAH--E---------
Lysinibacillus sphaericus	WP_125100926	--R---Q--E---DVSA-NG-QH- I --HK-Q--Q----E---------
Lysinibacillus xylanilyticus	WP_100542797	--R---N------TA-ASLG-HTE E --GK-A--KAL--E---------
Paenisporosarcina quisquiliarum	WP_090567731	--Q---D-LV------ANLG-HD- I --QK-AAVK-Q------------
Pseudomonas sp. EGD-AK9	ERI49895	-L----S-LQ---NSS-NLTEDD- Q --EKVE-H-K---E---------
Psychrobacillus psychrodurans	WP_093495447	--Q---D-LV------ANLG-HD- I --QK-AAVK-Q------------
Psychrobacillus psychrotoleran	WP_093537883	--R---D-LV------ANLG-HD- I --QK-ADVK-Q------------
Psychrobacillus sp. OK032	WP_093274901	--R---A--L---NV-A-LG-HH- I --HK-A-VQTQ------------
Rummeliibacillus stabekisii	WP_066790970	--R-S---L----QVSANLE--DT S D-DE-EK--LD------------
Sporosarcina koreensis	WP_040285463	--R---E-IG---AVSA-NG-QH- I --HK-RQ-QIL--E---------
Sporosarcina newyorkensis	WP_040759938	--R---H--Q----MSA-LAEDPQ S --SR-D--IT---N---------
Sporosarcina psychrophila	WP_067204697	--R---N-L----A-SA-LG-QK- I -IHKVD-IKND--V---------
Sporosarcina sp. BI001-red	WP_116018380	--R---Q-IE---DVSA-NG-QQ- I -IHK-Q---T---K---------
Sporosarcina sp. D27	WP_025782836	--R---Q--E---DVSA-NG-QQ- I --HK-Q-LQN---E---------
Sporosarcina sp. HY008	WP_067407687	--Q---N-L------SA-LG-QK- I -MHKVD--IT---Q---------
Sporosarcina sp. P13	WP_099687596	--Q---N-LQ---TLSA-LAEDSQ Q -I-G-D--IT-------------
Sporosarcina sp. P17b	WP_099624838	--R---S-LQ----ISA-LAEDSE L --NK-N--IK-------------
Sporosarcina sp. P18a	WP_099675761	--Q-S-S-LQ----ISA-LAENSE S -SNK-Q--IN---K---------
Sporosarcina sp. P20a	WP_099678365	--Q-S-S-LQ---TISA-LAENSE S --NK----IN---KS--------
Sporosarcina sp. P26b	WP_099692968	--R---S-LQ----ISA-LAEDSE L --NK-N--IK-------------
Sporosarcina sp. P34	WP_099694842	--Q-S-S-LQ----ISA-LAESSE S -SNK-Q--IN---K---------
Sporosarcina sp. P7	WP_099635782	--Q-S-S-LQ----ISA-LAENSE S -SNK-Q--IN---K---------
Sporosarcina sp. PTS2304	WP_114925012	--R---N-LQ---TLSA-LA-DSQ M -VRG-E--IT-------------
Sporosarcina sp. ZBG7A	WP_039043327	--R---Q--E---DVSA-NG-QH- I --HK-Q--QA---E---------
Sporosarcina ureae	WP_085427479	--R---S-LQ----ISA-LAEDSE L --NK-N--IK-------------
Streptococcus pneumoniae	COP76403	------G-L---MESS--LT-HNE K --ADLEKFQT---E--N-------
Tetzosporium hominis	WP_094942233	--R-S----L---EHSA-LG-QD- M -TF-LN--IK---D---------

Supplemental Figure 36

A partial sequence alignment of the Cysteine--tRNA ligase protein containing a one amino acid deletion (boxed) that is exclusively shared by all members belonging to the Jejuensis clade and absent in all other bacteria. One unnamed *Oceanobacillus* species also shared this CSI.

			435		478
**Jejuensis Clade (3/3)**	Lysinibacillus sp. BF-4	WP_036144197	SIYNIVIMEEVQTIIGIGCGASSKLVD		ANGRITQFHNAKDPAAY
	Lysinibacillus saudimassiliensis	CEA03200	---------------------------		-----------------
	Lysinibacillus jejuensis	WP_108308071	---------------------------		-----------------
**Other Bacteria (1/>200)**	Bacillus massiliosenegalensis	WP_019153375	-----M----Q-----L----A--FI-		TD-K-NH-S-P---KT-
	Aeribacillus pallidus	WP_063387300	-----I----M-----L--------I-	P	VSRE--H-A-P---KS-
	Aureibacillus halotolerans	WP_133581883	-L---M----M----------A--F--	P	-T-K-QR-A-P----S-
	Bacillus acidicola	WP_066265821	-L---I---------------A--FI-	P	KT-H-Q--S-P---KS-
	Bacillus aciditolerans	WP_121447102	-----M----M-----L----A--FI-	P	MT-K----A-P---KS-
	Bacillus aerophilus	WP_041507516	-L---I---------------A--FI-	P	RT-K---YA-P---KS-
	Bacillus aerophilus	WP_129548205	-L---I---------------A--FI-	P	RT-K---YA-P---KS-
	Bacillus alkalitelluris	WP_078548981	-----M----Q-S---L----A--F--	P	VS-K----S-P-E-K--
	Bacillus altitudinis	WP_035703450	-L---I---------------A--FI-	P	RT-K---YA-P---KS-
	Bacillus anthracis	AIM10229	------------S---L-------F-H	P	KT-A--H-A-P---KS-
	Bacillus aquimaris	WP_032089285	-----M---------------A--FI-	P	KT-K--H-A-P-E-N--
	Bacillus atrophaeus	WP_106272112	-----M---------------A--FI-	P	KT-K--H-A-P---KS-
	Bacillus australimaris	WP_060699471	-L---I---------------A--FI-	P	RS-K---YA-P---KS-
	Bacillus badius	WP_063383892	-----M---------------A--FI-	R	KT-K-EH-A-P---KT-
	Bacillus boroniphilus JCM 2173	GAE46060	-L---I----Q-----L-------FI-	P	ET-V----S-P---KS-
	Bacillus cecembensis	WP_057987204	--------------L---------FIH	P	ET-K------P------
	Bacillus cellulasensis	WP_057079749	-L---I---------------A--FI-	P	RT-K---YA-P---KS-
	Bacillus dakarensis	WP_077211091	-----M----Q-----L----A--FI-	P	KT-K----A-P---KT-
	Bacillus enclensis	WP_058298173	-----M---------------A--FI-	P	KT-K--H-A-P-E-N--
	Bacillus endophyticus	WP_113750868	-----M----Q-S---L-------F--	P	TT-K--R-A-P---KS-
	Bacillus filamentosus	WP_019393921	-----M----Q-S---L-------F--	P	TT-K--R-A-P---KS-
	Bacillus firmus	WP_048010727	-----M----Q-----L-------FI-	P	ET-K--H-A-P---KS-
	Bacillus fumarioli	WP_066367956	-L---I----T-----L-------FI-	P	VT-K--H-A-P---KS-
	Bacillus glycinifermentans	WP_048353710	-----M----K----------A--F--	P	KT-K--H-A-P---KS-
	Bacillus gottheilii	WP_066447849	-----M----L----------A--FI-	P	QS-K--H-A-P---KT-
	Bacillus halotolerans	WP_106022388	-----M---------------A--FI-	P	DT-K--H-A-P---KS-
	Bacillus haynesii	WP_076793305	-----M----K----------A--F--	P	GT-K--H-S-P---KT-
	Bacillus humi	WP_057996245	-----M----M-----L----A--F--	P	VT-K----A-P---KS-
	Bacillus intestinalis	WP_087988020	-----M---------------A--FI-	R	DT-K--H-A-P---KS-
	Bacillus jeotgali	WP_079507894	-L---I----Q-----L-------FI-	P	DT-V----S-P---KS-
	Bacillus korlensis	WP_066058934	-----M----Q-----L----A--FI-	P	ST-K----S-P---KT-
	Bacillus kribbensis	WP_026692640	-----M----M----------A--FIH	P	ET-K----A-P---KS-
	Bacillus licheniformis	WP_061576259	-----M----K----------A--F--	P	RT-K--H-S-P---KT-
	Bacillus marisflavi	WP_048004415	-----M---------------A--FI-	P	ET-K--H-A-P-E-N--
	Bacillus mesonae	WP_066394616	-----M----Q-----L-------FI-	P	VS-K--H-A-P---KS-
	Bacillus mojavensis	WP_010333579	-----M---------------A--FI-	P	DT-K--H-A-P---KS-
	Bacillus ndiopicus	WP_042476736	-----A--------L---------F-H	P	DT-K------P------
	Bacillus niacini	WP_063253227	-----M----Q-------------FI-	P	LS-K----A-P---KT-
	Bacillus oceanisediminis	WP_110065464	-----M----Q-----L-------FI-	P	QT-K--H-A-P---KS-
	Bacillus onubensis	WP_099353372	-----M----M-----L----A--FI-	P	VT-K----A-P---KS-
	Bacillus paralicheniformis	WP_101561217	-----M----K----------A--F--	P	GT-K--H-S-P---KT-
	Bacillus pseudomycoides	PEM13318	------------S---L-------F-H	P	ET-A--H-A-P---KS-
	Bacillus pumilus	WP_088004012	-L---I---------------A--FI-	P	RS-K---YA-P---KS-
	Bacillus rubiinfantis	WP_042356558	-----M----Q-------------FI-	P	VS-K--H-A-P---KS-
	Bacillus safensis	WP_081124165	-L---I---------------A--FI-	P	RS-K---YA-P---KS-
	Bacillus sinesaloumensis	WP_077617126	-----M----M-----L----A--FI-	P	VT-K----A-P---KS-
	Bacillus smithii	WP_048622828	-----L----------L----A--FI-	P	DT-K-ER-A-P---KS-
	Bacillus sonorensis	WP_029419042	-----M----K----------A--F--	P	NT-K--H-A-P---KS-
	Bacillus sp. 2B09	WP_125589150	-L---I---------------A--FI-	P	RT-K---YA-P---KS-
	Bacillus sp. 2D02	WP_125587361	-L---I---------------A--FI-	P	RT-K---YA-P---KS-
	Bacillus sp. 7788	WP_095284966	-L---I---------------A--FI-	P	RS-K---YA-P---KS-
	Bacillus sp. 7884-1	WP_095248521	-----M----Q-----L----A--FI-	L	QT-K----A-P---KT-
	Bacillus sp. 7894-2	WP_095243808	-----M----Q-----L-------FI-	P	GT-K--H-A-P---KS-
	Bacillus sp. AFS002410	WP_098117174	-L---L----E-----L-------F-N	P	NTRQ--R-A-----KS-
	Bacillus sp. AFS015802	WP_098354335	-----M---------------A--FI-	P	KT-K--H-A-P-E-N--
	Bacillus sp. AFS017336	WP_098171432	-L---L----E-----L-------F-N	P	NTRQ--R-A-----KS-
	Bacillus sp. BK100	WP_132106327	-----M---------------A--FIH	P	ET-K--H-A-P---KS-
	Bacillus sp. BT1B_CT2	EFV72129	-----M----K----------A--F--	P	RS-K--H-S-P---KT-
	Bacillus sp. CC120222-01	WP_085186360	-----M---------------A--FI-	R	DT-K--H-A-P---KS-
	Bacillus sp. F56	WP_069839101	-----M---------------A--FI-	R	DT-K--H-A-P---KS-
	Bacillus sp. FJAT-27231	WP_049660605	-----M---------------A--FI-	R	KT-K-EH-A-P---KT-
	Bacillus sp. HNG	WP_116351310	-----M----M-----L----A--F--	P	VT-K----A-P---KS-
	Bacillus sp. J33	WP_026583968	-----M----Q-----L-------FI-	P	QT-K--H-A-P---KS-
	Bacillus sp. JS	WP_014663384	-----M---------------A--FI-	R	DT-K--H-A-P---KS-
	Bacillus sp. LLTC93	WP_105927830	-L---I---------------A--FI-	P	RS-K---YA-P---KS-
	Bacillus sp. LNXM65	WP_106039566	-L---I---------------A--FI-	P	RS-K---YA-P---KS-
	Bacillus sp. L_1B0_12	WP_046527376	-L---I---------------A--FI-	P	RT-K---YA-P---KS-
	Bacillus sp. Leaf406	WP_056538055	-----M---------------A--FI-	P	ET-K--H-A-P-E-N--
	Bacillus sp. Leaf49	WP_056705102	-L---I---------------A--FI-	P	RT-K---YA-P---KS-
	Bacillus sp. MKU004	WP_064564970	-----M---------------A--FI-	P	KT-K--H-A-P-E-N--
	Bacillus sp. MSP13	WP_039073430	-----M---------------A--FI-	R	GT-K--H-A-P---KS-

Other Bacteria (1/>200)

Bacillus sp. NMCN1	WP_106055867	-L---I---------------A--FI- P RS-K---YA-P---KS-
Bacillus sp. NMCN6	WP_106048901	-L---I---------------A--FI- P RS-K---YA-P---KS-
Bacillus sp. PK3_68	WP_120038136	-----M---------------A--FI- R ET-K-EH-A-P---KT-
Bacillus sp. Root920	WP_056766065	-L---I---------------A--FI- P RS-K---YA-P---KS-
Bacillus sp. SG-1	WP_006837987	-----M----M----------A--FI- P ET-K--H-A-P-E-K--
Bacillus sp. SKP7-4	WP_119548366	-----M----M----------A--FI- P ET-K--H-A-P-E-K--
Bacillus sp. SYSU K30001	WP_124565252	----------------L-------FIH P ET-V----S-P---KS-
Bacillus sp. T33-2	WP_101585503	-L---I----L-----L-------FI- P VT-K--H-A-P---KS-
Bacillus sp. TE3	WP_129506257	-----M---------------A--FI- R DT-K--H-A-P---KS-
Bacillus sp. TH007	WP_058337524	-L---I---------------A--FI- P RT-K---YA-P---KS-
Bacillus sp. UMB0893	WP_101567611	-----M----Q-S---L----A--F-- P KT-K----A-P---KS-
Bacillus sp. Y1	WP_119707113	-----M----Q-----L----A--FIE P ST-K----S-P---KT-
Bacillus sp. es.034	WP_098441151	-----M---------------A--FI- P KT-K--H-A-P-E-N--
Bacillus stratosphericus	WP_103132125	-L---I---------------A--FI- P RS-K---YA-P---KS-
Bacillus subterraneus	WP_044393828	-L---I----Q-----L-------FI- P ET-S----S-P---KS-
Bacillus subtilis	WP_122895205	-----M---------------A--FI- R DT-K--H-A-P---KS-
Bacillus swezeyi	WP_076761670	-----M----K----------A--F-- P NT-K--H-A-P---KT-
Bacillus tequilensis	WP_024712870	-----M---------------A--FI- R DT-K--H-A-P---KS-
Bacillus timonensis	WP_010283542	-----M----M-----L----A--F-- P VT-K----A-P---KS-
Bacillus tuaregi	WP_071396046	-----M----Q-----L---------- P NT-K--R-A-P---KTF
Bacillus vallismortis	WP_010329232	-----M---------------A--FI- R DT-K--H-A-P---KS-
Bacillus velezensis	WP_106080397	-----M---------------A--FIH P ET-K--H-A-P---KS-
Bacillus vietnamensis	WP_034763249	-----M---------------A--FI- P KT-K--H-A-P-E-N--
Bacillus wudalianchiensis	WP_065410853	-----M---------------A--FI- R KT-K-EH-A-P---KT-
Bacillus xiamenensis	WP_008361323	-L---I---------------A--FI- P RT-K---YA-P---KS-
Bacillus zhangzhouensis	WP_034321525	-L---I---------------A--FI- P RS-K---YA-P---KS-
Bhargavaea beijingensis	WP_092097495	----------------L-------F-H P ET--------P---S--
Bhargavaea cecembensis	WP_063183403	----------------L-------F-H P ET--------P---S--
Bhargavaea ginsengi	WP_092055704	----------------L-------F-H P ET--------P---S--
Butyricicoccus sp. 1XD8-22	RKJ14831	--------------L---------F-N P KT-K------P------
Caryophanon latum	WP_066466221	--------------L---------F-N P ET-K-W----P------
Caryophanon tenue	WP_066543994	--------------L---------F-N P ET-K-W----P------
Domibacillus antri	WP_075397103	-----M----M----------A--FI- P ET-K-E--A-P---KS-
Domibacillus epiphyticus	WP_076765502	-----M----M----------A--FI- P ET-K-E--A-P---KS-
Edaphobacillus lindanitolerans	WP_076759761	----------------L-------F-H P QT--------P------
Falsibacillus pallidus	WP_114744083	-----M---------------A--F-- P ET-K--H-N-P---KS-
Falsibacillus sp. GY 10110	WP_121679259	-----M---------------A--FIH P ET-K--H-N-P---KS-
Filibacter sp. TB-66	VDC25860	----------------V-------FI- P GT-K----Y-P------
Filibacter sp. TB-66	WP_124069718	----------------V-------FI- P GT-K----Y-P------
Jeotgalibacillus campisalis	WP_041058949	-L---L----------L----A--FI- P ET-K--H-A-P---KS-
Jeotgalibacillus proteolyticus	WP_104056570	-L---I--------V-L----A--FI- P -T-K--H-A-P---KS-
Jeotgalibacillus soli	WP_041088251	-L---I----------L----A--FI- H SS-S----A-P---KS-
Jeotgalibacillus sp. S-D1	WP_133376230	-L---I----------L----A--FI- P ET-K--H-A-P---KS-
Kurthia gibsonii	WP_121177033	-----A----------L-------F-Y A KT-K-E-Y--P-E----
Kurthia huakuii	WP_029500856	------------------------F-- A KT-----H--P-E----
Kurthia massiliensis	WP_010290306	------------------------F-- A KT-----H--P-E----
Kurthia senegalensis	WP_010307067	----------------L-------F-- A KT-----H--P-E----
Kurthia sp. 11kri321	WP_068451027	-----A----------L-------F-H A DT-K-E-Y--P-E----
Kurthia sp. 3B1D	WP_126991204	----------------L-------F-- A QT-----H--P-E----
Kurthia zopfii	WP_109348480	-----A----------L-------F-- A -T-K-E----P-E----
Lysinibacillus acetophenoni	WP_097149640	--------------L-------T-F-N P ET-K------P------
Lysinibacillus boronitolerans	WP_036075876	--------------L---------F-H P ET--------P------
Lysinibacillus chungkukjangi	WP_107936609	--------------L-------T-F-N P TS-K------P------
Lysinibacillus composti	WP_124764414	-----L----AH--L---------F-H P ET-K---Y--P------
Lysinibacillus contaminans	WP_053582072	--------------L-V-------F-- P ET-K------P------
Lysinibacillus endophyticus	WP_121214950	--------------L-------T-F-N P ET-K------P------
Lysinibacillus fluoroglycofeni	WP_107943309	--------------L---------F-H P ET-K------P------
Lysinibacillus fusiformis	WP_004230799	--------------L---------F-H P ET--------P------
Lysinibacillus halotolerans	WP_122970882	--------------L-------T-F-N P VT-K------P------
Lysinibacillus macroides	WP_053995161	--------------L---------F-H P ET-K------P------
Lysinibacillus manganicus	WP_036182690	--------------L-L-----T-F-N P ET-K------P------
Lysinibacillus mangiferihumi	WP_107895409	--------------L---------F-H P ET-K------P------
Lysinibacillus massiliensis	WP_036175842	--------------L---------F-N P KT-K------P------
Lysinibacillus meyeri	WP_107841937	--------------L---------F-H P KT-K------P------
Lysinibacillus odysseyi	WP_036157513	--------------L---------F-H P VT-K------P------
Lysinibacillus parviboronicapi	WP_107926328	--------------L---------F-H P ET-K------P------
Lysinibacillus sinduriensis	WP_036200265	--------------L-------T-F-N P DT-K------P------
Lysinibacillus sp. B2A1	AVK86116	--------------L---------F-H P ET-K------P------
Lysinibacillus sp. BK089	WP_132364043	--------------L---------F-H P ET-K------P------
Lysinibacillus sp. FJAT-14222	WP_053593328	--------------L---------F-H P ET-K------P------
Lysinibacillus sp. FJAT-14745	WP_053485753	--------------L---------F-H P ET-K------P------
Lysinibacillus sp. LK3	WP_048391787	--------------L---------F-H P ET--------P------
Lysinibacillus sp. Marseille-P	WP_106780995	--------------L-------T-F-N H ET-K-S----P------
Lysinibacillus sp. OL1	WP_131521087	--------------L---------F-H P ET--------P------

Other Bacteria (1/>200)

Lysinibacillus sp. PB300	WP_115674054	--------------L---------F-H P ET--------P------
Lysinibacillus sp. SYSU K30002	WP_126657289	--------------L-------T-F-- P KT-K------P------
Lysinibacillus sp. YLB-03	WP_118876123	-----L--------L-------T-F-N P ET-K------P------
Lysinibacillus sp. YR326	TDU96059	--------------L---------F-H P ET-K------P------
Lysinibacillus sp. YR326	WP_134024551	--------------L---------F-H P ET-K------P------
Lysinibacillus sp. YS11	WP_103118806	--------------L---------F-H P ET--------P------
Lysinibacillus sp. ZYM-1	WP_054611153	--------------L---------F-H P DT-K------P------
Lysinibacillus sphaericus	WP_010857324	--------------L-V-------F-H P ET-K------P------
Lysinibacillus sphaericus	WP_012292165	--------------L-V-------F-H P ET-K------P------
Lysinibacillus sphaericus	WP_024362316	--------------L---------F-H P ET-K------P------
Lysinibacillus sphaericus	WP_036118097	--------------L---W-----F-H P ET--------P------
Lysinibacillus sphaericus	WP_036165454	--------------L-V-------F-H P ET-K------P------
Lysinibacillus sphaericus	WP_036220494	--------------L---------F-H P ET-K------P------
Lysinibacillus sphaericus	WP_054548654	--------------L---------F-H P ET--------P------
Lysinibacillus sphaericus	WP_069514705	--------------L---------F-H P ET-K------P------
Lysinibacillus sphaericus	WP_075526998	----------------V-------FIH P ET-K----Y-P------
Lysinibacillus sphaericus	WP_099806325	--------------L-V-------F-H P ET-K------P------
Lysinibacillus sphaericus	WP_103977672	--------------L---------F-H A ET-K------P------
Lysinibacillus sphaericus	WP_125102912	-----M------S-----------FR- P KT--------P------
Lysinibacillus tabacifolii	WP_108031303	--------------L---------F-H P ET-K------P------
Lysinibacillus telephonicus	WP_126293453	--------------L---------F-N P NS-K------P------
Lysinibacillus varians	WP_025220525	--------------L---------F-H P ES-K------P------
Lysinibacillus xylanilyticus	WP_049668916	--------------L---------F-H P ET-K------P------
Lysinibacillus xyleni	WP_097074162	--------------L-------T-F-N P DT-K------P------
Mycobacteroides abscessus subs	SHR41168	-----M----Q-----L----A--FI- P ST-K----S-P---KT-
Paenibacillus sp. FSL R5-0490	WP_076262442	-----M----Q-----L-------FI- P ET-K--H-A-P---KS-
Paenisporosarcina sp. K2R23-3	WP_119883900	------------------------FI- Q QT-K---YY-P------
Quasibacillus thermotolerans	WP_039233596	-----M---------------A--FI- R HT-K-EH-S-P---KT-
Rummeliibacillus pycnus	WP_102694046	----------------L-----T---N S D--K---YY-P------
Rummeliibacillus stabekisii	WP_066789615	-----A----A-----L-----T---N N -T-K-N-Y--P-E--Q-
Sinobaca qinghaiensis	WP_120193532	-L---I----L-----L----A--W-- P ET---SR-A-P---IS-
Solibacillus isronensis	WP_079524490	--------------L---------F-H P TT-K------P------
Solibacillus kalamii	WP_087618331	--------------L---------F-H P TT-K------P------
Solibacillus silvestris	WP_065216703	--------------L---------F-H P TT-K------P------
Solibacillus sp. R5-41	WP_099425200	--------------L---------F-H P ET-K------P------
Sphingobacterium sp. A3	TAI19342	-----M---------------A--FI- R DT-K--H-A-P---KS-
Sporosarcina globispora	WP_053436912	-----L----Q-----L-------FI- P QS-K--H-A-P---KS-
Sporosarcina koreensis	WP_060210618	-L---I------------------FM- P -T-K----Q-P------
Sporosarcina newyorkensis	WP_009498304	-L---L----A-----L-------FM- P -T-KL-----P-E----
Sporosarcina pasteurii	WP_115362917	----------------V-------FIH P ET-K----Y-P------
Sporosarcina psychrophila	WP_067205904	----------------V-------FI- P ET-K----Y-P------
Sporosarcina sp. BI001-red	WP_116020222	-----M------S-----------FR- P KT--------P------
Sporosarcina sp. D27	WP_025783115	-----M------S-----------FR- P KT--------P------
Sporosarcina sp. HY008	WP_067408372	------------------------FM- P ET-K-I----P------
Sporosarcina sp. P13	WP_099689674	-L---L----A-----L-------FM- P -T-KL-----P-E----
Sporosarcina sp. P16b	WP_099673782	-L---L----A-----L-------FME P -T-KL-----P-E-G--
Sporosarcina sp. P18a	WP_099676500	-L---L----A-----L-------FME P -T-KL-----P-E-G--
Sporosarcina sp. P19	WP_099692329	-L---L----A-----L-------FME P -T-KL-----P-E-G--
Sporosarcina sp. P20a	WP_099678924	-L---L----A-----L-------FME P -T-KL-----P-E-G--
Sporosarcina sp. P26b	WP_099693733	-L---L----A-----M-------FME P -T-KL-----P-E-G--
Sporosarcina sp. P29	WP_099662526	-L---L----A-----L-------FME P -T-KL-----P-E-G--
Sporosarcina sp. P3	WP_099639373	-L---L----A-----L-------FME P -T-KL-----P-E-G--
Sporosarcina sp. P33	WP_081242725	-L---L----A-----L-------FME P -T-KL-----P-E----
Sporosarcina sp. P34	WP_099696435	-L---L----A-----L-------FME P -T-KL-----P-E-G--
Sporosarcina sp. PTS2304	WP_114924505	-L---L----A-----L-------FMN P ST-KL-----P-E----
Sporosarcina sp. ZBG7A	WP_039042575	-----M------S-----------FR- P KT--------P------
Sporosarcina ureae	WP_029054129	-L---L----A-----L-------FME P -T-KL-----P-E-G--
Streptococcus pneumoniae	COF80242	------------S---L-------F-H P KT-A--H-A-P---KS-
Thalassobacillus sp. C254	WP_054635640	-L---I----------L----A--W-E P GT-K--R-A-P---VS-
Ureibacillus thermophilus	QBK26843	---------------------A--F-N P ET-A---YY-P-E-FQ-
Ureibacillus thermosphaericus	WP_016838159	--------------L------AT-F-N P KT-K---Y--P-E-YQ-
Viridibacillus arvi	WP_053418767	----------------L-------F-N A ET-K------P------
Viridibacillus sp. FSL H7-0596	WP_076034456	----------------L-------F-N A ET-K------P------

Supplemental Figure 37

A partial sequence alignment of the coproporphyrinogen III oxidase protein containing a one amino acid deletion (boxed) that is exclusively shared by all members belonging to the Jejuensis clade and absent in all other bacteria. *Bacillus massiliosenegalensis* is the only exception which also shares this CSI.

Group	Species	Accession	153		206
Jejuensis Clade (3/3)	Lysinibacillus jejuensis	WP_108306067	FNDEYNDYTNVTGNDIAPYLTHDY	Y	AAKTGTTNSDQWIVGFSP
	Lysinibacillus sp. BF-4	WP_036145046	--PN----AY----------Q-S	-	---------------Y--
	Lysinibacillus saudimassiliensis	CEA01695	--PN----AY----------Q-S	-	---------------Y--
Other Bacteria (1/>200)	Massilibacterium senegalense	WP_074018191	-DT-L-G--T---AK--SLFRGYE	V	-G---S----S-M---T-
	Alkalibacillus haloalkaliphilu	WP_017186179	-D-RLS--LR---AS-SHA-E-E-		-G-S---EN-A-M-----
	Anaerobacillus macyae	WP_048311325	-DESL----R---SG-DHL--RPA		-------ST-S-M---T-
	Anoxybacillus gonensis	WP_009360998	-DPTL---AS---QS-I-R--RP-		-G-S---KT-S-MI-YT-
	Anoxybacillus kamchatkensis	WP_026011433	-DPTL---AS---QS-I-RI-RP-		-G-S---KT-S-MI-YT-
	Anoxybacillus vitaminiphilus	WP_111644784	-DARL----S---QT--REM-RP-		-G-S---KT-S-MI-YT-
	Aquisalibacillus elongatus	WP_124220757	-D-RLS--MS---AG--NQ--QQ-		-G-S---DP-S-MI----
	Aureibacillus halotolerans	TDQ38769	-D-RL-G--T---R-LI-L--RP-		-G------A-S-MI----
	Bacillus abyssalis	WP_078414453	-DSRL-G--T---TP--K--SRT-		-G-S---ST-S-MI-Y--
	Bacillus acidiceler	WP_088012575	--TSFSS-LP---TS-I-E-SK--		YG-----KT-S-MI----
	Bacillus aciditolerans	WP_121448550	-DEKL----A---ST-SDK--RF-		-G-S-S-DA-N-MI-Y--
	Bacillus aerophilus	WP_129547946	---QL-G--S---RT-SKD--RN-		GG-S---GA-S-MI----
	Bacillus alkalitelluris	WP_078543381	-DEKL----K---ST--DK--RH-		-G-----KT-S-MI-Y--
	Bacillus altitudinis	WP_047945769	--HQL-G--S---RT-SKD--RT-		GG-S---GA-S-MI----
	Bacillus alveayuensis	WP_044749312	-DSRL----S---QT--KDM-RP-		-G-S---KT-S-MI-YT-
	Bacillus andreraoultii	WP_033827479	-DERL-G--K---QN-I-KISRI-		-G-S-S--T-N-MI--T-
	Bacillus aquimaris	WP_032086967	-DPKL---ST---IQ-RKDA-RH-		-G-S----T-N-MI----
	Bacillus aryabhattai	WP_099000247	--QKL-G--T---KP-SK-VSRP-		-G-S---ST-S-MI-Y--
	Bacillus asahii	WP_127762239	-DETL-G--K---ST-SKQ---V-		-G-S-S-ST-S-MI-YT-
	Bacillus australimaris	WP_060698651	---QL-G--S---RT-MKD--RT-		GG-S---GA-S-MI----
	Bacillus azotoformans	WP_035195439	-DKKL-S--S---QS--NG--RL-		-G-S---KT-S-MI--T-
	Bacillus bingmayongensis	WP_017153609	---KLSG-AS---HSLL-K-SRT-		-G-S-S-ET-S-MI--T-
	Bacillus cavernae	WP_126863562	-DKKL-G--K---SS--SQ--RK-		-G-S---ET-S-MI-YT-
	Bacillus cecembensis	WP_057986664	-DPV-S--SPA--LSLRSRM--T-		---S--------ML-YT-
	Bacillus cellulasensis	WP_041091390	---QL-G--S---RT-SKE--RN-		GG-S---GA-S-MI----
	Bacillus cereus	WP_000372927	---KLSG-AS---HSLL-K-SRT-		-G-S-S-ET-S-MI--T-
	Bacillus coagulans	WP_046721733	-DKRL-G-AR---AS-VKS--RP-		-G-S-S--T-S-MI----
	Bacillus enclensis	WP_058299464	-DPKL---ST---IQ-RKDA-RH-		-G-S----T-N-MI----
	Bacillus endophyticus	WP_124051796	-DERL----T----S-NEKT--I-		GG-S---QT-S-MI----
	Bacillus fastidiosus	WP_066230826	-DKRL-S--S---QGV-NM--R--		-G-S---ET-S-M--LT-
	Bacillus filamentosus	WP_085118961	-DERL----T----S-NEKT--I-		GG-S---QT-S-MI----
	Bacillus funiculus	WP_129727436	--KKLSG-AT---QA-I-K--RP-		-G-S-S-KT-S-MI--T-
	Bacillus halosaccharovorans	WP_078432039	-DTTL----S---HS--DS--R--		-G-S---S--S-MI--A-
	Bacillus halotolerans	WP_127696376	-DTDL-G--S---RT--DR--RT-		-G-S---SA-S-MI--N-
	Bacillus humi	WP_057998159	-DEKL----A---TT-SDK--RF-		-G-S-S-DT-S-MI-Y--
	Bacillus indicus	WP_029283245	-DEQL----S---HA-I-K-SRE-		-G-S---KT-S-MI-YA-
	Bacillus intermedius	ACE75739	---QL-G--S---RT-MKD--RT-		GG-S---GA-S-MI----
	Bacillus koreensis	WP_053403758	-DPKL----S---TP-LKQ-S---		SG-S---LT-S-MI-S--
	Bacillus kwashiorkori	WP_087941722	-DERF-G-AT----S--KKM-RE-		-G-S---DT-S-MI-Y--
	Bacillus litoralis	WP_066338053	-DTAL----S---HS--DS--R--		-G-S---ST-S-MI----
	Bacillus manliponensis	WP_034635481	--KKLSS-AA---QS-L-E-S-T-		-G-S-S-ET-S-M---T-
	Bacillus marinisedimentorum	WP_066176038	-DESL----R---KP-KHK--RP-		-G-S---RA-S-MI----
	Bacillus massiliglaciei	WP_110928290	-DEAL-G--K---STV-KS-HRP-		---S-S-ST-S-MI-YA-
	Bacillus massilioanorexius	WP_019243250	--QQL-G-ST---STLI-K-SRQ-		-G-S---KT-S-MI-YT-
	Bacillus massiliogorillae	WP_042351100	--QKL-G-AS---ST-V-R--RQ-		-G-S-S-QT-S-MI-YT-
	Bacillus megaterium	WP_129705504	--QKL-G--T---KP-SK-VSRP-		-G-S---ST-S-MI-Y--
	Bacillus ndiopicus	WP_042470745	-DPI-S--SPA--VA-RGRM--T-		---S---I----L--YT-
	Bacillus onubensis	WP_099360867	-DEKL----A---ST-SDK-SRF-		-G-S-S-DA-N-MI-Y--
	Bacillus oryziterrae	WP_017756652	-DLRL----S---RA--NL--RP-		SG-S---QT-S-MI--N-
	Bacillus pseudomycoides	PEA56636	--KKLSS--S---QNLV-K-SRT-		-G-S-S-ET-S-MI--T-
	Bacillus pumilus	WP_117732386	---QL-G--S---RT-MKD--RT-		GG-S---GA-S-MI----
	Bacillus safensis	WP_126682429	---QL-G--S---RT-IKE--RT-		GG-S---GA-S-MI----
	Bacillus salsus	WP_090853479	-DEKL-N--R---RTVMDQ--RH-		-G-----RT-S-MI-Y--
	Bacillus sinesaloumensis	WP_077619952	-DEKL----S---ST-SDK--RF-		-G-S-S-DT-S-M--Y--
	Bacillus solimangrovi	WP_069716707	-DPSL----T---R--RHS--RP-		-G-S---ET-S-M-----
	Bacillus sp. 123MFChir2	WP_020059753	--KKLSS--S---QNLV-K-SRT-		-G-S-S-ET-S-MI--T-
	Bacillus sp. 171095_106	WP_028409716	--QKL-G--T---KP-SK-VSRP-		-G-S---ST-S-MI-Y--
	Bacillus stratosphericus	WP_039963005	---QL-G--S---RT-SKE--RN-		GG-S---GA-S-MI----
	Bacillus subtilis	WP_003235916	-DTDL-G--S---RT--NR--RT-		-G-S---SA-S-MI--N-
	Bacillus testis	WP_050616632	--KKW-G-AD---TTLI-R-SRQ-		-G-S---ST-S-MI-YT-
	Bacillus thermocopriae	WP_128999579	-DPTL---AS---QS-I-RI-RP-		-G-S---KT-S-MI--T-
	Bacillus timonensis	WP_010677244	-DEKL----S---TT-SNK--RF-		-G-S-S-DT-S-MI--T-
	Bacillus trypoxylicola	WP_061949087	-DRQL----S---GSV--FIKRPV		-G-S-S-ST-S-MI--T-
	Bacillus weihaiensis	WP_072578118	-DTDL----S---SS--DS--R--		-G-S---ST-S-MI-LT-
	Bacillus xiamenensis	WP_071168420	---QL-G--S---RT-TKD--RT-		GG-S---GA-S-MI----
	Bacillus zhangzhouensis	WP_034318210	---QL-G--SI--RT-MKD--RT-		GG-S---GA-S-MI----
	Caenibacillus caldisaponilytic	WP_077614165	-DPKL---AK---ESVRHL---KI		-----S--T-S-MI--T-
	Citricoccus massiliensis	WP_102414419	-DRSL-G-MA---SS--DK--RT-		-G-S------S-MI-Y--
	Fictibacillus gelatini	WP_081412712	-DERL----K---AS-SK---RPA		-G-S-S-PN-S-MI--T-
	Fictibacillus phosphorivorans	WP_066399114	-DEKL----S---SS-NH---RPM		-G-S-S-PN-S-M--Y--
	Fictibacillus sp. FJAT-27399	WP_062238243	-DEKL----K---SS-DQ---RPA		-G-S-S-PT-S-M---T-

Other Bacteria (1/>200)

Filobacillus milosensis	WP_134340446	-DSRM---MY---AS--QR---Q-	-G-S---PN-S-MI----
Geobacillus galactosidasius	WP_089098266	-DPKL----T---QS-RKEI-RP-	-G-S---ET-S-MI----
Geobacillus sp. 44B	WP_081159796	-DPKL----T---QS-RKQI-RP-	-G-S---ET-S-MI--A-
Geobacillus sp. 44C	WP_081188514	-DPKL----T---QS-RKEI-RP-	-G-S---ET-S-MI----
Geobacillus sp. WCH70	WP_015865355	-DPKL----T---QS-RKEI-RP-	-G-S---KT-S-MI--A-
Geobacillus sp. Y4.1MC1	WP_013401861	-DPKL----T---QS-RKQI-RP-	-G-S---KT-S-MI--A-
Geobacillus yumthangensis	WP_097353991	-DPKL----T---QS-RKEI-RP-	-G-S---KT-S-MI----
Geomicrobium sp. JCM 19038	GAK06976	--S-L----S----S-SHLI-RPM	-G-S-S-PN-N-MI-YT-
Gracilibacillus boraciitoleran	GAE92550	-RE-L-G-MS---AS--NK-SN--	-G-S----V-N-MI----
Gracilibacillus dipsosauri	WP_109985436	-DQ-L-G-MS---AS--DQ-----	-G-S---E--S-M-----
Gracilibacillus halophilus	WP_003471361	-RE-L-G-MP---ASVSNQISR--	---S---DG---MI----
Gracilibacillus kekensis	WP_073199601	-QE-L-G-MS---AS--NQ---E-	-G-S------N-MI----
Gracilibacillus lacisalsi	WP_083939065	-EQ-L-G-MS---AS--DK---E-	-G-S----V-N-MM----
Gracilibacillus massiliensis	WP_058308731	--E-L-G-MS---AS--NK---E-	-G-S----V-N-MI----
Gracilibacillus orientalis	WP_091484724	-QE-L-G-MS---AS-NDQ---E-	-G-S----G-N-MI--T-
Gracilibacillus phocaeensis	WP_130859505	-DESL-G-MR---AS--DE---E-	-G-S----V-N-M--Y--
Gracilibacillus timonensis	WP_078059968	-DESL-G-MR---AS--DE---E-	-G-S----V-N-M--Y--
Gracilibacillus ureilyticus	WP_089741435	-KQ-L-G-MS---AS-SNQ---E-	-G-S------S-MI----
Halalkalibacillus halophilus	WP_051189180	-DERL-G-M----SS-SDI--R--	-G-S---PN-S-MI-Y--
Halobacillus sp. Marseille-P38	WP_101842348	-DSAL-G-M----SS--GE-SRT-	GG-S---D--S-MI----
Jeotgalibacillus alimentarius	WP_041123523	-DPAL---ST---ITLLDEK-RE-	---S----T---M-----
Jeotgalibacillus malaysiensis	WP_052268763	-DPAL---ST---ITLLDEK-RE-	---S---DT----I----
Jeotgalibacillus proteolyticus	WP_104058675	-DPAL---ST---ITLLNEK-RE-	G--S--------MI----
Jeotgalibacillus salarius	WP_134379236	-DPAL---ST---ITLLDEK-RE-	---S---DT---MI----
Jeotgalibacillus soli	WP_131272595	-DPAL---ST---ITLLQEK-RE-	---S----T---MI----
Jeotgalibacillus sp. R-1-5s-1	WP_134376482	-DPVL---ST---ITLLDQK-RE-	---S--------MI-Y--
Lentibacillus sp. Marseille-P4	WP_106495996	-DRSL-G-MA---ST-VDQ---T-	-G-S------S-MI-Y--
Lysinibacillus acetophenoni	WP_097147593	-DPVF---SPA--VSLR-RM--T-	---S-S-V--N-LI-YT-
Lysinibacillus boronitolerans	WP_036078210	-DPVFS--SPA--IS-RSRM--T-	---S-S------LI--T-
Lysinibacillus chungkukjangi	WP_107932429	-DPVFS--SPA--VSLR-RM--T-	---S---V----L--YT-
Lysinibacillus composti	WP_124763462	-DPVFS--SPS--IS-R-RM--T-	---S-S-I----------
Lysinibacillus contaminans	WP_053585224	-DPVFS--SPA--IA-RSRM--T-	---S-S------LI-YT-
Lysinibacillus endophyticus	WP_121215479	-DPVFS--SPA--VSLR-RM--T-	---S---V----LI-YT-
Lysinibacillus fluoroglycofeni	WP_107942014	-DPV-S--SPA--VS-RGRM--T-	---S---I----LI-YT-
Lysinibacillus fusiformis	WP_069479959	-DPVFS--SPA--IS-RSRM--T-	---S-S------LI--T-
Lysinibacillus halotolerans	WP_122972421	-DPV-S--SPA--VSLR-RM--T-	---S---V----L--YT-
Lysinibacillus macroides	WP_053993164	-DPVFS--SPA--IS-RSRM--T-	---S-S------L--YT-
Lysinibacillus mangiferihumi	WP_107896671	-DPVFS--SPA--IS-RSRM--T-	---S-S------LI--T-
Lysinibacillus meyeri	WP_107841868	-DPI-S--SPA--VS-RGRM--T-	---S---I----L--YT-
Lysinibacillus odysseyi	WP_036153124	-DPVLS--SPA--VS-RSRM--T-	---S-S------M--Y--
Lysinibacillus parviboronicapi	WP_107923700	-DPVFS--SPA--IA-RSRM--T-	---S-S------LI-YT-
Lysinibacillus sinduriensis	WP_036200064	-DPV-S--SPA--VSLR-RM--T-	---S---V----L--YT-
Lysinibacillus sp. 2017	WP_108711487	-DPV-S--SPA--LSLR-RMS-T-	---S--------MI--T-
Lysinibacillus sp. B2A1	AVK82635	-DPVFS--SPA--IS-RSRM--T-	---S-S------LI--T-
Lysinibacillus sp. BK089	WP_132360272	-DPVFS--SPA--IG-RSRM--T-	---S-S------LI--T-
Lysinibacillus sp. FJAT-14222	WP_053595453	-DPVFS--SPA--IS-RSRM--T-	---S-S------LI--T-
Lysinibacillus sp. FJAT-14745	WP_053484498	-DPVFS--SPA--IG-RSRM--T-	---S-S------LI--T-
Lysinibacillus sp. Marseille-P	WP_106783436	-DPVF---SPA--VSLR-RM-QT-	---S-S-L----L--Y--
Lysinibacillus sp. SYSU K30002	WP_126658867	-DPV-S--SPA--VSLR-RM--T-	---S---V----L--YT-
Lysinibacillus sp. YLB-03	WP_118875054	-DPVFS--SPA--VSLR-RM--T-	---S---D-------YT-
Lysinibacillus sp. YR326	WP_134020408	-DPVFS--SPA--IG-RSRM--T-	---S-S------LI--T-
Lysinibacillus sp. YS11	WP_103117410	-DPVFS--SPA--IS-RSRM--T-	---S-S------LI--T-
Lysinibacillus sp. ZYM-1	WP_054611199	-DPVFS--SPA--IS-RSRM--T-	---S-S------LI--T-
Lysinibacillus sphaericus	WP_010857919	-DPVFS--SPA--IA-RSRM--T-	---S-S------LI-YT-
Lysinibacillus sphaericus	WP_012292711	-DPVFS--SPA--IS-RSRM--T-	---S-S------LI--T-
Lysinibacillus sphaericus	WP_024362850	-DPVFS--SPA--IS-RSRM--T-	---S-S------LI--T-
Lysinibacillus sphaericus	WP_031418462	-DPVFS--SPA--IS-RSRM--T-	---S-S------LI--T-
Lysinibacillus sphaericus	WP_036121518	-DPVFS--SPA--IS-RSRM--T-	---S-S------LI--T-
Lysinibacillus sphaericus	WP_036218165	-DPVFS--SPA--IS-RSRM--T-	---S-S------LI--T-
Lysinibacillus sphaericus	WP_036222525	-DPVFS--SPA--IS-RSRM--T-	---S-S------LI--T-
Lysinibacillus sphaericus	WP_054548574	-DPVFS--SPA--IS-RSRM--T-	---S-S------LI--T-
Lysinibacillus sphaericus	WP_069512862	-DPVFS--SPA--IS-RSRM--T-	---S-S------LI--T-
Lysinibacillus sphaericus CBAM	EWH34946	-DPVFS--SPA--IS-RSRM--T-	---S-S------LI--T-
Lysinibacillus tabacifolii	WP_108030630	-DPVFS--SPA--IS-RSRM--T-	---S-S------LI--T-
Lysinibacillus telephonicus	WP_126293246	-DPV-S--SPA--VSLR-RM--T-	---S---V-----L--T-
Lysinibacillus varians	WP_025218563	-DPVFS--SPA--IS-RSRM--T-	---S-S------LI--T-
Lysinibacillus xylanilyticus	WP_100544175	-DPVFS--SPA--IG-RSRM--T-	---S-S------LI--T-
Lysinibacillus xyleni	WP_097071637	-DPV-S--SPA--VSLR-RM--T-	---S---V----LI-YT-
Melghiribacillus thermohalophi	WP_132370715	-DESL-G-SS---AS-NHM--RT-	-G-S------S-MI--T-
Oceanobacillus bengalensis	WP_121131515	-DPKL-G--A---ST--DE--RT-	-G-S-S--A-S-MI----
Oceanobacillus damuensis	WP_084268787	-DV-L-G--A---SS-SDQ--RV-	-G-S------S-MI-Y--
Oceanobacillus halophilus	WP_121204970	-DRMLDG--S---AP--DE--RT-	-G-S---S--S-M-----
Oceanobacillus limi	WP_090872524	-DESL-G-MS---SS--DQ-SRT-	-G---S--T-S-MI-Y--
Oceanobacillus manasiensis	WP_042221378	-DR-LDG-MS---SS-SDQ-SRI-	-G-S------S-MI-Y--

Other Bacteria (1/>200)	Oceanobacillus massiliensis	WP_010648428	-DE-M-G--S---SG-TDQ--RI-	-G-S---D--S-MI-Y--
	Oceanobacillus profundus	WP_118888488	-DV-M-G--S---AT-SKE-SRL-	-G-S---D--S-MI-Y--
	Oceanobacillus rekensis	WP_087973665	-DK-L-G--S---SR--SK--KL-	-G-S-S----S-MI-Y--
	Oceanobacillus senegalensis	WP_085994006	-DRKL-G--S---SP-VEQ--RT-	-G-S-S----S-MI----
	Oceanobacillus sp. E9	WP_084822410	-DE-L----S---SA-NNQ-SNI-	-G-S---DY-S-M--Y--
	Ornithinibacillus contaminans	WP_047980915	-DESL-G-MS---SS--DK--RT-	-G---S-D--S-MI-Y--
	Ornithinibacillus halophilus	WP_072891710	-DESL-G--S---AS---L--RT-	-G-S------S-MI-YT-
	Paenibacillus sp. 7884-2	WP_095307777	-DV-M-G--S---AT-SKE-SRL-	-G-S---D--S-MI-Y--
	Paenisporosarcina sp. K2R23-3	WP_119882534	-DPVF----PS--LA-RSKQ-RP-	-G-S--------ML----
	Parageobacillus caldoxylosilyt	WP_017436918	-DPKL----T---QS-RKQI-RP-	-G-S---ET-S-MI--A-
	Parageobacillus genomosp. 1	WP_043906298	-DPKL----T---QS-RKQI-RP-	-G-S---ET-S-MI--A-
	Parageobacillus thermantarctic	WP_090948121	-DPKL----T---QS-RKQI-RP-	-G-S---ET-S-MI--A-
	Parageobacillus thermoglucosid	WP_125010747	-DPKL----T---QS-RKQI-RP-	-G-S---KT-S-MI--A-
	Parageobacillus toebii	WP_062678160	-DPKL----T---QS-RKEI-RP-	-G-S---KT-S-MI----
	Paraliobacillus ryukyuensis	WP_113867734	-DSNL-G-MH---ES---T-H-T-	-G-S---A--S-MI----
	Paraliobacillus sediminis	WP_117168389	--PNL-G-MH---ES--DQ-Q-T-	-G-S---ET-S-MI-Y--
	Piscibacillus halophilus	WP_091773122	-DNRLS--MS---GN--DQ---Q-	-G-S---PN-S-MI-Y--
	Pontibacillus chungwhensis	WP_036780101	-DESL---MT---SS--NQ--RT-	-G-S----T-S-MI----
	Pontibacillus halophilus	WP_081658140	-DESL---MR---ASVS-K--RP-	-G-S---ET-S-MI--T-
	Pontibacillus marinus	WP_051255177	-DQSL---MR---SS--ND--RT-	-G-S---RT-S-MI----
	Pseudogracilibacillus auburnen	WP_110395005	-DRRL-G-ME---SS-IDK-S-S-	-G-S---DT-N-MI----
	Salinibacillus kushneri	WP_093133028	-DTSL---SR---AG-TDQ--RE-	-G---S-DT-N-MI----
	Salirhabdus sp. Marseille-P466	WP_102026390	-DESL---ST---AS-NHM--RT-	-G---S--T-S-MI----
	Saliterribacillus persicus	WP_114351503	--E-L-G-MS---SS--NE--RE-	-G-S---DT-S-MI----
	Scopulibacillus darangshiensis	WP_132747447	-DSKL---SK---STVSKM---KM	-----S-AA-S-M---T-
	Sediminibacillus halophilus	WP_051382172	-DESL-G-MR---AS--DE--RS-	-G-S---KT-N-M-----
	Solibacillus isronensis	WP_079524604	-DPVFS--SPA--LSLRSRMS-T-	---S--------MI--T-
	Solibacillus kalamii	WP_087615697	-DPVFS--SPA--LSLRSRMS-T-	---S--------MI--T-
	Solibacillus silvestris	WP_014824750	-DPVFS--SPA--LSLRSRMS-T-	---S--------MI--T-
	Solibacillus sp. R5-41	WP_099422363	-DPV-S--SPA--LSLRSRM--T-	---S--------ML--T-
	Sporolactobacillus laevolactic	WP_023509722	-DTKL-G--K---GPV-GL---KV	-----S-ST-S-MA--T-
	Sporolactobacillus nakayamae	WP_093670811	-DKRL-G--R---SPV-GL---KV	-----S-SA-S-MA--T-
	Sporolactobacillus sp. THM19-2	WP_129928428	-DKRL-G--K---AQV-GQ---KI	-----S-S--S-MA--T-
	Sporolactobacillus sp. THM7-4	WP_130030886	-DHRL-G--K---STV-GE---QM	-----S-ST-S-MA--T-
	Sporolactobacillus sp. THM7-7	WP_130001482	-DKRL-G--K---STV-GR---TI	-----S-ST-S-MA--T-
	Sporolactobacillus terrae	WP_037563684	-DKRL-G--K---SK-SGL---KV	-----S-P--S-MA--T-
	Sporolactobacillus vineae	WP_010630647	-DTRL-G--T---APV-GQ---RI	-------AT-S-MA--T-
	Streptococcus pneumoniae	CJF94884	--QKL-G--T---KP-SK-VSRP-	-G-S---ST-S-MI-Y--
	Tenuibacillus multivorans	WP_093855424	-DKRL-G-MY---SS--DQ---E-	-G-S---P--S-MI-Y--
	Thalassobacillus cyri	WP_093044878	-DSSL-G-MR---SS--DQ-SRP-	-G-S----T-S-MM----
	Tuberibacillus calidus	WP_051263069	-DPKL----K---YSVRNL-S-KV	-----S-DT-S-M---T-
	Turicibacter sanguinis	WP_055305536	--MQQ-NHLS---LS-I-N---Q-	-G-S-S--T-S-MI-YT-
	Turicibacter sp. H121	WP_068759136	--MQQ-NHLS---LS-I-N---Q-	-G-S-S--T-S-MI-YT-
	Ureibacillus thermophilus	QBK25210	-DPVFS--SPA--VSLR-RM--T-	---S---V----LI-Y--
	Ureibacillus thermosphaericus	WP_016837289	-DPVFS--SPA--VSLRSRM--T-	---S---V----LI-Y--
	Virgibacillus dakarensis	WP_088052136	-DRSL-G-MA---SS--DK--RT-	-G-S------S-MI-Y--
	Virgibacillus profundi	WP_095653764	-DR-LDG-MA---SS--ND---L-	-G-S---D--S-MI----
	Virgibacillus senegalensis	WP_110943434	-DESL-G-MR---AS--DE--RP-	-G-S---KT-N-M-----

Supplemental Figure 38

A partial sequence alignment of the PBP1A family penicillin-binding protein containing a one amino acid insertion (boxed) that is exclusively shared by all members belonging to the Jejuensis clade and absent in all other bacteria. *Massilibacterium senegalense* is the only exception which also shares this CSI.

Group	Species	Accession	179		225
**Ureibacillus clade (12/12)**	Lysinibacillus endophyticus	WP_121213073	GIAILLKISRWYVRQLQAEKLA	M	KKKKVLVSSLPPLTKKQVGIALTL
	Lysinibacillus xyleni	WP_097074282	-------------L--------	V	------------------------
	Lysinibacillus composti	WP_124765850	--G---------Y---M---SE	K	---RI-I----------I----A-
	Lysinibacillus acetophenoni	WP_097150276	--VL-F---K------E---NS	K	----L------------IQ--I--
	Lysinibacillus chungkukjangi	WP_107934724	-------L----L----E---E	K	----N-----------------G-
	Lysinibacillus halotolerans	WP_122972143	---L--Q-----I---E---SQ	Q	----I-I---------------G-
	Lysinibacillus manganicus	WP_036187474	---L-F---K------E---NS	K	-N--T-I------S----R--IA-
	Lysinibacillus massiliensis	WP_036172709	------------I---E--RTS	Q	----I-I-----------SF-
	Lysinibacillus sinduriensis	WP_036200551	------------MH-------E	K	-R----I---------------G-
	Lysinibacillus telephonicus	WP_126294517	------------MQ--E----S	K	Q---I-I-----------------
	Ureibacillus thermophilus	QBK27105	--IL-SY--K--A---RL---D	Q	N---I-I---------------L-
	Ureibacillus thermosphaericus	WP_050988855	--VL-FN--K--A---N----N	Q	-Q----I---------------I-
**Other Bacteria (0/>100)**	Lysinibacillus boronitolerans	WP_036075309	--IL-S--AT--KK--EQ-RTT		---R----T---------------
	Lysinibacillus contaminans	WP_053585563	--VV-AN--K--KK--EQ-RTS		---RI--------------T----
	Lysinibacillus fluoroglycofeni	WP_107941754	--VM-T--AK--KE--E----S		-V-RQ-I-----------
	Lysinibacillus fusiformis	WP_096363379	--IL-T--AK--KK--EQ-RT-		---R--I-T----------V----
	Lysinibacillus macroides	WP_053993527	--IL-S--AG--KK--EQ-RI-		---R----T-----Q-------A-
	Lysinibacillus mangiferihumi	WP_107896882	--LL-S--AK--KK--EQ-R-S		N--RT-------------------
	Lysinibacillus meyeri	WP_107838477	---M-T--AG--KA--EE---S		-V-RQ-I-----------TM--A-
	Lysinibacillus odysseyi	WP_036151621	--FM-T--AL--KAR-EE----		NV--------------------G-
	Lysinibacillus parviboronicapi	WP_107926065	--LL-S--AK--KK--EQ-RTS		N---T-------------------
	Lysinibacillus sp. 2017	WP_108711231	--FM-T--AA--K---E----S		NV-----------S---I-M--G-
	Lysinibacillus sp. B2A1	AVK82210	--VV-S--AA--KKR-EQ-R--		---R----T------N--------
	Lysinibacillus sp. BK089	WP_132363609	--VL-S--AA--RK--EQ-RI-		---R----N---------------
	Lysinibacillus sp. FJAT-14222	WP_053592664	--LL-S--AT--KK--EQ-RI-		---R----N---------------
	Lysinibacillus sp. FJAT-14745	WP_053485398	--V--S--AV--RK--EQ-R--		---R----N---------------
	Lysinibacillus sp. OL1	WP_131522143	--LL-S--AT--KK--EQ-RTT		---R----T---------------
	Lysinibacillus sp. YR326	WP_134026121	--LL-S--AT--KK--EQ-RS-		---R----N---------------
	Lysinibacillus sp. ZYM-1	WP_054611329	--IL-S--A---KN--EL-RQ-		---R----T---------------
	Lysinibacillus sphaericus	WP_012292433	--IL-T--A---KK--EQ-RA-		---R----T----------V----
	Lysinibacillus xylanilyticus	WP_100546162	--V--S--AA--RK--EQ-R--		---R----N---------------
	Bacillus cecembensis	WP_057985421	--FM-S--AA--K---EE---S		-V---------K----------S-
	Bacillus ndiopicus	WP_042470134	---M-T--AQ--KA--E----S		-V-RQ-------------MM--V-
	Bacillus sp. FJAT-22090	WP_053592279	--FL-SR--I--KKE-EV---S		-R--TIL------S----------
	Bacillus sp. OxB-1	WP_041075730	A-F--S---A--KQ--EQ---N		NR----L--MAN------------
	Caryophanon tenue	WP_066544604	--VL-T---A--K-E-EK----		-V-RT-I--------R-I------
	Filibacter sp. TB-66	VDC33508	AVF--T---A--KK--EQ---N		SR----L---GD---R------G-
	Filibacter sp. TB-66	WP_124071729	AVF--T---A--KK--EQ---N		SR----L---GD---R------G-
	Kurthia huakuii	WP_035944038	--FV-SR-AL--RDV-EE--RS		---RM-I-KFTA------
	Kurthia sp. 3B1D	WP_126989583	--FV-SR-AL--RGV-EE--RS		N--RT-I-KFTA-S----YV-
	Kurthia zopfii	WP_109349866	--FT-S---V--KK--EI--E-		RR-NPIL---SK----KIAY--VI
	Paenisporosarcina antarctica	WP_134208864	--F--M---A--KA--EQ---L		-R-R--L--MAN-----I-V--G-
	Paenisporosarcina indica	WP_075619409	A-F------A--KG--EQ---L		NH-R-----FAS-------
	Paenisporosarcina quisquiliaru	WP_090566119	--FL-S---A--KK--EL---S		-R-RT-L----A-------V----
	Paenisporosarcina sp. OV554	WP_108587399	--F--M---S--KA--EQ-R-L		NR-R--L--MAN-----I-V--G-
	Paenisporosarcina sp. TG-14	WP_017379043	--F--M---A--KA--EQ---L		NR-R--L--MAN-----I-V--G-
	Paenisporosarcina sp. TG20	WP_019414878	--F--M---A--KE--EQ-R-L		NR-RI-L--MDY----------G-
	Planococcus citreus	WP_121300986	--FV-S---A--KA--ERD--E		-V--AVL------------T-
	Planococcus halotolerans	WP_112224284	--F--S------KE--EQ---Q		-V----L----EM-R----V----
	Planococcus maitriensis	WP_112233074	--FV-S---A--KA--ERD--E		-V--AVL------------T-
	Planococcus maritimus	WP_068488850	--FV-S---A--KE--ERD---		-V--AVL------------
	Planococcus massiliensis	WP_052653736	--F--S---V--KEE-AQQ--Q		-G---RL----A-------
	Planococcus plakortidis	WP_068872656	--FV-S---A--KA--ERD--E		-VT-AVL------------T-
	Planococcus rifietoensis	WP_058383724	--FV-S---A--KA--ERD--E		-V--TVL------------T-
	Planococcus salinarum	OHX48571	--F--T------KE--ER---Q		-V---FL----EM-R----VV---
	Planococcus salinus	WP_123166143	--FV-S---L--KDT-EK---S		-V---IR----EM------V--I-
	Planococcus sp. CAU13	WP_052131585	--F--SR--K--KE--EQ---Q		-V----L----EM--R---M----
	Planococcus versutus	WP_049693494	--F--S---I--KQ--EQ--RQ		-V--I-L----EM--Q-I-V--V-
	Planomicrobium flavidum	WP_088008352	-VL--TA--K--KNE-AQ---G		-R-RTVL--M-D------------
	Planomicrobium glaciei	WP_074511825	--F--S---F--KE--AK---Q		-G--A-L----A-------V--A-
	Planomicrobium okeanokoites	WP_117313569	--F--S------KE--EQ---Q		-V----L----EM-R--I-
	Planomicrobium soli	WP_106533073	--F--TR--F--KER-AQ---Q		-Q--SIL----EM-R---------
	Planomicrobium sp. MB-3u-38	WP_101802461	--F--S------KE--EQ---Q		-V----L----EM-R--I-
	Planomicrobium sp. Y74	WP_121635041	--F--S------KE--EQ---Q		-V----L----EM-R----V----
	Psychrobacillus insolitus	WP_111439971	--F--S---A--KK--EE---S		-T----L----S------V-----
	Psychrobacillus psychrodurans	WP_093495684	--FL-S---A--KK--EL---S		-R-RT-L----A-S-----V----
	Psychrobacillus psychrotoleran	WP_093537462	--FL-S---A--KK--EE---S		NR-RT-L----A-----I-V--A-
	Psychrobacillus sp. FJAT-21963	WP_056831153	--FL-SR--I--KKE-EV---S		-R--TIL------S----------
	Psychrobacillus sp. OK028	WP_093061676	--F--SR--A--K---E----S		-R-RT-L----A--NR-I-V----
	Psychrobacillus sp. OK032	WP_093272282	--F--T---A--KK--EE-S-S		---RT-L----S-S----------
	Rummeliibacillus pycnus	WP_102694407	--LL-SR--V--KK--EE-NVT		-T------N--T-----I---IG-
	Rummeliibacillus sp. POC4	WP_119416294	--VL-SR--S--KN--EE-R-T		N---T-I-N--N---R------G-
	Rummeliibacillus sp. TYF005	WP_124218216	--VL-SR--S--KN--EE-R-T		N---T-I-N--N---R------G-
	Rummeliibacillus stabekisii	WP_066791353	--FL-SR--V--KE--EK-QSQ		----TII-N--V-----I----G-

Other Bacteria (0/>100)

Solibacillus isronensis	WP_079523219	--FL-T---M--K---E---KS	-I--M-I------------M--
Solibacillus kalamii	WP_087618524	--FL-T---M--K---E---KS	-I--M-I------------M--
Solibacillus silvestris	WP_014825054	--FL-T---M--K---E---RS	-I--M--------------M--
Solibacillus sp. R5-41	WP_099422130	--FM-T--AT--K---EE---S	-V---------R----------S-
Sporosarcina koreensis	WP_060206395	--F--S---A--K---ESD-KN	NR--L-L---GD-----I------
Sporosarcina newyorkensis	WP_009497749	--VL-FR--V--KK--NE-R-G	-R--AIL-TMEN-S---IT---I-
Sporosarcina pasteurii	WP_115362382	A-F--M---A--KE--IQ--KN	NR----L--IGN-----I-V--G-
Sporosarcina psychrophila	WP_067207351	--F--M---A--KK--EQ---N	NR----L--IGN----KI----G-
Sporosarcina sp. EUR3 2.2.2	WP_024533717	--Y--M---A--KE--EQ---L	NR-R--L--MAN--N--I-V--G-
Sporosarcina sp. P13	WP_099687493	---L-S---F--KK--DE-R-G	-R----LTTMEN---N--I---I-
Sporosarcina sp. P16b	WP_099672547	---L-TR--I--KK--NE-R-G	TR----LTT-ES-S-R-ITL----
Sporosarcina sp. P17b	WP_099624714	---L-SR--I--KK--DE-R-G	-R----LTTMEN------V-----
Sporosarcina sp. P18a	WP_099674349	---L-TR--I--KK--NE-R-G	TR----LTT-ES-S-R-ITL----
Sporosarcina sp. P19	WP_099690582	---L-TR--I--KK--DE-R-G	NR----LTTMES-S-R--TV----
Sporosarcina sp. P2	WP_099631490	---L-TR--I--KK--NE-R-G	TR----LTT-ES-S-R-IT-----
Sporosarcina sp. P20a	WP_099678312	---L-TR--F--KK--DE-R-G	NR----LTTMES-S-R--T---V-
Sporosarcina sp. P26b	WP_099692533	---L-SR--I--KK--DE-R-G	-R----LTTMEN------V-----
Sporosarcina sp. P3	WP_099639587	---L-TR--I--KK--DE-R-G	-R----LTTMEN-S-R--
Sporosarcina sp. P33	WP_081242318	---L-TR--F--K---DE-R-G	-R--A-LTTMES---N--
Sporosarcina sp. P34	WP_099695078	--VL-TR--I--KK--NE-R-G	TR----LTT-ES-S-R-ITL----
Sporosarcina sp. P7	WP_099636867	---L-TR--I--KK--NE-R-G	TR----LTT-ES-S-R-IT-----
Sporosarcina sp. PTS2304	WP_114924127	---L-SR--F--KK--EE-R-G	-R----LTTMES---N--V---I-
Sporosarcina ureae	WP_085426620	---L-SR--I--KK--DE-R-G	-R----LTTMEN------V-----
Viridibacillus arvi	WP_053418234	--IV-S---A--KK--EE-R-S	---RGI-----QM---------G-
Viridibacillus sp. OK051	WP_100795174	A-IV-S---D--KK--EE-R-S	-T---II----KM------V--G-

Supplemental Figure 39

A partial sequence alignment of the methionine MFS transporter protein containing a one amino acid insertion (boxed) that is exclusively shared by all members belonging to the Ureibacillus clade and absent in all other bacteria.

Group	Species	Accession	177		229
**Ureibacillus clade (12/12)**	Lysinibacillus telephonicus	WP_126296406	PAVAMTFTICALILLPFSGNGV		SWVFTGQNFAPMVFMGLAATSLAYIFFLSGL
	Lysinibacillus acetophenoni	WP_097150370	--------L--IL-----QD--		T----E--LL--L---F---------Y-G--
	Lysinibacillus chungkukjangi	WP_107936918	--------L---L------D--		I-A-DN--LF--I--------V--LL--G--
	Lysinibacillus composti	WP_124766959	-------SL---L-----QD-I		----SS--SW--IL---G------VL--A--
	Lysinibacillus endophyticus	WP_121213449	-------S--SIM------D--		--AMDK--LF-I---------V---L--G--
	Lysinibacillus halotolerans	WP_122971811	--------L-S-M-I---QD--		K---SS--LV--L--------I---L--A--
	Lysinibacillus manganicus	WP_036184320	--------L---M-----QQ--		T--LE-G-LL--L--A----------Y-A--
	Lysinibacillus massiliensis	WP_052126258	--------L---------QD--		--I--S--LI--L-------------Y-A--
	Lysinibacillus sinduriensis	WP_036199652	--------L---L------D--		M-A-E---LL--I--------I--LL--G--
	Lysinibacillus xyleni	WP_097073563	-------S---VM------D--		--A-DK--LF-I-------------L--A--
	Ureibacillus thermophilus	QBK24661	-S-----IL---F-----QD--		T---E----L--L----F--------Y----
	Ureibacillus thermosphaericus	WP_016837663	-S--I---LS-IF-----Q---		---LNT--LW--L----F-------LY-A--
**Other Bacteria (0/>100)**	Lysinibacillus contaminans	WP_053585455	-------SL---L-----ISDG	Y	--IAQSH-LWS-F---VMC--V--LL-----
	Lysinibacillus boronitolerans	WP_016994455	-------S--T-F-----LE-G	F	--LGNVD-LWT-L---IMC--V--LL-----
	Lysinibacillus fusiformis	WP_069479872	-------SL-T-F-----LSTG	F	A-LENV--LWTIL---TMC--V--LL--N--
	Lysinibacillus mangiferihumi	WP_107897381	-------S---VL-----ISSG	F	--LAEM--LWT-I---VMC--I--LL-----
	Lysinibacillus macroides	WP_053993594	-------SL--VL-V--AWQ-G	F	--MGISE-IWT-TY---M------VL--G--
	Lysinibacillus meyeri	WP_107839148	--------L---F-V-LAWADG	W	--LAAPS-ALS--Y--IMT--V---L-----
	Lysinibacillus fluoroglycofeni	WP_107943155	-------SL---F-I-LAWSDG	V	--LAMPS-ALA-LY--IM---V--VL--N--
	Lysinibacillus odysseyi	WP_036153423	-------SL--A--A-IALMEG	V	L-ITDKG-LL--LY--IIT--I--LL--A--
	Lysinibacillus sp. 2017	WP_108711366	-------SM---F-M--AARDG	L	G-LTE-T-LSA-L--A-----V--VL--G--
	Lysinibacillus sp. FJAT-14222	WP_053594070	-------S----F-----I--G	F	N-LAVPH-LWT-L---IMC--I--LL--N--
	Lysinibacillus sp. LK3	WP_048393285	-------S----F-----IQDG	F	--LVDVH-LWT-L---IMC--V--FL-----
	Lysinibacillus sp. OL1	WP_131520690	-------S----F-----LQ-G	F	--LVNVH-LWT-L---IMC--V--FL-----
	Lysinibacillus sp. YR326	WP_134022667	-------S----F-----I-RG	F	--LAIPH-LWT-L--EIMC--V---L--K--
	Lysinibacillus sp. YS11	WP_103117365	-------S----F-----IQDG	F	--LVDVH-LWT-L---IMC--V--FL-----
	Lysinibacillus sp. ZYM-1	WP_082389548	-------SL-TIL-----LHNG	F	D-LGSMH-LWT-L---IMC--V--LL--N--
	Lysinibacillus sphaericus	WP_112117204	-------S----F-----IQ-G	F	--LVNAH-LWT-L---IMC--V--FL--R--
	Lysinibacillus tabacifolii	WP_108030680	-------S---VL-----ISSG	F	--LAEM--LWT-I---VMC--I--LL-----
	Lysinibacillus varians	WP_025218504	-------S---VL-----ISSG	F	--LAEM--LWT-I---VMC--I--LL-----
	Lysinibacillus xylanilyticus	WP_100545175	-------S----F-----I-RG	F	--LAIPH-LWT-L---IMC--V---L--K--
	Bacillus cecembensis	WP_057988049	------------F----AARDG	L	G-IYE-N-LWA-L--A--------LL--G--
	Bacillus bogoriensis	WP_026672182	--VV--CS-IL-V-LLFFFD	L	--L-QINGIVSSLYI-IV--AI-----VR--
	Bacillus clausii	WP_095304392	--FI-SFS-IV---TFFLFE	V	--LLQW--GLVVLHL--I--G---ML-TK--
	Bacillus ndiopicus	WP_042476217	--------L--IF-I-LAWSDG	I	--LGATS-TLA--Y--IM---V---L-----
	Bacillus patagoniensis	WP_078391052	--IV-SFS--L-V-IFFFYD	V	--L-QW-SMVVALHL-AI--AI--LL-AK--
	Bacillus sp. B14905	EAZ86281	-------SL-T-F-----LSNG	F	A-LENM--LWTIL---IMC--V--LL--N--
	Bacillus sp. B14905	WP_043990093	-------SL-T-F-----LSNG	F	A-LENM--LWTIL---IMC--V--LL--N--
	Bacillus sp. FJAT-22090	WP_053590976	-------SVS--L-M--FFIYD	V	--LKEAG-IGIIFYL---T--I--VLYAR--
	Bacillus sp. OxB-1	WP_041072786	S--VI-SVS-AM----LFIFE	T	EGLL--RGV-TTIYL-I---TI---L-SK--
	Bacillus subtilis	WP_029316928	-VV-M-S-IL-T-LLWQSD	I	--IL-PRGLGTSLYI--I--CA--F--VK--
	Candidatus Planktophila sulfon	WP_095673605	-T--GVA--VSF--LFTEN	P	K-I--T-G-LTVLWL--VT--IG-VL-MF--
	Chryseomicrobium excrementi	WP_100353043	------SVA--L-T--YFING	A	A-TVEPI-A-ILLYL-IGT-T---V-YTT--
	Denitrovibrio sp.	PLX71056	--VM-FGG---M---LVMND	V	---L-M-GAVT--HL-ILT-T-S-VL-AK--
	Halobacillus massiliensis	WP_082232044	--VV--LS-IF-S-LLFIYD	I	--ITE-RG--VSLHL-II--G---FL-AR--
	Jeotgalicoccus halophilus	WP_092595997	----VV--LSSIL-S--LFILD	M	--I--VEGMITGLHL-VI--GI--YL-AT--
	Jeotgalicoccus marinus	WP_026865939	----VV---SG-M-S--LFIFH	A	T----SDGLITGLYL-VIV-GI--FM-AR--
	Jeotgalicoccus nanhaiensis	WP_135097477	-S--VV--LSG-L-S--LFVFD	M	--I-SREGIIAGLHL-VV--G---YL-AI--
	Kurthia gibsonii	WP_121176456	-S------M-GV------LFLG	V	E-IGIPS-WL-LFV--V-G--I--LL--R--
	Kurthia sp. 11kri321	WP_068456307	-S------M-GV------LFLG	V	E-IGIPS-WL-LFV--V-G--I--LL--R--
	Kurthia zopfii	WP_109350188	----------G-----I-LFLG	V	--ITMPA-WL-LLV--VFG--I--LL--T--
	Paenisporosarcina antarctica	WP_134210803	-------S-S-V--M--LFIFD	S	---TEPA-IITILYL-FMT--V---LY-K--
	Paenisporosarcina indica	WP_075618556	-------SMS--M-M--ILSFD	N	T-IVDPSSISIVLYL--LT--V---LY-K--
	Paenisporosarcina quisquiliarum	WP_090569973	-----SLS--L-T--YFILD	V	--LRDAG-VGIIFYL-I-T--I--VLYGW--
	Paenisporosarcina sp. HGH0030	WP_016428414	-------SLS--M-M--LFIFD	S	---SEPS-IWTILYL-FMT--V---LY-L--
	Paenisporosarcina sp. K2R23-3	WP_119882394	S--V--S-S--F-S--FLLFP	I	G--VD-A--TTILYL-WMT-T----L--T--
	Paenisporosarcina sp. OV554	WP_108584984	-------S-S-T--M--LFIFD	S	---TEPS-I-TILYL-FMT--V---LY-T--
	Paenisporosarcina sp. TG-14	WP_017381796	-------S-S-V--M--LFIFD	S	---TEPA-ILTILYL-FMT--V---LY-K--
	Paenisporosarcina sp. TG20	WP_019412791	-------S-S-IM-M--LFIFD	S	T--LEPS-SLVILYL-IMT--I---L--K--
	Planococcus halotolerans	WP_112221322	----LI-S-A-VF-I-SLFFFD	I	-YILIPADAGIILYL-F-T------L-SR--
	Planococcus massiliensis	WP_052650567	----VI-S-S-CF----LFVLD	L	-YIADPV-IGVIAYL-IG----S--L-ST--
	Planococcus plakortidis	WP_068870248	----VI-SLSGIG----LIFLD	I	GYLAEP--LMIVAYL---------LL-S---
	Planococcus rifietoensis	WP_058382846	----VV-SLSG-G-V--LFFLD	I	GYLALP--LVIVAYL---------LL-S---
	Planococcus salinarum	TAA73612	---SLI-S-A-IF-M-SLLFLD	L	-YILIPA-VGII-YL-F--------L-SR--
	Planococcus salinus	WP_123164639	----VL-SLS-VV---CLLFFD	L	TFITAS--L-VLLYL-I---TV---L-S---
	Planococcus sp. CAU13	WP_033541666	----LI-SVA-VF-M-SLFFFD	I	-YIRIPE-VGIILYL-I-T------L-SK--
	Planomicrobium glaciei	WP_036805690	----VI-SLS--Y-M--LFFFD	L	-YIAATE-LGVILYL--G----S--L-S---
	Psychrobacillus insolitus	WP_111440554	-------SVS-VL----FFLYG	V	--LKDVG-IGVVFYL-I-T-TV--VLYAK--
	Psychrobacillus psychrodurans	WP_093495335	-----SLS--L-T--YFILD	V	--LRDAG-VGIIFYL-I-T--I--VLYGW--
	Psychrobacillus psychrotolerans	WP_093536544	-----SLS--L-T-SYFLFD	V	--LNDAG-IGIIFYL-I-T--I--VLYGW--
	Psychrobacillus sp. FJAT-21963	WP_056828920	-------SVS--L-M--FFIYD	V	--LKEAG-IGIIFYL---T--I--VLYAR--
	Psychrobacillus sp. OK028	WP_093062024	-----SLS--L-T--YFILD	V	--LKDAG-IGIIFYL-I-T--I--VLYGW--
	Psychrobacillus sp. OK032	WP_093271771	-V-----SVS--L-S--WFLYD	V	--LQSTG-VGIIFYL---T--V--VLYGR--
	Rubrobacter taiwanensis	WP_132691735	--V--SLGGVL-S--LLA-D	L	---VEPRGT-VALHL--V--AV--LL-GR--

Other Bacteria (0/>100)

```
Salinicoccus kekensis            WP_097039789    S---I-SLS----S--FFIND M --LGETNGLLVALHI-VL--A---FL-S---
Salinicoccus luteus              WP_031545828      -IV---S--M-T--LLFND Q --LM-PNGISAVLYI--FG-G---FL-AY--
Salinicoccus qingdaonensis       WP_092987220    S---I-CLS----S--LFIND M --IGETNGLLVALHI--F--T---FL-S---
Salinicoccus roseus              WP_040106235      -IV---S--M-T--LMLND Q --MI-ASGISVVLYI--FG-G---FL-AY--
Salinicoccus sp. YB14-2          WP_052254473    S---I-CLS----S--LFIND M --LGETNG-LVALHI--L--T---FL-S---
Solibacillus isronensis          WP_079523842   -------S----F---LAAKDG F G-LTE-V-IW-IL--A---------L--G--
Solibacillus kalamii             WP_087617157   -------S----F----AAKDG F G-LTE-V-IW-IL--A---------L--G--
Solibacillus silvestris          WP_014824877   -------S----F---LAAKDG F G-LTE-V-IW-IL--A---------L--G--
Sporosarcina koreensis           WP_060204969   -S--VI-SVS-IM-M--LFIFE T EGLL-VPGIST-IYL-I----V---L-ST--
Sporosarcina pasteurii           WP_115359778   ----VI-S-S--M-F--LFIFE T EGL---RGI-VVL-L-F-T--I---L-ST--
Sporosarcina psychrophila        WP_067213566   ----VI-SMS-IM-M--LFLFE T EGLM--RGISVVLYL-IVT--V---L-SA--
Sporosarcina sp. EUR3 2.2.2      WP_024535724   -------S-S-I--M--LFVFD S ---TEPS-I-TILYL-FMT--V---LY-T--
Tetzosporium hominis             WP_094944520    ------SMA--L-T--YFING A A-TVEPI-A-ILLYL-IGT-T---V-YTT--
Viridibacillus arvi              WP_053418038   -------S-S--L-C-I-LVYG I E--GQSA-WL-LA---VFG--I--LL--T--
Viridibacillus sp. FSL H7-0596   WP_076034261   -------S-S--L-C-I-LIYG I E--GQ-A-WL-LA---VFG--I--LL--T--
Viridibacillus sp. OK051         WP_100794987   -------S----L-S-IALIYG I E--GQSA-WL-LL----FG--I--LL--T--
```

Supplemental Figure 40

A partial sequence alignment of the methionine EamA family transporter protein containing a one amino acid deletion (boxed) that is exclusively shared by all members belonging to the Ureibacillus clade and absent in all other bacteria.

Group	Organism	Accession	260		320
**Ureibacillus clade (11/12)**	Lysinibacillus endophyticus	WP_121213400	LPIYGILAGGAPSVWRAVTVVELLLISR		LKWRLSVDDALSITFILFVFIEPWSIFQIGFQL
	Lysinibacillus acetophenoni	WP_097148649	-----M------------I-A-II-LTQ		-YKKIP-----A-S--F--L------Y------
	Lysinibacillus chungkukjangi	WP_107934660	----AL-----------------IM---		--GK-AAA-----S--F--YL-----Y------
	Lysinibacillus composti	WP_124762164	----A-----T-----S-LM---VVLI-		Y-SH-T-----CVS--C--LL-----Y-V----
	Lysinibacillus manganicus	WP_081976220	-----V-----------L---FI-LTQ		KFKK--M----A-S--F---L-----Y-V----
	Lysinibacillus massiliensis	WP_036176149	--V-A------------S----I-LT-		F----PI----A-S--A--ML-----Y------
	Lysinibacillus sinduriensis	WP_036202311	----AV-----------M----IM---		-RGK-AA------S-----LLQ---VY------
	Lysinibacillus telephonicus	WP_126292796	----A--V---------S----TIL--		I----P-----A-S----II-----VY------
	Lysinibacillus xyleni	WP_097074616	----------------------I-L--		V------------S-----LL-----Y------
	Ureibacillus thermophilus	QBK27186	--L-A--S---------A-----ML--		--H---M----A-S--G--LL--GV-Y------
	Ureibacillus thermosphaericus	WP_050988846	--L-A-V------------L--II---		Y-R--AI----A-S--G--M----V-Y-V----
	Lysinibacillus halotolerans	WP_122971369	----------------I-I--IMM---	I	KGNK-----C-A-S-----WL---V-Y-V----
**Other Bacteria (1/>100)**	Butyricicoccus sp. 1XD8-22	RKJ43193	--V-A------------S----I-LT-		F----PI----A-S--A--ML-----Y------
	Lysinibacillus boronitolerans	WP_036076263	-----VI----------S----M-LL-	F	F--V--M----A-S--G--LL--GV--------
	Lysinibacillus contaminans	WP_053582763	--V--FI----------S---FVMLM-	Y	V---VPI----A-S--G--LL--GV---V----
	Lysinibacillus fluoroglycofeni	WP_107942457	--T-A-V------I----L---IVMLA-	Y	F-----AL--MA-S--V--IWQ-AVLY------
	Lysinibacillus fusiformis	WP_096365199	-----VI----------S----MMLL-	Y	---A--I----A-S-TG--LL--GV--------
	Lysinibacillus jejuensis	WP_108308341	--L-AM-------------TV-V-AVQ	L	K-R-IA-----AC---F--LWH-Y----V-L--
	Lysinibacillus macroides	WP_053996723	--T--VI----------S----I-LL-	Y	---AITI----A-S--G--LL--GA---V----
	Lysinibacillus meyeri	WP_107838788	--A-AL-----------L---VVMLA-	Y	---Q--AL-----C----LIWQ-AVFY------
	Lysinibacillus odysseyi	WP_036156829	--V-AL-----------L----V-LAK	Y	FR-H-PM------S-----LW--G--Y------
	Lysinibacillus parviboronicapiens	WP_107951113	--M--------------S---FV-LM-	Y	V--QVPI----A-S--G-ILL--GV---V----
	Lysinibacillus saudimassiliensis	CDZ99981	--L-AM-------------TV-V-VLQ	L	KQR-IA-----AV-L-G--LWQ-S--L-V----
	Lysinibacillus sp. 2017	WP_108713322	--M-AV-------I----S---FIMLAQ	Y	FRLKIPM-----LS--F--LL--GA---V----
	Lysinibacillus sp. AC-3	SKC03528	-----VI----------S----IMLM-	Y	FR-HV-I----A-S--C--LL--GV-Y------
	Lysinibacillus sp. AR18-8	WP_066036469	--V--VI----------S----MMLL-	F	F--V--I----A-S--G--LL--GV--------
	Lysinibacillus sp. B2A1	AVK84236	-----V-----------S----I-LM-	H	A-LQ-PI----ALS--G--LL--GV--------
	Lysinibacillus sp. BF-4	WP_036144532	--L-AM-------------TV-V-VLQ	L	KQR-IA-----AV-L-G--LWQ-S--L-V----
	Lysinibacillus sp. BK089	WP_132356902	-----VI----------S----IMLM-	F	AR-QI-I----A-S-LC--LL--GV--------
	Lysinibacillus sp. FJAT-14222	WP_053596245	-----VI----------S----IMLM-	Y	AR-QI-I----A-S-LC--LL--GV-Y------
	Lysinibacillus sp. FJAT-14745	WP_053484725	-----VI----------S----IMLM-	Y	AR--I-I----A-S--C--LL--GV-Y------
	Lysinibacillus sp. LD79	SCY66645	-----VI----------S----MMLL-	Y	---E--I----A-S-TG--LL--GV--------
	Lysinibacillus sp. LK3	WP_048392156	--V--VI----------S----MMLL-	F	F--V--I----A-S--G--LL--GV--------
	Lysinibacillus sp. OL1	WP_131520523	--V--VI----------S----MMLL-	F	F--V--I----A-S--G--LL--GV--------
	Lysinibacillus sp. PB300	WP_115674129	--V--VI----------S----MMLL-	F	F--V--I----A-S--G--LL--GV--------
	Lysinibacillus sp. SG8	SCX70120	-----GI----------S----MMLL-	Y	---E--I----A-S-TG--LL--GV--------
	Lysinibacillus sp. SG9	SCY74158	--V--VI----------S----MMLL-	F	---T--I----A-S--G--ML--GV--------
	Lysinibacillus sp. YR326	WP_134018547	-----V-----------S----IMLM-	Y	AR-QI-I----A-S-LC--LL--GV-Y------
	Lysinibacillus sp. YS11	WP_103118465	--V--VI----------S----MMLL-	F	F--V--I----A-S--G--LL--GV--------
	Lysinibacillus sp. ZYM-1	WP_054609263	-----VI----------S---IMMLL-	Y	---K--I----P-S-VG--LL--GV-Y------
	Lysinibacillus sphaericus	WP_054550074	--V--VI----------S----MMLL-	F	F--V--M----A-S--G--LL--GV--------
	Lysinibacillus tabacifolii	WP_108029823	--M--V--------V---S----VMLM-	F	AR-Q-P-----A-S--G--LL--GV---L----
	Lysinibacillus varians	WP_038509818	--M--V--------V---S----VMLM-	F	AR-Q-P-----A-S--G--LL--GV---L----
	Lysinibacillus xylanilyticus	WP_100546032	-----V-----------S----I-LM-	Y	AR-QI-I----A-S-LC--LL--GV-Y------
	Bacillus cecembensis	WP_057988270	--M-AV-----------S---FVMLAQ	Y	ARLKIPI-----LSL-V-LC---GA---V----
	Bacillus fordii	WP_018705866	--G-AV-S-AN-P-I---IMAI---S-K	K	WRMP--TL-IF--S----LLFD-MLVYH-----
	Bacillus fortis	WP_120068645	--G-AV-S--N-P-I---IMAI---S-K	K	WRLP--TL-IF-LS----LIFD-LL-YH-----
	Bacillus freudenreichii	WP_126431842	----AV-S--N-P-I---IMAL---V-K	K	WRLS--TL-TF-LS----LLFD-FL-YN-----
	Bacillus galactosidilyticus	WP_064467691	--V-ALIT-AN-P-I--TLMMF---SAK	K	WRLPFTTM-TIA-S-L--IMFN-YI-Y-A----
	Bacillus lentus	WP_066137351	----T--S--N-P-I---IMSL---AKQ	K	WRLPYTTF--F--S--V-L-FD-YL-YHV----
	Bacillus megaterium	WP_026681206	--V-ALM---E------SIMAIFFI-LH	K	T--KI--T-V------VLILFDKLI-YHV----
	Bacillus ndiopicus	WP_042478155	--A-ALI----------L---VVMLA-	Y	-----GAL-----C--V--LW--GAFY------
	Bacillus niameyensis	WP_062104624	--L-AVIS--N-P-I--IIMTL---S-Q	R	WRFP-TTL-T--LS--V-LLFD-YVVYNV----
	Bacillus sp. 3-2-2	WP_126051265	----AV-S--N-P-I---IMAL---V-K	K	WRLS--TL-TF-LS----LLFD-FL-YN-----
	Bacillus sp. B14905	EAZ83739	-----VI----------S----MMLL-	Y	---A--I----A-S-TG--LL--GV--------
	Bacillus sp. FJAT-22090	ALC84911	----AL----------S-S-T-IV---M	A	YRKKIAM---F-LSI-G----S--VVY------
	Bacillus sp. FJAT-22090	WP_082355339	----AL----------S-S-T-IV---M	A	YRKKIAM---F-LSI-G----S--VVY------
	Bacillus sp. OxB-1	BAQ11507	--F-AV-----------S-TVMV-LAA	S	GRL-IRL---V--SA-V-ILYQ-FVV--P----
	Bacillus sp. OxB-1	WP_070098213	--F-AV-----------S-TVMV-LAA	S	GRL-IRL---V--SA-V-ILYQ-FVV--P----
	Bacillus sp. VT-16-64	WP_077110613	--L--FVS--N-P-V---LMAL-IFSAK	R	WRLP--TL-----S-L--L-VD-YVVYH-----
	Bacillus terrae	WP_120114910	--V-AV-S--N-P-I---IMAI-I-SAN	R	WRLP--TL-IF-LS-M--LLFD-LL-YH-----
	Caryophanon latum	WP_083995150	--V--V-----------SF--IA--AQ	F	FRKP-PLLTIIAASCLAYLC-N-GTL--V----
	Caryophanon tenue	WP_083998145	--V--V-----------SF--IA-LLQ	F	-RKPIPLSSIIC-S-LGY-LVN-GVV-------
	Kurthia gibsonii	WP_131004912	--T-A-----------S-IM-LI-YVAL	L	FNH--K-ES--GLC-LF--L---NI-Y-V----
	Kurthia huakuii	WP_081715014	--T-A----SS------AIMTMIVVLLQ	M	RQTK-TAA---AL---G--LYN-SVVY-V----
	Kurthia massiliensis	WP_010288005	--T-A----ST------AMMTLIV--VQ	F	KQTKI-AA---ALS-L---LYR-AV---V----
	Kurthia senegalensis	WP_010303440	----AV----------S-MMTVIV-LIQ	M	RSEK-TAS--I--S--V--WLQ-F----V----
	Kurthia sibirica	WP_109306538	--L-ALFV----------MM-L-I-LAK	K	-EHV-TLEN-IA-S--I--LVS-N--Y------
	Kurthia sp. 11kri321	WP_082701388	--T-A-----------S-IM-LI-YGAL	L	FNH--K-ES--GLC-L---L---NI-Y-V----
	Kurthia zopfii	WP_126344131	--V-AL-V----------LT-LV---IS	S	FTRKP-LLTS--LC--I--SV--N---------
	Lentibacillus amyloliquefacien	WP_068443068	--L-AV----E---M--SMM-L-FM-AG	K	-N-KF--T-VI--V-L-LILTN-LI-YH-----
	Lentibacillus halodurans	WP_090232045	--C-ALI---E---L--S-M-V-F-LAG	K	MN-KF--T-V---V-M-LIVAD-YMLYN-----
	Lentibacillus jeotgali	WP_010531051	--L-ALI---E---L--SAM-L-FM-AN	K	IN-KF--T-V---V--TLILWN-YILYH-----
	Lentibacillus persicus	WP_090081261	--L-AL----E---M--SMM-L-FI-AG	K	MN-KF--T-VI--V-L-LI-MN-YI-YHV----
	Lentibacillus salicampi	WP_135108858	--F-ALI---E---L--SAM-L-FMLAN	K	MN-KF--T-V---V--MLTLLD-YMLYH-----
	Lentibacillus sp. Marseille-P4	WP_106497088	--F-A-V---E------SVMAMVFIVLA	K	--LQY--T-----V-F-LILANKYIVYSV----
	Oceanobacillus arenosus	WP_115772984	--V-------E------CVM-M-FILIN	E	F-LKF-IT-V------ILILVDKYI-YHV----
	Oceanobacillus bengalensis	WP_121128503	--V-AL----E------SLM-MCFILLS	K	--VTY--T-I---V---LIL---HV-YHV----
	Oceanobacillus damuensis	WP_067727358	--V-AL----E------SVM-VAFILFN	K	YRFKF-YT-V---V-L-LILFDKYIVYHV----
	Oceanobacillus iheyensis	WP_106896981	--A-AVV---E--I---SMM-VCFILL-	K	F-RAF-YT-V--FV-L---L-N-YMVYHV----

Other Bacteria (1/>100)

```
Oceanobacillus manasiensis        WP_042223402   --G-AVV---E--I---SMM-VCFV-L- K -NSTFNYT-V--FV--S--ILN-YLVY-V----
Oceanobacillus massiliensis       WP_010650959   --V-AL----E------ASMIIF-IFLN K SRF-F-YT-I---V-L-LILFDKHIVYH-----
Oceanobacillus picturae           WP_036572482   --A-AVV---E--I---SMM-LCFILL- K F-RAF-YT-V--FV-L---L-N-YMVYHV----
Oceanobacillus rekensis           WP_087971733   M-V-AM----E------TVM-VIFILLN K Y-FTF-YT-V---V-L-LILFDAYIVYHV----
Oceanobacillus senegalensis       WP_085991851   ----A-----E------SVM-IMVILLH K -RF-F-AL-VF-FA--VLI-LN-YFVY-V----
Oceanobacillus sp. 160            WP_114916803   --V-------E------CMM-MIFILIN Q F-LKF-II-V------FLILADKYI-HH-----
Ornithinibacillus californiens    WP_047986344   --V-AV-----------SLM-IIVILIQ K --VK-T-T-II---CLALIMAD-YI-Y------
Ornithinibacillus scapharcae      WP_081472409   --V-AL----E------SLMIL-VI-LQ K F-QKF-IS-VI----LGLLLVN-YYMYHV----
Paenisporosarcina antarctica      WP_134209527   ----A--V---------G---I--L-K Q MNK---IE--FALSILFSI--Q-GVLL------
Paenisporosarcina indica          WP_075618252   --V-AC-V---------G---II---Q Q FRR--ALE--FA-SILVYILLQ-GVLL------
Paenisporosarcina quisquiliaru    WP_090563868   --LFAL----------S-S-T-IV---T L FQRKIPIE--F-LSIMG---LT--I--------
Paenisporosarcina sp. HGH0030     WP_016426869   --V-AC-V---------G---I--L-K Q -HK--A----FA-SLLI-IYLQ-GVLL-V----
Paenisporosarcina sp. TG-14       WP_017380160   ----A--V---------G---I--L-K Q MNK---IE--FALSLLFSI--Q-GVLL------
Paucisalibacillus globulus        WP_096270705   ----A-----E---L--CSM-I-VILI- K IRLK---T-VI-V--L-LIAMDKYI-YHV----
Planococcus antarcticus           WP_081487825   --L-AVI-----------SM-TVV-AGK L FGF--PIASVMLTSI-V-ILWN-YAMYK-----
Planococcus halotolerans          WP_112223792   --F-AL------------SMTAAV-VF- L F-I---IAYI-LLS--F-IVVD-YV-YK-----
Planococcus salinus               WP_123163751   --M-AVV-----------SM-LTV-VAS L FGA--PIAQV-LLSLCA--LLD-YVMY------
Planococcus sp. CAU13             WP_052131671   --L-AL------------SMTSAV-LL- L IGA-VPAAHI-LVC-TV-IIL--YV-YK-----
Planococcus sp. Y42               WP_077590933   --L-AF----S---I---SMA--V--L- L VGT-I--MPV-LAS--T-ILVD-YI-Y------
Planococcus versutus              WP_083553675   --L-A-I-----------SM-SVV-LGK L FNFKFPLANIMLTSM-V-ILWN-YIMYK-----
Planomicrobium okeanokoites       WP_117312163   --F-AL------------SMTAAV-AF- L FRI-MPIAFI-LLS--F-ILLD-YV-YK-----
Planomicrobium soli               WP_106532764   --M-AVV---------S-GM-SAI-LC- L -GLKMP-ANV-L-S--C-LLWD-Y-LYM-----
Planomicrobium sp. MB-3u-38       WP_101803726   --F-AL------------SMTAAV-AF- L FRI-MPIAFI-LLS--F-ILLD-YV-YK-----
Planomicrobium sp. Y74            WP_121633941   --F-AL------------SMTSAV-AF- L F-FS-PIAYI-LLS----IAVD-YV-YK-----
Pontibacillus litoralis           WP_036831052   --M-AVI-------L---SMA-MA-LLT F TNV-IPLV-V--A-M---LL-N-LIA--L----
Pseudogracilibacillus auburnen    WP_110395458   ----A----SQ------SLMIVFVIFIN K I-LKYNYT-I---V-L-LII--KYIVYH-----
Psychrobacillus insolitus         WP_111437842   --L-AF----------S-S-T-IV---M L C-KK-AM---F-LSV-G--WLN--V--------
Psychrobacillus psychrodurans     WP_093496128   --L-AL----------S-S-T-IV---V L FQRKIAIE--F-LS-MG---LT--I--------
Psychrobacillus psychrotoleran    WP_093538543   --L-AL----------S-S-T-IV---M L -QRKIAI---F-LSI-G---LT--I--------
Psychrobacillus sp. FJAT-21963    KQL34180       ----AL----------S-S-T-IV-L-M A YRNKIAME--F-LSI-G----S--IVY------
Psychrobacillus sp. FJAT-21963    WP_082461048   ----AL----------S-S-T-IV-L-M A YRNKIAME--F-LSI-G----S--IVY------
Psychrobacillus sp. OK032         SER83901       --L-AFI---------S-S-T-II---L M F-KK-AI---F-LSI-G--LLT--V--------
Psychrobacillus sp. OK032         WP_093267147   --L-AFI---------S-S-T-II---L M F-KK-AI---F-LSI-G--LLT--V--------
Rummeliibacillus pycnus           WP_102691267   ----ACI---------S-L---MVML-K L WSSK--I----ALS--GL-LYQ-SI--------
Rummeliibacillus sp. POC4         WP_119414859   --L-ACI---------S-----MIM--K I -SKK--I-----LS--AL-LYQ-SI--------
Rummeliibacillus sp. TYF005       WP_124217190   --L-ACI---------S-----MIM--K I -SKK--I-----LS--AL-LYQ-SI--------
Rummeliibacillus stabekisii       WP_066786250   --L-AC----------S--M--IMM--- A S-SL-T-------S---LLAYQ-AM---V----
Solibacillus silvestris           WP_065215630   --L-AV-------I----S----IMLAQ Y FR-KIPI------S--M--LL--GAV--V----
Solibacillus sp. R5-41            WP_099424617   --L-AMM-----------N----VMLAH F -RV-IPI-----MSM-A-LC---GA---V----
Sporosarcina ureae                WP_085428901   --T-ACI----------A--TMIV-LAV T GNL--RL----AMSALF-IVMQ-HIV--P----
Terrabacteria group               WP_088050701   --L-A-I---E------CVM-M-FI-LQ K --L-Y-IT-----V-L-LILSDKYIVYH-----
Virgibacillus alimentarius        WP_029266834   --L-AL----E------SLSIMIFI-LN K A-LKFNYT-V---V-L--ILFDKYIVYHV----
Virgibacillus indicus             WP_094883512   --V-AVI---E-----SA-M-L-FI-I- K IRLDF--T-I---V-L-LILVDKYIVYHV----
Virgibacillus salinus             WP_092492504   --L-ALI---E------SSM-L-F-LIN K VRYKF--T-V---V---QILMDKYI-YS-----
Virgibacillus soli                WP_057983012   --V-ALIT-AN-P-I--TLMMF---SAK K WRLPFTTM-TIA-S-L--IMFN-YI-Y-A----
Virgibacillus sp. Bac332          WP_121605285   --V-ALM---E------SIMAIFFI-LH K T--KI--T-V------VLILFDKLI-YHV----
Virgibacillus subterraneus        WP_092503723   --L-ALI---E------SSM-L-F-LIN K VRYKF--T-V---V---QILMDKYI-YS-----
Viridibacillus arenosi            WP_038187188   ----AC-----------S----VML-- I VSN--AI----ALS--G--LLQ-SVV-------
Viridibacillus arvi               WP_053416417   ----AC-----------S----VM--- I VSNH-AT----ALS--G--LLQ-SVV-------
Viridibacillus sp. FSL H7-0596    WP_076034368   ----AC-----------S----VML-- I VSN--AI----ALS--G--LLQ-SVV-------
Viridibacillus sp. FSL H8-0123    WP_076064900   ----AC-----------S----VML-- I VSN--AI----ALS--G--LLQ-SVV-------
Viridibacillus sp. OK051          WP_100794314   ----ACV----------S----VM--- I VSN--AI----A-S--G--LLQ-SVV-------
```

Supplemental Figure 41

A partial sequence alignment of the DNA internalization-related competence protein ComEC/Rec2 containing a one amino acid deletion (boxed) that is exclusively shared by all members belonging to the Ureibacillus clade and absent in all other bacteria. *Lysinibacillus halotolerans* does not share this CSI despite branching within the Ureibacillus clade in phylogenetic trees. *Butyricicoccus* sp. 1XD8-22 is the only exception which shares this CSI despite not belonging to the Ureibacillus clade.

Group	Organism	Accession	23	Site	62
**Meyeri Clade (3/3)**	Lysinibacillus meyeri	WP_107841432	PIIPSVLFIAAGFILYGLFYTF		ALPWWFWVIEVLFVVLLF
	Lysinibacillus fluoroglycofenilyticus	WP_107942522	--------------V--V----		S------------A---
	Bacillus ndiopicus	WP_042473978	--------------V-------		------------------
**Other Bacteria (0/>200)**	Aeribacillus pallidus	WP_130156339	--L--A-L-V-A-FI--ICFS-	S	Q-TIS---V-SM-II---
	Anaerobacillus macyae	WP_048308885	----A---LYI--MM----FE-	A	PMTIY----QL-LT----
	Bacillus abyssalis	WP_078413783	-------------L---V-FS-	E	PFGFL--SVQIVLIIA--
	Bacillus acanthi	WP_108670787	---------FG-IL---V-FS-	E	PFN-F--TVQGT--IM--
	Bacillus acidicola	WP_066262161	--------LV-A-V---V-F--	S	P-H-I--AVQI---A---
	Bacillus acidiproducens	WP_018661494	----GM--LFGAYL---ICFS-	A	P-H-L--AV-I-LGI---
	Bacillus altitudinis	WP_073413913	------V-MVL--------FS-	E	K-TLT--L-QAM-TAV--
	Bacillus aquimaris	WP_071619308	--------LL-S-L---V-FD-	S	PFN-L--TVQ----L---
	Bacillus aryabhattai	WP_063248508	--------V-L--VI-----D-	S	FYS-L----QG-L--A--
	Bacillus asahii	WP_127761385	---------F-------F--S-	E	PFH-L--T-QI---L---
	Bacillus atrophaeus	WP_010789222	------V--V---V---FLF--	S	PYSYM--LV-AV-AAV--
	Bacillus australimaris	WP_060697357	------V-MVL------F-FS-	E	P-TLT--L-QAM-TAV--
	Bacillus butanolivorans	WP_116820718	-V-------FG-IL------S-	E	PFG-F--TVQI---I---
	Bacillus camelliae	WP_101354062	---------LGA-----ILF--	H	P-N-L--S--I--I----
	Bacillus campisalis	WP_046526328	----G----F---V---V-FS-	E	-FN-L--TVQ----I---
	Bacillus cavernae	WP_126865182	---------FG-IL---FLF--	E	QFN-I----QI-L-I---
	Bacillus cecembensis	WP_057984097	------I--VG---A-----S-	A	D------L--I---I---
	Bacillus cellulasensis	WP_041091011	------V-MVL------F-FS-	E	K-TLT--L-QAM-TAV--
	Bacillus cihuensis	WP_028391807	---------LG-IL---I--S-	E	PYN-G--T-QI-L-L---
	Bacillus circulans	WP_095320793	------I--L--YL-----FS-	E	E-SIL--I-QGM--I---
	Bacillus cohnii	WP_066421039	------F--VGA-L---FAFS-	A	S-NI---LFQGV--L---
	Bacillus cucumis	WP_101651285	--------LL---L---VLFS-	E	PFN-L--L-QG-------
	Bacillus deserti	WP_101640659	---------IG-IL-----FS-	D	PFSGL--AVQI-L-I---
	Bacillus drentensis	WP_066257645	--V-----LL---L---V-FN-	H	PFQ-Y--L-QG--I----
	Bacillus endophyticus	WP_061805939	---------I--YL-----FS-	E	QFSIL----QAM--I---
	Bacillus fastidiosus	WP_066225166	------V--VG--L-----FD-	K	SFNYL--I--G---AV--
	Bacillus filamentosus	WP_026009729	---------I--YL-----FS-	E	QFSIL--I-QAM--I---
	Bacillus firmus	RBP94131	--------LLG------ILFS-	E	PFN-L--T-Q----L---
	Bacillus foraminis	WP_121611776	-V-------IG--L---V-FS-	E	D-N-L--AVQGM------
	Bacillus freudenreichii	WP_126431892	-------LLVCA-L---F----	E	P-SFL--TVQI---L-I-
	Bacillus ginsengihumi	WP_025726699	--------LF-A-L---F-FS-	S	PFN-L--S-QAV------
	Bacillus glycinifermentans	WP_048356143	------V--I---V---F-F--	A	HYSYM--IV-GI-TAA--
	Bacillus gottheilii	WP_080845061	-------------L---FLFS-	E	PFS-L--T-QI---I---
	Bacillus halmapalus	WP_078379690	---------VG--L---V-FS-	E	E-NI---L-TA---I---
	Bacillus halosaccharovorans	WP_078432551	------I--GI--L-----F--	A	E-T-I--I-QG--IA---
	Bacillus haynesii	WP_043928703	------V--V---V---FLF--	G	HYSYL--I--GI-TAV--
	Bacillus hisashii	WP_095141299	-V----V-LVLAY-------S-	E	P-NFT--AVQL--ML-I-
	Bacillus horikoshii	WP_088018874	--------LVG--L---V-FS-	E	E-TFL--T-T----I---
	Bacillus horneckiae	WP_066399416	---------LG------I-F--	E	PFN-F---VQG--II---
	Bacillus jeotgali	WP_079510327	--L-G--LMLG------V--S-	E	PFN-L--S-QGM--A---
	Bacillus kochii	WP_095372554	--------------V-----S-	E	PFGVL--S-QI---I---
	Bacillus koreensis	WP_053402103	--------V-I---------G-	A	SYSVV--FVQGFLTIA--
	Bacillus korlensis	WP_066049708	----G---LVG---V---LFS-	E	PFN-L--S-QG-L-L---
	Bacillus kribbensis	WP_026692886	------I-VFG-IL---V-F--	E	HFGYF--A--IV--A---
	Bacillus licheniformis	WP_075223417	------V--V---V---FLF--	G	HYSYL--I--GI-TAV--
	Bacillus litoralis	WP_121662849	---------V---L---W-FE-	T	E-T-L--M-QGF-IA---
	Bacillus loiseleuriae	WP_049682161	---------LG-IL---V--S-	E	TFN-R--A-QI-L-L---
	Bacillus marisflavi	WP_079516398	----G---LLI-Y----V-FS-	E	P-N-L--T-QI---I---
	Bacillus massiliglaciei	WP_110927396	---------FI-I----F--S-	E	PFN-F--TVQI---I---
	Bacillus massiliogabonensis	WP_102275855	--------V-G------I-FS-	E	PFN-L----QG---L---
	Bacillus massiliogorillae	WP_042350082	------I--IGPI----FLF--	E	HFN-L--T-QG---I---
	Bacillus massiliosenegalensis	WP_019154521	--V------LG-Y------FS-	E	PFN-F---VQG-LII---
	Bacillus mediterraneensis	WP_071460140	-V--G--ALFG--L---V-F--	E	PFD-M--TVQG---L---
	Bacillus megaterium	WP_098526517	--------V-L--VI-----D-	S	SYS-L----QG-L--A--
	Bacillus methanolicus	WP_004435731	-V--------G--L---I-FS-	E	PFS-Y--L-QG---I---
	Bacillus muralis	WP_064463707	---------FG-IV------S-	A	PFG-F--TVQI---I---
	Bacillus nealsonii	WP_016203005	---------L--YL-----FS-	E	E-TIL----QGM--I---
	Bacillus niacini	WP_045517646	--------LLT-Y----V-FS-	E	PFG-F--I-QS--II---
	Bacillus notoginsengisoli	WP_118920422	-V-------F---LI--F-VS-	E	PFN-F---VQG--TI---
	Bacillus novalis	WP_066090100	--------LL---L---V-FS-	E	SFH-L-----G--I----
	Bacillus oceanisediminis	WP_110066152	---------MG------VLFS-	E	PFN-L--T-QA-------
	Bacillus paralicheniformis	WP_065644172	------V--V---V---FLF--	A	HYSYL--I--GI-TAV--
	Bacillus persicus	WP_090741000	------V-LL---V---V--S-	E	PFNLL--SVQA---I---
	Bacillus sporothermodurans	WP_066225904	--------LFGS-------FS-	H	SFN-L--T-QI---I---
	Bacillus subterraneus	WP_125479915	--L-G---LFG------I-F--	E	PFN-I--TVQG---I---
	Bacillus subtilis group	WP_023855216	------V--V---V---FLF--	G	HYSYL--I--GI-TAV--
	Bacillus swezeyi	WP_076761879	------V--V-------FLF--	G	HYSYI--I--GV-TAV--
	Bacillus taeanensis	WP_113804993	---------VL------V-F--	E	SFTFL----QGI-LL---
	Bacillus terrae	WP_120114964	-V-----LLVSA-V---V--S-	S	S-NLL----QI-L-I-I-
	Bacillus testis	WP_050616793	-V--A-----G-I----FLF--	E	HFNSV--TVQI---I---
	Bacillus thermoamylovorans	WP_108899126	-V----V-LVLAY-------S-	E	P-NFT--AVQL--ML-I-

Other Bacteria (0/>200)

Bacillus tuaregi	WP_071395766	-V-------F-------FLFS-	S	-FN-I--L-QG---L---
Bacillus velezensis	WP_129193103	------V-MVL------F-FS-	E	P-TLT--L-QAM-TAV--
Bacillus vietnamensis	WP_034757578	--L---V-LMGS-L---V-FS-	E	PFS-L--T-QI---A---
Bacillus vireti	WP_024030092	--------LL---L---V-FS-	E	RFH-L----QG--I----
Bacillus weihaiensis	WP_072579161	--------LI---V---YLFS-	T	R--FS----QG-L-AV--
Bacillus xiamenensis	WP_008360040	------V-MVL--L---FLFS-	E	Q-TLT--L-QAM-TAV--
Bacillus zeae	WP_119113609	----G--ALLG------V-F--	E	PFN-L--T-QG---I---
Bacillus zhangzhouensis	WP_034319560	------V-MVL------F-F--	E	PFTLT--L-QAM-TAV--
Bhargavaea beijingensis	WP_092096852	-V--AS--VFL--V------S-	A	N-------A-----I---
Bhargavaea cecembensis	WP_063178369	-V--AS--VFL---------S-	A	E------LA-I---I---
Bhargavaea ginsengi	WP_092051194	-V--AS--VFL--V------S-	A	E------LA-I---I---
Caryophanon latum	WP_066464520	---------V----V--FA-S-	S	D-------GQA---I---
Caryophanon tenue	WP_066542369	---------L---LV--FV-S-	S	D------LGQS---I---
Chryseomicrobium excrementi	WP_100352982	------V-LVL-Y----V-FS-	E	Q-NV-----QI-----I-
Cohnella phaseoli	WP_116062187	--L-GA-A-Y-A-FV--W-FS-	E	PFG----I-QT-I--V--
Edaphobacillus lindanitolerans	WP_076757730	----AS--VFL--------FS-	G	E------TA-I--A----
Falsibacillus pallidus	WP_114744875	-V-------LG-Y------FS-	K	DFS-L--SVQI---I---
Filibacter sp. TB-66	WP_124071476	-----AF--LS--L-----D--	E	GMTVL----Q---IL---
Jeotgalibacillus alimentarius	KIL48537	---------LL--------F--	D	DFS-L--T-QI--II---
Jeotgalibacillus campisalis	WP_041059342	------I--V---------FS-	T	PFN-L--T-QL-------
Jeotgalibacillus malaysiensis	AJD91467	---------LL--------F--	S	DFT-F--TVQI--II---
Jeotgalibacillus proteolyticus	WP_104056911	---------L---L---ALF--	A	DFN-L--T-QI--IL---
Jeotgalibacillus salarius	WP_134381791	---------LL--------F--	S	DFT-F--T-QI--II---
Jeotgalibacillus soli	WP_041090282	---------I---L------S-	E	PFN-L--TVQF---A---
Jeotgalibacillus sp. R-1-5s-1	WP_134376635	------V--LL--------FS-	E	PFN-L--T-QG--II---
Jeotgalibacillus sp. S-D1	WP_133375376	---------L---L---M-FS-	D	PFN----T-QI---I---
Kurthia huakuii	WP_029499177	----A-VC-F----V----FS-	G	D------L--I-LT--I-
Kurthia massiliensis	WP_010287921	----A-IC-F----V----FS-	S	D------T--I-LTA---
Kurthia senegalensis	WP_010303288	------FCVFV--LI--FYFG-	D	HFH------QI--MI---
Kurthia sibirica	WP_109305915	--------VFL-Y-I----FS-	A	DF-------QI-LTL---
Kurthia sp. 3B1D	WP_126990290	----A-VC-F----V----FN-	G	D------L--I-LT--I-
Kurthia zopfii	WP_109350750	------I--FL--TI-----S-	G	E-H-L---VQI-LT----
Lysinibacillus boronitolerans	WP_016995357	----G---LVG--L-----FS-	A	E-S----I--I-------
Lysinibacillus chungkukjangi	WP_107935448	------I--VL---V-----S-	S	D-G-L--T--I--II---
Lysinibacillus composti	WP_124762038	---------LI---V----FS-	A	E-S----T--I---I---
Lysinibacillus contaminans	WP_053582861	----G-I-LIG--L---A-FS-	A	E-S----I--I-------
Lysinibacillus endophyticus	WP_121213275	---------LL---V--M-FS-	T	E-G-L--SV-I---I---
Lysinibacillus fusiformis	WP_025116028	----G---LVG--L-----FS-	A	E-S-------I-------
Lysinibacillus halotolerans	WP_122972528	---------LL---V-----S-	A	DFG-L--T--I-------
Lysinibacillus jejuensis	WP_108306993	---------F----V----F--	S	-------LGQ--L-IV--
Lysinibacillus macroides	WP_053996115	----G---LLG--LV----FS-	A	E-S-------I-------
Lysinibacillus mangiferihumi	WP_107893970	----G---LLG--------FS-	A	D-S----A--I-------
Lysinibacillus massiliensis	WP_036173610	---------LL---I-----S-	S	VYS-------I-L-I---
Lysinibacillus odysseyi	WP_036157952	-----A---LG--VV-----S-	S	D------L------A---
Lysinibacillus parviboronicapi	WP_107950187	----G-I-LVG--L-----FS-	A	D-S-------I-------
Lysinibacillus saudimassiliens	CEA04902	---------F----V----F--	S	-------LGQ--L-IV--
Lysinibacillus sinduriensis	WP_036201888	---------VL---A-----S-	A	D-G-I--T--I--I----
Lysinibacillus sp. 2017	WP_108713260	--------LLG---A-----S-	S	D---------L---A---
Lysinibacillus sp. B2A1	AVK84384	----G-I-LLG---V----FS-	T	D-S-L--T--I-------
Lysinibacillus sp. BF-4	WP_036141949	---------F----V----F--	S	V------LGQ--L-I---
Lysinibacillus sp. BK089	WP_132356758	----G-I-LIG--LV----FS-	A	E-S-L--AT-I---II--
Lysinibacillus sp. FJAT-14222	WP_053595163	----G---LLG--LV----FS-	T	E-S----AS-II---I--
Lysinibacillus sp. FJAT-14745	WP_053485467	----G-I-LLG---V----FS-	T	E-S----A--I-------
Lysinibacillus sp. Marseille-P	WP_106779927	------I-MLL------V-FS-	D	T-S----TV---L-I---
Lysinibacillus sp. SYSU K30002	WP_126657114	---------LL---V----FS-	T	E-G-L--S--I-------
Lysinibacillus sp. YLB-03	WP_118875564	---------LL--VI----FS-	G	E-G-I--A--I-------
Lysinibacillus sp. YR326	WP_134018325	----G-I-LIG---V----FS-	A	E-S----AT-II---I--
Lysinibacillus sp. ZYM-1	WP_054609379	----G---LVG--L-----FS-	A	E-S----I--I-------
Lysinibacillus sphaericus	WP_012295185	----G---LVG--L-----FS-	A	E-S----I--I-------
Lysinibacillus tabacifolii	WP_108029770	----G---LLG------S-FS-	A	D-S----A--I-------
Lysinibacillus telephonicus	WP_126296137	---------LL---M-----S-	A	D-G-L-----I-------
Lysinibacillus xylanilyticus	WP_068983547	----GM---IG---V----FS-	A	E-S----A--I-------
Lysinibacillus xyleni	WP_097074583	---------LL---V----FS-	T	E-G-L--S--L---I---
Mycobacteroides abscessus subs	SLL32653	---------L--YL---I-FS-	E	E-SVL--I-QGM--I---
Paenibacillus sp. FSL R5-0490	WP_076258028	--------LLG------ILFS-	E	PFN-L--T-QG---L---
Paenibacillus taiwanensis	WP_028546864	--L-GA-A-Y-A-FV----F--	E	PFGF---S-QT-I--VM-
Paenibacillus thiaminolyticus	WP_087440851	--L-GA-A-Y-A-FV----FG-	E	SFGF---S-QT-I--M--
Paenisporosarcina antarctica	WP_134209569	------I--LG--L---V-FS-	A	E------L--L---G---
Paenisporosarcina indica	WP_075618093	----A-V-VIG--L-----FS-	T	E---G-----SF------
Paenisporosarcina quisquiliaru	SEM46394	------V-MLL-YV-----FS-	T	E-TLL----QI---I---
Paenisporosarcina sp. HGH0030	WP_016426925	------I--VG--L---V-FS-	A	E------L------I---
Paenisporosarcina sp. OV554	WP_108586237	------I--LG--L---V-FS-	A	E------L----------
Paenisporosarcina sp. TG-14	WP_017380109	------I--LG--L---V-FS-	A	E------L--L---G---
Paenisporosarcina sp. TG20	WP_019413693	---------L---L---V-FS-	T	E------L-QA---I---

Group	Organism	Accession	Sequence
Other Bacteria (0/>200)	Paracoccus sp. DMF	TFE52334	--------LLG------VLFS- E PFN-L--T-QG---L---
	Planococcus antarcticus	WP_006830476	----A---VFG---M----FS- S D------A-QS---I---
	Planococcus citreus	WP_121299987	-V--AS---FG---I----FS- A D------L--I---L---
	Planococcus donghaensis	WP_065526516	----A----FG--VI----FS- S D------A-QS-------
	Planococcus faecalis	WP_078080351	----A----FG--VM----FS- G D------A-QS---I---
	Planococcus halocryophilus	WP_008499024	----A----FG--VM----FS- S D------A-QS---I---
	Planococcus halotolerans	WP_112223987	-V--A----FG---M----FD- A D------L-QL---G---
	Planococcus kocurii	WP_058385534	----A----FG--VM----FS- S D------A-QS---I---
	Planococcus maitriensis	WP_112232315	-V--AS---FG---I----FS- D D------L--I---L---
	Planococcus maritimus	WP_068462013	-V--AS---FG---I----FS- A D------L--I-------
	Planococcus massiliensis	WP_052651771	----A----FG--VV----FE- S D------L--S-------
	Planococcus plakortidis	WP_068869044	-V--AS---FG---I----FS- A D------L--I---L---
	Planococcus rifietoensis	WP_058380908	----AS---FG---I----FS- A D------L--I---L---
	Planococcus salinarum	TAA70712	-V--A----FG--VM----FD- A D------L-QL---G---
	Planococcus salinus	WP_123163793	----A----FG--LI----FS- S E------A-Q----A---
	Planococcus sp. PAMC 21323	WP_038703720	----A----FG---M----FS- S D------A-QS---I---
	Planococcus sp. Y42	WP_077588877	----A---L-G--VT----FG- A E------A--I-------
	Planococcus versutus	WP_065524688	---------FG--VM----FS- S D------A-QI---I---
	Planomicrobium flavidum	WP_088006345	----G---L-G--VA----FG- A E-S----A--A---I---
	Planomicrobium glaciei	WP_074510993	----A----FG--VI----FD- S D------L-QT---I---
	Planomicrobium okeanokoites	WP_084246812	-V--A----FG---M----FD- A D------L-QL---G---
	Planomicrobium soli	WP_106532683	----AA---FG--VV----FN- A D------T--I--L----
	Planomicrobium sp. MB-3u-38	WP_101803788	-V--A----FG---M----FD- A D------L-QL---G---
	Planomicrobium sp. Y74	WP_121633945	-VV-A----FG---M----FD- A D------L-QL---A---
	Psychrobacillus insolitus	PZX07212	------V-MVI-Y-V----FS- T E-T-L--L-QL-------
	Psychrobacillus psychrotoleran	SFQ74203	------V-MLL-YVV----FS- A E-T-L----QI-------
	Psychrobacillus sp. FJAT-21963	WP_056832096	--L---V-MIL-Y-I----FS- S E-T-L----QL-------
	Psychrobacillus sp. OK028	SDM85510	------V-MLL-YVV----FS- N E-T-L--I-QI-------
	Psychrobacillus sp. OK032	WP_093267127	------V-MLL-YV-----FS- V E-N-L----QA-------
	Rhodococcus qingshengii	WP_133367347	--------LL--Y----V-FS- E PFG-F--L-QSF-II---
	Rummeliibacillus pycnus	WP_102691348	---------FL--VIH---HG- G EF-----------T----
	Rummeliibacillus stabekisii	WP_066786540	---------FL---IH---HS- S E------------TL---
	Solibacillus isronensis	WP_079527824	--------LLG---V-----S- T E---------L---A---
	Solibacillus kalamii	WP_087616042	--------LLG---V-----S- T E------L--L---A---
	Solibacillus silvestris	WP_014823406	--------LLG---V-----S- T E------L--L---A---
	Solibacillus sp. R5-41	WP_099424551	------I-LLG---A-----S- A D------L--I---I---
	Sporosarcina globispora	WP_053437610	--------LLG-I----ILFS- E PFN-L--T-QG---L---
	Sporosarcina koreensis	WP_060210371	------F--VG--L----IAS- E EMS-L----QT-------
	Sporosarcina newyorkensis	WP_078816543	-V----V--L---L---VIVS- E E-T-L-----G---I---
	Sporosarcina psychrophila	WP_067210206	----TA---VG--L----ID-- E GMTI---I-Q----I---
	Sporosarcina sp. BI001-red	WP_116016855	---------IG--L----ID-- S SMG-L--T-QL--TI---
	Sporosarcina sp. D27	WP_025784759	-V-------IG--L----ID-- S SMG-Q--T-QI--TI---
	Sporosarcina sp. EUR3 2.2.2	WP_024534501	------I--LG--L---V-FS- A D------L------I---
	Sporosarcina sp. HYO08	WP_067403512	-----A---F---L-F-FIH-- E GMDVL--I-QI--IA---
	Sporosarcina sp. P13	WP_099687433	------I-LF---L---AIVS- E E-T-L--------L----
	Sporosarcina sp. P20a	WP_099676856	-V----V-LL---L---ALVS- E E-T-L----Q----L---
	Sporosarcina sp. P3	WP_099637674	-V----I--L---L---A-VS- E E-T-L--A-QI---L---
	Sporosarcina sp. P33	WP_081241854	-V----V------L---VIVS- E E-T-L--I-QL---L---
	Sporosarcina sp. P34	WP_099694423	-V----V-LLV--L---AIVS- E E-T-L----Q----L---
	Sporosarcina sp. P7	WP_099637281	-V----V-LLV--L---AIVS- E E-T-L----Q----L---
	Sporosarcina sp. PTS2304	WP_114923619	------I--L---L---AIVS- E E-T-L--I--C-------
	Sporosarcina sp. ZBG7A	WP_039041668	-V-------IG--L----ID-- S SMG-L--T-QI--TI---
	Sporosarcina ureae	WP_083030622	-V----I--L---L---A-VS- E E-T-L--A-QI---L---
	Tetzosporium hominis	WP_094942068	------V-LLL-Y----V-FS- E E-NI-----QI-----I-
	Ureibacillus thermophilus	QBK26252	--------LLL--------FS- T E-SLG--L--I---I---
	Ureibacillus thermosphaericus	WP_026019114	---------LL---V----FS- A E-S----I--I---I---
	Viridibacillus arvi	WP_053416329	---------LL----H-V--S- G D--------QL---A---
	Viridibacillus sp. OK051	WP_100794244	---------LV------A--S- G D------L-QI---A---

Supplemental Figure 42

A partial sequence alignment of the DUF456 domain-containing protein containing a one amino acid deletion (boxed) that is exclusively shared by all members belonging to the Meyeri clade and absent in all other bacteria.

			178		230
**Meyeri Clade (3/3)**	Lysinibacillus fluoroglycofenilyticus	WP_107942781	LYALNEQYFAEINIFIAAGELKLADVKNKLPAVQPIDPM		ELQKLNDQKMAIEWLDRRLYDMQISRE
	Bacillus ndiopicus	WP_042475579	-------------VY----------A-KQ--MTE-T---		---R----------------I-----
	Lysinibacillus meyeri	WP_107839321	--K----------VY------------KQ--I-E-TE--		A--Q----------------------
**Other Bacteria (0/>200)**	Aeribacillus pallidus	ASS92386	--EH-KE--HAL--Y--------EELHT-VIPELQKKAE	TTGDQM	AV-EV--MLQFADR-EK-IH-LKL--Q
	Aerococcus suis	WP_084098792	--QQ-F---EAL-V----AQ---KKLQQEDLPKRYEQAQ	ETQNQM	TI-EV--L-QF-NR-EK-I--L-L--Q
	Alkalibacterium gilvum	WP_091635083	--DK-YE--EAL-VY-----V--EE-Q--SIPRAIERAE	SSNNQM	-V--V--LNQFLSR--K-V--LKTA-Q
	Alkalibacterium olivapovliticu	WP_106190644	--EK-YD--EAL-VY-----V-IEELS-EVIPQAVKHAE	TSGNQM	-V--V--LNQFLNR--K-V--LKTA-Q
	Alkalibacterium pelagium	WP_091478984	--EK-YE--EAL-VY-----V-IDELS-EVIPQAVKHAE	DSGNQM	-V--V--LNQFLNR--K-V--LKTA-Q
	Alkalibacterium sp. AK22	WP_034302440	--EK-YE--EAL-VY-----I-MDELSQEIIPQAVKTAE	DSGDQM	-V--V--LNQFLNR--K-V--LKTA-Q
	Alkalibacterium subtropicum	WP_091527802	--DK-YD--EAL-VY-----V-MEELQ-DIIPKAIEHAE	NSSNQM	-V--V--LNQFLNR--K-V--LKTA-Q
	Alkalibacterium thalassium	WP_091264547	--EK-YE--EAL-VY-----V-IDELS-EVIPQAVKHAE	ESGNQM	-V--V--LNQFLNR--K-V--LKTA-Q
	Ammoniphilus sp. CFH 90114	WP_129200916	--DN-KE--HTL--Y--------DELHE-VIPALKKEAE	TSKDEM	KF-EVK-MLQFADR--K----LKL---
	Ammoniphilus sp. YIM 78166	WP_134701901	-FEH-KE--HAL--Y--------DQLYQ-TIPELKKQAE	ASNDQM	KV-EV--MMQFADR--K--H-LKL---
	Bacillus acanthi	WP_108669960	--EN-KE--QAL-VY--------EELQQVTIPALQKQAE	ATNDHM	KF-EV--MIQFADR--K----LKL---
	Bacillus alkalitelluris	WP_078550031	--EN-KE--NAL-VY--------EELHKTTIPALKSEAE	VSNDQM	KF-EV--MMQFADR--K-IH-LKL---
	Bacillus asahii	WP_119118838	--ET-KD--HAL-VY-----I--EELY--TIPELKQSAQ	LSNDQM	KF-EV--MIQFADR--K--H-LKL---
	Bacillus bataviensis	WP_007084341	--ET-KE--HAL--Y-----I--EEMQT-TIPQLKKTAE	SANDQM	KF-EV--MIQFADR--K--H-LKL---
	Bacillus butanolivorans	WP_098177008	--ET-KE--QAL-VY-----I--EEIQE-TIPELKKSAE	LSNDQM	KF-EV--MRQFA-R--K----LKL---
	Bacillus campisalis	WP_046523069	--EH-KE--NAL--Y--------EELN--TIPELKKAAA	ATQDQM	KF-EV--MIQFADR--K--H-LKL---
	Bacillus canaveralius	WP_101578710	--EH-KE--HAL-VY--------EELYE-TIPELKKKAE	STQDQM	AF-DV--MRQFADR--K----LKL---
	Bacillus cavernae	WP_126867048	--EN-KE--QAL-VY-----I--EELHE-TIPELKKSAE	STNDHM	KF-EV--MVQFADR--K----LKL---
	Bacillus cecembensis	WP_057985237	---V--E--Q-V-V---SL-I-KQHLQEVILPTIQQALF	ESTDPF	KQNE---LH-Q-------M--LE----
	Bacillus cihuensis	WP_028389763	--ER--E--HML--Y-----SR-EELHAHSIPKAEHIAK	MEMDPL	KK-E-D-LLSFTDL-EK-V--LK----
	Bacillus circulans	WP_047944659	--EH-KT--EDL-VY---A-M-YEEMTK--IPE-KKLAE	ESGDRM	K--EV--LIHFA-L-EK-I--LKV---
	Bacillus cucumis	WP_101650393	--ET-KE--HAL--Y-----I--EEMET-IIPQLKKEAE	TTNDQM	KF-EV--MIQFADR--K--H-LKL---
	Bacillus dakarensis	WP_077211269	--EH-KD--NAL-VY--------EEMQT-IIPELKKSAE	ASQDQM	KF-EV--MLQFADR--K----LKL---
	Bacillus dielmoensis	WP_042462601	--DN-KE--HAL-VY--------EEMYQ-TIPELKRQAE	LTSDQM	KF-EV--MIQFADR--K--H-LKL---
	Bacillus drentensis	WP_066258995	--ET-KE--HAL-VY-----I--EEMQE-TIPQLKRAAE	VANDQM	KF-EV--MIQFADR--K--H-LKL---
	Bacillus firmus	WP_035331745	--DN-KE--HAL-VY--------EELHE-TIPELKKRAE	ESNDQM	KF-EV--MIQFADR--K----LKL---
	Bacillus foraminis	WP_121610986	--EN-KE--NAL--Y--------EELNE-TIPELKRAAQ	QTQDQM	KF-EV--MIQFADR--K--H-LKL---
	Bacillus fordii	WP_018708470	--EK-KE--HAL-----------EEIRTETIPALKKKAE	ASGDQM	AF-DV--MIQFADR--K----LKL--Q
	Bacillus fortis	WP_120068262	--EK-KE--HAL--------V--EEIRTETIPALKKKAE	ASGDQM	AF-DV--MIQFADR-EK----LKL--Q
	Bacillus freudenreichii	WP_126431965	--EK-KE--HAL----------IEEMRTETIPSLKKKAE	ASGDQM	AY-DV--MIQFADR-EK----LKL--Q
	Bacillus fumarioli	WP_066370348	--ET-KE--HAL--Y--------EEMHE-IIPALKKEAE	ATNDQM	KF-EV--MIQFADR--K--H-LKL---
	Bacillus gobiensis	WP_053605082	--ER-RD--QAL-VY-------IEEIRT--LPELQKKAD	SGHDQM	AV-DV--MMQFVDR-EK-I--LTL--Q
	Bacillus gottheilii	WP_066450045	--ES-KE--HAL-VY--------EELHE-TIPALKQEAE	KSNDQM	KF-EV--MIQFADR--K----LKL---
	Bacillus horneckiae	WP_066400734	--EN-KE--NAL-VY-----I--EELHE-TIPELKKAAE	ASNDQM	KF-EV--MIQFADR--K----LKL---
	Bacillus indicus	WP_051827203	--ET-RE--HSL-TY-E-A----HELH-ETIPALKKTNE	CSSDPM	KR-EEE-LLQFADQ--Q----LKV---
	Bacillus jeotgali	WP_079506562	--ET-KE--NAL--Y--------EELNE-TIPELKRAAE	TSQDQM	KF-EV--MIQFADR--K----LKL---
	Bacillus kochii	WP_095370331	--ES-KE--HAL--Y--------EEMREQSIPDLKKKAE	SSQDQM	KV-EV--LVQFA-R--K----LKL---
	Bacillus korlensis	WP_066055611	--EH-KE--HAL-VY--------DELNQSTIPKLKQKAE	QTQDQM	AF-EV--MMQFADR--K-I--LKL---
	Bacillus kwashiorkori	WP_062350762	--ET-K---QAL--Y-----V--EELET-VIPDLRKRAE	LSGNQM	DV-EV--MIQFA-R-EK--H-LKL--Q
	Bacillus lentus	WP_066137549	--EK-KE--HAL--Y-------IEELRT-VIPEAKQKAE	ASGDQM	AY-EV--MVQFADR-EK----LKL--Q
	Bacillus loiseleuriae	WP_049683544	--EH-KE--HML--Y--------EELHGNSIPTAEHVAK	METDPL	KK-E-D-ILSYTDL-EK-V--LK----
	Bacillus massiliglaciei	WP_110926235	--ET-KE--HAL-VY--------EELY--TIPELKKAAE	TANDQM	KF-EV--MIQFADR--K--H-LKL---
	Bacillus massiliogabonensis	WP_102272906	--EN-KE--HAL-VY-----I--EELHQ-TIPELKKAAE	ASNDQM	KF-EV--MIQFADR--K----LKL---
	Bacillus massilionigeriensis	WP_075982999	--DN-KE--HAL-V---------DELHD-VIPELKRKAE	ASQDQM	AF-EV--MMQFADR--K--H-LKL--Q
	Bacillus massiliosenegalensis	WP_019153058	--EN-KD--NAL-VY--------E-LRE-TIPELQKKAE	ASQDQM	KY-EA--MIQFADR--K----LKL---
	Bacillus mediterraneensis	WP_071460869	--EN-KE--HAL-VY--------EELQ--TIPEMKKAAA	ATSDQM	KF-EV--MIQFADR--K--H-LKL---
	Bacillus mesonae	WP_066387629	--ET-KE--HAL-VY-----I--EEMHE-IIPELKKQAE	ATKDQM	KF-EV--MIQFADR--K----LKL---
	Bacillus methanolicus	WP_003348290	--EK-KE--QAL-VY--------EELYK-TIPELKKAAE	ETNDQM	KV-EV--MIQFANR--K--H-LKL---
	Bacillus muralis	WP_064462282	--ET-KE--QAL-VY-----I--EEIHG-TIPELKKSAE	LSNDQM	KF-EV--MRQFA-R--K--H-LKL---
	Bacillus niacini	WP_045518294	--ET-KE--HAL-VY-----I--EELHQ-TIPEMKRAAE	VTNDQM	KF-EV--MIQFADR--K----LKL---
	Bacillus niameyensis	WP_062104748	--EK-KD--HAL----------IEELRT-SIPELKKKAE	ASGDQM	TY-EV--MVQFADR-EK----LKL--Q
	Bacillus notoginsengisoli	WP_118922121	--EH-KE--NAL-VY-------YEELQREVIPALRKTAE	ASGDQM	KF-EV--MLQFADR--K-VH-LKL---
	Bacillus novalis	WP_066092081	--ET-KE--HAL-VY-----I--EEMQE-TIPQMKRAAE	AANDQM	KF-EV--MIQFADR--K--H-LKL---
	Bacillus oceanisediminis	WP_019380106	--EN-KE--HAL-VY--------EELHE-TIPELKKKAE	ESNDQM	KF-EV--MMQFADR--K----LKL---
	Bacillus persicus	WP_090743501	--EN-KE--NAL--Y--------DELT--TIPDLKRAAQ	STNDQM	KF-EV--MIQFADR--K----LKL---
	Bacillus praedii	WP_057766551	--EN-KE--HAL-VY-----I--EELHQ-TIPELKKAAE	SSNDQM	KF-EV--MIQFADR--K----LKL---
	Bacillus rubiinfantis	WP_042354048	--DT-KE--HAL--Y--------EEMHQ-IIPELKKQAE	ATNDQM	KF-EV--MIQFADR--K--H-LKL---
	Bacillus selenatarsenatis	WP_041967818	--ET-KE--NAL--Y--------EELHD-TIPELKKAAE	TSQDQM	KF-EV--MIQFADR--K----LKL---
	Bacillus simplex	WP_034306047	--ET-KE--QAL-VY-----I--EEIHE-TIPELRNSAE	SSNDQM	KF-EV--MLQFA-R--K--H-LKL---
	Bacillus soli	WP_066067409	--ET-KE--HAL-V------I--EEMQE-TIPQLKKAAE	AANDQM	KF-EV--MIQFADR--K--H-LKL---
	Bacillus sp. 1NLA3E	WP_015595848	--ET-KE--HAL--Y-----I-IEELNE-TIPALKKAAE	ASQDQM	KY-EVS-MIQFADR--K----LKL---
	Bacillus sp. AFS006103	WP_098262235	--ET-KE--HAL-VY-----I--EEMQE-TIPQLKRAAE	VTNDQM	KF-EV--MIQFADR--K--H-LKL---
	Bacillus sp. AFS017274	WP_098370387	--ET-KD--QAL-VY-----I--EEIHG-TIPELRKSAE	SSNDQM	KF-EV--MLQFA-R--K--H-LKL---
	Bacillus sp. AFS026049	WP_098184969	--ET-KE--QAL-VY-----I--EEIHE-TIPELRKSAE	TSNDQM	KF-EV--MLQFA-R--K-IH-LKL---
	Bacillus sp. AFS031507	WP_098932622	--ET-KE--HAL-VY-----I--EEMQE-TIPQLKKAAE	VTNDQM	KF-EV--MIQFADR--K--H-LKL---
	Bacillus sp. AFS073361	WP_098575052	--ET-KE--HAL-VY-----I--EEMQE-TIPQLKKAAE	QTNDQM	KF-EV--MIQFADR--K--H-LKL---
	Bacillus sp. AFS094228	WP_098937622	--ET-KE--QAL-VY-----I--EEIHE-TIPELRKSAE	TSNDQM	KF-EV--MLQFA-R--K-IH-LKL---
	Bacillus sp. B-jedd	WP_048827183	--EH-KE--NAL-VY-------YDELQREVIPALRKTAE	ESGDQM	KF-EV--MLQFADR--K-IH-LKL---
	Bacillus sp. BA3	WP_101224993	--ET-KD--QAL-VY-----I--EEIHG-TIPELRKSAE	SSNDQM	KF-EV--MLQFA-R--K--H-LKL---
	Bacillus sp. EB01	WP_043929999	--EH-KE--NAL-VY-------YDELVQ-TIPAMRREAE	VSGDQM	KV-EV--MMQFADR--K-IH-LKL---
	Bacillus sp. FJAT-18017	WP_053597740	--EH-KE--NAL-VY-------YDELVQ-TIPAMRREAE	VSGDQM	KV-EV--MMQFADR--K-IH-LKL---
	Bacillus sp. FJAT-20673	WP_063577511	--ET-KE--QAL-VY-----I--EEIHE-TIPELRKSAE	SSNDQM	KF-EV--MLQFA-R--K--H-LKL---
	Bacillus sp. FJAT-21352	WP_053533601	--ET-KE--QAL-VY-----I--EEIHE-TIPELRKSAE	SSNDQM	KF-EV--MLQFA-R--K--H-LKL---
	Bacillus sp. FJAT-22058	WP_054398092	--ET-KE--QAL-VY-----I--EEIHE-TIPELRKSAE	SSNDQM	KF-EV--MLQFA-R--K--H-LKL---
	Bacillus sp. FJAT-25496	WP_057775822	--EN-KE--HAL-VY-----I--EELNE-IIPELKKAAE	ESNDQM	KF-EV--MIQFADR--K----LKL---
	Bacillus sp. FJAT-26652	WP_053355833	--EH-KE--QAL-VY--------EELNG-IIPALRKEAE	TTNDQM	KY-EV--MVQFADR--K--H-LKL---
	Bacillus sp. FJAT-27225	WP_066206061	--EH-RE--NAL-VY-------YDELIQ-TIPAMRREAE	ASGDQM	KV-EV--MMQFADR--K-IH-LKL---
	Bacillus sp. FJAT-27245	WP_053368332	--EY-KE--NAL-VY-------YEELQREVIPALRKEAE	ASGDQM	KF-EV--MLQFADR--K-VH-LKL---
	Bacillus sp. FJAT-27251	WP_053360627	--EH-KE--NAL--Y--------EELN--TIPELKKAAA	ATQDQM	KF-EV--MIQFADR--K--H-LKL---
	Bacillus sp. FJAT-27445	WP_059171892	--EY-KE--NAL-VY-------YEEMQREVIPAMKK-AE	ASGDQM	KF-EV--MLQFADR--K-VH-LKL---
	Bacillus sp. FJAT-29814	WP_066321165	--ET-KE--HAL-VY-----I--EEMHE-IIPRLKQEAQ	STNDQM	KF-EV--MIQFADR--K--H-LKL---
	Bacillus sp. FJAT-29937	WP_066295272	--EN-KE--HAL-VY-----I--EELNDITIPALKKKAE	ESNDQM	KF-EV--MIQFADR--K----LKL---
	Bacillus sp. FJAT-42315	WP_100402719	--EK-KE--QAL-----------EELQTETIPALRQKAE	QSGDQM	AY-DV--MVQFADR-EK--H-LKL--Q
	Bacillus sp. FJAT-46582	WP_100331200	--EK-KE--QAL-----------EELET-TIPALRQKAQ	ESGDQM	AF-EV--MVQFANR-EK--H-LKL--Q

Other Bacteria (0/>200)

Bacillus sp. HMSC76G11	WP_070879570	--DN-KD--HSL-VY-----M--EELH-RIIPELKNANS GSNDPM KQ-E-E-LLQFADQ--Q----LK----
Bacillus sp. J33	WP_026582629	--EN-KE--HAL-VY--------EELHE-IIPELKKKAE ETNDQM KF-EV--MIQFADR--K----LKL---
Bacillus sp. LF1	WP_090632239	--DT-KD--QAL-VY-----I--EEIHE-TIPALKQEAE VTNDQM KF-DVK-MIQFADR--K----LKL---
Bacillus sp. M5HDSG1-1	WP_127741695	--ES-K---HDL--L-Y-A-T---Q--EHKLPEMRKLAK ELQDEM KM-EAE-MEDFADR-EK-I--LK---Q
Bacillus sp. M6-12	WP_101597684	--EN-KE--HAL-VY-----I--EELY--TIPELKKIAE ETNDQM KF-EV--MTQFADR--K--H-LKL---
Bacillus sp. MRMR6	WP_075690393	--ET-KE--HAL-VY--------EELHH-TIPAMKKAAE AMNDQM KF-EV--MIQFADR--K----LKL---
Bacillus sp. MUM 116	WP_071354563	--ET-KE--HAL-VY-----I--EEMHE-IIPQLRKAAE ATNDQM KF-EV--MLQFADR--K--H-LKL---
Bacillus sp. NRRL B-14911	EAR68780	--DT-KE--HAL-VY-----I--EELHEQTIPQMRKEAE SSGDQM KF-EV--MLQFADR--K----LKL---
Bacillus sp. OG2	WP_094768623	--DT-KE--HAL-VY-----I--EELHEQTIPQLRKEAE ASGDQM KF-EV--MLQFADR--K----LKL---
Bacillus sp. OK048	WP_090761116	--ET-KE--HAL-VY-----I--EELHQ-TIPEMKRAAE TTNDQM KF-EV--MIQFADR--K----LKL---
Bacillus sp. OK085	WP_132091430	--ET-KE--HAL-VY-----I--EELHQ-TIPEMKKAAE ATSDQM KF-EV--MIQFADR--K----LKL---
Bacillus sp. OV166	WP_088089681	--ET-KE--HAL-VY-----I--EEMQE-TIPQLKKAAE VTNDQM KF-EV--MIQFADR--K--H-LKL---
Bacillus sp. OV194	WP_091008609	--EH-KE--QAL-VY--------EELNG-IIPALRKEAE TTNDQM KY-EV--MVQFADR--K--H-LKL---
Bacillus sp. RJGP41	WP_106027486	--ET-KE--QAL-VY-----I--EEIHE-TIPELRKSAE SSNDQM KF-EV--MLQFA-R--K--H-LKL---
Bacillus sp. T33-2	WP_101582331	--EN-KE--HAL-VY--------EELHE-TIPELKKAAA ATNDQM KF-EV--MIQFADR--K----LKL---
Bacillus sp. UMB0893	WP_101568592	--EN-KE--QSL-VY--------EELHT-SIPA-KKVIE GTHDPM KQ-EMS-LLQFADQ-EA-I--LKM---
Bacillus sp. UNC41MFS5	WP_026564047	--ET-KE--HAL-VY-----I--EEMQE-TIPQLKKAAE QTNDQM KF-EV--MIQFADR--K--H-LKL---
Bacillus sp. UNC438CL73TsuS30	WP_026575860	--ET-KE--HAL-VY-----I--EEMHE-TIPQLR-AAE ATNDQM KF-EV--MLQFADR--K----LKL---
Bacillus sp. URHB0009	WP_027322223	--EH-KE--HAL--Y--------EELYE-TIPQLKQESE ASQDQM KY-EV--MVQFADR--K----LKL---
Bacillus sp. V33-4	WP_101666154	--EH-KE--HAL-VY--------EELYE-TIPELKKKAE STQDQM AF-DV--MRQFADR--K----LKL---
Bacillus sp. V44-8	WP_117323660	--ES-KE--QAL-VY--------EELTA-TIPELKKAAE TTNDQM KF-EV--MVQFADR--K-VH-LKL---
Bacillus sp. V47-23a	WP_117327125	--EN-KE--HAL-VY-----I--EELYH-TIPELKKAAE ETNDQM KF-EV--MTQFADR--K--H-LKL---
Bacillus sp. V59.32b	WP_117305553	--EN-KE--QAL-VY-----I--EELHGITIPELRKMAE ATNDQM KF-EV--MVQFADRF-K----LKL---
Bacillus sp. VT-16-64	WP_077113248	--EK-KE--HAL-----------EEIRKETIPALKKKAE ASGDQM AF-DV--MMQFADR-EK----LKL--Q
Bacillus sp. WN066	WP_133333893	--ET-KE--HAL-VY-----I--EEMHE-TIPQLRKAAE ATNDQM KF-EV--MLQFADR--K----LKL---
Bacillus sp. X1(2014)	WP_038537420	--ET-KE--HAL-VY-----I--EELHE-IIPQLKKEAE ASNDQM KF-EV--MIQFADR-EK--H-LKL---
Bacillus sp. XXST-01	WP_126408378	--EN-KD--NAL-VY-----I--EELYE-TIPAMRA-AE ATQDQM KF-EV--MIQFADR--K----LKL---
Bacillus sp. Y1	WP_119710192	--EH-KE--HAL-VY--------DELNQSTIPKLKQKAE STQDQM AF-EV--MMQFADR--K-I--LKL---
Bacillus sp. YLB-04	WP_115451212	--EY-KE--NAL-VY-------YEELQRDVIPALRKEAE ASGDQM KF-EV--MLQFADR--K-VH-LKL---
Bacillus sp. mrc49	WP_100533314	--ET-KD--QAL-VY-----I--EEIHG-TIPELKKSAE LSNDQM KF-EV--MRQFA-R--K--H-LKL---
Bacillus sporothermodurans	WP_066234600	--EK-KD--HAL--Y--------EELQS-TIPELKNKAA ATGDQM AV-DV--MIQFADR-EK--H-LKL--Q
Bacillus subterraneus	WP_044390283	--ER-KE--QAL--Y-----I--EELHE-TIPALKKAAA ETNDQM KY-EV--MIQFADR--K--H-LKL---
Bacillus terrae	WP_120115051	--EK-KE--HAL-----------EEIRSETVPALKKKAE ASGDQM AF-DV--MIQFADR-EK--H-LKL--Q
Bacillus testis	WP_050614489	--EK-KE--HAL--Y---A-----ELNE-TIPELRKKAE ETGDQM AV-EV--MLQFVDR-EK-M--L-L--Q
Bacillus thermoamylovorans	WP_034767777	M-EK-KE--QAL--Y-----V--EELHT--IPELKRKAE QSGDQM AY-EV--MIQFADR-EK--H-LKL--Q
Bacillus timonensis	WP_010677505	--EN-KD--DAL-VY--------EEMHQ-IIPELKKQAE TTNDQM KV-EV--MVQFADR--K-VH-LKL---
Bacillus tuaregi	WP_071394598	--EK-KE--LAL--Y--------EELNQ-SIPELKRKAE MSQDQM DF-DV--MLQFADR-EK-M--LKL--Q
Bacillus vireti	WP_024028537	--ET-KE--HAL-VY-----I--EEMQE-TIPQLKRAAE AANDQM KF-EV--MIQFADR--K--H-LKL---
Bacillus zeae	WP_119114322	--EN-KE--HAL--Y--------EELQ--TIPEMKKAAA ASNDQM KF-EV--IIQFADR--K--H-LKL---
Bhargavaea beijingensis	WP_092097303	--DQ-KA--QAL-VY---A---RDEIA-EIIPAMRRKAE ETNDQM AI-EV--MAQFVDR-EK-I--L-L--Q
Bhargavaea cecembensis	WP_040228593	--EQ-KA--QAL-VY---A---RDEIM-ETLPALRAKAE ASNDQM AF-EV--MAQFVDR-EK-V--L-L--Q
Butyricicoccus sp. 1XD8-22	RKJ57068	--N---EFYHN---H---LDI-KQHLITN-LPKLQNESV SDSDSW QQ---S-LEN----I-K-M--L-L---
Carnobacterium sp. ZWU0011	WP_052181731	---K-LD--EAL--Y-------QEELLT--IPAAVEKAE KSTDQM DV--V--LNQFLDR-EK-TH-LKLA-Q
Desulfamplus magnetovallimorti	WP_080802720	--ER-QE--DSLE-L-----I--QEMEQEFEIRRSGLEA MENTDP MDLQKLKDFGDTIQSLD-RLYNLKLAR
Domibacillus aminovorans	WP_018393386	--DK-KE--QAL--Y--------EEIET-TIPELRQKAE RTGDQM DF-DV--MIQFADR-EK--H-LKL--Q
Domibacillus antri	WP_075398439	--DK-KE--QAL--Y--------EELET-TIPELRQKAE RTGDQM DF-DV--MVQFADR-EK--H-LKL--Q
Domibacillus enclensis	WP_045849587	--EK-KE--QAL--Y--------EEIET-TIPELRQKAE RTGDQM DF-DV--MIAFADR-EK--H-LKL--Q
Domibacillus epiphyticus	WP_076767254	--DK-KE--QAL--Y--------EELET-TIPELRQKAE STGDQM DF-DV--MVQFADR-EK--H-LKL--Q
Domibacillus indicus	WP_046173384	--EK-KE--QAL--Y--------EEIET-TIPELRQKAE RTGDQM DF-DV--MIAFADR-EK--H-LKL--Q
Domibacillus iocasae	WP_069937709	--EK-KE--LAL--Y--------EEIET-TIPELRQKAE RTGDQM DF-DV--MIAFADR-EK--H-LKL--Q
Domibacillus mangrovi	WP_073711367	--DK-KE--QAL--Y--------EEIET-TIPELRQKAE RSGDQM DF-DV--MIQFADR-EK--H-LKL--Q
Domibacillus robiginosus	WP_050184276	--EK-KE--QAL--Y--------EEIET-TIPALRQKAE QTGDQM DF-DV--MVAFADR-EK--H-LKL--Q
Domibacillus tundrae	WP_046181359	--EK-KE--QAL--Y--------EEIET-TIPDLRQKAE RTGDQM DF-DV--MIAFADR-EK--H-LKL--Q
Enterococcus aquimarinus	OJG11243	--QK-KD--DAL--Y-------MEELQ-DIIPAAMKKAE ETGDQM DV-IV--YTQFLDR--K-TH-LRLT-Q
Enterococcus timonensis	WP_071130988	--HK-KD--DAL--Y-------MEELQTTIIPQAMQKAQ QSGDQM DV-IA--YTQFLDR--K-T--LKLA--
Facklamia hominis	WP_006907385	--HK-KD--DAL--Y--------NNMQE-DLKKAIEKAN ASKNQM DV-VV--LQQF--R--K-I--LKLT-Q
Facklamia languida	WP_040447688	--QK-KD--DAL--Y-------IEELQSEIIPQAIEKAS QTNSSM DV-EVH-LNQFL-R--K-TH-LRLT-Q
Facklamia languida CCUG 37842	EHR38067	--QK-KD--DAL--Y-------IEELQSEIIPQAIEKAS QTNSSM DV-EVH-LNQFL-R--K-TH-LRLT-Q
Fictibacillus aquaticus	WP_094253629	--EH-KE--QAL-VY-----V--DELNQ-TIPALRKEAE ASGDQM KF-EV--MVQFADR--K--H-LKL---
Fictibacillus arsenicus	WP_066285918	--EN-KE--QAL-VY--------DELNQVTIPEMRKVAE QTNDQM KF-EV--MVQFADR--K-VH-LKL---
Fictibacillus enclensis	WP_061974622	--DH-KE--QAL-VY--------EEINT-VIPALRKEAE DTNDQM KY-EV--MVQFADR--K--H-LKL---
Fictibacillus phosphorivorans	WP_066243056	--EN-KE--QAL-VY--------DELNQVTIPEMRKVAE QTNDQM KF-EV--MVQFADR--K-VH-LKL---
Fictibacillus solisalsi	WP_090237249	--DH-KE--QAL-VY--------EEINT-VIPALRKEAK DTNDQM KY-EV--MVQFADR--K--H-LKL---
Fictibacillus sp. BK138	WP_130297175	--EN-KE--QAL-VY--------DELNQVTIPEMRKVAE QTNDQM KF-EV--MVQFADR--K-VH-LKL---
Fictibacillus sp. FJAT-27399	WP_062238839	--EH-KE--QAL-VY--------E-LNG-IIPALRKEAE TTNDQM KY-EV--MVQFADR--K--H-LKL---
Fictibacillus sp. S7	WP_129477935	--DH-KE--QAL-VY--------EEINT-VIPALRKEAE DTNDQM KY-EV--MVQFADR--K--H-LKL---
Gracilibacillus boraciitoleran	WP_035723458	--EH-KE--KGL-V---------EEIQE-ELPALRKAAE ATDDQM KF-EV--MLQFADR-EK--H-LKL--Q
Gracilibacillus kekensis	WP_073202352	--EH-KE--KGL-VY--------EEIQS-EIP-LRKEAE ATNDQM KF-EV--MLQFSDR-EK--H-LKL--Q
Gracilibacillus massiliensis	WP_058306386	--EH-KE--KGL-VY--------EEIQL-EIPALRKEAE ATNDQM KF-EV--MLQFTDR-EK--H-LKL--Q
Gracilibacillus ureilyticus	SES13319	--EH-KE--KGL-VY--------EEL-ENEIPALKKAAE STNDQM KY-EV--MIQFADR-EK--H-LKL--Q
Jeotgalibacillus alimentarius	WP_041122599	--NE-KE--QAL--Y---A---RE-LESNVIPALREKAE KSSDQM AY-EV--MMQFLDR--K-IH-L-L--Q
Jeotgalibacillus malaysiensis	WP_039809189	--NE-KE--QAL--Y---A---RE-LEQNVIPSLRSKAE KSSDQM AY-EV--MMQFLDR--K-IH-L-L--Q
Jeotgalibacillus salarius	WP_134379895	--NE-KE--QAL--Y---A-I-RE-LE-NVIPTLRNKAE KSTDQM AY-EV--MMQFLDR--K-IH-L-L--Q
Jeotgalibacillus soli	WP_041089804	--QQ-KD--QAL--Y---A---RDELEKETIPAIRKKAE QSDDQM AY-EV--MLQFLDR--K----L-L--Q
Kurthia senegalensis	WP_010302992	--DQ-KT--EAL--Y---A---RDELV-EIIPRAHAEAQ QSDDQM KV-EV--LNQFVDR-EK-M--L-L--Q
Kurthia sibirica	WP_109307241	--DQ-KT--EAL-VY---A---RDEIN-DIIPAMRKKAE ASNDQM AV-EV--MAQFVDR-EK-I--L-L--Q
Lactobacillus backii	WP_068224829	--TK-KD--DAL--Y--------NET-TT-LPAANKKAQ TSGNQM DV-AV--LTQFD-R--K-I--LKLA--
Lactobacillus bifermentans	WP_057903561	--DK-KD--DAL--YL------VQELRANTIPAAEKKAK TTGDQM D--TVT-LNQFVDR-EK----LKLA--
Lactobacillus concavus	WP_057825445	--EQ-KA--DAL-VY----KV--EELEQQQIPALLAEAE QSGDQM KV-EA--L-QFADR-EK-TH-L-LA--
Lactobacillus coryniformis	WP_003677501	--QK-KD--DAL----------RTELQT--IPEANQKAQ ASGSQM D--TV--LNQFE-R--K-V--LKLA--
Lactobacillus dextrinicus	WP_057757541	--EQ-KD--DAL-VY----KV--Q-LQENQIPQLLAAAE QSGDQM QV-EA--L-QFADR-EK-TH-LELA-Q
Lactobacillus iwatensis	WP_125980253	--TK-KD--DAL--Y--------NET-TT-LPAANKKAQ TSGNQM DV-AV--LTQFD-R--K-I--LKLA--
Lactobacillus rennini	SFZ87893	--QK-KD--DAL-V--------RTELQT--IPEASQKAE QSGDQM D--AV--LNQFE-R--K-V--LKLA--
Lentibacillus persicus	WP_090083243	VFDK-KD--DAL--Y-----V-RDELE-E-IPDLKQKAE QSGNQM DV--V--MIQFA-R--K-VH-LRL--Q
Listeria aquatica	WP_036071879	--DK-KD--QAL--Y--------E-IQD--LPELKKKAE QTGDQM DF-EY--LMQFADR-EK-VH-LKL--Q
Listeria booriae	WP_036085884	--DK-KD--QAL--Y-------QEEIST--LPELRKKAE QSGDQM DF-EV--LTQFADR-EK-VH-LKL--Q
Listeria cornellensis	WP_036078239	--DK-KD--QAL--Y-------QEEINT--LPELRKKAE QSGDQM DF-EV--LTQFADR-EK-VH-LKL--Q

Other Bacteria (0/>200)

```
Listeria costaricensis              WP_099222294  --DK-KD--QAL--Y-------IEEINTQMLPALRKKAE ESGDQM DF-EV--LTQFADR-EK-VH-LKL--Q
Listeria fleischmannii              WP_007472466  --DK-KD--QAL--Y--------EEIND--LPELRKKAE QTGDQM DF-EV--LTQFADR-EK-VH-LKL--Q
Listeria grandensis                 WP_036064310  --DK-KD--QAL--Y-------QEEINT--LPELRKKAE QSGDQM DF-EV--LTQFADR-EK-VH-LKL--Q
Listeria ivanovii                   WP_003720288  --DK-KD--QAL--Y--------EEINT-MLPELRKKAE QTGDQM DY-EV--LTQFADR--K-V--LRL--Q
Listeria kieliensis                 WP_115752045  --DK-KD--QAL--Y--------E-IQD--LPELKKKAE QTGDQM DF-EY--LMQFADR-EK-VH-LRL--Q
Listeria marthii FSL S4-120         EFR87209      --DK-KD--QAL--Y--------EEINT-MLPELRKKAE QTGDQM DY-EV--LTQFADR--K-V--LRL--Q
Listeria monocytogenes              WP_061104984  --DK-KD--QAL--Y--------EEINT-MLPELRKKAE QTGDQM DY-EV--LTQFADR--K-V--LRL--Q
Listeria rocourtiae                 WP_036072353  --DK-KD--QAL--Y-------QEEINT--LPELRKKAE QSGDQM DF-DV--LTQFADR-EK-VH-LKL--Q
Listeria seeligeri                  WP_012986083  --DK-KD--QAL--Y--------EEINT-MLPELRKKAE QTGDQM DY-EV--LTQFADR--K-V--LRL--Q
Listeria thailandensis              WP_122865341  --DK-KD--QAL--Y--------E-IQD--LPELKKKAE QTGDQM DF-EY--LMQFADR-EK-VH-LRL--Q
Listeria weihenstephanensis         WP_036062457  --DK-KD--QAL--Y-------QEEINT--LPELRKKAE QSGDQM DF-EV--LTQFADR-EK-VH-LKL--Q
Listeria welshimeri                 WP_011702758  --DK-KD--QAL--Y--------EEINT-MLPELRKKAE QTGDQM DY-EV--LTQFADR--K-V--LRL--Q
Lysinibacillus acetophenoni         WP_097148829  --R---E-YHN--VY----Q--KQ-MMSNQLPKLQNEAI EQNDMI SK-----FLQ------K-I--L-----
Lysinibacillus chungkukjangi        WP_107936173  --Q---D-YHN--MY---A-M-KK-LILNQLPKIEQAYE LSNNPL DK-R---LQAS-D---K-Q--L-----
Lysinibacillus endophyticus         WP_121213819  --R---D--KNLTL--------KR-LI-HTLPALEQSLA TSTNPM DK--VV-L-N------K-I--L-----
Lysinibacillus halotolerans         WP_122971751  -FD---D--HR--L------M-KK-LL-HDLPQLEKKAS TSANPM DQ-L---L-SS-----KKI--L-----
Lysinibacillus manganicus           WP_052124086  --R---E-YHN--------Q--KQ-MMTNQLPKLQNEAI EKNDLI SK-R---FMQ------K-I--L-L---
Lysinibacillus massiliensis         WP_052126302  --N---EFYHN---H---LDI-KQHLITN-LPKLQKESV SDSDSW QQ---S-LEN----I-K-M--L-L---
Lysinibacillus odysseyi             WP_036158798  --K---E--H---VY-----I-RHHMQDV-LPAFQQKAR TSENPM IN-E-Q-LHTSF------I--LE----
Lysinibacillus saudimassiliens      CEA05122      M-R--QS-HD-LSVIL------QY-FTSRELPRIEAMAA QDPQSL AP-AFE-A-NQLQR--Q----L--A--
Lysinibacillus sinduriensis         WP_036198515  --Q---E-YHNL-VY---A-M-KK-LCDNHLPKLEQSIR LSNNPL DK-R---LQNS---V-K----L-M---
Lysinibacillus sp. 2017             WP_108712690  --E---E--HN---Y--SL-I-KQHMEEVILPAISQAVI EQEDPF KRHE-Q-V--Q-------M--LEV---
Lysinibacillus sp. BF-4             WP_081892651  M-R--QS-HD-LS-IL------QY-FTSRELPRIEAMAA QDPQSL AP-AFE-A-NQLQR--Q----L--A--
Lysinibacillus sp. Marseille-P      WP_106778905  --R---E-YHN--VY----Q--KQELLT-ALPQYQNEAV EYNDLI RK---D-LNQ------Q-I--L-L---
Lysinibacillus sp. SYSU K30002      WP_126658551  --H---D--HNLT-Y-----M-RD-LLKNELPKLEALVA TSSNPM DQ-R---L-TS---I-K-I--L-----
Lysinibacillus sp. YLB-03           WP_118875944  --Q---D-YHNLT-Y-----M-KK-LY-HQLPKLKQEFV DSSNPM DQ-R---L-NS-----K----L-----
Lysinibacillus telephonicus         WP_126296021  M-Q---D--HN---Y-------KQ-LL-NVLPQVEQSVV ESKNLM DK-R-V-L-NS-----K----L-----
Lysinibacillus xyleni               WP_097074965  --R---D--QNL--Y-------KQ-LIKNSLPALEQSLA ASTNPM DK-RVV-LQSS-----K----L-----
Mycobacteroides abscessus subs      SHP74254      --EH-KE--HAL-VY--------DELNQSTIPKLKQKAE QTQDQM AF-EV--MMQFADR--K-I--LKL---
Paenibacillus sp. FSL R5-0490       WP_076258248  --DN-KE--HAL-VY--------EELHE-TIPELKKKAE ESNDQM KF-EV--MIQFADR--K----LKL---
Paenisporosarcina sp. K2R23-3       WP_119883207  --DQ-KA--QAL--Y---A---RDEITK--IPELQRKAE SSSDQM AY-EV--MAQFVDR-EK-I--L-L--Q
Paraliobacillus ryukyuensis         RBO97154      --EH-KE--KGL-VY-------MEEIQK-EIPALRKAAE TTNDQM KY-EV--MIQFADR-EK----LKL--Q
Planococcus halotolerans            WP_112222536  --DQ-KM--QAL-VY---A---RDEIL-ETIPALRKKAE ASDDQM AY-EV--MVQFVDR--K----L-L--Q
Planococcus massiliensis            WP_052651500  --DQ-KT--QAL-VY---A---RDEIL-ETIPALRKKAE QSNDQM AY-EV--MAQFLDR-EK----L-L--Q
Planomicrobium glaciei              WP_036803231  --DQ-KT--QAL-VY---A---RDEIL-ETIPALRRRAE QSNDQM AY-EV--MAQFLDR-EK----L-L--Q
Rummeliibacillus pycnus             WP_102692019  --EQ-KT--QAL-VY---A---KEEIVT--LPALKQKAE SSQDQM DV-E---LSQFV-R-EK-V--L-L--Q
Salirhabdus sp. Marseille-P466      WP_102029120  --EH-KE--KAL-VY--------EELQKTEIPQIRKAAE ATNDQM KF-EV--LIQFADR-EK-IH-LKL--Q
Solibacillus isronensis             WP_079526791  ---I--D--Q---VY--TL-I-KQHMRDVVLPALQKEII DGHNPF KQHE-K-IE-Q---I---M--LEL---
Solibacillus kalamii                WP_087616649  ---I--D--Q---VY--TL-I-KQHMRDIVLPSMQKEIV EGQNPF KQHE-K-IE-Q---I---M--LEL---
Solibacillus silvestris             WP_065215941  ---I--D--Q---VY--TL-I-KQHMRDVVLPSMQKEIV EGQNPF KQHE-K-IE-Q---I---M--LEL---
Solibacillus sp. R5-41              WP_099423642  ---V--E--Q-V-V---SL-I-KQHLQEVILPTIQQALL ENTDPF KQNE---LQ-Q-------M--LE----
Sporosarcina globispora             WP_053432984  --EN-KE--HAL-VY--------EELHENTIPELKKKAE ESNDQM KF-EV--MIQFADR--K----LKL---
Sporosarcina newyorkensis           WP_009496633  --EQ-KT--QAL-VY---A---IEEIN-TIIPELHRKAE LSDDQM MV-EV--MAQFVDR-EK----L-L--Q
Sporosarcina sp. P13                WP_099690084  --EQ-KT--QAL-VY---A---VEEITQTIIPELHKKAQ QADDQM MV-EV--MAQFVDR-EK----L-L--Q
Sporosarcina sp. P18a               WP_099674753  --EQ-KT--QAL-VY---A---IDEINSTIIPELHKKAQ QSDDQM MV-EV--MAQFVDR-EK----L-L--Q
Sporosarcina sp. P19                WP_099690852  --EQ-KT--QAL-VY---A---IDEINSTIIPELHKKAQ QSDDQM MV-EV--MAQFVDR-EK----L-L--Q
Sporosarcina sp. P2                 WP_099632335  --EQ-KT--QAL-VY---A---IDEINSTIIPELHKKAQ QADDQM MV-EV--MAQFVDR-EK----L-L--Q
Sporosarcina sp. P20a               WP_099677069  --EQ-KT--QAL-VY---A---IDEINSTIIPELHKKAQ QSDDQM MV-EV--MAQFVDR-EK----L-L--Q
Sporosarcina sp. P34                WP_099695659  --EQ-KT--QAL-VY---A---IDEINSTIIPELHKKAQ QSDDQM MV-EV--MAQFVDR-EK----L-L--Q
Sporosarcina sp. PTS2304            WP_114923808  --EQ-KT--QAL-VY---A---VDEINQTIIPELHKKAK QADDQM MV-EV--MAQFVDR-EK----L-L--Q
Sporosarcina ureae                  WP_029053830  --EQ-KT--QAL-VY---A---IHEINSTIIPELHKKAQ QSDDQM MV-EV--MAQFVDR-EK----L-L--Q
Trichococcus alkaliphilus           WP_106450708  --QK-KD--EAL--Y-----V-ME-LQKNAIPKAIQTAE VSQSQM DV-IV--L-QFLDR-EK-TH-LRLT-Q
Trichococcus collinsii              WP_086985413  --QK-KD--EAL--Y-----V-ME-LQKNAIPKAIQTAE VSQSQM DV-IV--L-QFLDR-EK-TH-LRLT-Q
Trichococcus flocculiformis         WP_086988520  --QK-KD--EAL--Y-----V-ME-LQKNAIPKAIQTAE ASQSQM DV-IV--L-QFLDR-EK-TH-LRLT-Q
Trichococcus pasteurii              WP_086943361  --QK-KD--EAL--Y-----V-ME-LQKNAIPKAIQTAE VSQSQM DV-IV--L-QFLDR-EK-TH-LRLT-Q
Trichococcus patagoniensis          WP_108031350  --QK-KD--EAL--Y-----V-ME-LQKNAIPKAIQTAE VSQSQM DV-IV--L-QFLDR-EK-TH-LRLT-Q
Vagococcus entomophilus             WP_126821675  ---K-KD--DAL--Y--------EELNTQ-IPDATTLAQ KTNDQM DV-IV--LQQFA-R--K-TH-LRLA-Q
Vagococcus fessus                   WP_126831989  --HK-KD--DAL--Y-----V-M-EL--E-IPAAIKHAE ETGDQM DV-TV--LNQFLDR--K-T--LNLA-Q
Vagococcus humatus                  WP_125943547  --QK-KD--EAL--Y-------KEELQT--IPEAVKEAE LSGDQM AV--V--LNQFL-R--K-T--LYLA-Q
Vagococcus sp. SS1994               WP_125957568  --HK-KD--DAL--Y-------IEELQT-TIPEAIAHAE QTGDQM DT-IV--LNQFVDR-EK-T--LKLA-Q
Vibrio vulnificus                   WP_133351607  --ET-KE--QAL-VY-----I--EEIHE-TIPELRKSAE SSNDQM KF-EV--MLQFA-R--K--H-LKL---
Weissella sp. 26KH-42               WP_133363539  -FKE-MD--HNL--Y---A---IQ-LQQNIIPAAQAKAE ASGDQM DF-TVS-RQQFL-R-EK-A--L-L--Q
```

Supplemental Figure 43

A partial sequence alignment of the DUF456 domain-containing protein containing a six amino acid deletion (boxed) that is exclusively shared by all members belonging to the Meyeri clade and absent in all other bacteria.

```
                                                     164                                                         228
Meyeri Clade (3/3)
Lysinibacillus meyeri                   WP_107839309 GSIIRQQLFEGSREKGLALCQFEQT A KKVLLVMGGSQGSVVLNDALRMNLTNLLQRYHIIHLCGK
Lysinibacillus fluoroglycofenilyticus   WP_107942786 ----------------E-------- - ----------L------------S---E----------
Bacillus ndiopicus                      WP_042475552 ---------Q-------K------A S --I-------L------------AD---K-N-------
Other Bacteria (12/>300)
Acetitomaculum ruminis                  WP_092871510 --P--SE--S-D---A-KF-G--DD G -P-IM-I---S---II-NT--GV-PE-VKE-KV------
Agathobacter ruminis                    WP_031542598 --P---E--N-DAQRAFEF-G-TDR S -P---II---S--K-I---V-EIVPT-VDSFN-------
Clostridium arbusti                     WP_035776513 -TP--KE-L---KIE--R--N-QD- H -P-IFII---L--K-I--TV-KAIDDI-LK-NV--I---
Clostridium botulinum                   WP_013725780 -TP--EE--K--KL--YKI-N--EK E -P--MII---L--K-I-ENI-N-IDE--KK-N---I---
Clostridium massiliodielmoense          WP_085829338 -TP--EE--K--KI--YEI-K-KDE S -P--MII---L--K-I-KSI-DA-SD--KN-N---I---
Clostridium novyi                       WP_039249011 -TP--EE--K--KI--YEI-K-KD- T -P--MII---L--K-I-KSV-DA-S--IKK-N---I---
Clostridium pasteurianum                WP_041711495 -TP--KE-L----MN--R--K-SDL N -P-VF-I---L--K-I--TI-K-IDSI-SK-NV--I---
Roseburia faecis                        WP_055067581 --P--RE--H-DA--S-HY-G-PDH S -P---IV---S--K-I-E-V-KV-PE--EQFY-------
Roseburia inulinivorans                 OLA66648     --P---E-LN-NPSNAIK--R--N- E -P-I-II---T--RAI-T-I-DL-PE--K--N-------
Geobacillus vulcani                     WP_031406058 -AVV-EE-KH--A-Q-RRI---DDR N -P---A----L--KKI-----A--ST--AEFD---I---
Lachnobacterium bovis                   WP_022748182 --P--AE--G-DPN-AIEY-H-TD- T -PI--IV---S--K-I--SV-KV-PE--EQFN-------
Legionella beliardensis                 WP_115302110 -TP--P---L-A-TQ---R---NDD K -PC---------ASS--AII-QS-SE-T-VFQ-------
Anaerocolumna aminovalerica             WP_091686971 -TP---E--S-NKLA--DF-G-TAN   -P-I--I---T----V-E-V-KV-PT--KQ-QV------
Anaerocolumna xylanovorans              WP_073590952 -TP---E--A-NKLL--DF-G-TSE   -P----V---T--AAV-E-V-QA-PE--KK-QV------
Anaerosporobacter mobilis               WP_073286891 --P--KE--S-NKL---DF-G-S-N   -PTI-IV---L--A-V-E-V-KI-DT--KQFQV------
Anaerostipes caccae                     WP_129700850 --P--EE--T-------K--D-TSS   RP---II---L---AI-N-V-E-IDE--EDFQ---I---
Anaerostipes hadrus                     WP_022374747 --P--EE--T-K--E--R--G-NED   -P----I---L---AI-N-I-N-IDA--EK-Q------R
Anaerotignum lactatifermentans          WP_087988513 -TP--AE--K-D-Q---EI-G-DG-   -P---Q----L-A-K------ES-PS--KDFD-------
Anaerotignum neopropionicum             WP_066089831 -AP--RE----KK---FEI-G-DG-   -P---M----L-A-K--QC--AQ-PQ---NF--------
Anaerotignum propionicum                WP_066052809 --P--KE--D-N-Q---ET-G-DGN   -P---M----L-A-K--QC--AE-PA---KFD-------
Anoxybacillus sp. UARK-01               WP_080862872 -AVV-EE-KQ--ADT-RKH----KG   -P--------L--KRI-----A--QT--ADFQ-V-I---
Anoxybacillus tepidamans                WP_027410272 -AVV-EE-KQ--AD--REH----KG   -P--------L--KRI-----A--RT--ADFQ-V-I---
Anoxybacillus vitaminiphilus            WP_111646284 -AVV-EE--K-DAN--FEY-R--KE   -P--------L--KRI--V--A--QT-TSDFQ-V-I---
Aureibacillus halotolerans              WP_133580452 -A-V-PD--K-NAA--RT--D-SSS   -P--------M-AQKI--TI-TE-DV--STF-V--I---
Bacillus cecembensis                    WP_057985217 ---------D-DKAR--S--D-TEE   ----------L---I-----E--DE--KE-Q-------
Bacillus oleivorans                     WP_097158530 -AVV-DE--K---D--F--TG-SSR   -P-I--T-----AKSI--FV-DQ-DE---TFQ-------
Bacillus sp. OxB-1                      BAQ11011     -AV--PE--D-V--Q--RYAGLDGL   -P---I-------A-I-----KE-PGI-E--DV------
Bacillus sp. SB49                       WP_026578312 -AVV-EE--T-D-L---SM-G-HKQ   -P-VM-----M--KRI---I-E--DD--KDVQ---I---
Bacillus sp. SG-1                       WP_006834765 -A---EE--T-DKAE-KR-TG-YED   -P---I----L--RKI-ESV-K--VL--EK-Q-------
Bacillus zeae                           WP_119110969 -PV--A-IKN---K---E--G-DDK   -P-I------L-AQKI--TV-NC-SD--RN-Q-------
Bariatricus massiliensis                WP_066735007 --P---E-MT----RAR-F-G--EI   -P-I-II---L-AAAV---V-NI-PQ--EK-QV------
Bhargavaea beijingensis                 WP_092097285 -PV---E-----KD---RISG-SGR   -P-----------A---E-V-S--PE--KTTD------R
Bhargavaea cecembensis                  WP_040228587 -P---PE-----KKE--R-AGLSGS   RP-----------A-----V-S--PE---THEV------
Bhargavaea ginsengi                     WP_092050998 -PV---E-----KDE--SIAG-SGR   -P-----------A---E-V-SG-PE--KTTD-------
Blautia sp. OF03-15BH                   WP_117766630 --P---E-LK-NKL-A-DF-G-TAD   -P----I-------IV--SI-GI-PE--KTFQV------
Brevibacillus brevis                    WP_012685998 -AVV-EE-KQ-RK-A---Y-S-TTK   -P--------L-ARRI-E-V-RT-P---EHFS-V-I---
Butyricicoccus sp. 1XD8-22              RKJ31702     ---V--E----NKRV-IES-G-DNS   -S--------L----------S--PE--VD-N-------
Butyrivibrio crossotus                  WP_005604660 --P--RE-LS-DKAA--K--G-NTQ   -P----I---L-A-HI---I-AI-PD-IRQ-QV--I---
Caldanaerobius fijiensis                WP_073342161 -NPV-S---T----E-----N-TSR   -P--------L---KI-NLV-AI-PE--R-FQVV-I---
Caldanaerobius polysaccharolyt          WP_026486398 -TPV-S--LK----E---F-S-TSK   -P----I---L---KI-SLV-AI-PD--R-FQVV-I---
Caminicella sporogenes                  RKD22276     -IPV-ES-LK-------GI-G-IEN   -P-IV-I------K----SI-H--DFI-KKFQVC-I--R
Candidatus Arthromitus                  WP_005806734 -TP--KE--L--EI--KEI-K-KNN   -PII---------TFI-NLI-K--DR--VKFN---I---
Candidatus Arthromitus sp. SFB          WP_014094437 -TP--KE--L--EI--KEI-K-NSN   -PIIF---------FI-NLI-K--DK--DKFN---I---
Caryophanon latum                       WP_066464260 -----SE--T-T-Q---S--G-DG-   RQ-V---------AII---V-Q--HT--PQFDV------
Caryophanon tenue                       WP_066542549 -----SE--Q----R---R-G-DG-   RQ-V-I-------AIV-N-V-E--HI-TPQFDV------
Clostridium aurantibutyricum            WP_077851076 -TP--DE--Q---I---EI-G-NGD   -P--MII---L--K-I---V-EE-NE--K--NVV-I---
Clostridium botulinum B str. E          CDH89712     --P--NEILH----E--KI-G-K-E   -E-I-I----L--KII--QI-GK-NL--RDFN---I---
Clostridium collagenovorans             WP_072829495 -TP--SE--K--K---KSF-G-KNN   -P-IML----L--K----S--GV-DK--KS-NV--I---
Clostridium collagenovorans DS          SHH43656     -TP--SE--K--K---KSF-G-KNN   -P-IML----L--K----S--GV-DK--KS-NV--I---
Clostridium fallax                      WP_072893742 -TP--ES----NKNN-RRFLN--TE   -DII------L--KII--TI-N--KE--LKFN-V-----
Clostridium felsineum                   WP_077892739 -TP--DE--Q---I---EI-G-NGD   -P--MII---L--K-I---V-EE-NE--K--NVV-I---
Clostridium frigidicarnis               WP_090041823 -TP--EE--F-N-D---QI-KLNKD   -P-IFI----L--KFI--TV-ES-D-I-ESFYVV-I---
Clostridium homopropionicum             WP_052220745 -TP--EE--K--KI--KLI-A--EE   -PI-MII---L--KII--TV-EM-DK--IK-N---I---
Clostridium intestinale                 WP_034856236 --P--EEIL---K---KKIAG-TDD   -EII-I----L--QII-N-V-G--SK--KEFN---I---
Clostridium intestinale URNW            ERK29721     --P--EEIL---K---KKIAG-TDD   -EII-I----L--QII-N-V-G--SK--KEFN---I---
Clostridium mediterraneense             WP_066872191 -TP--ED-LH--K-E-RRF-N-AKN   -P---II---L--K-I--SI-D--N---IGFN-------
Clostridium oryzae                      WP_079425031 -TP--KE-L----I--MRF-G-DKS   RPI--II---L--K-I---I-K-INQI-EK-QV--I---
Clostridium roseum                      WP_077834065 -TP--DE--Q---I---EI-G-NGD   -P--MII---L--K-I---V-EE-NE--K--NVV-I---
Clostridium sp. 27_14                   OKZ78011     -TP--EEILK---I--KEI-G-KDE   -EI---I---L-AKSI--EV-K----I-KEFNV--I---
Clostridium sp. TF01-11                 WP_055944979 --P--EE--K-DK----KF-G-DNS   -P-I-II---L-A-AV-N-V-GI-PE--KKFQV------
Clostridium sporogenes                  WP_045884963 -TP--KE-L---KA--IQ--G-KDD   -TI--II---L--KII-EIV-K--DDI-LKFN---I---
Clostridium sporogenes ATCC 15          EDU36093     -TP--KE-L---KV--IE--G-KDD   -PI--II---L--KII-EIV-K--DDI-LKFN---I---
Clostridium tetani 12124569             CDI49882     -TP--EE--L-NE-E-KKI-G-KNN   -PIV-L----L--KI--NLI-E-IEE--KKFN---I---
Clostridium vincentii                   WP_106059443 --P--EEI-K---L--FDI-G-TGK   -E--F-----L--K-I--VI-E--DE-INEWD---I---
Desnuesiella massiliensis               WP_055668429 -TP--EE-LL-------SF-G-T-N   RP---II---L--KYI--LV-NS-ME--KK-NV--I---
Dorea sp. OM02-2LB                      WP_117604197 --P---E-LS-NKIAA-D--G-SAD   -P-I--I---L----V-N-V-LA-PE--EQF-V------
Edaphobacillus lindanitolerans          SIT80731     -PV--PE-----K-A--R-AGLSGK   RP-----------A---E-V-SQ-PA--RTMDV-----Q
Epulopiscium sp. Nuni2H_MBin00          OOB77728     -TPL--EI-T--KSQ-MK--K-STQ   -P---I----S--ATV---V-AAVLD-VKQFNVV-I---
Eubacterium sp. 38_16                   OLA05253     --P---E--S-NK-E--R--G-DDS   -P--------L-A-AI-NSI-E--NE--KQFQ------R
Eubacterium sp. An11                    WP_087314486 --P--KE--D----E--R--G--GD   -P--MA------AAAI-K---S--DA--RQFD-------
Eubacterium sp. An3                     WP_087281170 --P--KE--D-R--E--K--G-KGD   -P--MA------AAAI-K---A--DA--EQFCV------
Eubacterium sp. CAG:274                 CDC19653     -TP--DV-YK--K----EFAHLDG-   -P-M-I--------KI-TSI-AL-PE--KK-D-------
Eubacterium xylanophilum                WP_026834345 --P--KE----DKS---EF-G-TDD   -P----I---L---SV-N-I-NS-DK--EK-Q-------
Falsibacillus sp. GY 10110              WP_121680876 -A-V-KE--N-NPD--YR-----KH   -D--------L-AKRI---V-SA-PS--PSFN-------
Filibacter sp. TB-66                    WP_124071167 -A---PE--N-R--E--RITGLTGD   -QI-II-------A-V-K-I-KD-AEIM-TFDL------
Geobacillus genomosp. 3                 WP_020958441 -A-V-EE-KH--AAQ-RQM---DGK   -P---A----L--KKI-----A--ST--AEFD---I---
Geobacillus jurassicus                  WP_066229507 -AVV-EE-KD-NA-Q-RKM---DGK   -P---A----L--KKI-----A--ST--AEFD---I---
Geobacillus kaustophilus                WP_044730371 -AVV-EE-KD-NA-Q-RKM---DGK   -P---A----L--KKI-----A--ST--AEFD---I---
Geobacillus sp. 44B                     WP_081161025 -AVV-EE-KR-DAD--RK-----KG   -P---A----L--KRI-----A--QT--SDFQ-V-I---
Geobacillus sp. A8                      WP_021322027 -AVV-EE-KD-NA-Q-RKM---DGK   -P---A----L--KKI-----A--ST--AEFD---I---
Geobacillus sp. Y4.1MC1                 WP_013401785 -AVV-E--KQ-NAD--R------KG   -P---A----L--KRI-----A--PK--ADFQ-V-I---
Geobacillus sp. ZGt-1                   WP_047757498 -AVV-EE-KD-NA-Q-RKM---DGK   -P---A----L--KKI-----A--ST--AEFD---I---
Geobacillus stearothermophilus          WP_053414270 -AVV-EE-KD-NA-Q-RKM---DGK   -P---A----L--KKI-----A--ST--AEFD---I---
Geobacillus thermocatenulatus           WP_025950337 -AVV-EE-KD-NA-Q-RKM---DGK   -P---A----L--KTI-----A--ST--AEFD---I---
```

Other Bacteria (12/>300)

Geobacillus thermoleovorans	WP_033845600	-A-V-EE-KD--A-Q-RKM---DGK	-P---A----L--KKI-----A--ST--AEFD---I---
Geobacillus zalihae	WP_081132832	-AVV-EE-KD-RA-Q-RKM---DGK	-P---A----L--KKI-----A--ST--AEFD---I---
Gracilibacillus dipsosauri	WP_109985545	-AVV-EE--N-N-K--YE-TGLDSR	-N---I----A--KKI---I-EG-DE--SDF----I---
Gracilibacillus orientalis	WP_091481241	-AVV-EE--T-N----F-MTA-TSQ	-N---I----A--QKI-Q-V-EG-ED--NQFQV--I---
Halobacillus aidingensis	WP_089653102	-AVV-EE----E-S------E-HKQ	-P-VM-----T--KRI---I-D--DE---EVQ-V-I---
Halobacillus alkaliphilus	WP_089754046	-AVV-EE--Q-K-S---SY-N-HKQ	-P-IM-----T--KR--E-I-N--DT--KNVQ---I---
Halobacillus dabanensis	WP_075035681	-AVV-EE----T-S------S-HKQ	-P-VM-----T--KKI--TI-D--DE--KEVQ-V-I---
Halobacillus mangrovi	WP_085030648	-AVV-DE----Q-A---SY-G-HKQ	-P-I------T--KA----V-H--DE--KDVQVV-----
Halobacillus trueperi	WP_115825090	-AVV-EE----D-S------E-HKQ	-P-VM-----T--KRI---I-D--DE---EVQ-V-I---
Hungatella hathewayi	RGC81492	--P--EE--T--K----K--D-TSS	-P---II---L---AI-N-V-E-IDE--EDFQ---I---
Jeotgalibacillus alimentarius	WP_041122625	-A---EE--A-A----I-FSGID--	-P-I------L-AKGI-ATV-SA-PE-T-H-S-------
Jeotgalibacillus sp. R-1-5s-1	WP_134371430	-A---KE--D-T-----E-TGLTSS	-P-M------L-AKGI---V-E--ST-TEH-SV------
Kurthia huakuii	WP_029498537	--V--PA--S-N--S--KY-G-SND	-PTIMI----L--KFI---V-A--EE--AT-N-------
Kurthia massiliensis	WP_026021959	--V--PA--S-N--S--TY-G-TNG	-PTIMI----L--KFI---V-D--EE---T-N-------
Kurthia sibirica	WP_109304972	-----SA----D-VR--EI-H-TNG	--TI------L--KFI---V-N---E--VT-NV------
Kurthia sp. 3B1D	WP_126990612	--V--PA--S-N--S--QY-G-SNN	-PTIMI----L--KFI---V-A--EE--KT-N-------
Kurthia sp. Dielmo	WP_044527218	--V--PA--S-N--S--QY-G-SNN	-PTIMI----L--KFI---V-A--EE--KT-N-------
Kurthia zopfii	WP_109350712	-----SE----D-QR---R-H-DNG	-STI-I----L--KFI---V-N---E--LENNV------
Laceyella sacchari	WP_132223445	-A---DE-KR----R--T--G-QAD	-P---I----L--QKI-QVV-Q--DS-MRYFQ-V-----
Legionella adelaidensis	WP_058461729	-TP--K---L-NK-------G-TKE	-PC--FI-----ANS--TCI-QS-NH-IEHFQV------
Legionella brunensis	WP_058441903	-TP--TL--K--KA------G-NDK	-PC---------AAP--N---QC-ST-CED-QV------
Legionella clemsonensis	WP_094091241	-TP--S---N--KK---S--G-TTE	-PCI------L---L--RT--ET-SAICEHFQ-------
Legionella donaldsonii	WP_115221730	-TP---E--K--K----D--G-NYD	-PCV----------K--T---SI-DP-TAS-QV------
Legionella massiliensis	WP_044012052	-TP---E--K--KSA--D--G-NKD	-PC----------LR--K---AA-PE-CEQ-QV------
Legionella nagasakiensis	WP_133127150	-TP--KA--H--KT------G-NED	-PC----------NAM-ACV-KA-DF-GE-FQV------
Legionella nautarum	WP_058503976	-TP---E--N--K-A-----G-KAE	-PC---V------MR--KVV-EA-PL-SGD-Q-------
Legionella rubrilucens	WP_058532198	-TP--R---N---QR-----G-NDK	QAI---I---L-AQAI-Q-V-EA-PR-S-N-Q-------
Lysinibacillus acetophenoni	WP_097148841	---V--E----KKN---RI-G-DDN	-Q-------------------S--PE--VDFN-V-----
Lysinibacillus boronitolerans	WP_036078983	-----PE----E-AR---F-G-STL	-P--------L------E---K--PE--KQF--------
Lysinibacillus chungkukjangi	WP_107936138	---V-E---K-NAA--KS--G-TDE	-----I----L-------S--S--PE--ASFNV------
Lysinibacillus composti	WP_124762930	---V--E--Q-NKSR-KE--D-KDD	--I--I----L----------S--PE--VN-NV------
Lysinibacillus contaminans	WP_053583270	------E--S-DKA----A-G-TPE	-P--------L----I-E---H--PE---TFQ-------
Lysinibacillus endophyticus	WP_121213833	---V-E---Q-DKDR-MK--G--EN	--T-------L----------S--PE--MEFN-------
Lysinibacillus fusiformis	WP_004230130	-----PE----E-AR---F-G-STL	-P--------L------E---K--PE--KQF--------
Lysinibacillus halotolerans	WP_122971738	---V-E---T-NASV-KKR-G--ER	----------L--A-------N--PE--KEFN-------
Lysinibacillus jejuensis	WP_108307123	-----SS--T-D-D--I-R-E-TGD	-EI--L-------KL---IV-D--PR--A-FD-------
Lysinibacillus macroides	WP_053994808	-----PE--K-EKAR--SF-G-TTL	-P--------L------E---K--PE--KQFY-------
Lysinibacillus manganicus	WP_036185648	---V--E--R-KKSTA-RI-G-DND	-P--------L----V-E---S--PE--VEFN-------
Lysinibacillus mangiferihumi	WP_107896079	------E--N-NKA----F-G-TAS	-P--------L----------H--PE---TFQV------
Lysinibacillus massiliensis	WP_036178848	---V--E----NKRV-IES-G-DNS	-S--------L----------S--PE--VD-N-------
Lysinibacillus odysseyi	WP_036158765	-----------DK-RA-R--G-VP-	----------L----------E--NQ--PMFN-V-----
Lysinibacillus parviboronicapi	WP_107925198	------E--T-NKAQ--S--G-TTS	-P--------L----------Q--PE---TFQV------
Lysinibacillus saudimassiliens	CEA00076	-----SS--T-D----IER-E-AGD	-EI--L-------KL---IV-E--DR--A-FD-------
Lysinibacillus sinduriensis	WP_036198501	---V-E--LK-KGAR-KK--G-TDE	--I-------L-------S--S--PE--AT-NV------
Lysinibacillus sp. 2017	WP_108712674	-----------NK-R---Y-G-TDE	----------L----V-----E--PA--KE-Q-------
Lysinibacillus sp. BF-4	WP_036144695	-----SS--T-D----IER-E-AGD	-EI--L-------KL---IV-E--DR--A-FD-------
Lysinibacillus sp. BK089	WP_132357921	------E--M-D-A---S--G-TTL	-P--------L----------Q-IPE-VKQFQ-------
Lysinibacillus sp. FJAT-14222	WP_053595531	------E--M-D-A---SQ-G-TTL	-P--------L----------Q--PE-VKQFQ-------
Lysinibacillus sp. FJAT-14745	WP_053485877	------E--A-D-A---S--G-TTL	-P--------L---------HQ--PE-IKQFQ-------
Lysinibacillus sp. YR326	WP_134024592	------E--A-D-AE--S--G-TTL	-P--------L----------Q--SE--KQFQ-------
Lysinibacillus sp. ZYM-1	WP_054611894	-----PE-LQ-E-A----Y-G-TTL	-P--------L----------K--PE--KQFY-------
Lysinibacillus sphaericus	WP_024361630	------E--N-NKA----F-G-TAS	-P--------L----------H--PE---TFQV------
Lysinibacillus tabacifolii	WP_108030304	------E--N-NKA----F-G-TAS	-P--------L----------H--PE---TFQV------
Lysinibacillus telephonicus	WP_126295597	---V-E---D-KKIR--S----DAS	----------L------E---S--PE--EA-QV------
Lysinibacillus varians	WP_025219559	------E--N-NKA----F-D-TAS	-P--------L----------H--PE---TFQV------
Lysinibacillus xylanilyticus	WP_049665426	------E--T-D-A---S--D-TTL	-P--------L----------Q--PE-VKQFQ-------
Lysinibacillus xyleni	WP_097074951	---V-E---Q-DKDR-MK--G--EN	--T-------L----------S-IPE--MNFNV------
Mediterraneibacter sp. KCTC 15	WP_119297888	--P---E-LS-NKIAA-D--G-SAD	-P-I--I---L----V-N-V-LA-PD--EQF-V------
Oceanobacillus halophilus	WP_121206094	-AV--EE--Q--------FAGLTKE	-P---I----G--QKI-ETV-SS-QE--SAFQ---I---
Oceanobacillus iheyensis	WP_106896220	-AV--DE----KK-A---FAGLSSA	-P---I----G-AQKI-ETI-NS-DH--ETF----I---
Oceanobacillus senegalensis	WP_085992838	-AV--EE--Q-D------YAGLSKG	-P---I----G--QKI--TV-ES-SE--NNFQ---I---
Oribacterium asaccharolyticum	WP_009536531	-AP--EE-LQ----E--K--G-SGE	-P--M-I---L---AI-T-V-E--RK-GEN-D---I---
Oribacterium parvum	WP_009534131	-AP--EE-LQ----E--S--S-SGE	-P--M-I---L---AI-T-V-E-IES-G-N-D---I---
Oribacterium sp. oral taxon 10	WP_009429331	-AP--EE-LQ----E--R--G-SGE	-P--M-I---L---AI-T-V-E--RK-GEN-D---I---
Ornithinibacillus contaminans	WP_047979836	-AV--EE--Q-NK-C-----G-TRE	-P---I----G-AKKI--TV-AS-AS----FQ---I--Q
Ornithinibacillus scapharcae	WP_010093803	-AV--EE--Q--K----T-AG-T-Q	-P---I----G-AQKI--TV-AS-DV--EKFQ-V-I---
Paenibacillus alvei	SYX83473	-AVV-SE-LL---SR--NA-G-TR-	-P---F-------KR--ERI-GS-PK---Q-Q-------
Paenibacillus alvei TS-15	EPY06494	-AVV-SE-LR---SR--NA-G-TR-	-P---F-------KR--ERI-SS-PK--EQ-Q---I---
Paenibacillus ginsengarvi	WP_120745596	-LP--DEMLR--AI---H--E-HKQ	-P--------L--K-I-E-V-GS-KR--E-FQ-V-----
Paenibacillus koleovorans	WP_127579637	-LP--DEVLQ--AAG-R-M-D-HRQ	-P---I-------RRI---V-EQ-HE---QFQ-V-----
Paenibacillus pinihumi	WP_036708245	-A---E--K--NAQ--REF-R-TSA	-P---I----L--QAI-N-V-AD-RS-AR-FQ-------
Paenibacillus sp. E194	WP_044356872	-AVV-SE-LL---SR--NA-G-TR-	-P---F-------KR--ERI-SS-PK--EQ-Q---I---
Paenibacillus sp. KS1	WP_065291179	-AVV-SE-LL---SR--NA-G-TR-	-P---F-------KR--ERI-SS-PK--EQ-Q---I---
Paenibacillus sp. N2SHLJ1	WP_131014139	-LP--DRML--KAQR-Y---D-HKQ	-P--F-----L-AQAI-E-V-GA-DE--E--Q---I---
Paenibacillus sp. NAIST15-1	GAV13532	-AVV-SE-LL---SR--NA-G-TR-	-P---F-------KR--ERI-SS-PK---Q-Q-------
Paenibacillus sp. UNC451MF	WP_028549285	-LP--E-ILQ-KAHR-FD--D-HKQ	-P--------L-AQKI-K-V-DS-DT--E--Q-V-----
Paenibacillus validus	WP_054798714	-LP--E-ILQ-AAAR--S--G-HSQ	-P--------L-AQRI-QTV-AS-DT-----Q---I---
Paenisporosarcina antarctica	WP_134209821	-----SE--D-KKQV--QF-D-TGW	-P---I---------V-E-I-S--PQ--EMFD-------
Paenisporosarcina indica	WP_075617779	-----SE----NKMN--KI-G-NGF	-SI--I-------T-----I-T--PR--EM-D-------
Paenisporosarcina sp. HGH0030	WP_016427317	-----SE----RK-T-MR--G--GF	-P---I-------A-V---I-S--PK--EK-DV------
Paenisporosarcina sp. OV554	WP_108585786	-----SE----RKVV--R--G-TGL	-P---I-------AII-ESI-S------DL-D-------
Paenisporosarcina sp. TG-14	WP_017378666	-----SE----KKQV--Q--D-AGW	-P---I---------V-E-I-S--PQ--KMFD-------
Paenisporosarcina sp. TG20	WP_019415497	-----SE--Q-KKDV--RM-G-VGF	-P---I-------E-V-E-I-S---QI-NM-DV------
Parageobacillus caldoxylosilyt	WP_017435880	-AVV-EE-KR-DAD--RK-----KG	-P---A----L--KRI-----A--QT--SDFQ-V-I---
Parageobacillus genomosp. 1	WP_043903381	-AV--EE-KQ-DAD--RQ----QKG	-P---A----L--KRI-----A--PK--ADFQ-V-I---
Parageobacillus thermantarctic	WP_090951464	-AVV-E--KQ-NAD--R------KG	-P---A----L--KRI-----A--QT--ADFQ-V-I---
Parageobacillus thermoglucosid	OUM91500	-AVV-E--KQ-NAD--R------KG	-P---A----L--KRI-----A--PK--ADFQ-V-I---

Other Bacteria (12/>300)

Parageobacillus toebii	WP_062678773	-AVV-EE-KR-NAD--REY----KG	-P--------L--KRI-----A--QT--DDFQ-V-I---
Parasporobacterium paucivorans	WP_073994221	--P--RE-LA----A-----G--EE	-P-I-II-------IV-ETV-KI-PA--EK-QL------
Paucisalibacillus globulus	WP_096273665	-AV--EE--Q-DKG------G-QNH	-P---I----G--QKI-ETV-QS-DE--TQFQ-V-I---
Paucisalibacillus sp. EB02	WP_042149310	-AV--EE--Q-DKG------G-QNR	-P---I----G--QKI-ETV-GS-DEI-NQFQV--I---
Planococcus antarcticus	WP_006831492	--L--SE-MK--A---QEI-P-DNE	LP--M--------AMI-S-V-D--ER--EGFN-------
Planococcus citreus	WP_121297504	--V--SE-LD--AAS-RE--H-DN-	H---MI-------ALI---V-E--DT-TKQFN-------
Planococcus donghaensis	WP_008431853	--V--NE-M--TA---REISS-TND	LP--M--------AMI-S-IHN--EQ--KQFN-------
Planococcus faecalis	WP_071154562	--L--SE-M---AI--QEI-S-DND	LP---I-------AMI-S-V-N--ER--KQVN-------
Planococcus halocryophilus	WP_008497515	--V--NE-M--TA---REI-S-ANE	LP--M--------AMI-S-IHN-VEE--KQFN-------
Planococcus halotolerans	WP_112222550	--V--SE-MD--AA--REI-H-DND	LP--MI-------E-I-T-I-S--QVI-ETFNV------
Planococcus kocurii	WP_058385244	--L--SE-M---AI--QEI-SLDND	LP---I-------AMI-S-V-N--ER--KQVN-------
Planococcus maitriensis	WP_112231798	--V--SE-LD--AAS-RE--H-DN-	H---M--------ALI---V-E--DM-TEQFN-------
Planococcus maritimus	WP_068462491	--V--SE-LD--AAS-RE--H-DN-	H---MI-------ALI---V-A--ET-TKQFN-------
Planococcus massiliensis	WP_052651482	--V--SE-M---A-I-KE--H--SD	LP--MI-------A-V-E-I-S--PA-MEA-NV------
Planococcus plakortidis	WP_068869229	--V--SE-LD--AAS-KE--H-DNN	H---M--------ALI-E-V-E--DR-TKQFN-------
Planococcus rifietoensis	WP_058380649	--V--SE-LD--AAS-RE--H-DN-	H---MI-------ALI---V-E--DT-TKQFN-------
Planococcus salinarum	TAA72332	--V--SE-MD--AA--REI-H-DND	LP--M--------E-I-T-I-S--QQ--KTFNV---V--
Planococcus salinus	WP_123164132	-AV--SE-MD--AA--KEI-Y-DND	LP--M--------AII-Q-V-D--KK-TENFS-------
Planococcus sp. CAU13	WP_033543598	--V--SE-M---VV--REIA-LDKE	LP--M--------E-I-T-I-S--QE-TRTFN-------
Planococcus sp. PAMC 21323	WP_038704001	--V--NE-MQ--V---REI-S-SKE	LP-----------AMI-S-IHN--EQ--EQFN-------
Planococcus versutus	WP_065524600	--L--SE-K--TAA--QEI-S-TSS	LP--M---------MI-S-I-N--DR-MEE-N-------
Planomicrobium flavidum	WP_088006284	-AV---E-LQ-TA-A-KRF-G-QR-	LP---I-------A-I-E-V-DR-GD-TEVFN-------
Planomicrobium glaciei	WP_036803211	--V--GE-M---A-I-RS--N-DNG	RP--MI-------A-I-N-I-A--PA--EEFN-V-----
Planomicrobium okeanokoites	WP_084245674	--V--SE-MD--AA--REI-H--ND	LP--MI-------E-I-T-I-S--QAIM-TFNV------
Planomicrobium soli	WP_106531690	--V--SE-MD--AA--KEI-S-DND	MP--MI-------A-I-E-I-T--AE--TQFN-------
Planomicrobium sp. MB-3u-38	WP_101802968	--V--SE-MD--AA--REI-H--ND	LP--MI-------E-I-T-I-S--QAIM-TFNV------
Planomicrobium sp. Y74	WP_121632014	--V--SE-MD--AA--REI-H-DND	LP--MI-------E-I-T-I-S--QVI-ETFNV------
Pontibacillus chungwhensis	WP_036786041	-AVV-EE--Q-DAAR-R---D-TRN	-P--------M--KKI---I-D--DE--KEFQVV-----
Pontibacillus halophilus	WP_026801164	-AVV-GE--Q--EQ---N--G-TR-	-P-M------M--KRI---V-D--DE--NEFQ-V-----
Pontibacillus litoralis	WP_036833505	-AVV-DE--T-NAQ---SMTN-TKS	-P-I------S--KKM---I-D--DE---T-Q------Q
Pontibacillus yanchengensis	WP_036823162	-AVV-DE--Q---Q---SMTN-TKS	-PII------S--KNI-N-I-N--SK--EQFQVV----E
Pseudogracilibacillus auburnen	WP_110395384	-AV--EE--Q-DKNR-YQ-TK-SPN	-----I----I--QKI--IV-Q--PE---KFQ---I--R
Pueribacillus theae	WP_116555464	--P--EEILK--K----KFLG-DLS	-P-ITI----L---K--TVI-D--ET--ED-Q-V-----
Roseburia sp. CAG:380	CDC96623	--P---E--R-------SF-G-TDD	-P-I-II---L-A-RV-E-V-AI-PQ--E-FR-------
Ruminococcus sp. AF14-10	WP_117888749	--P---E-LS-NKIAA-D--G-AAD	-P-I--I---L----V-N-V-LA-PE--EQF-V------
Rummeliibacillus	WP_119415465	-----PA--D-TK----NM-G-QNG	-ETI-I-------KFI---V-S-MVE--KDHN-------
Rummeliibacillus pycnus	WP_102692342	-----PA----K-----EM-G-NNG	-ETI-I-------KFI---V-S-MVE--KNHN-------
Sediminibacillus albus	WP_093216585	-AVV-EE--S-KK-----IAN-DNS	-P---I----G--AKI-Q-V-SQ-DI--EE-----I---
Sediminibacillus halophilus	WP_074600440	-AVV-DE--Q-N------IANLDNS	-P---I----G--AKI-ESV-G--DE--EE-Q---I---
Solibacillus isronensis	WP_079526817	-----------N--R-----G-TTE	----------L---II-----E--PV--EK-Q-------
Solibacillus kalamii	WP_087616670	-----------N--R-----G-TAE	----------L---II-----E--PS--EK-Q-------
Solibacillus silvestris	WP_014823821	-----------N--R-----G-TAE	----------L---II-----E--SS--EK-Q-------
Solibacillus sp. R5-41	WP_099423632	---------D-DKAR--SY-G-TEE	----------L----I-----E--DE--ED-Q-------
Sporosarcina koreensis	WP_040286418	-A---PE--D-D-KT--RMAGLSEG	-P--I--------A---EGI-GC-PE--S-FQV------
Sporosarcina newyorkensis	WP_078816641	-P---PE--N--K-E--RIAGLKSD	RPIFI--------A---E-I-GE-PQ--KN-Q-------
Sporosarcina psychrophila	WP_067209741	-A---PE--N-T--E--RIAGLSGE	-T-QII-------SI----V-KD-PAI-HDFD-------
Sporosarcina sp. BI001-red	WP_116019034	-A---PE----D-KT--RMSGLTEE	-P-VI--------A-------G--QTI--DFQ-------
Sporosarcina sp. D27	WP_025784290	-A---PE----D-KT--RMSGLSEE	-P-VI--------A-------G---AI-KDFQ-------
Sporosarcina sp. EUR3 2.2.2	WP_024534741	-----SE----RKVV--R--G-SGL	-P---I-------A-V-E-I-S------KL-D-------
Sporosarcina sp. P1	WP_099627587	-P---PE--K-D-QE--RKTGLNSK	-P-FII-------A---E-V-KE-PA--KK-Q-------
Sporosarcina sp. P33	WP_081241996	-P---PE--S-DK-R--RMAGLTAE	-P-FII-------A---E-I-AE-PE--A--QL------
Sporosarcina sp. P34	WP_099695656	-P---PE--K-D-QE--RRTGLNSE	-P-FII-------A---E-V-KE-PA--EK-Q-------
Sporosarcina sp. PTS2304	WP_114923812	-P---PE--T-DE-R--QFTG-THD	-PIFI--------A-----V-KS-PE--KQ-Q-------
Sporosarcina sp. ZBG7A	WP_039044222	-A---PE----D-KT--RMSGLSEE	-P-VI--------A-------G--PTI-KDFQ-------
Sporosarcina ureae	WP_029053834	-P---PE--M-D--E--RRTGLNSE	-P-FII-------A---E-V-NE-PD--KK-Q-------
Staphylococcus pasteuri	WP_107999794	-ATV-ED-KQ-DKQR-YQ-TD-K-D	----------L--KK----I-E--EA---Q-QV---T--
Tatlockia micdadei	WP_045099616	-TP--PE--Q--K-A-----G-KKN	-PC----------LK--SIV-NA-PA-SLQ-Q-------
Thalassobacillus cyri	WP_093046917	-AV--EE--K-DKQ---EM-G-SAS	-P--M-----M--KKI--SI-NQ-DE--RKMQ---I---
Thalassobacillus devorans	WP_085506797	-AV--EE----NKQ---EM-G-TAS	-P--M-----M--KKI--SI-NQ-DE--PKMQ---I---
Thalassobacillus sp. TM-1	WP_062443927	-AV--EE----NKQ---EM-G-SAS	-P--M-----M--KKI--SIQNQ-DE--AKMQ---I---
Thermoactinomyces daqus	WP_033099721	-AV--EE-KQ-D--Q---I-R-SPN	-P---I----L-AKRI-E-V-E--VS--EQFQ-V-I---
Thermoactinomyces vulgaris	WP_054095622	-A---DE-KR----R--S--G-QAD	-P---I----L--QKI-QVV-Q--DS-MRYFQ-V-----
Tumebacillus avium	WP_087458987	--P--HEIL--NADR-R-F-G-TKS	-P-I-A----L---KI-----A--DT--K-FQ-V-----
Tumebacillus sp. BK434	WP_132945378	--P--HEIL--NA-R-RSF-G-TPS	-P-I-A----L---KI-----A--DS----FQ-V-----
Ureibacillus thermophilus	QBK26008	--VV-E---K-NKMR--K--G--TR	-P--------L--------I-S--PEI-KDFNV------
Ureibacillus thermosphaericus	WP_016839360	--VV-E---K--KMR--K-----SK	-P--------L--------I-S--PE--KD-NV------
Vallitalea guaymasensis	WP_113672768	-TP--EEILQ-D-A---S--E-STS	-P---MT---L-A-RI--V--ES-DMI-EK-QLV-----
Virgibacillus phasianinus	WP_089060200	-AVV-DE--Q-NKK--F---G-TNN	-P---I----G--IKI-ESV-AG-DK--KEFQ-------
Virgibacillus senegalensis	WP_053217084	-AVV-DE--Q-N------FADLDNS	-P---I----G--AKI-E-V-SH-DE--KD-Q---I---
Virgibacillus sp. LJ137	WP_123314465	-AV---E--Q-DKDT-----G-DRS	-P--------G-AQKI-ESI-ES-PI--KSFQV--I--E
Viridibacillus arvi	WP_053417002	-----PA----RKST-----G-KER	-E-I-I-------KFI---V-A-IVQ--ENHNV------
Viridibacillus sp. OK051	WP_100796974	-----PA----RKA------G-KES	-G-I-I-------KFI---V-S-IVQ--EKHN-------

Supplemental Figure 44

A partial sequence alignment of the DUF456 domain-containing protein containing a one amino acid insertion (boxed) that is exclusively shared by all members belonging to the Meyeri clade and absent in all other bacteria. 12 other bacteria also share this CSI.

Group	Organism	Accession	215		275
**Meyeri Clade (3/3)**	Lysinibacillus meyeri	WP_107841857	GFVVSSITFSAMGYSATYQYTNKEGNTSVIEYHNPAIFG	MNDYVEVKLVDAKGK	SYEPVVEADRSFQPLMEMPPLVNAKKLTTVFYSILFGTVIYLLIRLI
	Lysinibacillus fluoroglycofenilyticus	WP_107942374	------V--G----------VD------RV---K-----	--K----E----EN-	I----AKENQ-----V---------------F-LI-----------
	Bacillus ndiopicus	WP_042477873	--I------N---------------K-G--------L--	--E---------EK-	K-------EQ-----A----I---------V---------------
**Other Bacteria (0/>200)**	Anoxybacillus flavithermus	AST06925	---IVTTS-RVA--E--FEWID-N-KQDEMV--M--LV-		PH-G-LKT-GRMT--VDT-AII--R--N--IW-LFA-LAL-G-L--V
	Anoxybacillus sp. 103	WP_077428654	---IVTTS-RVA--E--FEWID-N-KQDEMV--M--LV-		PH-G-LKT-GRMT--VDT-AII--R--N--IW-LFS-LAL-G-L--V
	Anoxybacillus vitaminiphilus	RAK22221	---LV-TS-K-AD-E-KFTWID-N-KEAETV-EM--LV-		PHKGELQTADR-E-IV---AII-----N--IW-LMG-F-L-W-L---
	Bacillus acidiceler	WP_129688686	----I-TA-T-S--E-K-NWV--DKVQEEMV-NL-SLV-		PH LTTKDK-EA-A---AM------N--IW--FA-AAL-AI----
	Bacillus alveayuensis	WP_044747973	---LV-TS-K-AD-E-KFTWVD-NDKEAEVV--M--LV-		PHKG R-E-II---AII-----N--IW-LMG-F-L-W-L---
	Bacillus andreraoultii	WP_033828514	-YIIVTSI--ILK-Q-SFEWV----KIVTSI-KL-----		LN-WQLL E--V-L-AIFD-Q-FN--LW-FIA-SFF-I----L
	Bacillus anthracis	AIM05383	--IGVTTV--S-K-E-KF-WVD-NEQQVEMK-NL--LV-		PH-GKLLTENKLE-TV-V-AI------N--IW-V-V--LL-IVL---
	Bacillus anthracis str. BF1	EJY91353	--IGVTTV--S-K-E-KF-WVD-NEQQVEMK-NL--LV-		PH-GKLLTENKLE-TV-V-AI------N--IW-V-V--LL-IVL---
	Bacillus aquimaris	RBP07722	----A-TA-TLGN-E-NF--VDQ--AEAIS--TL--LV-		PN-GQLLT-KVME--V---A-I-----N--IW-F-I--G----L---
	Bacillus asahii	WP_119115382	---IV-S--AMT--KESITWVD-K-AEAEL---I-SLI-		PN-WT ENQMK--I-V-AII-----N--VW-VGA-FIL-G-F---
	Bacillus badius	WP_041097027	---AVTT--TLS--E-KFE-V--D-KAAEETF-I---V-		PN EDK-E-IVD--AFI-----N-TLW-LAA-ALL-G----L
	Bacillus bataviensis	WP_007084916	----V-SV--GI--HEKFNW-D-H-TQA-V--KM---T-		PHD -K--I---AFI----AN--IW-LMS-L-L-G-----
	Bacillus butanolivorans	WP_053346822	---IITS--A--D-K-NFDWVD-HDELAKQ-FNL--LV-		PH R-E--VN--AII-----N--IW-MGI-L-L-G-----
	Bacillus campisalis	WP_046524117	--I-VASI--FAD-Q-EFT--D-N-VQATY---M--LI-		PN EGR-A-IV-T-AII-S---N--IW-LAA-LLL-W----V
	Bacillus cecembensis	WP_057982164	----ATS---I-N-ESKFE-VDTLD-VKQTT-MM-----		MN-YTELSEDKMT-V----A-ID-R-----VW-----S-L------V
	Bacillus cereus	KWW52717	--IGVTTV--S-K-E-KF-WVD-NEQQVEMK-NL--LV-		PH-GKLLTENKLE-TV-V-AI------N--IW-V-V--LL-IVL---
	Bacillus cereus group	WP_061677825	--IGVTTV--S-K-E-KF-WVD-NEQQVEMK-NL--LV-		PH-GKLLTENK-E-TV-V-AI------N--IW-V-I--LL-IVL--V
	Bacillus cihuensis	WP_028391590	A--IITS----LD-K-NFTWM--YNKED-F---L--LI-		PH-G-LLTEGKMK-FV---AIID-N-AN--IW-LFS-LIL-G-L---
	Bacillus coahuilensis	WP_059282968	-YI-AVTG--LA--E-SFD--S---TEA-AD-RV---V-		P--GILTTEGAMK--FST-DY-S-I--N--IW-FIV--IL-I-----
	Bacillus cohnii	WP_066418574	----ITVI--S-D-E-NFK-ID-R-AEATV--VL-PLV-		PN LLTE-- MN--VSL-AIIHTQ--N--IW--IT-AIL-V----L
	Bacillus cucumis	WP_101650941	---AV-SI-------EKFNWVD-HE-QVAV--KM---T-		PN LLTKNG-N--V---AFI-----N--IW-LFG-LIL-GI--S-
	Bacillus cytotoxicus	SCL88397	--IAVTT-Y---K-E-KF-WVD-NKQKAEM---L--LV-		PN -GK-E-VID--A-I-----N--IW-F-I--GL-VIA--V
	Bacillus drentensis	WP_066257307	---TV-SI--GT--HEKFNWID-HD-AQ-V--TM-S-S-		PH LLTKDG-K--F-V-AFI-----N--IW--MA-L---G-----
	Bacillus enclensis	SCB85268	--I-A-TG-TLSN-E--FN-V-QQDQEA-SK-TL---V-		PH AME--A---GVI-----N--IW-F-T--I---IL---
	Bacillus fumarioli	WP_066366570	----V-SG---A--HEKFNWID-ND-QVTVD-TM---T-		PH LLTKGG---WV---AI------N--IW-LIG-LFL-SI---L
	Bacillus funiculus	WP_129728103	---SATTA--ISK-E-KFE--D-NDQKTEMV-QL--LV-		P--G-LQTKGKLD-VLHT-AI------N--IW-L---IA--TVL--L
	Bacillus infantis	WP_129612012	--IAV-SI---AD-K-SFTWID-GDQE--LD-TM---T-		PH-GTLTT-GR-E--V---AII-----N--IW--GA-LIL-G-L---
	Bacillus kribbensis	WP_026694780	--IAV-SA--SA--ETKFSWVD-R-SDAETV--M--LV-		PHKY NMA--A---AM---E--N--IW-LFG-II---I---V
	Bacillus kwashiorkori	WP_062356214	--ILITV--NLAN-ETEFEWQT---EV-KQV--L-P-V-		PN-WEYVSGNTME--V---AIIH-QR-NS-LW-FIA--IL---V---
	Bacillus lentus	SQI56473	--IIITSA--FVD-K-DLNWID---KEK-AA-TL--LV-		PHD LIT-GKM---FN--AIID-Q-FN-MIW-LII--LL-------
	Bacillus loiseleuriae	WP_049681990	---IITS----ID-K-NFKWV--YDEEA-F---L--LI-		PH-GELMTEGKME-FVK--AIID-N-AN--VW-LFS-LIL-V----L
	Bacillus manliponensis	WP_034636616	--IGITTA--STK-E-KFEWI----QQAEQV--L--LV-		PN TNKLE-FV---AII--A--N--IW-VFV--IL-IVL---
	Bacillus marisflavi	WP_121620579	--IAV--G--VN--Q-SF--VD-T-EAAIA--TL--LV-		PH-W-L-TEDTME--V---AII-----N--IW---I--G--------
	Bacillus massiliogabonensis	WP_102274707	---IVTT---LLD-K-EFNWID-N-QELKQ-FNM---T-		PN -E--V---GII-----N--IW-L-S--I----F---
	Bacillus massilionigeriensis	WP_075982177	--I-I-TA--ISS-HSSFDWMD-NN-Q-KL--KM--LV-		PH-GELTTEGK-S--F-V-AYI-----N--IW-LAT-LLL-CV---V
	Bacillus massiliosenegalensis	WP_019154350	--IAVTS--AITD-Q-S-S-ID-N-AE-QMD-TM--VI-		PN-WQAIE-GT-E-WIQV-GII-----N--VW-LAA-LIL-IVL---
	Bacillus megaterium	WP_098449466	---AVTL----AH-ESSFNWV--S-EQEQVV--VGPHQS		EWTD GNDI---ID--AII-----N--IW-LAG-F-L-W-----
	Bacillus mesonae	WP_066385138	---IV-SI-AGT--QEKFNWID-H--QAEV--LM--LT-		PH LLTKDG-K--V---AFI-----N--IW-FAA-IIL-G-----
	Bacillus muralis	WP_057914330	---IITS--A-LD-K-NFDWID-K-AADKQ--NL--LV-		PH- LI---- -E--VS--AII-----N--LW--GA-LIL-G-----
	Bacillus niacini	WP_034677291	---LV-TL----D-K-EFNWID-NNQVAT---TM---T-		PH-GELLTKDK-E--V---AII-G--MNS-IW-FIG-FIL-G-L---
	Bacillus niameyensis	WP_062104772	--IIITTS-ALAD-ETQFSWV----KE--AT--L---V-		PH-G-KISETG-E--VS--AI---Q--N--IW-L-V---L---V---
	Bacillus notoginsengisoli	WP_118920583	--IAV-VG---A--E-KFNWVD-A-ESA-A---L---T-		PHDMIT-G KIK--V---AII-----N--IWALIG-SLL-G----V
	Bacillus novalis	WP_066089421	---AV-TI---S--HE-FNW-D-H-KVN-M--TM---T-		PH -K--V---AII-----N--IW-LMA-L-L-G----V
	Bacillus oceanisediminis	WP_110064275	---II-S-------K-EFNWID-N-QEA-L---M--LT-		PN -E--A---AII-----N--IW-LAS-IIL---L-A-
	Bacillus panaciterrae	WP_028400451	---AATTS---TK-E-KFEWV----QKAEMV-NL--LVA		PH LDSALQ--AII----MN--IWAL----G--AVL--V
	Bacillus persicus	WP_090744690	A-ILVTTA-TSVN-K-EFN-ID-K-VEA-Y---I--LI-		PN-L-T-GK -E--I-T-AIIDS---N--VW-LVI-L-L-WVL---
	Bacillus praedii	WP_057764295	---LVTT---LLD-K-EFNWID-N-QQLKQ--TM---T-		PN -E--V---GII-----N--VW-L-S--I----F---
	Bacillus proteolyticus	WP_071744878	--IAVTTV--S-K-E-KF-WID-NEQQVAMK-NL--LV-		PH-GKLLTENKLE-TV-V-AI------N--IW-VIV--LL-IVL--V
	Bacillus pseudomycoides	PEA55877	--I-ATTA--SVK-E-KF-WM--NEQQAEMV-NL--LV-		PH K-E-VI-T-AII-----N--IW-VFV--GL-V-L---
	Bacillus rubiinfantis	WP_042355227	---AV-SL--GI--HEKFNWID-H--ED-L--TM---T-		PH -E--L---AYI-----N--IW-FMG-A-L-G-L---
	Bacillus salsus	WP_090857530	--ILV--V--V-N-ES-FSWID-NEKQAEMV--L--LV-		PN-GELLTEGK-D-VI-L-AILS-R-VN--LW-F-V--IL-G-F--M
	Bacillus selenatarsenatis	WP_041965634	---FV-SF-A-TD---NFN-I--D-QPATE--TI--LV-		PHQFLTDK-- -E--F-T-AIIS----N--IW-LAA-I-L-V-L-A-
	Bacillus simplex	WP_061463915	---IITSS-A--D-Q-KFDWVD-H-AEVKQ-FNL--LV-		PN- LI-T-- -E---D----I-----N--LW-FGM-L-L-G-----
	Bacillus smithii	WP_003355203	---AVTTI-RV-D-E-AF--V----QPAEVV-NL--LI-		PH-GDLQTKNVMN--FYV-AII-----N--IW-LIA--IL-V---VV
	Bacillus testis	WP_050615595	--IIV-TV-AID--K-EFN-VD-N-EEA-AD-TL--LV-		QHDA-LKTKGAME--V-L-AIF----VN--LW-LGV---L---L---
	Bacillus thermoamylovorans	WP_041847782	--IMIKLV--I-N-Q-VFEWVD------TLK-TL---L-		PN-WLLT-G- ME-IL---AILE-Q-FN--IW-FIA---L-I-----
	Bacillus thuringiensis	WP_061666892	--IGVTTV--S-K-E-KF-WVD-NEQQLEMK-NL--LV-		PH-GKLQTENKLE-AV-V-AI------N--IW-V-V--LL-IVL--V
	Bacillus vietnamensis	WP_034760586	----A-TA-TLGH-E-NF--V-QQ-EEAIS--TL--LV-		PN LLSEDVME--V---A-I-----N--IW-FMV-------L---
	Bacillus vireti	WP_024026773	----V-TV---T--HEKFNW-D-H-KVN-M--TM--LT-		PN -K--V---AII-----N--IW-LMA-L-L-GF---V
	Bacillus wiedmannii	WP_098111386	--IAVTTV--S-K-E-KF-WID-NEQQVEMK-NL--LV-		PH-GKLLTENKLE-TV-V-AI------N--IW-V-V--LL-IVL--V
	Bacillus wudalianchiensis	WP_065409195	---AVTT--TLS--E-QFE-V--D--VAKETFTI---V-		PN -GK-E-IVD--AFI-----N-TLW-LAA-A-L-G-L--L
	Bacillus zeae	WP_119111846	---AV-SS--LAN-ESKFT-ID-N-QED----NL--VV-		PH-GKLQTAAK-E--A-T-AII-----N--LW-LVS-LAL-W-V---
	Bhargavaea beijingensis	WP_092095238	--I-I-VG-R-V--E--FD-VD-N-APAEVV----PL--		MN-YSAKTEGTME--A----II---T---FTW-VIA--L--F-----
	Bhargavaea cecembensis	WP_008297038	--I-ITVG-R-A--E-AFD-VD-NDMQAEVV--M-PL--		MN-Y TEGTMD--A----II---T---FTW-VIA--IL-F-----
	Bhargavaea ginsengi	WP_092048768	--I-VTVG-R-A--E-AFD-VD-N-AQAEVV--M--L--		MN-YSAKTEGTM---A---AII---T---FTW-VIT-IIL-F-----
	Caldibacillus debilis	OUM89072	-Y-LI-GV-QGT--K-EFEWID-N-Q-A-Q---L--LV-		PNGWQLQTEGRME-FA-L--IL----VN--LW-FIA---L-------
	Caryophanon latum	WP_066462665	----AT----S-Q-ESNFN-ID---KEAS-T-TM---W-		MNEYEQL-DT-T--V-T--VI--------TWAF-----L-V----L
	Caryophanon tenue	WP_066543577	---LA-V---VI--ESSFD-VD---RDAE-T-TM---W-		MN-Y-PQQEDT-T--A-T--VI--------TWA------L-G---V-
	Domibacillus aminovorans	WP_063966874	--IIVTTG-ALAD----FE-V---EAAD-Q--NV--LV-		PN-G-LVT-GKMN--VD--A-I-----N--LW-LGA-IIL--A----
	Domibacillus antri	WP_075397560	--ILITTG-ALSD-K-EFE-V---DAAA-Q-FNV--LV-		PN-G-LIT-GKMN-IV---AFI-----N--LW-LGA-VIL-V-----
	Domibacillus enclensis	WP_045852616	-ILITTG-A-AD-K-EFD-V----AAANL-FTV--LV-		PN-GSLLTEGKME--A---AFI-----N--LW-LIG-V-L-V----V
	Domibacillus mangrovi	WP_073710028	--IIVTTG-ALAD----FD-V---EAAA-M--NV--LV-		PN-G-LVT-GKMS-FVD--A-I-----N--LW-LGA-IIL--A----
	Edaphobacillus lindanitolerans	WP_076756433	--I-V-AG-G-S--E-KFT-VD-NDKPAEMV----PL--		MN-YEAKTEGVME-IA----II---T---FVW---A-S---------
	Falsibacillus sp. GY 10110	WP_121680710	---AATT---L-D-K-SFNWE-RK-EIE-MD-SL-PVV-		PHKADLQTNDRME--I-L-AFF----FN--LW-LAA-LI--GI----
	Filibacter sp. TB-66	WP_124070695	------TA--IA--E-KFT-ID-NEAPAK---NY-PL--		MH-YKALTEGAME-IA----I----T---FVW--GT-I-L-V-L---
	Jeotgalibacillus sp. R-1-5s-1	WP_134373681	--I----S--IA-AG-EFS-V-P-DQQVTA--AM--LVV		PN-GELLTEGA-ATSL---AI---AR-N--IW-L---AG--------
	Kurthia huakuii	WP_029498396	---AVTTY-NMT--E---T-VD---EQAS-T-AT-PLI-		MHQY ME--AAT-AWI--------IW-V-A--LL-GVL--V
	Kurthia massiliensis	WP_010286160	--IGATVY-NTTH-E-PFT-VD-D-QAAQ-T--M-P---		MHEI-D --AWI--------AW-L-I---L-I-L---
	Kurthia senegalensis	WP_010301197	--ILATL--Q-TN-Q-DF--VDQK-ERMP-T-TM--LV-		MNDSTALTENHLA-FV-A-AW--G--V-S-IW-L-Y-I-L-GIL-IL
	Kurthia sp. Dielmo	WP_020189573	---AITSY-N-T--E-S-T-VD---ESAA-T--T--LI-		MHQYDAQTTEA-E--V-V-AWI--------IW-L-A---L-GAL-IL
	Lysinibacillus acetophenoni	WP_097149030	-NI-V-TV-NVA--E-Q-S-VD-N-IEQQ-I-NV-----		MN-STTLTEGAMD--I-V-AFIHGL-----VW--IA--I--I-----
	Lysinibacillus chungkukjangi	WP_107935333	--ILATVI-KGI--E-DFT-IDTQDSEKE-T-YM--V--		MNDSTLLT-NAME-IF---AVI---------WAFII-S---I-V--V
	Lysinibacillus contaminans	WP_053584135	----I-S-----D-ESK-T-VD---A-RQ-V-NM-P---		MN-FEAVEEHGMS--V---GII--------AW-L-V--IL-WA----
	Lysinibacillus endophyticus	WP_121215588	--I-VTV--RS---E-VFN-VD-N-AQHE-I-NV--V--		MN-Y-LLT-DVME-IA----VI------V--W-FIV-SI----F---
	Lysinibacillus halotolerans	WP_122972876	--ILATVI-KSI--E-AFT-VD---AQQE-T-NM--V--		MN-Y-LLT-DVME-FV-TA-VI--Q------W-FV--SIL-I-----
	Lysinibacillus macroides	WP_053994131	---AVTS--N--D-ESS-T-ID---APHK-I-NL-P---		MH-YEAIEEHGMS-IV---A-I--------VW-FIV-SIL-V-----
	Lysinibacillus manganicus	WP_036183871	-NI-T-AV-N-V--E-Q-S-VD-N-MSQE-I-NV-----		MN-SATLTEGAMD-MI-V-AFIH-L-----VWA-IV--IL-I-----
	Lysinibacillus mangiferihumi	WP_107895154	---IV-S-----D-ESK-T-ID---MSRQ-T-NL-P---		MN-YE-VEEHGMS--V---A-I--------VW-L-I-SLL--F----
	Lysinibacillus massiliensis	WP_036177016	--IAITVG-RMA--E-QFA-VD-D-LQKE-T-NL---I-		MN-SELLTEGAME--FNV--ILHGV-----VW--IA--IL--I----
	Lysinibacillus odysseyi	WP_036154284	----ATTA-R-A--E-SFEHVTQ--K-E-V---M-----		PNDY-ALSEDRMT-FV-V-A-FD-S-M---VW-----S-L-V-----
	Lysinibacillus parviboronicapi	WP_107922935	---LVTS-----D-ES--T-VD---KDRQ-I-NM-P---		MNDYKAVEEHGMS--V---GII--------VW-L-V-SIL-------
	Lysinibacillus sinduriensis	WP_036203538	--ILATVL-KGV--E-DFT-IDTQKAEQE-T-NM--V--		MNDYTLLTENVMN-IV---AVI---------W-FII-S---IIV--V
	Lysinibacillus sp. 2017	WP_108712112	---IAT---NVV--E-KFE-VD-LDQV-TTT-NM-----		MN-F-ELTEDKMT--A-L---ID-R-----IW-L----IL-I-----
	Lysinibacillus sp. B2A1	AVK83377	---IA-S--K--D-ESK-S-VD---TERQ-T-NM-P---		MN-F -IV---S-I--------VW-L-V-SIL-------
	Lysinibacillus sp. BK089	WP_132363131	---IATCF--LL--DS--T-VD----GRQ---KM-PL--		MN-YKAIEEHGMS--V-----I--------VW-L-V-S-L-------
	Lysinibacillus sp. Marseille-P	WP_106784605	-YIAVT---RSI--E-QFT-ID-T-IEQQAI-DM-----		MN-STLLT-GVME--V-T-AII--Q-----VW-LIV--IL-I-----
	Lysinibacillus sp. SYSU K30002	WP_126657925	---AVTVI-RG---E-AFT-VD-N-AQQE-I-NM--V--		MN-SELLT-NVME--A----VI------V--W-FIA-SIL------V
	Lysinibacillus sp. YLB-03	WP_118874419	--ILATVI-KG---E-NFS-V-AQ-KELE-T-NM--V--		MNDYTLLT-GKME--V----VI---------W-FVI-S-V---V---
	Lysinibacillus sp. ZYM-1	WP_054612293	---TVTS--G--D-ESS-N-VD---T-RK-I-NL-P---		MN-Y --V---A-I--------AW-L-V-S-L-------
	Lysinibacillus sphaericus	WP_069510898	---IV-S-----D-ESK-T-ID---VSRQ-T-NL-P---		MN-YEAVEEHGMS--V---A-I--------VW-L-V-SLL-------
	Lysinibacillus tabacifolii	WP_108029605	---IV-S-----D-ESK-T-ID---MSRQ-T-NL-P---		MN-YE-VEEHGMS--V---A-I--------VW-L-I-SLL--F----

Other Bacteria (0/>200)

Lysinibacillus telephonicus	WP_126295442	---LITVV-RS---E-VFN-V----LEQK-T-NM--V--	MN-YTLLT-DVME--V----VI---------W-FIV---L-------
Lysinibacillus xylanilyticus	WP_068982998	---IATSI--G---ESN-T-VD---TERQ-T-NM-PL--	MN-YKAVEEHGMS--V---A-I--------VW-L-V-SIL-------
Lysinibacillus xyleni	WP_097072317	--I-VTVI-RS---E-VFN-VD-N-AEQE-I-NM--V--	MNDY-LLT-DVME-IA----VI------V--W-FIA-SI--------
Paenisporosarcina antarctica	WP_134210002	---A-TT--TL-D-E-SFT-VD--QKP-EMT-NA-P---	MN-YESLTPAAMT--A----I---------TWAL-----L-G-----
Paenisporosarcina indica	WP_075617645	---A-TT--KV-D-E-SFN-ID--EKADE-T-NV-P---	MN-YEALTPGTMT--V-T--I---------TW-LI--L-L-V-----
Paenisporosarcina sp. HGH0030	WP_016427447	---A-TT--TV-D-E-KFS-VD--EKEKE-T-NA-PL--	MN-YESLTPGTMA-VA--------------TW-LV--VI--G-----
Paenisporosarcina sp. K2R23-3	WP_119883097	---A--SL-Q----ESNFT-VD---APQE-T-NM--L--	MN-YEQVTTDVMS--A---A----------TWA-M--V---------
Paenisporosarcina sp. OV554	WP_108587643	---A-TT---V-D-E-KFN-VD--QKEKE-I-NT-P---	MN-FEALTPGTMT--A---SI---------TW-L-----L-V-L---
Paenisporosarcina sp. TG-14	WP_017380388	---A-TT--TL-D-E-SFT-VD--QKP-E-T-NA-P---	MN-YESLTPAAMA--A----I---------TWAL-----L-G---I-
Paenisporosarcina sp. TG20	WP_019415245	---A-TT---L-D-E-SFN-VD--QKAKS-I-NT-PL--	MN-YEALTPEAMT--V-T-SII--------TW-L----LL-G-----
Planococcus antarcticus	WP_006829835	---AV-TF---T--E-EFT-VD--DKEAA-T-TM-P---	MN-Y-ALT-EAMT--I-T-AI---G-----LW-VIV---L-W----V
Planococcus citreus	WP_121297600	----T-TF-TLTN-E-DFA-V--D-QP-E-T-NM-----	MN-FEPMT-GA-T--L----I-H-G-----LW-VVV---L-A-----
Planococcus donghaensis	WP_008430981	---IA-SF--LT--E-EFT-VD--AKDAAVT-TM-P---	MN-Y-ALT-DAMT--V---AI-H-G-----TW-------L-W----V
Planococcus faecalis	WP_078080518	---LA-SF--VT--E-EFN-VD--AKEAA-T-TM-P---	MN-YEALT-DAMT--I-T-AI-D-A-----LW-VMV---L-W----V
Planococcus halocryophilus	WP_008497815	---IA-SF--LT--E-EFT-VD--SKDA-VT-TM-P---	MNDY-ALT-DAMT--L---AI-H-G-----VW-------L-W----V
Planococcus halotolerans	WP_112222720	---IA-SY--LT--Q-DFT-V--D--E-S-T-NM-PLV-	MN-YTSLTEGAME-FF-T-AII--S-I---LW-FFV--IL-W----V
Planococcus kocurii	WP_058385016	---LA-SF--VT--E-EFN-VD--AKEAA-T-TM-P---	MN-YEALT-AAMT--V-T-AI-D-A-----LW-VMV---L-W----V
Planococcus maitriensis	WP_112231615	----T-TF-TLTN-E-DFA-V--D-QP-E-T-NM-----	MN-FEAMT-GA-T--V----I-H-G------W-VMV---L-A----V
Planococcus maritimus	WP_068462701	----T-TF-TLTN-E-DFA-V--D-QP-E-T-NM-----	MN-FEPMT-GALT--V----I-H-G------W-LVV---L-A----V
Planococcus massiliensis	WP_052651371	----A-SY--LT--ESEFA-VD---KNAE-T--M-----	MHQYEQLNEDKMT----T-AV---G-----MW-LIV--I--W-----
Planococcus plakortidis	WP_068869322	----T-TF-TLTN-E-DFA-V--D-QP-E-T-NM-----	MN-FEPMT-GA-T--V----I-H-G------W-VMV---L-G-----
Planococcus rifietoensis	WP_058380536	----T-TF-TLTN-E-DFA-V--DEQP-E-T-NM-----	MN-FESMT-GA-T--V----I-H-G------W-VMV---L-A-----
Planococcus salinarum	TAA72518	----ANSY-TLT--E-EFT-ID-DD-E-S-T-NM-PLV-	MN-YTALT-DAME--FQT--II--G-V--ILW-LI---AL-WS----
Planococcus salinus	WP_123164228	---I--TY--LTN-E-NFE-VD---QPAE-T-NM-P---	MN-Y-ALT-DAMT--A-T-AVI--G-----TW-FIV--IL-W-----
Planococcus sp. CAU13	WP_033543784	---IA-TF-TLTD-Q-DFN-ID-D--EAS-G-NM-PLV-	MN-YTSLTEDAMD-FF-T-AVI--G-V--ILW-FI---IL-W-----
Planococcus sp. PAMC 21323	WP_038704181	---IA-SF--LT--E-EFT-VD--AKDAAVT-TM-P---	MN-Y-ALT-DAMT--V---AI-H-G-----VW---I--IL-W----V
Planococcus sp. Y42	WP_077589552	---AV-SY-NLT--E-EFN-V----AEDSVV-EM--L--	MN-S-AVT -FV-V-AII-GA-----VW-L-I--LL-WFV---
Planococcus versutus	WP_049693188	---LA-SF--LT--E-EFT-VD---AEAA-T-AM-P---	MN-Y-ALTEDALT--V-T-AI-H-G-----VW---V--IL-W----V
Planomicrobium flavidum	WP_088007484	----ANSY-MLTD-E-AFD-VD---EAASVV-DM-PL--	MN-G-ALDESTME-IF-V-AFI-GS---A-AW-VIV--IL-G-V--V
Planomicrobium glaciei	WP_036801710	---I--TY-TLTD-Q-DFT-IDEKDAETK-T-NM-PV--	MN-Y-QLTEGTMT-WV-T-AV---S------W-FV---IL-WI---V
Planomicrobium okeanokoites	WP_084245454	---IA-SF--LT--QSDFS-V--D--E-T-T-NM--LV-	MN-YTSLTEGTME-FF-T-AV---S-V---LW-VFV--IL-W----V
Planomicrobium soli	WP_106531804	---AA-TY-TVTD-E-NFA-VD---LDKE-A-KM-P---	MN-YLS-GKMT --V---AI---S-----TW-VIL--I--W----V
Planomicrobium sp. MB-3u-38	WP_101802893	---IA-SY--LT--E-DFT-V--D--E-S-T-NM-PLV-	MN-YTSLTEGAME-FF-T-AII--S-I---LW-FFV--IL-W----L
Planomicrobium sp. Y74	WP_121632239	---IA-SY--LT--Q-DFT-V--D--E-S-T-NM-PLV-	MN-YTSLTEGAME-FF-T-AII--S-I---LW-FFV--IL-W----V
Psychrobacillus sp. FJAT-21963	WP_056829430	---I-TTA-TIAD-K-E-S-V-----AAEM--NM-----	MNKSEAITENAME--V-----------I-TW-VI---IL-VIL---
Psychrobacillus sp. OK032	WP_093273236	--IAATT--TLAD-KVEFN-V---EQVLTM--NV-P---	MN-SEAITENAM--------------SV-TW-VI----L-I-L---
Quasibacillus thermotolerans	WP_039237728	----ITTS-RLA--E-EFN-V----QAASETFTL---V-	PN-GELLTENR-E-IA---AFI-----N--IW-LAT-F-L-W----L
Rhodococcus qingshengii	WP_133367229	---LV-SL----D-K-EFNWID-S-SEAK---TM---T-	PH-GELLTSGK-E--V---A-I----MNS-IW-FIG-FIL-G-----
Rummeliibacillus pycnus	WP_102692548	---IV-S--NLL--K-NFT-VD-N-AQQE-V-N---L--	MHKYELKSPDKME--A---AV---------VW--IV--IL-G-L-I-
Rummeliibacillus sp. POC4	WP_119416441	---IVTS--RI---E-DFT-VD-N-AQQE-T-NT--L--	MHQYELKSSGKME--A---AI---------VW-V-V--IL-G-----
Solibacillus isronensis	WP_079525765	---AATV--N-V--E-K-E-VDTLDQVNTST-LM--V--	MN-Y-ELTEDRMT--F-L-A-ID-R------W-V----I--I-----
Solibacillus kalamii	WP_087618239	---AATV--N--D-E-K-E-VDTLDQV-TST-LM-----	MN-Y-ELSEDRMT--F-L-A-ID-R------W-A----I--I-----
Solibacillus sp. R5-41	WP_099423021	----ATS-----N-E-KFE-VDTLDKV-EVT-TM-----	MN-YKELSEDKMT-IKA--A-ID-R------W--I--SAL------V
Sporosarcina globispora	WP_053434533	--III-SS---A--K-EFNWID-N-QEA-L---M--LT-	PN-GQLTTEGK-E--A---AII-----N--IW-LAS-IIL--AL---
Sporosarcina koreensis	WP_040286758	------SA-TLS--E-KFN-VD-NNQPAE-T-NY-PL--	MHDYEQVTTDTMK-WA---AI----T---FVW-LAS-I-L-V-L---
Sporosarcina newyorkensis	WP_009497173	---IL-S--K-T--E-QFT-ID-NEMPAE-T-NF-PL--	MN-Y T-WA---AI----T--SFVW--FG---L---L---
Sporosarcina pasteurii	WP_115360642	--IIATTG-RVAD-E-QFTFV--NEQIEQVT--M-PL--	MHQYLT-G TM--WA-T-AIID------TTW-FFV---L-I-L---
Sporosarcina sp. BI001-red	WP_116016569	------TA--LS--E-DFN-VD-N-KA-EMT-NM-PL--	MS-YEQVTTDTMK-WF---AI----T---FVW-LAT-I-L---L---
Sporosarcina sp. EUR3 2.2.2	WP_024534897	---A-TT---V-D-E-KFN-VD--QKEKE-T-NT-P---	MN-FEALTQGTMT-IA---A----------TW-------L-G-----
Sporosarcina sp. HY008	WP_067407157	--I---T--KVT--E-AFT-VDQNEKPAQMV--Y-PL--	MHQY GAME-WA---AVI---T---FVW-VGT-I-----M---
Sporosarcina sp. P13	WP_099686985	----T-T--KT---E-EFT-ID-NEAPAE-T--I-PL--	MN-Y GAMK-WV-L--II--QR--SLTW-VAV-A-L---L---
Sporosarcina sp. P17b	WP_099624566	----A-T--KV-D-Q-EFT-I--DQVPAE-T--V-PL--	MN-Y -K MT-WV-L--II--Q-MSSLVW--AI-S-L-V-L---
Sporosarcina sp. P19	WP_099690422	------T--KV-D-E-EFT-I--DEVPAE-T--V-PL--	MN-Y -K MT-WV-L--II--Q--SSLVW--AV-S-L-V-L---
Sporosarcina sp. P3	WP_099638152	------T--KV-D-E-EFT-I--DEVPAE-T--V-PL--	MN-Y -K MT-WA-L--II--Q-MSSLVW--AV---L-V-L---
Sporosarcina sp. P33	WP_081244490	----T-T--KV-N-E-EFT-ID-NEASAE-T--V-PL--	MN-Y DTMT-WV----II--Q--SSLVW--AV-S-L---L---
Sporosarcina sp. P34	WP_099694263	------T--NV-N-E-EFT-ID-DQAPAE-T--I-PL--	MN-Y EKMT-WV-L--II--Q--SSLVW--AI-S-L-V-L---
Sporosarcina sp. PTS2304	WP_114923457	----T-T--KV---E-EFT-ID-NQAPAE-T--V-PL--	MN-Y NAMT-WV----II--Q---SLTW-VAV-A-L------L
Sporosarcina ureae	WP_029053071	------T--KV-D-E-EFT-I--DEVPAE-T--V-PL--	MN-Y -K MT-WV-L--II--Q--SSLVW--AV-S-L-V-L---
Streptococcus pneumoniae	CEY40058	--IGVTTV--S-K-E-KF-WID-TEQQVEMK-NL--LV-	PH-GKLLTENKLE-AV-V-AI------N--IW-V-V--LL-IVL--V
Ureibacillus thermosphaericus	WP_016836918	---LVT---RG---E-QFT-V-NN-ME-E-V-NM--L--	MN-SELKTEGVME--V---AII--------VW-F-V--IL---Y-F-
Vibrio vulnificus	WP_133347646	---IITSS-A--D-K-NFDWVD-K-VPAKQ-FNL--LV-	PH-ELT-T-- -E--VD--A-I-----N--LW-FGM-L-L-G-----
Viridibacillus arvi	WP_053417367	---AVTT--NVT--E-EFS-ID-D---KE-T-NT--L--	MHQELLT--K ME-IANV-AI---------VW---I---L-GI----
Viridibacillus sp. OK051	WP_100796616	----VTS--NV-N-E-EFT-VD-D-VQKE-T-NT--L--	MHQEA-T--K ME--ANV-AI---------VW--VV--IL-G-----

Supplemental Figure 45

A partial sequence alignment of the c-type cytochrome biogenesis protein CcsB containing a 15 amino acid insertion (boxed) that is exclusively shared by all members belonging to the Meyeri clade and absent in all other bacteria.

Group	Organism	Accession	123		165
**Meyeri Clade (3/3)**	Bacillus ndiopicus	WP_042477869	FPVVIDKTRSVQKAYNVGA	A	LPATVLVTPEGKVKKIITGEMTE
	Lysinibacillus fluoroglycofenilyticus	WP_066171039	-------------------	-	----I----D----------S-
	Lysinibacillus meyeri	WP_107841855	------------R-----V	-	---------------------S-
**Other Bacteria (0/>200)**	Anoxybacillus flavithermus	WP_088223244	------QQDQ-MN---I-P		----F-IDK---IVD----A---
	Anoxybacillus pushchinoensis	WP_091700493	------QQDQ-MN---I-P		----F-IDK---IVD----M---
	Anoxybacillus sp. B2M1	ANB58956	------REDQ-MN--GIDP		--T-F-IDKD--IT-----S---
	Anoxybacillus sp. P3H1B	KXG11115	------REDQ-MN--EIDP		--T-F-IDKD--IT-----T---
	Anoxybacillus suryakundensis	WP_055440692	------QQDQ-MN--HI-P		--T-F-INKD--IVDM---T---
	Anoxybacillus tepidamans	WP_027408659	------REDQ-MN--EIDP		--T-F-IDKN--I------T---
	Anoxybacillus vitaminiphilus	WP_111644078	--I---QQDQ-MN--DIDP		--T-F-IDKD--IV-----T---
	Bacillus altitudinis	WP_096881585	---A---D-Q-TE--DITP		--T-F-IN-----I-V-K-T---
	Bacillus alveayuensis	WP_044747971	--I---QQDQ-MN--DIDP		--T-F-INKD--IV-----T---
	Bacillus amyloliquefaciens	WP_065981792	---AL-TD-Q-LD--G-AP		I-T-F-IN-D---V-VV--T---
	Bacillus amyloliquefaciens gro	WP_032866334	---AL-TD-Q-LD--G-SP		I-T-F-IN-D---V-VV--T---
	Bacillus aquimaris	WP_064090984	--ILV--EDQ--H--GIDP		--T-L-IN---EIE-----T---
	Bacillus atrophaeus	WP_094232303	----L-SD-Q-LN--G-SP		I-T-F-IN----IV-VV--T---
	Bacillus australimaris	WP_060697213	---AM--D-Q-TE--DITP		--T-F-IN-----I-V-K-T---
	Bacillus cecembensis	WP_057982160	---A----K--FT---IDP		--TSIFIK-D-T-DR------S-
	Bacillus cereus	KLA14663	--ILK--D-A-TE--DITP		I-T-F-IDKN---L-V---S---
	Bacillus circulans	WP_047943218	--ILK--D-A-TE--DITP		I-T-F-IDKK---L-V---S---
	Bacillus clausii	WP_095236227	---A--ER-E-TR--GI-P		----I--DEH-I-Q-VH--A---
	Bacillus firmus	WP_061792440	--I----DSQ--S--GINP		----F-IDKD---V-Y-------
	Bacillus glycinifermentans	WP_096892169	----L--D-Q-LD--D-TP		--T-F-IN-D--IL-VV--T---
	Bacillus gobiensis	WP_098945211	-------D-Q-LE--D-NP		--T-F-IS-----V-VV--S---
	Bacillus halotolerans	WP_024121902	----L-TD-Q-LD--G-SP		--T-F-IN-----V-VV--T---
	Bacillus haynesii	WP_094777438	----L--D-Q-LN--D-TP		--T-F-IN-D-EIV-VVS-T---
	Bacillus horneckiae	WP_066395608	--ILL--DGQ--T--KI--		----Y-IDK----V-YH--QL--
	Bacillus infantis	WP_129612014	--I-T--DGQ--N--AIDP		----F-IN---E-V--H--QL--
	Bacillus kwashiorkori	WP_062356206	--IIV-EDGE--H--G-LP		----F-ID----IV-YH---LD-
	Bacillus licheniformis	WP_107661742	----L--D-Q-LN--D-TP		--T-F-IN-D-EIV-VV------
	Bacillus litoralis	WP_066330048	---IL--D-Q-LD--G--P		--T-F-IN---E-VH-TS-SL--
	Bacillus marisflavi	WP_048005924	--I-V--EEE--N--GIDP		--T-M-IN-D--ID-----T-S-
	Bacillus massiliogabonensis	WP_102274705	--I----DGQ--T--KI--		----FMIDKD---V-YH--QL--
	Bacillus mediterraneensis	WP_071460012	---M--EDGQ--A--GI-Q		--I-F-IDE----VRSY---L--
	Bacillus mojavensis	WP_010334780	----L-TD-Q-LD--G-SP		--T-F-IN-----V-VV--T---
	Bacillus muralis	WP_057914328	---M--QG-E-EN--R-DI		--V-F-IDK--Q-ID----AL--
	Bacillus nakamurai	WP_061520912	---AL-TD-Q-LD--G-SP		--T-F-IN----IV-VV--T---
	Bacillus notoginsengisoli	WP_118920581	--I-L--D-Q-MN--G--Q		----F--NK---IVRYH--QL--
	Bacillus oceanisediminis	WP_019380357	--I----NTQ--S--GINP		----F-IDKD---V-Y----L--
	Bacillus paralicheniformis	OCI07013	----L--D-Q-LN--D-TP		--T-F-IN-D-EIV-VVS-T---
	Bacillus patagoniensis	WP_078391229	--IA---R-ELTR--G--P		----I--DEH-Q-QRYHE-----
	Bacillus praedii	WP_057764292	--I----DGQ--T--KI--		----FMIDKD---V-YH--QL--
	Bacillus pumilus	WP_106067107	--IAM--D-Q-TE--DI-Q		--T-L--N---R-I-V-K-T---
	Bacillus rubiinfantis	WP_082193049	--ILM-RKSE-T-L--I-P		I-T-F-IDEN--IL-----S---
	Bacillus safensis	WP_111290505	---AM--D-Q-TE--DI-Q		--T-L-IN-----I-V-K-T---
	Bacillus siamensis	WP_016939135	---AL-TD-Q-LD--S-SP		I-T-F-IN-D---V-VV--T---
	Bacillus sp. UMB0899	WP_102229252	--IPL--D-Q-LN--G--P		--T-F-IN-----VD-TS-TL--
	Bacillus sp. UNC125MFCrub1.1	WP_035391621	---AM--D-Q-TE--DITP		--T-F-IN-----I-V-K-T---
	Bacillus sp. V59.32b	WP_117304997	---L---GEE-RM--DINP		--S-L-IDK----VDF----L--
	Bacillus sp. YLB-04	WP_115453916	--ILL--ESQ-MN--G--Q		----F--DKD--II-YH--QL--
	Bacillus sp. YR335	WP_111614213	--I-L--D-Q-LN--G--P		--T-F-IN-D---EF-TS-SL--
	Bacillus sp. es.034	WP_098439974	--ILV--EEE--N--GIDP		--T-F-IN-Q-EIE-----T---
	Bacillus stratosphericus	WP_039963986	---AM--D-Q-TE--DITP		--T-F-IN-----I-V-K-T---
	Bacillus stratosphericus LAMA	EMI12114	---AM--D-Q-TE--DITP		--T-F-IN-----I-V-K-T---
	Bacillus subtilis	WP_009967646	----L-TD-Q-LD--D-SP		--T-F-IN-----V-VV--T---
	Bacillus subtilis group	WP_004429691	----L-SD-Q-LN--G-SP		I-T-F-IN----IV-VV--T---
	Bacillus subtilis subsp. subti	OTQ84718	----L-TD-Q-LD--D-SP		--T-F-IN-----V-VV--T---
	Bacillus swezeyi	WP_076758749	----L--D-Q-LD--DTIQ		--T-F-INS--EIV-V---T---
	Bacillus tequilensis	WP_024715207	----L-TD-Q-LD--D-SL		----F-ID-----V-VV--T---
	Bacillus terrae	WP_120115120	---LK--SKDIMNM-G-FN		----L--N-----I-VEE--L--
	Bacillus thermocopriae	WP_129000465	------QQDQ-MN---I-P		----F-IDK---IVD----A---
	Bacillus vallismortis	WP_010328151	----L-TD-Q-LD--D-SP		--T-F-IN-----V-VV--T---
	Bacillus velezensis	ATX84348	---AL-TD-Q-LD--G-SP		I-T-F-IN-D---V-VV--T---
	Bacillus vietnamensis	WP_060672300	--ILV--EEE--N--GIDP		--T-F-IN-D-EIE-----T---
	Bacillus weihaiensis	WP_072579345	----L--QSQ-F---G--K		----Y-IDSD---VDV---TL--
	Bacillus xiamenensis	WP_008359709	---AM--D-Q-TE--DITP		--T-F-IN-----I-V-K-T---
	Bacillus zhangzhouensis	WP_034318809	---AM--D-Q-TE--DITP		--T-F-IN-----I-V-K-T---
	Bhargavaea beijingensis	WP_092095243	--I-----N--RD----RP		--T-F--G-D-------QQ--S-
	Bhargavaea cecembensis	WP_063179254	--I-----N--RD----RP		--T-F--G-D-------ET--S-
	Bhargavaea ginsengi	WP_092048773	--I-----K--RD---IRP		--T-L-IG-D-------QQ--S-
	Brevibacillus laterosporus	WP_003338476	--ILM--QKE-T----I-K		M-S-FYIDQD-I-QE--I-Q-D-
	Brevibacillus laterosporus GI-	CCF14280	--ILM--QKE-T----I-K		M-S-FYIDQD-I-QE--I-Q-D-
	Caryophanon tenue	WP_066543575	------T-GD-KQ--SIIP		--T---IN---T-T---------
	Chryseomicrobium excrementi	WP_100353130	------T-KD--DS-TI-P		--T-L-IN---E-I-V-------

Other Bacteria (0/>200)

Desmospora sp. 8437	WP_009710680	L---L-RD-E-TNL----P	I-SSFFIS----IV-H---Q-D-
Domibacillus robiginosus	WP_050182508	--IL--TG-E---V---NP	--V-FMIS-D---ED--V-GLV-
Falsibacillus pallidus	WP_114746268	----V--NKD--T--GIDP	--T-F-IN-K-EIVD--K-T---
Falsibacillus sp. GY 10110	WP_121680708	---LV--S-D--T--GIDP	--TSF-ID----IV-----TL--
Filibacter sp. TB-66	WP_124070697	--IA----E--KEV--IIP	--T-F-INK--RIEQ---R--S-
Geobacillus genomosp. 3	WP_020960397	--I---RQDQ-LN-----P	--T-F-IDKN-E-------T--K
Geobacillus icigianus	WP_033021084	--I---QEDQ-LN-----P	--T-F-IDKN----Q----T--K
Geobacillus jurassicus	WP_066230282	--I---RQDQ-LN-----P	--T-F-IDKN-E--Q----T--K
Geobacillus kaustophilus	WP_044730958	--I---RQDQ-LN-----P	--T-F-IDKN-E--Q----T--K
Geobacillus sp. 12AMOR1	AKM19509	--I---RQDQ-LN-----P	--T-F-IDKN-E-------A--K
Geobacillus sp. FJAT-46040	WP_096224964	--I---RQDQ-LN-----P	--T-F-IDKN-E-------A--K
Geobacillus stearothermophilus	WP_033017125	--I---RQDQ-LN-----P	--T-F-IDKN-E-------T--K
Geobacillus vulcani	WP_031408552	--I---RQDQ-LN-----P	--T-F-IDKN-E--Q--E-T--K
Jeotgalibacillus alimentarius	WP_041122736	----R-VNSD-MD--SI-P	--T-L--NSD-EIE-V-----S-
Jeotgalibacillus campisalis	KIL48106	----R-AQKD-MY--AI-P	--T-L-IN---E-V-VVK-----
Jeotgalibacillus malaysiensis	WP_039806421	------QKKD--N--TI-P	--T-L-IN---EII-V-------
Jeotgalibacillus salarius	WP_134380194	----R-VNKD-MD--SI-P	--T-M-IS-D-EII-V-K-----
Jeotgalibacillus soli	WP_041090143	------TQKD--N--TI-P	--T-L-IN---EIV-V-----S-
Jeotgalibacillus sp. R-1-5s-1	WP_134373647	----R-EHKD-MD--SI-P	--T-L-IS---E-TQV-----S-
Jeotgalibacillus sp. S-D1	WP_133375537	----R-AQ-D-YD--SI-T	--T-L-IS---E-VDVVK-----
Kurthia huakuii	WP_029498394	---A--H-K--VRT--IEP	--T-M-INEH-EIV---------
Kurthia massiliensis	WP_010286163	---A--DKK--MR---IDP	--T-IFIDAT-HIAN--------
Kurthia sibirica	WP_109304638	--MT----K--MRT---DQ	----F-IDQT--I---L-YGI--
Kurthia sp. 3B1D	WP_126990688	---A--TSK--LR---IDP	--T-M-IDEH--IV--------D
Kurthia sp. Dielmo	WP_020189571	---A--TSK--LR---IDP	--T-M-IDEH--IV--------D
Kurthia zopfii	WP_109348675	--MT--RSK--MR----DQ	----F-INK---ITE---HGI--
Lysinibacillus acetophenoni	WP_097149032	--T------D-MT----ID	---SIFID-N---V--K----K-
Lysinibacillus boronitolerans	WP_036079193	---L---NK--ME---INP	--T---INS---IE-------K-
Lysinibacillus chungkukjangi	WP_107935331	--A---N-KA-MT----NP	--T-I-IN---R-E-------S-
Lysinibacillus composti	WP_124765127	--------K--MT---INP	--T-M-IS-----E---------
Lysinibacillus contaminans	WP_053584137	--------KD-MTT----N	-----MID-D---A---------
Lysinibacillus endophyticus	WP_121215590	---A--E--D-MTE-TIDL	----I-IN---E-E-------N-
Lysinibacillus fusiformis	WP_069480468	--------K--MT-----Q	------ID-----V--------D
Lysinibacillus fusiformis ZB2	EKU41046	---L---NK--ME---INP	--T-I-INS---IE-------K-
Lysinibacillus halotolerans	WP_122972874	---A--P-K--MT----DL	----I-IN---E-E-------N-
Lysinibacillus jejuensis	WP_108306724	--------KG-RN---IIG	----I--S-----T--V---L--
Lysinibacillus macroides	WP_053994129	--------K--MT-----Q	------ID-D---T---------
Lysinibacillus manganicus	WP_036183876	--T------D-MT----ID	---SIFIN-D---L--K----K-
Lysinibacillus mangiferihumi	WP_107895152	--------K--MT----DN	------ID-D---V--T------
Lysinibacillus massiliensis	WP_036177020	---A----K--MT----QP	--T---IS-D-T--D------N-
Lysinibacillus odysseyi	WP_036154280	---A--Q-K--ME----TN	----I-INK---IE--V------
Lysinibacillus parviboronicapi	WP_107949731	--------K--MT-----N	-----MID-----A---------
Lysinibacillus saudimassiliens	CEA04730	--------KG-RN---IIG	----I--S-----T--V---L--
Lysinibacillus sinduriensis	WP_036203533	--A---Q-KA-MT----NP	--T-I-IN---R-E-------S-
Lysinibacillus sp. 2017	WP_108712110	---A----K--FT---I-P	--TSIFIK-D-EIER-V----S-
Lysinibacillus sp. AR18-8	WP_066036707	---L---NK--ME---INP	--T-M-INS---IE-------K-
Lysinibacillus sp. B2A1	AVK83375	--------K--MT-----Q	------ID-D---A---------
Lysinibacillus sp. BF-4	WP_036141737	--------KG-RN---IIG	----I--S-----T--V---L--
Lysinibacillus sp. BK089	WP_132363135	--------K--STT----N	----LVID-D---V------LE-
Lysinibacillus sp. FJAT-14222	WP_053594398	--------K--MT-----Q	----L-ID-D---A---------
Lysinibacillus sp. FJAT-14745	WP_053483117	--------KD-MTT----N	----L-ID-D---V--T------
Lysinibacillus sp. LK3	WP_048395832	---L---NK--ME---INP	--T-I-INS---IE-------K-
Lysinibacillus sp. Marseille-P	WP_106784602	--TA--H-K--MT---INP	--TSI-IS-D-T--D------S-
Lysinibacillus sp. PB300	WP_115673636	---L---NK--ME---INP	--T-I-INS---IE-------K-
Lysinibacillus sp. SYSU K30002	WP_126657927	---A--E-KD-MTT---TL	----I-IN-----E-------N-
Lysinibacillus sp. YLB-03	WP_118874421	---A--Q-K--MT----DL	----I-IN---N-E-------S-
Lysinibacillus sp. YS11	WP_103118176	---L---NK--ME---INP	--T-I-INS---IE-------KK
Lysinibacillus sp. ZYM-1	WP_054612291	--------K--MT-----Q	------ID-----A--------D
Lysinibacillus sphaericus	WP_012293419	--------K--MT-----Q	------ID-----V--------D
Lysinibacillus telephonicus	WP_126295444	---A--S-K--MT----DL	----I-IN-----E-------N-
Lysinibacillus xylanilyticus	WP_068983000	--------K--MT-----Q	----L-ID-D---V---------
Lysinibacillus xyleni	WP_097074687	---L---SK--MS---IKP	--T----N----IE--------D
Marininema mesophilum	WP_091734838	--I-L-QD-V-T-R-D--R	----Y-ISR---IIRKMK-Q-L-
Paenibacillus etheri	KTD86772	------V-GKIST--G-S-	----FIING---I-EA-L--I-D
Paenisporosarcina antarctica	WP_134210004	--I-----K--ME---INP	--T-L-IN-D--IEQ-LR-----
Paenisporosarcina indica	WP_075617643	--------K--MD---INP	--T-M-IN-Q--IE---------
Paenisporosarcina quisquiliaru	SEM10056	--IA--RNK--MES--IDP	--T-F-IN----IE---K-----
Paenisporosarcina sp. HGH0030	WP_016427449	--I-----K--MET--IDP	--T-L-IN----IE--VR-----
Paenisporosarcina sp. K2R23-3	WP_119883095	--I----EK--MQ---IDP	--T-F-IN----IER------S-
Paenisporosarcina sp. OV554	WP_108587640	--I-----K--ME---IDP	--T-L-IN----IEQ-VR-----
Paenisporosarcina sp. TG20	WP_019414657	--I--------MD---IRP	--T-I--N-Q--IV-------S-
Parageobacillus caldoxylosilyt	WP_017435309	--IM--REDQ-MN--D-NQ	----F-IDKN---------T---
Parageobacillus genomosp. 1	WP_043905333	--IM--REDQ-MN--D--Q	----F-IDKN---------T---
Parageobacillus thermantarctic	WP_090949283	---M--REDQ-MN--D-DR	----F-IDKN---------T---

Other Bacteria (0/>200)	Parageobacillus thermoglucosid	WP_064550074	---M--REDQ-MN--D-DQ	----F-IDKN---------T---
	Planifilum fimeticola	PRX41645	--ILL-QN-E-TQL-GI-P	I-SSIF-S-----VRKVS-Q-Q-
	Planococcus antarcticus	WP_006829833	--------K--MT---IRP	--T-I--N---NIQR--------
	Planococcus donghaensis	WP_065526033	--------K--MT---IRP	--T----N---NIQR--------
	Planococcus halocryophilus	WP_008497817	--------K--MT---IRP	--T----N---NIQR--------
	Planococcus halotolerans	WP_112222891	--I----NK--ME---IRP	--T-I--N-D-EIE--V----S-
	Planococcus maritimus	WP_068487686	--I----DK--ME---IRP	--T-L--N----IE-------S-
	Planococcus massiliensis	WP_052654461	--I-----K--MT---IRP	--T---IN---EIER-V------
	Planococcus plakortidis	WP_068870850	--I----DK--ME---IRP	--T-L--NQ---IE-------S-
	Planococcus rifietoensis	WP_058380534	--I---RDK--MT---IRP	--T-M--N-Q-EIQR-V------
	Planococcus salinarum	TAA72516	-------SK--MT---IRP	--T-I--N---EIER-V----S-
	Planococcus salinus	WP_123164230	-------SK--MS---INP	--T----N-----E---------
	Planococcus sp. CAU13	WP_033543786	---A---SK--MT---IRP	--T----N---EIQR-V------
	Planococcus sp. PAMC 21323	WP_038704183	--------K--MT---IRP	--T----N---NIQR--------
	Planococcus sp. Y42	WP_077589554	---A--ESK--ME--S-VP	--T---INS--I-TD---R----
	Planococcus versutus	WP_049693190	--I-----K--MT---IRP	--T-I--NS--EIQRV-------
	Planomicrobium flavidum	WP_088007480	--IA--EAK--ME--S-VP	--T---IS-D-T-TD---R----
	Planomicrobium glaciei	WP_115650534	--------K--MT---IIP	--T----N---EIERVV------
	Planomicrobium glaciei CHR43	ETP69670	--------K--MT---IIP	--T----N---EIERVV------
	Planomicrobium okeanokoites	WP_117312385	--IA---S---MT---IRP	--T----N---DIQR--------
	Planomicrobium soli	WP_106531806	--------K--MT---IIP	--T----N-D-EIQR--------
	Planomicrobium sp. MB-3u-38	WP_101803217	--I----DK--ME---IRP	--T-I--N-D-EIE-------S-
	Planomicrobium sp. Y74	WP_121632677	--I----NK--ME---IRP	--T-I--N-D-EIE--V----S-
	Psychrobacillus insolitus	WP_111439683	---L--NNK--MET--INP	--T-F--N----IE-------S-
	Psychrobacillus psychrodurans	WP_093493721	---L--DKK--MET-KINP	--T-L-IN-N--IE-V-----S-
	Psychrobacillus psychrotoleran	SFQ12806	--IA--RNK--MES--IDP	--T-F--N----IE---K-----
	Psychrobacillus sp. FJAT-21963	WP_056828164	---L--DNK--MET--INP	--T-F--N----IE-------S-
	Psychrobacillus sp. OK028	SDM99995	--IA--RNK--MES--IDP	--T-F-IN----IE---K-----
	Psychrobacillus sp. OK032	WP_093267677	---L--HNK--MET--INP	--T-M--N----IE-------SD
	Rummeliibacillus pycnus	WP_102692550	--ILL---K--MRV---DP	--T----DK---IAR--------
	Rummeliibacillus sp. POC4	WP_119416439	--IA----K--MR----DP	--T---IDKD--IV---------
	Rummeliibacillus sp. TYF005	WP_124217290	--IA----K--MR----DI	--T---IDKD--IV---------
	Rummeliibacillus stabekisii	WP_066791844	--I--------MR----DP	----I-INKD--IEN------K-
	Sinobaca qinghaiensis	WP_120192511	--I-L--N-N-VDS-GI-P	--S-F-IDEN-V-QD----S---
	Solibacillus isronensis	WP_079524103	---L--RY---MG---IKP	--T-I-INS---IE-------D
	Solibacillus silvestris	WP_065216983	---A----K--FT---I-P	--TSIFIK-N-TIDR--------
	Solibacillus sp. R5-41	WP_099423019	---A----K--FT---IDP	--TSIFIK-D-T-DR------S-
	Sporosarcina globispora	WP_053434531	--I----DSQ--S--GINP	----F-IDKD---V-Y-------
	Sporosarcina koreensis	WP_060209963	--TL----KT-MQT---KP	--T-F-IN----IVR------S-
	Sporosarcina newyorkensis	WP_081464526	--TL----KG-MQS--IKP	--T-L-IN----IV-------S-
	Sporosarcina pasteurii	WP_115364068	--TL---NKG-MQ---IKP	--T-L-ID----II-------S-
	Sporosarcina psychrophila	WP_067210457	--IA---DK--KEV--IVP	--T-F-INKD--IEQ---R--S-
	Sporosarcina sp. EUR3 2.2.2	WP_024534899	--I-----K--ME---IKP	--T-L-IN-D-EIEQ-VR-----
	Sporosarcina sp. HYO08	WP_067407161	--IA---DE--KE----VP	--T-FFINK--EIEQ---R--S-
	Sporosarcina sp. P16b	WP_099673968	--TL----KG-MQS--IKP	--T-L-INT---IV-------S-
	Sporosarcina sp. P2	WP_099630271	--TL----KG-MQS--IKP	--T-L-IN----IV-------S-
	Sporosarcina sp. P33	WP_081242607	--TL----K--MQT---KP	--T-F-IN----IVR------S-
	Sporosarcina ureae	ARF18834	--TL--T-K--MQT---KP	--T-L-IN-D--IV-------S-
	Tetzosporium hominis	WP_094941735	------RQKN-MEK--I-P	--T-F--D-D--IIR--Q-----
	Thermolongibacillus altinsuens	WP_132947018	-------QDQ-MN--DI-P	--T-F-IDKN--IVD----T---
	Ureibacillus thermophilus	QBK25814	--------K--MQ-----Q	----F-IS-D----R-PP--LS-
	Ureibacillus thermosphaericus	WP_096550776	--------K--MQ-----K	----Y-I--------VPP--LS-
	Viridibacillus arvi	WP_053417369	--------K--TR----NN	---SIFID-T---VRVHE--L--
	Viridibacillus sp. OK051	WP_100796614	--------K--TR----NK	---S-FID-----VRVYE----Q

Supplemental Figure 46

A partial sequence alignment of the thiol-disulfide oxidoreductase ResA protein containing a one amino acid insertion (boxed) that is exclusively shared by all members belonging to the Meyeri clade and absent in all other bacteria.

Group	Species	Accession	5		46
Meyeri Clade (3/3)	Lysinibacillus fluoroglycofenilyticus	WP_066164326	INQFLNDFFTETGC		DVVWQNDTFNIQLTIEMDKKIMNRPFY
	Lysinibacillus meyeri	WP_107840758	-----MN--S----		E--R-H-ALHV---V-----------
	Bacillus ndiopicus	WP_042475783	-----H---I----		--ER-EELLHV--S------------
Other Bacteria (0/>200)	Aeribacillus pallidus	WP_063388101	-ST--KR--QA-N-	P	IIEEK-GY-TV---V----EL------
	Amphibacillus xylanus	WP_015009891	NLHDFLVDYFTASQ	C	Q-EVI---IS----E---ERL------
	Anoxybacillus amylolyticus	WP_066326183	-Q---ERY-RANE-	D	I-ETEDGYMTV---VA---EL------
	Anoxybacillus ayderensis	WP_085788970	-QS--ER--IA-H-	P	ILERSDGHIRV---A----QL------
	Anoxybacillus flavithermus	WP_004889552	-QS--ER---A-H-	T	ILEHSDGHIRV---VD---QL------
	Anoxybacillus flavithermus WK1	ACJ33291	-QS--ER---A-H-	A	ILERSDGHIRV---VD---QL------
	Anoxybacillus gonensis	WP_035066773	-QS--ER---A-H-	P	I-ERNDGHIRV---V----QL------
	Anoxybacillus kamchatkensis	WP_019416935	-QS--ER---A-H-	P	I-ERNDGHIRV---V----QL------
	Anoxybacillus pushchinoensis	WP_091700609	-QS--ER--AA-H-	P	VLERSDGHIRV---VD---QL------
	Anoxybacillus suryakundensis	WP_055440954	-QS--ER---A-H-	H	ILERSDGHIRV---A----QL------
	Anoxybacillus tepidamans	WP_027407776	-Q---KRY-RAHE-	D	I-EDYDEYLTV--S--L--EL------
	Anoxybacillus thermarum	WP_043964041	-QS--ER--IA-H-	P	ILERS-GHIRV---A----QL------
	Bacillus acidiceler	WP_088010958	-HDYIEY--RYND-	E	I-KKTPTFLE----------L------
	Bacillus alkalitelluris	WP_088074869	-H-Y-KE--I-NDS	E	IIEETDSHLHV--S-D---AL------
	Bacillus alveayuensis	WP_044896049	-QH--KR--ISNQ-	E	I-EEGTGY-TV--------EL------
	Bacillus amyloliquefaciens	WP_071348019	VH---LR--EANH-	P	IIDHGPGFMTV---V----Q-------
	Bacillus amyloliquefaciens gro	WP_015240204	VHE--LR--EANH-	P	ITEHGPGFMTV---V----Q-------
	Bacillus andreraoultii	WP_033828619	-HSY-DQ---T-N-	D	I-ENTETYMTV---N----LL------
	Bacillus aquimaris	WP_113970482	-H---ERY-VAN--	D	L-ANGDGHMTV----DL--EL------
	Bacillus aryabhattai	WP_071270655	-H--KRY-LANN-	S	I-TETPSYLTV-----L--EL------
	Bacillus asahii	WP_119115513	-HK--VTY-EANH-	E	ILKNH-TYLTV--------EL------
	Bacillus atrophaeus	WP_106044154	VH---LR--QANS-	P	IIDQGPGHMTV---V----Q-------
	Bacillus cereus	WP_001183953	-HNY--N--EANN-	E	ILGRSPHLLDV--------LL------
	Bacillus cereus group	WP_061529601	-HNY--N--EANN-	E	ILGRSPHLLDV--------LL------
	Bacillus circulans	WP_095258951	-H---ER--VAND-	E	I-ENKPGYLTV--------EL------
	Bacillus cohnii	WP_066416491	-HNY-ERY--ANN-	E	IIENEQHHLTV--------ELI-----
	Bacillus dakarensis	WP_077213952	-H---EKY-LAN--	E	IIENKDGYMTV-----L--EL------
	Bacillus enclensis	WP_058297731	-H---ERY--AN--	E	L-ANGEGYMTV----DL--EL------
	Bacillus fastidiosus	WP_066225095	-HT--EN--ETNQ-	D	IIENNSHYLTV---V----EL------
	Bacillus firmus	WP_048008256	-HK--ERY-HAN--	E	IIDRGPGYMTV----DL--EL------
	Bacillus flexus	WP_119543436	-H---KKY--AHN-	D	LTEDNPSYLKV--------EL------
	Bacillus foraminis	WP_132008612	-YK--HNY-DSN--	E	F-ELADGYMTV---E-L--EL------
	Bacillus fordii	WP_018705945	-H-Y-EQ--MA---	T	IEENGQGFLTV---V----EL------
	Bacillus fortis	WP_120068446	-H-Y-EQ--MA---	T	IEENGQGFLTV---V----EL------
	Bacillus freudenreichii	WP_126431909	VH-Y-EQ--LA---	T	IEEYDPGYLTV---V----EL------
	Bacillus funiculus	WP_129727200	-HNY-HR---ANQ-	E	IGTVT-ALLD----V----LL------
	Bacillus ginsengihumi	WP_025726722	-H-Y-EQY--TN--	E	ILENHPGYLKV----DL--EL------
	Bacillus glycinifermentans	WP_048356322	-QR--ER---AN--	D	ITGKSPGHLTV---ADV--Q-------
	Bacillus gottheilii	WP_066443694	-HR--VKY-QANE-	D	IIEQGPGFLRV-----L--EL------
	Bacillus halmapalus	WP_078379749	-HK--ERY--ANS-	E	VMENT-SHLAV--S-----EL------
	Bacillus halosaccharovorans	WP_078432616	-HH--ES---SNS-	E	VLENKEGYITV--------LL------
	Bacillus halotolerans	WP_106293707	VH---LR--QANS-	P	IIDQGPGYMTV---V----Q-------
	Bacillus horikoshii	WP_064099900	-HR--ERY--ANS-	E	I-ENT-SHLAV----D---EL------
	Bacillus indicus	WP_029279731	-SE--ET---ANS-	D	ITEK-PGYMT----VD---EL------
	Bacillus intestinalis	WP_079287956	VHD--LH--QANS-	Q	I-DQGSGYMTV---V----Q-------
	Bacillus jeotgali	WP_079509723	-HN--ERY--ANN-	E	LIENDKGHMTV---V-L--EL------
	Bacillus korlensis	WP_066047087	-HN--VEY--ANN-	E	ILENNGSYLSV-----L--EL------
	Bacillus kwashiorkori	WP_062355920	-HNY-C---ESVE-	S	IEEKTK-YLTV---V----AL------
	Bacillus lentus	WP_066137460	-H-Y-EH--QAND-	Q	ILENGDGFLTV----D---EL------
	Bacillus litoralis	WP_121662767	-HSY-ES---ANS-	D	IIEKGQGYMTV--------EL------
	Bacillus luciferensis	WP_088071130	-HDYIEY--HNND-	E	I-TKTPTYLE----------L------
	Bacillus marinisedimentorum	WP_070120692	-HH--LK---VN--	E	IKQN-PGLLH----E----TL------
	Bacillus marisflavi	WP_121620694	-H-Y-EHY--AN--	E	LRASGEGFMEV-----L--EL------
	Bacillus massiliogabonensis	WP_102274586	-QH--ERY-HANS-	E	MIESGSGYLTV-----L--QL------
	Bacillus massilionigeriensis	WP_075982285	-H---ERY-HAN--	E	IFENGEGHMCV-----L--EL------
	Bacillus massiliosenegalensis	WP_019154483	-Q---KNY-QAN--	D	I-EETDGYMTV-----L--EL------
	Bacillus megaterium	WP_098333368	-H---KRY-LANN-	S	I-TETSSYLTV-----L--EL------
	Bacillus methanolicus	WP_004435620	-H---ERY-HAN--	E	IIENSPGHITV-----L--EL------
	Bacillus mojavensis	WP_010334915	VH---LR--QANS-	P	IIDQGPGYMTV---V----Q-------
	Bacillus nakamurai	WP_061527516	VH---LR--EANH-	P	VTEQGPGFMTV---V----Q-------
	Bacillus nealsonii	WP_016204892	-H---ER--SAND-	E	I-ENKLGYLTV--------EL------
	Bacillus niameyensis	WP_062104696	-H---KT----NN-	D	IEEDK-GYMT----VD---AL------
	Bacillus oceanisediminis	WP_110064396	-HK--ERY-HAN--	E	V-DKGPGYMTV-----L--EL------
	Bacillus oleivorans	WP_097157355	-K-Y-KN--LLNQ-	D	L-EDGPGHLT-----D---EL------
	Bacillus panaciterrae	WP_028399179	-HDY-HR---ANH-	D	ILEQTDHFLDV--------LL------
	Bacillus pseudalcaliphilus	KMK76970	LHR--T-Y-NSNE-	T	I-EETPAYLSV---P-L--L-------
	Bacillus pseudomycoides	PEA56330	-HHY-HC--EAN--	E	ILQRFPHLLDV--------LL------
	Bacillus salsus	WP_090855407	-HS--ERY--SNH-	E	ITEN-HGYMTV---V-L--QL------
	Bacillus shackletonii	WP_055739295	-H-Y-ERY-LANE-	E	ILEN--GYLTV----D---EL------
	Bacillus siamensis	WP_045926713	VH---LR--EANH-	P	ITEHGPGFMTV---V----Q-------
	Bacillus simplex	WP_061464021	-HD--ITY-KANE-	D	ILDNKPTHLTV--------EL------

Other Bacteria (0/>200)

Bacillus smithii	WP_003355322	-H-Y-ER---ANK-	P	ILENKKGFLHV---K----EL------
Bacillus soli	WP_066074123	-HS--TKY-QANE-	E	IIENTDGYLTV-----L--EL------
Bacillus solimangrovi	WP_069717056	-HH--KRY--S-N-	N	ILNQTPGMLQV--------LL------
Bacillus solisilvae	WP_087998946	-HD-IEY--INNE-	E	L-KKTPTYLE----V-----L------
Bacillus sonorensis	WP_077736495	-HR--ER---AN--	D	ITDKSDGHLTV---P-V--Q-------
Bacillus sporothermodurans	WP_066234406	-H-Y--QY-EANE-	E	ILENKDGYMTV-----L--EL------
Bacillus subterraneus	WP_125479874	-HN--ERY--ANN-	D	VIENNKGYMTV---V-L--EL------
Bacillus subtilis	WP_019714953	VHE--LR--QANS-	Q	I-DQGSGYMTV---V----Q-------
Bacillus subtilis group	WP_003325443	VH---LR--QANS-	P	IIDQGPGHMTV---V----Q-------
Bacillus swezeyi	WP_076761842	-HR--ER---AN--	T	ITDKSAGHLTV---A-V--Q-------
Bacillus tequilensis	WP_024715076	VHD--LR--QANS-	Q	IIDQSLGYMTV---V----Q-------
Bacillus terrae	WP_120114982	-H-Y-EQ--IA---	T	IEESGAGALTV--------EL------
Bacillus thuringiensis	WP_087949273	-HNY--N--EANN-	E	ILGRSPHLLDV--------LL------
Bacillus toyonensis	WP_098157818	-HNY--N--KANN-	E	ILGRSPHLLDV--------LL------
Bacillus vallismortis	WP_121642891	VHD--LR--QANS-	P	I-DQGSGYMTV---V----Q-------
Bacillus velezensis	WP_104842681	VH---LR--EANH-	P	ITEHGPGFMTV---V----Q-------
Bacillus vietnamensis	WP_034757958	-HR--ERY-VAN--	D	M-ANG-GYMTV----DL--EL------
Bacillus weihaiensis	WP_072579203	-HH--ES--KANS-	A	I-ENEPGYMTV----D---EL------
Butyricicoccus sp. 1XD8-22	RKJ52965	VHDY-KT-----N-	E	IISDEGHQLTV----DI----------
Caenibacillus caldisaponilytic	WP_077616602	-HSY-E---RAS--	D	ILDLRPRFIKVK--------L------
Caldibacillus debilis	WP_120668249	-RR--KH--AVN--	E	I-EETDQSVTV---E---RA-------
Falsibacillus pallidus	WP_114746156	-H---EQY-LANK-	E	LTGSG-GYLEV----D---EL------
Falsibacillus sp. GY 10110	WP_121678939	-H---KHY-EAN--	E	MIDQSQGHMTV--------EL------
Fictibacillus aquaticus	WP_094251852	-HR--ERY-IANE-	E	M-EKNFSYMKV-----L--VL------
Fictibacillus gelatini	WP_026676797	-HR--ER--VSNE-	E	ILEKTA-SLKV-----L--LL------
Filibacter sp. TB-66	WP_124070256	-HSY-QQ--K-NN-	Q	ILNENDHFI-V--------R-------
Geobacillus galactosidasius	WP_089097911	-H---ERY-RANE-	D	ILEADDGYIKV--S-----EL------
Geobacillus genomosp. 3	WP_020960561	-RR-VERY-AAN--	T	FLEAND-YMTV---A----EL------
Geobacillus jurassicus	WP_066230523	-RR-VERY--AN--	T	F-EAND-YLI----A----EL------
Geobacillus kaustophilus	WP_044737296	-RR-VERY-AAN--	T	F-EAND-YLIV---A----EL------
Geobacillus sp. 46C-IIa	WP_081209482	-RR-VERY-AAN--	T	FLEAND-YMT----A----EL------
Geobacillus sp. C56-T3	WP_013144748	-RR-VERY-AAN--	T	F-EASD-YLTV---A----EL------
Geobacillus sp. FJAT-46040	WP_096224913	-RR-VERY-AAN--	T	F-EASD-YLTV---A----EL------
Geobacillus sp. LEMMY01	WP_079936394	-RR-VERY-AAN--	T	F-EASD-YLTV---A----EL------
Geobacillus sp. Y4.1MC1	WP_013400392	-Q--VERY-RANE-	D	IIEADDGYIKV--SV----EL------
Geobacillus stearothermophilus	WP_033016406	-RR-VERY-AAN--	T	LLEAND-YMTV---P----EL------
Geobacillus thermoleovorans	WP_069304412	-RR-VERY-AAN--	T	F-EASD-YLTV---A----EL------
Geobacillus vulcani	WP_031408301	-RR-VERY-AAN--	T	F-EAND-YLTV---A----EL------
Geobacillus zalihae	WP_060787691	-RR-VERY-AAN--	T	F-EAND-YLTV---A----EL------
Gracilibacillus dipsosauri	WP_109984186	LH---H----IQD-	A	VREETEH-LEV---R-L-QAL------
Halobacillus litoralis	WP_128526476	-VQQ---SY--	S	V-EQSDSH-KV---N---EE-------
Halobacillus mangrovi	WP_085029549	-VQN--VSY--	S	V-QKSDSFLK----S---EE-------
Halobacillus salinus	WP_079479739	-VRE---SN--	T	ISEEGPTHMT----S---EE-------
Halobacillus sp. BBL2006	WP_035547964	-VRN--LSY--	S	ITEQS-SHLK----A---EE-------
Lentibacillus amyloliquefacien	WP_068442863	LHT--H-Y--AHH-	E	I-YS--GMITV---E---REL------
Lentibacillus persicus	WP_090080963	LHT--Y-Y--AHH-	E	I-LN--GMLTV---E---RAL------
Lysinibacillus acetophenoni	WP_097148117	VHEY-RT--H--N-	E	IESDEGYMLTV----DI----------
Lysinibacillus boronitolerans	WP_016995268	VHHY-KE--M-ND-	A	ILGEEDHYLTV---VDI--R-------
Lysinibacillus chungkukjangi	WP_107933243	VHDY-KT--K--D-	E	I-SDEGHMLSV----D---R-------
Lysinibacillus composti	WP_124761587	-H-Y-ES--R--D-	E	ILKN-PNFLSV---NDI----------
Lysinibacillus contaminans	WP_053585081	VHKY-QQ----ND-	C	ILGDDEHYITV----D---R-------
Lysinibacillus endophyticus	WP_121213764	-HDY-KT--K--N-	E	I-SDEGFQLTV----D---R-------
Lysinibacillus fusiformis	WP_069479831	VHEY-KG----NE-	P	ILGEEDHSLTV---VDI--R-------
Lysinibacillus halotolerans	WP_122970516	-HNY-KS--N----	E	VLSDEGHLLMV---VDI----------
Lysinibacillus macroides	WP_053993336	VQHY-KE--M-NN-	T	ILGEEHHYLTV---VDI--R-------
Lysinibacillus manganicus	WP_036186512	VHDY-RT--R--K-	E	IESDEGHIMTV----DI----------
Lysinibacillus mangiferihumi	WP_107897009	VHNY-RQ--L-NE-	D	L-FEDEHYLTV----A---R-------
Lysinibacillus massiliensis	WP_036178272	VHDY-KT-----N-	E	IISDEGHQLTV----DI----------
Lysinibacillus odysseyi	WP_036157060	VHDY-QT--Q--N-	P	ILTEHPYYMVV---E------------
Lysinibacillus parviboronicapi	WP_107948470	VHSY-KQ--M-ND-	N	ILGEDACYLTV----DI--R-------
Lysinibacillus sinduriensis	WP_036197945	VHDY-KV--K--N-	D	ILSDEGYMLSV---TD---R-------
Lysinibacillus sp. B2A1	AVK82409	VHSY-RE----ND-	T	ILNEDRHYLTV---VDI----------
Lysinibacillus sp. BK089	WP_132363773	VHSY-RQ--N--N-	P	ILGEDQHYLTV---VDI----------
Lysinibacillus sp. FJAT-14222	WP_053595866	VHSY-RQ----ND-	C	ILGEDHHYLTV---VDI----------
Lysinibacillus sp. FJAT-14745	WP_053485248	VHSY-RQ--I-ND-	R	ILGEDHHYLTV---VDI--R-------
Lysinibacillus sp. OL1	WP_131522480	VHHY-KE--M-NN-	A	IIGEE-HYLTV----DI--R-------
Lysinibacillus sp. SYSU K30002	WP_126660295	-HDY-RT--N--N-	Q	I-ADEGHQLTV----D------S----
Lysinibacillus sp. YLB-03	WP_118875400	-HEY--N--K--N-	D	ILEDEGFLLSV---VD---R-------
Lysinibacillus sp. YR326	WP_134022760	VHSY-RQ--N-ND-	P	ILGEDHHYLTV---VDI--R-------
Lysinibacillus sp. YS11	WP_103117350	VHHY-KE--M-ND-	A	ILGEE-HYLTV----DI--R-------
Lysinibacillus sp. ZYM-1	WP_054612762	VH-Y-KG--A-NE-	P	IIGEE-HSLTV---VDI--R-------
Lysinibacillus sphaericus	WP_075526920	-HDY-QR-------	P	ILTENDYYMIV---VD-----------
Lysinibacillus tabacifolii	WP_108030696	VHNY-RQ--L-NE-	D	M-FEDEHYLTV----A---R-------
Lysinibacillus telephonicus	WP_126292581	VHDY-YT--K--N-	D	ILLDEEHYLTV----DI----------

Other Bacteria (0/>200)

Lysinibacillus varians	WP_025218417	VHNY-RQ--L-NE- D M-FEDEHYLTV----A---R-------
Lysinibacillus xylanilyticus	WP_068985890	VHSY-RQ----ND- Y I-GEEHHYLTV---VDI----------
Lysinibacillus xyleni	WP_097073056	VHDY-RT-----N- E I-SDEGFQLTV----D----------
Mycobacteroides abscessus subs	SLL32697	-H---ER--IAND- E I-ENKPGYLTV--------EL------
Oceanobacillus bengalensis	WP_121130430	L----H-Y-SFHH- E IIENNDGVLRV---E---RAL------
Oceanobacillus profundus	WP_118889649	L-E--T-Y--AKD- E IISNHDGVIQ---NEQ--REL------
Oceanobacillus sp. Castelsardo	WP_068671898	L----Q-Y-VSHR- Q ILENNDGILK-R--E---RAL------
Oceanobacillus sp. YLB-02	WP_121520938	L----H-Y--NHH- Q ILTN-DGVLQ----E---RA-------
Paenibacillus lentimorbus	WP_128574831	VH---LR--EANH- P ITEHGPGFMTV---V----Q-------
Paenibacillus sp. 7884-2	WP_095306985	L-E--K-Y--AKD- E IISNHDGVIQ---NEQ--REL------
Paenibacillus sp. FSL R5-0490	WP_076258062	-HK--ERY-HAN-- E IIDRGPGYMTV----DL--EL------
Paenisporosarcina antarctica	WP_134209888	-HGY-QK----SN- P ILNSN-HFMTV---VD----------
Paenisporosarcina indica	WP_075619633	-HSY-HN--D-NN- H I-KNHDHYMTV----------D--Y-
Paenisporosarcina quisquiliaru	WP_090564988	VQHY-RT----NN- Q F-NDTDHYLTV----D---R-----Y-
Paenisporosarcina sp. HGH0030	WP_016428671	-HEY-VR--K-NN- Q I-KEHDHYITV----D---------Y-
Paenisporosarcina sp. OV554	WP_108586517	-HGY-EK----NN- D IIIGNDHYMTV----D----------
Paenisporosarcina sp. TG-14	WP_017378589	-HGY-QN----NN- H I-NSN-HYMTV----D----------
Paenisporosarcina sp. TG20	WP_019414801	-HSY-LK--Q-ND- H IINGNDHY-SV----D----------
Parageobacillus caldoxylosilyt	WP_061579912	-RH-VERY-RANE- A ILEANDEYMTV--------EL------
Parageobacillus genomosp. 1	WP_043905461	-RH-VERY-RANE- A ILEANDEYMTV--------EL------
Parageobacillus thermantarctic	WP_090948597	-QR--ERY-RANE- D ILETDDGYMKV--SV----EL------
Parageobacillus thermoglucosid	WP_125009488	-Q---ERY-RANE- D IIEADDGYIKV--SV----EL------
Psychrobacillus insolitus	WP_111439081	VHDY-VK--E-NE- R ILNNDGHYI-V----D---------Y-
Psychrobacillus psychrodurans	WP_093493420	VQHY-RT----NN- Q F-NDTDHYLTV----D---R-----Y-
Psychrobacillus psychrotoleran	WP_093536221	VQ-Y-RT--L-NN- Q F-NDTDHYLTV----D---R-----Y-
Psychrobacillus sp. FJAT-21963	WP_056828756	VHRY-QK----NN- Q IIQNHDYYMTV---VD---------Y-
Psychrobacillus sp. OK028	WP_093061878	VQ-Y-RT--S-NN- E I--DTDHYLTV----D---R-----Y-
Psychrobacillus sp. OK032	WP_093271588	VHDY-QK--K-NN- E I-DGNEHYMTV---VDI---------H
Rummeliibacillus pycnus	WP_102691496	VHRY-R---QQ-D- A IIEEADHYLTV--------R-------
Rummeliibacillus stabekisii	WP_066786775	V-EY-SR--E--D- S IIERHPHYITA----D---R-----Y-
Salipaludibacillus agaradhaere	WP_078577026	-HS--TH--K-NQ- D ITLLSPK--QV---K----AL------
Salipaludibacillus sp. KQ-12	WP_110607961	-H----E----YQ- P VTNISATSMVV---P----AL------
Sporolactobacillus sp. THM19-2	WP_129930312	NQYLNRF-TVSGCR L LPETNGHVLSVK--E----IL------
Sporolactobacillus sp. THM7-4	WP_130032119	HEFLV-Y-TASGCQ L LP-SEK-QIKVK--K----LL------
Sporosarcina globispora	WP_053434421	-HK--ERY-HAN-- E IIDKGPGYMTV----DL--EL------
Sporosarcina koreensis	WP_040285095	-K-YIHR--E-NR- G IIEESD-HLTV---T----R-----Y-
Sporosarcina newyorkensis	WP_009498767	-HGY-RQ--N-NN- P I-NGNEHYMTV---V-----------
Sporosarcina pasteurii	WP_115363077	-HDY-RH------- P ILTENDHYMIV---VD----------
Sporosarcina psychrophila	WP_067210785	-HSY-LQ--N-NH- Q I-NDNDHYI-V---VD---------Y-
Sporosarcina sp. BI001-red	WP_116019895	-H-YIQR--H-NN- G VLDSSY-HLTV----D---R-----Y-
Sporosarcina sp. D27	WP_025783984	-H-YIQR--H-NK- R VLDESY-HMTV----D---R-----Y-
Sporosarcina sp. EUR3 2.2.2	WP_024535963	-HGY-EK--K-NN- A I-RGNDHYLTV----D----------
Sporosarcina sp. HYO08	WP_067406881	-HDY-HQ--K-NN- P ILSRHDHFICV--------R-------
Sporosarcina sp. P1	WP_099627169	-HTY-HR----ND- E ILHGNDHYLTV----D---T-------
Sporosarcina sp. P13	WP_099687807	--EY-HH--RNSN- E ILTQHPHYM-V---------------
Sporosarcina sp. P16b	WP_099673196	-HTY-HR----NN- E ILHGNEHYLTV----D---R-------
Sporosarcina sp. P17b	WP_099625507	-HTY-HR--K-NN- E VLHGNEHYLTV----D---R-------
Sporosarcina sp. P18a	WP_099676285	-HTY-HR----ND- E ILHGNEHYLTV----D---R-------
Sporosarcina sp. P19	WP_099691447	-HTY-HR--K-ND- E ILHGNEHYLTV----D---R-------
Sporosarcina sp. P20a	WP_099677706	-HTY-HR----ND- E ILHGNEHYLTV----D---R-------
Sporosarcina sp. P26b	WP_099693462	-HTY-HR--K-NN- E VLHGNEHYLTV----D---R-------
Sporosarcina sp. P3	WP_099639773	-HTY-HK----ND- E ILHGNEHYLT-----D---R-------
Sporosarcina sp. P33	WP_081242964	-HT--HQ--K-NH- E VLHGNEHYLTV----D----------
Sporosarcina sp. P34	WP_099695766	-HTY-HR----ND- E ILHGNEHYLTV----D---R-------
Sporosarcina sp. PTS2304	WP_114924755	-HDY-YH--K-ND- E ILNSHSHYM-V--------R-------
Sporosarcina sp. ZBG7A	WP_039043623	-H-YIQR--H-NK- R VLDESY-HMTV----D---R-----Y-
Sporosarcina ureae	WP_083034031	-HTY-HK----ND- E ILHGNEHYLTV----D---R-------
Ureibacillus thermophilus	QBK26157	-HDY-RT---V--- E ITEEAP-YLTV---ADI--R-------
Ureibacillus thermosphaericus	WP_016837837	-HHY-RT--I--N- K IEDETS-YLTV---ADI--R-------
Viridibacillus arvi	WP_053416173	VHSY-R---L-ND- H I-NESDHYITV--S-D---R-------
Viridibacillus sp. OK051	WP_100797842	VHGY-RN--E-NK- Q I-NDSDHYMTV----D---RL------

Supplemental Figure 47

A partial sequence alignment of the hypothetical protein containing a one amino acid deletion (boxed) that is exclusively shared by all members belonging to the Meyeri clade and absent in all other bacteria.

Group	Species	Accession	232		273
**Meyeri Clade (3/3)**	Lysinibacillus fluoroglycofenilyticus	WP_107942795	MDMIITMEVEGITAKIDQKSTTKYEDFNN		TTITIPQEALDNA
	Bacillus ndiopicus	WP_042475499	L--V-S--M--------------------		-------------
	Lysinibacillus meyeri	WP_107840483	L----S--I--T-------T--T-D----		-------DV----
**Other Bacteria (0/>100)**	Bacillus alkalinitrilicus	WP_078429899	-EISMS-TI-DE-M-ST-RM-GVLTN---	V	GE-V----VF-T-
	Bacillus alkalitelluris	WP_088076352	INLELS--FM-E-ISVE-Q-HILMSKY-E	L	NDF---E-V-N-S
	Bacillus aquimaris	WP_032086178	VN-DFD--I--EKVQSV--M-GS-SK--E	I	ED----A-VKE--
	Bacillus camelliae	WP_101354787	-NQEME-N---QKLSLK--MKGT-S-Y-K	I	NE-KV---V----
	Bacillus cecembensis	WP_057989655	--FG-G-NM--VEMG-KN------S---A	V	P-----T---E--
	Bacillus enclensis	WP_058297106	VN-DFD--I--EKVQSV--M-GS-SK--E	I	ED----A-VKE--
	Bacillus farraginis	WP_058005636	-TQELE-AI-DQKMTMK-EMKST-TEY-K	I	KE-KV---V----
	Bacillus gottheilii	WP_080849112	-T-DME-G---D-VNVV-TVNGE-T----	I	ES--V---V--S-
	Bacillus infantis	WP_129613889	---TME-A---Q-VFLN--IEG--S-Y-E	V	KE------V--T-
	Bacillus infantis NRRL B-14911	AGX05936	---TME-A---Q-VSLN--IEG--S-Y-E	V	KE------V--T-
	Bacillus lentus	WP_066145625	IK-DT-LNMD-QKMSMR-NIKSD-A-Y-K	I	DS-EV--------
	Bacillus litoralis	WP_121663835	TI-DMEITA--E-L-MK-NMKG--S---T	I	EEVKV---VI---
	Bacillus mannanilyticus	WP_025026976	---DM--TI--E-MEMK-SLKG-IHNY-K	V	AE-VV-EDVIK--
	Bacillus massiliogabonensis	WP_102274167	---DLE-TI--Q-ISMK--MKGQ-SNY-S	V	EA--V---VI-S-
	Bacillus obstructivus	WP_071976904	-TQELE-AI-DQKMTMK-EMKST-T-Y-K	I	KE-KV---V----
	Bacillus oleronius	OOP69701	-TQELE-AI-DQKMTMK-EMKST-T-Y-K	I	KE-KV---V----
	Bacillus praedii	WP_131236678	---DLE-TI--Q-IAMK--MNGQ-SNY-S	V	NA--V---VI-S-
	Bacillus sp. B14905	EAZ84918	---SLS-K---N-I-TK-QMSI--H---A	V	------KDV----
	Bacillus sp. FJAT-25496	WP_057772287	V--EME-TA--Q-MVMK--VNGQ-S-Y-K	V	D---V---II-S-
	Bacillus sp. FJAT-27916	WP_049672136	IV-N-N-KA--QKVTLK-AID-T-S-Y-T	I	DS--V-K---EQ-
	Bacillus sp. FJAT-45037	WP_100374029	-F-EFS--T--ESISMV-NT-GTFSH--D	I	DS--V-T--IET-
	Bacillus sp. FJAT-46582	WP_100332550	VE-DM--DM--NQM-TS-TMSAL-SN--Q	V	KP--V-E-VVK--
	Bacillus sp. JCM 19041	WP_054707355	-SYDVI--E--T-INMTGNAEGT-SS--E	I	EE-E---------
	Bacillus sp. MKU004	WP_064566275	VN-DFD--I--EKVQSV--M-GS-SK--E	I	ED----A-VKE--
	Bacillus sp. NSP22.2	WP_051390392	V--D-DI-EN-E-TN-S--MEST-RN--E	L	DS-EV---VT-T-
	Bacillus sp. OG2	WP_094769588	---TME-A---Q-VSLN--IEG--S-Y-E	V	KE------V--T-
	Bacillus sp. TS-2	WP_045485200	-N-TM---A--E--T-T--MVGTFYGY-E	L	DS-E--EDVIE--
	Bacillus sp. UMB0728	WP_035403269	---TME-A---Q-VSLN--IEG--S-Y-E	V	KE------V--T-
	Bacillus sp. es.036	WP_098445538	--IDMD-KI-DESMNMK-SMEMSIDN--Q	V	D---V--NVI-S-
	Bacillus trypoxylicola	WP_061947780	-N-TM---A--E--T-T--MVGTFYGY-E	L	DS-E--EDVIE--
	Bhargavaea beijingensis	WP_092098908	-V-SMSV-DQAE-LN--ID-K-A-SNI-G	I	EK-E----II-S-
	Bhargavaea cecembensis	WP_008297506	-V-SM---EQ-E-LN--ID-N-A--NI-G	I	EK-EV---VI-S-
	Bhargavaea ginsengi	WP_092055264	-V-AM---EQ-EELN--MD-K-T-SNI-G	I	EK-E----II-S-
	Caryophanon latum	WP_066462492	--FFM------E-MEM-TN-NIT-TE--H	L	QS-D---NII-S-
	Caryophanon tenue	WP_066544696	VNLNM--DM--QPMTVETTTNMTFT-Y-H	L	D--D----VI-T-
	Domibacillus indicus	WP_052712239	-M-DM---I--EKMEMN-VMNAD-SNY-G	V	E---V-E-VVSG-
	Domibacillus iocasae	WP_069937140	-I-DT---M--E-MQ-N-VMNAD-SNY-D	V	E---V-EDIVNS-
	Edaphobacillus lindanitolerans	WP_076759099	-K-TM---A--EEMT-AMDMKSV-TNI-G	I	EN-EV--DVI-S-
	Halobacillus alkaliphilus	WP_089749398	-KLDMD--M-DMSTNMV-TFDMSIDN--G	V	E-----S-VV---
	Halobacillus halophilus	WP_014643609	-NLDMD--M-DMSTNMV-TFDMSIDN--G	V	G-----DQVV---
	Halobacillus salinus	WP_079478390	V--NM-I-QD-EKVT---SMEGS-SK--E	V	GE--V-E-VIQ--
	Halobacillus sp. KGW1	WP_062513606	-MIDMSLSM--Q-MN-S-S-K-TVSE---	V	G-VE----VING-
	Jeotgalibacillus alimentarius	WP_041122976	L--TMN--FAEQSLA---SLQSE-T-Y-A	I	DELKV---VI-S-
	Jeotgalibacillus campisalis	KIL45541	---TMI-TM--ESLE-E-Q-E-V--N--T	I	DE--V---I--S-
	Jeotgalibacillus malaysiensis	WP_039810578	L--TMN-KFAEESM-ME--LESE-T-Y-E	V	EK-EV---VI-S-
	Jeotgalibacillus proteolyticus	WP_104057232	---TMV--M--ESLTLV-NTEAN-LEY-H	M	DE------V--T-
	Jeotgalibacillus salarius	WP_134382955	L--TMN-NFAEQSM--E-NLESD-T-Y-A	I	DE--V---VI-S-
	Jeotgalibacillus sp. R-1-5s-1	WP_134374946	L--TMN-DM--E-MS-K-HLESD-TEY-T	V	DE--V---V----
	Jeotgalibacillus sp. S-D1	WP_133374882	TSL-M--D---ESLQLE-NTEST-KEY-T	I	DS-EV-D-VIE--
	Lentibacillus sp. Marseille-P4	WP_106496038	---D---KQD-SELN-V-NVESE-SNI-K	V	DA-EV-ADVKE--
	Lysinibacillus boronitolerans	WP_016994224	---TLK-Q---NSI-TR-QT-I--H---A	V	------KDV----
	Lysinibacillus chungkukjangi	WP_107934752	NNINF--NI--ESISTISN-KISFNN--G	V	EE------VI-T-
	Lysinibacillus composti	WP_124765908	-VLDF-IDM--ESMS-SNDTKVTFNN-DG	V	EA--V---VI-Q-
	Lysinibacillus contaminans	WP_053583352	T--TLA-DM--VKM------NII-DE--K	I	--------V-N--
	Lysinibacillus fusiformis	WP_069483360	---TLS-K---N-I-TK-QM-I--H---A	V	------KDV----
	Lysinibacillus halotolerans	WP_122972112	-NVTF--DI--ESMT-KTDMVS-FDN--G	V	KE------VI-K-
	Lysinibacillus macroides	WP_053995675	---T-N-Q---NSI-TR-Q--I--H---A	V	-------DV-E--
	Lysinibacillus odysseyi	WP_052124973	------L-E-DGKMT---SA-VD-S----	V	DA------VI---
	Lysinibacillus parviboronicapi	WP_107923506	-NFDFK-K---EELAMTT--VVT-SE--H	L	K--D---NII---
	Lysinibacillus sp. AR18-8	WP_066037379	---TLK-Q---NSI-TR-QT-I--H---A	V	------KDV----
	Lysinibacillus sp. B2A1	AVK84953	I--TLA-NMADVKM--N---AIT-DA--T	L	S------DV-K--
	Lysinibacillus sp. BK089	WP_132357022	-NFDLK-K--DQELSMKT--VVT-TE--H	L	K--D----II-K-
	Lysinibacillus sp. F5	WP_058844877	-NFDLK-A---DELIMNT--VVT-T---H	L	K--D---SII---
	Lysinibacillus sp. FJAT-14222	WP_053596722	VN-DLKIK--DEEFSSKT-TVVT-TE---	L	K--D----II---
	Lysinibacillus sp. FJAT-14745	WP_053484860	-NFDLK-K--DQELSMKT--VVT-T---H	L	KS-D--K-VI---
	Lysinibacillus sp. LD79	SCY84374	---SLS-K---N-I-TK-QMSI--H---A	V	------KDV----
	Lysinibacillus sp. OL1	WP_131520121	---TLK-Q---NSI-TR-QT-I--H---A	V	------KDV----
	Lysinibacillus sp. PB300	WP_115673673	---TLK-Q---NSI-TR-QT-I--H---A	V	------KDV----
	Lysinibacillus sp. SYSU K30002	WP_126658758	-V-DV-IDI--EVMSMSMNTDITFNN--G	V	KN-VV---VI-Q-
	Lysinibacillus sp. YR326	WP_134018755	INFDLK-K--DQELSMKT--VVT-T---H	L	K--D----II-K-
	Lysinibacillus sp. ZYM-1	WP_054613366	---TMS-K---NSI-TK-QT-I--H---A	V	------KDV-E--

Other Bacteria (0/>100)

Lysinibacillus sphaericus	WP_036227348	---SLS-K---N-I-TK-QM-I--H---A V ------KDV----
Lysinibacillus sphaericus C3-4	ACA41457	-NFDLK-A---DELLMNT--VVT-T---H L K--D---NIV---
Lysinibacillus telephonicus	WP_126293373	TIFD--IDID-KSM--STDAKIAFNN--E V EA------VI-Q-
Lysinibacillus xylanilyticus	WP_100545017	-NFDLK-K--DQELSMKT--VVT-T---H L K--D----II---
Oceanobacillus bengalensis	WP_121134670	I--EM--G---DEV-LS-QVS---TNI-T I QS-E----VK---
Oceanobacillus damuensis	WP_067727720	--LDMALSA--EEMS-A-QVNAV-TGI-T I DS-EV---V----
Oceanobacillus limi	WP_090868811	L--SMV-TI--EEVS-T-QVNSE-KGI-T V D--EV--DVK-S-
Oceanobacillus rekensis	WP_087973199	--LYM-IAE--EEMT-A-QINAE-TGI-T V DS-EV---V----
Oceanobacillus sp. YLB-02	WP_121525056	V--DM-LS---EELN-S-SID-E-KGI-T V EN-EV---VK-S-
Ornithinibacillus californiens	WP_047986016	---KMS-T---E-VD-V-HIESD-K-I-T V DN-E----VK-S-
Paenibacillus aceti	WP_120464901	VE-TMD--A--QSVSMEM-MDSTFSKY-E I EA-EV--------
Paenibacillus contaminans	WP_113036561	V--DME-DIQ-QKISMV-SIDGT-SNY-S V KE-VV-K---ES-
Paenibacillus pabuli	WP_076291718	VN-VMN--Q--QKISM-M-M-STFSNH-G V KE-K-------S-
Paenibacillus sp. 276b	WP_090808169	VN-VMN--Q--QKISM-M-M-STFSNH-G V KE-K-------S-
Paenibacillus sp. A59	WP_053781706	VN-VME--Q--QKISM-M-M-STFSNH-G V KE-K-------S-
Paenibacillus sp. AD87	WP_064639993	VN-VMN--Q--QKISM-M-M-STFSNH-G V KE-K-------S-
Paenibacillus sp. GM2	WP_068782501	VE-NMD--A--QQVSMEM-MDSAFSK--E I DA-EV--------
Paenibacillus sp. LK1	WP_099855292	VN-VMN--Q--QKISM-M-M-STFSNH-G V KE-K-------S-
Paenibacillus sp. MBLB1234	WP_126996113	VE-KMD--A--QQVSMEM-MDSSFSKY-E I EA-EV--------
Paenibacillus sp. OK003	WP_090998831	V--VME--Q--QKISM-M-M-SMFSNH-G V KE-K-------SS
Paenibacillus sp. OK076	WP_090894435	VN-VMD--Q--QKISM-M-M-STFSNH-G V KE-K-------S-
Paenibacillus sp. VT-16-81	WP_079350812	VN-VME--Q--QQISM-M-M-STFSNH-G V EE-K-------S-
Paenibacillus taichungensis	WP_094938722	VN-VME--Q--QKISM-M-M-STFSNH-G V EE-K-------S-
Paenisporosarcina indica	WP_075619338	-I-DM--A---EEM-LS-DLKSIFSNY-K V EP-KV---V--S-
Paenisporosarcina sp. OV554	WP_108585574	--LKMSVS---EEM-MS-DLKSTFNK--E I EP--V---V----
Paucisalibacillus globulus	WP_051359312	L--DMS-K---E-IS-V-NMKSE-TNI-A I DK-EV---V--S-
Paucisalibacillus sp. EB02	WP_042148658	L--EMS-I---E-VE-V-NVNS--SNI-T I DK-EV---VI-S-
Planococcus halotolerans	WP_112224088	-T-DFD--M--ESVN-QSDMQAE-SN--G I D---V-A-VIE--
Planococcus sp. Y42	AQQ54059	LLLDMDISA--E-ISFT-SIDVT-S---- V GP--V---II-G-
Planomicrobium okeanokoites	WP_117313451	-T-DFD--M--ESVN-QSDMQAE-SN--G I DE-IV-A-VIE--
Planomicrobium sp. MB-3u-38	WP_101802139	-T-DFD--M--ESVN-QSDMQAE-SN--G I DE--V-A-VIE--
Planomicrobium sp. Y74	WP_121634862	-T-DFD--M--ESVN-QSDMQAE-SN--G I D-----A-VIE--
Pseudogracilibacillus auburnen	WP_110395640	VTLDLE--ID-EKMTSQ-VIQST-SNH-E V EE-KV----I---
Ruegeria sp. NKC1-1	WP_114372795	-N-DLE--Q--Q-MEMA-EMQGTFS--DE I DE-EV----I---
Salimicrobium flavidum	WP_076558040	LS-D--I-E--Q-VHMT-E-NIT-S-Y-- I GEL-V-D-VMN--
Salipaludibacillus aurantiacus	WP_093054713	I--TM--TIMEE-MTTQ---NMIFS--DK I DP-E---DV----
Salipaludibacillus neizhouensi	WP_110938956	IN-VMG-DIMDQSIT---DTHMTLSE--E I DP-E----V----
Salirhabdus sp. Marseille-P466	WP_102028118	---DMK-NI--EELELA-QA-ME-TSI-K V EK-EV---VK-SS
Sediminibacillus halophilus	WP_051382224	V--D-DI-EN-E-TN-S--MEST-RN--E L DS-EV---VT-T-
Sporosarcina globispora	WP_053434023	-I-EMEITA--Q-IAMK--MNGQ-ANY-K V EA--V---V--T-
Sporosarcina newyorkensis	WP_078818287	---VL---EQ-QQV-VA--IKGDISKI-E I DE-KV-K-VI-G-
Sporosarcina pasteurii	WP_115363473	LV-DME-IN--EEMR-K-NIQS-FS-I-E V EE-K----VV---
Sporosarcina psychrophila	WP_067205128	-K-DM------EEMH----VKADISKI-E I D--K----I----
Sporosarcina sp. EUR3 2.2.2	WP_024536696	-NLEL-VT---EEM-MS-NMKSTFNK--E I EP-KV---VI-S-
Sporosarcina sp. P13	WP_099687684	I--VM-IDE--QKV----NIIGTMSKI-E I DE-NV-K-VI---
Sporosarcina sp. P3	WP_099638278	----M--DEQ-QQV-VT-NIKGTMTKI-E I DE-K--K-I----
Sporosarcina sp. P34	WP_099694776	----M--DEQ-QKI-VT-NV-GTMTKI-E I DE-K--K-II---
Sporosarcina sp. P7	WP_099635755	----M--DEQ-QKI-VT-NV-GTMTKI-E V DE-K--K-II---
Sporosarcina ureae	WP_083034192	----M--DEQ-QQV-VT-NIKGTMTKI-E I DE-K--K-I----
Terrabacteria group	WP_088052189	---DMA--QN-SALN-V-NMESE-SNI-K V DA-EV-ADVKE--
Terribacillus halophilus	WP_077305436	LE-NMNIT-D-E-GQLKMV-DNTF----E T EP-E--A--K-A-
Virgibacillus halodenitrifican	WP_060681105	---DM--QP--DAIN-V--VDST-SNI-- I DK-EV-E-VKND-
Virgibacillus senegalensis	WP_053218820	V--DLDI-EN-T-IN-S-QMEAA-SN--E L DN-Q----VT-T-

Supplemental Figure 48

A partial sequence alignment of the hypothetical protein containing a one amino acid deletion (boxed) that is exclusively shared by all members belonging to the Meyeri clade and absent in all other bacteria.

Group	Species	Accession	92		130
Meyeri Clade (3/3)	Lysinibacillus meyeri	WP_107840234	PLVLGGDHSIAIGTLAGLKK		YKNLGVIWFDAHADINTPE
	Lysinibacillus fluoroglycofenilyticus	WP_107943830	------------------E-		-------------------
	Bacillus ndiopicus	WP_042477767	------------------E-		-------------------
Other Bacteria (0/>200)	Acetoanaerobium noterae	WP_079589154	--------------IS-VL-	Y	K-S---------G------
	Acetonema longum	WP_004096395	-----------V-SI--AA-	H	-SRM---------------
	Anoxybacillus amylolyticus	ANB62381	-----------------VA-	H	-E------Y---G-L--A-
	Anoxybacillus flavithermus	WP_004577755	-----------------VA-	H	--------Y---G-L--E-
	Anoxybacillus flavithermus NBR	GAC92406	-----------------VA-	H	--------Y---G-L--E-
	Anoxybacillus gonensis	KGP59506	-----------------VA-	H	--------Y---G-L--E-
	Anoxybacillus pushchinoensis	WP_091704008	-----------------VA-	H	--------Y---G-L--E-
	Anoxybacillus tepidamans	WP_027410618	-----------------VA-	H	--------Y---G-L--A-
	Anoxybacillus thermarum	WP_084221231	-----------------VAQ	H	--------Y---G-L--E-
	Anoxybacillus vitaminiphilus	WP_111646257	-----------------VA-	H	--------Y---G-L--A-
	Bacillus acidicola	WP_066268051	--I------V---S---IA-	N	--K-----Y---G-L----
	Bacillus aerophilus	WP_041508132	--I--------------VA-	H	--S-----Y---G-L--E-
	Bacillus alkalitelluris	WP_088073267	-----------------VAN	K	-E----------G-L--GD
	Bacillus altitudinis	WP_050826852	--I--------------VA-	H	-E------Y---G-L--E-
	Bacillus alveayuensis	WP_044893645	-----------------VA-	H	--------Y---G-L--A-
	Bacillus andreraoultii	WP_033827222	---F-------------VS-	Y	-----L------G------
	Bacillus anthracis	WP_033648642	-----------------VA-	H	--------Y---G-L--E-
	Bacillus aryabhattai	WP_048022388	-----------------VSR	H	--------Y---G-L--AD
	Bacillus aryabhattai B8W22	SDE61333	-----------------VSR	H	--------Y---G-L--AD
	Bacillus asahii	WP_119118784	----------G------VA-	H	-R----------G-L--E-
	Bacillus australimaris	WP_060698038	--I--------------VA-	H	-E------Y---G-L--E-
	Bacillus bataviensis	WP_007084063	-----------------VS-	H	--------Y---G-L--A-
	Bacillus boroniphilus JCM 2173	GAE47737	-------------S-T-VS-	H	--------Y-----L--AD
	Bacillus butanolivorans	WP_098176661	-----------------VS-	H	-E-V----Y---G-L--E-
	Bacillus camelliae	WP_101356543	-----------------IS-	H	--------Y---G-L-I--
	Bacillus campisalis	WP_046525576	-----------------VS-	H	--------Y---G-L--A-
	Bacillus caseinilyticus	WP_090890064	-----------------VA-	K	-E------Y---G-L--G-
	Bacillus cecembensis	WP_057986203	------------------S-	H	-Q-M----------L----
	Bacillus cellulasensis	WP_017366476	--I--------------VA-	H	-E------Y---G-L--E-
	Bacillus cereus	WP_000711578	-----------------VA-	H	--------Y---G-L--E-
	Bacillus cereus BAG3X2-1	EJQ05277	-----------------VA-	H	--------Y---G-L--E-
	Bacillus cereus group	WP_070140545	-----------------VA-	H	--------Y---G-L--E-
	Bacillus coagulans	KYC65147	--IF---------I--IS-	H	-R------------M----
	Bacillus dakarensis	WP_077210512	-----------------VS-	H	--------Y---G-L--A-
	Bacillus dielmoensis	WP_042454366	-----------------VA-	H	--------Y---G-L--A-
	Bacillus drentensis	WP_066249480	-----------------VS-	H	--------Y---G-L--A-
	Bacillus fastidiosus	WP_066226443	-----------------VS-	H	--------Y---G-L--A-
	Bacillus fumarioli	WP_066370819	------------------A-	H	-E------Y---G-L--A-
	Bacillus gaemokensis	WP_033679194	-----------------VA-	H	--------Y---G-L--E-
	Bacillus ginsengihumi	WP_025731613	--T------M----I--IS-	H	FE------------L----
	Bacillus glycinifermentans	WP_048356505	------------------AR	H	-Q------Y-----V--K-
	Bacillus gottheilii	WP_080849002	-----------------VS-	H	-Q------Y---G-L--A-
	Bacillus haynesii	WP_043925801	------------------V-	H	-Q------Y-----L--K-
	Bacillus hisashii	WP_095142479	-----------------IS-	Y	F-R---------G-L----
	Bacillus horneckiae	WP_066399852	-----------------VA-	H	--------Y---G-L--A-
	Bacillus jeotgali	WP_079504308	-----------------VS-	H	--------Y---G-L--A-
	Bacillus korlensis	WP_066059168	-----------------VS-	H	-N------Y---G-L--A-
	Bacillus licheniformis	OLF93390	------------------A-	H	-Q------Y-----L--K-
	Bacillus litoralis	OAS86880	--IF-------------VS-	H	--------Y---G-L--S-
	Bacillus massilionigeriensis	WP_075980360	-----------------VS-	H	F-------Y---G-L--E-
	Bacillus massiliosenegalensis	WP_019156650	-----------------VSR	H	-Q------Y---G-L--A-
	Bacillus megaterium	WP_063670415	-----------------VSR	H	--------Y---G-L--AD
	Bacillus megaterium DSM 319	ADF37116	-----------------VSR	H	--------Y---G-L--AD
	Bacillus mesonae	WP_127484474	-----------------VA-	H	--------Y---G-L--S-
	Bacillus methanolicus	WP_003348551	-----------------VA-	H	--------Y---G-L--A-
	Bacillus mycoides	WP_131232306	-----------------VA-	H	--------Y---G-L--E-
	Bacillus mycoides DSM 2048	EEM01346	-----------------VA-	H	--------Y---G-L--E-
	Bacillus nealsonii	WP_016201460	-----------------VS-	H	--------Y---G-L--A-
	Bacillus niacini	WP_045522809	-----------------VS-	H	--------Y---G-L--A-
	Bacillus novalis	WP_066094812	-----------------VA-	H	--------Y---G-L--A-
	Bacillus oceanisediminis	WP_110067201	-----------------VS-	H	--------Y---G-L--A-
	Bacillus okhensis	WP_034631122	----------S--SI--IA-	H	--------Y-----L--A-
	Bacillus onubensis	WP_099354658	-----------------VA-	H	--------Y---G-L--AD
	Bacillus paralicheniformis	WP_105979390	------------------M-	H	-Q------Y-----L--K-
	Bacillus polygoni	WP_088036216	-----------------IAN	K	-E------Y---G-L--G-
	Bacillus pseudofirmus	WP_075681240	-----------------VT-	E	R-------Y---G-L--S-
	Bacillus pseudomycoides	PEA55642	-----------------IA-	H	--------Y---G-L--E-
	Bacillus psychrosaccharolyticu	WP_040376773	-----------------AS-	H	-E----------G-L--D-
	Bacillus pumilus	WP_088003683	--I--------------VS-	H	-E------Y---G-L--E-
	Bacillus rubiinfantis	WP_082193454	-----------------VA-	H	--------Y---G-L--A-
	Bacillus safensis	WP_111291580	--I--------------VS-	H	-E------Y---G-L--E-

Other Bacteria (0/>200)

Bacillus safensis FO-36b	KDE25652	--I--------------VA-	H	-E------Y---G-L--E-
Bacillus subterraneus	WP_044396614	-----------------VS-	H	--------Y---G-L--A-
Bacillus subtilis group	WP_020449919	------------------V-	H	-Q------Y-----L--K-
Bacillus swezeyi	WP_076757819	------------------A-	H	-Q---I--Y---S-L--K-
Bacillus thermoamylovorans	WP_041902783	-----------------IS-	Y	F-R---------G-L----
Bacillus thuringiensis	WP_079244966	-----------------VA-	H	--------Y---G-L--E-
Bacillus tuaregi	WP_071396679	-----------------VA-	H	------------G-L--A-
Bacillus velezensis	WP_133309035	--I--------------VA-	H	-E------Y---G-L--E-
Bacillus vireti	WP_024030704	-----------------VA-	H	--------Y---G-L--A-
Bacillus wiedmannii	WP_098632572	-----------------VA-	H	--------Y---G-L--E-
Bacillus xiamenensis	WP_008360591	--I--------------VA-	H	-E------Y---G-L--E-
Bacillus zhangzhouensis	WP_034324142	--I--------------VA-	H	-E------Y---G-L--E-
Butyricicoccus sp. 1XD8-22	RKI98627	------------------G-	Y	--------Y-----L---D
Chryseomicrobium excrementi	WP_100354439	------------------A-	N	-E------Y-----M--E-
Clostridiales bacterium 38-18	OJV66118	--------------I--VLQ	N	K-----------G----E-
Cohnella lupini	WP_115995767	---------V---S----TA	H	-R----------S-M--E-
Dendrosporobacter quercicolus	SDN32438	------------------IG	H	-D---I--Y---------A
Domibacillus iocasae	WP_069936948	-----------L-----VA-	K	H-------Y---G-L---D
Fictibacillus aquaticus	WP_094253990	-----------------VA-	N	--------Y---G-L--AD
Fictibacillus arsenicus	WP_077365739	-----------------VS-	H	--------Y---G-L--AD
Fictibacillus gelatini	WP_026678231	--I--------------VA-	H	-E------Y---G-L--A-
Fictibacillus phosphorivorans	WP_066239292	-----------------VS-	N	--------Y---G-L--AD
Filibacter sp. TB-66	WP_124069190	---------------S--AV	K	--------Y-----L--E-
Geobacillus sp. 44B	WP_081162065	-----------------VA-	H	--------Y---G-L--A-
Halobacillus massiliensis	WP_082235728	-----------M-----IS-	H	F-------Y---G-L--G-
Halobacillus sp. Marseille-P38	WP_101846923	-----------M-----VS-	H	-E------Y---G-L--E-
Kurthia huakuii	WP_029500564	-I----------------AE	N	-E------Y-----M--E-
Kurthia sibirica	WP_109307367	--I------------S--AQ	H	--------Y-----M--AD
Kurthia sp. 3B1D	WP_126991514	-V----------------AE	N	-E------Y-----M--E-
Kurthia zopfii	WP_109349747	--I---------------AH	H	--------Y-----M--A-
Lucifera butyrica	WP_122627948	------------------AR	H	-H------Y-----M----
Lysinibacillus acetophenoni	WP_097150217	---------------S--A-	H	--------Y-----L-S--
Lysinibacillus boronitolerans	WP_036075010	---F--------------GE	H	--------Y-----L----
Lysinibacillus chungkukjangi	WP_107937204	------------------AA	H	--------Y-----L---A
Lysinibacillus composti	WP_124765332	-----------------VS-	H	--------Y---G-L--A-
Lysinibacillus contaminans	WP_053584992	---F----------F---GE	H	--------------L---D
Lysinibacillus endophyticus	WP_121215622	--I------------T--GT	K	--------Y-----L----
Lysinibacillus fusiformis	WP_069483030	---F--------------GE	H	--------Y-----L----
Lysinibacillus halotolerans	WP_122973674	------------------AT	K	--------Y----------
Lysinibacillus macroides	WP_053993058	---F--------------SE	H	-N------------L---D
Lysinibacillus manganicus	WP_036187613	---------------S--A-	R	--------Y-----L----
Lysinibacillus mangiferihumi	WP_107897641	---F--------------GE	H	FQ------------L----
Lysinibacillus massiliensis	WP_036178395	------------------G-	Y	--------Y-----L---D
Lysinibacillus parviboronicapi	WP_054768622	---F--------------GE	H	--------------L---D
Lysinibacillus sinduriensis	WP_036197178	------------------AE	H	-D------Y-----L---A
Lysinibacillus sp. 2017	WP_108714248	------------------ST	R	-E-M----------L---D
Lysinibacillus sp. B2A1	AVK86647	---F--------------GE	H	--------Y-----L----
Lysinibacillus sp. BK089	WP_132361147	------------------GE	H	--------------L----
Lysinibacillus sp. FJAT-14222	WP_053595561	---F--------------GE	H	--------------L-I--
Lysinibacillus sp. FJAT-14745	WP_053481736	------------------GE	H	--------------L----
Lysinibacillus sp. Marseille-P	WP_106781767	------------------A-	H	-N------Y-----L----
Lysinibacillus sp. OL1	WP_131521039	---F--------------GE	H	--------Y-----L----
Lysinibacillus sp. PB300	WP_115673091	---F--------------GE	H	--------Y-----L----
Lysinibacillus sp. SYSU K30002	WP_126658662	------------------GT	K	--------Y-----M----
Lysinibacillus sp. YLB-03	WP_118876907	--I---------------T-	K	-T------Y-----L---D
Lysinibacillus sp. YR326	WP_134025292	------------------GE	H	F-------------L----
Lysinibacillus sp. ZYM-1	WP_054613281	---F-----------S--GE	H	--------Y-----L----
Lysinibacillus sphaericus	WP_012296037	---F--------------GE	H	--------Y-----L----
Lysinibacillus tabacifolii	WP_108030731	---F--------------GE	H	FQ------------L----
Lysinibacillus telephonicus	WP_126295719	--I-------S---F---AT	R	--------Y----------
Lysinibacillus xylanilyticus	WP_100542807	------------------GE	H	--------------L----
Lysinibacillus xyleni	WP_097075104	--I------------T--GT	K	--------Y-----L----
Melghiribacillus thermohalophi	WP_132373120	-----------------VA-	H	-Q------Y-----L--G-
Mycobacteroides abscessus subs	SLL35538	-----------------VT-	H	--------Y---G-L--A-
Paenibacillus lautus	WP_096773171	------------------TR	H	-R------I---S-L---D
Paenibacillus sp. 18JY21-1	WP_132417848	--I--------M-SI---T-	H	------------T----E-
Paenibacillus sp. HGF5	WP_036672411	------------------TR	H	-R------I---S-L---D
Paenibacillus sp. cl141a	WP_090912875	------------------TR	H	-R------I---S-L---D
Paenibacillus swuensis	WP_068603936	---------------G--VQ	K	--------------L--E-
Paenisporosarcina antarctica	WP_134208202	--I---------------GD	R	-E------Y-----L--G-
Paenisporosarcina indica	WP_075619488	------------------GD	R	-E------Y-----L--G-
Paenisporosarcina sp. K2R23-3	WP_119884282	---------------S--AD	R	-Q------Y-----L--AD
Paenisporosarcina sp. OV554	PUB08422	--I---------------GD	R	-E------Y---S----G-
Paenisporosarcina sp. TG-14	WP_017379025	--I---------------GD	R	-E------Y-----L--G-

Other Bacteria (0/>200)

```
Paenisporosarcina sp. TG20          WP_019414261  -----------------GD R -E------Y-----L--GD
Parageobacillus caldoxylosilyt      WP_042409155  ----------------VA- H --------Y---G-L--A-
Parageobacillus genomosp. 1         WP_043903349  ----------------VA- H --------Y---G-L--A-
Parageobacillus toebii              WP_062678981  ----------------IA- H --------Y---G-L----
Paucisalibacillus globulus          WP_026908807  --I---------S---IS- H --------Y---G-L--S-
Pelosinus fermentans                WP_007955003  ----------M-----IA- H ---I----Y-----M----
Pelosinus propionicus               WP_090932779  ----------M-----IA- H -N-V----Y-----M----
Pelosinus sp. UFO1                  WP_038674654  ----------M-----IA- H ---I----Y-----L----
Planococcus citreus                 WP_121300648  ----------------ISE H -E------Y-----M--S-
Planococcus maitriensis             WP_112232687  ----------------ISE H -E------Y-----M--S-
Planococcus maritimus               WP_068487968  ----------------ISE H -E------Y-----M--S-
Planococcus plakortidis             WP_068871402  ----------------ISE H -E------Y-----M--S-
Planococcus rifietoensis            WP_058382128  ----------------ISE H -E------Y-----M--S-
Planococcus sp. MB-3u-03            WP_101189214  ----------------ISE Y -E------Y-----M--S-
Propionispora sp. 2/2-37            CUH97281      ----------M--I--IAR H -Q------Y-----M---Q
Propionispora vibrioides            WP_091750871  -----------------A- H --------Y-----M----
Pseudomonas sp. GW456-E7            PNB54009      ----------------TA- H -D------Y---G-L--L-
Psychrobacillus insolitus           WP_111441080  -----------------AD R -E------Y-----L--E-
Psychrobacillus psychrotoleran      WP_093537353  -I---------------AD R -E------Y-----L--A-
Psychrobacillus sp. OK032           WP_093272778  -----------------AD R -E------Y-----L--A-
Rhizophagus irregularis             PKC51413      -----------------G- Y --------Y-----L---D
Rhodococcus qingshengii             WP_133372086  ----------------VS- H --------Y---G-L--A-
Rummeliibacillus pycnus             WP_102693581  ----------------ISD H -E------Y-----M--S-
Salimicrobium halophilum            WP_093194882  ----------M-----IS- H -E------Y-----L-SG-
Salirhabdus sp. Marseille-P466      WP_102029514  -----------------A- T Q-------Y---G-L--S-
Scopulibacillus darangshiensis      WP_132746299  ----------------VT- P --------Y---G-L--A-
Solibacillus isronensis             WP_079523425  -----------------SM K ---Q-I--------L----
Solibacillus sp. R5-41              WP_099425560  -----------------S- H -Q-M----------L----
Sporolituus thermophilus            WP_093688192  --I-------------ISR H -A------Y---G-L----
Sporosarcina globispora             WP_053433155  ----------------VS- H --------Y---G-L--A-
Sporosarcina koreensis              WP_060205520  -------------I---TS K ---I----Y---V-M--A-
Sporosarcina newyorkensis           WP_009498956  -----------------AD H --------Y-----M--R-
Sporosarcina pasteurii              WP_115363579  -----------------ST K ---M----Y-----M--D-
Sporosarcina psychrophila           WP_067204869  -----------------SE K --------Y-----F--A-
Sporosarcina sp. BI001-red          WP_116018286  ---F----M--------SE K -N------Y--------EK
Sporosarcina sp. D27                WP_025783821  ---F----M--------SE K -N------Y--------E-
Sporosarcina sp. EUR3 2.2.2         WP_024536544  --I--------------GD R -E------Y-----L--G-
Sporosarcina sp. HY008              WP_067407759  -------------I---TD H -E------Y--------A-
Sporosarcina sp. P1                 WP_099626737  -----------------AE H --------Y-----M--C-
Sporosarcina sp. P13                WP_099687754  -----------------AD H --------Y-----M--S-
Sporosarcina sp. P16b               WP_099672977  -----------------AE H --------Y-----M--S-
Sporosarcina sp. P18a               WP_099675736  -----------------GE H --------Y-----M--S-
Sporosarcina sp. P19                WP_099691345  -----------------AE H -T------Y-----M--S-
Sporosarcina sp. P20a               WP_099677984  -----------------SE H --------Y-----M--S-
Sporosarcina sp. P3                 WP_099638288  -----------------AE H --------Y-----M--S-
Sporosarcina sp. P34                WP_099694786  -----------------AE H --------Y-----M--S-
Sporosarcina sp. PTS2304            WP_114924964  --I--------------AD H -S------Y-----M--S-
Sporosarcina sp. ZBG7A              WP_039043284  ---F----M--------SE K -N------Y--------EK
Sporosarcina ureae                  WP_083034209  -----------------AE H --------Y-----M--S-
Staphylococcus cohnii               RIM27984      --I---------SIS-VS- H -E------Y---G-L-I--
Staphylococcus sciuri               RIO55833      ------------SIS-VS- H -E------Y---G-L-V--
Streptococcus pneumoniae            CEY38631      ----------------VA- H --------Y---G-L--E-
Tetzosporium hominis                WP_094943895  -----------------A- N -E------Y-----M--E-
Thermosinus carboxydivorans No      EAX46682      --I-------------ISR H -A------Y---G-L----
Tuberibacillus calidus              WP_027725289  ----------------VS- H --S-----Y---G-L--A-
Tumebacillus permanentifrigori      WP_109685020  -VI---------SI--ITQ K --K-----------F--ED
Ureibacillus thermophilus           QBK25019      -VI------S--SI--IR- H -Q----------T------
Ureibacillus thermosphaericus       WP_016839642  -VI------S--SI--IR- H -N----------T----S-
```

Supplemental Figure 49

A partial sequence alignment of the arginase protein containing a one amino acid deletion (boxed) that is exclusively shared by all members belonging to the Meyeri clade and absent in all other bacteria.

			291		339
Meyeri Clade (3/3)	Lysinibacillus fluoroglycofenilyticus	WP_066168906	FFGENSVTRFITSMFDYKQTTGLI	GLT	LYIALIIAFTYFYAFVQVNPEN
	Lysinibacillus meyeri	WP_107839040	---K----I--E-------M----	---	---V------------------
	Bacillus ndiopicus	WP_042478658	--------T--KN-----------	--S	V---------------------
Other Bacteria (0/>300)	Acinetobacter baumannii	RXD25530	-LP-SG--KW--TI---T-PI-F-		--VV--V-----------S--K
	Aeribacillus pallidus	WP_063387178	---P-D---W-QKT---T-PI-M-		I-V------A---T-------Q
	Anoxybacillus amylolyticus	WP_066322038	---S-D--NW-KKT---T-PV-MM		I-V------------------Q
	Anoxybacillus ayderensis	WP_042535887	---S-D--LW-KKV---T-PV-ML		I-V---V--------------Q
	Anoxybacillus flavithermus	WP_012573862	---S-D--LW-KKV---T-PV-M-		I-V---V--------------Q
	Anoxybacillus flavithermus NBR	GAC91819	---S-D--LW-KKV---T-PV-M-		I-V---V--------------Q
	Anoxybacillus pushchinoensis	WP_091701793	---S-D--LW-KKV---T-PV-ML		I-V---V--------------Q
	Anoxybacillus sp. 103	WP_077429976	---S-D--LW-KKV---T-PV-M-		I-V---V--------------Q
	Anoxybacillus sp. BC01	KHF28893	---S-D--LW-KKV---T-PV-ML		I-V---V--------------Q
	Anoxybacillus suryakundensis	WP_055442041	---S-D--LW-KKV---T-PV-M-		I-V---V--------------Q
	Anoxybacillus tepidamans	WP_027410253	---S-D--LW-KKT---T-PV-M-		I-V---V--------------Q
	Anoxybacillus vitaminiphilus	WP_111646134	---S-D--LW-RKT---T-PV-M-		I-V------------------Q
	Bacillus acanthi	WP_108671998	---P-D---W--NT---TKPV-M-		------V--S-----I-----Q
	Bacillus aciditolerans	WP_121448790	---P-A--EW-QKY---T-PV-M-		I------------T-------Q
	Bacillus alkalitelluris	WP_078543127	---Q-D--MW-QQT---TKPV-MV		--V---V------T-------Q
	Bacillus altitudinis	WP_039169523	---T-N--TW--NN---TKPI-MT		I-V---V--------------Q
	Bacillus alveayuensis	WP_044895211	---S-D--LW-RKT---T-PV-M-		I-V------------I-----Q
	Bacillus boroniphilus	WP_035209556	---T-D--LW-QKT---T-PI-M-		V-V------------I-----Q
	Bacillus butanolivorans	WP_098174989	---K----SW--EHLV-TNPI-M-		I-V------A-----I-----Q
	Bacillus camelliae	WP_101356133	---S-N--DT-QWV---TKPL-A-		I-V------------------Q
	Bacillus cavernae	WP_126865958	---P-DI--W-DKT---THPI-MV		V-V------A-----I-----Q
	Bacillus cecembensis	WP_057986262	---D-K--A---NT---SKPV-M-		I-VG--V--------I------
	Bacillus coahuilensis	WP_010169914	-V-S----DT--YI---T-PV-ML		I-V------------I-----Q
	Bacillus cohnii	WP_066414090	---T--T-EW-QTV---T-PI-MV		I-V----------T---S---Q
	Bacillus dakarensis	WP_077210493	--EQ-D--LW-QRV---T-PI-MV		I-S------------I-----Q
	Bacillus dielmoensis	WP_042453992	--PT-D--LW--KV---THPI-MT		--A------------I-----Q
	Bacillus drentensis	WP_066249431	--PT-D--LW--KI---SHPI-MT		--A------------I-----Q
	Bacillus endophyticus	WP_111924232	---S-D--EW-QKT---T-PI-MS		V-V---V------T-------Q
	Bacillus fastidiosus	WP_066227027	---P-D--DW-SKNL--T-PI-M-		I-V----------T-------Q
	Bacillus firmus	WP_048011514	--EQ-D--LW-QRI---TSPI-MV		I-S------------I-----Q
	Bacillus foraminis	WP_121610288	---T-D--AW-QNT---T-PI-M-		I-V------A-----I-----K
	Bacillus fordii	WP_018708750	---SSELAST-Q-F--HTKPV-M-		I-V------------------Q
	Bacillus fortis	WP_120072868	---SSELAST-Q-F--HTKPV-M-		I-V------------------Q
	Bacillus freudenreichii	WP_126434586	---SSE-AST-Q-F--HTKPV-M-		IFV------------------Q
	Bacillus glycinifermentans	WP_048355851	---S-D--NW-QKT---THPV-MC		V-A------------------Q
	Bacillus gobiensis	WP_053605192	---S-A--DW-QNT-N-S-PV-M-		I-V------------------Q
	Bacillus gottheilii	WP_080849032	--ES----NW-INV---T-PI-MV		I-S------------I-----Q
	Bacillus halmapalus	WP_078382540	---P-N--EW-QRT---TNPI-MV		V-V----------T---S---Q
	Bacillus halotolerans	WP_059352665	---T-D--KW-QAN--NTHPV-MT		I-V------------------Q
	Bacillus haynesii	WP_043925513	---T-D--NW-QKT---THPV-MG		V-A------------------Q
	Bacillus hisashii	WP_095141920	---D-K---W--EW-N-SHPF-MS		I-VI-----------I-----Q
	Bacillus horikoshii	WP_088016685	--N--A---W-I-S---TNPI-MV		V-V----------T---S---Q
	Bacillus humi	WP_058000142	---P-A--E---KY---T-PV-M-		I--------S---T-------Q
	Bacillus indicus	WP_029282792	---S-D--NW--KT---T-PI-MV		V-V------------------Q
	Bacillus jeotgali	WP_102264866	---T-D--LW-QKT---T-PI-M-		V-V------------I-----Q
	Bacillus kochii	WP_095371205	--ES----SW-QKI---TEPI-MV		I-A------------I-----K
	Bacillus koreensis	WP_053403299	---S-D---W-EKT---T-PI-M-		V--V-----S---T-------Q
	Bacillus korlensis	WP_066058508	--EQ----IW-ERI---THPV-MV		I-C------------I-----Q
	Bacillus licheniformis	OLO17383	---S-D--NW-QKT---THPV-MG		V-A------------------Q
	Bacillus litoralis	WP_121664189	---P-D--EW-SKT---T-PI-MV		I-V---L------T-------Q
	Bacillus loiseleuriae	WP_049679557	---S-K--EW--NNLT-QHPI-MV		I-V------S-----I-----Q
	Bacillus nakamurai	WP_061521277	---T-D--KW-QAN--NTHPV-MT		V-VV-----------------Q
	Bacillus niacini	WP_045516514	--PT-D--LW--KV---SHPI-MT		--A------------I-----Q
	Bacillus novalis	WP_066094735	---T-D--NW-NNNL--SRPI-MT		--AV-----------I-----Q
	Bacillus oceanisediminis	WP_110067557	--EQ-D--LW-QRI---TTPI-MV		I-S------------I-----Q
	Bacillus oleivorans	WP_097160685	--ED-A--NW-IRI---T-PI-MA		I-V------------I-----Q
	Bacillus oleronius	WP_078109865	---S-G--DT-QWV---TKPV-M-		I-V---V--------------Q
	Bacillus onubensis	WP_099364829	---P-A--EW-QKY---T-PV-M-		I------------T-------Q
	Bacillus oryziterrae	WP_017754844	---D-T--NW-ITT-N-SHPI-MV		V-V----------T-------K
	Bacillus paralicheniformis	WP_095323780	---T-D--NW-QKT---THPV-MG		V-A------------------Q
	Bacillus persicus	WP_090749640	---T-D--IW-QNT---TRPL-M-		I-V------------I-----K
	Bacillus salsus	WP_090858970	---Q-D--MW-QRT---T-PV-M-		--V---V------T-------Q
	Bacillus selenatarsenatis	WP_041966515	---T-D--LW-QKT---T-PI-M-		V-V------------I-----Q
	Bacillus shackletonii	WP_055737819	---S-N--DT-QWV---TKPV-A-		I-V------------------Q
	Bacillus siamensis	WP_045927002	---T-D--KW-QAN--NTHPV-MT		V-V------------------Q
	Bacillus simplex	WP_125162625	---K----SW--ENLA-TNPI-M-		I-V------A-----I-----Q
	Bacillus sinesaloumensis	WP_077620232	---P-E--DW-QTY---TRPV-M-		I------------T-------Q
	Bacillus smithii	WP_048622381	---NEK--ST-HNI---TKPV-ML		I-V------S-----------Q
	Bacillus soli	WP_066072358	--SQ-D--LW--KI---SHPI-MT		--A------------I-----Q
	Bacillus sonorensis	WP_006640334	---S-D--NW-QKT---THPV-MA		I-A------------------Q
	Bacillus subterraneus	WP_044393882	---T-D--LW-QKT---T-PI-MT		V-V------------I-----Q

Other Bacteria (0/>300)

Bacillus subtilis	WP_015482706	---T-D--KW-QNN--NTHPV-MA	I-V------------------Q
Bacillus testis	WP_050613650	--KQ-D--QW-INT---TKPV-M-	--V------S-----------Q
Bacillus thermoamylovorans	WP_041847225	---D-K---W--EW-N-SHPF-MS	I-VI-----------I-----Q
Bacillus thermocopriae	WP_128999496	---S-D--LW-KKV---T-PV-M-	I-V---V--------------Q
Bacillus timonensis	WP_010285091	---P-A--EW-QKY---T-PV-M-	I------------T-------Q
Bacillus tuaregi	WP_071396698	--EQ-D--LW-QKV---TNPI-MV	M-G------------I-----Q
Bacillus velezensis	RAP14068	-----D--KW-QAN--NTHPV-MT	V-V------------------Q
Bacillus vireti	WP_024027889	--SQ-D--LW-NRYL--SHPV-MT	--A------------I-----Q
Bacillus weihaiensis	WP_072581443	---S-D--EW-SKT---T-PV-M-	I------------T-------Q
Bacillus xiamenensis	WP_008356546	---T-N--TW--NN---TKPI-MT	I-V---V--------------Q
Bacillus zeae	WP_119112309	---T-D--LW-QKT---T-PI-M-	V-V------------I-----Q
Bhargavaea beijingensis	WP_092093273	---Q-RW-AA-IEI---TKPV-M-	I-V-------F----I------
Bhargavaea cecembensis	WP_040225818	---Q-KW-AA-IEI---TKPL-MV	I-V-------------------
Bhargavaea ginsengi	WP_092052994	---Q-RW-AA-IEI---TKPV-M-	I-V------------I------
Caenibacillus caldisaponilytic	WP_077616037	--H--A---W-IRT---TTA--MV	I-AL---------T-------K
Caryophanon latum	WP_066462964	-----D--TK--NF---TKP--ML	I-V------------I------
Caryophanon tenue	WP_066542818	-----DI-TN--NF---TKPI-MT	I-VV-----------I------
Chryseomicrobium excrementi	WP_100354152	---T----EAM-RVL--T-PI-MV	-FV---V--------I------
Domibacillus antri	WP_075397842	---S-DI-NK-QNI---T-PI-MA	I-V---V--------I-----Q
Domibacillus enclensis	WP_045851870	---S-D--ST-QNI---TKPV-MA	I-V---V--------I-----Q
Domibacillus epiphyticus	WP_076764323	---S-D--NT-QNI---TKPV-M-	I-V---V--------I-----Q
Domibacillus indicus	WP_046176566	---S-D--ST-QNI---TKPI-MA	I-V---V--------I-----Q
Domibacillus iocasae	WP_069937415	---S-D--TT-QNI---TKPV-MA	I-V---V--------I-----Q
Domibacillus mangrovi	WP_073712276	---S-D--TT-QNI---TKPI-MT	I-V------------I-----Q
Domibacillus robiginosus	WP_050182382	---S-D--ST-QNV---Q-PI-MA	I-V---V--------I-----Q
Domibacillus tundrae	WP_046180673	---S-D--TT-QNI---TKPV-MA	I-V---V--------I-----Q
Edaphobacillus lindanitolerans	WP_076758847	---Q-KW-AA-IEI---TKPV-M-	I-V---L---------------
Falsibacillus pallidus	WP_114746831	---Q-D--SA-EYI---TKPV-ML	I-V------S-----I-----Q
Falsibacillus sp. GY 10110	WP_121682429	---Q-D--TT-EWI---TKPV-ML	I-V------------I-----Q
Fictibacillus aquaticus	WP_094253174	--EQ----KW-INA---S-PI-M-	I-A----G-S---T-I-----Q
Filibacter sp. TB-66	WP_124068517	---T----TT--NV---TKPI-MV	---------------I------
Geobacillus genomosp. 3	WP_020958376	---T-D--LW-RRT---THPV-MT	I-VV-----------------Q
Geobacillus jurassicus	WP_066232471	---T-D--LW-RRT---THPV-MT	I-VV-----------------Q
Geobacillus kaustophilus	WP_044733004	---T-D--LW-RRT---THPV-M-	I-VV-----------------Q
Geobacillus kaustophilus GBlys	GAD13892	---T-D--LW-RRT---THPV-M-	I-VV-----------------Q
Geobacillus lituanicus	WP_094239434	---T-D--LW-RRT---THPI-MT	I-VV-----------------Q
Geobacillus thermoleovorans	WP_062898299	---T-D--LW-RRT---THPV-M-	I-VV-----------------Q
Geobacillus vulcani	WP_031406197	---T-D--LW-RRT---THPV-M-	I-VV-----------------Q
Jeotgalibacillus alimentarius	WP_041120846	---Q-NI-TT-QEV---T-PI-MT	I-V---V--------I------
Jeotgalibacillus campisalis	WP_041054729	---S-NI-TT-QQV--HT-PI-MT	I-VG-----------I------
Jeotgalibacillus malaysiensis	WP_039806315	---Q-NI-TT-QEV---T-PI-MT	I-V---V--------I------
Jeotgalibacillus marinus	AAT90906	-S-NI-VT-QEI---T-PI-MT	I-VG-----------I------
Jeotgalibacillus proteolyticus	WP_104059755	---S-NI-TT-QEV---T-PI-MT	I-VG-----------I------
Jeotgalibacillus salarius	WP_134382725	---Q-NI-TT-QEV---T-PI-MT	I-VG--V--------I------
Jeotgalibacillus soli	WP_041085463	---S-N--TT-QNV---T-PI-MA	I-V---V--------I------
Jeotgalibacillus sp. R-1-5s-1	WP_134374697	---Q-NI-TT-QDV---T-PI-MT	I-V---V--------I------
Jeotgalibacillus sp. S-D1	WP_133378452	---S-N--TT-QNV---T-PI-MT	I-V------A-----I------
Kurthia huakuii	WP_029500578	---D-E-AT--QNT---SKPV-M-	I-V------A-----------K
Kurthia massiliensis	WP_010290390	---DGK-AT--QNT---SKPV-M-	I-V------A-----------K
Kurthia senegalensis	WP_010307919	---DGK-AT--QNT---SKPV-M-	I-V------A-----------K
Kurthia sibirica	WP_109305658	L---GKTAT--ENT---SKPV-M-	I-----V--------------K
Kurthia sp. 3B1D	WP_126991956	---DGE-AT--QNT---SKPV-M-	I-V------A-----------K
Kurthia zopfii	WP_109349848	---DSKTATI-ANT---SKPV-ML	I-V------------------K
Lysinibacillus acetophenoni	WP_097149250	---Q----T--SNT---TKPV-ML	I-VG--A--------I------
Lysinibacillus chungkukjangi	WP_107932329	---Q----S---TT---TKPV-M-	I-----V--------I------
Lysinibacillus composti	WP_124766186	---Q-DI-S--QNT---TKPV-ML	I-V---V--------I------
Lysinibacillus endophyticus	WP_121214200	---Q-D--T--QNT---TKPV-M-	I-----VL-------I------
Lysinibacillus fusiformis	WP_025116435	---Q-D--T--ENT---TKPV-M-	I-V---V---------------
Lysinibacillus halotolerans	WP_122971289	---Q----S--QNT---TKPI-M-	I--------------I------
Lysinibacillus jejuensis	WP_108305996	-----N--T--QNT---TKPI-MM	I-V---V---------------
Lysinibacillus macroides	WP_053997244	-----TA-TW-ANNLL-T-PI-MT	I-VI------------------
Lysinibacillus manganicus	WP_036183097	---Q----T---NT---TKPV-ML	V-----T--------I------
Lysinibacillus mangiferihumi	WP_107894996	---Q-DI-T--QNT---QKPV-ML	I-V---V---------------
Lysinibacillus massiliensis	WP_036172473	---Q-DI-T--SNT-N-TEPV-ML	I-V---VL-------I------
Lysinibacillus odysseyi	WP_036151923	---Q----TW--NNLN-T-PI-MA	I--I------------------
Lysinibacillus parviboronicapi	WP_107925088	---Q-N--S--ENT---TKPV-M-	I-----V---------------
Lysinibacillus sinduriensis	WP_036200727	---Q-GF-T--LNT---NKPV-M-	I-----V--------I------
Lysinibacillus sp. 2017	WP_108714268	---D-N--S--SNT---TKPV-ML	I-VV-----A-----I------
Lysinibacillus sp. B2A1	AVK86673	-----G--T--ENT-N-EKPV-M-	V---------------------
Lysinibacillus sp. BF-4	WP_036143438	-----NI-T--QHT---TKPV-MM	I-V---V---------------
Lysinibacillus sp. BK089	WP_132362703	----GA--T--ENT---SKPV-MV	V-----V---------------
Lysinibacillus sp. FJAT-14222	WP_053596067	----GA--T--ENT--NTKPV-M-	V-V------------I------
Lysinibacillus sp. FJAT-14745	WP_053482804	---DGP-ST--EHT-N-TNPV-M-	I--V------------------
Lysinibacillus sp. Marseille-P	WP_106781811	---Q-DF-T--SNT---SKPV-ML	I-V---V--------I------

Other Bacteria (0/>300)

Lysinibacillus sp. SYSU K30002	WP_126658188	---Q-DI-T--QNT---TKPV-M-	I-V---V--------I------
Lysinibacillus sp. YLB-03	WP_118876555	---Q-Q--T---NT---TKPV-M-	I-V---V--------I------
Lysinibacillus sp. ZYM-1	WP_054610123	-----G--T--NNT--HTKPV-M-	I-V-------------------
Lysinibacillus sphaericus	WP_031416715	---Q-D--T--ENT---TKPV-M-	I-V---V---------------
Lysinibacillus sphaericus C3-4	ACA42038	---Q-D--T--ENT---TKPV-M-	I-V---V---------------
Lysinibacillus telephonicus	WP_126292307	---Q----S--Q-T---TKPV-M-	I------L--F----I------
Lysinibacillus xylanilyticus	WP_068986536	----SA--T--ENT---TKPV-M-	I-V---V---------------
Lysinibacillus xyleni	WP_097073491	---Q-DI-S--QNT---TKPV-M-	I-----V--------I------
Mycobacteroides abscessus subs	SHS94735	--EQ----IW-ERI---THPI-MV	I-C------------I-----Q
Paenibacillus sp. FSL R5-0490	WP_076262054	--EQ-D--LW-QRI---TSPI-MV	I-S------------I-----Q
Paenisporosarcina indica	WP_075620179	---Q-D--D--QNT---SKPV-ML	I-V---V--------I------
Paenisporosarcina sp. HGH0030	WP_016429902	---Q-K--D--QNT---S-PV-ML	I-V---V--------I------
Paenisporosarcina sp. K2R23-3	WP_119884307	---Q----D--QNT---TKPL-ML	I-V------------I-----Q
Paenisporosarcina sp. OV554	WP_108586015	---Q-N--D--QNT---SKPV-ML	I-V------------I------
Paenisporosarcina sp. TG20	WP_019415642	-----N--L--QNT---SKPV-MM	I-V------------I-----K
Parageobacillus caldoxylosilyt	WP_042410113	---S-D--LW-RKT---THPV-MV	--VL-----------------Q
Parageobacillus genomosp. 1	WP_043903332	---S-D--LW-RKT---AHPV-MV	I-VL-----------------Q
Parageobacillus thermantarctic	WP_090949469	---S-D--LW-RKT---T-PV-MV	I-V------------------Q
Parageobacillus thermoglucosid	WP_125010803	---S-D--LW-RNT---T-PV-MV	I-VV-----------------Q
Parageobacillus toebii	WP_062677098	---S-D--LW-RKT---T-PV-MV	I-VL-----------------Q
Planococcus antarcticus	WP_006828948	---A-AF-EA-QNT---TRPV-M-	I-V---V--------I------
Planococcus donghaensis	WP_065527563	---A-AF-EA-QNT---TRPV-M-	I-V---V--------I------
Planococcus halocryophilus	WP_008497446	---A-AF-EA-QNT---TRPV-M-	I-V---V--------I------
Planococcus maritimus	WP_068459716	---A-AF-EA-QNT---TRPV-M-	I-V------------I------
Planococcus massiliensis	WP_052654082	---A-AF-DA-QNT---TRPV-M-	I-V---V--------I------
Planococcus plakortidis	WP_068871373	---A-AF-EA-QNT---TRPV-M-	I-V------------I------
Planococcus rifietoensis	WP_058382146	---A-AF-EA-QNT---TRPV-M-	I-V------------I------
Planococcus salinarum	TAA68385	---A-AF-DAVQNT---T-PV-M-	I-V------------I------
Planococcus salinus	WP_123166168	---A-AF-DA-QNT---TRPV-MV	I-V---V--------I------
Planococcus sp. CAU13	WP_033542781	---A-AF-EA-ENT---T-PV-M-	I-V------------I------
Planococcus sp. PAMC 21323	WP_038701930	---A-AF-EA-QNT---TRPV-M-	I-V---V--------I------
Planococcus sp. Y42	WP_077587667	---S-TF-DA-QRT---T-PV-ML	I-V------------I------
Planococcus versutus	WP_049694560	---A-AF-EA-QNT---TRPV-M-	I-V------------I------
Planomicrobium flavidum	WP_088006769	---S-TF-DA-QRT---TEPV-ML	I-V------------I------
Planomicrobium glaciei	WP_036803942	---A-AF-DAVQNT---TRPV-M-	I-V------------I------
Planomicrobium soli	WP_106534453	---A-AF-DA-QNT---TRPV-M-	I-V---V--------I------
Pseudomonas sp. GW456-E7	PNB36031	---T-D--KW-QNY--NTHPV-MA	I-V------------------Q
Psychrobacillus insolitus	WP_111440061	---Q-N--DA--KAL--T-PI-MT	I-V------------I------
Psychrobacillus psychrotoleran	WP_093537846	---Q-NT-DA--RAL--T-PI-MT	I--------------I------
Psychrobacillus sp. FJAT-21963	WP_056831183	---Q-K--D--SNAL--T-PV-M-	I--------S-----I------
Psychrobacillus sp. OK028	WP_093062501	---Q-N--DS--RAL--T-PI-MT	I--------------I------
Psychrobacillus sp. OK032	WP_093274708	---Q--T-D---RAL--T-PI-M-	I-----V--------I------
Quasibacillus thermotolerans	WP_039234451	---S-D--TT-QNI---TKPV-M-	I-V---V--------I-----Q
Rhizophagus irregularis	PKC51895	---Q-DI-T--SNT-N-TEPV-ML	I-V---VL-------I------
Rhodococcus qingshengii	WP_133371729	--PT-D--LW--RV---SHPI-MT	--A------------I-----Q
Rummeliibacillus pycnus	WP_102693562	--EDTK--Q--EKT---G-PI-M-	--V------A-----------K
Rummeliibacillus stabekisii	WP_066790875	---DT------EKT---S-PI-M-	I-V---V--------------K
Solibacillus isronensis	WP_079523478	---D-N--A---NT---TKPV-ML	I-V------------I------
Solibacillus kalamii	WP_087617854	---D-N--A---NT---TKPV-ML	I-V------------I------
Solibacillus sp. R5-41	WP_099425580	---D-K--A---NT---SKPV-MV	I-----V--------I------
Sporosarcina globispora	WP_053433180	--EQ-D--LW-QRI---TSPI-MV	I-S------------I-----Q
Sporosarcina koreensis	WP_060203450	---D----K---RT---N-PV-M-	I-V---L--------I------
Sporosarcina newyorkensis	WP_009499037	--------KT-VRI---TNPI-M-	--VT--V--------I------
Sporosarcina pasteurii	WP_115363616	-----K--AT-INV---TNPV-MV	I-L------------I------
Sporosarcina psychrophila	WP_067204799	---T----TT--NV---TKPV-MV	F-LI-----------I------
Thermolongibacillus altinsuens	WP_132949320	---S-D--LW-KKV---T-PV-M-	I-V------------------Q
Turicibacter sanguinis	WP_055165242	-LP-SG--KW--TI---T-PI-F-	--VV--V-----------S--K
Ureibacillus thermophilus	QBK24951	---Q-K--T--AN----TKPV-M-	---------------------R
Ureibacillus thermosphaericus	WP_016839388	-----KI-T--INT---TKPV-M-	I--------------I-----R
Vibrio vulnificus	WP_133352913	---K----SW-STNL--TKPI-M-	I-V------A-----I-----Q
Viridibacillus arvi	WP_053418548	---D-KA-Q--ERT---S-PV-M-	I-V---V--A-----------K
Viridibacillus sp. OK051	WP_100795212	---D-KA-Q--QRT---S-PV-MV	M-V---M--A-----------K

Supplemental Figure 50

A partial sequence alignment of the preprotein translocase subunit SecY protein containing a three amino acid insertion (boxed) that is exclusively shared by all members belonging to the Meyeri clade and absent in all other bacteria.

			336		373
**Meyeri Clade (3/3)**	Lysinibacillus fluoroglycofeni	WP_107942022	TNTVVMLTALAAKNAVI		ESYADGLVLIAVTAGIDNAVDV
	Bacillus ndiopicus	WP_042470765	----M-----E-Q----		--------IV-----VG-----
	Lysinibacillus meyeri	WP_107841861	----M----I--Q-VI-		--------IV------R-----
**Other Bacteria (0/>100)**	Viridibacillus sp. FSL H8-0123	WP_076065806	S---A-M--V---HV--	RE	F-N-N-SIIV-----VG-----
	Viridibacillus sp. FSL H7-0596	WP_076034284	S---A-M--V---HV--	RE	F-N-N-SIIV-----VG-----
	Viridibacillus arenosi	WP_038180116	S---A-M--V---HV--	RE	F-N-N-SIIV-----VG-----
	Viridibacillus arvi	WP_053417924	S---A-M--V---HV--	RE	F-NTT-SIIV-----VG-----
	Rummeliibacillus pycnus	WP_102693132	----A-M--V-S-HV--	RE	F-SSA-SIV------VG-----
	Sporosarcina sp. P13	WP_099687941	-D--A-M--VSPEHV-V	EE	YQGDF-SIV-------G-----
	Rummeliibacillus	WP_119414057	S---A-M--V-S-HV--	RE	Y-SQV-SIVV-----VG-----
	Solibacillus sp. R5-41	WP_099422373	---------V-T-C-E-	QS	F-SGNCEIVVM----VG-GI--
	Sporosarcina sp. PTS2304	WP_114925645	-D--A-M--VSPEHVEV	EE	FAGDF-SIVV------G-----
	Paenisporosarcina sp. TG20	WP_019413051	-D--G-M--VRTE----	EE	YKGTF-SII------VG-----
	Paenisporosarcina antarctica	WP_134210691	-D--G-M--VQT-HV--	EE	YKGTF-SII-------G-----
	Lysinibacillus sp. B2A1	AVK82651	-S-------VPSRLVA-	NE	YTASF-S--V-----VG-----
	Planococcus sp. PAMC 21323	WP_038704752	-D--G-M--VQT-D-IV	KE	YEGPF-S-V------VG-G---
	Kurthia massiliensis	WP_010287123	EQ--A-M--V--EH-I-	RH	YTEG--DIFVM-----G-----
	Lysinibacillus sp. ZYM-1	WP_054611224	---------VPTALVA-	NE	FYAPF-S-IV-----VG-----
	Viridibacillus sp. OK051	WP_100794873	S---A-M--V-VEHV--	RE	F-SSI-SIIV-----VG-----
	Psychrobacillus insolitus	WP_111439284	-E--G-M--VYT-D---	RE	YAGDF-S-V-M----VG-----
	Sporosarcina pasteurii	WP_115359900	-E--G-M--AKTEFVE-	ES	YEGDF-T--V-----LG-----
	Planomicrobium sp. Y74	WP_121635638	-D--G-M--VQTEH--V	KE	YEGPF-T-V-------G-G---
	Lysinibacillus sphaericus	WP_075529034	-E--G-M--AKTEFVE-	ES	YEGDF-T--V-----LG-----
	Sporosarcina psychrophila	WP_067213220	-D--G-M--VMTEH-EV	EE	YTGDF-TI-------VG-----
	Paenisporosarcina sp. TG-14	WP_017382187	-D--G-M--VQT-HV--	EE	YKGTF-SII------VG-----
	Sporosarcina sp. EUR3 2.2.2	WP_024535599	-D--G-M--VQT-HV--	EE	YVGTF-SII------VG-----
	Bacillus cecembensis	WP_057986688	---------V-MNC-E-	QA	F-SGSCEIVVM----VG-GI--
	Lysinibacillus sp. BF-4	WP_036146914	EE--A-M--VY--H---	RT	FTHGELSLTVM----LG----I
	Lysinibacillus saudimassiliens	CEA00477	EE--A-M--VY--H---	RT	FTHGELSLTVM----LG----I
	Paenisporosarcina sp. HGH0030	WP_016428304	-D--G-M--VQT-HV--	EE	YDGTF-SII------VG-----
	Lysinibacillus odysseyi	WP_036153110	---------V-S-L-AV	RS	FKGNQCSITV-----VG-----
	Bacillus sp. FJAT-22090	WP_053590561	-E--G-M--VQT-D---	KE	YEGDF-S-F-M----VG-----
	Paenisporosarcina sp. OV554	WP_108586138	-D--G-M--VQT-HV--	EE	YNGTF-SII------VG-----
	Psychrobacillus sp. FJAT-21963	WP_056828105	-E--G-M--VQT-D---	KE	YEGDF-S-F-M----VG-----
	Kurthia sp. 3B1D	WP_126990953	EQ--A-M--VDV-H---	RR	YKEE-HEIFVM----VG-----
	Paenisporosarcina sp. K2R23-3	WP_119882541	-E-MA-M--ISPHK---	KQ	Y-GTEFV-TVM-----G--M--
	Kurthia zopfii	WP_126342202	PM--A-M--V--EH-I-	RS	YED-GVSIVVM----VG-----
	Lysinibacillus sp. PB300	WP_115674202	---------VPTALVA-	NE	F-APF-SIIV-----VG-----
	Lysinibacillus sp. YS11	WP_103117414	---------VPTALVA-	NE	F-APF-SIIV-----VG-----
	Kurthia sibirica	WP_109306172	SK--A-M--V-V-H-I-	KE	YEEDGLSIVVV----VG-----
	Lysinibacillus sp. LK3	WP_048393746	---------VPTALVA-	NE	F-APF-SIIV-----VG-----
	Lysinibacillus sp. OL1	WP_131520775	---------VPTALVA-	NE	F-APF-SIIV-----VG-----
	Bacillus sp. B14905	WP_008181894	D---A-M--VQ--F-T-	RD	FT-EGIHLV-MI---LG----I
	Lysinibacillus	WP_089984787	D---A-M--VQ--F-T-	RD	FT-EGIHLV-MI---LG----I
	Sporosarcina sp. P33	WP_081243811	-D--A-M--VSPEHVEV	HE	YEADF-SIVVV----VG-----
	Planococcus sp. CAU13	WP_033541791	-D--G-M--VQTRH-IV	KE	YEGTF-S-V------VG-G---
	Sporosarcina	WP_085431016	-D--A-M--VSPEHVEV	HE	YEADF-SIVVV----VG-----
	Planococcus halotolerans	WP_112221245	-D--G-M--VQTEH--V	KE	YEGPF-T-V-T----VG-G---
	Lysinibacillus boronitolerans	WP_036078229	---------VPTALVA-	NE	F-APF-SIIV-----VG-----
	Kurthia sp. Dielmo	WP_020189064	EQ--A-M--VDV-H---	RR	YKED-HELFVM----VG-----
	Planococcus sp. Y42	WP_077590207	-D--A-M--VRTED-IV	KE	YEG-F-S-I------VG-----
	Lysinibacillus fusiformis	WP_009371951	---------VPTALVA-	NE	F-APF-SIIV-----VG-----
	Solibacillus isronensis	WP_079524614	---------VETSC---	RH	F-KNNVEI-VM----VG-S---
	Tetzosporium hominis	WP_094943583	-E--MQ---V-T-G---	RE	YDAPF-S-V-L----VG--L--
	Planococcus kocurii	WP_058384426	-D--G-M--VQTQD-IV	KE	YAGPF-S-V------VG-G---
	Planococcus faecalis	WP_071153026	-D--G-M--VQTQD-IV	KE	YAGPF-S-V------VG-G---
	Sporosarcina koreensis	WP_040287774	-G--G-M--VHT-D-E-	RR	YEEDGFS-V------VG-----
	Rummeliibacillus stabekisii	WP_066791688	----A-M--VP-----V	RQ	FTSHEADIVVV----VG--I-I
	Lysinibacillus sp. AR18-8	WP_066036947	---------VPTALVA-	NE	F-APF-SIIV-----VG-----
	Lysinibacillus contaminans	WP_053585235	-G-------VSTNMVAL	NE	YKTDF-S-FVM----VG-----
	Planococcus donghaensis	WP_008432914	-D--G-M--VQTQD-IV	KE	YEGPF-S-V------VG-G---
	Lysinibacillus sp. Marseille-P	WP_106784492	ED--A-M--VNMQF-H-	DL	YEDGETS-T-F----LG-T---
	Planococcus versutus	WP_049692958	-D--G-M--VQTQD-IV	KE	YESLF-S-V------VG-G---
	Psychrobacillus sp. OK032	WP_093267767	-E--G-M--VYT-D---	KQ	YDGDF-S-IVM----VG-----
	Sporosarcina sp. ZBG7A	WP_039044509	-D--G-M--VQTLH-E-	AS	FADEGISIV------VG-----
	Sporosarcina ureae	WP_029053489	-D--A-M--VSPEHVEV	EE	YEGEF-SIVV-----VG-----
	Sporosarcina sp. P20a	WP_099678751	-D--A-M--VSPEHVEV	EE	YEGEF-SIVV-----VG-----
	Sporosarcina sp. D27	WP_025785556	-D--G-M--VQTLH-E-	AS	FADKGISIV------VG-----
	Sporosarcina sp. P3	WP_099638813	-D--A-M--VSPEHVEV	EE	YEGPF-SIVV-----VG-----
	Sporosarcina sp. P19	WP_099691880	-D--A-M--VSPEHVEV	EE	YEGEF-SIVV-----VG-----
	Bacillus taeanensis	WP_113805238	GH--G-M--ANL-DG--	KE	IGHEQFS---I----LS-----
	Paenisporosarcina indica	WP_075618409	SD--G-M--VQT-DV--	EE	YHGTF-SI-------VG-----
	Kurthia gibsonii	WP_087682479	DQ--A-M--V--RY-I-	RE	FEADGIH-V-M----VG-----
	Lysinibacillus sphaericus C3-4	ACA39994	D---A-M--VQ--F-T-	RD	FT-EGIHLV-MI---LG----I

Other Bacteria (0/>100)

Filibacter sp. TB-66	WP_124068764	-D--G-M--VTTQH-EV	EE	YHADF-S-I-V----VG-----
Solibacillus kalamii	WP_087615686	---------VETSC-A-	RH	F-KNNAEI-VM----VG-S---
Chryseomicrobium excrementi	WP_100353365	-E--MQ---V-TRG---	RE	YEAPF-S-V-L----VG--L--
Solibacillus silvestris	WP_065216624	---------VETSC-A-	RH	F-KNNAEI-VM----VG-S---
Solibacillus	WP_008404521	---------VETSC-A-	RH	F-KNNAEI-VM----VG-S---
Lysinibacillus chungkukjangi	WP_107932444	----G-M--VDT-K--V	KN	Y-MPF-E-IVV----VG--I--
Sporosarcina sp. P17b	WP_099625024	-D--A-M--VSPEHVEV	EE	YEGEF-SIVVV-----G-----
Sporosarcina sp. BIOO1-red	WP_116019618	-D--G-M--VQTLH-E-	AT	YADE-ISIV------VG-----
Sporosarcina sp. P7	WP_099637525	-D--A-M--VSPEHVEV	EE	YEGEF-SIVV------G-----
Butyricicoccus sp. 1XD8-22	RKJ62922	----G-M--VDTQD---	KS	YDASF-T-FVI----VG--I--
Lysinibacillus sinduriensis	WP_036200070	----G-M--VDTSK---	KS	YEMPF-E-I------VG--I--
Bhargavaea beijingensis	WP_092097429	-Q--A-M--AMTED---	GE	YGPP-APIV-C----VG-----
Kurthia senegalensis	WP_010302556	EQ--A-M--VDV-H-I-	RH	FKND-DDIFVMI---VG-----
Planococcus maitriensis	WP_112230415	-D--A-M--VRTEH-IV	RE	YAGDF-SIVV-----VG-G---
Lysinibacillus xylanilyticus	WP_068986692	-S----I--VP-NLVA-	NE	FKASF-S-FV-----VG-----
Sporosarcina sp. P1	WP_099627773	-D--A-M--VSPEHVEV	EE	YEGQF-SIVV-----VG-----
Lysinibacillus sp. BK089	WP_132360298	-S-------VPTNLVA-	NE	YKASF-S--V-----VG-----
Planococcus citreus	WP_121298395	-D--A-M--VRTEH-IV	RE	YASGF-SIVV-----VG-G---
Lysinibacillus macroides	WP_053993154	-S-------VPTSLVA-	QE	F-ASF-NMIV-----VG-----
Lysinibacillus sp. FJAT-14222	WP_053596482	-S-------VPTELVA-	NE	FKASF-S--V------G-----
Sporosarcina sp. P16b	WP_099672286	-D--A-M--VSPEHVEV	EE	YEGEF-SIVV-----VG-----
Sporosarcina sp. P18a	WP_099675578	-D--A-M--VSPEHVEV	EE	YEGEF-SIVV-----VG-----
Sporosarcina sp. P2	WP_099631219	-D--A-M--VSPEHVEV	EE	YEGEF-SIVV-----VG-----
Sporosarcina sp. P34	WP_099695448	-D--A-M--VSPEHVEV	EE	YEGEF-SIVV-----VG-----
Lysinibacillus parviboronicapi	WP_107947560	-S-------VSTEL-AL	NK	YKAPF-GIFVV----VGK----
Lysinibacillus sp. YR326	WP_134026563	D---A-M--VY-RY-T-	RE	FT-EGIHLV-MI---LG----I
Sporosarcina newyorkensis	WP_009766251	-D--A-M--VSPEYVNV	QE	FKAEF-SIV----V--G-----
Sporosarcina sp. HYOO8	WP_067405395	-D--G-M--VQ-EHVEF	GK	YEEDF-SI--------G-----
Planomicrobium glaciei	WP_036809679	-D--G-M--VRTED-IV	KE	YEAPF-S-V------VG-----
Bhargavaea cecembensis	WP_040227538	SQ--A-M--AMTED---	GE	YGPE-AP-VVC----VG-----
Planomicrobium okeanokoites	WP_084244184	-D-IG-M--VQTEH-IV	KE	YEGPF-TII------VG-G---
Planomicrobium sp. MB-3u-38	WP_101801404	-D-IG-M--VQTEH-IV	KE	YEGPF-TII------VG-G---
Lysinibacillus mangiferihumi	WP_107896686	-S-------VPTQLFAL	NE	Y-TEF-S-FVV----VG-----
Planococcus antarcticus	WP_006829277	-D--G-M--VQTQDVIV	KE	YEGLF-SIV------VG-G---
Planococcus massiliensis	WP_052650651	-E--G-M--VRTEH--V	KE	YQGDF-S-V------VG-----
Lysinibacillus massiliensis	WP_036171189	----G-M--VDTQD---	KS	YDASF-T-FVI----VG--I--
Lysinibacillus endophyticus	WP_121214078	----G-M--VESSK-I-	KQ	YNASF-E-IVI-----GG-I--
Bacillus daliensis	WP_090843467	EQSIG-M--VKVE----	KK	-VLNGIAI-VV----TG----I
Bacillus sp. Leaf406	WP_056536219	SR--G-M---DLSL-AH	GR	YEDG-KA-FVV----TG--I--
Bacillus marisflavi	WP_079516172	SR--G-M---DLSL-AH	GR	YEDG-KA-FVV----TG--I--
Planococcus maritimus	WP_068487055	-D--A-M--VRTEH-IV	RE	YESEF-SIVV-----VG-G---
Planococcus	WP_101805551	-D--A-M--VRTEH-IV	RE	YAGDF-SIVV-----VG-G---
Bacillus sp. V5-8f	WP_101631471	EE--G-M--VNV-D-AC	QF	YEEDGIQ-F-V----TG-----
Bhargavaea ginsengi	SEJ65360	-Q--A-M--AMTED---	GE	YGPP-APIVVC----VG-----
Lysinibacillus sp. SYSU K3OOO2	WP_126657994	EQ-IA-M--VDMEF-F-	DC	YEEGETSIV-M----LG-----
Planococcus halocryophilus	WP_008496862	-D--G-M--VQTQDVIV	KE	YEGPF-S-I------VG-G---
Lysinibacillus sp. FJAT-14745	WP_053484478	-S-------VPTNLVA-	NE	FKASF-S--V-----VG-----
Planomicrobium soli	WP_106532814	-D--G-M--VRTED-IV	KE	YMGPF-S-V-V----VG-----
Ureibacillus thermophilus	QBK25719	DK--A-M--VDM-F-FS	RF	YHDG-TSI-LL----LN-----
Planococcus rifietoensis	WP_058382924	-D--A-M--VRTEY-IV	RE	YAGDF-SIVV-----VG-G---
Lysinibacillus jejuensis	WP_108306501	AA--G-M--VP-RH-L-	RT	FCEQQTHITVM----LG----I
Planococcus plakortidis	WP_068870112	-D--A-M--VRTEH-IV	RE	YKADF-SIVV-----VG-G---
Lysinibacillus xyleni	WP_097071649	----G-M--VE-RK-I-	KQ	YETPF-E-YVI-----GG-I--
Caryophanon latum	WP_066466331	--C-G-M---TTDH---	RE	FNSEA-HLIV-----VGK-I--
Bacillus nealsonii	WP_101177177	EQ--G-M--VYIED-AY	KS	VVCK-LS-F-V----VG-----
Lysinibacillus sp. F5	WP_058845014	H---A-M--VQ-QF-T-	RD	FTFEGIQLVVMI---LG----I
Edaphobacillus lindanitolerans	WP_076756817	SQS-A-M--AMTED---	RE	YGPKEAPIVVC----VG-----
Jeotgalibacillus soli	WP_041086943	SE--G-M--VRVEDV--	KE	YKEN-LSLVV-----VG-----
Bacillus	WP_040209562	AE--G-M--VMLEDV-F	RR	FVHE-FSIFVV----VS--I--
Bacillus tuaregi	WP_071396459	SE--G-M--VYT-DV-F	RH	YEEQGFSLMVI----VG-----
Bacillus oleivorans	WP_097159716	SD--A-M--IPLSTG-V	KE	YRDG-TSLFVV-----GG----
Bacillus thermoamylovorans	WP_041846849	S---G-M--VKME-VQY	KQ	YRDK-FSI-VV----VG-----
Bacillus hisashii	WP_095141619	S---G-M--VKME-VQY	KQ	YLDK-FSI-VV----VG-----
Bacillus sp. CGMCC 1.16541	WP_110111947	E-V-GLM--VYL-EC-V	YT	YQSD-LIISSII---VG----I
Planococcus salinus	WP_123164563	-D--G-M--VKTEDVI-	KE	YTGDFSSLTV-----VG-----
Bacillus sp. WNO66	WP_133333519	SE--G-M--VILEDL-Y	RS	YEEN-FSIF-V----VG--I--
Oceanobacillus sojae	WP_077602282	SQ--G-M--V-LEDNSY	RV	YEGDGFS-M-V----VG-----
Bacillus sp. SAJ1	WP_126649763	E---G-M--VMIED--S	EF	VMDDGFSLF-V----VG-----
Bacillus sp. OxB-1	WP_052483901	AE--G-M--VSTEHVE-	GQ	YDGDF-SI-V-----VG-----
Bacillus shackletonii	WP_055740018	E---G-M--VMIED--S	EF	VMDDGFSLF-V----VG-----
Bacillus pseudofirmus	WP_075385101	EG--A-M--AILED-SF	KT	YTNEAFT-FVV----VS-----
Bacillus alveayuensis	WP_044747552	KE--G-M--AKLEDVA-	RE	MNEGNASIVVV----VS-----
Bacillaceae bacterium B16-10	WP_129076225	SE--G-M--AVLED-EF	II	KEFEEFT-FVM----LS-----
Bacillus massilionigeriensis	WP_075980463	AD--G-M--VMLEDVIY	KM	YEGENFS-F-V----VG-----

Other Bacteria (0/>100)	Kurthia huakuii	WP_029497786	EQ--A-M--VDVQH---	RR	YTNDEHEIFVM----VG-----
	Oceanobacillus jeddahense	WP_040978223	SQ--G-M--V-LEDNSH	RV	YEGDGFS-M-V----VG-----
	Bacillus methanolicus	WP_034669473	VD--G-M--VNLED--Y	QL	FRGDGFS-FVVI---AG-----
	Bacillus methanolicus MGA3	EIJ81167	VD--G-M--VNLED--Y	QL	FRGDGFS-FVVI---AG-----
	Tetragenococcus halophilus	WP_061840945	SQ--G-M--VTLEDNSY	CL	YEGDGFS-M-V-----G-----
	Bacillus litoralis	WP_066339535	YD-IG-M--VNL-DMS-	LK	-T-E-VQIIAVI---VG--I-I
	Planomicrobium flavidum	WP_088007630	-E--G-M--VRTEG--V	RE	YTF-A-TLV-M-----GT-T--
	Bacillus soli	WP_066064677	SE--G-M--VVLEDVIF	KL	FKQE-FS-FVV----VG--I--
	Bacillus oceanisediminis	WP_019381229	GE--G-M--VYV-DHSS	KF	YEDGTFS-YVV----VG-----
	Bacillaceae bacterium SAS-127	WP_094834240	NE--G-M--VDLEY-SY	RL	VEQDGMS-FVV----VG-----
	Parageobacillus caldoxylosilyt	WP_042410995	SS-IG-M--VDIHD--C	IY	-KQDAFSLWAV----VG-----
	Geobacillus sp. 44B	WP_081161288	SS-IG-M--VDIHD--C	IY	-KQDAFSLWAV----VG-----
	Bacillus cavernae	WP_126863650	EE--G-M--VNLND-SF	QL	YQEGEIS-F-V----TG-----
	Thermoplasma acidophilum	WP_010901304	ED---T---ADVSRY-H	AS	KHVEGWHID-FL---F---ISI
	Caryophanon tenue	WP_066547040	-S--G-M---SMDAV-A	ND	YDTPA-KFTVI----VGK-I--
	Thalassobacillus sp. C254	WP_054637372	AD--G-M--AVLED-AF	RR	F-TE-ADL--M----TS-----
	Bacillus praedii	WP_057766424	GE--G-M--VYPEDVSY	QL	YQADGFSAF-V----VG--I--
	Halomonas gudaonensis	WP_134845676	GES-A-M--VRLAELAV	VE	-RVSGIALTV-----VS----I

Supplemental Figure 51

A partial sequence alignment of the ATP-binding cassette domain-containing protein containing a two amino acid deletion (boxed) that is exclusively shared by all members belonging to the Meyeri clade and absent in all other bacteria.

Group	Organism	Accession	368		416
**Meyeri Clade (3/3)**	Bacillus ndiopicus	WP_042470344	VACAVGLGIGVSVVPELFAALPK	EF	GLNILTSNGIVCGSVTAIVLNIVF
	Lysinibacillus fluoroglycofeni	WP_107941886	-----------A-------S--S	K-	------------------------
	Lysinibacillus meyeri	WP_107841676	--IS--------------V---S	K-	---D-A-------A----------
**Other Bacteria (0/>200)**	Anoxybacillus amylolyticus	WP_131873770	I--S--V-L--TA--N---E--T		--R---DS---A--M---I--A--
	Anoxybacillus tepidamans	WP_027409174	I--S--M-L--TA--N---D--S		SIR---D----A--M---I-----
	Bacillus acidicola	WP_083952992	I--S--M-L--T---N---H--Q		SVQ--------I--L---L--L--
	Bacillus andreraoultii	WP_033828658	---S----L--T----M-RS--V		--R---ES---L--L--V----L-
	Bacillus azotoformans	WP_035197095	I--S--M-L--TT---M--Q--S		SVQ--------A--L---A---M-
	Bacillus badius	WP_041096869	I--S--M-L--T-------G--S		--QV--G----A--L---F---L-
	Bacillus boroniphilus JCM 2173	GAE44443	I--S--M-L--TA------QM-S		SIR---D----A--L------L--
	Bacillus butanolivorans	WP_098176866	I--S--M-L--T-------QI-E		SIQ---D----A--L---F-----
	Bacillus cereus	PFJ41953	---S--I-L--T---T--SQ--E		NIR---D----L--AS-VL-----
	Bacillus dakarensis	WP_077213740	I--S-CM-L--T---D---Q--S		-IS--------A--L---I--LI-
	Bacillus drentensis	WP_066254746	I--S--M-M--TA--DM--H--E		-VK----S---T--L---F-----
	Bacillus endophyticus	WP_061803665	I--S--M-L--T---D---N--Q		S-Q--------A--L-------F-
	Bacillus filamentosus	WP_019393134	I--S--M-L--T---D---N--Q		S-Q--------A--L-------F-
	Bacillus firmus	WP_035333549	I--S--M-L--TA------QM-S		SIR---D----A-----L------
	Bacillus flexus	WP_119543028	I--SI-M-L--T---D---Q--E		-IQ---G----A--F---I-----
	Bacillus fordii	WP_026319710	I--S----L--T-A-N--S---P		--Q--------A------A---I-
	Bacillus fortis	WP_128205420	I--S----L--T-A-D--S---S		--Q--------A------A---I-
	Bacillus galactosidilyticus	KRG11504	I--S--I-L--T-A-D------T		--Q--------A--I---I---L-
	Bacillus horneckiae	WP_066395292	I--S--M-L------G--SE--E		N-R---E----A--I---F---L-
	Bacillus indicus	WP_029280368	I--S--M-L--TA--DM-----E		SVK---N----A--L---L-----
	Bacillus intestinalis	WP_088679241	---S----L--T---DI-KQ--S		A-TL--T----A--F--V----IY
	Bacillus jeotgali	WP_102262366	I--S--M-L--TA------QM-S		SIR---D----A--L------L--
	Bacillus koreensis	WP_053399908	I--S--M-L--T---D---KF-T		A-Q---G----A--I------LI-
	Bacillus lentus	WP_066137915	I--SI-I-L--T-------Q--S		S-Q--------A--M---I---L-
	Bacillus litoralis	WP_066332479	---S----L--T----I--G--E		SVK---NS---A--I---------
	Bacillus loiseleuriae	WP_082191118	I--S--M-L--T---DI-LK--E		S-Q--------A--IS--F---L-
	Bacillus massiliosenegalensis	WP_019156700	---SI-M-L--TT--AI-DN--E		-IH--------I--L------VI-
	Bacillus mediterraneensis	WP_071459919	I--S--M-L--TA------K--E		NVQ--------A--L---I--V--
	Bacillus methanolicus	WP_004437213	I--S--M-L--TA--D---KM-E		S-R---D----A--L---F-----
	Bacillus muralis	WP_057915144	I--S--M-L--T------SQI-E		SFQ---E----A--L---F-----
	Bacillus nakamurai	WP_061521007	---S----L--T---DM-KH--S		Y--L--T----A--F--VL----Y
	Bacillus niacini	WP_045521773	I--S--M-L--TA--D---Q--E		SVK---N----A--L---F-----
	Bacillus niameyensis	WP_062104325	I--S----L--T-A-N------S		--Q--------A----------I-
	Bacillus oceanisediminis	WP_110064162	I--S--M-L--TA------QM-S		NIR---D----A--M---I--L--
	Bacillus oleivorans	WP_097158659	I--S--M-L--T---DI--K--Q		SVQ---D----A------L---I-
	Bacillus onubensis	WP_099364733	M--SI-M-L-ITT--T---N--E		-VQ--------I--L----M-LI-
	Bacillus sporothermodurans	WP_066228503	I--S--M-M--T---G---N--E		NIQ--------T--L---I---L-
	Bacillus subtilis	WP_103746485	---S----L--T---DI-KQ--S		A-TL--T----------V-----Y
	Bacillus subtilis group	WP_075750646	---S----L--T---DI-KQ--S		A-TL--T-------I--V-----Y
	Bacillus tequilensis	WP_024715302	---S----L--T---DI-KQ--S		A-AL--T----A-----V-----Y
	Bacillus terrae	WP_126646572	I--S----L--T-A-N--S---S		--Q--------A------A---L-
	Bacillus testis	WP_050615372	I--S--M-L--T---D---Q--E		NIK---N----A--L---F---I-
	Bacillus vallismortis	WP_100740086	---S----L--T---DM-KQ--S		A-TL--T----A--L--V-----Y
	Bacillus velezensis	WP_077199867	---S----L--T---DM-KH--S		Y--L--T----A--F--VI----Y
	Bacillus velezensis NAU-B3	CDH95484	---S----L--T---DM-KH--S		Y--L--T----A--F--VI----Y
	Bacillus wudalianchiensis	WP_065409258	I--S--M-L--T---G---G--S		SVQ--------A--L---F---L-
	Bacillus zeae	WP_119113462	I--S--M-L--TA-----SKM-E		S-Q---G----A--I---L-----
	Bhargavaea beijingensis	WP_092098109	---S----L------D--S---Q		-VQ--------A--F---------
	Bhargavaea cecembensis	WP_035001690	---S----L------D--S---Q		-VQ--------A--F---------
	Bhargavaea cecembensis DSE10	EMR06801	---S----L------D--S---Q		-VQ--------A--F---------
	Bhargavaea ginsengi	WP_092050935	---S----L------D--SS--Q		-VQL-------A--F---L-----
	Brochothrix campestris	WP_035315118	I--S----L--TT------T---		FVQLF------A---C--------
	Brochothrix thermosphacta	WP_120473038	I--S----L--TT------V---		FVQLF------A---C--I-----
	Caryophanon tenue	WP_083998345	----I---V------G---S--D		M-ET-F-----T-AT---------
	Chlamydia trachomatis	CRH87430	I--S--M-L--T-------QI-E		SFQ---E----A--L---F-----
	Clostridiales	WP_120378691	---S--M-L--T-------NF-E		SIQ--------A---------VL-
	Domibacillus aminovorans	WP_063966910	I--S--M-L--T-------G--D		-VK---G----A--L--L---VL-
	Domibacillus enclensis	WP_045852471	I--S----L--T---D---S--G		-VQ---G----A--L---------
	Domibacillus indicus	WP_046173682	I--S----L--T---D---G--G		-VQ---G----A----------I-
	Domibacillus mangrovi	WP_073709964	I--S--M-L--T---D---G--D		-VE---G----A--L--L---VL-
	Domibacillus robiginosus	WP_050180682	I--S----L--T---D---G--S		-IQ---G----A------------
	Domibacillus tundrae	WP_046180543	I--S----L--T---D---G--G		-IQ---G----A------------
	Edaphobacillus lindanitolerans	WP_076757589	----I---L------D--Q---S		-VRL-------A------------
	Fictibacillus	WP_082635840	I--S--I-L--T-E-D---K--Q		-IQ---D----A--L------L--
	Fictibacillus enclensis	KSU83338	I--S--I-L--T-E-D---K--Q		-IQ---D----A--L------L--
	Fictibacillus solisalsi	SDM96593	I--S----L--T-E-D---K--Q		-IQ---D----A--L------L--
	Fictibacillus sp. FJAT-27399	WP_062233046	I--S----L--T-E-D---K--Q		AVR---D----A--L------L--
	Filibacter sp. TB-66	WP_124070718	I--SI---L------D---Q--D		-FQV-------A--L---S---I-
	Geobacillus	WP_011231045	I--S--V-L--TA--N---E--T		--R---DS---A--M---I--A--
	Geobacillus sp. BCO2	KPC98245	I--S--V-L--TA--N---E--T		--R---DS---A--M---I--A--
	Geobacillus stearothermophilus	ATA59867	I--S--I-L--TA--N---E--A		--R---DS---A-GL---L--A--

Other Bacteria (0/>200)

Gracilibacillus orientalis	WP_091479649	---S--M-L--T----I--E--E	-IT---ES---I--L---C---I-
Halobacillus aidingensis	WP_089654823	---S--M-L--T-------K--E	SIQ--------A------------
Halobacillus dabanensis	WP_075037871	---S--M-L--T-------K--D	SVQ--------A------A-----
Halobacillus hunanensis	WP_079526010	---S--M-L--T-------Q--Q	SIQ--------A---------V--
Halobacillus karajensis	WP_074733065	---S--M-L--T-------K--S	SIQ--------A------------
Halobacillus kuroshimensis	WP_027954649	---SI-M-L--T-A-----Q--N	SIQ--------A--L---G-H---
Halobacillus mangrovi	WP_085030205	---S--M-L--T-------K--E	SFQ--------A--L-------L-
Halobacillus trueperi	WP_115825074	---S--M-L--T-------K--E	SIQ--------A--I---------
Kurthia massiliensis	WP_010290947	---SL-M-L--T-L--I-KV--E	SVQL-------A--L---------
Lentibacillus amyloliquefacien	WP_068448197	---S--M-M--TA------QF-S	-VQ--------A------T-----
Lentibacillus halodurans	WP_090233870	---S--M-M--T------KQF-S	-IQ--------A------------
Lentibacillus jeotgali	WP_029329024	---S--M-M--TA------QF-S	-VQ--------A------------
Lentibacillus persicus	WP_090080621	---S--M-M--TA------QF-S	-VQ--------A------------
Lentibacillus salicampi	WP_135108009	---SA-M-M--TA------QF-S	-IQ--------A------A-----
Lentibacillus sp. Marseille-P4	WP_106494717	I--S--I-L--T-A-D--SS--A	SIQ--------A--I---T---I-
Lentibacillus sp. SSKP1-9	WP_129674739	---S--M-L--T------QQF-S	-IQ--------A------------
Lysinibacillus composti	WP_124766740	-IS----A--AF--NI--V--P	S-S---------------------
Lysinibacillus endophyticus	WP_121213652	-I-----A--VF--DI--V--E	-IS---------------------
Lysinibacillus jejuensis	WP_108307384	-I-I-M-V----------N--A	-IS--V-----A----------L-
Lysinibacillus massiliensis	WP_036178422	-I-----A--VF---I--V--E	--SV------------------L-
Lysinibacillus saudimassiliens	CEA01307	-I-I-M-V----------N--D	R-S--V-----A----------L-
Lysinibacillus sp. BF-4	WP_036146501	-I-I-M-V----------N--D	R-S--V-----A----------L-
Lysinibacillus sphaericus	WP_125102278	---S--I-L----------K--E	-VQL-------A--I---T---I-
Mycobacteroides abscessus subs	SHR99649	I--S--I-L--TA--D---QM-S	SIR---D----A--I---L-----
Oceanobacillus	WP_017796201	I--S--M-L--T-------QI-N	S-Q--------A----------I-
Oceanobacillus arenosus	WP_115773300	I--S--M-L--T-------Q--L	SIQ--------A--IM--I---I-
Oceanobacillus bengalensis	WP_121127729	I--S--M-L--T-------Q--N	SVQ--------A----------IY
Oceanobacillus caeni	KPH79250	I--S--M-L--T------SV--A	S-Q--------A---I------I-
Oceanobacillus chungangensis	WP_115749160	I--S--M-L--T------VQ--Q	SIQ--------A------T-----
Oceanobacillus halophilus	WP_121203986	I--S--M-L--T-------T--S	SVQ--------A------------
Oceanobacillus iheyensis	WP_106896173	I--S--M-L--T-------Q--N	S-Q--------A----------I-
Oceanobacillus jeddahense	WP_040982108	I--S--M-L--T------EI--N	S-Q--------A----------I-
Oceanobacillus massiliensis	WP_010650245	I--S--M-L--T----I--Q--S	-IQ--------A------T-----
Oceanobacillus oncorhynchi	WP_042533724	I--SI-M-L--T------DV--N	S-Q--------A----------I-
Oceanobacillus picturae	WP_058950789	I--S--M-L--T-------Q--N	S-Q--------A----------I-
Oceanobacillus profundus	WP_118888647	I--S--M-L--T-------Q--S	SIQ--------A------A-----
Oceanobacillus senegalensis	WP_085992862	---S--M-L--T-------I--S	-VQ--------A------------
Oceanobacillus sojae	WP_077603388	I--S--M-L--T------DI--S	S-Q--------A----------I-
Oceanobacillus timonensis	WP_080874005	I--SI-M-L--T---D---V--N	S-Q--------A----------I-
Ornithinibacillus californiens	WP_047982817	---S--M-L--T-------Q--N	SVQ--------A--I---------
Ornithinibacillus scapharcae	WP_010093911	---S--M-L--T-------Q--T	SVQ--------A--I---------
Paenibacillus anaericanus	WP_127190059	I--S--M-L--T----V--Q--D	K-R--VD----A--F---I--LL-
Paenibacillus crassostreae	WP_068659868	I--S--I-L--T----V--Q--D	S-R--VD----A------LM-LI-
Paenibacillus pini	WP_036651108	I--S--M-L---------DV--D	-AR--VD----A---------LL-
Paenibacillus rhizosphaerae	WP_076172870	I--S--M-L------D--K-I-E	D-R--VD----A---------LM-
Paenibacillus sp. 7884-2	WP_095309865	I--S--M-L--T-------Q--S	SIQ--------A------A-----
Paenibacillus sp. VMFN-D1	WP_116232724	I--S--M-L------D--K-I-E	E-R--VD----A---------LM-
Paenibacillus sp. XY044	WP_094599670	I--S--M-L------D--K-I-E	DFR--VD----A------I--LM-
Paenibacillus terrigena	WP_018754791	I--S--M-L--T---N--H---G	EWK---E----A--F---I---L-
Paenibacillus tianmuensis	SCW80969	I--S--M-L--T---T--NQ--Q	AVQ--------A--F------L--
Paenibacillus vortex V453	EFU42757	I--S--M-L--TT--D---N--D	SIR--AD----A--L------LI-
Paenisporosarcina indica	WP_075617735	I--SI-M-L--T----I--Q--S	SFQ--------A------I-----
Paenisporosarcina quisquiliaru	WP_090568324	I--SI-I-L--T------DS--T	SMK--------A------------
Paucisalibacillus globulus	WP_096273555	---S--M-L--T-------Q--N	-IQ--------A--L---A---I-
Paucisalibacillus sp. EB02	WP_042149477	---S--M-L--T-------Q--N	-VQ--------A--I---A-----
Planococcus antarcticus	WP_040852826	I--S--I-L--T------VQ--S	SIQ--------A----------L-
Planococcus antarcticus DSM 14	EIM05567	I--S--I-L--T------VQ--S	SIQ--------A----------L-
Planococcus citreus	WP_121300781	---S--I-L--T-------L--A	-VQ--------A--L---A-----
Planococcus faecalis	WP_078080808	I--S--I-L--T------IQ--S	SVQ--------A----------L-
Planococcus halocryophilus	WP_040851133	I--S--I-L--T---D--LQ--S	SVQ--------A------A---L-
Planococcus halocryophilus Or1	EMF45597	I--S--I-L--T---D--LQ--S	SVQ--------A------A---L-
Planococcus halotolerans	WP_112224230	---S--I-L--T----I--LV-P	SFQ--------A--I---------
Planococcus kocurii	WP_058386982	I--S--I-L--T------IQ--S	SVQ--------A----------L-
Planococcus maitriensis	WP_112232869	---S--I-L--T-------L--A	-VQ--------A--L---G--V--
Planococcus maritimus	WP_068488720	---S----L--T-------L--A	-VQ--------A--L---G--V--
Planococcus massiliensis	WP_052653538	I--S----L--T----I--Q--S	S-Q--------A----------L-
Planococcus plakortidis	WP_068871678	---S--I-L--T-------L--A	-VQ--------A--L---G-----
Planococcus rifietoensis	WP_058381990	---S--I-L--T-------L--A	-VQ--------A--L---G-----
Planococcus salinus	WP_123166595	---S--I-L--T-------L--S	SIQ--------A------A--F--
Planococcus sp. CAU13	WP_033543617	---S--I-L--T----I--MV-P	SFQ--------A------------
Planococcus sp. PAMC 21323	WP_038702239	I--S--I-L--T------VQ--S	SIQ--------A----------L-
Planococcus sp. Y42	WP_077587781	I--SI-I-L--T------T---S	-VQ--------A------A-----
Planococcus versutus	ANU26611	I--S--I-L--T------VQ--S	SIQ--------A----------L-
Planomicrobium flavidum	WP_088009270	I--S--I-L--T------T---S	-VQ--------A--A---A-----

Other Bacteria (0/>200)

Planomicrobium glaciei	WP_036810764	---S--I-L--T----I--Q--A	--Q--------A------A---L-
Planomicrobium okeanokoites	WP_117313531	---S--I-L--T----I--MV-P	SFQ--------A------A-----
Planomicrobium soli	WP_106533149	I--S----L--T-------Q--S	SIQ--------A------------
Planomicrobium sp. MB-3u-38	WP_101802508	---S--I-L--T----I--LV-P	SFQ--------A------A-----
Planomicrobium sp. Y74	WP_121634991	---S--I-L--T----I--LV-P	SFQ--------A--I---------
Pontibacillus marinus	WP_027447065	I--S--M-L--T-A-D---Q---	QVQ---D----A--I---L--L--
Psychrobacillus insolitus	WP_111440276	I--SI-M-L--T---D--LS--E	SFQ--------A--L-------I-
Psychrobacillus psychrodurans	WP_093495104	I--SI-I-L--T------DS--T	SMK--------A------------
Psychrobacillus psychrotoleran	WP_093538127	I--SI-I-L--T-------E--T	SMK--------A--I---------
Psychrobacillus sp. OK028	WP_093062776	I--SI-I-L--T------DV--S	SMK--------A--M---I--I-
Psychrobacillus sp. OK032	WP_093276007	I--SI-M-L--T---D--IS--N	SIR--------A----------I-
Quasibacillus thermotolerans	WP_039237813	I--S--M-L--T---D---S--S	--Q--------A--M---I-----
Rhodococcus qingshengii	WP_133371566	I--S--M-L--TA--D---Q--A	SVK---N----A--L---F-----
Rummeliibacillus pycnus	WP_102693295	I--SI-M-L--T---DI-KV--E	SVR--------A----------I-
Rummeliibacillus sp. POC4	WP_119416962	I--SI-M-L--T---DI-KV--E	TVR--------A------A---I-
Rummeliibacillus sp. TYF005	WP_124218587	I--SI-M-L--T---DI-KV--E	TVR--------A------A---I-
Rummeliibacillus stabekisii	WP_066791425	------M-L--T---QI-DI--E	SVR--------A------AM----
Saccharibacillus sacchari	WP_037284968	I--S--M-L------G---G--E	E-R--VD---IA--F---IM-LL-
Salimicrobium album	WP_093107146	---SI-M-L--TT-----RS--E	-IQ--------A--L---------
Salimicrobium flavidum	WP_076557536	---SI-M-L--TA-----T---S	-VQ--------A--M---I-----
Salimicrobium halophilum	WP_093193019	---S--M-L--TA------S--E	-VQ--------A--L---------
Salimicrobium jeotgali	WP_008586869	---SI-M-L--TT-----RS--E	-IQ--------A--L---------
Salimicrobium salexigens	WP_076570708	---SI-M-L--TT-----RS--E	-IQ--------A--L---------
Salinibacillus kushneri	WP_093134113	---S--M-L--T------SR--E	SVQ--------A----------I-
Scopulibacillus darangshiensis	WP_132747290	I--SI---L--T-Q-D---K--I	-VQ-I-N----A--M---I--LI-
Sediminibacillus albus	WP_093212618	------M-L--TA---I--N--E	SIR---DS---A--M-------L-
Sediminibacillus massiliensis	WP_077624304	I--S--M-L--T----I--Q--E	SIR---ES---A--F---L---L-
Sporosarcina globispora	WP_053434641	I--S--M-L-ITA------QM-S	SVR---D----A------L-----
Sporosarcina koreensis	WP_040286776	I--S--I-L----------K--A	--QL-------A------S---M-
Sporosarcina newyorkensis	WP_078817704	---SI-I-L------D---K--D	SVQ--------L--L---F---I-
Sporosarcina pasteurii	WP_115360366	I--SI-I-L--T-A-D---H---	S-QMF------A--L---A-----
Sporosarcina psychrophila	AMQ08565	I---I-I-L--T-------QF-S	D-K--------A----------L-
Sporosarcina sp. BI001-red	WP_116016531	I--S--I-L----------K--V	SIQL-------A--IM--T---I-
Sporosarcina sp. D27	WP_025784951	---S--I-L----------K--E	-VQL-------A--I---T---I-
Sporosarcina sp. HY008	WP_067406110	I---I-M-L--T---D---G--A	--Q--------A--I------MM-
Sporosarcina sp. PTS2304	WP_114923437	---SI-I-L------D--SN--E	TIQ--------L--I---L---I-
Sporosarcina sp. ZBG7A	WP_039041819	---S--I-L----------K--E	-VQL-------A--I---A---I-
Sporosarcina ureae	WP_083035543	---SI-I-L------D--KE--E	SVQ--------L--I---F---I-
Thalassobacillus cyri	WP_093046939	---S--M-L--T-------Q--S	-IQ--------A------------
Thalassobacillus devorans	WP_028782445	---S--M-L--T-------Q--S	-IQ--------A------------
Thalassobacillus sp. TM-1	WP_062445290	---S--M-L--T-------Q--S	-VQ--------A------------
Thermolongibacillus altinsuens	WP_132948301	I--SI-M-L--T---D---KM-A	SIR---D----A---------VF-
Tuberibacillus sp. Marseille-P	WP_085521570	I--S----L--T-Q-D---Q--N	SVQ-I-N----A--I---L---I-
Virgibacillus chiguensis	WP_073005952	---S--M-L--T-------KF-E	SIQ--------A----------L-
Virgibacillus dokdonensis	WP_077702966	---S--M-L--T-------KF-E	SIQ--------A----------L-
Virgibacillus halodenitrifican	WP_019377088	I--S--M-L--T------SHF-N	SVQ--------A------T---L-
Virgibacillus indicus	WP_094886490	I--S--M-L--T------SQF-S	SVQ--------A------A-----
Virgibacillus ndiopensis	WP_099159954	I--S--M-L--T-------QF-S	SIQ--------A------------
Virgibacillus necropolis	WP_089534008	I--S--I-L--T-A-D---N--S	SFQ--------A--I---S---L-
Virgibacillus pantothenticus	WP_077297084	---S--M-L--T-------NF-E	SIQ--------A---------VL-
Virgibacillus phasianinus	WP_089063092	I--S--I-L--T-A-D---N--S	AIQ--------A--I---S---L-
Virgibacillus proomii	WP_077317922	I--S--M-L--T----I--K--N	SIQ--------A------F--ML-
Virgibacillus siamensis	WP_077325143	I--ST-I-L--T-A-----Q--A	-FQ--------A--L---A-----
Virgibacillus soli	WP_057985152	I--S--I-L--T-A-D------T	--Q--------A--I---I---L-
Virgibacillus sp. SK37	WP_040954522	I--S--M-L--T------SHF-T	SVQ--------A------T---L-
Viridibacillus arenosi FSL R5-	ETT87427	I---I-M-L--T---DI-KV--S	SVQ--------A------L---I-
Viridibacillus arvi	WP_053418336	I---I-M-L--T---DI-KV--D	SVQ--------A----------I-
Viridibacillus sp. OK051	WP_100795069	I---I-M-L--T---DI-KI--P	-VQ--------A--I---------

Supplemental Figure 52

A partial sequence alignment of the purine permease protein containing a two amino acid insertion (boxed) that is exclusively shared by all members belonging to the Meyeri clade and absent in all other bacteria.

Group	Species	Accession	123		154
**Meyeri Clade (3/3)**	Bacillus ndiopicus	WP_042477869	FPVVIDKTRSVQKAYNVG	A	ALPATVLVTPEGK
	Lysinibacillus meyeri	WP_107841855	------------R-----	V	-------------
	Lysinibacillus fluoroglycofenilyticus	WP_066171039	------------------	-	-----I----D--
**Other Bacteria (0/>100)**	Aliicoccus persicus	WP_091476527	--IAL-RN-D-T---Q--		P--T-IAINK---
	Anoxybacillus flavithermus	WP_088223244	------QQDQ-MN---I-		P----F-IDK---
	Anoxybacillus pushchinoensis	WP_091700493	------QQDQ-MN---I-		P----F-IDK---
	Anoxybacillus suryakundensis	WP_055440692	------QQDQ-MN--HI-		P--T-F-INKD--
	Bacillus acanthi	WP_108672299	---L---KSQ--N--K-N		P--V-F-IDK---
	Bacillus acidiproducens	WP_018660492	---L----GD--T---ID		P--T---IG-D--
	Bacillus altitudinis	WP_096881585	---A---D-Q-TE--DIT		P--T-F-IN----
	Bacillus andreraoultii	WP_033828512	--I-V-NGVQKAYGI YP		----F-IS----
	Bacillus aquimaris	WP_064090984	--ILV--EDQ--H--GID		P--T-L-IN---E
	Bacillus asahii	WP_119115384	---M---GQE--V--R-D		P--V-F-IDK---
	Bacillus atrophaeus	WP_010789090	----L-SD-Q-LN--G-S		PI-T-F-IN----
	Bacillus australimaris	WP_060697213	---AM--D-Q-TE--DIT		P--T-F-IN----
	Bacillus badius	WP_063440279	--IA--S-KE--D---IT		P----FMIS-D--
	Bacillus camelliae	WP_101353323	--IL---D-D--SV-G-D		D--HS--IG-D--
	Bacillus cecembensis	WP_057982160	---A----K--FT---ID		P--TSIFIK-D--
	Bacillus cihuensis	WP_028391592	--IM---NKQ--T--K-D		P--V-F-IDKN--
	Bacillus clausii	WP_063609356	---A--ER-E-TR--GI-		P----I--DEH--
	Bacillus coagulans	WP_014098122	---L---NGD--H--HID		P--T-F-IG-D--
	Bacillus deserti	WP_101644954	---L---KSE--N--K-D		P--V-F-INK---
	Bacillus firmus	WP_061792440	--I----DSQ--S--GIN		P----F-IDKD--
	Bacillus fordii	WP_018708928	--ILK--NKDIMNM-G-F		N----L-------
	Bacillus ginsengihumi	WP_025729027	---L--RNKD--H---IA		P--T-I-IG-D-R
	Bacillus glycinifermentans	WP_096892169	----L--D-Q-LD--D-T		P--T-F-IN-D--
	Bacillus gobiensis	ALC81454	-------D-Q-LE--D-N		P--T-F-IS----
	Bacillus halotolerans	WP_044154915	----L-TD-Q-LD--G-S		P--T-F-IN----
	Bacillus haynesii	WP_094777438	----L--D-Q-LN--D-T		P--T-F-IN-D-E
	Bacillus hemicellulosilyticus	WP_035340074	--I----G---TN--G-H		P--T-I-INEH--
	Bacillus hisashii	WP_095141196	--I-V-D-GE-----GIY		P----F-IS-D--
	Bacillus horneckiae	WP_066395608	--ILL--DGQ--T--KI-		-----Y-IDK---
	Bacillus infantis	WP_129612014	--I-T--DGQ--N--AID		P----F-IN---E
	Bacillus kwashiorkori	WP_062356206	--IIV-EDGE--H--G-L		P----F-ID----
	Bacillus licheniformis	WP_107661742	----L--D-Q-LN--D-T		P--T-F-IN-D-E
	Bacillus litoralis	WP_066330048	---IL--D-Q-LD--G--		P--T-F-IN---E
	Bacillus marisflavi	WP_048005924	--I-V--EEE--N--GID		P--T-M-IN-D--
	Bacillus nakamurai	WP_061520912	---AL-TD-Q-LD--G-S		P--T-F-IN----
	Bacillus notoginsengisoli	WP_118920581	--I-L--D-Q-MN--G--		Q----F--NK---
	Bacillus oceanisediminis	WP_019380357	--I----NTQ--S--GIN		P----F-IDKD--
	Bacillus oleivorans	WP_097157497	---LR-TKS-EVQRAYNI		-PLP-TILINPEG
	Bacillus paralicheniformis	WP_134996414	----L--D-Q-LN--D-T		P--T-F-IN-D-E
	Bacillus praedii	WP_057764292	--I----DGQ--T--KI-		-----FMIDKD--
	Bacillus safensis	WP_111290505	---AM--D-Q-TE--DI-		Q--T-L-IN----
	Bacillus simplex	WP_076365574	---M---G-E-EN--R-D		I--V-F--DK---
	Bacillus sonorensis	WP_006636455	----L--D-Q-LE--D-T		P--T-F-INS---
	Bacillus stratosphericus	WP_039963986	---AM--D-Q-TE--DIT		P--T-F-IN----
	Bacillus stratosphericus LAMA	EMI12114	---AM--D-Q-TE--DIT		P--T-F-IN----
	Bacillus subtilis	WP_009967646	----L-TD-Q-LD--D-S		P--T-F-IN----
	Bacillus subtilis group	WP_004429691	----L-SD-Q-LN--G-S		PI-T-F-IN----
	Bacillus tequilensis	WP_024715207	----L-TD-Q-LD--D-S		L----F-ID----
	Bacillus terrae	WP_120115120	---LK--SKDIMNM-G-F		N----L--N----
	Bacillus thermoamylovorans	WP_034770729	--I-V-H-GE-----GIY		P----F-IS-D--
	Bacillus thermocopriae	WP_129000465	------QQDQ-MN---I-		P----F-IDK---
	Bacillus vallismortis	WP_010328151	----L-TD-Q-LD--D-S		P--T-F-IN----
	Bacillus vietnamensis	WP_034758241	--ILV--EEE--N--GID		P--T-F-IN-Q-E
	Bacillus weihaiensis	WP_072579345	----L--QSQ-F---G--		K----Y-IDSD--
	Bacillus xiamenensis	WP_008359709	---AM--D-Q-TE--DIT		P--T-F-IN----
	Bacillus zhangzhouensis	WP_034318809	---AM--D-Q-TE--DIT		P--T-F-IN----
	Bhargavaea beijingensis	WP_092095243	--I-----N--RD----R		P--T-F--G-D--
	Bhargavaea cecembensis	WP_063179254	--I-----N--RD----R		P--T-F--G-D--
	Bhargavaea ginsengi	WP_092048773	--I-----K--RD---IR		P--T-L-IG-D--
	Brevibacillus laterosporus	WP_003338476	--ILM--QKE-T----I-		KM-S-FYIDQD--
	Caldibacillus debilis	WP_061568602	----V-REGE--D--DIF		P----F-IDAD--
	Caryophanon tenue	WP_066543575	------T-GD-KQ--SII		P--T---IN----
	Chryseomicrobium excrementi	WP_100352655	------RQKN-MER--I-		P--T-F--D-D--
	Desmospora activa	WP_107726084	--I-L-RN-E-T-Q--I-		PI-SS-FLDK---
	Domibacillus epiphyticus	WP_076767795	--IA---GKE--E--GIN		P--T-FMIR-D--
	Domibacillus robiginosus	WP_050179651	--IP---GKE-------N		P--V-FMIS---E
	Edaphobacillus lindanitolerans	WP_076756435	--I-----N--RD---IR		P--T-F--G-D--
	Falsibacillus pallidus	WP_114746268	----V--NKD--T--GID		P--T-F-IN-K-E
	Fictibacillus arsenicus	WP_066290581	--IPM--Q-E-T---GI-		PI-T-I-IDKN--
	Fictibacillus phosphorivorans	WP_066241966	--IPM--E-E-T---GI-		PI-T-I--DKN--
	Halopolyspora algeriensis	WP_114454329	--S-H-RN-RISA-LQ-P	S	----SYV---S-R

Other Bacteria (0/>100)

Jeotgalibacillus malaysiensis	WP_039806421	------QKKD--N--TI-		P--T-L-IN---E
Jeotgalibacillus soli	WP_041090143	------TQKD--N--TI-		P--T-L-IN---E
Jeotgalicoccus halophilus	WP_092594642	Y-IAL--N---T----I-		P--T-IAINK---
Jeotgalicoccus halotolerans	WP_115884326	Y-IAL-SN---T----I-		P--T--A-NK---
Jeotgalicoccus marinus	WP_026866345	Y-IAL-PN---T----I-		P--T-IAINKD-E
Jeotgalicoccus nanhaiensis	WP_135096805	Y-IAL-PN---T----I-		P--T--A-NK---
Kurthia sibirica	WP_109304638	--MT----K--MRT---D		Q----F-IDQT--
Kurthia zopfii	WP_109348675	--MT--RSK--MR----D		Q----F-INK---
Lysinibacillus acetophenoni	WP_097149032	--T------D-MT----I		D---SIFID-N--
Lysinibacillus boronitolerans	WP_036079193	---L---NK--ME---IN		P--T---INS---
Lysinibacillus chungkukjangi	WP_107937868	---L---NK--MLV--IK		P--T-I--N----
Lysinibacillus composti	WP_124765127	--------K--MT---IN		P--T-M-IS----
Lysinibacillus contaminans	WP_053584137	--------KD-MTT----		N-----MID-D--
Lysinibacillus endophyticus	WP_121215590	---A--E--D-MTE-TID		L----I-IN---E
Lysinibacillus fusiformis	WP_069480468	--------K--MT-----		Q------ID----
Lysinibacillus halotolerans	WP_122972874	---A--P-K--MT----D		L----I-IN---E
Lysinibacillus jejuensis	WP_108306724	--------KG-RN---II		G----I--S----
Lysinibacillus macroides	WP_053994129	--------K--MT-----		Q------ID-D--
Lysinibacillus manganicus	WP_036183876	--T------D-MT----I		D---SIFIN-D--
Lysinibacillus mangiferihumi	WP_107895152	--------K--MT----D		N------ID-D--
Lysinibacillus massiliensis	WP_036178552	---L---SK--MGT--IN		P--T---IN----
Lysinibacillus odysseyi	WP_036154280	---A--Q-K--ME----T		N----I-INK---
Lysinibacillus parviboronicapi	WP_107949731	--------K--MT-----		N-----MID----
Lysinibacillus saudimassiliens	CEA04730	--------KG-RN---II		G----I--S----
Lysinibacillus sinduriensis	WP_036203533	--A---Q-KA-MT----N		P--T-I-IN---R
Lysinibacillus sphaericus	WP_012293419	--------K--MT-----		Q------ID----
Lysinibacillus telephonicus	WP_126293464	-------NK--ME---IN		P--T-F--S----
Lysinibacillus xylanilyticus	WP_068983000	--------K--MT-----		Q----L-ID-D--
Lysinibacillus xyleni	WP_097074687	---L---SK--MS---IK		P--T----N----
Marininema halotolerans	WP_091834540	----L-RD-V-T-R-D--		R----Y-IDRN--
Marininema mesophilum	WP_091734838	--I-L-QD-V-T-R-D--		R----Y-ISR---
Melghirimyces profundicolus	WP_108021819	---LL-DG--TYD-FGIT		SV--S--ID---R
Melghirimyces thermohalophilus	WP_091567321	---LL-HG--TYD--EIS		SV-TS--IN---R
Mycolicibacterium novocastrens	WP_067387740	--S-Y-PDK---R-LG-P	P	V--VNY----D-R
Paenibacillus etheri	WP_082651088	------V-GKIST--G-S		-----FIING---
Paenisporosarcina antarctica	WP_134210004	--I-----K--ME---IN		P--T-L-IN-D--
Paenisporosarcina indica	WP_075617643	--------K--MD---IN		P--T-M-IN-Q--
Paenisporosarcina quisquiliaru	SEM10056	--IA--RNK--MES--ID		P--T-F-IN----
Planifilum fimeticola	WP_106344381	--ILL-QN-E-TQL-GI-		PI-SSIF-S----
Planifilum fulgidum	WP_092035635	--ILL-PD-D-TRL-GI-		PI-SSIF-S----
Planococcus antarcticus	WP_006829833	--------K--MT---IR		P--T-I--N----
Planococcus donghaensis	WP_008430978	--------K--MT---IR		P--T----N---N
Planococcus halocryophilus	WP_008497817	--------K--MT---IR		P--T----N---N
Planococcus halotolerans	WP_112222891	--I----NK--ME---IR		P--T-I--N-D-E
Planococcus maritimus	WP_068487686	--I----DK--ME---IR		P--T-L--N----
Planococcus massiliensis	WP_052654461	--I-----K--MT---IR		P--T---IN---E
Planococcus plakortidis	WP_068870850	--I----DK--ME---IR		P--T-L--NQ---
Planococcus rifietoensis	WP_058380534	--I---RDK--MT---IR		P--T-M--N-Q-E
Planococcus salinarum	TAA72516	-------SK--MT---IR		P--T-I--N---E
Planococcus salinus	WP_123164230	-------SK--MS---IN		P--T----N----
Planococcus versutus	WP_049693190	--I-----K--MT---IR		P--T-I--NS--E
Planomicrobium flavidum	WP_088007480	--IA--EAK--ME--S-V		P--T---IS-D--
Planomicrobium glaciei	WP_074509433	--------K--MT---II		P--T----N---E
Planomicrobium glaciei CHR43	ETP69670	--------K--MT---II		P--T----N---E
Planomicrobium soli	WP_106531806	--------K--MT---II		P--T----N-D-E
Pontibacillus litoralis	WP_084600238	----Q-T-SE-MQL-D--		----SYF-G-D--
Proteobacteria bacterium	PIE20229	----V-EDTAIAQ---PT	-	-A-F---IGRD--
Psychrobacillus insolitus	WP_111439683	---L--NNK--MET--IN		P--T-F--N----
Psychrobacillus psychrodurans	WP_093493721	---L--DKK--MET-KIN		P--T-L-IN-N--
Psychrobacillus psychrotoleran	SFQ12806	--IA--RNK--MES--ID		P--T-F--N----
Quasibacillus thermotolerans	WP_083965760	--IA--SSKE-----G-N		P----FMIS----
Rummeliibacillus pycnus	WP_102692550	--ILL---K--MRV---D		P--T----DK---
Rummeliibacillus stabekisii	WP_066791844	--I--------MR----D		P----I-INKD--
Salinicoccus albus	WP_020008362	--IAL-SS---TE---I-		P--T-IAIDK---
Salinicoccus halodurans	WP_046791486	--IAL----D-TE--SI-		P--T-IA-NQ---
Salinicoccus luteus	WP_031544893	--IAL-RSK--TD---I-		P--T-IA-NK---
Salinicoccus qingdaonensis	WP_092985850	--IAL-RN-D-T---Q--		P--T-IAINKD--
Salinicoccus roseus	WP_094905560	--IAL-RSK--TE---I-		P--T-IA-NK---
Salinicoccus sediminis	WP_046514921	--IAL-REK--TD---I-		P--T-IA-NK---
Solibacillus isronensis	WP_079524103	---L--RY---MG---IK		P--T-I-INS---
Solibacillus silvestris	WP_065216983	---A----K--FT---I-		P--TSIFIK-N--
Sporosarcina globispora	WP_053434531	--I----DSQ--S--GIN		P----F-IDKD--
Sporosarcina koreensis	WP_060209963	--TL----KT-MQT---K		P--T-F-IN----
Sporosarcina newyorkensis 2681	EGQ19671	--TL----KG-MQS--IK		P--T-L-IN----

**Other Bacteria (0/>100)**	Sporosarcina pasteurii	WP_115364068	--TL---NKG-MQ---IK	P--T-L-ID----
	Sporosarcina psychrophila	WP_067210457	--IA---DK--KEV--IV	P--T-F-INKD--
	Sporosarcina ureae	ARF18834	--TL--T-K--MQT---K	P--T-L-IN-D--
	Tetzosporium hominis	WP_094941735	------RQKN-MEK--I-	P--T-F--D-D--
	Thermolongibacillus altinsuens	WP_132947018	-------QDQ-MN--DI-	P--T-F-IDKN--
	Thiohalobacter thiocyanaticus	WP_096364770	---LF-RDS--AE--G-K	G--T---ID-Q-R
	Ureibacillus thermophilus	QBK25814	--------K--MQ-----	Q----F-IS-D--
	Ureibacillus thermosphaericus	WP_096550776	--------K--MQ-----	K----Y-I-----
	Viridibacillus arvi	WP_053417369	--------K--TR----N	N---SIFID-T--

Supplemental Figure 53

A partial sequence alignment of the thiol-disulfide oxidoreductase ResA protein containing a one amino acid insertion (boxed) that is exclusively shared by all members belonging to the Meyeri clade and absent in all other bacteria.

Group	Organism	Accession	176		213
**Solibacillus Clade (7/7)**	Solibacillus sp. R5-41	WP_099422418	GQAQTFQLGITALQKPQVME	Q	IQGGTYIGLPNNLDELG
	Solibacillus silvestris	WP_065216607	-TPLK-----SE----N---	K	L---------E---A--
	Solibacillus kalamii	WP_087615658	-TPLK-----SE----N---	K	L---------E---A--
	Solibacillus isronensis	WP_079524658	-TPLK-----SE----N---	K	L---------E---A--
	Bacillus cecembensis	WP_057986779	-PV-------ST--------	-	--------F----A---
	Lysinibacillus sp. 2017	WP_108711546	--Q-N------S--------	S	LN--------Q------
	Lysinibacillus odysseyi	WP_036150777	--VIQ-D-AVSQIT------	-	LN----LS--S--A---
**Other Bacteria (0/>100)**	Lysinibacillus meyeri	WP_107839955	-DVI-VD-AV-E-----T--		HVS-------T--AA--
	Lysinibacillus fluoroglycofeni	WP_107942047	-DVIAVD-AVSE-----A--		QIS-------T--AA--
	Bacillus ndiopicus	WP_042470929	-DVM-VD-AV-R--------		QIS-------T-VAA--
	Bacillaceae bacterium EAG3	WP_104848566	NSEE-ID-A-ISVE---FL-		KVSDNTFT----MAQ--
	Bacillus aquimaris	WP_044339972	EAN-Q-N---VTVE---FLQ		QF--NLL---V---G--
	Bacillus boroniphilus JCM 2173	GAE46682	-IS-EVN--VILIN---FL-		QK-ANLL---D--A---
	Bacillus butanolivorans	WP_053348508	FGEKS-G--VVSVK---FLD		QL--NLL---D-MRG--
	Bacillus canaveralius	WP_101576029	-TT-A-N--VILAN---FL-		QK--NLL---E-MGQ--
	Bacillus cavernae	WP_126863643	-NR-I-N---ISVK---LL-		QI-ENLL---A-F-Q--
	Bacillus dakarensis	WP_077215029	-TD-I-DI-VVQVN---FLT		AN-NNLY---D--N---
	Bacillus deserti	WP_101639774	-GV---E---ISVK---FF-		QK-TNLYA--ER-NT--
	Bacillus foraminis	WP_121610607	-SS-QRN--VVRVD---FL-		QK-HNLY---G--G---
	Bacillus gottheilii	WP_066442371	-GTASYD---SYVN---FL-		QK--NLL---E-MAD--
	Bacillus horneckiae	WP_066398183	-TTRE-N--VVQVN---FL-		QK--NLL---E-IAD--
	Bacillus jeotgali	WP_079506442	-TS-D-N--VVLVE---FL-		QK-ANLL---E--A---
	Bacillus korlensis	WP_066057951	-ATEV-N--VVRVN---FL-		QR--NLL---A-VA---
	Bacillus kribbensis	WP_026696023	RGT---D---VSV----FL-		QK-ENMLA-----NS--
	Bacillus kwashiorkori	WP_062351648	NEE-------VQIE---LL-		LA-DSMLK---HF----
	Bacillus loiseleuriae	WP_049683302	RGT---E---VSVK---F--		QL--NLLA--A--NQM-
	Bacillus massilioanorexius	WP_019243159	-AS-S-N---ISVKN--YL-		HKQ-NIV--AE-YAD--
	Bacillus massiliogabonensis	WP_102273643	-GTSE-N--VVQ-N---FL-		QK--NLL---E-MA---
	Bacillus massiliogorillae	WP_042350965	-TT-S----MVSV-N--YL-		HKQ-NVV--SE-AAQ--
	Bacillus massilionigeriensis	WP_075983153	-RV--YN--VVQVN---FL-		QK-NNLF---Y-YA---
	Bacillus massiliosenegalensis	WP_019153926	-AT-I-N--VVQVD---FLV		AK-NNLF---P-F----
	Bacillus mediterraneensis	WP_071460946	-ST-SAN--VVLVK---LL-		QK--NLF---D-FAG--
	Bacillus methanolicus	WP_003346760	-TR--IN--VSLVK---FLV		QK-DNLL---E-FA---
	Bacillus muralis	WP_057911934	YGSES-G---VSVK---FLD		RL--NLL---E-INN--
	Bacillus oleivorans	WP_097156855	-D-WNLGVIQINRPQAL-Q		---NR-A--D--A---
	Bacillus praedii	WP_057760235	-GTSE-N--VVQVN---FL-		QK--NLL---E-MA---
	Bacillus selenatarsenatis	WP_041964564	-TS-EVN--VILIN---FL-		QK--NLL---D--A---
	Bacillus simplex	WP_034315759	YGEKS-G---VSVK---FLD		RL--NLL---K-FNA--
	Bacillus sp. 17376	ESU34330	-IS-EVN--VILIN---FL-		QK-ANLL---D--A---
	Bacillus sp. AFS015802	WP_098351527	-GEER-N--VVTVE-A-FL-		QF--NLL-FAD------
	Bacillus sp. AFS017274	WP_098371634	YGEKS-G---VSVK-T-FLD		RL--NLL---K-FNA--
	Bacillus sp. AFS026049	WP_098187444	YGEKS-G---VSVK---FLD		RL--NLL---K-FNA--
	Bacillus sp. AFS043905	WP_098864888	YGEKS-G---VSVK---FLD		RL--NLL---K-FNA--
	Bacillus sp. AFS094228	WP_098937360	YGEKS-G---VSVK---FLD		RL--NLL---K-FNA--
	Bacillus sp. BA3	WP_101223814	YGKHS-G---VSVKN--FLD		RL--NLL-M-E-FNS--
	Bacillus sp. FJAT-20673	WP_063574660	YGEKS-G---VSVK---FLD		RL--NLL---K-FNA--
	Bacillus sp. FJAT-22090	WP_053590505	-STVQRE-AVSV-----L--		QLSE---V-----A---
	Bacillus sp. FJAT-25496	WP_057772894	-STSE-N---SLIK---FL-		QK--NLL---E-FE---
	Bacillus sp. FJAT-27916	WP_049669338	NGTE-ID---ISVE---FL-		NVSDNTFT----MAQ--
	Bacillus sp. FJAT-27986	WP_066105706	--SFNLGIISVKKPQFME-I		NNN-FT --K-MAQ--
	Bacillus sp. FJAT-29937	WP_066296891	-STSS-N--VVHIK-S-FL-		QK-ENLL---E-MA---
	Bacillus sp. Leaf13	WP_056524244	FG-KS-G--VVSVK---FLD		QL--NLL---D-MRG--
	Bacillus sp. MUM 13	WP_071352314	-TV-N-D--VVS-K---F--		QI-ENLL-M-A--NR--
	Bacillus sp. OV322	WP_090988631	-TV-N-D--VVS-K---F--		QI-ENLL-M-A--NR--
	Bacillus sp. RJGP41	WP_106027239	YGEKS-G---VSVK---FLD		RL--NLL---K-FNA--
	Bacillus sp. Soil745	WP_057278174	YGEKS-G---VSVK---LLD		RL--NLL---K-FNA--
	Bacillus sp. Soil768D1	WP_057216044	FGEKS-G--VVSVKN--FLD		QL--NLL---D-MRG--
	Bacillus sp. T33-2	WP_101583849	-GT-E-N--V-YIK---FL-		QK--NLF---D--AG--
	Bacillus sp. V3-13	WP_101661800	-TT---D--VILAN---FL-		QK--NLL---E-M-Q--
	Bacillus sp. V44-8	WP_117324366	-AV---N--MISIK---FL-		QI-ENLL---A-F-D--
	Bacillus sp. V59.32b	WP_117308861	DGT---N---VSVK---FL-		QI-ENLL---A-I-Q--
	Bacillus sp. es.034	WP_098438740	-GEER-N--VVTVE-S-FLQ		QY--NLL-FAD------
	Bacillus subterraneus	WP_044396291	-TS-QVN--VAIVN---FL-		QK-ANLL---E--AA--
	Bacillus thermoamylovorans	KIO72721	-NNV--N---AQIH---T-A		RV-DNLMT--D-MA---
	Bacillus vietnamensis	WP_060672843	-GEER-N--VVTVE-S-FLQ		QY--NLL-FAD------
	Bacillus zeae	WP_119113829	-SK-EAN--VVFVK---FL-		QK-DNLL-F-D---A--
	Butyricicoccus sp. 1XD8-22	RKJ59670	--TA--E----EI-------		HVSD---D--E-FA---
	Caldibacillus debilis	OUM84031	-TVREIP---VEVH---LLQ		QI-DNLLS--E-FA---
	Falsibacillus pallidus	WP_114745954	--KVSYN--VI-VN---FL-		QK-QNLF---Q-MNG--
	Falsibacillus sp. GY 10110	WP_121680923	--TES----VISVK---FL-		QK-NNLV---E-MNS--
	Filibacter sp. TB-66	WP_124068815	-TMD-IS--V-LMER-NL-T		HLS-----V-A-MA---
	Jeotgalibacillus alimentarius	WP_041123436	-IQ--VEM--VDI-R--L-D		RVSSV-L-I-D------
	Jeotgalibacillus campisalis	WP_041061694	-TE-QVE---VDI-R--L-D		RVSSV-L---A------
	Jeotgalibacillus malaysiensis	WP_039811496	-AQ--VE---VDI-R--L-D		RVSSV-L-I-D--AA--

Other Bacteria (0/>100)

Jeotgalibacillus proteolyticus	WP_104058571	-TE-QVE--VVNI-R--L--	RVSSI-F-I-E-AE---
Jeotgalibacillus salarius	WP_134379046	-AQ-EVE--VVDI-R--L-D	RVSSI-L---D------
Jeotgalibacillus soli	WP_041087017	-GV-AVE---VNV-R--M--	RVSNE-YSI-D--A---
Jeotgalibacillus sp. R-1-5s-1	WP_134375502	-AQ-EI---VVTIER--L-D	RVSSV-L-I-G--A---
Jeotgalibacillus sp. S-D1	WP_133376816	-NE-QAE---VTI-R--L--	RVSSV-L---D------
Kurthia massiliensis	WP_010287030	-TTEKIG----QIER-DL-V	HRA-N-LII-D-F----
Kurthia senegalensis	WP_010302401	-NKD-IDV-V-EVYRSEMLN	HIS---FTM-S-F----
Lysinibacillus boronitolerans	WP_016991805	-NVIRTD-AV-EF----L--	HVA-S-M------A---
Lysinibacillus chungkukjangi	WP_107932528	-DSL--E----EI-TS----	HISD------A--N---
Lysinibacillus composti	WP_124763349	-EVL-VE----E-----L--	HVK----A--S-M----
Lysinibacillus contaminans	WP_053585280	-DVIRTD-AV-EF----L--	HIS---V------A---
Lysinibacillus endophyticus	WP_121214028	-TT-G-E-AV-RVD---S--	KLS-S---V-G--EQ--
Lysinibacillus fusiformis	WP_004269488	-DIIRTN-AV-EF----L--	HVA-S-M------A---
Lysinibacillus halotolerans	WP_122971480	-ETLN-E--V-E-HT--LL-	HVS----A--A-F----
Lysinibacillus macroides	WP_053993105	-DIIRTE-AV-EF----L--	HIT-S-----A--A---
Lysinibacillus manganicus	WP_036182271	-ETLSVE----E-----A--	HISD----I-E-FA---
Lysinibacillus mangiferihumi	WP_107897830	-DVIRTD-AV-EF----L--	HIS---V---D--A---
Lysinibacillus massiliensis	WP_036171328	--TA--E----EI-------	HVSD---D--E-FA---
Lysinibacillus parviboronicapiens	WP_107923650	-NIIRTD-AV-E-----L--	HIS----------A---
Lysinibacillus sinduriensis	WP_036200119	-DVL--E----EV-T-----	HVSD---A--A--G---
Lysinibacillus sp. B2A1	AVK82706	-DIIRTD-AV-EF----L--	HIS-Q-V------AQ--
Lysinibacillus sp. FJAT-14222	WP_053596432	-DIIRTD-AV-EF----L--	HIS-S-V------AQ--
Lysinibacillus sp. FJAT-14745	WP_053484431	-DVIRTDVAV-EF----L--	KVS-A-V------AQ--
Lysinibacillus sp. Marseille-P	WP_106783554	-TT-A-E----E------LQ	HVS---FD--T-FA---
Lysinibacillus sp. OL1	WP_131520799	-DIIRTD-AV-EF--T-L--	HVA-S-ME-----A---
Lysinibacillus sp. SYSU K30002	WP_126658924	-TSLN-E-AV-QI----S--	QLS----A--T------
Lysinibacillus sp. YR326	WP_134020541	-DVIRTDVAV-EF----L--	KLS-S-V---D--AQ--
Lysinibacillus sp. ZYM-1	WP_054611271	-DIIRTD-AV-EF----L--	HVS-S-V---DD-A---
Lysinibacillus sphaericus	WP_010858007	-NIIRTDIAV-E-----L--	HIS----------A---
Lysinibacillus telephonicus	WP_126295540	-ETLS-E--V-E--------	HVS----A--A--N---
Lysinibacillus xyleni	WP_097071700	-TT-G-E-AV-QVE---T--	RLS----AV-T------
Paenisporosarcina quisquiliarum	WP_090565596	-TT-QRE-AVSV----NA--	HLSA---AM-D--E---
Paenisporosarcina sp. K2R23-3	WP_119882590	-TTDQ-D----V--R--L--	HLSA---T--D--N---
Planomicrobium flavidum	WP_088009553	-TE-AIP--VVEV-R--L--	-L-------DT -A--
Psychrobacillus psychrodurans	WP_093493850	-ST-QRE-AVSV----NA--	HLSA---AM-D--E---
Psychrobacillus psychrotoleran	WP_093538701	-TT-QRE-AVSV----NA--	QLS----A-----E---
Psychrobacillus sp. FJAT-21963	WP_056828035	-STVQRE-AVSV-----L--	QLSE---A-----A---
Psychrobacillus sp. OK028	WP_093061298	-TT-QRE-AVSV----NA-V	HLSA---AM----EQ--
Psychrobacillus sp. OK032	WP_093267920	-TTVQRE-AVSV-----L--	HISS---SM-T------
Rummeliibacillus pycnus	WP_102693078	NTNAKIEI-V-EI----L--	HISD--------FNQ--
Rummeliibacillus stabekisii	WP_066791760	RSVKIE--V-QI---EL--	HIS-------E-FG-F-
Sporosarcina koreensis	WP_040287536	-TE--VT----RMER-NL-T	RLSS-HFKV-D------
Sporosarcina pasteurii	WP_115359950	-TVTSIE----VIER-NF--	HLS-------R-MA---
Sporosarcina psychrophila	WP_067213083	-TT--IE-AV-VMER-NL-Q	RLS--NFA--E--AD--
Sporosarcina sp. D27	WP_025785611	-AE--AE-AV-LIER-NL-T	RLSS-HLT--A--ED--
Sporosarcina sp. HY008	WP_067405574	-SMEEIT----TIER-NL--	HISS----F---FAQ--
Sporosarcina sp. ZBG7A	WP_039041529	-TD--VE-AV-LIER-NL-T	RLSS-HLT--A--E---
Ureibacillus thermophilus	QBK25270	DTEE-----V-EI-L-----	AVS-P-LA--A-F----
Vibrio vulnificus	WP_133350988	YGEKS-G---VSVK---FLD	RV--NLL---K-FNA--
Viridibacillus arvi	WP_053417876	-SQR-VE-AV-E--R--L--	HIS-------T--N---
Viridibacillus sp. OK051	WP_100794819	-SQR-VE-AV-E--R--L--	HIS-A-----D--N---

Supplemental Figure 54

A partial sequence alignment of the flagellar hook-basal body protein containing a one amino acid insertion (boxed) that is exclusively shared by all members belonging to the Solibacillus clade and absent in all other bacteria.

Group	Species	Accession	50		93
**Solibacillus clade (7/7)**	Solibacillus silvestris	WP_008408248	RLLEALKEYRIHFPYTLQNIEEVIEKL		NAQDGKEGYFRLNVSAG
	Solibacillus isronensis	WP_079523109	---------------------------		-----------------
	Solibacillus kalamii	WP_087618568	---------------R-----------		-----------------
	Solibacillus sp. R5-41	WP_099422070	-----------A-----TEL--I-RQ-		-----QD----------
	Bacillus cecembensis	WP_057989468	-----------A-----TEL--I-HQ-		-----QD----------
	Lysinibacillus sp. 2017	WP_108711207	--I--------A----I-E--TI-HQ-		-----------------
	Lysinibacillus odysseyi	WP_036150880	--ET--HHFH--M---IKEFRT---R-		-EAV-G-----------
**Other Bacteria (0/>100)**	Lysinibacillus fluoroglycofenilyticus	WP_107943716	--T-S-NM---YM----ND-A--MHR-	N	ELNG-GD----I-----
	Lysinibacillus meyeri	WP_107839600	--T-S-NT---YM----NDVV-IMQR-	N	ELNG-GD----I-----
	Bacillus ndiopicus	WP_042479023	--T---NSF--YM----ND-A--MQR-	N	ELEG-ND----I-----
	Anaerobacillus alkalidiazotrop	WP_071389355	--ERSV--LN-I-DLKKEDLR-IVNH-	L	TIN-LEDA-V-F-----
	Anaerobacillus macyae	WP_048313410	--KAS-E-LQ-TL---KLKLLDEVKRT	I	Q-N-MVDA-V---I---
	Bacillus alkalitelluris	WP_078543180	--TSG---LN---EL-KES-LDILHRT	L	SSNGLQDA-V-I-----
	Bacillus cereus	AR060583	--Y-SA-SILLTI-M-VEEM--AVLHT	L	QKNEYADA-I--I--R-
	Bacillus daliensis	WP_090844758	--K-GAG-LNVKL-SS-ETDIREAVPA	L	LKAN-WKDGYFRLNIS-
	Bacillus mannanilyticus	WP_025028141	--V-SCQQIG-EWTPERLKLQ-Q-HD-	L	QVN-LHD----------
	Bacillus panaciterrae	WP_028400949	---T--SFLH-EWHMEKKELLQILHE-	L	ERNGIEDA-V-------
	Bacillus polygoni	WP_088036609	--H-GA--IG-EL-EYKREDTCR-IHQ	L	LELNNL-DGYFRWNISA
	Bacillus safensis	WP_073206721	--YDSARSIMLNI--S-EELT-KMIHT	V	ERN-L-DA-I--V--R-
	Bacillus salsus	WP_090859500	--RI--Q-LN-R-E-SKEDLLQMVHRT	L	E-NGLSDA-I-F-----
	Bacillus sp. 7_6_55CFAA_CT2	WP_000528157	--Y-SA-SILLTI-M-VEEM--AVLHT	L	QKNEYADA-I--I--R-
	Bacillus sp. ABP14	WP_070805746	--Y-SA-SILLTI-L-VDEM--AVLHT	L	QKNEYADA-I--I--R-
	Bacillus sp. BK245	WP_131844564	--RSS-SSLN-EWN-SNEE-RNILH--	I	DLNGLRDA-I-------
	Bacillus sp. FJAT-22090	WP_053591067	--AR--ADF--TM-FDQKT-M-AVKV-	T	TMNNEED------I---
	Bacillus sp. HMA207	WP_116344907	--Y-SA-SILLTI-M-VEEM--AVLHT	L	QKNEYTDA-I--I--R-
	Bacillus sp. L27	WP_071708810	--Y-SA-SILLTI-L-VEEM--AVLHT	L	QKNEYADA-I--I--R-
	Bacillus sp. OxB-1	WP_041072516	--RA--T-F--SM--GDRE-LDAVRR-	D	RDAGDGD----------
	Bacillus sp. S66	WP_121868397	--Y-SA-SILLTI-M-VEEM--AVLHT	L	QKNEYTDA-I--I--R-
	Bacillus sp. es.036	WP_098445940	--RDS-ENLQ-SL---YDELLLQ-KQT	I	E-NEM-DA-V-------
	Bacillus thuringiensis	WP_000528158	--Y-SA-SILLTI-M-VEEM--AVLHT	L	QKNEYADA-I--I--R-
	Bacillus thuringiensis serovar	EEM23067	--Y-SA-SILLTI-L-VEEM--AVLHT	L	QKNEYADA-I--I--R-
	Butyricicoccus sp. 1XD8-22	RKJ60304	Q-IST-N----KM---VIELM----EI	T	-KAN-QD-MI---I---
	Candiadtus Scalindua japonica	WP_096894167	-MRHSA-QL--S-D--NAE-G-W-N--	I	EKNC-QDA-I-ITL-R-
	Candidatus Scalindua brodae	KHE90820	-MRHSA-QL--L-E--NTE-S-I-KT-	I	DKNNIQDA-I-ITL-R-
	Chryseomicrobium excrementi	WP_100354584	--EQT-SAF--DM---SDE-LG-V-E-	T	RLNG-ED----F-----
	Desulfofundulus thermosubterra	WP_084062102	--EKGCTALGMAL-LL-TELGSLVHQT	I	KLN-TTD-AL--TL---
	Domibacillus antri	WP_075397352	--NNS-R-LN-A-EL-REEA-RI-AL-	Q	E-NGWENA-I-------
	Domibacillus epiphyticus	WP_076764166	--NNS-RKLN-V-EF-REEVKQI-AL-	E	E-NGW-NA-I-------
	Exiguobacterium chiriqhucha	WP_021068195	--EKS-V-LW-DL---K-EM-QA-QDV	V	RQNGTPNL-V-------
	Exiguobacterium marinum	WP_026824797	--NKSMEALW-D----KAE--TA-QR-	C	RQN-APNL-I-------
	Exiguobacterium mexicanum	WP_034781814	--EKS-A-LW-DL---K-EM-QA-QDV	V	RQNGTPNL-V-------
	Exiguobacterium profundum	WP_074035325	--IKS-D-LW-D--F-KEE-GTA-QQ-	C	RQN-APDL-I-------
	Exiguobacterium sp. AM39-5BH	WP_128124087	--EKS-A-LW-DL---K-E--QA-QDV	V	RQNGNPNL-V-------
	Exiguobacterium sp. AT1b	WP_074036646	--MKS-D-LW-D----KEE--TA-QQ-	C	RQNEAPDL-I-------
	Exiguobacterium sp. HVEsp1	WP_078145923	--IKS-D-LW-D--F-KEE-GTA-QQ-	C	RQN-APDL-I-------
	Exiguobacterium sp. NG55	WP_031421834	--MKS-D-LW-D--F-KEE-GTA-QQ-	C	RQN-APDL-I-------
	Exiguobacterium sp. SL-10	WP_131438118	--ERS-A-LW-DL---K-EM-QA-QDV	V	RQNGTSNL-V-------
	Exiguobacterium sp. SL-9	WP_131486143	--ERS-A-LW-DL---K-EM-QA-QDV	V	RQNGTSNL-V-------
	Exiguobacterium sp. TNDT2	WP_114167113	--EKS-A-LW-DL---K-EMGQA-QDV	V	RQNGTPNL-V-------
	Filibacter sp. TB-66	WP_124068608	--RT--I-FQ-TL--EDSV-L-TVAR-	N	ELSGEQD----------
	Jeotgalibacillus alimentarius	WP_041120798	--QF--DQMN-QAELSTASVMQM--E-	R	V-NG-ED----------
	Jeotgalibacillus campisalis	WP_041054593	--NHS-E-MQ-DKTFSVEEVMQMVST-	R	E-NG--D----------
	Jeotgalibacillus proteolyticus	WP_104059846	--H-S-Q-MG---SLNITEAADM-DN-	L	E-NG-ED----------
	Jeotgalibacillus salarius	WP_134382907	--QF-MDKMG-EADL-IDDLKLMV-Q-	R	V-NG-ED----------
	Jeotgalibacillus soli	WP_041085352	--HAS-N-MG-RYHIKNEEVAQM----	Q	T-NG-ED----------
	Jeotgalibacillus sp. R-1-5s-1	WP_134372564	--NTS--DMN-DYEMRFEQTC-I-RR-	L	E-NG-ED------I---
	Jeotgalibacillus sp. S-D1	WP_133378562	--N-----MG-NYSLGESEAAGIVQ--	Q	Q-NG-SD--Y-------
	Kurthia gibsonii	WP_121178090	-IRA--AT---KL---DVELK---RE-	T	AKSN-ED----------
	Kurthia huakuii	WP_029501055	-MEQ--AA-H-AL---EEQLRD--ME-	N	VRSG-DD----I-----
	Kurthia massiliensis	WP_010291118	--QR--DA-H-VL---SAELRD--MQ-	N	EREG-TD----------
	Kurthia senegalensis	WP_010309041	-ME----A---QL---E-QLRD--IE-	N	VRNGTED----V-----
	Kurthia sibirica	WP_109307076	--T---AT---AM--SKETLLV---E-	N	-RHGQVD----------
	Kurthia sp. 11kri321	WP_068456675	-IR---AT---QL---DVELK---RE-	T	AKSN-ED----------
	Kurthia zopfii	WP_109350084	-MKT--N--K-KM--SNDELM---RT-	N	AKSGNQD----------
	Lysinibacillus boronitolerans	WP_016993602	--QH--SQ-H--LN-SQSVLL--VQQ-	N	QLAG-QD----------
	Lysinibacillus composti	WP_124766629	--CM--AR---KV---ISE-Q---SE-	T	-RSG-ED-V--I-----
	Lysinibacillus contaminans	WP_053582303	---A--TQ----M---AGELIA---H-	N	EQAG-HD----------
	Lysinibacillus endophyticus	WP_121215323	--CN--NNF--PM---IRD-QN--KE-	W	L-ASREDSI--V-----
	Lysinibacillus fusiformis	WP_004229128	--QR--SQ-H--LN-PESVLL-AVQQ-	N	QLAG-QD----------
	Lysinibacillus jejuensis	WP_108307959	--KQ--QH---TM----EEMTAI-KR-	V	GLA--VDC-----I---
	Lysinibacillus macroides	WP_053997018	--QL--SQ----LT-NEHELLD-VQQ-	N	KLV--QD----------
	Lysinibacillus manganicus	WP_036190663	--ITK-NQFH-EM--SVPE-LR--KD-	T	KKANNQD-L-Y-T----
	Lysinibacillus mangiferihumi	WP_107896431	--QA--TQFH--L---K-DLLA---Q-	N	L-AG--D----------
	Lysinibacillus massiliensis	WP_036179557	--IST-N----KM---IRELM-I-KEM	T	-KAN-RD-KIL--I---

Other Bacteria (0/>100)

Lysinibacillus parviboronicapi	WP_107925782	--QAT-AQ---YMS--EDDLLT---Q- N LQAG-QD----I-----
Lysinibacillus saudimassiliens	CDZ99612	---KS-AT---TM----SEVADIVV-- V ELAG-VD----------
Lysinibacillus sp. AR18-8	WP_066038168	--QH--SQ-H--LN-PESVLL-AVQQ- N QLAG-QD----------
Lysinibacillus sp. B2A1	AVK82177	--HA--SQ-H--LT-SSDTLL-AVRQ- N KQSN-QD----------
Lysinibacillus sp. BF-4	WP_036145237	---KS-AT---TM---MSEVADIVV-- V ELAG-VD----------
Lysinibacillus sp. BK089	WP_132364382	--QT--SQ----M---EEDLLK-VQQ- N EQAN-QD----I-----
Lysinibacillus sp. FJAT-14222	WP_053594133	--QT--SQ----M---DEDVLK-VQL- N EQAN-QD----I-----
Lysinibacillus sp. FJAT-14745	WP_053485345	--QM--SQ----MS--EADLLN-VQQ- N EQA--QD----I-----
Lysinibacillus sp. LK3	WP_048395318	--QH--SQ-H--LN-PESVLL-AVQQ- N QLAG-QD----------
Lysinibacillus sp. Marseille-P	WP_106782191	--ISN-AQL--K----KSE-I--VA-- T ERAN-ED-A---I----
Lysinibacillus sp. OL1	WP_131522621	--QH--SQ-H--LN-PESVLL-AVQQ- N QLAG-QD----------
Lysinibacillus sp. SYSU K30002	WP_126660126	--CSS-NH---SM---IKS--NA-FE- L KRGSN-DSV--------
Lysinibacillus sp. YR326	WP_134025754	--QI--SQ----M---EEDLLN-VQR- N EQD--QD----I-----
Lysinibacillus sp. YS11	WP_103117256	--QH--SQ-H--LN-PESVLL-AVQQ- N QLAG-QD----------
Lysinibacillus sp. ZYM-1	WP_054612561	--QQ--SQ-----T-PENVLL-IVQQ- N EQAG-QD----------
Lysinibacillus sphaericus	AOV09010	--RQ--GD---SM--ENKE-LT--R-- N E-AG--D----------
Lysinibacillus sphaericus C3-4	ACA37734	--QQ--SQ----LT-PESVLL--VQQ- N QQAR-QD----------
Lysinibacillus tabacifolii	WP_108031347	--QA--TQFH--L---K-DLLA--KQ- N L-AG-QD----------
Lysinibacillus varians	WP_025218336	--QA--TQFH--L---K-DLLA--KQ- N L-AG-QD----------
Lysinibacillus xylanilyticus	WP_049667386	--QI--SQ----M---EEDLLN-VQR- N EQD--QD----I-----
Lysinibacillus xyleni	WP_097075301	--CN--NI---PM---IKD-QN---E- W T-ASREDCI--------
Paenisporosarcina antarctica	WP_134211712	--QK--D-F--EVKLDVEMLV-I-DE- N VRSG-TD----------
Paenisporosarcina indica	WP_075620319	--QK--D-FHL-VDLKVLD-SAIVRE- N ERS--YD------I---
Paenisporosarcina quisquiliaru	WP_090570866	--CR--EDF-VAM--EERD-LSAV-A- T A-NNMED------I---
Paenisporosarcina sp. HGH0030	WP_016429931	--QL--D-F-MTIKLDIGTLTS--QE- N ERSG-ED----M-----
Paenisporosarcina sp. K2R23-3	WP_119884388	--HD--Q----AM--A-DELLQ--RE- N EKNE-SD-------T--
Paenisporosarcina sp. OV554	WP_108587555	--RK--D-F--ELELD-EMLKAI-LE- N ARSG--D----------
Paenisporosarcina sp. TG-14	WP_017382097	--QK--D-F--EVKLDVEMLV-I-DE- N VRCV-TD----------
Paenisporosarcina sp. TG20	WP_019415321	-MQN--D-F--DHQLNTNTLYPL-CE- T KLSG-GD------I---
Parageobacillus thermantarctic	WP_090949683	--NHS-R-MN-AKSF-RCEVM-ILHR- L E-NRLQNA-V-------
Parageobacillus thermoglucosid	WP_064550627	--NHS-R-MN-AKSF-RCEVM-ILHR- L E-NRL-NA-V-------
Phycisphaera mikurensis	WP_014437514	--F-SARAI-LAL---PEQL--ATRRT V AKNALSD--I--VATR-
Planctomycetes bacterium	RLS64251	---ASA-SI-LTMH--EREL-QAVRET V A-NGSPD--I--V-TR-
Psychrobacillus insolitus	WP_111440768	--SR--ADF--TM--DKET-ASAVKE- T K-NNNED------I---
Psychrobacillus psychrodurans	WP_093496491	--CR--EDF-VAM--EERD-LSAV-A- T A-NNMED------I---
Psychrobacillus psychrotoleran	WP_093538660	--CR--SDF--EL-CEE-ELIAAVAA- T E-NH-ED------I---
Psychrobacillus sp. FJAT-21963	WP_056833363	--SL--ADF--TM-FDQET-M-AVKV- T TMNNEED------I---
Psychrobacillus sp. OK028	WP_093063078	--CR--SDF--AM--EEHELLS-V-A- T E-NN-ED------I---
Psychrobacillus sp. OK032	WP_093276366	--SR--ADF-MTM--DKKT-LAA-KA- T E-NN-ED------I---
Rummeliibacillus pycnus	WP_102693357	-IQG--E----EMN--FEELRN--ME- N RRNGQTD------I---
Sporosarcina koreensis	WP_060204361	--RL--R----AM--SDEE-A---QG- H VRSG-RD----------
Sporosarcina newyorkensis	WP_009499451	--RR--S----KMQ--DEELRQA-YS- F VENGNED----------
Sporosarcina pasteurii	WP_115359676	--KQ--AD---AM--EDDE-LT--R-- D E-A--RD----------
Sporosarcina psychrophila	WP_067213716	--HT--T----TM--DETE-LAAVR-- D QLNG-QD----------
Sporosarcina sp. EUR3 2.2.2	WP_024536364	--RK--D-F--NVELDIDMLTVI-DE- N TRSG-ED----------
Sporosarcina sp. HYO08	WP_067404321	--KF--S-FN-SL--EADDFFQA-RT- N ERAG-TD--------G-
Sporosarcina sp. P1	WP_099627860	--RS--SD---EMS-SDEE-LSA-HT- Y KENGNED--Y-------
Sporosarcina sp. P13	WP_099689044	--RS--F----AM--GDKE-QQA-SS- Y V-NGSED--Y-------
Sporosarcina sp. P16b	WP_099673749	--RS--ADF--KM--SDEE-LSA-HS- Y KENGCVD--Y-------
Sporosarcina sp. P17b	WP_099625973	--RS--ID-H-EM--SDEE-LMAVHS- Y KENGCED--Y-------
Sporosarcina sp. P18a	WP_099676521	--RS--ADF--KM--SDEE-LSA-HS- Y KENGCVD--Y-------
Sporosarcina sp. P19	WP_099692264	--RS--AD---EMR-SDEE-LSA-HS- Y KENGYED--Y-------
Sporosarcina sp. P20a	WP_099679176	--RS--AD---DM--SDEE-LAA-HS- Y EENGYED--Y---I---
Sporosarcina sp. P21c	WP_099629580	--RS--SD---EMS-SDEE-LSA-HT- Y KENGNED--Y-------
Sporosarcina sp. P26b	WP_099693974	--RS--ID-H-EM--SDEE-LMA-HS- Y KENGCED--Y-------
Sporosarcina sp. P3	WP_099639876	--RS--AD---EMR-SDEE-LSA-YS- Y KENGYED--Y-------
Sporosarcina sp. P33	WP_081243539	--RS--AD---EM---DEE-LAAVYS- Y QDNG-ED--Y-------
Sporosarcina sp. P34	WP_099696534	--RS--A----KM--SDEE-LSA-HS- Y RENGCVD--Y-------
Sporosarcina sp. PTS2304	WP_114925323	--R---S-F--SL--NDED-RQA-HS- Y VTNGSED----------
Sporosarcina ureae	WP_029054896	--RS--AD---EMR-SDEE-LSA-HS- Y KENGYED--Y-------
Streptococcus pneumoniae	CKH79946	--Y-SA-SILLTI-M-VEEM--AVLHT L QKNEYADA-I--I--R-
Tetzosporium hominis	WP_094942901	--EQT-SAF--KM---SDEMLR-V--- T QLNG-ED----F-----
Viridibacillus arvi	WP_053418355	--QR--DD-H-VL--SFDELLA--RE- D -RCN-ED----------
Viridibacillus sp. OK051	WP_100795461	--QH--DD-H-VL--SFVELLA--RE- D ERSN-ED----------

Supplemental Figure 55

A partial sequence alignment of the aminodeoxychorismate lyase protein containing a one amino acid deletion (boxed) that is exclusively shared by all members belonging to the Solibacillus clade and absent in all other bacteria.

Group	Species	Accession	67		116
Solibacillus clade (5/5)	Bacillus cecembensis	WP_057987106	MFSDTFPGSPQ	V	TKGDNITLAYTSDNEQELRKIFELFADGGNVTMPLQET
	Solibacillus sp. R5-41	WP_099422747	-----------	-	-------------------T------N--S--------
	Solibacillus kalamii	WP_008403332	-----Y-----	-	-V----SI----KD---I-F--DGLKE--KIV-E----
	Solibacillus isronensis	WP_079525127	-----Y-----	-	-V----SI----KD---I-F--DGLRE--K-V-E----
	Solibacillus silvestris	WP_014824513	-----Y-----	-	-V----SI----KD---I-F--YGLKE--KIV-E----
Other Bacteria (0/>200)	Bacillus ndiopicus	WP_042474820	--------Q-H		-V-N-V--SVLT-D-EK--YA-DVLKRD---Q-E----
	Lysinibacillus fluoroglycofenilyticus	WP_107942940	--------H-Y		-A-N-V---VVTND-AQ--HA-DVLKRE---Q-E----
	Lysinibacillus meyeri	WP_107840718	--------H-Y		KV-N-V---VVTND-AK--HA-DVLKRE-K-Q-E----
	Anaerobacillus alkalidiazotrop	WP_071390253	--------M-Y		QP--SVQI-IHPTV-ESA-E--AALQ---Q-I----K-
	Anaerobacillus alkalilacustris	WP_071308104	--------M-Y		QP--SVQI-IHPTV-ESA-E--AALQ---Q-I----K-
	Bacillus acidiceler	WP_088014074	--------F-F		NQ-N--H-SFL-EDLE---SV-SKLS---K-E-E----
	Bacillus aciditolerans	WP_121449051	----N---MEY		RE-N-F----I-SD-AAMKDA--KLKE--T-Q-E---V
	Bacillus akibai	WP_035660924	--------T-Y		VA-N--S--VI-Q-QDNITHY-NQLKN--K-GLE----
	Bacillus azotoformans	WP_003329629	----S---R-Y		V--N--E--IV-TDMD-VKS--NQLKE--K-E-E----
	Bacillus bataviensis	WP_007084675	--------Q-H		ES-TQV-ICI-T-DAEKA--F-DALQ---Q-QV-----
	Bacillus bogoriensis	WP_026674796	----N---T-Y		-V-N-----IIM-KQE-ITAA--KLKQ--R-G-N----
	Bacillus butanolivorans	WP_053346132	----V---M-F		VE-N--S--VVNK-MD-IKS--NKLKE--T-V-D----
	Bacillus cohnii	WP_066411701	----N-----H		VE-N-----VI--DREKIESY-HKLKE--K-G-E----
	Bacillus cucumis	WP_101648859	----N---A-H		QS-NQV-ICIST-DAEQA--F--ALS---H-G------
	Bacillus dakarensis	WP_077212264	-----Y--Q-F		VV-N-V---LI-K-MEDIKSWY-QLK---S-G-E----
	Bacillus deserti	WP_101644874	--------Q-Y		QL-NQ--VCIST-DVEKAT-M-DALQQ--K-N------
	Bacillus dielmoensis	WP_042460315	--------Q-A		QR-SQV-ICL-T-DAEKSKQF-NAL----QIQ------
	Bacillus drentensis	WP_066250907	--------Q-H		QS-NQV-ICISTNDADQAH-F--ALQE--Q--------
	Bacillus firmus	WP_035326514	--------M-F		-I-N--N--VVINDVDN---Y-NNL--------E----
	Bacillus fordii	WP_018706074	--------NEV		VN-NS----VV-K-LDG-KSA-YKLSE--K-E-E----
	Bacillus fortis	WP_120071251	---------EV		EN-S-----FV-K-MDDVKST-HKLSE--T-E-E----
	Bacillus freudenreichii	WP_126433358	--------NEV		VY-NG----FV--DMEK-KAA-DKLSE--T-E-E----
	Bacillus gaemokensis	WP_033674187	-I------K-Y		EI-SQV-I-IRMNDSEKAKEV--KLQ---E-L------
	Bacillus halmapalus	WP_078382442	----V---M-F		VE-N--S--VV-K--E-V-SV-HKLQE--K-G-E----
	Bacillus humi	WP_058000398	----N---MEF		KE-N-F----I-SD-AAM-DA--KLKE--T-Q-E---V
	Bacillus lentus	WP_066138089	--------M-F		VV-N--S--FV-E-LD-VKSA-NQLKE--K-G-E----
	Bacillus litoralis	WP_066325293	--------M-F		VE-N--S--IV-K-LD-IKSA-NKLK-----G-E----
	Bacillus loiseleuriae	WP_049681522	----V---M-F		I--N--S--IV-E-LD-IKSQ-DKLKE--T-G-E----
	Bacillus luciferensis	WP_088072643	--------F-F		IP-N-VH-SFI-EDLE-I-SV-NKLS---K-G-E----
	Bacillus marisflavi	WP_048007110	--------T-F		IE-N-----IVK-DKEV-TAL-DRLKEH-K-D------
	Bacillus massiliogabonensis	WP_102272256	--------Q-A		QE-NLV-ICV-F--ADKA-Q---ALQ---Q-E---AD-
	Bacillus massilionigeriensis	WP_075981942	----------F		VQ-N--S-T-M-KDIDDIKSV-NKLK---K-E-D----
	Bacillus muralis	WP_057914947	----V---M-F		IE-N--S--FV---LE-IKS--HNLKE--T-R-E----
	Bacillus nakamurai	WP_061521669	-I------Q-Y		QI-SQV-V-V-I--A-KSKEV--KLKE----E------
	Bacillus niacini	WP_045522417	--------M-F		IE-N-V---LV-K-MDD-KSW--QLK---T-G-E----
	Bacillus notoginsengisoli	WP_118919043	--------A-F		-V-N--S---H-FDADK-QAV-NRMKE--T-GVE----
	Bacillus oceanisediminis	WP_019383461	--------M-F		-V-N--N--VVINDLDK---Y-NNLQA--T---E----
	Bacillus okhensis	WP_034629016	--------T-H		VV-NHV-ICIMA-TKEQATEYYQALQE--K--------
	Bacillus onubensis	WP_099351410	----N---Q-F		VE-N-V---LL-E-MDD-KSWY-QLK---T-G-E----
	Bacillus praedii	WP_057760567	----------F		-Q-N-----IV-KDIN-IKSV-NKLKE--TIG-E----
	Bacillus pseudofirmus	WP_075387326	----------F		MV-N--S---H-FDADK-QSVYNRLKEC-T-D-E----
	Bacillus pseudomycoides	PEA55035	-I------Q--		QG-SQV-I-IHIQ-AEKSQEV--KLQ---Q-I------
	Bacillus simplex	WP_034311993	--------M-F		VE-N--S---VGNDVE-IKS--NKLSE--T-GLE----
	Bacillus sinesaloumensis	WP_077619593	----NY--MEY		RE-N-F----I-SD-AAM-DA-DKLKE--T-Q-E---V
	Bacillus soli	WP_066072592	--------Q-H		QS-TQV-ICI-T-DAEKAH-F-DALQ---Q-G------
	Bacillus solisilvae	WP_088002246	--------R-F		VE-N--N-SFQ-N-VE-IQAVYNKLKE--Q-G-E----
	Bacillus sporothermodurans	WP_107958743	--------T-F		VV-N--S--IV-Q-QD-ITSYYNQLKE--K-N-E----
	Bacillus subterraneus	WP_125478780	-L--N---G-Y		QL-SQV-I-LLINDVS-ANEV--KLQ--------I---
	Bacillus terrae	WP_120118890	--------NEV		EN-N-----FV-K-MDDVKSV-HKLSE--T-E-E----
	Bacillus timonensis	WP_010676785	----N---MEF		KM-N-F----I-SD-AAMKDA--KLKE--T-Q-E---V
	Bacillus tuaregi	WP_071394887	----NY--M-Y		QT-N-F----V-N--AAI-EA--KLKEE-S-E-E----
	Brevibacillus borstelensis	WP_003391183	--------Q-H		NV-NQV-ICI-TNSPE-AKQLY-ALQQ--Q-N------
	Brevibacillus brevis	WP_015891652	----I---Q-H		QP--QV-ICIST-DKEQSH---NALQE--Q-I------
	Brevibacillus centrosporus	WP_122960261	--------Q-V		QI-NQV-ICLST-SADKSKQL--ALQE--S-V------
	Brevibacillus formosus	WP_047067721	--------Q-H		QL--QV-ICIST-DKEQAH---DALQE--Q-I------
	Brevibacillus gelatini	WP_122903802	----A---M-Y		SA-T-FS--VI---HD--KGY-DKLKE--T-R-E----
	Brevibacillus laterosporus	WP_003337309	--------Q-H		Q--NQV-ICIATS-KE-STQ-Y-ALQE--Q-N------
	Brevibacillus nitrificans	WP_122925479	--------Q-V		QI-NQV-ICLST-SADKS-QL--ALQE--S-V------
	Brevibacillus parabrevis	WP_122966835	----V---M-Y		VA-T--S--II-E-LD--KGY-AKLKE----R-E----
	Brevibacillus reuszeri	WP_049736858	----S---QSI		P---HV-ICIV--DVEKSKR--DALQ---Q-L------
	Caldibacillus debilis	OUM90455	-----Y--M-Y		QP--TVQI-IHPKD-ARA----AAL----Q-VL---K-
	Cohnella luojiensis	TFE30087	----V---M-F		VQ-N--S-SIV-K-LE-VKSY-HKLKE--KIG-E----
	Cohnella panacarvi	WP_027084839	--------T-F		-S-N-VN-VVN--SKEKMNGL-GRLK---T---E----
	Domibacillus aminovorans	WP_063966071	----V---M-F		VE-N--S--LVNK-MD-IKS--NKLRE--T-V-D----
	Domibacillus enclensis	WP_045849037	--------M-F		QQ-ST---TIV-AD--K-TSY--GLSE--E-A------
	Domibacillus indicus	WP_046175966	-----L--M-Y		QQ-SA----VV-S-ADK--SC--ELSE--E---A----
	Domibacillus iocasae	WP_069938658	--------M-Y		HP-SA----LV-SDAEK--SY--GLS---E-L------
	Domibacillus robiginosus	WP_050180369	L-------T-Y		AQ-SA-S--LVGSDMDK--SY--GL-V--T----I---
	Domibacillus tundrae	WP_046180150	----A---M-Y		QP-SA----LI-SDAEK--AY--GLSEN-E-I------

**Other Bacteria (0/>200)**	Exiguobacterium chiriqhucha	WP_021066645	----AM-DG-V	-F-Q-----VVT-DLHAI-RE--ALS---R-L------
	Exiguobacterium mexicanum	WP_034776645	----AM-DG-V	-F-Q-----VVT-DLEAI-RE--ALS-E-H-L------
	Fictibacillus arsenicus	WP_066290307	--------M-F	VE-N-V---IVNKDIEQIKS-Y-GLKED---V-E----
	Fictibacillus gelatini	WP_026676303	-I------HSY	QL--QV-V-L-V-DVEKSKEV-DKLQ---K-I------
	Gorillibacterium timonense	WP_058304313	--------Q-V	-P-NL-S-CI-A-GFEKSK-L-DAL-N--Q-L--F---
	Hungatella hathewayi	WP_006774775	--------M-Y	EQ-NH--I-VAPED-ETA-R--GHLKE--K-E-E-S--
	Jeotgalibacillus malaysiensis	WP_039812512	---------AL	---------VVINE-EK--AQ-DRIKE--V-Q-D--K-
	Jeotgalibacillus salarius	WP_134381344	---------DL	R--------VVL-DAEK-KTQ-NKLSE--T-Q-E----
	Lihuaxuella thermophila	WP_089964534	--------Q-S	QS--QV-ICI-I--LERSKQ-Y-ALLQ--Q-K------
	Lysinibacillus acetophenoni	WP_097147766	-----W--Q-Y	VV-N-V-V-VL--D-EY--SS--ALRE--Q---E----
	Lysinibacillus composti	WP_124763719	-----W----F	-V-N---V-VVDSD-DF--SS-KALSE----V-E----
	Lysinibacillus endophyticus	WP_121213925	----NW----Y	VV-N--SVTLV-KD-EFI-SS--GLKQ--T-N-E----
	Lysinibacillus fusiformis	WP_004233493	--------M-F	ST-N-V---VV--D-EQM-QA-DKLKE----R-E----
	Lysinibacillus halotolerans	WP_122971044	-----W----F	-V-N---I-VV-ED-ELI-SA-DGLKE--K-N-E----
	Lysinibacillus macroides	WP_053997162	----------F	-V-N-----IV----EQM-QA-DKLK---E-K-E----
	Lysinibacillus manganicus	WP_036182059	-----W--Q-Y	VV-N-V-V-VL--D-EY--NS-DALKE--T---E----
	Lysinibacillus mangiferihumi	WP_107894952	----------F	-I-N-V---VVT-D-AQ--QA--KLKE--K---E----
	Lysinibacillus parviboronicapi	WP_054767882	----------F	-V-N-V---IVL-D-AQ--SAYKGLKE--K-N-E----
	Lysinibacillus sinduriensis	WP_036198993	-----W----F	VA-N--S-TVV-ED-DLI-SS--RLSE--T-S-E----
	Lysinibacillus sphaericus	VDG99511	----------V	VV-N--S--FV--DTD--TSS--KLKQN-Y-S-E----
	Lysinibacillus telephonicus	WP_126293720	-----W----Y	VV-N-V-V-LV-ED-AL--SA--GLKE--T-K-E----
	Lysinibacillus xylanilyticus	WP_049662719	----------F	-T-N-V---VV--D-AQM--A-DGLK--SE-K-E----
	Lysinibacillus xyleni	WP_097072351	----NW----Y	VV-N--SVTVV--D-AFI-SS--GLKE--K-S-E----
	Oceanobacillus arenosus	WP_115774186	----N---M-F	VE-N----SFINK--D-I-SAYDKLSK--EIVLE----
	Oceanobacillus chungangensis	WP_115751166	----N---M-F	VE-N----SLINK--D-I-AAY-KLSK--E-VLE----
	Paenibacillaceae bacterium	REJ17101	----A---M-Y	QQ-SAVEI-VVL-D-AQA----DALSE--E-V----K-
	Paenibacillus alginolyticus	WP_029199273	--------QTV	Q----V-ICI--KDKEKSQ-FYNALK---Q-I-E----
	Paenibacillus amylolyticus	WP_062837702	-L------M-Y	-V-NHV-VTVNTNTAD-AKA--NQLQ---E-K--I---
	Paenibacillus baekrokdamisoli	WP_125654259	----V---M-F	VL-N--S--VG-KDMD-VKSL-HKMKE--T-V-E----
	Paenibacillus beijingensis	WP_045668872	----V---M-F	-A-N--S-SIVT--KD-IESA-NKL----T-G-E----
	Paenibacillus bouchesdurhonens	WP_110932598	----V---M-Y	VQ-N-----FV-TDLD-IKSV-HKLKE--T-N-E----
	Paenibacillus camerounensis	WP_042200543	----V---M-F	-A-N--S--LVTR--EDVKSW-HQLKE--S---E----
	Paenibacillus castaneae	WP_102711044	----V---M-F	VV-N-----IV---KD-I-SF-DKLKV--K-G-E----
	Paenibacillus catalpae	WP_091189107	----V---M-F	IV-N------VVED-DKI-SA-DQLKV--TIV-E----
	Paenibacillus cellulosilyticus	WP_110043306	--------M-F	SV-T----S-QGSS-E---S--NKL----K-G-E----
	Paenibacillus chondroitinus	WP_047674397	--------M-Y	VA-N--S-SLV-KDID-VKSA-HKLKE--K-G-E----
	Paenibacillus crassostreae	WP_068658087	--------M-F	VV-N--S--IV-KDLDDVKSL-AKLQE--K-G-E----
	Paenibacillus daejeonensis	WP_020616525	----V---M-F	-P-N--S-SLI-RDMDA-KDA-HKLK---H-Q-E----
	Paenibacillus dendritiformis	WP_006677332	----V---M-F	-V-N--S--AICE--DQ-QS-YDKLQA--K-V-E----
	Paenibacillus donghaensis	WP_087916791	----V---M-F	-V-S--S-SVVT--AE--KTA-HQLQE--K-E-E----
	Paenibacillus ehimensis	WP_036715076	--------T-F	-A-N--S-SVV-K-ID-IKTY-NKLKE----G-D----
	Paenibacillus elgii	WP_010497699	----I---M-F	-A-N--S-SVM-K-ID-IKTY-NKLKE----G-E----
	Paenibacillus ferrarius	WP_079420833	--------M-Y	IA-N--S-SLV-P-LV-VQNA-HKLKE--K-G-E----
	Paenibacillus fonticola	WP_019639766	----V---M-F	VQ-N--S--FV-TDLEQVKSV-HQLKE--T-G-E----
	Paenibacillus ginsengarvi	WP_120745318	----V---M-F	-Q-N--S-SFV-ESRE-VETA-NRLKE--S-I-E----
	Paenibacillus glacialis	WP_068530780	----V---M-F	VV-N--S--VVTK-ID-VKSL--QLKE--K-G-E----
	Paenibacillus glycanilyticus	WP_127499194	----V---M-F	-V-N--S--FVEED-AK--SA-DRLKV---IA-E----
	Paenibacillus ihuae	WP_054941238	----V---M-F	-V-N--S-TLVTRD-AKVKTW-DQLKE--S---E----
	Paenibacillus ihumii	WP_055107883	----V---M-Y	VQ-N-----FI--DLE-VKSV-HKLKE--T-N-E----
	Paenibacillus illinoisensis	WP_110821800	-L------M-H	VI-N--SVTVNT-TPD-AKH--NKLE---E-K--I---
	Paenibacillus kobensis	WP_127533607	----V---M-F	-V-S----S-VTED-DKV-SV-NRLS---S---E----
	Paenibacillus koleovorans	WP_127579464	--------M-F	V--N-V---LVLKDKEAVESA-HKLKV---I--E----
	Paenibacillus kribbensis	WP_068501436	----V---M-F	VA-N--S--FVNKD-ERI-SV-HKLKE--T-N-E----
	Paenibacillus lentus	WP_125083317	----V---T-Y	IQ-N-----FV-TDLD-IKSV-HKLKE--T-H-E----
	Paenibacillus macerans	OMG46114	--------Q-H	QS-TQV-ICL-TNDAEQAKRM--AL-E--RIN------
	Paenibacillus macquariensis	WP_068585976	--------M-F	VV-N--S--VVTKDID-VKSL-DKLKE--K-S-E----
	Paenibacillus massiliensis	WP_018882960	-L------M-H	-----V-VSVQA-SPE-A-T--GKLQE----I--I---
	Paenibacillus montanisoli	WP_112881300	--------M-F	-M-N--S--IVI-DAE--KRVYAKLGE--Q-V------
	Paenibacillus odorifer	WP_076218478	----V---M-F	IE-N--S-SVV-KDLDNIK-Y--KLQE--K-V-E----
	Paenibacillus pabuli	WP_062326582	-L------M-H	II-NH-SVTVNT-TAD-AKA--NQLQ---E-K--I---
	Paenibacillus peoriae	WP_010345980	----V---M-F	VA-N--S--FVNKD-EKI-SV-HKLKE--T-N-E----
	Paenibacillus phocaensis	WP_068785463	----V-----F	-V-T--S--LV-THRE-IVSA-DRLKE--K-S-E----
	Paenibacillus pinihumi	WP_028563319	--------M-F	VP-N--S--VVL-D-EA--SA-ARLQ--AE-K-D----
	Paenibacillus pinisoli	WP_120113744	-LY-------Y	-R-N-M-VCV-LP-AERATQV--KL----HIVQ---A-
	Paenibacillus polymyxa	WP_081276818	----V---M-F	VT-N--S--IVNKD-EKI-SYYHKLKE--T-N-E----
	Paenibacillus polysaccharolyti	WP_090924229	-L---L--M-H	-V-----VTVNT-TAEQAKD--SKLEV--K-N--I---
	Paenibacillus prosopidis	WP_114379778	--------M-F	QP--TVQI-IHPRE-ARA-E--AALE---Q-V----K-
	Paenibacillus rhizosphaerae	WP_076174869	L-------N-Y	QL--QLSI-I--NDSER-QSV-AGLS---Q-L-E--K-
	Paenibacillus riograndensis	WP_060819139	----V---M-Y	VE-N-VN-TL-N-DAGKIKDW-NKLKE----I-E----
	Paenibacillus rubinfantis	WP_059043155	----V-----Y	-V-T-LS--LV-S-RE-ITSA--RLKE--S-K-E----
	Paenibacillus senegalensis	WP_010268713	--------M-Y	QQ-N--S--FV-KD--AI-DA-HKLSE--D-K-E----
	Paenibacillus silvae	WP_111270377	-L---L--M-H	-V-----ITVNT-TAE-AKN--GKLEV--K-N--I---
	Paenibacillus sonchi group	WP_020430708	----V---M-Y	VE-N-VN-TL-N-DAGKIKDW-NKLKE----I-E----
	Paenibacillus taihuensis	WP_116188628	--------M-L	-M-N-----LIINDAE--TRVYNRISGD-TA-------

Other Bacteria (0/>200)

Paenibacillus terrae	WP_014280921	----------F	SS-NQV-ICI-T-SVEQSQ-M-DALQQ--R-G------
Paenibacillus terrigena	WP_018754451	--------Q-F	-Y--QLSI-IV-NDMER-KS-YHAL----Q-I-E--K-
Paenibacillus thiaminolyticus	WP_087440065	----V---M-F	-V-N--S--IV-E--DD-QTF-DKLKV--K-V-E----
Paenibacillus typhae	WP_090714250	----V---M-F	-Q-N--S--LVTR--EDVKSW-NQLQE--S---D--Q-
Paenibacillus uliginis N3/ 975	SMF86893	----V---M-F	VE-N-V---VVTN-KE-IDSA-HILKE--TIE-E----
Paenibacillus xylaniclasticus	WP_127566741	----V---M-F	-I-S-----FVTVS-DKA-TV-KRLS---K-E-E----
Paenisporosarcina indica	WP_075617811	----V---M-F	IV-N--S---VNE-LEDVTSK-NKLKED-K-G-E----
Paenisporosarcina quisquiliaru	WP_090565204	----------F	ME-N--S--FV-KE-DLVKSA-NALKE--R-S-E----
Proteiniclasticum ruminis	WP_031574472	----A-----V	-V-E-VSIMV--KDME--KAQ-HKIKE--R-E-D----
Psychrobacillus insolitus	WP_111438877	----------F	IQ-N--S--FVGK--E-I-SA-DKLKE--S-G-E----
Psychrobacillus psychrodurans	WP_093496611	----------F	ME-N--S--FV-KE-DMVKSA-NALKE--R-S-E----
Psychrobacillus psychrotoleran	WP_093537244	----------F	-E-N--S--FV-NE-DTVKSA-NALKE--K-S-D----
Rhodococcus qingshengii	WP_133370510	----N-----F	IE-N-V-M-LV-K-LDDIQSWYDQLKE--T---E----
Risungbinella massiliensis	WP_044640108	--------M-Y	QQ--SVQI-LHPSD-ARS-E--NAL----QIV----K-
Rummeliibacillus pycnus	WP_102693476	--------N-Y	-V-N-V---VV--D-GYI-SA-RALQ---E-I-E--K-
Seinonella peptonophila	WP_073154582	L---V-----F	VE-N--H-V-MDNDIEKIKHT-DQLKA--K---D----
Shimazuella kribbensis	WP_028777219	--------Q-H	QI-S-VSI-IVVESKE-SK----KLSQ--Q-Q----K-
Sporolactobacillus inulinus	WP_010025256	--------Q-A	QE-NLV-ICV-F--ADKAHQ---ALQ---Q-E---S--
Sporolactobacillus laevolactic	WP_023510299	----N---QSS	QQ--Q--ICI---DKEKST----SLSQ--Q-K----D-
Sporolactobacillus pectinivora	WP_100488192	----N---QSS	KT--QV-ICI----KDKST-M--ALSE--A-K----D-
Sporosarcina globispora	WP_053437113	--------M-F	-V-N--N--VVI-DLDN---Y-NNLQE--T---E----
Sporosarcina koreensis	WP_060206158	--------F-F	VE-N-VS--FVNKDFDLIKLV--KLKE--T-K-D----
Sporosarcina ureae	WP_029055070	----------F	-V-N-VS---H-FD-GK-Q-VYDRLKE--T-E-E----
Tumebacillus avium	WP_087459095	--------Q-H	AI-SQV-ICL-T-SKETAERF-NAL----Q-S------
Ureibacillus thermosphaericus	WP_016838027	--------N-Y	-I-N---V-FID-DLDFMKRA--MLK---Q-E-E----
Vibrio vulnificus	WP_133346247	--------M-F	VE-N--S---V-NDLD-IKST-NKLSE--T-GLE----
Viridibacillus arvi	WP_053416706	----V-----F	IV-N-VS--VV----EQ-KSA--ALKV--K-A-E----

Supplemental Figure 56

A partial sequence alignment of the VOC family protein containing a one amino acid insertion (boxed) that is exclusively shared by all members belonging to the Solibacillus clade and absent in all other bacteria.

Group	Organism	Accession	266		297
**Solibacillus clade (6/7)**	Solibacillus kalamii	WP_087617253	VNKANMIRKMDELRV	N	DRRLDGISEIRDESDR
	Solibacillus silvestris	WP_014823878	---------------	-	----------------
	Solibacillus isronensis	WP_079526712	---------------	-	----------------
	Solibacillus sp. R5-41	WP_099423716	-----L---------	S	----E-----------
	Bacillus cecembensis	WP_057989369	-----L---------	S	----E-----------
	Lysinibacillus sp. 2017	WP_108712744	---------------	-	----------------
	Lysinibacillus odysseyi	WP_036159047	-----LVK-I--Q-L	-	-K--E--A--------
**Other Bacteria (0/>200)**	Lysinibacillus fluoroglycofenilyticus	WP_107942741	-----L-K-I--Q-S		-K--E--ADV------
	Lysinibacillus meyeri	WP_107838011	-----L-K-I--Q-A		-K--E--ADV------
	Bacillus ndiopicus	WP_042472650	-----L-K-I--Q-S		----E--ADV------
	Anaerobacillus macyae	WP_048309379	I----LVK-I-----		--KVE----V---T--
	Aquisalibacillus elongatus	WP_124219851	-----LVK-F----I		--KIE----V---T--
	Aureibacillus halotolerans	WP_133579313	----TLV--I----L		--KVE----V------
	Bacillus abyssalis	WP_078410818	-----LVK------L		--KVE--A-V---T--
	Bacillus acanthi	WP_108669265	-----LVK------L		--KVE--------T--
	Bacillus acidiproducens	WP_018663581	-----LVK----F-I		--KVE-M--V---T--
	Bacillus alkalitelluris	WP_078546888	-----LVK----F-L		--KIE----V---T--
	Bacillus amyloliquefaciens	WP_042635234	-----LVK----Y-I		-KKVE----V---T--
	Bacillus amyloliquefaciens gro	WP_021493413	-----LVK----Y-I		-KKVE----V---T--
	Bacillus aquimaris	WP_044336984	-----LVK----F--		--KVE----V---T--
	Bacillus aryabhattai	WP_045291689	-----LVK------L		-KKVE--A-V---T--
	Bacillus aryabhattai B8W22	SDD63613	-----LVK------L		-KKVE--A-V---T--
	Bacillus atrophaeus	WP_010788828	-----LVK----Y-I		-KKVE----V---T--
	Bacillus australimaris	WP_060697812	-----LVK----F-I		E-KVE----V---T--
	Bacillus bataviensis	WP_007087024	-----LVK----F-L		--KVE----V---T--
	Bacillus camelliae	WP_101353669	-----LVK------L		--KVE----V---T--
	Bacillus cavernae	WP_126865449	-----LVK-I----I		--K-E--A-V---T--
	Bacillus cereus	WP_002146338	-----LVK------I		-KK----A-V---T--
	Bacillus cereus group	WP_002121333	-----LVK------L		-K-----A-V---T--
	Bacillus cihuensis	WP_028390758	-----LV-----F-L		--KVE----V---T--
	Bacillus coagulans	WP_013859317	-----LVK-I----I		--KVE----V---T--
	Bacillus coahuilensis	WP_059350933	I----LVK----F--		--KVE--------T--
	Bacillus cohnii	WP_066411089	-----LVK------L		--KVE----V---T--
	Bacillus daliensis	WP_090840210	I----LVK------I		-KKVE--A-V---T--
	Bacillus deserti	WP_101643084	-----LVK----F-L		--KVE----V---T--
	Bacillus dielmoensis	WP_042456761	-----LVK----F-I		--KVE----V---T--
	Bacillus drentensis	WP_066254818	I----LVK----F-L		--KVE--------T--
	Bacillus endophyticus	WP_061804035	-----LVK------L		--KVE--A-V---T--
	Bacillus filamentosus	WP_019392293	-----LVK------L		--KVE--A-V---T--
	Bacillus freudenreichii	WP_126432171	-----LVK----F-L		--K-E----V---T--
	Bacillus ginsengihumi	WP_025728633	-----LVK-I--F--		--KIE----V---T--
	Bacillus glycinifermentans	WP_048355566	-----LVK----F-I		--KVE----V---T--
	Bacillus gobiensis	WP_053602922	-----LVK----Y-L		--KVE--------T--
	Bacillus gottheilii	WP_066445536	-----LVK------L		--KVE--A-V---T--
	Bacillus halotolerans	WP_059291732	-----LVK----Y-I		-KKVE----V---T--
	Bacillus intestinalis	WP_061186391	-----LVK----Y-I		-KKVE----V---T--
	Bacillus koreensis	WP_053400443	-----LVK------I		--KV---A-V---T--
	Bacillus kribbensis	WP_026695789	-----LV-----F-L		--KVE----V---T--
	Bacillus licheniformis	WP_080626920	-----LVK----F-I		E-KVE----V---T--
	Bacillus loiseleuriae	WP_049681208	-----LV-----F-L		--KVE----V---T--
	Bacillus mannanilyticus	WP_025026828	-----LV--I-----		--KVE--A-V---T--
	Bacillus marisflavi	WP_048003660	I----LVK----F--		--KVE--A-V---T--
	Bacillus massiliglaciei	WP_110929553	-----LVK------L		--KVE----V---T--
	Bacillus massilioanorexius	WP_019241398	-----LVK------I		--KVE--------T--
	Bacillus massiliogorillae	WP_042348485	-----LVK-I----I		--KVE----V---T--
	Bacillus megaterium	RBN41959	-----LVK------L		-KKVE--A-V---T--
	Bacillus mesonae	WP_066398461	-----LVK----F-L		--KVE----V---T--
	Bacillus mobilis	WP_071722454	-----LVK------I		-KK----A-V---T--
	Bacillus mojavensis	WP_029441413	-----LVK----Y-I		-KKVE----V---T--
	Bacillus mycoides	WP_012261394	-----LVK------I		-KK----A-V---T--
	Bacillus niameyensis	WP_062104968	-----LVK----F-L		--K-E----V---T--
	Bacillus notoginsengisoli	WP_118919809	-----LVK----F-L		--K-E--------T--
	Bacillus novalis	WP_066087285	-----LV-----F-L		--KVE----V---T--
	Bacillus oleivorans	WP_097160242	-----LVK----F-L		--KVE----V---T--
	Bacillus pumilus	WP_012010117	-----LVK----F-I		E-KVE----V---T--
	Bacillus safensis	WP_024427093	-----LVK----F-I		E-KVE----V---T--
	Bacillus subtilis	CUB42574	-----LVK----Y-I		-KKVE----V---T--
	Bacillus testis	WP_050614912	-----LVK------I		--KVE----V------
	Bacillus vallismortis	WP_010330654	-----LVK----F-I		-KKVE----V---T--
	Bacillus velezensis	ASB65558	-----LVK----Y-I		-KKVE----V---T--
	Bacillus vietnamensis	WP_060672026	I----LVK----F--		--KVE--A-V---T--
	Bhargavaea beijingensis	WP_092097339	----HLVK------L		--K-E----V------
	Bhargavaea cecembensis	WP_008298350	----HLVK------L		--K-E----V------
	Bhargavaea ginsengi	WP_092051038	----HLVK------I		--K-E----V------

Other Bacteria (0/>200)

Caryophanon latum	WP_066464092	-----LVK----Q-S	-K--E--A-V------
Domibacillus aminovorans	WP_063964430	----SLVK------I	--K------V---T--
Domibacillus mangrovi	WP_073711513	----SLVK------I	--K------V---T--
Edaphobacillus lindanitolerans	WP_076757647	-----LVK-I----L	--K-E----V------
Enterococcus sp. 6C8_DIV0013	WP_086343277	----VLVK------L	N---E----V------
Falsibacillus pallidus	WP_114744663	-----LVK-------	-KKVE----V---T--
Falsibacillus sp. GY 10110	WP_121680002	-----LVK----F--	--KVE--A-V---T--
Filobacillus milosensis	WP_134339166	-----LVK------I	E-KVE----V---T--
Halalkalibacillus sp. B3227	WP_101330481	I----LVK------L	--KVE----V---T--
Halobacillus massiliensis	WP_082234086	-----LVKR-----I	--KVE--A-V---T--
Jeotgalibacillus campisalis	WP_041057727	-----LVK------A	--KVE----V---T--
Jeotgalibacillus sp. S-D1	WP_133375762	-----LVK------A	--KVE----V---T--
Kurthia gibsonii	WP_121177368	-----LVK-I-----	-K--E--A-V------
Kurthia massiliensis	WP_010287585	----HLVK---D---	-K-----A-V------
Kurthia senegalensis	WP_010300495	----HLVK---D--L	-K--E----V------
Kurthia sibirica	WP_109307383	-----LV--------	-K-------V------
Kurthia sp. 11kri321	WP_068453541	-----LVK-I-----	-K--E--A-V------
Kurthia zopfii	WP_109349134	-----LVK-------	-K--E--A-V------
Lentibacillus jeotgali	WP_010530737	------VK------I	--KVE--A-V---T--
Listeria floridensis	WP_036096260	-----LVKR-----I	-KKIE----V---T--
Lysinibacillus	WP_036126105	-----LVK-I--Q--	-K--E--A--------
Lysinibacillus boronitolerans	WP_036079287	-----LVK-I--Q--	-K--E--A--------
Lysinibacillus chungkukjangi	WP_107936371	-----LVK-I--Q--	-K--E-----------
Lysinibacillus fusiformis	WP_004227925	-----LVK-I--Q--	-K--E--A--------
Lysinibacillus mangiferihumi	WP_107895343	-----LVK-I--Q--	-K--E--A--------
Lysinibacillus parviboronicapi	WP_107926872	-----LVK-I--Q--	-K--E--A--------
Lysinibacillus sinduriensis	WP_036201557	-----LVK-I--Q--	-K--E-----------
Lysinibacillus sp. AR18-8	WP_066036681	-----LVK-I--Q--	-K--E--A--------
Lysinibacillus sp. B2A1	AVK84992	-----LVK-I--Q--	-K--E-----------
Lysinibacillus sp. BK089	WP_132360656	-----LVK-I--Q--	-K--E--A--------
Lysinibacillus sp. FJAT-14222	WP_053593447	-----LVK-I--Q--	-K--E--A--------
Lysinibacillus sp. FJAT-14745	WP_053485550	-----LVK-I--Q--	-K--E--A--------
Lysinibacillus sp. OL1	WP_131520152	-----LVK-I--Q--	-K--E--A--------
Lysinibacillus sp. PB300	WP_115673658	-----LVK-I--Q--	-K--E--A--------
Lysinibacillus sp. YR326	WP_134023157	-----LVK-I--Q--	-K--E--A--------
Lysinibacillus sp. YS11	WP_103118203	-----LVK-I--Q--	-K--E--A--------
Lysinibacillus sp. ZYM-1	WP_054611782	-----LVK-I--Q--	-K--E--A--------
Lysinibacillus sphaericus	WP_010859374	-----LVK-I--Q--	-K--E--A--------
Lysinibacillus tabacifolii	WP_108030854	-----LVK-I--Q--	-K--E--A--------
Lysinibacillus varians	WP_025219468	-----LVK-I--Q--	-K--E--A--------
Lysinibacillus xylanilyticus	WP_049665167	-----LVK-I--Q--	-K--E--A--------
Marinilactibacillus piezotoler	WP_091895179	-----LV-----I-I	N-KIE--A-V------
Marinilactibacillus psychrotol	WP_087060189	-----LV-----I-I	N-KIE--A-V------
Marinilactibacillus sp. 15R	WP_072694880	-----LV-----I-I	N-KIE--A-V------
Melissococcus sp. OM08-11BH	WP_117972268	-----LVK------L	TK-VE----V---T--
Oceanobacillus arenosus	WP_115772660	------VK------L	--KVE--A-V------
Oceanobacillus caeni	WP_060669180	-----LVK------L	--KVE--A-----T--
Oceanobacillus chungangensis	WP_115749805	----S-VK-------	--KVE--A-V------
Oceanobacillus iheyensis	WP_106896746	------VK------I	--KVE--A-V---T--
Oceanobacillus picturae	WP_036572924	------VK------I	--KVE--A-V---T--
Oceanobacillus senegalensis	WP_085992097	------VK------I	--KVE--A-V---T--
Oceanobacillus sp. Castelsardo	WP_068672243	-----LVK------I	--KVE--A-V---T--
Ornithinibacillus halophilus	WP_072887171	-----LVK-I----I	--KVE--A-V---T--
Paenibacillus polymyxa	SPY12831	-----LVK----F-I	-KKVE----V---T--
Paucisalibacillus globulus	WP_026907675	-----LVK------I	--KVE--A-V---T--
Planomicrobium soli	WP_106532576	-------K---D--F	--K-E----V------
Pontibacillus chungwhensis	WP_036778996	-----LVK------I	-KKVE--A-V---T--
Pontibacillus halophilus	WP_026799290	------VK------I	-KKVE--A-V---T--
Pontibacillus litoralis	WP_036833309	-----LVK------L	-KKVE--A-V---T--
Pseudogracilibacillus auburnen	WP_110396405	-----LV------AA	--KVE--A-V---T--
Pueribacillus theae	WP_116555543	-----LVK------L	--K-E--A-V---T--
Rummeliibacillus pycnus	WP_102691757	-----LVK----M-L	-K--E--A-V------
Rummeliibacillus sp. POC4	WP_119415740	-----LVK----M-L	-K--E--A-V------
Rummeliibacillus sp. TYF005	WP_124217053	-----LVK----M-L	-K--E--A-V------
Rummeliibacillus stabekisii	WP_066787030	-----LVK----M-L	-K-----A-V------
Sediminibacillus massiliensis	WP_077621856	-----LVKR-----I	--KVE--T-V---T--
Sphingobacterium sp. A3	WP_130570954	-----LVK----Y-I	-KKVE----V---T--
Sporosarcina koreensis	WP_040286497	-----LVK------L	---------V------
Sporosarcina newyorkensis	WP_009496676	-----LVK------H	--K----A--------
Sporosarcina sp. BI001-red	WP_116019182	-----LVK-------	--K----A-V------
Sporosarcina sp. D27	WP_025784512	-----LVK-------	--K----A-V------
Sporosarcina sp. P1	WP_099627606	-----LVK------H	-------A--------
Sporosarcina sp. P13	WP_099688971	-----LVK------H	--K----A--------
Sporosarcina sp. P16b	WP_099673868	-----LVK------H	-------A--------

	Sporosarcina sp. P17b	WP_099625218	-----LVK------H	-------A--------
	Sporosarcina sp. P18a	WP_099674776	-----LVK------H	-------A--------
	Sporosarcina sp. P19	WP_099690833	-----LVK------H	-------A--------
	Sporosarcina sp. P2	WP_099632305	-----LVK------H	-------A--------
	Sporosarcina sp. P20a	WP_099677045	-----LVK------H	-------A--------
	Sporosarcina sp. P29	WP_099661761	-----LVK------H	-------A--------
	Sporosarcina sp. P3	WP_099637778	-----LVK------H	----E--A--------
	Sporosarcina sp. P33	WP_081241967	-----LVK------H	-------A--------
	Sporosarcina sp. P34	WP_099695683	-----LVK------H	-------A--------
	Sporosarcina sp. P7	WP_099636265	-----LVK------H	-------A--------
	Sporosarcina sp. PTS2304	WP_114923781	-----LVK------H	--K----A--------
	Sporosarcina sp. ZBG7A	WP_039043500	-----LVK-------	--K----A-V------
	Sporosarcina ureae	WP_029053803	-----LVK------H	-------A--------
	Streptococcus pneumoniae	COD29893	-----LVK----Y-I	-KKVE----V---T--
**Other Bacteria (0/>200)**	Tenuibacillus multivorans	WP_093856977	-----LVK------I	E-KVE----V---T--
	Thalassobacillus cyri	WP_093046350	-----LVKR-----I	--KVE--A-V---T--
	Thalassobacillus devorans	WP_085508532	-----LVKR-----I	--KVE--A-V---T--
	Thalassobacillus sp. TM-1	WP_062446043	-----LVKR-----I	--KVE--A-V---T--
	Vagococcus carniphilus	WP_126795608	----VLVK------L	S-K-E----V------
	Vagococcus fluvialis	WP_086951483	----VLVK------L	N---E----V------
	Vagococcus martis	WP_079348256	-----LVK------L	TK-VE----V---T--
	Vagococcus sp. AM17-17	WP_118249672	-----LVK------L	TK-VE----V---T--
	Vagococcus sp. SS1994	WP_125955566	-----LVK------L	TK-VE----V---T--
	Vagococcus teuberi	WP_071456798	-----LVK------L	TK-VE----V---T--
	Virgibacillus dokdonensis	WP_077703796	------VK------I	--KVE--A-V---T--
	Virgibacillus indicus	WP_094883805	------VK------I	--KVE--A-V---T--
	Virgibacillus profundi	WP_095655597	------V--I----I	--KVE--A-V---T--
	Virgibacillus sp. SK37	WP_040955561	------VK------I	--KVE--A-V---T--
	Viridibacillus arvi	WP_053415460	-----LVK-I--M-L	-K--E--A-V------
	Viridibacillus sp. FSL H8-0123	WP_076065020	-----LVK-I--M-L	-K--E--A-V------
	Viridibacillus sp. OK051	WP_100797153	-----LVK-I--M-L	-K--E--A-V------

Supplemental Figure 57

A partial sequence alignment of the DNA topoisomerase IV subunit A protein containing a one amino acid insertion (boxed) that is exclusively shared by all members belonging to the Solibacillus clade and absent in all other bacteria. The peripherally branching *Lysinibacillus odysseyi* does not share this CSI.

Group	Species	Accession	242		297
Solibacillus clade (6/7)	Solibacillus isronensis	WP_079526835	PLIEQVKATGYTGQ	V	EVAYTSPVISTHTG
	Solibacillus silvestris	OBW60427	--------A-----	-	---F----------
	Solibacillus kalamii	OUZ39439	--------A-----	-	---F----------
	Solibacillus sp. R5-41	WP_099423520	-----I-----EKE	I	--------------
	Bacillus cecembensis	WP_057985088	-----I-----DKE	I	-I------------
	Lysinibacillus sp. 2017	WP_108712655	-----L--A--N-E	-	-I-F----------
	Lysinibacillus odysseyi	WP_036158725	--L-L--E--F-NI		DI-F---I------
Other Bacteria (0/>100)	Lysinibacillus meyeri	WP_107840485	--LDLIQS--FNDS		-I-F----------
	Lysinibacillus fluoroglycofenilyticus	WP_107942794	--LDLI-S--FDNA		-I-F---I------
	Bacillus ndiopicus	WP_042475505	--LDLI-S--FDNA		-I-F---I------
	Bacillus sp. B14905	EAZ86861	--K-LIEG--FNDV		-I-F---I------
	Bacillus sp. FJAT-22090	WP_053589636	--MKLIEE--FKNI		Q-SF----------
	Bhargavaea cecembensis	WP_040228568	--KALIEES--EDV		KLMF----------
	Bhargavaea ginsengi	WP_092050979	--KQMIEED-FQDV		KLMF----------
	Caryophanon latum	WP_066464294	--K-L-EQ--FKNV		DI-F-C-T------
	Chryseomicrobium excrementi	WP_100352552	--K-AIE-S-FRDV		KMS--T--------
	Kurthia sibirica	WP_109304991	--A-LI-QS-FNDL		KFGF---I------
	Lysinibacillus acetophenoni	WP_097148849	----KI-E--FDNV		--KF---I------
	Lysinibacillus boronitolerans	WP_016993321	--KALIEE--FNDV		-I-F---I------
	Lysinibacillus chungkukjangi	WP_107936111	----L-ES--IKDI		-IKF---I------
	Lysinibacillus composti	WP_124762954	---GLIES--FKDV		-IKF---I------
	Lysinibacillus contaminans	WP_053583255	--KALIEE--FNNV		-I-F---I------
	Lysinibacillus endophyticus	WP_121215393	--K-LIES--FN-V		-IKF---I------
	Lysinibacillus fusiformis	WP_025117229	--K-LIEG--FNEV		-I-F---I------
	Lysinibacillus halotolerans	WP_122971729	--KQLIES--FHHI		-TKF---I------
	Lysinibacillus jejuensis	WP_108306803	--K-KIE----DNV		R-QF---IV-----
	Lysinibacillus macroides	WP_053994770	--K-L-EG--FQDV		-I-F---I------
	Lysinibacillus manganicus	WP_036185676	----KI-TS-FENI		--KF---I------
	Lysinibacillus mangiferihumi	WP_107896149	--K-L-EG--FDEV		-I-F---I------
	Lysinibacillus massiliensis	WP_036178987	--K-LIEQS-FNEV		-IKF---I------
	Lysinibacillus parviboronicapi	WP_054768374	--K-LIES--FDEV		-I-F---I------
	Lysinibacillus saudimassiliens	CEA05394	--K-KIE----HDV		R-QF---IV-----
	Lysinibacillus sinduriensis	WP_036198485	----MIQS--FKNV		-IKF---I------
	Lysinibacillus sp. AR18-8	WP_066036831	--KALIEE--FNDV		-I-F---I------
	Lysinibacillus sp. B2A1	AVK85197	--KALIEG--FNDV		-I-F---I------
	Lysinibacillus sp. BF-4	WP_036146680	--K-KIE----HDV		R-QF---IV-----
	Lysinibacillus sp. BK089	WP_132358007	--K-LIEG--FNEV		-I-F---I------
	Lysinibacillus sp. FJAT-14222	WP_053592762	--K-LIEG--FNEV		-I-F---I------
	Lysinibacillus sp. FJAT-14745	WP_053485924	--K-LIEG--VKEV		-I-F---I------
	Lysinibacillus sp. LK3	WP_048395696	--KALIEE--FNDV		-I-F---I------
	Lysinibacillus sp. Marseille-P	WP_106778869	-----IQS--FEHV		--KF---I------
	Lysinibacillus sp. OL1	WP_131520299	--KALIEE--FNDV		-I-F---I------
	Lysinibacillus sp. SYSU K30002	WP_126657868	--M-LI-S--F-DI		--KF---I------
	Lysinibacillus sp. YLB-03	WP_118875973	-----IHT--FSDV		--KF----------
	Lysinibacillus sp. YR326	TDU95316	--K-LIEG--FNEV		-I-F---I------
	Lysinibacillus sp. ZYM-1	WP_054611840	--K-LIEG--FDEV		-I-F---I------
	Lysinibacillus sphaericus	POZ56319	--K-L-EG--FDEV		-I-F---I------
	Lysinibacillus sphaericus C3-4	ACA40267	--K-LIEG--FDKV		-I-F---I-T----
	Lysinibacillus sphaericus CBAM	EWH32297	--K-LIEG--FDKV		-I-F---I------
	Lysinibacillus telephonicus	WP_126295922	--KQLIES--FNEV		-IKF---I------
	Lysinibacillus xylanilyticus	WP_049665506	--K-LIEG--FNDV		-INF---I------
	Lysinibacillus xyleni	WP_097074004	---DL--SS-FNDV		--KF---I------
	Paenisporosarcina indica	WP_075617767	--KKLIEE--FPNV		Q--F---I------
	Paenisporosarcina quisquiliaru	WP_090561459	--MNLI-ES-FLDI		Q-SF----------
	Paenisporosarcina sp. OV554	WP_108585803	--KKLIEES-FNDV		K--F---I------
	Planococcus antarcticus	WP_006831503	--R-KI-G--F-DI		KFDF-T--------
	Planococcus donghaensis	WP_065526195	--R-KI-E--FSDI		QFDF-T--------
	Planococcus donghaensis MPA1U2	EGA88966	--R-KI-E--F-DI		QFD--T--------
	Planococcus faecalis	WP_071154570	--R-KI-E--FSDI		QFDF-T--------
	Planococcus halocryophilus	WP_008497527	--R-KI-E--FDDI		QFDF-T--------
	Planococcus halotolerans	WP_112222572	--R-KIIES-FKDI		DFDF-T--------
	Planococcus kocurii	WP_058385233	--R-KI-E--FSDI		QFDF-T--------
	Planococcus maritimus	WP_068462514	--R-KI-E--F-DI		RYDF-T--------
	Planococcus plakortidis	WP_068869238	--R-KI-E--F-DI		RYDF-T--------
	Planococcus sp. PAMC 21323	WP_038704012	--R-KI-E--F-DI		QFDF-T-I------
	Planococcus versutus	WP_065524589	--RKKI-E--F-DI		QFDF-T-I------
	Planomicrobium glaciei	WP_036803188	---QELE-A-F-DI		KMD--T--------
	Planomicrobium okeanokoites	WP_084245654	--R-RIIES-FKDI		KFDF-T--------
	Planomicrobium sp. MB-3u-38	WP_101802961	--R-RIIES-FKDI		KFDF-T--------
	Planomicrobium sp. Y74	WP_121632039	--R-KIIES-FKDI		NFDF-T--------
	Psychrobacillus insolitus	WP_111438219	---KLIEQ--FKDI		K-SI----------
	Psychrobacillus psychrodurans	WP_093494767	--MNLI-ES-FLDI		Q-SF----------
	Psychrobacillus psychrotoleran	WP_093534389	--MNLI-ES-FLDI		Q-SF----------
	Psychrobacillus sp. FJAT-21963	WP_056829714	--MRLIEE--FKNI		Q-SF----------

**Other Bacteria (0/>100)**	Psychrobacillus sp. OK028	WP_093060986	---KLI-ES-FQDI	Q-SI----------
	Psychrobacillus sp. OK032	WP_093268755	--KNLIEE--FKQI	QMSF----------
	Rhizophagus irregularis	PKC51297	--K-LIEQS-FNEV	-IKF---I------
	Rummeliibacillus sp. TYF005	WP_124218510	--KRMIEES--QNV	KLGF---I------
	Rummeliibacillus stabekisii	WP_066788113	--KTRIEE---HDV	KFG----I------
	Sporosarcina koreensis	WP_060210452	--LQL-EES-WKDV	KFSF---I------
	Tetzosporium hominis	WP_094941948	--K-AIE-S-FRDV	KMSF-T--------
	Viridibacillus arvi	WP_053417024	--KK-LEES--MDT	KF-----I------

Supplemental Figure 58

A partial sequence alignment of the DegV family protein containing a one amino acid insertion (boxed) that is exclusively shared by all members belonging to the Solibacillus clade and absent in all other bacteria. The peripherally branching *Lysinibacillus odysseyi* does not share this CSI.

Group	Organism	Accession	206		248
Solibacillus clade (6/7)	Solibacillus sp. R5-41	WP_099422418	PNNLDELGYTEENIVTNLQGANR		QVGIQKGALEMSNVDVSKEM
	Bacillus cecembensis	WP_057986779	----A------------------		----------A---------
	Lysinibacillus sp. 2017	WP_108711546	-Q---------AE-L--------		---------------L-Q--
	Solibacillus silvestris	WP_065216607	-E---A--F-QAQVL-D------		--------------------
	Solibacillus kalamii	WP_087615658	-E---A--F-QAQVL-D------		--------------------
	Solibacillus isronensis	WP_079524658	-E---A--F-QAQVL-D------		--------------------
	Lysinibacillus odysseyi	WP_036150777	-S--A----A-GDVL-Q---QT-	N	E-SM-I---------L----
Other Bacteria (0/>100)	Bacillus ndiopicus	WP_042470929	-T-VAA----QAD-L-D----D-	A	RI-LNIQ--------I----
	Lysinibacillus meyeri	WP_107839955	-T--AA----QAD-L-D------	G	GI-LSIQT-------I----
	Lysinibacillus fluoroglycofeni	WP_107942047	-T--AA----QAD-L-D----G-	A	EI-LSIQT-------I----
	Bacillus aquimaris	WP_044339972	-V---G--V--DE----VL-RG-	A	DISMIQNS--A----I----
	Bacillus asahii	WP_119115760	-D-Y-G--V-ADDVL-E-T--S-	T	DISMKQ----S----L----
	Bacillus boroniphilus JCM 2173	GAE46682	-D--A---AI--E-M---A-PL-	N	-IA--Q-V--Q----M----
	Bacillus butanolivorans	WP_053348508	-D-MRG--V--DD-M-A-T-DS-	S	EIAVSQ-V--S----L----
	Bacillus canaveralius	WP_101576029	-E-MGQ--VA--E-F-E-N-PL-	N	EIS-KQ----Q---EM----
	Bacillus jeotgali	WP_079506442	-E--A---VD-AD-L-V-N-QQ-	N	R-S--Q-V--Q----M----
	Bacillus kribbensis	WP_026696023	----NS--VSANEVY-D-N-GL-	N	TISL-Q----S----L----
	Bacillus kwashiorkori	WP_062351648	--HF---TLPV-E--ME-N--E-	G	EIN-AHQ---Q-----AE--
	Bacillus lentus	WP_066144053	-E--QPGVNPALIYTELTGAERN		--AV-QK---A----L----
	Bacillus loiseleuriae	WP_049683302	-A--NQMNVNA-D-L-S-T-GL-	G	EISMTQ----N----L----
	Bacillus marisflavi	WP_121617636	-V-M-NTAV--QE-L---E--G-	N	GIQLSQ-T--A----MG---
	Bacillus massiliogabonensis	WP_102273643	-E-MA--NVPMNE-L-E-N-PM-	N	-IS--QR---Q----M----
	Bacillus massilionigeriensis	WP_075983153	-Y-YA--DA-V-D-L-E-N-PL-	E	-I---GS---Q----L----
	Bacillus massiliosenegalensis	WP_019153926	-P-F---NVGLDE-L-D-A-PL-	T	EI---QR---S----MA---
	Bacillus mediterraneensis	WP_071460946	-D-FAG--LN--D-F---A-GE-	G	AIS--Q----Q----L----
	Bacillus methanolicus	WP_003346760	-E-FA---VLKQD-L-E-N-PL-	A	-IAMKQ----Q----L----
	Bacillus muralis	WP_057911934	-E-INN--V-VDD-M-S-T-DS-	D	-IAVRQ----S----L----
	Bacillus oleivorans	WP_097156855	-D--A---VEL-E-L-PMN--L-	E	NIAM-Q----Q----L-V--
	Bacillus praedii	WP_057760235	-E-MA--NVPVNE-L-E-N-PM-	N	--S--QR---Q----M----
	Bacillus selenatarsenatis	WP_041964564	-D--A---V---EVM---A-PL-	N	-IA--Q-V--Q----M----
	Bacillus simplex	WP_034315759	-K-FNA--V--VD-M-A-T-DS-	N	RAAVRQ-T--S----L----
	Bacillus sp. 17376	ESU34330	-D--A---AI--E-M---A-PL-	N	-IA--Q-V--Q----M----
	Bacillus sp. AFS015802	WP_098351527	AD------V--DEV---VT-GA-	S	DISMVQNS--G----I----
	Bacillus sp. AFS017274	WP_098371634	-K-FNA--V--DD-M-A-T-DS-	N	RAAVRQ-S--S----L----
	Bacillus sp. M6-12	WP_101591975	-A-----NV---DVL-S-T-GG-	D	EISL-QR---T----L----
	Bacillus sp. MUM 13	WP_071352314	-A--NR--AAPSD-M-S-T-GL-	S	NISM-Q----T----L----
	Bacillus sp. OK838	WP_089362010	-E-IN--DV-DDD-M-A-T-DS-	N	-IA-RQ-T--S----L----
	Bacillus sp. OV322	WP_090988631	-A--NR--AAPAD-M-S-T-GL-	S	NISM-Q----T----L----
	Bacillus sp. OxB-1	WP_041073165	-E--AD--VN-AD-L-EI---A-	N	GI-L-NQ---A---NYE---
	Bacillus sp. P14.5	WP_113928299	-Q---G--VN-DE-L--MN--A-	N	NIAAAQSS--G----TG---
	Bacillus sp. RJGP41	WP_106027239	-K-FNA--V--VD-M-A-T-NS-	N	-AAVRQ-T--S----L----
	Bacillus sp. SG-1	WP_006838364	-DD-QGF-VN-NE-L-S-E--L-	D	NI-LAQST--G----IG---
	Bacillus sp. SKP7-4	WP_119546112	-DD-QG--V--DE-L--MN--L-	N	NI-LAQST--G----IG---
	Bacillus sp. Soil745	WP_057278174	-K-FNA--V--DD-M-A-T-DS-	N	-AAVRQ-T--S----L----
	Bacillus sp. Soil768D1	WP_057216044	-D-MRG--V--DD-M-A-T-DS-	S	ELAVRQ-V--S----L----
	Bacillus sp. T33-2	WP_101583849	-D--AG--VA-ND-L---D-QL-	N	GIS--Q----Q----L----
	Bacillus sp. V3-13	WP_101661800	-E-M-Q--VA--E-F-E-N-PL-	N	-I---Q----Q---EM----
	Bacillus sp. V44-8	WP_117324366	-A-F-D--IDQ-D-M-A-E-GL-	Q	DISMRQ----N----L----
	Bacillus sp. V47-23a	WP_117327700	-A-M---AVA-G----A-T--A-	G	EISL-QRS--A----L----
	Bacillus sp. V5-8f	WP_101631371	-A-F-Q--IERGD-M-P-D-GL-	Q	EISLRQ----S----L----
	Bacillus sp. V59.32b	WP_117308861	-A-I-Q--VG--D-M-A-N-GL-	D	DISLRQ-V--S---EL----
	Bacillus sp. XXST-01	WP_126408083	-E-FA--NVGVDE-L-DMV-PL-	G	EI---SH---S----MA---
	Bacillus sp. es.034	WP_098438740	AD------V--DEV---VT-GA-	T	DISMVQSS--G----I----
	Bacillus sp. mrc49	WP_100533008	-E-I-G-DV-VDD-M-A-T-DS-	N	-IAVRQ----S----L----
	Bacillus subterraneus	WP_044396291	-E--AA--V--GEVI-D-T-PL-	N	-IA--Q-V--Q----M----
	Bacillus vietnamensis	WP_060672843	AD------V--DEV---VT-GA-	S	DISMVQNS--G----I----
	Bacillus zeae	WP_119113829	-D---A--V--ND-I-EMA-PE-	G	GIS-KQ----Q---EM----
	Butyricicoccus sp. 1XD8-22	RKJ59670	-E-FA---LE-QEVLVDML----	V	NI-V-N----Q---NL----
	Caldibacillus debilis	OUM84031	-E-FA--NVP-GE-L-----EA-	I	GI-LEQQ---Q----IGE-L
	Caldibacillus debilis GB1	RKO62817	-E-FA--NVP-GE-L-----GA-	I	GI-LEQQ---Q----IGE-L
	Caryophanon latum	WP_066466374	-A-FE----N--DVLID---QA-	G	AIAL-NN---A-----G---
	Caryophanon tenue	WP_066546912	-T-FE----N-ADVAVQ-E-QA-	G	AIAV-NNV--S----M---L
	Falsibacillus pallidus	WP_114745954	-Q-MNG--VSAND-Y-EMT----	A	AISMRQ-V--Q----MG---
	Falsibacillus sp. GY 10110	WP_121680923	-E-MNS--VKAND-Y-DMT-N--	G	SIA--Q-S--Q----L----
	Filibacter sp. TB-66	WP_124068815	-A-MA---V--AD-M-EM---A-	S	VI-LENN---A----YQ---
	Jeotgalibacillus campisalis	WP_041061694	-A------VDPGEVLVQ-E--L-	G	GI-LEQ----Q----L-QSF
	Jeotgalibacillus proteolyticus	WP_104058571	-E-AE---IDPAEA-VQME-GL-	L	N--LEQ----Q----L--SF
	Jeotgalibacillus sp. S-D1	WP_133376816	-D------AERGDVLVQAE--L-	N	-I-LEQ-S--Q----L--AF
	Kurthia massiliensis	WP_010287030	-D-F---DINIDDVL-D-T-EE-	S	-I-L-N----Q----M-A--
	Kurthia senegalensis	WP_010302401	-S-F---KVDAKDVYS--T-NA-	A	NI-M-N-V--G----L----
	Lysinibacillus boronitolerans	WP_016991805	----A-----QAEVL-E-V----	Q	-I-M-N-T------NL----
	Lysinibacillus chungkukjangi	WP_107932528	-A--N----NQAD-HID----A-	T	E---TS-S--L---NL-T--
	Lysinibacillus composti	WP_124763349	-S-M----F-ANEVL-D---IG-	A	EI--TN-S------NL----
	Lysinibacillus contaminans	WP_053585280	----A-----QAEVL-E------	Q	-I-M-S-M------------

Group	Species	Accession	Sequence
Other Bacteria (0/>100)	Lysinibacillus endophyticus	WP_121214028	-G--EQ--LN-ADLY-----GL- T EI-LAN-K-------I----
	Lysinibacillus fusiformis	WP_004269488	----A-----QAEVL-E-V---- Q -I-M-S-T------NL----
	Lysinibacillus halotolerans	WP_122971480	-A-F-----N--DVL-----GA- V NI-MMN----S----L-T--
	Lysinibacillus jejuensis	WP_108306022	-D--N---V--AELL-P-R--G- Q E-NV-Q-V--------GR--
	Lysinibacillus macroides	WP_053993105	-A--A-----QAEVL-E-A-VD- Q -I-M-S-T------NL----
	Lysinibacillus mangiferihumi	WP_107897830	-D--A-----QAEV--E------ Q -I-M-ND---L---SL----
	Lysinibacillus massiliensis	WP_036171328	-E-FA---LE-QEVLVDML---- V NI-V-N----Q---NL----
	Lysinibacillus odysseyi	WP_036150777	-S--A----A-GDVL-Q---QT- N E-SM-I---------L----
	Lysinibacillus parviboronicapi	WP_107923650	----A-----QAEVL-E------ Q -I-M-S-M------NL----
	Lysinibacillus saudimassiliens	CEA01854	-Q-MN---V--ADLL-P-R--G- A --NV-Q-M--------GR--
	Lysinibacillus sinduriensis	WP_036200119	-A--G----N-AD-LVD---V-- D E---TN-S--L---NL-T--
	Lysinibacillus sp. B2A1	AVK82706	----AQ----QAEV-AE------ Q -I-M-S-T------NL----
	Lysinibacillus sp. BF-4	WP_036145632	-Q-MN---V--ADLL-P-R--G- T --NV-Q-M--------GR--
	Lysinibacillus sp. BK089	WP_132360386	----AQ----QAEV--E-R---- Q -I-M-N-S------NL----
	Lysinibacillus sp. FJAT-14222	WP_053596432	----AQ----QAEV--E-R---- Q -I-L-S---------L----
	Lysinibacillus sp. FJAT-14745	WP_053484431	----AQ----QAEV--E------ Q -I-M-S-S------NL----
	Lysinibacillus sp. Marseille-P	WP_106783554	-T-FA----D-AEVIND----A- E -I--MNR---Q---NL----
	Lysinibacillus sp. OL1	WP_131520799	----A-----QAEVL-E-V---- Q -I-M-S-T------NL----
	Lysinibacillus sp. SYSU K30002	WP_126658924	-T------L--ADLY-D-L--A- T ---MTNQKI-----------
	Lysinibacillus sp. YLB-03	WP_118874999	-D-FNGM--NQAD-LVD----A- T E--MMNR---S----I----
	Lysinibacillus sp. YR326	WP_134020541	-D--AQ----QAEV--E-R---- Q -I-M-S-S------NL----
	Lysinibacillus sp. ZYM-1	WP_054611271	-DD-A---F-QAEVL-Q-V---- Q -I-M-S-T------NL----
	Lysinibacillus sphaericus	WP_010858007	----A----NQAEVL-E------ Q -I-M-G-T------NL----
	Lysinibacillus telephonicus	WP_126295540	-A--N----NQGDVL------A- N EI--A--K-------L----
	Lysinibacillus xylanilyticus	WP_049668037	-D--AQ----QAEV--E-R---- Q -I-M-S-S------NL----
	Lysinibacillus xyleni	WP_097071700	-T------LN-ADLY-----GL- T EI--AN-K-------I----
	Paenisporosarcina quisquiliaru	WP_090565596	-D--E---LNA-D-M-D-L--D- G EIAMANRM--L----T----
	Paenisporosarcina sp. K2R23-3	WP_119882590	-D--N---V-AQD-L------D- A -I-LGN-Q--Q---NM---F
	Psychrobacillus insolitus	WP_111439337	--------V-QQDVL-D---EE- N EISLVN-I--L----I----
	Psychrobacillus psychrodurans	WP_093493850	-D--E---LNA-D-M-D-L--D- G EIAMANRM--L----T----
	Psychrobacillus psychrotoleran	WP_093538701	----E---LNA-D-M-D------ E EIA-AN-M--L----T----
	Psychrobacillus sp. FJAT-21963	WP_056828035	----A---VNAQDVIID----E- G EIALTNQV-------M----
	Psychrobacillus sp. OK028	WP_093061298	----EQ--LNA-D-M-D------ G EIA-AN-M--L----T----
	Psychrobacillus sp. OK032	WP_093267920	-T------VNADDVM-D------ G EIAVANQV-------I----
	Rummeliibacillus pycnus	WP_102693078	---FNQ--VAQGTVLQ------- N --AM-N-G--L----I----
	Rummeliibacillus stabekisii	WP_066791760	-E-FG-F-VQ-ASVLQ----G-- N -IAM-N-G--A----I----
	Sporosarcina koreensis	WP_040287536	-D------V--AD-L-E-T--AE S EIA-RQ----G----YQ---
	Sporosarcina pasteurii	WP_115359950	-R-MA---V---E-F------D- N II-L-N-V--T---HYE---
	Sporosarcina psychrophila	WP_067213083	-E--AD--V-QQQVL-E----A- N VI-LEGQ---A---NSQ---
	Sporosarcina sp. HYO08	WP_067405574	---FAQ--IR--EVAI----EQ- Q MI-LEN----G---N-Q---
	Sporosarcina sp. P1	WP_099627797	-TD-AA--L-AND-L-EMT--R- N -I-L-NQ---T---EYE---
	Sporosarcina sp. P16b	WP_099672255	-TD-AAI-L-ANDLL-E-T--G- N -I-L-NQ---T---EYE---
	Sporosarcina sp. P17b	WP_099625053	-TD-AA--LAANDLL-EMT--G- N -I-L-NQ---T---EYE---
	Sporosarcina sp. P18a	WP_099675542	-TD-AAI-L-ANDLL-E-T--G- N -I-L-NQ---T---EYE---
	Sporosarcina sp. P19	WP_099691261	-TD-AAI-L-ANDLL-EMT--G- N -I-L-NQ---T---EYE---
	Sporosarcina sp. P2	WP_099631268	-TD-AAI-L-ANDLL-E-T--G- N -I-L-NQ---T---EYE---
	Sporosarcina sp. P20a	WP_099679038	-T--AA--V-ANDLL-EMT--G- D -I-L-NS---T---EYE---
	Sporosarcina sp. P21c	WP_099628229	-TD-AA--L-ANDVL-EMT--R- N -I-L-NQ---T---EYE---
	Sporosarcina sp. P26b	WP_099693372	-TD-AA--LVANDLL-EMT--G- N -I-L-NQ---T---EYE---
	Sporosarcina sp. P3	WP_099638850	-TD-AA--L-ANDVL-EMT--G- S -I-L-NR---T---EYE---
	Sporosarcina sp. P33	WP_081243875	-ED-AA--V-ANDLL-EMT--G- E -I-L-NQ---T---QFE---
	Sporosarcina sp. P34	WP_099695410	-TD-AAI-L-ANDLL-E-T--G- S -I-L-NQ---T---EYE---
	Sporosarcina sp. P7	WP_099637240	-TD-AAI-L-ANDLL-E-T--G- N -I-L-NQ---T---EYE---
	Sporosarcina ureae	WP_029053432	-TD-AAI-L-ANDLL-EMT--G- S -I-L-NQ---T---EYE---
	Ureibacillus thermophilus	QBK25270	-A-F-----D-ND-L---V-IV- E NI-LENQK------NL----
	Ureibacillus thermosphaericus	WP_016839309	-A-------D-ND-L---V-FG- E SI-LENQK------NLT---
	Vibrio vulnificus	WP_133350988	-K-FNA--V--DV-M-A-T-DS- N -AAVRQ-T--S----L----
	Viridibacillus arvi	WP_053417876	-T--N---V--AEVLR-----D- A EI-L-N-S-------T----
	Viridibacillus sp. OK051	WP_100794819	-D--N---V--ADVLRD----D- A EI-L-N---------T----

Supplemental Figure 59

A partial sequence alignment of the flagellar hook-basal body protein A containing a one amino acid deletion (boxed) that is exclusively shared by all members belonging to the Solibacillus clade and absent in all other bacteria. The peripherally branching *Lysinibacillus odysseyi* does not share this CSI.

Group	Organism	Accession	233	Insertion	286
Solibacillus clade (6/6)	Solibacillus kalamii	WP_087615584	LPMEDPQMDLLEGSVFYRAAMTYDKI	KNELQK	LRFERGIDIEEEPRVHLEVNYRATNRDLLQIE
	Solibacillus isronensis	WP_079524758	--------------------------	------	-----------------------------H--
	Solibacillus silvestris	WP_065216524	-------------C------------	------	-----------------------------H--
	Solibacillus sp. R5-41	WP_099422539	--F-NE-L-K---A-------S-E-L	QH--H-	IQ--QN-E----Q-I---M---S--S--H---
	Bacillus cecembensis	WP_057984300	--F--A-R-K---A-------S-E-L	QH--K-	IQV-NN-T----Q-I--DM---SKHP--YH--
	Lysinibacillus sp. 2017	WP_108711657	---D--TV-K-----------S-E-L	QH-I--	-------E--V---I--QT---SMHS---HV-
Other Bacteria (0/22)	Bacillus ndiopicus	WP_042471198	--FD-V-DAADSQAL-H---F----L		MD-ATQHH--V-N----S--H-T-QL---H--
	Lysinibacillus fluoroglycofenilyticus	WP_107942145	--F--V-DAADPQAL-H---F----L		ID-ATQHH--V-N----A----TAQA---H--
	Lysinibacillus meyeri	WP_107840365	--FD-V-DAMDPQAL-H---F----L		MD-AAQHHL-I-N----MT-C-TQQS---H--
	Lysinibacillus sp. SYSU K30002	WP_126658318	--F-NE-DKDDDQA----P-VM---L		KQEAENFKV-M-Q-L---TC--YN-Q--YH--
	Lysinibacillus sp. YLB-03	WP_118874896	--F-NE-DKYN-QA----P-LM---L		-QEAYKFQ--V---I--DKT--FQ-N----V-
	Lysinibacillus massiliensis	WP_036176423	--F-NE-DKDDDQAL---S-VM---L		MDEAKQLQ--V------TG---FL-N--R---
	Butyricicoccus sp. 1XD8-22	RKJ43084	--F-NE-DKDDDQAL---S-VM---L		IEEAKQLQV-V------TG---FL-N--R---
	Ureibacillus thermosphaericus	WP_096550501	--F--E-DKHN-QA------L----L		KQEATKLG--I---I---TTH-YK----FHV-
	Ureibacillus thermophilus	QBK25404	--F-NENDKDN-QA-----SLM---L		RQEAVKLG--V---I-MQTT--YQ-F--QH--
	Lysinibacillus endophyticus	WP_121213908	--F-NE-DMYDDQA----P-VM---L		-QETANFKV-M------HTC--YN-N--QH--
	Lysinibacillus xyleni	WP_097071902	--F-ND-DKYDDQA----P-VM---L		-QETTNFKV-M------DTC--YN-H--AH--
	Lysinibacillus sinduriensis	WP_036198969	--F---NDMYDDQA----P--V---L		-QEAYKVQM-V---I---T-H-FH-H--QH--
	Lysinibacillus halotolerans	WP_122971030	--F--EKDKYNDQA----P-VM---L		-QEAYK-QL-I---I--DNC--FH-H--QHV-
	Lysinibacillus telephonicus	WP_126295461	--F--VNDKDDDQA----S-VM---L		IQEAHNFQ--I---I---NC--YH-Y--QHV-
	Lysinibacillus chungkukjangi	WP_107932759	--F---NDKYNDQA----P-VM---L		-QEAYKFQ-DV---I---NS--YQ-H--QHV-
	Lysinibacillus acetophenoni	WP_097147785	--L--EHDKDD-QAL---G-VM---L		-EEANNLQV-V---E--FGS--YN-L--KH--
	Lysinibacillus manganicus	WP_036182027	--L--EHDKDDDQAL-F-G-VM---L		MEEAKNLQVDI---E-FFGS--YN-L--KH--
	Lysinibacillus odysseyi	WP_036155700	--F-S-LDAQDTDAL------S---L		-SEAMQLGVDI--------GH-FE-K--QHV-
	Lysinibacillus composti	WP_124763701	--F--ENDKDD-QAL-H--SVM---L		MQEAHNLQ--I-D-E-MFGT--FH-Y----V-
	Lysinibacillus sp. Marseille-P	WP_106783804	--F--EHDKDN-QAL---P-LM---L		IDTA-DLAV-I---E--TGD--FN-K--SH--
	Planococcus rifietoensis	WP_058383372	---D-TSSIED-Q-L--QS-L-ASRL		TDLAATEG--L--E-Y-QQPK-FM-EE-RHV-
	Chryseomicrobium excrementi	WP_100352892	---DN--EAFDSQ-L--S--K-RQQL		VEVAQQHQVAV--D---

Supplemental Figure 60

A partial sequence alignment of the Helicase-exonuclease AddAB subunit AddB protein containing a six amino acid insertion (boxed) that is exclusively shared by all members belonging to the Solibacillus clade and absent in all other bacteria.

Group	Species	Accession	36		80
**Solibacillus clade (6/7)**	Bacillus cecembensis	WP_057988067	SLRYFFMLPFLIAIVAIRGK	NG	FRVLSNEVKAKPGAWLIWSFVGF
	Solibacillus silvestris	WP_014824884	--------------------	V-	--I--S-M-EH------------
	Solibacillus kalamii	WP_087617150	--------------------	V-	--I--S-M-EH------------
	Solibacillus isronensis	WP_079523851	-----------F--------	G-	--I---M-DH-----V-------
	Solibacillus sp. R5-41	WP_099422244	--------------------	--	----A----V-----F-------
	Lysinibacillus sp. 2017	WP_108711345	------------I---M---	G-	--NMA--M----YL--L------
	Lysinibacillus odysseyi	WP_036153456	---------------WM-KG		GKP-TK-LRQA-IP--G------
**Other Bacteria (0/>300)**	Bacillus ndiopicus	WP_042470442	---------------LY--N		Y-Q-H--M-SHSRQ--L------
	Lysinibacillus meyeri	WP_107840069	---------------LY-KN		Y-Q-H--M-TH-RQ-FV------
	Aeribacillus pallidus	WP_044899965	----L--V---L---WL--N		L-P-WR-MG-R-W---V------
	Alicyclobacillus acidoterrestr	WP_021297944	----L----I-LIL--A--R		Q-T-WS-MRRH-WQ-VW------
	Aneurinibacillus migulanus	CEH31434	----L--V---LL--MK-RN		LG--LTHMRRH--P-F----I--
	Anoxybacillus sp. B2M1	ANB56982	---FI--V---FL---S-KN		T---FQ-MRKQ-SS-FV--L---
	Anoxybacillus sp. P3H1B	KXG09646	---FI--V---FL---S-KN		T-A-FQ-MRKQ-SS-FV--L---
	Archangium gephyra	AKJ02939	-----I---LMSVL--AH-G		L-P-GA-LRRH--E--L--S---
	Archangium sp. Cb G35	OJT21647	-----I---LMSVL--AH-G		L-P-GA-LRRH--E--L--S---
	Archangium violaceum	WP_043408551	-----I---LMAVL--AH-G		L-P-GA-LRRH--E--L--S---
	Bacillus abyssalis	WP_078409389	----L--I-L-FV---G-KN		--P-LQVM-EN--R-VL------
	Bacillus acidiproducens	WP_051086239	---V--V---LV--LA-N		-Q--WK-M-KH-FQ--L---F--
	Bacillus aciditolerans	WP_121445288	-----V----G--L---N		LKE-LH-L-TN-VT-IL--TI--
	Bacillus akibai	WP_035663625	---FI--V-----L-LW-N		-KP-WK-LMSN-KP---------
	Bacillus alcalophilus	WP_003323307	---FL--I---VL--SL--G		LGP-FA-L-KA-IY---------
	Bacillus alveayuensis	WP_044748124	----I--V---LL--MA-N		V-S-LQVMREQ----IL---I--
	Bacillus aryabhattai	WP_045295013	-------V-L-LVL-YFW-G		IKPVVA-I--Q-QK--L--T---
	Bacillus aryabhattai B8W22	SDC24974	-------V-L-LVL-YFW-G		IKPVVA-I--Q-QK--L--T---
	Bacillus azotoformans	WP_035192342	----I--I---LL--G---N		MKPVFE-M-K--FE--L--TI--
	Bacillus badius	WP_041095132	----L--I-L-AV--GS-KG		M-P-LM-M-RN-F---L--T---
	Bacillus bataviensis	WP_007087196	-------I---FI--IA-N		LKP-LL-M-Q-----VL--SI--
	Bacillus butanolivorans	WP_083450928	----I--V---VC--LS-N		L-P-LQVMRER--P--L------
	Bacillus camelliae	WP_101354382	----I--I----C---L--N		LKGVFK-L--S--S--L------
	Bacillus campisalis	WP_046523240	-------V---L---LA-N		LSS-IKVM-EQ-KS--L------
	Bacillus cereus	WP_098339504	----Y--V-L--M--MY--N		LKQ-FQYM-SN-KE--L--I---
	Bacillus cihuensis	WP_028393100	----I--V---LI--IS-N		L-P-LKAM-EQ-KT--L------
	Bacillus circulans	SPT78635	---FL------LV--Y--TG		-KA-WLS-RKN-FP-FG------
	Bacillus clausii	WP_035203686	---FL------LV--Y--TG		-KA-WLS-RKN-FP-FG------
	Bacillus clausii KSM-K16	BAD65986	---FL------LV--Y--TG		-KA-WLS-RKN-FP-FG------
	Bacillus coagulans	KWZ81000	----IL-V---LV--LA-N		-QA-WA-M-KH-AG--L---T--
	Bacillus cucumis	WP_101649325	-------I----I--IG-KN		LKP-LL-M-KQI----L--T---
	Bacillus dakarensis	WP_077210863	----I--I---LI--IW--N		LKQ-LKVMRE--V---S------
	Bacillus deserti	WP_101642425	----I--I---AL--MVQKG		L-P-LKIM-E---S--L-G----
	Bacillus dielmoensis	WP_042455662	-------I---FI--IT-N		LKP-WR-M-N--V---L------
	Bacillus drentensis	WP_066256416	-------V---FV--LG-KN		LKP-LA-M-NN--V-VL--T---
	Bacillus eiseniae	WP_066189138	-------I-M-LI---L-RN		L-QVFT-MRRA--K-VL---I--
	Bacillus farraginis	WP_058004443	-------I---LI--LW-N		LKD-LR-M-KH-VQ--L--C---
	Bacillus firmus	WP_035327982	-------I-L-G---IF--N		L-P-FT-M-KA--Q--L---I--
	Bacillus foraminis	WP_121613222	-------I---L---MV-RN		LQH-IKAM--R-L--ML------
	Bacillus fordii	WP_018706293	----M--V---LM--L---N		L-K-IQ-M--S-RE--L--TI--
	Bacillus fortis	WP_120071166	----I--V---LV--MV--N		--R-IQ-M--N--E--L--T---
	Bacillus freudenreichii	WP_126431281	----I--I---LI--FF--N		L-P-FQ-MR-R--Q--L--T---
	Bacillus fumarioli	WP_066368722	-------I---FL--LA-N		LKP-LH-M-RQ-LP-----S---
	Bacillus funiculus	WP_129728397	A------I-L-LV--GV---		LTS-FLTM-KQ-FQ-F----I--
	Bacillus ginsengihumi	WP_025731611	----I--V----L--IS-N		-LA-LKIM-E--LS--C------
	Bacillus gottheilii	WP_066442317	-------I-L--I--SF---		LKPVFT-L-NA-RK-ML--TI--
	Bacillus horikoshii	WP_064099099	-F---V---LL--W---N		LGQ-FR-MRSA-L---L---T--
	Bacillus horneckiae	WP_066398294	----I--V---LVL-IG-RN		LKP-LQ-M-R---R---------
	Bacillus indicus	WP_029280683	---FL--I---VL---L-NS		-Q-FS-M-DQ-SK-VL---T--
	Bacillus jeotgali	WP_079509184	----I--V---V------N		LKP-IHTLFRN-YP--L------
	Bacillus kochii	WP_095371570	-------I-L-LI--LY-KS		L-A-MI-L-KA--K-ML--TI--
	Bacillus kribbensis	WP_026695229	----I--V---LG--IGQK-		L-G-LRVM-EH--L--L------
	Bacillus ligniniphilus	WP_017729348	-------V---LIL-LW-RN		-YQ-WL-M-KN-KP---------
	Bacillus loiseleuriae	WP_049680462	----I--V---LI--VA-RN		L-P-LKAM-EQ-KT--L---I--
	Bacillus marisflavi	WP_121620291	--I--V---MV--ML--N		L-P-IK-MI-R-SI--F------
	Bacillus marmarensis	WP_022626800	---F---V---LL--WL--G		LAD-FK-M-KN-RE-F----F--
	Bacillus massiliglaciei	WP_110926190	----M--V---LC--WA--N		L-EVFR-MLSR--K--L------
	Bacillus massiliogabonensis	WP_102273599	----L--V---LI--MF-RN		LKP-FL-M-R---I--V---I--
	Bacillus massiliogorillae	WP_042350514	-------VI--LI--GA-N		LHI-FH-M-KA-TS-I---T---
	Bacillus massilionigeriensis	WP_075981729	------V---LV--LW-N		-HS-WL-M-KQ-W---L--TI--
	Bacillus massiliosenegalensis	WP_019155282	---FI--I---LM--RRKN		LKP-FHMM-EN-S--ML------
	Bacillus mediterraneensis	WP_071458588	----I--V---LC--IM-N		L-P-LT-MRKQ--K-IL------
	Bacillus megaterium	AUO10665	-------V-L-LVL-YFW-G		IKPVVA-I--H-QK--L--T---
	Bacillus mesonae	WP_066384232	-------V---FI--MA-N		LKP-FV-M-NA--T--L------
	Bacillus persicus	WP_090746946	----L--V----I--LA-N		L-P-I-VM-EQ-KS--L---I--
	Bacillus praedii	WP_057760146	----L--V---LM--VC-RN		LKP-LL-M-RR--V--L---I--
	Bacillus pseudalcaliphilus	KMK75946	----L--I----VL--MK-G		LKPIIV-I-KA-LQ-FL---I--

Other Bacteria (0/>300)

Bacillus pseudofirmus	WP_012958092	---F---V---LL--WL--G	LAD-FK-M-KH-RE-F----F--
Brevibacillus borstelensis	WP_003391774	----L--V---LL------N	LGP-WQ-MRKH-WQ--L------
Brevibacillus centrosporus	WP_122961168	----L--V---LV--GM-RG	LQP-WE-M--T-WQ-GV------
Brevibacillus choshinensis	WP_055745395	----L--I---LL--GL--R	L-P-WV-MR-H-WQ-VV---I--
Brevibacillus nitrificans	WP_122923809	----L--V---LV--GM-RG	LQP-WE-M--N-WQ-GV------
Brevibacillus sp. CFH S0501	WP_134684281	-------I---LV--GM-NM	PQL-A -M--NWRE--L------
Brevibacillus sp. OK042	WP_092273728	----L--V---LV--GM-RG	LQP-WE-M--T-WQ-GV------
Brevibacillus sp. WF146	WP_065067283	----L--V---L---WL--N	L-P-WR-MG-R-W---V------
Brevibacillus thermoruber	WP_029098230	----L--V---L---WL--N	L-P-WR-MR-R-W---V------
Butyricicoccus sp. 1XD8-22	RKJ22689	-----------FI--FY-KG	WKPM---M-RQ-VQ-FV------
Caryophanon tenue	WP_066547427	-------V---VV---W-Q-	SAP-KQSM-THRTE-M---T---
Chryseomicrobium excrementi	WP_100353035	-------V---VI--WM--G	LPP-ARDMRTS--P-F-------
Dendrosporobacter quercicolus	WP_092074171	A-----T----L----F-RN	L-P-LE-LT-Q-A--I---T---
Desmospora sp. 8437	WP_009711530	---FL------MM--G----	LAG-WV-MRRH-V---G--L---
Desulfosporosinus meridiei	WP_014904833	---L--V-P-LL--I---N	-KP-LMDLGKR--V-----T---
Desulfosporosinus orientis	WP_014185207	----------VI--MT--N	LKP-LSALHSR-L---G--T---
Desulfosporosinus sp. HMP52	WP_034597510	---L--V-P-LL--MV--N	LKP-LLDLRKR--V-----T---
Domibacillus aminovorans	WP_063964460	----I--V---LC--I--N	L-P-L--MRKQ-RT-VL------
Domibacillus enclensis	WP_045851895	---F------FVI---M---	L-P-FS-M-QH-V--VV---F--
Domibacillus indicus	WP_046176026	---F------FVL---M--R	L-P-FE-M-RH-A--VV---F--
Domibacillus iocasae	WP_069938074	---F---M--FAI---M--R	L-P-FT-M-RN-I--VV---F--
Domibacillus robiginosus	WP_050181885	---FL-----FVV---M--T	L-Q-LS-M-QH-A--IV---F--
Domibacillus tundrae	WP_046179360	---F---V--FAI---M--R	L-P-FT-M-RN-I--VV---F--
Fictibacillus enclensis	WP_061967673	----L--V---LI--G---N	L-S-VQVM-ED-K--G---M---
Fictibacillus gelatini	WP_026676245	---FI--I---WL--RM--N	L-P-FL-MRKR-MP-I-------
Fictibacillus solisalsi	WP_090231973	----L--V---FI--G---N	L-A-LQVMRED-K--G---II--
Fictibacillus sp. FJAT-27399	WP_062236050	-------V---LI--ML--N	LGQ-MQTM-EQ-KS--L--L---
Fictibacillus sp. S7	WP_129477276	----L--V---FI--G---N	L-S-VQVM-ED-K--G---M---
Filibacter sp. TB-66	WP_124068980	-------V---V----Y--G	LGSVKR-MV---APFF---VTA-
Flavihumibacter solisilvae	WP_039140329	---F-W----FLV--LS-N	-S--W--I-RN-LQ-IV--T---
Jeotgalibacillus sp. S-D1	WP_133376672	---FL------LV--LA-NG	KQ-- K-LTKN-KQ-VL------
Kurthia gibsonii	WP_121176485	-----------LI---A-RG	LPKMTK-I-QNT----L------
Kurthia huakuii	WP_029497688	-----------FI---LQRH	LTPMTE-M-KA-----L------
Kurthia sibirica	WP_109304787	-----------LI---S-KG	LPYMVK-I-KNTS----------
Kurthia sp. 11kri321	WP_068456363	-----------LI---A-RG	LPKMTK-I-QNT----L------
Kurthia sp. Dielmo	WP_020188964	-----------FI---MQHN	VKPMTT-M-KA-IQ--L------
Kurthia zopfii	WP_109350180	-------I---LI---A-HG	LPSMTK-I--N-S---L------
Kyrpidia spormannii	WP_100668085	---F---V-L-M---WV-KG	I-P-FV-MRRH-WL--W------
Lysinibacillus acetophenoni	WP_097150378	------------VL-HF-KG	WKP-VR-M-TQ-SK--L------
Lysinibacillus boronitolerans	WP_036078053	-----------LI--FY-KG	-SQ--G-I--Q-I---L------
Lysinibacillus chungkukjangi	WP_107936941	-----------LV--LS-KG	WQP-TK-MR-Q-TK--L------
Lysinibacillus composti	WP_124766969	-----------LV--LY-RG	MNL-TK-M--Q-TK--L------
Lysinibacillus contaminans	WP_053585111	-----------LV--FY-KG	-TQ--G-M--Q-V---S------
Lysinibacillus endophyticus	WP_121213432	-----------FV--LS-KG	LKP--H-M-GQ-IK--L------
Lysinibacillus fusiformis	WP_004229766	-----------LI--FY-KG	-SQ--G-I--Q-I---L------
Lysinibacillus halotolerans	WP_122971795	-----------LI--FS-KG	WKP-TF-I-SQ-TK--L------
Lysinibacillus macroides	WP_053993304	-----------LV--FY-KG	-SQ--G-M--Q-I---L------
Lysinibacillus manganicus	WP_036184309	------------LL-HF-KG	WKP-AH-M-TQ-SK--L------
Lysinibacillus mangiferihumi	WP_107897389	-----------L---FY-KG	-SP-T--M-N--R---L------
Lysinibacillus massiliensis	WP_036178792	-----------FI--FY-KG	WKPM---M-RQ-VQ-FV------
Lysinibacillus parviboronicapi	WP_107924516	-------V---LV--FY-KG	-SQ-TT-M-TQ-I---L---I--
Lysinibacillus saudimassiliens	CEA04040	------------V---M-EG	L-P-RQ-MRQQ-R---GF--I--
Lysinibacillus sinduriensis	WP_036199630	---F-------LV--LS-KG	MKP-A--M-SQ-AR--L------
Lysinibacillus sp. B2A1	AVK82499	-----------LV--FY-KG	-PQ--G-M--Q-I---L------
Lysinibacillus sp. BF-4	WP_036144395	------------V---M-KG	L-P-RQ-MRQQ-RV--G---I--
Lysinibacillus sp. BK089	WP_132363694	-----------LV--FY-KG	-SK--G-M--Q-V---L---I--
Lysinibacillus sp. FJAT-14222	WP_053595810	-----------LV--FY-KG	-SQ--G-I--Q-V---L------
Lysinibacillus sp. FJAT-14745	WP_053485296	-----------L---FY-KG	-SQ--G-M--Q-I---L---I--
Lysinibacillus sp. Marseille-P	WP_106782420	-------V---LV--YF-KG	LKP--K-M-SQ-LK-T-------
Lysinibacillus sp. OL1	WP_131520681	-----------LI--FY-KG	-SQ--G-I--Q-I---L------
Lysinibacillus sp. PB300	WP_115674294	-----------LI--FY-KG	-SQ--G-I--Q-I---L------
Lysinibacillus sp. SYSU K30002	WP_126659183	-----------LI--FS-KG	WHL-TS-M-SQ-SK--L------
Lysinibacillus sp. YLB-03	WP_118876613	-----------LV------G	WKP--A-M-SQ-RQ--L------
Lysinibacillus sp. YR326	WP_134022687	-----------LT--FY-KG	-SQ--G-M--Q-VS--L---I--
Lysinibacillus sp. YS11	WP_103117363	-----------LI--FY-KG	-SQ--G-I--Q-I---L------
Lysinibacillus sp. ZYM-1	WP_054612790	-----------LI--FY-KG	-SQ----I--Q-T---L------
Lysinibacillus sphaericus	WP_010857757	-----------L---FY-KG	-SQ--G-M-SQ-I---L---I--
Lysinibacillus sphaericus C3-4	ACA38447	-----------LI--FY-KG	-SQ--S-I--Q-T---L------
Lysinibacillus tabacifolii	WP_108030683	----------L---FY-KG	-SP-T--M-N--R---L------
Lysinibacillus telephonicus	WP_126295340	-----------LV--LS-KG	WKP--S-M--Q-TK--L------
Lysinibacillus varians	WP_025218498	-----------L---FY-KG	-SP-T--M-N--R---L------
Lysinibacillus xylanilyticus	PJ041370	-----------LT--FY-KG	-SQ--G-M--Q-VS--L---I--
Lysinibacillus xyleni	WP_097073553	-----------LG--FS-KG	LKP-TH-M-GQ-VK--L------
Marinococcus halophilus	WP_079475263	---------M--V---AQNR	L-PVFHHLRSR-AE--L--T---

Other Bacteria (0/>300)

Marinococcus halotolerans	WP_022793255	---------M-----GAQKR	L-PVFHHLL-R-SE--L--T---
Marinococcus luteus	WP_091613924	---------M-----GAQKR	L-PVFHHLRTR-SE--L--T---
Mycobacteroides abscessus subs	SLL34586	-------V-L-ML--IC-N	IT-VWQ-L--N-VS--L--T---
Paenibacillus	WP_076354040	-------V-L-LLL-GA--R	L-P-FR-MRLR-SS--L------
Paenibacillus beijingensis	WP_045669519	----L--V-L-LI--IAKNN	LKP-LY-MRKR--T--L------
Paenibacillus borealis	WP_042210835	------T----LV---G--R	L-P-LAAMRER-----L-GT---
Paenibacillus ihuae	WP_054939017	----L-T----V----G--R	LKP-LSAM-ER-A---L-GT---
Paenibacillus mucilaginosus	WP_014371188	-------V-L-LLL-GRHHL	KP-WA-IRRA-----L--T---
Paenibacillus sp. A3	WP_054972989	----L--V-L-LGV-LA-KS	L-P-FA-M-KQ--T--L-GT---
Paenibacillus stellifer	WP_038694098	---I-T----L-L--G--R	L-P-LR-MADR-L---L-GT---
Paenibacillus tianmuensis	WP_090675988	----L----L-LGV-LA-N	L-P-LT-MRKQ--T--L-GT---
Paenibacillus typhae	WP_090719723	----L-T----V----G--R	LKP-LAAM-ER-----L-GT---
Paenibacillus tyrfis	WP_036686541	----L--V-L-LG--LA-N	L-P-LT-MRKQ--T--L-GT---
Paenisporosarcina antarctica	WP_134210807	-------V---LV--IL-SG	LKP-LV-M-SR-VS-FS------
Paenisporosarcina quisquiliaru	SEN79908	----------FF--YY-KG	LSTTFK-I-SA-A---L------
Paenisporosarcina sp. HGH0030	WP_016428660	----I--I---LS--LM-N	LLA-IK-MQKQ--K-IL------
Paenisporosarcina sp. OV554	WP_108584963	-------V---ML--VL--G	MAT-LA-M-SR-LP-FG----A-
Paenisporosarcina sp. TG-14	WP_017381786	-------V---L---LF--G	LKP-FV-M-NQ-IP-FS----A-
Paenisporosarcina sp. TG20	WP_019413424	-------V---L---LF--G	MKP-IT-M-TH-VP--G----A-
Planococcus citreus	WP_121301203	----L--I---LL--ML--N	LLP-LH-M-KHKVK--L---I--
Planococcus faecalis	WP_078080251	-------I---LL--VL-N	LKP-LQ-MNKNKR--TL------
Planococcus halotolerans	WP_112223480	-------V-------LM-N	L-PVFS-MRKQ--K--L------
Planococcus kocurii	WP_058385833	-------I---LL--VL-N	LKP-LQ-MNKNKR--TL------
Planococcus maritimus	WP_068461501	----L--I---LL--I---N	LYP-FK-M-KHKIK-IV------
Planococcus plakortidis	WP_068868791	----L--I---LL--VL--N	LLP-LH-M-KHKVK--L---I--
Planococcus rifietoensis	WP_058381167	----L--I---LL--I---N	L-P-LQ-MRNRKVK--L------
Planococcus salinus	WP_123166500	----I--V---VI--LA-KN	MGP-LK-M-KN--T--L------
Planococcus sp. CAU13	WP_033542144	-------V---LC--LM-N	L-PVL--M-KQ--K--L------
Planococcus sp. PAMC 21323	WP_038703414	----L--I---LL--IS-N	LKP-LQ-MNKSKK----------
Planococcus sp. Y42	WP_077588360	----I--V---VL---L-N	LKP-LR-MRKQ--Q-ML-G----
Planomicrobium glaciei	KOF08955	----L--V---LV--LA-RN	LKP-IA-M-NN-V---L---I--
Planomicrobium okeanokoites	WP_084242826	-------V---V---LM-N	L-PVFS-M-KQ--K--L------
Planomicrobium soli	WP_106534368	A---I--V---LV--MV-N	LKP-LE-M-RA-T---L---I--
Planomicrobium sp. MB-3u-38	WP_101803089	-------V---V---LM-N	L-PVFS-M-KQ--K--L------
Planomicrobium sp. Y74	WP_121634326	T------V-------LM-N	L-PVFI-MRKQ--K--L------
Pseudomonas sp. FW305-BF6	PNB76841	-------V---F---FL-NS	KD-IEHM-KHL-S-IL--SI--
Psychrobacillus psychrodurans	SFM76056	----------FF--YY-KG	LSTTFK-I-SA-A---L------
Psychrobacillus psychrotoleran	WP_093536572	----------FI--YY-KG	LSPTYK-I-SA-A--FL------
Psychrobacillus sp. OK028	WP_093063004	----------FI--YY-KG	LSATFK-I-SA-A---L------
Quasibacillus thermotolerans	WP_039238520	----L--I-L-VLL--G-KG	LGQMLG-M-KN-A---V------
Rubeoparvulum massiliense	WP_048601972	---FI-------L--MW---	LGL-IQ-M-RR-WE-ML--T---
Rummeliibacillus pycnus	WP_102693241	----L------LI--SS-KG	LTAVVQ-I-TQ-KD--L------
Rummeliibacillus stabekisii	WP_066791467	-----------LI--SA-KG	MP--TK-M----LP--C------
Sinobaca qinghaiensis	WP_120192394	---FL--I---LL--LL-RG	VKQVFI-IA---LP-I----T--
Sporomusa sphaeroides	WP_075757868	A---L-TV---LLL-LS-NN	LQA-IA-MR-R-D---L--T---
Sporosarcina globispora	WP_053433452	-------I-L-F---VF--N	L-P-FT-M-KA--Q-ML------
Sporosarcina koreensis	WP_040287686	-------V---A----M--G	-TPVQR-MRRH-WQFF---T-A-
Sporosarcina newyorkensis	WP_009766529	A------V---FV---M--G	LSGMKR-MT-A-RPFF-----A-
Streptococcus pneumoniae	CJF73263	-------V-L-LVL-YFW-G	IKPVVA-I--Q-QK--L--T---
Streptomyces albulus	WP_078488725	----L--TVP-L-L--L--G	LPAVFRSLR-R--H--L--TI--
Streptomyces tsukubensis	OON82306	----L---APMVVL--V--G	LGGTLRSLR-R--T--L--T---
Tetzosporium hominis	WP_094944507	-------A---LV--WM--G	ISS-VGDM-IS-RP---------
Thermoflavimicrobium dichotomi	WP_093228046	----I--V---LF--MA-N	L-P-LQMMREQ--T-FL------
Thermoflavimicrobium sp. FBKL4	WP_113658143	-------I---LV--LY-N	LST-LH-MRKQ-I---L--TI--
Tumebacillus flagellatus	WP_038090263	----L--A-MM--L--V-SE	LKPVWRALR-Q--V--G------
Ureibacillus thermophilus	QBK26159	-F-----------SY-TG	-EN-HR-MRENTSK--L------
Ureibacillus thermosphaericus	WP_016837652	-F-----------SY-TG	-EN-RR-MRENTSK--L------
Vibrio vulnificus	WP_133350631	----I--V---LC--LS-N	L-P-LRVMRE---P--L------
Viridibacillus arenosi	WP_038180417	-------I---LV--MY-KG	VQPMTK-M-KR-KV--L------
Viridibacillus arvi	WP_053418046	-------I---LV--MY-KG	IQSMTK-M-KQ-K---L------
Viridibacillus sp. OK051	WP_100794998	-----------LI--MY-KG	MQA-TQ---KQ-----L------

Supplemental Figure 61

A partial sequence alignment of the Multidrug resistance efflux transporter family protein containing a two amino acid insertion (boxed) that is exclusively shared by all members belonging to the Solibacillus clade and absent in all other bacteria. The peripherally branching *Lysinibacillus odysseyi* does not share this CSI.

Group	Species	Accession	32		66
Solibacillus clade (6/7)	Bacillus cecembensis	WP_057986646	EREAATQEFIQYLEQLN		TATGSHAFYAIIGQKADF
	Solibacillus isronensis	WP_008404556	------K---AF--D--		KD---N---S--------
	Solibacillus silvestris	WP_014824754	------K---AF--D--		KD---N---S--------
	Solibacillus kalamii	WP_079524598	------K---AF--D--		KD---N---S--------
	Solibacillus sp. R5-41	WP_099422355	------K---E---H--		-----N------------
	Lysinibacillus sp. 2017	WP_108711481	------K-------D--		KD---N---S--------
	Lysinibacillus odysseyi	WP_036153140	--Q--VE-------K--	EADT	AK--A------V------
Other Bacteria (0/>300)	Bacillus ndiopicus	WP_042470721	--T--VE--VA---K--	EADV	AK--A------V------
	Lysinibacillus meyeri	WP_107838217	--T--VE--VA-V-K--	EADV	AK--A------V------
	Anaerobacillus alkalidiazotrop	WP_071388410	-Q-QI-S--VSI-KKWE	NAEK	RKA----L-S-V------
	Anaerobacillus alkalilacustris	WP_071310474	-Q-QI-N--VSI-KKWE	DAEK	GKA----L-S-V------
	Anaerobacillus isosaccharinicu	WP_071319077	--QSI-N-LLGL--KWE	TTEA	RKA----L-S-V-----L
	Anaerobacillus macyae	WP_048311311	---Q--N--LTF--KWE	VTAG	HKQ----L-S-V------
	Anoxybacillus tepidamans	WP_027408073	--Q--IH--LSLV-KWQ	ETEN	RKE----I-TVV------
	Bacillus acidicola	WP_066267469	---S-IY--Q-F--K--	KTQE	NKK---SL-T-V------
	Bacillus acidiproducens	WP_018661286	---S-IA--EEF-AK-D	GIQN	NHE-AY-L-T-V------
	Bacillus aciditolerans	WP_121448345	--Q--IH--LGLVDKW-	KTQA	ENQ----L-T-V------
	Bacillus alkalitelluris	WP_088077123	---QIMG--LNL--NW-	ETQT	KFE----L-S-V------
	Bacillus aquimaris	WP_032086981	--AE-IA-YRRF-DK--	TTQA	AKE----L-T-V------
	Bacillus aryabhattai	WP_045293877	D-Q--IH--LGLI-KWK	GTEA	EQQ----L-T-V------
	Bacillus asahii	WP_119116248	--QL-IS--QGL-DKW-	TTQS	NNE----L-S-V------
	Bacillus badius	WP_041095483	--QT-IY--Q-F-NK--	TVHE	AKE------S-V------
	Bacillus bataviensis	WP_007087476	--QT-IH--LSLV-KW-	QTGE	QKK----L-T-V------
	Bacillus bogoriensis	WP_026672750	--QQIVN--L-TI-KWS	TAET	NGE----I-S-V------
	Bacillus camelliae	WP_101354439	--QT-IY--Q-FINK--	QTQN	EKQ----L-T-V------
	Bacillus campisalis	WP_046523813	--Q--IH--LGL--KW-	ATQN	EDK----L-TVV------
	Bacillus canaveralius	WP_101578114	--Q--IH-YLGLV-KWS	AAQE	RKE----L-T-V------
	Bacillus cavernae	WP_126863571	--QS-IS--NGL--KW-	KTQD	KKE----L-S-V------
	Bacillus cellulosilyticus	WP_013490687	---TIMA--FSL--KW-	NTQS	KFE---GL-T-V------
	Bacillus cihuensis	WP_028393392	--KE-IS--NRLVDKW-	TTQS	NNE----L-T-V------
	Bacillus circulans	WP_047940794	--QE-IT--LGL--KW-	TVQE	NNS----L-T-V------
	Bacillus coagulans	WP_013860784	--D--VK--R-F-Q--D	AIQS	KHE-AY-L-T-T------
	Bacillus coahuilensis	WP_010175666	--A--I--YH-F-NK--	QTQE	EEK----L-T-V------
	Bacillus cohnii	WP_066421946	--Q--IN--LGLV-KW-	TVEK	AKN----L-S-V------
	Bacillus cucumis	WP_101648359	-----IH--LSLV-KW-	ATQA	RKE----L-T-V------
	Bacillus cytotoxicus	WP_012096352	---Q-IV--LNII-KW-	KVAA	EKQ----M-T-V------
	Bacillus dakarensis	WP_077215114	--QT-IA--LSLV-KW-	KTQE	EKK---TL-T-V------
	Bacillus deserti	WP_101641254	--QE-VN--LGLV-KW-	KTQE	NKE----I-T-V------
	Bacillus dielmoensis	WP_042463702	--Q--IH--LGLV-KW-	STQE	SKE----L-T-V------
	Bacillus drentensis	WP_066248786	--Q--IH--LSLV-KW-	ATQE	RKE----L-T-V------
	Bacillus enclensis	WP_058299477	--A--IA-YRRF-DK--	TTQA	AKE----L-T-V------
	Bacillus fastidiosus	WP_066230799	--Q--ID--LGL--KWG	AVEN	KGE----L-S-V------
	Bacillus firmus	WP_061793815	--Q--IH--MALVDKW-	TTQS	EKN----L-T-V------
	Bacillus flexus	WP_119543832	---T-IH--LALT-KWT	NTEN	A-E----L-S-V------
	Bacillus foraminis	WP_121610505	--Q--IH--LGL--KW-	SAQN	EDK----L-T-V------
	Bacillus fordii	WP_018708377	--Q--IH--K---AK-D	ETQD	RKE----L-T-V------
	Bacillus fortis	WP_120071639	--Q--IH--K---AK-D	ETQD	RKE----L-TVV------
	Bacillus freudenreichii	WP_126430803	-QQ--IH--K---AK-D	DTQE	RKE----L-TVV------
	Bacillus fumarioli	WP_066367512	--QT-IH--LSLV-KW-	TTQK	QNE----L-T-V------
	Bacillus ginsengihumi	WP_025727600	---K-IF--Q-F-DR--	EIQE	SGN-AN-L-T-V------
	Bacillus gottheilii	WP_066442245	--QE-IH--LSLV-KW-	KTQA	EKK----L-TVV------
	Bacillus halmapalus	WP_078378565	--QT-IN--LGLV-KW-	NTQA	EKN----L-T-V------
	Bacillus horikoshii	WP_064099617	--QT-IN--LGLV-KW-	TVQL	EKN----L-S-V------
	Bacillus horneckiae	WP_066398391	--Q---H--LSL-DKW-	TTQS	EKK----L-T-V------
	Bacillus humi	WP_057998142	--Q--I---LGLV-KW-	NTQT	ENQ----L-T-V------
	Bacillus jeotgali	WP_079506206	--QEII---LGLV-KW-	KTQA	EKQ----L-T-V------
	Bacillus kochii	WP_095371534	-----IH--LSLVDKW-	TTQA	AEQ----L-T-V------
	Bacillus koreensis	WP_053403768	--Q--IH--LGLV-KW-	KTQT	DGQ----I-S-T------
	Bacillus korlensis	WP_066056949	D-QT-IH--LGLV-KW-	TTQD	KKE----L-T-V------
	Bacillus kwashiorkori	WP_062351409	--D--VE--LAL-NKWA	EVDE	NKQ------S-V-----L
	Bacillus lehensis	WP_038484186	--DGMIE--TAL-N-W-	DTET	NGK----L-S-V-----L
	Bacillus lentus	WP_066143857	--Q--IH--S----K--	DVQD	KKE----L-S-V------
	Bacillus litoralis	WP_066338139	--Q--IH--LGL--KW-	VAED	AEQ---TL-S-V------
	Bacillus loiseleuriae	WP_049683359	--QE-IS--NRLVDKW-	TTQS	NNE----L-TVV------
	Bacillus marinisedimentorum	WP_066176011	---T-IH--QGLI-KW-	KTEE	QKE----L-T-V------
	Bacillus marisflavi	WP_121617757	D----IS--HRF-HK--	KTHE	AKE----L-T-V------
	Bacillus massiliglaciei	WP_110928280	---I-IS--QGL-DKW-	TVQN	NNE----L-S-V------
	Bacillus massilioanorexius	WP_019243271	--Q--IS--LSLV-KW-	KTES	EKK----L-T-V------
	Bacillus massiliogabonensis	WP_102273569	--Q--IH--LSL-DKW-	TTQS	EKK----L-TVV------
	Bacillus massiliogorillae	WP_042351126	--Q--IS--LGLV-KW-	KTEA	EKA----L-S-V------
	Bacillus massilionigeriensis	WP_075983238	--Q--IN--LGLI-KW-	KTQV	EKQ----L-T-V------
	Bacillus massiliosenegalensis	WP_019154032	--Q--IH--LGLV-KW-	STQL	DKK----L-T-V------
	Bacillus megaterium	RBN36156	--Q--IH--LGLI-KWK	GTEA	EKQ----L-T-V------
	Bacillus mesonae	WP_066389619	--QT-IH--LSLV-KW-	KTQE	RKE----L-T-V------

Other Bacteria (0/>300)

Bacillus methanolicus	WP_003346907	--Q--IH--LGL--KW-	ATQN	RKE---VL-T-V------
Bacillus muralis	WP_064467068	--QL-IS--QGL--KW-	MTQS	DNE----L-S-V------
Bacillus nealsonii	WP_016202535	--QE-IN--LGL--KW-	VVQE	NNS----L-T-V------
Bacillus niacini	WP_045515996	--QS-IH--LSLV-KW-	HTQE	RNE----L-T-V------
Bacillus niameyensis	WP_062105445	--QN-ID--K---NR--	DSQE	QKE----L-T-V-H----
Bacillus notoginsengisoli	WP_118921008	--Q--ID--KGLV-KW-	GTME	NKE----L-TVV------
Bacillus terrae	WP_120119151	--Q--IH--K---AK-D	EAQD	RKD----L-T-V------
Bacillus testis	WP_050613383	--QT-IA--LALV-KWK	KTEN	EKA----L-T-V------
Bacillus timonensis	WP_010677259	--Q--IH--LGLV-KW-	KTQT	EQQ----L-T-V------
Bacillus tuaregi	WP_071393234	--D--I---QGLVDKW-	RTEK	EEQ---VI-S-V------
Bacillus vireti	WP_024027442	--Q--IH--LSLV-KW-	TTQE	RKE----L-T-V------
Bacillus weihaiensis	WP_072578107	--Q--IH--QGL--KW-	GVQE	AEK----L-S-V------
Bacillus zeae	WP_119112029	--Q--IH--RAL-DKW-	TTQA	GKE----L-T-V------
Bhargavaea beijingensis	WP_092097417	K-K---D---AF-DK-Q	KADD	EK-------TVV------
Bhargavaea cecembensis	WP_008296594	--K---D---A--DK-Q	KADD	EK-------TVV------
Bhargavaea ginsengi	WP_092054635	--K---D---TF-DK-Q	TADN	EK-------T-V------
Caenibacillus caldisaponilytic	WP_077614155	--QK-LD--GRL-DKWD	ETMK	AKA--Y---S-V------
Caryophanon latum	WP_066466121	--QE-IE--QA--AN--	EADV	AK--A----T-V------
Chryseomicrobium excrementi	WP_100353353	--Q---A--LVF-DEMQ	QVDE	SKE-A----TVV------
Dietzia cinnamea	WP_063973145	--QEII---LGLV-KW-	RTQA	EKQ----L-T-V------
Domibacillus aminovorans	WP_063966614	---D-IS--R-F-TK-D	ATQE	SKA---S--SVV------
Domibacillus antri	WP_075397025	---G-IS--RKF--K-D	AVQD	VRA---S--S-V------
Domibacillus enclensis	WP_045852525	---S-IA--RKF-FK-E	ATQE	IKA---S--SV-------
Domibacillus epiphyticus	WP_076766146	---G-IS--RKF--K-D	SAHE	QK----S--SVV------
Domibacillus indicus	WP_046174624	---S-IA--RKF-YK-E	AVQE	VKA---S--SV-------
Domibacillus iocasae	WP_069938480	---N-LS--RKF-YK-E	ATQE	LKE---S--SVV------
Domibacillus mangrovi	WP_073710696	---N-IS--R-F-TK-D	ATQE	SK----S--S-V------
Domibacillus robiginosus	WP_050180424	---G-LA--RKF-YK-E	ETQE	LKS---S--S--------
Domibacillus tundrae	WP_046179079	---S-IS--RKF-YK-E	ATQE	SKE---S--SVV------
Edaphobacillus lindanitolerans	WP_076756830	--K---N--MAF-DK-Q	KADD	EK-------T-V------
Falsibacillus pallidus	WP_114745852	--Q--IV--HR--DK--	DTQA	NQN----L-T-V------
Falsibacillus sp. GY 10110	WP_121681906	--Q--IV--HR--DK--	EVQD	NEK----L-S-V------
Fictibacillus aquaticus	WP_094252714	Q--L-IR--MTF--KWE	NTEA	QNA----L-S-V------
Fictibacillus arsenicus	WP_066294075	Q--Y-IN--LTF--KWE	NVEA	NK-----L-S-M------
Fictibacillus enclensis	WP_061973364	-----IK--TAF--KWE	GAEA	RKE----L-S-V------
Fictibacillus gelatini	WP_026678814	--DY--K--LTF--KWE	NVEA	KKE----L-T-V------
Fictibacillus macauensis	WP_007203922	---H-IH--LGF--KSE	GVQA	RKE----L-S-V------
Geobacillus thermoleovorans B2	GAJ57115	-----IS--LALVD-WE	TTES	EKQ----V-T-V------
Geobacillus thermoleovorans CC	AEV21138	-----IS--LALVD-WE	TTES	EKQ----V-T-V------
Geobacillus vulcani	WP_031406569	-----IS--LALVD-WE	TTES	EKQ----V-T-V------
Jeotgalibacillus malaysiensis	WP_039811744	--T--IN--Q-F-KK--	DVQD	QDQ---S--S-V------
Jeotgalibacillus proteolyticus	WP_104058690	D-SL-IT--Q-F-KK-H	IVQD	EEQ---S--S-V------
Jeotgalibacillus sp. S-D1	WP_133376923	--TQ-IA--QHF-QK-D	VVQN	EEQ---S--S-V------
Kurthia huakuii	WP_029497773	--KE--D---AF-AE--	KADE	AK-------S-V------
Kurthia massiliensis	WP_010287148	--KQ--D---AF-AE--	KADE	AK-------S-T------
Kurthia senegalensis	WP_010302604	--Q---D---AF-AE--	KADE	EK-------S-V------
Kurthia sibirica	WP_109307310	--TE-VE--L----R--	EADL	AK-----I-S-V------
Kurthia zopfii	WP_109350814	--TE-VE-L-----K--	EADV	AK-------S-V------
Lysinibacillus acetophenoni	WP_097147586	--TK-VE--V----K--	EAHV	AK-------S-L------
Lysinibacillus boronitolerans	WP_016993375	--Q--VE---T---K--	EADV	AK--A------V------
Lysinibacillus chungkukjangi	WP_107936790	--TN-VD--V----KI-	ESHV	AK-------S--------
Lysinibacillus composti	WP_124763476	--QQ--E--LK---N--	EAHV	AK-------S--------
Lysinibacillus contaminans	WP_053585217	--Q--VE---T--DK--	EADV	AK--A------V------
Lysinibacillus endophyticus	WP_121215487	--SK-ID--V----K--	ETHV	AK-------S--------
Lysinibacillus fusiformis	WP_025115434	--Q--VE--LA---K--	EADI	AK---------V------
Lysinibacillus halotolerans	WP_122972428	--TK-VD--V----K--	EAHV	AK-------S--------
Lysinibacillus jejuensis	WP_108306083	--K---D---AF--K--	QADE	AK-------S-V------
Lysinibacillus macroides	WP_053993171	--Q--VE---A---K--	EADV	AK--A------V------
Lysinibacillus manganicus	WP_036182389	--TK--E--L----KI-	EAHV	AK-------S-L------
Lysinibacillus mangiferihumi	WP_107896666	--Q--VE---A---K--	EADV	AK--A------V------
Lysinibacillus massiliensis	WP_036171134	--TK-VD--V----K--	EAHV	AN-------S-L------
Lysinibacillus parviboronicapi	WP_107923707	--Q--VE---A--DK--	EADV	AK--A------V------
Lysinibacillus saudimassiliens	CEA01630	--K---D---AF--K--	QADE	AK-------S-V------
Lysinibacillus sinduriensis	WP_036200051	--TN-VN--V----K--	EAHV	AK-------S--------
Lysinibacillus sp. B2A1	AVK82628	--Q--VE--VT---K--	EADI	AK--A------V------
Lysinibacillus sp. BF-4	WP_036145426	--K---D---AF--K--	QADE	AK-------S-V------
Lysinibacillus sp. BK089	WP_132360254	--Q--VE---A---K--	EADI	AK--A------V------
Lysinibacillus sp. YR326	WP_134020387	--Q--VE---A---K--	EADV	AK--A------V------
Lysinibacillus sp. YS11	WP_103117408	--Q--VE---A---K--	AADV	AK--A------V------
Lysinibacillus sp. ZYM-1	WP_054612801	--Q--VE---A---K--	EADV	AK--A------V------
Lysinibacillus sphaericus	WP_010857910	--Q--VE---A--DK--	DADV	AK--A------V------
Lysinibacillus telephonicus	WP_126293233	--TK-VD--V----K--	EAHV	AK-------S--------
Lysinibacillus varians	WP_025218559	--Q--VE---A---K--	EADV	AK--A------V------
Lysinibacillus xylanilyticus	WP_049667959	--Q--VE---A---K--	EADI	AK--A------V------

Other Bacteria (0/>300)

Lysinibacillus xyleni	WP_097071630	--SK-ID--V----K--	ETHV	AK-------S--------
Massilibacterium senegalense	WP_062200235	--QQ-IH--L-L--KF-	QTEE	KKE------S-V------
Mycobacteroides abscessus subs	SHP68314	D-QT-IH--LGLV-KW-	ATQD	KKE----L-T-V------
Paenibacillus sp. N2SHLJ1	WP_131017087	--N--QD-LLSL-H-YK	QAEE	NK---T---S-V------
Parageobacillus caldoxylosilyt	WP_017436929	-----IH--LSLV-KWQ	ETED	KKE----I-T-V-----L
Parageobacillus genomosp. 1	WP_043906308	-----IR--LSLV-KWQ	ETED	KKE----I-T-V-----L
Parageobacillus thermantarctic	WP_090948129	-----IR--LSLI-KWQ	ETED	KQE----V-T-V-----L
Parageobacillus thermoglucosid	WP_064552837	-----IR--LSLI-KWQ	ETED	KQE----I-T-V-----L
Planococcus antarcticus	WP_006829291	--QF-ID--QAFMDKI-	QADE	NK--A--L-S-V-----L
Planococcus citreus	WP_121298416	--QF-ID--NTFM-KV-	QADE	NK--A--L-S-------L
Planococcus donghaensis	WP_008430450	--QF-ID--QAFMDKI-	QADE	NK--A--L-S-V-----L
Planococcus halocryophilus	WP_008496847	--QF-ID--QAFMDKI-	QADE	NK--A--L-S-V-----L
Planococcus halotolerans	WP_112221257	--QF-ID-YQAFMDKV-	QADE	NK--A--L-S-------L
Planococcus maitriensis	WP_112230393	--QF-ID--NGFM-KV-	QADE	NK--A--L-S-------L
Planococcus maritimus	WP_068464297	--QF-ID--NNFM-KV-	QADD	NK--A--L-S-------L
Planococcus massiliensis	WP_052650637	--QF-ID--QAFMDKI-	QADE	NK--A--L-S-V-----L
Planococcus plakortidis	WP_068870134	--QF-ID--NTFM-KV-	QADE	NK--A--L-S-------L
Planococcus rifietoensis	WP_058382911	--QF-ID--NMFM-KV-	QADE	NK--A--L-S-------L
Planococcus salinarum	OHX57192	--QF-IN-YQAFMDKV-	IADE	NK--A--L-S-V-----L
Planococcus sp. PAMC 21323	WP_038704765	--QF-ID--QAFMDKI-	QADE	NK--A--L-S-V-----L
Planococcus sp. Y42	WP_077590220	--R--VE--TAF-NKI-	KTDE	AK--A--L-T-V------
Planococcus versutus	WP_049692970	--QF-ID--QAFMNKI-	QADE	NK--A--L-S-V-----L
Planomicrobium flavidum	WP_088007658	--R--ID--QAF-NKIG	KADE	EK--A--L-T-V------
Planomicrobium glaciei	WP_036802934	--QF-ID--QAFMDRV-	QADE	NK--A--L-S-V-----L
Planomicrobium okeanokoites	WP_084244160	--QF-ID-YQAFMDKV-	QADE	NK--A--L-S-V-----L
Planomicrobium soli	WP_106532801	--QF-ID-YQAFI-KV-	QADE	NK--A--L-S-V-----L
Planomicrobium sp. MB-3u-38	WP_101801394	--QF-ID-YQAFMDKV-	QADD	NK--A--L-S-V-----L
Planomicrobium sp. Y74	WP_121635625	--QF-ID-YQAFMDKV-	QADE	NK--A--L-S-------L
Pontibacillus chungwhensis	WP_036780113	---S-IH--H-L--KW-	QTEQ	NKE----L-TVV------
Pontibacillus litoralis	WP_036834476	-----IH--H-L--KW-	KTQD	EKQ----I-TVV------
Pontibacillus marinus	WP_027447924	---S-IY--N-L--KW-	QTEK	NKE----L-T-V-----L
Pontibacillus yanchengensis	WP_036815779	---T-IH--N-L-DKWK	DTED	KKE---SL-TVV------
Pueribacillus theae	WP_116555856	---K-IS--LGF--KWD	VVKH	NRE-DY-L-S-V------
Quasibacillus thermotolerans	WP_039231135	--QT-IY--Q-F-NN--	DVHE	AKE------SVV------
Rhodococcus qingshengii	WP_133369789	--QS-IH--LSLV-KW-	HTQE	RNE----L-T-V------
Rummeliibacillus pycnus	WP_102693147	--K--VD---N---KI-	EADI	AK-------S-L------
Rummeliibacillus stabekisii	WP_066791659	--K--VD------DK--	EADE	NK-------S-V------
Salipaludibacillus neizhouensi	WP_110938285	K--SIMS--MKL--KWD	KTQA	KFE---GL-S-L------
Salipaludibacillus sp. KQ-12	WP_110611813	----VIA--LKL-AKWD	VTQS	KFE---GL-S-L------
Scopulibacillus darangshiensis	WP_132747893	---S-I---NSF-DKWD	IMQQ	NKE--F---S-V------
Sediminibacillus albus	WP_093213814	--Q--IF--Q-L--KWE	QTEK	DKK----L-TVV------
Sediminibacillus halophilus	WP_074599838	--Q--IH--Q-L--KWE	QVEQ	NRN----L-T-V------
Sediminibacillus massiliensis	WP_077623501	--Q--IH--Q-M-GKWE	QVEK	DRN------SVL-H----
Sinobaca qinghaiensis	WP_120194538	--QSIVK---HM--DWQ	QVED	NGG----V-T-V-----L
Sporolactobacillus pectinivora	WP_100488036	--QT-M---KEFVDH-S	QNEE	NKE--Y---S--------
Sporolactobacillus sp. THM19-2	WP_129928460	-QQD-V---NH--S-WD	ENEQ	DHK--Y---S-V------
Sporolactobacillus sp. THM7-4	WP_130030852	-QL--I---NEF-NK-E	QNQL	NKE--Y---S--------
Sporosarcina globispora	WP_053433421	--Q--IH--MALVDKW-	TTQS	EKN----L-T-V------
Sporosarcina koreensis	WP_040287595	--QH-VD--VAF-DK--	KADE	NK--A----T-V------
Sporosarcina newyorkensis	WP_009766265	--Q--VN--LA---R-Q	QADE	AK-------TVV------
Sporosarcina pasteurii	WP_115359888	--QTIIE--H---DK-Q	HAHD	ENN------TVV------
Sporosarcina psychrophila	WP_067213253	--Q--VE--LV---K-Q	KTDD	DK--A----TV-------
Sporosarcina ureae	WP_029053503	--Q--VN--LTF--RMQ	EADD	AK-------TVV------
Staphylococcus lentus	WP_016999454	D-NTIVE--KKF--G-E	TVHN	NKE------N-T-----L
Staphylococcus stepanovicii	WP_095089690	D-NTMV-D-KRF--G-E	TVHQ	NKE------N-T-----L
Tetzosporium hominis	WP_094943560	--Q---E--LAF-DEMQ	QVDD	AKE-A----TVV------
Thalassobacillus cyri	WP_093044892	---S-IH--H-LINKWE	KTES	NK-----I-S-V------
Thalassobacillus devorans	WP_028784160	---S-IH--H-LINKWE	KTES	NK-----I-S-V------
Thalassobacillus sp. TM-1	WP_062442569	---S-IH--N-LISKWE	KTES	NKA----L-S-V------
Tuberibacillus sp. Marseille-P	WP_085522572	--T--L---Y-F-DAWD	VVQQ	NKN--YS--S--------
Ureibacillus thermophilus	QBK25202	--Q--LD--YE--DK-E	EVHQ	AK-------ST-------
Ureibacillus thermosphaericus	WP_016837297	--Q--IE--N---DK--	QVHE	AK-------ST-------
Virgibacillus senegalensis	WP_053218048	--Q--ID--Q-L--KWE	QVEQ	DRN----L-T-V------
Viridibacillus arvi	WP_053417937	--Q--VE---K---KI-	EADI	AK-------S-V------
Viridibacillus sp. OK051	WP_100794887	--QETID---K---KI-	EADI	AK-------S-V------

Supplemental Figure 62

A partial sequence alignment of the heme-dependent peroxidase protein containing a four amino acid deletion (boxed) that is exclusively shared by all members belonging to the Solibacillus clade and absent in all other bacteria. The peripherally branching *Lysinibacillus odysseyi* does not share this CSI.

Group	Organism	Accession	220		265
**Solibacillus clade (6/7)**	Solibacillus sp. R5-41	WP_099424991	VEQGEVLQVFQNPQAPITKNFMSQISG	E	TQEMQTSLEQILANYPSG
	Solibacillus silvestris	WP_008408682	----D------H---------V--A--	-	---T-A----------E-
	Solibacillus kalamii	WP_087618372	----D------H---------V--A--	-	---T-A----------E-
	Solibacillus isronensis	WP_079524197	----D------H---------V--A--	-	---T-A-----F----G-
	Bacillus cecembensis	WP_057983637	---------------------A----	-	---I-A------------
	Lysinibacillus sp. 2017	WP_108713673	----D------Q---------V--A--	A	---T-A--Q--I------
	Lysinibacillus odysseyi	WP_036156978	--T-K--D----------R-V--V--		-N-TKE-I----E-----
**Other Bacteria (0/>300)**	Bacillus ndiopicus	WP_042476191	----Q--N-------T-----V--V-D		SS-TKE-I---I----T-
	Lysinibacillus fluoroglycofeni	WP_107943150	----K--E---S---T-----V--V-D		SS-TKE-I---I----T-
	Lysinibacillus meyeri	WP_107839158	----K--E-------T-----V--V-D		SS-TRE-I---I-S--T-
	Aeribacillus pallidus	WP_063386934	--H-D--T--R--K-N---R-VK-VTE		PD-TMETIDYL-EK-KH-
	Anoxybacillus amylolyticus	WP_066322847	--M-------RK-EQ----R-VK-VTE		PE-TKEAMV-L-DQ--N-
	Anoxybacillus ayderensis	WP_085787833	--M----H--RK-EQ----R-VQ-LTE		PE-TKEAMLHL-ER--N-
	Anoxybacillus flavithermus	AST06248	--M----H--RK-EQ----R-VQ-VTE		PE-TKEAMLHL-ER--N-
	Anoxybacillus geothermalis	WP_044742706	-------H--R---Q----R-VK-LVE		PE-TEEAISHLFGQ----
	Anoxybacillus gonensis	WP_009362266	--M----H--RK-EQ----R-VQ-VTE		PE-TKEAMLHL-ER--N-
	Anoxybacillus kamchatkensis	WP_019417426	--M----H--RK-EQ----R-VQ-VTE		PE-TKEAMLHL-ER--N-
	Anoxybacillus pushchinoensis	WP_091700990	--M----H--RK-EQ----R-VQ-VTE		PE-TKEAMLHL-ER--N-
	Anoxybacillus sp. 103	WP_077429560	--M----H--RK-EQ----R-VQ-VTE		PE-TKEAMLHL-ER--N-
	Anoxybacillus sp. P3H1B	WP_066148811	--E-K--D------QM---R-VQ--TE		PE-TKGTIAHLFNQ--N-
	Anoxybacillus sp. UARK-01	WP_080862752	--E-K--D------QM---R-VQ--TE		PE-TKGTIAHLFNQ--N-
	Anoxybacillus suryakundensis	WP_055441467	--M----H--RK-EQ----R-VQ-VTE		PE-TKEAMLHL-ER--N-
	Anoxybacillus tepidamans	WP_027410053	--E----H---R--QM---R-VQ--TE		PE-TKETIAHL-DQ--T-
	Anoxybacillus thermarum	WP_043964959	--M----H--RK-EQ----R-VQ-VTE		PE-TKEAMLHL-ER--N-
	Anoxybacillus vitaminiphilus	WP_111645610	--E----R--RK-EQ----R-VQ-VTE		PE-TKETISHL-EK--Q-
	Bacillus abyssalis	WP_078414213	-------E--R--TQS---R-VK-VTE		PEQTRETI-HLVDQ--N-
	Bacillus acanthi	WP_108670639	--M-D--E-----KQ----R-VQ-VTE		PE-TKDTVDHL-ER--E-
	Bacillus aciditolerans	WP_121446225	----R--D--KK---N---R-VQ-VTE		PE-TKETV-NL-DR--N-
	Bacillus albus	WP_071758622	--M-P--D--R---KD---R-VQ-LTD		SEDTNETI-SLIEK--D-
	Bacillus alkalitelluris	WP_078544161	-------E--KK-KQ----R-VQ-VTE		PE-TKETIAHLVDQ--N-
	Bacillus alveayuensis	WP_044748591	--E----R--RQ-EQ----R-VQ-VTE		PE-TKETISHL-EK--Q-
	Bacillus amyloliquefaciens	WP_088613415	--E-------R--RE-M--R-VQ-VTE		PE-TKETIQHF-QETA--
	Bacillus anthracis	WP_097841504	--T-P--D--R---KD---R-VQ-LTD		SEDTNETI-NLIEK--D-
	Bacillus aquimaris	WP_044338175	--L-D--E--R---EQV--R-VKEV-Q		AK-TGETIDHL-TQ--N-
	Bacillus aryabhattai	WP_033580446	--E-Q--E--RK-KQ----R-VQ---E		PE-TFETI--VKEL----
	Bacillus asahii	WP_119118476	----P--E--K--T-----R-VQ-VTE		PE-TRETI-NL-ERF-K-
	Bacillus azotoformans	WP_003330873	----S--E--RK--Q----R-VN-VTE		PE-TKETI-HV-KK--D-
	Bacillus bataviensis	WP_007087922	--L-S--D--KH-EQ----R-VQ-VTE		PE-TKETI-HL-EL--H-
	Bacillus boroniphilus JCM 2173	GAE48129	--T-P--E--K---Q----R-VH-VTE		PE-TKETVDHL-ER--H-
	Bacillus butanolivorans	WP_098177284	----P--E--K--KEQM--R-VQ-VTE		PE-TKETM-HL-ER--A-
	Bacillus campisalis	WP_046522396	--S-P--N--K---Q----R-VQ-VTE		PE-TKETV-HL-QR--H-
	Bacillus canaveralius	WP_101577290	--L-D--D--K---Q---RR-VQ-VTE		PE-TKETI-HL-ES--H-
	Bacillus cereus	WP_000601749	--T-P--D--R---KD---R-VQ-LTD		SEDTNETI-SLIEK--D-
	Bacillus cereus group	WP_016112401	--S-P--D--R---QE---R-VQ--TD		SEDTNETI-SL-EK--D-
	Bacillus cihuensis	WP_028393684	----P--D-----A-Q---R-VQ-VTE		PA-TKDTVDHL-E---Q-
	Bacillus coahuilensis	WP_010175372	--L----D--R--KE----R-VQ-LTE		GE-TEA-IH-V-ES--N-
	Bacillus cucumis	WP_101649487	--L-S--D--K---Q----R-VQ-V-E		NEDTKETADHL-ER-N--
	Bacillus cytotoxicus	WP_012096034	--T-P--E--R---QE---R-VQ-LTD		SEDTNETI-NL-EK--D-
	Bacillus deserti	WP_101645258	--M-N--D--KR---Q---R-VQ-VTE		PE-TKETI-HL-E---N-
	Bacillus dielmoensis	WP_042461777	--L-S--D--K---Q----R-VK-VTE		PE-TNETV-HL-SR-H--
	Bacillus flexus	WP_078989123	-------E--RK--QN---R-VQ---E		PE-TFETV--VKEL----
	Bacillus foraminis	TCN26896	--T-P--H--K---EQ--RR-VQ-VTE		PE-TKETI-HL-SR--R-
	Bacillus fumarioli	WP_066372489	--L-P--D--R--------R-VQ-VTE		PE-TKDTV-HL-ER-H--
	Bacillus funiculus	WP_129729469	-------E--KH-KHAT--K-VQ-VTE		SD-ARETIG-L-HK--G-
	Bacillus gaemokensis	WP_033673071	--S-P--E--R--KQE---R-VQ--TD		SEDTNETI-SL-EK--D-
	Bacillus gottheilii	WP_066451373	--E-P--E--K----S---R-VQ-VTE		PE-TKETVQHL-DL--Q-
	Bacillus halmapalus	WP_078379086	----N--E--RK-EQ----R-VK-VTV		PE-TLETI-HL--DF-K-
	Bacillus halosaccharovorans	WP_078431319	-------D--K---KE---R-VK-VTE		PE-TNEVVAHL-EL--K-
	Bacillus horikoshii	WP_064098840	----D--N--RK-EQ----R-VK-VTE		PDDT-ETV-HL-SE--Q-
	Bacillus humi	WP_057998983	----R--D--KK---T---R-VQ-VTE		PE-TKETV-HL-EI--N-
	Bacillus indicus	WP_029284221	-------D--RR--QA---R-VK--TE		PDDT-ETM-L--EK-KQ-
	Bacillus jeotgali	WP_079507069	--T-P--E--K---Q----R-VQ-VTE		PE-TKETA-HL-SL--H-
	Bacillus kochii	WP_095371991	--I----D---K-KE----R-VQ-VTE		PE-TKETITHL-SA--N-
	Bacillus litoralis	WP_066329925	--E-D--D--KH-KQ----R-VK-VTE		PE-TNEVVANL-E---K-
	Bacillus loiseleuriae	WP_049683062	----P--D--K--T-Q---R-VQ-VTE		PE-TKDTVAHL-E---Q-
	Bacillus massiliglaciei	WP_110928581	----P--E--K--EQD---R-VQ-VTE		PDDTKETV-HL-ER--K-
	Bacillus massilionigeriensis	WP_075982789	--I-S--D--K---QS---R-VQ-VTE		PE-TKETVDHL-----Q-
	Bacillus massiliosenegalensis	WP_019156198	--T-R--E--K---QQ---R-V--VTE		PE-TKETI-HL-ER--Q-
	Bacillus mediterraneensis	WP_071461303	--R-P--H--K--EK-V--R-VQ-VTE		PE-TKETIGHL-----R-
	Bacillus megaterium	RBN41904	--E-Q--E--RK-KQ----R-VQ---E		PE-TFETI--VQEL----
	Bacillus mesonae	WP_066386868	--I-P--E--R---Q----R-VQ-VTE		PE-TKETV-HL-KQ-K--
	Bacillus methanolicus	WP_003349501	--L-S--E--K---Q----R-VQ-VTE		PE-TKETVQHL-E---H-
	Bacillus thermocopriae	WP_128999744	--M----H--RK-EQ----R-VQ-VTE		PE-TKEAMLHL-ER--N-
	Bacillus thuringiensis	WP_087959398	--T-P--D--R---KD---R-VQ-LTD		SEDTNETI-NLIEK--D-

Other Bacteria
(0/>300)

Bacillus timonensis	WP_010676455	----R--D--KK---N---R-VQ-VTE	PE-TKETI-HL-DRF-N-
Bacillus tuaregi	WP_071393653	--M-T--E--K---QA---R-VQ-VTE	PE-TKATIDHL-SE-H--
Bacillus vietnamensis	WP_034764016	--L-D--E--R--KEQV--R-VKEV-Q	VE-TGETIDHL--Q--E-
Bacillus weihaiensis	WP_072578568	--E-N--D--R--KQ----R-VK-VTE	PE-TNEVVGHL-EL--K-
Bacillus wiedmannii	WP_088091976	--T-P--D--R---KD---R-VQ-LTD	SEDTNETI-SLIEK--D-
Bacillus zeae	WP_119112163	--T-P-----K--EK-V--R-VQ-VTE	PE-TKETMGHLV----R-
Bhargavaea beijingensis	WP_092097843	--M-D-----RS--Q----R-V--VTE	S-DAKQAIS-LIREN-D-
Bhargavaea cecembensis	WP_008300338	--M-D-----RS--Q----R-V--VTE	-HDIKQAIG-LVREN-D-
Bhargavaea ginsengi	SEJ83122	--M-N-----RS--Q----R-V--VTE	SPDVKQAIG-LIKEN-D-
Butyricicoccus sp. 1XD8-22	RKJ52323	--E-K----------------II-V--	AK-SKE-M----Q---T-
Caldibacillus debilis	WP_020155164	--V-P--EI-KR-K-E---Q-VR-VTE	-E-PSETI-HLFRK--E-
Caryophanon latum	WP_066462405	--M-D------S--QK---R-V-K---	EDQTKEAIRELNKS-R--
Caryophanon tenue	WP_066544784	--M--------A--QK---R-V--V--	EDQTKEAIRELNKS-R--
Chryseomicrobium excrementi	WP_100353754	--E-Q-ID--K---QA---R-V----D	GAHFSDTVDHF-EM--N-
Edaphobacillus lindanitolerans	SIT91417	--E-D-----KS-EQ----R-V--VTE	PGDARQAIS-LGEA--D-
Exiguobacterium alkaliphilum	WP_034817230	--I-Q-AD---H-EQ----E-VK--GA	VDD-SLTF-HLK-R----
Exiguobacterium aurantiacum	WP_029334630	--I-Q-AD---H-EQ----E-VK--GA	VDD-SLTF-HLK-R----
Exiguobacterium chiriqhucha	WP_029595093	--T-Q-AD---H-EQ----E-VK--GA	VED-SLTF-HLK-R----
Exiguobacterium mexicanum	WP_034778457	--T-Q-AE---H-EQ----E-VK--GA	VED-SLTF-HLK-R----
Exiguobacterium profundum	WP_074036352	--T-Q-SD---H-KQ----E-VK--GA	VDDLSLTF-HLKER----
Exiguobacterium sp. AB2	WP_034807057	--I-Q-AD---H-EQ----E-VK--GA	VDD-SLTF-HLK-R----
Exiguobacterium sp. AM39-5BH	WP_128122086	--T-Q-AD---H-EQ----E-VK--GA	VED-SLTF-HLK-R----
Exiguobacterium sp. AT1b	WP_074033544	--T-Q-SD---H-EQ----E-VK--GA	VDDLSLTF-HLKER----
Exiguobacterium sp. HVEsp1	WP_078147273	--T-Q-SD---H-EQ----E-VK--GA	VDDLSLTF-HLKER----
Exiguobacterium sp. S17	EPE60785	--T-Q-AD---H-EQ----E-VK--GA	VDD-SLTF-HLK-R--T-
Exiguobacterium sp. SH0S7	WP_131443108	--T-Q-AD---H-EQ----E-VK--GA	VDD-SLTF-HLK-R----
Exiguobacterium sp. SH3S1	WP_131469783	--T-Q-AD---H-EQ----E-VK--GA	VDD-SLTF-HLK-R----
Exiguobacterium sp. SH5S4	WP_131455897	--T-Q-AD---H-EQ----E-VK--GA	VDD-SLTF-HLK-R--T-
Exiguobacterium sp. TNDT2	WP_114166833	--T-Q-AE---H-EQ----E-VK--GA	VED-LLTF-HLK-R----
Exiguobacterium sp. ZOR0005	WP_047376276	--I-Q-AD---H-EQ----E-VK--GA	VDD-SLTF-HLK-R----
Geobacillus subterraneus	WP_033843137	----D--H--R---Q----R-VK-LVE	PE-TEEAVSHLFGQ--N-
Geobacillus thermodenitrifican	WP_008880235	-------H--R---Q----R-VQ-LIE	PE-TEEAVSHLFGQ--N-
Geobacillus thermoleovorans	WP_081130780	-------H--R---Q----R-VK-LIE	PE-TEEAISHLFGQ----
Geobacillus vulcani	WP_031407272	----D--H--R---Q----R-VQ-LIE	PE-TEEAISHLFGQ--N-
Halobacillus aidingensis	WP_089654169	-------D--LH--K-V--K-VD-VM-	DP-KE---QL-KD--R--
Halobacillus dabanensis	WP_075035196	----D--D--LH--RQV--K-VD-VM-	DP-KE--MKL-KD--QA-
Halobacillus litoralis	WP_128526092	----D--D--LH-EKKV--R-VD-VM-	DP-KE--MQL-KD--RT-
Halobacillus sp. SKP4-6	WP_128541426	----D--D--LH--Q-V-RK-VD-VM-	DP-KEN--QL-NET-K--
Halobacillus trueperi	WP_115822660	-------D--LH--K-V--K-VD-VM-	DP-KE---QL-KD--R--
Jeotgalibacillus soli	WP_041091072	--L----D--K---E----R-VQ-VTE	PE-TRETI-HLVEE-QD-
Jeotgalibacillus sp. R-1-5s-1	WP_134375891	--L-N--E--KQ-KQ----R-VQ-VTE	PE-TRETI-HL-KD-KG-
Kurthia gibsonii	WP_121176978	--E-D-----DA----V-QR-VQ-VTN	-VGQ-E-I---KEQ-QN-
Kurthia senegalensis	WP_010306678	----D--D-----------R-V----D	SGTQKD-IA-MV-E-NQ-
Kurthia sp. 11kri321	WP_068451415	--E-D-----DA----V-QR-VQ-VTN	-VGQ-E-I---KEQ-QH-
Lysinibacillus acetophenoni	WP_097149998	-------R-----------K-VM-V--	SK-SKD-I----KA--T-
Lysinibacillus boronitolerans	WP_036078638	--R--------A---A---K-V---TD	-K-T-DTVA--KT---T-
Lysinibacillus chungkukjangi	WP_107935072	--S-D--H-------E-----VM-V--	-K-SKE-I----V---T-
Lysinibacillus composti	WP_124765459	---------------D-----VH--T-	SK-S-G-I----I---T-
Lysinibacillus contaminans	WP_053582205	--S--------A---D---K-V---TD	-K-T-ETVA-LK-H--T-
Lysinibacillus endophyticus	WP_121214265	----D--R-------E-----VM-V--	-K-SKE-I--------T-
Lysinibacillus fusiformis	WP_025116635	--R--------A---A---K-VA--TN	-K-T-DTV---K----T-
Lysinibacillus halotolerans	WP_122972500	----D------Q---D-----VM-V--	-KDSKN-I--------T-
Lysinibacillus macroides	WP_053995222	--R--------S---A---K-VA--TD	-K-TLETVA--K----T-
Lysinibacillus manganicus	WP_036186982	---------------------VM-V--	KK-AKE-I----EHFT--
Lysinibacillus mangiferihumi	WP_107894885	--R--------A---A---K-V---TD	-K-T-ETVA-LR----T-
Lysinibacillus massiliensis	WP_036173103	--E-K----------------VI-V--	AK-SKE-M----Q---T-
Lysinibacillus parviboronicapi	WP_054767628	--R--------A---A---K-V---TD	-K-T-ETMA-LKV---T-
Lysinibacillus saudimassiliens	CEA04175	--A-------R--------R-V--LT-	-EDTNATFAT-R-H--T-
Lysinibacillus sinduriensis	WP_036201130	--S-D--D-------E-----VM-V--	-R-SKE-I----V---T-
Lysinibacillus sp. B2A1	AVK87079	--R--------A---A---K-V---TD	-K-T-ETVA--K----T-
Lysinibacillus sp. BF-4	WP_036145545	--A-------R--------R-V--LT-	-EDTNATFAT-R-H--T-
Lysinibacillus sp. BK089	WP_132361829	--R--------A---A---K-V---TD	-K-T-ETVA--K----N-
Lysinibacillus sp. FJAT-14222	WP_053593189	--R--------A---A---K-V---TD	-K-T-ETVA--K----N-
Lysinibacillus sp. FJAT-14745	WP_053484238	--R--------A---A---K-V---TD	-K-T-ETVA--K----N-
Lysinibacillus sp. Marseille-P	WP_106780667	--K-D----------A-----VL-V--	SK-SKD-I--M-K---T-
Lysinibacillus sp. OL1	WP_131521246	--R--------A---A---K-V---TD	-K-T-DTVA--KT---T-
Lysinibacillus sp. SYSU K30002	WP_126660461	----N----------D-----VM-V--	-K-SKD-I----V---T-
Lysinibacillus sp. YLB-03	WP_118876297	--S------------A-----VM-V--	AK-SKE-I----V---T-
Lysinibacillus sp. YR326	WP_134022511	--R--------A---A---K-V---TD	-K-T-ETVA--K----E-
Lysinibacillus sp. ZYM-1	WP_054610697	--R--------A---A---K-VA--TN	-K-T-DTV---K----T-
Lysinibacillus sphaericus	WP_010857443	--R--------A---A---K-V---TD	-K-T-ETVA-LRL---T-
Lysinibacillus sphaericus C3-4	ACA38167	--R--------A---A---K-VA--TN	-K-T-DTV---K----T-
Lysinibacillus tabacifolii	WP_108031001	--R--------A---A---K-V---TD	-K-T-ETVA-LR----T-
Lysinibacillus telephonicus	WP_126294288	--T----R-------E-----VM-V--	-K-TKV-I--V-----T-

Other Bacteria (0/>300)

Lysinibacillus varians	WP_025220415	--R--------A---A---K-V---TD	-K-T-ETVA-LR----T-
Lysinibacillus xylanilyticus	WP_049669083	--R--------A---A---K-V---TD	-K-T-ETVA--K----E-
Lysinibacillus xyleni	WP_097073857	----D----------D-----VM-V--	-K-SKE-I--------T-
Macrococcus bovicus	WP_133451452	----P-ME--E---STV-QR-VKDDLK	DA-VEA-IDE-M-H--T-
Macrococcus brunensis	WP_133430781	----P-M---E---SSV-QR-VKDDLK	DA-VEA-IDE-M-H--T-
Macrococcus hajekii	WP_133429609	----P-MH--E---SSV-QR-VKDDLK	DS-LE--MNE-MEH--T-
Oceanobacillus chungangensis	WP_115748430	----D--N---K--Q-V--K-VE-VM-	NN-DNEVIN-LIT-TA--
Oceanobacillus sp. YLB-02	WP_121521783	----D--D--S--KK-V--K-VQ--M-	ETDEAEDT-K---T-KE-
Paenisporosarcina indica	WP_075619945	--M-S--DI--K--QD---R-VA--N-	PE-ANQTIAHLVEQ----
Paenisporosarcina quisquiliaru	WP_090567046	--E-K--D------Q----R-V--V-D	SNKAIQTIQHLKEL--N-
Paenisporosarcina sp. HGH0030	WP_016429040	--M-S---I--T-EQ----R-VA-VN-	PESASQTIAHLVEQ--T-
Parageobacillus caldoxylosilyt	GAJ39782	----------RQ-KQE---R-VQ-VVE	PE-TKETITHL-DK--N-
Parageobacillus genomosp. 1	WP_043905944	----------RQ-KQE---R-VR-VVE	PE-TKETITHL-DK--N-
Parageobacillus thermantarctic	SFA47714	----------RQ-KQA---R-VQ-VVE	PE-TKETITHL-DK--N-
Parageobacillus thermoglucosid	WP_064552343	----------RQ-KQA---R-VQ-VVE	PE-TKETITHL-DK--N-
Parageobacillus toebii	WP_062755800	----------RQ-KQEV--R-VQ-VIE	PE-TKETIAHL-DK--K-
Planococcus massiliensis	WP_052652185	--L-D------S-K-E---R-VA-VTD	-EDS-ETIKNLRKL--T-
Planomicrobium flavidum	WP_088006513	--L-D--TI--S-RE----R-VA-VTE	A-GAEETVRRLREA--A-
Pontibacillus chungwhensis	WP_036782530	--E-D--D--LH-KEKV--R-VE--M-	D-DQDEAV-T---Q-QT-
Pontibacillus marinus	WP_027445960	----D--D--LH--Q-V--R-VD--M-	DRDEEDA-ST-VSQHD--
Pontibacillus yanchengensis	WP_036819622	----D--D--R---QKV--R-VE-VM-	DRDQDD--QE--TH-D--
Psychrobacillus insolitus	PZX04804	--E-D--E-----KQ----R-V--V-E	KGQAEQTIQRLVEL--N-
Psychrobacillus psychrodurans	WP_093494919	--E-K--D------Q----R-V--V-D	SNKAIQTIQHLKEL--N-
Psychrobacillus psychrotoleran	WP_093537756	--E-N--D------QT---R-V--V-D	SNKALQTIQ-LKEL--N-
Psychrobacillus sp. FJAT-21963	WP_056832757	--E-D--E------Q----R-VT-V-E	NNQS-QTIQNLQEL--N-
Psychrobacillus sp. OK028	WP_093062578	--E-N--D------QA---R-V---ND	SSKALQTIQ-LKEL--D-
Psychrobacillus sp. OK032	WP_093269839	--E-D--E------Q----R-VT-V-D	-TQTMQTIQNLVDL--N-
Quasibacillus thermotolerans	KKB33157	--M-P--D--KS-EQ----R-VQ-VTE	PE-SKETI-HL-EQ--D-
Sediminibacillus albus	WP_093213318	------D--LH--Q-V--R-VE-VM-	DKDQED--KILME--QK-
Sediminibacillus massiliensis	WP_077624925	-------E--LH-KEQV--R-VE-VM-	D-DQDE---TM-KT-DA-
Sporosarcina pasteurii	WP_115362743	--I-D--E---T-KE----R-VA-LTE	SADAKEAI-HLKEEF-T-
Sporosarcina psychrophila	WP_067206317	--L-D--D---S-KE----R-V--VTE	PV-AKQAMAH-KEEF-A-
Sporosarcina sp. BIOO1-red	WP_116020305	--T-D---L--S-KE----R-V--VTE	PEDAKKVMSLMRQD--D-
Terrabacteria group	WP_095250805	--L-H--D--K---Q----R-VQ-VTE	PE-TKETA-HL-ER-Q--
Tetzosporium hominis	WP_094944341	--E-Q-ID--K---QA---R-V----D	GAHFSDTVDHF-EM--N-
Thermolongibacillus altinsuens	WP_132949053	--L-------RK--Q----R-VQ-VTE	PE-TKETI-HL-NK--N-
Turicibacter sanguinis	WP_055242897	----D--T--S---H-V-ER-VK-VAS	KD-SIKTV-HLIKKF-K-
Turicibacter sp. H121	WP_068759213	----D--T--S---H-V-ER-VK-VAS	EDDSIETV-HLIKKF-K-
Ureibacillus thermophilus	QBK26650	----T--E--R----E-----VM-V-D	FK-TKEAI-H--K---T-
Ureibacillus thermosphaericus	WP_016838663	----D--E-------E-----VM-V-D	SR-SKE-I-H--K-----
Vibrio vulnificus	WP_133351222	----P--D--K--KEQM--R-VQ-VTE	PE-TKETMDHL-ER--A-
Virgibacillus sp. LJ137	WP_123315126	----D--D--HR--QAV--K-VE--M-	LTDDEDEV-P---T-NQ-
Viridibacillus arvi	WP_053418649	--N-D--E---S---DV--R-V---TD	-N-RKE-I---IE-----

Supplemental Figure 63

A partial sequence alignment of the methionine ABC transporter ATP-binding protein containing a one amino acid insertion (boxed) that is exclusively shared by all members belonging to the Solibacillus clade and absent in all other bacteria. The peripherally branching *Lysinibacillus odysseyi* does not share this CSI.

Group	Organism	Accession	71		177
**Solibacillus clade (6/7)**	Solibacillus sp. R5-41	WP_099424859	FGIQSFSPVASCGKDLDEMKALALEILEDYRD	V	GDITFKVEVHRTDK
	Solibacillus isronensis	WP_079528470	-----I------E----V-----F--------	K	--L-----------
	Solibacillus silvestris	WP_065215504	-----I------E----V-----F-------E	K	--L-----------
	Solibacillus kalamii	WP_008407795	-----I------E----V-----F-------E	K	--L-----------
	Bacillus cecembensis	WP_057989274	-------------------E--I--------	A	--------------
	Lysinibacillus sp. 2017	WP_108713547	-----------E----A-----FD-------	K	--L-----------
	Lysinibacillus odysseyi	WP_036156210	------------E--MEKI----IQ-M-L-Q-		QN-------R----
**Other Bacteria (0/>100)**	Lysinibacillus meyeri	WP_107838065	----------A-E--I-A--Q--ID-MAQ-G-		EPL------R----
	Bacillus ndiopicus	WP_042474619	-----------N-NM-D--Q--I--MAQHSN		QPL------R----
	Caryophanon latum	WP_066465063	-------A---AE-NM-AI-EV-HK-M-Q-KE		P-------K----
	Anoxybacter fermentans	WP_127018295	--V-L---VKT-L-ME-I-EM--RV---ALP		DGG-----TK-AN-
	Bacillaceae bacterium SAOS 7	WP_095477429	---------VRTA-E-P---EA--ALVQKNVT		ESC----STR-S--
	Bacillus acanthi	WP_108671301	--------AVKVE---E-I-KA--FYVQ-IKE		DIR----TTK-A--
	Bacillus andreraoultii	WP_033828180	---------IRVQ--IETI-ES--NLFQKLNA		QGK----QTR-A--
	Bacillus aquimaris	WP_044339434	---------VKVAQ-IE-ISKAG-ALVQKNFE		PGK----SAR-A--
	Bacillus badius	WP_041097803	---------VRT-Q-IE----A---LVKRNFS		PGN----SAR-S--
	Bacillus campisalis	WP_046523479	-------LAVKVE----K--QA---LFRSLYS		EGK---ITAK-S--
	Bacillus circulans	WP_095332411	--------AIKLER-IEII--A----VTSLYE		EGS---ISAR-A--
	Bacillus dielmoensis	WP_042459722	--------AIKVR--M--L-QSS-DLVSSLYR		EGQ----TTK-S--
	Bacillus firmus	WP_035330328	--------AVKVN--IE----A--ALFLKHF-		EGK---ITAK-A--
	Bacillus fordii	WP_018708795	--------AIRTE----QI--A--HVV-QSHI		PGR---ISAR-S--
	Bacillus fortis	WP_120074199	--------AIRTE----QI--A--H-VKQAHI		PGG---ISAR-S--
	Bacillus freudenreichii	WP_126431494	--------AIRTE----QI--A--HVVKKAHS		PGQ---ISAR-A--
	Bacillus halotolerans	WP_059293418	-------LAIK-ESR--DI-TT--QAIK-QYQ		PGD-----TK-AY-
	Bacillus hisashii	WP_095142417	---------VRVN--MEFI-ESS-KLFQKLHA		QGK----QAR-A--
	Bacillus infantis	WP_129613917	--------AVKTE--IEKI-GA--AL-KRVYK		DGD----SAR-A--
	Bacillus intestinalis	WP_079288095	-------LAIK-DSR--DI--T--QAI--QYQ		PGD----ATK-AY-
	Bacillus jeotgali	WP_079510065	--------AVKVE---EK--DA---LFRNIYK		PGQ---ITAK-S--
	Bacillus kochii	WP_095372269	---------LKVE----T--EK--QLIQKVYQ		EGD---ITAK-A--
	Bacillus korlensis	WP_066050504	--------IKT-LE-E-I-EA--QLFLNKYK		VGD---ISTR-AN-
	Bacillus lentus	WP_066136868	--------AIKTE--I-AIRSA--H-IDQAHI		PGN----SAR-S--
	Bacillus licheniformis	WP_075747796	-------LAIK-DSR--DI--T--QAI--QYQ		PGD----VTK-AY-
	Bacillus loiseleuriae	WP_049682614	Y----L--ALKI-R-IE-I--A--HYVDQLSG		EIE----STK-S--
	Bacillus marisflavi	WP_048007454	---------VMT-R-V---MEAG-ALVKKSFE		PGK----SAR-G--
	Bacillus massilioanorexius	WP_019243882	---------MEVE--I-A---G--QYIKNLDR		KIE----TTK-A--
	Bacillus massiliogabonensis	WP_102274258	--------AIKTDRE-EN--NA--DLFKKIHQ		AGE---ITTK-S--
	Bacillus massilionigeriensis	WP_075982578	--------AVKT----EI--DT---LFKGIYK		EGD---ITAK-A--
	Bacillus massiliosenegalensis	WP_019154860	--------AIKTS---E---SA--QLFREVYT		EGKS--ITAR-A--
	Bacillus nealsonii	WP_101175687	--------AIKLE--I-T--ET--KLVSSIYQ		EGK---ITAK-V--
	Bacillus niacini	WP_045523650	--------AIKVKR-IEQ--VA--SLVKRLYK		EGQ---ITPK-S--
	Bacillus niameyensis	WP_062104248	---------LKTE-NI-AI--A--HLV-QAHS		PGN----SAR----
	Bacillus novalis	WP_066090843	--------AIKVAR-VESL-QA--QLIQNLYK		EGQ----TTK-S--
	Bacillus oceanisediminis	WP_110066304	--------AVKTN--MEA--DA--ALFQKKYE		EGK---ITAK-A--
	Bacillus persicus	WP_090741625	--------AVKVE--ITI---A---LFKRLYS		DGK---ITGK-S--
	Bacillus praedii	WP_057761379	--------AIKTD-E-EN--NA--DLFKKIHQ		AGE---ITTK-S--
	Bacillus rubiinfantis	WP_042354789	--------AVKVD--IEVI-EV--KLVRSLYR		DGA----TAK-S--
	Bacillus selenatarsenatis	WP_041965442	--------ALKVE----K--DA---LFRKIHK		PGQ---ITAK-S--
	Bacillus smithii	WP_003355663	---------VRTE-NIEKV-EA--TLVKANFS		PGN---INAR-S--
	Bacillus subterraneus	WP_044390737	--------AVKVD---EK--DA---LFKKIHQ		PEQ---ISAK-S--
	Bacillus subtilis	AAC00308	-------LAIK-DSR--DI--T--KAIK-QYK		PGD----ATK-AY-
	Bacillus tequilensis	WP_024714605	-------LAIK-DSR---I--T--QAM--QYQ		PGD----ATK-AY-
	Bacillus terrae	WP_120115478	--------AIRTE----QI--A--LVVKKSHS		PGK---ISAR-S--
	Bacillus thermoamylovorans	WP_034771688	---------VRVN--MELI-ESS-KLFQKLHA		QGK----QAR-A--
	Bacillus tuaregi	WP_071393826	---------IKT---IE---TAV-DLFKKVYS		EGQ---ITTK-A--
	Bacillus vallismortis	WP_010329657	-------LAIK-DSR---I--T--QAI--QYQ		PGD----ATK-AY-
	Bacillus wudalianchiensis	WP_065408771	---------VRTDQNIE---TA---LVKRNFS		LGK----SAR-S--
	Bhargavaea beijingensis	WP_092097650	--------AVR-KAN--SI-ET-ER--SGMET		EGK------R-A--
	Bhargavaea cecembensis	WP_008300211	--------AVR-KA--ETI-ET-ER--S-MDT		EGK------R-A--
	Bhargavaea ginsengi	WP_092051923	--------AVR-RA--ETI-ET-ER--SGMDT		EGK------R-A--
	Brochothrix thermosphacta	ANZ94568	-----Y---IKVEQ-IEQI-EM-VAL-QRHYK		EGQS---RTK-A--
	Brochothrix thermosphacta DSM	EUJ37440	-----Y---IKVEQ-IEQI-EM-VAL-QRHYK		EGQS---RTK-A--
	Carnobacterium divergens	WP_034569122	----TY---VKVER-IEAL--A-I-MIRNVYQ		EGQ---ITTR-A-
	Chryseomicrobium excrementi	WP_100352251	----------KAE-TI-A-A-TTIA--SELET		SGK-C--T-K-S--
	Domibacillus aminovorans	WP_063964528	--------VKTSQ------EA--GLMKRAMI		-TT-----TR-A--
	Domibacillus antri	WP_075398309	--------VKTSQE---V--A---LVKKAMS		-GT-----TR-A--
	Domibacillus epiphyticus	WP_076764579	--------VKTSQE-N-I--A---LVKRSMS		-KT-----TR-A--
	Domibacillus mangrovi	WP_073710337	--------VKTSQE-----EA---LVKRAMI		-AT-----TR-A--
	Edaphobacillus lindanitolerans	WP_076758201	--------AVR-DAEIE-I-QT-A---GRMET		EGK-----AR-A--
	Enterococcus timonensis	WP_071131869	----N---MIKVP---EII--T---LMKEAYE		PGL----NTK-A-
	Falsibacillus pallidus	WP_114745156	--------TVRT-L-IE-I-DA--SL-KKLFS		EGQ----SAR-S--
	Granulicatella balaenopterae	WP_089746131	---------YIVE--MEQV--VV-DL-K-LNP		TEKN--ITTK-S-
	Jeotgalibacillus alimentarius	WP_041120898	---------IKSDR----V-RN-VDLVRSVVS		EGTL---KAR-S--
	Jeotgalibacillus campisalis	WP_041060122	---------VKTAAN-T---EQ-VKLV-SLAH		EQA----SAR-A--
	Jeotgalibacillus malaysiensis	WP_039810399	---------VKSDRA---V-QH-A-LVKSIAK		EGTL---KAR-A--

Other Bacteria (0/>100)

Jeotgalibacillus proteolyticus	WP_104057138	---------VKTA-E---I-EQ-VLLVSSIKK	TSA----SAR-A--
Jeotgalibacillus salarius	WP_134381539	---------VKS-R----V-NH-SQLVKSVAT	EGTL---KAR-A--
Jeotgalibacillus soli	KIL43860	---------VKTS-----I-SASTVLVAELV-	-PT---IAAR-A--
Kurthia huakuii	WP_029499465	----------E-AT--EA----GK--VSGLDY	EGKS---N-K-SY-
Kurthia massiliensis	WP_010288305	----------E-PT---A----GK--VSTLDY	AGKS---N-K-SF-
Kurthia senegalensis	WP_010303783	--V--Y----E-PT---A-----K--VQTLDY	EGK----N-K-SY-
Kurthia sibirica	WP_109305245	----------E-PTT-ED--K--V--VSSIDF	EGK----N-K-SY-
Kurthia zopfii	WP_109349057	----------E-PTT-ED--N--V--VKGIDY	EGK----N-K-SY-
Lactobacillus concavus	WP_057825497	----N---SLKIA--VETL-QT---MMQELVP	VAK---IDTR-S-
Lactobacillus coryniformis	WP_003679620	----N---SIRVER-M-QVR-T-IAMIKEQYQ	PGD----NTR-S-
Lactobacillus kosoi	WP_108982344	---ET---AMKVE--I-AV-KV---MVK-QYK	PGM---IDTR-Q--
Lactobacillus micheneri	WP_105964843	---ET---AMKVE--I-AV-KV---MVK-QYK	PGM---IDTR-Q--
Lactobacillus mudanjiangensis	WP_130845671	----N---SIRVAQ-M--VY-T-I-MMKAQFK	PGM----NTR-S-
Lactobacillus parabrevis	WP_020088376	---EN---SVKVA--I--I-QT-IAMVKEVFK	PGM---INTR-Q--
Lactobacillus paracasei	WP_016373196	----N---SIAVE--M-KVH-V--QLMNETAP	KG-SY--NTR-S-
Lactobacillus paracasei subsp.	EPC72797	----N---SIAVE--M-KVH-V--QLMNETAP	KG-SY--NTR-S-
Lactobacillus pasteurii	WP_009559994	----TY---IKTE-S-ESI-ET----MQATYE	AGM----NTR-S-
Lactobacillus pasteurii DSM 23	KRK08709	----TY---IKTE-S-ESI-ET----MQATYE	AGM----NTR-S-
Lactobacillus paucivorans	WP_057878078	---EN---SVKVD----AI--A-VAMVKEVFE	PGM---INTR-Q--
Lactobacillus pobuzihii	WP_017868325	---------IKVTR-VE-V-KT-IS-FNEIAQ	DGM---INTR-S-
Lactobacillus sharpeae	WP_054679877	--------SVQVE--M-KV--A---LVNATAA	NGV-----TK-A--
Lactobacillus timberlakei	WP_105988245	---ET---AMKVE--I-AV-KV---MVK-QYK	PGM---IDTR-Q--
Listeria booriae	WP_036087737	--------AVRVPQ-VE---RAG--LVRAMHQ	PGGS---NAR-S-R
Listeria cornellensis	WP_036079376	--------AVRVPQ--E---QAG-SLVRAMHQ	AGGS---NAR-A-R
Listeria innocua	WP_010991623	--------AVRVNL----V--A--SLVQ-AHE	ENG----AAR-SHR
Listeria ivanovii	WP_111125109	--------AVRVDL--E-V-KA--ALVQ-THE	-NG----AAR-SHR
Listeria monocytogenes	WP_070752986	--------AVRVNL----V--A--SLVQ-AHE	ENG----AAR-SHR
Listeria newyorkensis	SQC56209	--------AVRVPQ--E---QAG-SLVRAMHQ	AGGS---NAR-A-R
Listeria rocourtiae	TDR53520	--------AVRVAQ--E---QAG-SLVRAMHQ	MGG----NTR-A-R
Listeria weihenstephanensis	WP_036061532	--------AVRVPQ--E---QAG-SLVRAMHQ	PGGS---NAR-A-R
Lysinibacillus acetophenoni	WP_097148564	----------I-E-NM-A--S--I--MN-FK-	Q--------R-S--
Lysinibacillus boronitolerans	WP_016991572	----------A-E--M-S--K--IT-M-TFK-	EQH------K----
Lysinibacillus chungkukjangi	WP_107935546	----------V-T-E-----S---I-M-QFKE	QEL------R-S--
Lysinibacillus composti	WP_124762503	----N-----V-E--V-SI-D--IK-MDL-KE	KE-------R-S--
Lysinibacillus contaminans	WP_053582442	----------A-E--M-I--D--IT-MDTFK-	EQL------K----
Lysinibacillus endophyticus	WP_121212687	----------Y-E---ES--N-S-L-M-QFKE	QQ-------R-S--
Lysinibacillus fusiformis	WP_004226153	----------A-E--M-S--K--IT-M-TFK-	EPH------K----
Lysinibacillus halotolerans	WP_122971091	----------V-E-------K-S-I-M-Q-KE	KE-------R-S--
Lysinibacillus jejuensis	WP_108306415	----------A----IE---Q--KDVMKQ-E-	QTY------K----
Lysinibacillus macroides	WP_053996475	----------A-E--IES--Q--VT-MDTFKE	AQHS-----K----
Lysinibacillus manganicus	WP_036188172	----------V-D-N--A--T--R--MD-FKN	KE-------K-S--
Lysinibacillus mangiferihumi	WP_107895054	----------A-E--M-A--Q---T-MDTFK-	QQL------K----
Lysinibacillus massiliensis	WP_036174810	----------V-E-T--A--K--I--MD-FKE	QQV------R-S--
Lysinibacillus parviboronicapi	WP_107923384	----------A-N--MEA--S--IT-MDTFKS	EKL------K----
Lysinibacillus saudimassiliens	CEA05479	----------A-E--IEVI-Q--KDVMKQ-E-	ETY------K----
Lysinibacillus sinduriensis	WP_036198619	----------VSS-N--D--K-S-I-M-QFK-	QQL------R-S--
Lysinibacillus sphaericus	WP_010860204	----------A-D--IEA--S--IT-MDTFKH	EQL------K----
Lysinibacillus tabacifolii	WP_108029960	----------A-E--M-A--R---T-MDTFK-	QQL------K----
Lysinibacillus telephonicus	WP_126294622	---------SI-E-N-ED--K---TVM-QFK-	RQ-------R-S--
Lysinibacillus varians	WP_025220314	----------A-E--M-A--R---T-MDTFK-	QQL------K----
Lysinibacillus xylanilyticus	WP_049667478	----------A-E---EA--K--IT-MDTFKN	EQL------K----
Lysinibacillus xyleni	WP_097072894	----------F-E--IES--N-S-L-MDQFKE	QQL------R-S--
Macrococcus canis	WP_086042986	--VH-V---VKVE--I-QI--S-VKLAQ-IDT	PGV----DAK-S--
Macrococcus caseolyticus	WP_086038954	--VH-V---VKVE--I-QI-SS-VKLAR-IDA	PGV----DAK-S--
Methanomassiliicoccales archae	OPY34176	--VA-V---VK--SNME--R-RVA-LSRNWLE	EGS---I-AR-S
Micrococcus lylae	WP_115113073	--VH-V---VKVE--I-QI-SS-VKLAR-IDA	PGV----DAK-S--
Mycobacteroides abscessus subs	SHQ01771	--------IKTSL--E-V-EA--QLFLNNYK	VGD---ISTR-AN-
Mycoplasma gallinarum	WP_027332607	---S-Y---VV-E---ETI-KT-AAL-SKNQA	K-----AR-S--
Novibacillus thermophilus	WP_077721277	---V-V---VRTEL-IG-V-GA--ALM-KHDM	PNV-----TQ-AN-
Paenisporosarcina antarctica	WP_134209351	------------SL--EDI-QT-TKVMDSLDR	T-K----S-K-SY-
Paenisporosarcina indica	WP_075620072	------------SQ--EDI-QT-IKVI-SLDR	ENK----T-K-SY-
Peptococcaceae bacterium DCMF	ATW25824	---Y-V---IKTE----AVQKA--RV-H-ALP	QGG-----TK-S--
Pilibacter termitis	WP_078806789	---------VRVE---E---KM-VS-VREQFF	EGM---ITSK-S-
Planococcus antarcticus	WP_006830000	---------TA-EL--EA--KT-EAVIAKIQ-	T-KS--IS-K-PF-
Planococcus citreus	WP_121301144	---------TQTSLE-----HT--S-VSKMDT	K-KS---S-K-PN-
Planococcus donghaensis	WP_008429084	---------TA-EL-IEA--KT-EAVIAKIE-	T-KS---S-K-PY-
Planococcus faecalis	WP_071153168	---------TA-EL-MEA--KT-EAVIAKIE-	T-KS---S-K-PF-
Planococcus maitriensis	WP_112233802	---------TQTSL---D--QT--S-ISKMDT	DGKS---S-K-PY-
Planococcus maritimus	WP_068461622	---------TQTSLE-----RT--S-VSKMDT	Q-KS---S-K-
Planococcus plakortidis	WP_068868848	---------TQTSLE-----HT--S-VRKMDT	AGKS---S-K-PY-
Planococcus rifietoensis	WP_058381107	---------TQTSLE--D--HT--A-VSKMDT	K-KS---S-K-PN-
Planococcus salinus	WP_123163575	---------TES-LSIE---NT-MS-VGKMDT	AGK----S-K-AN-
Planococcus versutus	WP_065524318	---------TA-NL-M-M--KT-EVVIAKLDN	T-KS---S-K-
Planomicrobium flavidum	WP_088006648	---------VQVP--IGG--EE-IKLVASLKP	EGK---TT-K-S--

Other Bacteria (0/>100)

Psychrobacillus insolitus	WP_111437643	------------SQ---DI--T--K-MDGLEY	EGK--R-T-K----
Psychrobacillus psychrotoleran	WP_093536870	------------S-EM-DIQ-T--Q-MDSLDS	Q-K--R-T-K-S--
Quasibacillus thermotolerans	WP_039234176	---------VRTEQNIE-IQ-A---LVRKNFS	KGK----SAR-S--
Rummeliibacillus	WP_119415087	---H------Q-EPTIEA--E--V--VKGMDY	KGK----NAK-SF-
Rummeliibacillus pycnus	WP_102691052	---H------Q-EPT-EV--D--V--VKGMDY	KGK----N-K-SF-
Rummeliibacillus stabekisii	WP_066788897	---H------E-EPTVEA--E--V--VQGLDY	EGK----NAK-SF-
Sporosarcina globispora	WP_053434104	--------AVKVN--VE---DA--ALFLKDYQ	EGKS--ITAK-A--
Sporosarcina koreensis	WP_040286179	----Y--I-Q-ESTVESIRST--GVF-HEET	LGR------K----
Sporosarcina newyorkensis	WP_009497687	--------I-I-EPT-EAI-QTS-DVIGNTET	KGK------K----
Sporosarcina psychrophila	WP_067207559	----------K-EAT-EAI-EK----IGANET	AGK------R----
Sporosarcina ureae	WP_029052871	--------IVI-APT-EDI-ETS-KV-GKTET	AGK----D-K-A--
Staphylococcus aureus	WP_037588526	--VH-V---VRIE----L--EV--KFAH-FK-	- ---ID-K-A--
Staphylococcus carnosus	RTX87725	---K-V---HKTTQ------N-CVKLAQNFSG	- ---ID-K-V--
Staphylococcus felis	WP_103208248	Y-VH-I---VKIK-TM--VYQY-VRFAKE---	- S--I--K-S--
Staphylococcus fleurettii	WP_119634187	--VN-V--IIRIE-E--I--EV-IKFA--FN-	- ---ID-K-A--
Staphylococcus haemolyticus	PNH24046	---K-I---HKTSQE---I-K-CVDLAQ-FKP	E ---ID-K-V--
Staphylococcus hominis	WP_002448303	---K-I---FKTTQN-ED--Q-CVSLAK-FAN	- ----D-K-V--
Staphylococcus lentus	WP_017000630	--VH-V---VRIE-E--V--EI--QFANEFN-	- ---ID-R-A--
Staphylococcus muscae	WP_095117155	--VH-I---LKIE-NM-SVY-H-IKFA-K-HE	- S-----K-S--
Staphylococcus petrasii subsp.	PNZ71052	---K-V---HKTTQE--DI-N-CV-LAQNFSG	- ---ID-K-V--
Staphylococcus saprophyticus	WP_115344851	---K-I---FKTTQN-ED--Q-CVSLAK-FAN	- ----D-K-V--
Staphylococcus schleiferi	WP_126477580	--VH-V---VRIE----L--EV--KFAH-FK-	- ---ID-K-A--
Staphylococcus sciuri	RIO74079	--VH-V---VRIE----L--EV--KFTH-FK-	- ---ID-K-A--
Staphylococcus stepanovicii	WP_095087431	--VH-V---VRID-EI-I--EV-IKFA--FKS	E ---ID-K-A--
Staphylococcus succinus	WP_046836705	---Y-I---LKIE-TI-AA--Q-V-FAK--AE	- ---ID-K-S--
Staphylococcus vitulinus	WP_016912208	--VH-V---VRIE-E--A--EI-SKFAD-FK-	- ---I--K-A--
Tepidanaerobacter acetatoxydan	WP_013778661	---VYI---L--EN--EQI-RA---VVKGQKF	EGK-----TR-PN-
Tetzosporium hominis	WP_094944064	----------KTE-TI-A-A-TTIA--SELET	SGK-C--T-K-S--
Trichococcus alkaliphilus	WP_106448804	----NY---YLVSR----V-RVLV-L-AKMET	AGK---IATR-A-
Trichococcus collinsii	WP_086985399	----N----YLVSR----V-RVLV-L-AKMET	AGK---IATR-A-
Trichococcus ilyis	WP_068623208	----NY---YLVSR----V-RVLV-L-AKMGT	AGK---IATR-S-
Trichococcus patagoniensis	WP_108031373	----N----YLVSR----V-RVLV-L-AKMET	AGK---IATR-A-
Turicibacter sanguinis	WP_055241181	--L--Y-LA-R-ENEI-AI-------INEQPK	VPT-----TK-S--
Turicibacter sp. H121	WP_068759505	--L--Y-LA-R-ENEI-AI-------INEQPK	VPT-----TK-S--
Ureibacillus thermophilus	QBK26531	----------M-E---GVIQQT-QK-MDR-K-	KS---------S--
Ureibacillus thermosphaericus	WP_026018967	----------I-DREIENIQQ--QK-MDR-K-	QQ---------S--
Vagococcus fluvialis	WP_114289587	---------IKIE--T-VICET-V-MVKEIWE	KGQ----QSR-S--
Vagococcus martis	WP_079347468	---------VRIE--PELICQT---MVK-IWE	PGK----QSR-S--
Vagococcus teuberi	WP_071457359	---------VRIE--PELICQT-V-MVK-IWE	PGK----QSR-S--
Viridibacillus	WP_038187845	---H------K-EPT--A--E--IN-MKSINT	DGK----NAK-S--
Viridibacillus arvi	WP_053416630	---H------K-EPT--A--E--IN-MKSIDT	DGK----NAK-S--
Viridibacillus sp. OK051	WP_100794527	---H------K-EPTI-A--E--VD-MKSIDT	DGK----NAK-SF-

Supplemental Figure 64

A partial sequence alignment of the tRNA 4-thiouridine(8) synthase ThiI protein containing a one amino acid insertion (boxed) that is exclusively shared by all members belonging to the Solibacillus clade and absent in all other bacteria. The peripherally branching *Lysinibacillus odysseyi* does not share this CSI.

Group	Organism	Accession	315		361
**Solibacillus clade (6/7)**	Solibacillus sp. R5-41	WP_099422549	WDIEKNGLQNVRDKKDLLKKLIAEAES	L	VHTNSNLARASELQYGKIP
	Solibacillus isronensis	WP_079524767	-EL------I------E---ME--RDD	-	FISG----------S---
	Solibacillus silvestris	WP_014824617	-EL------I------E---ME--RDD	-	FISGN---------S---
	Solibacillus kalamii	WP_087615578	-EL------I------E---ME--RDD	-	FISGN---------S---
	Bacillus cecembensis	WP_057984285	---------I------R--NY-----E	-	-Q--------G----S---
	Lysinibacillus sp. 2017	WP_108711666	-EL--S---V------Q---YQ----Y	-	IN-G--F--------SQ--
	Lysinibacillus odysseyi	WP_081977990	-EL------KLQK-REQ-D-YRRDL-D		AESKYD-NK-A--RH----
**Other Bacteria (0/>200)**	Lysinibacillus meyeri	WP_107838802	-EA--AS--SIQN-REQ-D-YRRDL-D		AESKYD-NK-A--RH----
	Lysinibacillus fluoroglycofenilyticus	WP_107942153	-EA--AD--LIQQ-REQ-G-YRRAL-E		AESKYD-NQ-A--RH----
	Caryophanon latum	WP_066461481	-KL--D-I-TLQK-REQ-D--RRDSDD		AQMRGDYNK-A----S---
	Caryophanon tenue	WP_066542970	-KF--E-I-TLQA-REQ-D--RR-S-D		AQMRGDYN--A----SQL-
	Acidobacteria bacterium	PZN88432	-QQ--AAI-G--TQ-EE-ER-RI---Q		AERAGDY------R--R--
	Actinokineospora bangkokensis	WP_075977858	-QN--GS-NR---L-EQ-ET-RG-S-R		AERDGD-GK-A--R--R--
	Actinokineospora enzanensis	WP_018686746	-QN--GSIDR--EL-EQ-EA-RG-S-R		AERD-D-G--A--R--R--
	Actinophytocola xanthii	WP_075128825	-QN--GSIEK--EL-EQ-EQ-RG-S-R		AERDGD-G--A--R--R--
	Actinophytocola xinjiangensis	OLF09441	-QN--GSIER--EL-EQ-EQ-RG-S-R		AERDGD-G--A--R--R--
	Aestuariimicrobium kwangyangen	WP_022909366	-EQ--S--NK-GEI-EEID--RT--DR		LMRQGD--K-A-I---R--
	Amycolatopsis alba	WP_020632408	-QN--GSIER---L-EQ-EQ-RG-S-R		AERDGD-GK-A--R--R--
	Amycolatopsis albispora	WP_113695466	-QN--GSIER---L-EQ-EQ-RG-S-R		AERDGD-G--A--R-----
	Amycolatopsis antarctica	WP_094864352	-QN--GSIE----L-EQ-EQ-RG-S-R		AERDGD-G--A--R--R--
	Amycolatopsis halophila	WP_034267826	-QN--GSIDKI--L-EE-ER-RG---R		AERDAD-G--A--R-----
	Amycolatopsis lurida	WP_034312365	-QN--GSIER---L-EQ-EQ-RG-S-R		AERDGD-GK-A--R--R--
	Amycolatopsis marina	WP_091668364	-QN--GSIEK--EL-EQ-EQ-RG-S-R		AERDGD-G--A--R-----
	Amycolatopsis methanolica	WP_017985662	-QN--GSIEK--EL-EQ-EQ-RG--DR		AERDAD-G--A--R-----
	Amycolatopsis nigrescens	WP_020669286	-QN--GSIEK---L-EQ-EQ-RG-S-R		AERDGD-G-----R-----
	Amycolatopsis palatopharyngis	WP_116050134	-QN--GSIEK---L-ER-EQ-RG-S-R		AERDGD-G--A--R-----
	Amycolatopsis ruanii	WP_116114708	-QN--GSIEK--EL-EQ-EQ-RG--DR		AERDAD-G--A--R-----
	Amycolatopsis sacchari	WP_091508096	-QN--GSIEK--EL-EQ-EQ-RG--DR		AERDAD-G--A--R--R--
	Amycolatopsis taiwanensis	WP_027941434	-QN--GSIEK---L-ER-EQ-RG--DR		AERDAD-G--A--R-----
	Amycolatopsis thermalba	WP_116102079	-QN--GSIEK--EL-EQ-EQ-RG--DR		AERDAD-G--A--R-----
	Amycolatopsis xylanica	WP_091288653	-QN--GAIER--EF-EQ-EQ-RG-S-R		AERDGD-G--A--R--R--
	Anaerolineaceae bacterium	OQY24838	-QQ--AAI-KL--I-ERIEQVRH-I-Q		-QRSG--E--------LL-
	Antarctobacter heliothermus	WP_089276957	-QA-RDK-ASA--I-EE-DH-R--L--		AKRDG-F-K-G-----R--
	Bacillus asahii	WP_119118133	-QM--AS--Q-Q-MREQ-E--RR-L-Q		AENDYD-N--A--RH----
	Bacillus butanolivorans	WP_053344987	-QM--SA--K-Q-QREE-E--RH-L-Q		AENDYD-N--A--RH-R--
	Bacillus canaveralius	WP_125927984	-QL--EAI----Q-RE--E--RR-LAE		AENQYD-N--A--RH----
	Bacillus cohnii	WP_066416676	-EL--QSI-GI-E-RE--ER-KR-L-E		AEEKYD-NK-A-IRH--L-
	Bacillus dakarensis	WP_077211177	-QA--E-I-A--E-RE--E--RR-L-D		AEN-YD-NK-A--RH-R--
	Bacillus horneckiae	WP_066392793	-EL--E-IHK-QQQRET-E--RR-L-E		AEN-YD-NK-A--RH----
	Bacillus massilioanorexius	WP_019240478	-EL--S---KIQE-RE--E--KRQL-D		AENAYD-NK-A--RH----
	Bacillus massiliogorillae	WP_042349517	-EL--S----IQH-REQ-ETYKRQL-E		AEN-YD-NK-A--RH----
	Bacillus muralis	WP_057912437	-QM--SA--K-Q-QREE-E--RH-L-Q		AENDYD-N--A--RH-R--
	Bacillus oceanisediminis	WP_110065342	-QL--E-I-K-QE-REQ-E-MRRDL-E		AEN-YD-NK-A--RH-R--
	Bacillus praedii	WP_057763845	-EL--E---R-QQ-REM-E--RH-LQD		AEN-YD-NK-A--RH----
	Bacillus simplex	WP_061143034	-QM--SA--K-Q-QREE-E--RH-L-Q		AENDYD-N--A--RH-R--
	Bacillus solani	WP_056683517	-EL--E-I-R-QH-REM-E--RH-LQD		AEN-YD-NK-A--RH----
	Candidatus Nomurabacteria bact	OGI96819	-QN---A-TDL--I-KS-EA-RL----		AEARAD---VA-IR--R--
	Candidatus Thiodiazotropha end	ODC00289	-KS--AA--GTTHI-ES-E-ARL-L-T		A-RAGD---M------R--
	Caulobacteraceae bacterium OTS	WP_066771688	-SA--QK--VTSKS-EE-E--QT-Y-Q		AVRQG--------K--R--
	Corynebacterium heidelbergense	WP_112769289	--N--A-IDSL-EI-EE-DN-RR-S-I		AERDG-F-KVA--R--R--
	Crossiella equi	WP_086781312	-KG---AIDRI-EL-EQ-EQ-RG-S-R		AERD-D-G--A--R--R--
	Ferrimicrobium acidiphilum	WP_035392520	-QL-RD-IGKI-EM-ER-EQ-KTD--G		DERVG--D--A-IR--Q--
	Gemmobacter intermedius	WP_128489855	-QA-RDR-EGA--L-EK-E-AR--LDI		AKRDG-F-K-G-----R--
	Gemmobacter megaterium	WP_076532188	-QA-RDR-EGT--L-EQ-D-AR--LDI		AKRDG-F-K-G-----R--
	Gordonia aichiensis	WP_005169009	-QS--TAIDA---T-EE-ER-RG--DR		AERDGD-G--A--R--R--
	Gordonia alkanivorans	WP_127962366	-QS--TAIDA---L-EE-DR-RG---R		AERDGD-G--A--R--R--
	Gordonia amicalis	WP_006438139	-QS--TAIDA---L-EE-DR-RG--DR		AERDGD-G--A--R--R--
	Gordonia araii	WP_007321829	-QS--SAIDA---T-EQ-EH-RG--DR		AERDGD-G--A--R--Q--
	Gordonia bronchialis	WP_012835768	-QS--TAIDA---L-EE-ER-RG--DR		AERDGD-G--A--R-----
	Gordonia desulfuricans	WP_059038575	-QS--TAIDA---V-EE-EN-RG--DR		AERDGD-GK-A--R-----
	Gordonia hirsuta	WP_005942737	-QA---V-DS---V-EE-ER-RG--DR		AERDGD-G--A--R--T--
	Gordonia hydrophobica	WP_066163434	-QS--TAIDA---V-EE-ER-RG--DR		AERDGD-GK-A--R-----
	Gordonia iterans	WP_105941213	-QS---VINA---L-EE-EN-RG--DR		AERDGD-GK-A--R-----
	Gordonia jacobaea	WP_049700013	-QS--TAIDA---T-EE-ER-RG--DR		AERDGD-G--A--R-----
	Gordonia lacunae	WP_086534033	-QS--TAIDA---L-EE-ER-RG--DR		AERDGD-G--A--R--RV-
	Gordonia malaquae	WP_008381737	-QG--TAIDA---V-EE-ER-RG--DR		AERDGD-G--A--R--R--
	Gordonia namibiensis	WP_006867617	-QS--TAIDA---L-EE-DR-RG---R		AERDGD-G--A--R--R--
	Gordonia neofelifaecis	WP_009680779	-QS--TAIDA---V-EE-ER-RG--DR		AERDGD-GK-A--R-----
	Gordonia otitidis	WP_007238800	-QS--TAIDA---T-EE-ER-RG--DR		AERDGD-G--A--R-----
	Gordonia polyisoprenivorans	WP_006371830	-QS--TAIDA---V-EQ-EN-RG--DR		AERDGD-G--A--R--R--
	Gordonia rhizosphera	WP_006334460	-QS--TAIDA---L-EE-E--RG---R		-ERSGD-G--A--R-----
	Gordonia rubripertincta	WP_119033187	-QS--TAIDA---L-EE-DR-RG---R		AERDGD-G--A--R--R--
	Gordonia shandongensis	WP_026917549	-QS--TAIDA---V-EK-EQ-RG--DR		AERDGD-G--A--R--Q--
	Gordonia soli	WP_007618300	-QS--TAIDA---L-EE-EQ-RG--DR		AERDGD-G--A--R--R--

Other Bacteria (0/>200)

Gordonia sputi	WP_005209214	-QS--TAIDA---T-EK-ER-RG--DR	AERDGD-G--A--R-----
Gordonia terrae	WP_004021913	-QS--TAIDA---L-EE-ER-RG--DR	AERDGD-G--A--R-----
Granulicoccus phenolivorans	WP_081684212	-ER--G--NE-G-L-KQIDE-RG---R	AQREGD-G-----L-----
Hamadaea tsunoensis	WP_027342919	-KT--SHI-AISQA-EE-ET-RG--DR	AERDAD-G-----R-----
Isobaculum melis	WP_092650528	-ET--EEVSK----RAI-EQ-RR-L-D	AES-Y--E--A--RH-T--
Kibdelosporangium phytohabitan	WP_054296162	-QN--GSIEK--NL-EE-EQ-RG--DR	AERDGD-------R--R--
Kroppenstedtia sanguinis	WP_124246943	-QS--SAIDA---S-EK-EQ-RG--DR	AERDGD-G--A--R--Q--
Kutzneria albida	WP_025361787	-QN--GSIEK--EL-EQ-EQ-RG-S-R	AERDGD-G--A--R--R--
Kutzneria buriramensis	WP_116172009	-QN--GSIEK--EL-EQ-EQ-RG-S-R	AERD-D-G--A--R--R--
Labedaea rhizosphaerae	WP_133848092	-QN--GSIE---EL-TQ-EQ-RG-S-R	AERDGD-G--A--R--R--
Lawsonella clevelandensis	PZP88500	-QN--AIDA---L-ET-EQ-RGQ--R	AERDGD-GK-A--R--R--
Listeria grayi	WP_036104855	-E----EISQI-E-REQIDI-RHQL-E	AEN-YD-TK-A--RH----
Luteococcus japonicus	WP_094763226	-ES--A--NR-G-L-TEID--RG---R	FQREGD--K-A-I---R--
Maliponia aquimaris	WP_094020792	-QA-RDK-ASA--I-EE-DH-R--L-Q	AKREG-F---G-----R--
Mameliella alba	WP_088716758	-QA-RDK-ASA--L-EE-DH-R--L--	AKRDG-F-K-G-----R--
Microlunatus phosphovorus	WP_013861520	--A--Q--NQ-G-L-ARIDE-RI--DR	ALREG--V----IN--E--
Microlunatus soli	SDS85459	-QA--E--NQ-G-L-EKIDE-RG---R	AQREGD-G-----L--E--
Mycobacterium alsense	WP_068209996	-QN--AIDV--EL-EQ-EA-RG-S-R	AERDGD--K-A--R--R--
Mycobacterium basiliense	VDM87004	-QN---SIEI--EL-EQ-ET-RG-S-R	AERDGD--K-A--R--R--
Mycobacterium branderi	WP_083133858	-QN---AIDT--EL-EQ-ET-RG-S-R	AERDGD--K-A--R--R--
Mycobacterium canettii	WP_014000201	-QN---AIEI---L-EQ-EA-RG-S-R	AERDGD--K-A--R--R--
Mycobacterium conspicuum	WP_085236275	-QN---AIDT--EF-EQ-EI-RG-S-R	AERDGD--K-A--R--R--
Mycobacterium decipiens	WP_085327161	-QN---AIEI---F-EQ-ET-RG-S-R	AERDGD--K-A--R--R--
Mycobacterium gastri	WP_036412553	-QN---AIEI--EL-EQ-EA-RG-S-R	AERDGD--K-A--R--R--
Mycobacterium gordonae	WP_055580394	-QN---AIDI--EL-EQ-EE-RG-S-R	AERDGD--K-A--R--R--
Mycobacterium heckeshornense	WP_048889437	-QN---AIDT--EL-EQ-ET-RG-S-R	AERDGD--K-A--R--R--
Mycobacterium intracellulare	WP_064935235	-QN---AIDV--EL-EQ-EA-RG-S-R	AERDGD--K-A--R--R--
Mycobacterium kansasii	ORB85220	-QN---AIEI--EL-EQ-EA-RG-S-R	AERDGD--K-A--R--R--
Mycobacterium lacus	WP_085160091	-QN---AIEI--EL-EQ-ET-RG-S-R	AERDGD--K-A--R--R--
Mycobacterium leprae	WP_010908927	-QN---AIDV--EL-EQ-ET-RG-S-R	AERDGD--K-A--R--R--
Mycobacterium lepraemurium	WP_128619199	-QN---AIDV--EL-EQ-ET-RG-S-R	AERDGD--K-A--R--R--
Mycobacterium malmoense	WP_071508744	-QN---AIDA--EL-EQ-ET-RG-S-R	AERDGD--K-A--R--R--
Mycobacterium noviomagense	WP_083088618	-QN---AIDT--EL-EQ-ET-RG-S-R	AERDGE--K-A--R--R--
Mycobacterium palustre	WP_085078842	-QN---AIDV--EL-EQ-ET-RG-S-R	AERDGD--K-A--R--R--
Mycobacterium paraffinicum	WP_073874418	-QN---AIDV--EL-EQ-EA-RG-S-R	AERDGD--K-A--R--R--
Mycobacterium paraseoulense	WP_083173369	-QN--SAIDV---L-EQ-EA-RG-S-R	AERDGD--K-A--R--R--
Mycobacterium persicum	WP_075546386	-QN---AIEI--EL-EQ-EA-RG-S-R	AERDGD--K-A--R--R--
Mycobacterium saskatchewanense	WP_085257842	-QN---AIDV--EL-EQ-ET-RG-S-R	AERDGD--K-A--R--R--
Mycobacterium scrofulaceum	WP_067309674	-QN---AIDV--EL-EQ-EA-RG-S-R	AERDGD--K-A--R--R--
Mycobacterium shimoidei	WP_069395981	-HN---AIDT--EL-EQ-ET-RG-S-R	AERDGD--K-A--R--R--
Mycobacterium tuberculosis	AIH62713	-QN---AIEI---L-EQ-EA-RG-S-R	AERDGD--K-A--R--R--
Mycobacterium xenopi	WP_003922462	-QN---AIDT--EL-EQ-ER-RG-S-R	AERDGD--K-A--R--R--
Mycobacteroides immunogenum	WP_043076833	-QN---AIDA---L-EQ-EG-KG--DR	AERDGD-GK-A--R--R--
Mycolicibacterium acapulense	WP_066810782	-QN---AIDI--EL-EQ-ES-RG--DR	AERDG--E--A--R--R--
Mycolicibacterium agri	WP_097942096	-QN---AIDT--EL-EQ-ET-RG--DR	AERDG--E--A--R--R--
Mycolicibacterium aichiense	WP_115318389	-QN---AIDV---FTEQ-D--RG--DR	AERDGD--K-A--R--R--
Mycolicibacterium aromaticivor	WP_036342969	-QN---AIDI---FTEQ-D--RG--DR	AERDGD--K-A--R--R--
Mycolicibacterium doricum	WP_085189481	-QN---AIDT--EL-EQ-EA-RG--DR	AERDG--EK-A--R--R--
Mycolicibacterium elephantis	KKW61960	-QN---AIDV--EL-EQ-EE-RG--DR	AERDG--E--A--R--R--
Mycolicibacterium fallax	WP_085095953	-QN---AIDV---F-EQ-DQ-RG--DR	AERDGD--K-A--R--R--
Mycolicibacterium flavescens	WP_069412539	-QN---AIDI--EL-EQ-ES-RG--DR	AERDG--E--A--R--R--
Mycolicibacterium holsaticum	WP_069404621	-QN---AIDV--EL-EQ-ET-RG--DR	AERDG--E--A--R--R--
Mycolicibacterium moriokaense	WP_110318353	-QN--SAID---EL-EQ-DT-RG--DR	AERDGD--K----R--R--
Mycolicibacterium mucogenicum	WP_133427979	-QN---AIDT--EL-EQ-ET-RG--DR	AERDGD--K-A--R--R--
Mycolicibacterium novocastrens	WP_067392085	-QN---AIDI--EL-EQ-ES-RG--DR	AERDG--E--A--R--R--
Mycolicibacterium rhodesiae	WP_083117981	-QN---AIDV---FTEQ-D--RG--DR	AERDGD--K-A--R--R--
Mycolicibacterium rutilum	WP_083406006	-QN---AIDI--EL-EQ-ET-RG--DR	AERDG--E--A--R--R--
Mycolicibacterium sphagni	WP_094480701	-QN---AIDI---F-EQ-ET-RG--DR	AERDGD--K-A--R--R--
Mycolicibacterium tusciae	WP_006246075	-QN--GAIDI---L-EQ-DR-RG--DR	AERDGD--K----R--R--
Nakamurella panacisegetis	WP_090474888	--Q--S--NR-GEL-KS-DE-RVQ--R	AQREGD--K----L--Q--
Nocardia farcinica	WP_068970187	-QS--TAIDA---L-EE-ER-RG--DR	AERDGD-G--A--R--RV-
Nocardia fluminea	WP_101465321	-QN---AIDS--GF-EQ-EA-RG-S-R	AERDGD-GK-A--R-----
Pelagibaca abyssi	WP_076698229	-QA-RDK-ASA--I-EE-DR-R--L-I	AKREG-Y-K-G-----R--
Phialophora attae	XP_018003923	--K-RGEIE-IKKT-EEIE-ARQDL-L	ARR-N-F--------SV--
Prauserella marina	WP_091805964	-QN--GSIEK--EL-EQ-EQ-RG---R	AERDAD--H-A--R--R--
Prauserella rugosa	WP_030533665	-QN--GSIER--EL-EQ-EQ-RG--DR	AERDAD-G--A--R--R--
Prauserella shujinwangii	WP_106179034	-QN--GSIEK--EL-EQ-EQ-RG-S-R	AERDGD-G--A--R--R--
Propionibacteriaceae bacterium	WP_094449995	-EA--S--NR-GEL-EQIDQ-RS--DR	AQREGD--K----T-----
Propionicicella superfundia	WP_028709254	-EA--S--NR-G-L-EQID--RG---R	AQREGD-GK---IL--R--
Pseudonocardia asaccharolytica	WP_028930808	-QN--TSIES--EL-EQ-EA-RG-S-R	AERDGD-G--A--R-----
Rhodobacter johrii	WP_112328905	-QA-RDK-EAA--L-EQ-DRAR--L-Q	-KREG-----G--S--V--
Rhodobacter sphaeroides	WP_015921675	-QA-RDK-EAA--L-EQ-DRAR--L-Q	-KREG-----G--S--V--
Rhodobacteraceae bacterium	PCJ08669	-QA-RDK-AGA--I-E--E-AR--L-I	AKREG---K-G--S--V--
Rhodococcus corynebacterioides	WP_068149194	-QN---AIDS---L-EQ-EA-RG-S-R	AERDGD-G--A--R--R--

Other Bacteria (0/>200)

Saccharomonospora azurea	WP_005442270	-QN--GSIER--EL-EQ-EQ-RG---R	AERDAD-G--A--R--R--
Saccharomonospora cyanea	WP_005460421	-QN--GSIER--EL-EQ-EQ-RG--DR	AERDAD-G--A--R--R--
Saccharomonospora glauca	WP_005466956	-QN--GSIER--EL-EQ-EQ-RG--DR	AERDAD-G--A--R--R--
Saccharomonospora marina	WP_009157127	-QN--GSIEK--EL-EQ-EQ-RG--DR	AERDAD-G--A--R--R--
Saccharomonospora paurometabol	WP_007023335	-QN--GSIER--EL-EQ-EQ-RG--DR	AERD-D-G--A--R--R--
Saccharomonospora saliphila	WP_019818856	-QN--GSIER--EL-EQ-EQ-RG---R	AERDAD-G--A--R--R--
Saccharomonospora viridis	WP_015788106	-QN--SAIER--GL-EQ-EQ-RG---R	AERDAD-G--A--R--R--
Saccharothrix australiensis	WP_121000641	-QN--GSIEK---L-ER-EQ-RG-S-R	AERDGD-G--A--R--R--
Saccharothrix carnea	WP_106616083	-QN--GSIEK---L-EQ-EQ-RG-S-R	AERDGD-G--A--R--R--
Saccharothrix espanaensis	WP_015105656	-QN--GSIEK---L-EQ-EG-RG-S-R	AERDGD-G--A--R--R--
Saccharothrix syringae	WP_033428306	-QN--TAIEK---L-EQ-EQ-RG-S-R	AERDGD-G--A--R--R--
Saccharothrix texasensis	WP_123744962	-QN--GSIEK---L-EQ-EQ-RG-S-R	AERDGD-G--A--R--R--
Saccharothrix variisporea	WP_121224208	-QN--TSIEK---L-EQ-EQ-RG-S-R	AERDGD-G--A--R--R--
Salipiger bermudensis	WP_007794953	-QA-RDK-ASA--V-EQ-DRMR-DL-I	AKREG-F-K-G-----R--
Salipiger thiooxidans	WP_089955885	-QA-RDK-ASA--L-EQ-DR-R--L-I	AKREG-F-K-G--------
Segniliparus rotundus	WP_013138523	-QH--HAIDA---V-EQ-EA-RG--DR	AERDGD-G--A--R--R--
Skermania piniformis	WP_066469445	-QN---AIDA---V-EQ-ET-RG--DR	AERDGD-GK-A--R--R--
Sporosarcina globispora	WP_053436812	-QL--E-I-K-QE-REQ-E-MRRDL-E	AEN-YD-NK-A--RH-R--
Streptoalloteichus hindustanus	WP_073479672	-QN--GSIER--EL-EQ-EQ-RG-S-R	AERDGD-G--A--R--R--
Thermocrispum agreste	PZM89806	-QN--RSIDRI--L-EQ-EQ-RG--DR	AERDAD-G--A--R-----
Thermocrispum municipale	WP_028851742	-QN--ASIDRI--L-EQ-EQ-RG--DR	AERDAD-G--A--R-----
Umezawaea tangerina	WP_106196034	-QN--GSIDK---L-EQ-EQ-RG-S-R	AERDGD-G--A--R--R--
Vibrio vulnificus	WP_133348719	-QM--SA--K-Q--REE-E--RY-L-Q	AENDYD-N--A--RH-R--
Williamsia limnetica	WP_110467928	-QS---AINS---L-ER-EH-RG-S-R	AERDGD-G--A--R--Q--
Williamsia sp. 1135	WP_084891771	-QS---AINS---L-ER-EH-RG-S-R	AERDGD-G--A--R--Q--
Williamsia sterculiae	SIR64968	-QG---AIDS---L-TE-EE-RI--DR	AERDGD-G--A--R--R--
Yangia pacifica	WP_108964479	-QA-RDK-ASA--I-EQ-DR-R-DL-I	AKREG-F-K-G--------

Supplemental Figure 65

A partial sequence alignment of the AAA family ATPase protein containing a one amino acid insertion (boxed) that is exclusively shared by all members belonging to the Solibacillus clade and absent in all other bacteria. The peripherally branching *Lysinibacillus odysseyi* does not share this CSI.

```
                                                                          435                                 479
Sporosarcina clade   Sporosarcina ureae                     WP_083031738  LFEYDEEDGRYYAAHHPFTMPA [  ] DVEELKTSPETVKAQAYDLVLNG
(9/9)                Sporosarcina newyorkensis 2681         EGQ22734      ---------------------- [  ] ---Q---H--S-----------
                     Sporosarcina koreensis                 WP_082023378  ----------F----------- [  ] --K--EE-------L--------
                     Sporosarcina pasteurii                 WP_115361426  ---------------------- [  ] N--Q-ESNRDE-----------
                     Sporosarcina psychrophila              WP_067208224  ----N--AR--Q---------- [  ] -----VA---------------
                     Lysinibacillus sphaericus LMG 22257    WP_075527774  -------A---F---------- [  ] NI-Q-ESNRDE-----------
                     Lysinibacillus sphaericus NCTC 11025   WP_039043074  ---------------------- [  ] -----VS-------L--------
                     Bacillus sp. OxB-1                     WP_041075310  -------------------R-S [  ] --D--VN---------------
                     Filibacter sp. TB-66                   WP_124071582  --------E-----------S  [  ] -----VS--Q------------
Other Bacteria       Lysinibacillus sphaericus FSL M8-0337  WP_069513343  ----S-------------R-F DE -I-LMD-N-AA-R-----I----
(0/>100)             Lysinibacillus sphaericus NCTC 7582    WP_112118166  ----S-------------R-F DE -IALMD-D-AA-R-----I----
                     Lysinibacillus sphaericus C3-41        WP_012295418  ----S-------------R-F DE -IPLMD-D-SA-R-----I----
                     Aliicoccus persicus                    WP_091473619  -------E---F-------S-Q DD -L-K-ESA-SE---K---I----
                     Alkalibacterium pelagium               WP_091478646  -L-----E---N---------V ES ---L------KAM-D---I----
                     Alkalibacterium thalassium             WP_091264289  -L-----E---N---------V ES ---L------KAM-D---I----
                     Anoxybacillus vitaminiphilus           WP_111645852  -L-----E-------------V RE -LPL-E-E-DK-R----------
                     Bacillus cereus                        WP_000840890  ------DAE--F---------F RE ---L-E-A--KAR----------
                     Bacillus cohnii                        WP_066420797  -L-----A---F---------V RE -L-L-N-D-GK-R----------
                     Bacillus deserti                       WP_101640552  -L-----S---F---------F RE -LDK-V-D-GS-----------
                     Bacillus nealsonii                     WP_016201662  ------AE--F----------Q RA -L-L-DSN-KA-------I----
                     Bacillus simplex                       WP_034308772  -L-----E-------------F RE -MDK-ESD-AS-R----------
                     Bacillus subtilis                      WP_019715218  -L-H-Q-E--F----------V RE -L-LIE-A--DM----------
                     Bhargavaea cecembensis                 WP_008299830  ---------------------V RE -I-K-G-D-QK-R-M---M----
                     Carnobacterium divergens               WP_074402823  -L-----N---S---------K ES -I-K-T---GE-Y-----I----
                     Domibacillus antri                     WP_075397743  -L-----E-------------- RE -LDK-ESD-GA-R-----I----
                     Edaphobacillus lindanitolerans         WP_076757800  ----------F----------V RE -LPK-E-E-QS-R-M--------
                     Geobacillus jurassicus                 WP_066228677  -L-----E-------------V RD -IPL-E-N-SA-R----------
                     Gracilibacillus orientalis             WP_091483186  -L-----E---H---------F EA -FDK-D-N-GE-R----------
                     Halobacillus aidingensis               WP_089652628  ------DE---H---------- GG RMD---KN-KH-R-E---I----
                     Jeotgalibacillus soli                  WP_041090398  -L--A----------------V RE -L-L-D-H--K-R-----I----
                     Lysinibacillus contaminans             WP_053582709  ----S-------------R-F DE -I-LMD-N-SA-R-----I----
                     Lysinibacillus fluoroglycofeni         WP_107942875  -------E----------R-F DE -IALME-N-QA-R-----I----
                     Lysinibacillus odysseyi                WP_036156636  -------E----------R-F DE -I-LMD-N-AA-R-----I----
                     Mycobacteroides abscessus              SLL32537      -------E--F----------Q RA -L-L-D-N-KA-------I----
                     Oceanobacillus senegalensis            WP_085991814  -L-----L---F-------S-V EE -MDK-ES-----R-N--------
                     Ornithinibacillus scapharcae           WP_010098972  -L-----L---F---------F EE -F-K-R-D-SS-R-N--------
                     Paenisporosarcina antarctica           WP_134209502  ------Q--------------F VE -L-LMD-N--K-R----------
                     Parageobacillus thermantarctic         WP_090947495  -L-------------------V RE -LPQ-E-N--K-R----------
                     Paraliobacillus quinghaiensis          WP_117154540  -L----NA---T---------S DE -LDK-D-H--Q------------
                     Piscibacillus halophilus               WP_091772674  ------DE---H---------Q VQ ---K-EQD-AN-------V----
                     Planococcus citreus                    WP_121300086  -L---DK--------------F EE -LDK-S-E-QN-R----------
                     Planomicrobium soli                    WP_106533791  -----DKA---F---------F EE -LDK-E-D-AS-R----------
                     Pontibacillus chungwhensis             WP_036785585  -------L---F---------E RE -I-K-E-E-QE-R----------
                     Salimicrobium halophilum               WP_093190645  ----N-D--------------- AE -EQH-LDR----Q-E---I----
                     Salinicoccus luteus                    WP_031548892  -------A---F-------S-K AE ---K-E-A--E-I-N---I----
                     Sediminibacillus albus                 WP_093216773  -L-----L---F---------V QE -M-K-E-N-GE---E--------
                     Sporosarcina globispora                WP_053434289  -L-----E-------------- RE -LDL-DKN-AQ-R----------
                     Streptococcus pneumoniae               CJG04213      -L----DAD--F---------F RE ---L-E-A--KAR----------
                     Terribacillus goriensis                WP_038561582  -------E-------------- AA -IDK-E-D-SS---E---I----
                     Thermolongibacillus altinsuens         WP_132947668  -L-----E-------------V RE -LPK-E-E--K-R----------
                     Vagococcus carniphilus                 WP_126793767  -L-----A---A---------K AS ---L---A--K-Y-E---V----
                     Vibrio vulnificus                      WP_133349422  -L-----E-------------F RE -MDK-ESD-AS-R----------
                     Virgibacillus soli                     WP_057986813  -L-----A---S---------V RE -IDK-E-N-AE-----------
```

Supplemental Figure 66

A partial sequence alignment of the aspartate-tRNA ligase protein containing a two amino acid deletion (boxed) that is exclusively shared by all members belonging to the Sporosarcina clade and absent in all other bacteria.

Group	Organism	Accession	183		222
**Sporosarcina clade (9/9)**	Sporosarcina ureae	WP_029055238	FNQALMELGATICTPN		PHCLLCPVRDFCIAFEEGKQQQLP
	Sporosarcina newyorkensis	WP_009498457	----------V-----		-R----------------------
	Sporosarcina psychrophila	WP_067205806	---G-----------K		-K----------L--Q--R-EE--
	Sporosarcina koreensis	WP_060207281	---G-----------R		-K----------T--H--R-EE--
	Sporosarcina pasteurii	WP_115362990	---G-----------K		-K-------EY-Q-YD--R-EE--
	Lysinibacillus sphaericus LMG 22257	WP_075526960	---G-----------K		-K-------EY-Q--Y--R-EE--
	Lysinibacillus sphaericus NCTC 11025	WP_125101230	----I----------K		-R----------S-------E
	Bacillus sp. OxB-1	WP_084212359	-------------I-R		-R----------A--Q--R-EE--
	Filibacter sp. TB-66	WP_124069304	---G-----------K		-K-------E--L--N--R-EEF-
**Other Bacteria (0/>200)**	Aeribacillus pallidus	WP_063386263	----------MV---T	S	-S------QEH-L--S--L-EK--
	Alteribacillus iranensis	WP_091663833	----------LV---K	S	-G------Q-Y-L-RA--V-TE--
	Anoxybacillus flavithermus	OAO76371	----------L----K	N	-S------QRH-R--A--MEK---
	Anoxybacillus suryakundensis	CUA81190	----------L----K	N	-S------QRH-R--A--VEK---
	Bacillus acanthi	WP_108670539	----------L----G	T	-S-------EH-T--K--V-SE--
	Bacillus acidicola	WP_066266292	----------L----T	S	-S-------EH-H-----TTGE--
	Bacillus alkalitelluris	WP_078549852	----------L----T	S	-S--------H-R--D--V-SE--
	Bacillus aquimaris	OIU68868	----------L----T	S	-S-------EH-R--D--V-EE--
	Bacillus aryabhattai	WP_033580775	---GM-----IV---T	S	-S-------EH-R-----V-NE--
	Bacillus aryabhattai B8W22	SDE21256	---GM-----IV---T	S	-S-------EH-R-----V-NE--
	Bacillus badius	KZO01366	----------L----T	S	-S-------EH-C--H--TMAE--
	Bacillus butanolivorans	WP_053344480	----------L----G	K	-A------QSH-L---A-V-SE--
	Bacillus camelliae	WP_101354857	----------L----T	S	-S-------EH-H-----T-TE--
	Bacillus canaveralius	WP_125928508	----------IV---T	S	-S-------NH-Q--H--V-TD--
	Bacillus cavernae	WP_126867155	----------L----G	V	-A-------EH-F--Q--V-AE--
	Bacillus cecembensis	WP_057984583	---G------L----T	S	-K--------Y-TV-H--DPTS--
	Bacillus cohnii	WP_066421424	---G------IV---T	S	-S-------EH-R--H--E-NE--
	Bacillus dakarensis	WP_077211014	----------L----T	S	-A--------Y-H--H--S-KE--
	Bacillus deserti	WP_101639702	----------M----G	T	-A-------EH-E--A--V-SR--
	Bacillus dielmoensis	WP_042454979	----------LV---T	S	-A--------H-Q-----I-DE--
	Bacillus drentensis	WP_066251584	----------L----T	S	-A--------H-Q--D--V-NE--
	Bacillus enclensis	WP_058296986	----------L----T	S	-S-------EH-R--D--V-EE--
	Bacillus endophyticus	WP_061802466	---G------L----K	S	-A------QEH-R-----T-KE--
	Bacillus farraginis	WP_058005718	----------L----T	S	-A-------EH-L-----V-NE--
	Bacillus filamentosus	WP_019390610	---G------L----K	S	-A------QEH-R-----T-NE--
	Bacillus firmus	WP_035333106	----------L----T	S	-S-------EH-T--Y--T--E--
	Bacillus flexus	WP_061786170	---G------IV---T	S	-S-------EH-R---Q-V--E--
	Bacillus foraminis	TCN18820	----M-----L----T	S	-S--------H-Q--N--V-DE--
	Bacillus fordii	WP_033369840	----------L----S	S	-S-------EH-Q--AK-V-E---
	Bacillus fortis	WP_120072618	----------L----T	S	-A-------EH-Q--SK-V-D---
	Bacillus freudenreichii	VEF48868	----------L----T	S	-S-------EH-Q---N-V-EE--
	Bacillus fumarioli	WP_066369456	----------L----A	S	-S--------Y-Q-----V-N---
	Bacillus gottheilii	WP_080848305	----------L----T	S	-S--------H-S--HG-T-HE--
	Bacillus halodurans	WP_010897103	---G------LV---T	S	-G--------H-R--AA-V-E---
	Bacillus horikoshii	WP_064099253	---G------MV---T	S	-S-------EY-R--A--V-RE--
	Bacillus horneckiae	WP_066391772	----------L----T	S	-S------QEH-D--R--T-NE--
	Bacillus humi	WP_057999663	----------L--V-G	N	-A-------EH-K--A--V-KE--
	Bacillus indicus	KEZ47511	----------IV---T	S	-S--I----EH-Q-----V-TE--
	Bacillus indicus LMG 22858	KEZ48009	----------IV---T	S	-S--I----EH-Q-----V-TE--
	Bacillus jeotgali	WP_079505925	----------L----T	S	-S--------H-E--H--V-TE--
	Bacillus koreensis	WP_053403039	---G------L----T	S	-S-------EH-R--D--V-DE--
	Bacillus korlensis	WP_066056392	----------L----T	S	-S--------H-H-----T-NE--
	Bacillus kribbensis	WP_035322323	----------L----G	T	-S-------EH-S--A--M-TE--
	Bacillus lentus	WP_066145476	----------L----T	S	-A--------H-L---D-V-SE--
	Bacillus litoralis	WP_066336000	----------L----T	S	-S-------EH-T-----V-AE-
	Bacillus marisflavi	WP_048007766	----------L----T	S	-S-------EH-H-----V-SE--
	Bacillus massilioanorexius	WP_019242934	----------L----G	T	--------NTH-V--SK-E-TE--
	Bacillus massiliogabonensis	WP_102273184	----------L----T	S	-S-------EH-H--H--TEKE--
	Bacillus massiliogorillae	WP_081707781	----------L----G	T	-A-M-----EH-V--A--V-AE--
	Bacillus massilionigeriensis	WP_075980251	------D---Q----T	S	-S-------EH--S-D--TSNE--
	Bacillus massiliosenegalensis	WP_019154987	----------L----T	S	-S-------EH-H--H--VEKE--
	Bacillus megaterium	WP_013081544	---GM-----IV---T	S	-S-------EH-R-----V-NE--
	Bacillus methanolicus	WP_003347782	----------I----T	S	-S-------EH-Q--H--V-EN--
	Bacillus muralis	WP_057914511	----------L----G	K	-A------QSH-L---S-V-SE--
	Bacillus ndiopicus	WP_042478964	---G------L----T	S	-K--------Y-------DAAE--
	Bacillus nealsonii	WP_101178868	----------L----T	S	-S--------H-Q--H--T-TD--
	Bacillus niacini	WP_045520039	----------L----T	S	-S-------EH-K--H--VESE--
	Bacillus niameyensis	WP_062105451	----------L----T	S	-A-------EH-L--Q--VA-E--
	Bacillus notoginsengisoli	WP_118924038	----------L----T	S	-S-------EH----H--V-A---
	Bacillus novalis	WP_066083817	----------L----T	S	-S--------H-H--S--V-NE--
	Bacillus oceanisediminis	WP_019383505	----------L----T	S	-S-------EH-T--Y--T-HE--
	Bacillus okuhidensis	WP_053432594	---G------LV---T	S	-G--------H-R--AA-V-E---
	Bacillus onubensis	WP_099352810	----------L--V-G	N	-A-------EH-R--A--V-KE--
	Bacillus patagoniensis	WP_078395265	---G------LV---T	S	-G-------EQ-M-YDQ---HE--
	Bacillus persicus	SEN87437	----------L----T	S	-S-------EH-Q--NT-M-NE--

Other Bacteria (0/>200)

Bacillus polygoni	WP_088035955	----------M----R S -A------QQH-L-R---V-E---
Bacillus praedii	WP_095396674	----------L----T S -S-------EH-H--H--TEKE--
Bacillus pseudomycoides	PEA53295	---G------L--V-K N -A-------EH-R--A--V-KE--
Bacillus psychrosaccharolyticu	WP_081704813	----------L--K-S N -A------QQH-Q-----V-TE--
Bacillus rubiinfantis	WP_042356737	----------LV---T S -A--------H-Q--A--V-NE--
Bacillus salsus	SDP91799	---G------L----T S -S-------EH-K-----V-AE--
Bacillus selenatarsenatis	WP_041967615	----------L----T S -S-------EH-E--H--V-NE--
Bacillus selenatarsenatis SF-1	GAM15969	----------L----T S -S-------EH-E--H--V-NE--
Bacillus shackletonii	WP_055741922	----------L----T S -S-------EH-C-----T-GE--
Bacillus simplex	WP_061461731	----------L----G K -A------QSH-L---A-V-SE--
Bacillus sinesaloumensis	WP_077620862	----------L--V-Q N -A------QEH-K--A--V-KE--
Bacillus smithii	AKP46149	----------L----T S -S-------EH-H-----V-EE--
Bacillus soli	WP_066070002	----------L----T S -S--------H-H--D--V-NE--
Bacillus sporothermodurans	KYD11382	----------L----T S -S-------EH-Q--H--V-D---
Bacillus subterraneus	WP_044396674	----------L----T S -S--------H-Q--H--V-KE--
Bacillus testis	WP_050616672	----------L----T S -S------MEH-Q--A----LE--
Bacillus timonensis	WP_026021831	----------L--V-G N -A-------EH-R--A--V-KE--
Bacillus trypoxylicola	WP_061949908	---G------LV---T S -G-------EH-R-Y-R---S---
Bacillus tuaregi	WP_071396105	----------IV---T S -S-------EH-V--H--V-GE--
Bacillus vietnamensis	WP_060673424	----------L----T S -S-------EH-Q-----V-TE--
Bacillus vireti	WP_024030818	----------L----T S -S--------H-H--F--V-NE--
Bacillus wakoensis	WP_034746014	---G------L----T S -G-------E--R-YDT---NE--
Bacillus wakoensis JCM 9140	GAE26385	---G------L----T S -G-------E--R-YDT---NE--
Bhargavaea beijingensis	WP_092095768	---G------VV---R R -K-------EL-G-YH--REEE--
Bhargavaea cecembensis	WP_008300573	---GM-----V----R R -K----------G-YH--REEE--
Bhargavaea ginsengi	WP_092054761	---G------V----R R -K-------E--G-YH--REEE--
Caryophanon tenue	WP_066544022	---G------L----T S -K--------Y-------EPEK-
Chryseomicrobium excrementi	WP_100354524	---G------L----Q K -K-------EQ-R------TES--
Domibacillus aminovorans	WP_063964800	----------I----T S -S------QNH-Q-----V-RE--
Domibacillus antri	WP_075399605	----------I----T S -S------QNH-Q-----V-RE--
Domibacillus enclensis	SIQ97578	-----------V---T S -A------QNH-E---Q-V-RE--
Domibacillus epiphyticus	OMP68172	----------I----T S -S------QNH-M-----V-RE--
Domibacillus indicus	WP_046174810	----------M----T S -A------QNH-Q-----V-RE--
Domibacillus iocasae	OES45069	---------------T S -A------QNH-Q-----I-RE--
Domibacillus mangrovi	WP_073712068	----------I----T S -S------QNH-Q-----V-RE--
Domibacillus robiginosus	WP_050181969	----------I----T S -S------QNH-Q-----V-RE--
Domibacillus tundrae	WP_046181115	-----------V---T S -A------QNH-Q-----V-RE--
Edaphobacillus lindanitolerans	WP_084186715	---G-------V---T R -K-------EW-G-YH--REES--
Falsibacillus pallidus	WP_114747216	----------L----T S -S-------EH----H---ERE--
Fictibacillus aquaticus	WP_094253461	----------I----R S -A--I---QQE-R--A--R--E--
Fictibacillus arsenicus	ANX10881	----M-----L----K S -A-----M-EL-KG--Q---HE--
Fictibacillus enclensis	WP_061974282	----------L----K S -A--T---HEH-R---Q-R-RE--
Fictibacillus macauensis	WP_007202736	----------L----Q S -A------QAH-R---Q-R-KE--
Fictibacillus phosphorivorans	WP_066242715	---G------I----K S -A-----M-EL-RG--S-R-RE--
Fictibacillus solisalsi	WP_090237496	----------L----K S -A--I---H-H-R---Q-R-RE--
Gracilibacillus lacisalsi	WP_018932583	---G------LV---K N -S------QEH----R--VE-S--
Jeotgalibacillus malaysiensis	WP_039808116	----------IV---T S -S-F----QSH-A--A--S--E--
Jeotgalibacillus salarius	WP_134382424	----------IV---T S -S-F----QAH-A-----S-HE--
Jeotgalibacillus soli	WP_041088052	----------L----T S -S------NEH-L--A--V-SE--
Kurthia gibsonii	WP_121177545	---G------LV---T S -K--------Y-G--A--RTAE--
Kurthia huakuii	WP_029500825	---G------MV---T S -S--------Y-T--HD-EPEK--
Kurthia massiliensis	WP_010290847	---G-------V---T S -S--------Y-T--H--DPEK--
Kurthia senegalensis	WP_010308402	---G------M----T S -S--------Y-T--H--EPEK--
Kurthia sibirica	WP_109306150	---G------LV---T S -K--------Y-G--A--QTSN--
Kurthia zopfii	WP_109349573	---G------LV---T S -K--------Y-G--A--RTTE--
Lysinibacillus acetophenoni	WP_097150994	---G--D---Q----T S -K-------EY-M--Q--HPEE--
Lysinibacillus boronitolerans	WP_016995054	---G------L----T S -K-------EY-T--N--EPEK--
Lysinibacillus chungkukjangi	WP_107937107	------D---M----T S -K--------Y----S--DADK--
Lysinibacillus endophyticus	WP_121213260	---G--D---M----T S -K--------Y----H--DMDK--
Lysinibacillus fluoroglycofeni	WP_107943666	---G------L----T S -K-------EY-----A-DAAE--
Lysinibacillus fusiformis	WP_004230484	---G------L----T S -K-------EY-T--N--EPEK--
Lysinibacillus halotolerans	WP_122971948	----I-D---M----T S -K--------Y----Q--DTE---
Lysinibacillus macroides	WP_053995532	---G------L----T S -K-------EY-L--N--EPEN--
Lysinibacillus manganicus	WP_036190336	---G--D---Q----T S -K-------EY-------HPEE--
Lysinibacillus massiliensis	WP_036177983	------D---Q----T S -K-------EY-L--Q---PEE--
Lysinibacillus meyeri	WP_107840133	---G------L----T S -K--------Y-----A-DAAE--
Lysinibacillus odysseyi	WP_036150601	---G------L----T S -K-------EY-R---A-DAA---
Lysinibacillus parviboronicapi	WP_107950099	---G------L----T S -K--------Y-T--H--FPEK--
Lysinibacillus sinduriensis	WP_036197690	------D---M----T S -K--------Y----N--IAD---
Lysinibacillus sp. 2017	WP_108713941	---G------V----T S -K--------Y-T--Q--DPST--
Lysinibacillus sp. B2A1	AVK86186	---G------L----T S -K-------EY----N--EPEK--
Lysinibacillus sp. FJAT-14745	WP_053483072	---G------L----T S -K-------EY----N--EPEK--
Lysinibacillus sp. SYSU K30002	WP_126659404	------D---M----T S -K-------EY-T--H--DAD---
Lysinibacillus sp. YLB-03	WP_118877038	------D---M----T S -K--------Y-T--Q--DAD---

Group	Species	Accession	Sequence
Other Bacteria (0/>200)	Lysinibacillus sp. YR326	WP_134025979	---G------L----T S -K-------EY-T--N--EPEK--
	Lysinibacillus sp. ZYM-1	WP_054612694	---G------L----T S -K-------EY-T--N--EPEK--
	Lysinibacillus telephonicus	WP_126294004	---G--D---M----T S -K--------Y----Q--DAD---
	Lysinibacillus xylanilyticus	WP_049668857	---G------L----T S -K-------EY-T--N--EPEK--
	Lysinibacillus xyleni	WP_097072667	---G--D---M----T S -K--------Y----H--DADK--
	Mycobacteroides abscessus subs	SHT32045	----------L----T S -S-------EH-H-----T-NE--
	Oceanobacillus halophilus	WP_121205066	----I-D--------T S -A------QEY-R--A--V-ED--
	Planococcus antarcticus	WP_081487866	---G------L----T S -K-------EH-A--H----NV--
	Planococcus antarcticus DSM 14	EIM05761	---G------L----T S -K-------EH-A--H----NV--
	Planococcus citreus	WP_121301213	---G------L----T S -K--------H-E--HA--ETE--
	Planococcus donghaensis	ANU23963	---G------L----T S -K-------EH-A--H----HE--
	Planococcus donghaensis MPA1U2	EGA89374	---G------L----T S -K-------EH-A--H----NE--
	Planococcus faecalis	AQU78675	---G------L----T S -K-------EH-A--H----NE--
	Planococcus halocryophilus	ANU12422	---G------L----T S -K-------EH-A--H----NE--
	Planococcus halocryophilus Or1	EMF47201	---G------L----T S -K-------EH-A--H----NE--
	Planococcus halotolerans	WP_112224967	---G------L----T S -K-------EH-A--H--R-NE--
	Planococcus kocurii	ALS79356	---G------L----T S -K-------EH-A--H----NE--
	Planococcus maitriensis	WP_112233846	---G------L----T S -K--------H-E--HA--ETE--
	Planococcus maritimus	KYG71130	---G------L----T S -K--------H-S--H---ETE--
	Planococcus massiliensis	WP_110925636	---G------L----T S -K-------EH-A--H----HE--
	Planococcus rifietoensis	WP_058381349	---G------L----T S -K--------H-E--HA--ETE--
	Planococcus salinarum	TAA71746	---G------L----T S -K-------EH-A--H--R-SE--
	Planococcus salinus	WP_123166634	---G------L----T S -K-------EH-H--H--R-HE--
	Planococcus versutus	ANU26027	---G------L----T S -K-------EH-S--Y----NE--
	Planomicrobium flavidum	WP_088008181	---G------I----S S -K--------H-AG-H--V-NE--
	Planomicrobium glaciei	KOF09700	---G------L----T S -K-------EH-A--H-----D--
	Planomicrobium okeanokoites	WP_084242273	---G------L----T S -K-------EH-S--H--R-HE--
	Planomicrobium soli	WP_106534782	---G------L----T S -K-------EH-A--DK---HD--
	Pontibacillus marinus	WP_027446736	---G------IV---K N -S-M----QEH-R--H---EKE--
	Psychrobacillus insolitus	WP_111440227	---G------V----T K -K-------EY-T--F--M-EG--
	Quasibacillus thermotolerans	WP_039238732	----------L----T S -A--------H-H--H--TADE--
	Rhodococcus qingshengii	WP_133370550	----------L----T S -S--------H-QG-H--VESE--
	Rummeliibacillus pycnus	WP_102693964	---G------LV---T S -K-------E--T--N--NPEE--
	Rummeliibacillus stabekisii	WP_066789723	---G------L----T S -K-------E--T--Q--RPEE--
	Shimazuella kribbensis	WP_051272074	---------------K S -Q--F---K-V-L-Y-K-LEKV--
	Solibacillus isronensis	WP_079523621	---G------L----T S -K--------Y-T--H--DPSS--
	Solibacillus kalamii	WP_087617404	---G------L----T S -K--------Y-T--H--DPSS--
	Solibacillus silvestris	WP_014822713	---G------L----T S -K--------Y-T--H--DPSS--
	Sporosarcina globispora	WP_053437062	----------L----T S -S-------EH-T--H--T-NE--
	Staphylococcus schweitzeri	WP_047450588	----M-----L----K N -L--------N-E--DK-TFEK--
	Streptococcus pneumoniae	CJG03073	---GM-----IV---T S -S-------EH-R-----V-NE--
	Tepidibacillus fermentans	TCS82580	-----------V---K S -L------M---EGRK--N-D---
	Tetzosporium hominis	WP_094944773	---G------L----Q K -K-------EE-R------TDS--
	Thermoactinomyces vulgaris	RMA97367	------D--------V S -G-------SV-E-Y-K-MEE---
	Ureibacillus thermophilus	QBK26964	------D---M----T S -K-------EY-LG-M--DAE---
	Ureibacillus thermosphaericus	WP_016837865	------D---M----T S -K-------EY-VS-M--DAD---
	Vibrio vulnificus	WP_133352037	----------L----G K -A------QSH-L---A-V-SE--
	Virgibacillus indicus	WP_094886793	----I-D--SS----K S -A------QEY----A--MEES--
	Viridibacillus arenosi	WP_038178547	---G------M----T S -K-------E--T--N--NPEK--
	Viridibacillus arvi	WP_053418813	---G------M----T S -K-------E--T--N--NPEK--

Supplemental Figure 67

A partial sequence alignment of the A/G-specific adenine glycosylase protein containing a one amino acid deletion (boxed) that is exclusively shared by all members belonging to the Sporosarcina clade and absent in all other bacteria.

Group	Organism	Accession	138		176
**Sporosarcina clade (6/6)**	Sporosarcina ureae	WP_029053848	GDLGPVYGKQWRAWDSANGV		IDQLAQVIDSIKHNPDSRR
	Sporosarcina newyorkensis	WP_078816654	---------------TDH--		--------E---T------
	Sporosarcina koreensis	WP_060210454	-----I------S-NAGA--		---IE-L--G--R------
	Sporosarcina psychrophila	WP_067209777	A-----------S-SNGEQS		---I-NL-EG--N------
	Sporosarcina pasteurii	WP_115361336	A-----------S-ATE--T		---IKN------N--N---
	Lysinibacillus sphaericus LMG 22257	WP_075527822	A-----------S-ATE--T		---IKN------N------
**Other Bacteria (0/>200)**	Abyssibacter profundi	WP_109720948	--------------EGP---	V	V----ELVHNL-T------
	Aeromonas caviae	WP_048209078	--------A---S-PA-D-S	V	---IQKAV-D-Q-------
	Aeromonas dhakensis	WP_124249368	--------A---S-P--D-S	V	---IQKAV-D---------
	Aeromonas hydrophila	WP_017409024	--------A---S-PA-D-S	V	---IQKAV-D---------
	Aeromonas jandaei	WP_104016033	--------A---S-PT-D-G	V	V--IQKAV-D---------
	Aestuariibacter salexigens	WP_026376377	--------H---S-TG-D-Q	V	V--ISE-VEQ---------
	Alteromonas addita	WP_062085878	-E----------S--NGD-T	T	---I-D---Q--T------
	Alteromonas facilis	WP_100643750	-E------H---S-SGE--E	S	---I-E--EQ---------
	Alteromonas flava	WP_100656231	-E----------S-TG---E	V	---I-D--QQ---------
	Alteromonas naphthalenivorans	WP_013785512	-E----------S--NGD-T	T	---I-D--EQ--T------
	Alteromonas stellipolaris LMG	ALM91943	-E----------S--NGD-T	T	---I-D---Q--T------
	Amphibacillus jilinensis	WP_017472154	-E------------Q-SK-E	T	---I-N--E----H-----
	Amphibacillus marinus	WP_091493525	-E------------QTSQ-E	T	----KD----L--------
	Anaerobacillus alkalidiazotrop	WP_071390516	------------S-TAPD-T	T	---ISN---E--R------
	Anaerobacillus isosaccharinicu	WP_071315977	------------S-TAP--K	T	---I-G--EE--R------
	Aquicella lusitana	WP_114833400	--------------QT-D-R	V	---MRALVEQ--T------
	Aromatoleum aromaticum	WP_011238317	------------R-ET---A	T	---I--L--GL--------
	Arthrobacter phage Correa	ASR80143	------------S--TG--K	V	---IHE-VK---YD-----
	Azoarcus communis	WP_108950844	-E----------R-ET-D-R	S	---IS--VEA--R------
	Azoarcus olearius	WP_065341164	------------R-ET-D-R	S	V-----LV-G--R------
	Azoarcus toluclasticus	WP_018990746	------------R-ET-D-R	T	V-----L--GLRR------
	Azonexus hydrophilus	WP_028994904	------------R-ECPD-R	L	V--I--LVH-L--------
	Bacillaceae bacterium B16-10	RXJ01806	------------S-TAP--Q	T	---ISS--EE--R------
	Bacillus aidingensis	WP_026700583	-E---I------S-EG-D-K	T	---IS--VEQ--T------
	Bacillus aquimaris	WP_113970757	-E------H---S-SG---E	T	---ITNL--T--N------
	Bacillus caseinilyticus	WP_090892619	------------S-PT-D-G	A	----GE-VKQ--T------
	Bacillus clausii	WP_094426376	------------S-EG---K	T	V--ITE-VEQ--M--N---
	Bacillus coagulans	AJ023224	------------S-QTRD-R	T	---IT---EQ--T------
	Bacillus halodurans	WP_010899587	-E----------S-EG---K	T	V--IS--VEA---Q-----
	Bacillus hemicellulosilyticus	WP_035342806	-N----------S-QG---K	T	V--ISEA--Q--N------
	Bacillus humi	WP_057997323	-E------------ETSR-E	T	----KN--NQ--N------
	Bacillus indicus	WP_029280463	-E------R---S-EG---E	T	V--I-KL--Q--N------
	Bacillus lehensis	WP_038485997	------------S-EG---K	T	V--IKE-VEQ--T------
	Bacillus ligniniphilus	WP_017726370	------------S-EG---K	T	V--IK----D-RK------
	Bacillus marmarensis	WP_022629028	------------S-EG---K	V	V--ISEL-EQ---------
	Bacillus ndiopicus	WP_042473263	------------S-PA---E	T	---I-N--EQ--K------
	Bacillus onubensis	WP_099362818	-E------------E-SR-E	T	----KN--NQ--N------
	Bacillus patagoniensis	WP_078392145	-E----------S-EG---K	T	---I-H-V-Q--N------
	Bacillus polygoni	WP_088032676	------------S-PDPD-G	T	----GKI-NE--S------
	Bacillus pseudalcaliphilus	KMK77645	------------S-QG---R	T	V--ISE--EQ--E--N---
	Bacillus pseudofirmus	WP_012960338	------------S-EG---K	V	V--ISEL-EQ---------
	Bacillus salarius	WP_125554949	-----I------S-EG-D-K	I	V--IS----Q--N------
	Bacillus sinesaloumensis	WP_077618508	-E------------E-SR-E	T	----KN--NQ--N------
	Bacillus trypoxylicola	WP_061947854	-E----------S-EG---K	T	---ISD--EQ--Q--N---
	Balneatrix alpica	WP_027311839	--------A---S-PA-D-R	Y	------ILEE--R------
	Candidatus Baumannia cicadelli	WP_038499119	-----I--R-----GT-D-H	Y	----SD--FQL--------
	Candidatus Competibacteraceae	RUQ37462	-----I--------QT-D-R	V	---M-R--EQLRCD-----
	Candidatus Fukatsuia symbiotic	WP_072550212	--------------ST-D-H	Q	-----R--EQL-CD-----
	Candidatus Peregrinibacteria b	PIP65706	-N------------EG-D-T	V	T--IT--L--L-N------
	Catalinimonas alkaloidigena	WP_089678222	-N----------S-EAPD-R	V	---IG--VE--RR------
	Chitinophaga arvensicola	WP_089894676	------------S-ETRD-K	V	---ITEAVNT--N------
	Chitinophaga eiseniae	WP_078672083	------------S-ETTS-E	V	---ISIAV-T--K------
	Chitinophaga filiformis	WP_089832805	------------S-ETKD-R	V	---ISEAVKT--N------
	Chitinophaga ginsengisegetis	WP_079468810	------------S-ETRS-K	V	---ITEAVNT--N------
	Chitinophaga sancti	WP_072361630	------------S-ETKD-K	V	---I-DA-NT--K------
	Chitinophagaceae bacterium	RYZ29259	-E----------S-EGR--E	V	---ITE--NQ--K------
	Chryseobacterium carnis	WP_125023792	--------A---S-TG---K	V	V--ITE---Q--K------
	Chryseobacterium chaponense	WP_076388187	--------A---S-TG---K	I	V--FSE--EQ--K------
	Chryseobacterium elymi	WP_116014438	--------A---S-QG---K	V	V--ITE---Q--K------
	Chryseobacterium haifense	WP_123903423	--------A---S-KG-DNK	I	V--ISE---Q--K------
	Chryseobacterium jeonii	WP_039353523	--------A---S-TG---K	V	V--ITE---Q--K------
	Chryseobacterium nakagawai	VEH21997	--------A---S-NG-D-K	V	V--ITE---Q--K------
	Chryseobacterium solincola	WP_039346425	--------A---S-EG---K	V	---ITE---Q--K------
	Chryseobacterium treverense	WP_089820399	--------A---S-EG---K	V	---ITE---Q--K------
	Chryseobacterium vrystaatense	WP_034745724	--------A---S-QG---K	V	V--ITE---Q--K------
	Cohnella kolymensis	WP_041065736	--------S-----ETPD-G	T	---IQ--VE---R------
	Cohnella luojiensis	TFE24875	--------S-----ETPD-R	T	---IQ--V-G--T------
	Cohnella lupini	WP_115992995	--------S-----ET-D-R	T	---IQ--V-G--N------

Other Bacteria (0/>200)

Cohnella phaseoli	WP_116061121	--------S-----ETPD-R T ---IQ--V-A--R------
Cohnella thermotolerans	WP_027091467	--------S-----ET-D-R T ---I---VEA--N------
Colwellia aestuarii	WP_077285384	--------V---N-PAQ--E T ------L--DL-N------
Dakarella massiliensis	WP_049686635	-N------A-----RT---D K ---ISE---Q-R-H-----
Deltaproteobacteria bacterium	TDJ09124	--------A---R-KN-K-E T V---T-LV-GL--------
Euryarchaeota archaeon	RZD39015	------------R-ET-D-R I ----S-AV-M--N--N---
Euryarchaeota archaeon TMED117	RPG80205	------------K-E-KD-R I ----E-A--Q--N--N---
Facklamia miroungae	WP_090288845	-----I------S-TDCE-Q V ---IQ------RKQ-----
Ferriphaselus amnicola	WP_062627343	------------S-GA-D-R T ---IKIAV-QL--------
Flavisolibacter ginsengisoli	WP_072833525	------------S-EG-H-Q V ---V-D--KQ--T------
Flavobacteriaceae bacterium JJ	WP_088358504	--------A---S-TG---K V ---ISE---Q--K------
Flavobacteriia bacterium	TAF85310	-E------S---S-PT-S-E H ---IQ---EQ---------
Fontibacillus phaseoli	WP_114495428	--------S-----EAPD-R R ---I-N--EA--N------
Gallionellales bacterium RIFCS	OGS95556	-N----------S-AT-D-R V V--ISE-V-Q--R------
Gammaproteobacteria bacterium	OGT06924	-N------------QASD-R M ---ISE--EQ-R---N---
Gilliamella apicola	WP_065561974	--------------GA-D-R Q ---IS-L-EQ---D-----
Gilliamella bombi	WP_085165202	--------------GA-D-R Q ---IS-L--Q--ND-----
Gilliamella intestini	WP_091124855	--------------GT-D-R Q ---IS-L--HV-ND-----
Hydrogenophilus thermoluteolus	WP_119334938	-----I------R-EAPD-R V ---I---VE-L-N------
Jeotgalibacillus salarius	WP_134379997	----S-------E-NKS--E T ----RE--S----S-----
Jeotgalibacillus soli	WP_041090045	-E--D---------KKS--D I ----HD-------------
Kurthia gibsonii	WP_087680566	------------S-P--D-G T ---IK---T---E--N---
Kurthia huakuii	WP_029498519	------------S-PD-S-G T ----HN--E---K--N---
Kurthia massiliensis	WP_010285983	------------S-PDND-G T ---MQ---E---T--N---
Kurthia senegalensis	WP_010300899	------------S-PDEK-G T ----Q----A--QT-----
Kurthia sibirica	WP_109304997	-N----------S-PD-T-G T ---IKN------N------
Lacimicrobium alkaliphilum	WP_099033360	-E----------S-QGID-E V ---I---VEQ--N------
Legionella maceachernii	WP_058452269	--------Q-----PA-D-R V ----SE-LQQ--T------
Legionella pneumophila	WP_010948554	------------S-PT-D-R T ----SE-VQQ--S------
Legionella quinlivanii	KTD51908	--------R---S-P----E T ----SE-LQQ-QS------
Legionella saoudiensis	WP_058534080	------------S-PT-D-R H ----SE-LEQ--K------
Legionella waltersii	WP_058480739	------------S-PT-D-R T ----SEIVQQ--M------
Luteibacter rhizovicinus	WP_046967265	--------R-----PT-D-K V V--I-W-V-E--R------
Lysinibacillus chungkukjangi	WP_107935626	------------S-PTS--E S ----KK---Q---------
Lysinibacillus fluoroglycofeni	WP_107942676	------------S-PA---E T ---I-N--QQ--N------
Lysinibacillus halotolerans	WP_122971716	-E----------S-PTS--E T ----KK---Q--N------
Lysinibacillus meyeri	WP_107841556	------------S-PA---E T ---I-N--EQ-NK------
Meiothermus hypogaeus	WP_119341563	-E----------S-EG---Q T ---M-W-VEE--R------
Mixta gaviniae	WP_104958417	------------S-GA-D-R Q -----K-L-QL-ND-----
Oceanimonas baumannii	WP_094278094	-E----------S-EA-D-R V ---IS-AMET--A------
Oceanimonas doudoroffii	WP_094201340	------------S-EA-D-R V ---IS-ALET--T------
Oceanimonas marisflavi	WP_107850843	-E----------S-AA-D-R V ---IS-ALET--T------
Oceanimonas smirnovii	WP_019936056	-S----------S-ET-D-R V ---IS-ALET--T------
Ornithobacterium rhinotracheal	WP_128502224	------------S-A-P-NK T ---IREAV-T--N------
Paenibacillus amylolyticus	WP_062834875	--------S---T-EAP--D K ---I-A------N------
Paenibacillus barcinonensis	WP_110896740	--------S---T-EAP--E K ---I-A------N------
Paenibacillus borealis	WP_042214444	-E------S-----E-KD-R H ---I-N--E----------
Paenibacillus camerounensis	WP_042198555	-E------S-----E-SD-R H ---I-A--G---N------
Paenibacillus chibensis	WP_127602596	-N------S---N-EAPD-T H ---I-N--E----------
Paenibacillus crassostreae	WP_068658365	-E------S-----E--E-K K ---I-N--E---N------
Paenibacillus darwinianus	WP_036579550	-E------S-----EA-D-R R ---I-E--E---R------
Paenibacillus dendritiformis	WP_111155682	--------S-----ETKD-R I ---I-N--EQ--T------
Paenibacillus durus	WP_025700806	-E------S-----E--D-R Q ---ISS--E---N------
Paenibacillus forsythiae	WP_025702215	-E------S-----E--D-R H ---ISN-----RN------
Paenibacillus glucanolyticus	WP_063479264	--------S-----ETPD-Q H ---I-N--E---N------
Paenibacillus graminis	WP_025706201	-E------S-----E--D-R H ---I-A------N------
Paenibacillus herberti	WP_089523511	--------S-----EDK--S T H--I-A-----RN------
Paenibacillus ihbetae	WP_077565593	--------S-----EAPD-R R ---I-N--E---T------
Paenibacillus ihuae	WP_054940866	-E------S-----E-KD-R T ---ISA--E----------
Paenibacillus illinoisensis	WP_110823255	--------S---T-EAP--E K ---ISA------N------
Paenibacillus jilunlii	WP_062528454	-E------S-----E--D-R H ---I-A------N------
Paenibacillus lautus	WP_076321177	-N------S-----ETPD-R Y ---I-N------N------
Paenibacillus pabuli	WP_062325377	--------S---T-EAP--E K ---I-A------N------
Paenibacillus pini	WP_036647114	--------S---T-E--D-N R ---ISN--E----------
Paenibacillus pinihumi	WP_028561136	--------S-----QAPD-R V ---I---V---RR------
Paenibacillus riograndensis	WP_020431198	-E------S-----E--D-R H ---I-A------N------
Paenibacillus sabinae	WP_025335206	-E------S-----E--D-R H ---ITN--E---K------
Paenibacillus senegalimassilie	WP_059053009	--------S---S-EAPD-R K ---I-A-VEA---------
Paenibacillus solani	WP_054401950	--------S-----ETPD-R H ---I--------N------
Paenibacillus sophorae	WP_036599818	-E------S-----E--D-R H ---ISN------N------
Paenibacillus taichungensis	WP_113054504	--------S---T-EAP--D K ---I-A------N------
Paenibacillus thiaminolyticus	SUA98236	--------S-----ETKD-R V ---I-N--EQ--T------
Paenibacillus uliginis N3/ 975	SMF86285	--------S-----ETPD-R H ---I-N--E---N------
Paenibacillus wynnii	WP_036655991	-E------S-----E--D-R H ---ISN--EA--N------

Other Bacteria (0/>200)

Paenibacillus xylanexedens	WP_074095463	--------S---T-EAP--E K ---I-A------N------
Paenibacillus zanthoxyli	WP_025692733	-E------S-----E--D-R H ---ISS------N------
Paenisporosarcina antarctica	WP_134209838	--------R---S-P-T--Q T ----KN--K---N------
Pantoea agglomerans	WP_069025334	------------S-GT-S-Q E ----S--MEQL--D-----
Parendozoicomonas haliclonae	WP_087107145	------------S-EGP--E V V--IKDL-NE--K------
Pedobacter duraquae	WP_133552987	--------S---S-PTPE-G T ---I-KIV-T--N------
Planococcus donghaensis	WP_065526192	------------S-ATTT-G T ----KN--E---K------
Planococcus maritimus	WP_069575963	------------S-AATD-G Q ----KN--E---N------
Planococcus plakortidis	WP_068869240	------------S-AATD-G Q ----KN-VE---------
Planococcus salinarum	TAA72318	------------S-QTTT-D T ----KN--E---K------
Prevotella fusca	WP_025078327	--------H---S-PDYK-G T ---IKN-V-M---------
Prevotella histicola	WP_008823149	--------H---S-PDYK-G T ---I-N---Q---H-----
Prevotella veroralis	WP_018910122	--------H---S-PDYK-G T ---I-N-L-Q-----N---
Pueribacillus theae	WP_116553225	--------Y---S-PDG--G T ---IKE-VHQ---------
Rhodospirillum centenum	WP_012567181	--------------ET-D-R V V--I--LV-G-RG--T---
Rummeliibacillus stabekisii	WP_066788119	------------S-PDGM-G T ---IQN--E---K------
Saccharibacillus kuerlensis	WP_018977664	-E------S-----EA-D-R K ---ISA------N------
Salibacterium qingdaonense	WP_090926749	-E---I------S-EG-D-K T V--IS----Q--T------
Salinisphaera japonica	WP_123657032	--------Y---S-PAP--Q A ----VR--EA---------
Salinisphaera shabanensis	WP_006913294	--------Y---S-PTPG-E S V----R-------------
Salipaludibacillus aurantiacus	WP_093055311	-----I------S-P-PD-E S ----G---EQ--T------
Salisediminibacterium haloalka	WP_093073916	--------R---S-PAP--E S V---RD--EQ---------
Salisediminibacterium halotole	WP_121439197	--------R---S-PAP--E S V---RD--EQ---------
Salsuginibacillus halophilus	WP_106588917	-E----------S-EG-D-Q T V----D--EA-R-------
Sediminibacterium salmoneum	WP_026763029	------------S-EG-D-K V ---I-D-LNQ--NQ-----
Sediminitomix flava	WP_109616399	-E------------EKP--E T V--I-N---QL-N--N---
Sinobaca qinghaiensis	WP_120194433	------------S-EG---R T V--ISD--KQ--E------
Sphingobacteriales bacterium	RTL59955	-E----------S-EA-D-K I ---VSDL-EQ--K------
Sphingobacteriia bacterium	TAF51502	------------S-AG---E T ---ITD-LHQLR-------
Sphingobacteriia bacterium RIF	OHC85779	------------S-EG-D-K V ---ITD-LHQL--------
Sporolactobacillus pectinivora	WP_100487916	--------S---S-PGK--K T ---I--LM-Q--N------
Succinatimonas hippei	WP_009142703	------------S-AAPD-K V ---ISNAV-M---------
Sutterellaceae	WP_116269888	-N------A-----RT---D K ---ISE---Q-R-H-----
Tatlockia micdadei	WP_045098073	-----I--Q-----PA-D-R I ----SE-LQQ--N------
Thauera hydrothermalis	WP_114649849	------------H-EA-D-R V ---I---VE-LRR------
Thermobacillus composti	WP_041854612	--------------EAPD-R M ---I-R-VEA-RT------
Thermobacillus composti KWC4	AGA59506	--------------EAPD-R M ---I-R-VEA-RT------
Thiohalobacter thiocyanaticus	WP_096367252	--------Y---S-PTPD-R H ---I---VEQL--------
Wohlfahrtiimonas larvae	WP_077925093	------------S-PT-D-R Y ---ISE-V-Q--N--N---
Xenorhabdus bovienii	WP_038213949	--------------G--D-R Q -----K-LEQL-SD-----
Xenorhabdus budapestensis	WP_099135060	--------------GA-D-R Q ----T---EQL-RD-----
Xenorhabdus ehlersii	WP_099133035	-----------------D-R Q ----T---EQL-SD-----
Xenorhabdus khoisanae	WP_047964181	--------------G--D-R Q ----T---EQL-SD-----
Xenorhabdus koppenhoeferi	WP_092548201	--------------G--D-C Q ----T--LEQLRSD-----
Xenorhabdus miraniensis	WP_099114001	--------------G--D-R Q V---T---EQL-ND-----
Xenorhabdus thuongxuanensis	WP_074020353	--------------G--D-R Q ----T---EQL-SD-----
Zetaproteobacteria bacterium	PCJ98181	--------E---S-KT---Q T ----SN--EQ--S------
Zobellella denitrificans	WP_094039155	--------H---S-QG-D-R V ---ISLAL-T-R-------
Zobellella endophytica	WP_106730888	--------H---S-QG-D-R V T--IS-ALET---------
Zooshikella ganghwensis	WP_027707131	-E----------S-EG---E V V---S-L-YD--T--N---

Supplemental Figure 68

A partial sequence alignment of the thymidylate synthase protein containing a one amino acid deletion (boxed) that is exclusively shared by all members belonging to the Sporosarcina clade and absent in all other bacteria.

Group	Organism	Accession	31		90
Sporosarcina clade (9/9)	Sporosarcina ureae	WP_083032299	WAFLIDMLILSAISGIFVKPVFRVLDIAITKPS	AF	LFSPYKVTALVLLLLYFIVMTKIAGQTVGK
	Sporosarcina newyorkensis	WP_009497682	------L-LI-S--------I--LM----N--F	--	-------A--L-------L---L-------
	Sporosarcina koreensis	WP_060209226	--YV--L--V-S-----I--I------K-SN--	FL	--------L-I-F-G--AF---M-------
	Sporosarcina psychrophila	AMQ08476	--YT--L-V-------II--I---AGWE--N--	F-	F--T----I-I-M---AL---YLQ-----
	Sporosarcina pasteurii	WP_115364127	--YC--IIV-F----L-I--I----E-PV-N--	FL	F-T---I-I-IVT----LF---YLQ-----
	Lysinibacillus sphaericus LMG 22257	AOV09128	--YC--LIV--S-G-L-I--I------PV-N--	FL	F-T---I-L-IVT----LL---YLQ-----
	Lysinibacillus sphaericus NCTC 11025	WP_125103261	--Y---II-VG----LL---I---V-YP-KD-I	FL	-Y----LIL-A-F-A--LL---FFS-----
	Bacillus sp. OxB-1	WP_052484117	--YV--L-VV------LL--A-HLSGM--SN-P	F-	------L-T-AVF----AL---LFR--I--
	Filibacter sp. TB-66	WP_124069881	--YT--L-VIA-----V---I---AH-D-SS--	FL	------I---L------TM---FLQ-----
Other Bacteria (0/>100)	Anoxybacillus amylolyticus	WP_066327682	--Y-L-L-VVGS-DRLI-Y-L--LF-LS-D-TT		M---IAIATV-VFYG--VL---SFQ--I--
	Anoxybacillus tepidamans	WP_051530032	--Y-L-L-VVASLNRLL-Y-L--LS-LSLEEKT		----IA-VTT-VFYT--VI---YFQ-----
	Anoxybacillus vitaminiphilus	WP_111644489	--Y-L-LVVIAS-N--I-Y----FM-LPLEENH		M-T-LSIATAATFY----F----FK--I--
	Bacillaceae bacterium EAG3	WP_104850098	--YI--I--VGS--RL-I--I--L---P-DSSG		I---FT-IDSIVFFG--FI--LLFA--L--
	Bacillaceae bacterium SAOS 7	WP_095477437	--Y---LIVIGS--RLIIY----L-EVD-HVSG		M-E-IN-ATAAVFY----IF--YW------
	Bacillaceae bacterium SAS-127	WP_094834333	--YV--LIVIGS--RLIIY----L-EVD-HASG		M-A-IN-ATAAVFY----IF--YW------
	Bacillus acidiproducens	WP_018662479	--Y-L-L--IQSVNWLILH----L-KLSLADD-		M---ISILSAAVFY----L---YFS--L--
	Bacillus aciditolerans	WP_121447456	--Y-L-LVVIGS-NR-LIY-I--A---PLSDSD		M--AAAIATA-VFY---VL---FFS--L--
	Bacillus alkalitelluris	WP_078545087	--Y-A-LI-IGSVNR-IIY----L---PV-ESN		M-A-VSILTALTFFA--VL---FFQ--L--
	Bacillus aquimaris	RBP03900	--Y-L-LIVIGS-TRLV-N---KL---SLSEG-		M-A-ISILTSIVFYG---L---YFS--I--
	Bacillus azotoformans	WP_035194307	--YV--LIVVGSLHR---Y--L-W---S-IET-		ML-TVAISTA-VMYA---I---LL---L--
	Bacillus badius	KZO01496	--Y-L-LIVVGSL-RLLIY-L--AA--DTAANG		I--AVNIATALVFY---VL---WR---I--
	Bacillus bataviensis	WP_007087412	--Y-L-LIVVGS-ERLLIN-F--A---SLSEFN		M-A-ISIASA-IFY---VL---YFR--L--
	Bacillus camelliae	WP_101354937	--Y-V-LIVIGS-DRLL--TI--L---S-DNTG		M---FS-ATAMVFYV--VL---YT---I--
	Bacillus campisalis	WP_046523473	--Y-L-LVVIGSLNRLI-F----A-GLPVAESG		M-A-AAIVTAIVFY---VL---YF---L--
	Bacillus canaveralius	WP_101576915	--Y-L-LIVVGS-DRLIMN-I------PLHEMG		I-A-ITIVTAITFYA--VL---FF---L--
	Bacillus cavernae	WP_126865749	--Y-T-II-VGS-NR-I---I-KA--LSPG W		---EAICTSIIFY----L---FLKK-L--
	Bacillus coagulans	WP_014097729	--Y-L-L-VVASVKWLVIH-I--LSGLSL-EEK		IYA-IT-ASA-VFYG--VL---FF---L--
	Bacillus dakarensis	WP_077214248	--Y-L-LVVIGS-DR-IIN-I-KM-GLELNAI-		I-A-ISIA-AITFYA--VL---FF---L--
	Bacillus dielmoensis	WP_081798060	--Y-L-LVVIGS-ER-L-N-L--M---SLSEFN		M-A-VSIASA-IFY---VL---FFN--I--
	Bacillus drentensis	WP_082797425	--Y-L-LVVVGS-VRLLIE----L---PLAEAN		M-A-ASIA-A-IFY---VL---YFN--L--
	Bacillus enclensis	SCB92809	--Y---LIVIGS-TRLI-----K---VSLVEG-		M-A-ISILSAIVFYG--VL---FFS--I--
	Bacillus fastidiosus	WP_082800052	--Y---LIVITS-NR-IIY-ILTLIGADTSN--		M---VTILSSITFF---VL---YLQ--L--
	Bacillus firmus	WP_035330320	--Y-L-LVVVGSVDR-LIN-I--A---PLHESN		--A-ISIATAITFYA--VL---FF---L--
	Bacillus fordii	WP_018708789	--Y-L-LAVIGGV---L---L--A--LQLSASG		I-T-INIASALIFY---VL---FFR--L--
	Bacillus fortis	WP_120074188	--Y-L-LAVIA-VG--I---L--A--LQLSASG		I-THVNIASALVFY---VL---FF---L--
	Bacillus freudenreichii	WP_126431500	--Y-L-LIVIASV---I---L--A-GLELGTGG		IIT-FNIVSALVFY---VL---FF---L--
	Bacillus ginsengihumi	WP_025731665	--Y-L-LVVIGS-DRLI-H-I--IA-VPLHDG-		M---ISIVSALVFYG--VL---WW---I--
	Bacillus gottheilii	WP_066443014	--Y-A-IIVIGS-DRLLIN-L--L--LPLYETG		---MMTIATALTFY---VL---FF---L--
	Bacillus halmapalus	WP_078379400	--Y-L-LI-IWS-G--VIF-I--M-N-PLSQSF		MY--ATIATTI-WFS---I---YFK--L--
	Bacillus horikoshii	WP_082891893	----L-L--IWS-GA-V-F-L--L-G-SESQSF		----ASIATTII-YG---L---YFK--L--
	Bacillus horneckiae	WP_066396785	--Y-L-LIVIGS-DRLI-N-I--L---SLKEH-		I-A-ISIITAITFYG--VL---FFN-----
	Bacillus humi	WP_057999512	--Y-L-LVVIGSMNR-LIY-A--A---PL-ESH		---GASIATALVFY---VL---FFN--L--
	Bacillus indicus	WP_051827225	--Y---IIVIWS-NR-VFH-L-TW---PVS-TF		I---IAICSAAVFYA--VL---FL---L--
	Bacillus jeotgali	WP_079510351	--Y-L-LIVIGS-NRMIIN-I--A---PLAEDG		I---MAIATA-VFY---VL---FL---L--
	Bacillus kochii	WP_095372277	--Y-L-WI-IGS-N--LIY----A--VSLNNS-		Y--AVAIATALTFY---VL---YL---L--
	Bacillus kwashiorkori	WP_077216182	--Y-V-IILIA--T-MIIT-L--YF-WPMNEI-		-----TLATAAVFY----L---FFQ--L--
	Bacillus lentus	SQI54934	--Y-L-CAVIFG-N-M-I-----M---SLS-VG		V--A-AIISGLVFY----L---LLD--L--
	Bacillus litoralis	WP_066326705	--Y---L-VIGSVNR-VIY-L-DLIGLNNNNSF		----VSIATA-IFFA--VL---YFN--L--
	Bacillus massilioanorexius	WP_019243874	--Y-L-LIVIFS----LIS---KA--LFQYSFW		I-T--ALAKA-IFY---VL---FL---L--
	Bacillus massiliogabonensis	WP_102274266	--Y-L-LIVIGS-ERLI-N-I--M---SLNEF-		I-A-ISIVTAIIFYA--VL--RFFN--L--
	Bacillus massilionigeriensis	WP_075982571	--Y-L-LIVIGSLNRLLIY-L--FM--DVHAT-		M-A-VSIATA-IFY---VL---YL---L--
	Bacillus mediterraneensis	WP_083413173	--Y-T-LI-IWSLN-LLIY-IL-G-G-STD-D-		--D-AT-LTAIIFY---VL---YL---L--
	Bacillus mesonae	WP_066386069	--Y-L-LIVIGS-VRLSIN----L--LSLAEY-		M-A-ISIASA-IFY---VL---YFR--L--
	Bacillus methanolicus	WP_003347398	--Y-L-L-VIGS-DRMI-N-L--LF-LPLHEL-		M-A-ISIATAITFYG--LL---FF---L--
	Bacillus ndiopicus	WP_042474632	---I--G--V--AI--VIN-I-YFM-WSLM-TP		WYA-LAIISTIVYYS--AI---FWQ-----
	Bacillus nealsonii	WP_040343187	--Y-A-LI-V-SVNR-LIY-IL-I--VPLHESG		I--TVNICTAITFY---VL---FLK--L--
	Bacillus niacini	WP_034676794	--Y-L-LIVIGS-ERLIIN-L--IFE-PLVEFN		M-A-IS-ASA-IFY---VL---YFH--L--
	Bacillus niameyensis	WP_062104254	--Y-L-L--IFS-N--I---I--AFG-SLSESG		IITG-GIASTIIFYA---L---FFK--L--
	Bacillus notoginsengisoli	WP_118920869	--Y-L-LIVAGS-FRLIA-----FFE-ESSGGL		---DTIVSA-IFY---VL---FFH--L--
	Bacillus novalis	WP_066090823	--Y-L-L-VVGS-ER-LI--L------SL-EFN		M-A-ISIASA-IFY---VL---YFN--L--
	Bacillus oceanisediminis	WP_019383063	--Y-L-LIVVGSVDR-LIN-I--A---PLHESN		--A-ISIATAITFYA--VL---FF---L--
	Bacillus onubensis	WP_099352307	--Y-L-LVVIGS-NR-LIY-A--A---PLSDSD		I--ATSIATA-VFY---VL---FFS--L--
	Bacillus persicus	WP_090741611	--Y-L-LVVIGSVNRLVIY----S--LS-AET-		M-T-IAIATAFTYY---VL---LT---L--
	Bacillus praedii	WP_057761398	--Y-L-LIVIGS-ERLIIN----L---SLNEF-		I-A-ISIVTA-IFYA--VL--RFFN--L--
	Bacillus salsus	WP_090849207	--Y-L-LIVIASLNR-IIY-IL-L---P-GD-N		M-A-VSILTAITYF---VL---YFK--L--
	Bacillus selenatarsenatis	WP_084135433	--Y-L-LIVIGS-NRMIIN----A--VPLIEDG		IL--MTIATA-VFY---VL---YF---L--
	Bacillus selenatarsenatis SF-1	GAM13602	--Y-L-LIVIGS-NRMIIN----A--VPLIEDG		IL--MTIATA-VFY---VL---YF---L--
	Bacillus shackletonii	KQL55404	--Y-V-LIVIGS-DRLL--SI--L-G-SLENTG		M---IAI-TALVFYG--VL---FT---I--
	Bacillus sinesaloumensis	WP_077621042	--Y-L-IVVIGSLNR-LIN-S--A---SLSES-		I--AASIATAIVFY---VL---FF---L--
	Bacillus smithii	WP_003355649	--Y-L-LIVIGSLKR-LIL----W--LPVFAHE		--A-IH-ASA--FYG--LLF--FT------
	Bacillus soli	WP_084374904	--Y-L-LIVVGS-ERLLIN-L--M---SL-EFN		I-A-ISIASA-IFY---VL---YFR--L--
	Bacillus sporothermodurans	WP_066226891	--Y-L-IVVVAS--RLIIR----IV--PL-DSG		--T--SIVTTL-FY----L---FLSA-I--
	Bacillus subterraneus	WP_044390744	--Y-L--IVIGS-NRLIIN----AM--PLLEEG		F---KTIMTA-VFY---VL---YL---L--
	Bacillus terrae	WP_120115519	--Y-L-LVVVA-V---I------A--FQLSASG		I-T-INIASALI-Y---VL---FF---L--

Other Bacteria (0/>100)

Bacillus timonensis	WP_042342354	--Y-L-LVVI-S-NR-LIN-I--A--LPLSES-	M--AASISTAIVFY---VL---FFS--L--
Bacillus tuaregi	WP_084786924	--Y-L-LVVIASVN--L-Y-I--A---K-SD--	I-A-VSI-TA-IFYG--VL---FF---L--
Bacillus vietnamensis	KPL57985	--Y-V-LIVIGS-TRLI-N-I-KL---SLSDG-	M-A-ISILTSIVFYG---L---YFS--I--
Bacillus vireti	KLT16262	--Y-L-LIVVGS-ER-LI--L------SL-EFN	M-A-ISIVSA-IFY---VL---YFN--L--
Bacillus weihaiensis	WP_072578871	--Y-V-L-VI-SVNR-I-H-L-SL-G-NTSESF	---LTIVTT-TFFA--VLL--YF---L--
Bacillus wudalianchiensis	OCA92245	--Y-L-LIVVGS--RLLIY-L--A---EVASSG	---AVNIATALVFY---VL---WK---I--
Bacillus zeae	WP_119114147	--Y-F-LIVIWS-N-LLIY----A--LSVSGEE	--K-VNIATA-TFY---VF---YL---L--
Caryophanon tenue	WP_066542133	--YI--L--IF-LTS-I---I-Y-IGTTTDEYT	WYA-FTIASA-IFYG--VL---FT------
Edaphobacillus lindanitolerans	SIT85177	-SY---LIV-GSV-MLVIR-LS-LGGLE-APDG	I-A--NIATAAVFF---VL---FF------
Falsibacillus pallidus	WP_114745149	--Y-L-LIVIGS-DRLLI-----L--FQLHDT-	M-A-ISILFAIVFYG--VL---YF-A-I--
Lysinibacillus acetophenoni	SOC36588	F--I--QG-V--MI--V-Y-I-K-F-LNL-DTR	Y-A-ISIISTIFYFS---L---YF---L--
Lysinibacillus boronitolerans	WP_016991567	----L-GF-I---G--L-N-I-YLM-WSLSDSV	WYA-ISIITAIFYYS--VL---FF---L--
Lysinibacillus contaminans	WP_082332541	------S-VI--VI--CIN-I-YIM-WSLAESI	WYA-ISIISA-IYYA--VL---FL---L--
Lysinibacillus endophyticus	WP_121212691	---A--S-LV---V--L-N----LFGWSLNDSN	WYA-ITIISAIIYYA--VLT---WN--I--
Lysinibacillus fusiformis	WP_004226185	----L-GF-I--VG--L-N-I-YLM-WSLSDSV	WYA-ISIITAIFYYS--VL---FF---L--
Lysinibacillus macroides	WP_053996468	----L-GF-V--VI--LIN-I-YLM-WSLSESV	WYA-ISIITAI-YYS--VL---FF---L--
Lysinibacillus odysseyi	WP_052124909	------S-VI---V--VIN-I-HLM-WSFDESV	WYR-IVILSAIVYYA----L--LW------
Lysinibacillus sinduriensis	WP_052129998	---VV-I--V--LV--VIN-L-HLF-WSLSEKN	WY--MSIVSG-FYYA--TIL--YWQ-----
Lysinibacillus sp. B2A1	AVK83898	----L-GF-IT-VG----N-I-YLM-WSLSETV	WYA-ISIITAIFYYS--VL---FF---L--
Lysinibacillus sp. BK089	WP_132357209	----L-GFVIT--G--L-N-I-YLM-WSLSETV	WYA-ISILSAI-YYS--VL---FF---L--
Lysinibacillus sp. FJAT-14222	WP_053592374	----L-G--IT-VG-VL-N-I-YLM-WSLSETV	WYA-MSIISAI-YYS--VL---FF---L--
Lysinibacillus sp. FJAT-14745	WP_053484994	----L-GFVIT-LC--L-N-I-YFM-WSLSETV	WYA-ISIISAI-YYS--VL---FF---L--
Lysinibacillus sp. LK3	WP_048393550	----L-GF-I--VG--L-N-I-YLM-WSLSDSV	WYA-ISIITAIFYYS--VL---FF---L--
Lysinibacillus sp. OL1	WP_131521337	----L-GF-I--VG--L-N-I-YLM-WSLSDSV	WYA-ISIITAIFYYS--VL---FF---L--
Lysinibacillus sp. YR326	WP_134021911	----F-GFVIT-VG--L-N-I-YLM-WSLSETV	WYA-ISIISAI-YYS--VL---FF---L--
Lysinibacillus sp. YS11	WP_103118657	----L-GF-I--VG--L-N-I-YLM-WSLSDSV	WYA-ISIITAIFYYS--VL---FF---L--
Lysinibacillus sp. ZYM-1	WP_054610845	----L-GF-V--VV--LIN-I-YLM-WSLSESV	WYA-ISIISAI-YYS--VL--RYF---L--
Lysinibacillus tabacifolii	WP_108029955	---VL-G--I---I--A-N-I-YLM-WSLSESV	WYA-ISIISA--YYC--VL---FF---L--
Lysinibacillus telephonicus	WP_126294626	---AF-S--V---V--L---I-HLF-WSLSESN	WYA-MTIISA-FYYS--VIT---WQ-----
Lysinibacillus xylanilyticus	WP_049667484	----L-GFVIT-VG--L-N-I-YLM-WSLSETV	WYA-ISIISAI-YYS--VL-A-FF---L--
Lysinibacillus xyleni	WP_097072890	---A--S-VV---V--L-N-I--LFGWSLSDSN	WYA-ITIVSAI-YYA--VLT--FWN-----
Mycobacteroides abscessus subs	SHQ01426	--Y-L-L-VIGS-DR-LIN-I--AIGLDLHSGD	F-A-ITIATALTFYG--VL---FF---L--
Paenisporosarcina quisquiliaru	WP_090564272	--YTV-I-V-AS-GMLLI--I--LFSLELNNSE	WYA-FT-ITA-IFYA--VL---LCS-----
Parageobacillus thermantarctic	WP_090947644	--Y-L-L-VVGS-NRLL-F-L-HL---SVERT-	M-A-ATIATT-IFYA--VL---FFQ--L--
Parageobacillus thermoglucosid	WP_013876454	--Y-L-L-VVGS-NRLL-F-L-HL---SVERT-	M-A-ATIATT-TFYA--VL---FFQ--L--
Planococcus citreus	WP_121301142	--Y---V-VI--V-S-LIR-L-ALFGWETAETA	WYA--AILTAIVFYG--VL---FFA-----
Planococcus donghaensis	WP_008429092	--Y---L-VVA-LTS-L-----ALSGLET-NMP	WYG--AIVSA-IFYG--V----FF------
Planococcus halocryophilus	WP_008497188	--Y---L-VVA-LTS-L-----ALSGLET-NMP	WYG-FA-VSA-IFYG--VL---FF------
Planococcus halotolerans	WP_112224638	--Y---L-VI--VTS-LL----AL-GLSMQSSN	WYA-FAILSAI-FYT--VL---FF---I--
Planococcus maitriensis	WP_112233804	--Y---L-VI--V-S-LIR-L-ALFGWETSESV	WYA--AILTAIVFYG--VL---FFS--I--
Planococcus massiliensis	WP_052652033	--Y---L-LI--VTS-S---I-LL-GLETSGSP	WYA--AIISA--FYA--VL---YLS-----
Planococcus plakortidis	WP_068868850	--Y-V-I-LI--V-S-LIR-L-ALFGWEA-ESV	WYA--AILTA-VFYG--VL---FFA-----
Planococcus rifietoensis	WP_058381105	--Y---I-VI--V-S-LIR-L-ALFGWETAETA	WYA--AILTAIVFYG--VL---FFA-----
Planococcus salinus	WP_123163579	--Y---LAVIWG-TS-LL----AAFGVETSDSN	WYA--TILSA--FYG--VL---FFA-----
Planococcus versutus	WP_065524314	--Y---L-VVGGLTS-LA----ALIGMET-EIP	WYA--AIVSG-IFYG--VM---FL------
Planomicrobium glaciei	KOF11925	--Y---L--IASATS-L---I-LLIGVDTAETV	WYA--AIISA--FYA--VL---FF------
Planomicrobium okeanokoites	WP_084246507	--Y---L-VI-GVTS-LL--A-ALIGVSVDSTN	WYA-FTILSAL-FYT--VL---FFS-----
Planomicrobium soli	WP_106534304	--YI--L-VIFSVTS-LI--I-AL-GLETSSAA	WYA--AILSAIVFYA--VL---YFK-----
Psychrobacillus insolitus	PZX06952	--YTV-L-V-AS-GMLLI--I--LFSLD-NN--	WYA-FA-ITATIFYG-------LCN-----
Psychrobacillus psychrodurans	WP_093496077	--YTV-I-V-AS-GMLLI--I--LFSLELNNSE	WYA-FT-ITA-IFYA--VL---LCS-----
Psychrobacillus psychrotoleran	WP_093536876	--YTV-I---ASVGMLLI--I--LFSLDLNN-V	WYA-FTLITAAIFYA--VL---LCS-----
Sporosarcina globispora	WP_053434112	--Y-L-LVVVASVDR-LIN-I--A---PLHESN	--A-ISIATAITFYA--VL---FF---L--
Terrabacteria group	WP_095248924	--Y-L-LIVVGS-ERLII--I--A-E-PLIEFN	M-A-ISIASAIIFY---VL---NFN--L--
Virgibacillus soli	KRG14599	--Y-L-LIMIFS-N-MII--I--GIGVSLVDTG	F--A-GIVSGIVFYA---L---YFQ--I--
Viridibacillus arenosi FSL R5-	ETT82613	--Y-L-LA-I-----LT-N-IVTI-G-DTDNAP	WYA-VAIVSS-IFY---VI---FWN-----
Viridibacillus arvi	KOO52463	--Y-L-LA-I-----LT-N-IVTI-G-NTDNAP	WYA-VTIVSS-IFY---VI---FLN-----

Supplemental Figure 69

A partial sequence alignment of the RDD family protein containing a two amino acid insertion (boxed) that is exclusively shared by all members belonging to the Sporosarcina clade and absent in all other bacteria.

Group	Organism	Accession	332		368
Sporosarcina clade (9/9)	Sporosarcina ureae	WP_029055098	DGEVLTLLSYPEERDYRKVSK		GIKTVQKVWYNGQLIE
	Sporosarcina newyorkensis	WP_009498481	----------A--------T-		----------S-N-V-
	Sporosarcina psychrophila	WP_067205742	------------------LT-		----------K-H-A-
	Sporosarcina koreensis	WP_082713596	--------------T---L--		-F-P------K-K-T-
	Sporosarcina pasteurii	WP_115363029	--------APH--G----LT-		-LHPTE-T-RH-K-Q-
	Bacillus sp. OxB-1	WP_041071287	----------A-------LT-		-M-PI--T--K---L-
	Filibacter sp. TB-66	WP_124069299	----------T-------LT-		-------T-H----V-
	Lysinibacillus sphaericus NCTC 11025	WP_125101214	------------------LA-		-Y-P-----HG-K---
	Lysinibacillus sphaericus LMG 22257	WP_075526940	--------APH--G----LT-		-LHSTE-I-RH-K-Q-
Other Bacteria (0/>100)	Lysinibacillus sphaericus OT4b.31	WP_010857208	----------HD-KT-K-WTR	EV	AG-P-----HD-E-V-
	Lysinibacillus sphaericus 1987	WP_036225626	----------QD-KT-K-WTR	EV	PG-P---I-HD-K-M-
	Lysinibacillus sphaericus DSM 28	WP_024364515	----------ND-KT-K-WTR	EV	PG-P---I-HD-K-V-
	Lysinibacillus endophyticus	WP_121213227	----------RD-KT-K-LTR	EM	-V--I-----Q-K-V-
	Lysinibacillus saudimassiliens	CEA03407	--K-------RD---F-RLT-	EL	PK-PI--I-HE-----
	Lysinibacillus xyleni	WP_097072635	----------RD-KT-K-LTR	EM	-V-------NQ-K-V-
	Lysinibacillus sp. SYSU K30002	WP_126659436	----------KD-KT-K-LTR	EM	NV-------HQ-EM--
	Lysinibacillus sp. BF-4	WP_036145967	--K-------RD---F-RLT-	EL	PK-PI--I-HE-----
	Lysinibacillus halotolerans	WP_122971547	----M-----KD-KT-K-LTR	EL	NV-P------Q--V--
	Lysinibacillus acetophenoni	WP_097150956	----------KD-KT-K-LTR	EL	E--A-----SQREI--
	Lysinibacillus boronitolerans	WP_016993482	----------RD-KT-K-WTR	EL	PC-P---I-HD-E---
	Lysinibacillus chungkukjangi	WP_107935248	---------FRD-K--K-LTR	DM	KV-A-----HK-SIV-
	Lysinibacillus composti	WP_124763903	--------A-KD-KT-K-LLR	EL	EK-P---I-FK-EIV-
	Lysinibacillus contaminans	WP_053581988	----------ND-KT-K-WTR	EI	PG-P---I-HD-E---
	Lysinibacillus fusiformis	WP_025115808	----------QD-KT-K-WTR	EV	PG-P---I-HD-K-M-
	Lysinibacillus jejuensis	WP_108306537	--K-I-----R----F-RLT-	QI	PQ-P--Q--H--G--D
	Lysinibacillus macroides	WP_053995503	----------RD-KT-K-WTR	EI	PG-P---I-HD-K-M-
	Lysinibacillus manganicus	WP_036188767	--------T-KD-KT-K-LTR	EL	DV-AI----NK-EIV-
	Lysinibacillus massiliensis	WP_036175314	----------KD-KI-K-LTR	EL	NV-P---M--K-K-E-
	Lysinibacillus meyeri	WP_107841141	----------RD-KT-K-WLR	EL	EL-A-----HK-E---
	Lysinibacillus odysseyi	WP_052124723	----------KD-KT-K-WTR	EI	SA-P-----AQ-M---
	Lysinibacillus parviboronicapiens	WP_107924690	----------HD-KT-K-WTR	EI	AG-P-----HD-E-V-
	Lysinibacillus sinduriensis	WP_036198896	----M-----KD-K--K-LIR	EM	KV-A-----NK-SIV-
	Lysinibacillus sp. 2017	WP_108713973	----------KD-KT-K-WVR	DL	SQ-A---I-AK-E-V-
	Lysinibacillus sp. B2A1	AVK86223	--------A-RD-KT-K-WIR	EI	PG-S-----HD-K-V-
	Lysinibacillus sp. BK089	WP_132363398	----------RD-KT-K-WTR	EI	SA-P---I-HE-K-V-
	Lysinibacillus sp. FJAT-14222	WP_053597129	----------RD-KT-K-WTR	EI	PG-P---I-HD-E-V-
	Lysinibacillus sp. FJAT-14745	WP_053483043	----------RD-KT-K-WTR	EI	SA-P-----HE-K-V-
	Lysinibacillus sp. Marseille-P	WP_106781190	----------KD-KT-K-LTR	EL	NV-SI----HK-EFV-
	Lysinibacillus sp. YLB-03	WP_118877004	----------QD-KT-K-LTR	EL	KV-A-----HK-M-L-
	Lysinibacillus sp. YR326	WP_134026033	----------RD-KT-K-WTR	EI	PG-P------E-K-V-
	Lysinibacillus sp. ZYM-1	WP_054611935	----------QD-KT-K-WTR	EV	PG-P---I-HD-K-M-
	Lysinibacillus tabacifolii	WP_108031122	----------ND-KT-K-WTR	EV	PG-P---I-HD-K-V-
	Lysinibacillus telephonicus	WP_126293963	----------KD-KT-K-LTR	EI	NV-P---I-HQ-KV--
	Lysinibacillus xylanilyticus	WP_049668828	----------RD-KT-K-WTR	EV	PV-P-----HE-K-V-
	Bacillus cecembensis	WP_057984904	----------RD-KT-K-WVR	DL	SQ-A-----HK-EF--
	Bacillus ndiopicus	WP_042478864	----------RD-KT-K-WLR	EL	-L-A-----HK-E---
	Bhargavaea beijingensis	WP_092095705	-----SFV---D-KT---LTR	AL	PG-P-----HK-G-L-
	Bhargavaea ginsengi	WP_092054823	-----SFV---D-KT---LTR	AL	PG-P-----HK-S-V-
	Caryophanon latum	WP_066463501	----IAF---HD-K--K-LTR	DL	PK-P---I-HD-A---
	Caryophanon tenue	WP_066544156	----IAF---RD-K-FK-LTR	DL	PK-PI--I-HD-A-V-
	Edaphobacillus lindanitolerans	WP_084186713	-----SFV---D-KT---LTR	EL	SE-P-----HK-A-T-
	Kurthia huakuii	WP_029500797	-------TG-A-KQ-KQITR	TL	DD-PTE---ED-G-VK
	Paenisporosarcina antarctica	WP_134209070	-----S--T-TD-KN-K-WT-	EL	PS-P-----DQ-K---
	Paenisporosarcina indica	WP_075619018	-----S--T-TD-KN-K-WT-	EL	PS-P-----DQ-H-V-
	Paenisporosarcina quisquiliarum	WP_090568361	----M---T-AD-KTFK-WT-	EL	PK-P------R-E---
	Planococcus antarcticus	WP_006830119	--------A-SD--H-K-FT-	EL	PS-P----IHG-K---
	Planococcus citreus	WP_121301239	--------A-AD-KH-K-LT-	EL	PN-P---IMHG-E-V-
	Planococcus donghaensis	WP_008431158	--------A-AD--H-K-FT-	EL	PS-P---IIHG-K---
	Planococcus faecalis	WP_071153265	--------A-AD--H-K-FT-	EL	PS-P---IVHG-K---
	Planococcus halocryophilus	WP_008496096	--------A-AD--H-K-FT-	EL	PS-P---IIHS-K---
	Planococcus halotolerans	WP_112224943	--------A-QD--H-K-FT-	DL	PS-P---IMHG-E---
	Planococcus kocurii	WP_058386035	--------A-AD--H-K-FT-	EL	PS-P---IVHG-K---
	Planococcus maitriensis	WP_112233874	--------A-AD-KH-K-LT-	EL	PD-P---IMHA-E---
	Planococcus maritimus	WP_068461133	--------A-AD-KH-K-LT-	EL	PG-P---IMHG-E---
	Planococcus massiliensis	WP_052652436	--------A-AD--H-K-FT-	EL	PS-P---ILHG---V
	Planococcus plakortidis	WP_068868594	--------A-AD-KH-K-LT-	EL	PG-P----M-G-N---
	Planococcus rifietoensis	WP_058381380	--------A-AD-KH-K-LT-	EL	PN-P---IMHG-E-M-
	Planococcus salinarum	OHX51611	--------A-QD--H-K-FT-	DL	PS-P---IMHS-K-V-
	Planococcus salinus	WP_123166361	--------A-AD--H-K-L--	QL	PS-P---IIHG-K-V-
	Planococcus versutus	WP_049695078	--------A-AD--H-K-FT-	EL	PS-P---IVHG-K---
	Planomicrobium flavidum	WP_088008228	---A------AD--Q-KRIA-	EL	QS-P---I-HR-R-M-
	Planomicrobium glaciei	WP_036805891	--------A-QD--H-K-FT-	EL	PS-P----LHG-----
	Planomicrobium okeanokoites	WP_084242301	--------A-QD--H-K-FT-	DL	PS-P---IMHG-E---
	Planomicrobium soli	WP_106534757	--------A--D--H-K-FT-	EL	PS-P----LHG-K---

Other Bacteria (0/>100)

Psychrobacillus insolitus	WP_111440262	--------T-TD-KT-K-WT- EL PS-P------R-E---
Psychrobacillus psychrodurans	WP_093495092	----M---T-AD-KTFK-WT- EL PK-P------R-E---
Psychrobacillus psychrotolerans	WP_093538137	----M---T-SD-KTFK-WT- EL PK-PI-----R-E---
Rummeliibacillus pycnus	WP_102693939	----I-----H--QA-H-RVR EL S--P-E-I-S--K---
Solibacillus isronensis	WP_079523658	----------KD-KTFK-WLR DL SL-G-----TQ---V-
Solibacillus kalamii	WP_087617429	----------KD-KTFK-WLR DL SL-G-----TQ---V-
Solibacillus silvestris	WP_014822683	----------KD-KTFK-WLR DL SL-G-----TQ---V-
Ureibacillus thermophilus	QBK27001	----------KD-KN-K-LLR QV -Q-P----I-K---M-
Ureibacillus thermosphaericus	WP_016837910	----I-----KD-KN-K-LLR EV -Q-P---II-K-----
Viridibacillus arvi	WP_053418845	--------AHH--HT---WTR EL PS-P-E-T-TA-K---

Supplemental Figure 70

A partial sequence alignment of the DEAD/DEAH box helicase protein containing a two amino acid deletion (boxed) that is exclusively shared by all members belonging to the Sporosarcina clade and absent in all other bacteria.

Group	Organism	Accession	77		124
Sporosarcina clade (8/8)	Sporosarcina ureae	WP_029054744	YKSQQEMKVKMDGLRPEMEDIQKRMKASK	ESQ	DKEEQMKLQQEMMGLY
	Sporosarcina newyorkensis	WP_040759904	-----D------A------------E--	-KN	----------------
	Sporosarcina pasteurii	WP_115363387	--T-----I---A----------L-EA-	-AG	----------------
	Sporosarcina psychrophila	WP_067205249	-----N------A----------L-EA-	-KE	Q-D--V----------
	Sporosarcina koreensis	WP_052461676	------------A-K----V---Q--EAQ	KAG	N--Q------------
	Lysinibacillus sphaericus LMG 22257	WP_075526765	--------I---A----------I-EA-	-AG	--D-------------
	Lysinibacillus sphaericus NCTC 11025	WP_125101458	------------A-K----T---Q--TA-	--G	N---------------
	Filibacter sp. TB-66	WP_124068871	-----N------A----------L-EA-	--A	S-D----I--------
Other Bacteria (0/>200)	Abyssicoccus albus	WP_123807815	----AV-RE--ALVK----RV--KV-VAT		TQ--K-EA-----A--
	Aeribacillus pallidus	WP_063387247	--D--N--L--EKVM---KEL-E-F-KA-		-QA--Q-I-K---DF-
	Auricoccus indicus	WP_077139801	----AV-RE--ALVK----RV--KV-VAT		TQ--K-EA-----A--
	Bacillus alveayuensis	WP_044894255	-KV--E---A-K-K-DEL--K--KA-		-SQ--Q-V-L---QF-
	Bacillus andreraoultii	WP_033828946	-K-KD-RE--EVMK----A--T-L--A-		T------I-----Q--
	Bacillus aquimaris	RBP08132	--N--D--G--ALIK---TEM--KI-EA-		--D--K-------L--
	Bacillus asahii	AZV42035	--T--V--S---A-K---TTL-EKI-STT		-ATKKAE--R---Q--
	Bacillus azotoformans	WP_003331625	--N--LL-S-IESIK---DK--EKI-N--		-----Q-F---IFA--
	Bacillus badius	KIL74287	-K--I--E--AVVK---DK--Q-L--A-		NP---R-I-----A--
	Bacillus bataviensis	WP_007086730	--N-MA--E---V-K---DV---K--TET		-PKKKQE--A------
	Bacillus boroniphilus JCM 2173	GAE44227	--Y--N--E--EV-K---DE---QL-GT-		-PA--R----------
	Bacillus butanolivorans	WP_053345091	--T-LS--T--N--K---TA--QKI-ET-		-PAK-KA------Q--
	Bacillus camelliae	WP_101356588	--K--V--E---V-K---TKL-AK--QA-		T----A----------
	Bacillus campisalis	WP_046523912	--T--S--D---K-K--LDE----L--T-		-EK--Q-------A--
	Bacillus caseinilyticus	WP_090890542	-----V-RE--SVIQ---KE--EKY-DA-		-QQSKQ-M-----A--
	Bacillus cavernae	WP_126864631	--A--T--G--EAFK---TA--AKI-QT-		-SVK-KE------L--
	Bacillus cecembensis	WP_057985958	-R--AV-R----EIK-K-DE---NL--VE		S----RA------QV-
	Bacillus cihuensis	WP_051405082	--T-IK--G---K-K---AA--EKI-QT-		-PVKKQE--K---A--
	Bacillus circulans	KLV25129	--N--L--S---IIK----A---KI--T-		-QK--Q---M---D--
	Bacillus coagulans	WP_035188566	F-K--I-QD--KV-K---DKL-TKI--A-		T----A----------
	Bacillus coahuilensis	WP_010172200	F-N-----G---K-K---QE---KL-ET-		-T---R-------Q--
	Bacillus cucumis	WP_101651326	--N-MV--E---V-K---DA---K--TE-		-PKKKQE--A------
	Bacillus dielmoensis	WP_042455794	--NSMA--E---K-K---DE----L--E-		-PQK-RAI-A------
	Bacillus drentensis	WP_066256618	--N-MG--E---V-K---DV--TK--TE-		-PKKKQE--A------
	Bacillus fastidiosus	WP_066234967	-----A--E--ELIK----I------EE-		-PAK-KEI----IK--
	Bacillus firmus	WP_035330840	--N-SL--E---K-K--L-K--EKL-TA-		KT---Q------FA--
	Bacillus flexus	WP_119543500	--KSTMA-QA------S---KYAGK-		-P-T-Q---K---A--
	Bacillus fordii	WP_018707532	--N--M------K------E----S-EA-		TP---Q-I-M---N--
	Bacillus fortis	WP_120074290	--N--L------K-K----E----S-QA-		NQ---Q-I-L---E--
	Bacillus freudenreichii	VEF46367	--N--L------K-K----E----S-QA-		T----Q-V-M---E--
	Bacillus fumarioli	WP_066370324	--N-MA--E---A-K---DA---K---E-		-PKKKQE--A---S--
	Bacillus galactosidilyticus	WP_064467831	--K---------MIK---D-----LR-T-		-P---K-I-----A--
	Bacillus ginsengihumi	WP_025729319	--N---------V-K---DK---QI-NA-		T----A----------
	Bacillus gottheilii	WP_066445136	--N--V--E---L-K----K---KL-ET-		NQK--Q----------
	Bacillus halosaccharovorans	WP_078433727	--N--Q--E---L-K---D----KI--A-		-QKK-LE-----V---
	Bacillus haynesii	EWH21104	--K-RT-QE--ALVK--LDA--S-L-KT-		-PVK-REI-----Q--
	Bacillus hisashii	WP_095141264	-K-K--RE--EVMK----A--A-L--A-		T----V-I-----T--
	Bacillus horikoshii	WP_082892160	--KNMLA-QK-K----A---KY-DK-		NPDD-K-M-T-L----
	Bacillus horneckiae	WP_066394409	-T--T--L---V-K----I---KI-ET-		-QK--Q------FA--
	Bacillus jeotgali	WP_079509359	--N--N--D--EL-K---DE---KL-TT-		-PA--R----------
	Bacillus massiliglaciei	WP_110929204	--N-AA--K--EAFK---TV--EKI-QTQ		-PSKKAE-----FQ--
	Bacillus massilioanorexius	WP_019240317	---YV-RD--TVMQ---KV--DKL-KA-		SP--KA-I-----K--
	Bacillus massiliosenegalensis	WP_019155656	-K-RT--L---VMK--L-V---KL-ET-		EAG--QR-----FA--
	Bacillus mediterraneensis	WP_071459817	--A--Q--E---IFK---DL--QKL-KT-		-PK--Q----------
	Bacillus mesonae	WP_066384442	--N-MA--E-----K---DA---K---E-		-PKKKQE--A------
	Bacillus methanolicus	WP_004434115	--N--A--E---L-K--LDE---KL-ST-		KP---Q------FA--
	Bacillus muralis	WP_057912329	--T-LS--T--N--K---TA--QKI-ET-		-PAK-KA------Q--
	Bacillus ndiopicus	WP_042476988	-RA-AA--S---IVK-K--E--QK--EAE		S----A------LS--
	Bacillus nealsonii	WP_016203777	--N--L------SIK----A---KI-TT-		-QK--Q---M---D--
	Bacillus niacini	WP_045523997	--N-MA--E---VMK---------L-EE-		-PKKKQE---------
	Bacillus niameyensis	WP_062106879	--N---------KMK-----V-----EA-		TP---K-V----LQ--
	Bacillus notoginsengisoli	WP_118919879	--N-LI--G---S-K---DEL--KI--E-		-AAK-KE--SQ-F---
	Bacillus novalis	WP_066088775	--N-MG--E---V-K---DV---K--TE-		-PKKKQE--A------
	Bacillus oceanisediminis	WP_019379844	--N-SL--E---K-K--L-K--QKL-TA-		KPQ--Q------F---
	Bacillus oleivorans	WP_097160644	--N--Q--I--EQ-K----E---KIRET-		S----Q------FA--
	Bacillus oleronius	WP_078111357	F-K--I-----EK-K---TA--TK--QA-		T------Y--------
	Bacillus onubensis	WP_099354446	--N-SV--E--GL-K----M--EKI-TET		-PKKKQE--T-L----
	Bacillus paralicheniformis	KFM90697	--K-RA-QE--ALVK--LDA--SKL-KT-		-PVK-REI-----Q--
	Bacillus rubiinfantis	WP_042355421	--N-MQ--E---K-K---DT---KL--ET		-PKKKQEI-A------
	Bacillus salsus	SDP33281	-----L-----AN-K---DS--EKL-NT-		-AK--KV--L---N--
	Bacillus selenatarsenatis	WP_041967479	--N--N--E--EVIK---DE--NQL-GT-		-PA--R----------
	Bacillus simplex	AMM92239	--T-LS--T--N--K---AA--QKI-ET-		-PVK-KA------Q--
	Bacillus smithii	WP_040340837	-K-----E---KIK--V-K----I-SA-		T------------Q--
	Bacillus soli	WP_066061569	--N-MV--E---V-K---DV---K--TE-		-PKKKQE--A------
	Bacillus sonorensis	WP_077736532	F-K-RV-QE--ALVK---DA--SKL-KT-		-PAK-KE------K--
	Bacillus sonorensis L12	EME74775	--K-RT-QE--ALVK---DA--SKL-KT-		-PVK-REI-----K--

Other Bacteria (0/>200)

Bacillus sporothermodurans	WP_066229749	F-K--I-----EK-K---TV--NKI-QA-	S--------------
Bacillus subterraneus	WP_044392524	-----S--E--EV-K---DE---KL-DT-	-SA--K---------
Bacillus terrae	WP_120118252	--N--L--L---K-K----E----S-EA-	TP---Q-I-M---E--
Bacillus thermoamylovorans	WP_034770066	-K-K--RE--EVMK----A--A-L--A-	T----V-I-----T--
Bacillus timonensis	WP_010677699	--N-SV--E--ELVK----Q---KIT-ET	-PKKKQD--R------
Bacillus tuaregi	WP_071395206	--T-AN--E---L-K---DI---KI-ET-	-PKD-QL--K------
Bacillus vietnamensis	WP_060671911	--N--D--G--ALIK---SA---KI-ET-	-----K-----L-M--
Bacillus vireti	WP_024029298	--N-MG--E---V-K---DAV--K--TE-	-PKKKQE--A------
Bacillus zeae	WP_119112437	--A--K--G--ELFK---DN---KL-QT-	-PK--Q-------A--
Bhargavaea beijingensis	WP_092097946	--T--A------A----------L-ET-	--D------------
Bhargavaea cecembensis	WP_008301301	--T--A------A----------L-ET-	--D------------
Bhargavaea ginsengi	WP_092055389	--T--A------A----------I-ET-	--D------------
Caldibacillus debilis	OUM83535	-K--I--Q--EII-----A----L--A-	T----A------IQ--
Chryseomicrobium excrementi	WP_100354206	-----G--L---K-K---D-----L-EA-	S-----A---------
Deinococcus radiophilus	WP_126352097	Q--ARTAA--QI-Q-K-KE--D-Y-DQ-	-R-S--QM-M--AA--
Domibacillus aminovorans	WP_063965823	-K-AV-RQ--EVVK-K-TA--D-L--E-	NP---R-I-M------
Domibacillus antri	WP_075398437	-K-AV-RQ--EIVK-K-TE--E-L--A-	NP---R-I-M------
Domibacillus enclensis	WP_045849589	-K-AL-RQ---VVK-Q-T---A-L--T-	NP---R-I-M---S--
Domibacillus epiphyticus	WP_076767250	-K-AV-RQ--EVVK-K-TE--E-L--A-	NP---R-I-M------
Domibacillus indicus	WP_046173383	-K-AV-RR---IVK-Q-M---T-L--A-	-QD--R-I-M---N--
Domibacillus iocasae	WP_069937707	-K-AV-RQ---VVK-Q-T---V-L--A-	NP---R-I-M---S--
Domibacillus mangrovi	WP_073711369	-K-AV-RQ--EVVK-K-TA--D-L--E-	NP---R-I-M------
Domibacillus robiginosus	WP_050184274	-K-AG-RY--EKVK-Q-T---T-L--A-	TP---R-I-M---S--
Domibacillus tundrae	WP_046181361	-K-AV-RQ---VVK-Q-T---A-L--T-	NP---R-I-M---S--
Edaphobacillus lindanitolerans	WP_076759179	-----A------A-K---DE---KI-ET-	--D--L---------
Falsibacillus pallidus	WP_114745486	--N--Q--S--EV-K---DK---KL--E-	SP-K-RE--A------
Halobacillus alkaliphilus	WP_089753086	F-NS-D-RG---AVK--LN-L-S-L-QAS	SQ-DKK-I-S---E--
Halobacillus halophilus	WP_014642358	F-NS-D-RG---AVK--LN-L-S-L-QAS	SQ-DKQ-I-S---E--
Halobacillus mangrovi	WP_085030664	--NS---R---EAVK---N---N-L-EA-	TQ-DKQEI-A---K--
Halobacillus massiliensis	WP_082235911	F-NS-D-R----AFK---T---N-L--AE	TQ-DKQ-I-A---A--
Halobacillus salinus	WP_079480797	--NS-A-RE---VMK-KLTE--E---KTE	--D-KQ---S------
Jeotgalibacillus campisalis	KIL48712	-RN--V--E--N--K---DE----L--TE	S------I--------
Jeotgalibacillus proteolyticus	WP_104057343	-RN--V--E--SE-K---DE--VK--KA-	N----TQ---------
Jeotgalibacillus soli	KIL52155	-RN--V--E--EV-K----E----L--TE	S------I--------
Jeotgalicoccus halophilus	WP_092599552	--N-MM-RE--KIVK----A--Q-V-VAT	TQ--K-AA--------
Jeotgalicoccus halotolerans	WP_115885087	--N-MM-RE--KIVK----A--Q-V-VAT	TQ--K-AA--------
Jeotgalicoccus marinus	WP_026865985	--N-MM-RE--KIVK----E--Q-V-VAT	TQ--K-AA-----A--
Jeotgalicoccus psychrophilus	WP_026859237	--N-MM-RE--KIVK----A--Q-V-VAT	TQ--K-AA--------
Jeotgalicoccus saudimassiliens	WP_035810904	--N-MM-RE--KIVK----E----V-VAT	TQ--K-AA--------
Kurthia huakuii	WP_029500450	--N--G--M---K----------KL--AS	S-----A---------
Kurthia massiliensis	WP_026022135	--N--G--A---K------A---KL--AS	S-----A---------
Kurthia senegalensis	WP_010307313	--N--G--A---K------E---KI--T-	S-----A---------
Kurthia sibirica	WP_109306451	--N--G------K-K---DA---KL-QT-	S-----V---------
Kurthia zopfii	WP_109348849	--N--G--S---K-K---DS---KL-QA-	S-----V---------
Listeria aquatica	WP_036073369	-A-MG-QS--AVAK--ID---A-L-RAA	T----T-I-----VV-
Listeria ivanovii	WP_014092759	-A-MG-QS--AVAK--IDE--A-L-RAT	S----A-I-----AV-
Listeria weihenstephanensis FS	EUJ36491	-A-MG-QS--AVAK--IDE--A-L-RAT	S----Q-I----AAV-
Lysinibacillus acetophenoni	WP_097150327	-RR-AL--S---IVK-K-DE--T-L--A-	T-----AI----LA--
Lysinibacillus boronitolerans	WP_016992710	-RQ--L--T---AFK----AV--K--EA-	T----TQY-----A--
Lysinibacillus chungkukjangi	WP_107934779	-R--SA--S---VVK-K-DE--E-L--AQ	T-----AI----LS--
Lysinibacillus composti	WP_124766903	-RN-SL--S---VVK-K-DE--G-L-EA-	T-----AI----LA--
Lysinibacillus contaminans	WP_053584968	-RQ--M--T---AFK--L-EV--K--EA-	T-----QY----V---
Lysinibacillus endophyticus	WP_121213131	-RQ-SL--S---VVK-KLDE--A-L-EA-	T-----TI----LT--
Lysinibacillus fusiformis	WP_069482837	-RQ--L--T---AFK----AV--K--EA-	T----TQY-----A--
Lysinibacillus halotolerans	WP_122972912	-RQ-SK--S---IVK-K--E--E-L--AQ	T-----AI----LA--
Lysinibacillus macroides	WP_053996949	-RQ--V--T---AFK----AV--K--EA-	T----TQY-----A--
Lysinibacillus manganicus	WP_036189537	-RR-AL--S---IVK-K-DEV-AKL--A-	T-----AI----LA--
Lysinibacillus massiliensis	WP_036172827	-RK-AL--S---IVK-K-DEL-D---KAT	T-----AV----IA--
Lysinibacillus meyeri	WP_107839647	-RA-AA--T---IVK-K--E---N--EAE	S----A------LS--
Lysinibacillus odysseyi	WP_036152660	-RN-AV--S---HVK-K--E--GKL-TAE	K--D--A-----LN--
Lysinibacillus parviboronicapi	WP_054768242	-RQ--I--S---AFK----EV--K--EA-	T-----QY-----A--
Lysinibacillus sinduriensis	WP_036200466	-RQ-SA--S---IVK-K-DE--E-L--AQ	T-----AI----LS--
Lysinibacillus sp. B2A1	AVK86399	-RQ--L--T---AFK----AV--K--EA-	T-----QH-----A--
Lysinibacillus sp. BK089	WP_132361598	-R---K--L---AFK----AV--K--EA-	T----TQY-----A--
Lysinibacillus sp. FJAT-14222	WP_053593999	-RQ--T--T---AFK----AV--K--EA-	T-----QH-----A--
Lysinibacillus sp. FJAT-14745	WP_053481948	-R---K--S---AFK----AV--K--EA-	T----TQY-----A--
Lysinibacillus sp. Marseille-P	WP_106781612	-RN-SL--S---IVK-K-DE--A-L--AE	T--Q--AI----LA--
Lysinibacillus sp. SYSU K30002	WP_126658788	-RQ-SR--A---VVK-K-DE--Q-L-EA-	T-----AI----LT--
Lysinibacillus sp. YLB-03	WP_118876777	-RR-SQ--S---VVK-K-DE--E-L--AQ	T-----AI----L---
Lysinibacillus sp. YR326	WP_134022897	-R---K--M---AFK----AV--K--EA-	T----TQY-----A--
Lysinibacillus sp. ZYM-1	WP_054609422	-RQ--L--T---AFK----AV--K--EA-	T----TQH-----A--
Lysinibacillus telephonicus	WP_126293321	-RQ-SL--S---VVK-K-DE--Q-L--AS	T-----AI-----T--
Lysinibacillus xylanilyticus	WP_049663397	-R---K--M---AFK----AV--K--EA-	T----TQY-----A--
Lysinibacillus xyleni	WP_097074343	-RQ-SL--S---VVK-K-DE--A-L-EA-	T-----AI----LT--

Other Bacteria (0/>200)

Mycobacteroides abscessus subs	SHQ77487	-----Q--E--EA-K--LDT--AKI-NT-	-SK--Q---------
Nosocomiicoccus massiliensis	WP_040928285	--N-ML-RE--KIVK-------R---VAT	TQ--K-AV-----A--
Oceanobacillus senegalensis	WP_085992930	-NSHH--E--KVIK---DAL-EKY-DN-	-T-SRT-M-----Q--
Paenibacillus agaridevorans	WP_108995516	----MT--G--AA-Q--LKELEAKY-DK-	-ADSLT-K-----A--
Paenibacillus alvei	SYX83831	-RN--K--Q---V-Q--LKLL-DAY-DK-	-A-SKA-M-----Q--
Paenibacillus paeoniae	WP_116047969	-----S-----AA-Q--LKVLEAKY-DK-	-QDTLA-K---T-E--
Paenibacillus pinisoli	WP_120113925	-----A--T--AA-Q--LKALEAKY-DK-	-QDALV-K---T-E--
Paenibacillus prosopidis	WP_114381685	-R--HQ-RQ--TV-Q--LKQL-EKF-GKS	-T-SKQ-----T-Q--
Paenisporosarcina antarctica	WP_134208555	--T--G--L---SMK---DE---KI----	-----TI--K------
Paenisporosarcina indica	WP_075619496	--N--G-----A-K---DA---KI--A-	----------------
Planococcus antarcticus	WP_006830218	--R--G-------M----------L-ET-	----------------
Planococcus donghaensis	WP_008432374	--R--G------AM----------L-ET-	----------------
Planococcus halocryophilus	WP_008498799	--R--G------AM----------I-ET-	----------------
Planococcus halotolerans	WP_112224142	--R--G------AM----------L-GT-	----------------
Planococcus maitriensis	WP_112233042	--R--G------KM----------L--T-	----------------
Planococcus maritimus	WP_068460368	--R--G------KM----------L-ET-	E---------------
Planococcus massiliensis	CEG24003	--R--G------K-----------L-QT-	-------I--------
Planococcus plakortidis	WP_068872035	--R--G------KM----------L--T-	----------------
Planococcus salinarum	TAA71848	--R--G------KM----------L-ET-	----------Q-----
Planococcus salinus	WP_123166246	--R--G------AM----------L-ET-	----------------
Planococcus versutus	WP_049695022	--R--G------VM----------I-ET-	----------------
Planomicrobium flavidum	WP_088006142	--R--A------AM----------L--T-	-R-----I--------
Planomicrobium glaciei	SDH05143	--R--G------K-----------L-ET-	-------I--------
Planomicrobium soli	PSL28816	--R--G------RM----------L-ET-	-------I--------
Pontibacillus chungwhensis	WP_036786096	-----Q-----A-MK---D---A---EAT	P-DKQ-M---L-A--
Pontibacillus halophilus	WP_036770312	--K--D--S--EVMK-------G-L-GAS	SE--RKEA---LVQ--
Pontibacillus marinus	WP_027447564	--K--V--Q--AKIK---D-V-N---E--	TQ--KQEI-K---A--
Pontibacillus yanchengensis	WP_036823195	--K--L--T--TAIK---D-V-N-L-EAT	SQ-DKQQI-N---E--
Psychrobacillus insolitus	WP_111439899	--N--G------K-K---DS---KL-ET-	V-----A---------
Psychrobacillus psychrotoleran	SFQ58245	--N--G--Q---K-K---D-----L-ET-	---Q--A---------
Quasibacillus thermotolerans	WP_039238348	-K--A-RE--EIVK----K---KL-EA-	TQ---R-I-----M--
Rhizophagus irregularis	PKC52000	-RK-AL--S---IVK-K-DEL-D---KAT	T-----AV----IA--
Rhodococcus qingshengii	WP_133367061	-RN-MN--E---VMK----A---K--DE-	-PKKKQEM---L----
Rummeliibacillus pycnus	WP_102694441	--N--G--E---K-K---DE---KL-EA-	T-----A---------
Rummeliibacillus stabekisii	WP_066790304	--N--G--E---Q-K---DE---KL--T-	S-----A---------
Salinicoccus albus	WP_020007227	--N-MM-RE--KIVK----E---KV-VAT	TQ--K-EA--------
Salinicoccus alkaliphilus	WP_072710928	--N-MM-RE--KIVK----E----V-VAT	TQ--K-EA--------
Salinicoccus carnicancri	WP_017549591	--N-MM-RE--KIVK----E---KT-VAT	TQ--K-EA-----A--
Salinicoccus halodurans	WP_046790954	--N-MM-RE--KIVK----E---KT-VAT	TQ--K-EA-----A--
Salinicoccus kekensis	WP_097041095	--N-MM-RE--KIVK----E----V-VAT	TQ--K-EA--------
Salinicoccus luteus	WP_031545215	--N-MM-RE--KIVK----E---KT-VAT	TQ--K-EA-----A--
Salinicoccus qingdaonensis	WP_092987738	--N-MM-RE--KIVK----A---KV-VAT	TQ--K-EA--------
Salinicoccus roseus	WP_040105935	--N-MM-RE--KIVK----E---KT-VAT	TQ--K-EA-----A--
Salinicoccus sediminis	WP_046514224	--N-MM-RE--KIVK----E---KT-VAT	TQ--K-EA-----A--
Salipaludibacillus agaradhaere	WP_078579953	-NN-LT-RE--AV-K---DEV--KY-DK-	NR-D-Q-M-----A--
Scopulibacillus darangshiensis	WP_132743828	--N--Q-R----LIK---DE-K----EE-	-PAV-R-M----LE--
Solibacillus isronensis	WP_079526040	-R--AT------LV--Q-----T-L--A-	TP--R-TV-----S--
Sporolactobacillus inulinus	WP_010027175	-FDT-Q---K-K--LSQ-----ADTA	NP--KQ-I-M---A--
Sporolactobacillus inulinus CA	KLI03658	-FDT-Q---K-K--LSQ-----ADTA	NP--KQ-I-M---A--
Sporolactobacillus nakayamae	SFG89279	-IR-FDV-K---K-K---T------S-AQ	-PN-KQ-I-M------
Sporolactobacillus terrae	WP_028977723	-FDT-Q---K-K--LSQ-----ADAA	SP--KQ---M---A--
Sporosarcina globispora	WP_053435813	--N-NL--E---K-K--L-K--QKL-T--	KPQ--Q------FS--
Sporosarcina sp. EUR3 2.2.2	WP_024536770	--N--G--I---AMK---DE---KI--T-	--A--TQ---------
Staphylococcus stepanovicii	WP_095085564	F---KK-QF--KQIK--IDE-N-K---T-	-EK-RA-YS--L-A--
Staphylococcus vitulinus	WP_016912063	F-G-KK-QF--KQ-K--IDE-N-K---A-	-DQ-RTQYS----A--
Tetzosporium hominis	WP_094943212	--A--G--Q---K-K---D-----L-EA-	S--D--A---------
Thalassobacillus cyri	SEA98918	--N--H--E--SVMQ-D-KI--EKL-GTD	KR---L-------E--
Thalassobacillus devorans	WP_051353151	--N--H--E--SVMQ---KV--EKL-ETD	KR---L-------E--
Ureibacillus thermophilus	QBK24598	-RQ-SI--S---IIK-K-DE--EKL-KAT	T------I----LA--
Ureibacillus thermosphaericus	WP_016838326	-RQ-SI--S---VIK-K-DE--E-L-KAT	T------I----LA--
Vibrio vulnificus	WP_133349297	--T-LS--T--S--K---TA--QKI-ET-	-PAK-KA------Q--
Virgibacillus ndiopensis	WP_099157533	-NSR---K---VI----NE-KEKY-DK-	-ADSKL-M----TQ--
Virgibacillus soli	WP_057988636	--K---------IIK---D-----LR-T-	-P---K-I-----A--
Viridibacillus arenosi FSL R5-	ETT82862	--H--G--T---K-K---D----KL--A-	T-----A---------
Viridibacillus arvi	KO047393	--H--G--T---K-K---D----KL--A-	T-----A---------

Supplemental Figure 71

A partial sequence alignment of the membrane protein insase YidC containing a three amino acid insertion (boxed) that is exclusively shared by all members belonging to the Sporosarcina clade and absent in all other bacteria.

Group	Organism	Accession	25		78
**Sporosarcina clade (9/9)**	Sporosarcina newyorkensis	WP_009765631	MRNQVAMFSLMIFLTLISFSMV	VAHLSD	VAGFSKFFVIPTLFLFAAVQVGLQLY
	Sporosarcina psychrophila	WP_067211550	--G-IM---------IM-----	M-YQA-	------YLI----L------------
	Sporosarcina koreensis	WP_060208971	--D--M----------V-----	--YEAG	------YLI--MVM------------
	Sporosarcina ureae	WP_083035331	--S-------------------	F---N-	--------I-----------------
	Sporosarcina pasteurii	WP_115360350	--A--I---M------T--AI-	L-SNA-	-I-----Y---VIL------------
	Lysinibacillus sphaericus LMG 22257	WP_075528534	--A--I---M------T---I-	L-SNA-	-I-----Y---VIL------------
	Lysinibacillus sphaericus NCTC 11025	WP_039044113	--G--M-------M--V--TF-	--YQQG	-E--T-YL---V--------------
	Filibacter sp. TB-66	WP_124071047	----IM--A----------T--	M-YN-G	-----EY----I-L------------
	Bacillus sp. OxB-1	WP_041073825	--G--T----------V--AI-	T-YQNE	IT---SY-I--IIL-------T----
**Other Bacteria (0/>200)**	Abyssicoccus albus	WP_123807662	--M--TV-G----F---A-ML-		AG-VA-A----IIL-L--L--I--F-
	Anoxybacillus amylolyticus	WP_131874107	--H-MIA-V---L----A-AA-	G	YEE--HW--V-FIL-L-----AF---
	Anoxybacillus ayderensis	WP_085788176	-KH-IVS-T--------A-FA-	G	YE---AW-SV-FIL-L-V---IF---
	Anoxybacillus flavithermus	WP_004891266	-KH-IIS-T--------A-FA-	G	YE---AW-SV-FIL-L-V---IF---
	Anoxybacillus geothermalis	WP_044742659	--H-LIA-V---L------AA-	G	YEE--HW--V-FIL-L-----AF---
	Anoxybacillus suryakundensis	WP_055440204	-KH-IIS-T--------A-FA-	G	YE---AW-AV-FIL-L-V---IF---
	Anoxybacillus vitaminiphilus	WP_111643594	-KH--IS-A-------VA-FAI	G	YDK--PW-SV-FIL-L-I----F---
	Aureibacillus halotolerans	WP_133579165	-KH--IT-----L--IV--IA-	A	YEE--RD--V-FIL-L-V---AY---
	Auricoccus indicus	WP_077140040	--M--TV-G----F---A-ML-		AG-VA-A----IIL-L--L--I--F-
	Bacillus abyssalis	WP_078409353	-KM--IS-A--------A-FA-	G	NEQ--GW-TV-FII-L-V--LLF---
	Bacillus acanthi	WP_108669823	--Y--IT-A--------A-VA-	G	YD---HW-IV-FIV-L-VI--IF---
	Bacillus acidicola	WP_066264867	-KF--VS-V------V-A-AA-		Y-D---W-T--FI--L-V---AF-I-
	Bacillus aciditolerans	WP_121449116	-KQH-IS-A------I-A-IA-	G	SEQ--PW--V-FIL-L-VI--I----
	Bacillus alveayuensis	WP_044893890	-KH--IS-A--------A-FA-	G	YEE--PW-T--FIL-L-----VF---
	Bacillus andreraoultii	WP_033829337	-QA--IT-A------I---IA-	G	SGS--AK-I--FIL-L-V----F---
	Bacillus aquimaris	WP_044337517	--M--TS-M-------VA-IA-		AGD-D-Y-IV-FIL-L-V--LIF---
	Bacillus aryabhattai	WP_098621486	-KL--IS-AM-L-----A-FA-		YQE--GWYIM-VIV-L-V---IF---
	Bacillus asahii	WP_119116181	-KH--IT-AM---F---A-AA-		GFD---W-IK-VIL-L-V---IF---
	Bacillus badius	WP_041099760	--I---T-T----F-VV--VA-		A-D---W--V-FIL-L-V----F---
	Bacillus bataviensis	WP_007086230	--Y-IIS-A--------A-AC-	A	IK---SW-TV-FIL-L-----VF--F
	Bacillus butanolivorans	WP_053345205	-KH--IS-T----F---A-AA-	G	IE---HW-IK-VIL-L-----VF---
	Bacillus camelliae	WP_101352581	-KY--IS-I--------A-GA-		Y-D---W-T--IIL-L-I---IF---
	Bacillus campisalis	WP_046522259	--Y--IT---------MA-AA-	G	FGD--SW-IA-FII-L-V---IF---
	Bacillus cavernae	WP_126864345	-KH--VT-TM---F---A-LA-	A	YD---HW-IR-FIL-L-V---AF---
	Bacillus cecembensis	WP_057985724	--K--VN-AI-----FVA-AS-		----PAT-IA-VIL-L-GI--V----
	Bacillus dielmoensis	WP_042456072	--Y--VS---------VA-GA-		MK- -AW-TV-FII-L-V---VF---
	Bacillus firmus	WP_035328686	--H--IT-A------IVA-VAA	G	YE---GW-IV-FILIL-L---IF---
	Bacillus fordii	WP_018705266	--H--IS-AIS-----VA-GA-		A---D-W----FIL-L-L---IF---
	Bacillus fortis	WP_120073944	--H--IS-VIS-----VA-GA-		A---D-W----FIL-L-L---IF---
	Bacillus gottheilii	WP_066446977	--Y-IIT-A-------VA-VA-	G	YE---AS-AV-FILVL-F---AF---
	Bacillus halmapalus	WP_078381026	-KY--VS-T------VLA-LA-		G-DM--L-IV-FII-L-V--CIF---
	Bacillus hisashii	WP_095141698	-QV--IT-A------IVA-IA-	G	TGD--VK-I--FIL-L-VI---F---
	Bacillus horikoshii	WP_064101235	-KY--VS-V--------A-LA-		G-EM--Y-IV-FII-L-V--CIF---
	Bacillus horneckiae	WP_066393277	--H--IS-A--------A-VA-	A	YEE--GW--V-FIL-L-G---IF--F
	Bacillus indicus	WP_029285089	-KY--VT-A------VVA-VAI	G	YD-IAEW-K--FIL-L-V---IF---
	Bacillus jeotgali	WP_079508310	--Y-IVS-A------IVA-VA-	G	YE---GW-TV-FIL-L-V---IF---
	Bacillus korlensis	WP_066052844	--Y-IVS--M------VA-IA-	G	YGD--AW-IV-FIL-L-I---VF---
	Bacillus kribbensis	WP_026692164	--Y-IIS-A--------A-GA-	Y	YE---HW--V-FIM-L--I--VF--F
	Bacillus lentus	WP_066140024	--Y--IS-AIS-----VA-GA-		Y---D-W----VIL-L-V---IF---
	Bacillus loiseleuriae	WP_049680657	-KH--IT--M---F---A-A--	A	METI-RW-I--VIL-L-VI--IF---
	Bacillus marisflavi	WP_048004170	--M-LTA-M--------A-IA-		AGD-D-Y--L-FIL-L-V--LVF---
	Bacillus methanolicus	WP_003348816	--E-IIS-T----F---A-GA-	A	YGK--GW-T--F-I-L-V---LF---
	Bacillus muralis	WP_064466385	-KH--VT-V----F-V---IA-	A	MED--HW-IK-VIL-L-VI--VF---
	Bacillus ndiopicus	WP_042471599	--S--TQ-AI--L---VA-ASA		--D-APN--K-FVL-L-G---VQ---
	Bacillus niacini	WP_034674194	--H--VS-A--------A-AA-	A	MK---AW-IV-FII-L-I---IF---
	Bacillus niameyensis	WP_062105189	--H--IS-AIS-----CA-GS-		Y---D-W--V-FIL-L-I---IF---
	Bacillus notoginsengisoli	WP_118922846	--Y-IIS--M-L---F-A-GA-		GV---GW-TV-LIM-L-S---IF--F
	Bacillus novalis	WP_066084992	--Y--VS----------A-AS-	A	MK--TSW-TV-FIL-L-V---VF--F
	Bacillus oceanisediminis	WP_110067833	-KH--IT-AM-----IVA-VAA	G	YE---AW-IV-FILIL-V---IF---
	Bacillus onubensis	WP_099353569	-KHH-IS-A------I-A-IA-	G	YGE--PW--V-FIL-L-VI--I----
	Bacillus persicus	WP_090747596	--Y--VT-T--------A-AA-	G	FGDL--W-TV-FIL-L-V---IF---
	Bacillus praedii	WP_057768195	-KH--IT-A--------A-VA-	A	YE--TGW-IV-FIL-L-V---IF--F
	Bacillus psychrosaccharolyticu	WP_040375478	-KH--VT-V----F---A-AA-	A	IE-I-PW-IR---L-L-----IF---
	Bacillus selenatarsenatis	WP_041965546	--Y-IVS-A-------VA-VA-	G	YD---DW-TV-FIL-L-VI--IF---
	Bacillus simplex	WP_061462296	-KH--IT-I----F--LA-AA-	G	IE---HW-IK-VIL-L-VI--VF---
	Bacillus sinesaloumensis	WP_077617695	-KH--IT-A------I-A-IA-	G	YGE--PYV-V-LIL-L-VI--I----
	Bacillus smithii	AKP46730	--M-IVS-T--L-----A-VA-		G--L--W--L-FVL-L-CI--LF-I-
	Bacillus soli	WP_066066404	--Y--VS---------VA-AA-	A	MK--TAW-TV-FIM-L-V---IF---
	Bacillus sporothermodurans	WP_066232083	-KY--VS-V---L----A-GA-		A-DL--W-T--VIL-M-V---IF---
	Bacillus subterraneus	WP_044391539	--Y-IVS-T------IV--VA-	G	YE---GW-TV-FII-L-VI--IF---
	Bacillus terrae	WP_120115279	--H--IS-AIS-----VA-GA-		A---D-W----FIL-L-L---IF---
	Bacillus testis	WP_050614657	-KY--IS---------V--IA-	G	YD---RW-IL--IL-L-V---IF---
	Bacillus thermoamylovorans	WP_034769538	-QV--IT-A------IVA-IA-	G	IGD--VK-I--FIL-L-VI---F---
	Bacillus timonensis	WP_010282766	-KHH-IS-A------I-A-VA-	G	SEK--PW--V-FII-L-VI--I----
	Bacillus tuaregi	WP_071395463	--H--IT-A--------A-VA-	G	YD---NW-T--FIL-L-G---AY---

Other Bacteria (0/>200)

Bacillus vietnamensis	WP_060669953	-KM-LTS-M-------VA-IA-		AGD-D-Y--V-FIL-L-V--LVF---
Bacillus vireti	WP_024029156	--Y--VS----------A-AS-	A	IK--TSW-TV-FIL-L-----VF--F
Bacillus wudalianchiensis	WP_065411569	--I---T-T----F-VV--VA-		A-D---W----FIL-L-V----F---
Bacillus zeae	WP_119111442	--Y--IT-A-------V--VA-	G	YGE--GW-IV-FIL-L-V---IF---
Bhargavaea beijingensis	WP_092096247	--S-LM--A----------AA-		Q----AY--V-VIL-L-TI-------
Bhargavaea cecembensis	WP_008297381	--S-LM--A----M-----AA-		Q----AY----VVL-L----------
Bhargavaea ginsengi	WP_092049159	--S-LM--A----M-----AA-		Q----AY----VVL-L----------
Caryophanon latum	OCS91398	-KM--VT-A--------A-IA-		N----PYY-T-IIL---GI--V----
Caryophanon tenue	WP_066543186	--M--VT-A--------A-LA-		G----FY--A-VIL-L-G---V----
Domibacillus aminovorans	WP_063964983	--M--VT-TM---F-V-A-AA-		G-----W-IL-FVL-L-I---VF---
Domibacillus antri	WP_075399780	--M--VT-T----F-V-A-AA-		G-----W-I--FVL-L-V---AF---
Domibacillus enclensis	WP_045851471	--M--IT-TM---F-V-A-AA-		G-----W-IV-FVL-L-V---IF---
Domibacillus epiphyticus	WP_076762817	--M--IT-T----F-VLA-AA-		G-----W-I--FVL-L-C---AF---
Domibacillus indicus	WP_046175156	--M--IT-TM---F-V-A-AA-		G-----W-IV-FVL-L-V---IF---
Domibacillus iocasae	WP_069939503	--M--IT-TM---F-V-A-AA-		G-----W-IV-FVL-L-I---IF---
Domibacillus mangrovi	WP_073711109	--M--VT-TM---F-V-A-AA-		G-----W-IL-FVL-L-I---IF---
Domibacillus robiginosus	WP_050181552	--M--IT-TM---F-V-A-VA-		G-----W-I--FVL-L-V---IF---
Domibacillus tundrae	WP_046179674	--M--VT-TM---F-V-A-AA-		G-----W-IV-FVL-L-V---IF---
Edaphobacillus lindanitolerans	WP_076756615	--G-LM--A----M-----TA-		Q----PL----VIL-L----------
Falsibacillus pallidus	WP_114743804	-KHH-VS-A-------VA-AA-		Y-DM--W-IV-FIL-L-----I----
Geobacillus icigianus	WP_033022149	--H-MIA-V---L---VA-AA-	G	YEE--HW--V-FIL-L-----AF---
Geobacillus jurassicus	WP_066233371	--H--IA-V---L----A-AA-	G	YEE--HW--V-FIL-L-----AF---
Geobacillus kaustophilus	WP_044731837	--H-MIA-V---L----A-AA-	G	YEE--HW--V-FIL-L-----AF---
Geobacillus lituanicus	WP_094239853	--H-MIA-V---L----A-AA-	G	YEE--HW--V-FIL-L-----AF---
Geobacillus stearothermophilus	KFL15888	--H-LIA-V---L------AA-	G	YEE--HW----FIL-L-G---AF---
Geobacillus thermoleovorans	WP_068895349	--H-MIA-V---L----A-AA-	G	YEE--HW--V-FIL-L-----AF---
Gracilibacillus boraciitoleran	WP_035721654	-KQ--IT-A---IF-I-A-A--		LS-LD-L-L--I-LVL------F---
Gracilibacillus dipsosauri	WP_054859565	-KQ--IT-A---IF-I-A-G--		M-EM--L----VIIVL------F---
Gracilibacillus kekensis	WP_073200571	-KQ--IT-A---IF-I-A-A--		AS-LD-L----I-LVL-GI---F---
Gracilibacillus lacisalsi	WP_018932796	-KQ--IT-A---IF-IVA-A--		E-ELD-L----V-LVL--I---F-I-
Gracilibacillus massiliensis	WP_058306995	-KQ--IT-A---IF-I-A-A--		TS-LD-L----I-LVL-----AF---
Gracilibacillus orientalis	WP_091483963	-KQ--IT-V---VF-IVA-A-I		E-ELDRL----V-LVL--I---F-V-
Gracilibacillus ureilyticus	WP_089741921	-KQ--IT-A---IF-I-A----		I-ELN-L----VI-VL------F---
Halobacillus alkaliphilus	WP_089751920	-KQ--IT-V---LF-FVA-G--		IMEVNSY------IIL-L---VF---
Halobacillus dabanensis	WP_075036518	-KQ--LT-A---LF---A-G--		IMEINSY------IIL-V---AF---
Halobacillus halophilus	WP_014643079	-KQ--IT-V---LF-FVA-G--		IMEVNSY------IIL-I---AF---
Halobacillus hunanensis	WP_079529723	-KQ--IS-VM--LF-IVA----		LLEVNSY-L----IIL-V---LF---
Halobacillus kuroshimensis	WP_027954078	-KH--LT-A---LF---A-G--		-MEVNSY-I----IIL-I---AF---
Halobacillus litoralis	WP_128522894	-KQ--IS-A---LF-F-A-G--		IMEI-S-------IIL-V---AF---
Halobacillus mangrovi	WP_085030066	-KQ--IS-V---LF---A-AL-		MFEVNSY-I----IIL-V---AF---
Halobacillus salinus	WP_079480408	-KH--IS-VM--IF-FVA-G--		MFEVNS--I----I-L-V---AF---
Halobacillus trueperi	WP_115824834	-KQ--LT-G---LF-M-A-G--		IFEVNSY-I----IIL-V---AF---
Jeotgalibacillus alimentarius	WP_041122287	--G--TS-AM---M-F-A-II-		L-D-D--YAF-I-LVL--A--V----
Jeotgalibacillus campisalis	WP_041057235	--KH-TS-A----V---A-I--		L---D-T--Y-V-LAL--L--V----
Jeotgalibacillus malaysiensis	WP_039808668	--G--TS-AM---M-F-A-II-		I-D-D--YAF-I-LVL--A--V----
Jeotgalibacillus proteolyticus	WP_104056201	--QH-TS-A----M---A-IA-		M---D-IY-Y-I-LVL--M--V----
Jeotgalibacillus salarius	WP_134382085	--G--TS-AM---M-F-A-II-		I-D-D--YAF-V-LVL-GA--V----
Jeotgalibacillus soli	WP_041088832	--AH-TS-AM-------A-I--		MS--D-YY-F-V-L-L-GI--V----
Kurthia sibirica	WP_109304418	-----TA-AI---F-F-A-AL-		M----PTL-V-VIL-L-V---I----
Kurthia zopfii	WP_109349933	--K---A-AM--LF-FVA-AA-		Q---PAN-IG-VIL-L-G---A--F-
Lentibacillus amyloliquefacien	WP_068440739	-QK--IT-A---LF-I-A-VI-	A	TGVMDSM-IV-V-IIL-LI---F---
Lentibacillus salicampi	WP_135108179	-QK-LIT-A---VF-I-A-VV-	A	AGVMDSM----I-LIL-V----F-F-
Lysinibacillus acetophenoni	WP_097147898	--K--VN-AI--LF-F-A-AV-		--D--PY-IK-FIL-L-----V----
Lysinibacillus chungkukjangi	WP_107934922	--K--IN-AI-----FLA-AV-		--D--AYYIK-VIL-L-G---V----
Lysinibacillus composti	WP_124764125	--K--VN-AI-----FVA-AS-		A-----Y--V-VIL-L-----V----
Lysinibacillus contaminans	WP_053584476	--K--IN-AI-----F-A-AV-		A-D--PYLIA-FVL-L-GI--V----
Lysinibacillus endophyticus	WP_121212871	-KK--VN-AI-----FLA-AV-		--D--PYLIK-FIL-L-GI--V----
Lysinibacillus halotolerans	WP_122970245	--K--IN-AI-----FLA-TV-		--D--AY-IK-FIL-L-----V----
Lysinibacillus macroides	WP_053993803	--K--IN-AI-----F-A-G-A		A-----YLIF-FIL-L-G---V----
Lysinibacillus manganicus	WP_036181841	--K--VN-AI--LF-F-A-AV-		--D--PY-IK-VIL-L-G---A----
Lysinibacillus mangiferihumi	WP_107895661	--K--IN-AI-----F-A-AV-		A-----Y-IA-LVL-L-C---V----
Lysinibacillus massiliensis	WP_036171955	--K--VN-AI---F-F-A-AV-		I-D--PY-IK-IIL-M-G---V----
Lysinibacillus meyeri	WP_107839287	--S--TQ-A---L--FVA-AAA		L-D--PN--K-VIL-L-GI--VQ---
Lysinibacillus odysseyi	WP_036155998	--K--MQ-AI--L--FVA-AA-		--D--PN-IK-LIL-L-G---VQ---
Lysinibacillus parviboronicapi	WP_107923188	--K--IN-AI-----F-A-AV-		A-D---YLIF-FVL-L-G---V----
Lysinibacillus saudimassiliens	CEA02511	-QR--TN-AI-------A-AS-		M-----TLIL-LIL-L-GI--V----
Lysinibacillus xylanilyticus	WP_068983280	--K--VN-AI---F-F-A-AV-		A-D---YLIF-LIL-L-G---V----
Lysinibacillus xyleni	WP_097072021	-KK--VN-AI-----FLA-AV-		--D--PYLIK-FIL-L-GI--V----
Macrococcus bohemicus	WP_111745670	--M--TT-AI-------A-A--		A--L--E----AVL-M-LI--I--F-
Macrococcus bovicus	WP_133451193	--Q--TT-AV-----F-A-G--		A--LD-AV-L-L-I-L-F---I--FF
Macrococcus brunensis	WP_133431062	--Q--TT-AV-----F-A-A--		A--LD-AI-L-I-I-L-FI--I--FF
Macrococcus canis	WP_086042268	--M--TT-AI-----FVA-A--		A--L--E----AVL-L-LI--I--F-
Macrococcus caseolyticus	WP_086038501	--M--TT-AI-----FVA-A--		A--L--E----AVL-L-LI--I--F-
Macrococcus hajekii	WP_133429398	--Q--TT-GI-----F-A-G--		A--LD-AI-L-I-V-L-FI--I--FF

Other Bacteria (0/>200)

Macrococcus lamae	WP_133444379	--M--TT-AV-----F-A-A--		AG-LHRD----VIS-L-LI--I--FF
Melghiribacillus thermohalophi	WP_132370332	-KY--IT-AM--IF-I-A-GL-		AG----Y-T--VILVL----FLF---
Mycobacteroides abscessus subs	SHT04123	--Y-IVS--M-----FVA-IA-	G	YGD--AW-IV-FIL-L-V---VF---
Oceanobacillus bengalensis	WP_121130280	-KH--IT-V---VF---A-LA-		ASDLD-I----LILIL-V----F-F-
Oceanobacillus damuensis	WP_067728248	--K-IIT-A----F-M-A-FA-		MM-T-ST----LILIL-V----F-F-
Oceanobacillus halophilus	WP_121202451	-KH--IT-V---AF---A-LV-		A-EL-RT----FIL-L-V----F-F-
Oceanobacillus massiliensis	WP_010649527	--K-IVT-A----F---A-FA-		LM-V-ST----LILIL-L----F-F-
Oceanobacillus rekensis	WP_087973047	--K-IIT-A----F-M-A-FA-		ML-V-ST----LILIL-I----F-F-
Oceanobacillus senegalensis	WP_085992501	-KH-LVS-A---IF--VA-LV-		G--MDRT----FI-IL-V----F-F-
Paenisporosarcina indica	WP_075617439	--GH-TT-AI-------A-T--		Y----AY--V-IIL-L-----I----
Parageobacillus thermoglucosid	WP_064551146	-KH--IS-V---L----A-FA-	G	YDK--HW-SV-FI--L-V---MF---
Parageobacillus toebii	WP_062678287	-KH--IS-V---L------AA-	G	YDK--HW-S--FIL-L-V---AF---
Piscibacillus halophilus	WP_091775474	-KHH-LT-AM--AF---A-GL-		I--V-PM-T--VILIL-----LF---
Planococcus antarcticus	WP_006828863	--GHL-T-AI-------A-T--		A----VYLIV-IIL-L-GI--V----
Planococcus citreus	WP_121297785	--G-LTS-AM-------A-TV-		A-D--NYLIV-IIL-L--I--V----
Planococcus donghaensis	WP_008429190	--GHL-T-AI-------A-T--		A----VYLIV-IIL-L-GI--V----
Planococcus halocryophilus	WP_065528119	--GNL-T-AI-------A-T--		A----VYLIV-IIL-L-GI--V----
Planococcus maritimus	WP_068463019	--G-LTS-A--------A-TL-		A----TYLIV-IIL-L-----V----
Planococcus massiliensis	WP_052651070	--G-LTS-AI-------A-TA-		A-D--PYLIT-IIL-L-----V----
Planococcus rifietoensis	WP_058383483	--S-LTS-AM-------A-TV-		A-D--NYLIV-IIL-L--I--V----
Planococcus salinarum	TAA72104	--Q-LTS-AI-------A-T--		A----TYLIV-IIL-L--I--V----
Planococcus salinus	WP_123164405	--G-LTS-AM-------A-TA-		A-D--PYLIV-IIL-L-----V----
Planococcus versutus	WP_049693375	--GHLGT-AI-------A-T--		A----VYL-A-I-L-M--I--V----
Planomicrobium flavidum	WP_088005798	--G---T-A-------VA-TA-		A-D--AYY-V--IL-L----------
Planomicrobium glaciei	WP_036806983	----LTS-AM-------A-TT-		A-D--PYLIT-IIL-L-----V----
Planomicrobium okeanokoites	WP_084244935	--H-LTS-AI-------A-T--		A-D--AYLIV-IIL-L-----V----
Planomicrobium soli	WP_106534925	--HMLTS-AM-------A-TA-		A----TYLIV-IIL-L-G---V----
Pontibacillus chungwhensis	WP_036780875	-KY-LIT-A---IF--VA-G--		I-EYNPM-T--VII-L-C----F---
Pontibacillus halophilus	WP_026799646	-KY--VT-A----F--VA----	A	FGD-GTQ----VIL-L-G---AF---
Pontibacillus litoralis	WP_036832253	-KF-LVT-AC--VF-----GL-		I-E-NPM-T--IIL-L-C----F---
Pontibacillus marinus	WP_027448158	-KHHLIT-AS--VF---A-G--		IGD-HRL-T--VII-M-V---AF---
Pontibacillus yanchengensis	WP_036822861	-KY-LVT-AA--VF--V--GL-		IGE-HPM-T--VII-L-C---AF---
Psychrobacillus insolitus	WP_111438677	--G--TT-AI-------A--A-		A----EY-IV-I-L-L--I--V----
Psychrobacillus psychrotoleran	WP_093535382	-----TT-GI---------TA-		A----VYLIV-VIL-L--I--V----
Quasibacillus thermotolerans	WP_039231899	--I---T-T----F-V---AA-		A-----W----VIL-L-VI---F---
Rhodococcus qingshengii	WP_133369495	--H--VS-A--------A-AV-	A	AK---AW-IV-FIL-L-I---IF---
Rummeliibacillus pycnus	WP_102692780	-----TV-AI-----F-A-AA-		Q----AYL---IIL---G--------
Rummeliibacillus stabekisii	WP_066785118	-----TV-AI-----FVA-AT-		Q----A-L-V-LIL---G---A----
Salimicrobium jeotgali	WP_008589709	-VQ--IT-A---LF---A----		ILDI-SY-IK---L-L-G---AF--F
Salinibacillus kushneri	WP_093136519	-KY--IT-AM--AF-I-A-GL-		-GD---YL-V-IILVL-V---LF---
Saliterribacillus persicus	WP_114352819	-KR--IT-A---VF-I-A-G--		-S-LD-L-I--IILVL------F---
Sediminibacillus albus	WP_093210989	-KQ--IT-V---LF-V-A-G--		IYELD-L------LIL-V----F---
Sediminibacillus halophilus	WP_074597485	-KQ--IT-A---VF---A-G--		K-DL--L----I-LVL------F---
Sediminibacillus massiliensis	WP_077622139	-KQ--IT-A---AF-V-A-G--		YG-LDSL--M-V-IIL-V----F---
Solibacillus isronensis	WP_079524962	--K--VN-AI-----F-A-AA-		L-D-APT-I--IIL-L-G---V----
Solibacillus kalamii	WP_087615498	--K--VN-AI-----F-A-AS-		L-D-APT-I--IIL-L-G---V----
Sporosarcina globispora	WP_053436602	-KH--IT-A------IVA-VAA	G	YE---GW-IV-FILIL-L---IF---
Sporosarcina sp. EUR3 2.2.2	WP_024535117	--G--TT-AI-------A-TA-		Y----ANL-V-IIL-L-VI--I----
Staphylococcus aureus	WP_037587459	--M--TN-AI---V-F-A-AL-		A-ELP-E----VILGL-VL--V--F-
Staphylococcus fleurettii	WP_107509327	--M--TN-AI---V-FVA-AL-		A-E-P-E----VILGL-VL--V--F-
Staphylococcus lentus	WP_017001117	--M--TN-AI---V-FVA-AL-		A-ELP-E----VILGL-VL--V--F-
Staphylococcus sciuri	WP_103361902	--M--TN-AI---V-FVA-AL-		A-ELP-E----VILGL-VL--V--F-
Staphylococcus stepanovicii	WP_095088608	--L--TN-AI---V-F-A-GL-		A-ELP-E-----ILAL-VL--V--F-
Staphylococcus vitulinus	WP_016911332	--M--TN--I---V-F-A-AL-		A-ELP-E----VILGL-VL--V--F-
Terrabacteria group	WP_012656643	--M--TT-AI-----FVA-A--		A--L--E----AVL-L-LI--I--F-
Thalassobacillus devorans	WP_085508007	-QH--IS-VM--IF-IAA-AL-		-SDV--Y-T--IIL-L-V---VF---
Thermolongibacillus altinsuens	WP_132948018	-KF--IS-I------IVA-FA-	G	YE---HW-SV-FIL-L-T---AF---
Ureibacillus thermophilus	QBK25523	L-K--IN-AI-----F-A-AT-		--D-APT-IK-VIL-L-GI--I----
Ureibacillus thermosphaericus	WP_016837537	--K--IN-AI-----FVA-AT-		--D-APT-IK-VIL-L-GI--V----
Virgibacillus dokdonensis	WP_101934057	-KK-LIT-A---GF-IVA--I-	A	TNAMD-M-AV-L-LIL-L----F-F-
Virgibacillus indicus	WP_094884029	-KK-LIT-A---GF---A-AI-	A	TEAMD-M--V-I-LIL-L----F-F-
Virgibacillus necropolis	WP_089532728	-K--LIT-A---VF--VA-GI-	V	GELMP-M-----IL-L-I---AF---
Virgibacillus phasianinus	WP_089062871	-KY-LIT-G---IF--VA-GL-	V	GELMP-M-----IL-L-I---AF-F-
Virgibacillus senegalensis	WP_053219795	-KQ--IT-A---VF---A-G--		M-DL--L----I-LVL------F---

Supplemental Figure 72

A partial sequence alignment of the cytochrome b6 protein containing a 5-6 amino acid insertion (boxed) that is exclusively shared by all members belonging to the Sporosarcina clade and absent in all other bacteria.

Group	Organism	Accession	18		53
Sporosarcina clade (9/9)	Sporosarcina ureae	WP_083035866	FVFKTRQFRTS	HM	FPIRKKMYASLAGTALGALLIFF
	Sporosarcina newyorkensis	WP_009497672	-----------	--	----------M--S---------
	Sporosarcina koreensis	WP_040286191	-Y---------	-V	-------F-AK--MF--VM-AV-
	Sporosarcina pasteurii	WP_115362259	-Y--M-----T	Y-	L-----LF--M--A---LF--T-
	Sporosarcina psychrophila	WP_067207609	-Y--------R	SL	----------MS-AS
	Lysinibacillus sphaericus LMG 22257	WP_075527357	-Y--M-----T	Y-	L-----LF--M--A---LF--S-
	Lysinibacillus sphaericus NCTC 11025	WP_125103255	---------	QV	-------FS-K--MF--L--V--
	Filibacter sp. TB-66	WP_124069872	-Y--------R	--	----------MS-AS--G---S-
	Bacillus sp. OxB-1	WP_041075558	-Y--------R	QI	----------M--AS--G--VS-
Other Bacteria (0/>100)	Lysinibacillus acetophenoni	WP_097148548	-YY--K---ST		L-----W-KAK--I---VFI-A-
	Lysinibacillus chungkukjangi	WP_107934281	-YY--KD--ST		L-----W-KAK--L---IFV-V-
	Lysinibacillus composti	WP_124762479	-YY--K---VN		L-----W-KAK--I---IFV-A-
	Lysinibacillus endophyticus	WP_121212700	-Y---K---SV		L-----W-KAQ--V---CFIV--
	Lysinibacillus halotolerans	WP_122971108	-YY------ST		L-----W-KAK--V---LFIMV-
	Lysinibacillus jejuensis	WP_108306426	-Y---K---S-		L--A--W-K-K--V--A-FIVL-
	Lysinibacillus macroides	WP_053996460	-Y---K---SP		L--A--W-K-K-NIGF-SFIL--
	Lysinibacillus manganicus	WP_081976275	-Y-------AT		L-----W-KAK--I---IFI-A-
	Lysinibacillus manganicus DSM	KGR77501	-Y-------AT		L-----W-KAK--I---IFI-A-
	Lysinibacillus massiliensis	WP_036174855	-Y---K----N		L-----W-KAK--V---IFI-A-
	Lysinibacillus meyeri	WP_107839773	-YY--K---ST		L-----W-TAK--V---TFI-A-
	Lysinibacillus odysseyi	WP_036156234	-YY--K---ST		L-----W-TAK------IFM---
	Lysinibacillus parviboronicapi	WP_107949853	-Y-------ST		L--A-NW-K-K-NVG---FILL-
	Lysinibacillus sp. 2017	WP_108713534	-Y---K---ST		L-----W-TAK--VT--IF-AL-
	Lysinibacillus sp. BF-4	WP_036142390	-Y---K---S-		L--A--W-K-K--V--A-FIVL-
	Lysinibacillus sp. BK089	WP_132357202	-Y---K---S-		L--A-NW-K-K-NVSF--FILL-
	Lysinibacillus sp. FJAT-14745	WP_053484986	-Y---K---S-		L--A-NW-K-K-NVSF--FILL-
	Lysinibacillus sp. Marseille-P	WP_106780397	-YY--K----N		L-----W-KAK--VS--IFI-A-
	Lysinibacillus sp. SYSU K30002	WP_126657485	-Y---K---AT		L-----W-KAQS-V---CF--S-
	Lysinibacillus sp. YLB-03	WP_118877343	-YY--KE--ST		L--Q--W-KAK--V---IFV-V-
	Lysinibacillus telephonicus	WP_126294640	-YY--K---ST		L-----W-KAK--V---MFI-A-
	Lysinibacillus xyleni	WP_097072881	-Y-------AV		L-----W-KAQ--V---CFI-A-
	Bacillus cecembensis	WP_057989291	-Y---K-L-AT		L-----W-TAK--VS--MF----
	Bacillus ndiopicus	WP_042474662	-YY--K---ST		L-----W-TAK--V---IFI-A-
	Chryseomicrobium excrementi	WP_100352266	-Y---K----D		L-----W--AKS-IC---FVFL-
	Paenisporosarcina antarctica	WP_134209365	-Y---K---NN		L--A--W--NRSLV---S--F--
	Paenisporosarcina indica	WP_075620059	-Y---K---ND		L--A--W--NR-LV---C--F--
	Paenisporosarcina sp. HGH0030	WP_016426678	-Y---K---SE		L--A--W-SNR-LI---S--F--
	Paenisporosarcina sp. OV554	WP_108585362	-Y---K---SE		L--A--W--NRSLI---S--F--
	Paenisporosarcina sp. TG-14	WP_017379921	-Y---K---NN		L--A--W--NRSLV---S--F--
	Paenisporosarcina sp. TG20	WP_019415966	-Y---K-I-SN		L--A--W-SNC-LVS--S--F--
	Planococcus maritimus	WP_068461654	-YY--K-I--R		L-V--NW---R-QI---SFIA--
	Planococcus massiliensis	WP_052652014	-YY--K---AT		L---R-W---I-SV---SFVL--
	Planococcus rifietoensis	WP_058381091	-YY--K-I--R		L-V--NW---R-QI---SFIA--
	Planococcus salinarum	TAA69660	-YY--K---AK		L-----W---T-SMF--SFIL--
	Planococcus salinus	WP_123163595	-YY--K---AT		L-----W---V-SV---SFVM--
	Planococcus sp. CAU13	WP_033541911	-YY--K----T		L-----W---N-SVF--SFGV--
	Planococcus sp. Y42	WP_077588687	-YY------SH		L--Q-NW-K-K------IF-LA-
	Planomicrobium flavidum	WP_088006635	-YY--K---SH		L-V--NW-K-K--V---LF-LA-
	Planomicrobium glaciei	WP_036804796	-YY--K---AV		L-----W--AV-AFS--SFIF--
	Planomicrobium soli	PSL35243	-YY--K-----		L-----W---M-SI---SFVL--
	Planomicrobium sp. Y74	WP_121633333	-YY--K-I-ST		L-----W---T-SIF--SFVF--
	Psychrobacillus insolitus	WP_111437663	-Y---K----T		------WFS-K-SVC---FIF--
	Psychrobacillus psychrotoleran	WP_093536887	-Y---K----P		L-----WLGAK-SVC---F-L--
	Psychrobacillus sp. OK028	WP_093060406	-Y---K----P		L-----WFGAK-SVC---F-L--
	Psychrobacillus sp. OK032	WP_093266893	-Y---K-L--N		L-----WF-AR-SVC---FMF--
	Rummeliibacillus pycnus	WP_102691066	-Y---K-I--N		L-----W-TNR--V---II--L-
	Rummeliibacillus stabekisii	WP_066791898	-Y---K----N		L-----W--GR--VS--V--VL-
	Solibacillus isronensis	WP_079528435	-Y---K---ST		L---R-W-TAK--V--A-F----
	Solibacillus kalamii	WP_087618414	-Y---K---ST		L---R-W-TAK--V--A-F----
	Solibacillus silvestris	WP_014823113	-Y---K---ST		L---R-W-TAK--V--A-F----
	Solibacillus sp. R5-41	WP_099424846	-Y---K---AT		L-----W-TAK--V----F----
	Tetzosporium hominis	WP_094944088	-Y---K----D		L-----W-SAKS-IC--VFVFL-
	Ureibacillus thermophilus	QBK26519	-YY--K---ST		L--Q--W-KAK--I---LFVV--
	Ureibacillus thermosphaericus	WP_016837120	-YY--K-----		L--Q--W-KAK--V---LFV---
	Viridibacillus arvi	WP_053416619	-YL--K---SD		L-----W-S-R--V---G--VI-
	Viridibacillus sp. OK051	WP_100794514	-YL--K---ST		L----NW---RS-V---G-----

Supplemental Figure 73

A partial sequence alignment of the hypothetical protein containing a two amino acid insertion (boxed) that is exclusively shared by all members belonging to the Sporosarcina clade and absent in all other bacteria.

			215		252
**Planococcus/Planomicrobium Clade (19/19)**	Planococcus faecalis	WP_078080352	EFARVSERLTD	PK	LKGVNTVTDWKRVKSSDLTILGSTT
	Planococcus antarcticus	WP_006830474	-----------	-N	------M------------------
	Planococcus citreus	WP_121299985	---V-------	--	-E----I-------MT---------
	Planococcus donghaensis	WP_008431661	-----------	-Q	------------I------------
	Planococcus halocryophilus	WP_008499025	-----------	-Q	------------I------------
	Planococcus halotolerans	WP_112223831	-----------	--	------I-------T----------
	Planococcus kocurii	WP_058385532	-----------	--	-------------------------
	Planococcus maitriensis	WP_112232311	---K-------	--	-E------------MT---------
	Planococcus maritimus	WP_068462016	---V-------	--	-E----I-------MT---------
	Planococcus massiliensis	WP_052651769	-----------	-D	------I-------T-------N--
	Planococcus plakortidis	WP_068869046	---V-------	--	-E------------MT---------
	Planococcus rifietoensis	WP_058380906	---V-------	--	-E------------MT---------
	Planococcus salinarum	TAA70651	-----------	--	------I-------T----------
	Planococcus salinus	WP_123163795	-----------	--	----------E-T-K----------
	Planococcus versutus	WP_065524690	-----------	--	-T----------I------------
	Planomicrobium glaciei	WP_036809392	-----------	--	---------------TE--------
	Planomicrobium okeanokoites	WP_117312181	-----------	--	------I-------T----------
	Planomicrobium soli	WP_106532681	-----------	--	------I-----T-T---V------
	Planomicrobium koreense	WP_135501277	-----------	--	--------------T----------
**Other Bacteria (0/>100)**	Planococcus sp. Y42	WP_077588879	---Q-----AE		-P--S-----S-E-A----V-----
	Planomicrobium flavidum	WP_088006347	---------SE		-P--------R-K-MT---------
	Bacillus cecembensis	WP_057984088	-------N---		-P----T------RL-P-AV--R--
	Bacillus ndiopicus	WP_042473968	---------A-		-P----T------RL-P-AL--R--
	Bacillus sp. FJAT-22090	WP_053588874	---------G-		-N----T---R---T-S-A---TAS
	Bacillus sp. OxB-1	WP_041075184	---A-----D-		-P----T---D---M-SNA------
	Bhargavaea beijingensis	WP_092096858	-Y-K-----HE		-P----T---E---N-E-A---N--
	Bhargavaea cecembensis	WP_040228799	-Y-L-----G-		-P----T---E--RN-P-A---T-S
	Bhargavaea ginsengi	WP_092051192	-Y-------HE		-P----T---E--RN-E-A---Q--
	Caryophanon latum	WP_083995377	----------E		-P----T---R---L-S-A---R--
	Caryophanon tenue	WP_066542371	----------E		-T----T-------L-S-A---R-S
	Chryseomicrobium excrementi	WP_100352443	---------NE		-Q----T---R--RN-N-A---T--
	Edaphobacillus lindanitolerans	WP_084186636	-Y-L-----G-		-P----T---E--RN-P-AV--T--
	Filibacter sp. TB-66	WP_124071474	---S-----NE		-P--D-T---D---L-TS----T--
	Kurthia gibsonii	WP_121175916	---V---H-SE		-P----T---Q-E-K---S---A--
	Kurthia huakuii	WP_029499175	---I-----N-		-P----T---A-K-K-------K--
	Kurthia massiliensis	WP_010287916	---I-----NE		-P--S-T---A-K-K---A---R--
	Kurthia senegalensis	WP_010303283	---T-----NE		MP--T-T---A-K-K---S---K--
	Kurthia sibirica	WP_109305913	---V---H---		-P----T---Q-ELK-K-----R--
	Kurthia sp. 11kri321	WP_068452710	---V---H-SE		-P----T---Q-E-K---S---A--
	Kurthia sp. 3B1D	WP_126990288	---V-----NN		-P----T---A-K-K-------K--
	Kurthia zopfii	WP_109350752	---V---H-SE		-P--D-T---Q-E-K---A------
	Lysinibacillus acetophenoni	WP_097148257	---------P-		-P-I--T------RLNS-S---R--
	Lysinibacillus boronitolerans	WP_016992375	----------E		-P----T-------L-S-S---R--
	Lysinibacillus chungkukjangi	WP_107935456	---------S-		-P----T-------L-S-A---R--
	Lysinibacillus composti	WP_124762673	---------S-		-P----T-------L-S-S---R--
	Lysinibacillus contaminans	WP_082332674	---L-----SE		-S----T------RHVP-S---R--
	Lysinibacillus endophyticus	WP_121215696	---------P-		-P----T-------L-SVA---R--
	Lysinibacillus fluoroglycofeni	WP_107942525	---------A-		-P----T------RL-P-AL--R--
	Lysinibacillus fusiformis	WP_069481971	----------E		-P----T-------L-S-S---R--
	Lysinibacillus halotolerans	WP_122971906	---------S-		-P----T-------L-S-A---R--
	Lysinibacillus jejuensis	WP_108306995	---------SE		-P----T-------L-P-A---R--
	Lysinibacillus macroides	WP_053996113	----------E		-P----T-------L-S-S---R--
	Lysinibacillus manganicus	WP_036186184	---------S-		-P----T-------LAP-SV--R--
	Lysinibacillus mangiferihumi	WP_107893972	----------E		-P----T-------L-S-SV--R--
	Lysinibacillus massiliensis	WP_036173551	---------P-		-P----T-------L-S-S---R--
	Lysinibacillus meyeri	WP_107842114	---------A-		-P----T------RLTPFSL--R--
	Lysinibacillus odysseyi	WP_036157972	---L-----SE		-P----T-----IRLAPVAV--R--
	Lysinibacillus parviboronicapi	WP_107924588	----------E		-P----T-------L-S-SV--R--
	Lysinibacillus saudimassiliens	CEA04905	---------SE		-P----T-------L-P-A---R--
	Lysinibacillus sinduriensis	WP_088291734	---------S-		-P----T-------L-S-A---R--
	Lysinibacillus sp. 2017	WP_108712477	-------Q-SE		-P---AT---R--HA-T-SV--R--
	Lysinibacillus sp. B2A1	AVK84386	----------E		FP----T-------L-S-S---R--
	Lysinibacillus sp. BF-4	WP_036141957	---------SE		-P----T-------L-P-A---R--
	Lysinibacillus sp. BK089	WP_132356755	----------Q		-P----T-----L-L-S-A---R--
	Lysinibacillus sp. BK089	WP_132356756	----------Q		-P----T-----L-L-S-A---R--
	Lysinibacillus sp. FJAT-14222	WP_053595161	----------E		-T----T-------L-S-S---R--
	Lysinibacillus sp. FJAT-14745	WP_053485465	----------Q		-P----T-----L-L-S-A---R--
	Lysinibacillus sp. Marseille-P	WP_106779896	---------S-		-P----T-----I-LTS-S---R--
	Lysinibacillus sp. OL1	WP_131520596	----------E		-P----T-------L-S-S---R--
	Lysinibacillus sp. SYSU K30002	WP_126657097	---------S-		-P-I--T-------L-S-A---R--
	Lysinibacillus sp. YLB-03	WP_118875781	---------S-		-P----T---R---L-S-G---R--
	Lysinibacillus sp. YR326	WP_134018321	----------Q		-P----T-----L-L-S-G---R--
	Lysinibacillus sp. ZYM-1	WP_054609381	----------E		-P----T-------L-S-S---R--
	Lysinibacillus sphaericus	WP_075528003	---M-----S-		FP----T---E--RK-TN----T--

Other Bacteria (0/>100)

Lysinibacillus telephonicus	WP_126296364	---------S-	-P----T-------L-SVS---R--
Lysinibacillus varians	WP_025219986	----------E	-P----T-------L-S-SV--R--
Lysinibacillus xylanilyticus	WP_049663904	----------Q	-P----T-----L-L-S-G---R--
Lysinibacillus xylanilyticus	WP_100543237	----------Q	-P----T-----L-L-T-G---R--
Lysinibacillus xyleni	WP_097074600	---------P-	-P----T-------L-SVAV--R--
Paenisporosarcina antarctica	WP_134209570	-------H-GE	MI----T---V--PK-S-A---R--
Paenisporosarcina indica	WP_075618091	---------S-	M-----T---V--PK-S-A------
Paenisporosarcina quisquiliaru	WP_090563765	---------GE	-E----T---R-I-T-S-A------
Paenisporosarcina sp. HGH0030	WP_016426927	----------E	MV----T---V--PK-P-A---R--
Paenisporosarcina sp. K2R23-3	WP_119883321	-----------	-P----T---N--PT-N-S---RV-
Paenisporosarcina sp. OV554	WP_108586239	----------E	M-----T---V--PK-T-S---R--
Paenisporosarcina sp. TG-14	WP_081503738	-------H-GE	MI----T---V--PK-S-A---R--
Paenisporosarcina sp. TG20	WP_019413691	-------H-GE	-N----T---V--PM-T-S---R--
Psychrobacillus insolitus	WP_111437898	---------GE	-T----T---R---T-S-A---T--
Psychrobacillus psychrodurans	WP_093496093	---------GE	-E----T---R-I-T-S-A------
Psychrobacillus psychrotoleran	WP_093538386	---------G-	-E----T---R---N-S-A---T--
Psychrobacillus sp. FJAT-21963	WP_056831687	---------G-	-I----T---R---T-S-A---TAS
Psychrobacillus sp. FJAT-21963	WP_056832203	---------GE	-E----T---R---T-S-A------
Psychrobacillus sp. OK028	WP_093060652	---------SE	-E----T---R---T-S-A------
Psychrobacillus sp. OK032	WP_093277398	---------DG	-E----T---R---T-S-A------
Rummeliibacillus pycnus	WP_102694302	---V---N-HE	-PN---T---A-K-K---S------
Rummeliibacillus stabekisii	WP_066786545	---I---H-NE	-PN---A-----I-N---S------
Solibacillus isronensis	WP_079527809	-------Q-A-	-P----T------RL-P-A---R--
Solibacillus kalamii	WP_087616050	-------Q-A-	-P----T------RL-P-A---R--
Solibacillus silvestris	WP_014823409	-------Q-A-	-P----T------RL-P-A---R--
Solibacillus sp. R5-41	WP_099424545	-------N---	-P----T------RL-P-AV--R--
Sporosarcina koreensis	WP_082713751	---A-----S-	FE----T---E---F--S----TM-
Sporosarcina newyorkensis	WP_078817001	---V-----NE	-P----T---D---K--SA------
Sporosarcina pasteurii	WP_115364080	---M-----S-	FP----T---E--RK-SN----T--
Sporosarcina psychrophila	WP_067210201	---T-----GE	-P--D-T---E--RK--N----T--
Sporosarcina sp. BI001-red	WP_116017194	---T---Q-SS	-P----T---E---M--SA---T--
Sporosarcina sp. D27	WP_025784757	---T---Q-GS	-P----T---E---M--SA---T--
Sporosarcina sp. EUR3 2.2.2	WP_024534503	----------E	M-----T---V--PM-T-S---R--
Sporosarcina sp. HY008	WP_067403517	---A-----SE	-P----T---E-MRR--N-------
Sporosarcina sp. P13	WP_099687338	---II----DE	-P-F--T---A---K-YSA------
Sporosarcina sp. P17b	WP_099625702	---V-----DQ	-P----T---D---K--SA------
Sporosarcina sp. P19	WP_099690536	-L-V-----DQ	-P----T---D---K--SA------
Sporosarcina sp. P2	WP_099630341	-L-V-----DQ	-P----T---D---K--SA------
Sporosarcina sp. P20a	WP_099677123	-L-V-----DQ	-P----T---D---K--SA------
Sporosarcina sp. P26b	WP_099693264	---V-----DQ	-P----T---D---K--SA------
Sporosarcina sp. P3	WP_099637675	---V-----DQ	-P----T---D---K--SA------
Sporosarcina sp. P33	WP_081241856	---V-----DQ	-P----T---D---K--SA------
Sporosarcina sp. P34	WP_099694642	-L-V-----DQ	-P----T---D---K--SA------
Sporosarcina sp. P35	WP_099662711	---V-----DQ	-P----T---D---K--SA------
Sporosarcina sp. P37	WP_085429872	---V-----DQ	-P----T---D---K--SA------
Sporosarcina sp. P7	WP_099637335	-L-V-----DQ	-P----T---D---K--SA------
Sporosarcina sp. PTS2304	WP_114923621	---II----DE	-P-F--T---E---K--SA------
Sporosarcina sp. ZBG7A	WP_039041666	---T---Q-GS	-P----T---E---M--SA---T--
Sporosarcina ureae	WP_085426052	---V-----DQ	-P----T---D---K--SA------
Tetzosporium hominis	WP_094942066	---------NE	-Q----T---R--RN-N-A---T--
Ureibacillus thermophilus	QBK26246	---------F-	-P----T-------L-S-A---R--
Ureibacillus thermosphaericus	WP_096551143	---------YE	-P----T-------L-S-SV--R--
Viridibacillus arvi	WP_053416328	---I---N-SK	-P----T---A---K-A-S------
Viridibacillus sp. FSL H8-0123	WP_076064883	---I---N-SE	-P----T---A---K-A-S------
Viridibacillus sp. OK051	WP_100794241	---I---H-SE	-P----T---A---K-A-S------

Supplemental Figure 74

A partial sequence alignment of the penicillin-binding protein 2 containing a two amino acid insertion (boxed) that is exclusively shared by all members belonging to the Planococcus/Planomicrobium Clade.

Group	Organism	Accession	56		96
Planococcus/Planomicrobium clade (19/19)	Planococcus halocryophilus	WP_065528121	WSREIMEGSTVPPTLNSEKTYK	SKE	KKEMLTGTFGILFKPF
	Planococcus antarcticus	WP_006828872	----------------------	---	----------------
	Planococcus kocurii	ALS78163	----------------------	---	----------------
	Planococcus faecalis	AQU79935	----------------------	---	----------------
	Planococcus versutus	WP_049693383	----------------------	---	----------------
	Planococcus donghaensis	WP_008429172	--------NV------------	---	----------------
	Planococcus salinarum	TAA72113	--------A----------K--	KQ-	-Q--------------
	Planococcus citreus	WP_121297792	--------A----------K--	RQ-	-QD-------MR-Q--
	Planococcus halotolerans	WP_112222968	--------A----------K--	KQ-	--D----------R--
	Planococcus maitriensis	WP_112231302	--------A----------K--	RQ-	-QD-------MR-Q--
	Planococcus maritimus	WP_068463032	--------A----------K--	RQ-	-QD-------MR-Q--
	Planococcus massiliensis	WP_052651061	--------A----------K--	RQ-	-Q--M-----MVL---
	Planococcus plakortidis	WP_068869488	--------A----------K--	RQD	-QD-------MR-Q--
	Planococcus rifietoensis	WP_058383474	--------A----------K--	RQ-	-QD-------MR-Q--
	Planococcus salinus	WP_123164414	--------A----------KF-	RQ-	-QD---K---ME----
	Planomicrobium koreense	WP_135500253	--------A-------------	RA-	-N--------MK-A--
	Planomicrobium soli	WP_106534663	--------AI-----------N	R--	-N--M-----MI----
	Planomicrobium okeanokoites	WP_084244917	--------A----------K--	KQ-	-Q--------------
	Planomicrobium glaciei	WP_036807040	--------A---Q--KT--R-S	RQ-	-N--I---Y--K-A--
Other Bacteria (0/>100)	Planococcus sp. Y42	WP_077589733	--------AVF----KT-RKF-		-S-L------MRLE--
	Planomicrobium flavidum	WP_088005863	--------AVF----KT-RKFQ		-TQ--------RLE--
	Anoxybacillus ayderensis	WP_085788183	-D---Q--AVS----QT--QFE		-EKI-S----MP-A--
	Anoxybacillus flavithermus	WP_081253652	-D---Q--AVS----QT--QFE		-EKI------MP-A--
	Anoxybacillus gonensis	WP_009373471	-D---Q--AVS----QT--QFE		-EKI-S----MVLA--
	Anoxybacillus kamchatkensis	WP_019418701	-D---Q--AVS----QT--QFE		-EKI-S----MVLA--
	Anoxybacillus pushchinoensis	WP_091703180	-D---Q--AVS----QT--QFE		-EKI------MP-A--
	Anoxybacillus sp. 103	WP_077429527	-D---Q--AVS----QT--QFE		-EKI------MP-A--
	Anoxybacillus sp. KU2-6(11)	KFZ43525	-D---Q--AVS----QT--QFE		-EKI------MP-A--
	Anoxybacillus suryakundensis	WP_082435657	-D---Q--AVS----QT--KFE		-EKI-I----MP-A--
	Anoxybacillus thermarum	WP_084221338	-D---Q--AVS----QT--QFE		-EKI-S----MP-A--
	Anoxybacillus vitaminiphilus	WP_111643583	-D---Q--AVF----KT----E		-EKVIN----MRLE--
	Bacillus andreraoultii	WP_033829346	-H---R---VS----KT--R-E		-EKL-------I-E--
	Bacillus aryabhattai	WP_098621491	-D---Q--AVF----KT--KF-		-Q-LVN-------A--
	Bacillus asahii	WP_127759671	-QK--R--AI-----KT-RKFE		-IKLM------P----
	Bacillus campisalis	WP_046522267	-D------AVF----K---K-E		-QKI-E-----PL---
	Bacillus cecembensis	WP_057985707	--------AVF----KT-RKFD		R-G-------MELN--
	Bacillus coahuilensis	WP_059350672	-----Q--QAF----KT--R-E		-EKLV-S-Y---LH--
	Bacillus cucumis	WP_101647618	--------A-Y----K---RFE		-DK--SSS---VL---
	Bacillus dielmoensis	WP_042456030	-D------AIF----KT--QFE		-VKVM------K-E--
	Bacillus flexus	WP_061785705	-D---Q--AVF----KT--K-E		-QKL-N-----S-A--
	Bacillus foraminis	WP_132005899	-D------AVF----K--RK-E		-QKL-E--Y--PL---
	Bacillus fumarioli	WP_066369725	-D------AIF----KT-RKFE		-EK-I------P----
	Bacillus halmapalus	WP_078381034	-E---R--AIQ----KT-RKFE		-QKL-------K-A--
	Bacillus horikoshii	WP_064101244	-E---R--AIH----K--RRFE		-QKL-N-----K-G--
	Bacillus jeotgali	WP_102262065	-D------AVH----KT-VKFE		-EKV-------P----
	Bacillus kochii	WP_095369660	-D------AVY----KT--KFE		-VRV-------P-L--
	Bacillus koreensis	WP_053399774	-D---Q--AVF----KT-RKF-		-Q-V-------S-RD-
	Bacillus lentus	WP_066140053	-D-V-K--A-A----K--RQFE		-VKI-------ILE--
	Bacillus litoralis	WP_066330521	-E---R--AIY----KT-R--E		-QRV-N-S--VPL---
	Bacillus marisflavi	WP_053427414	-----Q--A-Y----KT--K-E		-ERV-------P-A--
	Bacillus massiliosenegalensis	WP_019152933	-D---I--AV-----KT--K-L		-E-L-----C-P----
	Bacillus mediterraneensis	WP_071459314	-D---A---VF----K---KFE		-QK-M------P-E--
	Bacillus megaterium	WP_080967530	-D---Q--AVF----KT--KF-		-Q-LVN-------A--
	Bacillus ndiopicus	WP_042471584	--------AVF----KT-RKFD		R-G-I------EI---
	Bacillus persicus	WP_090747621	-D------AVF----K--RKFE		-QRL-E-S---P--S-
	Bacillus selenatarsenatis	WP_041965555	-D------AVY----KT-VKFE		-EKV-------PL---
	Bacillus simplex	WP_125162529	-QK--Q--A-M----KT-RKFE		-QRL-E-----P-E--
	Bacillus sp. 7504-2	WP_095313180	-D------AIL----KT-EK-L		-E-L-----C-P----
	Bacillus sp. 7586-K	WP_095298940	-E---R--AIH----KT--I-E		-QKV-N-----PL---
	Bacillus sp. AFS040349	WP_098799400	-E---R--AIY----KT-R--E		-EKI-N-----P----
	Bacillus sp. Aph1	WP_051137122	-D---Q--AVF----KT--KF-		-Q-LVN-------A--
	Bacillus sp. CBEL-1	WP_131886959	-D---Q--AVF----KT--K-E		-QKL-N-----S-A--
	Bacillus sp. CGMCC 1.16541	WP_110112394	-D---R--AVF----KT--KFQ		-ERI-------P-Q--
	Bacillus sp. CHD6a	WP_060666863	-E---R--AIY----K--RKFE		-EKL-N-----Q-G--
	Bacillus sp. FJAT-21351	KOP77492	-D---Q--AVF----KT--KF-		-Q-LVN-------A--
	Bacillus sp. FJAT-27251	WP_053362041	-D------AVF----K---KFE		-QKI-E-----PL---
	Bacillus sp. FJAT-29814	WP_066317992	-D------AVF----KT-RI-E		-QK-M------P----
	Bacillus sp. IHB B 7164	WP_082888750	-D---Q--AVF----KT--KF-		-Q-LVN-------A--
	Bacillus sp. J37	WP_026560505	-E---R--AVF----KT-QK-E		-IKV-------P----
	Bacillus sp. LL01	WP_047970725	-E---R--AIY----K--RKFE		-EKL-N-----K-G--
	Bacillus sp. Leaf406	WP_056537498	-----Q--A-Y----KT--K-E		-ERV-------P-A--
	Bacillus sp. M6-12	WP_101593488	-D---R--AVF----KT--K-E		-ERI-------P----
	Bacillus sp. OK838	WP_089362389	-QK--Q--A-M----KT-RKFE		-QRL-E-----P-E--
	Bacillus sp. OxB-1	WP_041073807	--------A-F----KT-AK-E		-TK---------LQQ-

Other Bacteria (0/>100)

Bacillus subterraneus	WP_044395054	-D------AVY----KT-VKFE	-EKV-------P-Q--
Bacillus thermocopriae	WP_129000062	-D---Q--AVS----QT--QFE	-EKI------MP-A--
Bacillus vietnamensis	WP_034755975	-----Q--A-Y----KT--R-E	-EKI-N---A-P-S--
Bacillus weihaiensis	WP_072580183	-E---R--AIY----KT-R--E	RQRI-N-----PLR--
Bacillus zeae	WP_119111435	-D-------IQ----K--RK-E	-QKI-N-----P-G--
Bhargavaea cecembensis	WP_040227916	--------A-Y----KT-RKF-	-T-L-N-----VLE--
Caldibacillus debilis	WP_020155669	-E---Q---VS----KT-RKFE	-MKV-------P-R--
Caryophanon latum	WP_066464039	--------AVY----KT-RKFD	R-G-Q-----MVLA--
Edaphobacillus lindanitolerans	WP_076756624	--------A-Y----KT-RKF-	-T-L-N-----VLD--
Jeotgalibacillus campisalis	WP_041057224	F----Q---IY----KT--KFQ	-QK--------PL---
Jeotgalibacillus soli	WP_041088818	-----Q--AAF----KT--KFE	-QKI-N-----PL---
Jeotgalibacillus sp. S-D1	WP_133375997	F----Q--A-Y---IKT-RKFE	-QKI-N------L---
Kurthia huakuii	WP_029498126	--------AVF----QT--K-T	H---R----A-AVA--
Kurthia massiliensis	WP_010286616	--------AVF----TT--K-T	H---R-----MMLE--
Kurthia senegalensis	WP_010301828	--------AVF----KT--KFS	H---R-----MHVE--
Kurthia sibirica	WP_109304410	--------AAF----K---KFD	RQ--K------V-Q--
Kurthia sp. 11kri321	AMA62813	--------AAY----ET--KFD	RQK-Q-----MVLE--
Kurthia zopfii	WP_109349940	--------ASY----K---KFE	RQQ-K---L---L---
Lysinibacillus fluoroglycofeni	WP_107943070	--------AVF----KT-RKFD	R-G-I------EI---
Lysinibacillus jejuensis	WP_108305674	--------A-F----K--RKFD	R-G-Q------H--H-
Lysinibacillus mangiferihumi	WP_107895652	--------A-F----K--RKFD	R-G-E------ELSH-
Lysinibacillus meyeri	WP_107839295	--------AVF----KT-RKFD	R-G--------EI---
Lysinibacillus odysseyi	WP_036155980	--------AVF----KT-RKFD	R-G-M-----MELG--
Lysinibacillus saudimassiliens	CEA02490	--------A-F----KT-RKFD	R-G-Q------H-NH-
Lysinibacillus sp. 2017	WP_108711768	--------AVF----KT-RKFD	R-G-M-----MEI---
Lysinibacillus sp. B2A1	AVK83008	--------A-F----K--RKFD	R-G-E------ELS--
Lysinibacillus sp. BF-4	WP_036146039	--------A-F----KT-RKFD	R-G-Q------H-NH-
Lysinibacillus sp. FJAT-14745	WP_053483433	--------A-F----K--RKFD	R-G-E------ELS--
Lysinibacillus varians	WP_025218739	--------A-F----K--RKFD	R-G-E------ELSH-
Paenisporosarcina antarctica	WP_134210182	--------A-F----KT--K-E	RTK--------YL-H-
Paenisporosarcina indica	WP_075617430	--------A-Y----K---K-E	-TK--------YL-H-
Paenisporosarcina sp. K2R23-3	WP_119882861	--------A------KT--K-E	-TK---------LE--
Paenisporosarcina sp. OV554	WP_108584461	--------A-F----K---K-E	-TK--------YL-H-
Paenisporosarcina sp. TG-14	WP_017379529	--------A-F----KT--K-E	RTK--------YL-H-
Paenisporosarcina sp. TG20	WP_019416081	--------A-F----K---K-E	-TK-M------YL-H-
Psychrobacillus insolitus	WP_111438687	-----I---------K---KFE	RTKL--S---VEL---
Psychrobacillus psychrotoleran	WP_093535400	-----I----N----QT--K-E	RTKA--S----DM---
Psychrobacillus sp. OK028	WP_093059853	-----I----N----QT--K-E	RTRA--S----EM---
Psychrobacillus sp. OK032	WP_093264080	-----I---------Q---K-E	RTKV--SSY-VEL-S-
Quasibacillus thermotolerans	WP_039231876	-DK--R--AVF---IK---KFE	-TKV------VP----
Rummeliibacillus pycnus	WP_102692789	--------A-F----KT-RKFD	R-K-M------KVE--
Rummeliibacillus stabekisii	WP_066785095	--------A-F----KT-RKFD	RQK-MS-----KIV--
Salinicoccus albus	WP_020008759	-N--------K----KT--K-N	--Q--E---V-CMA--
Salinicoccus luteus	WP_031548383	---Q-I--T-K----K---KF-	-Q-L-D---V-CL---
Salinicoccus roseus	WP_052443801	---Q-I--T-K----K---KF-	-Q-L-D---V-CL---
Solibacillus isronensis	WP_079524947	--------AVF----KT-RKFD	R-G-M-----MEI---
Solibacillus kalamii	WP_087615503	--------AVF----KT-RKFD	R-G-M-----MEI---
Solibacillus silvestris	WP_065216480	--------AVF----KT-RKFD	R-G-M-----MEI---
Solibacillus sp. R5-41	WP_099422647	--------AVF----KT-RKFD	R-G-------MEL---
Sporosarcina koreensis	WP_060205157	--------A-F----KT--KFD	RQK-V------SLDH-
Sporosarcina newyorkensis	WP_040758291	--------A-Y----K---R-E	-DK----S-AMVLE--
Sporosarcina psychrophila	WP_067211568	--------A-F----ET--KFE	-RKV----Y--QLRH-
Sporosarcina sp. BI001-red	WP_116016118	----------Y----K---K-E	-QK--------I-E--
Sporosarcina sp. D27	WP_025785203	--------A-Y----K---K-E	-QK--------I-E--
Sporosarcina sp. EUR3 2.2.2	WP_024535126	--------A-F----KT--K-E	RTK--------YL-H-
Sporosarcina sp. HYO08	WP_067408032	--------A-F----KT--I-E	-TK--------FLHN-
Sporosarcina sp. P13	WP_099688359	--------A-Y---IR---R-E	-DK---SS-AVVLQ--
Sporosarcina sp. P34	WP_099694058	--------A-Y----K---R-E	-DK--SSS---VL---
Sporosarcina sp. P7	WP_099635685	--------A-Y----K---R-E	-DK--SNS---VL---
Sporosarcina sp. PTS2304	WP_114923208	--------A-Y---IK---R-E	-DK---SS--MVIE--
Viridibacillus arvi	WP_053417578	--------A-F----K--RKFE	R-H-M------IL---
Viridibacillus sp. OK051	WP_100796340	--------A-F----Q--RKFE	R-K-M------EL---

Supplemental Figure 75

A partial sequence alignment of the hypothetical protein containing a three amino acid insertion (boxed) that is exclusively shared by all members belonging to the Planococcus/Planomicrobium clade.

Group	Organism	Accession	14		65
**Planococcus/ Planomicrobium clade (18/18)**	Planococcus faecalis	WP_071153391	LLGNQNTKYKFEYDPDILEPIDNLH		SRDYFVKFNCPEFTSLCPQTGQPDFAT
	Planococcus antarcticus	WP_006828137	-------A-------E---------		---------------------------
	Planococcus donghaensis	WP_008428689	----------------V--------		T--------------------------
	Planococcus halocryophilus	WP_008496278	-------------A-E---------		T--------------------------
	Planococcus kocurii	WP_058386617	-------------------------		---------------------------
	Planococcus maritimus	WP_069577388	-------R-------G---A-----		T--------------------------
	Planococcus massiliensis	WP_052653722	-------------------------		T--------------------------
	Planococcus salinus	WP_123166051	-----D----YA-A-EV--A---M-		T--------N-----------------
	Planomicrobium glaciei	WP_036802747	---------Q---------A-----		T--------------------------
	Planomicrobium okeanokoites	WP_084243287	-----D----Y--A-EV--A---M-		---------N-----------------
	Planomicrobium soli	WP_106533077	-------------------------		T--------------------------
	Planomicrobium koreense	WP_135503470	---------------N---A-----		T--------------------------
	Planococcus rifietoensis	ALS75328	-------R-------G---A-----		T--------------------------
	Planococcus plakortidis	ANU21061	-------R-------G---A-----		T--------------------------
	Planococcus citreus	RLJ86208	-------R-------G---A-----		T--------------------------
	Planococcus halotolerans	RAZ75466	-----D----Y--A-EV--A---M-		T--------N-----------------
	Planococcus salinarum	TAA66322	-----D----Y--A-EV--A---M-		T--------N-----------------
	Planococcus maitriensis	WP_058382035	-------R-------G---A-----		T--------------------------
**Other Bacteria (0/>100)**	Planococcus sp. Y42	WP_077587733	-----H---T-----S---AV----	V	N--F--------------M--------
	Planomicrobium flavidum	WP_088009110	-------A-S---------AV----	T	---F--------------M--------
	Bacillus acidiproducens	WP_018663417	-----K---PAD---AV--AV----	A	G-----------------L--------
	Bacillus aciditolerans	WP_121446832	---------S---A-EV--SV----	P	D-----------------L-H------
	Bacillus alkalitelluris	WP_078547887	-----G---L---S-----SF--K-	P	D------------S----K--------
	Bacillus anthracis	WP_098880351	---------L---S-E---VF--N-	P	N-----------------K--------
	Bacillus asahii	WP_127758965	-----G---L---T--V--TF--K-	I	------------------M--------
	Bacillus camelliae	WP_101354290	-----G-------A-E---SV----	S	N-----------------I-S---Y--
	Bacillus cereus	WP_098888874	---------L---S-E---VF--N-	P	N-----------------K--------
	Bacillus circulans	WP_047940920	-------Q--SD-A-EV--SV----	S	D-----------------I--------
	Bacillus coagulans	WP_035188704	-----K---PAD---GV--AV----	A	D-----------------L--------
	Bacillus cytotoxicus	WP_011984146	---------L---S-E---TF--N-	P	N-----------------K--------
	Bacillus fastidiosus	WP_066225693	-----G---I---S-----SF--K-	P	N-----------------M--------
	Bacillus foraminis	WP_132007858	---------S-Q-A-EV--AV--M-	S	------------------M-H------
	Bacillus fordii	WP_018706214	---------L---S-QV--------	A	D----I--------------N------
	Bacillus fortis	WP_120072524	---------L---S-EV--S-----	P	D-------------------N------
	Bacillus funiculus	WP_129727996	---------L---S-S---TF--N-	P	-------L----------K--------
	Bacillus galactosidilyticus	KRG12657	-----H---LDH-S--V--------	S	G-----------------K--------
	Bacillus ginsengihumi	WP_025727398	-------Q-S-D-A-EV--AV----	S	D-----------------L-H------
	Bacillus gottheilii	WP_080848446	-----G-Q-L---S-----SF--K-	P	N-----------------K--------
	Bacillus koreensis	WP_053399674	-----G---L---A--V--AF--K-	P	N-----------------K--------
	Bacillus lentus	WP_066136682	---------L-K-S--V--S-----	P	N-------------------H------
	Bacillus ligniniphilus	WP_017727515	-----G---Q-----S---VFE-K-	Q	------------------M--------
	Bacillus manliponensis	WP_034636342	---------L---S-E---VF--N-	P	N-----------------K--------
	Bacillus massiliosenegalensis	WP_019152499	---------S-D-A-EV--SV----	S	D-----------------I-S------
	Bacillus mesonae	WP_066383455	------NQ-L---S--V--AV----	A	D-----------------L-K------
	Bacillus mycoides	WP_078178262	---------L---S-E---VF--N-	P	N-----------------K--------
	Bacillus nealsonii	WP_016201606	-------Q--SD-A-EV--SV----	S	D-----------------I--------
	Bacillus niacini	WP_063252441	-------Q-S---A-EV--AV----	P	E-----------------L-H------
	Bacillus oceanisediminis	WP_110066745	-----G---L---S-E---AF--K-	P	N-----------------K--------
	Bacillus onubensis	WP_099354032	---------S---A-EV--SV----	P	D-----------------L-H------
	Bacillus pseudomycoides	WP_098716462	---------L---S-K---TF--N-	P	N-----------------K--------
	Bacillus sp. 123MFChir2	WP_020061890	---------L---S-G---TF--N-	P	N-----------------K--------
	Bacillus sp. AFS018417	WP_098309564	---------L---S-E---TF--N-	P	N-----------------K--------
	Bacillus sp. FJAT-27231	WP_049660373	-----G---T-----S---SF--K-	P	N-----------------V--------
	Bacillus sp. FJAT-27245	WP_053368496	-----G---A-Q-A-EV--AV----	S	N-----------------L-N------
	Bacillus sp. FJAT-27986	WP_066103416	------V--A-N-A-EV--SV----	S	D-----------------I-Q------
	Bacillus sp. FJAT-29814	WP_066303564	-----G---V---A-EV--SV----	P	N-----------------L--------
	Bacillus sp. FJAT-42315	WP_094834220	-----G-T---A---S---TF--K-	Q	N-----------------M--------
	Bacillus sp. FJAT-45350	WP_096199666	-------Q-H---N-S---SF--K-	P	N-----------------K--------
	Bacillus sp. H1a	WP_025150970	---------L---S-E---VF--N-	P	N-----------------K--------
	Bacillus sp. HMF5848	WP_125905749	-----G---------S---TF--K-	P	D-----------------I-A------
	Bacillus sp. HNG	WP_116353844	-------Q-S---A-EV--AV----	T	N-----------------L-H------
	Bacillus sp. J13	WP_028406450	-----G---T-----G---SF--K-	P	Y-----------------I--------
	Bacillus sp. M6-12	WP_101592600	-----G---L---S-QV--SF--K-	P	N--------------------------
	Bacillus sp. MUM 116	WP_071357160	-------Q-S---A-EV--AV----	S	N-----------------I-K------
	Bacillus sp. OK048	WP_090759583	---------S---A-EV--AV----	P	D-----------------L-H------
	Bacillus sp. OK085	WP_132095747	---------S---A-EV--AV----	P	D-E---------------L-H------
	Bacillus sp. PK3_68	WP_120036332	-----G---T-----S---SF--K-	P	N-----------------V--------
	Bacillus sp. SN10	WP_101166116	---------L---S-E---VF--N-	P	N-----------------K--------
	Bacillus sp. SYSU K30001	WP_124562866	-----H---L-----A---TF--N-	P	N-----------------K--------
	Bacillus sp. UMB0893	WP_101564623	-----G-N-L---S-----SF--K-	E	N-----------------K--------
	Bacillus sp. UNC438CL73TsuS30	WP_026573785	-------Q-S---A-EV--AV----	V	N-----------------L-K------
	Bacillus sp. V44-8	WP_117322995	-----G---L---S-----SF--K-	P	N-----------------M--------
	Bacillus sp. V47-23a	WP_117328142	-----G---L---S-QV--SF--K-	P	N--------------------------
	Bacillus sp. V5-8f	WP_101634584	-----G---L---S-E---SF--K-	P	N-----------------M--------

Other Bacteria (0/>100)

Bacillus sp. V59.32b	WP_117308515	-----G---L---S-----SF--K- P N-----------------M--------
Bacillus sp. WN066	WP_133332834	-------Q-S---A-EV--AV---- V D-----------------L-K------
Bacillus sp. XXST-01	WP_126407934	---------S-D-A-EV--SV---- S D-----------------I-A------
Bacillus sp. Y1	WP_119706693	-----G---L---S-S---AF--K- P N-----------------K--------
Bacillus terrae	WP_120117750	-------E-L---S-GV--S----- S D----I--------------K------
Bacillus thuringiensis	WP_000918896	---------L---S-K---VF--N- P N-----------------K--------
Bacillus toyonensis	WP_099010031	---------L---S-E---VF--N- P N-----------------K--------
Bacillus wiedmannii	WP_098096735	---------L---S-E---VF--N- P N-----------------K--------
Bacillus wudalianchiensis	WP_065411659	-----G---T-----S---SF--K- P N-----------------V--------
Bhargavaea beijingensis	WP_092093745	-------Q-ST--A-EV--AV---- T N-----------------I--------
Bhargavaea cecembensis	WP_008297641	-------Q-ST--A-EV--SV---- T N-----------------I--------
Bhargavaea ginsengi	WP_092052400	-------Q-ST--A-EV--AV---- S N-----------------I--------
Cohnella luojiensis	TFE29433	-----G-R-V-----GV--AF--K- P G-----------------M--------
Cohnella lupini	WP_115991663	-----G---V-----GV--TF---- P G-----------------M--------
Cohnella sp. 6021052837	WP_080834324	-----G---V---S-----TF--K- P D-----------------M--------
Cohnella sp. M2MS4P-1	WP_120977782	-----G---V-----GV--AF--K- P G-----------------M--------
Falsibacillus pallidus	WP_114745665	-----G-N-L---S-----TF--K- Q N-----------------K--------
Falsibacillus sp. GY 10110	WP_121682200	-----G-N-L---S-----AF--K- P N-----------------K--------
Filibacter sp. TB-66	WP_124071678	---------L---S--V--A----- M N--------------------------
Intestinibacter bartlettii	WP_007285244	-----G-----G-------VF--K- P NN----------------I--------
Lysinibacillus composti	WP_124766813	---------S-D-A-EV--AV---- S N-----------------L-H------
Lysinibacillus endophyticus	WP_121215048	---------A---A-EV--AV---- S N-----------------L-N------
Lysinibacillus fusiformis	WP_025116401	-----G---S---A-EV--AV---- S N-----------------L-N------
Lysinibacillus halotolerans	WP_122973455	-------Q-S-D-A-EV--AV---- S N-----------------L-H------
Lysinibacillus odysseyi	WP_036155154	------V--S-D-A-EV--AV---- T N-----------------I-S------
Lysinibacillus sp. BF-4	WP_036146420	---------L---A-EV--AVP--- T G--F--------------M--------
Lysinibacillus sp. BK089	WP_132363257	---------L---S-E---AV---- T KN-F--------------M--------
Lysinibacillus sp. SYSU K30002	WP_126658087	---------A-D-A-EV--AV---- S N-----------------L-N------
Lysinibacillus sp. YLB-03	WP_118876686	-----G---A---A-EV--AV---- S N-----------------L-N------
Lysinibacillus sphaericus	WP_031418567	-----G-N-S---A-EV--AV---- S N-----------------L-N------
Lysinibacillus telephonicus	WP_126295574	-----G---L---A-EV--AV---- S N-----------------L-H------
Lysinibacillus xylanilyticus	WP_068983048	---------L---S-EV--AV---- T GN-F--------------M--------
Lysinibacillus xyleni	WP_097073375	---------A-N-A-EV--AV---- S N-----------------L-N------
Mycobacteroides abscessus subs	SLL36168	-------Q--SD-A-EV--SV---- A D-----------------I--------
Ornithinibacillus californiens	WP_047983358	-------Q-S---A-EV--AV-S-- S N-----------------I-K------
Ornithinibacillus scapharcae	WP_010095477	-------N-S---A-EV--AV---- S N-----------------I-K------
Paenibacillus amylolyticus	WP_062832992	------V--T-----G---SF--K- P Y-----------------I--------
Paenibacillus bovis	WP_060533372	-----G---T-----S---SF--K- T Y-----------------I--------
Paenibacillus dauci	WP_046225586	-----G---T-----S---SF--K- S Y-----------------I--------
Paenibacillus etheri	WP_082651064	-----G-Q---G-------VF--K- P G-----------------V--------
Paenibacillus ihbetae	WP_077569313	-----G---T-----G---SF--K- P Y-----------------I--------
Paenibacillus lactis	WP_007130817	-----G---T-----G---SF--K- P Y-----------------I--------
Paenibacillus odorifer	WP_076304130	-----G-Q---G-------VF--K- P G-----------------V--------
Paenibacillus panacisoli	WP_028591132	-----G---T-----G---SF--K- P Y-----------------I--------
Paenibacillus polysaccharolyti	WP_090918481	------V--T-----G---SF--K- P Y-----------------I--------
Paenibacillus sp. 18JY21-1	WP_132417636	-----Q-Q-S-D-T--V--AV---- S N-----------------L-R------
Paenibacillus sp. DMB20	WP_046678334	-----G---------S---SF--K- P Y-----------------I-----Y--
Paenibacillus sp. FF9	WP_054956242	-----G---H-----S---SF--K- P Y-----------------I--------
Paenibacillus sp. FSL R5-0345	AIQ36087	-----G-Q---G-------VF--K- P G-----------------V--------
Paenibacillus sp. FSL R5-0765	WP_076209554	------V--T-----G---SF--K- P Y-----------------I--------
Paenibacillus sp. GM2FR	WP_100540733	-----G---T---------SF--K- P Y-----------------I--------
Psychrobacillus insolitus	WP_111439054	-----G---S-D-A-E---SV---- S T-----------------I--------
Psychrobacillus psychrotoleran	WP_093536326	-----G---S-D-A-EV--SV---- S T-----------------I-N------
Rummeliibacillus pycnus	WP_102692061	---------SY--N--V--AV---- T EN-F--------------M--------
Saccharibacillus kuerlensis	WP_018975987	-----G---T-----G---SF--K- A Y-----------------I--------
Sporosarcina globispora	WP_053437177	-----G---L---S-E---AF--K- P N-----------------K--------
Sporosarcina newyorkensis	WP_009497601	-------V-DYS-N-NL--S----- Q G--------------------------
Sporosarcina psychrophila	WP_067207771	---------VY--N--V--AV--M- V D-----------------M--------
Sporosarcina sp. P13	WP_099689112	-----E-V-DYS-N--V--A----- H ---------------------------
Staphylococcus chromogenes	WP_119510776	------N--E-D-R--V--TF--K- Q G-----------------I--------
Staphylococcus xylosus	WP_119615174	------N----D---TV--SF--K- P G-----------------I--------
Thermolongibacillus altinsuens	WP_132948453	-----G---V---N-----VF--K- P D-----------------K--------
Viridibacillus sp. OK051	WP_100795337	-----H-Q-S---A-EV--AV---- S N-----------------M-H------

Supplemental Figure 76

A partial sequence alignment of the NADPH-dependent 7-cyano-7-deazaguanine reductase QueF protein containing a one amino acid deletion (boxed) that is exclusively shared by all members belonging to the Planococcus/Planomicrobium clade. *Planococcus versutus* does not have a homolog of this protein.

Group	Organism	Accession	11		64
Planococcus/ Planomicrobium clade (19/19)	Planococcus maitriensis	WP_112232475	LVREDVLTEAMVKTLEVKKLLQRDRMS		ILDAVAKTGLSRSAFYKYRDAVFPFHS
	Planococcus donghaensis	WP_008431510	-----------Q------RM--ND-I-		-M---N---------------------
	Planococcus antarcticus	WP_006828761	-----------Q-------M--N--I-		-M---N---------------------
	Planococcus faecalis	WP_078080323	-----------Q-------M--N----		-M---N---------------------
	Planococcus halotolerans	WP_112223748	-----------Q-------M---GNI-		-----E---------------------
	Planococcus kocurii	WP_058385627	-----------Q-------M--N--I-		-M---N---------------------
	Planococcus maritimus	WP_068461855	---------------------------		-----S---------------------
	Planococcus massiliensis	WP_052651858	-----------Q------R----EH--		-----NQ--------------------
	Planococcus plakortidis	WP_068868964	---------------------------		------Q--------------------
	Planococcus salinarum	TAA69192	-----------Q-------M----KI-		-----E---------------------
	Planococcus salinus	WP_123164025	-------------------M-----V-		---------------------------
	Planococcus versutus	WP_065524208	-----------Q-------M--K--IT		--E--N---------------------
	Planomicrobium glaciei	WP_036806235	-----------Q------R--------		-----N---------------------
	Planomicrobium soli	WP_106533810	-----------Q---------------		-----SQ--------------------
	Planococcus rifietoensis	ALS74283	---------------------------		------Q--------------------
	Planococcus citreus	RLJ87208	---------------------------		------Q--------------------
	Planomicrobium okeanokoites	WP_084246263	-----------Q-------M----NI-		-----E---------------------
	Planomicrobium koreense	WP_135500684	-----------Q------R--------		-----NHA-------------------
	Planococcus halocryophilus	WP_065527857	E-YESKQFT		LAEIKE---I---TL-R-
Other Bacteria (0/>100)	Aeribacillus pallidus	WP_063387958	-------P---K---------E-GKV-	S	VQE--QLVD-----------------T
	Anoxybacillus vitaminiphilus	WP_111644646	-------P---K-V--A-E--E-KKAN	S	-A---QMV-V---V------------A
	Bacillus alcalophilus	WP_003320572	-----I--D--Q------S--ESGK-K	K	-NE--HRV----------K-GI----T
	Bacillus alkalinitrilicus	WP_078428989	-----M-----Q----A-E--ESGKVK	K	-NE--KEV------------GIY---T
	Bacillus alveayuensis	WP_044892895	-------P---R---------E-GKAL	S	VQE--QRVDM-----------I----T
	Bacillus atrophaeus	WP_106360459	-------P---R--------ID-KKAE	S	VAE--Q-VD----------------YT
	Bacillus azotoformans	WP_035194387	-I---F-P---K----A-A-IE-GKAD	S	VA---QMV------------GI----T
	Bacillus bogoriensis	WP_026672465	-----M-S--------A-AM--SGKAK	K	-SE--HAV-I--------K-GI-----
	Bacillus boroniphilus JCM 2173	GAE43939	-------P---K----A-EMIE-GKAE	S	VW---QRVD-----------T-----T
	Bacillus camelliae	WP_101354190	-------S---K------E--E-GKVT	S	VWQ-AQEA----------K-T-----T
	Bacillus cecembensis	WP_057988159	--------D--Q----A-Q---NGSVA	S	-W---KEVD------------------
	Bacillus cellulasensis	WP_041090934	-------PD--R---------D-KKAD	S	VA---Q-AD----------------YT
	Bacillus coagulans	WP_014097873	-------P---I---QA-E--E-GQAV	S	VG--AK-A------------T------
	Bacillus cucumis	WP_101652023	-------P---K--I---EM-E-GKAE	S	-A---L-VD-----------T----ST
	Bacillus dakarensis	WP_077214112	-------P---I----A-EM-E-KKVE	S	VWE--Q-VD-----------T-----T
	Bacillus deserti	WP_101641315	-------P---Q----A-E--D-GKAD	S	V-E--QQVDI----------T-----T
	Bacillus firmus	WP_035331302	-------P---K---DA-EMIE-GKAE	S	VW---Q-VD-----------T-----T
	Bacillus foraminis	WP_121611559	-------P---K----A-EMIE-GKAE	S	VW---QRVD-----------T-----T
	Bacillus fordii	WP_018708260	-------P---K----A-A--E-GSAE	S	-W---QQVD-----------T-----T
	Bacillus freudenreichii	WP_126431648	-------P---K----A-A--E-GSAE	S	-W---QQVD-----------T-----T
	Bacillus glycinifermentans	WP_048353528	-------P---R--------IE-KKAE	S	VAE--Q-VD----------------YT
	Bacillus indicus	WP_029279393	-------P---R------Q-IE-KKAE	S	VAE--QRVDM----Y-----------T
	Bacillus intestinalis	WP_087986771	-------PD--R---------D-KKAD	S	VA---Q-VD----------------YT
	Bacillus jeotgali	WP_079509897	-------P---K----A-EMIE-GKAE	S	VW---QRVD-----------T-----T
	Bacillus korlensis	WP_066050085	-------P---K---DA-EM-E-GKVE	S	VW---Q-VD-----------T-----T
	Bacillus kribbensis	WP_026693788	-------P---Q--I-A-E--D-GKAE	S	V-E--QQVDI----------T-----T
	Bacillus litoralis	WP_066334341	-----I-P---K--------IE-GKAD	S	VAE--QRVDM----------------T
	Bacillus marisflavi	WP_121620873	-----I-P---K----A-E--E-GKVD	S	VWE--Q-VD-----------T-----T
	Bacillus massilioanorexius	WP_019242755	-------P---Q---DA-E-I--GKAE	S	VWE--Q-VD-----------T-----T
	Bacillus massiliogabonensis	WP_102274421	-------P---K----A-EMIE-GKAE	S	VW--AQ-VD-----------T-----T
	Bacillus massiliogorillae	WP_042345615	-------P---Q----A-E-I--GKAE	S	VF---Q-VD-----------T-----T
	Bacillus massilionigeriensis	WP_075983594	-------P---K----A-EM-E-GKVE	S	VWE--Q-VD-----------TI----T
	Bacillus massiliosenegalensis	WP_019154666	-------P---R----A-EMIE-KKVE	S	VW---QQVD-----------T-----T
	Bacillus mediterraneensis	WP_071460259	-------P---K----A-EMIE-KKAE	S	VW---QRVD-----------T-----T
	Bacillus ndiopicus	WP_042475047	--------D--Q----A-A---TGAVA	S	-W---KEVD------------------
	Bacillus paralicheniformis ATC	AGN37361	-------P---R--------IE-KKAE	S	VAE--Q-VD----------------YT
	Bacillus pseudalcaliphilus	KMK75703	-----I--D--E------S--ESGKVK	K	-NE--HRV----------K-GI----T
	Bacillus pseudofirmus	WP_075387591	-----M-----Q----A-E--ESGKVK	K	-NE--KEV------------GIY---T
	Bacillus psychrosaccharolyticus	WP_040376203	-------P---K---DA-E-IE-G-AE	S	-W---Q-VD-----------T-----T
	Bacillus pumilus	OLP65386	-------PD--R---------D-KKAD	S	VA---Q-AD----------------YT
	Bacillus safensis	WP_065214575	-------PD--R---------D-KKAD	S	VA---Q-AD----------------YT
	Bacillus selenatarsenatis	WP_041964008	-------P---K----A-EMIE-GKAE	S	VW---QRVD-----------T-----T
	Bacillus smithii	WP_048623763	-------P---K------E--E-GKAD	S	VQ---N-VD-----------T-----T
	Bacillus soli	WP_066063841	-------P---I----A-EM-D-GKAE	S	-A---Q-AD-----------T----ST
	Bacillus sonorensis	WP_006637841	-------P---R--------IE-KKAE	S	VAE--Q-VD----------------YT
	Bacillus sp. 1NLA3E	WP_015595116	-------P---K----A-EMIE-GKAE	S	VW---NRVD-----------T-----T
	Bacillus sp. 3-2-2	WP_126048458	-------P---K----A-A--E-GSAE	S	-W---QQVD-----------T-----T
	Bacillus sp. 7504-2	WP_095307251	-------P---K----A-EMIE-KKVE	S	VW---QQVD-----------T-----T
	Bacillus sp. 7586-K	WP_095302402	-----I-P---K--------IE-GKVD	S	VAE--QRVDM----------------T
	Bacillus sp. AFS031507	WP_098934446	-------P---K---D--EM-E-GKAE	S	-A---H-AD-----------T----S-
	Bacillus sp. BK100	TCN03873	-------PD--R---------D-KKAE	S	VA---Q-VD----------------YT
	Bacillus sp. Co1-6	WP_052586392	-------PD--R---------D-KKAE	S	VA---Q-VD-----------T----YT
	Bacillus sp. FJAT-14578	WP_028395036	-------P---Q---QA-R-IE-GKVD	S	-A---QMVD-----------T-----T
	Bacillus sp. FJAT-25496	WP_057771725	-------P---K---DA-EMIE-GKAE	S	VW---Q-VD-----------T-----T
	Bacillus sp. FJAT-27231	WP_049663427	-------P---K----A-E-ID-H-VD	S	VWE--K-VD-----------T-----T

Other Bacteria (0/>100)

Bacillus sp. FJAT-27916	WP_049670016	-------P---Q---QA-EMIE-GKVL S VW---N-A------------T------
Bacillus sp. FJAT-29937	WP_066292905	-------P---K---DA-EMIE-GKAE S VW---Q-VD-----------T-----T
Bacillus sp. FJAT-42376	WP_123918143	-------P---K---D---MME-GKAA S VAE--QRAD-----------------T
Bacillus sp. FJAT-46582	WP_100331709	-------P---K----A-E-IE-RKAD S VW---K-VD-----------T-----T
Bacillus sp. HMSC76G11	WP_070879467	-------P---R--------IE-KKAD S VAE--QRVDM----Y-----------T
Bacillus sp. M5HDSG1-1	WP_127737300	-------S---K----A-E--E-GKAE S VF---Q-VD-----------TI----T
Bacillus sp. MUM 116	WP_071353987	-------P---K--I-A-E--E-GKVE S VQE--QQ-D-----------T----S-
Bacillus sp. NRRL B-14911	EAR65510	-------P---K----A-EMID-GKAE S VW---Q-VD-----------T-----T
Bacillus sp. OV166	WP_088090542	-------P---K---D--EM-E-GKAE S -A---H-AD-----------T----S-
Bacillus sp. PK3_68	WP_120033407	-------P---K----A-E-ID-H-VE S -WE--K-VD-----------T-----T
Bacillus sp. SA1-12	WP_046515793	-----I-P---K--------IE-GKVD S VAE--QRVDM----------------T
Bacillus sp. SJS	WP_082883714	-------P---K---D---MME-GKAG S VAE--QRVD-----------------T
Bacillus sp. T33-2	WP_101584293	-------P---K----A-EMIE-GKAE S VW---HRVD-----------T-----T
Bacillus sp. TS-2	WP_045485308	-----I--D--Q------S--ESGKIK K -NE--HHV------Y---K-GI----T
Bacillus sp. UMB0893	WP_101566971	-------P---R--------IE-KKAD S VAE--QRVDM----Y-----------T
Bacillus sp. UMB0899	WP_102230817	-----I-P---K--------IE-GKVD S VAE--QRVDM----------------T
Bacillus sp. V3-13	WP_101662713	-------P---K----A-EMIE-GKAE S VW---H-VD-----------T-----T
Bacillus sp. VT-16-64	WP_077114367	-------P---K----A-A--E-GTAE S -W---QQVD-----------T-----T
Bacillus sp. XXST-01	WP_126407382	-------P---K----A-EMIE-KKVE S VW---QQVD-----------T-----T
Bacillus sp. Y1	WP_119709261	-------P---K---DA-EM-E-GKVE S VW---Q-VD-----------T-----T
Bacillus sporothermodurans	WP_107920257	-------P---K------E-IE-GKAD S VA--AQSVD-----------T------
Bacillus subterraneus	WP_125481844	-------P---K----A-EMIE-GKAE S VW---QRVD-----------T-----T
Bacillus subtilis	WP_019846899	-------PD--R---------D-KKAD S VA---Q-VD----------------YT
Bacillus swezeyi	WP_076762976	-------P---R--------IE-KKAE S VAE--Q-VD----------------YT
Bacillus taeanensis	WP_113804308	-------S-S-Q-A--A-S--E-GKVE T VN---KHV------------GI----A
Bacillus testis	WP_050615908	-------P---Q---DA-E-I--GKAE S VWE--Q-VD-----------T-----T
Bacillus trypoxylicola	WP_061948224	-----I--D--Q------S--ESGKIK K -NE--HHV------Y---K-GI----T
Bacillus tuaregi	WP_071395900	-------P---K----A-EMIE-GKAE S VW---QRVD-----------T-----T
Bacillus velezensis	WP_129003637	-------PD--R---------D-KKAE S VA---Q-VD----------------YT
Bacillus weihaiensis	WP_072579035	-----I-P---K--------IE-GKVD S VAE--QRVD-----------------T
Bacillus wudalianchiensis	WP_065408926	-------P---K----A-E-ID-H-VD S VWE--K-VD-----------T-----T
Bacillus xiamenensis	WP_008357896	-------PD--R---------D-KKAD S VA---Q-AD----------------YT
Bacillus zeae	WP_119114135	-------P---K----A-EMIE-GKAE S VW---HRVD-----------T-----T
Bhargavaea beijingensis	WP_092096592	---------S-Q----A-R---NGEET T -W---KRV------------T------
Bhargavaea cecembensis	WP_008299804	---------S-Q----A-R---NGEE- T -W---KRV------------T------
Bhargavaea ginsengi	WP_092052161	---------S-Q----A-R---NGEE- T -W---KRV------------T------
Caryophanon latum	WP_066461042	--------D--Q---QA-H---TGAVA S -W---KEVD------------------
Caryophanon tenue	WP_066542244	--------D--Q----A-H---TGAVA S -W---KEVD------------------
Domibacillus robiginosus	WP_050181236	-------P---K---DA-E-IE-K-AD S VW---QQVD-----------T-----T
Edaphobacillus lindanitolerans	WP_076758417	---------S-Q----A-R---NGEE- T -W---KRV------------T------
Falsibacillus pallidus	WP_114745018	-I-----P---K----A-N--E-GKA- S VW---QRVD-----------T-----T
Falsibacillus sp. GY 10110	WP_121678762	---D---P---K----A----E-GKVD S VWE--R-VD-----------T-----T
Fictibacillus gelatini	WP_026676433	M------S-S-Q-A--A-S-IE-GKVK T VAE-A-QV------------GI----T
Fictibacillus phosphorivorans	WP_066396518	M------S-S-Q-A--A-S--E-GKVK T VAE-AE-V------------GI----T
Halobacillus dabanensis	WP_075034677	---S-I-P---M--I-A-E--E-GKVE S -FE--QQV-----------------QA
Halobacillus kuroshimensis	WP_027955271	---S-I-P---M--I-A-E--E-GKVG S -YE--KHV-----------------QA
Halobacillus litoralis	WP_128526336	---S-I-P---M--IDA-A--E-GKVE S -F---Q-V-----------------QA
Halobacillus trueperi	WP_115822560	---S-I-P---M--I-A-E--E-GKVE S -FE--QQV-----------------QA
Jeotgalibacillus sp. R-1-5s-1	WP_134376740	-------P---Q----A-Q--E-GKAG S VWE--REVD-----------T-----T
Kurthia huakuii	WP_029499325	--------D--Q---QA-H---TGMV- S -W---KEVD------------------
Kurthia massiliensis	WP_010288118	--------D--Q---QA-H---TGAV- S -W---KEVD------------------
Kurthia senegalensis	WP_010303563	--------D--Q---QA-H---TGAV- S -W---KEVD------------------
Kurthia sibirica	WP_109307039	--------D--H----A-Q---SGTV- S -W---KQVD------------------
Kurthia sp. 3B1D	WP_126991088	--------D--Q---QA-H---TGSV- S -W---KEVD------------------
Kurthia zopfii	WP_109348267	--------D--H----A-H---TGTV- S -W---KQVD------------------
Lysinibacillus acetophenoni	WP_097148397	--------D--Q----A-Q--ESGSV- S -W---K-VD------------------
Lysinibacillus chungkukjangi	WP_107934605	--------D--Q----A-H---SGAV- S -W---KEVD------------------
Lysinibacillus composti	WP_124762283	--------D--Q----A-H---SGAV- S -W---KEVD------------------
Lysinibacillus contaminans	WP_053582677	--------D--Q----A-H--SSGTV- S -W---KQVD------------------
Lysinibacillus endophyticus	WP_121212833	--------D--Q---QA-H---SGSV- S -W---KEVD------------------
Lysinibacillus halotolerans	WP_122973212	--------D--Q----A-H---SGAV- S -W---KEVDI-----------------
Lysinibacillus macroides	WP_053996302	--------D--Q----A-H--SSGSV- S -W---KQVD------------------
Lysinibacillus manganicus	WP_036185820	--------D--Q----A-H--ESGSV- S -W---K-VD------------------
Lysinibacillus massiliensis	WP_036176785	--------D--Q----A-Q--ESGAV- S -W---K-VD------------------
Lysinibacillus meyeri	WP_107841918	--------D--Q----A-E---TGAVA S -W---KEVD------------------
Lysinibacillus odysseyi	WP_036156597	--------D--Q----A-Q---SGQVA S -W---KEVD------------------
Lysinibacillus parviboronicapi	WP_107923538	--------D--Q----A-H--SSGTV- S -W---KQVD------------------
Lysinibacillus sinduriensis	WP_036201730	--------D--Q----A-H---SGAV- S -W---KEVD------------------
Lysinibacillus sp. 2017	WP_108713374	--------D--Q----A-Q---NGTVA S -W---KEVD------------------
Lysinibacillus sp. B2A1	AVK84141	--------D--Q----A-H--SSGSV- S -W---KQVD------------------
Lysinibacillus sp. Marseille-P	WP_106780127	--------D--Q----A-Q--ESGTV- S -W---K-VD------------------
Lysinibacillus sp. SYSU K30002	WP_126657247	--------D--Q---QA-H---SGAV- S -W---KEVD------------------
Lysinibacillus sp. YLB-03	WP_118875703	--------D--Q----A-H---SGAV- S -W---KEVD------------------
Lysinibacillus sphaericus	WP_010860048	--------D--Q----A-H--SSGTV- S -W---KQVD------------------

Other Bacteria (0/>100)

```
Lysinibacillus telephonicus       WP_126292701   --------D--Q----A-H---SGAV- S -W---KEVD------------------
Lysinibacillus xyleni             WP_097072740   --------D--Q---QA-H---SGAV- S -W---KEVD------------------
Mycobacteroides abscessus subs    SHP93651       -------P---K---DA-EMIE-GKVE S VW---Q-VD-----------T-----T
Natribacillus halophilus          WP_090398334   -----M-----Q----A-RM-DN-QSL T VG---Q-A-------------IL---T
Paenisporosarcina antarctica      WP_134209476   -----------Q---DA-H---SGEVA S -W---K-VD------------------
Paenisporosarcina indica          WP_075620453   -----------Q----A-H---SGEVA S -W---K-VD------------------
Paenisporosarcina sp. HGH0030     WP_016426802   -----------Q---DA-Q---SGEVA S -W---K-VD-----Y------------
Paenisporosarcina sp. OV554       WP_108586421   -----------Q----A-E---SGEVA S -W---K-VD------------------
Paenisporosarcina sp. TG-14       WP_017381195   -----------Q---DA-H---SGEVA S -W---K-VD------------------
Paenisporosarcina sp. TG20        WP_019413879   -----------Q---DA-Q--HSGEVA S -W---K-VD------------------
Psychrobacillus insolitus         WP_111437787   -----------Q---DA-Q---SGKV- S -W---KEVD------------------
Psychrobacillus psychrotoleran    WP_093537018   -----------Q---DA-H---SGKA- S -W---KEVD------------------
Psychrobacillus sp. OK028         WP_093060538   -----------Q----A-H---SGKA- S -W---KEVD------------------
Psychrobacillus sp. OK032         WP_093266243   -----------Q----A-H---SGKV- S -W---KEVD------------------
Pueribacillus theae               WP_116554902   ---------S-R----A-A--ESGKAE Q -N-----V------------S-IA---
Ruegeria sp. NKC1-1               WP_114375847   -----M-----Q----A-R--ENEPST T -G---Q-V-------------IL---T
Rummeliibacillus pycnus           WP_102691202   --------D--Q----A-H---TGAVT S -W---KQVD------------------
Rummeliibacillus stabekisii       WP_066788500   --------D--Q----A-H---TGAV- S -W---KQVD------------------
Salmonella enterica               WP_076927571   -------PD--R---------D-KKAD S VA---Q-AD----------------YT
Scopulibacillus darangshiensis    WP_132742745   -------S-S-L-V-QA-T--E-GKAE S VT---KRV------Y-----GI----T
Solibacillus sp. R5-41            WP_099424673   --------D--Q----A-Q---NGTVA S -W---KEVD------------------
Sporosarcina pasteurii            WP_115361471   ---------S-L--I------ASGEA- T -QE-TKNI------------T----E-
Sporosarcina sp. EUR3 2.2.2       WP_024534386   -----------Q---DA-E---SGEVA S -W---K-VD------------------
Ureibacillus thermophilus         QBK26380       --------D--Q----A-R--ESGKVN S -W---KQVD------------------
Ureibacillus thermosphaericus     WP_016838403   --------D--Q----A-R--ETGKVN S -W---RQVD------------------
Viridibacillus arvi               WP_053416474   --------D--Q---DA-Y---TGAV- S -W---KQVD------------------
Viridibacillus sp. OK051          WP_100794388   --------D--Q----A-Y---TGAV- S -W---KQVD------------------
```

Supplemental Figure 77

A partial sequence alignment of the ACT domain-containing protein containing a one amino acid deletion (boxed) that is exclusively shared by all members belonging to the Planococcus/Planomicrobium clade.

			1009		1053
Planococcus/ Planomicrobium clade (18/18)	Planococcus donghaensis	WP_065525509	MEIARATTEEKETQISNLRAFQERN		DSTEALNRLKQVAKTGGNIF
	Planococcus antarcticus	WP_006829260	---------------G---------		------E----A--------
	Planococcus citreus	WP_121298366	-------K-------N---------		P-D---Q-------E-----
	Planococcus faecalis	WP_078080671	-------I-------G--------H		--D-----------------
	Planococcus halocryophilus	WP_008496882	-------------------------		--------------------
	Planococcus halotolerans	WP_112221229	-------K----A--EK-----D--		N-D---E----A--------
	Planococcus kocurii	WP_058384442	-------I-------G--------H		--S-----------------
	Planococcus maitriensis	WP_112230437	-------K-------N---------		P-D---E---K---D-----
	Planococcus maritimus	WP_083389106	-------K-------N---------		P-D---Q-------E-----
	Planococcus massiliensis	WP_052650666	---------------A---------		A-E-------HA--S-----
	Planococcus plakortidis	WP_068870085	-------K-------N---------		P-D---E---K---E-----
	Planococcus rifietoensis	WP_058382941	-------Q----A--N---S-----		P-D---E---R---------
	Planococcus salinarum	TAA72943	-------K----A--DK-----G--		N-D---E-------------
	Planococcus salinus	WP_123164546	-------K----S--EK--------		P-D---D---H---------
	Planomicrobium glaciei	WP_036809729	---------------A---------		S-E---AH--H---S-----
	Planomicrobium okeanokoites	WP_117313678	-------K-------T---------		--D---D---H-V-------
	Planomicrobium soli	WP_106532830	---------------N------A--		N-E---D-------------
	Planomicrobium koreense	WP_135500804	---------------A---------		A-E---A-------S-----
Other Bacteria (0/>100)	Planomicrobium flavidum	WP_088009458	-Q----SK-------T--------H	GD	KTE-------E---------
	Planococcus sp. Y42	WP_077590191	-Q----GKD------N---G----H	ED	KTG-------E---------
	Alkalibacillus haloalkaliphilus	WP_029425059	--V----Q---QQ--A---------	AD	EAEQ--K------ASE--V-
	Anoxybacillus amylolyticus	WP_066322571	IQL---SY----L--T---------	KD	KVE---K---H--VS-----
	Anoxybacillus geothermalis	WP_044745382	IQL----Y-------R---E-----	KD	KAGP--E------TS-----
	Anoxybacillus sp. P3H1B	WP_066150663	I-L---SY----L--K--Q------	RD	KAE-------HA-VS-----
	Anoxybacillus sp. UARK-01	WP_080862199	I-L---SY----L--K--Q------	RD	KAE-------HA-VS-----
	Anoxybacillus tepidamans	WP_027408050	IQL---SY----L--A---------	KD	KIE---K---H--VS-----
	Anoxybacillus vitaminiphilus	WP_111644771	IQL---SY----L--N---------	KD	KVD---K---H--VS-----
	Bacillus acanthi	WP_108668826	------------G--Q---E-----	KH	AAD---T---EA-MS-----
	Bacillus alkalitelluris	WP_088077088	--L----K----E------K---QH	KE	EAQV--A---E--L----L-
	Bacillus alveayuensis	WP_044749324	IQL---SY----L--N---------	KD	KVD---K------VS-----
	Bacillus aquimaris	WP_113969262	--L----K-------H---D--SQH	KD	K-E---A----A-VD-----
	Bacillus bataviensis	WP_007087452	--L---------L--H-------VH	AG	E-AD--K---EA-VS-----
	Bacillus campisalis	WP_046523790	--L----K----L--A---D--G--	AD	K-E---R----A-VS---V-
	Bacillus canaveralius	WP_125927726	-QL----K----L--N---K-----	SV	HAD---V---KA-V----V-
	Bacillus dakarensis	WP_077215092	--L---SK----L--H---E-----	GD	QTEQ------AT-VS-----
	Bacillus dielmoensis	WP_042463636	--L----Q----L---------TAH	AE	EASA--T----T-TS-----
	Bacillus enclensis	WP_058299455	--L----K-------K---D--A--	ES	-ID---Q---KA-VD-----
	Bacillus firmus	WP_061794032	--L----K-------R---S-----	KD	K-E---KQ--EA-VS-----
	Bacillus foraminis	WP_121610526	------SK---V---A--E--K---	KG	Q-K---AA---T-VS-----
	Bacillus fordii	WP_018708402	I-L----KQ------R---N---N-	KD	VVE---S---K--VS-----
	Bacillus fumarioli	WP_066367454	--L----K----L--Q-------EH	KD	EAQA--Q---E--VS-----
	Bacillus halmapalus	WP_078378591	I-L--S-E-------R--T------	SD	KVE----------IN-E---
	Bacillus horikoshii	WP_064099598	I-L--S-E---Q---Q--A------	AG	EVE---D------IS-----
	Bacillus horneckiae	WP_066398323	--L------------H---S-----	KD	KME---I----T-VS-----
	Bacillus infantis	WP_129613015	-------KG------R---S-----	KD	HAG---S---EA-V-D----
	Bacillus jeotgali	WP_102265199	--L----K-------Q--E--KGK-	KD	Q-E-------EA-VS-----
	Bacillus kochii	WP_095371559	------SK-------R--QL-----	QA	VAQ---Q---E--VS-----
	Bacillus marisflavi	WP_079514240	--L----K-------H--K---QGH	GD	KTE---K----S-VE-----
	Bacillus massiliogabonensis	WP_102273587	--L----K-------Q---S-----	KG	KTDI--KH--ET-VS-----
	Bacillus mediterraneensis	WP_071461016	-------Q-------R--E--KG--	ED	K-A--IA---EA-V------
	Bacillus methanolicus	WP_003351718	--L----K----L--Q---S-----	KD	RAN---I----T-VS-----
	Bacillus muralis	WP_064467086	-------K----G--T--Q------	NQ	FTD---T---AA-MN-D---
	Bacillus oceanisediminis	WP_019380587	--L----K-------R---S--D--	KG	K-E---KQ--EA-VS-----
	Bacillus pseudofirmus	WP_075681864	--L----K----Q--T-------KH	QD	KAP---K--QET-I------
	Bacillus simplex	WP_061141301	-------K----G--K---------	KQ	FTD---T---AA-MN-D---
	Bacillus sp. 17376	ESU34430	--L----K----------ES-KGK-	KD	QTE-------ET-VS-----
	Bacillus sp. 1NLA3E	WP_015596106	------SN----S--T---D--A-H	ND	QVA----------VS---V-
	Bacillus sp. 2_A_57_CT2	WP_009332303	--L----K-------H---S--D--	KD	K-E---KQ--EA-VS-----
	Bacillus sp. 7894-2	WP_095243204	--L----K-------R---S-----	KD	K-E----Q--EA-VS-----
	Bacillus sp. AFS015802	WP_098351478	--L----K-------H---D--AQH	ND	KTE---A----A-VG-----
	Bacillus sp. CHD6a	WP_060664642	I-L--S-E---Q---Q--A------	AA	EVEA--G------IS-----
	Bacillus sp. FJAT-22090	WP_053590545	--L----K-------T---S--KK-	EL	EAA----------M------
	Bacillus sp. FJAT-25496	WP_057772784	--L----Q----I--L---S-----	KD	NTE---K---ET-V------
	Bacillus sp. FJAT-27245	WP_053368411	--L---SK----L--A---E-----	GE	KTAD--K---AT-VS-----
	Bacillus sp. FJAT-27251	WP_053361091	--L----K----L--A---D--G--	AD	K-E--------A-VS---V-
	Bacillus sp. FJAT-29814	WP_066310140	--L---------L--H---E---AH	KD	E--A--K---EA-VS-----
	Bacillus sp. FJAT-29937	WP_066296660	--L----Q-------Q---S-----	KD	KTE--IK---ET-VS-----
	Bacillus sp. FJAT-45037	WP_100374209	--L----K----Q--T--------H	QN	ETEQ--KN-Q-T-IN-----
	Bacillus sp. FJAT-45350	WP_096203020	--L----K----Q--T------D--	KD	ETQT--K--QET-VS-----
	Bacillus sp. HMSC76G11	WP_070877959	------SK-------Q---------	ET	YAA--ID----A-IHNQ---
	Bacillus sp. J33	WP_026583788	--L----K-------R---S-----	KD	KAE---K---ET-VS-----
	Bacillus sp. LF1	WP_090634770	--L---------L--H-------A-	VE	VAAP--K---ET-VS-----
	Bacillus sp. Leaf406	WP_056539241	--L----K-------H--K---QGH	GD	KTE---K----S-VE-----
	Bacillus sp. MKU004	WP_064567856	--L----K-------K---D--A--	ES	-ID---Q---KA-VD-----

Other Bacteria (0/>100)

Bacillus sp. OG2	WP_094769140	-------K-------R---S-----	KD	HAG---S---EA-V-D----
Bacillus sp. SA5d-4	WP_094925133	--L---SK----H--T-------TH	KD	-AEH---S-----VS-----
Bacillus sp. SG-1	WP_006837443	--L----K-------T---S-----	QD	STE---KK--EA-VS-----
Bacillus sp. SKP7-4	WP_119546162	--L----K-------T---S--G--	ED	STE---K---EA-VND----
Bacillus sp. T33-2	WP_101581990	------S--------R--EQ-KS--	KE	TCEQ--A----T-VS-----
Bacillus sp. UMB0728	WP_101548879	-------K-------R---S-----	KG	HAG---S---EA-V-D----
Bacillus sp. URHB0009	WP_027323497	--L----K----M------E--NK-	KD	N-QQ--A---EA-VN-----
Bacillus sp. V33-4	WP_101665803	-QL----K----L--N---K-----	SV	HGD---V---KA-V----V-
Bacillus sp. V47-23a	WP_117325486	-------K----L--A---E---K-	KN	C-V--I-S--AA-VS-----
Bacillus sp. V59.32b	WP_117306371	-------K----G--Q---E-----	RS	HYEA-I-----A-VN-E---
Bacillus sp. es.034	WP_098438699	--L----K-------H---D--SQH	KD	QTE---T----A-VG-----
Bacillus sp. m3-13	WP_010197458	I-L--S-E---Q---R--A------	SA	EVE---S------IS-----
Bacillus sp. mrc49	WP_100531232	-------K----G--K---------	KQ	FTD---T---AA-MN-D---
Bacillus subterraneus	WP_125480256	--L-----------R--ET-KDK-	KD	Q-E---K---ET-VN-----
Bacillus vietnamensis	WP_060674338	--L----Q-------Q---D--SQH	KD	KTE---T----A-VG-----
Bacillus vireti	WP_024030726	-------Q----L--N-----W-AH	GG	E-AD--K---EA-VS-----
Bacillus zeae	WP_119112054	-------K-------R--E--KA-H	DD	LNR-S-D---KA-VS-----
Bhargavaea beijingensis	WP_092097444	--L----K----------KT----H	GD	RT----G----A-VS-----
Bhargavaea cecembensis	WP_063179950	--L----K-------R--KT----H	GD	R-Q---D---KA-V------
Bhargavaea ginsengi	WP_092054564	--L----K-------R--KS-----	GE	RTQ---G---TA-VS-----
Brevibacillus panacihumi	WP_023555161	I-L---------Q--A---------	RD	AAPA--S--QE--MA-----
Brevibacillus sp. CFH S0501	WP_134683388	I-L----E----Q--Q---------	KH	LAPA--K--QE--VS-----
Caldalkalibacillus thermarum	WP_007504624	L-L----K---DQ--A---------	KD	KAPQ--E---ET-RS-----
Caryophanon tenue	WP_066547008	------SN---SL--T------QSH	GD	KTEQ--Q---E-------L-
Chryseomicrobium excrementi	WP_100353372	------S--------T---S--QTH	AD	A-EA--A---D---------
Edaphobacillus lindanitolerans	WP_076756805	--L----K-------R---T---G-	KD	ETA---R----A-VAN----
Effusibacillus pohliae	WP_018132316	V-L----R----L--A---E-----	KD	KAP-M-Q------QS-----
Falsibacillus pallidus	WP_114745876	I-L----K-------Q---S--DA-	KD	ETQA-IDK---T-M------
Filibacter sp. TB-66	WP_124068781	--L----K-------T------RTH	EE	QTAD--KK--E--VS-----
Geobacillus jurassicus	WP_066232337	IQL----Y-------R---E-----	KD	KVGP--E------TS-----
Geobacillus kaustophilus	WP_044732851	IQL----Y-------R---E-----	KD	KAGP--E------TS-----
Geobacillus lituanicus	WP_050367984	IQL----Y-------R---E-----	KD	KAGP--E------TS-----
Geobacillus sp. 12AMOR1	AKM20600	IQL----Y-------R---E-----	KD	KAGP--E------TS-----
Geobacillus sp. 15	KZM55808	IQL----Y-------R---E-----	KD	KAGP--E------TS-----
Geobacillus sp. 44B	WP_081159808	IQL----Y----L--K---E-----	KD	KVEA--E------VS-----
Geobacillus sp. 46C-IIa	WP_081207884	L-L----Y----L--H---E--A--	KD	KAGP--E------VS-----
Geobacillus sp. BC02	KPC99841	IQL----Y-------R---E-----	KD	KAGP--E------TS-----
Geobacillus sp. C56-T3	WP_013146627	IQL----Y-------R---E-----	KD	KAGP--E------TS-----
Geobacillus sp. FJAT-46040	WP_096226043	IQL----Y-------R---E-----	KD	KAGP--E------TS-----
Geobacillus sp. JS12	WP_063193457	IQL----Y-------R---E-----	KD	KAGP--E------TS-----
Geobacillus sp. PK12	WP_129447230	IQL----Y-------R---E-----	KD	KAGP--E------TS-----
Geobacillus sp. T6	WP_047752494	IQL----Y-------R---E-----	KD	KAGP--E------TS-----
Geobacillus sp. WCH70	WP_015865342	IQL----Y----L--K---E-----	KD	KVDA--E------IS-----
Geobacillus sp. WSUCF1	EPR29347	IQL----Y-------R---E-----	KD	KAGP--E------TS-----
Geobacillus sp. Y4.1MC1	WP_013401852	IQL----Y----L--R---E-----	KD	KVE---E------VS-----
Geobacillus stearothermophilus	WP_053414181	IQL----Y-------R---E-----	KD	KAGP--E------TS-----
Geobacillus thermocatenulatus	WP_025949634	IQL----Y-------R---E-----	KD	KAGP--E------TS-----
Geobacillus thermodenitrifican	WP_029761115	L-L----Y----L--R---E-----	KD	KVGP--E------IS-----
Geobacillus vulcani	WP_031406611	IQL----Y-------R---E-----	KD	KAGP--E------TS-----
Geobacillus zalihae	WP_081132711	IQL----Y-------R---E-----	KD	KAGP--E------TS-----
Geobacter metallireducens	WP_004513381	-L----K----Q--A--------H	RN	EAPQ--K--QE--VN-----
Geobacter pelophilus	WP_085813239	-L----A----Q--R---------	KE	KAPL--K--QE--VS-----
Geobacter sp. M21	WP_015836685	-L----Q----Q--K---------	KE	KAPL--K--QE--VS-----
Geobacter uraniireducens	WP_041245316	-L----I----Q--R---------	RV	KAPL--K--QE--VS-----
Halalkalibacillus halophilus	WP_027965181	------SQ---QQ--K---D--K--	ET	EAEV------E--S-D----
Jeotgalibacillus proteolyticus	WP_104058645	--L----K-------E---S--NTH	KD	ETE-------KS-VS---L-
Jeotgalibacillus soli	WP_041087574	--L-----------N---T-HASR	QN	-LA---A---ET-VS---L-
Jeotgalibacillus sp. R-1-5s-1	WP_134376522	--L----K-------A-------QH	AN	EADA--A---ET-V----L-
Kurthia gibsonii	WP_121178206	--V----K----L-LK---T---KH	RN	EAE-Q-KK--H----N----
Kurthia massiliensis	WP_010287104	--V---S----D---M---K--QAH	ED	KAQ-M-Q--QE---S-----
Kurthia sp. 11kri321	WP_068455972	--V----K----L-LK---T---KH	RN	EAE-Q-KK--H----N----
Laceyella sacchari	WP_102993670	I-LT---P---QA--DR-----QAH	RN	EA----E---E--RS---L-
Laceyella sediminis	WP_106341808	I-LT---P---QA--DR-----QAH	RN	EA----E---E--RS---L-
Lentibacillus amyloliquefacien	WP_068446818	--L---SK----H---E-HK---A-	KA	ET-A------D--AS-----
Lentibacillus persicus	WP_090085636	--L---SK----H---E-HK---K-	KD	ETEA------D--AS-----
Lysinibacillus fluoroglycofeni	WP_107942026	-------Q---DL--A--E--KA-H	AN	EAD--IK---HA-V------
Lysinibacillus meyeri	WP_107840504	-------K---DL--A--Q---T-H	TA	QAD--IK----A-V------
Lysinibacillus odysseyi	WP_052124777	-Q----SK---DA--N--KT----H	SD	K---------KA-V------
Lysinibacillus sp. SYSU K30002	WP_126658885	------SK----L--Q---E--K--	ET	AI--S--K--ET-LS---V-
Melghiribacillus thermohalophi	WP_132370727	--L---SK---DK--E---K--KEH	EH	EAK---E------R------
Melghirimyces thermohalophilus	WP_091569563	QLT---E---K---Q--L----KH	RD	QAQ----------RE---L-
Mycobacteroides abscessus subs	SIC43832	-------K-------R---S-----	KD	HAG---S---EA-V-D----
Oceanobacillus halophilus	WP_121204981	--L----K----H--KE--K-----	KA	YTED------E--TS-----
Oceanobacillus sp. YLB-02	WP_121524234	--L----K----H--NE--R---K-	KN	EAEQ---------A-N----

Other Bacteria (0/>100)

Ornithinibacillus californiens	WP_047983943	--L----K-------Q--KK--AQH QD KTEP--KS-----A------
Paenibacillus sp. FSL R5-0490	WP_076257072	--L----K-------R---S----- KD K-E----Q--EA-VS-----
Paenisporosarcina antarctica	WP_134210677	-------S-------R--L-----H SA E-QA--D---E--VS-----
Paenisporosarcina indica	WP_075618392	-------I----S--L--------- SA E-EA--K---E--V------
Paenisporosarcina quisquiliaru	WP_090565673	-------K----------N--QK- DT EAE---A------V------
Paenisporosarcina sp. HGH0030	WP_016428287	---------------L------QL- SV EAEA--K---E--V------
Paenisporosarcina sp. K2R23-3	WP_119882547	--L----K-------------QTH ET ETEA--RK--D--VS---L-
Paenisporosarcina sp. OV554	WP_108586122	-------SD---S--QS------LH AD E-VA--A---E--IS-----
Paenisporosarcina sp. TG-14	WP_017379499	-------S-------R--L-----H SA --QA--D---E--VS-----
Paenisporosarcina sp. TG20	WP_019414985	-------I---DI--A--L------ SS E-QD--K------VS-----
Parageobacillus caldoxylosilyt	WP_061578880	IQL----Y----L--K---E----- KD KVEA--E------VS-----
Parageobacillus genomosp. 1	WP_043906285	IQL----Y----L--K---E----- KD KVEA--E------VS-----
Parageobacillus thermantarctic	WP_090948109	-QL----Y----W--K---E----- KD KVG---E------IS-----
Parageobacillus thermoglucosid	WP_125010742	IQL----Y----L--R---E----- KD KVE---E------VS-----
Parageobacillus toebii	WP_062678149	IQL----Y----L--K---E----- KD KVDA--E------IS-----
Paucisalibacillus globulus	WP_026907745	--L----I----H--QE-K----KH QD QVEP--K------AS-----
Psychrobacillus insolitus	WP_111439298	-------KD--D---V---N--AKS PT ETE----------V------
Psychrobacillus psychrodurans	WP_093493808	-------K----------N--QK- EA EAE---A------V------
Psychrobacillus psychrotoleran	WP_093535781	---------------T---N--QK- EA ETE---A------V------
Psychrobacillus sp. FJAT-21963	WP_056828076	--L----K-------T---S--KK- EL EAA----------M------
Psychrobacillus sp. OK028	WP_093061336	-------K-------T---N--QK- EA ETEK--A------V------
Psychrobacillus sp. OK032	WP_093267814	------------S--T---S--GKH AT EASI--AQ-----V------
Solibacillus isronensis	WP_079524622	------SK---DL--A--AS-K-A- AD N-QK--D---E---------
Solibacillus silvestris	WP_014824736	------SK---DL--A--AG-K-A- AD N-QK--DQ--E---------
Sporosarcina koreensis	WP_060206810	-------R-------A------SQH SD AAE---RK-Q---V------
Sporosarcina sp. EUR3 2.2.2	WP_024535581	-------S-------ES------LH AD EAA---K---V--VS-----
Sporosarcina sp. P17b	WP_099625034	-------Q---QI--------HSTH AD E-EQ--A--QE--V----V-
Sporosarcina sp. P26b	WP_099693633	-------Q---QI--------HSTH AD E-EQ--A--QE--V----V-
Sporosarcina ureae	WP_085428128	-------Q---QI--------HSTH AD E-EQ--A--QE--V----V-
Tetzosporium hominis	WP_094943595	------SK-------T---S--QSH AD A-EA--A---D---------
Thermoactinomyces vulgaris	WP_022737347	I-LT---P---QA--DR-----QAH RN -AM---E---E--RS---L-

Supplemental Figure 78

A partial sequence alignment of the methylmalonyl-CoA mutase protein containing a two amino acid deletion (boxed) that is exclusively shared by all members belonging to the Planococcus/Planomicrobium clade. *Planococcus versutus* does not have a homolog of this protein.

			72		111
**Flavidum Clade (2/2)**	Planomicrobium flavidum	WP_088008171	TKNIFETLVNFGEQDTEVVP	ENM	GLAESWEISEDGLTYTF
	Planococcus sp. Y42	WP_077588385	----------------D---	--N	-----------------
	Aeribacillus pallidus	WP_044899652	-E--Y-----YEDTS-K-I-		---------P-------
	Anoxybacillus flavithermus	WP_003397938	-----D--LDYNDG--SIQ-		A---K--V-Q-------
	Anoxybacillus sp. BC01	KHF29402	-----D--LDYNDG--TIQ-		A---E-T--D-------
	Anoxybacillus sp. P3H1B	WP_066146790	-----D--LDYN-G--S-Q-		----K-D--P-------
	Anoxybacillus sp. UARK-01	WP_080860109	-----D--LDYN-G--S-Q-		----K-D--P-------
	Bacillus abyssalis	WP_078408442	---L-N--IE-------LN-		---KE-SV--------L
	Bacillus acanthi	WP_108670548	----Y---IE--P----LH-		---TE-KA--------L
	Bacillus aquimaris	WP_113970148	---------K--------N-		----E-KV-D-------
	Bacillus bataviensis	WP_007083076	-E------LEY--K--T-Q-		----K--A---------
	Bacillus canaveralius	WP_101576950	--------IE-------IH-		----E-SNTD---K--L
	Bacillus cecembensis	WP_057984574	-Q------L--S-G--T-Q-		---KE-TV---------
	Bacillus coahuilensis	WP_010170947	-E--------------QIE-		N--KE-T----------
	Bacillus cucumis	WP_101647567	-E------IQY-D---TIH-		-------VTP-----K-
	Bacillus dielmoensis	WP_042454935	-E-------SY--K-VTIN-		----K-DV-D-------
	Bacillus drentensis	WP_066259729	-E--Y---LQY-D---TIN-		----K--VTP-------
	Bacillus endophyticus	WP_061802473	-Q---D--IL--K-N--LE-		---TE-ST--------L
	Bacillus filamentosus	WP_026009437	-Q---D--IL--K-N--LE-		---TE-ST--------L
	Bacillus firmus	WP_035333116	----Y---IE-------IQ-		---TE--T-----KH-L
	Bacillus foraminis	WP_121611504	-E------LEY-----TIH-		----E-NV-P-N-----
	Bacillus fumarioli	WP_066370240	-I----N---Y-K---TLH-		---K---V-P-------
	Bacillus gobiensis	WP_053602138	-Q------LEY-----TIQ-		---KK--V-D-------
	Bacillus infantis	WP_129613409	-V------I--------IQ-		---TE--E-----KH-L
	Bacillus jeotgali	WP_102261613	----Y---IE-------IH-		-------E-----KH-L
	Bacillus kochii	WP_095369998	---------E-------LH-		---TD--T------Q-L
	Bacillus korlensis	WP_066056369	--------IE-------LA-		---TE-TN--------L
	Bacillus litoralis	WP_121662751	-E-------EY-----TLH-		-------V-K-A----L
	Bacillus marinisedimentorum	WP_070121082	---V-D--IDY-D----IQ-		A---E-DA--------L
	Bacillus marisflavi	WP_121618967	---------D--Q---TIH-		---KE-DV-S-------
	Bacillus massiliogabonensis	WP_102273193	--------I--------IEA		---TE--T-----KH-L
	Bacillus massiliogorillae	WP_042351738	--------IQ--DK--SLQ-		---KE-SV-D---K--M
	Bacillus mediterraneensis	WP_071458755	-E--Y---I--------IE-		------KE-D---KH-L
	Bacillus mesonae	WP_066393931	-I----Q-L-Y-----TIH-		----K--V-D-------
	Bacillus ndiopicus	WP_042479012	-Q-L----L----E--TIH-		---TE-D----------
	Bacillus oceanisediminis	WP_110066772	----Y---I--------IEA		---TE--T-----KH-L
	Bacillus oleivorans	WP_097158427	----Y----E--P----LY-		---TE-TP-D------L
**Other Bacteria (0/>200)**	Bacillus praedii	WP_057760479	-D-L----I--------IE-		---TE--T-----KH-L
	Bacillus rubiinfantis	WP_042356898	-I-L----L-Y-----TIQ-		----K--V-D-A-----
	Bacillus selenatarsenatis	WP_041967597	----Y---IE-------IH-		----D--E-----KH-L
	Bacillus sonorensis	WP_006639847	-T------L-Y-----TLH-		---VK--A-D------L
	Bacillus sp. 17376	WP_023625550	----Y---IE-------IH-		-------E--G--KH-L
	Bacillus sp. 7504-2	WP_095314062	-V-LY---I--------IH-		---TE-ST------H-L
	Bacillus sp. AFS006103	WP_098263502	-E--Y---LQY-D---TIN-		----K--VTP-------
	Bacillus sp. AFS040349	WP_098796013	-E-------EY-----TLH-		-------V---A----L
	Bacillus sp. AFS073361	WP_098576261	-E------IQY-D---TIN-		-------VTP-------
	Bacillus sp. B-jedd	WP_048823596	-E--YD-------S--TTQ-		---------D-S-----
	Bacillus sp. B14905	WP_008173493	-A--Y----------VTIQ-		---K---A---------
	Bacillus sp. CGMCC 1.16541	WP_110114311	-Q---D--IS----S-DLE-		---TE-NA--------L
	Bacillus sp. EB01	WP_043934807	-E-------QY-D---TIN-		----K--V-D-------
	Bacillus sp. FJAT-14578	WP_028397065	---L-N--IE-------LN-		---KE-SV--------L
	Bacillus sp. FJAT-18017	WP_053601809	-E-------QY-D---TIN-		----K--V-D-------
	Bacillus sp. FJAT-22090	WP_053592032	-Q-V----L-------TINE		---TK--P---------
	Bacillus sp. FJAT-25496	WP_057774715	-E------LEY-D---T-N-		----K-TV-D---S---
	Bacillus sp. FJAT-27225	WP_066204034	-E-------QY-D---TIN-		----D-KV-D-------
	Bacillus sp. FJAT-29814	WP_066305433	-E-M-----E------TIH-		-------P---------
	Bacillus sp. J33	WP_026581717	----Y---IE-------IQ-		---TE--T-----KH-L
	Bacillus sp. J37	WP_026558831	-E------LEY-----TLH-		-------V-D-A----L
	Bacillus sp. LF1	WP_090633313	-Q--------Y-V---TLH-		----K-DV-P-------
	Bacillus sp. Leaf406	WP_056534717	-V---------------PN-		----K-DVA--------
	Bacillus sp. MUM 116	WP_071355830	-Q------LEY-P---TIH-		---K---V-P-------
	Bacillus sp. NRRL B-14911	EAR64270	-V------I--------IQ-		---TE--E-----KH-L
	Bacillus sp. OG2	WP_094769932	-V------I--------IQ-		---TE--E-----KH-L
	Bacillus sp. OxB-1	WP_041071224	---V----L-------TIH-		---TE--P---------
	Bacillus sp. P14.5	WP_113929690	---------E-------IQA		---KE-DVA--------
	Bacillus sp. SG-1	WP_006836300	---------K--------QA		---KE-DV---------
	Bacillus sp. SJS	WP_035405981	-E--Y-S-LSY-DE--T-Q-		----K--A-D-------
	Bacillus sp. SKP7-4	WP_119547622	---------K--------QA		---KE-DV---------
	Bacillus sp. UNC41MFS5	WP_026564379	-E--Y---IQY-D---TIN-		-------VTP-------
	Bacillus sp. UNC438CL73TsuS30	WP_026573471	-E-V----LDY-P---I-K-		----K--V-K-------
	Bacillus sp. URHB0009	WP_027320991	-D----N-LSY-P---T-Q-		----K--VTP-------
	Bacillus sp. V-88	WP_079531083	---------K--------N-		----K--V-D---N-K-
	Bacillus sp. V3-13	WP_101662011	--------IE-------IH-		----E-SNT----K--L
	Bacillus sp. V33-4	WP_101665396	--------IE-------IH-		----E-SNTD---K--L

Other Bacteria (0/>200)

Bacillus sp. WN066	WP_133337364	-E------LDY-P---V-Q-	----K--V-K-------
Bacillus sp. X1(2014)	WP_038539868	-E-------EY-D---TIN-	----K--V---------
Bacillus sp. Y1	WP_119707032	--------IE-------LA-	---TE-SN--------L
Bacillus sp. es.034	WP_098441922	---------K--------N-	----K--V-D---N-K-
Bacillus subterraneus	WP_044394077	----Y---I--------IQ-	-------E-A---KH-L
Bacillus vietnamensis	WP_061810825	---------K--------N-	----K--V-D---N-K-
Bacillus vireti	WP_024029132	-Q------LVY-----TIH-	----E-NT-D-------
Bacillus weihaiensis	WP_072579221	-E------IEY-----TLH-	------KV---A-----
Bacillus zeae	WP_119113238	-E-VY---I--------IE-	----K--E-A---KH-L
Bhargavaea beijingensis	WP_092095848	AA------I-------TLQ-	---KE--A--------L
Bhargavaea cecembensis	WP_082194096	AA------L-------TIN-	---KE--AA--------
Bhargavaea ginsengi	WP_092054743	AA------I-------TLQ-	---KE--A--------L
Brevibacillus agri	WP_005833136	-E---D---GYA-ES-----	S---K----P-------
Brevibacillus borstelensis	WP_003390708	-E--Y----SYEDTN-T-I-	-----------------
Brevibacillus gelatini	WP_122904625	-E-V-D---GYA-ES-----	S---K----P-------
Brevibacillus invocatus	WP_122907225	-E--YD---GYE-ES-K---	S---K--V-P-------
Brevibacillus nitrificans	WP_122925521	-E-V-D---GYE-ES-A---	S---K----P-------
Brevibacillus panacihumi	WP_031305380	-E---D---GYE-ES-K---	S---K------------
Brevibacillus parabrevis	WP_063231367	-E-V-D---GYA-ES-----	S---K----P-------
Brevibacillus reuszeri	WP_103109076	-E-V-D---GYA-ET-----	S---K---AP-------
Brevibacillus sp.	REK64633	-E--Y----SYEDTS-K-I-	---------P-------
Brevibacillus sp. CFH S0501	WP_134687514	-E---D---SYE-EN-N---	--------AP-------
Brevibacillus thermoruber	WP_029097332	-E--Y-----YEDTS-K-I-	---------P---I---
Butyricicoccus sp. 1XD8-22	RKJ50876	-E------LE------T-H-	---KE--V---------
Caldibacillus debilis	WP_120667370	-E--Y-K-I--K--S--LE-	---TD--V-D-------
Chryseomicrobium excrementi	WP_100354529	-Q-V------------TIN-	---KE-TV---------
Edaphobacillus lindanitolerans	WP_076760057	AA------L-------TIH-	---KE--A---------
Falsibacillus pallidus	WP_114747240	---------E--R----IHE	---TK-DV-P-------
Falsibacillus sp. GY 10110	WP_121682318	---------E--K---QIHE	---TK-DV-D-------
Fictibacillus sp. BK138	WP_130297061	-EQ-Y-P---Y-KDN-DI--	---KK-D----------
Filibacter sp. TB-66	WP_124069283	-V-LY---L-------TIQ-	---KE--P---------
Jeotgalibacillus alimentarius	WP_041121275	-------I-E-------IN-	----E--V---------
Jeotgalibacillus campisalis	WP_041056114	---V----IE--RE---IN-	---TD--V---------
Jeotgalibacillus malaysiensis	WP_039807230	-------I-E-------IN-	----E--V---------
Jeotgalibacillus proteolyticus	WP_104057388	---V----IE--KE----N-	---AE-DV---------
Jeotgalibacillus salarius	WP_134380640	---------E-------IN-	----E--V---------
Jeotgalibacillus sp. R-1-5s-1	WP_134372335	-----D--IF-------IQ-	---AD--V---------
Jeotgalibacillus sp. S-D1	WP_133377720	---------E--RE---IN-	---AE---A--------
Kurthia gibsonii	WP_121176612	--------I-----N-DL--	---SK--T-KN--E---
Kurthia huakuii	WP_029498410	-Q------IR------TLQ-	---KE-KV-D-------
Kurthia sp. 11kri321	WP_068454136	--------I-----N-DL--	---SK--T-KN--E---
Kurthia sp. 3B1D	WP_126990675	-Q------IR------TLQ-	---KE-KV-D---E---
Kurthia sp. Dielmo	WP_020189586	-Q------IR------TLQ-	---KE-KV-D---E---
Kurthia zopfii	WP_109350228	----M---I----GS--L--	----K--T-K-------
Lysinibacillus boronitolerans	WP_036076983	-A--Y--------R-VTIQ-	---K---A---------
Lysinibacillus chungkukjangi	WP_107937119	-E------L-------TIN-	---HD--V-----E---
Lysinibacillus contaminans	WP_053582024	-A-LY----------VTIN-	---K---P---------
Lysinibacillus endophyticus	WP_121215743	-E------LE------TIQ-	S--KE--V-D-------
Lysinibacillus fluoroglycofeni	WP_107943669	-Q-L----L-------TIH-	---TK-D--D-------
Lysinibacillus fusiformis	WP_096365875	-A--Y----------VTIQ-	---K---A---------
Lysinibacillus halotolerans	WP_122971954	-E------L-------T-Q-	---KE--V---------
Lysinibacillus macroides	WP_053995537	-G--Y----S--QK-MTIN-	-------P---------
Lysinibacillus mangiferihumi	WP_107897504	-V--Y---I------VTLK-	---K--GA-D-------
Lysinibacillus massiliensis	WP_036177973	-E------LE------T-H-	---KE--V---------
Lysinibacillus meyeri	WP_107840160	-Q-L----L-------AIH-	---KE-S--D-------
Lysinibacillus odysseyi	WP_036150585	-V-LY---L-------TIQ-	----E--V-----E-K-
Lysinibacillus parviboronicapi	WP_107924663	-A--Y----------VTIH-	---K---P---------
Lysinibacillus sinduriensis	WP_036197697	-E------LS--DE--TIN-	---KE-TV---------
Lysinibacillus sp. 2017	WP_108713935	-Q------L--N-E--T-KA	---KE--V---------
Lysinibacillus sp. B2A1	AVK86180	-A--Y--------R-VTIQ-	---K---P---------
Lysinibacillus sp. BK089	WP_132363328	-V-LY---I------VTLQ-	---K---P--------M
Lysinibacillus sp. FJAT-14222	WP_053597094	-A--Y--------K-VTIQ-	---K---P---------
Lysinibacillus sp. FJAT-14745	WP_053483078	-A--Y--------R-VTIQ-	---K---P-K-------
Lysinibacillus sp. Marseille-P	WP_106781126	-E--Y---L---D---T-N-	---KD--V---------
Lysinibacillus sp. OL1	WP_131521933	-A--Y--------R-VTIQ-	---K---A---------
Lysinibacillus sp. SYSU K30002	WP_126659398	-E------L-------TIN-	---KE--V---------
Lysinibacillus sp. YLB-03	WP_118877044	-E--Y---L-------TIN-	---KE--V---------
Lysinibacillus sp. YR326	WP_134025969	-A--Y--------R-VTIQ-	---K---P---------
Lysinibacillus sp. ZYM-1	WP_054612690	-A--Y-------DR-VTIQ-	---K---A---------
Lysinibacillus sphaericus	WP_125101233	-V-L------------T-Q-	---KE-ST---------
Lysinibacillus tabacifolii	WP_108031110	-V--Y---I------VTLK-	---K---A---------
Lysinibacillus telephonicus	WP_126295933	-E--Y---L-------TIH-	---KE-KV---------
Lysinibacillus varians	WP_025220605	-V--Y---I------VTLK-	---K---A---------
Lysinibacillus xylanilyticus	WP_100545164	-A--Y--------K-VTIQ-	---K---P---------

Other Bacteria (0/>200)

Lysinibacillus xyleni	WP_097072673	-E------L-------TIH-	---KE--V---------
Mycobacteroides abscessus subs	SHT32332	---V----IE-------LA-	---TE-TN--------L
Numidum massiliense	WP_054951409	----YD---DY-----TIHE	A--TE-DV-D-------
Paenibacillus sp. FSL R5-0490	WP_076260352	----Y---IE-------IQA	---TK--M-----KH-L
Paenisporosarcina antarctica	WP_134209106	-Q-L----L-------TINE	---TK--P---------
Paenisporosarcina indica	WP_075618985	---L--------D----IHE	---K--VP-----S---
Paenisporosarcina quisquiliaru	WP_090568496	-Q-VL---L-------TINE	---TK--P---------
Paenisporosarcina sp. HGH0030	WP_016429228	-Q-V--------K----IN-	---KE-KP---------
Paenisporosarcina sp. K2R23-3	WP_119884529	---V--------D----IH-	---KE--A-D-------
Paenisporosarcina sp. OV554	WP_108587816	-Q-V----L---DK--TINE	---TK--P---------
Paenisporosarcina sp. TG-14	WP_017381156	-Q-L----L-------TINE	---TK--P---------
Planococcus antarcticus	WP_006830862	-----D-----------IE-	A--SD-TA---------
Planococcus citreus	WP_121301209	-----D--I--------IEA	---SD-QA---------
Planococcus donghaensis	WP_065526934	-----D-----------IE-	---SE-TP---------
Planococcus faecalis	WP_071153286	-----D-----------IEG	A--SK--P-----S---
Planococcus halocryophilus	WP_008497131	-----D-----------IE-	---SE-TP---------
Planococcus halotolerans	WP_112224973	-----D-----------IEG	---SD--AA--------
Planococcus kocurii	WP_058385999	-----D-----------IEG	A--SK--P-----S---
Planococcus maitriensis	WP_112233841	-----D--I--------IEA	---SE--AA--------
Planococcus maritimus	WP_068461187	-----D--I--------IEA	---SD-QAE--------
Planococcus massiliensis	WP_052652375	-----D-----------IH-	A--TE--AA--------
Planococcus plakortidis	WP_068868627	-----D--I--------IEA	---SD--AA--------
Planococcus rifietoensis	WP_058381343	-----D--I--------IEA	---SD--A---------
Planococcus salinarum	TAA71741	-----D------A----IE-	---SEYSA---------
Planococcus salinus	WP_123166640	-----D--I--------IE-	---TD--AA-------L
Planococcus sp. CAU13	WP_033541594	-----D-----------IE-	A--SD-TAE------V-
Planococcus sp. PAMC 21323	WP_038703249	-----D-----------IEA	---SE-TP---------
Planococcus versutus	WP_065524406	-----D-----------IEA	---SK--P---------
Planomicrobium glaciei	WP_036805790	-----D-----------IH-	---TE--AA--------
Planomicrobium okeanokoites	WP_117313940	-----D-----------IE-	---SEYTAA--------
Planomicrobium soli	WP_106534800	-----D-----------IH-	---KE-TAAK-----N-
Planomicrobium sp. MB-3u-38	WP_101803531	-----D-----------IE-	---SEYTAA--------
Planomicrobium sp. Y74	WP_121636664	-----D-----------IE-	---SEYTAA--------
Psychrobacillus psychrodurans	WP_093495064	-Q-VL---L-------TINE	---TK--P---------
Psychrobacillus psychrotoleran	WP_093538179	-Q-VQ---L-------TINE	---TK--P-----S---
Psychrobacillus sp. FJAT-21963	WP_056833283	-Q-V----L-------TINE	---TK--P---------
Psychrobacillus sp. OK028	WP_093062831	-Q-VL---L---P---TINE	---TK--P-----S---
Psychrobacillus sp. OK032	WP_093276228	-Q-V----L---K---TINE	---TK--A---------
Rhizophagus irregularis	PKC50709	-E------LE------T-H-	---KE--V---------
Rummeliibacillus pycnus	WP_102693970	-Q------I---K---TLQ-	---TK--T-K-------
Rummeliibacillus sp. POC4	WP_119414491	-Q-V----I-------KLQ-	N--TK--A---------
Rummeliibacillus sp. TYF005	WP_124217753	-Q-V----I-------KLQ-	N--TK--A---------
Rummeliibacillus stabekisii	WP_066789703	--------IE---E--TLH-	---TK--A-K------L
Salinibacillus kushneri	WP_093135598	--------L-Y-----TIQ-	---KE--V-D-------
Scopulibacillus darangshiensis	WP_132746999	----YD---EYKG-T-D-G-	------D--D-------
Solibacillus isronensis	WP_079523616	-Q-L----L-------TIN-	---KE--V---------
Solibacillus silvestris	WP_014822718	-Q-V----L-------TIN-	---KE--V---------
Solibacillus sp. R5-41	WP_099425246	-Q-L----L---P---TIH-	---K--TV-D---S---
Sporosarcina koreensis	WP_040285829	-V-L------------TIQ-	---KE--TTD-------
Sporosarcina newyorkensis	WP_078818414	-V-L----L-------T-Q-	---KE--P---------
Sporosarcina psychrophila	WP_067205827	-V--Y---L----S---IN-	---TE--P---------
Sporosarcina sp. BI001-red	WP_116020181	-V-L---------E--TIQ-	---KE--P---------
Sporosarcina sp. D27	WP_025782775	-V-L---------S--T-Q-	---KE-KT---------
Sporosarcina sp. EUR3 2.2.2	WP_024533886	-Q-V----L----E--TINE	---TK--P---------
Sporosarcina sp. P33	WP_081242811	-V-L----L-------T-Q-	---KEY-T---------
Sporosarcina sp. PTS2304	WP_114924583	-V-L----L-------T-Q-	---KE--P---------
Sporosarcina sp. ZBG7A	WP_039042819	-V-L------------T-Q-	---KE-ST---------
Tetzosporium hominis	WP_094944781	-Q-V------------TIN-	---KE-TV---------
Ureibacillus thermosphaericus	WP_016837859	-I--Y-----------TIH-	-------V---------
Viridibacillus arvi	WP_053418807	-E-LY-K-I-------TLN-	---K---AA----S---
Viridibacillus sp. OK051	WP_100795872	-E-LY-K-I-------TLN-	---K--KAAK---S---

Supplemental Figure 79

A partial sequence alignment of the ABC transporter substrate-binding protein containing a 3 amino acid insertion (boxed) that is exclusively shared by all members belonging to the Flavidum clade and absent in all other bacteria.

Group	Organism	Accession	125		165
**Flavidum Clade (2/2)**	Planomicrobium flavidum	WP_088008409	YEGWYCTPCESFYTESQLDE	H	GHCPDCGRPVHKVKEESYFF
	Planococcus sp. Y42	WP_077587581	------------F-------	-	Q-------------------
**Other Bacteria (0/>200)**	Alkalibacillus haloalkaliphilu	WP_017185063	------------F-DR--E-		-N--------E---------
	Amphibacillus marinus	WP_091497679	------------F--R----		-K-----G--ER--------
	Anoxybacillus ayderensis	WP_085788858	---------------R--V-		-N--------E----Q----
	Anoxybacillus flavithermus	WP_041637542	---------------R--V-		-N--------E----Q----
	Anoxybacillus flavithermus WK1	ACJ32416	---------------R--V-		-N--------E----Q----
	Anoxybacillus sp. P3H1B	WP_082741731	---------------R--VD		-N--------E---------
	Anoxybacillus sp. UARK-01	WP_080859998	---------------R--VD		-N--------E---------
	Anoxybacillus thermarum	WP_043967573	---------------R--V-		-N--------E----Q----
	Anoxybacillus vitaminiphilus	WP_111646242	---------------R--VD		-N--------E---------
	Aquisalibacillus elongatus	WP_124222921	------------F-DR--ED		-N--------E---------
	Bacillus acanthi	WP_108672142	------------F--R-VEN		-N--------E---------
	Bacillus acidicola	WP_066267815	------------F--R--VN		-N--------E---------
	Bacillus acidiproducens	WP_018663637	------------F--R--VD		-K--------E---------
	Bacillus alveayuensis	WP_044747316	------------F--R--V-		-N--------E---------
	Bacillus aquimaris	WP_071618367	------------F--R--EN		-N--------E---------
	Bacillus boroniphilus	WP_035209872	--------------DR--ED		-N--------E---------
	Bacillus campisalis	WP_046525190	--------------DR--EN		-N--------E---------
	Bacillus coagulans	WP_061574354	------------F--R--VD		-K--------E---------
	Bacillus enclensis	WP_058300015	------------F--R--EN		-N--------E---------
	Bacillus firmus	WP_035332956	-----------Y---R--AD		-N------------------
	Bacillus foraminis	WP_132011504	--------------DR--EG		-N--------E---------
	Bacillus fumarioli	WP_066369996	--------------DR--I-		-N--------E---------
	Bacillus ginsengihumi	WP_025730353	------------F--R--VD		-K--------E---------
	Bacillus indicus	WP_029286035	------------F--R--EN		-N--------E---------
	Bacillus jeotgali	WP_102264790	--------------DR--ED		-N--------E---------
	Bacillus kochii	WP_095371296	-----------YF--T--V-		-N---------R--------
	Bacillus kwashiorkori	WP_062350068	------------F--R--N-		-N--------E---------
	Bacillus lentus	WP_066146154	------------F--I--EN		-N--------E---------
	Bacillus marinisedimentorum	WP_070121526	------------F-DR--N-		-S--------E---------
	Bacillus massilioanorexius	WP_019244523	------------F--R-IEN		-N--------E---------
	Bacillus massiliogabonensis	WP_102273321	------------F--R--EN		-N--------N---------
	Bacillus methanolicus	WP_003351519	--------------DH--VD		-N--------E---------
	Bacillus niameyensis	WP_062107799	------------F--R--VN		-N--------E---------
	Bacillus oceanisediminis	WP_110067680	-----------Y---R--SD		-N------------------
	Bacillus oleivorans	WP_097160774	------------F--R--AD		-N--------E---------
	Bacillus onubensis	WP_099353530	------------F--R--AD		-N--------E---------
	Bacillus praedii	WP_057767543	------------F--R---N		-N------S-N---------
	Bacillus selenatarsenatis	WP_041968000	--------------DR--ED		-N--------E---------
	Bacillus sp. 17376	WP_023613329	--------------DR--ED		-N--------E---------
	Bacillus sp. 1NLA3E	WP_015591886	-----------Y---I--V-		-N--------Q---------
	Bacillus sp. 2_A_57_CT2	WP_009336673	-----------Y---R--VD		-N------------------
	Bacillus sp. 7894-2	WP_095246036	-----------Y---R--AD		-N------------------
	Bacillus sp. AFS015802	WP_098353710	------------F--R--EN		-N--------E---------
	Bacillus sp. B-jedd	WP_048827593	--------------DR--V-		-N--------E---------
	Bacillus sp. B14905	WP_008181620	------------F--T--VD		-N------------------
	Bacillus sp. FJAT-22090	WP_053591101	------------F--T--VD		-N------------------
	Bacillus sp. FJAT-27225	WP_066207249	--------------DR--V-		-N--------E---------
	Bacillus sp. FJAT-27231	WP_049666011	------------F--R--EN		-N--------E----Q----
	Bacillus sp. FJAT-27245	WP_053368665	--------------DR--V-		-N--------E---------
	Bacillus sp. FJAT-27251	WP_053361249	--------------DR--EN		-N--------E---------
	Bacillus sp. FJAT-27986	WP_066103195	------------F-DR-VE-		-N--------E---------
	Bacillus sp. FJAT-44742	WP_100400531	----------------H--ED		-K--------E--R------
	Bacillus sp. HMSC76G11	WP_070875711	------------F--R--EN		-N--------E---------
	Bacillus sp. J33	WP_026585207	-----------Y---R--TD		-N------------------
	Bacillus sp. MKU004	WP_064563558	------------F--R--EN		-N--------E---------
	Bacillus sp. OxB-1	WP_041072467	-----------YF--G--ED		-N--------Q---------
	Bacillus sp. P14.5	WP_113928167	------------F--R--EN		-N--------E---------
	Bacillus sp. PK3_68	WP_120035801	------------F--R--EN		-N--------E----Q----
	Bacillus sp. SKP7-4	WP_119548690	------------F--R--ED		-K--------E---------
	Bacillus sp. UMB0893	WP_101569451	------------F--R---N		-N--------E---------
	Bacillus sp. V3-13	WP_101662606	--------------DR--EN		-N--------E----A----
	Bacillus sp. X1(2014)	WP_038538256	--------------DR--V-		-N--------G---------
	Bacillus sp. es.034	WP_098438564	------------F--R--EN		-N--------E---------
	Bacillus subterraneus	WP_125481636	--------------DR--ED		-N--------E---------
	Bacillus taeanensis	WP_113808137	------------F--L----		-N--------E---------
	Bacillus thermocopriae	WP_128999512	---------------R--VD		-N--------E----Q----
	Bacillus vietnamensis	WP_034762948	------------F--R--EN		-N--------E---------
	Bacillus vireti	WP_024026430	----------A---DR--V-		-N--------E---------
	Bacillus wudalianchiensis	WP_065409966	------------F--R--EN		-N--------E----Q----
	Bhargavaea cecembensis	WP_063183955	-----------YF--A---D		-N------------------
	Bhargavaea ginsengi	WP_092056031	------------F-DA--ED		-N------------------
	Butyricicoccus sp. 1XD8-22	RKJ65648	---------------T--EN		-N------------------

Other Bacteria (0/>200)

Caenibacillus caldisaponilytic	WP_077614550	---------------H--VD	-K--------E---------
Clostridium tetani	WP_130004608	------------W------D	HN--------E-T---A---
Desmospora activa	WP_107728455	------------W--R--KD	-N--------E--R------
Edaphobacillus lindanitolerans	WP_076760168	-----------YF-----VD	-N--------E---------
Faecalitalea cylindroides	WP_081459249	------------W-----VD	-K--------K-A---A---
Fictibacillus aquaticus	WP_094253271	----------A-F--R--ED	-N--------K---------
Filibacter sp. TB-66	WP_124068639	-----------YF-----EN	EN--------K---------
Filobacillus milosensis	WP_134340191	------------F-DR--ED	-N--------E---------
Geobacillus jurassicus	WP_066231060	---------------R--VD	-N--------E---------
Geobacillus sp. Y4.1MC1	WP_013399804	---------------R--VD	-N--------E---------
Geobacillus stearothermophilus	WP_033014212	---------------R--VD	-N--------E---------
Geobacillus vulcani	WP_031406343	---------------R--V-	-N--------E---------
Halalkalibacillus halophilus	WP_027964437	------------F-DR--ED	-N--------E---------
Halalkalibacillus sp. B3227	WP_101332315	------------F-DR--ED	-K--------E---------
Halobacillus alkaliphilus	WP_089753617	-------S----F--R----	-------G--E---------
Halobacillus halophilus	WP_014641348	-------S----F--R----	-------G--E---------
Halobacillus hunanensis	WP_079524843	------------F--R----	-------G--E---------
Halobacillus mangrovi	WP_085027027	-------S----F--R----	-------G--E---------
Halobacillus salinus	WP_079477418	-------S----F--R---D	-------G--E---------
Halobacillus sp. BBL2006	WP_035542962	-------S----F--R----	-------G--E---------
Halobacillus sp. Marseille-P38	WP_101847165	------------F--R--N-	-------G--E---------
Kurthia huakuii	WP_029501090	------------F--T--VD	-N---------T--------
Kurthia massiliensis	WP_010291168	------------F--T--ED	-N------------------
Kurthia senegalensis	WP_010309151	------------F--T--ED	-N------------------
Kurthia sibirica	WP_109307104	---L--------F--A-VEN	-N---------T--------
Kurthia sp. 11kri321	AMA61806	------------F--M--VD	-N--------N---------
Kurthia sp. 3B1D	WP_126988931	------------F--T--VD	-N------------------
Kurthia zopfii	WP_109350116	------------F--M--VN	-N-------------Q----
Lentibacillus amyloliquefacien	WP_068446235	-------S----F--R---D	-------A--E---------
Lentibacillus jeotgali	WP_010531230	-------S----F--R---D	-------N--E---------
Lentibacillus sp. Marseille-P4	WP_106495620	-------S----F--R---D	-------G--E---------
Lysinibacillus acetophenoni	WP_097151204	---------------T--EN	-N------------------
Lysinibacillus boronitolerans	WP_036075703	------------F--T--VD	-N------------------
Lysinibacillus chungkukjangi	WP_107936284	---------------T--EN	EN------------------
Lysinibacillus composti	WP_124766662	---------------T---N	-N------------------
Lysinibacillus contaminans	WP_053582343	------------F--T--VD	-N------------------
Lysinibacillus endophyticus	WP_121215687	---------------T--AD	-N------------------
Lysinibacillus fusiformis	WP_069483067	------------F--T--VD	-N------------------
Lysinibacillus halotolerans	WP_122972295	---------------T--VD	-N------------------
Lysinibacillus macroides	WP_053997053	------------F--T--VD	-N------------------
Lysinibacillus manganicus	WP_036189096	---------------T---N	-N------A-----------
Lysinibacillus mangiferihumi	WP_107896449	------------F--T--ED	-K------A-----------
Lysinibacillus massiliensis	WP_036179618	---------------T--EN	-N------------------
Lysinibacillus parviboronicapi	WP_107950831	------------F--T--V-	-N------------------
Lysinibacillus sinduriensis	WP_036197880	--------------DT--ED	-N------------------
Lysinibacillus sp. AR18-8	WP_066038155	------------F--T--VD	-N------------------
Lysinibacillus sp. B2A1	AVK82142	------------F--T--EN	-N------------------
Lysinibacillus sp. BK089	WP_132364293	---S--------F--T--VD	-N------------------
Lysinibacillus sp. FJAT-14222	WP_053594097	------------F--T--VD	-N------------------
Lysinibacillus sp. FJAT-14745	WP_053485312	------------F--T--VD	-N------------------
Lysinibacillus sp. Marseille-P	WP_106782136	---------------T--EN	-N------------------
Lysinibacillus sp. OL1	WP_131522705	------------F--T--VD	-N------------------
Lysinibacillus sp. SG9	SCZ08594	------------F--T--VD	-N------------------
Lysinibacillus sp. SYSU K30002	WP_126660162	---------------T---N	-N------D-----------
Lysinibacillus sp. YLB-03	WP_118877708	---------------K--ED	-N------------------
Lysinibacillus sp. YR326	WP_134025810	---S--------F--T--VD	-N------------------
Lysinibacillus sp. YS11	WP_103117245	------------F--T--VD	-N------------------
Lysinibacillus sp. ZYM-1	WP_054612528	------------F--T--VD	-N------------------
Lysinibacillus sphaericus	WP_010857016	------------F--T--V-	-N------------------
Lysinibacillus tabacifolii	WP_108031369	------------F--T--ED	-K------A-----------
Lysinibacillus telephonicus	WP_126295486	---------------T--VD	-N------------------
Lysinibacillus varians	WP_025218326	------------F--T--ED	-K------A-----------
Lysinibacillus xylanilyticus	WP_049667351	---S--------F--T--VD	-N------------------
Lysinibacillus xyleni	WP_097075266	---------------T--VD	-N---------T--------
Mageeibacillus indolicus	WP_012992864	----------A-W-----KD	-C----------T-------
Melghiribacillus thermohalophi	WP_132372738	------------F-DR--E-	-N--------E---------
Melghirimyces thermohalophilus	WP_091572032	------------W--R--E-	-N--------E--R------
Natranaerovirga hydrolytica	WP_132278712	----------T----R----	-N----N-K-E---------
Paenibacillaceae bacterium GAS	WP_090777458	------------F--R---K	-N--------EL--------
Paenibacillus alvei	WP_040735921	------------F--R---N	-N--------EL--------
Paenibacillus sp. FSL R5-0490	WP_076262367	-----------Y---R--AD	-N------------------
Paenisporosarcina antarctica	WP_134211684	--------------DN---N	-N------------------
Paenisporosarcina indica	WP_075620353	---L-------YF--T--EN	-N--------N---------
Paenisporosarcina sp. HGH0030	WP_016429967	------------F-----EN	-N--------K---------

Other Bacteria (0/>200)

Paenisporosarcina sp. K2R23-3	WP_119884422	------------F--K--VD	-N--------N---------
Paenisporosarcina sp. OV554	WP_108587500	-----------YF-----ED	-N--------N---------
Paenisporosarcina sp. TG-14	WP_017380249	--------------DK---N	-N------------------
Paenisporosarcina sp. TG20	WP_019414132	--------------DK---N	EN------------------
Paludifilum halophilum	WP_094265781	------------W--R--E-	----------E--R-Q----
Parageobacillus caldoxylosilyt	WP_017434462	---------------R--VD	-N--------E---------
Parageobacillus thermantarctic	WP_090949734	---------------R--VN	-N--------E---------
Parageobacillus thermoglucosid	RDE29914	---------------R--VD	-N--------E---------
Parageobacillus toebii	WP_062677420	---------------R--VD	-N--------E---------
Piscibacillus halophilus	WP_091772964	------------F-DR--ED	-N--------E---------
Planococcus antarcticus	WP_065536332	------------F--N--EN	-N------------------
Planococcus citreus	WP_121301355	------------F-----ED	-N------------------
Planococcus donghaensis	WP_065527620	------------F--N--EN	-N--------Q---------
Planococcus faecalis	WP_071153224	------------F--N--EN	-N------------------
Planococcus halocryophilus	WP_008498579	------------F--N--EN	-N--------Q---------
Planococcus halotolerans	WP_112225040	-------------------N	-N------------------
Planococcus kocurii	WP_058386793	------------F--N--EN	-N------------------
Planococcus maitriensis	WP_112233987	------------F-----ED	-N------------------
Planococcus maritimus	WP_068459548	------------F-----ED	-N------------------
Planococcus massiliensis	WP_052649524	---------------A--VD	-K------------------
Planococcus plakortidis	WP_068871224	------------F-----ED	-N------------------
Planococcus rifietoensis	WP_058382237	------------F-----ED	-N------------------
Planococcus salinarum	TAA69702	------------------EN	-N------------------
Planococcus salinus	WP_123166464	---------------G---N	-A------------------
Planococcus sp. CAU13	WP_033543415	---------------G--EN	-N------------------
Planococcus sp. PAMC 21323	WP_038705294	------------F--N--EN	-N--------Q---------
Planococcus versutus	WP_049695096	------------F--N--EN	-N------------------
Planomicrobium glaciei	WP_074511524	---------------A--VD	DN------------------
Planomicrobium okeanokoites	WP_084242245	-------------------N	-K------------------
Planomicrobium soli	WP_106534679	---L-----------A--VD	-N------------------
Planomicrobium sp. MB-3u-38	WP_101803427	--------------V----N	-K------------------
Planomicrobium sp. Y74	WP_121636605	-------------------N	-N------------------
Pontibacillus marinus	WP_027447145	------------F--R---N	-------G--E---------
Pontibacillus yanchengensis	WP_036822673	-------S----F--R----	-------N--E---------
Psychrobacillus insolitus	WP_111440836	------------------ED	-N------------------
Psychrobacillus psychrotoleran	WP_093538629	------------F--T--V-	-N--------Q---------
Psychrobacillus sp. FJAT-21963	WP_056833330	------------F--T--VD	-N------------------
Psychrobacillus sp. OK028	WP_093063113	------------F--T--I-	-N--------Q---------
Psychrobacillus sp. OK032	WP_093276538	---S--------F-----VD	-N------------------
Pueribacillus theae	WP_116554304	---------------T--VD	-K--------E---------
Quasibacillus thermotolerans	WP_039234775	------------F--L---N	-N--------ER---Q----
Rummeliibacillus pycnus	WP_102693394	------------F-DT---N	-N---------R--------
Rummeliibacillus stabekisii	WP_066791273	------------F--T---N	-K------------------
Salimicrobium flavidum	WP_076560554	-------S----F--R----	-------G--E---------
Salirhabdus sp. Marseille-P466	WP_102029480	------------F--R--EN	-N--------E---------
Sporosarcina globispora	WP_053433254	-----------Y---R--VD	-N------------------
Sporosarcina psychrophila	WP_067213756	------L---TYF--G--EN	-N--------K---------
Sporosarcina sp. EUR3 2.2.2	WP_037578292	-----------YF-----ED	-N--------N---------
Sporosarcina sp. PTS2304	WP_114925292	----K-V----YF-------	-N--------QR-E------
Tenuibacillus multivorans	WP_093855092	------------F-DR--ED	-N--------E---------
Thermolongibacillus altinsuens	WP_132949377	---------------R--VD	-N--------E---------
Ureibacillus thermophilus	QBK24771	-----------Y---T--VD	-N--------Q-L-------
Ureibacillus thermosphaericus	WP_016838865	------------F--T--ID	-N--------Q-L-------
Vallitalea guaymasensis	WP_113673041	------------F--R--ED	-K--------D-----A---
Virgibacillus subterraneus	WP_092506326	-------S----F--R---D	-------G--E---------
Viridibacillus arvi	WP_053418386	------------F------N	-N------------------
Viridibacillus sp. OK051	WP_100795422	------------F-----EN	-N------S-----------

Supplemental Figure 80

A partial sequence alignment of the methionine--tRNA ligase containing a 1 amino acid insertion (boxed) that is exclusively shared by all members belonging to the Flavidum clade and absent in all other bacteria.

Group	Organism	Accession	70		129
Flavidum Clade (2/2)	Planomicrobium flavidum	WP_088008611	PNIALAEGELDLNAFQTISYFDAFMEEHNID	N	VVPIGSTVIAPMGLYSDKYESVEELPEG
	Planococcus sp. Y42	WP_077590293	---------------------E--K---GL-	-	I--------------E----L------
Other Bacteria (0/>100)	Bacillus andreraoultii	WP_033826508	--Q---Q--I---S--H-AFLKQ-KK--KL-		L----T-------I--E--KD-ADI-K-
	Bacillus aquimaris	WP_032086661	--Q---D-DI-----------S-V----L-		L------I-----I--E--K--DD--N-
	Bacillus aurantiacus	WP_026691670	--M------V-F---------N-I----L-		LE--AG-Y------------H-DI---
	Bacillus butanolivorans	WP_083450632	--E-----DI-I----HVA-L-S-NAKNDT-		I----T-I---L----S--K----I-N-
	Bacillus camelliae	WP_101355446	--T--ND-S---------T-MNQ-KK---L-		ITA------------H--KKLSDI-N-
	Bacillus cihuensis	WP_028392563	--L------I-A-S--------E-INKQKL-		LEA---------A---K---KL------
	Bacillus clausii	WP_035205273	--Q---D--I-A---------H-I---DL-		LTA-----L-------T--D--DDI-DS
	Bacillus coahuilensis	WP_010174751	--M---D-DV-------V------IK--SL-		LA---T-I-----V--EE-SN-ADI-D-
	Bacillus enclensis	WP_058299608	--Q---D-DI-----------S-V----L-		L------I-----I--E--K--DD--N-
	Bacillus endophyticus	WP_113753175	--Q--S--DI-V----HVA-L-T-N-KNHT-		I----T-I---L----N--KDLKQ--N-
	Bacillus firmus	WP_035329252	--V-----D--V-----VA---T-IK---M-		LS--AT--L-------E-VK---DI---
	Bacillus glycinifermentans	WP_048353489	--M--SS-DI-A---------NS-KK-R-L-		LS---T-I-----I---H-K--KDI-D-
	Bacillus gottheilii	WP_080845925	--T-------------VA---V-I----L-		L---AT--L----I------D-NDI-D-
	Bacillus halmapalus	WP_078380500	--L---QK---A---------N--IK--DL-		LT--AT------------DN--DI---
	Bacillus haynesii	WP_043926466	--Q---G-DI-A---------N--KK-R-LE		LS---T-I-----I--E--G-PDDI-N-
	Bacillus horneckiae	WP_066397691	----------V-----VA---T-I----L-		LS--AT--L-------------DDI---
	Bacillus indicus	WP_029284348	--L--S-----A---------N--KK---L-		LT---T-----L-------K---DI---
	Bacillus jeotgali	WP_079507688	--L--------A-S---V------IK---L-		L---AT-Q-----I-----KD-KDI---
	Bacillus licheniformis	WP_134972933	--Q---S-DI-A---------N--KK-R-LE		LS-V-T-I-----I--E--D-PGDI-N-
	Bacillus loiseleuriae	WP_049682905	--L------I-A-S---V----E-IKKQKL-		LEA--A----------K--KKL------
	Bacillus marisflavi	WP_048005338	--L--E-----A---------NE-KK--DL-		LT---T-----L----E--K--DDI-D-
	Bacillus massiliogabonensis	WP_102273828	----------A-----VA---S-IK---L-		L---AT--L----I--E-FK--DDI---
	Bacillus massiliosenegalensis	WP_019155167	--T--------V-----VA---V-IK---L-		L---AT--L----I--E----D-KDI---
	Bacillus megaterium	WP_116515599	--Q------I-V----HVA-L-S-NQKNGT-		I----T-L---L----E--K-LKDI-N-
	Bacillus muralis	WP_064465474	--L--S---I-A-S---VA---E-IQDQ-LK		LEA------------K-HK-IKDI-D-
	Bacillus niacini	WP_045518364	--T--------V-----VA---V-IK--KL-		L---AT--L----I--E--KD-KDI-D-
	Bacillus notoginsengisoli	WP_118919391	--L--------A-S---V---N--IK--KL-		L---AT-Q--------E--KD-K-I-K-
	Bacillus oceanisediminis	WP_110064582	--V-----D--A-----VA---T-I----M-		LS--AT--L-------E-VT-PD-I---
	Bacillus paralicheniformis	WP_096747972	--Q---G-DI-A---------N--KK-R-LE		LS---T-I-----I--E--D-PDDI-N-
	Bacillus patagoniensis	WP_078392532	--Q---D--I-A-----V----H-I----Q-		LSA-----L-------S-----DDI-D-
	Bacillus polygoni	WP_088036085	--Q---D--I-I-----V----H-K-Q--L-		L----A-YL---------HD-P-DI---
	Bacillus praedii	WP_057763362	----------A-----VA---S-IK--KL-		L---AT--L----I--E--K--DDI---
	Bacillus psychrosaccharolyticu	WP_040373510	--V-----D--A-S---V---N--IK-R-L-		LA-VAT----------E-HK-I-DI-D-
	Bacillus shacheensis	WP_059105965	--L--D-----A-----V----N-V---HLS		-EA--------------R-----DI-D-
	Bacillus shackletonii	WP_055738114	--T--ND-S---------T-MNQ-KQD--L-		ISA-------------N--KNLSDI-N-
	Bacillus simplex	WP_034304170	--L--S---I-A-S---VA---E-VQDQ-LK		LEA------------K-HK-L-D--D-
	Bacillus sonorensis	WP_006636576	--M---S-DI-A---------NS-KK-R-L-		LS-V-T-I-----I---H-K--KDI-D-
	Bacillus sp. 7504-2	WP_095309633	--T--------V-----VA---V-I---DL-		L---AT--L----I--E--KD-NDI-D-
	Bacillus sp. 7884-1	WP_095246500	--T------I-V-----VA---V-IK--KL-		L---AT--L----I--E--KD-KDI-D-
	Bacillus sp. 7894-2	WP_095243472	--V----DD--V-----VA---T-IK---M-		LT--AT--L-------E-VK---DI---
	Bacillus sp. AFS002410	WP_098121738	--T--ND-S---------T-MNQ-KKD--L-		ISA----------I--H--KNLNDI---
	Bacillus sp. AFS015802	WP_098350479	--Q---D-DI-----------S-V----L-		L------L-----I-AE--K--D--K-
	Bacillus sp. AFS017274	WP_098370545	--A--E--DI-I----HVA-L-S-NAKNDT-		I----T-I---L----S--K----I-N-
	Bacillus sp. AFS017274	WP_098372064	--L--S---I-A-S---VA---E-VQDQ-LK		LEA------------K-HKNLKD--D-
	Bacillus sp. AFS043905	WP_098863891	--L--S---I-A-S---VA---E-VQDQ-LK		LEA------------K-HK-LKD--D-
	Bacillus sp. AFS043905	WP_098864673	--E-----DI-I----HVA-L-S-NANN-T-		I----T-I---L----S--K----I-N-
	Bacillus sp. B-jedd	WP_048826478	--L--------V-S---V------IK--KL-		L---AT-Q-----I--E--KD-KDI-K-
	Bacillus sp. BA3	WP_101223329	--L--S---I-A-S---VA---E-VQDQ-LK		LEA------------K-HKDLKD--D-
	Bacillus sp. EB01	WP_043932359	--T------V-I-----VA---T-I----V-		L---AT--L-------E--KDIK-I---
	Bacillus sp. FJAT-18017	WP_053598456	--T------V-I-----VA---T-I----V-		L---AT--L-------E--KD-K-I-D-
	Bacillus sp. FJAT-20673	WP_063575212	--L--S---I-A-S---VA---E-VQDQ-LK		LEA------------K-HK-LKD--D-
	Bacillus sp. FJAT-20673	WP_082854785	--E-----DI-I----HVA-L-S-NANN-T-		I----T-I---L----S--K----I-N-
	Bacillus sp. FJAT-21352	WP_053536239	--L--S---I-A-S---VA---E-VQDQ-LK		LEA------------K-HK-LKD--D-
	Bacillus sp. FJAT-26652	WP_053355378	--Q--SD-DI-A---------NS-KK--KL-		LA--AT-------V--K--KNIKDI-A-
	Bacillus sp. FJAT-27225	WP_066198365	--T------V-I-----VA---E-I----V-		L---AT--L-------E--KD-KDI-K-
	Bacillus sp. FJAT-27245	WP_053367645	--L--------A-S---V------IK--KL-		L---AT-Q-----I--E--KD-KDI-K-
	Bacillus sp. FJAT-27445	WP_059173545	--L--------A-S---V---N--IK--KL-		L---AT-Q--------Q--KD-KDI-K-
	Bacillus sp. FJAT-44876	WP_096187804	--M---D--I-I---------EN-IN--SL-		L---A--YL----M--E-HP-PN-I---
	Bacillus sp. HMSC76G11	WP_070874840	--L--S-----A---------N--KK--DL-		LS---T-----L----E--K--NDI---
	Bacillus sp. J33	WP_026583507	--V-----D--A-----VA---T-I----M-		LS--AT--L-------E-VT-A--I---
	Bacillus sp. JCM 19035	WP_054794296	--Q--EG--V-I-----V----H-K----L-		LTA-----L----------D---DI-D-
	Bacillus sp. JCM 19045	GAF13069	--Q--EG--V-I-----V----H-K----L-		LTA-----L----------D---DI-D-
	Bacillus sp. JCM 19046	WP_035440130	--Q--EG--V-I-----V----H-K----L-		LTA-----L----------D---DI-D-
	Bacillus sp. JCM 19047	WP_035392911	--Q---D-SI-I-----V-F--N-K----L-		LTA-----L--------------DI-D-
	Bacillus sp. KQ-3	WP_122899761	--M---D--V-F---------N-I----L-		LE--AG-Y------------H--I---
	Bacillus sp. LL01	WP_047971488	--L---QK---A---------N--IK--DL-		LS--AT----------E---N--DI---
	Bacillus sp. Leaf13	WP_056522381	--E-----DI-I----HVA-L-S-NAKNDT-		I----T-I---L----S--K----I-N-
	Bacillus sp. Leaf406	WP_056533828	--L--E-----A---------NE-KK--DL-		LT-V-T-----L----E--K--DDI-D-
	Bacillus sp. MKU004	WP_064566582	--Q---D-DI-----------S-V----L-		L------I-----I--E--K--DD--N-
	Bacillus sp. Marseille-P3661	WP_102347558	--M---D-DITI-----V---N--I----L-		L----T-I----------T--D-I-D-
	Bacillus sp. Marseille-P3800	WP_099303007	--Q---D-SI-I-----V-F--N-R---DL-		LAA-----L----------DN-DDI-D-
	Bacillus sp. OG2	WP_094768505	----------A---------N--KK--DL-		LS--AT-------I--E--KD-KDI---

Other Bacteria (0/>100)

Bacillus sp. OK085	WP_132091594	--T--------V-----VA---V-IK--KL-	L---AT--L----I--E-HKD-KDI-D-
Bacillus sp. OK838	WP_089362834	--E-----DI-I----HVA-L-S-NANN-T-	I----T-I---L----S--K----I-N-
Bacillus sp. P14.5	WP_113927248	-----------A---------N--KK--DL-	L---AT-------I-----K-AD-I---
Bacillus sp. RJGP41	WP_106026236	--L--S---I-A-S---VA---E-VQDQ-LK	LEA-------------K-HK-L-D--D-
Bacillus sp. SAJ1	WP_126647298	--T--ND-S---------T-MNQ-KKD--L-	IAA-------------N--KNLSDI-N-
Bacillus sp. SG-1	WP_006836087	-----------A---------N--KK--DL-	LA--AT-------I--E------DI---
Bacillus sp. SKP7-4	WP_119549046	-----------A---------N--KK--DL-	LT--AT-------I--E--K-AD-I---
Bacillus sp. Soil531	WP_057274656	--Q------I-V----HVA-L-S-NQKNGT-	I----T-I---L----E--K-LKDI-N-
Bacillus sp. Soil745	WP_082609436	--E-----DI-I----HVA-L-S-NANN-T-	I----T-I---L----S--K----I-N-
Bacillus sp. T33-2	WP_101580361	--T--------V-----VA---V-IK--KL-	L---AT--L----I-----KD-KDI-Q-
Bacillus sp. UMB0893	WP_101567974	--L--------A---------N--KK-----	LS---T-----L----E--K-IK-I---
Bacillus sp. XXST-01	WP_126408632	--T--------V-----VA---V-IK---L-	L---AT--L----I-----KD-KDI-D-
Bacillus sp. YLB-04	WP_115451802	--L--------A-S---V---N--IK---L-	L---AT-Q-----------KD-KDI-K-
Bacillus sp. YSP-3	WP_110521741	--M---D--V-F----------N-I----L-	LE--AG-Y-------------H--I---
Bacillus sp. es.034	WP_098439444	--Q---D-DI------------S-V----L-	L------I-----I--E--K---D--K-
Bacillus sp. mrc49	WP_100533375	--L--S---I-A-S---VA---E-IQDQ-LK	LEA-------------K-HK-IKDI-D-
Bacillus subterraneus	WP_125481059	--L--------A-S---V------IK--KL-	L---AT-------I-----KD-KDI---
Bacillus subtilis group	WP_041817169	--Q---G-DI-A---------N--KK-R-LE	LS---T-I-----I--E--D-PDDI-N-
Bacillus swezeyi	WP_076761513	--Q---S-DI-A---------NT-KK-RGL-	LS---T-I-----V-----Q--GDI-D-
Bacillus testis	WP_050613917	--T--------A-----VA---Q-IK--KL-	L---AT------AIF-E--KK--DI-D-
Bacillus vietnamensis	WP_034756406	--Q---D-DI------------S-V----L-	LI-----I-----I--E--K---D--K-
Caldibacillus debilis	WP_020156839	--Q---DK-I------H-AFLNQ-K---HL-	I----T-------I--E--KD-S-I-D-
Cohnella thermotolerans	WP_027093895	--L---NK-V-I----HLAFLSQ-NV---LN	I-------V--L----K--K--D-I-D-
Exiguobacterium acetylicum	WP_050676974	--M------I-A---------N--KK---LK	LS--AT-------I-----KD-KDI-T-
Exiguobacterium antarcticum	WP_014971437	--L------I-A-S-------N--KK---LK	LS--AT-------I----VKD-KDI-V-
Exiguobacterium enclense	WP_058264514	--M------I-A---------N--KK--KLK	LS--AT-------I-----KD-KDI-T-
Exiguobacterium sibiricum	WP_012371582	--L------I-A-S---V---N--KK--KLK	LS--AT-------I----VKD-KDI-V-
Exiguobacterium sp. B203-G5 25	TCK77285	--L------I-A-S---V---N--KK--KLK	LS--AT-------I----IKD-KDI-V-
Exiguobacterium sp. BMC-KP	WP_053454428	--M------I-A---------N--KK--KLK	LS--AT-------I-----KD-KDI-V-
Exiguobacterium sp. KRL4	WP_071499930	--L------I-A-S-------N--KK--DLK	LS--AT-------I----VKD-KDI-V-
Exiguobacterium sp. Leaf196	WP_056064144	--M------I-A---------N--KK---LK	LS--AT-------I-----KD-KDI-V-
Exiguobacterium sp. RIT341	WP_035410708	--M------I-A---------N--KK--KLK	LS--AT-------I-----KD-KDI-V-
Exiguobacterium sp. RIT452	WP_119923994	--L------I-A-S-------N--KK--DLK	LS--AT-------I----VKD-KDI-V-
Exiguobacterium sp. RIT594	WP_114597351	--L------I-A-S---V---N--KK--KLK	LS--AT-------I----VKD-KDI-V-
Exiguobacterium sp. U13-1	WP_069940047	--M------I-A---------N--KK--KLK	LS--AT-------I-----KD-KDI-V-
Exiguobacterium sp. ZWU0009	WP_047394453	--M------I-A---------N--KK--KLK	LS--AT-------I-----KD-KDI-V-
Exiguobacterium undae	WP_028105552	--L------I-A-S-------N--KK---LK	LS--AT-------I----VKD-KDI-V-
Fictibacillus enclensis	WP_061967558	--Q---D-DI-A-S-------NS-KK--KL-	LA--AT-------V--E--KDIKDI-D-
Fictibacillus solisalsi	WP_090231878	--Q---D-DI-A-S-------NS-KR--KL-	LA--AT-------V--E--KDIKDI-D-
Fictibacillus sp. S7	WP_129477385	--Q---D-DI-A-S-------NS-KK--KL-	LA--AT-------V--E--KDIKDI-D-
Fonticella tunisiensis	WP_133627984	--M------I-A------A--NK-K-D--L-	LTSL-Y--L----M--K-IK-LS--KD-
Halobacillus alkaliphilus	WP_089753932	--M---D-DI-------V----S-IK--DL-	LA---T-L-----I--EE--D-KNI---
Halobacillus dabanensis	WP_075038247	--M---D-DI-------V----S-I---DL-	LA---T-L-----V--E---D-SNI---
Halobacillus halophilus	WP_014645161	--M---D-DI-------V----S-IK--DL-	LA---T-L-----I--E---D-TNI---
Halobacillus salinus	WP_079477703	--M---D-DI-------V------I---DL-	LA-V-T-L-----I----F-D--QI---
Halobacillus sp. BBL2006	WP_035546048	--M---D-DI-------V----S-IK---L-	LA---T-L-----I--E---D-NNI---
Jeotgalibacillus campisalis	WP_041056250	-----S----------------D-I--REL-	LA--A-------A---E-V--I----D-
Jeotgalibacillus proteolyticus	WP_104057329	-------N--------------E-I--REL-	LS------L---A---E-V-------D-
Jeotgalibacillus sp. R-1-5s-1	WP_134375715	--V-----DI------------E-I-DR-L-	LA--A-------A---E-VS--D-----
Jeotgalicoccus nanhaiensis	WP_135097435	--T---D--I---S---VA---E-I--R-L-	L---AT-NL----I---SLTD-NDI-D-
Jeotgalicoccus saudimassiliens	WP_035810085	--T---D--I---S---VA---E-I--R-L-	L---AT-NL----I---SLTD-NDI-D-
Lentibacillus halodurans	WP_090240430	--L--------A-----V------I---DL-	LA--AT----------E--D-P------
Lentibacillus sediminis	WP_100011730	--L------I-A-----V------I---DL-	LT--AT----------E-HS----I---
Lentibacillus sp. Marseille-P4	WP_106495665	--L------I-V-----V------IK--DL-	LT--AT----------E--D--DD--D-
Oceanobacillus manasiensis	WP_042220842	--L--S---I-A------------I---DL-	LT--AT----------E-H-T--DI---
Oceanobacillus sp. 160	WP_114915901	--Q--V--DI-I----HVA-L-S-N-NTASE	I----T-I---L-------D--D-I-D-
Paenibacillus sp. FSL R5-0490	WP_076257485	--V-----D--V-----VA---T-IK---M-	LT--AT--L-------E-VK---DI---
Paenisporosarcina sp. OV554	WP_108586042	---------I-A----------E-KADQKL-	LEA-A-----------K-HKAL-D--D-
Paenisporosarcina sp. TG20	WP_019414795	---------I-A----------E-KTAQ-L-	IEA----------I--K-HTAL-D--D-
Piscibacillus halophilus	WP_091772765	--L---D-DV-I-----V------I---DL-	LE--AT-I-----I--E-----D-I---
Planococcus antarcticus	WP_006830883	--L-------------------E-V----V-	I-------------------AID-I---
Planococcus citreus	WP_121298550	--L-------------------E-V----V-	I--------------------I------
Planococcus donghaensis	WP_065525417	--L-------------------E-VA-----	I--------------------I----Q-
Planococcus faecalis	WP_071153129	--L-------------------E-VA--S--	I--------------------IDDI---
Planococcus halocryophilus	WP_008496774	--L-------------------E-VA-----	I--------------------I------
Planococcus halotolerans	WP_112221718	----------------------E-V------	I---------------E--D-ID-----
Planococcus kocurii	WP_058384341	--L-------------------E-VA-----	I--------------------IDDI---
Planococcus maitriensis	WP_112230271	--L-------------------E-V----V-	I--------------------I------
Planococcus maritimus	WP_068464526	--L-------------------E-V----V-	I--------------------I----D-
Planococcus massiliensis	WP_052650547	--L-------------------E-VA--D--	I--------------------ID---N-
Planococcus plakortidis	WP_068870262	--L-------------------E-V----V-	I---------------E--D-I----D-
Planococcus rifietoensis	WP_058382840	--L-------------------E-V----V-	I--------------------I------
Planococcus salinarum	TAA73302	----------------------E-VA--DL-	I---------------E---------D-
Planomicrobium glaciei	WP_036805630	--M-------------------E-VT--KL-	I---------------E--KT-D---D-

Group	Species	Accession	Sequence	Sequence
Other Bacteria (0/>100)	Planomicrobium okeanokoites	WP_117312893	---------------------E-V------	I--------------E--D-ID-----
	Planomicrobium soli	WP_106533936	-----G---------------E-VA--KL-	I-------------------L------
	Planomicrobium sp. MB-3u-38	WP_101801196	---------------------E-V------	I--------------E--D-ID-----
	Planomicrobium sp. Y74	WP_121635180	---------------------E-V------	I--------------E--D-ID---D-
	Pontibacillus chungwhensis	WP_036783443	--L---D-DI-------V----S-IK--DL-	LE---T-L-----V--EE-DNL-NI---
	Pontibacillus halophilus	WP_026800165	--M---D-DI-------V----Q-IKD-DL-	LA---T-L-E---I---E--D-SNI-D-
	Pontibacillus marinus	WP_027446016	--L---D-DI-------V------IK--DL-	LA---T-L-----I--E--KD-G-I---
	Rhodococcus qingshengii	WP_133368919	--T------I-V-----VA---V-IK--KL-	L---AT--L----I--E--KD-KDI-D-
	Rubeoparvulum massiliense	WP_048600283	--L-----DI-A-----Y---QS-TS--HLS	LT-LAT--L----I---H-QN-ADI---
	Salimicrobium salexigens	WP_076571205	--M---D-DI-------V----S-I--R-L-	LE--AT-I-----V--EE--D-KNI---
	Salinibacillus kushneri	WP_093131869	--L--SN--I-M-----V----S-I--R-L-	LE--AT-L------------NPADI-D-
	Sporosarcina globispora	WP_053433698	--V-----D--V-----VA---T-IK--DM-	LS--AT--L-------E-VK---DI-D-
	Sporosarcina sp. EUR3 2.2.2	WP_024535487	---------I-A----------E-K-DQ-L-	LEA-A-----------K-HKAL-D--D-
	Tenuibacillus multivorans	WP_093855255	--L---D-DI-------V----S-I---DL-	LA--A--Y-----I--------D-I---
	Tuberibacillus sp. Marseille-P	WP_085522516	--L---A-DI-M-----V----S-IK-RDL-	LK--AT-I----------FK-PKNI-D-
	Vibrio vulnificus	WP_133350405	--L--S---I-A-S---VA---E-VQDQ-LK	LEA-------------K-HK-LKD--D-
	Virgibacillus chiguensis	WP_073010609	--L------I-A-----V------IK--DL-	LT--AT----------E-HGTP-HI-K-
	Virgibacillus dokdonensis	WP_101933101	--L------I-A-----V------IK--DL-	LT--AT----------E-HGTT-DI-K-
	Virgibacillus halodenitrifican	WP_019377802	--M------I-A-----V------IK--EL-	LT--AT----------E-HD---SI---
	Virgibacillus ndiopensis	WP_099158948	--L--------A-----V------IK---L-	L---AT----------E-HDA--D----
	Virgibacillus necropolis	WP_089531754	--L------I-A-----V----S-IK--DL-	L---AT-------------D-P--I---
	Virgibacillus pantothenticus	WP_077298732	--L------I-A-----V------IK--DL-	LT--AT-----L----E-HDKP-DI-K-
	Virgibacillus proomii	WP_077317633	--L------I-A-S-------EN-IK--DL-	LA--AT----------E-HD-P-DI-D-
	Virgibacillus senegalensis	WP_053219266	-----------A---------N--KK--DL-	LA--AT-L-----I----------V---
	Virgibacillus sp. Bac330	WP_121641127	--L------I-A-----V------IK--DL-	LT--AT----------E-HGTP-DI-K-
	Virgibacillus sp. LJ137	WP_123314771	--M---A--I-A-----V------IK--DL-	L----T----------E-HDD-SDI---
	Virgibacillus sp. SK37	WP_040953852	--M------I-A-----V------IK--EL-	LT--AT----------E-HDR--SI---

Supplemental Figure 81

A partial sequence alignment of the MetQ/NlpA family ABC transporter substrate-binding protein containing a 1 amino acid insertion (boxed) that is exclusively shared by all members belonging to the Flavidum clade and absent in all other bacteria.

Group	Species	Accession	122		182
**Flavidum Clade (2/2)**	Planomicrobium flavidum	WP_088005980	KGQTDTIAVNFSRIDIGWFNQIPIIGDLYWTSI		LAIVLAFIAWGIIYYTPFGLRLRAVGEH
	Planococcus sp. Y42	WP_077589601	-----I-S----Q-R-SFLRD------M----L		---AI---S-AV----------------
**Other Bacteria (0/>200)**	Anoxybacillus amylolyticus	WP_066323979	-----Q-Q-G-DKM--PVLSH--V--P-FFANG	YIPSY	I--A----V-Y-LFK-------------
	Anoxybacillus flavithermus	WP_099668997	-----QVQ-G-DK---PILSK--V--PILFSNG	YLPSY	-------VV-Y---K-------------
	Anoxybacillus kamchatkensis	WP_019418464	-----QVQ-G-DK---PILSK---L-PILFSNG	YIPSY	-------VV-YV--K-------------
	Anoxybacillus pushchinoensis	WP_091699935	-----QVQ-G-DK---PILSK--VL-PILFSNG	YLPSY	-------VV-YV--K-------------
	Anoxybacillus sp. BCO1	WP_042894118	-----QVQ-G-DK---PILSK---L-PILFSNG	YIPSY	-------VV-YV--K-------------
	Anoxybacillus suryakundensis	WP_055441098	-----QVQ-G-DK---PILSK--V--PILFSNG	YLPSY	-------VV-YV--K-------------
	Anoxybacillus tepidamans	WP_027408750	-----Q-QIG-DK--VPILSH-----P-FFSNG	YIPSY	I--A---VV-Y-LFK-------------
	Anoxybacillus thermarum	WP_043966516	-----QVQ-G-DK---PILSK--VL-PILFSNG	YVPSY	-------VV-YV--K-------------
	Anoxybacillus vitaminiphilus	WP_111643787	-----Q-Q-G-DK---PVLSD-----P-LFENG	YIPSY	I--A---VV-Y--FK--------S----
	Bacillus acanthi	WP_108669880	-----S-QEL-EK--LPVLSK--VL-KIFFSNG	YYVSY	-------VV-FV--K------I-S----
	Bacillus acidicola	WP_066264576	-----F-VN--KQV--PVLKH------IFF-NV	TYPSY	---IM-VVV-Y---K--------S----
	Bacillus alveayuensis	WP_044893708	-----Q-QIG-DK---PLLSD-----P-LFKNG	YIPSY	I------VV-YV-FK--------S----
	Bacillus amyloliquefaciens	WP_071347700	-A---K-PEP-YKTN-PGLSD--VL-KIFFSDV	YYTSI	-------VS-F-LFK------I-S----
	Bacillus anthracis	WP_098758304	-----F-QYRIEK--VPGLSD-----KIFFSNV	PLTSY	I--I---VV-Y---K-------------
	Bacillus aquimaris	WP_113968165	-----I-SEG-GKV-VPLL-K--V---IFFSNT	YWPPF	V--LVSV-V-F-MFK--------S----
	Bacillus atrophaeus	WP_106359949	-A---K-PEP-YKTN-PGLSD--VL-KIFFSDV	YYTSI	--------S-F-LFK------I-S----
	Bacillus badius	WP_063383554	-----I-K---KKV--PVLSD--V--K-IFSN-	SYPAI	V--A---V--YVL-K-------------
	Bacillus camelliae	WP_101355720	-----M-VQ--RK---PGLSH-------FFKNV	TYASY	---IIS-VV-YV--K--------S----
	Bacillus canaveralius	WP_101576717	-----I-QKG--KV--PLLSD--LL-E-FFSNT	YYTSY	V---F-----YVMFK-------------
	Bacillus cereus	WP_000008849	-----F-QYRIEK--VPGLSD--V--EIFFSNV	PLTSY	I--I---VV-Y---K-------------
	Bacillus cihuensis	WP_051405077	-----MVTE--D-V--KFLSD--F--KIFFSNG	YYISY	I--I--I-V-YV-FK------I------
	Bacillus circulans	WP_123257478	----G--RYS-KK---PLLKD--V----FFSNT	YWTSY	----V-I-V-FVM-K--------S----
	Bacillus cucumis	WP_101647466	--E--I-SQG-NK---PVLKD--V--KIFFQNT	YWTSF	---IV-I-V-YVVFK--------S----
	Bacillus cytotoxicus	WP_048724461	-----F-QYRIEK--VPGLSDV-V--KIFFS-V	PVTSY	I--I----V-Y---K-------------
	Bacillus deserti	WP_101639969	--E--SVT-T-PKV-VPLLSD--VL-P-FFSDA	YYTSY	I--A--VL--YV--K-S------S----
	Bacillus dielmoensis	WP_042456473	--E-GI-SKG--K---PVLKD--FL-KIFFQNT	YWTSF	----V-----Y-VFK--------S----
	Bacillus farraginis	WP_058002713	-----F-KE--RK---PFLSD--V----FFSGV	TYASY	---I-S-VV-FV--K--------S----
	Bacillus firmus	WP_035329089	-----I-QKG--KV--PFLSD--V--K-FFSNT	YYTSF	V--AV--L--FVMFK-------------
	Bacillus fumarioli	WP_066364888	--E--I-QKG-NK---PVLKD-----KIFFQNT	YWVSF	---IV-I---Y---K--------S----
	Bacillus gobiensis	WP_053603980	-----K-P-P-YKVN-PYLSE--F--KILFSDV	YYTSV	------I---Y---K------I-S----
	Bacillus gottheilii	WP_080849193	-----I-QKG--K---PVLSD--V--E-FFSNT	YYTSF	V--FV-V---Y---K-------------
	Bacillus halosaccharovorans	WP_078434291	-----R-STS-NKM--PLLSD--V--KIFFSGG	YITSY	---I--V-V-YV--K--------S----
	Bacillus halotolerans	WP_024122669	-A---K-PEP-YKTS-PGLSD--VL-KIFFSDV	YYTSI	--------S-F-LFK------I-S----
	Bacillus horneckiae	WP_066393738	-----I-QKG--KV--PLLSD-----KIFFSNT	YYTSY	V--IV-I---YV-FK--------S----
	Bacillus koreensis	WP_053401648	-----F-Q-RIDK--VPVLSK--V----FFK-V	PVTSY	I--A---LV-Y---K--------S----
	Bacillus korlensis	WP_066053465	-----I-QKG--K---PVLSD---L-K-FF-NT	YYTSF	V--IV-----FVMFK-------------
	Bacillus lentus	WP_066139494	-------LE-IGK---PLLSD-----K-FFSNV	SYMAY	V--L-S-VV-F-LFK--------S----
	Bacillus licheniformis	WP_075748980	-A---K-PEP-YKTN-PGLSN---L-KIFFSDV	YYTSI	---A----S-F-LFK------I-S----
	Bacillus loiseleuriae	WP_049683634	-----MVTE--D-V-VKFLSD-----KIFFSNG	YYISY	I-----VVV-Y--FK------I------
	Bacillus marinisedimentorum	WP_066173156	R---PM-QER-M-F-VPFLAE--VL-P-FFKNE	YWTSY	---LF---V-FV-FK----V--------
	Bacillus marisflavi	WP_048003993	-----I-SEG--K--VPVLHK--V---IFFSNT	YWPPF	V--IA--LV-F-MFK--------S----
	Bacillus massilioanorexius	WP_019242240	-----F-QQT--K---PILSK--V---IFFSHQ	YAIAY	I-LIIP----YVLFK------I------
	Bacillus massiliogabonensis	WP_102271397	-----I-QKG--KV--PILSD---L-RIFFSNT	YYTSY	V--II-----YV-FK--------S----
	Bacillus massiliogorillae	WP_042348833	-----R-D-R-DKM--PFLSD--V---IFFKDV	YIVSY	I--LV-VVV-YV--K-------------
	Bacillus mesonae	WP_066396252	--E--I-KKG--KV--PILKD--V--KIFFQNT	YWTSF	V--IV-I---Y-VFK--------S----
	Bacillus mojavensis	WP_010331679	-A---K-PEP-YKTS-PGLSD--VL-KIFFSDV	YYTSI	--------S-F-LFK------I-S----
	Bacillus nakamurai	WP_061522327	-A---K-PEP-YKTN-PGLSD--VL-KIFFSDV	YYTSI	--------S-F-LFK------I-S----
	Bacillus ndiopicus	WP_042472047	R----F-SER-P-F-VPLLAD--FL-P-LFH--	YGTSI	--F-V--L--F---K-------------
	Bacillus nealsonii	WP_101177733	-------QYS-KKV--PVLKD--V--E-FF-NT	YWTSY	---IV-I---FVM-K--------S----
	Bacillus novalis	WP_066096334	--E--I-KHS-GKV--PVLKK--Y--EIFFQNT	YWTSF	V--IV-V---FF--K--------S----
	Bacillus oceanisediminis	WP_110065630	-----I-QKG--KV--PFLSD--V--K-LFSNT	YYTSF	A--AV--L--FVMFK-------------
	Bacillus oleivorans	WP_097160553	-----R-SDP-YSTN-PFLSD-------LF-KV	YWTSF	I--G--I-V-IVV---------------
	Bacillus panaciterrae	WP_028399044	-----V-QYRIDK---PGLSD--VL-K-FF---	PLTSY	I--A-V-VV-Y---K-------------
	Bacillus pumilus	WP_024719727	-A---K-TEP-YKG--PLLSD------IFFKDV	YYTSI	--L---VV--FVLFKM------------
	Bacillus rubiinfantis	WP_042356210	--E-GI-SEG-RK---PVLKD-----EIFFQNT	YWTSF	V--IV-V---YVV-K--------S----
	Bacillus siamensis	WP_016938126	-A---K-PEP-YKTN-PGLSD--VL-KIFFSDV	YYTSI	-------VS-F-LFK------I-S----
	Bacillus soli	WP_066068794	--E--I-KQG--K---PILKD--VL-KIFFQNT	YWTSF	V--FV-----YV--K--------S----
	Bacillus sporothermodurans	WP_066233232	-----F-KE--RK---PVLKD---V---FFSNV	TYASY	---I---VV-F--FK--------S----
	Bacillus stratosphericus	WP_052320814	-A---K-TEP-YKG--PLLSD------IFFKDV	YYTSI	--L---VV--FVLFKM------------
	Bacillus subtilis	WP_064816501	-A---K-PEP-YKTN-PGLSD---L-KIFFSDV	YYTSI	---A----S-F-LFK------I-S----
	Bacillus velezensis	WP_100001584	-A---K-PEP-YKTN-PGLSD--VL-KIFFSDV	YYTSI	-------VS-F-LFK------I-S----
	Bacillus vireti	WP_034724966	--E--I-KHS-GKV--PILKK--Y--EIFFQNT	YWTSF	V--IV-V---FF--K--------S----
	Bacillus weihaiensis	WP_072579987	-----R-TIS-NKM-VPFLSD------IFFSGG	YITSY	I--LF-V-V-YV--K-------------
	Chryseomicrobium excrementi	WP_100352701	-----YVSEQ-I-F--PVLSD--F--PMFFSDV	YSTSI	---AV-IL--FV--K-------------
	Citricoccus massiliensis	WP_102415243	-----MVTEP-YTT--PLLGD--V--PIFFQNT	YLTSY	-------V--Y-L-K-------------
	Domibacillus indicus	WP_046174999	-----M-DSR-I-K--PFLSD--V---IFFS--	ARPTI	W-FII--VS-FVL-K--------S----
	Falsibacillus pallidus	WP_114743615	-----M-QNA-RKV--PVLSD--V--KIFFANT	YYTSF	F--IV---V-F--FK--------S----
	Geobacillus genomosp. 3	WP_020959411	-----Q-Q-G-DK--VPILSD--V--P-LFSNA	YAPSY	I-VL---LS-Y---K-------------
	Geobacillus jurassicus	WP_066229768	-----Q-Q-G-DK--VPVLSD--V--P-LFSNA	YVPSY	I------VS-YV--K-------------
	Geobacillus kaustophilus GBlys	GAD12225	-----Q-Q-G-DK--VPVLSD--V--P-LFSNA	YAPSY	I--A---VS-YV--K-------------
	Geobacillus lituanicus	WP_094239903	-----Q-Q-G-DK--VPVLSD--V--P-LFSNA	YVTSY	I--L---VS-YV--K-------------
	Geobacillus sp. 46C-IIa	WP_081206929	-----Q-Q-G-DK--VPVLSD--V--P-LFSNA	YAPSY	I--I---VS-YV--K-------------
	Geobacillus sp. FJAT-46040	WP_096225498	-----Q-Q-G-DK--VPVLSD--V--P-LFSNA	YAPSY	I--A---VS-YV--K-------------
	Geobacillus sp. GHH01	WP_015374443	-----Q-Q-G-DK--VPVLSD--V--P-LFSNA	YAPSY	I--A---VS-YV--K-------------
	Geobacillus sp. LC300	AKU27761	-----Q-Q-G-DK--VPVLSD--V--P-LFSNA	YVTSY	I--L---VS-YV--K-------------
	Geobacillus stearothermophilus	WP_121625496	-----Q-Q-G-DK--VPVLSD--V--P-LFSNA	YVTSY	I--L---VS-YV--K--------S----
	Geobacillus thermodenitrifican	WP_100660319	-----Q-Q-G-DK--VPVLSD--V--P-LFSNA	YVTSY	I--I---VS-Y---K-------------
	Geobacillus vulcani	WP_031410313	-----Q-Q-G-DK--VPVLSD--V--P-LFSNA	YAPSY	I--A---VS-YV--K-------------
	Geobacillus zalihae	WP_060788134	-----Q-Q-G-DK--VPVLSD--V--P-LFSNA	YVTSY	I--L---VS-YV--K-------------
	Halobacillus alkaliphilus	WP_089751003	-----MVDRP-YT---PVL-K---L---FFSGW	YLTSY	---I---A--F---K--------S----
	Halobacillus dabanensis	WP_075037648	-----F-ESPIYSSNVPLL-K------IFF-GW	YWTSY	---A---FV-FV--K--------S----
	Halobacillus halophilus	WP_014643339	-----MVDRP-YT---PVL-K---L---FFSGW	YLTSY	---I---A--F---K--------S----
	Halobacillus hunanensis	WP_079529905	-----MVERP-YTT--PVLSK-------FFSGM	YLTSY	---L---V--Y-LFK--------S----
	Halobacillus karajensis	WP_074734152	-----F-ESPIYSSHVPLL-K-------FFSGW	YWTSY	--V-A---V-FV--K--------S----
	Halobacillus kuroshimensis	WP_027954266	-----F-KQP-YSS-VPVL-N---V-EMLFSGW	YWTSY	---AM--A--FVL-K--------S----

Other Bacteria
(0/>200)

Halobacillus litoralis	WP_128522702	-----I-SRP-YSM--PVLSK-------FF-GW YLTSY ---F---V--FF--K--------S----
Halobacillus sp. BBL2006	WP_052158927	-----MVERP-YTT-VPLLSK--VL---FFSGM YITSY -------V--F---K-S------S----
Halobacillus trueperi	WP_115823359	-----F-ESPIYSSNVPLL-K---L--MFF-GW YWTSY ---AM---V-F--FK--------S----
Klebsiella pneumoniae	OON72022	-----F-QYRIEK--VPGLSD--V--KIFFSNV PLTSY I--I---VV-YV--K------------
Kurthia huakuii	WP_029498309	-----S-SER-D-Y--PVLSD--V--PMFFHDV YLTSV ---AV--L--FV--K------------
Kurthia massiliensis	WP_010286277	-----S-SER-N-Y-VPVLGD--V--KMFFHDL HITSV ---AI-----FV--K------------
Kurthia sibirica	WP_109304575	-----S-EER---F-FPLLSD--VL-KMFFSD- YGTSI --FGI-IL--FV--K------------
Kurthia sp. 3B1D	WP_126990732	-----S-SER-G-Y-VPVLGD--V--RMFFHDL HMTSV ---AV--L--FV--K------------
Kurthia sp. Dielmo	WP_020189503	-----S-SER-G-Y-VPVLGD--V--RMFFHDL HMTSV ---AV--L--FV--K------------
Kurthia zopfii	WP_109349274	-----S-NER---F-VPGLSE--V--KMFF-DV YGTSI ---GV-IL--FVLFK--------S----
Lentibacillus amyloliquefacien	WP_068441432	-----MVSQQ-FTQ--PLLSK-----EIFFQG- YLSSY -------V--YVL-K--------S----
Lentibacillus salicampi	TFJ94718	R----MVSHQ-FTR--PFLSD--V---IFFQG- YWTSY ---A-S-V--YVL-K--------S----
Lysinibacillus acetophenoni	WP_097149197	-----S-SQR---F-VPFLSD--F--P-FFEDA YATTF V-FAVV----FVL-K------I------
Lysinibacillus chungkukjangi	WP_107932837	-----S-TER---F--PVLGD---L-KMFFVDV YATSI ----V-IV--FV--K------------
Lysinibacillus composti	WP_124765984	----EF-TER-A-F--PGLAS--VL-PMFFHNV YGTSI ----V-IV--FV--K------------
Lysinibacillus endophyticus	WP_121215015	-----S-TER---F--PLLSD--V--KMFFVDV YATSI --VAV-I---F--TK------------
Lysinibacillus fluoroglycofeni	WP_107942308	R----F-SKR-A-F--PVLSD--F--R-LFHDV YGTSI ---AV-IF--FV-FK------------
Lysinibacillus halotolerans	WP_122970422	-----S-TER---F--PLLSD--V--KMFFVDV YATSI --FAV-IV--F---KS-----------
Lysinibacillus manganicus	WP_036189726	-----S-QQR---F-VPYLSD--F--P-FFEDA YATTF V-FAVV----FVL-K------I------
Lysinibacillus meyeri	WP_107839506	R----F-SAR-P-F-VPLLAD--FF-P-LFHN- YGTSI --F-V--L--FV-FK------------
Lysinibacillus sp. Marseille-P	WP_106784314	-----S-EER---M-VPGLAD--FL-E-FFKDV YATSF ---FM--VG-FVL-K------I------
Lysinibacillus sp. SYSU K30002	WP_126658044	-----S-TER---F--PILSD-----KMLFVDV YATSI ----I-----F---K------I------
Lysinibacillus sp. YLB-03	WP_118874595	-----A-SER---F--PLLSD---L-KMFFVDV YATSI --FAV-----YV--K--------S----
Lysinibacillus telephonicus	WP_126293020	-----S--ER---F--PVLSD--L--KMFFVDV YATSI --F-V-IV--FV--K------------
Lysinibacillus xyleni	WP_097072201	-----S-TER---F--PLLSD-----RMFFVDV YATSI ---AV-L---F--TK------------
Mycobacteroides abscessus subs	SHR11943	-----I-QKG--K---PVLSD--VL-K-FFSNT YYTSY V--IV-----FVMFK------------
Oceanobacillus iheyensis	WP_011067783	-----MVSQP-YTT--AILEK--V--PIFFQGV YVTSY ---I---V--YVL-K------------
Oceanobacillus profundus	WP_118888906	-----MV-QP-YTT--PLLSK--V--SIFFQG- YITSY ---I---V--FVL-K------------
Paenibacillus sp. 7884-2	WP_095308750	-----MV-QP-YTT--PLLSK--V--SIFFQG- YITSY ---I---V--FVL-K------------
Paenibacillus sp. FSL R5-0490	WP_076255875	-----I-QKG--KV--PFLSD--F--K-FFSNT YYTSF V--AV--L--FVMFK------------
Paenisporosarcina antarctica	WP_134210058	-----F-QER-L-D--PFLKD--VL-PMFFQNV YATSV ----I-IL--VVL-F------------
Paenisporosarcina indica	WP_075617580	-----FVQER-L-DG-PFLEK--FF-PMFFQNV YWTSV ---GI-ILS-VVL--------------
Paenisporosarcina quisquiliaru	WP_090562411	-----Y-QER-I-F--PLLSD-----PMLFKDV YGTSI ---GV-VL--FV--K------------
Paenisporosarcina sp. HGH0030	WP_016427578	-----FVQER-L-D--PFLKD---L-PMFFQNV YWTSV ---GI-IL--VVL--------------
Paenisporosarcina sp. K2R23-3	WP_119883038	-----F-QER-L-T--PILEN--V--PMFFQHV YYTSI --FAV-IL--VVL-F------I------
Paenisporosarcina sp. OV554	WP_108584689	-----FVQER-L-D--PFLKD---L-PMFFQNV YWTSV ---GI-IL--VVL--------------
Paenisporosarcina sp. TG-14	WP_017380887	-----F-QER-L-D--PFLKN---L-PMFFQNV YATSV ---AI-IL--VVL-F------------
Paenisporosarcina sp. TG20	WP_026023066	-----FVQER-L-DS-PLLEK-----PMFFQNV YGTSV ---SI-IL--IVL--------------
Parageobacillus caldoxylosilyt	WP_042407069	-----Q-Q-G-DK--VPVLSH--V--P-FFSNA YIPSY ---I---V--Y---K------------
Parageobacillus genomosp. 1	WP_043904173	-----Q-Q-G-DK--VPVLSD-----P-FFSNA YIPSY ---I---V--YV--K------------
Parageobacillus thermantarctic	WP_090947968	----NQ-QIG-DK--VPILSD--V--P-FFSNT YVPSY ---I---V--YV--K------------
Parageobacillus thermoglucosid	WP_064550976	-----Q-QIG-DK---PVLSD--V--P-FFSNT YVPSY ---I---V--YV--K------------
Planococcus antarcticus	WP_006828226	-----F-TER-A-Y-VPFLSD--F--P-FF--V YNTSF F--AV-----FV--K------------
Planococcus citreus	WP_121297639	-----F-SER-A-YNVPVLSE--V--P-FF--V YNTSF F--GV--L--FV--K------------
Planococcus faecalis	WP_071154156	-----F-TER---YNVPFLSD--V--P-FFK-V YNTSF F--AV--V--FV--K------------
Planococcus kocurii	WP_058384965	-----F-TER---YNVPFLSD--V--P-FFK-V YNTSF F--AV--V--FV--K------------
Planococcus maitriensis	WP_112231529	-----F-SER-A-YNVPVLSE--V--P-FF--V YNTSF F--AV--L--FV--K------------
Planococcus plakortidis	WP_068869365	-----F-SER-A-YNVPVLSE--V--P-FF--V YNTSF F--GV--L--FV--K------------
Planococcus versutus	WP_049693250	-----F-TER---YNVPFLSD--V--P-LFK-V YNTSF F--AV--V--FV--K------------
Planomicrobium glaciei	WP_036806598	-----F-TER---N--PVLSD--V--PMFFS-V YYTSV ---AV-IF--FV--K--------S----
Psychrobacillus insolitus	WP_111438427	-----Y-QER-I-D--PYLSE---L-PMFFKDV YGTSI --LIV-LL--FV--K------------
Psychrobacillus psychrodurans	WP_093494497	-----Y-QQR-I-F--PLLSD-----PMLFKDV YGTSI ---GV-VL--FV--K------------
Psychrobacillus psychrotoleran	WP_093535041	-----Y-QER-I-F--PFLSDL-V--PMLFKDV YGTSI ---GI-VM--FV--K------------
Psychrobacillus sp. FJAT-21963	WP_056829337	-----Y-QER-I-F--PFLSD--V--PMFFKDV YSTSI --VAI-IL--FV--K------------
Psychrobacillus sp. OK028	WP_093060199	-----Y-QER-I-F--PLLSE--V--PMFFKDV YGTSI --VGV-IM--FV--K------------
Psychrobacillus sp. OK032	WP_093262988	-----Y-QER-I-F--PFLSD--VL-PMFFKDV YSTSI ---IV-IG--FV--K------------
Rummeliibacillus pycnus	WP_102692605	-----S--ER---F--PGLKN---L-KMFF-DV YGTSI ---AV--F--F---K------------
Rummeliibacillus stabekisii	WP_066785780	-----S-SER---F--PVLKD--LL-KMFF-DV YGTSI F---V-----F---K--------S----
Sediminibacillus albus	WP_093212290	-----MVSQP-YSL--PFLSDV--L-P-FFQDM YMTSY ---FI-----F-L-K--------S----
Sediminibacillus halophilus	WP_074598234	-----MVSQP-YTL--PVLSD--VL-T-FFQNM YLTSY ---I--I---Y-L-K--------S----
Sporosarcina globispora	WP_053436414	-----I-QKG--KV--PFLSD--V--K-FFSNT YYTSF A--AV--L--FVMFK------------
Sporosarcina sp. EUR3 2.2.2	WP_024534969	-----FVQER-L-D--PFLKD--VL-PMFFQNV YWTSI ---GI-IL--VVL--------------
Tetzosporium hominis	WP_094941684	-----YVTEQ-I-F--PVLSD--V--PMFFSDV YSTSI ---AV-IL--FV--K------------
Thermolongibacillus altinsuens	WP_132949530	-----QVE-G-DK---PILSK-----P-FFSNG YIPSY I-------V-YV--K--------S----
Virgibacillus dakarensis	WP_088050257	-----MVTEP-YTT--PLLGD--V--PIFFQNT YLTSY -------V--Y-L-K------------
Virgibacillus indicus	WP_094883858	-----MVTEP-F-T--PLLSE--L--PMFFQG- YTTSY V--LF-----YVL-K------------
Virgibacillus ndiopensis	WP_099158159	-----MVTKP-YTM--PLLSKV-V---IFFQG- YLTSY ---I---V--YVLHK------------
Virgibacillus necropolis	WP_089534373	-----MVSKP-YTT--KYLSD--VL-PIFFQN- YLTSH ---A---V--YVL-K------------
Virgibacillus phasianinus	WP_089063682	-----MVSKP-YTT--PLLSD--VL-PILFQG- YLTSY ---A------YVL-K------------
Virgibacillus siamensis	WP_077326246	-----MVSQP-YTN--PILKE--V--PVFFEN- YLTSY I--I---V--YVL-K------------
Viridibacillus arvi	WP_053417416	-----M-K-R-P-FN-PYLED--L--PMLFKDV YGTSI ---AV-IL--V---K------------

Supplemental Figure 82

A partial sequence alignment of the ABC transporter permease containing a 5 amino acid deletion (boxed) that is exclusively shared by all members belonging to the Flavidum clade and absent in all other bacteria.

			98		139
**Flavidum Clade (2/2)**	Planomicrobium flavidum	WP_088009144	DSCLCSIGSVETICIENKPLKPGA		FNAELPETGDWRILGNVN
	Planococcus sp. Y42	WP_077587920	---------L----V---------		------------------
**Other Bacteria (0/>100)**	Alkaliphilus metalliredigens	WP_012065736	-A--GKVDRIGYMTVS-G------	G	V-K---AV--IH-T-I--
	Ammoniphilus sp. CFH 90114	WP_129199573	-A--GQFN--GN-T-SHG------	G	VKK----V-NLH-T-I--
	Ammoniphilus sp. YIM 78166	WP_134702323	-A--GQFS--GN-T-AHG------	G	VKK----V-NFH-T-I--
	Anoxybacillus ayderensis	WP_042534882	-A--GRVKNIGMVT--KG------	A	V-KN--SV--AH-T-I--
	Anoxybacillus flavithermus	WP_003397575	-A--GRVKNIGMVT--KG------	A	V-KN--SV--AH-T-I--
	Anoxybacillus gonensis	KGP59836	-A--GRVKNIGM-S--KG------	A	V-KN--SV--AH-T-I--
	Anoxybacillus kamchatkensis	WP_050982316	-A--GRVKNIGM-S--KG------	A	V-KN--SV--AH-T-I--
	Anoxybacillus mongoliensis	WP_075039603	-A--GRVKNIGM-S--KG------	A	V-KN--SV--AH-T-I--
	Anoxybacillus sp. 103	WP_077429091	-A--GRVKN-GMVT--KG-I----	A	V-KN--SV--AH-T-I--
	Anoxybacillus sp. BCO1	WP_042895279	-A--GRVKNIGMVT--KG------	A	V-KN--SV--AH-T-I--
	Anoxybacillus sp. KU2-6(11)	KFZ42409	-A--GRVKNIGMVT--KG------	A	V-KN--SV--AH-T-I--
	Anoxybacillus suryakundensis	WP_055440867	-A--GRVKNIGMVTV-KG------	A	V-KN--SV--AH-T-I--
	Anoxybacillus tepidamans	WP_027408134	-A--GRLK--GA-T-AEG-VR---	G	V-KQ--PV--FH-T-V--
	Anoxybacillus thermarum	WP_043964356	-A--GRVKNIGM-S--KG------	A	V-KN--SV--AH-T-I--
	Bacillus aquimaris	WP_113969409	-A--GRLK--GA-QLSEG------	G	V-K----V-EIHLT-I--
	Bacillus australimaris	WP_060698951	-A--GRVK--GSFQ-GDG------	G	VQK----V--IH-N-I--
	Bacillus bogoriensis	WP_026675527	-A--GRMN--GK-TVAQG-I----	A	V-KN---V--VHVT-I--
	Bacillus campisalis	WP_046524973	-A--GRLK--GS-H-NSG------	G	V-KN---V--IHLT-I--
	Bacillus coahuilensis	WP_059349923	-A--GRMK--GA-QVG-G-V----	G	V-K----V--IH-T-I--
	Bacillus firmus	WP_048010208	-A--GRLK--GV-Q-GDG-V----	G	V-KD---V--MH-T-I--
	Bacillus foraminis	WP_121610424	-A--GRLK--G--Q-ASG-V----	G	V-K----V--IH-T-I--
	Bacillus freudenreichii	VEF50061	-A--GRLA-IGE-KVGTG------	G	V-KK---V--AF-T-I--
	Bacillus gottheilii	WP_080843624	-A--GRVK--GI-QVGDG-V----	G	V-KD---V--MH-T-I--
	Bacillus infantis	WP_129613047	-A--GRLK--GV-QVGAG-V----	G	V-K----V--MH-T-I--
	Bacillus jeotgali	WP_102264744	-A--GRLK--GS-QVG-G-V----	G	V-K----V-NMH-T-I--
	Bacillus kochii	WP_095371340	-A--GRVK--GI-Q-ADG-V----	G	V-KD---V--IH-A-I--
	Bacillus korlensis	WP_066059787	-A--GRLK--GF-QLVDG-V----	G	V-K---SV--YH-T-I--
	Bacillus marisflavi	WP_048004582	-A--GRLK--GA-QLSHG------	G	V-K----V-NIHLT-I--
	Bacillus massiliogorillae	WP_042352969	-A--GRMKN-G-VQ-GDG------	G	V-KN---V--IHVT-I--
	Bacillus massiliosenegalensis	WP_019157216	-A--GRLK--GV-Q-GEG-V----	G	VKK----V--YH-T-I--
	Bacillus notoginsengisoli	WP_118921182	-A--GRFK--GI-QVCDG-V----	G	V-K----V--MH-T-I--
	Bacillus persicus	WP_090748355	-A--GRMK--GF-QVGSG-V----	G	V-K----V--IH-T-I--
	Bacillus pumilus	WP_088002906	-A--GRVK--GSFQ-GDG------	G	VQK----V--IH-N-I--
	Bacillus safensis	WP_048238328	-A--GRVK--GSFQ-GDG------	G	VQK----V--IH-N-I--
	Bacillus selenatarsenatis	WP_041965149	-A--GRLK--GS-QVGDG-V----	G	V-K----V-NMH-T-I--
	Bacillus sp. 1NLA3E	WP_015596221	-A--GRLK--GV-QVSDG-V----	G	V-KD---V--IH-T-I--
	Bacillus sp. 7504-2	WP_095314738	-A--GRLK--GV-Q-GEG-V----	G	VKK----V--YH-T-I--
	Bacillus sp. AFS015802	WP_098351367	-A--GRLK--GA-QLSEG------	G	V-K----V-EIHLT-I--
	Bacillus sp. AFS073361	WP_098577046	-A--GRVKN-GV-QVG-G-V----	G	V-K---AV--IH-T-I--
	Bacillus sp. EB01	WP_043934275	-A--GRFK--GVLQ-C-G-V----	G	V-KD---V--MH-T-I--
	Bacillus sp. FJAT-18017	WP_053597531	-A--GRFK--GVLQ-C-G-V----	G	V-KD---V--MH-T-I--
	Bacillus sp. FJAT-27225	WP_066205270	-A--GRFK-IGVLQ-C-G-V----	G	V-KD---V--MH-T-I--
	Bacillus sp. FJAT-27245	WP_053368613	-A--GRFK--GI-QVCDG-V----	G	V-K----V--MH-T-I--
	Bacillus sp. FJAT-27251	WP_053364471	-A--GRLK--GS-H-NSG------	G	V-KN---V--IHLT-I--
	Bacillus sp. FJAT-27445	WP_059171620	-A--GRFK--GV-QVCDG-V----	G	V-K----V--MH-T-I--
	Bacillus sp. FJAT-45086	WP_100405872	-A--GQSS--GKVSLSSG------	A	V-KQ---V--IHMT-I--
	Bacillus sp. GBSW19	WP_106073039	-A--GRVK--GSFQ-GDG------	G	VQK----V--IH-N-I--
	Bacillus sp. HMF5848	WP_125908196	-A--GRLN--GV-S-GEG-V----	G	V-K---AV--AH-T-I--
	Bacillus sp. I-2	WP_076840624	-A--GRVK--GSFQ-GDG------	G	VQK----V--IH-N-I--
	Bacillus sp. Leaf406	WP_056539917	-A--GRLK--GA-QLSHG------	G	V-K----V-NIHLT-I--
	Bacillus sp. Nf3	WP_107164230	-A--GRVK--GSFQ-GDG------	G	VQK----V--IH-N-I--
	Bacillus sp. P14.5	WP_113927352	-A--GRLK--GA-QLGDG-V----	G	V-K---DV--MH-T-I--
	Bacillus sp. SG-1	EDL64167	-A--GRLK--GA-QLG-G-V----	G	V-K---DV--IH-T-I--
	Bacillus sp. SG-1	WP_044022814	-A--GRLK--GA-QLG-G-V----	G	V-K---DV--IH-T-I--
	Bacillus sp. T33-2	WP_101584143	-A--GRLK--GS-QVGSG-V----	G	V-K----V--IH-T-I--
	Bacillus sp. UNC41MFS5	WP_026568060	-A--GRVKN-GV-QVG-G-V----	G	V-K---AV--IH-T-I--
	Bacillus sp. Y1	WP_119710423	-A--GRLK--GF-QLADG-V----	G	V-K---SV--YH-T-I--
	Bacillus sp. YLB-04	WP_115450936	-A--GRFK--GV-QVCDG-V----	G	V-K----V--MH-T-I--
	Bacillus subterraneus	WP_044392029	-A--GRLK--GS-QVG-G-V----	G	V-K----V--MH-T-I--
	Bacillus zhangzhouensis	WP_034323860	-A--GRVK--GSFQ-GDG------	G	VQK----V--IH-N-I--
	Caloranaerobacter azorensis	WP_035165124	-A--GN-DRIGYVKV-KG------	G	V-KN---I--IH-T-I--
	Clostridium acetireducens	WP_070110734	-A--G--QNIGN-I--S---S--S	A	LKKN--KV--LS-T-I--
	Clostridium acetobutylicum	WP_034586355	-A--G-TKT-GN-V-Q-T--S--S	A	M-KS--TV--LS-T-I--
	Clostridium amylolyticum	WP_073006144	-A--G-VQN-GK-V---S-IL---	A	V-KN--PV--LS-T-I--
	Clostridium arbusti	WP_026038540	-A--G-LQ--GG-I-----IM---	A	M-KD--PV--LS-T-V--
	Clostridium beijerinckii	WP_012061241	----G--NNIGKVF-QK---L--L	A	L-KN--AV--MS-T-I--
	Clostridium botulinum	WP_096045026	-A--G--QNIGK-IVDS---Y--S	A	V-KD--AI--LS-N-V--
	Clostridium carboxidivorans	WP_007063403	-A--G--QN-GN-VL-S------S	A	M-KD--QV--LS-T-I--
	Clostridium cellulovorans	WP_010074392	-A--GNVE--G-V-LKD---A---	A	LKKS--PV--IS-T-V--
	Clostridium chauvoei	WP_021876874	-A--GD-HNIGKVF-Q-R--Q--L	A	M-KD--AI--MS-T-V--
	Clostridium chromiireducens	WP_079440834	----G--NNIGK-F-QK---L--L	A	L-KN--SV-EMS-T-I--
	Clostridium cochlearium	WP_089864650	-A--GH-QNIGKVFV--------S	A	V-KD--SI-NLS-T-I--

Other Bacteria (0/>100)

Clostridium combesii	WP_099839744	-A--G--QNIGK-IVDS---Y--S A V-KD--AI--LS-N-V--
Clostridium drakei	WP_032078462	-A--G--QN-GN-VL-S------S A M-KD--QV--LS-T-I--
Clostridium estertheticum	WP_071615086	-A--G-LQN-GK-VV-E---S--- A M-KD--KV--LS-T-I--
Clostridium fallax	WP_072896294	-A--GTVQN-GK-L--K------L A VSKD--PV--LS---I--
Clostridium frigidicarnis	WP_090042446	-A--G-LPN-GKVL-D----F--S A V-KD--AV--LS-S-V--
Clostridium homopropionicum	WP_052220877	-A--G--Q-IGN-I--D---S--- A M-KQ--KV-NLS-T-I--
Clostridium ihumii	WP_040329784	-A--G--H--GNVI-QS--IT--S A L-KD--PV--LS-T-V--
Clostridium isatidis	WP_119866532	-A--GK-NNIGK-F------S--L A L-KD--KV--LS-T-I--
Clostridium kluyveri	WP_012104227	-A--GN-QN-GK-IL-S---H--S A M-KS--QV--LS-T-I--
Clostridium liquoris	WP_106062555	-A--GNVQNIGK----E---S--S A M-KN--PV-NLS-T-I--
Clostridium ljungdahlii	WP_063557101	-A--GT-QD-GK-I--T---T--S A MKKS--QV--LS-T-I--
Clostridium lundense	WP_027626062	-A--G--QNIGN--V-E---S--S A M-KK--PV-NLS-T-I--
Clostridium luticellarii	WP_106011216	-A--GA-QN-GK-IV-T---H--S A M-KS--QV--LS-T-I--
Clostridium novyi	WP_011721007	-A--GK-D-IGN-I-D-T-IT--- A MHKK---V--LS-T-I--
Clostridium oryzae	WP_079426886	-AS-G--HN-GK-I------T--- A M-KK--PV--LS-T-I--
Clostridium pasteurianum	WP_003444885	-A--G--QN-GK-I-----VI--- A M-KD--PV-NLS-T-I--
Clostridium peptidivorans	WP_097028237	-A--G--QNIGKV-V-E---S--S A M-KN---V-NLS-T-I--
Clostridium perfringens	WP_131439065	-A--GVVDN-GHVL--KR-----I A L-KN---V--LS---I--
Clostridium puniceum	WP_077849778	----G-LNNIGK-F-QK---M--L A L-K----V--MS-T-I--
Clostridium ragsdalei	WP_065078765	-A--GT-QD-GK-I--T---T--S A MKKS--QV--LS-T-I--
Clostridium saccharoperbutylac	WP_015395855	-A--G-FKNIGN-F-QK------L A L-KV--SV-EMS-T-I--
Clostridium scatologenes	WP_029161058	-A--G--QN-GN-VL-S------S A M-KD--QV--LS-T-I--
Clostridium sp. ATCC 25772	WP_061996694	-A--G-LP--GNVI-DS---T--- A L-KD--SV-NLS-T-V--
Clostridium sp. AWRP	WP_127723808	-A--GT-QD-GK-I--T---T--S A MKKS--QV--LS-T-I--
Clostridium sp. CAG:221	CDB16781	-AA-G-VNNIGKVI------A--- A --KS--AI-NMS-K-I--
Clostridium sp. CAG:265	CDB74179	-AA-G-VHNIGK-I------S--- A --KS--PI-NMS-K-I--
Clostridium sp. CT4	WP_128210868	-A--GD-DN-GK-Y--K---L--M A V-KN--PV--IS---V--
Clostridium sp. HMP27	KGK86791	-A--G--QNIGKV-V-E---S--S A M-KN---I--LS-T-I--
Clostridium sp. JN-1	WP_123054923	-A--G--QN-GK-I------N--S A V-KS--KV--LS-T-I--
Clostridium sp. JN500901	WP_119974134	-A--G--QN-GK-I------T--S A V-KA--KV--LS-T-I--
Clostridium sp. K25	WP_003374901	-A--GKLNNIGN-IL-----S--- A M-KD--KV--LS-T-I--
Clostridium sp. L74	WP_053467155	-A--G--QNIGK-I-DS--IY--S A V-KD--AI--LS-S-V--
Clostridium sp. Marseille-P420	WP_125154218	-A--G-VQNIGK----E---S--S A M-KN--LV-NIS-T-I--
Clostridium sp. N3C	WP_074366236	-A--GALHN-GK-IV-A---T--S A M-KD--QV-NLS-T-V--
Clostridium sp. ND2	WP_055071986	-A--G--NN-GK-I-QKA--T--I A --KN--KV-HLS---I--
Clostridium sporogenes	WP_045884056	-A--G--QNIGK-IVDS---Y--S A V-KD--AI--LS-N-V--
Clostridium sulfidigenes	WP_035135532	-AS-GGLQ--GK-I--S---Y--- A L-KK--SV--LS-V-I--
Clostridium taeniosporum	WP_069680993	----GK-NNIGK-F-DK---Y--K A L-K---PV--IS-T-I--
Clostridium tagluense	WP_124998819	-A--G-LQN-GK-IV-E---S--- A M-KD--KV-NLS-T-V--
Clostridium tepidiprofundi	WP_066826100	----G--DNIGK-I------S--- A LKKD--KV-NIS-T-I--
Clostridium tetani	WP_035141189	-A--GH-QNIGKVFV-D------S A V-KD--PV-NLS-T-I--
Clostridium tunisiense	WP_017415144	-AS-GALQN-GKVV--S---S--- A L-KD--KV--LS-T-I--
Clostridium tyrobutyricum	WP_039657843	-A--G-LQN-GK-IL-A---H--S A M-KS--SI--LS-T-I--
Clostridium ventriculi	WP_055259140	-A--G-LDN-GK-I--K--IY--M A V-KD--PV--LS-M-V--
Cohnella luojiensis	TFE23564	-A--G-V--IG-VH-V-G--R--- G V-KT-TPF--IH-S-V--
Desmospora activa	WP_107728179	-A--GQLS--GW-QVG-G------ G V-KQ---V-QVH-T-I--
Desnuesiella massiliensis	WP_055669976	-A--GN-QN-GK-M--R---H--S A M-KD--PV--LS-T-I--
Desulfosporosinus lacus	WP_073031012	-A--GKS-E-GK-TLS-S--T--- A L-K---AV--IS-M-I--
Desulfuribacillus stibiiarsena	WP_069701095	-A--GKLN--GN-T-GTGS----- G V-K----V--VY-T-T--
Effusibacillus lacus	WP_096180813	-A--GQLS--GM-SVGVG------ G V-KN---V--IH-T-I--
Effusibacillus pohliae	WP_018131418	-A--GQLS--GM---GEG------ G V-KN---V--VH-T-I--
Falsibacillus sp. GY 10110	WP_121681489	-A--GR-K--GS-Q-GDG-V---- G V-K---SV--MH-T-I--
Fischerella thermalis	WP_102177091	-A--GQLA--GW-QVG-G------ G V-KQ---V-HVH-T-I--
Garciella nitratireducens	WP_087679396	-A--GRHN-IGN-I-SSG------ G V-KN---I--MS-I-I--
Marininema halotolerans	WP_091833198	-A--GQLS--GW-QVG-G------ G V-KQ---V-QIH-T-I--
Melghirimyces profundicolus	WP_108022227	-A--GQLA--GW-QVG-G------ G V-KQ---V-QVH-T-I--
Mycobacteroides abscessus subs	SHT35673	-A--GRLK--GF-QLADG-V---- G V-K---PV--YH-T-I--
Oceanobacillus jeddahense	WP_040977728	-A--GKSS--GS-IA--------L A L-KQ--QV-NMN-T-V--
Oceanobacillus massiliensis	WP_010648891	-A--GRNA-IGH-IT--G-----S A L-KA--SI--VN-T-V--
Oceanobacillus oncorhynchi	WP_042528680	-A--GKSS--GS-IA--------L A L-KQ--QV-NMN-T-V--
Oceanobacillus sojae	WP_077602773	-A--GKSS-IGS-IA--------L A L-KQ--QV-NMN-T-V--
Oceanobacillus timonensis	WP_080871896	-A--GKSS--GS-IA--R-----L A L-KQ--QV-NINMT-V--
Paenibacillus alginolyticus	WP_029196150	-A--GQVS--GC-QVADG------ G V-KD--PV--IHVT-I--
Paenibacillus algorifonticola	WP_046234575	-A--GQVS-IGS-H-GTG-VR--- G V-K---PV--MH-T-I--
Paenibacillus amylolyticus	WP_123067002	-A--GQSS--GC-QVV-G------ G V-K---PV--IHLT-I--
Paenibacillus barcinonensis	WP_110896914	-A--GQSS--GC-QVV-G------ G V-K---PV--IHLT-I--
Paenibacillus chitinolyticus	WP_042235663	-A--GQVS--GS-QVGHG------ G V-K---SV--IHVT-I--
Paenibacillus chondroitinus	WP_047684042	-A--GQVS--GC-QVADG------ G V-KD--PV--IHVT-I--
Paenibacillus contaminans	WP_113032879	-A--GQLS--GC-QLASG------ G V-K---AV--IHMT-I--
Paenibacillus ferrarius	WP_079415841	-A--GQVS--GC-QVADG------ G V-KD--PV--IHVT-I--
Paenibacillus pabuli	WP_062327701	-A--GQSS--GC-QVV-G------ G V-K---PV--IHLT-I--
Paenibacillus pectinilyticus	WP_065851212	-A--GQVA--GC-QVAHG------ G V-KD--PV--IHVT-I--
Paenibacillus polysaccharolyti	WP_090923592	-A--GQSS--GC-QVV-G------ G V-K---PV--IHLT-I--
Paenibacillus senegalensis	WP_010279198	-A--GQLS--GS-K-GDG------ G V-KD--QV--IH-T-V--

Group	Species	Accession	Sequence
**Other Bacteria (0/>100)**	Paenibacillus sp. 1ZS3-15	WP_068667009	-A--GQVA--GC-QVAHG------ G V-KD--PV--IHVT-I--
	Paenibacillus sp. DCT19	WP_128104231	-A--GQSS--GC-QVV-G------ G V-K---PV--IHLT-I--
	Paenibacillus sp. FJAT-26967	WP_068776492	-A--GQVS--GS-QVGHG------ G V-K---AV--IHVT-I--
	Paenibacillus sp. FSL R5-0490	WP_076262932	-A--GRLK--GV-Q-GDG-V---- G V-KD---V--MH-T-I--
	Paenibacillus sp. GP183	WP_090794503	-A--GQAS--GS-Q-A-G------ G V-K---PV-HMHMT-I--
	Paenibacillus sp. LK1	WP_099857731	-A--GQSS--GC-QVV-G------ G V-K---PV--IHLT-I--
	Paenibacillus sp. MEC069	WP_134755625	-A--GQVS--GC-Q-A-G------ G V-K---PV-NIHVT-I--
	Paenibacillus sp. PAMC 26794	WP_017691426	-A--GQSS--GC-QVV-G------ G V-K---PV--IHLT-I--
	Paenibacillus sp. PCH8	WP_105406419	-A--GQSS--GC-QVV-G------ G V-K---PV--IHLT-I--
	Paenibacillus sp. Root52	WP_056697544	-A--GQSS--GC-QVV-G------ G V-K---PV--IHLT-I--
	Paenibacillus sp. Soil750	WP_056622658	-A--GQVA--GC-QVAHG------ G V-KD--PV--IHVT-I--
	Paenibacillus sp. Soil766	WP_057314203	-A--GQVA--GC-QVAHG------ G V-KD--PV--IHVT-I--
	Paenibacillus sp. Soil787	WP_056838727	-A--GQVS--GC-QVADG------ G V-KD--PV--IHVT-I--
	Paenibacillus sp. UNC499MF	WP_103995353	-A--GQVS--GS-QVGHG------ G V-K---SV--IHVT-I--
	Paenibacillus sp. URHA0014	WP_028557229	-A--GQVA--GC-QVAHG------ G V-KD--PV--IHVT-I--
	Paenibacillus sp. yr247	WP_090824603	-A--GQVS--GC-QVADG------ G V-KD--PV--IHVT-I--
	Paenibacillus taichungensis	WP_113053315	-A--GQSS--GC-QVV-G------ G V-K---PV--IHLT-I--
	Paenibacillus whitsoniae	WP_126143518	-A--GQVA--GC-QVAHG------ G V-KD--PV--IHVT-I--
	Parageobacillus thermantarctic	WP_090948180	-A--GRLK--GA-T-AKG-VR--- G V-KQ--PV--FH-T-V--
	Salipaludibacillus agaradhaere	WP_078579124	-A--GRTS-IG--V-GEG-I---- A L-KD-----HIH-A-I--
	Salipaludibacillus aurantiacus	WP_093055483	-A--GRVT-IGS-V-ADG------ A M-KN-----NMH-A-I--
	Tepidibacillus fermentans	WP_132769616	-A--GQL---GS-QVVDG------ G VQKN---I-HFH-S-V--
	Tepidibacter formicigenes	WP_072887466	-A--GD-NR-GC-KVS-G------ G V-KN--PI--IN---I--
	Tepidibacter thalassicus	WP_072723144	-A--G--NRIGC-KVS-E------ G V-KT--SV--IS---I--
	Thermoclostridium stercorarium	WP_015360221	-A--GTH-NIGY-KVAKG-----S G LKKD------IH-T-I--
	Thermolongibacillus altinsuens	WP_132947244	-A--GKLK-IGM-TV-KG------ G V-KQ--PV--IH-T-V--
	Tumebacillus permanentifrigori	WP_109685820	-A--GQ-Q--GLVT-G-G------ G V-KV---V-NIHVT-I--

Supplemental Figure 83

A partial sequence alignment of the spore protease YyaC protein containing a 1 amino acid deletion (boxed) that is exclusively shared by all members belonging to the Flavidum clade and absent in all other bacteria.

Group	Species	Accession	92		132
**Flavidum Clade (2/2)**	Planomicrobium flavidum	WP_088007583	HVHYAIPHAVCAILGREMAGR	P	ETKIVTTLHGTDITVLGYD
	Planococcus sp. Y42	WP_077589509	----------------D--NA	-	D------------------
**Other Bacteria (2/>100)**	Staphylococcus pettenkoferi	WP_002472138	-M---V-----G----Q--KK	K	DV--M--------------
	Staphylococcus argensis	WP_103371290	-M---V-----G----Q--KK	K	DV--M--------------
	Anoxybacillus tepidamans	WP_027408554	------------V-A-Q-S--		DV-----------------
	Bacillus acidicola	WP_066262737	---------I-----KQ---T		DL-----------------
	Bacillus aquimaris	WP_113970690	---------------KQ-S-K		DV-----------------
	Bacillus badius	WP_063385665	----------------QI-RS		SI---------------H-
	Bacillus bataviensis	WP_007084851	--------------AKQ-SN-		DI-----------------
	Bacillus campisalis	WP_046524177	--------------A---S--		DL-----------------
	Bacillus circulans	WP_047940384	--------------AKQ-SN-		DV-----------------
	Bacillus dakarensis	WP_077213779	--------------AKQ-S-K		DI-----------------
	Bacillus deserti	WP_101645019	--------------A-Q---T		DV-----------------
	Bacillus drentensis	WP_066257148	--------------AKQ-CN-		DI-----------------
	Bacillus foraminis	WP_132008974	--------------A---S-K		DL-----------------
	Bacillus fordii	WP_018707178	---------------KQ---T		DV-V---------S-----
	Bacillus fortis	WP_120073056	---------------KQ-S-T		DV-V---------S-----
	Bacillus freudenreichii	WP_126432054	--------------AKQ---T		DV-V---------S-----
	Bacillus fumarioli	WP_066369844	--------------AKQ--DH		-V-----------------
	Bacillus gottheilii	WP_080845475	--------------AKQ-S--		DV-----------------
	Bacillus horneckiae	WP_066395413	--------------AKQ-CNK		-I-----------------
	Bacillus jeotgali	WP_102263387	--------------A-Q-S--		DL-----------------
	Bacillus kochii	WP_095372781	-----V---I-----KQ-S--		DV-----------------
	Bacillus korlensis	WP_066047624	--------------AKQ-S--		DV-----------------
	Bacillus kribbensis	WP_026694847	--------------AKQ---T		DV-----------------
	Bacillus lonarensis	WP_090774967	-----V--------A-Q-S--		DV---------------W-
	Bacillus marisflavi	WP_121620509	---------------KQ-S-T		DV-----------------
	Bacillus massiliogabonensis	WP_102274775	--------------AKQ-CD-		DI-----------------
	Bacillus massilionigeriensis	WP_075982109	---------------KQ-S-K		NV-----------------
	Bacillus mediterraneensis	WP_071459948	---------I----A---C--		DL--------------AN-
	Bacillus mesonae	WP_127487926	--------------AKQ----		DL-----------------
	Bacillus methanolicus	WP_004435237	--------------AKQ-CKK		-L-----------------
	Bacillus nealsonii	WP_101176769	--------------AKQ-S--		DV-----------------
	Bacillus notoginsengisoli	WP_118920643	--------------A---S--		DL---------------N-
	Bacillus obstructivus	WP_071976089	-----M---------KQ---T		NV-----------------
	Bacillus oceanisediminis	WP_110064209	--------------AKQ-S-K		DI-----------------
	Bacillus panaciterrae	WP_028402725	---------I----AKQ--AH		PV--I---------I----
	Bacillus persicus	WP_090744525	--------------A---S--		DL-----------------
	Bacillus praedii	WP_057764429	--------------AKQ-CD-		DI-----------------
	Bacillus psychrosaccharolyticu	WP_040376265	--------------AKQ---T		-V-----------------
	Bacillus rubiinfantis	WP_042355288	---------I----AKQ-S--		DF-----------------
	Bacillus selenatarsenatis	WP_041964542	--------------A-Q-S--		DL-----------------
	Bacillus shacheensis	WP_059105136	-----V--------AKQ-S--		-V--M------------W-
	Bacillus smithii	WP_003355118	---------------KQ---S		NS-----------------
	Bacillus soli	WP_066061022	--------------AKQ-SQ-		DI-----------------
	Bacillus sp. 7894-2	WP_095244667	--------------AKQ-S-T		DI-----------S-----
	Bacillus sp. AFS006103	WP_098261622	--------------AKQ-CD-		-I-----------------
	Bacillus sp. AFS015802	WP_098350214	---------------KQ-S--		DV-----------------
	Bacillus sp. AFS073361	WP_098574785	--------------AKQ-SH-		DF-----------------
	Bacillus sp. B-jedd	WP_048825244	--------------A---S--		DL--I------------N-
	Bacillus sp. FJAT-27231	WP_049662861	---------------KQI-RS		NI---------------H-
	Bacillus sp. FJAT-27245	WP_053366939	--------------AK--S--		DL---------------N-
	Bacillus sp. FJAT-27251	WP_053363940	--------------A---S--		DL-----------------
	Bacillus sp. FJAT-27445	WP_059170940	--------------A---S--		NL--I------------H-
	Bacillus sp. FJAT-29814	WP_066312938	--------------AKQ-S--		DI-----------------
	Bacillus sp. FJAT-29937	WP_066294351	--------------AKQ-CE-		DI-----------------
	Bacillus sp. J33	WP_026581086	--------------AKQ-S-K		DI-----------------
	Bacillus sp. Leaf406	WP_056535767	---------------KQ-S--		DV-----------------
	Bacillus sp. M5HDSG1-1	WP_127737632	--------------AKQ-S--		DV-----------------
	Bacillus sp. MUM 13	WP_071351202	--------------AKQ---H		PV-----------------
	Bacillus sp. OV322	WP_090989353	--------------AKQ---H		PV-----------------
	Bacillus sp. PK3_68	WP_120032560	----------------QI-RS		NI---------------H-
	Bacillus sp. SYSU K30001	WP_124563901	---------I----AKQ--Q-		DV------------I----
	Bacillus sp. T33-2	WP_101580064	--------------A------		DI-----------------
	Bacillus sp. UNC41MFS5	WP_026563905	--------------AKQ-SH-		DF-----------------
	Bacillus sp. URHB0009	WP_027323844	-A-------------KQ-S--		DM-----------------
	Bacillus sp. V44-8	WP_117321694	--------------AKQ---T		DV-----------------
	Bacillus sp. VT-16-64	WP_077113330	--------------AKQ-S-Q		DI-----------S-----
	Bacillus sp. X1(2014)	WP_038535830	--------------AKQ----		DL-----------------
	Bacillus sp. Y1	WP_119708929	--------------AKQ-S--		DV-----------------
	Bacillus sp. YLB-04	WP_115452360	--------------A---S--		DL---------------N-
	Bacillus sp. es.034	WP_098440036	---------------KQ-S-K		DV-----------------
	Bacillus subterraneus	WP_044396109	--------------A-Q-S--		DL-----------------
	Bacillus terrae	WP_120117407	---------------KQ-S-T		DV-----------S-----

Other Bacteria (2/>100)

Bacillus tuaregi	WP_071395570	--------------AKQ-S--	NI------------------
Bacillus vietnamensis	WP_034758410	---------------KQ-S-K	DV------------------
Bacillus wudalianchiensis	WP_065409237	---------------KQI-RS	DI----------------H-
Bacillus zeae	WP_119114350	------------------S--	DL------------------
Candidatus Parcubacteria bacte	RJR08849	------------Y-AKK-T-D	-L------------------
Clostridiales bacterium mt7	WP_101730960	--------------AKQ-SN-	DV------------------
Falsibacillus pallidus	WP_114745338	---------------KQ-S-M	DL------------------
Falsibacillus sp. GY 10110	WP_121680777	--------------AKQ---T	-L------------------
Lysinibacillus sp. B2A1	AVK83636	------------V-A---S--	-VG-----------S---Q-
Macrococcus hajekii	WP_133429085	-M---V---I-G----QI-RK	DV------------------
Mycobacteroides abscessus subs	SHS34594	--------------AKQ-S--	DV------------------
Paraliobacillus sp. PM-2	WP_090853451	---------I----AK---N-	-V----------------I-
Planococcus citreus	WP_121299425	----------------D---S	DIG---------------T-
Planococcus donghaensis	WP_065526073	----------------D---S	NIG---------------S-
Planococcus faecalis	WP_078080513	----------------D---S	NIG---------------S-
Planococcus halocryophilus	WP_008497775	----------------D---S	NIG---------------S-
Planococcus halotolerans	WP_112222673	----------------H---S	DIG---------------S-
Planococcus kocurii	WP_058385059	----------------D---S	NIG---------------S-
Planococcus maitriensis	WP_112231962	----------------D---S	DIG---------------T-
Planococcus maritimus	WP_068465707	----------------D---S	DIG---------------T-
Planococcus massiliensis	WP_110925699	----------------D---S	DIG---------------T-
Planococcus plakortidis	WP_068869282	----------------D---S	DIG---------------T-
Planococcus rifietoensis	WP_058383628	----------------D---S	DIG---------------T-
Planococcus salinus	WP_123164464	----------------D--DS	NIG---------------S-
Planococcus sp. CAU13	WP_033541215	----------------D---S	DIG---------------S-
Planococcus sp. PAMC 21323	WP_038704138	----------------D---S	NIG---------------S-
Planomicrobium glaciei	WP_036801963	----------------D---S	DIG---------------S-
Planomicrobium soli	WP_106531759	----------------D---A	DIG---------------T-
Rummeliibacillus pycnus	WP_102692493	------------V-A-D-S-Q	DIG---------------F-
Rummeliibacillus sp. POC4	WP_119415384	------------A-A-D-S-K	DVG-----------------
Rummeliibacillus sp. TYF005	WP_124217840	------------A-A-D-S-K	DVG-----------------
Sporosarcina globispora	WP_053434600	--------------AKQ-S-T	DI------------------
Staphylococcus argenteus	WP_047434720	-M---V---I-G--A---S-K	DI--M---------------
Staphylococcus aureus	WP_086040118	-M---V---I-G--A---S-K	DI--M---------------
Staphylococcus carnosus	WP_015900341	-M---V---I-G----Q-S-K	DV--M---------------
Staphylococcus hominis	RLY74578	-M---V-----G--A-Q---K	DI--M---------------
Staphylococcus schweitzeri	WP_047558804	-M---V---I-G--A---S-K	DI--M---------------
Staphylococcus simiae	WP_002463071	-M---V---I-G--A---S--	DI--M---------------
Staphylococcus simulans	WP_105992765	-M---V---I-G----Q-S-K	DV--M---------------
Staphylococcus sp. EZ-P03	WP_114603338	-M---V---I-G----Q-S-K	DV--M---------------
Staphylococcus sp. SDB 2975	WP_123144728	-M---V---I-G----Q-S-K	DV--M---------------
Viridibacillus arvi	WP_053417231	------------V---D---E	DVG-----------------
Viridibacillus sp. OK051	WP_100796734	---------I--V---D---E	DVG-----------------

Supplemental Figure 84

A partial sequence alignment of the N-acetyl-alpha-D-glucosaminyl L-malate synthase BshA protein containing a 1 amino acid insertion (boxed) that is exclusively shared by all members belonging to the Flavidum clade and absent in all other bacteria. 2 *Staphylococcus* species are exceptions which also share this CSI.

Group	Organism	Accession	149		205
**Flavidum Clade (2/2)**	Planomicrobium flavidum	WP_088005873	GVVCSVSEAQAIGEACGTGFIRLTPGIR	☐	PGNESHDQKRTATPAEALKAGATHIVVGR
	Planococcus sp. Y42	WP_077589676	------K--KR---V--ED-L-V-----		L--D---------EQ-ARE---------
**Other Bacteria (1/>100)**	Desulfovibrio aminophilus	WP_027175274	-----GL--AR-K----A--RC------		-AGG-D--R--V---Q-VA---DYL----
	Absiella dolichum	WP_004797854	------H--R--H----KD-LTV-----	L	SDDSKD----V----F-KEE-CD------
	Aliidiomarina maris	WP_111568953	-----AK--HLLR-SL-AD-LLV-----	P	A-S-Q-----VM--E--IR--VSYL-M--
	Azospirillum brasilense	WP_119508063	-----PA-VAL-R----PD--LMV----	P	AWAAAN----VM------A---D-L-I--
	Azospirillum sp. TSH58	WP_109070692	-----PA-VAL-R----PD--LMV----	P	AWAAAN----VM------A---D-L-I--
	Azospirillum sp. TSO35-2	WP_109110578	-----PA-VALVRA---P---LMV----	P	AWAATN----IM------A---D-L-I--
	Bacillus coahuilensis	WP_010172326	-----PY--DLVRKL--DE-L-V-----	L	-HGDH-----VT--CD-R-L-S-------
	Bacillus ndiopicus	WP_042471769	------H--K--A----ND-L-V-----	M	L-GDA-----I---DG-K-D-SSL-----
	Bacillus sp. FJAT-22090	WP_053590070	------L--KK-A----ND-L-V-----	L	AEGDT---V-V---SN-KVL-SS------
	Bacillus sp. OxB-1	WP_041073928	------H--G--SDT--PS-FKV-----	M	ADGGQ-----I---D--K---S-------
	Bhargavaea beijingensis	WP_092096385	------L--S--RD---EE-LKV-----	L	ADDDT-----V----D-AR--S-------
	Bhargavaea cecembensis	WP_008297289	------H--S--RD---EE-LKV-----	L	ADGDT-----I----D-GR--S-------
	Bhargavaea ginsengi	WP_092049041	------L--S--RD---EN-LKV-----	M	ADGDL-----VS--TD-AR--S-------
	Brochothrix thermosphacta	WP_029092445	-----AH--K-----TAAN-L-V-----	P	LSATVG--R-IM---Q-RAN-S-------
	Caryophanon latum	WP_066463299	------H--R--A----ED-L-V-----	M	L-G-A-----I---DG-K-D-S-Q-----
	Caryophanon tenue	WP_066543271	------H--R--ADV--ED-L-V-----	M	L-GDA-----I---DG-KQD-S-Q-----
	Chlamydia trachomatis	CRH86007	------H--K--H----DD-LTV-----	L	ASDSVG----V----F-KEQ-CDY-----
	Chromohalobacter japonicus	WP_040241706	-----AQ--T-LRAL--DT-LKV-----	P	SASNGD--R--L---R--A--S--L-I--
	Clostridioides difficile	WP_103989486	-----AQ-VDK-V-----D--TVC----	P	KSA-VG----VV--SD-I-K--HYL----
	Deltaproteobacteria bacterium	OGP14168	-----PQ-IE-VRRE--DN-VIV-----	L	-DQ-PD----I----A-I----DY-----
	Desulfallas geothermicus	WP_092484228	-----PR-IELVR----P--KII-----	P	D-AKLG--R-VM------VR---SY--I--
	Desulfofarcimen acetoxidans	WP_015757861	---A-PQ-IR--RQ---PE-VII-----	P	S-GAV-----VT--G--IA----Y-----
	Desulfofarcimen intricatum	WP_114638053	---A-PW-IS--RQ---RE-VII-----	P	V-GDI-----VM--K--VA----Y-----
	Desulfovirgula thermocuniculi	WP_027717204	---A-AR--A--RQS--PD-LIV-----	P	A-APAG----V------------L-----
	Domibacillus robiginosus	WP_050181507	------L--A--R----EA-YKV-----	L	KDGQAD----I-D----R-I-S-M-----
	Ectothiorhodospira haloalkalip	WP_025281607	-----AR--PVLRQEL--E-LKV-----	P	R-S-AG----IV---D-IRD----L-I--
	Ectothiorhodospira marina	WP_090250412	-----AR--A-LR-DL-AA-LKV-----	P	R-S-AG----IV---D--RD----L-I--
	Ectothiorhodospira sp. BSL-9	WP_063465682	-----AR--PGLRQEL----LKV-----	P	R-S-AG----IV---D-IRD----L-I--
	Edaphobacillus lindanitolerans	WP_076756562	------H--S--RSL--DS-LKV-----	L	A-SGTQ----I---E--R---S-------
	Enterococcus casseliflavus	WP_005226112	-----AH--KQ-KQRTTDA---------	P	A-S-VG----IM---Q-R-----M-----
	Faecalibaculum rodentium	WP_075885244	------H-----H----PE-LTV-----	L	ASDSKD----V----L-RER-CDY-----
	Faecalitalea sp. Marseille-P37	WP_102267136	------H--K--H----SD-LTV-----	M	ASDSVD----V----Y-KEQ-CDY-----
	Fictibacillus arsenicus	WP_077361401	------Q--NS-KKI--DD-LTV-----	P	Q-ADT---A-I------ARM-SDFM-I--
	Fictibacillus gelatini	WP_026675932	-----PL--GL-K-K--SA-LTV-----	M	E-D-KN--------S--R-L-SDF-----
	Fictibacillus phosphorivorans	WP_066393516	------Q--MK-K-V--KA-LTV-----	P	S-ADT---A-V---K--ALK-SDYM-I--
	Fictibacillus sp. BK138	WP_130295739	------Q--SH-KKR--AD-LTV-----	P	Q-ADT---A-I------ARM-SDFM-I--
	Filibacter sp. TB-66	WP_124070976	------H-SAV-A----NE-LKV-----	L	ADSGT-----I---E--HL--S-------
	Geothrix fermentans	WP_052572053	-----AW--A--RD---E--H-------	P	A-AATQ--A-VM---Q---Q-S-WL----
	Halomonas aestuarii	WP_071941556	-----AQ--RRLQAL---D-LKV-----	P	SFAAAG--R-VM------R--S--L----
	Halomonas axialensis	WP_058576291	-----AQ--AQ-EAQ--SA-LKV-----	P	SFAAAN--Q-IM---D-MQ--S--L----
	Halomonas caseinilytica	WP_064702600	-----SQ--ERLR-S--PS-LKV-----	P	ASAAAD--R-IM--SA-MA--S--L----
	Halomonas daqingensis	WP_103968256	-----AQ-STRLRQL---D-LKV-----	P	RSA-HG--R-VM-----MA--S--L-I--
	Halomonas desiderata	WP_086508799	-----AQ-STRLRQL---D-LTV-----	P	RSA-HG--R-VM-----MA--S--L-I--
	Halomonas elongata	WP_112049466	-----SM--ERLR-T--DA-LKV-----	P	TTAAAD--R-IM---S-MA--S--L----
	Halomonas ilicicola	WP_072822267	-----SQ--E-VRR---E--LKV-----	P	ENSA-D--R-IL--SG--A--S--L-I--
	Halomonas jeotgali	WP_017430006	-----AR--E-LA-S--EA-LKV-----	P	AFAAAN--Q-IM--SA-VQ--S--L----
	Halomonas lionensis	WP_083024582	-----AQ--AQ-KAQ--SA-LKV-----	P	SFAAAN--Q-IM---D-MQ--S--L----
	Halomonas lutea	WP_019018232	-----AQ-SARLR-I--DA-LKV-----	P	AFA-TG--R-VM-----MA--S--L----
	Halomonas shengliensis	WP_089680270	-----AQ---RLR-R--DA-LKV-----	P	SFAAAG--Q-IM---A-MA--S--L----
	Halomonas sinaiensis	WP_064700259	-----SQ--ERLR-S--PS-LKV-----	P	ASAAAD--R-IM--SA-MA--S--L----
	Halomonas songnenensis	WP_106376076	-----AQ--AHLKQV--E--LKV-----	P	SFAAAN--Q-IM--S--MR--S--L-I--
	Halomonas sp. 362.1	WP_129138851	-----AQ---RLRDV--DT-LKV-----	P	SFAAAD--R-VL-----IR--S--L----
	Halomonas sp. KM-1	WP_010629006	-----AQ-STRLRQL--AD-LKV-----	P	RSA-HG--R-VM-----MA--S--L-I--
	Halomonas taeanensis	WP_092524409	-----AQ--T-LRDL--ED-LKV-----	P	ASAAAD--R-VL--SA-MA--S--L----
	Halomonas urumqiensis	WP_102586298	-----AQ--A-LRTR--DD-LKV-----	P	SFADAF--R-IM---K-MA--S--L----
	Halomonas xianhensis	WP_134017233	-----AR-SAR-R-I--DS-LKV-----	P	ANG-AG--R-IM---D-MA--S--L----
	Idiomarina woesei	WP_055437859	-----AQ---M-AQQ--D----V-----	P	V-A-QG--R-VM--P--AAL-IEVL-I--
	Kurthia gibsonii	WP_087682423	------L--KG-E----KT-FKV-----	L	A-G-------V---DF-KSE-S-Q-----
	Kurthia huakuii	WP_029498171	------L--NM-E----KE-LKV-----	L	A-GAT-----V---QV-REE-S-Q-----
	Kurthia massiliensis	WP_010286523	------H--KM-E----KA-LKV-----	L	A-G-T-----V---QV-RQE-S-Q-----
	Kurthia senegalensis	WP_010301692	------H--KM-E----KD-LKV-----	L	A-GAT-----V---QV-RIE-S-Q-----
	Kurthia sibirica	WP_109304460	------L--HD-EK---KA-LKV-----	L	V-G-A-----V---NF-KEQ---Q-----
	Kurthia sp. 11kri321	WP_068455035	------L--KG-E----KT-FKV-----	L	A-G-------V---DF-KSE-S-Q-----
	Kurthia sp. 3B1D	WP_126990792	------L--KM-E----KA-LKV-----	L	A-GAT-----V---QV-RAE-S-Q-----
	Kurthia sp. Dielmo	WP_020189388	------L--KM-E----KA-LKV-----	L	A-GAT-----V---QV-RAE-S-Q-----
	Kurthia zopfii	WP_126341874	------L--KV-A----DE-FKV-----	L	A-G-A-----V---QF-KSE-S-Q-----
	Listeria monocytogenes	PDF99363	-----PL--EM-SKEL--D-LKV-----	P	K-AARN--Q-IT--E--KTL-S-------
	Lysinibacillus acetophenoni	WP_097147948	------H--AT-S-V--ED-L-V-----	L	A-G-------V---NF-RNH-SSL-----
	Lysinibacillus boronitolerans	WP_036077160	------H--K--A----DE-L-V-----	L	A-GAA-----I---DG-KRD-SSL-----
	Lysinibacillus chungkukjangi	WP_107933901	------H--GQ------QE-L-V-----	L	--G-A-----I---NG-KAS-SSL-----
	Lysinibacillus composti	WP_124764070	------H--A--RDL--ES-L-V-----	L	A-G-A-----I---DG-K-D-SSL-----
	Lysinibacillus contaminans	WP_053584418	------H--K--A-L--ED-L-V-----	L	A-G-------I---NG-KRD-SSL-----

Other Bacteria (1/>100)

Lysinibacillus endophyticus	WP_121212923	------H--G--R-V--EE-L-V----- L A-G-T-----I---DG-R-D-SSL-----
Lysinibacillus fluoroglycofeni	WP_107942259	------H--K--A-V--ED-L-V----- M L-GDA-----I---DG-KRD-S-------
Lysinibacillus fusiformis	WP_009373525	------H--K--A-V--D--L-V----- L A-GAA-----I---DG-KRD-SSL-----
Lysinibacillus jejuensis	WP_108305728	------H--AM---V--AD-L-V----- L A-G-T-----I---DG-KRD---Q-----
Lysinibacillus manganicus	WP_036181717	------H--GL------DA-L-V----- L A-G-------V---DY-RNN-SSL-----
Lysinibacillus mangiferihumi	WP_107895697	------H-----ADV--QD-L-V----- L A-GDA-----I---DG-RRD-SSL-----
Lysinibacillus massiliensis	WP_036171812	------H--E--RDI--ND-L-V----- M A-GD------I---DG-RSD-SSL-----
Lysinibacillus meyeri	WP_107839220	------H----VA-V--ED-L-V----- M L-GDA-----I---DG-KHD-SSL-----
Lysinibacillus parviboronicapi	WP_107947692	------H--K--A-T--EN-L-V----- L A-GDA-----I---DG-KHD-SSL-----
Lysinibacillus saudimassiliens	CEA02661	------H--AM------AD-L-V----- L A-G-T-----I---DG-KRD---Q-----
Lysinibacillus sinduriensis	WP_036202820	------H--GQ-A-S--QE-L-V----- L --G-A-----I---DG-R-G-SSF-----
Lysinibacillus sphaericus	WP_069510227	------H-----A-V--QD-L-V----- L A-GDA-----I---DG-KRD-SSL-----
Lysinibacillus xylanilyticus	WP_049668369	------H--K--A-V--ED-L-V----- L A-GDA-----I---DG-KRD-SSL-----
Lysinibacillus xyleni	WP_097072075	------H--G--R-T--EE-L-V----- L A-G-A-----I---DG-K-D-SSL-----
Mariprofundus micogutta	WP_072659140	-I----H--A--KQT---N--TV----- W G-QDTQ----V-D--S-ITG-SDYL----
Merdibacter massiliensis	WP_075877735	------H--R--H-----D-LTV----- L ADDSKD----V----Y-KEQ-CD------
Nitrospirae bacterium RBG_16_6	OGW59023	---A-PN-IRL-R-S--PK-VIVS---- P A-S---------E--Q--SD--DYL-I--
Oceanobacillus senegalensis	WP_085992447	------H--T--K----SD-LTV----- L DNS-H-----V---KF-KEN-SDIV-I--
Paenibacillus sp. P1XP2	WP_036707596	---A-AQ--GD-AA----D--TV----- P S-S-QG----VL--G--IRN-SSY-----
Planococcus antarcticus	WP_006828811	------L--E---D---QE-L-V----- P AAVSAD----V----Q-REK-SS------
Planococcus citreus	WP_121297746	------H--K---V---DD-L-V----- P AAS-------I---SD-KAQ-S-------
Planococcus donghaensis	WP_008430771	------L--E-------QN-L-V----- P ATVSAD----I----Q-RAK---------
Planococcus faecalis	WP_071153625	------L------D---QE-L-V----- P A-VLAN----I----Q-REK-SS------
Planococcus halocryophilus	WP_008497997	------L--E-------QD-L-V----- P ASVSAD----V----Q-RIK-S-------
Planococcus halotolerans	WP_112222914	------H--E---D---EE-L-V----- P SQVSAD----V------SRQ-S-------
Planococcus kocurii	WP_058384892	------L------D---QE-L-V----- P A-VLAD----I----Q-REK-SS------
Planococcus maitriensis	WP_112231399	------H--K----V--DD-L-V----- P AAS-------I---SD-KAQ-S-------
Planococcus maritimus	WP_069576028	------H--K-------KD-L-V----- P AAA-------I---SD-KTQ-S-------
Planococcus plakortidis	WP_068869437	------H--E---N---ED-L-V----- P ASS-------I---YD-KMQ-S-------
Planococcus rifietoensis	WP_058383532	------H--K---V---DD-L-V----- P AAS-------I---SD-KAQ-S-------
Planococcus salinarum	TAA68723	------H--E---D---EE-L-V----- P AQVSVD----V---D--RAK-S-------
Planococcus salinus	WP_123164356	------H--K----V--ED-L-V----- P ADVS-D--I-V---RQ-REQ-S-------
Planococcus versutus	WP_049693326	------L-----RD---QE-L-V----- P AAVSAD----I----Q-REK-SS------
Planomicrobium okeanokoites	WP_117312470	------H-SE---D---EE-L-V----- P AQASVD----V------SRQ-S-------
Planomicrobium soli	WP_106534907	------L--K---A---EE-L-V----- P ADVSAD----V---EQ-RQN-S-------
Planomicrobium sp. MB-3u-38	WP_101802004	------H-SE---DS--EA-L-V----- P AQASVD----V------SRQ-S-------
Planomicrobium sp. Y74	WP_121632752	------H--E---D---EE-L-V----- P SQVSAN----V------SRQ-S-------
Pontibacillus yanchengensis	WP_036823072	------A--SI-A-V--DS-Y------- L KDQPHQ--V-I---EK-R----SA-----
Psychrobacillus psychrotoleran	WP_093535268	------L--KK-ASV--EE-L-V----- L AEG-A---V-V----N-RIL-SS------
Psychrobacillus sp. FJAT-21963	WP_056827354	------L--KK-A----ND-L-V----- L AEGDT---V-V---SN-RVL-SS------
Psychrobacillus sp. OK032	WP_093263758	---S--L--KK-T----NE-L-V----- L AEG-A---I-V---SN-RLL-SS------
Rummeliibacillus pycnus	WP_102692725	------L--K--EKV--KE-LKV----- L S-GA------V----F-KEN-S-------
Rummeliibacillus stabekisii	WP_066785266	------L--K--E----KE-LKV----- L E-G-A-----I----V-REKRS-------
Selenomonas ruminantium	WP_073089497	---A-AQ--AL-R----DD-LIV----- P A-SDVN--S-I----S---D-S--L-I--
Selenomonas sp. AE3005	WP_028128473	---A-AQ--AL-R----DD-LIV----- P A-SDTN--S-VV------SN--S-L----
Selenomonas sp. FOBRC9	WP_009657933	---A-PQ--AR-RR---A--LIV----- P A-VSND--R-VT-----IH---S------
Solibacillus isronensis	WP_079525076	------H-----R----DD-L-V----- M L-GD----Q-I---DG-K-D-SSL-----
Solibacillus kalamii	WP_087615457	------L-----R----DD-L-V----- M L-G-A---Q-I---DG-K-D-SSL-----
Solibacillus silvestris	WP_014824529	------H-----R----DD-L-V----- M L-G-A---Q-I---DG-K-D-SSL-----
Solibacillus sp. R5-41	WP_099422716	------H--R--R----ED-L-V----- M L-GQA-----I---DG-RQD-SSL-----
Sporosarcina koreensis	WP_040286913	------L-TGK-AD---KE-LKV----- M ADDAA-----I---TQ-REL-S-------
Sporosarcina newyorkensis	WP_078817795	------Q--KI-E-V--KE-FKV----- L AQGDV-----V----K-RQE-S-------
Sporosarcina psychrophila	WP_067211406	------H--T--ADV--KP-FKV----- L ADGGA-----I---E--RL--S-------
Sporosarcina sp. PTS2304	WP_114923272	------Q--KI-EKV--KD-FKV----- L ASGDV-----V----K-WQE-S-------
Sporosarcina ureae	WP_029052349	------Q--KI-E-V--KD-FKV----- L AQGDV-----V----K-KQE-S-------
Staphylococcus chromogenes	WP_119512723	-----PL-T-L-T-H--SS-LKV----- P ANSARN----VT--EG-KQL-S-------
Staphylococcus epidermidis	RLY71898	-----PL--EM-SKEL--D-LKV----- P K-AARN--Q-IT--E--KTL-S-------
Staphylococcus schleiferi	WP_050331261	-----PL--SLLKAE--EH-LKV----- P ANSR-D----IT--ED-K-R-S-------
Thalassobacillus cyri	WP_093046655	------Q-VK--KA---SS-LTV----- P K-AQQN----S---L--KQQ-TDA-----
Thalassobacillus devorans	WP_028782716	------Q-VK--KA---SS-LTV----- P E-VQQN----S---V--KQQ-TDA-----
Thalassobacillus sp. TM-1	WP_082412047	------H-VK--K----DN-LTV----- P E-SDRN----A---K--KQE-TDS-----
Thermodesulfovibrio sp. RBG_19	OHE60407	---S--R--EI-RNH--K---IV----- P SWTPPD------I-K--IR---DYL-I--
Ureibacillus thermophilus	QBK25578	------H--KQ-A-R--EA-L-V----- L L-GDA-----V---DF-REN-SSY-----
Ureibacillus thermosphaericus	WP_016837478	------H--KN-ADV--ET-L-V----- L --GDA-----I---DY-RIN-SSY--I--
Veillonella dispar	WP_129823789	-----AL--KM-R----DD-LIV----- P SFATTD----V----S--QD--SRL-I--

Supplemental Figure 85

A partial sequence alignment of the orotidine-5'-phosphate decarboxylase protein containing a 1 amino acid deletion (boxed) that is exclusively shared by all members belonging to the Flavidum clade and absent in all other bacteria. *Desulfovibrio aminophilus* is an exception which also shares this CSI.

Group	Species	Accession	105		153
**Flavidum Clade (2/2)**	Planomicrobium flavidum	WP_088005773	KRNLGLTSLQKLLAQIGIAIITFLLL		RNVFDTGIGVPFTDWEIELSWVY
	Planococcus sp. Y42	WP_077589695	----------------AV-LV--FV-		-D----AVSI---AF-V----A-
**Other Bacteria (1/>100)**	Streptohalobacillus salinus	WP_110250489	------------MG--VVGAVF--MA		QDLLVSE-V---SSLT-D-A-L-
	Alkalibacillus haloalkaliphilu	WP_017186035	--------K--MFF-LI---VVYII-	S	NST-P-D-AI-G-NYS-D-G-A-
	Alteribacillus persepolensis	WP_091270230	--------K---AG-LVV---F--V-	N	NQG---SVA--G-N-SV-FG-F-
	Ammoniphilus oxalaticus	WP_120189356	-------AK---FG-LL--V-LYYV-	L	QYH---K-Y--G-S-G---G-A-
	Ammoniphilus sp. CFH 90114	WP_129196854	-------AK---FG-LA--V-LYYV-	M	QYN---RVY--G-S-GV--G-M-
	Aneurinibacillus migulanus	WP_043072682	-------AK---FG-LV-GAVFYYV-	I	--GY--T-SI-GIH-S-DMG-L-
	Aquisalibacillus elongatus	WP_124220175	--------K--M-F-LF--VVVYIV-	H	NHG---T-TI-G-EFSM-FA-F-
	Bacillus aidingensis	WP_026700141	--------K---AG-FL---VF--V-	N	NQG-S-AVA--G-N-SVDFG-F-
	Bacillus aquimaris	WP_113968468	--------K----G--V-SV-FY-IF	K	Q-D-P-TVSI-L--FSF--G-F-
	Bacillus bogoriensis	WP_026674376	--------K---V--LL--VVFYIS-	Q	QLG-S-E-AI-A-T-A-DIG-L-
	Bacillus cecembensis	WP_057985785	------------IG--A--VAA----	R	LGA---S--L-Y--IS-D-GIF-
	Bacillus circulans	WP_123257422	--------R----G--I-SL-FYFV-	R	E-E-S-A-SI--V-KPL--G-F-
	Bacillus dielmoensis	WP_042456175	--------K----G--I-SV-FY-VY	R	QSG-S-E-SI---NTT---G-FF
	Bacillus fastidiosus	WP_066232454	--------R----G-VI--V-FYIVY	T	QFG-S-E--F-G-E-S-D-G-G-
	Bacillus foraminis	WP_132005723	--------K----G--V-SV-FYIIF	R	QSD-S-E-S--L--LSFD-G-A-
	Bacillus horneckiae	WP_066393365	------------IG--I-SV-FYIV-	R	Q-D-S-E-HI-L--YSFD-G-M-
	Bacillus humi	WP_057996828	--------K----G--V----FYFIF	K	Q-N-S-S-SI-G--Y-FD-KIL-
	Bacillus lentus	WP_066139914	--------K---IG--I--VVFYVV-	K	I-G-S-I-SI-L--FS-D-G-G-
	Bacillus marisflavi	WP_121619465	--------K----G--V--A-FYFV-	R	QTG--SSLTI-L--FS-D-G-G-
	Bacillus massilionigeriensis	WP_075981019	--------R----G--V-SV--YFI-	Q	Q-N-S-E-HI-LI-YSLD-G-G-
	Bacillus massiliosenegalensis	WP_019152878	------------IG--IVSVVF--I-	Q	HYE-S-VVSI-L--FS-D-G-G-
	Bacillus mesonae	WP_127486284	--------R----G--I-SV-FY-VY	K	QMN-S-E-NI----SS-D-G-FF
	Bacillus methanolicus	WP_003348884	--------K----G--I-SAVFYFV-	K	T-E-S-DLHI-L--YSLD-G-L-
	Bacillus ndiopicus	WP_042471688	------------IG--I-S-AA-M--	R	LGT---VVEI----FS-DFGLF-
	Bacillus nealsonii	WP_016201008	--------R----G--I-SL-FYFV-	R	E-E-S-A-SI--VEKPL--G-F-
	Bacillus niacini	WP_034673109	--------R----G--I-SV-FY-VY	K	Q-G-P-E-S--GSNYS---G-F-
	Bacillus notoginsengisoli	WP_118922777	--------K----G--I-SV-FY--Y	R	SSELPSA-RL--S-EP-H-G-I-
	Bacillus oleivorans	WP_097159493	--------K---FG--L-S--V-WIM	K	D-GIS-E--I---NI-F--G-F-
	Bacillus sp. 7504-2	WP_095313098	------------IG--IVSV-F--I-	Q	HYD-S-V-SI-L-GFS-D-G-G-
	Bacillus toyonensis	WP_098684462	--------K----G-LI---VF--IG	K	GQA-H-Y-MI-G--VKF----A-
	Bacillus tuaregi	WP_071395429	--------K----G--IVSVVVYF--	K	QME-S-AVSI----YSL--GFF-
	Bacillus vietnamensis	WP_034755857	--------K----G--V-SV-FY-IF	K	Q-D-P-TVSI-L--ISF--G-F-
	Bhargavaea beijingensis	WP_092096321	------------IG--A--VVSY---	R	LGP-E-TV-L---SF--D-GPF-
	Bhargavaea cecembensis	WP_008297323	------------IG--V--VVSY---	R	LGP-E-TV-L---SF--D-GPF-
	Bhargavaea ginsengi	WP_092049097	------------IG--V--V-SY---	R	LGP-E-TV-L---SF--D-GPF-
	Butyricicoccus sp. 1XD8-22	RKJ20111	------------VG--I-S-LS----	R	VGA-Q-TVSI---E-SMD-GII-
	Caenibacillus caldisaponilytic	WP_077614488	--------K---IG--LV---FY---	V	H-G-S-RVD----P-SFH---A-
	Chryseomicrobium excrementi	WP_100352788	------------IG--V----AYF--	Q	LGP---A-AI---T-S-DFGIL-
	Edaphobacillus lindanitolerans	WP_076756587	------------IG--A--VVSY---	K	LGP-E-T--L--SS---D-GPL-
	Filibacter sp. TB-66	WP_124071003	------N-----IG--I--VAA-F--	K	LGP---AVHL--V-FR---GVF-
	Filobacillus milosensis	WP_134339371	--------K--M-G-LF---VVYVV-	H	N-GY--T-T--G--LSV-FG-F-
	Geobacillus genomosp. 3	WP_020959244	--------R--FVG-LI--V-F--VY	R	QSG-S-ALHI-G---SFD-G-A-
	Geobacillus sp. 46C-IIa	WP_081206796	--------R--FVG-LI--V-F-FVY	R	QSG-S-ALHI-G---SFD-G-A-
	Geobacillus thermodenitrifican	WP_108437733	--------R--FIG-LI--V-F-FVY	R	QSG-S-ALHI-G---SFD-G-A-
	Halalkalibacillus halophilus	WP_027963104	--------K--M-G-LV--V-VYFI-	Q	SQG-N-AV-I-G-NLDL-FG-F-
	Halalkalibacillus sp. B3227	WP_101330281	--------K--M-C-VF---VVSII-	Y	NQG---AVDI-A---S--FG-F-
	Halobacillus dabanensis	WP_075036551	--------K--M-G--V--LVVY-I-	R	NSD-P-F-SI-G-SI-LD-G-G-
	Halobacillus litoralis	WP_128522859	--------K--M-G--V--LVVYFI-	R	QTD-P-Y-SI-G-SFDL--G-G-
	Halobacillus mangrovi	WP_085030034	--------K--M-G--I---VVYMI-	R	Q-D-P-T----G-E--V--GFG-
	Halobacillus massiliensis	WP_082234260	--------K--M-G--V---VVYI--	Q	Q-G-P-Y-SI-G--LQL--GIL-
	Halobacillus salinus	WP_079480374	--------K--M-G--V---VVY-I-	R	Q-D-P-T----G--M-F--GFG-
	Halobacillus sp. BBL2006	WP_035544360	--------K--M-G--I---VVY-I-	S	Q-G-P-T----G-SF-V--GFG-
	Halobacillus sp. SKP4-6	WP_128539989	--------K--M-G--V---VVY-I-	Q	Q-G-E-TV-I-G-GI-L--G-G-
	Jeotgalibacillus proteolyticus	WP_104056127	------------IG--F----FYVIF	Q	A-G-SSI-AI---TV--N-GFL-
	Jeotgalibacillus sp. R-1-5s-1	WP_134371987	------------IG--V--L-FYFIF	R	A-D-SSV-TI---EF-LN-GI--
	Jeotgalibacillus sp. S-D1	WP_133375957	--------K----G--I--V-FY-IF	Q	A-G-SSIVSI---SF--N-GF--
	Kurthia zopfii	WP_109349160	--------I---I---V-SVVAY---	K	GAG--NHLSI-Y--I-FN-GYF-
	Lysinibacillus boronitolerans	WP_016992541	------------IG--V---LAYF--	H	VGS---TLAI---E-T-D-DVF-
	Lysinibacillus chungkukjangi	WP_107933843	------------IG--I-S-AA----	R	LGS---S-S----E-S-D-GIL-
	Lysinibacillus composti	WP_124764097	--------I---VG--V-S-AA----	R	LGT---S-TI-Y--LS-D-GIL-
	Lysinibacillus contaminans	WP_053584447	--------I---IG--I-S-LVYF--	H	VAS---TLAI-----S-D-GIF-
	Lysinibacillus endophyticus	WP_121212896	--------I---IG--I-S-AA----	R	LGT---S-SI----IS-DFGIL-
	Lysinibacillus fusiformis	WP_004224845	------------IG--V---LAYF--	H	VGS---TLAI---E-T-DHDVF-
	Lysinibacillus halotolerans	WP_122970270	------------IG--I-S-AA----	R	LGS---SV-I-----S-D-GIL-
	Lysinibacillus macroides	WP_053993832	------------IG--V---LAYF--	H	IAS---TLAI---E-I-D-EVF-
	Lysinibacillus manganicus	WP_036181791	------------VG--I-SVLA----	R	QGT---TVT---VE-S-N-GIF-
	Lysinibacillus massiliensis	WP_036171886	------------VG--I-S-LS----	R	VGA-Q-TVSI---E-SMD-GII-
	Lysinibacillus meyeri	WP_107839252	------------IG--I-S-AA-M--	R	LGT-N-VVEI---GYS-DFGLF-
	Lysinibacillus odysseyi	WP_036156061	------------IG--V---AA----	R	LGT---A-AI---E-QVD-GVL-
	Lysinibacillus parviboronicapi	WP_107923161	--------I---IG--V---LAYF--	H	VGS---TLAI---E-T-D-GIF-
	Lysinibacillus saudimassiliens	CEA02590	--------I---IG--V----AYV--	R	G-E---SVTI-LV--SVD-GML-
	Lysinibacillus sinduriensis	WP_036202749	------------IG--I-S-AA----	R	LGS---S-N---I--S-D-GIL-

Other Bacteria (1/>100)

Lysinibacillus sp. B2A1		AVK83057	------------IG--V--VLAYF--	H	VGS---TLAI----MTFD-GIF-
Lysinibacillus sp. BF-4		WP_036141113	--------I---IG--I---VAY---	R	G-E---SVTI-LV--SVD-GML-
Lysinibacillus sp. BK089		WP_132357555	------------IG--V---LAYF--	H	VGS---TLAI-----T-D-GIF-
Lysinibacillus sp. FJAT-14222		WP_053594643	------------IG--V---LAYF--	H	VGS---TLAI-----T-D-GIF-
Lysinibacillus sp. FJAT-14745		WP_053483395	------------IG--V--VLAYF--	H	VGS---TLAI-----T---GNF-
Lysinibacillus sp. Marseille-P		WP_106784042	------------IG--I-S-LS----	R	LGA-E-S-TI--I--SMD-GI--
Lysinibacillus sp. OL1		WP_131521629	------------IG--V---LAYF--	H	VGS---TLAI---E-T-D-DVF-
Lysinibacillus sp. SYSU K30002		WP_126657654	------------IG--I-S-AS----	R	LGS---S-SI-Y---S-D-GIL-
Lysinibacillus sp. YLB-03		WP_118874749	------------IG--I-SVAA----	R	LGS---S--I-----S-D-GIF-
Lysinibacillus sp. ZYM-1		WP_054611688	------------IG--V---LAYF--	H	VGS---TLAI---ELTVD-GVF-
Lysinibacillus sphaericus		WP_010858331	--------I---IG--V---LAYF--	H	VGS---TLAI-----T-D-GIF-
Lysinibacillus xylanilyticus		WP_068983251	------------VG--V--VLAYF--	H	VGS---TLAI---E-TVD-GNF-
Lysinibacillus xyleni		WP_097072047	--------I---IG--I-S-AA---I	R	LGD---T-SI----ISVDFGIL-
Marinococcus halophilus		WP_079476096	--------I---VG--V--F-F-IV-	Y	NGT---SVA--G-G-SL-FG-A-
Marinococcus halotolerans		WP_022792693	--------I---IG--V--F-F-IV-	Y	NGT---SVA--G-E-SL-FG-A-
Marinococcus luteus		WP_091610207	--------I---IG--V--F-F-IV-	Y	NGT---SVA--G-E-SL-FG-A-
Melghiribacillus thermohalophi		WP_132370269	RQ------R------VL----VYII-	D	-HG-P-Y-A--L--IQL--GFG-
Mycobacteroides abscessus subs	E	SHT22772	--------K---FG--V-SF-FYFI-	R	Q-E-S-TVSI-L--ISFD-G-L-
Oceanobacillus arenosus		WP_115773646	-----------FIG--V--LVF-FI-	R	SHD-P-Y-QI-G--IQW--G-A-
Oceanobacillus rekensis		WP_087973009	--------K----G--I--LVF-FI-	R	GHE-S-A-HI-G--IQWD-G-A-
Paenisporosarcina antarctica		WP_134210149	------------IG--V---AS-M--	I	QGP-E-TVS------S-D-GIA-
Paenisporosarcina sp. TG20		WP_019413889	------------IG--I---AS----	Q	QGP-A-TLSI---E-SVDFGVA-
Piscibacillus halophilus		WP_091771987	--------K--M-F-LF--VVVYIV-	H	NQGY--TVTI-G--FS--FG-F-
Planococcus antarcticus		WP_006828835	------------I---V--V-S-VV-	S	SVD-E--LSI----IS-----L-
Planococcus citreus		WP_121297762	----------------V--VLS-F--	R	A-G---AL-I----VS---G---
Planococcus donghaensis		WP_065525913	------------I---I--VVS-FV-	S	GSD-E--VDI---NIS-----L-
Planococcus faecalis		WP_071153615	------------I---V--V-S-FV-	S	GTD-E--VTI----ISF----L-
Planococcus halocryophilus		WP_065528558	------------I---I--V-S-FV-	S	GSD-E--VNI---NIS-----L-
Planococcus halotolerans		WP_112222938	------------I---L--VVS----	G	MVE----LVI---SIS----GL-
Planococcus kocurii		WP_058384869	------------I---V--V-S-FV-	S	GTD-E--VTI----ISL----L-
Planococcus maitriensis		WP_112231365	----------------V--VLS-F--	R	A-G---AL-I----AS---G---
Planococcus maritimus		WP_068462969	----------------V--VLS-F--	R	A-G---AL-I---EAS---G---
Planococcus massiliensis		WP_110925592	------------IG--V--A-S-F--	N	GVG---AVSI---SVSL---M--
Planococcus plakortidis		WP_068869458	-------------G--V--VLS-F--	R	A-G---AL-I----AS---G-I-
Planococcus rifietoensis		WP_058383509	----------------V--VLS-F--	R	A-G---AL-I---EVS---G---
Planococcus salinarum		TAA72078	------------I---F--VVS-F--	G	MIE----LVI---TFS----GL-
Planococcus salinus		WP_123164378	------------V---V--V-S-F--	N	EVD----LM----GIS-D-AGI-
Planococcus sp. CAU13		WP_033543522	------------IG--V--V-S----	G	MIE----LTI---SIS----GL-
Planococcus sp. PAMC 21323		WP_038705434	------------I---I--VVS--V-	S	NGN-E--VNI---NFS-----L-
Planococcus versutus		WP_049693349	----------------V--V-S--V-	N	GID-E--LSI---SIS-----L-
Planomicrobium glaciei		WP_115650566	------------I---V--V-S----	N	GTE---SVSI----AA----V--
Planomicrobium soli		WP_106534883	------------V---V--VVS-F--	N	SID---ALSI---SIS----GL-
Planomicrobium sp. Y74		WP_121632799	------------I---L--VVS----	G	MVE----LVI---SIS----GL-
Pontibacillus chungwhensis		WP_036778450	--------K--MVG-LF--AVFYII-	R	-HD-P-Y--I-G--FQVD-G-G-
Pontibacillus halophilus		WP_026799681	--------K---A--FV--L--YII-	E	--G-S-Y-EI---SIQF--G-A-
Psychrobacillus sp. OK028		WP_093059933	--------I---IG--L--VLAYF--	K	QGP---T-EI---QLDLP-GQL-
Psychrobacillus sp. OK032		WP_093263889	--------I---IG--V-S-VAYF--	K	LGP---SLAI---NVDFS-GQF-
Rummeliibacillus pycnus		WP_102692751	------------I---V-S-AAYF-M	K	LGT-HNTVQI----FA-H-GVF-
Rummeliibacillus sp. POC4		WP_119414203	------------I---V-S-AAYF--	K	MGT-HNTVQI----FA-H-GLT-
Rummeliibacillus sp. TYF005		WP_124217209	------------I---V-S-AAYF--	K	MGT-HNTVQI----FA-H-GLT-
Rummeliibacillus stabekisii		WP_066785199	------------IG--V-SVAAYF-M	R	LGT-HNT-QI----YA-H-GAI-
Saliterribacillus persicus		WP_114352795	--------K---IG--L---VFYVV-	K	MQN---S--I-G-AFD---G-F-
Solibacillus sp. R5-41		WP_099422684	------------IG--A--VAA----	R	LGA-E-S--L-Y--IS-D-GIF-
Sporosarcina koreensis		WP_040286937	------------IG--I---FA-F--	K	LGP---V-SI--M-F--DMGVF-
Sporosarcina pasteurii		WP_115360380	------------IG--VV--LV-F--	K	MGP-E-TVAI--L-IKFD-GIF-
Sporosarcina psychrophila		WP_067211472	--------I---IG--IV-VAA-F--	K	LGP---AVQI---EF----GVF-
Tenuibacillus multivorans		WP_093855760	--------K--M-C--F--VVAYIV-	H	NQGY--T-T--G--FTL-FG-F-
Tetzosporium hominis		WP_094941584	------------IG--V----AYF--	Q	LGP---A-SI---T-S-DFGIL-
Thalassobacillus cyri		WP_093046621	--------K------FV--VVVYMI-	R	QHN-P-Y----A--IQLD-GLL-
Thalassobacillus devorans		WP_085508078	--------K------FV--VVVY-I-	R	QHN-P-Y-A--A--IQVD-GLL-
Thalassobacillus sp. TM-1		WP_062445744	--------K------FV--VVVY-I-	R	QHN-P-Y-A--A--IQVD-GLL-
Ureibacillus thermophilus		QBK25551	------------IG--V-S-LA----	R	LGT---S-SI--K--S-D-GIL-
Ureibacillus thermosphaericus		WP_016837508	------------VG--I-S-LA----	R	LGT---S-SI---E-SVD-GIL-

Supplemental Figure 86

A partial sequence alignment of the phospho-N-acetylmuramoyl-pentapeptide-transferase protein containing a 1 amino acid deletion (boxed) that is exclusively shared by all members belonging to the Flavidum clade and absent in all other bacteria. *Streptohalobacillus salinus* is an exception which also shares this CSI.

			282		331
***Caryophanon* (2/2)**	Caryophanon latum	WP_066462903	KNRLFNQILAETLVNPETGEV	YQDEEGNT	LFTKGTLLDRRTLDLLIPLLE
	Caryophanon tenue	WP_066542789	-------V-------------	--------	---------------------
**Other Bacteria (0/>100)**	Anoxybacillus tepidamans	WP_027410228	-------R------------I		IAE---MI------RIL-Y--
	Bacillus acanthi	WP_108672023	-------RI-----D-----I		IVE-----------RI--H--
	Bacillus alcalophilus	WP_003322601	-------R-----ID------		-AE---M-------RI--Y--
	Bacillus alkalinitrilicus	WP_078429456	-------R------D------		IADE-------A--RI--Y--
	Bacillus anthracis	WP_047399141	-------R------D-----I		-AAE--I-------RIL-Y--
	Bacillus aquimaris	WP_071617112	-----G-T-----AD-----I		-AE---V-------KV--Y--
	Bacillus azotoformans	WP_035198181	-------RV-----D------		-VE---V-------RIL-Y--
	Bacillus cecembensis	WP_057986325	-------TI-----DS----I		-VEA----------K---Y--
	Bacillus cereus	WP_098207359	-------R------D-----I		-AAE--I-------RIL-Y--
	Bacillus circulans	SPU18633	-------R---K--D------		-AEE----------K---H--
	Bacillus clausii	WP_095336812	-------R---K--D------		-AEE----------K---H--
	Bacillus cohnii	WP_066414009	-------T------D-----I		IAE---M-------RIL-Y--
	Bacillus fastidiosus	WP_066227096	-------R------D-----I		IAE----I------RI--N--
	Bacillus glycinifermentans	WP_048406282	-------R------D-----I		-AE---I-------KVL-Y--
	Bacillus hemicellulosilyticus	GAE31479	-------R-----ID------		IAEE----------RIL-Y--
	Bacillus krulwichiae	WP_066159537	-------R------D------		IAEE----------RIL-YI-
	Bacillus litoralis	WP_121664179	-------R------D-----I		IAE---MI------RI--N--
	Bacillus manliponensis	WP_034642626	-------R------D-----I		-AAE--I-------RIL-Y--
	Bacillus marisflavi	WP_048007602	-------T-----AD-----I		-AE---V----A--KI--Y--
	Bacillus marmarensis	WP_022630048	-------R------D------		IAEE----------RIL-N--
	Bacillus massiliosenegalensis	WP_019157078	-------R------D-----I		IAE--V--------RI--Y--
	Bacillus mycoides	AAK74098	-------R------D-----I		-AAE--V-------RIL-Y--
	Bacillus ndiopicus	WP_042478608	-------TI----AD-----I		-VE---V-------KI--Y--
	Bacillus okhensis	WP_034631510	-------R------D------		IAEE----------RIL-Y--
	Bacillus panaciterrae	WP_028400850	-------R------D-----I		-VSE--I-------RIL-Y--
	Bacillus patagoniensis	WP_078393387	-------R---K--D------		-AEE----------K---H--
	Bacillus pseudalcaliphilus	KMK74856	-------R-----ID------		-AE-----------RI--Y--
	Bacillus pseudofirmus	WP_012957129	-------R------D------		IAEE----------RIL-N--
	Bacillus pseudomycoides	PGZ98875	-------R------D-----I		-AAE--I-------RIL-Y--
	Bacillus rhizosphaerae	SHM02853	-------R---K--D------		-AEE----------K---H--
	Bacillus shacheensis	WP_059105429	-------R------D------		-AEE--V----V--R---H--
	Bacillus sp. 123MFChir2	WP_020062286	-------R------D-----I		-VAE--I-------RIL-Y--
	Bacillus sp. 7504-2	WP_095313704	-------R------D-----I		IAE--A--------RI--H--
	Bacillus sp. AFS001701	WP_098232297	-------RI-----D-----I		IVE---T-------RI--F--
	Bacillus sp. AFS018417	WP_098309994	-------R------D-----I		-AAE--I-------RIL-Y--
	Bacillus sp. AFS040349	WP_098798331	-------R------D-----I		IAE---MI------RI--N--
	Bacillus sp. AFS041924	WP_098809318	-------RI-----D-----I		IVE---T-------RI--F--
	Bacillus sp. AFS053548	WP_098862510	-------RI-----D-----I		IVE---T-------RI--F--
	Bacillus sp. AFS088145	WP_098428742	-------RI-----D-----I		IVE---T-------RI--F--
	Bacillus sp. B14905	EAZ83453	-------TI-----D-----I		-VE---V-------KIL-Y--
	Bacillus sp. DL7	ADZ96245	-------R------D-----I		-AAE--I-------RIL-Y--
	Bacillus sp. DSL-17	WP_136357937	-------R------D-----I		IAE---MI------RI--N--
	Bacillus sp. EAC	WP_088044566	-------RI-----D-----I		IVE---T-------RI--F--
	Bacillus sp. FJAT-25509	WP_056467386	-------RI-----D-----I		IVE---T-------RI--F--
	Bacillus sp. FJAT-44742	WP_100400585	-------R------D------		-AEE-AVI------R---Y--
	Bacillus sp. FJAT-45086	WP_100405987	-------R------D------		IAEE----------RI--Q--
	Bacillus sp. FJAT-45385	WP_088106039	-------R------D------		IAEE----------RIL-Y--
	Bacillus sp. JCM 19045	GAF14837	-------R---K--D------		-AEE----------K---H--
	Bacillus sp. JCM 19046	GAF18692	-------R---K--D------		-AEE----------K---H--
	Bacillus sp. JCM 19047	GAF22578	-------R---K--D------		-AEE----------K---H--
	Bacillus sp. M6-12	WP_101596649	-------KI-----D-----I		IVE-----------RI--S--
	Bacillus sp. Marseille-P3800	WP_099305481	-------R---K--D------		-AEE----------K---Y--
	Bacillus sp. SYSU K30001	WP_124565494	-------RV-----D-----I		-VSE--I-------RIL-Y--
	Bacillus sp. UMB0899	WP_102232780	-------R------D-----I		IAE---MI------RI--N--
	Bacillus sp. V47-23a	WP_117326943	-------KI-----D-----I		IVE-----------RI--SI-
	Bacillus testis	WP_050613625	-------RI-----D-----I		-VE-----------RIL-Y--
	Bacillus thuringiensis	WP_080711266	-------R------D-----I		-AAE--I-------RIL-Y--
	Bacillus velezensis	AYO51734	-------R------D-----I		-AE--QI-------KVL-Y--
	Bacillus wakoensis	WP_034745200	-------R------D------		IAEE----------RIL-Y--
	Bacillus wiedmannii	WP_098831010	-------R------D-----I		-AAE--I-------RIL-Y--
	Butyricicoccus sp. 1XD8-22	RKJ07608	------HTI-----D-----I		-VE---V-------R---S--
	Chryseomicrobium excrementi	WP_100354134	-------TI-----D-----I		-VEE--MI---V--R---N--
	Jeotgalibacillus alimentarius	WP_041120828	-----G-V-----AD-----I		IAE--------N--K---N--
	Jeotgalibacillus proteolyticus	WP_104059731	-----G-V-----AD-----I		-AE---T----N--K---Y--
	Jeotgalibacillus salarius	WP_134382771	-----G-V-----AD-----I		-AE--------N--K---N--
	Jeotgalibacillus soli	WP_041085425	-----G-V-----AD-----I		-AE--------N--K---Y--
	Jeotgalibacillus sp. R-1-5s-1	WP_134374643	-----G-V-----AD-----I		-AE---T-------K---Y--
	Klebsiella pneumoniae	OON81170	-------R------D-----I		-AAE--I-------RIL-Y--
	Kurthia senegalensis	WP_010308019	-------T------------I		-AE---VI---V--RIT-F--
	Kurthia sibirica	WP_109305689	-------TI----ID-----I		-VE----I---V--R---H--
	Kurthia zopfii	WP_109349823	-------TI-----D-----I		-VE---VI---V--KI--N--
	Listeria innocua FSL S4-378	EFR92086	-------T------D-----I		IAS--DI----N--QI--N--

Other Bacteria (0/>100)

Lysinibacillus acetophenoni	WP_097149277	-------TI-----D-----I	-VE---I----N--R---Y--
Lysinibacillus composti	WP_124766158	-------TV-----D-----I	-VE--KM-------K---Y--
Lysinibacillus endophyticus	WP_121214177	-------TI-----D-----I	VVE---T-------RI--Y--
Lysinibacillus fusiformis	WP_025116451	-------TI-----D-----I	-VE---V-------KIL-Y--
Lysinibacillus halotolerans	WP_122971261	-------TV-----D-----I	-VE---T-------RI--Y--
Lysinibacillus macroides	WP_053997223	-------TI-----D-----I	-VE---V-------KIL-Y--
Lysinibacillus manganicus	WP_036183188	-------TV-----D-----I	-VE---V----N--R---Y--
Lysinibacillus massiliensis	WP_036172410	-------TI-----D-----I	-AE---V----N--R---Y--
Lysinibacillus meyeri	WP_107839063	-------TI----AD-----I	-VE---V-------KI--Y--
Lysinibacillus odysseyi	WP_036151986	-------TI----AD------	-VE--A-I------K---Y--
Lysinibacillus parviboronicapiens	WP_107925080	-------TI-----D-----I	-VE---V-------KIL-Y--
Lysinibacillus sp. B2A1	AVK86705	-------TI-----D-----I	-VE---V-------KIL-F--
Lysinibacillus sp. BK089	TCJ69729	-------TI-----D-----I	-VE---V-------KIL-Y--
Lysinibacillus sp. FJAT-14222	WP_053596049	-------TI-----D-----I	-VE---I-------KIL-Y--
Lysinibacillus sp. FJAT-14745	WP_053482821	-------TI-----D-----I	-VE---V-------KIL-Y--
Lysinibacillus sp. LK3	WP_048395558	-------TI-----D-----I	-VE---V-------KIL-Y--
Lysinibacillus sp. Marseille-P	WP_106781849	-------TV-----D-----I	-VE---I----N--R---Y--
Lysinibacillus sp. SYSU K30002	WP_126658161	------HTI-----D-----I	-VE---V-------RI--Y--
Lysinibacillus sp. YLB-03	WP_118876527	-------TI-----D-----I	IVE---T----N--RI--Y--
Lysinibacillus sp. YR326	WP_134022302	-------TI-----D-----I	-VE---V-------KIL-Y--
Lysinibacillus sp. ZYM-1	WP_054610137	-------TI-----D-----I	-VE---V-------KIL-Y--
Lysinibacillus sphaericus	WP_010860616	-------TI-----D-----I	-VE-----------KIL-Y--
Lysinibacillus telephonicus	WP_126292330	-------TI--A--D-----I	-VE---V-------RI--Y--
Lysinibacillus varians	AHN23753	-------TI-----D-----I	-VE---V-------KIL-Y--
Lysinibacillus xylanilyticus	WP_068986552	-------TI-----D-----I	-VE---V-------KIL-Y--
Lysinibacillus xyleni	WP_097073461	------HTI-----D-----I	IVE---T-------RI--Y--
Planococcus massiliensis	WP_052654196	-------TI-----D-----I	-VEA--VI---V--R---Y--
Planococcus versutus	WP_049694532	-------TI-----D-----I	-VEA---I---V--R---N--
Planomicrobium glaciei	WP_036803863	-------TI-----D-----I	-VEA--VI---V--R---Y--
Planomicrobium koreense	WP_135500614	-------TI-----D-----I	-VEA---I---V--R---Y--
Planomicrobium soli	WP_106534485	-------TI-----D-----I	-VEA---I---V--R---N--
Psychrobacillus psychrotoleran	WP_093537873	-------TI-----D-----I	-VEA---I---V--R---H--
Psychrobacillus sp. OK028	WP_093062478	-------TI-----D-----I	-VEA---I---V--R---H--
Psychrobacillus sp. OK032	WP_093274848	-------TV-----D-----I	-VEA---I---V--K---H--
Rhizophagus irregularis	PKC50745	-------TI-----D-----I	-AE---V----N--R---Y--
Rummeliibacillus pycnus	WP_102693537	-------TI----AD-----I	-VEA---I---V--R---Y--
Salipaludibacillus aurantiacus	WP_093052684	-------R------D-----I	-AEE---I---V--K---Y--
Salipaludibacillus sp. KQ-12	WP_110611189	--------------D-----I	-AEE-S-I---L--K---Y--
Scopulibacillus darangshiensis	WP_132747082	-------R---K-ID-----I	VADE----------R-L-II-
Solibacillus isronensis	WP_008406991	-------TI-----D-D---I	IVEA-------N--K---Y--
Solibacillus sp. R5-41	WP_099425607	-------TI-----DS----I	-VEA----------K---Y--
Sporolactobacillus inulinus	WP_047035059	-------R--QK--D-----I	VADE----------R-L-TI-
Sporolactobacillus laevolactic	WP_023511464	-------R---K--D-----I	VADE----------R-L-TI-
Sporolactobacillus nakayamae	WP_093674093	-------R---K--D-----I	VADE----------R-L-TI-
Sporolactobacillus terrae	WP_028977333	-------R--QK--D-----I	VADE----------R-L-TI-
Streptococcus pneumoniae	CJF84031	-------R------D-----I	-AAE--I-------RIL-Y--
Tetzosporium hominis	WP_094942220	-------TI-----D-----I	-VEE--MI---V--R---N--
Tuberibacillus sp. Marseille-P	WP_085520799	-------R---K--D------	VADE----------R-L-TI-
Ureibacillus thermophilus	QBK24918	-------RI-----D-----I	IVE---I-------KIL-Y--

Supplemental Figure 87

A partial sequence alignment of the DNA-directed RNA polymerase subunit beta protein containing an eight amino acid insertion (boxed) that is exclusively shared by all members belonging to the genus *Caryophanon* and absent in all other bacteria.

Group	Species	Accession	63		113
Caryophanon (2/2)	Caryophanon tenue	WP_083998246	NNLRVFREAGLVKELTYGDSSSRFDFV	LG	ATDHYHMICEDCGTIVDFHYPG
	Caryophanon latum	WP_083995309	---------------------------	--	----------------------
**Other Bacteria (0/>100)**	Aeribacillus pallidus	WP_063386250	--------V---------A-------		T-E---V---E--K--------
	Anoxybacillus amylolyticus	WP_066323085	------K-V-----------------		TS----I---R--K--------
	Anoxybacillus sp. B2M1	ANB57914	------K-V-----------------		TS----I---R--K--------
	Anoxybacillus tepidamans	WP_027410669	------K-V-----------------		TS----I---Q--K--------
	Anoxybacillus vitaminiphilus	WP_111646305	------K-V-----------------		TS----I---E--K--------
	Bacillus acanthi	WP_108670561	------K-V---------------Y-		T-----V--QK--A--------
	Bacillus acidicola	WP_066266241	--------V-----------------		T-E---V---G--K--------
	Bacillus acidiproducens	WP_018663883	--------I---------A-------		TSN---I------K--------
	Bacillus aerophilus	WP_041506701	--------S----------------A		TS----A---N--K--------
	Bacillus altitudinis	AHI58976	--------S----------------A		TS----A---N--K--------
	Bacillus alveayuensis	WP_044749483	--------V---------A-------		TS----I---E--K--------
	Bacillus aquimaris	WP_113970028	--------V---------A-------		T-----V--DG--K--------
	Bacillus bacterium	HBZ11255	--------V---------A-------		TS----I---E--K--------
	Bacillus badius	WP_041101653	--------V---------A-------		T-----V---E--K--------
	Bacillus camelliae	WP_101354871	--------V-----------------		TSN---I------K--------
	Bacillus campisalis	WP_046525806	--------V-----------------		T-Q---V--QS--K--------
	Bacillus cavernae	WP_126867120	--------V--I--------------		T-N---V--QN--K--------
	Bacillus cellulasensis	KIL29018	--------S----------------A		TS----A---N--K--------
	Bacillus cihuensis	WP_028390314	--------V-----------A-----		T-N---V---K---------A
	Bacillus coagulans	WP_029142942	------K-I---------A-------		TSN---I------K--------
	Bacillus coahuilensis	WP_029326715	------K-V---------A-------		T-----A------K--------
	Bacillus dakarensis	WP_077211025	--------V---------A------I		T-N---V---K--K--------
	Bacillus deserti	WP_101639634	--------V-----------------		T-----V--QN-NK--------
	Bacillus dielmoensis	WP_042455008	--------V-----------------		T-Q---V---K--K--------
	Bacillus fastidiosus	WP_066229079	--------V----------------I		T-E---V---Q--K--------
	Bacillus foraminis	WP_121609841	--------V-----------------		T-H---V--QE--K--------
	Bacillus fordii	WP_033369792	--------V---------A-------		TS----I---E--K--------
	Bacillus fortis	WP_120072602	--------V---------A-------		TS----I---E--K--------
	Bacillus freudenreichii	WP_126433102	--------V---------A-------		T-----I------K--------
	Bacillus fumarioli	WP_066372540	--------V-----------------		T-N---I---Q--K----Y---
	Bacillus ginsengihumi	WP_025727974	--------I---------A-------		TSS---I------K--------
	Bacillus gobiensis	WP_053602144	--------T-----------------		TSN---A---N--K--------
	Bacillus gottheilii	WP_066449157	--------V----------------I		T-Q---V---Q--K--------
	Bacillus halmapalus	WP_078381738	--------V---------N-----Y-		T-----L--DE--K--------
	Bacillus halotolerans	WP_024120666	--------S---------A-------		TS----A---S--K--------
	Bacillus halotolerans	WP_059336068	--------S---------A-------		TS----A---S--K--------
	Bacillus horikoshii	WP_064099219	--------V---------N-----Y-		T-----L--D---K--------
	Bacillus indicus	WP_029285997	--------V---------A-------		T-E---V---K--K--------
	Bacillus kochii	WP_095369991	--------V-----------------		T-Q---V---G--K--------
	Bacillus korlensis	WP_066060055	--------V----------------I		T-H---V---K--K--------
	Bacillus kribbensis	WP_026693444	--------V-----------------		T-E---V------K--------
	Bacillus kwashiorkori	WP_062353229	--------V----------------T		TS----A---S--K--------
	Bacillus lentus	WP_066145440	--------V---------A-------		TS----I------K--------
	Bacillus litoralis	WP_066336074	--------V---------A-------		TS----C---N--K--------
	Bacillus loiseleuriae	WP_049680208	--------V-----------A-----		T-N---V---K---------A
	Bacillus massilioanorexius	WP_026089313	-------------------------T		TS----V--VK-----------
	Bacillus mediterraneensis	WP_071458769	--------V-----------------		T-E---V------K--------
	Bacillus methanolicus	WP_034644757	--------V---------A-------		T-H---V---S--K--------
	Bacillus ndiopicus	WP_042478985	--------V---------AA-----A		TG--------T--K--------
	Bacillus niameyensis	WP_062105440	--------V---------A-------		TS----I---N--K--------
	Bacillus oceanisediminis	WP_110066613	--------V---------A-------		T-H---V---S--K--------
	Bacillus pumilus	OLP64194	--------S----------------A		TS----A---N--K--------
	Bacillus salacetis	RIW32345	--------V---------A-----Y-		T-----V--DE--K--------
	Bacillus salacetis	WP_136035709	--------V---------A-----Y-		T-----V--DE--K--------
	Bacillus sinesaloumensis	WP_077620886	--------V------P---------I		T-Q---V---E--K--------
	Bacillus smithii	WP_040341209	--------V-----------------		T-----V---E--K--------
	Bacillus sp. 3-2-2	WP_126051952	--------V---------A-------		T-----I------K--------
	Bacillus sp. B14905	EAZ87737	--------V---------A-------		TN--------C--K--------
	Bacillus sp. CGMCC 1.16541	WP_110114322	--------V-I-------------Y-		T-----V---N--K--------
	Bacillus sp. CHD6a	WP_060666521	--------V---------N-----Y-		T-----V--DE--K--------
	Bacillus sp. DSL-17	WP_136356078	--------V-----------------		TS----C---K--K--------
	Bacillus sp. FJAT-22090	WP_082355335	-------------------------I		TN-------NS--K--------
	Bacillus sp. FJAT-27231	KMY53116	--------V---------A-------		T-----V---E--K--------
	Bacillus sp. FJAT-27986	WP_066107879	------K-V-----------------		SS----I---K--K--------
	Bacillus sp. FJAT-29814	WP_066305267	--------V-----------------		TSH---V---E--K--------
	Bacillus sp. FJAT-42315	WP_100403398	--------V---------A------I		T-----A------K--------
	Bacillus sp. FJAT-42376	WP_123914230	------K-V-----------------		T-E---V---T--K--------
	Bacillus sp. FJAT-46582	WP_100330447	--------V---------A-------		T-----I---E--K--------
	Bacillus sp. HMF5848	WP_125905166	--------V---------A-------		T-E---V---E--K--------
	Bacillus sp. HNG	WP_116354024	--------V------P---------I		T-Q---V------K--------
	Bacillus sp. KQ-3	WP_122901350	------K-----R-----------SN		T-----V------K--------
	Bacillus sp. P14.5	WP_113930459	--------V---------A-----Y-		T-----V--DE--K--------

Other Bacteria (0/>100)

Bacillus sp. PK3_68	WP_120036390	--------V----------A-------	T-----V---E--K--------
Bacillus sp. RRD69	SIT87017	--------S-----------------A	TS----A---N--K--------
Bacillus sp. SJS	WP_035406029	------K-V------------------	T-E---V---T--K--------
Bacillus sp. UMB0893	WP_101568743	--------V----------A-------	T-E---V---K--K--------
Bacillus sp. UNC125MFCrub1.1	WP_035391512	--------S-----------------A	TS----A---N--K--------
Bacillus sp. V44-8	WP_117323143	--------V------------------	T-N---V--QN--K--------
Bacillus sp. V47-23a	WP_117327170	--------V----------A-------	T-Q---V---K--K--------
Bacillus sp. V5-8f	WP_101634720	--------V------------------	T-N---V--QN--K--------
Bacillus sp. V59.32b	WP_117304683	--------V------------------	T-N---V--QN--K--------
Bacillus sp. X1(2014)	WP_038539977	--------V----------A-------	TSH---V--Q---K--------
Bacillus sp. YR335	WP_111617902	--------V------------------	TS----C---T--K--------
Bacillus sp. es.034	WP_098441245	--------V------------------	T-----V--DG--K--------
Bacillus sporothermodurans	WP_066226474	--------V----------A-------	T-S---V---N--K--------
Bacillus stratosphericus LAMA	EMI14967	--------S-----------------A	TS----A---N--K--------
Bacillus subtilis	WP_124073275	--------S----------A-------	TS----A---N--K--------
Bacillus terrae	WP_120117793	--------V----------A-------	TS----I---E--K--------
Bacillus timonensis	WP_136379446	--------V------P----------I	T-Q---V------K--------
Bacillus tuaregi	WP_071396094	--------V------------------	T-Q---V---S--K--------
Bacillus velezensis	WP_130571780	-------------------A-------	TS----A---S--K--------
Bacillus vietnamensis	WP_061810831	--------V----------A-------	T-----V--DG--K--------
Bacillus xiamenensis	WP_008361043	--------S-----------------A	TS----A---N--K--------
Bacillus zeae	WP_119113225	--------V----------A-------	T-E---V------K--------
Bhargavaea beijingensis	WP_092095850	---------------P-----------	TH----I--S---K--------
Bhargavaea cecembensis	WP_069201933	---------------P-----------	TN----I--S---K--------
Caldibacillus debilis	WP_026499599	--------------------------A	TS----A---K--K-------S
Chryseomicrobium excrementi	WP_100354535	--------S------------------	TN-------TG--K--------
Domibacillus antri	WP_075399587	--------V----------T-------	T-E---V---S--K--------
Domibacillus enclensis	WP_045852481	--------V----------T-------	T-E---V---S--K--------
Domibacillus epiphyticus	WP_076764292	--------V----------T-------	T-E---V---S--K--------
Domibacillus indicus	WP_046176705	--------V----------T-------	T-----V---S--K--------
Domibacillus iocasae	WP_069937942	--------V----------T-------	T-E---V---T--K--------
Domibacillus robiginosus	WP_050181705	--------V----------T-------	T-----V------K--------
Domibacillus tundrae	WP_046181335	--------V----------T-------	T-E---V---T--K--------
Edaphobacillus lindanitolerans	WP_076760069	---------------P-----------	TN----I--N---K--------
Falsibacillus pallidus	WP_114747237	--------V------------------	T-----V---S--K--------
Falsibacillus sp. GY 10110	WP_121682244	--------V------------------	T-----V---S--K--------
Jeotgalibacillus alimentarius	WP_041122036	--------V----------A-------	T-----A------K--------
Jeotgalibacillus campisalis	WP_041058725	--------V------------------	T-----A--Q---K--------
Jeotgalibacillus malaysiensis	WP_039808143	--------V-----------------I	T-----A------K--------
Jeotgalibacillus proteolyticus	WP_135122145	--------V------------------	T-----A--Q---K--------
Jeotgalibacillus salarius	WP_134382435	--------V------------------	T-----A------K--------
Jeotgalibacillus soli	WP_041088104	--------V------------------	T-----A--QN--K--------
Jeotgalibacillus sp. R-1-5s-1	WP_134373321	--------V------------------	T-----A---E--K--------
Jeotgalibacillus sp. S-D1	WP_133378250	--------V------------------	T-----A--QE--K--------
Kurthia huakuii	WP_035944995	-------------------NA-----I	TSK---V---S--K--------
Lysinibacillus boronitolerans	WP_016994016	--------V----------A-------	TN--------C--K--------
Lysinibacillus chungkukjangi	WP_107937143	--------S----------AA------	TN--------S--K--------
Lysinibacillus composti	WP_124767037	--------------------A------	TN--------C--K-------A
Lysinibacillus contaminans	WP_082332517	--------V----------A-------	TN--------C--K--------
Lysinibacillus fusiformis	WP_025115856	--------V----------A-------	TN--------C--K--------
Lysinibacillus halotolerans	WP_122971965	-------------------AA------	TN--------C--K--------
Lysinibacillus macroides	WP_083448556	--------V----------A-------	TN--------C--K--------
Lysinibacillus manganicus	WP_036190318	-------------------AA------	TN-------QC--K--------
Lysinibacillus massiliensis	WP_036177959	-------------------TA------	TN-------QC--K--------
Lysinibacillus meyeri	WP_107840153	--------V----------AA-----A	TG--------T--K--------
Lysinibacillus parviboronicapiens	WP_107924650	--------V----------A-------	TN--------C--K--------
Lysinibacillus sinduriensis	WP_036197718	--------S----------AA-----I	TN--------S--K--------
Lysinibacillus sp. B2A1	AVK86162	--------V----------A-------	TN--------C--K--------
Lysinibacillus sp. Marseille-P	WP_106781114	-------------------AA------	TN-------QC--K--------
Lysinibacillus sp. OL1	WP_131521920	--------V----------A-------	TN--------C--K--------
Lysinibacillus sp. SGAir0095	QCR33739	--------S----------AA------	TN--------S--K--------
Lysinibacillus sp. SYSU K30002	WP_126659388	-------------------AA------	TN-------QS--K--------
Lysinibacillus sp. YLB-03	WP_118877055	-------------------AA------	TN--------S--K--------
Lysinibacillus sp. ZYM-1	WP_082389536	--------V----------A-------	TN--------C--K--------
Lysinibacillus sphaericus	WP_010857265	--------V----------A-------	TN--------C--K--------
Lysinibacillus telephonicus	WP_126295940	-------------------AA------	TN-------QC--K--------
Lysinibacillus varians	AHN23294	--------V----------A-------	TN--------C--K--------
Paenisporosarcina indica	WP_075618978	---------------------------	TN----I--DL--K--------
Paenisporosarcina quisquiliarum	SEN48815	---------------------------	TN-------DT--K--------
Paenisporosarcina sp. HGH0030	WP_036660126	---------------------------	TN----I--DN--K--------
Paenisporosarcina sp. K2R23-3	WP_119883934	---------------------------	TN----I--DS--K--------
Paenisporosarcina sp. OV554	WP_108587824	--------S------------------	TN----I--DS--K--------
Parageobacillus thermantarctic	WP_090952383	------K-V----------A-------	TS----V---Q--K--------
Parageobacillus thermoglucosid	WP_064550614	------K-V----------A-------	TS----V---Q--K--------

**Other Bacteria (0/>100)**	Planococcus antarcticus	WP_006830854	-------K-------------------	TH----I--N---K--------
	Planococcus donghaensis	WP_008432144	-------K-------------------	TH----I--N---K--------
	Planococcus halocryophilus	WP_008497122	-------K-------------------	TH----I--N---K--------
	Planococcus massiliensis	WP_052652364	-------K-------------------	TN-------N---K--------
	Planococcus salinarum	OHX50324	------KK-------------------	TH-------NE--K--------
	Planococcus salinus	WP_123166648	-------K-------------------	TH-------N---K--------
	Planococcus sp. PAMC 21323	WP_038703257	-------K-------------------	TH----I--N---K--------
	Planococcus sp. Y42	WP_077588393	---------------------------	TH----I--N---K--------
	Planococcus versutus	WP_049694230	-------K-------------------	TN----I--N---K--------
	Planomicrobium flavidum	WP_088008156	--------------------------I	TH----I--N---K--------
	Planomicrobium glaciei	WP_036805768	-------K-------------------	TH----I--N---K--------
	Planomicrobium koreense	WP_135505116	------KS-------------------	TN----V--N---K--------
	Planomicrobium soli	WP_106534795	-------K-------------------	TH-------S---K--------
	Psychrobacillus sp. FJAT-21963	WP_082461119	--------------------------I	TN-------NS--K--------
	Psychrobacillus sp. OK028	SDO12870	---------------------------	TN-------DA--K--------
	Psychrobacillus sp. OK032	WP_093276259	--------------------------I	TN-------NT--K--------
	Quasibacillus thermotolerans	WP_039238758	--------V----------A-------	T-----V------K--------
	Solibacillus isronensis	WP_079523609	--------V----------AA------	TG-------DG--K--------
	Sporosarcina sp. EUR3 2.2.2	WP_081697108	--------S------------------	TN----I--DS--K--------
	Tetzosporium hominis	WP_094944795	--------S------------------	TN-------TG--K--------
	Thermolongibacillus altinsuens	WP_132949156	------K-V------------------	TS----I---Q--K--------
	Ureibacillus thermophilus	QBK26951	-------------------AA------	TN--------S--K-T-----A
	Virgibacillus soli	KRG10553	-------------------A-------	TS----A---H--K--------

Supplemental Figure 88

A partial sequence alignment of the peroxide-responsive transcriptional repressor PerR protein containing a two amino acid insertion (boxed) that is exclusively shared by all members belonging to the genus *Caryophanon* and absent in all other bacteria.

			76		131
Caryophanon (2/2)	Caryophanon tenue	WP_066543353	LLGKVLVTHQTGPEGKEVKRLYIEEGC	P	NIEKEYYISLVVDRATSRVTVMGSAEGG
	Caryophanon latum	WP_066463390	--------------------I------	-	--A-------------------------
Other Bacteria (0/>100)	Abyssicoccus albus	WP_123807393	---------------------------		D-QN---L-F------D--VL---E---
	Anoxybacillus amylolyticus	WP_066323757	---------------------L-----		D-K-----G----------VL---E---
	Anoxybacillus ayderensis	WP_021094224	---------------------L-----		D-K-----G----------VL---E---
	Anoxybacillus flavithermus	WP_003394931	---------------------L-----		D-K-----G----------VL---E---
	Anoxybacillus sp. BCO1	WP_042894132	---------------------L-----		D-K-----G----------VL---E---
	Anoxybacillus sp. UARK-01	WP_080860835	---------------------L-----		D-K-----G----------VL---E---
	Anoxybacillus tepidamans	WP_027408670	---------------------L-----		D-K-----G--L-------VL---E---
	Anoxybacillus vitaminiphilus	WP_111643706	---------------------L-----		D-K-----G----------VL---E---
	Auricoccus indicus	WP_077140396	---------------------------		D-QN---L-F------D--VL---E---
	Bacillus alveayuensis	WP_044893784	---------------------L-----		D-K-----G----------VL---E---
	Bacillus beveridgei	WP_069365209	I--------------------W-----		D-QN---VGI--------I-M-A-E---
	Bacillus cecembensis	WP_057987069	----I------------I--------S		D-R----L---L-----C--M---E---
	Bacillus gottheilii	WP_080846462	I---T----------------L-----		D-Q-----G--L--------L-A-E---
	Bacillus ndiopicus	WP_042471886	----I---------------------S		D-R----LG--L--------L---E---
	Bacillus thermoamylovorans	WP_034769750	----T----------------L--K--		D-K-----GI-L--------L-A-S---
	Bhargavaea beijingensis	WP_092096485	--------------------------S		D-L-------------D--VL---E---
	Bhargavaea cecembensis	WP_008297210	--------------------------S		D-L-------------D--VL---E---
	Bhargavaea ginsengi	WP_092048970	--------------------------S		D-L-------------D--VL---E---
	Chryseomicrobium excrementi	WP_100352734	--------------------I-----S		D-Q-----G--L--------L---E---
	Cohnella kolymensis	WP_041064624	---------------------L-----		D-K-----GV----G-G--VM---E---
	Cohnella laeviribosi	WP_019003743	---------------------L-----		D-K-----G-----S-G-IV----E---
	Cohnella luojiensis	WP_135152976	---------------------L-----		D-K-----GV----G-G--VM---E---
	Cohnella lupini	WP_115990966	-----------------I---L-----		D-K-----GV----G-G--VM---E---
	Cohnella panacarvi	WP_027084288	---------------------L-----		D-K-----GV------G--VM---E---
	Cohnella sp. HS21	WP_130611803	---------------------L-----		D-K-----GV----G-G--VM---E---
	Cohnella sp. M2MS4P-1	WP_120976201	-----------------I---L-----		D-K-----GV----G-G--VM---E---
	Edaphobacillus lindanitolerans	WP_076756523	--------------------------S		D-L-----------S-D--VL---E---
	Fictibacillus aquaticus	WP_094250829	I--------------------L-----		D-K-----G--L-------VM---E---
	Geobacillus icigianus	WP_033022978	---------------------L-----		D-Q-----G----------VL---E---
	Geobacillus sp. WSUCF1	EPR26506	---------------------L-----		D-Q-----G----------VL---E---
	Geobacillus stearothermophilus	WP_095859150	---------------------L-----		D-Q-----G----------VL---E---
	Geobacillus subterraneus	WP_033843507	---------------------L-----		D-Q-----G----------VL---E---
	Geobacillus thermoleovorans	WP_014195443	---------------------L-----		D-Q-----G----------VL---E---
	Geobacillus vulcani	WP_031410443	---------------------L-----		D-Q-----G----------VL---E---
	Kurthia gibsonii	WP_121178138	-------------Q--------V---S		D-K----L------Q-----L---E---
	Kurthia huakuii	WP_029498222	-----------------I--------S		D-K----V--L---------L---E---
	Kurthia massiliensis	WP_010286444	-I------------------------S		D-K----V--L---------L---E---
	Kurthia senegalensis	WP_010301575	-----------------I--------S		-------V--L---------L---E---
	Kurthia sibirica	WP_109304505	----I---------------I-----S		--K----L-F----Q-----L---E---
	Kurthia sp. 11kri321	WP_068454917	-------------Q--------V---S		D-K----L------Q-----L---E---
	Kurthia zopfii	WP_109349225	-----------------I----V-Q-S		D-K----L--L---D-----L---E---
	Lysinibacillus acetophenoni	WP_097147994	----I---------------------S		D-K----L-F-L--------L---E---
	Lysinibacillus chungkukjangi	WP_107933981	----I---------------------S		D-K----L-F-L--------L---E---
	Lysinibacillus composti	WP_124764024	----I---------------------S		D-R----L---L--------L---E---
	Lysinibacillus contaminans	WP_053584355	I---T-----------V---------S		D-Q----L---L------------E---
	Lysinibacillus endophyticus	WP_121212964	----I---------------------S		D------L-FIL--------L---E---
	Lysinibacillus fusiformis	WP_004229281	----I---------------------S		D-Q----L---L------------E---
	Lysinibacillus halotolerans	WP_122970343	----I---------------------S		D-K----L-F-L--------L---E---
	Lysinibacillus jejuensis	WP_108305782	----I-------------------Q-S		D-K----L------------L---E---
	Lysinibacillus macroides	WP_053993908	----I---------------------S		D-Q----L---L--------M---E---
	Lysinibacillus manganicus	WP_036181609	----I---------------------S		D-K----L-F-L-------VL---E---
	Lysinibacillus mangiferihumi	WP_107894696	----I---------------------S		D-Q----L---L--------F---E---
	Lysinibacillus massiliensis	WP_036171688	----I---------------------S		D-K----L-F-L--------L---E---
	Lysinibacillus meyeri	WP_107837896	----I---------------------S		D-R----LG--L--------L---E---
	Lysinibacillus odysseyi	WP_036151133	----I---------------------S		D-Q----L-F-L--------L---E---
	Lysinibacillus parviboronicapiens	WP_107923089	----I---------------------S		D-Q----L---L------------E---
	Lysinibacillus sinduriensis	WP_036202940	----I---------------------S		D-S----L-F-L--------L---E---
	Lysinibacillus sp. B2A1	AVK83156	----I---------------------S		D-Q----L---L--------M---E---
	Lysinibacillus sp. BF-4	WP_036141348	----I-------------------Q-S		D-Q----L------------L---E---
	Lysinibacillus sp. BK089	TCJ73581	----I---------------------S		D-Q----L---L--------M---E---
	Lysinibacillus sp. FJAT-14222	WP_053594194	----I---------------------S		D-Q----L---L--------M---E---
	Lysinibacillus sp. FJAT-14745	WP_053483313	----I------------I--------S		D-Q----L---L--------M---E---
	Lysinibacillus sp. Marseille-P	WP_106784170	----I---------------------S		D-K----L-F-L--------L---E---
	Lysinibacillus sp. SGAir0095	QCR31784	----I---------------------S		D-K----L-F-L--------L---E---
	Lysinibacillus sp. SYSU K30002	WP_126657567	----I---------------------S		D-K----L-F-L--------L---E---
	Lysinibacillus sp. YLB-03	WP_118874674	----I---------------------S		D-K----L-FI---------L---E---
	Lysinibacillus sp. YR326	WP_134018974	-I--I---------------------S		D-Q----L---L--------M---E---
	Lysinibacillus sp. ZYM-1	WP_054610301	----I---------------------S		D-Q----L---L------------E---
	Lysinibacillus sphaericus	WP_010858428	--------------------------S		D-Q----L---L------------E---
	Lysinibacillus telephonicus	WP_126292946	----I---------------------S		D-K----L-F-L--S-----L---E---
	Lysinibacillus xylanilyticus	WP_100543951	-I--I---------------------S		D-Q----L---L--------M---E---
	Lysinibacillus xyleni	WP_097072122	----I---------------------S		D------L-F-L--------L---E---

Other Bacteria (0/>100)

Marininema halotolerans	WP_091835246	-I--T----------------L-----	P-D----LGV-I----N---L-A-S---
Marininema mesophilum	WP_091737121	----T----------------L-----	P-D----LGV-I----N---L-A-S---
Paenibacillus assamensis	WP_028595590	I--------------------L--Q--	D-K-----GI------G--VM---E---
Paenisporosarcina indica	WP_075617537	-----------------I---LV----	D-K-----G--L--------L---E---
Paenisporosarcina sp. HGH0030	WP_016427698	-----------------I---LV----	D-K----LG--L--------L---E---
Paenisporosarcina sp. K2R23-3	WP_119882967	--------------------------S	D-Q----LG--L---S----L---E---
Paenisporosarcina sp. OV554	WP_108584626	-----------------I---LV----	D-K----LG--L----A---L---E---
Paraburkholderia tropica	RQN32920	----Q----------------------	D-Q----VGF-I----DK--L-A-E---
Parageobacillus thermantarctic	WP_090948040	---------------------L-----	D-K-----GF---------VL---E---
Parageobacillus thermoglucosid	WP_064551049	---------------------L-----	D-K-----G----------VL---E---
Planococcus maitriensis	WP_112231464	------------------------A-S	-------LG--L--E-----L---E---
Planococcus massiliensis	WP_052651234	------------------------A-S	D-Q----LG--L--E-----L---E---
Planococcus sp. CAU13	WP_033543926	-I----------------------A-S	-------LG--L--E-----L---E---
Planococcus sp. Y42	WP_077589639	--------------------------S	D-Q----LG--L--E-----L---E---
Planomicrobium flavidum	WP_088005911	------------------------A-S	D-Q----LG--L--E-----L---E---
Planomicrobium glaciei	WP_036806698	----------------------V-A-S	D-Q----LG--L--E-----L---E---
Planomicrobium koreense	WP_135503819	-----------------I------A-S	S------LG--L--E-----L---E---
Planomicrobium soli	WP_106531952	------------------------A-S	D-Q----LG--L--E-----L---E---
Pontibacillus halophilus	WP_026799761	---ST----------------L-----	D-Q-----G--L--------M---E---
Psychrobacillus insolitus	WP_111438551	---------------------L----S	D-K----VG--L--------L---E---
Psychrobacillus psychrotoleran	WP_093535180	-----------------I---L----S	D-K----VG--L--S-----L---E---
Psychrobacillus sp. OK028	WP_093060075	-----------------I---L----S	D-K----VG--L--S-----L---E---
Psychrobacillus sp. OK032	WP_093263286	-----------------I---L----S	D-K----VG--L--------L---E---
Rummeliibacillus pycnus	WP_102692679	------------------------A-S	D-K----L-FL-------------E---
Rummeliibacillus sp. POC4	WP_119414269	------------------------A-S	D-K----L-FL-------------E---
Rummeliibacillus sp. TYF005	WP_124217243	------------------------A-S	D-Q----L-FL------K------E---
Rummeliibacillus stabekisii	WP_066785376	------------------------A-S	D-Q----V--L---------L---EA--
Seinonella peptonophila	WP_073157350	---------------------L-----	P-------GV-I--S-N--VM---E---
Solibacillus isronensis	WP_079525179	----I--------D------------S	D-K----L---L-------VM---E---
Solibacillus sp. R5-41	WP_099422766	----I------------I--------S	D-R----L---L-----C--M---E---
Sporosarcina koreensis	WP_040286875	----T----------------L----A	D-Q-------------D---L---E---
Sporosarcina sp. BIO01-red	WP_116016312	----T----------------LV---A	D-K-------------D---L---E---
Sporosarcina sp. EUR3 2.2.2	WP_024535015	----T------------I---LV----	D-K----LG--L----A---L---E---
Staphylococcus agnetis	WP_107401391	--------------------------A	--Q----VGF-I----D---L-A-E---
Staphylococcus aureus	PKR14675	----T------------I---------	A-Q----VGF-I----DQ--L-A-E---
Staphylococcus cohnii	WP_107525978	----T------------I---------	D-Q----VGF-I----D---L-A-E---
Staphylococcus delphini	WP_019165484	--------------------------A	--Q----VGF-I----D---L-A-E---
Staphylococcus edaphicus	WP_099089404	----T------------I---------	D-Q----VGF-I----D---L-A-E---
Staphylococcus gallinarum	WP_119485862	----T------------I---------	D-Q----VGF-I----D---L-A-E---
Staphylococcus hominis	WP_071859448	----Q----------------------	D-Q----VGF-I----D-I-L-A-E---
Staphylococcus hyicus	WP_107633181	--------------------------A	--Q----VGF-I----D---L-A-E---
Staphylococcus kloosii	WP_061854845	----Q----------------------	D-Q----VGF-I----D-I-L-A-E---
Staphylococcus lentus	WP_017000097	---------------------L-----	D-Q-----GF-I----D-I-L-A-E---
Staphylococcus lutrae	WP_085238141	-------------Q------------A	--Q----VGF-I----D---L-A-E---
Staphylococcus microti	WP_044360972	--------------------------A	D-K-----GF-I----D---L-A-E---
Staphylococcus pettenkoferi	WP_049410664	----Q----------------------	D-Q----VGF-I----D---L-A-E---
Staphylococcus pseudintermediu	WP_063278813	--------------------------A	--Q----VGF-I----D---L-A-E---
Staphylococcus saprophyticus	WP_048794076	----Q----------------------	D-Q----VGF-I----D-I-L-A-E---
Staphylococcus sciuri	WP_119494720	---------------------L-----	D-Q-----GF-I----D-I-L-A-E---
Staphylococcus simulans	WP_107539904	----T--------Q-------------	D-Q----VGF-I----D---L-A-E---
Staphylococcus sp. AtDRG32	WP_133944731	----Q----------------------	D-Q----VGF-I----D-I-L-A-E---
Staphylococcus sp. HMSC057A02	WP_070647562	----Q----------------------	D-Q----VGF-I----D-I-L-A-E---
Staphylococcus sp. HMSC072E01	WP_070672539	----Q----------------------	D-Q----VGF-I----D-I-L-A-E---
Staphylococcus succinus	PTI75699	-I--T------------I---------	D-Q----VGF-I----DK--L-A-E---
Staphylococcus vitulinus	WP_103323025	-----------------I---L-----	D-Q-----GF-I----D-I-L-A-E---
Staphylococcus xylosus	WP_107544070	----T----------------------	D-Q----VGF-I----D---L-A-E---
Streptococcus pneumoniae	WP_061815414	----Q----------------------	D-Q----VGF-I----D-I-L-A-E---
Tetzosporium hominis	WP_094941646	--------------------I-----S	D-A-----G--L--------L---E---
Thermoflavimicrobium dichotomi	WP_093229357	----I----------------L-----	P-------GV-I--S-G--VM---E---
Thermoflavimicrobium sp. FBKL4	WP_113657995	---------------------L-----	P-D-----GV-I--S-E--VM---E---
Thermolongibacillus altinsuens	WP_132948126	----K----------------L-----	D-K-----G----------VL---E---
Ureibacillus thermophilus	QBK25617	----K---K----Q-------------	E-K----L-FIL--S---I-----S---
Ureibacillus thermosphaericus	WP_016837429	----I---Q----Q-------------	E-K------FIL--S---I---------
Viridibacillus arvi	WP_053417481	--------------------------S	D-K----LGF--------I-L---E---
Viridibacillus sp. OK051	WP_100796466	----I---------------------S	D-K----LGV----------F---E---

Supplemental Figure 89

A partial sequence alignment of the ADP-forming succinate--CoA ligase subunit beta protein containing a one amino acid insertion (boxed) that is exclusively shared by all members belonging to the genus *Caryophanon* and absent in all other bacteria.

			324		369
***Caryophanon* (2/2)**	Caryophanon tenue	WP_066542314	FSELHVFPFSPRTGTPAARMEDM	F	VDEDVKNERVHRLISLNDQLAK
	Caryophanon latum	WP_066460915	----------------------I	-	-A--------------------
**Other Bacteria (0/>100)**	Lysinibacillus boronitolerans	WP_016994110	----------------------Q		I---I-----------------
	Lysinibacillus fusiformis	WP_069482030	----------------------Q		I---I-----------------
	Lysinibacillus sp. PB300	WP_115674111	----------------------Q		I---I-----------------
	Lysinibacillus sp. ZYM-1	WP_054609311	----------------------Q		I---I-----------------
	Lysinibacillus macroides	WP_053996187	----------------------Q		I---I---------A-------
	Lysinibacillus parviboronicapiens	WP_107950301	----------------------Q		I---I--D------A-------
	Lysinibacillus sp. B2A1	AVK84302	----------------------Q		I---I---------A-------
	Lysinibacillus sp. BK089	TCJ74419	----------------------Q		I---I---------A-------
	Lysinibacillus sp. FJAT-14222	WP_053595262	----------------------Q		I--NI---------A-------
	Lysinibacillus sp. FJAT-14745	WP_053484671	----------------------Q		I---I--D------A-------
	Lysinibacillus sp. YR326	WP_134018454	----------------------Q		I---I---------A-------
	Lysinibacillus xylanilyticus	WP_049663752	----------------------Q		I--GI---------A-------
	Lysinibacillus sphaericus	WP_010861506	----------------------Q		I---I---------A--N----
	Lysinibacillus contaminans	WP_053582795	--------------------D-Q		I---I---------A-------
	Bacillus acanthi	WP_108669599	--------Y-K-----------Q		---EI---------A-S-----
	Bacillus aciditolerans	WP_121447253	--------Y-K-----------Q		I--E------------S-----
	Bacillus altitudinis	WP_098680699	--------Y-K-----------Q		---N----------A-S-----
	Bacillus australimaris	WP_060697386	--------Y-K-----------Q		---N----------A-S-----
	Bacillus bataviensis	WP_007087609	--------Y-K-----------Q		---E------------S-----
	Bacillus butanolivorans	WP_053347008	--------Y-K-----------Q		----------------S-----
	Bacillus campisalis	WP_046526297	--------Y-K-----------Q		I-------------A-S-----
	Bacillus canaveralius	WP_101575557	--------Y-K-----------Q		---E----------A-S-----
	Bacillus cellulasensis	WP_057079299	--------Y-K-----------Q		---N----------A-S-----
	Bacillus cereus	AUZ27143	--------Y-K-----------Q		---N----------A-S-----
	Bacillus cohnii	WP_066420975	--------Y-K-----------Q		I--E------------S-----
	Bacillus cucumis	WP_101651858	--------Y-K-----------Q		I--E------------S-----
	Bacillus dakarensis	WP_077214025	--------Y-K-----------Q		I-------------T-S-----
	Bacillus dielmoensis	WP_042458552	--------Y-K-----------Q		---E----------T-------
	Bacillus fastidiosus	WP_066225248	--------Y-K-----------Q		---E------------S-----
	Bacillus foraminis	WP_121611697	--------Y-K-----------Q		I-------------A-S-----
	Bacillus horneckiae	WP_066396076	--------Y-K-----------Q		---E----------T-S-----
	Bacillus humi	WP_057998713	--------Y-K-----------Q		---E------------S-----
	Bacillus intestinalis	WP_088679289	--------Y-K-----------Q		---N----------A-S-----
	Bacillus jeotgali	WP_102263640	--------Y-K-----------Q		--------------E-SN----
	Bacillus kyonggiensis	WP_136830857	--------Y-K-----------Q		--------------A-S-----
	Bacillus licheniformis	WP_075747378	--------Y-K-----------Q		---N----------A-S-----
	Bacillus massiliogabonensis	WP_102274513	--------Y-K-----------Q		----------------S-----
	Bacillus massiliogorillae	WP_042345811	--------Y-K-----------Q		-------D------A-S-----
	Bacillus niacini	WP_063251344	--------Y-K-----------Q		---E----------T-S-----
	Bacillus novalis	WP_066090228	--------Y-K-----------Q		---E------------S-----
	Bacillus onubensis	WP_099361964	--------Y-K-----------Q		I--E------------S-----
	Bacillus persicus	WP_090741831	--------Y-K-----------Q		-------D------A-S-----
	Bacillus praedii	WP_057764937	--------Y-K-----------Q		-E-E------------S-----
	Bacillus pumilus	WP_099681219	--------Y-K-----------Q		---H----------A-S-----
	Bacillus safensis	WP_024427954	--------Y-K-----------Q		-E-N----------A-S-----
	Bacillus salsus	WP_090855046	--------Y-K-----------Q		---E----------A-S-----
	Bacillus selenatarsenatis	WP_041966901	--------Y-K-----------Q		--------------E-SN----
	Bacillus sinesaloumensis	WP_077618858	--------Y-K-----------Q		I--E------------S-----
	Bacillus soli	WP_066064077	--------Y-K-----------Q		---E------------S-----
	Bacillus sp. 1NLA3E	WP_015595028	--------Y-K-----------Q		---E---D--------S-----
	Bacillus sp. 7504-2	WP_095306871	--------Y-K-----------Q		--------------A-S-----
	Bacillus sp. 7788	WP_095285403	--------Y-K-----------Q		---N----------A-S-----
	Bacillus sp. AFS006103	WP_098259579	--------Y-K-----------Q		---E---D--------S-----
	Bacillus sp. AFS031507	WP_098931377	--------Y-K-----------Q		---E------------S-----
	Bacillus sp. B-jedd	WP_048825602	--------Y-K-----------Q		---E----------A-S-----
	Bacillus sp. BA3	WP_101222608	--------Y-K-----------Q		--------------T-S-----
	Bacillus sp. BK450	TDU14642	--------Y-K-----------Q		---N----------A-S-----
	Bacillus sp. EB01	WP_043933926	--------Y-K-----------Q		--------------A-S-----
	Bacillus sp. F56	WP_069839512	--------Y-K-----------Q		---N----------A-S-----
	Bacillus sp. FJAT-18017	WP_053598901	--------Y-K-----------Q		-----------K--A-S-----
	Bacillus sp. FJAT-27225	WP_066196279	--------Y-K-----------Q		----I-----------S-----
	Bacillus sp. FJAT-27245	WP_053367186	--------Y-K-----------Q		---E----------A-S-----
	Bacillus sp. FJAT-27445	WP_059171183	--------Y-K-----------Q		---E----------A-S-----
	Bacillus sp. FJAT-29937	WP_066293290	--------Y-K-----------Q		I--E------------S-----
	Bacillus sp. FJAT-45066	WP_096153339	--------Y-K-----------Q		---E----------A-S-----
	Bacillus sp. FJAT-45122	WP_100333402	--------Y-K-----------Q		---E----------A-S-----
	Bacillus sp. FJAT-45350	WP_096201316	--------Y-K-----------Q		--------------E-SN----
	Bacillus sp. HNG	WP_116351529	--------Y-K-----------Q		I--E------------S-----
	Bacillus sp. I-2	WP_076840450	--------Y-K-----------Q		---H----------A-S-----
	Bacillus sp. LF1	WP_090637614	--------Y-K-----------Q		I--E------------S-----
	Bacillus sp. LLTC93	WP_105925905	--------Y-K-----------Q		---H----------A-S-----
	Bacillus sp. M 2-6	WP_008342461	--------Y-K-----------Q		---N----------A-S-----

Other Bacteria (0/>100)

Bacillus sp. M6-12	WP_101597014	--------Y-K-----------Q	I--E---D--------S-----
Bacillus sp. YLB-04	WP_115453490	--------Y-K-----------Q	---E------------S-----
Bacillus sporothermodurans	WP_066226016	--------Y-K-----------Q	I--N------------S-----
Bacillus stratosphericus	WP_039964036	--------Y-K-----------Q	---N----------A-S-----
Bacillus subtilis	WP_090558118	--------Y-K-----------Q	---N----------A-S-----
Bacillus tequilensis	WP_024715004	--------Y-K-----------Q	---N----------A-S-----
Bacillus timonensis	WP_010678177	--------Y-K-----------Q	I--E------------S-----
Bacillus timonensis	WP_136377731	--------Y-K-----------Q	I--E------------S-----
Bacillus tuaregi	WP_071395817	--------Y-K-----------Q	----------------S-----
Bacillus vireti	WP_024030996	--------Y-K-----------Q	-N-E------------S-----
Bacillus xiamenensis	WP_008356240	--------Y-K-----------Q	---N----------A-S-----
Bacillus zeae	WP_119113573	--------Y-K-----------Q	I------D------A-S-----
Bacillus zhangzhouensis	WP_034319471	--------Y-K-----------Q	---H----------A-S-----
Lysinibacillus meyeri	WP_107839456	--------Y-K-----------Q	---EI---------G-------
Lysinibacillus odysseyi	WP_052125034	----------K-----------Q	---EI---------A-------
Massilibacterium senegalense	WP_062198707	--------Y-K-----------Q	I--E------------S-----
Planococcus salinus	WP_123163762	--------Y-Q-----------Q	---E------------------
Planococcus sp. Y42	WP_077588844	--------Y-K-----------Q	---E----------G-------
Planomicrobium flavidum	WP_088008022	----------K-----------Q	---E----------E-------
Planomicrobium glaciei	WP_036809485	--------Y-Q-----------Q	I--NI---------A-------
Solibacillus isronensis	WP_079525584	--------Y-Q-----------Q	I--EI---------A-------
Solibacillus silvestris	WP_014824426	--------Y-Q-----------Q	---EI---------A-------
Solibacillus sp. R5-41	WP_099422888	----------Q-----------Q	---E----------A-------
Sporosarcina koreensis	WP_040286651	--------Y-K-----------Q	---N----------E-------
Sporosarcina psychrophila	WP_067208362	--------Y-K-----------Q	-P-E----------E-------
Sporosarcina sp. D27	WP_025784796	--------Y-K-----------Q	--------------E-------
Sporosarcina sp. P17b	WP_099625679	--------Y-K-----------Q	---N----------T-------
Sporosarcina sp. P19	WP_099690516	--------Y-K-----------Q	---N---------LT-------
Sporosarcina sp. P2	WP_099630224	--------Y-K-----------Q	---S---------LT-------
Sporosarcina sp. P33	WP_081244672	--------Y-K-----------Q	---N---------LT-------
Sporosarcina sp. P34	WP_099694393	--------Y-K-----------Q	---N---------LT-------
Sporosarcina ureae	WP_029054678	--------Y-K-----------Q	---N---------LT-------
Streptococcus pneumoniae	CVM18812	--------Y-K-----------Q	---N----------A-S-----
Bacillus sp. VT-16-64	WP_077110639	--------Y-M---------K-Q	IA-S----------A-S-----
Chryseomicrobium excrementi	WP_100352413	--------Y-K---------D-Q	---E----------T-------
Domibacillus enclensis	WP_045851021	--------Y-K---------D-Q	-----------K--A-S-----
Bacillus sp. Y1	WP_119709181	--------Y-K---------D-Q	----I-----------S-----
Bacillus sp. FJAT-42376	WP_123917944	--------Y-K---------D-Q	--------------E-S-----
Bacillus sp. M5HDSG1-1	WP_127737405	--------Y-K---------D-Q	I-------------A-S-----
Bacillus funiculus	WP_129727315	--------Y-K---------D-Q	-A---------K--A-S-----
Bacillus gottheilii	WP_080844963	--------Y-K---------D-Q	I---I---------A-S-----
Bacillus massiliosenegalensis	WP_019154567	--------Y-K---------D-Q	--------------A-S-----
Tetzosporium hominis	WP_094942098	--------Y-K---------D-Q	---E----------T-------
Bacillus circulans	WP_095258985	--------Y-K---------DNQ	IE--------------S-----
Bacillus nealsonii	WP_016202964	--------Y-K---------DNQ	I---------------S-----
Fictibacillus gelatini	WP_026676355	--------Y-K----------NQ	-P------------A-S-----
Lysinibacillus chungkukjangi	WP_107935398	-A------Y-K-----------Q	--------------T-------
Bacillus cecembensis	WP_057989634	-A------Y-Q-----------Q	---E----------T-------
Butyricicoccus sp. 1XD8-22	RKJ60490	-A--------K-----------Q	---E----------T-------
Kurthia massiliensis	WP_044504402	-A--------K-----------Q	-------------L--------
Kurthia zopfii	WP_109348358	-A--------K-----------Q	-------------LT-------
Lysinibacillus acetophenoni	WP_097148321	-A--------K-----------Q	---E---------LT-------
Lysinibacillus endophyticus	WP_121213330	-A--------K-----------Q	I------------LT-------
Lysinibacillus halotolerans	WP_122971352	-A--------K-----------Q	I--E---------LT-------
Lysinibacillus manganicus	WP_036186293	-A--------K-----------Q	I--E----------T-------
Lysinibacillus massiliensis	WP_036176027	-A--------K-----------Q	---E----------T-------
Lysinibacillus composti	WP_124762134	-A--------K-----------Q	I--EI--------LT-------
Lysinibacillus sp. Marseille-P	WP_106780012	-A--------K-----------Q	------T-------T-------
Lysinibacillus sp. SYSU K30002	WP_126657169	-A--------K-----------Q	-------------LT-------
Lysinibacillus sp. YLB-03	WP_118875614	-A--------K-----------Q	I--N---------LA-------
Lysinibacillus telephonicus	WP_126292757	-A--------K-----------Q	---E---------LT-------
Lysinibacillus xyleni	WP_097074528	-A--------K-----------Q	----I--------LT-------
Rummeliibacillus pycnus	WP_102691282	-A--------K-----------Q	---E---------LT-------

Supplemental Figure 90

A partial sequence alignment of the tRNA (N(6)-L-threonylcarbamoyladenosine(37)-C(2))-methylthiotransferase MtaB protein containing a one amino acid insertion (boxed) that is exclusively shared by all members belonging to the genus *Caryophanon* and absent in all other bacteria.

Group	Organism	Accession	325		374
Caryophanon **(2/2)**	Caryophanon latum	WP_066461515	FIPLISGTSGNSGTQALAVAVRGLATKDASN	D	ESKMRLLLRELSTGAIMG
	Caryophanon tenue	WP_066543012	---------------------------E-GD	E	---LK---------MV--
**Other Bacteria (0/>100)**	Bacillus abyssalis	WP_078408689	-----A-MA--T--------------G-VE-		---WK-II--AG--L-T-
	Bacillus acanthi	WP_108670025	-----A-MA--T-----------I--G-LEQ		---WK-I---AG--V-T-
	Bacillus alkalitelluris	WP_078548042	-----A-MA--T-----------I--G-HEK		-T-KK-I---AG--L-T-
	Bacillus aquimaris	WP_113970021	-----A-MA--T-----------I--G-LEK		-N--S-VI--AG--L-T-
	Bacillus aryabhattai	WP_088567726	-----G-MA--T--------------GEIEE		---FK--I--AA--M-T-
	Bacillus cecembensis	WP_057984247	-----------------------I--G-IGG		K--FK-V----G--V---
	Bacillus cihuensis	WP_028393123	-----G-MA--T-----------I--G-LEH		---WK-II--AG--L-T-
	Bacillus coahuilensis	WP_082686248	-----A-MA--T-----------I--GEIQ-		K--RT-----AG--L-T-
	Bacillus dakarensis	WP_077211206	-----A-MA--------------I--G-VEK		---SK-I---AG--L-T-
	Bacillus koreensis	WP_053399485	-----A-M---T--------------G-IER		-GIWKMIM---G--L-V-
	Bacillus korlensis	WP_066054891	-----A-MA--T--------------G-LEK		-E-SK-IV--AG--V-T-
	Bacillus loiseleuriae	WP_049680440	-----G-MA--T-----------I--G-LEH		---WK-V---AG--L-T-
	Bacillus marinisedimentorum	WP_070119725	-----A-MA--------------I--G-FEE		--RW--IR--AG--V-T-
	Bacillus massiliogorillae	WP_042349470	-----A-MA--------------I--G-LEE		---WK-II--AG--L-T-
	Bacillus megaterium	WP_043980456	-----G-MA--T--------------GEIEE		---FK--M--AA--M-T-
	Bacillus muralis	WP_064466567	-----G-MA--T-----------I--G-LEQ		---WK-V---AG--L-I-
	Bacillus ndiopicus	WP_042471259	-----------------------I--GEVDE		Q--LK--IK-CI--LLT-
	Bacillus oceanisediminis	WP_110067609	-----A-MA--T-----------I--G-LEK		-N--K-II--AG--L-T-
	Bacillus psychrosaccharolyticu	WP_040373273	-----G-MA--T-----------I--G-LE-		---WK-II--AG--L-T-
	Bacillus salacetis	WP_119549702	-----A-MA--T-----------I--G-LEE		---WK-VI--AG--L-T-
	Bacillus salsus	WP_090851098	-----A-MA--T-----------I--G-VDK		--RAK-I---AG--V-T-
	Bacillus simplex	WP_137017266	-----G-MA--T-----------I--G-LEQ		D--WK-V---AG--L-T-
	Bacillus solimangrovi	WP_069718337	-----A-MA--T----------NI--G-TEH		----K-IA--AG--I-T-
	Bacillus sp. AFS015802	WP_098353047	-----A-MA--T-----------I--G-LEK		-N--S-VI--AG--L-T-
	Bacillus sp. B14905	WP_008174266	--------------------I--I--G-VEE		H--LK-----AG--LMS-
	Bacillus sp. BA3	WP_101224434	-----G-MA--T-----------I--G-LEQ		D--WK-V---AG--L-T-
	Bacillus sp. CGMCC 1.16541	WP_110113514	-----A-MA--T--------------GEVAE		---WK-IV--AG--F-T-
	Bacillus sp. CMAA 1363	WP_128821162	-----G-MA--T--------------GEIEE		---FK--I--AA--M-T-
	Bacillus sp. FJAT-14578	WP_028398417	-----A-MA--T--------------G-VE-		---WK-II--AG--L-T-
	Bacillus sp. M5HDSG1-1	WP_127736707	-----A-MA--T-----------I--G-LEK		---LK-II--AG--L-T-
	Bacillus sp. M6-12	WP_101596799	-----A-MA--T-----------I--G-LED		---WK-II--AG--L-T-
	Bacillus sp. OxB-1	WP_041076568	-----A--A--------------I--R-VED		---FK-----AG--L-T-
	Bacillus sp. P14.5	WP_113930517	-----A-MA--T-----------I--G-LEE		---WK-VV--AG--L-T-
	Bacillus sp. Root147	WP_057239464	-----G-MA--T--------------GEIEE		---FK--I--AA--M-T-
	Bacillus sp. SG-1	WP_006837732	-----A-MA--T-----------I--G-LEE		---WK-VI--AG--L-T-
	Bacillus sp. V3-13	WP_101658278	-----A-MA--T-----------I--G-LEK		---LK-V---AG--I-T-
	Bacillus sp. V44-8	WP_117322294	-----A-MA--T-----------I--G-LEE		-N-WK-----AG--L-T-
	Bacillus sp. V5-8f	WP_101633919	-----A-MA--T-----------I--G-LED		-N-WK-----AG--L-T-
	Bacillus sp. mrc49	WP_100533842	-----G-MA--T-----------I--G-LEQ		---WK-V---AG--L-I-
	Bacillus sporothermodurans	WP_084347947	-----G-MA--T-----------I--G-VNE		---WK-V---AG--L-T-
	Bacillus testis	WP_050614343	-----G-MA--------------I--G-LEQ		---WK-V---AG--I-T-
	Bhargavaea beijingensis	WP_092096009	-----A-MA--------------I--G-IEE		--RVK--V--AG--L-T-
	Bhargavaea cecembensis	WP_063179718	-----A-MA--------------I--G-IEE		--R-K--M--AG--L-T-
	Bhargavaea ginsengi	WP_092054074	-----A-MA--------------I--G-IEG		--R-K--A--AG--L-T-
	Butyricicoccus sp. 1XD8-22	RKJ31452	-----------------------I--GEIDG		KN-LK--F--I---L-T-
	Chlamydia trachomatis	CRH70204	-----G-MA--T-----------I--GELEQ		---WK-V---AG--L-I-
	Chryseomicrobium excrementi	WP_100352866	-----A-MA--------------I--G-IEE		Q---K--I--AG--L-T-
	Edaphobacillus lindanitolerans	WP_076756693	-----A-MA------S-------I--G-IEE		----K--F--AG--L-T-
	Falsibacillus pallidus	WP_114744234	-----G-M---T-------V---I--G-LER		---WK------G--L-T-
	Filibacter sp. TB-66	WP_124069976	-----A--A--------------I--R-IED		---FK-----AG--FMT-
	Jeotgalibacillus alimentarius	WP_041122186	-----A-MA--T----------SI--G-IND		---TK-I---AG--L-T-
	Jeotgalibacillus campisalis	WP_041056950	-----A-MA--T--------I-SI--G-IQD		---WK-IF--AG--L-T-
	Jeotgalibacillus malaysiensis	WP_039808461	-----A-MA--T----------SI--G-IKD		----K-II--AG--L-T-
	Jeotgalibacillus proteolyticus	WP_104056650	-----A-MA--T--------I-SI--G-IED		---W--I---AG--L-T-
	Jeotgalibacillus salarius	WP_134382679	-----A-MA--T----------SI--G-IKD		----K-IM--AG--L-T-
	Jeotgalibacillus soli	WP_041088423	-----A-MA--T--------I--I--G-IKE		---WK-IM--AG--L-T-
	Jeotgalibacillus sp. R-1-5s-1	WP_134375190	-----A-MA--T----------SI--G-IKD		----K-I---AG--F-T-
	Kurthia gibsonii	WP_087682367	-----G-----------------I--G-VEE		----K--V--GL--V---
	Kurthia huakuii	WP_029498043	-----G-----------------I--G-VEE		----K-VV---L--V---
	Kurthia massiliensis	WP_010286736	-----G-----------------I--G-VEE		----K-VM--FL-SL---
	Kurthia senegalensis	WP_010302008	-----G-----------------ISNG-TEK		---WK-VT--ML--LV--
	Kurthia sibirica	WP_109305616	-----G-----------------I--G-VTG		VN-FK--I--AL--IV--
	Kurthia sp. 11kri321	WP_068455379	-----G-----------------I--G-VEE		----K--V--GL--V---
	Kurthia sp. 3B1D	WP_126990853	-----G-----------------I--G-VEE		----K-VI---L--I---
	Kurthia sp. Dielmo	WP_020189256	-----G-----------------I--G-VEE		----K-VI---L--I---
	Kurthia zopfii	WP_109350379	-----G-----------------I--G-SE-		--RIK--I--AL--V---
	Lentibacillus sp. SSKP1-9	WP_129677202	-----A-MG--T--------------GNF-Q		-G-LK-I---A---L-T-
	Lysinibacillus acetophenoni	WP_097147815	-----------------------I--GEVDG		KN-LK--I--IC--L-T-
	Lysinibacillus boronitolerans	WP_036076671	--------------------I--I--G-VEE		H--FK-----AG--LMS-
	Lysinibacillus chungkukjangi	WP_107935000	-----------------------I--GEIGG		N--IKM----VL--L-T-
	Lysinibacillus composti	WP_124763670	-----------------------I--GEIND		K--VKM----IC--L-T-
	Lysinibacillus contaminans	WP_053585434	--------------------I-----G-VEE		H--VK--I--AG--

Other Bacteria (0/>100)

Lysinibacillus endophyticus	WP_121214081	-----------------------I--GEIQE	K--FKM-M--IM--L-T-
Lysinibacillus fusiformis	WP_004227432	--------------------I--I--G-VEE	H--FK-----AG--LMS-
Lysinibacillus halotolerans	WP_122970997	-----------------------I--GEIDE	K---KM----VL--L-T-
Lysinibacillus jejuensis	WP_108306288	-----A--------------I--I--G-LED	---LK--V---F--M---
Lysinibacillus macroides	WP_053993685	--------------------I--I--G-VEE	H--FK-----AG--LMS-
Lysinibacillus manganicus	WP_036181980	-----------------------I--GEIDG	KN-FK--I--IC--L-T-
Lysinibacillus mangiferihumi	WP_107895592	--------------------I--I--G-VEE	H---K-----AG--ILT-
Lysinibacillus massiliensis	WP_036176523	-----------------------I--GEIDG	KN-LK--F--I---L-T-
Lysinibacillus meyeri	WP_107838820	-----------------------I--GEVNE	Q--LK--MK-CL--LFT-
Lysinibacillus odysseyi	WP_036155760	-----------------------I--GEV-E	K---K--M---V--L---
Lysinibacillus parviboronicapiens	WP_107925318	--------------------I--I--G-VEE	H---K-----AG--LMT-
Lysinibacillus saudimassiliensus	CEA02256	-----A--------------I--I--G-LEE	---LK--I---F--M---
Lysinibacillus sinduriensis	WP_036202483	-----------------------I--GEIGD	K--LKM----VL--L-T-
Lysinibacillus sp. B2A1	AVK82873	--------------------I--I--G-VEE	H--LK--A--AG--LMS-
Lysinibacillus sp. BF-4	WP_036143628	-----A--------------I--I--G-LEE	---LK--I---F--M---
Lysinibacillus sp. BK089	TCJ68539	--------------------I--I--G-VEE	H--LK-----AG--LMS-
Lysinibacillus sp. FJAT-14222	WP_053596292	--------------------I--I--G-VEE	H--LK-----AG--LMS-
Lysinibacillus sp. FJAT-14745	WP_053483576	--------------------I--I--G-VED	H--LK-----AG--LMS-
Lysinibacillus sp. Marseille-P	WP_106783855	-----------------------I--GEIDG	KN-FK-----IC--L-T-
Lysinibacillus sp. SGAir0095	QCR31597	-----------------------I--GEIGE	K--IKM----VL--L-T-
Lysinibacillus sp. SYSU K30002	WP_126658375	-----------------------I--GEIQE	K--IKMF---IM--L-T-
Lysinibacillus sp. YLB-03	WP_118874863	--------------------I--I--G-IDE	K---KM----VL--LT--
Lysinibacillus sp. YR326	WP_134025331	--------------------I--I--G-VEE	H--LK-----AG--LMS-
Lysinibacillus sp. ZYM-1	WP_054611601	--------------------I--I--G-VEE	H--FK-----AG--LMS-
Lysinibacillus sphaericus	WP_125102130	-----A--A--------------I--R-IED	---LK--I--AG--L-T-
Lysinibacillus telephonicus	WP_126295513	-----------------------I--GEIDE	K--AKMI---IM--L-T-
Lysinibacillus xylanilyticus	WP_100545847	--------------------I--I--G-VEE	H--LK-----AG--LMS-
Lysinibacillus xyleni	WP_097071934	-----------------------I--GEIQE	K--IKM-F--IM--L-T-
Oceanobacillus arenosus	WP_115773463	-----G--A--A--------I-----GEYEK	QG--K-I---GG--L-T-
Oceanobacillus damuensis	WP_067729603	-----A-MA--T-----------I--GEYG-	QG--K-IM--AY--L-T-
Oceanobacillus halophilus	WP_121204635	-----A-MA--T------------SNG-YE-	QG-L--V---AT--L-T-
Oceanobacillus limi	WP_090868735	-----A-MA--T--------------GEYGS	DG-LK-I---A---M-T-
Oceanobacillus rekensis	WP_087973287	-----A-MA--T--------------G-YG-	DG--K-I---AA--L-T-
Oceanobacillus senegalensis	WP_085992703	-----A-MA--T------------SNG-YE-	-G-LK-I---AT--F-T-
Oceanobacillus sp. YLB-02	WP_121523122	-----A-MA-----------------G-YE-	TG-IK-IF--TA--F-T-
Ornithinibacillus contaminans	WP_047982366	-----A-MA--T--------------GEYG-	-G-LK-I---AG--L-T-
Paenisporosarcina antarctica	WP_134210277	-----A-MA--------------I--G-IEE	Q---K--M--AG--L-T-
Paenisporosarcina indica	WP_075617327	-L---A-MA--------------I--G-IAE	Q--IK--I--AG--L-T-
Paenisporosarcina quisquiliarum	WP_090563168	-----A-MA--------------I--G-IEE	Q--TK--F--AG--L-T-
Paenisporosarcina sp. HGH0030	WP_016427999	-----A-MA--------------I--G-IAE	Q--LK--M--AG--L-T-
Paenisporosarcina sp. K2R23-3	WP_119882769	-----A-MA--------------I--G-IEE	----S-MV--AG--L-T-
Paenisporosarcina sp. OV554	WP_108584268	-----A-MA--------------I--G-IAE	Q---K--M--AG--L-T-
Paenisporosarcina sp. TG-14	WP_017381603	-----A-MA--------------I--G-IEE	Q---K--M--AG--L-T-
Paenisporosarcina sp. TG20	WP_019413403	-----A-IA-----------I--I--G-IK-	Q-NLK--M--GG--L-T-
Planococcus antarcticus	WP_006829717	-----A-MA--------------I--G-IEE	Q---K--F--AG--L-
Planococcus citreus	WP_121297852	-----A-MA--------------I--G-IEE	----K--F--AG--L-T-
Planococcus donghaensis	WP_065525818	-----A-MA--------------I--G-IEE	----K--F--AG--L-T-
Planococcus faecalis	WP_071153554	-----A-MA--------------I--G-IEE	----K-----AG--L-
Planococcus halocryophilus	WP_008498122	-----A-MA--------------I--G-IEE	----K--F--AG--L-
Planococcus kocurii	WP_058384756	-----A-MA--------------I--G-IEE	----K--F--AG--L-
Planococcus maitriensis	WP_112231177	-----A-MA--------------I--G-IEE	---IK--F--AG--L-T-
Planococcus maritimus	WP_068463147	-----A-MA--------------I--G-IEE	----K--F--AG--L-
Planococcus massiliensis	WP_052650983	-----A-MA--------------I--G-IEE	-------F--AG--L-T-
Planococcus plakortidis	WP_068869554	-----A-MA--------------I--G-IEE	---LK--F--AG--L-T-
Planococcus rifietoensis	WP_058383408	-----A-MA--------------I--G-IEE	---LK--F--AG--L-T-
Planococcus salinarum	TAA73157	-----A-MA--------------I--G-IEE	Q---K--Y--AG--L-T-
Planococcus salinus	WP_123166309	-----A-MA--------------I--G-IEE	---IK--F--AG--L-
Planococcus sp. CAU13	WP_033541623	-----A-MA--------------I--G-IED	---L---F--AG--L-T-
Planococcus sp. PAMC 21323	WP_038704429	-----A-MA--------------I--G-IEE	----K-----AG--L-
Planococcus sp. Y42	WP_077589796	-----A-MA--------------I--G-IEE	---LK--A--AG--L-T-
Planococcus versutus	WP_049693458	-----A-MA--------------I--G-IEE	---TK--F--AG--L-
Planomicrobium flavidum	WP_088005650	-----A-MA--------------I--G-IEE	---LK--A--AG--L-T-
Planomicrobium glaciei	WP_036807298	-----A-MA--------------I--G-IEE	----K--F--AG--L-
Planomicrobium koreense	WP_135505604	-----A-MA--------------I--G-IEE	K--LK---Q-AG--L-T-
Planomicrobium soli	WP_106534743	-----A-MA--------------I--G-IEE	---VK-----AG--L-
Psychrobacillus insolitus	WP_111438766	-----A-MA--------------I--G-IEK	Q---K--F--AG--L-T-
Psychrobacillus psychrodurans	WP_093494301	-----A-MA--------------I--G-IEE	Q--TK--F--AG--L-T-
Psychrobacillus psychrotolerans	WP_093535572	-----A-MA--------------I--G-IEE	---TK--F--AG--L-T-
Psychrobacillus sp. OK028	WP_093060267	-----A-MA--------------I--G-IAE	Q--TK--F--AG--L-T-
Psychrobacillus sp. OK032	WP_093265450	--------A-----------I--I--G-IEK	Q---K--A--AG--LTT-
Rummeliibacillus pycnus	WP_102692872	-----G-------------S---I--G-VE-	TK-LK--F---I--I---
Rummeliibacillus stabekisii	WP_066784818	-----G-----------------I--GNSEE	DN-WA-M---MV--I---
Salimicrobium album	WP_093105411	-----A-MA--T--------I--I--GEIRK	QG---MM---A---L-T-
Salimicrobium jeotgali	WP_008590057	-----A-MA--T--------I--I--GEIRK	QG---MM---A---L-T-

**Other Bacteria (0/>100)**	Salimicrobium sp. WN024	WP_095821681	-----A-MA--T--------I--I--GEIRK	QG---MM---A---L-T-
	Salinibacillus kushneri	WP_093137388	-----A-MA--T-----------I--GESK-	DGQV-I-M--AL--L-T-
	Solibacillus isronensis	WP_079524784	----------------------I--G-IGG	K--F--IV------L---
	Solibacillus silvestris	WP_065216512	----------------------I--G-IGG	K--F--IV------L---
	Solibacillus sp. R5-41	WP_099422565	----------------------I--G-IGG	K--F--I----G--V---
	Sporosarcina koreensis	WP_060206124	-----A--A--------------I--R-VED	---FK-----AG--L-T-
	Sporosarcina newyorkensis	WP_009765710	-----A--A------S-------I--R-IED	---FK--V--AG--L-T-
	Sporosarcina pasteurii	WP_115364041	-----A--A--------------I--R-IED	---FK-----AG--LLT-
	Sporosarcina psychrophila	WP_082786837	-----A--A--------------I--G-VED	---FK--I--AG--LMT-
	Sporosarcina sp. BI001-red	WP_116015971	-----A--A--------------I--R-IEE	---LK--I--AG--L-T-
	Sporosarcina sp. D27	WP_025786072	-----A--A--------------I--R-IED	---FK--I--AG--L-T-
	Sporosarcina sp. EUR3 2.2.2	WP_024535265	-----A-MA--------------I--G-IAE	Q---K--M--AG--L-T-
	Sporosarcina sp. HYO08	WP_067407964	-----A--A--------------I--R-IED	---FK-----AG--I-T-
	Sporosarcina sp. P13	WP_099688303	-----A--A------S-------I--R-IED	---LK--I--AG--L-T-
	Sporosarcina sp. P16b	WP_099674209	-----A--A------S-------I--R-IED	---LK--I--AG--I-T-
	Sporosarcina sp. P18a	WP_099675243	-----A--A------S-------I--R-IED	---LK--I--AG--I-T-
	Sporosarcina sp. P19	WP_099692197	-----A--A------S-------I--R-IED	---IK--I--AG--I-T-
	Sporosarcina sp. P20a	WP_099679158	-----A--A------S-------I--R-IED	---LK--M--AG--I-T-
	Sporosarcina sp. P33	WP_081244184	-----A--A------S-------I--R-IED	---LK--I--AG--I-T-
	Sporosarcina sp. P34	WP_099694011	-----A--A------S-------I--R-IED	---LK--I--AG--I-T-
	Sporosarcina sp. P37	ARK26329	-----A--A------S-------I--R-IED	---LK--I--AG--I-T-
	Sporosarcina sp. PTS2304	WP_114925800	-----A--A------S-------I--R-IED	---FK--I--AG--L-T-
	Sporosarcina sp. ZBG7A	WP_039042250	-----A--A--------------I--R-IED	---LK--I--AG--L-T-
	Sporosarcina ureae	WP_029052469	-----A--A------S-------I--R-IED	---IK--I--AG--I-T-
	Streptococcus pneumoniae	CJF97607	-----G-MA--T--------------GEIEE	---FK--I--AA--M-T-
	Tetzosporium hominis	WP_094941500	-----A-MA--------------I--G-IEE	----K--I--AG--L-T-
	Ureibacillus thermophilus	QBK25440	-------------------V---I--GEIDE	K--LKMIF--MG--L-T-
	Ureibacillus thermosphaericus	WP_016838240	-------------------V---I--GEIDE	K--LKMIF--MG--L-T-
	Virgibacillus chiguensis	WP_073010811	-----A-MA----------------NG-YGK	QG--K-V---A---F-N-
	Virgibacillus dokdonensis	WP_116277252	-----A-MA----------------NG-YGK	QG--K-V---A---F-N-
	Virgibacillus pantothenticus	WP_077297324	-----A-MA--A-------------SG-YGK	QG--K-V---A---L-N-
	Virgibacillus sp. Bac330	WP_121639343	-----A-MA----------------NG-YGK	QG--K-V---A---F-N-
	Viridibacillus arvi	WP_053417717	-----G-----------------I--G-VEE	----K--I--AI--I-T-
	Viridibacillus sp. FSL H8-0123	WP_076065657	-----G-----------------I--G-VEE	----K-----AI--I-T-
	Viridibacillus sp. OK051	WP_100794637	-----G-----------------I--G-VED	----K-----AI--I-T-

Supplemental Figure 91

A partial sequence alignment of the magnesium transporter protein containing a one amino acid insertion (boxed) that is exclusively shared by all members belonging to the genus *Caryophanon* and absent in all other bacteria.

Group	Organism	Accession	80		120
Caryophanon **(2/2)**	Caryophanon tenue	WP_066542157	LNGIIHPAIRAEMIRQRDEYVAQG		KHVVMDIPLLFESKL
	Caryophanon latum	WP_066461930	--A-----------A---A---E-		---I-----------
**Other Bacteria (0/>100)**	Anoxybacillus tepidamans	WP_027410192	--A-V---V-KT-MA--ES-IQS-	A	-T--L--------N-
	Bacillus aryabhattai	WP_074677755	--A-V---V-K--LS-KER-IEE-	Y	DA--L--------D-
	Bacillus atrophaeus	WP_106046073	----V---V-----SK--LA-SNN	E	PF--L----------
	Bacillus cecembensis	WP_057989778	------------------H--K-	E	QT-I-----------
	Bacillus cereus	WP_002145438	--K-V---V-E--NA-KEL-IKE-	M	QS--L----------
	Bacillus gaemokensis	WP_033672864	--K-V---V-N--NA-K-M-IKE-	V	QAI-L----------
	Bacillus manliponensis	WP_034639748	--H-V---V-K--QK-KEA-IRE-	R	EV--L----------
	Bacillus marmarensis	WP_022628827	--Q-V---V-R--LA-K-R-E-E-	H	ETIIF--------N-
	Bacillus megaterium	WP_116514963	--A-V---V-K--LS-KES-IEE-	Y	EA--L--------D-
	Bacillus ndiopicus	WP_042474726	--D-------T--L-----LLHG-	A	E--------------
	Bacillus panaciterrae	WP_028400936	--S-V---V----NA-K-A-IQK-	E	-A--L--------N-
	Bacillus pseudofirmus	WP_075681418	--Q-V---V-R--LA-K-R-E-E-	H	ETIIF--------N-
	Bacillus pseudomycoides	WP_006096183	--K-V---V-K--NV-K-M-IKE-	V	QA--L----------
	Bacillus sp. Aph1	WP_034265604	--A-V---V-K--LS-KER-IEE-	Y	DA--L--------D-
	Bacillus sp. B14905	EAZ86003	--D-M-----K--LQ---A-LED-	H	E--------------
	Bacillus sp. B14905	WP_043990216	--D-M-----K--LQ---A-LED-	H	E--------------
	Bacillus sp. FJAT-21351	WP_053487771	--A-V---V-K--LS-KES-IEE-	Y	EA--L--------D-
	Bacillus sp. FJAT-42376	WP_123918342	----V--QV-Q--V------IQ--	K	-A-IL----------
	Bacillus sp. NSP22.2	WP_026569737	--D-V---V-EK-LE---AFA-K-	K	EA--L-----Y--S-
	Bacillus sp. OxB-1	WP_041075519	----------K--H--K--WLEK-	A	NT-IL----------
	Bacillus sp. PK3_68	WP_120033713	--S-V---V-QF--AEK-AA--A-	K	QTII---------E-
	Bacillus sp. Root147	WP_057233248	--A-V---V-K--LS-KES-IEE-	Y	EV--L--------D-
	Bacillus sp. Root239	WP_057244995	--A-V---V-K--LS-KES-IEE-	Y	EV--L--------D-
	Bacillus sp. SA5d-4	WP_094923284	--S-V---V-LR-NDKK-M-ISE-	R	NT-IF----------
	Bacillus sp. SJS	WP_035410935	----V--EV-K--------LIQ--	S	-A-IL----------
	Bacillus sp. UNC437CL72CviS29	WP_026593623	--K-V---V-K--NA-K-M-IKE-	V	QA--L----------
	Bacillus subtilis group	WP_003325119	----V---V-----SK--LA-SNN	E	PF--L--------N-
	Bacillus taeanensis	WP_113804340	--N-V---V-KK--E-K-T-LQ--	Y	H-I-L----------
	Bhargavaea beijingensis	WP_092097620	--D-M-----K--L----RHFER-	A	-T-----------R-
	Bhargavaea cecembensis	WP_008299699	--D-M-----Q--L----GHFDR-	A	-T-----------R-
	Bhargavaea ginsengi	WP_092051841	--D-V-----Q--L----RHFEN-	A	RT-----------R-
	Butyricicoccus sp. 1XD8-22	RKJ08779	--N----------L--KE-HL-N-	A	-T-I-----------
	Chryseomicrobium excrementi	WP_100352284	----------Q--L--K-V-----	E	-I-IL----------
	Edaphobacillus lindanitolerans	WP_076758140	--D-------T--L----GHL-A-	K	-T-----------R-
	Fictibacillus gelatini	WP_035350039	--S-V---V-Q--R--A-QFL-E-	Y	ET--L----------
	Fictibacillus macauensis	WP_040340494	--A-V---V--QLKKEAES-L---	Y	P--------------
	Filibacter sp. TB-66	WP_124069854	--S-------K--L--KESWLSN-	A	QT-I-----------
	Geobacillus sp. 44C	WP_081188836	--A-V---V-RR-LAEKEA--QK-	A	-TI-L--------E-
	Geobacillus sp. Y4.1MC1	WP_013400247	--A-V---V-RR-LAEKEA--QK-	A	-T--L--------E-
	Jeotgalibacillus alimentarius	WP_041120939	--Q-V---V--D-LS-K-QAF-A-	K	HT-FL--------E-
	Jeotgalibacillus soli	WP_041090928	--E-V---V--D-LA-KEQLLNA-	K	QTII-----------
	Kurthia gibsonii	WP_121177877	----------K--L-----WLNN-	A	EV-I-----------
	Kurthia huakuii	WP_029499436	--D-------K--L------LSD-	H	P------------G-
	Kurthia massiliensis	WP_010288264	--D-------K--L------LSD-	H	L------------G-
	Kurthia sp. 11kri321	WP_068451970	----------K--L-----WLNN-	A	EV-I-----------
	Kurthia sp. 3B1D	WP_126989228	--E-------K--L----KHLSD-	H	P------------G-
	Kurthia zopfii	WP_109349029	--D-M-----K--LA---CW-E--	A	EV-------------
	Lentibacillus amyloliquefacien	WP_068443660	--N-V---V-EK--ER--AFA-S-	E	-S--L----------
	Lentibacillus halodurans	WP_090239213	--A-V---V-GK-LKK--N--GT-	V	-S--L----------
	Lentibacillus jeotgali	WP_010532421	--S-V---V-EK--ER--AFA-A-	E	RS--L----------
	Lentibacillus sp. SSKP1-9	WP_129672914	--S-V---V-ER-VSK--V--SS-	A	-S--L-----Y--N-
	Lysinibacillus acetophenoni	WP_097148528	--D----------L-----HI-N-	A	-T-I-----------
	Lysinibacillus boronitolerans	WP_036078324	--D-M-----K--L----A-LEA-	H	E--------------
	Lysinibacillus chungkukjangi	WP_107934222	----------Q--L-----H-EN-	E	-T-------------
	Lysinibacillus composti	WP_124762437	--D-------Q--L-R----I-N-	E	-T-I-----------
	Lysinibacillus contaminans	WP_053582479	--D-M-------------ALL-A-	E	---------------
	Lysinibacillus endophyticus	WP_121212719	--D----------L-----H--N-	A	-T-I-----------
	Lysinibacillus fluoroglycofeni	WP_107942958	--D----------L-----LLLG-	A	E--------------
	Lysinibacillus fusiformis	WP_004226271	--D-M-----K--L----A-LEA-	H	E--------------
	Lysinibacillus halotolerans	WP_122971128	--R-------Q--L-----HI-K-	A	QT-I-----------
	Lysinibacillus jejuensis	WP_108306444	--D-------T--L-------TN-	H	Q--------------
	Lysinibacillus macroides	WP_053996439	--D-M-----K--L----A-LEA-	S	-------------Q-
	Lysinibacillus manganicus	WP_036188243	--D----------L-----HL-N-	A	-T-I-----------
	Lysinibacillus mangiferihumi	WP_107895030	--D-M-----Q--L----A-IEA-	Q	---------------
	Lysinibacillus massiliensis	WP_036173931	--N----------L--KE-HL-N-	A	-T-I-----------
	Lysinibacillus meyeri	WP_107839797	--D----------L-----F-QG-	A	A--------------
	Lysinibacillus odysseyi	WP_036156280	--D-------V-----KE--LK--	A	P--I-----------
	Lysinibacillus parviboronicapiens	WP_107923412	--D-M-----E--L----AFIKA-	Q	--I------------
	Lysinibacillus saudimassiliensis	CEA05651	--D-------T--L-------TN-	H	Q--------------
	Lysinibacillus sinduriensis	WP_036198699	----------Q--L-----HFGN-	A	NT-------------
	Lysinibacillus sp. AC-3	SKB74538	--DMM-----K--L----A-IDA-	E	------------N--
	Lysinibacillus sp. B2A1	AVK83930	--D-M-----Q--L----A-IEA-	N	---------------

Other Bacteria (0/>100)

Lysinibacillus sp. BF-4	WP_036142318	--D-------T--L-------TN-	H	Q--------------
Lysinibacillus sp. BK089	TCJ74815	--D-M-----Q--L----A-LEA-	E	---------------
Lysinibacillus sp. FJAT-14222	WP_053592408	--D-M-----K--L----A-LES-	E	---------------
Lysinibacillus sp. FJAT-14745	WP_053484962	--D-M-----H--L----A-LEA-	E	---------------
Lysinibacillus sp. LK3	WP_048393604	--D-------K--L----A-LEA-	H	E--------------
Lysinibacillus sp. Marseille-P	WP_106780369	--D----------L-----HI-K-	A	-T-I-----------
Lysinibacillus sp. OL1	WP_131521356	--D-M-----K--L----A-LEA-	H	E--------------
Lysinibacillus sp. PB300	WP_115674489	--D-M-----K--L----A-LEA-	H	E--------------
Lysinibacillus sp. SG9	SCY95637	--D-M-----K--L----A-LEA-	H	E--------------
Lysinibacillus sp. SGAir0095	QCR33221	--S-------Q--L-----HIEN-	S	-T-I-----------
Lysinibacillus sp. SYSU K30002	WP_126657322	--D----------L-------TN-	E	-T-I-----------
Lysinibacillus sp. YLB-03	WP_118877327	--S-------Q--L------LDA-	A	-T-------------
Lysinibacillus sp. YR326	WP_134021865	--D-M-----Q--L----A-LEA-	E	---------------
Lysinibacillus sp. ZYM-1	WP_054610814	--D-M-----K--L----A-LEG-	Y	E--------------
Lysinibacillus sphaericus	WP_010860168	--D-M-----Q--L----AF-EV-	Q	T-I------------
Lysinibacillus telephonicus	WP_126295377	--A-------Q--L------LEN-	E	-T-------------
Lysinibacillus varians	WP_025220298	--D-M-----Q--L----A-IEA-	Q	---------------
Lysinibacillus xylanilyticus	WP_068983953	--D-M-----Q--L----A-LEA-	E	---------------
Lysinibacillus xyleni	WP_097072862	--D-------S--L-----H--N-	E	-T-I-----------
Oceanobacillus chungangensis	WP_115748051	--S-V--EV-KK--AE--ALI-E-	E	RCI-L--------N-
Oceanobacillus limi	WP_090870464	----V---V-ER-LEK--T--EA-	E	-CI-L----------
Oceanobacillus manasiensis	WP_042223258	--N-V---V-EK--TK--A--KE-	L	PI--L----------
Paenisporosarcina indica	WP_075620038	--DLM--------L----KLIQD-	H	STII-----------
Paenisporosarcina sp. HGH0030	WP_016426698	--DLM--------L-----LFES-	E	ETLI---------R-
Paenisporosarcina sp. OV554	WP_108585344	--DLM-----S--F-----HLKS-	Q	-TLI---------R-
Paenisporosarcina sp. TG20	WP_019415942	--EL------T--L-M---ILQS-	E	-DM------------
Parageobacillus caldoxylosilyt	WP_017437240	--A-V---V-RR-MAEKE---QS-	A	-TI-L--------E-
Parageobacillus genomosp. 1	WP_043905728	--A-V---V-RR-MAEKEA--QN-	A	-TI-L--------E-
Parageobacillus thermantarctic	WP_090947612	--A-V---V-RR-LAEKEA--QK-	A	-T--L--------E-
Parageobacillus thermoglucosid	WP_125009364	--A-V---V-RR-LAEKEA--QK-	A	-T--L--------E-
Parageobacillus toebii	WP_062679222	--A-V---V-RR-LAEKEA--QK-	A	-TI-L--------E-
Paucisalibacillus globulus	WP_096269657	--S-V---V-KR-LE-KET-LKD-	E	SC-------------
Planococcus antarcticus	WP_006831139	--D-------Q--L---Q-LL---	F	-TII---------R-
Planococcus citreus	WP_121300244	--D-------R--L---QAFLDE-	Y	ET-I-----------
Planococcus faecalis	WP_071153188	--D-------Q--L---QDFL---	F	-TII---------R-
Planococcus kocurii	WP_058385713	--D-------Q--L---QDFL---	F	-TII---------R-
Planococcus maitriensis	WP_112232624	--D-------R--L---QAFL-G-	H	ET-I-----------
Planococcus maritimus	WP_068488493	--D-------R--L---QVFLEA-	N	ET-I-----------
Planococcus massiliensis	WP_052651996	--DV------Q--L---S-LLE--	H	ETII---------R-
Planococcus plakortidis	WP_068868884	--D-------R--L---QAFL-E-	Y	ET-I-----------
Planococcus rifietoensis	WP_058381072	--D-------R--L---QAFLDD-	Y	ET-I-----------
Planococcus salinarum	TAA70456	--D-------E--L---NA-I---	F	ETII-----------
Planococcus salinus	WP_123163614	--DV------K-------H--Q--	F	DI-I-----------
Planococcus sp. CAU13	WP_033542166	--D----------L---N--LEA-	H	-TI------------
Planococcus sp. Y42	WP_077588702	--D-------Q--L---E-WL-N-	A	QT-I-----------
Planomicrobium flavidum	WP_088006619	--EVM-----Q--L----GWLEN-	A	DT-I---------R-
Planomicrobium glaciei	WP_053165458	--DV------M--L---G-FLD--	F	ET-I---------R-
Planomicrobium koreense	WP_135500323	--D-------N--L---AD-M-E-	Q	-V-I-----------
Planomicrobium okeanokoites	WP_117312072	--D-------T--L--KSVFMED-	H	RTI------------
Planomicrobium soli	WP_106533901	--N-------K-IL----AFL---	H	-T--L----------
Planomicrobium sp. MB-3u-38	WP_101801719	--D----------L--KSVFIED-	H	RTI------------
Planomicrobium sp. Y74	WP_121633367	M-D----------L---SAFM-E-	H	QTI------------
Pontibacillus yanchengensis	WP_036822086	--SFV---V-E--VQ---F--TE-	Y	-A--L--------D-
Pseudogracilibacillus auburnen	WP_110396201	--E-------K-------R-IE--	E	-S--L-----Y--G-
Psychrobacillus insolitus	WP_111437682	--D----------L--KE-LIKE-	H	PTI------------
Rummeliibacillus pycnus	WP_102691083	--E-------K--L-----WLEK-	E	--I------------
Rummeliibacillus stabekisii	WP_066788806	--S-M-----Q--L-----LTEA-	E	P-I----------H-
Sediminibacillus albus	WP_093216114	--K-V---V-KK-L----N--EA-	E	SA--L----------
Sediminibacillus halophilus	WP_074601073	--S-V---V-KK-LE---T-ADK-	E	EA--------Y--D-
Sediminibacillus massiliensis	WP_077621280	--E-V---V-K---AEKER-LS--	E	-A--L--------N-
Solibacillus isronensis	WP_079528373	----------E--L-----FISF-	E	-NIF-----------
Solibacillus kalamii	WP_087615792	--S-------E--L-----LMSY-	E	-NIF-----------
Solibacillus silvestris	WP_014823131	--S-------E--L-----LMSY-	E	-NIF-----------
Solibacillus sp. R5-41	WP_099424817	-------------L-----HL-K-	E	-T-I-----------
Sporosarcina koreensis	WP_082713687	--------V-K--L--KE-WLEK-	A	NT-I-----------
Sporosarcina newyorkensis	WP_078816870	--ELM-----G--VS-KEQ-LQ--	Y	QT-I-----------
Sporosarcina pasteurii	WP_134268479	--T-------T--L----KHISN-	A	NTII-----------
Sporosarcina sp. BI001-red	WP_116019485	--A-------K-L-N-KEAHLVA-	A	-T-I--------N--
Sporosarcina sp. EUR3 2.2.2	WP_024534243	--DLM-----S--L-----LFKS-	H	-TLI---------R-
Sporosarcina sp. HY008	WP_067402841	----------K--LS-KEKWI-A-	A	GTII-----------
Sporosarcina sp. P19	WP_099690649	--ELM-----GR-VS-KED-LT--	Y	QT-I-----------
Sporosarcina sp. P20a	WP_099677448	--ELM-----GR-VS-KED-LT--	Y	ETII-----------
Sporosarcina sp. P3	WP_099639892	--ELM-----GR-VS-KED-LT--	Y	QT-I-----------
Sporosarcina sp. ZBG7A	WP_039043972	--N-------K-L-A-KEAHL-A-	E	-T-IL-------N--

**Other Bacteria (0/>100)**	Sporosarcina ureae	WP_029052902	--ELM-----GR-VS-KED-LT-- Y QT-I----------
	Tetzosporium hominis	WP_094944119	----------Q--L--K-V-L--- E -I-IL---------
	Ureibacillus thermophilus	QBK26498	--S-------K--L-----HL-N- E -T-I----------
	Ureibacillus thermosphaericus	WP_016837141	----------K--L------I-N- E -T-I----------
	Virgibacillus phasianinus	WP_089062253	--E-V---V-GN-LK--EA--DA- E -C--L---------
	Virgibacillus profundi	WP_095655011	--E-V---V-EN-LK---A--NAE A NC--L---------
	Virgibacillus senegalensis	WP_053218944	--K-V---V--K-LE---Q--DK- E -A--L-----Y--S-
	Virgibacillus siamensis	WP_077327408	----V---V-EK-LSK--A-MES- E RCIIL--------N-
	Viridibacillus arvi	WP_053416604	--D-------Q--L-----LLEN- A -DI---------R-
	Viridibacillus sp. OK051	WP_100794497	--D-------R--L-----LLEN- A R-II--------R-

Supplemental Figure 92

A partial sequence alignment of the dephospho-CoA kinase protein containing a one amino acid deletion (boxed) that is exclusively shared by all members belonging to the genus *Caryophanon* and absent in all other bacteria.

			1		53
***Caryophanon* (2/2)**	Caryophanon latum	WP_066466362	MLDLHQIYAAQKRGLFFLLAGCALGWGF	L	TSYQTIFAGIALGALFGTYNFWIL
	Caryophanon tenue	WP_066546948	---------T------------------	-	-------------F----------
**Other Bacteria (0/>100)**	Aeribacillus pallidus	WP_063389019	-DRIEKLFVRS-KY-L----IYV-A---		-P--D--L-L---TS-SL--L---
	Bacillus abyssalis	WP_078414415	-Q--Q-MFPRLRSYI--I--LFV-----		-E--SV-L-L---TV-SL--L-T-
	Bacillus alveayuensis	WP_044896343	-YRYNNLWTRSRKYILY----YV-----		-P-----L-LI--TS-SL--L-L-
	Bacillus aquimaris	WP_044339992	-PE-Q-VFNRHRKYI----SFYV-----		----S--L-LI--TGISLFSH-LI
	Bacillus bataviensis	WP_007086160	-PE-QFKFSR-R-WM-S--SIYV-----		-------L-LVF-TSLSLF-L-LM
	Bacillus campisalis	WP_046523764	-PEIQVVF-R-RKYI----SIYV-----		-V-KP----LVI-TIVSLF-L-L-
	Bacillus cavernae	WP_126863527	-P--QTT-KRHLKY-LYF--I--F----		-D--DV---L---TA--VF-L-LI
	Bacillus cecembensis	WP_057986745	-----HVF-L--KAFL----L-----V-		SP-ES---------F---------
	Bacillus cereus	WP_098349048	-I-V-GLVQR--KYMYY---LLV-----		---KDV-L-LII-TI-S
	Bacillus coahuilensis	WP_059283595	-P--SHMFKRHQKYI-Y--SLAV-----		-P-K-L-L-FI--FSI-S--H-L-
	Bacillus cucumis	WP_101646688	-P-FRVM-NR--KWI-Y---IYV-----		-----L-L-LI--TC--FL-L-L-
	Bacillus dakarensis	WP_077215064	-P-VKT--SR-S-YI-T--SLYV--Y--		---K---L-LI--TSLSLF-I-L-
	Bacillus dielmoensis	WP_042463561	-P-FRTMFNR-RKWM-Y--SIYV-----		-T--P--L-LI--TC--YL-L-M-
	Bacillus drentensis	WP_066248641	-P-FRVM-NR-RKWI-Y--SIYV-----		-------L-LI--TC-SYL-L---
	Bacillus fastidiosus	WP_066230910	---I-LMFHRYRKFI-----VYV-----		-E--SV-L-LI--TAVSL--L-VM
	Bacillus flexus	WP_061785833	-QE-QHLFPRLRSYI-Y---LYV-----		-P---V-L-LI--TALSL--L-V-
	Bacillus foraminis	WP_132008064	-P-IQV-FTR-RKYI----SIYV-----		-E-K-V---LI--TGISL--M-L-
	Bacillus fordii	WP_018708431	-D--Q-LFNRHRKYILY---IFV-----		S-F-S--M-LI--TSISFF-L-MM
	Bacillus freudenreichii	WP_126430840	-D--Q-LFNRHRKYILY---IFV-----		-P--S--M-LI--TSISFF-H-MM
	Bacillus horneckiae	WP_066398256	-PEFKATFVR-RKYM----SFYV-----		-P--PV-L-LF--TS-SLF-L-LM
	Bacillus indicus	WP_029566598	-F----M-RRYRKYM-L--SLY------		-GH-SV-L-L---TTLSLF-L-
	Bacillus infantis	WP_129612994	-P--KTMFIR-RKYI-L--SVYV-----		----S--M-LI--TSLSLF-L-LM
	Bacillus kochii	WP_095371587	-PEIQERFSR-RKYML---SFFV-----		----S--L-LI--TVLSFF-L-LI
	Bacillus korlensis	WP_066059976	-PEIQS--IR-RKYI-----LYV-----		-------L-LI--TSLSLF-L-L-
	Bacillus kwashiorkori	WP_062351551	---VVNLFGRYRKY-Y----FFV-----		-T--H----LFI-TSISFI-L---
	Bacillus kyonggiensis	WP_136831336	-P-VQT--RQ-QKYIL---SIYV-----		-T-K---L-LI--TA--LF-M-L-
	Bacillus litoralis	WP_121663869	-F-M-LMFRRYRKYI-Y---IYV-----		-E--SV-L-LI--TCITL--L--M
	Bacillus massiliogabonensis	WP_102273616	-PE-KAT-VR-RKYI-L--SIYV-----		----S--L-LV--TSCSLF-L-L-
	Bacillus massilionigeriensis	WP_075983187	-PE-QAM-IRERKYI----SFYV-----		---KS--L-LI--TSLSLF-L-LM
	Bacillus massiliosenegalensis	WP_019153963	-PEIKT--RR-Q-YI-----IYV-----		-------L-LI--TAL-LF-M-L-
	Bacillus megaterium	WP_098580333	-Q--QHVFPRLRSYILY---LYV-----		---KAV---LI--TALSL--L-
	Bacillus mesonae	WP_066389521	-PE-QE-FTRS-KWM-Y---IYV-----		----SV-L-LI--TGLSLF-L-LM
	Bacillus methanolicus	WP_003346811	-PEMNEMI-R-RKYI-Y---VYV-----		----S--L-LIF-TSLSFF-L-LI
	Bacillus muralis	WP_057911908	-PEMKIMFNR-SKYII-----YVV---L		----AV---LI--TSLSL--L-LI
	Bacillus ndiopicus	WP_042470889	-E---Y-F-K--KV------L-V---A-		-P-AS-----G---F---------
	Bacillus niacini	WP_063254595	-PEFQTMF-R-RKWM----SFYV-----		-AH-S--L-LI--TSLSLF-L-LM
	Bacillus novalis	WP_066093872	-PEYQL-FSRLR-WI-----IYV-----		----P--L-LVF-TSLSLF-L-LM
	Bacillus oceanisediminis	WP_110063728	-PEIKA-FQR-RKYI-L---VYV-----		-P--S--L-LI--TSLSLF-L-LM
	Bacillus oleivorans	WP_097156804	-D--KA-FIRGSKYIL---SFYV-----		-P------LV--TTLSLF-----
	Bacillus persicus	WP_090748826	-QEWQ-MTIRYRKYM-Y---LYV-----		----SV---LI--TSL-FF-M-L-
	Bacillus praedii	WP_057760176	-PE-KAT-VR-RKYI-L--SIYV-----		----S--L-LV--TSCSLF-L---
	Bacillus rubiinfantis	WP_042354360	-PE-QA--NRS-KWMLY-M-LYI-----		----S--L-L-F-TSLSLF-L-L-
	Bacillus simplex	WP_137017340	-PEMKIMFNR-SKYII----VYVV---L		----AV---LI--TSLSL--L-LI
	Bacillus solani	WP_056686779	-PE-KAT-VR-RKYI-L--SIYV-----		----S--L-LV--TSCSLF-L-L-
	Bacillus soli	WP_066072969	-PEIQVMF-R-R-WM-S--SIYV-----		----SV-L-LVF-TSL-LF-L-LM
	Bacillus sp. 1NLA3E	WP_015596074	-S-FKAM-KREQKYI-----FYCI---V		---KSV-L-LI--TSLSL--L-MM
	Bacillus sp. 3-2-2	WP_126046720	-D--Q-LFNRHRKYILY---ILV-----		-P--S--M-LI--TSISFF-H-MM
	Bacillus sp. 7504-2	WP_095312067	-Q-IKTT-MR-R-S-----SFYV-----		-D-KP--L-LI--TALSLF-L-L-
	Bacillus sp. 7586-K	WP_095301536	-F-M-LMFRRYRKYI-Y---IYV-----		-E--SV-L-LI--TSITL--L--M
	Bacillus sp. AFS006103	WP_098262254	-PEFRVM-NR-RKWI-Y--SIYV-----		----P--L-LI--TC-SYL-L---
	Bacillus sp. AFS040349	WP_098795459	-FEM-LMFRRYRKYI-Y---VYVI----		-E--SV-L-LI--TCITL--L--M
	Bacillus sp. AFS073361	WP_098571525	-P-FRVM-NR-RKWI-Y--SIYV-----		-----V-L-LI--TC-SYL-L-F-
	Bacillus sp. B-jedd	WP_048827235	-S--RA--FRVR-Y-L---SIYV-----		-DH-Q----LI--TALSLF-L-LM
	Bacillus sp. CGMCC 1.16541	WP_110112622	-QE---MFPRLRSYM-N---LYV-----		----SV-L-LI--TSLSL-SL-V-
	Bacillus sp. EB01	WP_043930051	-S-IGT--SRIR-Y-----SIYV-----		-D-PH--G-LI--TALSMF-L-LM
	Bacillus sp. FJAT-21351	WP_053487820	-Q--QHVFPRLRSYILY---LYV-----		---KAV---LI--TTLSL--L-
	Bacillus sp. FJAT-21352	WP_053536890	-PEMKIMFNR-SKYII----VYVV---L		----AV---LI--TSLSL--L-LI
	Bacillus sp. FJAT-21945	WP_053478545	-PE-KAT-VR-RKYI-L--SIYV-----		----S--L-LV--TSCSLF-L-L-
	Bacillus sp. FJAT-25496	WP_057772841	-S--KTM-VR-RKYI-L--SFYV-----		----S--L-LI--TVMSL--L-LM
	Bacillus sp. FJAT-27225	WP_066205917	-P--RA---RTRKY-----SLYV-A---		-DHRQ----LFM-TALSLF-L-M-
	Bacillus sp. FJAT-27245	WP_053368382	-S--RA--FRIR-Y-----SLYV-----		-GHKQ----LI--TALSLF-L-LM
	Bacillus sp. FJAT-27251	WP_053361066	-P--Q-MTSRYRQYM-Y---VYV-----		----PV---LI--TSL-FF-V-L-
	Bacillus sp. FJAT-27445	WP_059171847	-T--TA--FRI--Y-----SLYV-----		-GHKP----LI--TALSLF-L-LM
	Bacillus sp. FJAT-29814	WP_066310015	-PE-QAMFSR-R-WI-Y--SIYV-----		-A-KS--L-LIF-TSLSLF-L-LM
	Bacillus sp. FJAT-29937	WP_066296774	-PEFKVMFIR-RKYI-L--SVYV-----		----S--L-LI--TVLSLF-L-LM
	Bacillus sp. J33	WP_026583389	-PEIKA--QR-RKYI-L--SVYV-----		-P--S--L-LI--TSLSLF-L-LI
	Bacillus sp. LF1	WP_090634827	-PEFRV--NR-RKWI-Y--SIYV-----		---K---S-LI--TC-SFL-L-M-
	Bacillus sp. MRMR6	WP_075686720	-PEFQTMFVR-RKWM----SFYV-----		-T--S--L-LV--TSLSLF-L-LM
	Bacillus sp. MUM 116	WP_071354912	-P-FREMFSR-RKWNLY--SIFV-----		-----V-L-LI--TC-SFL-I-L-
	Bacillus sp. OK048	WP_090762677	-PEFQTMF-R-RKWM----SFYV-----		-A--S--L-LI--TSLSLF-L-LM
	Bacillus sp. OxB-1	WP_041073135	-QT-QE-HKR---V------LFV---A-		-GFPQ----LI--S---L------
	Bacillus sp. P14.5	WP_113928279	-TEIQ-M-NRHRKYI-Y--SIYV-----		-D--NV-L-L-F-TAISLF-H-L-

Other Bacteria (0/>100)

Bacillus sp. SA1-12	WP_046588568	-N-V-LM-RRYRSYI-Y--SFYV----L	-D--SV-L-LI--TSISL-HL--M
Bacillus sp. T33-2	WP_101582019	--EIND-FTR-RKYI-Y--SVYV-----	---KSVYL-LI--TSLSLF-L-L-
Bacillus sp. UMB0893	WP_101567338	-F----M-RRYRKYM-Y--SLY-I----	-E--SV-L-L---TGLSLF-L-
Bacillus sp. UMB0899	WP_102230389	-S-MQLMFQRYRKYI-Y---LYV-----	-E--SV-L-LL--TCITL--L--M
Bacillus sp. UNC41MFS5	WP_026568216	-P-FRVM-NR-RKWI-Y--SIYV-----	--F----L-LI--TC-SYL-L---
Bacillus sp. URHB0009	WP_027323469	-P-FR-M-KK-Q-LV----SAYV-----	---KS--L-LI--TC-SFL-L-L-
Bacillus sp. V3-13	WP_101661157	-PEINEMF-R-RKYI-Y-M-VYI-----	-P--S--L-L-F-TSLSL--L-LM
Bacillus sp. V59.32b	WP_117307389	-PEMQLMFNRHLKY-LY---I-V-----	-DFR-----L---VA-SFF-L-LI
Bacillus sp. VT-16-64	WP_077109626	-D--Q-LFNRHRKYILY---IFV-----	-KH-S--M-LI--TILSFF-L-
Bacillus sp. X1(2014)	WP_038537789	-P--QAMFTR-RKWM----SFYV-----	-A--P--L-LIF-TSLSFF-L-LM
Bacillus sp. XXST-01	WP_126408152	-P-VQT--RQ-Q-YIL---SIYV-----	-------L-LI--TA--LF-M-L-
Bacillus sp. Y1	WP_119710253	-PEIQS--IR-RKYI-----LYV-----	-------L-LI--TSLSLF-L-L-
Bacillus sp. YR335	WP_111616039	-T-MQ-MFQRYRKYI-Y---LYVI----	-E--S--L-LI--TCITL--L---
Bacillus terrae	WP_120114830	-DN-Q-LFNR-RKYILY---ILV-----	-P--S--M-LI--TSISFF-L-MM
Bacillus testis	WP_050616595	-P-IQYMFKR-LKYILY---LYV-----	-N--AV---LI--TTV-LF-L-L-
Bacillus tuaregi	WP_071393286	--QFKTMFTRERKYI-Y---VYV-----	----S--L-LI--TSLSLF-L-L-
Bacillus weihaiensis	WP_072578180	-F--QLMFHSY-KYI-Y---LYV----L	-D--Q--L-LII-TSISF--L-M-
Bacillus zeae	WP_119114469	-PEIQT--QR-RKYI----SIYV-----	-EFKPV---LV--TGISLF-L-L-
Bhargavaea beijingensis	WP_092097466	-EN-R--HNR---A------ML------	S-WP-V---LI--V---L------
Bhargavaea cecembensis	WP_063179904	-EN-R--HNR---A------ML------	SPWP-V---LI--V---L------
Bhargavaea ginsengi	WP_092054507	-EN-R--HNR---A------LL------	SPWP-V---LI--V---L------
Chryseomicrobium excrementi	WP_100353393	--N-FH--KR--KYIL----A-V--AA-	-PQP------G---V--L------
Domibacillus antri	WP_075396982	-PE-QHSIKR-RKY-LY---IFV-----	--A-A-Y--LV--TSLSL--H--I
Domibacillus enclensis	WP_045852012	-PE-Q-SIKR-RTY-IY---IFV-----	--A-D----LF--TALSL--H--I
Domibacillus epiphyticus	WP_076766229	-PE-QHSIKR-RKY-IY---IFV-----	--A-S-Y--LI--TTLSL--H--I
Domibacillus indicus	WP_046174588	-PE-QHSIKR-RKY-IY---IFV-----	--A-EVY--LI--TVLSL--H--I
Domibacillus mangrovi	WP_073710732	-PE-Q-SIKR-RKY-LY---IFV-----	--TP--Y--LI--TALSL--H--I
Edaphobacillus lindanitolerans	WP_076756780	-EN-R--HNR---A------LLV-----	-PWPKV---LI--I---L------
Falsibacillus pallidus	WP_114745920	-PE-Q-MFNRHRKYI-Y---IYV-----	-E-K---L-LV--TAISLF-H-LM
Falsibacillus sp. GY 10110	WP_121681965	-PE---MFNRHRKYI-Y--SIYV-----	---KS--L-LV--TCLSLF-H-LM
Filibacter sp. TB-66	WP_124068802	-QT-QD-HKR--KV------LFV-----	-DFKQV---LI--S---L------
Halobacillus massiliensis	WP_082232670	-EEYQ-MMTR-RKWM-Y---ILV----L	-PW-P--L-LL--STISF--L-LM
Kurthia huakuii	WP_029497828	-VEM-L--RRI-KYILLF-LV-------	-T--QV---L-----C-LF---
Kurthia massiliensis	WP_010287054	-VEM-R--RRLRKFILLF-LV-------	---N-V---L-----C-LF---
Kurthia senegalensis	WP_010302440	-VEM-R--RRLQKYILVF-LV-----S-	-----V---L-I---C-LF---S-
Kurthia sibirica	WP_109306213	-VEM-R-FKR-IALIL---LV--I--S-	SP--VV---L-----C-LF---
Kurthia zopfii	WP_109350880	-VEM-R-FKK-SLMIIA--IV--I----	---HA----L-----C-LF---N-
Lysinibacillus acetophenoni	WP_097147639	-----H-FII--KA------L-I----L	-PFGS--L--G---F---------
Lysinibacillus boronitolerans	WP_128568277	RRGRY-----L-------	-P---V-----V--F---------
Lysinibacillus chungkukjangi	WP_107932498	-----H-F-M--KA------L-------	---P-V-----V--C---------
Lysinibacillus composti	WP_124763381	-------FSM--KA----F-L-------	-P--S--L--G---F---------
Lysinibacillus contaminans	WP_053585266	-----H-F-V---A------L-------	-----------I--F---------
Lysinibacillus endophyticus	WP_121214042	-----H-F-M--KA------L-------	--HP-V-----V--C---------
Lysinibacillus fluoroglycofeni	WP_107942041	-E---Y-F-K--KV------L-V---A-	-P-AS----LG---F---------
Lysinibacillus fusiformis	WP_009371933	-----H-F-V---A------L-------	-P---V-----V--F---------
Lysinibacillus halotolerans	WP_122971466	-----H-F-M--KA------L-------	---P-V-----V--C---------
Lysinibacillus jejuensis	WP_108306034	-----H-FTM---A------I--I--AA	---E-V------------------
Lysinibacillus macroides	WP_053993120	-------F-M---A------L-------	-P--SV-----V--F---------
Lysinibacillus manganicus	WP_036182305	-------FIM--KA------L-V----I	-P-GS--L-LG---F---------
Lysinibacillus massiliensis	WP_036171291	-----H-F-M--KA----F-LS------	----S--L--S---F---------
Lysinibacillus meyeri	WP_107839940	-E---Y-FTK--KV------L-V---A-	-P-AS-----G---F---------
Lysinibacillus odysseyi	WP_036150815	-----L-F-M--KA------L-V-----	--F------L-I--F---------
Lysinibacillus parviboronicapi	WP_107923660	-----H-F-V---A------L------L	----SV-----I--F---------
Lysinibacillus sinduriensis	WP_036200104	-----H-F-M--KA------L-------	---P-V-----V--C---------
Lysinibacillus sp. B2A1	AVK82690	-----H-F-M---A------L-------	-P---F-----I--F---------
Lysinibacillus sp. BF-4	WP_036146937	-----H-FTM---A------I--I--TA	---E-V------------------
Lysinibacillus sp. BK089	TCJ71492	-----H-F-V---A------L-------	-P---V-----I--F---------
Lysinibacillus sp. FJAT-14222	WP_053596448	-----H-F-V---A------L-------	-P---V-----I--F---------
Lysinibacillus sp. FJAT-14745	WP_053484445	-----H-I-V---A------L-------	-P---V-----I--F---------
Lysinibacillus sp. Marseille-P	WP_106783528	-----H-F-M--KA------L--I----	-P-G-M-L-LGI--F---------
Lysinibacillus sp. SGAir0095	QCR31425	-----H-F-M--KA------L-------	--NP-V-----V--C---------
Lysinibacillus sp. SYSU K30002	WP_126658909	-----H-F-M--KA------L-------	---P-V-----I--C---------
Lysinibacillus sp. YLB-03	WP_118875014	-----H-FTM--KA------L-------	--IPAV-----V--C---------
Lysinibacillus sp. ZYM-1	WP_054611259	-----H-F-V---A------L-------	-----V-----V--F---------
Lysinibacillus sphaericus	WP_075528995	-QN-QE-HSR-R-ATY-I--LFV-----	-EWN-V---LI--T---L------
Lysinibacillus telephonicus	WP_126295292	-----H-F-M--KA------L-------	-A-S-V-----V--C---------
Lysinibacillus xylanilyticus	WP_068986231	-----H-F-V---A------L-------	-P---V-----I------------
Lysinibacillus xyleni	WP_097071684	-----H-F-M--KA------L-------	--HP-------V--C---------
Mycobacteroides abscessus subs	SHP70831	-PEIQS--IR-RKYI-----LYV-----	-------L-LI--TSLSLF-L-L-
Paenisporosarcina antarctica	WP_134210657	-Q--Q-V--R--KLV-----F-------	-PV-SV---L-I-S---L----L-
Paenisporosarcina indica	WP_075618367	-Q--Q-V--R--KLV-----F------L	-P-NSV---L-------L----L-
Paenisporosarcina quisquiliarum	WP_090565626	--EMQS-FTK--KYM-----V------L	-P-S-----L---S---L------
Paenisporosarcina sp. TG-14	WP_017382145	-Q--QLV--R--KLV-----F-------	-PV-SV---L-I-S---L----L-
Paenisporosarcina sp. TG20	WP_019414225	-Q--QEV--R--KLVIY---F-------	-PF-SV---LS------L------

Other Bacteria (0/>100)

Planococcus antarcticus	WP_006829236	-DG-RE--TRL-KMI--I--FYVI----	----EV---LII-T---I--M---
Planococcus citreus	WP_121298338	-QE--KRL--LMY-I--VFV----V	-P-PEV---LIV-S---I--M---
Planococcus donghaensis	WP_065525528	-DG-RE--TRL-KMII-I--FYVI----	----EV---LII-T---I--M---
Planococcus halocryophilus	WP_008496900	-RE--TRL-KMII-I--FYVI-L--	----EV---LII-T---I--M---
Planococcus maitriensis	WP_112230473	-DG-QE--KRL--LMY-I--AFV----V	-P-PEV---LIV-S---I--M---
Planococcus maritimus	WP_068464148	-QE--KRL--LMY-I--AFV----V	-P-PEV---LIV-S---I--M---
Planococcus massiliensis	WP_052650686	-RE--TRL--MIY-I--AFVI----	-P--EV---LLI-T---I--M---
Planococcus plakortidis	WP_068870045	-DG-QE--KRL--LMY-I--AFV----V	-P-PEV---LIV-S---I--M---
Planococcus rifietoensis	WP_058382964	-QE--KRL--LMY-I--VFV----V	-P-PEV---LIV-S---I--M---
Planococcus salinarum	TAA72921	-RE-FTRL--III-I--IFV-----	-P--D----LII-T---I--M---
Planococcus salinus	WP_123164524	-RE--TRL-KMI--I--AFV----L	-P--AV---LII-T---L--M---
Planococcus sp. CAU13	WP_033542496	-RE-FTR---II--I--LFV-----	-P--D----LII-T---I--M---
Planococcus sp. PAMC 21323	WP_038704712	-RE--TRL-KMII-I--LYVI----	----EV---LII-T---I--M---
Planococcus sp. Y42	WP_077590168	-S-F-E--EKL-KMV--I--LF------	-P-PSV---L---TA--L------
Planococcus versutus	WP_049694101	-EG-RE--TRL-KMI--I--FYVI----	-P--EV-L-LII-T---I--M---
Planomicrobium flavidum	WP_088009578	-S---E---KLRKMV--V--L-------	-P-PSV---L---TA--F------
Planomicrobium glaciei	WP_036809798	-DG-RE--TRL-KMMY-I--AFVI----	----EV---LII-S---I--M---
Planomicrobium koreense	WP_135500189	-RE--ERL-KMMY-I--AFV----L	-P--A----LII-S---I--M---
Planomicrobium soli	WP_106532850	-DG-KE--TRL--MIY-I--AFVI----	-P-------LII-I---I--M---
Pontibacillus halophilus	WP_026799010	-PQYDSMI-R-RK-M--V--IFV-----	----QV-L-LL--SVISFF-LRL-
Psychrobacillus insolitus	WP_111439322	--EMQG-FTK--KYE-----V--I----	-P-S-F-------S---L------
Psychrobacillus psychrodurans	WP_093493835	--EMQS-FTK--KYM-----V------L	-P-S-----L---S---L------
Psychrobacillus psychrotolerans	WP_093538716	--EMQR-FTR--KYIY----A-------	-P-S-----L---S---L------
Psychrobacillus sp. OK028	WP_093061313	--EMQR-FTK--KY------V------L	-P-S-----L---S---L------
Psychrobacillus sp. OK032	WP_093267882	--EMQR-FTK--IYI-----V-------	-P-S-----L---S---L------
Rummeliibacillus pycnus	WP_102693093	--E----F-K--KV------FF------	-PLK-V---L-V-V---Q------
Rummeliibacillus stabekisii	WP_066791744	--EM---F-R--KM------F-------	--FK-V---LIV-V---H------
Solibacillus isronensis	WP_079524647	--N--HVF-M--KAF-----V-------	SP-DS---------F---------
Solibacillus silvestris	WP_065216611	--N--HVF-M--KAF-----I-T-----	SP-DS---------F---------
Solibacillus sp. R5-41	WP_099422402	------VF-M--KAF-----V-------	SP-ES---------F---------
Sporosarcina globispora	WP_053433473	-PEIKA-FQR-RKYI-L--SVYV-----	-P--S--L-LI--TS-SLF-L-LI
Sporosarcina koreensis	WP_060209507	-HT-RH--NR--KA------LFVI--A-	SDFRPF---MI--S---L------
Sporosarcina newyorkensis	WP_078817031	-QSMQE-FNR--KSF-----LFG---F-	LD-R-----LI--SS--M------
Sporosarcina pasteurii	WP_115359936	-QN-QE-HSR-RKLT--TI-LF----VI	-GWN-V---LI--T---L------
Sporosarcina psychrophila	WP_067213126	-QT-QE-HRK--KA------LFV---AL	-DLKP----LI--S---L------
Sporosarcina sp. EUR3 2.2.2	WP_024535552	----Q-V--R--KLI-----F-------	-P-P-V---L---G---L----L-
Sporosarcina sp. HYO08	WP_067405509	-QT-QEVHNR-RKA------LFVI----	-NF--V---LI--S---L------
Sporosarcina sp. P13	WP_099688717	-QSMAE-FSR--KSF-----LFG---FL	LDFR-----LI--S---M----V-
Sporosarcina sp. P19	WP_099691268	-QSMQE-FKQ--KSF-----LFV---F-	LD-R-----LI--S---M------
Sporosarcina sp. P33	WP_081243856	-QSMQE-FKQ--K-F-----LFV---FL	LD-R-----LI--S---M------
Sporosarcina sp. PTS2304	WP_114925699	-QSMAE-FSR--KSF-----LF----LV	LD-R-----LI--SV--M------
Sporosarcina ureae	WP_085132712	-QSMQE-FKQ--KSF-----LFV---F-	FD-R-----LI--S---M------
Tetzosporium hominis	WP_094943629	--N-FH--KR--KYIL----A-V--AA-	-P-P------G---V--L------
Ureibacillus thermophilus	QBK25255	-----RTFDK--KA------L-V---F-	-P-P---S--GI--I---------
Ureibacillus thermosphaericus	WP_016837241	-------FIK--KA------L-II--F-	-P-P---S--GI------------
Viridibacillus arvi	WP_053417888	--E----F-K---M------F-----A-	-P---V---L-------H------
Viridibacillus sp. OK051	WP_100794833	--E----F-K---M------F-----A-	-P---V---L-------H------

Supplemental Figure 93

A partial sequence alignment of the ATP synthase subunit I protein containing a one amino acid insertion (boxed) that is exclusively shared by all members belonging to the genus *Caryophanon* and absent in all other bacteria.

			240		288
Caryophanon (2/2)	Caryophanon latum	WP_066461928	ISDYRNVNGEAGGMQHRLKMYGKKQC		ACGAATESLTIAGRTSVYCPACQ
	Caryophanon tenue	WP_066542156	------------------------I-		S--TE---------------L--
**Other Bacteria** (2/>100)	Gemella sanguinis	WP_006363515	----VHAD-GE-N--NFHQI----K-		PL-HDV-NIN-K--S-H---V--
	Gemella asaccharolytica	WP_066129746	----V-SE--K-N--NY--I----V-		PL-H--KNIK-K--STYF--E--
	Bacillus cecembensis	WP_057989779	-----SI--G--T-----------E-	L	---TE-N-MV-G--------Y--
	Bacillus ndiopicus	WP_042474724	-----T---G--S--N-----SH---	P	L-QTE-T-VV------T---S--
	Bacillus sp. FJAT-22090	WP_053588652	-----QI---S-K--N--Q--A--T-	P	I-N-K-LQK--G----T--GK--
	Bacillus sp. OxB-1	WP_041075521	------I--Q--S--D------R-T-	P	---ET-K-MK-------------
	Bhargavaea beijingensis	WP_092097621	------I--------D--R---RQY-	P	---T--KRM-----------K--
	Bhargavaea cecembensis	WP_063177840	------I--------E--R---R-S-	P	E---E-RRV-----------K--
	Bhargavaea ginsengi	WP_092051843	------I-----N--E--R---R-N-	P	G---E-KRM--G--------K--
	Chryseomicrobium excrementi	WP_100352283	---------G--N---F---------	P	T--TE-KQMV-G----T---V--
	Edaphobacillus lindanitolerans	WP_076758142	------I-----S--D--Q-----H-	P	S--SE-KRR--G--------K--
	Filibacter sp. TB-66	WP_124069855	------I-----T--D------R-V-	Y	N--MP-K-VK----------K--
	Jeotgalibacillus alimentarius	WP_041120938	---------------E--Q--A-SV-	P	V-ESPVKKAVVG----H--TT--
	Jeotgalibacillus campisalis	WP_041060015	---------Q--S--D--Q---R-E-	P	K--SNVKQAV-G--N-H--GK--
	Jeotgalibacillus malaysiensis	WP_039810304	---------------E--Q--A-ST-	P	V--S-VKKAVVG----H--TT--
	Jeotgalibacillus proteolyticus	WP_104057100	------------S--E--Q-----V-	P	S----VKQAV-G--N-H--SS--
	Jeotgalibacillus salarius	WP_134381574	---------------E--Q--A-SV-	P	V-QSPVKKAVVG----H--TT--
	Jeotgalibacillus soli	WP_041090717	---------Q--E--D--Q---R-V-	P	---S-IKQAV-G--N-H--TT--
	Kurthia huakuii	WP_029499437	------I-----S---H-Q---M-K-	P	M-HTN--QQI----N-F---N--
	Kurthia massiliensis	WP_010288267	------I-----------Q---M-K-	P	M-HTN--QQI----N-F---N--
	Kurthia senegalensis	WP_010303734	------I-----S--T--Q---M-K-	P	M-HTN--QEI----N-F---N--
	Kurthia sibirica	WP_109305208	------I---S-S--N------M-K-	P	M-HTNSQQIL--N-N-YF--N--
	Kurthia sp. 3B1D	WP_126989227	------I---------H-Q---M-K-	P	M-HTN--QQI----N-F---N--
	Kurthia zopfii	WP_109349030	------I--------D------MNI-	P	M-HTN-KQK-----N-Y---N--
	Lysinibacillus acetophenoni	WP_097148529	------I-------------------	P	T--SN-K-M--G--------Q--
	Lysinibacillus boronitolerans	WP_016993202	------I-----S--N--------V-	P	--ET--SQM--G-----F--N--
	Lysinibacillus chungkukjangi	WP_107934224	------I--------D--------T-	P	Q-NT--K--ILG--------K--
	Lysinibacillus composti	WP_124762439	------I-----------------I-	P	N-SSN-K-M-----------K--
	Lysinibacillus contaminans	WP_053582478	------I-----S--N--Q-----V-	P	---TP-SQM--G-----F--N--
	Lysinibacillus endophyticus	WP_121212718	------I-----------------I-	P	Q--SN-K-MV-G--------Q--
	Lysinibacillus fusiformis	WP_004226269	------I-----S--N--------V-	P	--ET--SQM--G--------N--
	Lysinibacillus halotolerans	WP_122971127	------I-----S-----------N-	P	T--TL-K--V-G-----F--K--
	Lysinibacillus jejuensis	WP_108306443	------I--------DY-Q-----V-	P	L--EP-LQA-------T--GR--
	Lysinibacillus macroides	WP_053996440	------I-----S--N--------I-	P	--ET--SQM--G-----F--N--
	Lysinibacillus manganicus	WP_036188104	------I-------------------	G	V--SN-K--V-G--------Q--
	Lysinibacillus mangiferihumi	WP_107895031	------I-----S--N--Q-----I-	P	V--TG-SQM--G--------N--
	Lysinibacillus massiliensis	WP_036173934	------------S-----------V-	P	N--TI-K--I-G--------Q--
	Lysinibacillus meyeri	WP_107839795	-----T---G--S--N------H---	P	L-QS----VV------T---S--
	Lysinibacillus odysseyi	WP_036156278	-----S---G--S--N-----S--T-	P	I-QK--K-VV------T---S--
	Lysinibacillus parviboronicapiens	WP_107923411	------I--G--S--N--------I-	L	E--TD-SQM--G-----F--N--
	Lysinibacillus saudimassiliensis	CEA05654	------I--------DY-Q-----V-	P	L--EP-LQE-------T--GR--
	Lysinibacillus sinduriensis	WP_036198697	------I--------N--------T-	P	E-DT--K--I-G--------K--
	Lysinibacillus sp. AR18-8	WP_066037546	------I-----S--N--------V-	P	--ET--SQM--G--------N--
	Lysinibacillus sp. ZYM-1	WP_054610815	------I-----S--N--------I-	P	---T--SQM-VG-----F--N--
	Lysinibacillus sphaericus	WP_125103297	------I--D--S--N--Q---R-V-	P	K--VE-A-IQ--N---T--KK--
	Lysinibacillus telephonicus	WP_126295376	------I-----------------S-	P	Q--TS-K--V-G--------Q--
	Lysinibacillus varians	WP_025220299	------I-----S--N--Q-----I-	P	V--TG-SQM--G--------N--
	Lysinibacillus xylanilyticus	WP_100544886	------I--------N--Q-----M-	P	T--T--SQM--G-----F--N--
	Lysinibacillus xyleni	WP_097072863	------I-----------------I-	P	Q--ND-K-MV-G--------Q--
	Macrococcus brunensis	WP_133431155	--S----E--S-Q--T-F-I--RQT-	G	T--REV-KAV----NTH--TH--
	Macrococcus caseolyticus	WP_133444924	------AQ--S-T--N-F-V----I-	G	T--NEIKTKV--T-NTH--TH--
	Macrococcus hajekii	WP_133428803	--S----E--S-Q--T-F-I--R-T-	G	T--SDVQTAV----NTH--TI--
	Macrococcus sp. DPC7161	WP_129064843	---------QS-Q--N-FYV------	G	Y--SDI-HIV----N-H--AT--
	Paenisporosarcina antarctica	WP_134209382	------I--------D--Q-------	P	N-IN--KQ-VLG--N-W---S--
	Paenisporosarcina indica	WP_075620039	------I--------D--Q--S--H-	P	N-LIP-KQVVLG--N-W---S--
	Paenisporosarcina quisquiliarum	WP_090564213	-----TI--G--N--N--Q-----E-	G	V--TK-KQK--G----T---T--
	Paenisporosarcina sp. HGH0030	WP_016426697	----I--------N--S-------	P	D-LIS-KQVVLG--N-W---S--
	Paenisporosarcina sp. K2R23-3	WP_119883562	-----SI-----S--D--Q-----N-	P	T--KTVLQKV----N-W--TS--
	Paenisporosarcina sp. OV554	WP_108585345	------I-----S-----Q-------	P	H-LVP-KQRVLG--N-W---S--
	Paenisporosarcina sp. TG-14	WP_017379901	------I--------D--Q-------	P	N-IN--KQ-VLG--N-W---S--
	Paenisporosarcina sp. TG20	WP_019415943	------I--------D--Q-------	P	V-SIP-KQ-VLG--N-W---S--
	Planococcus antarcticus	WP_006831140	------I---S-S--N-FG-------	A	D--T--KT-K-G--A-M---S--
	Planococcus citreus	WP_121300246	------I---S-S--N-FG-----T-	T	V--TE-KTVK--S-A-----S--
	Planococcus donghaensis	WP_065526669	-----DI---S-N--N-FG-------	A	D--T--KT-K-G--A-----T--
	Planococcus faecalis	WP_071153187	------I---S-S--D-FG-------	M	V--TM-KT-K-G--A-----T--
	Planococcus halocryophilus	WP_065527882	-----DI---S-S--N-FG-------	A	N--TQ-KT-K-G--A-----T--
	Planococcus halotolerans	WP_112224666	------I---S----N-FA-------	M	T--SV-KT-K-G--------T--
	Planococcus kocurii	WP_058385714	------I---S-S--D-FG-------	M	V--TM-KT-K-G--A-----T--
	Planococcus maitriensis	WP_112232626	------I---S-S--D-FG-----T-	T	V--TE-KTVK--S-A-----S--
	Planococcus maritimus	WP_068461690	------I---S-S--N-FG-----A-	S	F--TE-KTVK----A-----S--
	Planococcus massiliensis	WP_052651997	----------S-N--N-FG-------	M	E--SM-K--Q-G--------T--
	Planococcus plakortidis	WP_068868883	------I---S-S--N-FG-----T-	T	I--TE-KTVK--S-A-----S--

Other Bacteria (2/>100)

Planococcus rifietoensis	WP_058381073	------I---S-S--N-FG-----T- T V--TE-KTVK--S-A-----S--
Planococcus salinarum	TAA70455	------I---S----N-FA------- M S--S--KT-K-G--------T--
Planococcus salinus	WP_123163613	------I---S-S--N-FG------- I G--S--KT-K-G--------K--
Planococcus sp. Y42	WP_077588701	----------S-S----------H- L E--HE--NMK-G----TF--V--
Planococcus versutus	WP_065524284	----------S-S--N-FG------- E V---M-KT-K-G--A-----S--
Planomicrobium flavidum	WP_088006620	------I---S-S------------ P D---E--KM--G-----F--S--
Planomicrobium glaciei	WP_074510648	------I---S-S--N-FG------- M V--S--K--K-G--------T--
Planomicrobium koreense	WP_135500324	------I---S-R--D-FG------- M E--T--K--Q----------T--
Planomicrobium okeanokoites	WP_084246453	------I---S----N-FA------- M T--SV-KT-K-G-----F--S--
Planomicrobium soli	WP_106533902	----------S-N--N-FG------- I E--S--KT-K-G--A--------
Planomicrobium sp. Y50	WP_135815787	------I---S----N-FA------- M T--SV-KT-K-G--------T--
Planomicrobium sp. Y74	WP_121633365	------I---S----N-FA------- M T--SV-KT-K-G--------T--
Psychrobacillus insolitus	WP_111437681	-----RI---T-N--N--Q-----I- P T-EKK-KQQ--G----T------
Psychrobacillus psychrodurans	WP_093496053	-----TI--G--N--N--Q-----E- G V--TK-KQK--G----T---T--
Psychrobacillus psychrotolerans	WP_093536906	-----TI--G--N--N--Q-----E- G F--TK-KQK--G----T------
Psychrobacillus sp. FJAT-21963	WP_056833029	-----QI---S-K--N--Q--A--T- P L-N-K-LQK--G----T--GK--
Psychrobacillus sp. OK028	WP_093060426	-----SI--G--N--N--Q-----E- G V--TK-KQK--G----T------
Psychrobacillus sp. OK032	WP_093266872	-----TI--G--N--N--Q--S--N- P I--RK-LQK--G----T--GS--
Rummeliibacillus pycnus	WP_102691082	------I--------D--Q-----A- P I-HTN-KQMV-S--N-----K--
Rummeliibacillus sp. POC4	WP_119417028	------I--------D--------T- P I-HTN-KQ-V-S--N-----N--
Rummeliibacillus sp. TYF005	WP_124218636	------I--------D--------T- P I-HTN-KQ-V-S--N-----N--
Rummeliibacillus stabekisii	WP_066788809	------I-----S--N--Q---R-A- P I-HTN-KQMV----N-----H--
Salinicoccus carnicancri	WP_017549079	-----G-S-QS-S--D-F-I-Q--K- A H--SDVKTKM--T-NTF--TK--
Salinicoccus halodurans	WP_046790415	-----G-S-QS-S--D-F-I-Q--R- S Q--GDVKTKV--T-NTF--TK--
Salinicoccus luteus	WP_031544544	-----G-S--K-S--D-FHI-Q--S- S S--GPVKTK---T-NTF--TK--
Salinicoccus roseus	WP_124009918	-----G-S--K-S--D-FHI-Q--S- S S--GPVKTK---T-NTF--TK--
Salinicoccus sediminis	WP_046512074	-----G-S-QS-S--D-F-I-Q--K- A C--GDVKTKV--T-NTF--TK--
Solibacillus isronensis	WP_079528375	-----SI--G--T------------ V V-ETN----V------T---Q--
Solibacillus kalamii	WP_087615791	-----SI--G--T------------ V V-ETN----V------T---Q--
Solibacillus silvestris	WP_014823130	-----SI--G--T------------ V V-ETN----V------T---Q--
Solibacillus sp. R5-41	WP_099424818	-----SI--G--T-----------E- L ---TE-K-MV-G--------Y--
Sporosarcina koreensis	WP_060209863	------I-----T--T--------L- G K--TD-K-MK----------Q--
Sporosarcina newyorkensis	WP_040759261	-----S---N--T--N--H-----V- S T-ETP-K-KQ-------------
Sporosarcina pasteurii	WP_115362223	------I-----S--D------Q-E- P T-E-E-K-MQ-G--------K--
Sporosarcina psychrophila	WP_067207669	------I-----T--D------R-F- P ---S----MK----------T--
Sporosarcina sp. BI001-red	WP_116019484	------I-----S--D--Q---R-V- P I--SN-A-IQ--N---T--KK--
Sporosarcina sp. D27	WP_025783753	------I-----S--N--Q---R-V- P I--SE-A-IQ--N---T--KK--
Sporosarcina sp. EUR3 2.2.2	WP_024534242	------I-----------Q------- P H-FIP-KQRVLG--N-W---S--
Sporosarcina sp. HYO08	WP_067402838	------I--Q--T--D------R-T- P D--KE-K-MK-------------
Sporosarcina sp. P13	WP_099687575	-----S---N--T--D--H-----V- T C--KL-K-KQ-------------
Sporosarcina sp. P16b	WP_099672488	-----S---N--T--D--H-----T- V --DTP-K-KQ-------------
Sporosarcina sp. P18a	WP_099674409	-----S---N--T--D--H-----T- V T-DTP-K-KQ-------------
Sporosarcina sp. P19	WP_099690648	-----S---N--T--D--H-----T- L --DTP-K-KQ-------------
Sporosarcina sp. P20a	WP_099677449	-----S---N--T--D--H-----T- L --DTP-K-KQ-------------
Sporosarcina sp. P26b	WP_099692566	-----S---N--T--N--H-----T- L E-DTP-K-KQ----------S--
Sporosarcina sp. P29	WP_099661232	-----S---N--T--D--H-----T- L S-DTP-K-KQ-------------
Sporosarcina sp. P3	WP_099639893	-----S---N--T--D--H-----T- V --DTP-K-KQ-------------
Sporosarcina sp. P33	WP_081242231	-----S---N--T--D--H-----I- T V--KP-K-RQ-------------
Sporosarcina sp. P34	WP_099695163	-----S---N--T--D--H-----T- V --DTP-K-KQ-------------
Sporosarcina sp. PTS2304	WP_114924043	-----S---N--T---H-H-----L- T S-NKP-K-KQ-------------
Sporosarcina sp. ZBG7A	WP_039044003	------I--D--S--N--Q---RRV- P K---E-A-IQ--N---T--KK--
Sporosarcina ureae	WP_029052901	-----S---N--T--D--H-----T- L --DTP-K-KQ-------------
Staphylococcus auricularis	WP_059107171	-----HAD-A--T--LH-NV-KQ-V- H N--SSI-TKIV---N-H------
Staphylococcus epidermidis	WP_032603461	-----HAD-KT-Q--LH-NV-KQ-K- K V--HSI-TKV----N-HF--N--
Staphylococcus simulans	WP_119550224	-----HAD-QT-H--EY--I-K--T- A V--GPI-TEV-G--N-HF--N--
Staphylococcus sp. HMSC055B03	WP_070682883	-----HAD-KT-Q--LH-NV-KQ-K- K V--HSI-TKV----N-HF--N--
Staphylococcus sp. SDB 2975	WP_123144891	V-S--HAD-KT-Q---Y--V-K--V- P V--GPIHTKV----N-H---H--
Tetzosporium hominis	WP_094944118	---------G--N---F--------- P S--TQ-KQMV-G----T---V--
Ureibacillus thermophilus	QBK26499	------I-----------------I- P N-S-N-KTM--G--------K--
Ureibacillus thermosphaericus	WP_016837140	------I-----------------V- P --N-S-K-MI-G--------K--
Viridibacillus arvi	WP_053416605	------I-----S-----------T- P S-NGD-KQKI-G--N-----N--
Viridibacillus sp. OK051	WP_100794498	------I-----S-----------N- P N-NEE-KQKI-G--N-----N--

Supplemental Figure 94

A partial sequence alignment of the bifunctional DNA-formamidopyrimidine glycosylase/DNA-(apurinic or apyrimidinic site) lyase protein containing a one amino acid deletion (boxed) that is exclusively shared by all members belonging to the genus *Caryophanon* and absent in all other bacteria. 2 *Gemella* species are exceptions which also share this CSI.
